# Supplementary figures and images for: Transcriptome reveals differential expression of flavor and color in closely related strains of tomato (Solanum lycopersicum)
Source: PeerJ. 2025 Oct 7;13:e20113. doi: 10.7717/peerj.20113 (PMC12513376; doi:10.7717/peerj.20113)

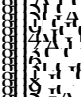

Supplement: Supplemental Information 3 [file peerj-13-20113-s003.pdf]
