# Supplementary material for: Transcriptome reveals differential expression of flavor and color in closely related strains of tomato (Solanum lycopersicum)
Source: PeerJ. 2025 Oct 7;13:e20113. doi: 10.7717/peerj.20113 (PMC12513376; doi:10.7717/peerj.20113)
Supplement: Supplemental Information 4 [file peerj-13-20113-s004.pdf]

Table S2: Transcriptome sequencing data from 12 samples

Sample	Raw Data		Valid Data		Valid Ratio(reads)	Q20%
	Read	Base	Read	Base		
Br19_1	43586696	6.54G	41360144	6.20G	94.89	98.55
Br19_2	37765316	5.66G	35608520	5.34G	94.29	98.54
Br19_3	44005926	6.60G	41448472	6.22G	94.19	98.52
Br20_1	43634662	6.55G	41136564	6.17G	94.27	98.39
Br20_2	43349532	6.50G	40847398	6.13G	94.23	98.49
Br20_3	37686158	5.65G	35527350	5.33G	94.27	98.58
MF19_1	44029070	6.60G	41576110	6.24G	94.43	98.56
MF19_2	43141790	6.47G	40541180	6.08G	93.97	98.46
MF19_3	37608686	5.64G	35303672	5.30G	93.87	98.44
MF20_1	38589638	5.79G	36771788	5.52G	95.29	98.47
MF20_2	39246528	5.89G	37501146	5.63G	95.55	98.09
MF20_3	40932462	6.14G	38639784	5.80G	94.4	98.22

Q30%	GC content%	Unique Mapped reads
93.58	42	37405135(90.44%)
93.8	42.5	32066535(90.05%)
93.5	42	38318733(92.45%)
93.23	42.5	37216810(90.47%)
93.51	42.5	37186659(91.04%)
93.82	42.5	32671129(91.96%)
93.74	42	38153960(91.77%)
93.36	42	36783944(90.73%)
93.53	42	31959539(90.53%)
93.69	42	33581369(91.32%)
92.05	42	34390920(91.71%)
92.17	42	35432656(91.70%)
