# Supplementary material for: Transcriptome reveals differential expression of flavor and color in closely related strains of tomato (Solanum lycopersicum)
Source: PeerJ. 2025 Oct 7;13:e20113. doi: 10.7717/peerj.20113 (PMC12513376; doi:10.7717/peerj.20113)
Supplement: Supplemental Information 5 [file peerj-13-20113-s005.pdf]

Table S3: Differential expressions of genes in Br19-vs-Br20

gene_id	FPKM.Br	FPKM.Br	FPKM.Br	FPKM.Br	FPKM.Br	FPKM.Br	fc	log2(fc)
Solyc09g0	90.86	96.51	95.79	209.35	212.39	210.41	0.45	-1.16
Solyc09g0	5.49	5.69	5.76	68.18	66.48	68.70	0.08	-3.59
Solyc09g0	160.62	162.06	154.44	75.70	76.08	74.80	2.11	1.07
Solyc09g0	769.76	775.45	790.13	2064.52	2126.85	2063.43	0.37	-1.42
Solyc10g0	1.17	1.03	1.20	40.63	39.44	40.32	0.03	-5.15
Solyc09g0	0.56	0.44	0.57	71.24	75.64	73.50	0.01	-7.13
Solyc07g0	11.54	11.79	10.86	68.10	68.73	62.02	0.17	-2.54
Solyc03g0	37.76	36.89	37.24	104.58	105.86	105.77	0.35	-1.50
Solyc02g0	335.50	347.97	343.34	750.62	792.83	781.19	0.44	-1.18
Solyc02g0	24.00	23.83	23.58	107.44	103.88	100.50	0.23	-2.13
Solyc03g0	121.32	114.56	109.52	1.83	2.04	1.86	60.27	5.91
Solyc12g0	138.75	139.24	143.24	503.95	498.15	500.77	0.28	-1.84
Solyc05g0	22.88	21.69	22.44	0.42	0.32	0.42	58.00	5.86
Solyc05g0	3.89	3.38	3.42	75.30	68.54	68.18	0.05	-4.31
Solyc02g0	4.18	4.62	4.35	31.47	31.88	32.46	0.14	-2.87
Solyc02g0	94.97	100.27	100.40	36.78	36.51	38.42	2.65	1.40
Solyc08g0	83.79	86.91	87.21	174.39	175.70	171.65	0.49	-1.02
Solyc05g0	40.26	38.14	37.82	132.08	132.31	134.65	0.29	-1.78
Solyc03g0	152.40	153.92	157.37	49.13	46.74	45.94	3.27	1.71
Solyc06g0	62.00	55.96	57.61	3.28	3.38	3.70	16.95	4.08
Solyc08g0	36.87	33.32	31.24	175.45	169.96	175.49	0.19	-2.36
Solyc09g0	313.48	305.73	316.10	678.35	701.41	687.31	0.45	-1.14
Solyc12g0	97.90	93.87	96.08	231.44	238.17	222.42	0.42	-1.27
Solyc02g0	1.50	1.59	2.20	90.22	85.25	84.10	0.02	-5.62
Solyc01g0	203.84	197.08	191.59	435.11	444.51	459.19	0.44	-1.18
Solyc05g0	1.52	1.19	1.77	184.82	185.07	185.64	0.01	-6.95
Solyc11g0	8.36	8.33	8.83	67.31	70.04	68.16	0.12	-3.01
Solyc09g0	5.95	5.06	6.29	40.35	41.16	40.20	0.14	-2.81
Solyc09g0	5.24	5.23	4.43	70.94	69.45	68.82	0.07	-3.81
Solyc10g0	127.72	113.50	108.45	2171.06	2143.92	2228.78	0.05	-4.23
Solyc09g0	2.33	1.91	2.37	24.87	24.86	25.27	0.09	-3.50
Solyc03g0	189.47	195.15	199.25	73.42	72.41	71.17	2.69	1.43
Solyc03g0	202.79	206.51	215.67	85.31	83.32	85.03	2.46	1.30
Solyc02g0	554.33	537.71	563.76	204.09	210.77	200.76	2.69	1.43
Solyc06g0	145.84	154.11	151.63	47.12	45.19	47.16	3.24	1.69
Solyc08g0	143.74	163.00	156.78	563.25	554.23	571.71	0.27	-1.87
Solyc02g0	345.57	363.37	348.42	81.60	80.46	84.65	4.29	2.10
Solyc02g0	106.53	114.32	118.24	8.98	9.35	8.70	12.54	3.65
Solyc01g0	98.35	112.28	106.25	384.08	398.19	378.66	0.27	-1.87
Solyc01g0	841.08	839.45	846.44	196.82	191.59	191.42	4.36	2.12
Solyc04g0	203.04	201.23	204.48	664.13	663.51	644.59	0.31	-1.70
Solyc04g0	79.26	78.90	77.20	166.43	168.96	167.50	0.47	-1.10
Solyc11g0	36.02	36.06	36.87	324.06	320.13	312.08	0.11	-3.13
Solyc09g0	127.69	127.79	126.84	54.23	56.67	55.00	2.30	1.20
Solyc03g1	10.61	11.54	10.64	38.59	37.63	40.02	0.28	-1.83
Solyc09g0	12.89	14.11	13.95	49.41	49.20	52.00	0.27	-1.88
Solyc02g0	62.88	59.27	59.57	19.75	20.33	19.82	3.03	1.60
Solyc06g0	88.45	90.96	90.77	34.44	33.81	33.60	2.65	1.41
Solyc01g1	41.06	40.42	41.82	89.48	92.27	93.09	0.45	-1.16

Solyc09g0	1.76	1.25	1.78	22.82	22.95	22.81	0.07	-3.84
Solyc09g0	0.27	0.12	0.16	24.67	26.21	26.58	0.01	-7.14
Solyc04g0	9.03	8.95	8.79	25.49	27.41	26.19	0.34	-1.56
Solyc11g0	35.05	34.09	33.43	78.63	83.42	78.45	0.43	-1.23
Solyc01g1	81.43	77.24	73.58	18.66	18.44	20.63	4.02	2.01
Solyc01g1	0.30	0.24	0.31	3.68	3.61	3.69	0.08	-3.71
Solyc03g1	430.85	400.80	393.71	882.50	867.54	853.00	0.47	-1.09
Solyc01g1	39.79	39.61	40.11	103.04	96.46	95.45	0.41	-1.30
Solyc11g0	2.63	2.77	2.43	18.73	19.71	20.56	0.13	-2.91
Solyc08g0	55.81	55.86	53.02	24.90	24.98	23.39	2.25	1.17
Solyc06g0	0.42	0.55	0.86	26.78	24.25	23.53	0.02	-5.35
Solyc09g0	34.73	36.64	35.91	97.82	95.31	99.72	0.37	-1.45
Solyc01g1	64.32	64.45	65.40	26.70	29.26	27.77	2.32	1.21
Solyc10g0	17.64	11.98	17.93	725.60	711.43	739.38	0.02	-5.52
Solyc01g0	30.02	28.74	29.66	13.20	12.66	13.10	2.27	1.18
Solyc01g1	28.56	27.94	28.34	8.94	9.57	9.41	3.04	1.60
Solyc02g0	148.91	168.24	155.89	351.51	345.28	363.16	0.45	-1.16
Solyc07g0	180.85	197.89	182.05	389.27	395.54	411.42	0.47	-1.09
Solyc03g1	55.79	55.56	56.97	25.99	26.00	24.14	2.21	1.14
Solyc07g0	11.49	10.97	11.62	38.65	37.47	36.23	0.30	-1.72
Solyc06g0	37.12	37.33	38.49	18.16	18.34	17.14	2.11	1.07
Solyc02g0	107.31	112.47	113.92	56.62	55.12	53.41	2.02	1.01
Solyc02g0	43.10	40.16	41.21	89.28	93.52	88.61	0.46	-1.12
Solyc09g0	64.66	55.03	58.43	157.02	162.19	160.12	0.37	-1.43
Solyc11g0	65.89	67.04	68.93	141.41	146.99	151.75	0.46	-1.12
Solyc06g0	6.43	6.59	5.90	25.90	26.76	26.00	0.24	-2.05
Solyc02g0	34.86	37.76	37.86	7.87	7.99	9.30	4.39	2.13
Solyc11g0	10.91	11.26	11.49	35.58	37.29	37.02	0.31	-1.71
Solyc02g0	92.34	92.25	93.89	42.48	41.64	43.87	2.18	1.12
Solyc10g0	0.55	0.62	0.46	8.71	9.23	10.35	0.06	-4.12
Solyc03g0	32.47	36.62	35.29	101.65	95.54	95.10	0.36	-1.49
Solyc06g0	535.51	495.37	461.71	17.58	17.54	23.36	25.52	4.67
Solyc07g0	4.14	4.46	3.89	27.13	25.65	23.74	0.16	-2.62
Solyc12g0	187.16	181.18	183.08	83.24	85.22	90.23	2.13	1.09
Solyc09g0	190.91	176.95	174.87	365.42	384.77	361.75	0.49	-1.03
Solyc01g0	2.76	3.01	2.79	9.00	9.02	9.55	0.31	-1.69
Solyc07g0	67.31	65.52	66.25	146.50	146.35	139.34	0.46	-1.12
Solyc03g0	210.50	187.06	210.68	64.99	60.40	55.85	3.36	1.75
Solyc09g0	2.24	2.31	2.08	6.96	7.17	6.64	0.32	-1.65
Solyc09g0	0.14	0.18	0.16	12.71	10.13	11.11	0.01	-6.18
Solyc11g0	14.87	14.69	15.80	30.57	31.36	31.10	0.49	-1.04
Solyc08g0	28.29	30.06	29.33	64.11	65.55	64.07	0.45	-1.14
Solyc06g0	57.96	54.68	57.34	23.03	23.81	23.29	2.42	1.28
Solyc09g0	50.71	48.09	48.83	99.78	101.91	100.89	0.49	-1.04
Solyc03g1	1.83	1.45	1.21	14.43	13.54	14.01	0.11	-3.23
Solyc12g0	19.21	18.74	17.29	53.30	54.35	55.90	0.34	-1.57
Solyc08g0	62.01	66.30	62.04	181.87	187.07	196.84	0.34	-1.57
Solyc05g0	1.85	2.11	1.75	10.94	10.59	11.09	0.18	-2.51
Solyc12g0	11.22	12.84	12.18	30.37	31.23	29.59	0.40	-1.33
Solyc05g0	2.50	2.70	2.35	11.35	11.23	11.01	0.22	-2.15
Solyc01g1	208.52	216.67	206.36	97.47	92.65	103.00	2.15	1.11

Solyc06g0	0.44	0.46	0.55	6.16	5.81	5.89	0.08	-3.62
Solyc06g0	432.68	435.64	380.39	1520.99	1592.12	1646.73	0.26	-1.93
Solyc06g0	13.15	12.69	12.30	1.88	1.78	1.80	6.98	2.80
Solyc01g1	74.57	84.48	83.59	170.58	174.22	172.48	0.47	-1.09
Solyc11g0	303.97	290.31	266.23	654.12	653.66	706.95	0.43	-1.23
Solyc01g0	52.15	52.59	51.40	108.42	103.00	105.70	0.49	-1.02
Solyc01g1	118.55	115.62	111.44	251.32	248.46	258.32	0.46	-1.13
Solyc12g0	19.91	17.78	19.42	45.42	45.39	45.10	0.42	-1.25
Solyc03g0	36.90	36.41	36.94	77.56	75.40	77.75	0.48	-1.07
Solyc03g0	103.46	101.27	87.73	223.82	233.83	229.09	0.43	-1.23
Solyc01g0	0.46	0.36	0.55	10.51	9.93	10.22	0.04	-4.49
Solyc05g0	0.39	0.29	0.33	13.37	12.85	12.37	0.03	-5.26
Solyc06g0	32.21	33.21	34.47	13.36	12.12	13.65	2.55	1.35
Solyc02g0	27.87	28.93	29.68	58.47	62.89	60.14	0.48	-1.07
Solyc08g0	72.49	67.86	64.37	140.83	141.49	137.19	0.49	-1.04
Solyc09g0	0.01	0.04	0.05	7.21	7.49	8.43	0.00	-7.76
Solyc01g0	61.78	64.04	61.08	17.92	17.09	15.73	3.68	1.88
Solyc09g0	12.35	12.78	12.70	4.80	4.78	4.75	2.64	1.40
Solyc09g0	33.48	31.56	33.19	11.78	12.44	11.94	2.72	1.44
Solyc07g0	64.35	61.30	63.05	27.23	25.58	26.94	2.37	1.24
Solyc06g0	15.75	17.39	15.82	44.05	44.55	44.64	0.37	-1.44
Solyc02g0	10.12	8.47	9.24	39.22	36.22	42.61	0.24	-2.08
Solyc04g0	147.04	136.17	128.66	272.69	285.87	282.87	0.49	-1.03
Solyc02g0	20.40	20.05	20.72	45.15	48.56	46.99	0.43	-1.20
Solyc02g0	24.97	25.80	25.71	52.77	52.66	53.27	0.48	-1.05
Solyc09g0	3.16	3.35	3.20	11.55	11.86	10.63	0.29	-1.81
Solyc05g0	14.02	15.31	14.74	31.68	32.83	31.52	0.46	-1.12
Solyc06g0	21.12	21.12	21.22	5.17	4.53	4.60	4.44	2.15
Solyc03g0	54.11	51.63	50.50	22.82	23.41	23.00	2.26	1.17
Solyc11g0	6.90	6.94	6.76	1.00	0.92	0.62	8.11	3.02
Solyc03g1	7.24	7.26	7.47	18.52	18.39	17.87	0.40	-1.32
Solyc12g0	27.88	27.12	30.32	60.51	61.56	57.89	0.47	-1.08
Solyc10g0	16.12	14.36	14.04	44.25	42.53	43.57	0.34	-1.55
Solyc09g0	130.33	148.15	116.36	32.05	36.96	39.01	3.66	1.87
Solyc10g0	106.46	100.02	98.70	52.16	46.80	47.13	2.09	1.06
Solyc01g0	63.16	61.80	59.43	30.55	29.19	29.73	2.06	1.04
Solyc05g0	42.49	41.58	43.10	21.30	21.31	20.59	2.01	1.01
Solyc08g0	10.90	10.83	11.58	2.23	2.12	2.21	5.08	2.34
Solyc07g0	10.56	10.10	10.13	22.42	21.77	20.31	0.48	-1.07
Solyc11g0	22.44	22.23	22.33	51.86	49.37	50.31	0.44	-1.18
Solyc10g0	18.57	21.30	19.15	46.55	50.06	46.81	0.41	-1.28
Solyc03g0	0.58	0.64	0.55	5.68	5.67	5.14	0.11	-3.22
Solyc01g1	3.03	3.18	3.36	10.28	10.34	10.01	0.31	-1.68
Solyc06g0	11.39	11.35	10.42	0.39	0.31	0.29	33.21	5.05
Solyc02g0	33.43	32.51	34.01	69.34	70.50	66.39	0.48	-1.05
Solyc05g0	44.96	45.15	46.66	22.15	21.97	23.72	2.02	1.01
Solyc08g0	1.36	1.54	1.53	7.19	7.25	6.89	0.21	-2.27
Solyc01g0	35.23	33.74	35.17	12.76	10.58	12.25	2.93	1.55
Solyc02g0	7.85	8.24	7.94	24.76	21.25	23.51	0.35	-1.53
Solyc08g0	63.60	73.36	71.21	139.54	143.67	145.46	0.49	-1.04
Solyc09g0	9.82	8.73	9.74	0.80	1.06	1.32	8.90	3.15

Solyc02g0	28.15	26.52	28.03	58.21	62.25	58.76	0.46	-1.12
Solyc04g0	19.70	18.83	18.77	42.76	42.33	40.66	0.46	-1.13
Solyc09g0	2.03	2.54	1.86	15.59	14.54	12.86	0.15	-2.74
Solyc11g0	39.11	39.49	36.63	13.31	11.50	13.65	3.00	1.58
Solyc06g0	19.53	18.43	18.54	43.70	42.57	43.05	0.44	-1.19
Solyc12g0	29.38	30.02	29.46	13.03	13.35	13.84	2.21	1.14
Solyc09g0	7.64	8.67	7.63	26.78	25.84	23.83	0.31	-1.67
Solyc09g0	49.97	45.10	45.39	104.81	104.99	106.64	0.44	-1.17
Solyc12g0	29.71	31.09	29.00	60.77	63.59	64.85	0.47	-1.08
Solyc01g0	48.03	45.82	43.17	97.52	103.57	104.09	0.45	-1.16
Solyc03g0	5.69	4.49	4.09	27.90	25.83	24.67	0.18	-2.46
Solyc10g0	15.86	16.56	17.73	34.00	36.66	36.21	0.47	-1.09
Solyc04g0	14.35	15.76	16.77	40.35	44.31	40.12	0.38	-1.41
Solyc04g0	17.42	17.10	15.95	34.22	36.64	34.64	0.48	-1.06
Solyc03g0	12.61	12.39	11.49	34.97	36.38	37.52	0.34	-1.58
Solyc08g0	8.03	7.37	7.90	20.32	20.37	18.88	0.39	-1.35
Solyc07g0	20.27	19.16	21.47	42.90	41.33	41.18	0.49	-1.04
Solyc02g0	5.41	5.10	5.58	0.58	0.44	0.34	11.83	3.56
Solyc03g1	3.76	3.53	3.93	12.51	12.90	11.61	0.30	-1.72
Solyc02g0	40.56	40.02	39.82	84.88	81.24	89.54	0.47	-1.09
Solyc08g0	18.78	18.05	18.60	8.76	7.84	7.88	2.26	1.18
Solyc09g0	13.39	15.18	15.82	5.12	5.33	5.00	2.87	1.52
Solyc03g0	16.76	16.42	15.98	6.94	7.00	6.44	2.41	1.27
Solyc05g0	1.84	1.91	1.64	7.03	7.94	7.13	0.24	-2.03
Solyc03g0	1.20	1.57	1.64	9.41	10.43	9.03	0.15	-2.71
Solyc11g0	0.59	0.70	0.47	4.84	5.01	4.24	0.13	-3.00
Solyc09g0	0.08	0.08	0.09	2.48	2.39	2.17	0.03	-4.88
Solyc09g0	1.91	1.23	1.12	13.98	13.41	17.42	0.10	-3.40
Solyc12g0	11.16	11.47	12.27	30.89	29.53	29.62	0.39	-1.37
Solyc03g0	10.86	12.27	11.69	25.85	26.22	25.51	0.45	-1.16
Solyc01g0	21.18	19.38	20.97	43.65	44.66	45.90	0.46	-1.12
Solyc05g0	18.31	17.10	18.97	9.12	9.17	8.35	2.04	1.03
Solyc05g0	18.39	17.55	18.17	5.27	5.68	5.49	3.29	1.72
Solyc08g0	1.02	1.09	1.01	5.63	5.46	5.06	0.19	-2.37
Solyc08g0	19.46	19.59	21.79	5.27	5.12	6.09	3.69	1.88
Solyc04g0	28.21	26.65	27.74	56.56	55.86	58.63	0.48	-1.05
Solyc10g0	18.88	19.69	17.61	45.61	41.89	43.93	0.43	-1.23
Solyc10g0	23.61	20.18	18.78	103.46	100.18	122.15	0.19	-2.38
Solyc06g0	8.64	9.18	9.27	26.01	25.61	21.94	0.37	-1.44
Solyc08g0	2.29	1.96	2.18	8.29	8.07	8.42	0.26	-1.95
Solyc01g1	106.77	111.56	99.00	207.39	208.61	225.13	0.49	-1.01
Solyc02g0	3.60	3.04	3.54	15.81	16.07	13.73	0.22	-2.16
Solyc04g0	34.71	33.23	34.83	11.46	12.67	11.05	2.92	1.55
Solyc09g0	7.18	7.33	8.88	18.65	18.67	17.25	0.43	-1.22
Solyc04g0	2.64	3.03	2.33	20.60	21.43	20.47	0.13	-2.97
Solyc07g0	32.79	33.62	31.79	71.03	70.76	77.02	0.45	-1.16
Solyc05g0	0.61	0.68	0.91	36.40	30.48	33.96	0.02	-5.52
Solyc03g0	7.59	9.13	8.40	0.50	0.27	0.55	18.97	4.25
Solyc04g0	2.92	2.87	2.99	6.10	6.26	5.95	0.48	-1.06
Solyc06g0	0.80	1.45	1.01	7.58	7.75	8.49	0.14	-2.87
Solyc01g0	21.70	21.98	18.62	49.14	49.70	50.05	0.42	-1.26

Solyc01g0	22.78	19.86	23.29	9.50	8.32	8.29	2.53	1.34
Solyc05g0	4.16	4.25	3.64	10.88	11.60	10.17	0.37	-1.44
Solyc02g0	36.07	35.54	34.42	16.35	16.53	16.57	2.14	1.10
Solyc07g0	0.33	0.19	0.38	3.70	3.99	3.29	0.08	-3.60
Solyc02g0	8.66	7.89	8.10	18.09	17.92	16.74	0.47	-1.10
Solyc01g0	18.25	18.65	18.81	7.88	8.08	7.07	2.42	1.27
Solyc09g0	9.74	10.52	9.90	0.58	0.61	0.61	16.68	4.06
Solyc01g1	5.47	5.23	5.99	1.15	0.87	0.76	5.99	2.58
Solyc08g0	2.20	1.97	2.10	6.78	7.16	6.46	0.31	-1.70
Solyc01g0	1.04	1.29	1.25	6.63	6.06	5.25	0.20	-2.32
Solyc04g0	0.81	0.67	0.83	4.33	4.31	3.92	0.18	-2.44
Solyc05g0	8.95	9.24	8.89	21.40	19.60	19.10	0.45	-1.15
Solyc04g0	5.69	6.29	4.70	19.77	21.02	21.24	0.27	-1.90
Solyc04g0	0.98	1.13	0.88	6.11	5.95	5.36	0.17	-2.54
Solyc09g0	34.16	29.98	31.51	11.69	9.11	10.36	3.07	1.62
Solyc03g0	119.06	119.96	123.09	39.95	41.83	38.77	3.00	1.59
Solyc04g0	35.40	35.97	34.04	16.37	15.59	17.03	2.15	1.11
Solyc07g0	0.05	0.06	0.03	1.70	1.77	1.62	0.03	-5.23
Solyc07g0	27.71	27.04	25.37	10.04	9.63	9.47	2.75	1.46
Solyc03g0	5.73	5.40	5.25	12.30	12.16	11.41	0.46	-1.13
Solyc04g0	8.25	8.54	9.60	20.87	21.11	20.23	0.42	-1.24
Solyc07g0	14.88	12.79	16.00	35.57	35.10	35.27	0.41	-1.28
Solyc01g0	70.85	75.62	64.02	27.51	27.21	24.72	2.65	1.41
Solyc04g0	23.95	22.06	21.85	8.29	9.22	8.98	2.56	1.36
Solyc08g0	15.33	16.50	16.01	8.05	7.56	7.90	2.03	1.02
Solyc08g0	1.97	2.45	2.60	7.44	8.08	8.15	0.30	-1.75
Solyc12g0	11.86	10.16	10.78	4.10	3.80	4.18	2.72	1.44
Solyc05g0	12.45	12.10	13.54	5.73	6.59	5.40	2.15	1.10
Solyc09g0	11.21	10.22	12.01	22.95	22.98	23.90	0.48	-1.06
Solyc07g0	2.97	0.86	2.08	142.78	140.79	149.40	0.01	-6.20
Solyc11g0	1.24	1.07	0.85	7.58	6.95	5.81	0.16	-2.69
Solyc03g0	19.30	20.23	19.56	9.26	9.78	10.11	2.03	1.02
Solyc08g0	1.95	1.84	2.21	7.49	8.47	6.86	0.26	-1.93
Solyc01g0	2.46	2.45	2.40	8.74	8.80	7.58	0.29	-1.78
Solyc07g0	13.07	11.11	13.00	31.85	30.78	27.56	0.41	-1.28
Solyc09g0	14.44	15.46	14.96	4.62	5.24	4.22	3.19	1.67
Solyc10g0	0.15	0	0.07	9.14	11.64	9.17	0.01	-7.08
Solyc04g0	3.98	4.19	3.99	8.81	9.12	8.87	0.45	-1.14
Solyc12g0	9.37	8.02	8.18	19.45	19.39	20.85	0.43	-1.22
Solyc04g0	10.45	9.84	9.54	21.16	23.24	21.89	0.45	-1.15
Solyc01g0	1.71	1.13	1.52	5.67	5.64	5.13	0.26	-1.92
Solyc02g0	2.30	2.41	2.33	5.61	5.46	5.50	0.42	-1.24
Solyc01g0	7.17	6.66	8.24	2.80	2.91	2.77	2.60	1.38
Solyc12g0	10.87	11.42	10.01	4.42	4.40	4.85	2.36	1.24
Solyc02g0	4.78	5.31	4.40	12.27	11.47	11.13	0.42	-1.27
Solyc10g0	716.98	673.35	611.42	1389.89	1475.50	1433.00	0.47	-1.10
Solyc12g0	1.46	1.15	1.50	6.40	6.73	7.37	0.20	-2.32
Solyc02g0	19.87	18.90	16.56	40.57	40.88	39.94	0.46	-1.13
Solyc03g0	12.63	13.55	14.23	2.55	2.53	2.08	5.64	2.50
Solyc04g0	83.58	88.24	74.26	33.27	30.29	32.95	2.55	1.35
Solyc01g0	12.13	13.51	14.16	5.23	4.78	5.30	2.60	1.38

Solyc02g0	0.65	0.67	0.67	1.72	1.65	1.50	0.41	-1.29
Solyc05g0	2.80	2.66	2.89	1.14	1.17	1.37	2.27	1.18
Solyc06g0	1.35	1.69	1.71	8.43	8.80	8.91	0.18	-2.46
Solyc01g0	10.60	10.19	11.22	32.61	32.30	31.12	0.33	-1.59
Solyc12g0	10.72	9.97	10.24	4.29	3.79	4.53	2.45	1.29
Solyc09g0	20.23	17.80	18.90	41.55	43.48	42.42	0.45	-1.16
Solyc08g0	7.01	6.81	6.74	2.82	2.48	2.23	2.73	1.45
Solyc01g0	10.43	8.81	9.29	20.30	20.69	20.02	0.47	-1.10
Solyc07g0	43.26	35.91	36.91	95.79	95.37	101.59	0.40	-1.33
Solyc02g0	0.46	0.58	0.90	4.50	4.48	3.77	0.15	-2.72
Solyc03g1	4.40	4.23	4.28	1.31	1.32	1.52	3.11	1.63
Solyc06g0	23.10	21.24	21.28	47.46	51.03	50.81	0.44	-1.19
Solyc01g1	4.58	5.01	4.45	1.33	1.30	1.43	3.45	1.79
Solyc03g0	1.24	1.48	1.51	4.24	4.29	4.24	0.33	-1.60
Solyc06g0	6.44	5.67	6.39	1.57	1.34	1.63	4.08	2.03
Solyc09g0	6.06	4.36	4.59	0.73	1.02	0.84	5.78	2.53
Solyc06g0	10.33	9.43	9.77	19.99	21.95	21.15	0.47	-1.10
Solyc01g1	1.24	0.94	0.83	4.91	6.01	4.71	0.19	-2.38
Solyc01g0	0.04	0.11	0.21	3.23	3.15	3.78	0.04	-4.82
Solyc01g0	14.32	13.69	15.94	32.75	32.40	30.82	0.46	-1.13
Solyc08g0	4.39	3.97	4.44	8.76	8.58	8.38	0.50	-1.01
Solyc09g0	9.98	10.96	7.72	33.68	32.96	32.01	0.29	-1.78
Solyc11g0	9.40	9.67	9.42	4.55	4.79	4.21	2.10	1.07
Solyc11g0	13.61	13.53	14.27	25.49	29.48	28.59	0.50	-1.01
Solyc09g0	2.37	2.70	2.66	0.32	0.40	0.46	6.55	2.71
Solyc01g0	23.02	26.20	23.08	44.64	49.53	50.95	0.50	-1.01
Solyc06g0	10.69	9.45	10.49	21.38	22.87	21.02	0.47	-1.09
Solyc04g0	5.18	4.26	5.03	11.17	12.61	12.68	0.40	-1.33
Solyc08g0	11.62	12.19	12.95	5.66	6.37	5.98	2.04	1.03
Solyc10g0	3.26	2.77	3.33	7.05	7.26	6.62	0.45	-1.16
Solyc02g0	4.29	4.30	3.50	0.73	1.01	0.79	4.79	2.26
Solyc09g0	7.92	8.34	8.44	16.77	16.08	17.53	0.49	-1.03
Solyc03g1	1.66	1.74	1.88	4.39	4.46	4.56	0.39	-1.35
Solyc10g0	0.20	0.27	0.13	2.25	2.17	2.44	0.09	-3.52
Solyc06g0	0.18	0.12	0.07	2.03	2.24	2.25	0.06	-4.15
Solyc08g0	0.82	0.95	0.81	4.03	4.19	4.26	0.21	-2.28
Solyc08g0	18.64	17.20	19.16	9.47	8.83	7.86	2.10	1.07
Solyc08g0	0.47	0.52	0.57	2.11	1.91	1.88	0.26	-1.92
Solyc02g0	27.87	24.01	28.03	52.35	56.30	52.91	0.49	-1.02
Solyc01g1	2.54	3.06	3.35	11.22	9.93	10.97	0.28	-1.84
Solyc09g0	3.00	3.04	2.65	6.71	6.63	6.17	0.45	-1.17
Solyc04g0	172.55	148.53	142.05	351.77	357.37	373.22	0.43	-1.22
Solyc12g0	2.80	2.99	2.57	8.18	8.39	8.11	0.34	-1.56
Solyc08g0	4.10	3.46	4.42	11.44	11.75	11.84	0.34	-1.55
Solyc07g0	1.47	1.49	1.83	4.87	4.43	5.12	0.33	-1.59
Solyc02g0	4.39	3.41	4.03	10.65	10.33	11.65	0.36	-1.46
Solyc03g1	3.69	3.25	3.43	8.04	8.65	8.35	0.41	-1.27
Solyc05g0	0.37	0.30	0.59	5.46	5.84	4.76	0.08	-3.67
Solyc07g0	3.07	2.91	2.85	7.91	7.45	7.43	0.39	-1.37
Solyc08g0	0.23	0.32	0.34	3.68	3.37	4.23	0.08	-3.67
Solyc11g0	1.35	1.08	1.45	7.51	7.29	5.74	0.19	-2.40

Solyc08g0	0.68	0.78	1.02	4.90	5.37	4.81	0.16	-2.60
Solyc09g0	0.56	0.80	0.64	3.10	3.31	3.14	0.21	-2.26
Solyc03g1	1.32	1.56	1.48	4.15	3.86	3.65	0.37	-1.42
Solyc01g0	2.26	2.31	2.14	7.60	7.08	7.54	0.30	-1.73
Solyc09g0	7.52	7.10	6.27	14.26	14.13	13.94	0.49	-1.02
Solyc06g0	4.12	4.12	4.06	0.78	0.60	0.36	7.07	2.82
Solyc02g0	3.82	3.92	4.45	1.36	1.78	1.47	2.64	1.40
Solyc06g0	5.54	5.61	5.04	2.13	1.96	2.11	2.61	1.39
Solyc07g0	6.21	5.99	5.83	12.79	13.97	14.25	0.44	-1.19
Solyc05g0	1.19	1.40	1.21	3.27	3.55	3.32	0.37	-1.42
Solyc09g0	8.65	6.93	7.46	19.12	19.24	21.04	0.39	-1.37
Solyc03g0	6.03	5.68	5.48	11.55	13.22	12.31	0.46	-1.11
Solyc09g0	11.45	14.23	10.28	26.57	29.16	28.10	0.43	-1.22
Solyc03g0	2.94	2.85	2.87	1.11	1.03	1.22	2.58	1.37
Solyc09g0	0.37	0.57	0.10	5.56	6.89	7.17	0.05	-4.24
Solyc01g0	1.28	1.35	1.16	5.47	4.49	5.06	0.25	-1.99
Solyc06g0	5.84	6.27	6.50	15.77	15.31	16.41	0.39	-1.35
Solyc09g0	18.62	20.59	22.19	9.93	8.30	8.69	2.28	1.19
Solyc10g0	7.08	6.69	6.59	15.32	14.85	13.43	0.47	-1.10
Solyc07g0	13.97	13.09	13.80	6.54	5.46	5.86	2.29	1.19
Solyc01g1	2.91	3.16	2.76	6.27	7.08	7.30	0.43	-1.23
Solyc02g0	6.63	7.41	6.03	16.96	19.22	18.05	0.37	-1.43
Solyc02g0	27.72	30.06	27.92	13.60	15.70	12.67	2.04	1.03
Solyc03g1	0.08	0.01	0.10	1.72	1.45	1.42	0.04	-4.59
Solyc11g0	3.34	2.53	2.87	9.14	10.26	7.95	0.32	-1.65
Solyc11g0	9.47	8.67	10.09	1.21	2.11	1.60	5.73	2.52
Solyc01g0	2.11	2.33	1.86	6.72	6.59	6.14	0.32	-1.62
Solyc08g0	9.41	8.32	9.72	20.02	16.94	18.27	0.50	-1.01
Solyc09g0	2.64	3.06	2.93	0.56	0.77	0.49	4.75	2.25
Solyc07g0	1.89	2.18	1.46	6.43	6.54	5.79	0.30	-1.76
Solyc11g0	7.69	8.42	8.48	18.27	18.41	18.83	0.44	-1.17
Solyc12g0	2.76	2.32	2.76	8.66	8.25	8.51	0.31	-1.70
Solyc03g1	13.99	14.19	16.01	32.36	34.69	33.32	0.44	-1.18
Solyc04g0	4.03	3.54	4.60	11.20	11.53	12.61	0.34	-1.54
Solyc12g0	2.24	2.14	2.36	5.12	4.85	5.20	0.44	-1.17
Solyc09g0	1.87	2.11	2.20	5.04	5.25	5.24	0.40	-1.33
Solyc09g0	4.19	3.68	3.42	9.99	11.22	11.91	0.34	-1.55
Solyc04g0	5.51	6.10	6.26	14.21	14.32	14.62	0.41	-1.27
Solyc07g0	11.22	14.05	11.03	36.25	36.01	29.81	0.36	-1.49
Solyc11g0	5.33	5.90	5.89	2.45	2.45	2.30	2.38	1.25
Solyc11g0	7.26	6.79	6.92	13.96	14.59	14.38	0.49	-1.03
Solyc01g0	2.81	2.73	1.94	7.95	8.01	8.19	0.31	-1.69
Solyc06g0	16.47	15.54	14.91	7.20	7.64	6.96	2.15	1.11
Solyc12g0	4.80	4.62	4.16	9.92	10.14	11.13	0.44	-1.20
Solyc07g0	14.70	16.84	16.38	33.26	42.89	42.50	0.40	-1.31
Solyc09g0	0.48	0.53	0.57	3.04	2.98	2.73	0.18	-2.47
Solyc03g0	2.63	2.79	2.82	6.47	7.32	7.65	0.38	-1.38
Solyc05g0	1.73	1.94	1.84	4.66	4.75	5.14	0.38	-1.40
Solyc06g0	2.18	1.56	1.94	8.90	6.86	8.61	0.23	-2.10
Solyc02g0	6.75	6.25	5.87	2.93	2.59	2.81	2.27	1.18
Solyc06g0	11.71	13.78	10.97	29.58	26.95	28.13	0.43	-1.22

Solyc09g0	6.52	7.69	7.49	2.64	2.45	3.17	2.63	1.39
Solyc09g0	5.07	5.00	5.09	2.41	2.18	2.64	2.10	1.07
Solyc06g0	5.42	5.20	5.08	1.53	1.55	1.15	3.71	1.89
Solyc01g0	0.50	0.37	0.50	4.97	3.01	4.27	0.11	-3.16
Solyc02g0	0.91	1.10	1.32	3.45	3.45	3.13	0.33	-1.59
Solyc04g0	1.29	1.33	1.28	3.66	3.31	2.99	0.39	-1.35
Solyc03g0	1.40	1.36	1.29	0.15	0.07	0.05	15.05	3.91
Solyc12g0	1.25	1.17	1.17	3.94	3.84	3.52	0.32	-1.65
Solyc01g0	2.41	2.41	3.28	9.13	8.01	9.18	0.31	-1.70
Solyc01g1	10.69	9.63	9.40	3.55	3.14	3.83	2.83	1.50
Solyc12g0	7.59	7.09	8.60	2.71	3.38	3.09	2.53	1.34
Solyc12g0	6.38	6.86	6.39	13.75	13.52	13.60	0.48	-1.06
Solyc10g0	27.92	27.41	23.68	12.38	9.97	11.25	2.35	1.23
Solyc09g0	5.61	5.69	5.22	11.21	11.12	11.03	0.50	-1.01
Solyc01g0	3.23	3.74	4.34	8.71	9.74	9.36	0.41	-1.30
Solyc06g0	11.00	8.64	9.78	23.43	23.76	22.57	0.42	-1.25
Solyc12g0	4.61	4.27	4.06	9.99	8.74	10.23	0.45	-1.16
Solyc10g0	3.24	2.94	2.79	0.12	0.12	0.08	27.72	4.79
Solyc11g0	1.16	1.21	1.14	3.76	3.34	3.49	0.33	-1.60
Solyc06g0	1.53	1.64	1.55	5.38	5.53	4.95	0.30	-1.75
Solyc10g0	8.38	6.98	7.22	2.41	2.46	3.21	2.80	1.48
Solyc12g0	3.19	3.87	3.23	0.74	0.75	0.89	4.32	2.11
Solyc02g0	0.89	0.97	1.05	7.70	8.93	6.34	0.13	-2.98
Solyc11g0	0.81	0.55	0.82	4.08	4.24	4.62	0.17	-2.57
Solyc09g0	12.32	10.66	10.56	26.04	25.47	26.64	0.43	-1.22
Solyc05g0	5.54	5.76	6.91	12.06	12.58	13.87	0.47	-1.08
Solyc12g0	5.25	4.37	3.24	16.03	17.58	18.60	0.25	-2.02
Solyc09g0	3.25	3.06	2.72	6.71	6.35	6.26	0.47	-1.10
Solyc06g0	10.57	11.10	10.91	31.69	32.55	30.75	0.34	-1.54
Solyc04g0	7.95	8.07	9.22	18.71	20.27	17.15	0.45	-1.15
Solyc04g0	0.34	0.54	0.37	3.18	3.34	2.91	0.13	-2.91
Solyc12g0	1.05	1.01	0.94	3.19	2.91	3.00	0.33	-1.60
Solyc05g0	2.68	3.33	3.14	6.90	6.81	6.08	0.46	-1.11
Solyc01g0	5.87	6.15	6.67	12.57	12.28	12.68	0.50	-1.01
Solyc02g0	3.55	2.94	3.33	7.21	8.36	8.54	0.41	-1.30
Solyc06g0	5.36	4.64	4.62	13.25	11.07	12.38	0.40	-1.33
Solyc10g0	2.87	3.01	3.08	1.09	1.07	1.13	2.72	1.45
Solyc09g0	1.41	1.61	1.97	5.12	4.06	4.55	0.36	-1.46
Solyc06g0	1.26	1.08	2.22	8.14	6.42	7.59	0.21	-2.28
Solyc05g0	0.72	0.87	0.71	4.07	3.54	3.37	0.21	-2.26
Solyc06g0	0.95	1.14	1.10	5.07	4.96	4.55	0.22	-2.19
Solyc09g0	1.33	1.20	1.50	3.43	3.72	3.23	0.39	-1.37
Solyc02g0	0.75	0.80	0.86	3.89	3.54	4.09	0.21	-2.26
Solyc04g0	0.70	0.68	0.41	3.29	4.13	3.82	0.16	-2.65
Solyc06g0	1.64	2.20	1.65	5.05	6.05	4.94	0.34	-1.55
Solyc02g0	1.50	1.93	2.08	5.32	4.98	5.18	0.36	-1.49
Solyc11g0	0.17	0.22	0.25	0.02	0.02	0.03	9.10	3.19
Solyc07g0	5.78	5.40	5.74	2.85	2.64	2.59	2.10	1.07
Solyc11g0	2.47	2.48	3.16	5.50	5.67	5.70	0.48	-1.06
Solyc06g0	6.57	6.76	6.07	12.48	14.76	12.33	0.49	-1.03
Solyc09g0	4.14	3.38	3.87	8.41	8.12	7.21	0.48	-1.06

Solyc06g0	5.99	5.39	5.94	1.86	2.34	1.94	2.82	1.49
Solyc02g0	9.83	10.02	10.26	4.61	5.14	4.02	2.19	1.13
Solyc09g0	1.42	0.77	1.17	5.80	5.16	5.86	0.20	-2.32
Solyc01g0	1.91	1.51	1.84	4.42	4.12	4.26	0.41	-1.28
Solyc07g0	3.59	3.22	3.61	1.34	0.95	0.86	3.31	1.73
Solyc11g0	15.00	15.14	14.52	6.44	6.65	8.48	2.07	1.05
Solyc09g0	2.86	2.63	3.02	1.02	0.94	0.96	2.92	1.55
Solyc03g0	2.54	2.38	2.01	0.69	0.41	0.36	4.75	2.25
Solyc12g0	5.07	4.60	4.91	9.27	10.48	10.06	0.49	-1.03
Solyc09g0	0.54	0.49	0.63	3.09	2.65	2.81	0.19	-2.37
Solyc08g0	3.96	3.54	4.15	12.47	11.12	10.35	0.34	-1.54
Solyc09g0	2.73	2.87	3.30	0.63	0.90	0.77	3.87	1.95
Solyc02g0	6.35	6.32	6.72	14.37	12.88	13.51	0.48	-1.07
Solyc01g0	27.77	29.91	28.73	12.53	12.42	13.78	2.23	1.16
Solyc05g0	6.11	5.13	4.42	12.22	12.48	13.00	0.42	-1.27
Solyc01g0	6.26	6.41	5.45	13.33	13.79	13.39	0.45	-1.16
Solyc06g0	0.24	0.14	0.41	3.48	3.35	3.27	0.08	-3.69
Solyc02g0	5.74	5.68	4.93	1.30	1.63	1.30	3.87	1.95
Solyc02g0	0.48	0.56	0.47	0.01	0.02	0.01	30.37	4.92
Solyc08g0	1.40	1.66	1.69	4.30	4.27	4.22	0.37	-1.43
Solyc08g0	2.39	2.46	2.30	6.75	6.98	6.61	0.35	-1.51
Solyc11g0	2.54	2.75	2.78	5.77	5.29	5.45	0.49	-1.03
Solyc05g0	10.04	9.71	9.37	2.08	0.63	1.30	7.26	2.86
Solyc05g0	3.70	3.48	3.23	0	0	0	34721.47	15.08
Solyc06g0	4.15	4.11	4.05	1.74	1.68	1.46	2.52	1.33
Solyc01g1	1.22	1.47	1.08	4.05	3.94	4.17	0.31	-1.69
Solyc04g0	3.11	2.77	2.84	5.51	6.75	5.62	0.49	-1.04
Solyc02g0	0.74	0.55	0.58	2.35	2.27	2.74	0.25	-1.99
Solyc07g0	4.39	4.90	5.22	9.57	9.78	9.87	0.50	-1.01
Solyc11g0	1.15	1.30	1.00	3.30	3.17	3.23	0.36	-1.49
Solyc03g1	9.31	10.09	8.53	21.65	19.49	19.69	0.46	-1.12
Solyc09g0	2.51	2.80	2.46	8.19	8.40	7.31	0.32	-1.62
Solyc01g0	0.88	0.81	0.61	2.75	3.82	2.83	0.24	-2.03
Solyc01g0	0.77	1.08	0.56	3.69	3.48	3.42	0.23	-2.14
Solyc11g0	1.64	1.91	1.41	3.92	4.14	4.21	0.40	-1.31
Solyc03g0	2.05	2.07	2.01	4.86	4.74	4.54	0.43	-1.21
Solyc01g0	4.08	3.95	2.83	8.48	8.37	9.07	0.42	-1.26
Solyc11g0	4.82	5.22	3.25	16.29	14.24	16.41	0.28	-1.82
Solyc03g0	2.47	2.13	2.58	4.85	5.40	5.16	0.47	-1.10
Solyc01g1	0.52	0.43	0.38	1.76	1.54	1.91	0.26	-1.97
Solyc01g0	0.70	0.91	0.62	2.47	2.35	2.61	0.30	-1.74
Solyc04g0	5.64	6.65	5.78	12.83	12.27	12.81	0.48	-1.07
Solyc10g0	4.91	4.27	4.79	10.07	10.65	9.95	0.46	-1.13
Solyc06g0	1.27	1.56	0.74	6.05	6.59	5.05	0.20	-2.31
Solyc02g0	0.15	0.13	0.07	1.10	1.17	1.20	0.10	-3.35
Solyc02g0	7.87	7.49	7.31	14.42	14.31	16.79	0.50	-1.01
Solyc05g0	6.19	6.20	5.32	1.91	1.31	1.76	3.55	1.83
Solyc09g0	1.07	1.02	0.90	0.28	0.41	0.36	2.86	1.51
Solyc05g0	1.10	0.78	1.06	2.70	2.68	2.89	0.36	-1.49
Solyc05g0	14.43	11.86	13.76	4.17	3.04	2.72	4.03	2.01
Solyc01g0	11.47	11.64	10.66	6.19	4.35	4.28	2.28	1.19

Solyc02g0	1.46	1.56	1.38	3.55	3.43	3.77	0.41	-1.29
Solyc11g0	2.32	2.12	2.43	0	0.03	0.06	81.02	6.34
Solyc01g1	6.45	5.49	7.01	1.39	2.27	2.04	3.32	1.73
Solyc04g0	0.11	0.12	0.14	1.23	1.34	1.36	0.09	-3.42
Solyc03g0	1.23	1.41	1.58	0.41	0.38	0.36	3.70	1.89
Solyc03g0	5.48	6.25	5.63	2.32	2.17	1.95	2.70	1.43
Solyc05g0	5.82	5.24	6.56	2.13	1.28	1.45	3.62	1.86
Solyc08g0	3.26	2.74	2.73	6.34	6.13	6.14	0.47	-1.09
Solyc02g0	20.45	20.28	19.29	10.45	9.67	9.77	2.01	1.01
Solyc08g0	8.04	7.28	6.98	3.75	3.77	3.27	2.07	1.05
Solyc02g0	4.89	6.64	7.47	17.55	21.12	17.45	0.34	-1.56
Solyc04g0	1.81	2.03	2.16	4.05	4.02	4.49	0.48	-1.06
Solyc04g0	0.41	0.67	0.42	2.97	2.47	2.50	0.19	-2.40
Solyc05g0	0.39	0.57	0.48	3.18	3.22	3.25	0.15	-2.75
Solyc06g0	1.82	2.07	1.92	0.54	0.51	0.69	3.34	1.74
Solyc06g0	6.12	4.55	4.96	10.94	9.74	10.72	0.50	-1.01
Solyc01g0	2.22	2.32	1.64	5.81	5.95	5.79	0.35	-1.51
Solyc09g0	9.38	7.83	9.06	3.41	3.74	4.01	2.35	1.24
Solyc09g0	6.08	5.77	6.31	0	0.09	0.10	91.78	6.52
Solyc04g0	10.99	12.43	10.33	23.17	24.15	24.74	0.47	-1.09
Solyc09g0	3.05	3.27	3.03	1.44	1.07	0.94	2.71	1.44
Solyc02g0	2.01	1.54	1.82	4.34	4.25	4.47	0.41	-1.28
Solyc06g0	3.04	3.18	3.55	6.93	6.70	6.53	0.49	-1.04
Solyc04g0	0.82	0.91	0.78	2.30	2.85	2.64	0.32	-1.63
Solyc02g0	5.20	4.02	4.64	8.88	9.66	9.65	0.49	-1.02
Solyc07g0	4.24	4.14	4.26	0.42	0.39	0.78	7.98	3.00
Solyc06g0	3.82	3.68	4.13	1.52	1.44	1.86	2.41	1.27
Solyc05g0	2.04	1.66	1.89	4.82	4.99	4.20	0.40	-1.33
Solyc03g0	1.95	1.74	1.65	0.77	0.79	0.73	2.33	1.22
Solyc01g0	0.59	0.59	0.59	1.65	1.39	1.45	0.40	-1.34
Solyc11g0	3.46	3.28	3.96	0.76	0.98	0.94	3.98	1.99
Solyc03g0	1.90	1.41	1.67	5.75	5.78	5.67	0.29	-1.79
Solyc03g0	3.52	3.82	3.68	7.18	7.09	7.97	0.50	-1.01
Solyc08g0	0.66	0.67	0.67	1.82	1.52	1.74	0.39	-1.34
Solyc04g0	0.95	1.33	1.43	3.79	3.41	3.62	0.34	-1.54
Solyc05g0	0.07	0.15	0.12	1.21	1.18	1.21	0.09	-3.40
Solyc03g0	2.25	2.43	2.39	5.27	5.65	5.58	0.43	-1.22
Solyc01g1	1.50	1.61	1.86	4.47	4.00	3.76	0.41	-1.30
Solyc03g1	5.26	4.12	5.00	2.07	2.26	1.95	2.29	1.19
Solyc01g0	1.37	1.76	1.71	0.37	0.42	0.35	4.27	2.09
Solyc05g0	5.43	5.19	4.66	12.03	11.73	10.88	0.44	-1.18
Solyc01g0	0.96	0.70	0.88	3.29	2.66	2.70	0.29	-1.77
Solyc11g0	0.22	0.17	0.18	1.28	1.44	1.50	0.14	-2.88
Solyc10g0	1.24	1.21	1.17	3.09	3.29	3.18	0.38	-1.40
Solyc02g0	0.46	0.37	0.45	1.89	1.76	1.65	0.24	-2.05
Solyc03g0	2.32	2.15	2.28	0.81	0.90	0.77	2.72	1.44
Solyc11g0	1.40	1.11	0.97	0.18	0.13	0.24	6.34	2.66
Solyc06g0	1.49	1.65	1.35	0.27	0.20	0.33	5.58	2.48
Solyc12g0	1.39	1.41	1.59	4.51	3.99	4.03	0.35	-1.51
Solyc06g0	3.27	3.65	3.69	1.27	1.27	1.23	2.81	1.49
Solyc03g0	12.50	12.75	12.90	0	0	0	127160.37	16.96

Solyc03g0	0.17	0.24	0.24	1.75	1.55	2.07	0.12	-3.06
Solyc12g0	3.18	3.89	3.41	8.60	8.57	7.68	0.42	-1.25
Solyc03g0	0	0	0	0.87	0.76	1.25	0.00	-13.23
Solyc07g0	3.44	5.08	4.61	1.42	1.02	1.24	3.56	1.83
Solyc04g0	1.95	2.07	2.44	0.90	1.21	1.02	2.07	1.05
Solyc04g0	4.51	4.05	3.82	10.04	9.80	9.32	0.42	-1.24
Solyc08g0	2.11	2.30	2.34	6.25	5.33	5.82	0.39	-1.37
Solyc10g0	1.21	1.69	1.42	3.09	3.77	3.21	0.43	-1.22
Solyc10g0	2.75	2.90	2.53	1.09	0.82	0.90	2.92	1.54
Solyc06g0	51.55	49.90	38.98	20.82	21.87	25.74	2.05	1.04
Solyc09g0	2.65	2.07	2.26	5.60	5.59	4.82	0.44	-1.20
Solyc04g0	0.59	0.85	0.80	2.46	2.24	2.58	0.31	-1.69
Solyc06g0	6.41	9.45	9.21	16.95	16.98	16.93	0.49	-1.02
Solyc06g0	1.42	1.13	1.58	6.52	5.49	7.25	0.21	-2.22
Solyc09g0	4.86	4.57	4.95	1.67	1.42	1.46	3.16	1.66
Solyc11g0	1.31	1.51	1.08	3.18	3.53	5.04	0.33	-1.59
Solyc04g0	5.13	5.40	5.47	2.30	1.89	2.86	2.27	1.18
Solyc01g1	3.07	2.99	3.24	1.51	1.24	1.36	2.27	1.18
Solyc01g0	0.61	0.58	0.68	1.83	1.63	1.44	0.38	-1.39
Solyc03g0	3.60	3.03	3.50	1.52	1.36	1.49	2.32	1.21
Solyc09g0	22.73	22.71	17.65	7.47	8.76	8.34	2.57	1.36
Solyc12g0	2.01	1.94	2.17	4.71	5.01	4.22	0.44	-1.19
Solyc04g0	41.13	30.32	36.01	12.48	13.79	16.65	2.50	1.32
Solyc04g0	2.82	3.02	2.74	1.10	1.15	1.49	2.30	1.20
Solyc05g0	1.41	1.58	1.54	3.58	3.11	3.14	0.46	-1.12
Solyc10g0	4.48	4.16	4.46	8.93	7.84	9.46	0.50	-1.00
Solyc07g0	3.11	3.05	2.90	1.44	1.31	1.30	2.24	1.16
Solyc10g0	6.66	5.53	5.98	11.27	12.41	13.14	0.49	-1.02
Solyc03g0	0.50	0.39	0.37	1.63	1.29	1.34	0.29	-1.76
Solyc04g0	0.21	0.27	0.27	1.21	1.37	1.37	0.19	-2.40
Solyc01g0	1.97	2.00	2.33	1.03	1.01	0.92	2.13	1.09
Solyc09g0	0	0	0	4.82	4.69	3.65	0.00	-15.42
Solyc02g0	1.46	0.92	1.23	3.89	3.98	3.11	0.33	-1.60
Solyc09g0	0.57	0.61	0.62	2.14	1.47	1.51	0.35	-1.51
Solyc11g0	5.40	6.24	6.58	2.76	2.76	2.88	2.17	1.12
Solyc05g0	14.41	14.38	17.34	7.09	7.19	7.92	2.08	1.06
Solyc04g0	1.45	1.15	1.24	2.78	3.19	3.39	0.41	-1.29
Solyc06g0	0.63	0.50	0.54	1.48	1.46	1.18	0.40	-1.31
Solyc03g0	2.06	1.86	1.58	0.37	0.42	0.51	4.24	2.08
Solyc08g0	2.44	2.22	2.73	4.68	5.65	5.43	0.47	-1.09
Solyc01g0	17.42	15.24	15.98	6.50	4.93	6.99	2.64	1.40
Solyc06g0	0.84	0.76	1.14	2.76	2.99	2.89	0.32	-1.65
Solyc02g0	0.09	0	0.10	2.04	3.49	2.65	0.02	-5.49
Solyc06g0	0.05	0.14	0.16	1.71	1.65	1.88	0.07	-3.94
Solyc03g1	3.45	3.74	3.33	1.71	1.35	1.25	2.45	1.29
Solyc06g0	5.25	4.28	4.69	2.18	2.53	2.28	2.04	1.03
Solyc05g0	0.37	0.37	0.59	1.75	1.71	1.56	0.26	-1.92
Solyc11g0	3.13	2.96	2.53	5.49	6.66	6.08	0.47	-1.08
Solyc03g1	2.50	2.74	2.55	4.73	6.51	5.74	0.46	-1.12
Solyc01g0	3.11	2.65	3.41	6.46	7.03	7.75	0.43	-1.21
Solyc01g1	1.30	1.18	1.16	3.54	3.72	3.16	0.35	-1.52

Solyc02g0	0.07	0.08	0	0.88	0.92	0.83	0.06	-4.14
Solyc01g0	0.57	0.63	0.63	0.15	0.18	0.12	4.01	2.00
Solyc04g0	3.64	3.47	3.38	7.32	6.53	7.77	0.49	-1.04
Solyc04g0	0.73	0.66	1.10	3.91	3.19	3.58	0.23	-2.11
Solyc09g0	4.10	4.70	4.23	9.13	10.24	8.22	0.47	-1.08
Solyc03g0	1.68	1.66	1.67	0.70	0.64	0.59	2.59	1.37
Solyc04g0	0.37	0.63	0.45	1.97	2.52	2.32	0.21	-2.22
Solyc11g0	0.90	1.28	1.25	0.10	0.11	0.10	10.65	3.41
Solyc07g0	0.86	1.32	1.09	3.63	3.78	3.42	0.30	-1.73
Solyc10g0	0.94	1.41	1.44	3.65	5.67	4.67	0.27	-1.88
Solyc01g0	0.09	0.20	0.21	1.74	1.53	1.10	0.11	-3.13
Solyc09g0	0.63	0.95	0.95	2.24	2.01	2.21	0.39	-1.35
Solyc05g0	0.52	0.73	0.43	1.89	2.47	2.16	0.26	-1.96
Solyc02g0	1.40	1.07	1.47	0.15	0.09	0.10	11.64	3.54
Solyc05g0	1.25	1.83	1.56	3.61	4.05	4.72	0.38	-1.41
Solyc06g0	2.84	2.78	2.45	6.76	6.53	6.36	0.41	-1.28
Solyc03g0	0	0	0	2.08	2.41	1.82	0.00	-14.36
Solyc08g0	1.30	1.87	1.08	5.54	4.81	7.13	0.24	-2.04
Solyc08g0	1.80	1.83	2.05	4.06	3.76	3.91	0.48	-1.04
Solyc05g0	0.38	0.41	0.45	1.15	1.17	1.42	0.33	-1.59
Solyc07g0	1.80	2.23	2.45	4.44	4.70	4.79	0.47	-1.10
Solyc06g0	0.27	0.30	0.37	1.52	1.79	1.80	0.18	-2.46
Solyc01g0	0.65	0.54	0.64	0.14	0.06	0.10	6.15	2.62
Solyc08g0	3.28	3.07	2.59	5.54	6.95	6.19	0.48	-1.06
Solyc11g0	1.42	1.41	1.36	2.55	3.00	3.46	0.46	-1.11
Solyc02g0	0.28	0.37	0.35	0.89	1.00	1.08	0.34	-1.57
Solyc06g0	1.42	2.47	1.64	5.11	5.73	5.79	0.33	-1.59
Solyc09g0	4.08	3.45	3.44	8.24	7.19	7.62	0.48	-1.07
Solyc02g0	1.77	1.73	1.51	4.12	3.76	3.92	0.43	-1.23
Solyc01g0	2.04	2.32	2.13	4.98	4.16	5.57	0.44	-1.18
Solyc11g0	4.22	4.85	3.76	2.14	1.81	1.96	2.17	1.12
Solyc05g0	2.37	2.09	2.38	5.29	5.69	6.02	0.40	-1.31
Solyc02g0	0.96	0.60	0.90	0.10	0.17	0.12	6.42	2.68
Solyc01g1	5.87	4.99	5.93	0	0.06	0.08	122.80	6.94
Solyc05g0	0.94	0.60	0.98	2.73	3.06	2.31	0.31	-1.69
Solyc05g0	0.58	0.80	0.66	2.20	1.89	2.31	0.32	-1.65
Solyc05g0	1.80	2.05	2.16	5.54	4.77	5.97	0.37	-1.44
Solyc02g0	2.63	2.55	2.39	4.80	4.87	5.82	0.49	-1.03
Solyc06g0	0.65	0.53	0.97	0.12	0.12	0.10	6.40	2.68
Solyc12g0	0.43	0.26	0.66	0.03	0.06	0.04	10.12	3.34
Solyc04g0	1.04	1.03	1.05	3.71	4.60	4.88	0.24	-2.08
Solyc12g0	1.50	1.39	1.65	3.38	3.35	3.71	0.43	-1.20
Solyc08g0	0.77	0.82	0.69	2.13	2.16	2.09	0.36	-1.48
Solyc03g1	3.18	3.53	3.40	0.70	0.48	0.48	6.09	2.61
Solyc02g0	0.06	0.05	0.09	0.51	0.44	0.64	0.12	-3.00
Solyc01g0	3.34	2.83	3.03	7.37	6.96	6.12	0.45	-1.15
Solyc01g0	2.08	1.93	1.81	0.77	0.42	0.68	3.11	1.64
Solyc01g0	1.68	1.66	2.52	4.99	6.68	5.48	0.34	-1.55
Solyc01g0	1.24	1.80	1.46	4.01	4.60	3.79	0.36	-1.46
Solyc02g0	0.69	0.80	0.84	2.72	2.13	1.71	0.35	-1.49
Solyc07g0	0.30	0.21	0.26	1.10	0.77	1.17	0.25	-1.97

Solyc03g0	8.87	9.17	9.02	4.75	4.47	4.30	2.00	1.00
Solyc06g0	2.01	1.84	2.04	0.93	0.86	1.00	2.11	1.07
Solyc07g0	0	0	0	3.87	3.77	3.52	0.00	-15.18
Solyc03g0	2.86	3.10	3.58	6.45	7.35	7.66	0.44	-1.17
Solyc02g0	1.12	1.51	1.15	0.19	0.15	0.30	5.84	2.55
Solyc09g0	12.09	8.46	8.84	3.51	2.96	3.72	2.89	1.53
Solyc04g0	0.73	0.94	0.83	0.16	0.24	0.18	4.34	2.12
Solyc04g0	4.05	4.12	4.30	8.58	10.82	10.15	0.42	-1.25
Solyc08g0	0.15	0.11	0.05	1.05	1.02	0.88	0.11	-3.24
Solyc09g0	0.06	0.16	0.12	0.70	1.10	0.89	0.13	-2.95
Solyc04g0	0.62	0.58	0.48	1.49	1.50	1.05	0.42	-1.26
Solyc03g1	5.53	4.27	5.68	0.93	0.70	0.91	6.09	2.61
Solyc02g0	32.87	29.19	25.08	13.57	15.10	13.54	2.06	1.05
Solyc08g0	1.59	2.36	2.09	0.85	0.76	0.84	2.48	1.31
Solyc04g0	0.31	0.40	0.38	1.48	2.13	2.65	0.17	-2.52
Solyc12g0	1.62	1.41	1.69	3.42	3.61	2.74	0.48	-1.05
Solyc03g1	1.17	1.15	0.93	2.72	3.28	2.57	0.38	-1.40
Solyc07g0	0.87	0.97	0.93	3.56	3.40	3.83	0.26	-1.96
Solyc03g0	0.41	0.28	0.48	1.68	1.34	1.11	0.28	-1.83
Solyc09g0	0.82	1.43	1.00	0.27	0.36	0.15	4.18	2.06
Solyc07g0	0	0.03	0	0.50	0.48	0.41	0.02	-5.66
Solyc12g0	0.49	0.64	0.71	0.09	0.07	0.17	5.61	2.49
Solyc01g0	0.13	0.20	0.11	0.83	0.99	0.70	0.18	-2.51
Solyc11g0	0.56	0.74	0.50	0	0	0	5992.64	12.55
Solyc06g0	0.67	0.81	0.91	0.16	0.25	0.23	3.73	1.90
Solyc01g1	0.57	0.31	0.48	2.28	1.98	1.84	0.22	-2.16
Solyc04g0	2.11	2.02	1.88	4.57	3.82	4.26	0.48	-1.07
Solyc05g0	23.54	32.88	25.15	13.40	11.60	14.24	2.08	1.06
Solyc11g0	3.50	3.88	4.74	1.63	1.15	1.65	2.74	1.45
Solyc11g0	0.14	0.28	0.22	0.91	0.98	0.92	0.23	-2.13
Solyc03g0	0.76	0.67	0.58	0.03	0	0.03	36.58	5.19
Solyc01g1	1.95	1.98	2.53	4.91	4.54	5.84	0.42	-1.24
Solyc02g0	4.44	3.51	3.19	10.54	9.25	10.24	0.37	-1.43
Solyc02g0	0.22	0.58	0.36	2.50	3.19	2.63	0.14	-2.83
Solyc02g0	1.96	1.50	1.91	0.44	0.54	0.36	3.98	1.99
Solyc12g0	0.18	0.16	0.12	1.17	1.43	1.01	0.13	-2.97
Solyc01g0	15.38	15.16	15.64	5.30	5.97	8.16	2.38	1.25
Solyc03g0	2.18	2.14	2.38	0.90	0.71	1.09	2.49	1.31
Solyc06g0	6.23	5.12	5.53	2.88	3.18	2.35	2.01	1.01
Solyc01g1	0.99	0.87	1.05	0.45	0.49	0.44	2.11	1.08
Solyc10g0	0.03	0	0.06	0.54	0.49	0.38	0.06	-4.05
Solyc01g0	2.35	2.07	2.38	0.95	1.24	0.93	2.19	1.13
Solyc01g0	7.49	7.13	7.30	17.41	16.69	18.97	0.41	-1.28
Solyc09g0	1.88	1.55	1.29	5.12	4.45	4.70	0.33	-1.60
Solyc04g0	0.10	0.07	0.08	0.29	0.57	0.61	0.17	-2.57
Solyc02g0	2.72	2.02	2.84	0.84	1.07	0.70	2.89	1.53
Solyc02g0	0.50	0.66	0.40	0.04	0	0	40.96	5.36
Solyc08g0	2.01	2.27	2.46	4.13	4.87	5.27	0.47	-1.08
Solyc02g0	0.02	0.09	0.03	0.55	0.41	0.38	0.10	-3.33
Solyc08g0	0.96	1.04	0.87	2.29	1.74	2.04	0.47	-1.07
Solyc09g0	0.48	0.49	0.62	1.27	1.31	1.07	0.44	-1.19

Solyc06g0	1.38	1.13	1.03	2.41	2.31	2.83	0.47	-1.09
Solyc01g1	11.55	11.06	11.66	4.63	5.79	5.36	2.17	1.12
Solyc11g0	0.84	0.61	0.72	0.19	0.20	0.20	3.66	1.87
Solyc11g0	5.57	6.48	6.97	3.18	1.98	2.28	2.55	1.35
Solyc01g0	0	0	0	0.46	0.43	0.43	0.00	-12.11
Solyc05g0	2.74	2.15	2.59	0.90	1.18	1.15	2.31	1.21
Solyc01g0	1.42	1.42	1.05	2.61	3.02	2.23	0.50	-1.01
Solyc03g0	1.22	1.01	1.09	1.96	2.57	2.38	0.48	-1.05
Solyc10g0	1.34	0.79	0.68	3.20	3.43	2.47	0.31	-1.69
Solyc02g0	0.84	1.29	0.64	2.49	2.55	2.46	0.37	-1.44
Solyc09g0	5.05	3.44	5.41	15.82	13.18	10.37	0.35	-1.50
Solyc05g0	0.49	0.47	0.30	0.04	0.04	0.02	12.80	3.68
Solyc06g0	3.07	3.17	3.75	8.11	10.36	7.98	0.38	-1.40
Solyc04g0	2.28	2.80	2.96	4.70	6.47	6.10	0.46	-1.11
Solyc01g1	0.95	1.11	1.28	0.18	0.17	0.13	7.06	2.82
Solyc02g0	1.08	1.56	1.03	2.73	3.52	4.07	0.36	-1.49
Solyc11g0	1.61	1.48	1.51	3.61	3.34	2.93	0.47	-1.10
Solyc11g0	1.75	1.65	1.77	3.24	3.83	3.68	0.48	-1.05
Solyc10g0	0	0	0	0.86	0.78	0.93	0.00	-13.06
Solyc03g0	1.10	1.03	0.91	0.50	0.41	0.41	2.30	1.20
Solyc05g0	9.61	9.19	9.88	3.54	2.25	3.44	3.11	1.64
Solyc03g0	1.51	1.28	1.87	3.44	3.55	3.39	0.45	-1.15
Solyc01g1	4.74	4.30	4.49	1.67	2.06	1.61	2.53	1.34
Solyc10g0	1.68	1.35	1.49	0.58	0.64	0.74	2.30	1.20
Solyc01g1	1.05	1.13	0.86	2.40	2.60	1.95	0.44	-1.20
Solyc01g1	0.14	0.36	0.21	1.18	1.48	0.96	0.20	-2.34
Solyc05g0	2.58	2.42	3.11	1.20	0.91	1.07	2.55	1.35
Solyc12g0	0.26	0.31	0.36	0.93	0.96	0.90	0.33	-1.58
Solyc01g0	16.16	21.69	15.65	8.27	5.28	8.88	2.38	1.25
Solyc01g0	0.66	0.37	0.32	2.16	2.07	1.34	0.24	-2.04
Solyc11g0	1.94	1.73	1.80	0	0	0	18258.90	14.16
Solyc02g0	0	0	0	0.23	0.38	0.44	0.00	-11.78
Solyc12g0	1.70	1.46	1.29	3.07	3.06	2.93	0.49	-1.03
Solyc08g0	2.93	2.99	3.35	7.50	6.51	5.93	0.47	-1.10
Solyc12g0	0.50	0.75	0.62	1.70	1.85	1.73	0.35	-1.50
Solyc03g1	0.98	1.00	1.07	2.25	2.15	2.04	0.47	-1.08
Solyc03g0	1.27	1.31	1.86	3.41	3.78	3.93	0.40	-1.33
Solyc11g0	1.41	1.18	0.81	3.42	2.69	3.98	0.34	-1.57
Solyc06g0	3.70	3.56	4.05	8.49	8.62	7.41	0.46	-1.12
Solyc02g0	2.34	2.71	3.58	0	0	0	28763.13	14.81
Solyc11g0	4.43	4.06	2.94	0.96	0.51	1.26	4.18	2.06
Solyc02g0	0	0	0	0	0	12.52	0.00	-15.35
Solyc06g0	1.40	1.54	1.89	0.60	0.69	0.50	2.69	1.43
Solyc11g0	14.35	13.19	11.42	5.20	4.66	5.98	2.46	1.30
Solyc02g0	0.33	0.05	0.21	1.34	1.45	1.37	0.14	-2.83
Solyc12g0	0.20	0.25	0.18	0	0	0	2112.44	11.04
Solyc02g0	0.23	0.20	0.20	0.93	1.08	0.75	0.23	-2.14
Solyc06g0	0	0.09	0.09	0.55	1.00	0.80	0.07	-3.75
Solyc06g0	2.48	2.44	2.49	1.09	1.31	1.31	2.00	1.00
Solyc09g0	0	0.06	0	8.14	7.57	7.88	0.00	-8.65
Solyc04g0	3.21	3.93	2.72	1.63	1.67	1.59	2.02	1.01

Solyc05g0	0.87	0.89	0.92	1.99	1.56	1.96	0.49	-1.03
Solyc10g0	0.27	0.35	0.66	2.25	3.41	3.78	0.14	-2.88
Solyc05g0	0.26	0.29	0.14	0.88	0.60	0.73	0.31	-1.67
Solyc12g0	1.63	1.40	1.47	0.41	0.42	0.61	3.12	1.64
Solyc07g0	0.46	0.98	0.59	0	0	0	6759.25	12.72
Solyc03g0	2.29	2.21	3.17	0	0	0	25578.61	14.64
Solyc01g1	1.52	1.81	1.57	0.54	0.46	0.36	3.62	1.85
Solyc05g0	2.47	1.99	1.90	0.75	0.55	0.83	2.98	1.58
Solyc08g0	3.47	3.57	3.67	6.95	7.59	7.88	0.48	-1.06
Solyc03g0	0.49	0.50	0.61	0.15	0.18	0.13	3.51	1.81
Solyc03g1	0	0	0	0.59	0.34	0.50	0.00	-12.22
Solyc09g0	0.03	0	0.05	0.37	0.59	0.59	0.06	-4.18
Solyc01g0	0.36	0.33	0.36	0.92	1.32	1.03	0.32	-1.64
Solyc02g0	0	0.02	0	0.20	0.34	0.20	0.02	-5.39
Solyc02g0	0.53	0.38	0.78	2.34	3.58	3.04	0.19	-2.41
Solyc01g0	0.54	0.44	0.64	1.34	1.37	1.20	0.42	-1.26
Solyc12g0	8.36	8.69	6.56	2.89	3.21	2.59	2.72	1.44
Solyc03g0	1.12	1.11	0.96	2.39	2.28	2.04	0.48	-1.07
Solyc01g0	10.93	12.71	9.62	4.47	4.89	4.57	2.39	1.25
Solyc11g0	0.74	0.76	0.60	0.33	0.31	0.24	2.41	1.27
Solyc10g0	0.22	0.63	0.64	2.45	1.53	2.41	0.23	-2.10
Solyc04g0	0.15	0.20	0.15	0.89	0.93	0.62	0.21	-2.26
Solyc07g0	0.36	0.23	0.17	1.09	1.17	0.96	0.24	-2.07
Solyc11g0	1.69	1.97	0.99	0.60	0.49	0.63	2.71	1.44
Solyc06g0	2.17	3.17	2.39	4.71	5.76	5.09	0.50	-1.01
Solyc06g0	7.00	7.85	6.56	2.96	3.80	3.01	2.19	1.13
Solyc01g0	1.04	0.97	1.02	0.31	0.40	0.43	2.66	1.41
Solyc02g0	0.56	0.49	0.41	1.06	1.12	1.30	0.42	-1.24
Solyc06g0	0.58	0.56	0.56	1.76	2.94	2.44	0.24	-2.07
Solyc02g0	0.94	0.88	0.73	0.06	0.05	0.17	9.14	3.19
Solyc04g0	0.45	0.50	0.48	1.02	0.99	0.98	0.48	-1.07
Solyc10g0	0.69	0.99	0.78	2.14	2.30	2.19	0.37	-1.43
Solyc06g0	1.10	1.10	0.86	0.24	0.30	0.16	4.39	2.14
Solyc08g0	0.66	0.57	0.45	1.45	1.79	1.23	0.38	-1.41
Solyc02g0	0	0	0	0	12.50	0	0.00	-15.35
Solyc05g0	1.23	1.47	1.35	2.49	2.84	3.03	0.48	-1.04
Solyc06g0	1.02	0.85	0.53	2.28	2.53	2.43	0.33	-1.60
Solyc12g0	0.63	0.71	0.73	1.46	1.59	1.21	0.49	-1.04
Solyc05g0	0.24	0.52	0.48	1.48	2.01	1.69	0.24	-2.05
Solyc06g0	1.17	0.96	0.93	3.17	2.21	2.48	0.39	-1.36
Solyc08g0	0	0	0	0.37	0.28	0.50	0.00	-11.90
Solyc02g0	1.51	1.05	2.23	0.26	0.27	0.41	5.07	2.34
Solyc09g0	0	0	0	0.34	0.37	0.39	0.00	-11.83
Solyc09g0	4.68	5.16	4.53	9.35	10.95	11.87	0.45	-1.16
Solyc09g0	0	0	0	0.52	0.17	0.45	0.00	-11.89
Solyc06g0	0.47	0.46	0.38	1.12	1.64	1.26	0.33	-1.61
Solyc12g0	1.26	1.23	1.28	0.46	0.52	0.54	2.48	1.31
Solyc08g0	0.43	0.97	0.74	2.36	2.68	2.57	0.28	-1.83
Solyc03g1	5.05	3.01	3.15	11.49	10.33	10.34	0.35	-1.52
Solyc11g0	0.30	0.37	0.33	1.02	0.88	1.16	0.33	-1.61
Solyc05g0	0.41	0.81	0.42	1.96	2.45	2.25	0.25	-2.02

Solyc02g0	0.24	0.03	0.48	3.21	3.93	2.21	0.08	-3.65
Solyc03g0	0	0	0	1.15	0	0	0.00	-11.91
Solyc06g0	0.02	0.03	0.03	0.17	0.17	0.19	0.15	-2.69
Solyc03g1	0	0	0	1.04	0	0	0.00	-11.76
Solyc09g0	1.61	1.74	1.59	0.39	0.24	0.43	4.66	2.22
Solyc03g0	0	0.13	0.30	1.96	1.68	1.97	0.08	-3.70
Solyc10g0	0	0	0	0.82	0.71	0.26	0.00	-12.54
Solyc04g0	0.81	0.53	0.94	2.50	2.92	2.52	0.29	-1.80
Solyc01g0	0.31	0.65	0.45	1.31	1.36	1.40	0.35	-1.53
Solyc02g0	3.08	3.63	3.53	1.51	1.38	1.61	2.27	1.18
Solyc02g0	0.27	0.23	0.14	0	0	0	2133.31	11.06
Solyc03g1	0	0	0.01	0.25	0.19	0.13	0.03	-5.30
Solyc05g0	0	0	0	0.22	0.30	0.22	0.00	-11.28
Solyc01g0	1.37	0.08	0.72	0	0	0	7223.16	12.82
Solyc01g0	0.16	0.16	0.13	0	0	0	1525.65	10.58
Solyc12g0	0	0	0	0.18	0.26	0.17	0.00	-11.01
Solyc05g0	0.46	0.43	0.39	0	0	0	4257.93	12.06
Solyc05g0	0.23	0.20	0.25	0	0	0	2266.47	11.15
Solyc02g0	1.49	0	0	0	0	0	4951.62	12.27
Solyc01g0	0	0.09	0.16	3.50	3.57	4.02	0.02	-5.46
Solyc07g0	0.72	0.85	1.00	0.18	0.28	0.10	4.55	2.19
Solyc11g0	1.75	1.69	1.91	5.27	6.45	6.60	0.29	-1.77
Solyc02g0	0.25	0.55	0.46	1.43	1.62	1.68	0.27	-1.90
Solyc10g0	1.20	0.69	0.93	2.28	2.39	2.15	0.41	-1.27
Solyc12g0	0.13	0	0.03	1.24	0.74	0.59	0.06	-4.02
Solyc02g0	1.66	1.28	2.14	4.02	3.46	3.54	0.46	-1.12
Solyc03g1	0	0	0	0.49	1.05	0.66	0.00	-12.84
Solyc09g0	0	0	0	0.24	0.52	0.65	0.00	-12.20
Solyc03g0	1.33	1.30	1.22	0	0.07	0.02	39.98	5.32
Solyc02g0	1.14	1.29	1.42	2.76	2.97	3.23	0.43	-1.22
Solyc07g0	0.75	0.86	0.89	0.26	0.31	0.17	3.41	1.77
Solyc04g0	0.14	0.20	0.21	0.56	0.83	0.65	0.27	-1.91
Solyc05g0	0.32	0.30	0.30	1.05	0.95	0.69	0.34	-1.56
Solyc09g0	2.94	3.49	3.97	1.12	1.52	0.84	2.99	1.58
Solyc11g0	1.92	1.82	1.53	4.62	3.84	3.66	0.43	-1.20
Solyc03g0	0.62	0.64	0.81	0	0	0	6906.40	12.75
Solyc04g0	0.78	0.89	0.64	0.32	0.34	0.29	2.43	1.28
Solyc05g0	0.60	1.04	0.79	3.56	2.79	2.00	0.29	-1.78
Solyc03g1	2.01	2.15	1.90	4.76	4.38	4.08	0.46	-1.12
Solyc05g0	0.28	0.30	0.34	0.64	0.75	0.85	0.41	-1.29
Solyc02g0	0.41	0.27	0.35	0.05	0	0.01	18.22	4.19
Solyc05g0	1.17	0.88	0.70	0	0.19	0.05	11.71	3.55
Solyc08g0	1.06	1.01	1.05	2.73	2.21	2.20	0.44	-1.20
Solyc11g0	3.21	0	0	0	0	0	10683.60	13.38
Solyc03g1	0	0	12.01	0	0	0	40017.21	15.29
Solyc07g0	0.03	0	0.09	0.49	0.47	0.70	0.07	-3.80
Solyc12g0	1.35	1.14	1.36	0.54	0.33	0.44	2.94	1.56
Solyc09g0	1.20	0.62	0.84	2.73	3.40	3.54	0.28	-1.86
Solyc03g1	1.75	1.87	1.92	0.91	0.76	0.94	2.13	1.09
Solyc01g0	0.20	0.36	0.18	0.59	0.72	0.81	0.35	-1.51
Solyc02g0	0	0	0	0.22	0.21	0.25	0.00	-11.14

Solyc03g0	0	0	0	0.26	0.34	0.12	0.00	-11.21
Solyc05g0	0	0.09	0	4.28	3.89	3.62	0.01	-6.96
Solyc04g0	0.76	0.97	0.62	2.10	3.34	2.42	0.30	-1.74
Solyc10g0	0.77	0.89	1.46	2.90	3.16	3.62	0.32	-1.63
Solyc06g0	0.20	0.11	0.12	0.59	0.59	0.48	0.26	-1.93
Solyc02g0	0.15	0.12	0.18	0.44	0.76	0.73	0.23	-2.10
Solyc07g0	0.43	0.59	0.63	0.25	0.24	0.26	2.22	1.15
Solyc09g0	0	0	0	0.50	0.44	0.51	0.00	-12.24
Solyc11g0	1.64	1.64	1.77	0.72	0.73	0.65	2.41	1.27
Solyc02g0	0.95	0.91	1.09	0.45	0.31	0.30	2.79	1.48
Solyc04g0	0.10	0.29	0.21	1.04	1.33	1.03	0.18	-2.51
Solyc11g0	1.16	1.60	1.49	3.37	3.50	2.41	0.46	-1.12
Solyc09g0	1.94	1.69	1.77	0.66	0.88	0.86	2.25	1.17
Solyc10g0	0.27	0.26	0.25	0.76	0.83	0.85	0.32	-1.64
Solyc08g0	0	0	0	0.58	0.41	0.57	0.00	-12.35
Solyc01g0	2.43	3.44	3.06	1.15	1.52	1.26	2.27	1.18
Solyc09g0	0.82	0.79	0.78	0.29	0.44	0.27	2.38	1.25
Solyc05g0	1.80	1.27	0.88	3.20	4.99	4.38	0.31	-1.67
Solyc01g1	1.88	1.77	2.09	0.64	0.72	1.02	2.41	1.27
Solyc04g0	0.65	0.73	0.68	0.24	0.24	0.21	3.02	1.59
Solyc06g0	3.03	3.02	2.95	8.15	6.77	4.84	0.46	-1.13
Solyc02g0	0.72	0.49	0.54	1.15	1.40	1.31	0.45	-1.14
Solyc03g0	0.44	0.45	0.47	1.18	1.21	1.03	0.40	-1.33
Solyc10g0	0	0	0	0.41	0.18	0.38	0.00	-11.65
Solyc02g0	4.47	4.29	5.19	0.54	0.44	0.62	8.70	3.12
Solyc01g1	0.06	0.08	0.07	0.36	0.29	0.39	0.20	-2.33
Solyc07g0	0.99	1.54	1.87	3.30	3.16	3.42	0.45	-1.16
Solyc11g0	0	0	0	0.19	0.13	0.22	0.00	-10.82
Solyc05g0	0.11	0.12	0	1.45	1.21	1.66	0.05	-4.21
Solyc09g0	0.79	0.55	0.78	0.12	0.19	0.21	4.04	2.01
Solyc03g1	0.59	0.51	0.40	1.31	1.14	1.26	0.40	-1.31
Solyc04g0	1.73	2.84	1.98	4.97	5.92	4.66	0.42	-1.25
Solyc02g0	0.21	0.06	0.27	1.06	1.06	0.78	0.19	-2.42
Solyc01g0	0	0	0	0.11	0.39	0.30	0.00	-11.38
Solyc06g0	0.57	0.59	0.55	0.21	0.21	0.29	2.40	1.26
Solyc09g0	0.51	0.87	0.84	2.70	2.26	1.55	0.34	-1.55
Solyc05g0	0.49	0.37	0.40	0.16	0.09	0.12	3.41	1.77
Solyc04g0	0.07	0.07	0.04	0.31	0.28	0.52	0.16	-2.68
Solyc06g0	0.21	0.30	0.25	1.00	0.91	0.89	0.27	-1.88
Solyc01g0	2.58	2.98	2.69	0.98	1.52	1.37	2.13	1.09
Solyc09g0	1.04	0.56	0.93	3.01	2.44	1.99	0.34	-1.56
Solyc01g1	0.65	0.63	0.81	0.28	0.32	0.26	2.42	1.28
Solyc06g0	0.05	0.05	0	0.61	0.25	0.31	0.08	-3.58
Solyc10g0	2.28	2.86	2.55	5.55	6.54	4.64	0.46	-1.12
Solyc05g0	1.48	1.54	0.98	2.90	2.47	2.81	0.49	-1.03
Solyc01g0	0.46	0.45	0.27	1.00	1.22	0.94	0.37	-1.43
Solyc01g1	5.24	5.74	5.58	2.70	2.20	2.99	2.10	1.07
Solyc07g0	0.69	0.75	0.49	1.32	1.32	1.52	0.46	-1.11
Solyc05g0	0.72	0.83	0.55	0.26	0.23	0.37	2.46	1.30
Solyc05g0	7.52	8.08	6.50	3.59	2.19	2.32	2.73	1.45
Solyc01g0	0	0	0	0.19	0.11	0.10	0.00	-10.39

Solyc01g0	0.22	0.22	0.18	0	0	0	2099.54	11.04
Solyc01g0	0.95	0.69	0.35	0.14	0.12	0.15	4.89	2.29
Solyc06g0	0.76	0.72	0.72	0.18	0.27	0.06	4.37	2.13
Solyc08g0	1.45	1.58	1.82	0.90	0.77	0.68	2.07	1.05
Solyc02g0	0.54	0.74	0.68	1.52	1.91	2.26	0.34	-1.54
Solyc12g0	0.37	0.28	0.31	1.20	0.96	1.05	0.30	-1.74
Solyc06g0	0.06	0.13	0.07	0.38	0.62	0.45	0.18	-2.48
Solyc02g0	0.74	0.51	0.77	1.97	1.88	1.77	0.36	-1.48
Solyc01g0	4.83	4.32	3.52	2.33	1.84	1.93	2.08	1.05
Solyc02g0	0.25	0.35	0.30	0.04	0.03	0.03	8.65	3.11
Solyc02g0	0.41	0.45	0.32	0.96	0.82	0.77	0.46	-1.11
Solyc08g0	1.19	1.24	1.42	0.63	0.60	0.58	2.14	1.10
Solyc08g0	0	0	0	0.17	0.23	0.21	0.00	-10.99
Solyc09g0	3.52	4.23	3.48	8.46	7.84	7.21	0.48	-1.07
Solyc01g0	1.33	1.55	1.14	0.44	0.33	0.62	2.88	1.52
Solyc02g0	0.16	0.16	0.22	0.79	0.49	0.64	0.28	-1.85
Solyc09g0	0.51	1.21	0.79	0.08	0	0	33.35	5.06
Solyc01g0	2.86	3.53	3.76	0	0.05	0	208.08	7.70
Solyc08g0	0	0	0.18	1.21	0.89	1.69	0.05	-4.43
Solyc02g0	1.22	1.16	0.90	0.44	0.41	0.36	2.73	1.45
Solyc03g1	0.17	0.40	0.17	0	0	0	2479.17	11.28
Solyc12g0	1.30	0.98	1.02	3.29	3.11	3.50	0.33	-1.58
Solyc01g0	0.90	0.86	1.23	1.83	2.33	1.85	0.50	-1.01
Solyc05g0	0	0	0	0.18	0.12	0.07	0.00	-10.27
Solyc08g0	0.03	0.01	0.02	0.09	0.11	0.15	0.20	-2.32
Solyc07g0	1.25	0.96	1.03	0.54	0.42	0.49	2.25	1.17
Solyc09g0	2.41	1.32	1.66	0.51	0.59	0.49	3.39	1.76
Solyc06g0	0.54	0.74	1.12	2.13	2.17	1.83	0.39	-1.35
Solyc06g0	0.51	0.29	0.50	0.90	0.90	1.01	0.46	-1.12
Solyc04g0	0.09	0.07	0.06	0.21	0.25	0.18	0.35	-1.51
Solyc05g0	0.72	0.82	1.08	2.01	2.13	1.99	0.43	-1.23
Solyc03g0	0.07	0.02	0.06	1.57	0.38	0.76	0.06	-4.13
Solyc06g0	2.13	2.15	1.61	3.70	4.15	4.09	0.49	-1.02
Solyc02g0	1.30	1.38	1.21	0.55	0.81	0.41	2.20	1.14
Solyc06g0	5.67	4.08	3.97	13.55	10.95	10.35	0.39	-1.34
Solyc05g0	3.76	3.55	2.79	1.67	1.84	1.47	2.03	1.02
Solyc09g0	0.45	0.85	0.72	0.14	0	0.10	8.52	3.09
Solyc02g0	2.25	1.91	1.88	0.66	0.58	0.77	3.01	1.59
Solyc10g0	1.80	1.28	1.02	2.98	3.04	2.91	0.46	-1.12
Solyc11g0	1.59	2.05	1.77	4.24	3.27	4.34	0.46	-1.13
Solyc12g0	0	0	0	0.58	0.66	0.68	0.00	-12.65
Solyc10g0	2.43	3.70	4.06	7.91	6.85	6.09	0.49	-1.03
Solyc03g0	0.11	0.20	0.10	0.57	0.48	0.40	0.28	-1.84
Solyc07g0	4.80	4.42	4.13	9.58	8.73	9.20	0.49	-1.04
Solyc03g1	1.25	2.04	1.52	3.79	4.43	3.03	0.43	-1.23
Solyc01g1	1.77	1.45	1.14	0.51	0.33	0.38	3.58	1.84
Solyc12g0	6.63	5.64	5.20	1.65	3.10	2.04	2.57	1.36
Solyc08g0	0	0.01	0	0.08	0.13	0.10	0.05	-4.43
Solyc08g0	0.65	0.67	0.72	1.64	1.38	1.19	0.48	-1.05
Solyc05g0	0.70	0.51	0.37	0.03	0	0	49.27	5.62
Solyc11g0	1.74	1.33	2.46	3.86	4.23	3.22	0.49	-1.04

Solyc08g0	0.30	0.44	0.19	1.36	1.25	1.55	0.22	-2.16
Solyc07g0	0.90	0.97	0.95	0.45	0.47	0.38	2.18	1.12
Solyc05g0	0	0	0	0.14	0.42	0.08	0.00	-11.04
Solyc01g0	0.83	0.64	0.84	0.43	0.36	0.28	2.16	1.11
Solyc12g0	0.28	0.10	0.31	0	0	0	2321.45	11.18
Solyc04g0	1.42	1.32	1.55	4.41	3.62	4.46	0.34	-1.54
Solyc02g0	0.14	0.09	0.13	1.05	0.76	0.59	0.15	-2.75
Solyc01g1	0.47	0.35	0.50	0.94	0.91	0.88	0.48	-1.05
Solyc12g0	1.03	1.12	0.69	0	0.22	0.17	7.30	2.87
Solyc08g0	0.02	0.06	0	0.23	0.30	0.16	0.12	-3.09
Solyc04g0	0.78	0.96	0.96	0.34	0.27	0.31	2.93	1.55
Solyc04g0	1.21	1.38	1.74	2.89	3.44	3.13	0.46	-1.13
Solyc04g0	1.17	0.87	0.86	2.12	2.03	1.88	0.48	-1.06
Solyc07g0	2.44	2.24	1.59	4.96	4.20	3.92	0.48	-1.06
Solyc07g0	0.02	0	0	0.14	0.23	0.12	0.04	-4.66
Solyc05g0	0.04	0	0.02	0.14	0.17	0.19	0.13	-3.00
Solyc11g0	3.49	3.55	3.30	1.42	1.61	1.52	2.27	1.19
Solyc09g0	0.97	1.20	0.93	2.29	2.28	2.28	0.45	-1.14
Solyc06g0	1.16	1.07	1.09	0.27	0.26	0.42	3.49	1.81
Solyc03g1	0.61	0.71	0.56	0	0.10	0	18.12	4.18
Solyc08g0	0.14	0.08	0.14	0.64	0.41	0.36	0.26	-1.95
Solyc08g0	0	0.01	0.02	0.32	0.53	0.53	0.03	-5.18
Solyc02g0	0.43	0.54	0.58	0	0	0	5185.11	12.34
Solyc09g0	0	0	0.20	0.64	1.28	1.31	0.06	-4.02
Solyc09g0	0.12	0.03	0.15	0.67	0.42	0.55	0.18	-2.45
Solyc12g0	0.93	0.62	1.54	2.96	4.41	4.22	0.27	-1.90
Solyc10g0	0.31	0.38	0.24	0.83	0.88	0.93	0.35	-1.50
Solyc06g0	0.09	0.18	0.24	0.43	0.54	0.74	0.30	-1.75
Solyc03g1	0.54	0.26	0.43	0.93	1.14	1.22	0.37	-1.42
Solyc04g0	0.44	0.53	0.30	1.03	1.02	1.34	0.38	-1.41
Solyc09g0	0.38	0.40	0.31	1.16	0.88	1.26	0.33	-1.59
Solyc06g0	0	0	0	0.18	0.20	0.23	0.00	-10.98
Solyc02g0	0.49	0.61	0.66	1.10	1.18	1.56	0.46	-1.13
Solyc05g0	0.27	0.27	0.29	0.91	0.86	0.81	0.32	-1.63
Solyc10g0	0.64	0.89	0.71	1.39	1.76	1.64	0.47	-1.09
Solyc11g0	1.24	1.36	1.11	2.78	2.68	2.33	0.48	-1.07
Solyc01g0	0.03	0	0.03	0.20	0.17	0.11	0.11	-3.14
Solyc06g0	0.88	0.88	1.53	0.41	0.56	0.57	2.13	1.09
Solyc04g0	2.22	2.85	2.89	5.32	5.21	5.91	0.48	-1.05
Solyc01g0	0.19	0.33	0.58	0	0	0	3695.64	11.85
Solyc03g1	0.04	0	0.04	0.29	0.19	0.37	0.09	-3.43
Solyc04g0	0.52	0.50	0.44	1.01	1.20	0.79	0.48	-1.05
Solyc03g1	0.48	0.86	0.37	0.24	0.12	0.03	4.39	2.13
Solyc11g0	6.79	9.82	5.83	15.13	15.16	20.50	0.44	-1.18
Solyc01g1	0.06	0.03	0.08	0.37	0.45	0.16	0.18	-2.46
Solyc08g0	1.21	1.28	1.28	3.40	3.81	3.10	0.37	-1.45
Solyc05g0	0	0	0	0.64	0.84	0.68	0.00	-12.82
Solyc11g0	0.75	0.90	0.87	0	0	0	8396.24	13.04
Solyc11g0	1.00	1.36	1.16	0.45	0.08	0.38	3.86	1.95
Solyc08g0	0.01	0	0.01	0.11	0.10	0.22	0.06	-4.18
Solyc08g0	0.12	0.35	0.30	1.03	1.02	1.53	0.21	-2.23

Solyc03g0	0.34	0	0.22	1.73	1.95	1.91	0.10	-3.33
Solyc03g0	1.50	2.61	2.51	0.30	0	0	22.13	4.47
Solyc06g0	8.74	4.14	7.13	13.12	16.04	13.38	0.47	-1.09
Solyc11g0	2.39	2.26	2.34	1.22	0.94	0.91	2.28	1.19
Solyc05g0	0.30	0.28	0.24	1.12	0.77	0.57	0.33	-1.60
Solyc11g0	0	0.04	0	0.22	0.19	0.23	0.06	-4.06
Solyc04g0	0.57	0.70	0.56	0.33	0.21	0.32	2.12	1.08
Solyc12g0	2.35	3.05	2.86	9.03	6.90	6.38	0.37	-1.43
Solyc06g0	1.03	0.99	1.46	0.37	0.28	0.53	2.93	1.55
Solyc11g0	0	0.54	0.39	3.35	2.53	2.50	0.11	-3.17
Solyc08g0	0.38	0.29	0.33	0.93	1.01	0.93	0.35	-1.53
Solyc03g0	1.31	1.31	1.55	0.62	0.51	0.55	2.48	1.31
Solyc09g0	0.68	0.74	0.70	0.32	0.21	0.13	3.21	1.68
Solyc03g1	0.63	0.35	0.36	0.93	1.15	0.92	0.45	-1.16
Solyc08g0	0.12	0.09	0.10	0.44	0.32	0.34	0.29	-1.80
Solyc07g0	0.27	0.11	0.44	0.96	1.34	1.24	0.23	-2.11
Solyc03g0	0.60	0.94	0.73	0.07	0.21	0.26	4.19	2.07
Solyc05g0	1.63	1.73	1.54	0.72	1.01	0.63	2.08	1.06
Solyc10g0	0.59	0.48	0.53	0.27	0.19	0.20	2.44	1.29
Solyc03g0	0.08	0.04	0.13	0.31	0.90	0.41	0.15	-2.72
Solyc11g0	0.31	0.36	0.47	1.03	1.18	0.79	0.38	-1.39
Solyc02g0	1.97	2.31	1.70	1.20	1.06	0.68	2.03	1.02
Solyc03g1	0.77	0.59	0.75	1.51	1.71	1.23	0.47	-1.08
Solyc03g1	0.39	0.50	0.42	1.10	1.05	0.83	0.44	-1.19
Solyc01g0	0.02	0.02	0	0.24	0.32	0.26	0.06	-4.16
Solyc06g0	0	0	0	0.05	0.07	0.06	0.00	-9.23
Solyc04g0	2.48	2.67	3.26	1.01	0.98	1.85	2.19	1.13
Solyc05g0	4.89	4.19	3.73	2.63	1.09	2.25	2.15	1.10
Solyc03g0	0.81	0.83	0.52	1.65	1.44	1.71	0.45	-1.15
Solyc06g0	0.57	0.96	0.44	2.04	1.66	1.56	0.38	-1.41
Solyc12g0	1.43	1.13	1.85	0.35	0.22	0.21	5.66	2.50
Solyc01g0	0.44	0.84	0.96	1.71	1.53	1.98	0.43	-1.22
Solyc01g0	0.11	0.06	0.07	0	0	0	778.53	9.60
Solyc01g0	0.05	0.17	0.18	0.42	0.46	0.61	0.28	-1.86
Solyc05g0	3.55	3.22	2.96	1.71	1.42	1.08	2.31	1.21
Solyc07g0	0.71	0.67	0.77	0.27	0.34	0.21	2.61	1.38
Solyc07g0	0.30	0.18	0.27	0.85	0.85	0.65	0.32	-1.65
Solyc02g0	0.26	0.38	0.03	0	0	0	2242.32	11.13
Solyc12g0	0.11	0.28	0.22	0.04	0	0.02	9.14	3.19
Solyc02g0	1.52	1.37	1.73	0.50	0.54	0.67	2.71	1.44
Solyc03g0	0	0	0.07	0.61	0.55	0.47	0.04	-4.59
Solyc05g0	2.39	1.12	1.67	4.45	3.67	4.15	0.42	-1.25
Solyc08g0	0.43	0.33	0.46	0.87	1.17	0.94	0.41	-1.30
Solyc08g0	0.13	0.20	0.18	0.04	0.01	0	9.73	3.28
Solyc09g0	0	0.03	0	0.17	0.15	0.34	0.04	-4.53
Solyc07g0	0.34	0.25	0.37	0.62	0.64	0.66	0.50	-1.01
Solyc08g0	0.03	0.01	0	0.18	0.08	0.11	0.11	-3.22
Solyc09g0	1.24	1.51	1.01	0.56	0.39	0.59	2.43	1.28
Solyc08g0	2.57	1.64	2.63	1.12	1.12	0.92	2.17	1.12
Solyc08g0	5.23	5.73	5.22	2.58	2.69	2.67	2.04	1.03
Solyc06g0	0.06	0.12	0.08	0.38	0.28	0.37	0.26	-1.94

Solyc01g1	0.21	0.34	0.39	1.64	1.79	1.58	0.19	-2.41
Solyc12g0	0.02	0	0	0.13	0.11	0.12	0.06	-4.02
Solyc02g0	1.60	1.21	1.72	3.12	3.45	3.39	0.45	-1.14
Solyc06g0	0.72	0.54	0.29	0.12	0.03	0.13	5.75	2.52
Solyc07g0	0.18	0.13	0.17	0.44	0.40	0.57	0.34	-1.55
Solyc02g0	0.27	0.07	0.09	0	0	0	1436.80	10.49
Solyc05g0	0	0	0	0.37	0.26	0.05	0.00	-11.15
Solyc10g0	0.67	0.50	0.71	1.69	1.32	1.09	0.46	-1.13
Solyc01g0	0.35	0.44	0.37	0.99	0.81	1.11	0.40	-1.33
Solyc12g0	0.02	0	0.08	0.25	0.39	0.39	0.09	-3.42
Solyc03g0	0.42	0.55	0.35	1.06	0.90	0.75	0.48	-1.05
Solyc09g0	1.24	0.73	0.63	2.97	3.27	2.92	0.28	-1.82
Solyc02g0	0.63	0.97	0.70	1.66	1.76	1.71	0.45	-1.17
Solyc12g0	0	0	0	0.06	0.12	0.07	0.00	-9.73
Solyc12g0	0	0	0	0.20	0.13	0.16	0.00	-10.69
Solyc07g0	2.12	2.50	2.73	0.72	1.55	0.86	2.34	1.23
Solyc04g0	0.09	0.13	0.13	0.35	0.40	0.65	0.25	-2.03
Solyc09g0	0.22	0.35	0.21	0.04	0.05	0.09	4.39	2.13
Solyc02g0	0.07	0	0.06	0.23	0.24	0.35	0.16	-2.63
Solyc07g0	0	0.13	0.07	0.67	0.42	0.53	0.12	-3.04
Solyc08g0	0.45	0.65	0.67	0.99	1.86	1.82	0.38	-1.40
Solyc11g0	0.15	0	0.13	0.93	0.82	0.52	0.12	-3.04
Solyc12g0	0.64	1.47	1.35	2.46	2.06	3.29	0.44	-1.17
Solyc09g0	0	0	0	0.07	0.08	0.04	0.00	-9.29
Solyc03g0	0.58	0.69	0.58	0.32	0.26	0.21	2.33	1.22
Solyc12g0	0.40	0.49	0.35	1.35	0.95	1.35	0.34	-1.55
Solyc05g0	0.74	0.44	0.34	1.14	1.45	1.12	0.41	-1.28
Solyc08g0	0.09	0.17	0.14	0.37	0.39	0.31	0.37	-1.42
Solyc06g0	2.33	2.43	1.31	4.59	4.09	3.80	0.49	-1.04
Solyc10g0	0.73	0.72	0.41	1.95	1.05	1.58	0.40	-1.31
Solyc06g0	0.15	0.10	0.11	0.35	0.35	0.49	0.31	-1.70
Solyc09g0	1.43	1.85	1.01	0.35	0.28	0.47	3.90	1.96
Solyc11g0	0	0	0.01	0.12	0.07	0.15	0.04	-4.78
Solyc02g0	1.22	0.93	1.35	0.47	0.64	0.34	2.43	1.28
Solyc05g0	2.16	3.19	3.97	6.21	7.33	6.36	0.47	-1.10
Solyc01g0	0	0	0	0.06	0.14	0.13	0.00	-10.07
Solyc05g0	1.37	1.22	1.06	3.08	2.66	2.46	0.45	-1.17
Solyc10g0	1.16	1.05	1.20	0	0	0.26	13.20	3.72
Solyc07g0	1.60	1.62	1.61	0.51	0.86	0.30	2.89	1.53
Solyc07g0	2.41	2.45	3.07	1.50	0.96	0.82	2.43	1.28
Solyc03g1	0.49	0.50	0.37	0.11	0.19	0.16	2.90	1.54
Solyc03g1	0.33	0.22	0.22	0.87	0.60	0.70	0.35	-1.51
Solyc02g0	0	0	0.03	0.13	0.15	0.24	0.06	-3.96
Solyc01g1	0.04	0.03	0.12	0.42	0.24	0.33	0.20	-2.34
Solyc07g0	0	0	0	0.10	0.18	0.12	0.00	-10.38
Solyc09g0	0.26	0.21	0.14	0	0	0	2049.83	11.00
Solyc12g0	0.72	0.66	0.55	2.22	1.72	1.30	0.37	-1.44
Solyc12g0	0.59	0.49	0.59	2.05	1.28	1.06	0.38	-1.40
Solyc01g0	0	0	0	0.59	0.30	0.25	0.00	-11.90
Solyc02g0	2.26	1.51	1.77	3.00	5.09	5.05	0.42	-1.25
Solyc02g0	0.92	0.28	0.61	0	0.18	0.07	7.32	2.87

Solyc07g0	1.43	1.30	0.84	0.26	0.36	0.33	3.80	1.93
Solyc08g0	1.40	1.14	1.63	0.63	0.75	0.47	2.25	1.17
Solyc04g0	2.84	2.85	2.67	0.93	1.38	1.52	2.18	1.13
Solyc12g0	0.97	0.72	1.02	2.33	2.29	1.69	0.43	-1.22
Solyc12g0	0.03	0.04	0	0.21	0.19	0.12	0.13	-2.93
Solyc01g0	0.55	0.53	0.17	0	0	0	4163.59	12.02
Solyc11g0	0.65	0.83	0.41	0.28	0.27	0.21	2.48	1.31
Solyc11g0	0.09	0.10	0.36	0	0	0	1843.65	10.85
Solyc04g0	0.21	0.18	0.17	0.53	0.30	0.48	0.43	-1.23
Solyc01g1	1.83	1.62	1.34	0.39	0.76	0.56	2.79	1.48
Solyc11g0	0.33	0.28	0.43	1.33	0.84	1.01	0.33	-1.60
Solyc09g0	0.10	0	0.11	0.70	0.74	0.57	0.10	-3.28
Solyc04g0	0	0.27	0.22	0.81	2.11	0.98	0.13	-2.98
Solyc09g0	0.58	1.02	0.66	2.13	1.26	2.70	0.37	-1.42
Solyc03g1	0.44	0.47	0.56	1.22	0.73	1.09	0.48	-1.05
Solyc02g0	0.69	1.13	1.78	2.72	2.95	2.35	0.45	-1.16
Solyc01g1	0.39	0.53	0.77	1.03	0.84	1.53	0.50	-1.01
Solyc06g0	0.60	0.59	0.76	1.59	1.44	1.07	0.48	-1.07
Solyc03g0	0.35	0.44	0.52	1.30	1.14	1.15	0.36	-1.46
Solyc02g0	0.06	0.11	0.11	0.21	0.37	0.33	0.31	-1.67
Solyc07g0	0.75	1.05	0.67	0.21	0.21	0	5.85	2.55
Solyc10g0	0.16	0.14	0	0.69	0.68	0.58	0.15	-2.71
Solyc02g0	0.39	0.11	0.09	0.83	0.76	1.05	0.22	-2.19
Solyc12g0	0.41	0.29	0.32	0.84	0.83	0.82	0.41	-1.30
Solyc10g0	0	0	0	0.19	0.17	0.31	0.00	-11.11
Solyc01g1	1.00	0.77	0.84	0.38	0.29	0.21	2.95	1.56
Solyc02g0	0.27	0.21	0.19	0.51	0.60	0.39	0.45	-1.15
Solyc04g0	0	0	0	0.18	0.15	0.15	0.00	-10.62
Solyc03g0	0.42	0.62	0.22	1.05	1.53	1.72	0.29	-1.76
Solyc03g1	0	0	0	0.09	0.02	0.09	0.00	-9.32
Solyc04g0	0.78	0.52	0.40	0.13	0.16	0.07	4.79	2.26
Solyc10g0	0.51	0.60	0.89	1.73	1.29	1.66	0.43	-1.22
Solyc05g0	1.83	1.25	1.57	0.64	0.90	0.48	2.31	1.21
Solyc11g0	0.22	0.26	0.19	0.06	0.06	0.07	3.60	1.85
Solyc12g0	0.96	0.60	0.67	2.01	2.35	1.25	0.40	-1.33
Solyc03g0	0.15	0.30	0.06	0.73	0.43	0.66	0.27	-1.86
Solyc02g0	0.15	0.19	0.25	0.59	0.78	0.43	0.33	-1.61
Solyc02g0	0	0	0	0.92	0.12	0.19	0.00	-12.00
Solyc05g0	0.70	1.25	0.97	1.76	2.23	2.10	0.48	-1.07
Solyc07g0	1.16	0.87	1.61	2.83	2.76	3.12	0.42	-1.26
Solyc01g0	0.12	0.11	0.10	0.34	0.43	0.28	0.31	-1.67
Solyc06g0	0.58	0.32	0.35	1.71	0.91	1.46	0.31	-1.71
Solyc07g0	0.06	0.04	0.07	0	0	0	558.88	9.13
Solyc07g0	0.10	0.09	0.06	0.36	0.22	0.33	0.29	-1.79
Solyc05g0	0.32	0.38	0.27	0.79	0.99	0.69	0.39	-1.35
Solyc06g0	0.46	0.28	0.44	1.08	1.70	0.91	0.32	-1.65
Solyc10g0	0.68	0.71	0.96	1.17	1.51	2.11	0.49	-1.03
Solyc10g0	2.99	3.21	2.71	1.39	1.31	1.22	2.27	1.18
Solyc05g0	0	0	0	0.36	0.31	0.16	0.00	-11.44
Solyc03g0	0.13	0.06	0.17	0.34	0.45	0.68	0.25	-2.02
Solyc05g0	1.33	2.29	1.79	0.71	0.90	0.79	2.25	1.17

Solyc07g0	0.06	0	0.12	0.39	0.28	0.34	0.18	-2.47
Solyc04g0	0.65	0.38	0.34	0.18	0.14	0.11	3.15	1.66
Solyc08g0	0.04	0.01	0.04	0.17	0.11	0.23	0.18	-2.44
Solyc03g0	0.02	0.01	0.02	0.14	0.08	0.10	0.16	-2.66
Solyc09g0	0	0	0	0.08	0.06	0.06	0.00	-9.38
Solyc12g1	0	0	0	0.16	0.14	0.10	0.00	-10.37
Solyc10g0	0.46	0.52	0.65	0.21	0.14	0.13	3.44	1.78
Solyc11g0	4.23	2.93	3.17	1.38	1.88	1.25	2.29	1.20
Solyc05g0	0.02	0	0	0.17	0.18	0.11	0.04	-4.72
Solyc10g0	1.75	1.40	1.10	3.41	3.34	2.70	0.45	-1.15
Solyc08g0	0.19	0.23	0.14	0.52	0.53	0.33	0.40	-1.32
Solyc04g0	0.02	0.06	0.11	0	0	0	607.22	9.25
Solyc09g0	0	0	0	0.08	0.08	0.07	0.00	-9.57
Solyc11g0	0	0	0	0.02	0.04	0.03	0.00	-8.26
Solyc07g0	0.25	0.42	0.32	0.12	0.09	0.07	3.47	1.79
Solyc11g0	1.42	0.80	1.02	0	0	0	10805.31	13.40
Solyc01g0	0.58	0.40	0.40	0	0	0.12	11.12	3.48
Solyc12g0	0.24	0.28	0.31	0.85	0.69	0.55	0.40	-1.34
Solyc02g0	0.21	0.19	0.33	0.57	0.51	0.66	0.42	-1.25
Solyc09g0	0.63	1.02	0.52	2.31	1.58	1.15	0.43	-1.22
Solyc04g0	0.83	0.97	0.84	0.37	0.52	0.43	2.01	1.01
Solyc09g0	0.39	0.48	0.58	0.81	1.03	1.17	0.48	-1.06
Solyc08g0	0	0	0	0.19	0.14	0.30	0.00	-11.03
Solyc02g0	0.15	0.17	0.10	0.29	0.31	0.34	0.45	-1.16
Solyc03g1	1.07	0.93	0.81	1.54	1.50	2.80	0.48	-1.06
Solyc02g0	0.26	0.33	0.24	0.10	0.14	0.12	2.27	1.18
Solyc04g0	0.65	0.59	0.66	0.23	0.21	0.21	2.91	1.54
Solyc05g0	0.15	0.33	0.42	0.86	0.61	0.81	0.40	-1.34
Solyc04g0	0.40	0.75	0.62	2.19	3.55	2.90	0.21	-2.28
Solyc02g0	0.58	0.45	0.37	1.71	1.26	1.18	0.34	-1.57
Solyc07g0	2.29	2.32	1.74	1.14	0.74	1.08	2.14	1.10
Solyc11g0	0.16	0.19	0.14	0.40	0.45	0.42	0.38	-1.39
Solyc02g0	0.60	0.37	0.56	0.84	1.10	1.14	0.50	-1.01
Solyc02g0	0	0	0	0.21	0.35	0.26	0.00	-11.41
Solyc07g0	2.59	1.79	3.25	1.19	1.07	0.89	2.42	1.28
Solyc07g0	0.55	0.58	0.81	1.38	1.03	1.72	0.47	-1.08
Solyc07g0	0.34	0.50	0.49	0.90	1.04	0.76	0.49	-1.02
Solyc05g0	0.92	0.98	0.59	0.34	0.29	0.52	2.16	1.11
Solyc03g0	0	0	0	0.13	0.27	0.06	0.00	-10.59
Solyc11g0	0.83	1.05	1.13	0.17	0	0.29	6.49	2.70
Solyc11g0	0.05	0	0.10	0.42	0.39	0.39	0.13	-2.94
Solyc08g0	0.13	0.32	0.20	0.54	1.18	0.97	0.24	-2.03
Solyc10g0	0	0	0	0.29	0.40	0.18	0.00	-11.52
Solyc08g0	0.06	0.02	0.03	0.28	0.18	0.14	0.19	-2.38
Solyc06g0	0.40	0.33	0.43	0.08	0.08	0.10	4.54	2.18
Solyc10g0	0.12	0.09	0.09	0.03	0	0	12.09	3.60
Solyc03g0	3.93	3.73	4.38	1.04	1.41	0.65	3.89	1.96
Solyc11g0	0	0	0	0.02	0.10	0.07	0.00	-9.30
Solyc01g1	0.36	0.30	0.44	0.83	0.62	0.98	0.45	-1.15
Solyc05g0	0.13	0.03	0	0.90	1.03	0.88	0.06	-4.14
Solyc11g0	0.45	0.34	0.37	0.14	0.18	0.18	2.39	1.25

Solyc03g0	0	0	0	0.00	0.06	0.07	0.00	-8.79
Solyc06g0	0.66	0.59	0.49	0	0	0	5812.44	12.50
Solyc03g1	0.12	0.14	0.26	0.64	0.47	0.42	0.34	-1.57
Solyc09g0	0.29	0.16	0.37	0.03	0.08	0.08	4.53	2.18
Solyc11g0	0.92	0.63	0.55	0.10	0.14	0.17	5.14	2.36
Solyc03g0	0.06	0.08	0.10	0.34	0.24	0.46	0.23	-2.14
Solyc01g1	1.05	1.28	1.18	0.43	0.26	0.34	3.40	1.76
Solyc12g0	0.19	0.17	0.16	0.30	0.37	0.43	0.48	-1.07
Solyc02g0	3.36	3.74	3.54	1.49	1.45	2.01	2.15	1.10
Solyc10g0	2.16	2.33	2.40	1.46	0.63	0.59	2.57	1.36
Solyc03g0	0.19	0.24	0.19	0.05	0.11	0.09	2.49	1.31
Solyc06g0	0.05	0.13	0.09	0	0	0	896.80	9.81
Solyc05g0	1.05	0.77	0.59	0.35	0.30	0.49	2.10	1.07
Solyc06g0	0.21	0.26	0.14	0.06	0.09	0.03	3.53	1.82
Solyc03g1	0.03	0.01	0	0.28	0.14	0.08	0.08	-3.61
Solyc03g0	0.22	0.37	0.38	0.17	0.14	0.14	2.14	1.10
Solyc07g0	0	0	0	0.09	0.48	0.21	0.00	-11.35
Solyc02g0	0.10	0.14	0.05	0.29	0.24	0.31	0.34	-1.56
Solyc10g0	0	0	0	0.29	0.15	0.44	0.00	-11.52
Solyc02g0	0.03	0.02	0.03	0.29	0.32	0.09	0.11	-3.14
Solyc06g0	0	0	0.03	0.15	0.09	0.10	0.08	-3.70
Solyc12g0	0.39	0.58	0.37	1.02	1.07	0.91	0.45	-1.16
Solyc07g0	0.31	0.16	0.28	0.61	0.59	0.50	0.44	-1.18
Solyc06g0	0.10	0.14	0.27	0.42	0.47	0.62	0.34	-1.56
Solyc03g1	0.49	0.69	0.69	1.37	1.30	1.26	0.47	-1.08
Solyc11g0	0.07	0	0.11	0.51	0.35	0.21	0.16	-2.61
Solyc05g0	0	0	0	0.09	0.03	0.02	0.00	-8.91
Solyc06g0	0.06	0	0	0.46	0.43	0.35	0.05	-4.31
Solyc12g0	0.55	0.35	0.24	1.23	1.57	1.07	0.30	-1.76
Solyc03g0	0.08	0.38	0.24	0.04	0	0	18.41	4.20
Solyc01g0	0.09	0.02	0.08	0.37	0.19	0.54	0.18	-2.50
Solyc03g0	0.38	0.30	0.33	0.09	0	0	11.59	3.53
Solyc02g0	0.22	0.47	0.34	0.90	0.69	0.67	0.46	-1.12
Solyc10g0	0.22	0.35	0.64	1.52	0.83	1.38	0.33	-1.62
Solyc03g0	0	0	0	0.01	0.01	0.02	0.01	-7.23
Solyc12g0	0.04	0.03	0.03	0.24	0.20	0.10	0.18	-2.45
Solyc01g1	0.04	0.14	0.04	0.59	0.48	0.42	0.15	-2.78
Solyc08g0	0.16	0.05	0.03	0	0	0	799.01	9.64
Solyc10g0	0.47	0.74	0.85	0.26	0.23	0.34	2.49	1.31
Solyc01g0	0.84	0.56	0.41	1.66	1.14	0.97	0.48	-1.06
Solyc03g0	0.40	0.24	0.31	1.11	0.71	0.57	0.39	-1.34
Solyc03g0	1.65	1.18	1.77	4.05	3.03	3.23	0.45	-1.17
Solyc06g0	0.11	0.16	0.23	0.49	0.45	0.40	0.37	-1.45
Solyc03g1	0.06	0	0	0.24	0.23	0.11	0.10	-3.33
Solyc08g0	0.98	0.85	0.49	1.62	2.54	1.93	0.38	-1.39
Solyc06g0	0.09	0.16	0.10	0.46	0.27	0.41	0.31	-1.70
Solyc07g0	0.27	0.19	0.45	0.79	0.72	0.74	0.40	-1.31
Solyc10g0	0.66	0.52	0.21	0.05	0.01	0.03	14.83	3.89
Solyc12g0	0.38	0.32	0.34	0.82	1.11	0.70	0.39	-1.35
Solyc01g0	0.37	0.35	0.23	0.08	0.16	0.12	2.66	1.41
Solyc02g0	0.39	0.40	0.46	0.13	0.19	0.25	2.19	1.13

Solyc03g1	2.74	2.69	3.20	6.04	6.55	5.35	0.48	-1.05
Solyc09g0	0.38	0.32	0.25	0	0	0	3152.56	11.62
Solyc03g0	1.40	1.50	1.83	0.62	1.06	0.53	2.14	1.10
Solyc06g0	0.26	0.24	0.39	0	0.09	0	9.98	3.32
Solyc01g0	2.17	1.83	2.20	0.67	1.35	0.88	2.13	1.09
Solyc08g0	0.08	0.10	0.04	0	0	0	745.09	9.54
Solyc08g0	0	0	0.03	0.14	0.13	0.14	0.07	-3.82
Solyc09g0	0	0	0.14	0.26	0.54	0.51	0.10	-3.29
Solyc04g0	0.40	0.52	0.18	0.77	0.84	0.90	0.44	-1.19
Solyc02g0	0	0.05	0.02	0.18	0.20	0.18	0.12	-3.05
Solyc10g0	0	0.05	0.11	0.41	0.25	0.32	0.16	-2.65
Solyc04g0	75.13	75.09	0	0	0	0	500727.74	18.93
Solyc01g0	0.12	0.09	0	0.49	0.59	0.60	0.12	-3.05
Solyc03g0	0	0	0	0.21	0.05	0.11	0.00	-10.30
Solyc09g0	0	0	0	0.52	0.33	0.36	0.00	-11.98
Solyc08g0	1.14	0.91	0.79	0.37	0.30	0.07	3.83	1.94
Solyc01g0	1.57	0.99	1.44	2.45	3.70	3.46	0.42	-1.26
Solyc12g0	0.15	0.19	0.08	0	0	0	1405.23	10.46
Solyc10g0	0.65	1.06	0.70	0.41	0.27	0.26	2.57	1.36
Solyc06g0	0.05	0.02	0.03	0	0	0	366.52	8.52
Solyc07g0	0.97	0.87	0.41	0.17	0	0.28	4.97	2.31
Solyc10g0	0.09	0.30	0.13	0	0	0	1745.96	10.77
Solyc01g1	0.23	1.06	0.40	2.05	1.14	1.64	0.35	-1.51
Solyc09g0	0.52	0.25	0.50	0.15	0.06	0	6.02	2.59
Solyc02g0	0.18	0.16	0.18	0.50	0.34	0.37	0.43	-1.23
Solyc01g0	0.06	0	0	0.37	0.39	0.40	0.06	-4.18
Solyc10g0	0	0	0	0.23	0.16	0.28	0.00	-11.12
Solyc09g0	0.46	1.26	1.62	3.48	3.52	3.04	0.33	-1.59
Solyc02g0	0.13	0.14	0.06	0.02	0.03	0	7.41	2.89
Solyc06g0	0	0	0	0.62	0.78	0.65	0.00	-12.74
Solyc09g0	0.41	0.32	0.63	0.95	1.07	0.85	0.47	-1.09
Solyc06g0	0	0	0	0.09	0.05	0.32	0.00	-10.57
Solyc10g0	0.43	0.69	0.45	1.67	1.42	1.85	0.32	-1.66
Solyc12g0	0.35	0.35	0.30	0.89	0.63	0.68	0.46	-1.12
Solyc01g0	0	0	0.11	0.25	0.34	0.30	0.12	-3.07
Solyc10g0	0.21	0.45	0.38	0.89	0.71	0.50	0.50	-1.01
Solyc10g0	1.24	1.40	1.01	2.12	2.67	2.79	0.48	-1.05
Solyc02g0	0.20	0.20	0.20	0.07	0.09	0.10	2.29	1.19
Solyc01g0	0.52	0.54	0.66	0.16	0.30	0.28	2.28	1.19
Solyc01g1	0.09	0.29	0.19	0.78	0.57	0.59	0.29	-1.76
Solyc02g0	0.07	0.09	0.05	0	0	0	722.84	9.50
Solyc08g0	0.02	0	0.13	0.23	0.34	0.32	0.18	-2.51
Solyc09g0	0.12	0.24	0.08	0.75	0.58	0.23	0.29	-1.78
Solyc06g0	0	0.06	0	0.43	0.21	0.26	0.06	-4.04
Solyc10g0	1.35	1.47	1.09	3.09	2.60	2.69	0.47	-1.10
Solyc01g1	0.15	0.20	0.31	0.60	0.52	0.55	0.39	-1.36
Solyc03g0	0.18	0.24	0.18	0	0.07	0.05	4.88	2.29
Solyc02g0	0.23	0.22	0.23	0.38	0.61	0.38	0.49	-1.03
Solyc04g0	2.27	1.22	1.86	3.88	2.90	4.98	0.45	-1.14
Solyc04g0	8.32	7.77	2.42	1.85	2.22	1.93	3.09	1.63
Solyc04g0	0.28	0.21	0.29	0.72	0.65	0.71	0.37	-1.43

Solyc03g1	0.12	0.28	0.26	0.02	0.11	0.04	4.07	2.02
Solyc03g0	0.13	0.19	0.22	0.02	0.04	0.06	4.40	2.14
Solyc12g0	0	0	0	0.19	0.36	0.30	0.00	-11.47
Solyc01g0	0.22	0.19	0.16	0.57	0.47	0.55	0.35	-1.50
Solyc03g0	0.58	0.42	0.41	1.17	1.10	1.13	0.41	-1.27
Solyc01g0	1.37	1.27	0.97	0.06	0.54	0.31	3.95	1.98
Solyc04g0	0	0	0	0.11	0.19	0.44	0.00	-11.26
Solyc05g0	0.94	1.39	1.01	0.30	0.31	0.57	2.82	1.49
Solyc01g0	0	0	0	0.26	0.34	0.21	0.00	-11.42
Solyc01g0	0.31	0.25	0.31	0.69	0.50	0.56	0.49	-1.02
Solyc02g0	0.96	0.40	0.78	2.11	1.56	1.64	0.40	-1.31
Solyc09g0	0.23	0.18	0.09	0.36	0.41	0.55	0.38	-1.40
Solyc03g0	2.09	2.16	2.25	0	0.18	0	36.70	5.20
Solyc03g1	1.50	1.26	0.51	2.15	2.64	2.38	0.45	-1.14
Solyc03g0	0.16	0	0	0.48	0.78	0.78	0.08	-3.68
Solyc02g0	0.04	0.10	0.04	0.21	0.30	0.56	0.17	-2.55
Solyc05g0	0.87	0.63	0.37	1.22	1.62	1.64	0.42	-1.26
Solyc08g0	0	0.05	0.12	0.91	0.78	0.60	0.07	-3.76
Solyc09g0	0.20	0.33	0.37	0.12	0.10	0.08	3.02	1.59
Solyc01g1	0	0	0	0.09	0.11	0.05	0.00	-9.69
Solyc04g0	0.22	0.35	0.28	0.97	0.66	0.54	0.39	-1.36
Solyc09g0	0.02	0	0	0.05	0.16	0.11	0.08	-3.73
Solyc04g0	0.23	0.42	0.34	0.94	1.12	0.82	0.34	-1.54
Solyc06g0	0.08	0.05	0.12	0.35	0.43	0.16	0.26	-1.94
Solyc03g0	0	0	0	0.01	0.03	0.05	0.00	-8.17
Solyc06g0	1.13	1.06	1.03	1.63	2.09	2.96	0.48	-1.05
Solyc01g1	0	0	0	0.06	0.03	0.13	0.00	-9.51
Solyc01g0	0.39	0.54	0.33	0.13	0.16	0.19	2.61	1.39
Solyc10g0	0.94	1.11	1.58	3.27	3.20	3.61	0.36	-1.47
Solyc04g0	1.43	1.40	1.59	0.56	0.11	0.12	5.58	2.48
Solyc02g0	0	0	0	0.03	0.04	0.02	0.00	-8.25
Solyc05g0	0.40	0.22	0.56	1.13	1.04	0.60	0.43	-1.23
Solyc04g0	0.06	0.12	0.03	0.27	0.15	0.32	0.27	-1.87
Solyc09g0	1.39	2.57	2.16	3.88	5.73	3.74	0.46	-1.13
Solyc08g0	1.05	0.72	0.69	0	0.08	0	29.09	4.86
Solyc03g1	0.07	0.10	0.03	0	0	0	668.93	9.39
Solyc06g0	0.22	0.25	0.25	0.39	0.84	0.76	0.36	-1.46
Solyc09g0	1.19	1.42	0.92	2.75	1.97	3.10	0.45	-1.15
Solyc09g0	0	0	0	0.17	0.17	0.07	0.00	-10.42
Solyc07g0	0.07	0.10	0.13	0.30	0.21	0.31	0.37	-1.43
Solyc11g0	0	0	0	0.03	0.03	0.04	0.00	-8.36
Solyc01g0	0	0	0	0.22	0.14	0.09	0.00	-10.54
Solyc12g0	0.08	0.17	0.12	0.04	0	0	8.56	3.10
Solyc01g0	0.10	0.33	0.53	0	0	0	3191.65	11.64
Solyc11g0	0	0	0	0.06	0.06	0.05	0.00	-9.16
Solyc10g0	0.59	0.69	0.99	0.29	0.25	0.25	2.87	1.52
Solyc09g0	0.02	0.03	0	0.11	0.13	0.20	0.13	-2.94
Solyc07g0	0	0	0	0.02	0.07	0.04	0.00	-8.72
Solyc12g0	0	0	0	0.17	0.07	0.05	0.00	-9.93
Solyc10g0	0.62	0.65	0.55	0.10	0.07	0.27	4.12	2.04
Solyc03g0	0	0	0.11	0.56	1.15	1.24	0.04	-4.71

Solyc11g0	2.21	1.09	1.96	4.10	3.31	3.83	0.47	-1.10
Solyc03g0	0.17	0	0.48	1.01	1.09	1.05	0.21	-2.28
Solyc01g0	0.79	1.23	1.31	0.71	0.50	0.44	2.01	1.01
Solyc05g0	0.64	1.10	0.65	1.58	1.57	2.04	0.46	-1.12
Solyc07g0	0.18	0.47	0.20	0	0	0	2831.71	11.47
Solyc08g0	0	0.12	0.05	0.40	0.20	0.34	0.17	-2.53
Solyc07g0	0.05	0.07	0.07	0	0	0	638.37	9.32
Solyc10g0	6.10	5.24	5.87	2.38	2.89	1.90	2.40	1.26
Solyc06g0	0.13	0	0.24	0.55	0.61	0.56	0.22	-2.19
Solyc06g0	0	0	0.05	0.26	0.45	0.58	0.04	-4.72
Solyc02g0	0.42	0.47	0.33	1.28	1.03	0.53	0.43	-1.20
Solyc11g0	0	0	0	0.07	0.06	0.11	0.00	-9.61
Solyc02g0	0.25	0.24	0.10	0	0	0	1965.81	10.94
Solyc09g0	0.29	0.21	0.22	0.12	0.09	0.10	2.30	1.20
Solyc10g0	0	0	0.06	0.26	0.17	0.22	0.08	-3.56
Solyc07g0	1.26	1.99	1.37	0.74	0.78	0.13	2.78	1.48
Solyc11g0	0.08	0.09	0.12	0.25	0.20	0.16	0.49	-1.03
Solyc02g0	0.04	0	0.05	0.61	0.24	0.42	0.07	-3.77
Solyc10g0	0.18	0.33	0.10	0.45	0.52	0.49	0.42	-1.27
Solyc06g0	0.62	0.47	0.35	1.45	1.14	1.13	0.39	-1.36
Solyc03g0	0.75	1.05	0.82	2.49	2.04	2.23	0.39	-1.37
Solyc01g0	0.31	1.00	0.18	1.25	1.55	1.84	0.32	-1.64
Solyc05g0	0.16	0.37	0.58	0.18	0.12	0.09	2.79	1.48
Solyc01g1	0.56	0.21	0.35	0.96	0.78	0.87	0.43	-1.22
Solyc01g1	3.64	4.86	4.52	1.50	1.40	2.05	2.63	1.39
Solyc07g0	0	0	0	0.04	0.13	0.04	0.00	-9.40
Solyc05g0	0.45	0.16	0.17	0.89	0.56	0.82	0.34	-1.55
Solyc12g0	0.13	0.16	0.19	0.30	0.43	0.46	0.40	-1.33
Solyc04g0	0.18	0.07	0.15	0.29	0.31	0.32	0.43	-1.23
Solyc01g0	0.09	0.08	0.05	0	0.01	0.02	6.71	2.75
Solyc10g0	0.19	0.27	0.09	0.46	0.44	0.52	0.38	-1.38
Solyc10g0	0.20	0.32	0.39	0.54	0.74	0.53	0.50	-1.00
Solyc10g0	0.06	0.18	0.19	0.40	0.43	0.41	0.35	-1.51
Solyc02g0	0.12	0	0.17	1.28	0.67	1.02	0.10	-3.34
Solyc03g0	0.33	0.11	0.14	0.91	0.49	1.38	0.21	-2.26
Solyc08g0	0.09	0.14	0.08	0	0.03	0	11.48	3.52
Solyc11g0	0	0	0	0.01	0.05	0.03	0.00	-8.21
Solyc02g0	0.14	0.20	0.18	0	0	0.05	10.07	3.33
Solyc02g0	0.02	0.04	0.03	0	0	0	313.46	8.29
Solyc11g0	0.38	0.64	0.55	0.84	1.43	1.06	0.47	-1.08
Solyc08g0	0.06	0.19	0.24	0.71	0.43	0.36	0.32	-1.65
Solyc09g0	0.10	0	0.16	0.92	0.78	0.73	0.11	-3.22
Solyc09g0	1.77	1.97	1.09	0.47	0.71	0	4.11	2.04
Solyc03g1	0	0.02	0	0.07	0.03	0.08	0.10	-3.35
Solyc03g1	1.22	0.66	1.21	1.95	1.98	2.86	0.46	-1.13
Solyc06g0	0.03	0.10	0.04	0.01	0.01	0	11.24	3.49
Solyc07g0	0.06	0	0.07	0.19	0.15	0.33	0.20	-2.33
Solyc09g0	0	0	0.06	0.18	0.16	0.26	0.11	-3.24
Solyc11g0	0.22	0.14	0.21	1.27	0.50	0.66	0.24	-2.07
Solyc05g0	0	0	0	0.30	0.13	0.29	0.00	-11.22
Solyc05g0	0.28	0.32	0	0	0	0	1995.76	10.96

Solyc11g0	0.12	0.10	0.13	0.23	0.68	0.46	0.25	-1.98
Solyc03g1	0.05	0	0.11	0.39	0.40	0.23	0.16	-2.67
Solyc05g0	0.40	0.39	0.41	1.02	0.82	0.78	0.46	-1.13
Solyc08g0	0.81	0.60	1.26	0.42	0.19	0.27	3.05	1.61
Solyc11g0	0.15	0.19	0.37	0.55	0.70	0.41	0.42	-1.25
Solyc12g0	0.14	0.30	0.14	0.47	0.61	0.41	0.40	-1.34
Solyc08g0	0.36	0.23	0.20	0.72	0.73	0.62	0.38	-1.38
Solyc10g0	0	0.05	0.09	0	0	0	476.24	8.90
Solyc04g0	0	0.06	0	0.23	0.44	0.12	0.07	-3.80
Solyc07g0	0.23	0.15	0.12	0.43	0.29	0.42	0.44	-1.19
Solyc06g0	0.10	0.08	0.11	0.04	0.05	0.03	2.48	1.31
Solyc06g0	0	0	0	0.11	0	0.12	0.00	-9.59
Solyc03g0	0.81	1.01	0.91	0	0.10	0	27.60	4.79
Solyc03g0	0.51	0.47	0.62	0.21	0.28	0.29	2.05	1.03
Solyc01g0	0	0	0	0.11	0.06	0.05	0.00	-9.50
Solyc08g0	0	0	0	0.11	0	0.11	0.00	-9.52
Solyc02g0	0.68	0.56	0.73	0.27	0.07	0.12	4.24	2.09
Solyc11g0	0.07	0	0.08	0	0	0	482.30	8.91
Solyc03g0	0.53	0.12	0.24	0	0	0	2979.20	11.54
Solyc01g0	0	0	0	0.03	0.03	0.05	0.00	-8.60
Solyc03g0	0.08	0.14	0.13	0	0.02	0.04	5.49	2.46
Solyc10g0	0.09	0.07	0.16	0.15	0.64	0.38	0.28	-1.85
Solyc05g0	0.00	0.03	0.04	0.37	0.09	0.10	0.13	-2.93
Solyc01g1	1.03	0.96	0.89	0.32	0.63	0.42	2.11	1.08
Solyc11g0	0	0	0	0.10	0.09	0	0.00	-9.30
Solyc12g1	0	0	0	0.53	0.31	0.79	0.00	-12.42
Solyc01g0	0.46	0.37	0	0	0	0	2748.30	11.42
Solyc02g0	0	0	0	0.02	0.02	0.07	0.00	-8.58
Solyc09g0	0.11	0.16	0.18	0.45	0.33	0.40	0.39	-1.37
Solyc01g0	0.43	0.21	0.33	0.13	0.04	0.06	4.29	2.10
Solyc02g0	0.46	0.63	0.46	0.33	0.12	0.17	2.46	1.30
Solyc06g0	0.05	0.16	0	0.29	0.53	0.32	0.19	-2.40
Solyc08g0	0.07	0	0.12	0.37	0.63	0.26	0.15	-2.74
Solyc08g0	0.89	1.60	1.06	1.60	3.32	2.26	0.49	-1.02
Solyc12g0	0	0	0	0.08	0.03	0.06	0.00	-9.16
Solyc09g0	1.51	1.50	2.43	3.16	4.28	3.76	0.49	-1.04
Solyc09g0	0.05	0.03	0.05	0	0	0	460.47	8.85
Solyc06g0	0	0	0	0.05	0.14	0	0.00	-9.26
Solyc07g0	0.16	0.07	0.10	0.40	0.25	0.45	0.30	-1.72
Solyc10g0	0	0.05	0.05	0.11	0.32	0.22	0.15	-2.72
Solyc06g0	1.28	1.33	1.43	0.22	0.57	0.87	2.45	1.29
Solyc01g0	0	0.09	0	0.12	0.47	0.30	0.10	-3.33
Solyc02g0	0.10	0.07	0.13	0.31	0.30	0.28	0.34	-1.54
Solyc12g0	0.15	0.17	0	1.09	0.97	0.66	0.12	-3.08
Solyc12g0	1.03	1.09	1.02	0.31	0.34	0.22	3.59	1.84
Solyc12g0	0.08	0	0	0.12	0.22	0.31	0.12	-3.02
Solyc09g0	0	0	0.06	0.15	0.32	0.16	0.10	-3.32
Solyc04g0	0.14	0.08	0.20	0.34	0.49	0.38	0.34	-1.54
Solyc03g1	0	0	0.06	0.15	0.20	0.23	0.11	-3.19
Solyc03g1	1.54	0.78	1.27	0.34	0.45	0.73	2.37	1.24
Solyc12g0	0.19	0	0.31	0.48	0.86	1.02	0.21	-2.24

Solyc09g0	0	0.07	0	0.40	0.13	0.13	0.10	-3.31
Solyc06g0	0.20	0.10	0.16	0.48	0.36	0.24	0.42	-1.25
Solyc01g0	0.11	0.40	0.05	0	0	0.04	12.75	3.67
Solyc01g1	1.95	1.60	1.13	0.52	0.79	0.87	2.14	1.10
Solyc02g0	0.10	0.25	0.18	0.40	0.39	0.39	0.44	-1.18
Solyc06g0	0	0.19	0	0.52	0.71	0.44	0.11	-3.16
Solyc02g0	0.36	0.32	0	0	0	0	2267.03	11.15
Solyc08g0	0.31	0.53	0.38	0.14	0.11	0.19	2.74	1.45
Solyc04g0	0.27	0.31	0.34	0.72	0.53	0.67	0.48	-1.05
Solyc08g0	0.24	0.64	0.79	1.08	0.99	1.45	0.48	-1.07
Solyc03g0	0.44	1.15	0.94	0.29	0	0.31	4.23	2.08
Solyc06g0	1.32	1.07	0.50	0.39	0.17	0.34	3.20	1.68
Solyc11g0	0	0	0	0.18	0.05	0.07	0.00	-9.98
Solyc10g0	0	0	0	0.07	0	0.11	0.00	-9.22
Solyc09g0	0.24	0.40	0.31	0.09	0.20	0.09	2.52	1.33
Solyc10g0	0.15	0.00	0.30	0.73	0.44	0.69	0.24	-2.04
Solyc12g0	0	0	0	0.38	0.30	0.34	0.00	-11.72
Solyc05g0	0	0	0	0.07	0	0.07	0.00	-8.91
Solyc06g0	0.13	0.14	0.42	1.00	0.60	0.46	0.33	-1.58
Solyc07g0	0.01	0	0.02	0.08	0.03	0.09	0.18	-2.49
Solyc01g0	0.09	0	0.11	0.61	0.21	0.22	0.20	-2.35
Solyc06g0	1.07	0.57	0.81	1.63	2.56	1.84	0.40	-1.30
Solyc03g0	0.51	0.27	0.30	1.42	0.85	0.95	0.34	-1.57
Solyc04g0	0.41	0.16	0.47	0.19	0.15	0.11	2.30	1.20
Solyc03g1	0.21	0.41	0.72	1.06	1.23	1.09	0.40	-1.33
Solyc03g1	0.15	0	0.09	0.20	0.40	0.41	0.24	-2.05
Solyc01g0	0	0.22	0.08	0.32	0.52	0.47	0.23	-2.09
Solyc04g0	0.33	0.32	0	0	0	0	2167.20	11.08
Solyc05g0	1.10	1.64	1.35	3.58	4.02	2.67	0.40	-1.33
Solyc01g1	0.21	0.09	0.16	0.32	0.46	0.37	0.40	-1.31
Solyc03g0	3.74	3.15	3.96	1.42	2.11	1.03	2.38	1.25
Solyc06g0	0.72	0.82	1.01	1.77	1.89	2.08	0.44	-1.17
Solyc04g0	0	0	0	0	0.10	0.02	0.00	-8.72
Solyc09g0	0.11	0.06	0.13	0.27	0.14	0.31	0.42	-1.26
Solyc11g0	0.43	0.75	0.47	0.23	0.13	0.25	2.70	1.43
Solyc01g0	0.30	0.35	0.27	0.97	0.44	0.72	0.43	-1.23
Solyc06g0	0.10	0.12	0.03	0.21	0.17	0.23	0.41	-1.27
Solyc09g0	0.15	0	0.23	0.43	0.60	0.52	0.24	-2.04
Solyc01g0	0.16	0.06	0.16	0.25	0.45	0.34	0.37	-1.43
Solyc02g0	0.47	0.49	0.26	0.76	0.93	1.13	0.43	-1.20
Solyc09g0	0.99	0.68	0.82	1.41	2.06	1.67	0.49	-1.04
Solyc09g0	0.30	0.22	0.23	0.74	0.60	0.52	0.40	-1.32
Solyc07g0	0.17	0.16	0.20	0	0	0	1759.77	10.78
Solyc06g0	0	0	0	0.05	0.03	0.06	0.00	-8.89
Solyc09g0	0.03	0.02	0.01	0	0	0	203.60	7.67
Solyc01g0	0.05	0.06	0.03	0.21	0.16	0.04	0.33	-1.61
Solyc08g0	0.38	0.71	0.43	1.00	1.01	1.39	0.45	-1.17
Solyc06g0	0.16	0.13	0.10	0.43	0.23	0.39	0.37	-1.42
Solyc09g0	0.10	0.40	0.28	0.75	0.46	0.82	0.38	-1.39
Solyc09g0	0.32	0.14	0.24	0.45	0.62	0.69	0.39	-1.34
Solyc09g0	0	0.54	0.44	0	0	0	3285.76	11.68

Solyc10g0	0.35	0.60	0.45	0.19	0.17	0.25	2.30	1.20
Solyc01g1	0.33	0.09	0.51	0.07	0.06	0.09	4.26	2.09
Solyc02g0	0	0	0	0.10	0.39	0.26	0.00	-11.31
Solyc07g0	0	0	0	0.04	0.14	0.12	0.00	-9.97
Solyc03g0	0.55	0.51	0	0.06	0	0	16.58	4.05
Solyc11g0	2.10	1.45	2.09	3.22	4.70	4.02	0.47	-1.08
Solyc08g0	0.07	0.07	0.16	0.33	0.50	0.19	0.29	-1.77
Solyc04g0	1.25	1.82	1.53	4.29	2.48	2.81	0.48	-1.06
Solyc11g0	0.03	0	0	0.12	0.14	0.17	0.08	-3.74
Solyc04g0	0.03	0	0.02	0.16	0.08	0.05	0.20	-2.32
Solyc08g0	0.04	0.06	0.12	0.21	0.18	0.17	0.39	-1.34
Solyc12g0	0.05	0.03	0.07	0	0	0	485.70	8.92
Solyc02g0	0.13	0.39	0.14	0	0.02	0.11	5.03	2.33
Solyc10g0	0.02	0	0.11	0	0	0	436.13	8.77
Solyc02g0	1.52	0.83	1.42	0.60	0.76	0.42	2.11	1.08
Solyc08g0	0	0	0	0.05	0.08	0.03	0.00	-9.01
Solyc03g1	0.26	0.35	0.33	0.91	0.76	0.49	0.44	-1.19
Solyc01g0	0	0.04	0.06	0.17	0.22	0.15	0.19	-2.40
Solyc12g0	0	0	0	0.22	0	0.15	0.00	-10.26
Solyc04g0	0.30	0.44	0	0	0	0	2447.39	11.26
Solyc06g0	0	0	0	0.04	0.07	0	0.00	-8.61
Solyc09g0	0	0	0	0.06	0.04	0	0.00	-8.38
Solyc04g0	0.09	0.15	0.08	0.24	0.27	0.16	0.49	-1.04
Solyc10g0	0.13	0	0.17	0.35	0.41	0.26	0.30	-1.75
Solyc05g0	0.10	0	0	0.25	0.22	0.27	0.14	-2.83
Solyc08g0	0	0	0	0.04	0.03	0.10	0.00	-9.13
Solyc06g0	0.19	0.28	0.16	0.52	0.46	0.41	0.45	-1.14
Solyc01g0	4.03	3.50	17.73	2.36	2.53	2.18	3.58	1.84
Solyc09g0	0.02	0.03	0	0.10	0.05	0.07	0.22	-2.21
Solyc10g0	0.07	0.05	0.12	0.27	0.21	0.21	0.35	-1.51
Solyc01g0	0.93	1.06	0.61	1.72	1.91	2.09	0.45	-1.14
Solyc01g1	0	0	0.01	0.05	0.04	0.04	0.11	-3.23
Solyc07g0	0.84	0.69	0.71	0.08	0.37	0.49	2.40	1.27
Solyc06g0	0.08	0	0	0.17	0.44	0.30	0.08	-3.56
Solyc11g0	0	0	0	0	0.03	0.18	0.00	-9.45
Solyc06g0	0	0	0	0.01	0.09	0	0.00	-8.39
Solyc02g0	0	0	0	0.46	0.04	0.17	0.00	-11.14
Solyc09g0	0	0	0	0.37	0	0.52	0.00	-11.54
Solyc11g0	0	0	0	0.12	0	0.18	0.00	-9.96
Solyc04g0	0.15	0.14	0.08	0.25	0.25	0.80	0.28	-1.84
Solyc08g0	0	0	0	0.02	0.07	0.13	0.00	-9.51
Solyc01g1	0	0	0	0.19	0.04	0	0.00	-9.56
Solyc07g0	0	0	0	0.07	0.10	0.06	0.00	-9.63
Solyc06g0	0.11	0.09	0.17	0.24	0.44	0.28	0.39	-1.37
Solyc02g0	0.11	0.14	0	0.46	0.38	0.23	0.23	-2.09
Solyc07g0	1.70	1.13	1.43	0.56	0.88	0.49	2.22	1.15
Solyc06g0	0	0.05	0.06	0	0	0	379.31	8.57
Solyc03g0	1.11	1.24	1.07	2.69	2.25	2.22	0.48	-1.07
Solyc05g0	0	0	0	0.03	0.07	0.04	0.00	-8.79
Solyc01g1	0.20	0.16	0.15	0.45	0.28	0.34	0.47	-1.09
Solyc03g0	0.06	0.04	0.03	0.18	0.08	0.13	0.35	-1.52

Solyc02g0	0	0	0	0.11	0.08	0	0.00	-9.32
Solyc06g0	0	0	0	0	4.87	5.61	0.00	-15.09
Solyc08g0	0.26	0.19	0.38	0.08	0.04	0.13	3.30	1.72
Solyc12g0	0	0	0	0.14	0.28	0	0.00	-10.45
Solyc11g0	0.77	0.97	0.51	0.12	0.08	0.28	4.59	2.20
Solyc08g0	0	0.08	0.01	0	0	0	311.75	8.28
Solyc07g0	0.04	0.05	0	0	0	0	314.95	8.30
Solyc06g0	0	0.04	0.45	0	0	0	1647.59	10.69
Solyc08g0	0.18	0.15	0.22	0.09	0.08	0.07	2.33	1.22
Solyc02g0	0	0.04	0.09	0.15	0.34	0.17	0.20	-2.30
Solyc05g0	0.10	0.39	0.21	0.60	0.51	0.41	0.46	-1.13
Solyc04g0	0.23	0.10	0.13	0.40	0.39	0.27	0.43	-1.22
Solyc11g0	0	0.04	0.06	0.19	0.11	0.07	0.26	-1.97
Solyc09g0	0.07	0	0	0.10	0.14	0.18	0.16	-2.61
Solyc10g0	0.36	0.35	0.10	0.06	0.09	0.09	3.52	1.82
Solyc01g0	0.12	0.11	0.06	0.24	0.26	0.17	0.42	-1.24
Solyc02g0	0.29	0.11	0.30	0	0	0.11	6.38	2.67
Solyc04g0	0.11	0.10	0.11	0	0	0	1096.07	10.10

pval	qval	regulation
0	0	down
0	0	down
0	0	up
0	0	down
0	0	down
0	0	down
0	0	down
0	0	down
0	0	down
0	0	down
0	0	up
0	0	down
0	0	up
0	0	down
0	0	down
0	0	up
0	0	down
0	0	down
0	0	up
0	0	up
0	0	down
0	0	down
0	0	down
0	0	down
0	0	down
0	0	down
0	0	down
0	0	down
0	0	down
0	0	down
0	0	down
0	0	up
0	0	up
0	0	up
0	0	up
0	0	down
0	0	up
0	0	up
0	0	down
0	0	up
0	0	down
0	0	down
0	0	down
0.00	0.00	up
0.00	0.00	down
0.00	0.00	down
0.00	0.00	up
0.00	0.00	up
0.00	0.00	down

0.00	0.00	down
0.00	0.00	down
0.00	0.00	down
0.00	0.00	down
0.00	0.00	up
0.00	0.00	down
0.00	0.00	down
0.00	0.00	down
0.00	0.00	down
0.00	0.00	up
0.00	0.00	down
0.00	0.00	down
0.00	0.00	up
0.00	0.00	down
0.00	0.00	up
0.00	0.00	up
0.00	0.00	down
0.00	0.00	down
0.00	0.00	up
0.00	0.00	down
0.00	0.00	up
0.00	0.00	up
0.00	0.00	down
0.00	0.00	down
0.00	0.00	down
0.00	0.00	down
0.00	0.00	up
0.00	0.00	down
0.00	0.00	up
0.00	0.00	down
0.00	0.00	down
0.00	0.00	up
0.00	0.00	down
0.00	0.00	up
0.00	0.00	down
0.00	0.00	down
0.00	0.00	down
0.00	0.00	up
0.00	0.00	down
0.00	0.00	down
0.00	0.00	down
0.00	0.00	down
0.00	0.00	up
0.00	0.00	down
0.00	0.00	down
0.00	0.00	down
0.00	0.00	down
0.00	0.00	down
0.00	0.00	down
0.00	0.00	down
0.00	0.00	up

0.00	0.00	down
0.00	0.00	down
0.00	0.00	up
0.00	0.00	down
0.00	0.00	down
0.00	0.00	down
0.00	0.00	down
0.00	0.00	down
0.00	0.00	down
0.00	0.00	down
0.00	0.00	down
0.00	0.00	down
0.00	0.00	up
0.00	0.00	down
0.00	0.00	down
0.00	0.00	down
0.00	0.00	up
0.00	0.00	up
0.00	0.00	up
0.00	0.00	up
0.00	0.00	down
0.00	0.00	down
0.00	0.00	down
0.00	0.00	down
0.00	0.00	down
0.00	0.00	down
0.00	0.00	down
0.00	0.00	up
0.00	0.00	up
0.00	0.00	up
0.00	0.00	down
0.00	0.00	down
0.00	0.00	down
0.00	0.00	up
0.00	0.00	up
0.00	0.00	up
0.00	0.00	up
0.00	0.00	up
0.00	0.00	down
0.00	0.00	down
0.00	0.00	down
0.00	0.00	down
0.00	0.00	down
0.00	0.00	up
0.00	0.00	down
0.00	0.00	up
0.00	0.00	down
0.00	0.00	up
0.00	0.00	down
0.00	0.00	down
0.00	0.00	up

0.00	0.00	down
0.00	0.00	down
0.00	0.00	down
0.00	0.00	up
0.00	0.00	down
0.00	0.00	up
0.00	0.00	down
0.00	0.00	down
0.00	0.00	down
0.00	0.00	down
0.00	0.00	down
0.00	0.00	down
0.00	0.00	down
0.00	0.00	down
0.00	0.00	down
0.00	0.00	down
0.00	0.00	down
0.00	0.00	up
0.00	0.00	down
0.00	0.00	down
0.00	0.00	up
0.00	0.00	up
0.00	0.00	up
0.00	0.00	down
0.00	0.00	down
0.00	0.00	down
0.00	0.00	down
0.00	0.00	down
0.00	0.00	down
0.00	0.00	down
0.00	0.00	down
0.00	0.00	up
0.00	0.00	up
0.00	0.00	down
0.00	0.00	up
0.00	0.00	down
0.00	0.00	down
0.00	0.00	down
0.00	0.00	down
0.00	0.00	down
0.00	0.00	down
0.00	0.00	down
0.00	0.00	up
0.00	0.00	down
0.00	0.00	down
0.00	0.00	down
0.00	0.00	down
0.00	0.00	up
0.00	0.00	down
0.00	0.00	down
0.00	0.00	down

0.00	0.00	up
0.00	0.00	down
0.00	0.00	up
0.00	0.00	down
0.00	0.00	down
0.00	0.00	up
0.00	0.00	up
0.00	0.00	up
0.00	0.00	down
0.00	0.00	down
0.00	0.00	down
0.00	0.00	down
0.00	0.00	down
0.00	0.00	down
0.00	0.00	up
0.00	0.00	up
0.00	0.00	up
0.00	0.00	down
0.00	0.00	up
0.00	0.00	down
0.00	0.00	down
0.00	0.00	down
0.00	0.00	up
0.00	0.00	up
0.00	0.00	up
0.00	0.00	down
0.00	0.00	up
0.00	0.00	up
0.00	0.00	down
0.00	0.00	down
0.00	0.00	down
0.00	0.00	up
0.00	0.00	down
0.00	0.00	down
0.00	0.00	down
0.00	0.00	up
0.00	0.00	down
0.00	0.00	down
0.00	0.00	down
0.00	0.00	down
0.00	0.00	down
0.00	0.00	down
0.00	0.00	up
0.00	0.00	up
0.00	0.00	down
0.00	0.00	down
0.00	0.00	down
0.00	0.00	down
0.00	0.00	up
0.00	0.00	up
0.00	0.00	up

0.00	0.00	down
0.00	0.00	up
0.00	0.00	down
0.00	0.00	down
0.00	0.00	up
0.00	0.00	down
0.00	0.00	up
0.00	0.00	down
0.00	0.00	down
0.00	0.00	down
0.00	0.00	up
0.00	0.00	down
0.00	0.00	up
0.00	0.00	down
0.00	0.00	up
0.00	0.00	up
0.00	0.00	down
0.00	0.00	down
0.00	0.00	down
0.00	0.00	down
0.00	0.00	down
0.00	0.00	down
0.00	0.00	up
0.00	0.00	down
0.00	0.00	up
0.00	0.00	down
0.00	0.00	down
0.00	0.00	down
0.00	0.00	up
0.00	0.00	down
0.00	0.00	up
0.00	0.00	down
0.00	0.00	down
0.00	0.00	down
0.00	0.00	down
0.00	0.00	down
0.00	0.00	up
0.00	0.00	down
0.00	0.00	down
0.00	0.00	down
0.00	0.00	down
0.00	0.00	down
0.00	0.00	down
0.00	0.00	down
0.00	0.00	down
0.00	0.00	down
0.00	0.00	down
0.00	0.00	down
0.00	0.00	down
0.00	0.00	down
0.00	0.00	down

0.00	0.00	down
0.00	0.00	down
0.00	0.00	down
0.00	0.00	down
0.00	0.00	down
0.00	0.00	up
0.00	0.00	up
0.00	0.00	up
0.00	0.00	down
0.00	0.00	down
0.00	0.00	down
0.00	0.00	down
0.00	0.00	down
0.00	0.00	up
0.00	0.00	down
0.00	0.00	down
0.00	0.00	down
0.00	0.00	up
0.00	0.00	down
0.00	0.00	up
0.00	0.00	down
0.00	0.00	down
0.00	0.00	up
0.00	0.00	down
0.00	0.00	down
0.00	0.00	up
0.00	0.00	down
0.00	0.00	down
0.00	0.00	up
0.00	0.00	down
0.00	0.00	down
0.00	0.00	down
0.00	0.00	down
0.00	0.00	down
0.00	0.00	down
0.00	0.00	down
0.00	0.00	down
0.00	0.00	down
0.00	0.00	down
0.00	0.00	up
0.00	0.00	down
0.00	0.00	down
0.00	0.00	up
0.00	0.00	down
0.00	0.00	down
0.00	0.00	down
0.00	0.00	down
0.00	0.00	down
0.00	0.00	down
0.00	0.00	up
0.00	0.00	down

0.00	0.00	up
0.00	0.00	up
0.00	0.00	up
0.00	0.00	down
0.00	0.00	down
0.00	0.00	down
0.00	0.00	up
0.00	0.00	down
0.00	0.00	down
0.00	0.00	up
0.00	0.00	up
0.00	0.00	down
0.00	0.00	up
0.00	0.00	down
0.00	0.00	down
0.00	0.00	down
0.00	0.00	down
0.00	0.00	up
0.00	0.00	down
0.00	0.00	down
0.00	0.00	up
0.00	0.00	up
0.00	0.00	down
0.00	0.00	down
0.00	0.00	down
0.00	0.00	down
0.00	0.00	down
0.00	0.00	down
0.00	0.00	down
0.00	0.00	down
0.00	0.00	down
0.00	0.00	down
0.00	0.00	down
0.00	0.00	down
0.00	0.00	down
0.00	0.00	down
0.00	0.00	up
0.00	0.00	down
0.00	0.00	down
0.00	0.00	down
0.00	0.00	down
0.00	0.00	down
0.00	0.00	down
0.00	0.00	down
0.00	0.00	down
0.00	0.00	down
0.00	0.00	up
0.00	0.00	up
0.00	0.00	down
0.00	0.00	down
0.00	0.00	down

0.00	0.00	up
0.00	0.00	up
0.00	0.00	down
0.00	0.00	down
0.00	0.00	up
0.00	0.00	up
0.00	0.00	up
0.00	0.00	up
0.00	0.00	down
0.00	0.00	down
0.00	0.00	down
0.00	0.00	up
0.00	0.00	down
0.00	0.00	up
0.00	0.00	down
0.00	0.00	down
0.00	0.00	down
0.00	0.00	up
0.00	0.00	up
0.00	0.00	down
0.00	0.00	down
0.00	0.00	down
0.00	0.00	up
0.00	0.00	up
0.00	0.00	up
0.00	0.00	down
0.00	0.00	down
0.00	0.00	down
0.00	0.00	down
0.00	0.00	down
0.00	0.00	down
0.00	0.00	down
0.00	0.00	down
0.00	0.00	down
0.00	0.00	down
0.00	0.00	down
0.00	0.00	down
0.00	0.00	down
0.00	0.00	down
0.00	0.00	down
0.00	0.00	down
0.00	0.00	down
0.00	0.00	down
0.00	0.00	down
0.00	0.00	down
0.00	0.00	down
0.00	0.00	up
0.00	0.00	up
0.00	0.00	down
0.00	0.00	up
0.00	0.00	up

0.00	0.00	down
0.00	0.00	up
0.00	0.00	up
0.00	0.00	down
0.00	0.00	up
0.00	0.00	up
0.00	0.00	up
0.00	0.00	down
0.00	0.00	up
0.00	0.00	up
0.00	0.00	down
0.00	0.00	down
0.00	0.00	down
0.00	0.00	down
0.00	0.00	up
0.00	0.00	down
0.00	0.00	down
0.00	0.00	up
0.00	0.00	up
0.00	0.00	down
0.00	0.00	up
0.00	0.00	down
0.00	0.00	down
0.00	0.00	down
0.00	0.00	down
0.00	0.00	up
0.00	0.00	up
0.00	0.00	down
0.00	0.00	up
0.00	0.00	down
0.00	0.00	up
0.00	0.00	down
0.00	0.00	down
0.00	0.00	down
0.00	0.00	down
0.00	0.00	down
0.00	0.00	down
0.00	0.00	down
0.00	0.00	up
0.00	0.00	up
0.00	0.00	down
0.00	0.00	down
0.00	0.00	down
0.00	0.00	down
0.00	0.00	down
0.00	0.00	up
0.00	0.00	up
0.00	0.00	up
0.00	0.00	down
0.00	0.00	up
0.00	0.00	up

0.00	0.00	down
0.00	0.00	down
0.00	0.00	down
0.00	0.00	up
0.00	0.00	up
0.00	0.00	down
0.00	0.00	down
0.00	0.00	down
0.00	0.00	up
0.00	0.00	up
0.00	0.00	down
0.00	0.00	down
0.00	0.00	down
0.00	0.00	down
0.00	0.00	up
0.00	0.00	down
0.00	0.00	up
0.00	0.00	up
0.00	0.00	down
0.00	0.00	up
0.00	0.00	up
0.00	0.00	down
0.00	0.00	up
0.00	0.00	up
0.00	0.00	down
0.00	0.00	down
0.00	0.00	up
0.00	0.00	down
0.00	0.00	down
0.00	0.00	down
0.00	0.00	up
0.00	0.00	down
0.00	0.00	down
0.00	0.00	down
0.00	0.00	up
0.00	0.00	up
0.00	0.00	down
0.00	0.00	down
0.00	0.00	up
0.00	0.00	down
0.00	0.00	up
0.00	0.00	down
0.00	0.00	down
0.00	0.00	down
0.00	0.00	up
0.00	0.00	up
0.00	0.00	down
0.00	0.00	down
0.00	0.00	down
0.00	0.00	down
0.00	0.00	down

0.00	0.00	down
0.00	0.00	up
0.00	0.00	down
0.00	0.00	down
0.00	0.00	down
0.00	0.00	up
0.00	0.00	down
0.00	0.00	up
0.00	0.00	down
0.00	0.00	down
0.00	0.00	down
0.00	0.00	down
0.00	0.00	down
0.00	0.00	up
0.00	0.00	down
0.00	0.00	down
0.00	0.00	down
0.00	0.00	down
0.00	0.00	down
0.00	0.00	down
0.00	0.00	down
0.00	0.00	down
0.00	0.00	up
0.00	0.00	down
0.00	0.00	down
0.00	0.00	down
0.00	0.00	down
0.00	0.00	down
0.00	0.00	down
0.00	0.00	down
0.00	0.00	up
0.00	0.00	down
0.00	0.00	up
0.00	0.00	up
0.00	0.00	down
0.00	0.00	down
0.00	0.00	down
0.00	0.00	down
0.00	0.00	up
0.00	0.00	up
0.00	0.00	down
0.00	0.00	down
0.00	0.00	down
0.00	0.00	up
0.00	0.00	down
0.00	0.00	down
0.00	0.00	up
0.00	0.00	down
0.00	0.00	down
0.00	0.00	down
0.00	0.00	down

0.00	0.00	up
0.00	0.00	up
0.00	0.00	down
0.00	0.00	down
0.00	0.00	up
0.00	0.00	up
0.00	0.00	up
0.00	0.00	down
0.00	0.00	down
0.00	0.00	down
0.00	0.00	down
0.00	0.00	up
0.00	0.00	up
0.00	0.00	up
0.00	0.00	down
0.00	0.00	down
0.00	0.00	down
0.00	0.00	down
0.00	0.00	down
0.00	0.00	up
0.00	0.00	down
0.00	0.00	up
0.00	0.00	down
0.00	0.00	up
0.00	0.00	up
0.00	0.00	down
0.00	0.00	down
0.00	0.00	up
0.00	0.00	up
0.00	0.00	down
0.00	0.00	up
0.00	0.00	down
0.00	0.00	down
0.00	0.00	down
0.00	0.00	up
0.00	0.00	down
0.00	0.00	up
0.00	0.00	up
0.00	0.00	up
0.00	0.00	up
0.00	0.00	down
0.00	0.00	up
0.00	0.00	down
0.00	0.00	down
0.00	0.00	down
0.00	0.00	up
0.00	0.00	up
0.00	0.00	down
0.00	0.00	down
0.00	0.00	down
0.00	0.00	down

0.00	0.00	down
0.00	0.00	up
0.00	0.00	up
0.00	0.00	up
0.00	0.00	down
0.00	0.00	up
0.00	0.00	down
0.00	0.00	down
0.00	0.00	down
0.00	0.00	down
0.00	0.00	down
0.00	0.00	up
0.00	0.00	down
0.00	0.00	down
0.00	0.00	up
0.00	0.00	down
0.00	0.00	down
0.00	0.00	down
0.00	0.00	down
0.00	0.00	up
0.00	0.00	up
0.00	0.00	down
0.00	0.00	up
0.00	0.00	up
0.00	0.00	down
0.00	0.00	down
0.00	0.00	up
0.00	0.00	down
0.00	0.00	up
0.00	0.00	down
0.00	0.00	up
0.00	0.00	down
0.00	0.00	down
0.00	0.00	down
0.00	0.00	down
0.00	0.00	down
0.00	0.00	down
0.00	0.00	down
0.00	0.00	down
0.00	0.00	up
0.00	0.00	up
0.00	0.00	down
0.00	0.00	up
0.00	0.00	up
0.00	0.00	down
0.00	0.00	up
0.00	0.00	down
0.00	0.00	down
0.00	0.00	up
0.00	0.00	down
0.00	0.00	up

0.00	0.00	down
0.00	0.00	down
0.00	0.00	down
0.00	0.00	up
0.00	0.00	up
0.00	0.00	up
0.00	0.00	up
0.00	0.00	up
0.00	0.00	down
0.00	0.00	up
0.00	0.00	down
0.00	0.00	down
0.00	0.00	down
0.00	0.00	down
0.00	0.00	down
0.00	0.00	down
0.00	0.00	up
0.00	0.00	down
0.00	0.00	up
0.00	0.00	up
0.00	0.00	down
0.00	0.00	down
0.00	0.00	down
0.00	0.00	up
0.00	0.00	down
0.00	0.00	up
0.00	0.00	up
0.00	0.00	down
0.00	0.00	down
0.00	0.00	up
0.00	0.00	down
0.00	0.00	down
0.00	0.00	up
0.00	0.00	down
0.00	0.00	down
0.00	0.00	down
0.00	0.00	down
0.00	0.00	down
0.00	0.00	down
0.00	0.00	down
0.00	0.00	down
0.00	0.00	up
0.00	0.00	down
0.00	0.00	down
0.00	0.00	down
0.00	0.00	down
0.00	0.00	up
0.00	0.00	down
0.00	0.00	down
0.00	0.00	down
0.00	0.00	down

0.00	0.00	down
0.00	0.00	down
0.00	0.00	down
0.00	0.00	down
0.00	0.00	up
0.00	0.00	down
0.00	0.00	down
0.00	0.00	down
0.00	0.00	down
0.00	0.00	up
0.00	0.00	up
0.00	0.00	down
0.00	0.00	down
0.00	0.00	up
0.00	0.00	up
0.00	0.00	down
0.00	0.00	up
0.00	0.00	up
0.00	0.00	up
0.00	0.00	down
0.00	0.00	up
0.00	0.00	down
0.00	0.00	down
0.00	0.00	down
0.00	0.00	down
0.00	0.00	down
0.00	0.00	down
0.00	0.00	down
0.00	0.00	up
0.00	0.00	down
0.00	0.00	up
0.00	0.00	down
0.00	0.00	down
0.00	0.00	up
0.00	0.00	down
0.00	0.00	up
0.00	0.00	up
0.00	0.00	down
0.00	0.00	down
0.00	0.00	down
0.00	0.00	up
0.00	0.00	up
0.00	0.00	down
0.00	0.00	up
0.00	0.00	up
0.00	0.00	down
0.00	0.00	up
0.00	0.00	down
0.00	0.00	up
0.00	0.00	down
0.00	0.00	down

0.00	0.00	down
0.00	0.00	down
0.00	0.00	down
0.00	0.00	down
0.00	0.00	down
0.00	0.00	down
0.00	0.00	up
0.00	0.00	down
0.00	0.00	up
0.00	0.00	up
0.00	0.00	down
0.00	0.00	down
0.00	0.00	up
0.00	0.00	down
0.00	0.00	down
0.00	0.00	up
0.00	0.00	up
0.00	0.00	down
0.00	0.00	up
0.00	0.00	up
0.00	0.00	down
0.00	0.00	down
0.00	0.00	down
0.00	0.00	down
0.00	0.00	up
0.00	0.00	down
0.00	0.00	down
0.00	0.00	down
0.00	0.00	down
0.00	0.00	up
0.00	0.00	down
0.00	0.00	down
0.00	0.00	down
0.00	0.00	down
0.00	0.00	up
0.00	0.00	down
0.00	0.00	up
0.00	0.00	down
0.00	0.00	down
0.00	0.00	up
0.00	0.00	down
0.00	0.00	up
0.00	0.00	down
0.00	0.00	down
0.00	0.00	down
0.00	0.00	down
0.00	0.00	up
0.00	0.00	down
0.00	0.00	up
0.00	0.00	up
0.00	0.00	down

0.00	0.00	up
0.00	0.00	up
0.00	0.00	up
0.00	0.00	up
0.00	0.00	down
0.00	0.00	down
0.00	0.00	down
0.00	0.00	down
0.00	0.00	up
0.00	0.00	up
0.00	0.00	down
0.00	0.00	up
0.00	0.00	down
0.00	0.00	down
0.00	0.00	up
0.00	0.00	down
0.00	0.00	up
0.00	0.00	up
0.00	0.00	down
0.00	0.00	up
0.00	0.00	up
0.00	0.00	down
0.00	0.00	down
0.00	0.00	down
0.00	0.00	down
0.00	0.00	up
0.00	0.00	up
0.00	0.00	down
0.00	0.00	down
0.00	0.00	down
0.00	0.00	down
0.00	0.00	down
0.00	0.00	down
0.00	0.00	up
0.00	0.00	down
0.00	0.00	up
0.00	0.00	up
0.00	0.00	up
0.00	0.00	down
0.00	0.00	down
0.00	0.00	down
0.00	0.00	down
0.00	0.00	down
0.00	0.00	down
0.00	0.00	down
0.00	0.00	up
0.00	0.00	up
0.00	0.00	down
0.00	0.00	down
0.00	0.00	up
0.00	0.00	down

0.00	0.00	down
0.00	0.00	up
0.00	0.00	down
0.00	0.00	up
0.00	0.00	up
0.00	0.00	down
0.00	0.00	down
0.00	0.00	down
0.00	0.00	up
0.00	0.00	down
0.00	0.00	up
0.00	0.00	down
0.00	0.00	down
0.00	0.00	down
0.00	0.00	down
0.00	0.00	down
0.00	0.00	up
0.00	0.00	down
0.00	0.00	up
0.00	0.00	up
0.00	0.00	down
0.00	0.00	down
0.00	0.00	up
0.00	0.00	down
0.00	0.00	down
0.00	0.00	down
0.00	0.00	down
0.00	0.00	down
0.00	0.00	down
0.00	0.00	down
0.00	0.00	down
0.00	0.00	down
0.00	0.00	down
0.00	0.00	down
0.00	0.00	down
0.00	0.00	down
0.00	0.00	down
0.00	0.00	up
0.00	0.00	down
0.00	0.00	up
0.00	0.00	down
0.00	0.00	down
0.00	0.00	up
0.00	0.00	down
0.00	0.00	down
0.00	0.00	down
0.00	0.00	down
0.00	0.00	up
0.00	0.00	up
0.00	0.00	down
0.00	0.00	down

0.00	0.00	down
0.00	0.00	up
0.00	0.00	down
0.00	0.00	up
0.00	0.00	down
0.00	0.00	down
0.00	0.00	up
0.00	0.00	down
0.00	0.00	up
0.00	0.00	down
0.00	0.00	down
0.00	0.00	up
0.00	0.00	up
0.00	0.00	down
0.00	0.00	down
0.00	0.00	down
0.00	0.00	up
0.00	0.00	up
0.00	0.00	up
0.00	0.00	down
0.00	0.00	down
0.00	0.00	up
0.00	0.00	down
0.00	0.00	down
0.00	0.00	down
0.00	0.00	down
0.00	0.00	up
0.00	0.00	up
0.00	0.00	down
0.00	0.00	down
0.00	0.00	up
0.00	0.00	down
0.00	0.00	up
0.00	0.00	down
0.00	0.00	up
0.00	0.00	up
0.00	0.00	down
0.00	0.00	up
0.00	0.00	up
0.00	0.00	up
0.00	0.00	down
0.00	0.00	down
0.00	0.00	down
0.00	0.00	up
0.00	0.00	down
0.00	0.00	down
0.00	0.00	down
0.00	0.00	up
0.00	0.00	up
0.00	0.00	up
0.00	0.00	down

0.00	0.00	down
0.00	0.00	down
0.00	0.00	down
0.00	0.00	up
0.00	0.00	down
0.00	0.00	up
0.00	0.00	down
0.00	0.00	down
0.00	0.00	down
0.00	0.00	down
0.00	0.00	down
0.00	0.00	down
0.00	0.00	down
0.00	0.00	down
0.00	0.00	down
0.00	0.00	up
0.00	0.00	down
0.00	0.00	up
0.00	0.00	down
0.00	0.00	down
0.00	0.00	down
0.00	0.00	down
0.00	0.00	down
0.00	0.00	down
0.00	0.00	up
0.00	0.00	down
0.00	0.00	down
0.00	0.00	down
0.00	0.00	down
0.00	0.00	down
0.00	0.00	down
0.00	0.00	up
0.00	0.00	down
0.00	0.00	up
0.00	0.00	down
0.00	0.00	down
0.00	0.00	down
0.00	0.00	up
0.00	0.00	up
0.00	0.00	up
0.00	0.00	up
0.00	0.00	down
0.00	0.00	down
0.00	0.00	down
0.00	0.00	down
0.00	0.00	up
0.00	0.00	down
0.00	0.00	down
0.00	0.00	down
0.00	0.00	down
0.00	0.00	up

0.00	0.00	up
0.00	0.00	up
0.00	0.00	up
0.00	0.00	down
0.00	0.00	down
0.00	0.00	up
0.00	0.00	up
0.00	0.00	up
0.00	0.00	down
0.00	0.00	up
0.00	0.00	down
0.00	0.00	down
0.00	0.00	down
0.00	0.00	down
0.00	0.00	down
0.00	0.00	down
0.00	0.00	down
0.00	0.00	down
0.00	0.00	down
0.00	0.00	down
0.00	0.00	up
0.00	0.00	down
0.00	0.00	down
0.00	0.00	down
0.00	0.00	down
0.00	0.00	up
0.00	0.00	down
0.00	0.00	down
0.00	0.00	down
0.00	0.00	down
0.00	0.00	up
0.00	0.00	down
0.00	0.00	up
0.00	0.00	up
0.00	0.00	down
0.00	0.00	down
0.00	0.00	down
0.00	0.00	down
0.00	0.00	down
0.00	0.00	down
0.00	0.00	down
0.00	0.00	down
0.00	0.00	up
0.00	0.00	down
0.00	0.00	down
0.00	0.00	down
0.00	0.00	down
0.00	0.00	up
0.00	0.00	down
0.00	0.00	down
0.00	0.00	up

0.00	0.00	down
0.00	0.00	up
0.00	0.00	down
0.00	0.00	down
0.00	0.00	down
0.00	0.00	down
0.00	0.00	up
0.00	0.00	up
0.00	0.00	down
0.00	0.00	down
0.00	0.00	down
0.00	0.00	up
0.00	0.00	down
0.00	0.00	down
0.00	0.00	up
0.00	0.00	up
0.00	0.00	up
0.00	0.00	down
0.00	0.00	down
0.00	0.00	down
0.00	0.00	up
0.00	0.00	down
0.00	0.00	down
0.00	0.00	down
0.00	0.00	down
0.00	0.00	up
0.00	0.00	up
0.00	0.00	down
0.00	0.00	down
0.00	0.00	down
0.00	0.00	up
0.00	0.00	down
0.00	0.00	down
0.00	0.00	down
0.00	0.00	up
0.00	0.00	down
0.00	0.00	down
0.00	0.00	up
0.00	0.00	down
0.00	0.00	up
0.00	0.00	down
0.00	0.00	down
0.00	0.00	down
0.00	0.00	down
0.00	0.00	up
0.00	0.00	up
0.00	0.00	up
0.00	0.00	down
0.00	0.00	down
0.00	0.00	down
0.00	0.00	up

0.00	0.00	down
0.00	0.00	up
0.00	0.00	down
0.00	0.00	up
0.00	0.00	up
0.00	0.00	down
0.00	0.00	up
0.00	0.00	down
0.00	0.00	up
0.00	0.00	up
0.00	0.00	up
0.00	0.00	up
0.00	0.00	up
0.00	0.00	up
0.00	0.00	down
0.00	0.00	up
0.00	0.00	down
0.00	0.00	down
0.00	0.00	down
0.00	0.00	down
0.00	0.00	down
0.00	0.00	down
0.00	0.00	down
0.00	0.00	down
0.00	0.00	down
0.00	0.00	down
0.00	0.00	down
0.00	0.00	down
0.00	0.00	down
0.00	0.00	up
0.00	0.00	down
0.00	0.00	up
0.00	0.00	down
0.00	0.00	down
0.00	0.00	down
0.00	0.00	down
0.00	0.00	down
0.00	0.00	up
0.00	0.00	up
0.00	0.00	down
0.00	0.00	down
0.00	0.00	down
0.00	0.00	down
0.00	0.00	down
0.00	0.00	down
0.00	0.00	down
0.00	0.00	down
0.00	0.00	up
0.00	0.00	down
0.00	0.00	up
0.00	0.00	up

0.00	0.00	down
0.00	0.00	up
0.00	0.00	up
0.00	0.00	up
0.00	0.00	up
0.00	0.00	up
0.00	0.01	down
0.00	0.01	down
0.00	0.01	down
0.00	0.01	down
0.00	0.01	down
0.00	0.01	up
0.00	0.01	down
0.00	0.01	down
0.00	0.01	down
0.00	0.01	up
0.00	0.01	down
0.00	0.01	up
0.00	0.01	up
0.00	0.01	up
0.00	0.01	up
0.00	0.01	up
0.00	0.01	down
0.00	0.01	up
0.00	0.01	down
0.00	0.01	down
0.00	0.01	down
0.00	0.01	down
0.00	0.01	up
0.00	0.01	down
0.00	0.01	down
0.00	0.01	down
0.00	0.01	down
0.00	0.01	down
0.00	0.01	down
0.00	0.01	down
0.00	0.01	down
0.00	0.01	up
0.00	0.01	up
0.00	0.01	down
0.00	0.01	up
0.00	0.01	down
0.00	0.01	down
0.00	0.01	down
0.00	0.01	down
0.00	0.01	down
0.00	0.01	up
0.00	0.01	down
0.00	0.01	down
0.00	0.01	up
0.00	0.01	down

0.00	0.01	up
0.00	0.01	up
0.00	0.01	down
0.00	0.01	down
0.00	0.01	down
0.00	0.01	up
0.00	0.01	down
0.00	0.01	up
0.00	0.01	down
0.00	0.01	down
0.00	0.01	down
0.00	0.01	down
0.00	0.01	up
0.00	0.01	down
0.00	0.01	down
0.00	0.01	down
0.00	0.01	down
0.00	0.01	down
0.00	0.01	up
0.00	0.01	down
0.00	0.01	down
0.00	0.01	down
0.00	0.01	down
0.00	0.01	down
0.00	0.01	down
0.00	0.01	down
0.00	0.01	down
0.00	0.01	up
0.00	0.01	down
0.00	0.01	up
0.00	0.01	down
0.00	0.01	down
0.00	0.01	down
0.00	0.01	down
0.00	0.01	up
0.00	0.01	up
0.00	0.01	down
0.00	0.01	down
0.00	0.01	down
0.00	0.01	down
0.00	0.01	down
0.00	0.01	down
0.00	0.01	up
0.00	0.01	up
0.00	0.01	down
0.00	0.01	up
0.00	0.01	down
0.00	0.01	down
0.00	0.01	down
0.00	0.01	up
0.00	0.01	down

0.00	0.01	down
0.00	0.01	down
0.00	0.01	up
0.00	0.01	down
0.00	0.01	up
0.00	0.01	down
0.00	0.01	up
0.00	0.01	up
0.00	0.01	down
0.00	0.01	down
0.00	0.01	down
0.00	0.01	down
0.00	0.01	up
0.00	0.01	up
0.00	0.01	down
0.00	0.01	up
0.00	0.01	down
0.00	0.01	down
0.00	0.01	down
0.00	0.01	down
0.00	0.01	down
0.00	0.01	down
0.00	0.01	up
0.00	0.01	down
0.00	0.01	up
0.00	0.02	down
0.00	0.02	down
0.00	0.02	down
0.00	0.02	down
0.00	0.02	up
0.00	0.02	down
0.00	0.02	down
0.00	0.02	down
0.00	0.02	down
0.00	0.02	down
0.00	0.02	up
0.00	0.02	down
0.00	0.02	up
0.00	0.02	up
0.00	0.02	down
0.00	0.02	down
0.00	0.02	down
0.00	0.02	up
0.00	0.02	down
0.00	0.02	down
0.00	0.02	up
0.01	0.02	down
0.01	0.02	down
0.01	0.02	down
0.01	0.02	down
0.01	0.02	up

0.01	0.02	down
0.01	0.02	down
0.01	0.02	down
0.01	0.02	up
0.01	0.02	down
0.01	0.02	down
0.01	0.02	down
0.01	0.02	up
0.01	0.02	down
0.01	0.02	down
0.01	0.02	up
0.01	0.02	down
0.01	0.02	up
0.01	0.02	up
0.01	0.02	down
0.01	0.02	down
0.01	0.02	up
0.01	0.02	up
0.01	0.02	up
0.01	0.02	down
0.01	0.02	up
0.01	0.02	down
0.01	0.02	down
0.01	0.02	up
0.01	0.02	down
0.01	0.02	down
0.01	0.02	up
0.01	0.02	down
0.01	0.02	down
0.01	0.02	up
0.01	0.02	up
0.01	0.02	down
0.01	0.02	down
0.01	0.02	down
0.01	0.02	down
0.01	0.02	down
0.01	0.02	up
0.01	0.02	down
0.01	0.02	down
0.01	0.02	down
0.01	0.02	up
0.01	0.02	down
0.01	0.02	down
0.01	0.02	down
0.01	0.02	up
0.01	0.02	down
0.01	0.02	down
0.01	0.02	down
0.01	0.02	down
0.01	0.02	up
0.01	0.02	down

0.01	0.02	down
0.01	0.02	down
0.01	0.03	up
0.01	0.03	up
0.01	0.03	down
0.01	0.03	down
0.01	0.03	up
0.01	0.03	up
0.01	0.03	down
0.01	0.03	down
0.01	0.03	up
0.01	0.03	up
0.01	0.03	down
0.01	0.03	down
0.01	0.03	up
0.01	0.03	down
0.01	0.03	down
0.01	0.03	down
0.01	0.03	down
0.01	0.03	down
0.01	0.03	down
0.01	0.03	down
0.01	0.03	down
0.01	0.03	up
0.01	0.03	down
0.01	0.03	down
0.01	0.03	down
0.01	0.03	up
0.01	0.03	down
0.01	0.03	down
0.01	0.03	up
0.01	0.03	down
0.01	0.03	down
0.01	0.03	down
0.01	0.03	up
0.01	0.03	down
0.01	0.03	down
0.01	0.03	down
0.01	0.03	down
0.01	0.03	down
0.01	0.03	down
0.01	0.03	down
0.01	0.03	up
0.01	0.03	down
0.01	0.03	up
0.01	0.03	down
0.01	0.03	down
0.01	0.03	down
0.01	0.03	down
0.01	0.03	down
0.01	0.04	up

0.01	0.04	up
0.01	0.04	up
0.01	0.04	down
0.01	0.04	down
0.01	0.04	up
0.01	0.04	down
0.01	0.04	down
0.01	0.04	down
0.01	0.04	down
0.01	0.04	down
0.01	0.04	down
0.01	0.04	up
0.01	0.04	up
0.01	0.04	up
0.01	0.04	up
0.01	0.04	down
0.01	0.04	down
0.01	0.04	down
0.01	0.04	down
0.01	0.04	up
0.01	0.04	down
0.01	0.04	down
0.01	0.04	down
0.01	0.04	down
0.01	0.04	down
0.01	0.04	down
0.01	0.04	down
0.01	0.04	up
0.01	0.04	down
0.01	0.04	down
0.01	0.04	down
0.01	0.04	down
0.01	0.04	up
0.01	0.04	down
0.01	0.04	down
0.01	0.04	down
0.01	0.04	down
0.01	0.04	down
0.01	0.04	down
0.01	0.04	down
0.01	0.04	down
0.01	0.04	down
0.01	0.04	down
0.01	0.04	down
0.01	0.04	down
0.01	0.04	up
0.01	0.04	up
0.01	0.04	down
0.01	0.05	down
0.01	0.05	down
0.01	0.05	down

0.01	0.05	down
0.01	0.05	down
0.01	0.05	up
0.01	0.05	down
0.01	0.05	up
0.02	0.05	up
0.02	0.05	up
0.02	0.05	up
0.02	0.05	up
0.02	0.05	down
0.02	0.05	down
0.02	0.05	down
0.02	0.05	down
0.02	0.05	down
0.02	0.05	up
0.02	0.05	down
0.02	0.05	up
0.02	0.05	up
