# Supplementary material for: Transcriptome reveals differential expression of flavor and color in closely related strains of tomato (Solanum lycopersicum)
Source: PeerJ. 2025 Oct 7;13:e20113. doi: 10.7717/peerj.20113 (PMC12513376; doi:10.7717/peerj.20113)
Supplement: Supplemental Information 6 [file peerj-13-20113-s006.pdf]

Table S4: Differential expressions of genes in MF19-vs-MF20

gene_id	FPKM.M	FPKM.M	FPKM.M	FPKM.M	FPKM.M	FPKM.M	fc	log2(fc)
Solyc08g0	43.79	42.20	41.58	2.75	2.11	1.80	19.15	4.26
Solyc07g0	760.07	755.32	772.17	270.77	278.18	263.45	2.82	1.49
Solyc11g0	5.15	5.66	6.10	709.12	606.37	706.11	0.01	-6.90
Solyc10g0	5627.82	5650.01	5542.07	627.02	588.45	558.03	9.48	3.25
Solyc01g1	35.46	35.86	36.43	7.46	7.75	7.12	4.83	2.27
Solyc09g0	67.60	65.29	69.48	11.46	11.24	11.06	6.00	2.58
Solyc05g0	0.79	0.89	1.00	62.09	58.62	61.24	0.01	-6.09
Solyc10g0	472.36	508.30	489.17	116.97	119.66	111.06	4.23	2.08
Solyc05g0	56.13	55.76	57.84	2.46	2.70	3.37	19.89	4.31
Solyc04g0	6.27	7.66	6.71	120.28	155.47	138.82	0.05	-4.33
Solyc09g0	5.39	5.32	5.31	97.12	111.82	109.71	0.05	-4.31
Solyc04g0	0.85	0.86	0.87	9.34	10.59	10.00	0.09	-3.54
Solyc03g0	108.50	113.95	111.88	16.30	13.49	13.93	7.65	2.93
Solyc12g0	126.52	123.46	122.31	37.69	38.67	39.10	3.22	1.69
Solyc04g0	8.63	9.29	9.29	139.39	150.66	149.83	0.06	-4.01
Solyc01g0	52.37	56.85	58.13	532.14	657.60	615.60	0.09	-3.43
Solyc08g0	249.00	248.36	246.56	94.68	94.41	91.46	2.65	1.41
Solyc07g0	57.02	53.64	53.89	6.58	5.91	5.42	9.18	3.20
Solyc04g0	6.48	7.29	6.68	37.04	40.13	38.66	0.18	-2.50
Solyc11g0	27.84	27.54	25.41	1.54	1.11	1.12	21.45	4.42
Solyc04g0	30.84	29.53	30.55	5.01	5.25	4.63	6.11	2.61
Solyc06g0	13.08	12.24	12.59	1.72	1.72	1.48	7.71	2.95
Solyc03g0	48.75	49.76	51.45	12.72	12.46	11.87	4.05	2.02
Solyc09g0	1.49	1.50	1.63	8.33	9.06	8.47	0.18	-2.49
Solyc10g0	26.56	26.70	27.81	6.15	6.54	6.36	4.26	2.09
Solyc04g0	78.46	77.82	80.74	13.01	10.36	11.64	6.77	2.76
Solyc09g0	203.99	195.86	200.65	33.20	26.58	26.15	6.99	2.80
Solyc01g0	0.49	0.92	1.34	88.14	78.91	82.39	0.01	-6.50
Solyc08g0	2.65	3.03	2.85	20.60	20.72	20.87	0.14	-2.87
Solyc07g0	41.23	40.69	39.15	6.44	6.34	5.21	6.73	2.75
Solyc03g0	156.85	158.17	157.58	62.10	64.49	60.50	2.53	1.34
Solyc01g1	25.93	24.06	24.46	5.42	5.02	5.25	4.75	2.25
Solyc07g0	271.82	279.31	288.62	793.96	794.82	805.04	0.35	-1.51
Solyc12g0	200.39	198.87	199.36	589.51	636.33	657.41	0.32	-1.65
Solyc09g0	0.42	0.33	0.36	7.18	8.26	7.97	0.05	-4.40
Solyc01g1	99.93	97.05	96.87	19.36	18.98	15.52	5.46	2.45
Solyc02g0	2.20	1.83	2.06	28.07	26.92	25.60	0.08	-3.73
Solyc07g0	97.53	108.54	102.80	25.04	26.06	23.55	4.14	2.05
Solyc01g1	11.21	10.29	10.49	1.70	2.00	1.85	5.77	2.53
Solyc06g0	120.32	115.99	119.21	47.31	47.97	45.93	2.52	1.33
Solyc04g0	282.46	277.71	292.96	107.66	107.14	100.94	2.70	1.43
Solyc01g0	587.55	605.88	581.94	271.13	265.57	276.43	2.18	1.13
Solyc02g0	555.91	539.35	541.94	138.10	170.15	155.10	3.53	1.82
Solyc01g1	35.79	35.73	34.76	3.72	3.20	2.42	11.39	3.51
Solyc10g0	600.42	604.51	620.27	283.61	301.55	289.13	2.09	1.06
Solyc03g0	23.42	23.55	22.32	88.58	101.09	95.41	0.24	-2.04
Solyc09g0	5.07	5.19	5.31	19.40	19.24	19.73	0.27	-1.91
Solyc01g0	119.59	121.05	116.28	42.62	45.78	47.97	2.62	1.39
Solyc04g0	14.84	15.23	15.72	88.45	117.48	105.81	0.15	-2.77

Solyc06g0	19.02	20.56	19.81	2.25	1.78	2.25	9.47	3.24
Solyc09g0	0.07	0.05	0.14	9.68	12.07	11.37	0.01	-7.01
Solyc07g0	56.18	56.87	57.08	24.41	25.44	24.43	2.29	1.20
Solyc08g0	489.99	465.12	469.56	188.25	194.98	179.34	2.53	1.34
Solyc11g0	1.80	1.99	1.74	17.10	16.89	18.84	0.10	-3.26
Solyc05g0	1.73	2.42	2.95	39.83	41.52	37.65	0.06	-4.07
Solyc07g0	89.88	86.23	85.54	31.99	29.47	31.98	2.80	1.49
Solyc02g0	49.39	53.53	53.38	15.27	14.65	13.92	3.57	1.83
Solyc09g0	18.83	18.97	18.22	2.75	2.33	2.25	7.65	2.93
Solyc01g0	93.51	90.00	92.11	38.18	37.59	36.45	2.46	1.30
Solyc11g0	111.55	111.82	108.77	35.99	35.14	31.16	3.25	1.70
Solyc02g0	103.03	102.09	99.48	35.46	31.93	31.50	3.08	1.62
Solyc05g0	15.54	15.38	13.91	3.41	3.47	3.18	4.45	2.16
Solyc01g1	12.48	12.91	12.77	0.71	1.10	0.91	14.06	3.81
Solyc11g0	25.20	27.11	26.97	89.93	102.39	90.62	0.28	-1.84
Solyc07g0	171.80	155.12	157.52	52.83	50.70	49.57	3.16	1.66
Solyc10g0	8.86	8.02	8.33	0.13	0.14	0.12	65.27	6.03
Solyc11g0	0.51	0.87	0.95	15.06	14.01	16.63	0.05	-4.30
Solyc03g0	24.28	26.46	24.01	92.25	116.25	102.83	0.24	-2.06
Solyc09g0	0.99	1.28	1.01	19.62	15.49	19.34	0.06	-4.05
Solyc09g0	16.09	14.33	15.61	91.07	91.14	81.06	0.17	-2.52
Solyc05g0	355.34	365.20	356.42	912.54	1054.40	1021.28	0.36	-1.47
Solyc06g0	21.08	19.89	20.13	6.51	6.18	6.74	3.14	1.65
Solyc12g0	138.47	136.40	139.18	52.44	48.55	45.59	2.82	1.50
Solyc06g0	9.68	9.39	8.87	1.25	1.01	1.31	7.83	2.97
Solyc07g0	74.91	71.15	75.49	26.68	26.31	26.91	2.77	1.47
Solyc05g0	42.04	44.15	43.32	15.87	15.81	14.57	2.80	1.49
Solyc09g0	23.78	23.89	23.97	7.76	8.10	8.17	2.98	1.58
Solyc04g0	25.24	24.66	23.15	6.20	5.54	6.25	4.06	2.02
ENSRNA(	2.03	1.32	2.53	134.24	148.31	136.91	0.01	-6.16
Solyc09g0	15.25	13.82	14.85	44.27	47.54	45.35	0.32	-1.64
Solyc06g0	18.49	18.50	17.85	2.93	1.97	2.40	7.51	2.91
Solyc04g0	15.26	15.72	14.83	4.46	4.28	4.23	3.53	1.82
Solyc11g0	59.13	59.45	58.86	26.89	26.69	24.57	2.27	1.18
Solyc09g0	9.22	8.85	8.47	0.55	0.65	0.54	15.27	3.93
Solyc09g0	20.97	20.91	21.16	57.05	62.33	63.57	0.34	-1.54
Solyc03g1	78.84	82.31	78.93	33.16	32.83	30.44	2.49	1.32
Solyc04g0	39.98	41.27	38.95	17.53	18.73	18.75	2.19	1.13
Solyc11g0	10.61	11.50	11.20	1.37	1.09	1.40	8.62	3.11
Solyc09g0	11.89	11.21	11.62	44.54	54.26	49.25	0.23	-2.09
Solyc01g1	221.61	220.48	215.48	90.52	76.84	79.01	2.67	1.42
Solyc08g0	16.74	17.66	15.93	3.90	3.80	3.45	4.51	2.17
Solyc09g0	4.07	3.83	4.15	15.42	17.16	16.28	0.25	-2.02
Solyc02g0	14.24	12.17	10.70	1.18	0.93	1.03	11.82	3.56
Solyc06g0	152.67	154.13	153.27	73.16	67.88	66.41	2.22	1.15
ENSRNA(	0.06	0.09	0.06	14.32	16.02	15.79	0.00	-7.78
Solyc11g0	27.76	27.73	25.71	73.61	78.19	72.63	0.36	-1.47
Solyc03g1	7.17	7.99	7.85	22.38	23.52	23.78	0.33	-1.60
Solyc08g0	19.81	18.83	18.22	5.23	4.88	5.24	3.70	1.89
Solyc05g0	0.19	0.39	0.38	12.21	14.36	12.57	0.02	-5.33
Solyc12g0	10.77	9.84	9.60	1.85	2.28	1.98	4.94	2.30

Solyc05g0	27.79	27.66	28.90	12.99	12.71	12.17	2.23	1.16
Solyc12g0	11.66	11.23	9.47	0.50	0.42	0.40	24.67	4.62
Solyc08g0	11.28	11.37	10.78	4.00	3.74	3.72	2.92	1.55
Solyc03g1	5.90	5.60	5.95	1.67	1.72	1.70	3.43	1.78
Solyc02g0	57.29	57.05	61.48	15.52	11.90	11.96	4.47	2.16
Solyc12g0	8.66	7.76	7.91	31.52	34.10	30.78	0.25	-1.99
Solyc03g0	18.78	18.95	17.97	7.85	8.17	7.34	2.38	1.25
Solyc01g0	28.05	27.04	25.49	8.26	7.49	7.17	3.52	1.81
Solyc02g0	30.67	30.77	30.29	112.91	103.44	98.83	0.29	-1.78
Solyc01g0	15.00	12.98	11.50	1.04	0.97	0.67	14.73	3.88
Solyc01g0	17.36	18.51	16.24	48.97	59.37	57.81	0.31	-1.67
Solyc04g0	52.06	51.63	49.49	20.51	19.56	18.36	2.62	1.39
Solyc04g0	21.04	20.47	22.05	55.82	62.62	60.93	0.35	-1.50
Solyc08g0	18.71	21.80	20.48	3.57	2.61	3.49	6.31	2.66
Solyc03g1	22.27	23.59	23.66	57.31	56.09	54.42	0.41	-1.27
Solyc02g0	1.41	1.61	1.56	6.88	7.42	7.11	0.21	-2.23
Solyc12g0	25.80	26.79	25.67	55.15	58.80	60.32	0.45	-1.16
Solyc12g0	63.12	55.60	63.70	19.61	16.52	17.30	3.41	1.77
Solyc01g0	67.09	66.67	70.12	32.51	35.82	32.95	2.01	1.01
Solyc03g0	10.77	9.87	9.51	2.14	1.68	1.86	5.30	2.41
Solyc09g0	234.00	231.35	232.11	114.55	101.60	97.99	2.22	1.15
Solyc06g0	23.91	19.34	21.78	2.55	3.61	2.95	7.14	2.84
Solyc03g0	15.00	16.61	16.19	41.74	41.37	41.39	0.38	-1.38
Solyc09g0	28.58	29.98	29.60	112.11	92.76	98.91	0.29	-1.78
Solyc11g0	22.56	22.56	21.50	6.44	7.72	6.63	3.20	1.68
Solyc02g0	6.48	6.34	6.61	1.05	0.70	0.89	7.35	2.88
Solyc02g0	13.36	13.67	14.36	41.03	37.59	38.20	0.35	-1.50
Solyc04g0	1.06	0.95	1.28	16.33	23.25	20.55	0.05	-4.19
Solyc10g0	12.79	13.22	12.68	2.10	1.83	1.64	6.94	2.80
Solyc08g0	181.18	176.20	180.04	71.93	60.32	58.48	2.82	1.49
Solyc09g0	12.51	12.57	13.07	5.35	5.63	5.32	2.34	1.23
Solyc01g0	143.38	137.83	131.36	54.20	47.62	51.85	2.68	1.42
Solyc07g0	12.30	13.62	13.13	2.00	1.71	2.10	6.72	2.75
Solyc01g1	9.07	8.68	7.86	1.92	1.67	1.55	4.98	2.32
Solyc03g1	740.92	811.65	811.28	227.43	297.39	280.45	2.94	1.55
Solyc12g1	10.20	10.36	10.34	4.10	3.87	4.23	2.53	1.34
Solyc05g0	24.67	23.76	23.43	7.46	8.75	7.56	3.02	1.60
Solyc02g0	369.35	341.41	343.71	39.02	30.24	25.83	11.09	3.47
Solyc03g1	578.07	565.64	529.12	234.75	285.77	254.19	2.16	1.11
Solyc11g0	15.10	14.37	12.76	1.59	1.76	1.09	9.52	3.25
Solyc07g0	20.01	21.41	23.26	6.63	5.50	6.26	3.52	1.81
Solyc04g0	39.36	37.66	38.90	16.51	17.56	18.63	2.20	1.14
Solyc04g0	44.09	48.28	42.83	16.28	16.88	16.31	2.73	1.45
Solyc03g1	18.92	18.30	18.06	6.60	7.99	8.10	2.44	1.28
Solyc04g0	12.18	13.33	13.91	3.72	3.38	3.33	3.78	1.92
Solyc02g0	2.02	2.01	2.06	0.20	0.14	0.18	11.83	3.56
Solyc02g0	54.68	50.74	52.66	21.64	18.80	18.23	2.69	1.43
Solyc01g1	11.26	11.79	12.40	37.27	41.31	39.05	0.30	-1.73
Solyc10g0	60.48	60.09	58.15	26.63	30.08	28.88	2.09	1.06
Solyc06g0	28.70	28.78	26.66	12.26	13.26	13.62	2.15	1.10
Solyc12g0	176.87	174.49	175.77	23.76	18.14	16.23	9.07	3.18

Solyc03g0	5.25	5.07	5.48	0.28	0.26	0.15	22.76	4.51
Solyc05g0	2.15	2.63	2.38	21.40	19.49	16.49	0.12	-3.00
Solyc09g0	21.91	22.16	23.20	7.95	6.98	8.44	2.88	1.53
Solyc11g0	10.12	9.95	10.59	2.53	2.73	2.18	4.12	2.04
Solyc03g1	31.95	34.99	33.11	16.08	15.68	15.83	2.10	1.07
Solyc01g0	0.27	1.01	0.73	25.97	21.75	22.25	0.03	-5.12
Solyc02g0	44.35	47.53	43.93	22.16	20.39	22.40	2.09	1.06
Solyc01g0	36.66	37.67	39.24	16.77	17.80	19.43	2.10	1.07
Solyc06g0	5.98	6.18	5.83	17.91	22.88	21.15	0.29	-1.78
Solyc12g0	12.21	11.63	10.59	3.34	3.81	3.36	3.28	1.71
Solyc09g0	10.37	10.92	10.65	2.23	2.68	3.01	4.03	2.01
Solyc07g0	3.62	3.62	3.55	0.61	0.38	0.50	7.24	2.86
Solyc01g1	67.96	68.95	66.53	141.50	160.45	164.31	0.44	-1.20
Solyc11g0	20.13	21.11	18.24	60.13	76.81	70.18	0.29	-1.80
Solyc06g0	12.08	10.85	12.10	1.51	2.19	1.38	6.89	2.78
Solyc04g0	15.79	16.57	16.78	5.32	4.33	4.40	3.50	1.81
Solyc03g1	24.28	22.76	25.33	10.73	10.73	11.48	2.20	1.14
Solyc03g0	28.13	25.20	26.89	6.02	4.12	4.16	5.61	2.49
Solyc05g0	3.20	3.39	3.43	9.93	11.59	11.49	0.30	-1.72
Solyc01g1	6.24	6.77	6.23	19.23	23.96	23.74	0.29	-1.80
Solyc01g0	129.81	138.02	127.90	42.93	32.65	30.82	3.72	1.90
Solyc03g1	24.59	22.34	24.04	70.06	64.21	71.45	0.35	-1.54
Solyc08g0	11.74	11.99	12.23	4.90	4.66	4.27	2.60	1.38
Solyc01g1	4.91	5.21	5.01	0.89	0.74	0.59	6.83	2.77
Solyc02g0	3.51	3.96	3.43	12.20	11.80	11.78	0.30	-1.71
Solyc12g0	29.79	30.66	31.68	15.08	15.18	15.67	2.01	1.00
Solyc01g0	25.27	23.66	23.40	10.98	10.50	10.59	2.26	1.17
Solyc06g0	24.42	23.34	22.28	1.69	0.50	1.14	21.01	4.39
Solyc10g0	71.23	68.04	66.84	31.76	29.79	27.38	2.32	1.21
Solyc11g0	9.21	8.60	9.50	26.20	33.64	29.82	0.30	-1.72
Solyc04g0	2.17	2.40	2.89	12.83	14.66	14.10	0.18	-2.48
Solyc05g0	6.63	7.05	7.28	1.67	1.16	1.55	4.79	2.26
Solyc03g1	39.08	40.94	40.26	6.51	3.53	4.03	8.55	3.10
Solyc07g0	45.50	49.34	49.51	14.77	10.13	11.04	4.02	2.01
Solyc01g1	29.26	30.77	29.61	62.56	69.33	71.39	0.44	-1.18
Solyc11g0	8.63	8.60	8.62	3.83	3.62	3.88	2.28	1.19
Solyc10g0	28.02	28.17	28.83	11.58	13.08	11.48	2.35	1.23
Solyc09g0	9.96	9.22	9.71	27.25	27.42	24.42	0.37	-1.45
Solyc04g0	61.83	61.05	58.28	138.26	161.11	140.40	0.41	-1.28
Solyc04g0	435.77	439.53	437.16	211.36	182.57	164.98	2.35	1.23
Solyc10g0	8.67	8.64	9.07	2.76	3.11	2.57	3.13	1.64
Solyc12g0	14.01	12.15	12.36	38.41	45.46	45.84	0.30	-1.75
Solyc02g0	29.46	32.73	31.66	14.56	14.75	13.99	2.17	1.12
Solyc06g0	0	0.15	0.13	18.56	24.63	24.49	0.00	-7.89
Solyc08g0	262.64	265.14	251.87	99.47	130.20	106.46	2.32	1.21
Solyc01g0	17.66	17.77	17.51	7.78	9.35	8.38	2.08	1.05
Solyc02g0	26.85	25.90	26.80	5.89	7.08	4.63	4.52	2.18
Solyc02g0	8.29	8.71	8.26	2.40	2.30	2.27	3.63	1.86
Solyc06g0	9.84	10.87	9.87	29.02	33.60	33.95	0.32	-1.66
Solyc11g0	6.69	5.47	5.85	0.65	0.65	0.41	10.55	3.40
Solyc04g0	24.38	23.50	23.06	6.96	4.56	4.72	4.37	2.13

Solyc03g0	0.37	0.38	0.30	2.26	2.68	2.56	0.14	-2.84
Solyc05g0	7.54	7.59	7.26	1.12	1.30	1.39	5.88	2.56
Solyc06g0	22.50	22.28	20.88	10.27	11.37	10.45	2.05	1.03
Solyc07g0	16.24	16.75	16.18	7.83	7.35	7.12	2.20	1.14
Solyc09g0	0.66	0.57	0.56	3.02	3.34	3.47	0.18	-2.45
Solyc07g0	23.96	23.40	22.53	8.85	7.25	7.16	3.00	1.59
Solyc08g0	18.86	19.76	21.01	8.05	6.96	7.61	2.64	1.40
Solyc05g0	13.51	14.99	14.47	5.63	4.99	5.60	2.65	1.41
Solyc02g0	18.55	19.05	17.77	46.37	60.85	57.27	0.34	-1.57
Solyc07g0	7.19	7.04	6.97	22.55	31.29	24.87	0.27	-1.89
Solyc09g0	22.71	23.96	22.96	45.82	52.55	49.67	0.47	-1.09
Solyc04g0	8.64	8.50	9.94	1.43	0.82	0.91	8.55	3.10
Solyc01g0	5.56	5.71	6.11	1.92	1.98	1.74	3.08	1.62
Solyc05g0	19.97	19.59	20.18	40.58	46.51	42.83	0.46	-1.12
Solyc01g0	0.19	0.34	0.65	29.22	31.01	34.11	0.01	-6.32
Solyc09g0	46.91	44.22	45.08	21.01	18.48	17.99	2.37	1.24
Solyc01g1	24.49	26.14	24.83	54.89	64.18	66.42	0.41	-1.30
Solyc10g0	6.19	5.58	5.44	20.46	20.42	20.75	0.28	-1.84
Solyc09g0	18.76	19.53	20.56	5.73	7.08	6.74	3.01	1.59
Solyc11g0	7.23	7.13	7.08	16.52	16.80	18.65	0.41	-1.28
Solyc06g0	11.14	10.85	10.82	24.31	25.56	26.80	0.43	-1.22
Solyc01g1	10.65	8.88	9.77	2.80	3.23	2.99	3.25	1.70
Solyc10g0	10.21	9.83	10.78	4.52	3.89	4.03	2.48	1.31
Solyc01g0	7.39	7.48	8.13	21.84	25.36	23.06	0.33	-1.61
Solyc04g0	21.62	19.19	18.17	5.04	6.43	5.99	3.38	1.76
Solyc03g0	45.89	45.63	44.23	22.95	20.73	20.82	2.10	1.07
Solyc04g0	12.44	13.61	13.34	32.71	31.70	33.52	0.40	-1.31
Solyc08g0	120.38	109.39	123.94	255.44	264.64	247.46	0.46	-1.12
Solyc01g0	2.52	2.91	2.77	12.11	14.05	10.97	0.22	-2.18
Solyc03g0	7.75	8.75	8.11	3.81	3.57	3.47	2.27	1.18
Solyc02g0	14.71	14.20	14.63	6.61	6.53	6.02	2.27	1.18
Solyc01g0	14.24	13.78	13.31	5.00	5.79	5.33	2.56	1.36
Solyc03g1	2489.85	2320.45	2468.66	1190.80	947.94	916.88	2.38	1.25
Solyc02g0	20.43	19.75	18.97	7.52	8.71	8.34	2.41	1.27
Solyc02g0	235.45	250.31	242.65	445.55	511.44	505.34	0.50	-1.01
Solyc08g0	8.58	9.06	7.89	2.15	2.82	2.37	3.48	1.80
Solyc11g0	2.37	3.15	2.24	0.11	0.15	0.13	20.23	4.34
Solyc02g0	20.37	19.53	21.69	7.48	8.18	7.67	2.64	1.40
Solyc09g0	2.12	2.35	2.33	6.73	6.75	6.37	0.34	-1.54
Solyc07g0	0.18	0.27	0.19	15.41	9.30	10.54	0.02	-5.77
Solyc03g0	16.84	14.64	14.33	4.38	3.48	3.53	4.02	2.01
Solyc09g0	2.93	3.13	2.87	8.93	11.07	10.64	0.29	-1.78
Solyc07g0	11.46	12.29	12.75	5.92	5.57	6.39	2.04	1.03
Solyc05g0	14.33	15.17	14.37	31.16	29.67	29.73	0.48	-1.05
Solyc04g0	2.67	2.63	2.52	0.69	0.69	0.58	3.99	2.00
Solyc04g0	23.55	25.50	23.00	9.10	10.82	9.76	2.43	1.28
Solyc02g0	10.71	10.15	9.45	23.33	22.78	25.89	0.42	-1.25
Solyc03g0	1.55	1.84	1.77	5.83	6.11	6.74	0.28	-1.86
Solyc04g0	12.35	10.77	12.15	3.94	3.94	4.33	2.89	1.53
Solyc12g0	6.47	7.03	7.34	1.54	1.80	1.30	4.50	2.17
Solyc01g1	11.29	11.81	10.85	4.12	4.56	5.08	2.47	1.30

Solyc01g1	75.09	69.90	73.31	34.34	30.74	27.44	2.36	1.24
Solyc06g0	0.71	0.80	0.78	4.49	5.12	5.05	0.16	-2.68
Solyc01g1	7.97	9.10	8.33	1.77	2.64	2.34	3.76	1.91
Solyc10g0	0.89	1.19	1.05	10.55	14.73	10.80	0.09	-3.53
Solyc01g0	30.28	31.83	32.55	62.65	64.19	63.07	0.50	-1.00
Solyc09g0	2.97	3.17	3.32	0.25	0.10	0.09	21.60	4.43
Solyc02g0	0.53	0.58	0.47	3.95	5.08	4.21	0.12	-3.07
Solyc03g0	13.86	12.90	14.63	5.91	5.66	6.21	2.33	1.22
Solyc02g0	11.49	12.17	12.37	23.82	26.41	26.91	0.47	-1.10
Solyc07g0	15.05	15.15	16.71	40.46	53.18	51.96	0.32	-1.63
Solyc03g0	32.10	34.78	36.62	77.14	89.47	82.48	0.42	-1.27
Solyc03g1	19.39	19.93	22.09	9.01	8.79	9.85	2.22	1.15
Solyc08g0	0.84	0.57	0.72	5.29	5.18	5.86	0.13	-2.93
Solyc07g0	18.36	17.89	17.15	8.15	7.67	8.79	2.17	1.12
Solyc08g0	8.24	6.49	6.68	0.58	0.38	0.33	16.60	4.05
Solyc12g0	10.32	10.64	10.51	3.79	4.18	3.92	2.65	1.40
Solyc11g0	10.62	9.49	10.54	2.82	2.59	2.47	3.90	1.96
Solyc05g0	24.71	22.14	22.94	10.39	10.23	9.46	2.32	1.21
Solyc01g0	3.56	4.40	3.77	0.41	0.16	0.19	15.46	3.95
Solyc06g0	15.33	16.93	16.18	6.86	7.90	7.80	2.15	1.10
Solyc11g0	0.32	0.29	0.31	1.53	1.40	1.34	0.22	-2.21
Solyc11g0	3.70	3.96	4.33	0.92	0.78	0.90	4.62	2.21
Solyc04g0	9.25	9.96	10.49	3.94	3.54	3.00	2.83	1.50
Solyc05g0	13.14	15.77	12.95	2.61	3.20	3.72	4.39	2.14
Solyc06g0	16.04	14.72	15.12	1.72	0.78	1.14	12.59	3.65
Solyc06g0	9.66	10.05	10.21	22.67	26.35	25.60	0.40	-1.32
Solyc05g0	15.04	15.85	12.94	3.46	2.53	3.20	4.77	2.25
Solyc07g0	6.32	5.79	6.75	1.78	1.54	1.48	3.93	1.98
Solyc12g0	8.69	8.57	7.97	2.51	2.74	2.39	3.30	1.72
Solyc12g0	59.48	54.60	52.25	22.11	21.19	17.15	2.75	1.46
Solyc12g0	35.31	33.27	33.79	9.51	11.77	13.24	2.97	1.57
Solyc12g0	21.68	21.06	21.11	9.96	9.85	9.17	2.20	1.14
Solyc07g0	2.30	2.31	2.28	8.58	8.88	9.14	0.26	-1.95
Solyc12g0	37.58	37.07	37.11	14.45	14.70	15.90	2.48	1.31
Solyc09g0	3.62	3.18	3.14	0.54	0.36	0.47	7.25	2.86
Solyc03g0	26.25	24.35	23.88	10.04	9.97	10.61	2.43	1.28
Solyc12g0	12.85	11.89	12.75	25.37	27.32	25.89	0.48	-1.07
Solyc02g0	6.26	5.88	5.74	0.71	0.87	0.43	8.87	3.15
Solyc10g0	14.92	13.62	13.39	6.37	6.79	6.36	2.15	1.10
Solyc01g0	25.84	25.06	25.10	13.52	11.37	11.46	2.09	1.06
Solyc12g0	2.07	2.02	2.29	7.29	6.81	7.24	0.30	-1.74
Solyc08g0	7.98	7.45	8.63	2.58	3.20	2.66	2.85	1.51
Solyc12g0	8.97	9.52	9.86	1.50	0.59	0.77	9.91	3.31
Solyc09g0	11.74	11.09	11.96	25.27	31.82	30.75	0.40	-1.34
Solyc01g0	24.21	25.01	23.98	53.38	66.02	56.85	0.42	-1.27
Solyc09g0	8.06	7.59	8.32	22.21	28.32	23.80	0.32	-1.63
Solyc09g0	7.74	7.23	7.98	0.93	1.45	0.94	6.92	2.79
Solyc01g0	21.84	21.72	21.83	45.76	47.72	49.47	0.46	-1.13
Solyc01g0	7.05	6.35	6.65	2.62	2.36	1.99	2.88	1.53
Solyc09g0	4.32	4.01	4.61	1.07	1.18	0.86	4.17	2.06
Solyc09g0	4.02	4.35	3.49	0.52	0.79	0.61	6.17	2.63

Solyc06g0	17.20	17.62	14.94	5.07	5.36	6.22	2.99	1.58
Solyc09g0	4.86	4.90	4.54	1.96	1.92	2.07	2.40	1.26
Solyc09g0	4.98	4.67	5.67	14.41	14.40	14.62	0.35	-1.50
Solyc06g0	32.09	34.50	33.75	63.83	75.91	67.45	0.48	-1.05
Solyc09g0	2.70	2.07	2.85	9.51	10.36	10.86	0.25	-2.01
Solyc08g0	1.76	1.88	1.70	0.43	0.38	0.36	4.60	2.20
Solyc02g0	17.99	17.98	18.41	34.01	37.54	40.05	0.49	-1.04
Solyc06g0	10.73	10.71	10.84	3.86	4.64	4.95	2.40	1.26
Solyc05g0	24.49	25.03	24.72	11.06	9.74	10.43	2.38	1.25
Solyc02g0	0.36	0.39	0.43	2.24	2.76	2.64	0.16	-2.68
Solyc01g0	23.79	22.64	22.09	71.66	59.47	61.11	0.36	-1.49
Solyc09g0	1.55	1.47	1.32	6.22	6.08	5.57	0.24	-2.04
Solyc01g0	3.03	2.82	2.90	7.89	8.99	8.50	0.34	-1.54
Solyc10g0	33.03	33.93	33.24	14.75	18.71	16.26	2.02	1.01
Solyc12g0	6.54	6.54	6.51	2.67	2.87	2.81	2.35	1.23
Solyc04g0	58.20	58.88	58.09	25.45	20.61	18.99	2.69	1.43
Solyc08g0	49.61	52.82	49.78	114.91	104.55	101.45	0.47	-1.08
Solyc09g0	0.82	0.87	0.96	0.20	0.19	0.20	4.52	2.18
Solyc08g0	2.11	2.12	2.46	0.49	0.47	0.47	4.70	2.23
Solyc06g0	4.84	4.85	4.55	10.28	12.91	11.73	0.41	-1.29
Solyc12g0	24.02	24.86	23.96	9.42	10.20	7.28	2.71	1.44
Solyc01g1	1.78	1.60	1.75	0.03	0	0.04	72.82	6.19
Solyc09g0	21.99	20.81	18.63	73.55	67.30	56.80	0.31	-1.69
Solyc03g1	6.71	9.13	8.19	1.02	0.49	0.45	12.24	3.61
Solyc03g0	12.09	13.30	12.67	3.06	2.80	3.26	4.17	2.06
Solyc01g1	11.48	10.43	10.79	3.67	3.82	3.77	2.90	1.54
Solyc10g0	4.83	4.76	4.40	10.44	13.14	11.80	0.40	-1.34
Solyc10g0	24.20	25.77	28.00	8.78	9.94	7.90	2.93	1.55
Solyc01g0	3.59	3.50	2.99	10.02	9.79	8.88	0.35	-1.51
Solyc07g0	11.08	11.11	10.26	3.63	4.90	3.80	2.63	1.40
Solyc04g0	9.08	8.70	9.41	3.73	4.16	3.99	2.29	1.19
Solyc11g0	11.41	12.15	12.44	23.87	27.31	25.84	0.47	-1.10
Solyc02g0	5.55	5.79	4.98	12.87	15.74	15.10	0.37	-1.42
Solyc06g0	5.33	4.81	4.98	2.13	1.92	2.07	2.47	1.31
Solyc03g0	2.28	2.37	1.99	0.66	0.75	0.68	3.18	1.67
Solyc06g0	19.25	21.14	21.84	9.08	9.79	10.06	2.15	1.10
Solyc01g0	1.68	1.75	1.81	0.27	0.16	0.21	8.17	3.03
Solyc11g0	12.84	14.34	12.69	6.70	6.71	6.00	2.05	1.04
Solyc11g0	5.64	5.09	5.13	1.75	1.81	1.46	3.16	1.66
Solyc06g0	1.48	1.53	1.50	0.16	0.04	0.07	16.51	4.04
Solyc06g0	2.70	2.48	2.26	7.60	9.70	8.73	0.29	-1.81
Solyc05g0	3.03	2.30	2.95	26.42	26.95	24.58	0.11	-3.23
Solyc08g0	8.69	9.27	9.03	2.80	1.78	2.18	3.99	2.00
Solyc03g1	0.56	0.48	0.58	2.57	3.11	3.70	0.17	-2.53
Solyc06g0	4.00	4.21	5.32	1.23	1.25	1.26	3.62	1.85
Solyc07g0	48.98	52.75	51.53	94.11	114.79	102.40	0.49	-1.02
Solyc09g0	14.12	14.87	12.28	2.52	2.14	1.09	7.18	2.84
Solyc09g0	5.23	5.50	6.02	1.82	1.45	1.37	3.61	1.85
Solyc09g0	12.80	13.14	12.62	3.05	3.08	3.11	4.17	2.06
Solyc09g0	32.19	31.50	27.47	63.75	80.77	75.30	0.41	-1.27
Solyc07g0	7.80	7.67	7.47	3.70	3.92	3.80	2.01	1.01

Solyc09g0	0.10	0.17	0.19	1.47	1.72	1.73	0.09	-3.42
Solyc06g0	1.35	1.37	1.75	7.14	10.34	6.78	0.18	-2.44
Solyc10g0	4.87	4.56	4.94	1.84	1.55	1.54	2.91	1.54
Solyc03g1	37.91	35.55	37.24	83.52	94.37	79.65	0.43	-1.22
Solyc05g0	8.37	7.84	8.36	3.27	3.30	3.66	2.40	1.27
Solyc09g0	19.41	22.22	20.08	10.24	9.49	9.26	2.13	1.09
Solyc09g0	8.94	10.25	9.32	20.22	22.95	21.50	0.44	-1.18
Solyc10g0	45.63	43.34	45.69	20.84	20.95	20.22	2.17	1.12
Solyc02g0	7.94	9.02	8.88	3.74	4.67	3.74	2.12	1.09
Solyc12g0	10.37	10.36	10.34	4.83	5.03	5.34	2.04	1.03
Solyc04g0	47.72	52.84	46.63	106.18	153.23	129.67	0.38	-1.40
Solyc11g0	52.77	50.17	50.26	22.04	15.67	14.72	2.92	1.55
Solyc04g0	15.10	15.48	15.17	45.40	44.47	35.01	0.37	-1.45
Solyc06g0	59.29	69.68	64.73	129.87	160.48	157.89	0.43	-1.21
Solyc05g0	7.88	5.93	5.89	1.23	1.66	1.23	4.78	2.26
Solyc08g0	6.59	5.58	6.72	2.53	2.53	2.88	2.38	1.25
Solyc09g0	10.32	11.07	9.50	2.25	1.13	1.90	5.85	2.55
Solyc01g0	6.24	6.57	6.02	2.10	2.04	1.65	3.25	1.70
Solyc04g0	5.33	6.07	5.12	2.06	1.65	2.02	2.89	1.53
Solyc01g1	0.85	0.93	0.82	3.22	3.72	3.83	0.24	-2.06
Solyc01g1	5.70	5.84	5.77	16.46	14.05	16.26	0.37	-1.43
Solyc03g1	1.98	2.69	2.41	7.84	7.54	8.43	0.30	-1.75
Solyc10g0	6.20	5.18	6.13	14.19	16.27	14.85	0.39	-1.37
Solyc03g0	3.22	2.77	2.92	0.75	0.91	0.73	3.72	1.89
Solyc07g0	15.90	15.58	16.28	5.94	6.70	5.23	2.67	1.42
Solyc05g0	10.47	11.47	9.85	1.82	2.86	2.66	4.33	2.11
Solyc07g0	4.00	3.99	3.88	0.53	0.37	0.69	7.42	2.89
Solyc09g0	1.49	1.51	1.60	4.29	4.82	4.62	0.34	-1.58
Solyc03g0	11.88	12.58	11.98	23.65	26.82	24.87	0.48	-1.05
Solyc08g0	3.70	3.94	4.13	11.72	14.79	13.83	0.29	-1.78
Solyc05g0	1.62	1.51	1.29	16.77	14.69	16.49	0.09	-3.44
Solyc04g0	29.35	30.31	33.65	14.86	12.80	12.53	2.32	1.22
Solyc03g0	12.72	11.91	11.04	5.26	6.34	4.94	2.16	1.11
Solyc04g0	0.92	0.40	0.83	5.03	5.57	5.86	0.13	-2.94
Solyc11g0	9.88	10.10	9.28	19.61	19.40	20.05	0.50	-1.01
Solyc11g0	26.62	30.79	29.12	11.14	13.37	13.30	2.29	1.19
Solyc06g0	10.60	10.75	10.83	4.69	5.72	4.99	2.09	1.06
Solyc02g0	6.44	6.39	7.20	13.17	15.21	14.74	0.46	-1.11
Solyc06g0	12.18	13.37	9.71	2.25	2.05	2.30	5.35	2.42
Solyc01g0	12.42	11.45	11.66	5.64	5.59	5.25	2.16	1.11
Solyc03g0	1.23	1.39	1.44	0.12	0.19	0.22	7.56	2.92
Solyc07g0	2.08	2.03	2.34	0.31	0.25	0.41	6.62	2.73
Solyc09g0	0.62	0.80	0.82	3.41	3.49	3.15	0.22	-2.17
Solyc03g1	3.54	3.82	3.66	1.57	1.46	1.53	2.42	1.27
Solyc06g0	1.06	0.88	0.96	3.27	4.01	4.09	0.26	-1.97
Solyc02g0	8.72	9.09	8.66	3.48	3.04	3.73	2.58	1.37
Solyc01g0	3.92	3.55	2.90	11.05	11.35	10.16	0.32	-1.65
Solyc10g0	2.79	2.92	2.86	6.58	7.46	7.70	0.39	-1.34
Solyc06g0	10.77	10.07	10.79	4.02	2.99	2.84	3.21	1.68
Solyc05g0	5.27	5.79	5.56	2.30	2.28	2.32	2.41	1.27
Solyc02g0	697.25	642.35	647.93	95.02	64.11	48.48	9.57	3.26

Solyc07g0	3.88	2.81	3.62	0.55	0.26	0.42	8.41	3.07
Solyc08g0	2.56	2.33	2.88	0.35	0.46	0.43	6.25	2.64
Solyc03g0	1.37	1.40	1.39	4.72	4.09	4.42	0.31	-1.67
Solyc12g0	11.90	12.03	10.55	22.80	26.02	25.94	0.46	-1.12
Solyc05g0	0.30	0.27	0.35	1.30	1.97	1.56	0.19	-2.39
Solyc02g0	6.51	7.12	7.54	2.36	2.75	2.31	2.85	1.51
Solyc09g0	10.06	11.23	11.18	24.96	28.30	24.51	0.42	-1.26
Solyc09g0	0	0.06	0.13	9.41	9.15	11.04	0.01	-7.29
Solyc12g0	1.73	1.70	2.05	0.31	0.18	0.22	7.81	2.96
Solyc06g0	5.97	5.88	5.63	0.55	1.24	0.55	7.48	2.90
Solyc04g0	1.71	1.56	1.46	7.26	6.91	6.92	0.22	-2.15
Solyc01g0	1.16	1.71	1.51	6.91	6.75	6.73	0.21	-2.22
Solyc06g0	0.61	0.68	0.37	5.60	5.19	4.32	0.11	-3.19
Solyc07g0	325.55	288.02	321.41	39.36	21.62	21.77	11.30	3.50
Solyc12g0	0.17	0	0.40	35.52	26.11	29.51	0.01	-7.31
Solyc07g0	5.06	5.23	5.42	10.47	11.07	10.93	0.48	-1.05
Solyc06g0	12.13	11.01	11.83	4.95	3.55	4.27	2.74	1.45
Solyc03g1	40.19	35.94	43.76	20.79	18.13	16.48	2.16	1.11
Solyc07g0	5.19	6.12	5.35	2.31	2.65	2.72	2.17	1.12
Solyc03g1	2.38	2.04	1.88	0.50	0.42	0.45	4.61	2.21
Solyc03g0	2.74	2.80	3.02	0.79	0.52	0.58	4.53	2.18
Solyc04g0	1.62	1.62	1.46	0.30	0.13	0.30	6.45	2.69
Solyc06g0	2.80	2.54	2.94	7.60	7.97	9.16	0.33	-1.58
Solyc10g0	0.31	0.17	0.29	1.76	1.96	2.22	0.13	-2.96
Solyc02g0	0.24	0.46	0.32	1.84	2.26	2.72	0.15	-2.74
Solyc08g0	2.40	2.13	2.32	0.60	0.44	0.53	4.36	2.13
Solyc09g0	43.14	45.45	41.87	77.92	102.63	94.24	0.47	-1.07
Solyc01g0	41.94	40.50	37.70	20.92	19.38	19.74	2.00	1.00
Solyc01g1	0.04	0.04	0.02	1.23	1.17	0.83	0.03	-5.02
Solyc12g0	4.57	4.64	4.26	12.42	12.40	11.51	0.37	-1.43
Solyc07g0	2.63	2.66	2.63	0.45	0.54	0.29	6.21	2.63
Solyc05g0	17.10	18.89	18.53	32.42	39.32	39.37	0.49	-1.03
Solyc10g0	8.38	7.76	7.87	3.18	2.16	1.91	3.31	1.73
Solyc11g0	6.45	7.06	7.16	19.35	17.28	16.07	0.39	-1.35
Solyc09g0	6.11	6.86	6.00	1.83	2.12	1.43	3.53	1.82
Solyc09g0	4.49	4.72	5.17	0.35	0.27	0.08	20.47	4.36
Solyc07g0	21692.13	20498.43	20744.43	6919.72	4983.38	4797.90	3.77	1.91
Solyc02g0	6.49	7.06	7.33	13.88	17.01	16.75	0.44	-1.19
Solyc09g0	12.57	12.82	13.11	23.56	29.18	27.27	0.48	-1.06
Solyc03g0	7.61	6.77	6.13	2.21	2.31	2.52	2.92	1.54
Solyc05g0	28.25	27.85	24.22	12.32	11.39	10.28	2.36	1.24
Solyc07g0	1.38	1.32	1.29	0.62	0.61	0.60	2.19	1.13
Solyc05g0	2.82	2.89	2.77	5.70	5.98	5.75	0.49	-1.04
Solyc11g0	1.64	1.42	1.52	4.53	4.10	4.40	0.35	-1.51
Solyc10g0	12.71	11.33	12.32	3.91	2.33	3.24	3.83	1.94
Solyc05g0	5.34	4.95	4.93	1.46	0.96	0.96	4.51	2.17
Solyc12g0	4.14	4.32	3.47	0.75	0.87	0.62	5.32	2.41
Solyc09g0	3.63	4.43	4.33	14.01	15.32	16.85	0.27	-1.90
Solyc06g0	19.50	19.69	18.44	36.75	47.18	40.82	0.46	-1.11
Solyc07g0	73.81	67.71	68.92	148.46	141.33	134.52	0.50	-1.01
Solyc01g1	2.04	2.13	2.26	0.94	0.90	0.93	2.32	1.22

Solyc08g0	15.15	18.42	16.19	34.11	43.88	41.54	0.42	-1.26
Solyc02g0	6.48	6.72	6.05	18.61	15.85	16.53	0.38	-1.41
Solyc01g1	642.80	605.44	606.76	365.10	276.98	256.82	2.06	1.05
Solyc03g0	27.63	32.76	33.39	15.12	16.59	13.72	2.06	1.05
Solyc05g0	3.38	4.12	3.29	0.70	0.90	0.82	4.45	2.15
Solyc11g0	0.33	0.31	0.30	1.20	1.33	1.19	0.25	-1.98
Solyc08g0	3.85	3.14	2.86	9.20	8.94	10.00	0.35	-1.51
Solyc10g0	7.60	9.34	7.88	3.53	3.72	3.19	2.38	1.25
Solyc02g0	140.31	143.93	144.47	84.57	63.90	64.35	2.01	1.01
Solyc04g0	3.42	3.56	3.14	7.28	7.86	8.66	0.43	-1.23
Solyc08g0	2.12	2.40	2.20	5.01	5.78	5.48	0.41	-1.27
Solyc09g0	1.74	1.38	1.65	5.54	8.04	7.23	0.23	-2.13
Solyc03g0	6.03	5.83	6.33	1.66	0.85	1.22	4.87	2.29
Solyc03g1	11.76	11.91	13.26	26.29	28.86	25.45	0.46	-1.13
Solyc01g0	0.40	0.26	0.23	3.53	2.70	2.88	0.10	-3.35
Solyc10g0	0.19	0.09	0.07	1.62	1.64	1.84	0.07	-3.89
Solyc05g0	18.52	21.51	20.30	10.07	8.64	9.86	2.11	1.08
Solyc01g0	3.79	3.90	4.35	0.49	0.93	0.87	5.24	2.39
Solyc07g0	4.31	3.46	4.29	1.40	1.35	1.31	2.96	1.57
Solyc12g0	26.74	19.59	25.58	7.21	7.93	5.86	3.42	1.78
Solyc02g0	3.91	4.64	4.38	9.16	9.66	9.66	0.45	-1.14
Solyc02g0	4.33	4.78	3.95	19.98	18.84	13.61	0.25	-2.01
Solyc11g0	6.32	6.42	6.83	2.76	2.70	2.82	2.36	1.24
Solyc01g0	13.11	13.19	11.67	5.27	5.84	4.39	2.45	1.29
Solyc01g0	15.59	14.60	13.43	32.58	36.54	29.70	0.44	-1.18
Solyc11g0	0	0.10	0.08	2.47	2.31	2.53	0.02	-5.38
Solyc10g0	2.78	2.52	2.63	6.49	6.25	7.56	0.39	-1.36
Solyc01g1	1.89	1.92	2.00	4.20	4.70	4.44	0.44	-1.20
Solyc03g0	0.13	0.18	0.30	2.96	2.36	3.11	0.07	-3.80
Solyc05g0	1.74	1.58	1.36	4.18	4.64	4.62	0.35	-1.52
Solyc06g0	3.59	3.57	3.77	1.41	1.19	1.02	3.01	1.59
Solyc06g0	15.32	15.12	16.50	29.63	34.32	31.64	0.49	-1.03
Solyc07g0	10.73	9.85	10.41	5.10	4.35	4.60	2.20	1.14
Solyc02g0	11.74	11.97	11.48	4.97	5.63	4.60	2.31	1.21
Solyc06g0	4.68	5.69	4.70	2.17	2.19	2.05	2.35	1.23
Solyc03g0	23.84	26.47	22.42	3.83	2.04	1.50	9.87	3.30
Solyc04g0	4.67	4.37	5.02	9.55	12.57	12.22	0.41	-1.29
Solyc08g0	2.16	2.36	2.09	4.13	4.69	4.60	0.49	-1.02
Solyc10g0	8.29	9.60	11.21	2.95	3.28	2.39	3.38	1.76
Solyc06g0	25.90	26.73	25.62	12.28	7.92	9.16	2.67	1.41
Solyc11g0	5.63	5.16	5.72	11.40	12.78	11.97	0.46	-1.13
Solyc05g0	5.41	4.60	4.89	1.98	1.68	1.65	2.81	1.49
Solyc01g0	0.15	0.68	0.31	9.10	8.37	9.50	0.04	-4.56
Solyc04g0	4.19	3.89	3.62	9.26	10.34	11.44	0.38	-1.41
Solyc06g0	1.14	1.33	1.36	5.95	5.22	6.27	0.22	-2.18
Solyc08g0	5.36	6.68	6.50	2.79	2.92	2.81	2.18	1.12
Solyc11g0	8.23	8.27	7.49	3.69	4.38	3.50	2.07	1.05
Solyc09g0	1.87	1.65	1.85	5.16	4.73	4.87	0.36	-1.46
Solyc11g0	4.04	3.56	4.48	9.41	10.10	11.04	0.40	-1.34
Solyc01g0	0.98	1.02	0.76	2.74	3.15	3.38	0.30	-1.75
Solyc04g0	1.12	1.13	1.84	6.76	6.56	7.36	0.20	-2.34

Solyc06g0	16.42	17.16	17.17	9.45	8.39	7.23	2.02	1.02
Solyc01g1	3.36	3.76	3.66	7.64	8.51	7.66	0.45	-1.14
ENSRNA(	273.86	214.78	281.74	134.77	101.40	129.04	2.11	1.08
Solyc04g0	1.96	2.22	1.79	4.59	4.72	5.18	0.41	-1.28
Solyc07g0	1.86	1.73	2.12	0.35	0.38	0.47	4.78	2.26
Solyc04g0	0.35	0.64	0.26	3.68	5.06	3.48	0.10	-3.29
Solyc01g1	3.66	3.96	4.02	1.03	1.35	1.48	3.01	1.59
Solyc11g0	3.18	2.83	3.35	1.04	0.99	1.15	2.94	1.56
Solyc01g0	2.19	2.15	2.43	0.70	0.66	0.55	3.54	1.82
Solyc06g0	4.77	4.39	5.00	1.32	0.91	0.72	4.81	2.27
Solyc06g0	7.56	6.93	7.29	18.96	18.51	17.46	0.40	-1.33
Solyc02g0	2.06	2.54	2.96	0.47	0.26	0.39	6.74	2.75
Solyc01g0	1.34	1.92	2.03	0.21	0.18	0.18	9.30	3.22
Solyc12g0	6.57	6.15	6.06	3.27	2.99	3.08	2.01	1.01
Solyc04g0	2.20	2.15	2.17	5.21	4.67	5.80	0.42	-1.27
Solyc11g0	63.76	65.44	65.46	33.91	21.25	21.66	2.53	1.34
Solyc02g0	3.68	3.88	3.80	6.73	8.14	7.87	0.50	-1.00
Solyc07g0	4.83	4.90	5.11	1.70	1.52	1.31	3.28	1.71
Solyc06g0	2.91	2.56	2.99	7.43	7.01	6.43	0.41	-1.30
Solyc06g0	4.79	5.49	5.03	2.46	2.33	2.52	2.09	1.07
Solyc03g0	2.46	2.88	2.59	12.35	10.97	9.74	0.24	-2.06
Solyc08g0	5.19	5.65	5.23	2.16	1.92	1.68	2.79	1.48
Solyc10g0	0.61	0.81	0.90	0.10	0.05	0.07	10.66	3.41
Solyc03g1	3.00	2.86	2.91	6.35	5.68	6.31	0.48	-1.06
Solyc06g0	10.39	9.39	11.15	4.90	4.39	4.77	2.20	1.14
Solyc06g0	579.23	529.52	552.25	46.71	23.03	17.24	19.10	4.26
Solyc12g0	21.83	21.78	22.47	12.62	9.09	9.71	2.10	1.07
Solyc03g1	5.96	6.20	6.28	14.90	18.21	15.90	0.38	-1.41
Solyc03g1	0.77	1.05	1.02	3.51	4.39	3.65	0.25	-2.02
Solyc12g0	0.72	0.69	0.73	1.99	2.43	2.58	0.31	-1.70
Solyc07g0	22.71	21.60	19.87	40.39	53.00	44.74	0.46	-1.11
Solyc08g0	8.57	8.07	11.00	2.47	2.22	2.25	3.98	1.99
Solyc04g0	32.82	32.85	31.64	17.58	13.73	12.55	2.22	1.15
Solyc06g0	13.40	12.14	14.81	7.03	7.23	5.77	2.01	1.01
Solyc02g0	3.04	3.55	3.63	1.14	0.89	0.85	3.55	1.83
Solyc10g0	4.32	4.13	4.26	1.31	1.24	1.13	3.46	1.79
Solyc09g0	5.78	5.83	6.86	11.95	15.13	13.85	0.45	-1.15
Solyc12g0	9.82	10.78	10.56	3.39	5.42	4.13	2.41	1.27
Solyc03g0	4.76	4.98	4.55	2.58	2.33	1.81	2.12	1.09
Solyc10g0	4.66	4.73	4.80	1.87	2.03	1.57	2.60	1.38
Solyc12g0	18.21	20.90	20.39	9.78	8.90	8.12	2.22	1.15
Solyc05g0	3.60	3.21	3.73	0.43	0.64	0.90	5.36	2.42
Solyc08g0	9.28	9.02	9.55	21.01	27.76	23.64	0.38	-1.38
Solyc06g0	37.84	38.35	38.57	114.21	81.15	79.78	0.42	-1.26
Solyc12g0	1.85	1.93	1.65	4.42	4.93	5.61	0.36	-1.46
Solyc03g0	4.40	4.34	4.21	1.54	1.28	1.82	2.79	1.48
Solyc04g0	3.66	2.70	3.33	0.81	0.56	0.75	4.57	2.19
Solyc02g0	8.54	6.67	7.32	3.01	2.59	3.00	2.62	1.39
Solyc05g0	0.52	0.52	0.65	1.79	2.42	2.00	0.27	-1.87
Solyc07g0	3.10	3.02	3.53	0.70	0.98	0.62	4.20	2.07
Solyc09g0	0.24	0.49	0.28	2.51	2.42	2.32	0.14	-2.85

Solyc04g0	2.89	3.08	2.95	1.40	1.51	1.44	2.05	1.04
Solyc12g0	14.88	13.32	13.34	4.63	2.08	2.93	4.31	2.11
Solyc10g0	9.41	9.32	8.41	4.03	2.79	2.50	2.91	1.54
Solyc03g1	4.35	4.53	4.52	2.06	2.61	1.91	2.04	1.03
Solyc06g0	3.38	3.01	3.19	7.81	9.06	6.92	0.40	-1.31
Solyc12g0	4.91	5.28	5.38	11.15	11.39	11.90	0.45	-1.15
Solyc11g0	0	0	0	5.85	7.74	6.03	0.00	-16.00
Solyc02g0	7.76	8.91	8.11	3.73	2.37	2.39	2.92	1.55
Solyc01g0	5.15	5.20	4.66	2.11	2.42	2.11	2.26	1.18
Solyc12g0	24.50	21.00	21.91	10.79	11.34	9.26	2.15	1.10
Solyc03g0	11.96	10.55	10.09	4.39	6.02	5.17	2.09	1.07
Solyc10g0	5.10	5.44	5.32	2.28	2.98	2.17	2.14	1.10
Solyc06g0	2.70	2.81	1.93	7.89	8.69	7.43	0.31	-1.69
Solyc08g0	7.42	7.53	8.42	2.04	0.82	1.46	5.40	2.43
Solyc05g0	4.98	6.60	7.64	1.75	2.48	1.79	3.20	1.68
Solyc04g0	122.52	111.64	111.18	62.18	38.19	39.57	2.47	1.30
Solyc02g0	0.78	0.81	0.83	0.03	0.10	0.05	14.41	3.85
Solyc03g1	0.72	0.75	0.82	1.88	1.96	2.10	0.38	-1.38
Solyc02g0	5.51	4.49	3.84	13.82	12.85	14.90	0.33	-1.59
Solyc10g0	17.46	20.21	18.60	8.84	7.03	9.15	2.25	1.17
Solyc05g0	2.31	2.64	2.91	0.96	0.80	0.68	3.22	1.69
Solyc04g0	7.05	7.02	7.83	16.02	18.53	17.34	0.42	-1.24
Solyc01g1	3.10	3.51	2.58	1.19	1.10	1.21	2.62	1.39
Solyc07g0	4.47	5.21	4.66	10.25	17.03	15.30	0.34	-1.57
Solyc02g0	4.50	3.55	4.42	9.32	9.38	9.25	0.45	-1.16
Solyc02g0	0.51	0.09	0	20.55	13.28	12.46	0.01	-6.27
Solyc10g0	3.17	2.95	3.34	1.00	1.20	1.15	2.83	1.50
Solyc10g0	9.35	10.53	9.98	4.50	4.15	3.00	2.56	1.36
Solyc09g0	3.57	3.44	3.40	11.20	9.71	8.73	0.35	-1.51
Solyc09g0	19.10	16.94	19.52	10.54	7.96	9.09	2.01	1.01
Solyc03g0	0.27	0.39	0.34	1.71	2.08	1.62	0.18	-2.45
Solyc02g0	2.52	2.88	3.21	9.97	9.25	11.32	0.28	-1.83
Solyc03g1	3.80	3.79	3.12	11.76	10.43	9.33	0.34	-1.56
Solyc08g0	0.68	0.57	0.93	3.55	3.65	3.90	0.20	-2.35
Solyc02g0	5.05	5.65	6.04	10.74	11.54	11.62	0.49	-1.02
Solyc05g0	0.23	0.44	0.43	4.58	3.91	4.59	0.08	-3.58
Solyc10g0	14.19	13.98	11.86	5.42	3.02	3.57	3.33	1.74
Solyc03g0	23.87	19.69	20.97	12.63	9.37	8.87	2.09	1.06
Solyc06g0	5.42	5.59	5.32	12.28	11.00	11.93	0.46	-1.11
Solyc02g0	2.08	2.42	2.09	5.11	5.21	5.21	0.42	-1.24
Solyc10g0	17.53	18.76	18.00	6.66	10.22	7.98	2.18	1.13
Solyc11g0	0	0.09	0.10	3.94	4.52	3.49	0.02	-5.93
Solyc02g0	2.71	2.99	3.00	9.69	9.81	12.28	0.27	-1.87
Solyc07g0	10.78	10.23	10.07	0.92	0.44	0	22.90	4.52
Solyc09g0	8.91	8.80	9.02	18.97	15.88	18.63	0.50	-1.00
Solyc03g1	9.41	9.45	8.89	3.08	1.29	1.74	4.55	2.18
Solyc08g0	2.20	2.22	2.19	4.76	5.08	5.42	0.43	-1.21
Solyc09g0	14.25	13.58	12.78	5.47	3.35	3.09	3.41	1.77
Solyc04g0	3.54	3.55	3.18	0.71	0.52	0.94	4.75	2.25
Solyc07g0	2.26	2.38	2.41	0.62	0.58	0.67	3.79	1.92
Solyc01g0	9.04	7.75	7.54	18.85	18.17	17.46	0.45	-1.16

Solyc05g0	4.28	3.45	3.82	1.70	1.69	1.78	2.24	1.16
Solyc06g0	2.33	2.35	2.86	6.85	7.65	5.89	0.37	-1.44
Solyc11g0	4.06	4.50	4.40	7.93	9.49	8.70	0.50	-1.01
Solyc06g0	10.60	10.60	8.62	4.95	4.79	3.86	2.19	1.13
Solyc06g0	2.09	2.71	2.76	0.60	0.76	0.38	4.34	2.12
Solyc01g0	2.97	3.08	3.23	7.14	6.35	6.62	0.46	-1.12
Solyc09g0	1.61	2.13	1.94	4.96	5.19	5.18	0.37	-1.43
Solyc03g0	1.93	2.14	2.36	0.67	0.52	0.46	3.90	1.96
Solyc09g0	2.89	2.53	2.97	5.80	5.62	5.80	0.49	-1.04
Solyc05g0	1.27	1.08	0.96	3.39	4.52	3.78	0.28	-1.82
Solyc06g0	0.98	1.13	0.81	3.19	4.00	3.71	0.27	-1.90
Solyc11g0	0.45	0.83	0.60	2.74	2.54	2.43	0.24	-2.04
Solyc10g0	1.48	1.56	1.46	0.56	0.40	0.66	2.78	1.48
Solyc02g0	1.18	0.94	0.88	0.08	0.03	0.04	19.98	4.32
Solyc08g0	10.96	9.63	9.27	4.49	3.52	3.47	2.60	1.38
Solyc01g1	1.42	1.62	1.28	0.61	0.66	0.71	2.18	1.13
Solyc08g0	0.06	0.09	0.16	0.79	0.79	0.93	0.12	-3.05
Solyc02g0	2.08	1.76	1.49	6.22	5.37	6.72	0.29	-1.78
Solyc02g0	24.07	25.57	22.82	6.59	4.42	3.42	5.02	2.33
Solyc11g0	13.12	13.82	12.91	22.19	31.08	28.57	0.49	-1.04
Solyc04g0	2.55	2.70	2.78	1.22	1.13	1.53	2.07	1.05
Solyc05g0	3.67	3.86	3.30	7.41	7.09	7.18	0.50	-1.00
Solyc08g0	0.62	0.77	0.80	2.13	1.90	2.46	0.34	-1.57
Solyc03g1	4.10	2.80	3.58	0.74	0.22	0.31	8.25	3.04
Solyc08g0	7.25	7.36	8.95	3.58	3.88	3.42	2.16	1.11
Solyc11g0	20.45	19.01	18.87	11.07	6.58	7.63	2.31	1.21
Solyc11g0	0	0	0	8.51	9.43	9.29	0.00	-16.47
Solyc04g0	1.22	1.80	1.80	0.29	0.16	0.20	7.43	2.89
Solyc05g0	2.20	2.06	1.90	0	0	0	20549.37	14.33
Solyc11g0	1.78	1.83	1.75	0	0	0	17863.20	14.12
Solyc06g0	2.20	2.59	2.77	7.54	7.69	7.20	0.34	-1.57
Solyc01g0	2.46	2.32	2.71	0.24	0.17	0.28	10.73	3.42
Solyc10g0	1.63	1.82	1.82	3.59	4.18	4.48	0.43	-1.22
Solyc03g0	57.11	51.28	52.00	20.72	12.01	8.20	3.92	1.97
Solyc08g0	2.76	3.41	3.28	6.44	8.12	7.30	0.43	-1.21
Solyc03g1	3.07	4.03	4.16	7.97	9.51	8.55	0.43	-1.21
Solyc09g0	0	0	0	9.56	8.14	10.53	0.00	-16.52
Solyc08g0	5.65	5.38	5.74	11.47	10.72	12.18	0.49	-1.03
Solyc02g0	5.22	3.74	3.66	1.37	1.15	1.60	3.06	1.62
Solyc01g0	7.58	7.82	7.67	15.57	17.26	14.71	0.49	-1.04
Solyc05g0	10.99	11.94	11.52	24.65	20.28	27.86	0.47	-1.08
Solyc09g0	13.95	14.56	16.78	6.02	5.77	5.57	2.61	1.38
Solyc01g0	2.24	1.96	2.03	0.88	0.57	0.61	3.02	1.60
Solyc10g0	4.51	3.43	3.67	1.57	1.62	1.73	2.36	1.24
Solyc07g0	3.91	4.72	5.19	0.22	0	0	61.65	5.95
Solyc01g0	25.99	22.98	25.42	12.93	8.99	9.47	2.37	1.24
Solyc08g0	10.58	10.09	9.50	25.51	23.20	19.99	0.44	-1.19
Solyc06g0	1.19	1.67	2.03	0	0	0	16269.40	13.99
Solyc02g0	1.86	1.52	2.17	5.27	6.08	5.04	0.34	-1.56
Solyc02g0	1.84	1.86	2.04	0.17	0	0.12	20.11	4.33
Solyc05g0	22.76	20.56	20.37	50.63	46.91	49.08	0.43	-1.20

Solyc01g1	1.61	1.40	1.56	0.39	0.42	0.52	3.44	1.78
ENSRNA(	30.85	23.09	31.46	13.62	8.68	12.52	2.45	1.29
Solyc02g0	5.18	5.26	4.59	2.38	1.53	2.05	2.52	1.33
Solyc01g0	2.84	3.32	3.03	1.22	1.36	1.65	2.17	1.11
Solyc05g0	2.64	3.21	2.83	1.03	1.56	1.38	2.18	1.12
Solyc05g0	0.46	0.41	0.35	1.96	1.63	1.86	0.22	-2.15
Solyc03g1	0	0.00	0.10	23.02	22.84	17.93	0.00	-9.23
Solyc08g0	1.72	1.37	1.60	0.55	0.47	0.40	3.31	1.73
Solyc11g0	2.54	2.25	2.99	0.67	0.56	0.97	3.53	1.82
Solyc02g0	7.83	6.70	7.88	3.48	3.42	3.88	2.08	1.06
Solyc09g0	1.13	0.71	0.55	3.99	4.94	4.64	0.18	-2.50
Solyc09g0	0	0.30	0.11	5.18	4.05	3.66	0.03	-4.98
Solyc02g0	0.82	0.87	0.83	2.23	2.43	2.53	0.35	-1.51
Solyc06g0	1.34	1.13	1.11	4.87	10.64	9.54	0.14	-2.80
Solyc02g0	0.81	0.76	0.63	2.38	2.07	3.45	0.28	-1.84
Solyc01g0	1.85	1.97	1.91	4.51	4.89	4.33	0.42	-1.26
Solyc08g0	1.67	1.38	1.87	0.54	0.40	0.36	3.77	1.91
Solyc02g0	3.72	3.27	2.97	1.16	1.50	1.26	2.54	1.34
Solyc01g1	3.15	3.70	3.95	8.86	9.69	10.11	0.38	-1.41
Solyc12g0	5.49	4.71	5.69	2.66	2.23	2.74	2.08	1.06
Solyc01g0	4.87	4.20	4.23	0	0	0	44322.86	15.44
Solyc09g0	0.13	0.28	0.16	2.89	4.60	4.38	0.05	-4.37
Solyc08g0	11.21	10.35	9.63	4.36	3.67	5.04	2.39	1.25
Solyc03g1	8.84	11.88	14.72	3.45	1.93	1.14	5.44	2.44
Solyc06g0	9.95	10.33	9.52	5.60	4.07	3.29	2.30	1.20
Solyc03g0	2.31	2.35	2.55	0	0	0	24042.82	14.55
Solyc05g0	45.84	48.10	49.05	26.67	18.28	16.05	2.34	1.23
Solyc08g0	1.34	1.05	1.32	0.40	0.38	0.34	3.33	1.74
Solyc03g0	5.76	5.01	4.25	29.63	19.83	16.68	0.23	-2.14
Solyc09g0	3.45	2.89	2.59	6.59	7.35	6.48	0.44	-1.19
Solyc10g0	3.51	3.59	3.79	1.66	1.86	1.36	2.23	1.16
Solyc10g0	399.66	361.89	381.97	95.52	47.71	51.10	5.88	2.56
Solyc09g0	1.23	1.08	1.33	0.43	0.47	0.44	2.71	1.44
Solyc12g0	0.26	0.39	0.30	1.69	1.86	1.87	0.17	-2.52
Solyc02g0	34.96	32.07	31.27	17.98	15.19	12.42	2.16	1.11
Solyc12g0	4.99	5.65	5.03	1.19	2.45	1.80	2.88	1.52
Solyc07g0	5.91	5.20	4.33	1.45	1.26	1.39	3.77	1.91
Solyc07g0	0.46	0.54	0.43	0	0	0	4759.57	12.22
Solyc05g0	10.20	11.62	11.31	6.13	5.30	4.61	2.07	1.05
Solyc06g0	60.10	53.49	55.96	32.07	18.35	18.03	2.48	1.31
Solyc06g0	0.17	0.19	0.08	1.10	0.86	1.03	0.15	-2.75
Solyc03g0	1.22	1.15	1.25	0.21	0.40	0.14	4.81	2.26
Solyc01g0	1.38	1.44	1.61	0.38	0.44	0.39	3.70	1.89
Solyc06g0	3.05	3.13	2.67	0	0	0	29487.63	14.85
Solyc05g0	2.14	2.64	2.86	0.50	0.87	0.57	3.96	1.99
Solyc07g0	8.87	9.37	6.45	2.63	1.10	0.97	5.25	2.39
Solyc01g1	4.71	3.48	5.24	0	0	0	44789.38	15.45
Solyc01g0	15.54	11.21	11.59	37.08	36.58	30.20	0.37	-1.44
Solyc03g0	0.43	0.39	0.56	2.00	2.19	1.95	0.22	-2.16
Solyc09g0	1.63	1.65	1.69	4.23	4.93	4.50	0.36	-1.46
Solyc07g0	3.18	2.32	2.49	7.41	6.84	7.21	0.37	-1.43

Solyc03g1	2.71	2.36	2.14	0.74	0.69	0.43	3.89	1.96
Solyc11g0	13.32	12.90	11.88	5.19	3.60	3.75	3.04	1.60
Solyc02g0	1.89	2.08	1.78	0.88	0.59	0.46	2.97	1.57
Solyc03g1	5.23	4.79	4.41	2.74	2.34	2.01	2.04	1.03
Solyc02g0	0.54	0.73	0.53	2.09	1.44	2.62	0.29	-1.77
Solyc09g0	6.02	5.61	5.32	2.95	3.09	2.13	2.07	1.05
Solyc04g0	0.49	0.58	0.71	1.63	2.54	2.19	0.28	-1.84
Solyc05g0	2.04	2.26	2.09	0.65	0.62	0.38	3.86	1.95
Solyc01g0	1.24	1.29	1.21	4.34	3.71	4.32	0.30	-1.72
Solyc05g0	3.97	3.24	4.56	1.47	1.13	0.95	3.31	1.73
Solyc12g0	4.58	3.93	3.87	8.33	10.47	8.69	0.45	-1.15
Solyc11g0	10.89	10.48	10.23	5.69	4.07	5.51	2.07	1.05
Solyc11g0	0.74	0.61	0.85	0.12	0.06	0.16	6.63	2.73
Solyc04g0	0.60	0.52	0.49	1.41	1.44	1.53	0.37	-1.44
Solyc09g0	0.31	0.34	0.40	2.69	3.37	2.37	0.12	-3.00
Solyc09g0	0.32	0.83	0.37	4.28	4.04	4.57	0.12	-3.08
Solyc02g0	25.14	26.76	25.98	15.45	8.45	10.16	2.29	1.19
Solyc05g0	3.27	4.65	4.43	1.34	1.03	0.57	4.20	2.07
Solyc01g0	2.08	2.19	1.98	4.43	5.07	4.76	0.44	-1.19
Solyc06g0	1.27	1.54	0.96	5.65	5.47	6.32	0.22	-2.21
Solyc02g0	5.98	6.12	5.66	3.08	2.09	1.77	2.56	1.36
Solyc06g0	1.89	2.74	1.95	0.61	0.56	0.30	4.45	2.15
Solyc11g0	1.01	1.00	1.15	0.23	0.14	0.08	7.03	2.81
Solyc11g0	0.13	0.11	0.11	0.32	0.35	0.42	0.32	-1.67
Solyc05g0	0.85	1.46	1.19	0	0	0	11629.84	13.51
Solyc08g0	0	0.05	0	7.40	6.98	6.88	0.00	-8.88
Solyc01g1	4.26	4.60	4.55	10.35	7.74	10.27	0.47	-1.08
Solyc02g0	1.23	1.61	1.32	2.92	2.84	3.06	0.47	-1.08
Solyc02g0	4.83	4.10	4.32	10.68	10.35	9.69	0.43	-1.21
Solyc03g0	0.49	0.55	0.50	1.08	1.25	1.18	0.44	-1.19
Solyc01g1	2.53	2.18	2.41	0.85	1.07	1.14	2.32	1.21
Solyc01g0	6.66	6.35	5.71	1.62	1.18	0.77	5.24	2.39
Solyc07g0	306.87	292.46	308.85	131.16	83.89	82.68	3.05	1.61
Solyc09g0	2.45	1.59	2.39	0.09	0	0	68.63	6.10
Solyc06g0	0.74	0.74	0.90	0.23	0.21	0.25	3.42	1.77
Solyc02g0	2.95	3.29	3.21	1.08	0.46	0.42	4.84	2.28
Solyc12g0	5.30	5.26	4.68	1.93	2.73	1.74	2.38	1.25
Solyc09g0	4.55	4.44	3.70	7.93	10.21	8.51	0.48	-1.07
Solyc05g0	1.61	1.32	1.72	0.68	0.60	0.57	2.50	1.32
Solyc10g0	1.51	2.10	0.99	11.90	8.99	8.14	0.16	-2.66
Solyc05g0	0.87	1.08	1.09	2.30	3.19	3.36	0.34	-1.54
Solyc01g0	0.53	0.45	0.67	0.13	0.09	0.10	5.13	2.36
Solyc07g0	1.44	1.87	1.61	0.57	0.58	0.70	2.66	1.41
Solyc01g1	0.43	0.37	0.22	0.04	0.01	0.02	14.96	3.90
Solyc02g0	1.06	1.12	0.98	4.05	4.08	3.66	0.27	-1.90
Solyc03g0	9.33	11.50	8.96	4.12	1.84	2.45	3.54	1.82
Solyc04g0	2.43	1.85	1.55	5.53	7.46	6.74	0.30	-1.76
Solyc01g1	1.18	1.26	1.35	0.40	0.48	0.39	2.98	1.58
Solyc04g0	4.93	4.29	4.73	1.76	0.63	0.68	4.53	2.18
Solyc01g0	2.00	2.31	2.36	4.31	4.33	5.01	0.49	-1.03
Solyc02g0	24.03	22.90	22.31	13.14	7.77	7.28	2.46	1.30

Solyc05g0	5.15	5.27	6.99	1.07	0.37	1.08	6.89	2.79
Solyc06g0	2.31	2.16	2.09	0.47	0.86	0.52	3.53	1.82
Solyc06g0	3.09	2.93	3.18	1.54	1.04	1.36	2.33	1.22
Solyc09g0	1.92	2.69	2.08	0.75	0.59	0.64	3.37	1.75
Solyc07g0	15.03	13.71	12.99	7.96	6.69	4.72	2.15	1.11
Solyc07g0	2.69	1.85	2.35	0.60	0.51	0.45	4.41	2.14
Solyc12g0	0.98	0.53	0.62	0	0	0	7107.62	12.80
ENSRNA(	18.28	13.98	17.03	8.77	5.61	7.06	2.30	1.20
Solyc04g0	6.72	7.16	6.94	2.74	2.28	1.36	3.26	1.71
Solyc01g0	1.22	1.36	1.57	0.50	0.64	0.60	2.38	1.25
Solyc09g0	5.46	4.69	3.65	1.32	1.34	1.65	3.19	1.68
Solyc11g0	0.08	0.17	0.31	3.50	1.69	2.76	0.07	-3.83
Solyc11g0	1.21	1.20	1.51	0.06	0.15	0	18.46	4.21
Solyc01g0	2.78	2.93	1.64	0.54	0.52	0.38	5.13	2.36
Solyc11g0	0.77	0.78	0.90	0.25	0.23	0.27	3.29	1.72
Solyc04g0	0.33	0.29	0.41	1.73	1.86	1.38	0.21	-2.27
Solyc05g0	0.34	0.28	0.40	0.09	0.13	0.11	3.13	1.65
Solyc07g0	2.90	3.23	3.11	6.13	8.17	7.80	0.42	-1.26
Solyc04g0	1.32	1.69	1.66	3.91	3.38	3.65	0.43	-1.23
Solyc07g0	1.59	1.78	1.88	4.05	4.30	4.69	0.40	-1.31
Solyc06g0	2.71	3.07	3.02	1.27	0.73	0.71	3.25	1.70
Solyc05g0	2.46	2.49	1.88	4.46	5.52	5.54	0.44	-1.19
Solyc02g0	2.51	1.76	2.47	5.00	6.58	6.05	0.38	-1.39
Solyc08g0	4.21	4.12	4.31	8.77	9.16	8.15	0.48	-1.05
Solyc06g0	0.65	0.58	0.88	0	0	0	7023.58	12.78
Solyc09g0	1.83	2.07	2.51	4.37	4.53	4.61	0.47	-1.07
Solyc01g0	1.53	1.11	1.03	0.29	0.08	0.18	6.67	2.74
Solyc12g0	1.38	1.28	1.45	0	0	0	13681.82	13.74
Solyc01g0	0.07	0.11	0	0.96	1.03	0.83	0.06	-3.97
Solyc05g0	1.79	1.45	2.11	3.93	4.35	4.20	0.43	-1.22
Solyc10g0	1.15	0.97	0.96	0.07	0	0	43.00	5.43
Solyc01g1	6.86	6.19	5.18	1.89	1.53	1.40	3.78	1.92
Solyc09g0	4.08	3.97	3.93	8.47	8.94	7.41	0.48	-1.05
Solyc01g1	9.19	7.80	7.54	18.07	16.63	19.53	0.45	-1.14
Solyc03g1	1.46	1.46	1.59	2.86	3.16	3.11	0.49	-1.02
Solyc03g1	3.35	3.55	2.82	0.84	0.78	1.14	3.52	1.82
Solyc02g0	0.33	0.58	0.35	1.09	1.38	1.46	0.32	-1.64
Solyc02g0	2.09	1.64	1.70	0.74	0.50	0.56	3.02	1.60
Solyc07g0	3.02	3.33	3.63	1.28	1.62	1.89	2.09	1.06
Solyc02g0	2.59	2.77	2.45	1.19	1.16	1.06	2.28	1.19
Solyc01g1	21.35	23.78	22.48	2.79	1.11	0.65	14.86	3.89
Solyc02g0	424.10	387.21	396.08	205.36	141.27	135.48	2.50	1.32
ENSRNA(	11.35	10.58	13.54	5.17	4.16	4.95	2.48	1.31
Solyc10g0	2.50	2.41	2.27	1.05	0.96	1.15	2.27	1.19
Solyc04g0	0.65	1.13	1.51	5.15	6.77	6.04	0.18	-2.45
Solyc03g1	0.81	0.85	0.83	1.97	2.15	2.21	0.39	-1.35
Solyc05g0	4.02	3.03	3.53	1.15	0.35	0.77	4.68	2.23
Solyc12g0	13.78	13.59	14.36	36.21	35.54	33.59	0.40	-1.34
Solyc05g0	3.99	3.43	3.02	7.82	8.96	7.89	0.42	-1.24
Solyc10g0	1.78	2.35	2.46	0.68	0.29	0.45	4.66	2.22
Solyc05g0	4.66	3.43	4.87	1.30	1.04	1.71	3.20	1.68

Solyc12g0	2.53	2.02	1.93	0.79	0.51	0.31	4.01	2.00
Solyc03g0	1.12	0.77	1.00	0.11	0	0.08	15.63	3.97
Solyc09g0	0.42	0.70	0.34	3.00	2.18	2.11	0.20	-2.33
Solyc01g0	2.66	2.49	2.61	1.03	0.52	0.90	3.17	1.66
Solyc10g0	15.24	16.78	20.15	9.80	5.63	8.51	2.18	1.12
Solyc10g0	0.57	0.51	0.46	0.04	0	0	40.50	5.34
Solyc03g0	1.63	1.84	1.95	0.32	0.55	0.43	4.15	2.05
Solyc07g0	2.45	2.17	1.83	1.04	0.61	0.81	2.62	1.39
Solyc07g0	18.99	18.43	18.73	11.72	9.44	6.90	2.00	1.00
Solyc06g0	5.40	4.95	5.56	2.09	1.83	1.55	2.91	1.54
Solyc04g0	0.41	0.44	0.48	1.31	1.89	1.90	0.26	-1.94
Solyc06g0	0.22	0.23	0.30	0	0	0	2513.21	11.30
Solyc10g0	1.47	1.36	1.86	0	0	0	15621.48	13.93
Solyc10g0	2.68	2.74	2.80	0.91	1.31	1.19	2.41	1.27
Solyc12g0	1.49	1.57	1.95	0.54	0.38	0.50	3.54	1.82
Solyc01g0	20.95	23.15	22.13	12.73	7.23	8.80	2.30	1.20
Solyc02g0	0.82	0.97	0.85	0	0.04	0	64.34	6.01
Solyc09g0	1.60	1.25	1.18	3.49	3.91	4.02	0.35	-1.51
Solyc06g0	2.10	1.33	1.81	0.70	0.55	0.43	3.13	1.64
Solyc11g0	390.74	375.96	387.76	219.36	154.38	151.23	2.20	1.14
Solyc03g0	1.31	1.19	0.97	0.15	0.18	0.23	6.17	2.63
Solyc08g0	0.38	0.61	0.39	0	0	0	4598.17	12.17
Solyc09g0	3.07	3.32	3.46	13.42	9.30	7.58	0.32	-1.62
Solyc01g1	0	0	0	54.96	0	0	0.00	-17.48
Solyc09g0	11.21	7.52	8.30	3.36	2.34	3.31	3.00	1.59
Solyc11g0	0.94	0.94	0.93	0	0	0	9351.68	13.19
Solyc05g0	0.72	0.50	0.48	1.67	1.54	1.68	0.35	-1.52
Solyc03g1	1.67	1.87	1.59	4.46	4.15	3.66	0.42	-1.26
Solyc01g0	1.82	1.73	1.99	0.67	0.88	0.62	2.56	1.36
Solyc03g0	2.60	2.18	3.25	1.06	0.45	0.59	3.84	1.94
Solyc01g0	1.57	1.56	1.87	0.58	0.84	0.74	2.33	1.22
Solyc01g0	1.09	1.04	0.92	2.76	3.37	3.46	0.32	-1.65
Solyc10g0	1.12	1.31	0.93	0.16	0.01	0.02	17.76	4.15
Solyc04g0	0	0	0	0.41	0.40	0.39	0.00	-11.98
Solyc11g0	2.50	2.55	2.44	0.66	0.19	0.39	5.99	2.58
Solyc02g0	6.57	4.14	5.89	2.40	1.22	1.15	3.48	1.80
Solyc03g0	0.46	0.45	0.83	0.09	0.07	0.09	7.07	2.82
Solyc02g0	2.26	2.96	2.87	11.36	11.08	13.48	0.22	-2.15
Solyc03g0	1.90	1.74	1.86	3.95	4.26	3.96	0.45	-1.14
Solyc02g0	2.12	2.22	2.31	4.18	5.03	4.24	0.49	-1.02
Solyc09g0	18.10	15.17	19.11	7.60	6.61	4.11	2.86	1.52
Solyc04g0	0	0	0	1.06	1.61	1.95	0.00	-13.91
Solyc06g0	2.40	3.03	3.08	0.39	1.03	0.47	4.50	2.17
Solyc02g0	318.17	283.72	286.32	146.90	92.68	97.74	2.63	1.40
Solyc09g0	5.09	4.64	5.58	8.73	11.25	11.00	0.49	-1.02
Solyc12g0	2.15	2.14	2.09	1.13	0.90	0.69	2.35	1.23
Solyc02g0	0.35	0.38	0.37	1.11	1.31	1.66	0.27	-1.89
Solyc01g1	2.42	3.22	2.91	5.87	6.58	6.55	0.45	-1.15
Solyc03g1	0.43	0.41	0.49	0	0	0	4459.91	12.12
Solyc09g0	5.68	5.50	5.31	1.54	1.59	1.64	3.45	1.79
Solyc03g1	1.12	1.10	1.18	2.41	2.32	2.23	0.49	-1.04

Solyc03g0	0	0	88.91	0	0	0	296363.47	18.18
Solyc01g0	0.56	0.58	0.69	1.35	1.24	2.06	0.39	-1.35
Solyc09g0	2.50	2.60	2.57	0.83	0.65	0.33	4.23	2.08
Solyc03g0	2.57	3.53	2.96	0	0	0	30231.28	14.88
Solyc07g0	3.78	4.41	4.29	1.62	1.10	1.16	3.21	1.68
Solyc10g0	0.17	0	0.07	2.90	2.80	3.21	0.03	-5.19
Solyc10g0	2.74	2.68	2.26	1.30	1.48	1.03	2.02	1.01
Solyc02g0	2.52	2.25	2.22	14.65	7.28	8.13	0.23	-2.10
Solyc02g0	2.46	2.44	2.25	6.15	5.00	5.76	0.42	-1.24
Solyc04g0	0.59	0.60	0.84	0	0	0	6800.94	12.73
Solyc11g0	0	0	0	1.14	0.91	0.98	0.00	-13.30
Solyc06g0	8.14	5.76	6.90	17.22	17.04	14.21	0.43	-1.22
Solyc02g0	1.80	2.29	4.43	20.01	14.27	13.73	0.18	-2.49
Solyc10g0	1.15	1.11	1.51	0.17	0.05	0	16.69	4.06
Solyc11g0	0	0	0	7.10	5.54	5.64	0.00	-15.90
Solyc07g0	0.60	0.49	0.50	0	0.07	0.05	12.95	3.70
Solyc02g0	3.86	4.72	5.26	2.01	2.68	2.05	2.05	1.04
Solyc09g0	0.10	0	0	4.34	2.91	3.13	0.01	-6.72
Solyc04g0	1.81	2.03	1.78	0.75	0.89	0.75	2.36	1.24
Solyc12g0	0.86	1.43	1.42	0	0	0	12341.84	13.59
Solyc12g0	4.56	5.67	4.42	1.91	1.24	2.41	2.63	1.40
Solyc10g0	0.61	0.75	0.54	0.09	0.04	0.04	11.34	3.50
Solyc04g0	2.78	2.94	2.64	5.13	7.19	5.27	0.47	-1.07
Solyc11g0	2.47	2.76	2.61	1.17	0.97	1.31	2.27	1.18
Solyc01g1	1.97	1.57	1.37	0	0	0	16398.44	14.00
Solyc09g0	0	0	0	2.80	3.85	1.83	0.00	-14.79
Solyc01g0	0.32	0.39	0.34	0	0	0	3528.16	11.78
Solyc01g0	0.52	0.60	0.66	0.12	0.24	0.19	3.26	1.71
Solyc03g1	3.23	2.72	2.70	1.35	1.16	1.55	2.14	1.09
Solyc10g0	3.79	3.22	3.19	1.82	1.45	1.49	2.14	1.10
Solyc09g0	0.88	1.08	1.15	0.37	0.15	0.27	3.93	1.98
Solyc05g0	0	0	0	0	16.10	0	0.00	-15.71
Solyc08g0	1.77	1.59	1.56	0.25	0	0.10	14.34	3.84
Solyc01g0	0.77	0.99	0.86	1.84	1.79	1.77	0.49	-1.04
Solyc02g0	0	0	0	0.26	0.47	0.41	0.00	-11.89
Solyc11g0	1.55	1.37	1.49	0.61	0.58	0.43	2.73	1.45
Solyc04g0	11.62	10.64	12.02	6.75	5.09	4.21	2.14	1.09
Solyc01g0	4.62	4.08	3.77	2.04	2.47	1.66	2.02	1.02
Solyc09g0	1.15	0.84	1.27	0	0	0	10879.94	13.41
Solyc05g0	0	0	0	2.08	3.75	3.22	0.00	-14.88
Solyc06g0	0.24	0.18	0.20	1.48	1.69	1.22	0.14	-2.82
Solyc03g1	1.30	1.23	1.40	2.28	3.46	3.26	0.44	-1.20
Solyc02g0	1.93	2.35	2.19	0.79	0.70	0.41	3.42	1.77
Solyc06g0	1.95	2.01	1.80	0.50	0.86	0.90	2.54	1.35
Solyc05g0	2.17	1.87	1.08	0.27	0.35	0.12	6.90	2.79
Solyc01g0	2.88	2.09	2.25	0.89	0.94	1.03	2.52	1.33
ENSRNA(	138.06	106.36	144.30	66.30	44.56	60.28	2.27	1.18
Solyc09g0	22.65	23.66	25.75	4.98	1.80	1.59	8.61	3.11
Solyc11g0	0.29	0.71	0.52	0	0	0	5096.34	12.32
Solyc02g0	2.44	2.25	2.31	5.42	4.02	6.03	0.45	-1.15
Solyc12g0	1.66	1.63	1.26	0.20	0	0.07	17.00	4.09

Solyc06g0	2.58	2.23	2.43	0.99	1.11	1.06	2.29	1.20
Solyc08g0	2.60	2.84	2.19	1.25	0.87	1.03	2.43	1.28
Solyc02g0	5.10	4.97	5.51	1.56	0.25	1.08	5.40	2.43
Solyc06g0	2.52	1.53	1.98	6.92	5.13	5.33	0.35	-1.53
Solyc10g0	0	0.03	0.04	0.74	0.62	0.58	0.04	-4.70
Solyc03g1	4.39	4.34	2.60	0.79	0.07	0.21	10.69	3.42
Solyc11g0	1.03	1.44	1.29	0.31	0.13	0.31	5.04	2.33
Solyc08g0	0	0	0	0.53	0.84	0.86	0.00	-12.86
Solyc10g0	256.92	238.64	252.69	63.89	24.73	24.33	6.62	2.73
Solyc06g0	1.41	1.47	1.31	0.57	0.63	0.82	2.07	1.05
Solyc10g0	0.54	0.75	0.33	0	0	0	5392.75	12.40
Solyc03g1	0.43	0.59	0.59	1.34	1.64	1.89	0.33	-1.59
Solyc06g0	4.93	3.62	4.39	10.82	11.76	10.57	0.39	-1.36
Solyc10g0	5.68	5.59	6.63	1.63	1.84	1.68	3.48	1.80
Solyc05g0	2.48	2.34	1.82	0.73	0.97	0.91	2.54	1.35
Solyc03g1	3.05	2.80	3.25	1.82	1.14	1.51	2.04	1.03
Solyc11g0	1.34	2.19	1.75	0.27	0	0.07	15.27	3.93
Solyc02g0	0.34	0.41	0.51	0	0	0	4235.90	12.05
Solyc08g0	1.17	1.28	1.46	3.27	3.47	3.16	0.39	-1.34
Solyc08g0	0.64	0.53	0.64	1.35	1.58	1.43	0.42	-1.27
Solyc01g0	28.92	0	0	0	0	0	96415.43	16.56
Solyc11g0	2.38	2.76	2.85	5.98	5.65	5.82	0.46	-1.13
Solyc11g0	0.69	0.76	0.95	2.32	2.57	2.03	0.35	-1.52
Solyc10g0	2.11	1.68	1.36	0	0.11	0	47.10	5.56
Solyc09g0	0	0.04	0.05	1.33	1.26	1.29	0.02	-5.38
Solyc08g0	0.35	0.44	0.35	0	0	0	3785.25	11.89
Solyc11g0	3.25	2.54	3.07	5.49	7.10	6.75	0.46	-1.12
Solyc06g0	1.73	2.10	1.65	0.56	0.41	0.41	3.98	1.99
Solyc01g0	1.21	0.97	0.86	0.34	0.31	0.35	3.07	1.62
Solyc11g0	2.28	2.91	2.16	4.60	4.46	5.67	0.50	-1.00
Solyc10g0	0	0	0	1.07	1.01	1.13	0.00	-13.39
Solyc09g0	0.67	0.48	0.39	0.03	0	0	51.95	5.70
Solyc03g0	1.63	1.86	1.89	3.27	3.88	3.74	0.49	-1.02
Solyc09g0	0.35	0.29	0.27	0	0	0	3042	11.57
Solyc09g0	1.12	0.12	0.04	6.54	10.50	6.36	0.05	-4.19
Solyc09g0	0	0	0	1.18	1.23	1.61	0.00	-13.71
Solyc02g0	2.17	2.05	1.89	0.62	0.96	1.14	2.24	1.17
Solyc08g0	0.34	0.48	0.39	0	0	0	4039.84	11.98
Solyc03g0	2.83	3.36	3.15	1.75	1.31	1.48	2.06	1.04
Solyc11g0	0	0	0	0.80	0.65	0.88	0.00	-12.92
Solyc10g0	5.48	4.71	5.23	2.41	2.02	2.91	2.10	1.07
Solyc10g0	19.43	19.83	19.53	12.50	6.89	7.33	2.20	1.14
Solyc01g1	0	0	0	0.33	0.23	0.27	0.00	-11.43
Solyc02g0	3.10	3.07	2.78	10.69	5.98	11.92	0.31	-1.68
Solyc11g0	0.59	0.67	0.62	1.44	2.54	1.82	0.32	-1.63
Solyc06g0	0	0	0	0	0	7.08	0.00	-14.53
Solyc10g0	0.29	0.16	0.14	0	0	0	1973.43	10.95
Solyc01g0	0.66	0.86	0.73	0.14	0.08	0.12	6.50	2.70
Solyc01g0	0	0	14.01	0	0	0	46703.99	15.51
Solyc02g0	0	0	0	20.60	0	0	0.00	-16.07
Solyc03g1	7.01	7.88	7.34	4.12	2.69	2.48	2.39	1.26

Solyc10g0	0	0	0	0	0	4.55	0.00	-13.89
Solyc09g0	15.74	16.48	16.38	3.38	0.92	1.38	8.56	3.10
Solyc06g0	0	0	0	0.11	0.26	0.20	0.00	-10.88
Solyc03g0	1.66	1.64	1.68	0.62	0.76	0.72	2.38	1.25
Solyc06g0	2.97	3.36	2.55	6.57	8.53	7.91	0.39	-1.37
Solyc11g0	0.64	0.58	0.87	0	0	0	6976.37	12.77
Solyc11g0	3.38	2.89	4.00	1.89	0.83	1.29	2.57	1.36
Solyc09g0	120.23	114.92	117.06	56.73	31.52	35.61	2.84	1.51
Solyc08g0	2.17	1.88	2.04	4.07	5.79	4.68	0.42	-1.25
Solyc12g0	0	0	0	0.93	2.03	1.61	0.00	-13.90
Solyc09g0	2.33	1.89	2.29	1.21	0.84	0.99	2.14	1.10
Solyc02g0	3.85	7.10	4.16	2.22	2.71	1.59	2.32	1.21
Solyc05g0	2.05	1.25	1.57	0.38	0.17	0.16	6.84	2.77
Solyc05g0	0	0	0	0	0	4.90	0.00	-14.00
Solyc03g0	1.83	2.36	2.10	0.96	1.21	0.90	2.05	1.04
Solyc10g0	1.13	0.66	1.18	0	0	0.07	43.03	5.43
Solyc11g0	0.57	0.19	0.38	0	0	0	3794.43	11.89
Solyc01g1	1.42	1.51	1.05	0.34	0.37	0.29	4.02	2.01
Solyc05g0	11.79	11.76	9.17	6.12	4.85	3.13	2.32	1.21
ENSRNA(	125.49	94.73	137.08	61.85	41.23	52.61	2.30	1.20
Solyc06g0	1.11	0.71	1.37	3.65	3.44	3.97	0.29	-1.80
Solyc04g0	2.95	3.26	2.45	7.25	7.12	6.64	0.41	-1.28
Solyc03g0	0.13	0.07	0	1.23	1.67	1.92	0.04	-4.63
Solyc02g0	0.49	0.62	0.45	1.63	1.26	1.55	0.35	-1.51
Solyc05g0	2.46	2.57	1.34	0	0	0	21240.81	14.37
Solyc01g0	0.91	1.11	0.97	0.09	0	0	34.25	5.10
Solyc08g0	1.85	1.49	1.80	0.56	0.21	0.38	4.49	2.17
Solyc07g0	0	0	0	0	1.51	0	0.00	-12.30
Solyc09g0	2.50	2.12	2.54	0.63	0.43	0.39	4.92	2.30
Solyc03g1	5.62	6.04	5.66	2.16	3.19	1.28	2.61	1.39
Solyc03g0	0.91	0.40	0.35	0	0	0	5511.05	12.43
Solyc08g0	0.58	0.50	0.38	0	0	0	4901.80	12.26
Solyc02g0	0.95	0.85	0.69	1.99	1.69	2.19	0.43	-1.23
Solyc10g0	0.79	0.76	0.96	5.49	5.07	5.41	0.16	-2.67
Solyc05g0	0.86	1.27	0.60	0.09	0.08	0.16	8.29	3.05
Solyc09g0	1.60	1.53	1.83	3.61	3.53	3.51	0.47	-1.10
Solyc02g0	6.11	4.44	4.63	10.39	12.62	14.36	0.41	-1.30
Solyc03g0	4.87	3.55	4.49	10.05	9.65	10.59	0.43	-1.23
Solyc09g0	0.04	0	0.06	0.90	0.93	1.13	0.03	-4.88
Solyc07g0	4.57	3.70	4.15	0.19	0	0.12	40.33	5.33
Solyc08g0	0.62	0.37	0.33	2.08	2.43	2.85	0.18	-2.47
Solyc11g0	1.07	1.02	1.12	0.29	0.37	0.49	2.78	1.48
Solyc12g0	0.87	0.78	0.69	0.23	0.33	0.20	3.10	1.63
Solyc01g0	0.97	0.94	0.82	0.34	0.43	0.36	2.42	1.27
Solyc10g0	15.31	12.88	13.95	3.75	5.34	2.14	3.75	1.91
Solyc12g0	0	0	0	0.30	0.15	0.29	0.00	-11.26
Solyc12g0	0.81	0.78	0.90	0.29	0.42	0.42	2.20	1.14
Solyc01g0	3.25	3.59	3.12	0	0.14	0.17	32.23	5.01
Solyc02g0	0.23	0.29	0.19	0	0	0	2381.72	11.22
Solyc04g0	5.14	4.44	4.65	2.37	2.40	2.06	2.09	1.06
Solyc06g0	1.78	1.13	1.07	0.45	0.38	0.47	3.04	1.60

Solyc03g0	0.36	0.36	0.33	0.05	0.11	0.12	3.84	1.94
Solyc05g0	1.70	1.60	1.59	0.68	0.48	0.49	2.96	1.57
Solyc03g1	0.92	0.80	0.76	1.78	1.81	1.64	0.48	-1.07
Solyc01g0	0.58	0.52	0.51	1.36	1.05	1.21	0.44	-1.17
Solyc04g0	2.85	2.86	2.10	1.12	1.10	1.11	2.35	1.23
Solyc05g0	3.85	3.75	3.37	2.13	1.58	1.45	2.13	1.09
Solyc02g0	1.06	1.58	1.57	0.61	0.31	0.32	3.41	1.77
Solyc03g0	0	0	0	0.23	0.31	0.49	0.00	-11.75
Solyc12g0	0	0	0	1.45	1.23	1.05	0.00	-13.60
Solyc07g0	2.17	1.73	1.73	7.05	5.85	4.94	0.32	-1.66
Solyc10g0	0.50	0.56	0.70	1.64	1.53	1.84	0.35	-1.51
Solyc11g0	1.11	0.89	1.42	0.34	0.29	0.36	3.46	1.79
Solyc02g0	2.43	2.27	2.16	3.78	6.03	6.60	0.42	-1.26
Solyc06g0	0.27	0.28	0.21	1.25	2.04	1.81	0.15	-2.74
Solyc05g0	0	0	0	1.27	2.15	2.14	0.00	-14.18
Solyc09g0	0	0	0	0.50	0.22	0.50	0.00	-11.98
Solyc09g0	5.09	4.84	5.42	1.15	2.49	1.60	2.93	1.55
Solyc06g0	1.52	1.51	1.56	0.64	0.42	0.80	2.46	1.30
Solyc10g0	0.02	0.02	0.03	0.26	0.30	0.36	0.08	-3.68
Solyc12g0	0.38	0.37	0.47	1.26	1.07	0.98	0.37	-1.44
Solyc05g0	7.74	7.42	7.14	5.29	2.72	2.80	2.06	1.05
Solyc06g0	0.81	0.77	0.77	0.16	0.17	0.23	4.17	2.06
Solyc07g0	1.79	1.89	1.92	5.36	3.79	4.36	0.41	-1.27
Solyc12g0	0.12	0.25	0.15	3.15	2.74	3.72	0.05	-4.20
Solyc12g0	2.11	1.49	1.93	0.80	1.06	0.86	2.04	1.03
Solyc08g0	2.50	2.73	2.30	10.57	9.12	11.55	0.24	-2.05
Solyc05g0	0	0	0	0.78	0.32	1.16	0.00	-12.88
Solyc02g0	1.83	1.81	1.53	0.28	0.34	0.37	5.17	2.37
Solyc01g0	5.27	4.92	4.14	0.76	1.87	0.94	4.00	2.00
Solyc06g0	0.14	0.06	0.07	0.73	0.84	1.01	0.11	-3.25
Solyc08g0	0.81	0.85	0.65	0.36	0.23	0.28	2.67	1.41
Solyc02g0	3.86	3.94	4.09	16.49	9.31	23.07	0.24	-2.04
Solyc09g0	1.29	0.89	1.21	0.26	0.28	0.29	4.06	2.02
Solyc08g0	0.42	0.49	0.28	1.03	1.09	1.28	0.35	-1.51
Solyc10g0	0	0.01	0	2.14	1.72	3.32	0.00	-9.03
Solyc02g0	1.20	1.16	1.37	3.37	3.91	3.55	0.34	-1.54
Solyc03g0	0.34	0.32	0.17	0	0	0	2761.02	11.43
Solyc10g0	15.55	13.55	17.95	34.60	32.72	29.83	0.48	-1.05
Solyc01g1	0	0	0.90	0	0	0	2993.63	11.55
Solyc09g0	3.36	4.42	3.73	7.35	8.89	8.38	0.47	-1.10
Solyc05g0	0.02	0	0.01	0.29	0.20	0.56	0.04	-4.82
Solyc02g0	7.15	6.47	5.84	2.89	3.41	3.22	2.04	1.03
Solyc02g0	0.57	0.58	0.46	0.11	0.06	0.17	4.67	2.22
Solyc06g0	0.27	0.26	0.15	0	0	0	2266.68	11.15
Solyc03g0	1.37	1.21	1.55	0.59	0.73	0.59	2.16	1.11
Solyc12g0	3.33	3.01	2.65	1.67	0.71	1.11	2.58	1.37
Solyc05g0	0	0	0	0.95	1.08	1.13	0.00	-13.36
Solyc09g0	15.90	13.22	16.69	8.92	6.76	5.77	2.13	1.09
ENSRNA(	727.39	567.47	772.61	394.05	262.02	346.98	2.06	1.04
Solyc11g0	0.35	0.21	0.38	0	0	0	3143.94	11.62
Solyc11g0	0.62	0.18	0.42	0	0	0	4049.02	11.98

Solyc07g0	1.98	1.97	2.22	0.82	0.52	0.20	4.00	2.00
Solyc01g0	1.42	1.87	1.87	4.64	4.39	3.59	0.41	-1.29
Solyc02g0	3.27	3.12	3.84	0.85	1.29	0.71	3.58	1.84
Solyc05g0	0	0	0	0.28	0.25	0.17	0.00	-11.21
Solyc01g1	32.65	34.83	33.00	2.15	0.22	0.32	37.32	5.22
Solyc04g0	1.33	1.16	1.38	3.48	3.34	2.70	0.41	-1.30
Solyc06g0	1.41	1.65	1.25	0.47	0.15	0.37	4.32	2.11
Solyc02g0	0.97	1.44	1.35	4.98	3.19	5.16	0.28	-1.83
Solyc02g0	1.51	1.81	1.84	0.86	0.26	0.33	3.57	1.83
Solyc04g0	0	0	0	1.65	1.29	1.30	0.00	-13.79
Solyc07g0	2.97	1.52	2.44	0.39	0.22	0.25	8.01	3.00
Solyc06g0	2.42	2.50	3.15	1.00	0.78	0.53	3.49	1.80
Solyc03g1	3.92	3.63	4.06	1.58	1.20	0.65	3.39	1.76
Solyc05g0	0	0.13	0.03	4.55	2.98	4.14	0.01	-6.23
Solyc01g1	1.05	1.03	1.23	0.30	0.26	0.42	3.40	1.77
Solyc02g0	2.12	2.11	1.70	0.82	0.80	0.97	2.29	1.19
Solyc12g0	6.08	7.18	5.56	2.97	2.61	2.25	2.41	1.27
Solyc05g0	0.33	0.32	0.45	0.03	0	0	31.58	4.98
Solyc03g0	0.16	0.45	0.12	2.14	2.49	1.86	0.11	-3.15
Solyc07g0	0.54	0.50	0.54	0.07	0	0	22.14	4.47
Solyc01g0	0.53	0.65	0.43	0.05	0	0	33.68	5.07
Solyc09g0	8.97	7.72	8.36	4.11	2.88	4.28	2.22	1.15
Solyc10g0	0	0	0	0.83	1.32	1.78	0.00	-13.68
Solyc04g0	1.50	0.72	0.92	3.31	4.18	3.17	0.29	-1.76
Solyc05g0	4.41	3.75	3.32	0.89	0.85	0.20	5.91	2.56
Solyc06g0	0.17	0.04	0	3.69	4.42	3.09	0.02	-5.77
Solyc08g0	0.45	0.56	0.43	0.95	1.37	1.46	0.38	-1.39
Solyc04g0	3.32	3.36	2.90	5.16	7.24	6.95	0.49	-1.01
Solyc06g0	0.04	0.17	0.06	0.61	0.58	0.69	0.15	-2.77
Solyc05g0	0	0.13	0	1.33	1.64	2.01	0.03	-5.29
Solyc04g0	1.29	1.03	1.01	0.54	0.43	0.24	2.74	1.46
Solyc01g0	0.85	1.23	0.92	0.13	0.32	0.21	4.54	2.18
Solyc03g0	0.23	0.23	0.24	0.69	0.53	0.73	0.36	-1.49
Solyc09g0	0.79	0.88	0.72	2.43	3.84	3.18	0.25	-1.98
Solyc10g0	0	0	0	1.10	1.45	1.54	0.00	-13.73
Solyc09g0	2.47	2.41	2.00	0.95	0.98	1.00	2.35	1.23
Solyc05g0	1.16	1.73	1.39	0.61	0.74	0.70	2.08	1.06
Solyc12g0	0.88	1.20	0.87	0.54	0.27	0.28	2.73	1.45
Solyc11g0	0.49	0.63	0.43	1.47	1.40	1.27	0.38	-1.41
Solyc06g0	1.44	1.36	1.28	5.28	3.82	5.57	0.28	-1.85
Solyc12g1	1.05	1.63	0.96	0.46	0.50	0.62	2.30	1.20
Solyc01g0	13.99	13.43	14.36	9.17	4.53	5.55	2.17	1.12
Solyc03g1	0.89	1.70	0.74	0.21	0	0.04	13.81	3.79
Solyc04g0	1.41	0.81	0.66	0	0	0	9579.06	13.23
Solyc11g0	0.25	0.27	0.09	1.05	1.12	0.82	0.20	-2.30
Solyc02g0	5.06	6.35	6.47	14.66	9.99	13.65	0.47	-1.10
Solyc05g0	6.55	5.14	4.33	1.99	2.45	2.06	2.46	1.30
Solyc07g0	0	0	0	0.21	0.36	0.27	0.00	-11.47
Solyc08g0	2.83	3.07	2.90	6.22	4.55	7.39	0.48	-1.04
Solyc10g0	0.96	1.19	0.79	3.10	3.19	2.57	0.33	-1.59
Solyc08g0	0	0	0.06	1.68	1.12	0.93	0.02	-6.06

Solyc12g0	0.78	0.65	0.60	0.23	0.21	0.22	3.08	1.62
Solyc09g0	10.09	8.99	8.54	15.28	23.75	17.62	0.49	-1.04
Solyc11g0	0.59	0.77	0.82	0.21	0.17	0.13	4.22	2.08
Solyc08g0	2.32	2.56	2.31	1.06	1.45	0.92	2.10	1.07
Solyc02g0	1.89	2.34	1.83	0.74	1.19	1.09	2.01	1.01
Solyc04g0	1.24	0.87	1.20	0.51	0.36	0.30	2.84	1.50
Solyc04g0	1.04	1.20	1.06	2.40	2.86	2.86	0.41	-1.30
Solyc11g0	2.72	2.63	1.99	1.14	0.69	1.19	2.43	1.28
Solyc04g0	0.80	0.68	0.75	1.72	2.23	1.51	0.41	-1.29
Solyc02g0	0.21	0.18	0.22	0.55	0.86	1.03	0.25	-1.99
Solyc01g0	1.63	1.44	1.89	2.83	4.11	3.38	0.48	-1.06
Solyc01g0	7.70	9.03	7.40	16.78	18.21	17.41	0.46	-1.12
Solyc06g0	0.40	0.45	0.37	0.86	0.76	1.00	0.46	-1.11
Solyc03g0	0.28	0.36	0.25	0.71	0.92	0.94	0.34	-1.54
Solyc07g0	2.41	2.77	2.08	1.18	1.14	0.75	2.37	1.24
Solyc09g0	1.21	1.25	1.01	0.32	0.17	0.48	3.57	1.84
Solyc08g0	2.52	1.81	2.19	6.40	6.76	4.58	0.37	-1.44
Solyc05g0	0.32	0.09	0.52	0	0	0	3068.36	11.58
Solyc03g0	0.11	0.38	0	11.16	6.19	7.29	0.02	-5.67
Solyc10g0	0.16	0.22	0.10	0	0	0	1604.71	10.65
Solyc07g0	0.54	0.56	0.51	0.05	0.18	0.10	4.69	2.23
Solyc03g0	0.13	0.16	0.17	0	0	0	1566.99	10.61
Solyc12g0	0.41	0.38	0.64	0.03	0	0	44.33	5.47
Solyc10g0	0.11	0.10	0.11	0	0	0	1081.58	10.08
Solyc09g0	0.23	0.18	0.20	0.96	0.75	0.68	0.26	-1.97
Solyc10g0	0.76	0.64	0.56	1.69	1.71	1.78	0.38	-1.41
Solyc01g0	0.77	0.75	0.54	0.25	0.25	0.14	3.21	1.68
Solyc11g0	2.96	3.16	3.07	0.74	0.20	0.21	7.96	2.99
Solyc06g0	0.20	0.21	0.21	0	0	0	2059.41	11.01
Solyc12g0	0.89	0.83	0.88	0.16	0.30	0.39	3.03	1.60
Solyc12g0	0.49	0.30	0.49	0	0	0	4279.43	12.06
Solyc05g0	1.68	2.11	1.53	1.06	0.66	0.60	2.29	1.20
Solyc07g0	0.14	0.14	0.16	0	0	0	1462.99	10.51
Solyc02g0	0.18	0.17	0.25	0.73	0.85	0.67	0.27	-1.89
Solyc04g0	0.47	0.47	0.62	1.06	1.18	1.58	0.41	-1.29
Solyc10g0	0.78	0.56	0.80	0.33	0.32	0.30	2.26	1.18
Solyc02g0	4.73	4.32	3.01	12.21	11.43	16.54	0.30	-1.74
Solyc07g0	0.14	0.51	0.31	0	0	0	3182.84	11.64
Solyc05g0	0.09	0.36	0.20	0	0	0	2143.40	11.07
Solyc02g0	3.79	3.62	3.77	8.21	9.01	8.65	0.43	-1.21
Solyc01g1	1.12	0.90	0.76	0	0	0	9262.43	13.18
Solyc03g1	0.56	0.38	0.45	1.23	1.09	1.12	0.41	-1.30
Solyc01g0	1.89	1.80	1.89	0.91	0.88	0.59	2.35	1.24
Solyc09g0	1.35	0.99	1.09	1.88	2.72	2.46	0.49	-1.04
Solyc02g0	8.31	6.39	6.15	4.01	2.99	3.08	2.07	1.05
Solyc07g0	0.23	0.22	0.21	0.04	0.01	0.02	8.50	3.09
Solyc09g0	12.32	11.77	11.56	2.86	0.75	0.79	8.11	3.02
Solyc06g0	1.32	1.01	1.18	0.53	0.41	0.61	2.25	1.17
Solyc01g1	1.10	1.25	1.15	2.02	3.07	2.66	0.45	-1.15
Solyc08g0	0.30	0.46	0.37	0	0	0	3738.24	11.87
Solyc09g0	0	0	0	1.50	1.48	1.41	0.00	-13.84

Solyc06g0	0.27	0.56	0.71	0	0	0	5133.09	12.33
Solyc08g0	0	0.09	0	1.61	0.91	1.48	0.02	-5.43
Solyc10g0	0	0	0	0.51	0.44	0.35	0.00	-12.09
Solyc10g0	0.43	0.25	0.20	0	0	0	2907.35	11.51
Solyc12g0	0.72	0.76	0.68	0.30	0.29	0.22	2.66	1.41
Solyc05g0	0.36	0.39	0.16	0	0	0	3037.21	11.57
Solyc02g0	0.68	0.54	0.63	1.37	1.71	1.56	0.40	-1.32
Solyc03g0	0.40	0.23	0.41	1.14	0.98	1.22	0.31	-1.69
Solyc03g0	0.82	0.84	0.89	0.43	0.36	0.47	2.02	1.02
Solyc04g0	253.20	250.36	264.52	103.92	47.22	46.51	3.89	1.96
Solyc02g0	1.16	1.55	1.34	0.44	0.77	0.57	2.28	1.19
Solyc11g0	0.20	0.26	0.21	0.63	0.70	0.84	0.31	-1.70
Solyc06g0	1.82	1.95	2.00	1.12	0.97	0.78	2.01	1.01
Solyc01g0	0.17	0.17	0.26	0	0	0	1991.27	10.96
Solyc10g0	0	0	0	0.41	0.41	0.50	0.00	-12.10
Solyc01g1	1.11	0.59	1.03	0.21	0.20	0.21	4.37	2.13
Solyc09g0	0	0	0	0.48	0.90	1.34	0.00	-13.14
Solyc04g0	0.03	0	0.07	0.43	0.52	0.45	0.07	-3.79
Solyc02g0	2.35	2.65	2.88	4.37	7.03	4.94	0.48	-1.05
Solyc06g0	1.00	1.20	1.15	2.41	3.22	3.03	0.39	-1.37
Solyc10g0	1.15	1.07	0.97	0.60	0.40	0.36	2.33	1.22
Solyc05g0	1.27	0.90	1.17	0.48	0.09	0.25	4.12	2.04
Solyc03g0	1.14	1.54	1.22	0.31	0.14	0.48	4.18	2.06
Solyc10g0	33.80	33.33	31.47	4.33	0.72	0.53	17.65	4.14
Solyc01g0	0.60	0.79	0.74	0.30	0.24	0.27	2.62	1.39
Solyc03g0	0.68	0.82	0.69	0.14	0.20	0.36	3.13	1.65
Solyc08g0	6.97	6.34	5.94	13.08	13.96	14.21	0.47	-1.10
Solyc09g0	0.59	0.26	0.41	0.15	0.06	0.07	4.54	2.18
Solyc11g0	0.62	0.83	0.59	0	0.04	0	57.14	5.84
Solyc01g0	9.09	7.58	7.63	5.54	3.54	2.94	2.02	1.02
Solyc10g0	0.14	0.26	0.28	0	0	0	2271.73	11.15
Solyc01g0	0.52	0.44	0.55	0.18	0.07	0.17	3.53	1.82
Solyc02g0	0.62	0.39	0.59	2.44	1.75	2.34	0.25	-2.02
Solyc08g0	0.58	0.59	0.74	0.29	0.31	0.20	2.38	1.25
Solyc06g0	0.56	0.27	0.58	0.05	0	0.05	14.49	3.86
Solyc05g0	0.98	0.78	1.20	0.32	0.14	0.09	5.37	2.42
Solyc03g0	0.40	0.63	0.43	1.70	1.81	1.98	0.27	-1.91
Solyc05g0	0.30	0.24	0.33	0.02	0	0	39.90	5.32
Solyc07g0	6.69	6.33	7.38	2.77	1.28	1.02	4.02	2.01
Solyc11g0	0	0	0	1.34	1.56	1.19	0.00	-13.74
Solyc01g1	0.30	0.31	0.24	0	0	0	2829.11	11.47
Solyc01g0	0.78	0.91	0.83	1.70	2.69	1.93	0.40	-1.32
Solyc02g0	0	0	0	0.33	0.25	0.18	0.00	-11.29
Solyc01g0	0.35	0.34	0.24	0.69	0.76	0.85	0.41	-1.30
Solyc02g0	3.97	3.55	3.59	7.19	8.79	7.09	0.48	-1.05
Solyc12g0	0.27	0.22	0.21	0.89	0.79	0.88	0.27	-1.89
Solyc02g0	0.54	0.79	1.07	0.24	0.13	0.04	5.76	2.53
Solyc06g0	0.45	0.89	0.65	2.60	2.31	2.06	0.29	-1.80
Solyc12g0	0.99	1.53	1.37	0.54	0.48	0.68	2.29	1.20
Solyc10g0	0	0	0	0.96	0.63	0.48	0.00	-12.76
Solyc04g0	1.05	1.38	1.39	2.73	3.13	2.73	0.45	-1.16

Solyc03g0	0.15	0.35	0.19	0	0	0	2300.48	11.17
Solyc03g0	1.70	1.78	1.32	0.34	0.51	0.56	3.43	1.78
Solyc12g0	0.78	1.15	0.94	2.68	3.02	3.09	0.33	-1.61
Solyc06g0	0.22	0.23	0.21	0	0	0	2169.21	11.08
ENSRNA0	675.76	477.92	663.46	343.18	199.95	249.43	2.29	1.20
Solyc12g0	0.46	0.45	0.63	0.19	0.14	0.17	3.03	1.60
Solyc06g0	1.48	1.25	1.42	0.34	0.70	0.23	3.27	1.71
Solyc07g0	9.19	9.30	8.79	4.85	3.40	3.20	2.38	1.25
Solyc06g0	0.12	0.13	0.29	0.76	0.71	0.91	0.23	-2.13
Solyc08g0	2.21	1.39	2.11	0.97	0.79	0.48	2.54	1.34
Solyc09g0	0.05	0.07	0.06	0	0	0	617.25	9.27
Solyc12g0	0	0	0	0.82	0.44	0.49	0.00	-12.51
Solyc02g0	3.78	3.36	4.24	0.98	0.91	0.64	4.49	2.17
Solyc07g0	1.22	0.86	1.31	0.49	0.47	0.50	2.33	1.22
Solyc10g0	0.33	0.28	0.29	0.04	0.03	0.03	8.99	3.17
Solyc01g0	3.22	3.10	2.86	6.30	5.84	6.22	0.50	-1.00
Solyc06g0	10.81	8.69	7.22	4.55	2.16	1.33	3.33	1.73
Solyc02g0	0.14	0	0.11	3.07	2.61	2.24	0.03	-4.99
Solyc11g0	1.76	2.62	1.71	0.84	1.25	0.63	2.24	1.16
Solyc05g0	1.62	1.64	1.56	0.70	0.53	0.68	2.52	1.33
Solyc08g0	0.23	0.15	0.37	0	0	0	2486.58	11.28
Solyc11g0	0	0	0	0.26	0.39	0.30	0.00	-11.62
Solyc04g0	0.27	0.17	0.07	0	0	0	1711.14	10.74
Solyc04g0	0.61	0.67	0.57	0.18	0.16	0.17	3.70	1.89
Solyc03g0	0.57	0.19	0.15	3.80	1.22	2.88	0.12	-3.11
Solyc09g0	0	0	0	0.38	0.58	0.25	0.00	-11.97
Solyc01g1	0.16	0.07	0.11	0.56	0.59	0.60	0.20	-2.35
Solyc03g1	0.29	0.39	0.37	0.59	0.79	0.83	0.47	-1.08
Solyc11g0	13.79	15.07	11.79	25.39	28.68	30.28	0.48	-1.05
Solyc11g0	0	0	0	0.15	0.17	0.23	0.00	-10.83
Solyc03g0	1.78	1.74	0.94	3.46	3.44	3.85	0.42	-1.27
Solyc03g0	2.14	2.21	3.34	1.30	0.83	0.71	2.71	1.44
Solyc11g0	0	0	0	0.98	1.26	1.03	0.00	-13.41
Solyc09g0	1.55	0.80	1.03	0.43	0.50	0.49	2.37	1.25
Solyc03g1	0.39	0.47	0.42	0.16	0.08	0.03	4.71	2.24
Solyc02g0	2.72	2.29	2.19	1.28	0.80	1.34	2.11	1.08
Solyc02g0	0.26	0.38	0.40	0.04	0	0	25.47	4.67
Solyc09g0	0	0	0	0.60	0.33	0.58	0.00	-12.30
Solyc07g0	0.57	0.32	0.36	0.04	0.02	0.14	6.31	2.66
Solyc09g0	0.80	1.11	1.12	2.78	1.87	3.79	0.36	-1.48
Solyc06g0	0.26	0	0	2.19	1.79	1.87	0.05	-4.46
Solyc11g0	0	0	0	1.00	1.13	0.46	0.00	-13.08
Solyc07g0	1.60	1.25	0.86	0	0	0	12376	13.60
Solyc12g0	1.12	0.86	0.87	0.34	0.30	0.32	2.97	1.57
Solyc07g0	0.88	0.92	1.04	0.31	0.51	0.43	2.26	1.18
Solyc04g0	0.27	0.38	0.36	0.06	0	0	17.20	4.10
Solyc07g0	0.16	0	0	0.98	1.81	1.28	0.04	-4.69
Solyc11g0	13.01	13.54	12.91	0.19	0	0	210.82	7.72
Solyc05g0	2.77	3.58	2.41	1.70	1.24	1.13	2.15	1.10
Solyc09g0	0	0	0	1.18	1.60	1.03	0.00	-13.63
Solyc05g0	8.02	7.37	6.08	3.84	1.58	2.34	2.76	1.47

Solyc04g0	0.25	0.26	0.36	0.06	0.04	0	8.84	3.14
Solyc07g0	22.63	24.51	22.80	60.89	39.42	42.37	0.49	-1.03
Solyc07g0	1.63	2.53	2.11	3.77	4.39	4.46	0.50	-1.01
Solyc10g0	1.77	1.97	1.56	4.80	4.51	3.86	0.40	-1.31
Solyc06g0	0.29	0.27	0.34	0.63	0.66	0.84	0.42	-1.25
Solyc12g0	0	0	0	0.18	0.15	0.26	0.00	-10.94
Solyc07g0	0.72	1.08	0.51	0.17	0.05	0.07	8.04	3.01
Solyc08g0	0	0	0.07	0.86	0.51	1.17	0.03	-5.23
Solyc12g0	2.11	1.20	1.25	3.60	3.51	3.97	0.41	-1.28
Solyc01g0	0.53	0.58	0.66	0.24	0.06	0.19	3.64	1.87
Solyc02g0	0.13	0.12	0.26	1.20	0.68	1.13	0.17	-2.56
Solyc02g0	0.63	0.63	0.81	0.23	0.22	0.33	2.66	1.41
Solyc08g0	1.97	1.71	1.31	0.65	0.69	0.61	2.56	1.36
Solyc04g0	1.83	2.09	2.45	0.27	0.61	0.84	3.72	1.90
Solyc03g0	0.21	0.25	0.12	0.02	0	0	23.80	4.57
Solyc06g0	0.43	0.45	0.69	1.03	1.40	1.26	0.43	-1.23
Solyc09g0	0.74	0.35	0.55	0.09	0	0.11	8.04	3.01
Solyc04g0	0.28	0.28	0.37	0.06	0	0	15.77	3.98
Solyc04g0	0.38	0.30	0.44	2.89	2.02	2.25	0.16	-2.67
Solyc03g1	0.03	0.03	0	0.24	0.30	0.49	0.06	-4.16
Solyc12g0	0	0.47	0.35	3.04	3.96	3.91	0.08	-3.72
Solyc02g0	0.05	0.23	0.17	1.04	0.67	0.55	0.20	-2.33
Solyc03g0	0	0	0	1.28	1.38	1.51	0.00	-13.76
Solyc10g0	0	0.04	0	0.49	0.39	0.70	0.03	-5.30
Solyc03g0	1.16	1.34	0.92	0.44	0.23	0.19	3.99	2.00
Solyc06g0	0.16	0.20	0.22	0	0	0	1942.11	10.92
Solyc12g0	3.46	2.25	2.64	1.35	0.67	1.01	2.76	1.47
Solyc04g0	0.52	0.38	0.42	0.92	0.97	0.97	0.46	-1.11
Solyc03g0	0	0	0	0.51	0.52	0.71	0.00	-12.50
Solyc06g0	0	0	0	0.04	0.10	0.08	0.00	-9.49
Solyc12g0	0	0.09	0	1.37	0.74	1.79	0.02	-5.42
Solyc05g0	0.03	0.09	0.14	0.36	0.52	0.58	0.18	-2.44
Solyc10g0	0.37	0.38	0.34	0.16	0.13	0.20	2.19	1.13
Solyc06g0	0	0	0	0.20	0.21	0.18	0.00	-10.94
Solyc09g0	0.12	0.05	0.23	0	0	0	1363.92	10.41
Solyc05g0	8.50	8.16	7.74	0	0.24	0	99.78	6.64
Solyc12g0	0.11	0.16	0.49	0	0	0	2529.18	11.30
Solyc06g0	0	0	0	0.11	0.12	0.13	0.00	-10.22
Solyc08g0	0.72	0.68	0.56	1.26	1.46	1.22	0.50	-1.00
Solyc08g0	0.45	0.37	0.42	1.70	1.03	1.59	0.29	-1.80
Solyc10g0	3.03	3.18	2.12	1.56	1.31	1.16	2.07	1.05
Solyc08g0	2.78	2.73	3.29	1.58	0.91	1.67	2.11	1.08
Solyc06g0	0.43	0.61	0.94	0	0.03	0	72.41	6.18
Solyc09g0	0	0	0	0.30	0.07	0.43	0.00	-11.38
Solyc08g0	1.28	0.91	0.88	0.35	0.43	0.48	2.43	1.28
Solyc08g0	2.19	1.31	1.96	0.76	0.84	1.09	2.02	1.02
Solyc10g0	0.37	0.81	0.51	0	0	0	5617.07	12.46
Solyc09g0	0	0.11	0.15	1.32	0.82	1.80	0.07	-3.94
Solyc11g0	2.07	1.37	2.14	0.84	0.90	0.49	2.51	1.33
Solyc12g0	2.13	1.23	1.42	4.77	4.87	3.81	0.36	-1.49
Solyc04g0	1.17	1.10	1.14	4.10	3.24	2.90	0.33	-1.58

Solyc07g0	0.39	0.22	0.40	0	0	0.03	29.42	4.88
Solyc06g0	1.01	0.92	1.64	0.19	0	0	18.67	4.22
Solyc08g0	1.44	1.25	1.48	2.74	2.60	3.47	0.47	-1.08
Solyc09g0	0.71	1.03	1.25	3.78	4.21	3.06	0.27	-1.89
Solyc02g0	0.22	0.22	0.24	0.06	0.08	0.06	3.38	1.76
Solyc02g0	0.51	0.46	0.31	1.49	2.60	1.04	0.25	-2.00
Solyc04g0	0	0	0	0.40	0.29	0.50	0.00	-11.95
Solyc02g0	0.26	0.32	0.18	0.68	0.57	0.64	0.40	-1.31
Solyc10g0	0.40	0.59	0.41	0.07	0.19	0.07	4.22	2.08
Solyc01g1	0.71	0.17	0.42	1.46	2.03	1.96	0.24	-2.07
Solyc09g0	0.09	0.06	0.08	0	0	0	767.92	9.58
Solyc04g0	0.36	0.45	0.31	0.68	0.97	1.22	0.39	-1.37
Solyc06g0	0.20	0.18	0.41	2.33	2.51	2.63	0.11	-3.23
Solyc09g0	0.20	0.20	0.40	0	0	0	2647.84	11.37
Solyc03g0	0.87	0.74	0.96	2.61	2.70	2.30	0.34	-1.57
Solyc07g0	0.41	0.61	0.64	0	0	0	5532.77	12.43
Solyc06g0	1.43	1.08	2.24	5.13	5.52	5.91	0.29	-1.80
Solyc07g0	2.41	2.84	3.77	0.46	1.59	0.49	3.55	1.83
Solyc03g1	0.46	0.16	0.37	0.02	0	0	57.07	5.83
Solyc04g0	0.46	0.28	0.15	1.97	1.56	2.08	0.16	-2.66
Solyc04g0	0.13	0.12	0.11	0	0	0	1191.32	10.22
Solyc08g0	0.22	0.15	0.11	0	0.02	0	21.56	4.43
Solyc01g0	1.30	2.05	1.38	2.98	3.80	3.89	0.44	-1.18
Solyc03g1	16.98	17.39	16.97	0.78	0	0.26	49.39	5.63
Solyc08g0	0	0	0	0.08	0.09	0.14	0.00	-10.00
Solyc01g0	0.09	0.15	0.15	0	0	0	1296.75	10.34
Solyc02g0	0.27	0.48	0.11	0	0	0	2831.77	11.47
Solyc01g1	1.33	1.73	1.29	3.30	3.15	2.73	0.47	-1.08
Solyc02g0	0.29	0.28	0.34	0	0	0	3056.62	11.58
Solyc01g1	0.72	0.87	0.84	2.08	1.24	2.36	0.43	-1.22
Solyc03g0	0.07	0.19	0.14	0	0	0	1336.08	10.38
Solyc09g0	0.46	0.10	0.25	0	0	0	2685.31	11.39
Solyc03g0	6.39	4.23	3.78	11.91	11.17	16.21	0.37	-1.45
Solyc02g0	0.80	0.51	0.95	2.35	2.14	3.38	0.29	-1.80
Solyc01g0	0.08	0	0	1.39	1.36	1.64	0.02	-5.72
Solyc06g0	4.07	3.81	3.43	2.22	2.21	1.00	2.08	1.06
Solyc05g0	0.09	0.17	0.20	0	0.02	0	21.99	4.46
Solyc12g0	0.04	0.40	0.11	0	0	0	1818.26	10.83
Solyc10g0	2.39	2.75	2.91	4.65	6.60	4.95	0.50	-1.01
Solyc10g0	1.55	2.06	1.67	0.96	0.73	0.91	2.03	1.02
Solyc10g0	0.45	0.52	0.44	0.20	0.18	0.29	2.14	1.09
Solyc12g0	1.34	1.07	1.07	2.23	2.86	2.45	0.46	-1.11
Solyc09g0	0.39	0.46	0.55	0.23	0.21	0.19	2.22	1.15
Solyc08g0	0.07	0.15	0.09	0	0	0	1033.20	10.01
Solyc04g0	0.58	0.47	0.67	0	0	0	5759.74	12.49
Solyc11g0	0.57	0.41	0.35	0	0	0	4436.07	12.12
Solyc03g0	0.53	0.76	0.70	1.54	1.89	1.46	0.41	-1.30
Solyc09g0	0.35	0.39	0.40	0.11	0.08	0.05	4.69	2.23
Solyc03g1	0.12	0.35	0.36	0	0	0	2773.71	11.44
Solyc06g0	1.32	1.34	1.06	0.33	0.30	0.62	2.99	1.58
Solyc12g0	0.34	0.24	0.18	0.08	0.02	0.06	4.85	2.28

Solyc01g1	0.07	0.12	0.13	0	0	0	1067.51	10.06
Solyc08g0	0	0	0	0.66	0.35	0.16	0.00	-11.92
Solyc01g0	0.11	0.07	0.02	0.32	0.34	0.39	0.19	-2.40
Solyc08g0	2.67	2.22	2.88	0.75	1.52	1.14	2.28	1.19
Solyc02g0	2.41	2.14	2.11	4.53	5.31	4.66	0.46	-1.12
Solyc05g0	0	0.16	0	2.11	2.23	0.88	0.03	-5.01
Solyc03g1	19.13	17.69	17.05	5.99	1.48	1.68	5.89	2.56
Solyc07g0	2.40	1.67	3.23	1.00	0.87	0.76	2.77	1.47
Solyc05g0	0.14	0	0.14	2.25	1.34	1.78	0.05	-4.24
Solyc06g0	3.08	2.02	2.16	5.87	7.64	6.11	0.37	-1.43
Solyc05g0	1.05	1.50	1.22	0.34	0.41	0.30	3.62	1.86
Solyc04g0	8.67	12.24	9.59	29.57	16.64	18.25	0.47	-1.08
Solyc05g0	3.26	2.36	3.14	1.43	0.70	1.37	2.50	1.32
Solyc03g1	3.27	2.30	3.05	1.16	1.66	1.38	2.05	1.04
Solyc04g0	0.35	0.39	0.27	0	0.05	0	19.54	4.29
Solyc07g0	1.93	2.39	2.35	0.83	1.52	0.94	2.02	1.02
Solyc07g0	1.15	0.91	0.73	0.42	0.47	0.45	2.07	1.05
Solyc01g0	45.16	43.14	51.29	27.26	12.33	14.76	2.57	1.36
Solyc03g0	0.36	0.50	0.23	0	0	0.05	20.56	4.36
Solyc06g0	1.68	1.43	1.64	0.81	0.61	0.81	2.13	1.09
Solyc02g0	0.04	0	0.03	0.36	0.29	0.37	0.07	-3.82
Solyc02g0	0.39	0.25	0.59	0.17	0.16	0.13	2.66	1.41
Solyc05g0	0.96	0.66	0.50	0.23	0.18	0.10	4.22	2.08
Solyc09g0	1.67	1.33	1.41	3.51	3.43	3.20	0.44	-1.20
Solyc08g0	0.14	0.06	0.16	0	0	0	1235.43	10.27
Solyc12g0	0.05	0	0	0.92	0.69	0.57	0.02	-5.43
Solyc02g0	0.91	0.52	0.88	0.43	0.27	0.29	2.33	1.22
Solyc08g0	1.19	1.58	1.74	3.62	3.29	2.93	0.46	-1.13
Solyc09g0	0.29	0.38	0.14	0.03	0	0	25.41	4.67
Solyc10g0	0.08	0.06	0.06	0.27	0.26	0.19	0.27	-1.87
Solyc10g0	0.17	0.03	0.09	0	0	0	950.12	9.89
Solyc08g0	0.55	0.20	0.27	0.08	0.03	0	8.90	3.15
Solyc12g1	0	0	0	0.09	0.11	0.13	0.00	-10.11
Solyc05g0	1.03	1.32	1.02	0.62	0.40	0.23	2.69	1.43
Solyc07g0	1.46	0.91	1.98	0.15	0.04	0.11	14.48	3.86
Solyc11g0	0.09	0.07	0.14	0	0	0	1008.81	9.98
Solyc02g0	0.04	0.11	0.05	0.25	0.31	0.38	0.22	-2.21
Solyc07g0	0.05	0.12	0.18	0	0	0	1180.56	10.21
Solyc06g0	0.78	0.67	0.62	0.27	0.20	0.25	2.85	1.51
Solyc03g1	0.07	0	0	0.89	0.63	1.69	0.02	-5.50
Solyc02g0	0.26	0.24	0.31	0.09	0.13	0.09	2.60	1.38
Solyc10g0	1.84	1.50	1.36	3.05	3.89	2.73	0.49	-1.04
Solyc12g0	1.15	0.68	1.01	0.51	0.20	0.18	3.17	1.66
Solyc12g0	2.52	3.05	2.68	4.70	7.39	5.74	0.46	-1.11
Solyc11g0	0.08	0.23	0.18	0.75	1.39	1.63	0.13	-2.96
Solyc10g0	0.26	0.31	0.29	0.79	0.97	0.56	0.37	-1.43
Solyc08g0	0.05	0.08	0.10	0.32	0.33	0.23	0.27	-1.90
Solyc12g0	1.76	0.84	1.34	0.58	0.44	0.48	2.62	1.39
Solyc06g0	0	0	0	0.43	0.46	0.37	0.00	-12.03
Solyc10g0	0.33	0.44	0.37	0.06	0.06	0.04	7.48	2.90
Solyc04g0	1.06	1.51	1.90	0.60	0.27	0.60	3.05	1.61

Solyc05g0	0.37	0.26	0.27	0	0	0	2989.49	11.55
Solyc06g0	3.70	4.59	4.56	1.64	2.88	1.81	2.03	1.02
Solyc01g0	0.97	0.59	0.80	0.26	0.12	0.19	4.12	2.04
Solyc01g0	4.41	4.75	5.03	1.81	2.68	1.77	2.27	1.18
Solyc09g0	1.28	1.39	1.22	2.35	2.95	2.94	0.47	-1.09
Solyc02g0	0.63	0.69	0.65	1.37	2.15	1.56	0.39	-1.37
Solyc07g0	0.23	0.18	0.15	0	0	0	1880.17	10.88
Solyc02g0	0.30	0.23	0.28	0.06	0.04	0	7.75	2.95
Solyc09g0	0	0	0	0.40	0.44	0.27	0.00	-11.85
Solyc12g0	6.20	6.21	4.11	11.06	13.13	13.37	0.44	-1.18
Solyc10g0	0	0	0	0.05	0.15	0.17	0.00	-10.24
Solyc09g0	1.51	1.61	1.87	0.64	0.78	0.47	2.65	1.40
Solyc11g0	1.22	0.82	1.52	0	0	0	11861.59	13.53
Solyc03g0	3.38	2.85	2.45	4.62	7.49	5.67	0.49	-1.03
Solyc04g0	0.12	0.39	0.10	0	0	0	2071.20	11.02
Solyc03g0	0	0	0	0.40	0.16	0.09	0.00	-11.07
Solyc12g0	0	0	0	0.94	2.02	1.33	0.00	-13.80
Solyc07g0	6.00	5.11	5.93	3.28	2.35	1.20	2.50	1.32
ENSRNA(	11.26	8.80	9.71	21.93	23.98	24.24	0.42	-1.24
Solyc01g0	0.17	0.31	0.12	0.02	0.02	0.04	6.48	2.70
Solyc06g0	0.38	0.51	0.40	0.97	1.17	1.35	0.37	-1.44
Solyc11g0	13.94	17.00	17.30	0.54	0	0	89.82	6.49
Solyc07g0	0.87	1.20	1.24	0.25	0.39	0.58	2.71	1.44
Solyc09g0	5.27	5.71	5.46	0.28	0	0.03	54.33	5.76
Solyc11g0	0.44	0.47	0.45	0.20	0.16	0.10	2.98	1.58
Solyc08g0	0.94	0.82	1.11	0.57	0.24	0.25	2.73	1.45
Solyc03g1	0.10	0.15	0.14	0	0	0.03	14.48	3.86
Solyc01g0	0.38	0.62	0.73	0.15	0	0.05	8.77	3.13
Solyc02g0	0.59	0.92	0.61	0.20	0	0.06	8.19	3.03
Solyc07g0	0	0	0	0.34	0.22	0.25	0.00	-11.40
Solyc10g0	0.05	0.15	0.28	0	0	0	1611.78	10.65
Solyc05g0	0.11	0.14	0.06	0	0	0	1020.85	10.00
Solyc02g0	0.64	0.54	0.71	1.27	1.12	1.73	0.46	-1.13
Solyc08g0	0.35	0.42	0.55	0.13	0.06	0.13	4.16	2.06
Solyc01g0	0.27	0.28	0.40	1.93	1.65	0.94	0.21	-2.26
Solyc04g0	0.07	0.05	0.17	0.60	0.60	0.75	0.15	-2.72
Solyc08g0	0.35	0.28	0.29	0.78	0.67	0.73	0.42	-1.25
Solyc02g0	0.73	0.89	0.87	0.31	0.47	0.30	2.29	1.20
Solyc03g0	0.50	0.33	0.43	1.19	1.38	0.80	0.37	-1.42
Solyc12g0	0.29	0.23	0.22	0.12	0.14	0.09	2.17	1.12
Solyc05g0	0.05	0.04	0	0.26	0.40	0.41	0.09	-3.43
Solyc12g0	4.31	3.80	3.81	8.26	8.15	9.78	0.45	-1.14
Solyc07g0	4.39	3.57	4.21	2.50	1.15	1.29	2.47	1.30
Solyc01g0	0.28	0.25	0.24	0.11	0.03	0.07	3.59	1.84
Solyc03g0	0.06	0.05	0.12	0	0	0	794.30	9.63
Solyc09g0	0.14	0.23	0.27	1.40	1.12	1.27	0.17	-2.56
Solyc04g0	0.20	0.18	0.19	0.40	0.38	0.50	0.44	-1.18
Solyc04g0	0.40	0.49	0.48	0.09	0	0.13	6.17	2.63
Solyc12g1	0.03	0.01	0.04	0	0	0	249.94	7.97
Solyc09g0	1.51	1.70	1.48	0.71	0.76	0.55	2.32	1.21
Solyc03g0	2.48	2.78	3.43	1.81	0.94	0.59	2.60	1.38

Solyc01g0	0.17	0.12	0.11	0.01	0	0	36.75	5.20
Solyc04g0	0.15	0.20	0.12	0	0	0	1564.01	10.61
Solyc07g0	1.85	1.13	1.87	0.60	0.20	0.41	3.98	1.99
Solyc04g0	0.08	0.07	0.12	0	0	0	900.23	9.81
Solyc11g0	0	0	0.06	0.48	0.50	1.05	0.03	-5.07
Solyc04g0	0.04	0.25	0.17	0	0	0	1523.79	10.57
Solyc06g0	1.34	1.14	1.10	0.43	0.17	0.25	4.18	2.06
Solyc03g1	1.29	0.60	0.88	1.97	2.14	2.26	0.44	-1.20
Solyc04g0	1.40	1.07	1.29	2.04	2.82	2.98	0.48	-1.06
Solyc08g0	6.10	5.10	5.11	10.90	11.98	11.10	0.48	-1.06
Solyc10g0	1.47	1.40	1.92	0.80	0.87	0.71	2.02	1.01
Solyc11g0	0.49	0.38	0.15	0.13	0.11	0.04	3.66	1.87
Solyc12g0	1.19	1.18	1.51	3.13	2.60	2.62	0.47	-1.10
Solyc03g0	0.90	1.36	0.89	1.95	2.04	2.45	0.49	-1.03
Solyc08g0	0.70	0.91	0.65	0.38	0.16	0.22	2.98	1.57
Solyc12g0	0.14	0	0.14	1.18	1.08	0.98	0.09	-3.53
Solyc11g0	0.73	0.44	1.08	0	0	0	7510.88	12.87
Solyc02g0	0.81	0.98	0.81	0.47	0.33	0.49	2.00	1.00
Solyc08g0	0.40	0.32	0.33	0.12	0.06	0.08	4.04	2.01
Solyc05g0	0.30	0.43	0.38	0.07	0	0.08	7.28	2.86
Solyc08g0	0.74	0.68	0.81	0.11	0.34	0.10	4.09	2.03
Solyc01g0	0.08	0.08	0.08	0	0	0	761.47	9.57
Solyc09g0	0.22	0.17	0.25	0	0	0	2135.62	11.06
Solyc06g0	1.55	2.36	1.75	3.68	3.89	4.20	0.48	-1.06
Solyc09g0	0.12	0.05	0.15	0	0	0	1048.85	10.03
Solyc06g0	0.29	0.11	0.29	0.04	0	0	17.36	4.12
Solyc02g0	1.01	1.22	0.86	0.05	0	0.11	19.78	4.31
Solyc03g1	6.04	6.02	6.25	2.04	0.44	0.43	6.29	2.65
Solyc09g0	0	0	0.03	0.12	0.15	0.14	0.07	-3.89
Solyc01g0	0.05	0.08	0	0.62	1.33	0.92	0.04	-4.48
Solyc12g0	0.90	1.25	0.69	2.25	2.41	1.73	0.45	-1.17
Solyc02g0	0.20	0.08	0.17	0	0	0	1496.74	10.55
Solyc01g1	0	0	0	0.68	0.71	1.05	0.00	-12.99
Solyc08g0	0.74	0.54	0.48	0.21	0.21	0.20	2.80	1.49
Solyc10g0	0	0.07	0	0.60	1.09	0.78	0.03	-5.05
Solyc11g0	0	0	0	0.27	0.51	0.20	0.00	-11.68
Solyc01g1	0.50	0.46	0.25	0.09	0.02	0.15	4.73	2.24
Solyc07g0	0.33	0.16	0.25	0.05	0	0	14.86	3.89
Solyc07g0	0.56	0.74	0.37	0.12	0	0	13.43	3.75
Solyc09g0	1.16	0.87	1.01	0.58	0.28	0.22	2.82	1.50
Solyc07g0	0.10	0.34	0.26	0	0	0	2327.11	11.18
Solyc10g0	2.16	1.39	1.84	0.44	0.51	0.27	4.43	2.15
Solyc05g0	0.09	0.17	0.06	0	0	0	1063.88	10.06
Solyc12g0	0.62	0.35	0.32	0.05	0.10	0.09	5.33	2.42
Solyc01g0	0.13	0.13	0.78	2.46	3.06	2.99	0.12	-3.04
Solyc01g0	1.88	2.03	2.15	1.17	1.10	0.75	2.01	1.00
Solyc03g0	0.76	0.52	0.55	0.34	0.29	0.19	2.22	1.15
Solyc09g0	1.86	1.45	1.86	0.64	0.10	0.27	5.11	2.35
Solyc10g0	1.18	1.01	0.86	5.31	2.78	2.43	0.29	-1.79
Solyc04g0	0.18	0.20	0.35	0.61	1.03	0.53	0.34	-1.57
Solyc06g0	2.42	2.23	1.36	4.32	5.03	4.63	0.43	-1.22

Solyc03g0	0.33	0.42	0.37	0.21	0.11	0.14	2.40	1.26
Solyc03g0	0.36	0.40	0.50	0.13	0.12	0.10	3.69	1.88
Solyc02g0	1.08	0.83	0.76	3.06	2.46	2.21	0.35	-1.53
Solyc03g1	1.27	1.93	1.44	0.77	0.93	0.58	2.03	1.02
Solyc07g0	0.04	0.01	0.04	0.10	0.35	0.22	0.14	-2.88
Solyc02g0	0.52	0.16	0.58	2.68	2.27	1.25	0.20	-2.29
Solyc09g0	0.56	0.37	0.39	1.43	0.96	0.88	0.41	-1.30
Solyc11g0	0.18	0.13	0.12	0.53	0.45	0.76	0.25	-2.02
Solyc02g0	0.82	0.58	0.61	1.85	1.85	1.60	0.38	-1.40
Solyc02g0	0.57	1.15	0.82	2.17	2.23	1.90	0.41	-1.30
Solyc03g1	1.17	1.01	1.61	2.57	3.03	2.87	0.45	-1.16
Solyc09g0	113.76	117.25	124.03	42.80	12.19	10.70	5.40	2.43
Solyc01g0	0.09	0.03	0.15	0	0	0	894.88	9.81
Solyc03g1	0.41	0.13	0.17	0	0	0	2361.02	11.21
Solyc03g1	0.36	0.38	0	2.31	2.75	2.03	0.10	-3.25
Solyc01g0	0.39	0.50	0.35	0.87	1.18	0.85	0.43	-1.23
Solyc08g0	0.03	0.13	0.04	0	0	0	629.04	9.30
Solyc09g0	0.36	0.86	0.87	0.11	0.19	0	6.94	2.79
Solyc10g0	0.15	0.07	0.30	0	0	0	1721.61	10.75
Solyc05g0	7.36	8.43	8.42	2.67	0.91	0	6.77	2.76
Solyc02g0	0.11	0.30	0.21	0.94	0.60	0.64	0.28	-1.83
Solyc04g0	3.83	3.47	4.23	0.63	0.03	0.07	15.69	3.97
Solyc01g0	0.52	0.51	0.54	0.06	0	0	25.40	4.67
Solyc04g0	0.06	0.00	0.01	2.31	0.03	0.40	0.03	-5.17
Solyc02g0	0.14	0.29	0.14	0.04	0	0.03	7.78	2.96
Solyc06g0	0.03	0.04	0.13	0.19	0.71	0.42	0.15	-2.76
Solyc07g0	0.56	0.75	0.77	0.35	0.16	0.09	3.48	1.80
Solyc12g0	7.58	6.41	6.59	5.29	2.09	2.84	2.01	1.01
Solyc05g0	1.27	0.94	1.13	1.99	2.79	4.00	0.38	-1.39
Solyc01g0	0.13	0.10	0.30	0.04	0.03	0.03	5.28	2.40
Solyc05g0	0.59	0.53	0.69	0.05	0	0.13	10.06	3.33
Solyc04g0	1.07	0.99	1.28	0.09	0	0	39.10	5.29
Solyc08g0	0.03	0.06	0.05	0	0	0	455.76	8.83
Solyc10g0	1.89	1.85	1.73	4.38	5.52	5.91	0.35	-1.54
Solyc11g0	0.11	0.05	0.10	0	0	0	856.16	9.74
Solyc03g0	0.24	0.14	0.15	0	0	0	1794.95	10.81
Solyc07g0	0.48	0.50	0.49	1.48	1.93	1.74	0.28	-1.81
Solyc04g0	0.77	0.80	0.77	1.42	2.13	1.32	0.48	-1.06
Solyc06g0	0.14	0.14	0.32	0.65	0.83	0.93	0.25	-2.01
Solyc04g0	0	0	0	0.31	0.33	0.62	0.00	-12.04
Solyc06g0	0.70	0.70	0.86	0	0	0	7542.61	12.88
Solyc02g0	0.07	0.10	0.07	0.61	0.28	0.47	0.18	-2.48
Solyc04g0	0.10	0.08	0.34	0	0	0	1733.54	10.76
Solyc05g0	0.13	0.11	0.12	0.52	0.61	0.50	0.23	-2.14
Solyc10g0	5.06	4.93	4.48	11.19	11.27	11.51	0.43	-1.23
Solyc11g0	0.27	0.23	0.23	0	0	0	2410.23	11.23
Solyc11g0	0.20	0.21	0.23	0.80	0.78	0.79	0.27	-1.90
Solyc07g0	0.44	0.52	0.42	0.89	1.27	0.80	0.47	-1.10
Solyc02g0	0.42	0.60	0.06	1.42	2.13	2.09	0.19	-2.37
Solyc03g0	1.71	2.71	2.67	5.55	5.72	4.10	0.46	-1.12
Solyc06g0	0.29	0.33	0.30	0.82	0.91	0.97	0.34	-1.55

Solyc06g0	0	0	0	0.48	0.46	0.22	0.00	-11.92
Solyc12g0	0.35	0.49	0.37	0.13	0.12	0.07	3.71	1.89
Solyc04g0	0	0	0	1.01	1.00	0.65	0.00	-13.11
Solyc12g0	0.31	0.40	0.27	0.81	1.00	1.08	0.34	-1.56
Solyc04g0	1.29	1.56	2.15	0.93	0.44	0.74	2.37	1.25
Solyc03g0	0.64	0.72	0.39	1.11	1.51	1.83	0.39	-1.35
Solyc08g0	0.99	0.87	0.92	0.30	0.03	0	8.58	3.10
Solyc01g0	0.06	0.03	0	0.21	0.42	0.24	0.11	-3.21
Solyc06g0	1.64	1.48	1.84	0.47	1.25	0.66	2.09	1.06
Solyc01g1	0.20	0.44	0.56	0	0.09	0	13.38	3.74
Solyc03g0	0.06	0.04	0.02	0.19	0.26	0.22	0.19	-2.38
Solyc06g0	0.14	0.08	0.06	0	0	0	912.98	9.83
Solyc03g0	0.33	0.23	0.51	0.08	0.10	0.08	4.00	2.00
Solyc01g0	0.46	0.30	0.36	0.71	1.30	1.21	0.35	-1.51
Solyc09g0	1.44	1.43	1.43	0.89	0.74	0.42	2.09	1.07
Solyc05g0	0	0	0	0.55	0.62	0.85	0.00	-12.71
Solyc11g0	0.58	0.64	0.40	0.22	0.06	0.19	3.46	1.79
Solyc12g0	0.15	0.18	0.19	0.58	0.73	0.77	0.25	-2.01
Solyc03g0	0.08	0.16	0.21	0	0.03	0	15.71	3.97
Solyc01g0	1.35	1.57	0.86	0.64	0.32	0.56	2.48	1.31
Solyc04g0	0.01	0.01	0.02	0.09	0.05	0.05	0.18	-2.51
Solyc05g0	0.36	0.50	0.35	0.14	0.17	0.19	2.42	1.27
Solyc05g0	0.73	1.27	1.00	0.20	0	0	14.97	3.90
Solyc06g0	0.50	0.32	0.38	0	0	0	4013.78	11.97
Solyc02g0	0.88	0.62	0.74	0.05	0	0	44.26	5.47
Solyc09g0	0	0	0	0.17	0.15	0.12	0.00	-10.53
Solyc10g0	0	0.05	0	0.26	0.55	0.29	0.04	-4.58
Solyc01g0	0	0	0	0.37	0.42	0.43	0.00	-11.99
Solyc10g0	0.46	0.56	0.34	1.15	2.33	1.17	0.29	-1.77
Solyc12g0	0.13	0.08	0.23	0	0	0	1446.11	10.50
Solyc07g0	0.64	0.70	0.65	0.36	0.35	0.29	2.00	1.00
Solyc12g0	36.93	37.69	37.04	6.76	0.38	0.59	14.44	3.85
Solyc12g0	3.32	4.03	3.74	2.49	1.33	1.03	2.29	1.19
Solyc01g0	1.31	1.27	0.59	0.15	0.15	0	10.72	3.42
Solyc02g0	0.89	0.69	0.60	0.08	0	0	25.89	4.69
Solyc08g0	0.35	0.43	0.34	0.83	0.89	1.04	0.41	-1.30
Solyc01g0	1.03	0.98	0.79	2.49	2.08	1.52	0.46	-1.12
Solyc04g0	0.09	0.11	0.16	0	0	0	1185.03	10.21
Solyc06g0	0.16	0.14	0.18	0.58	0.47	0.36	0.34	-1.57
Solyc09g0	0.08	0.03	0.03	0	0	0	429.36	8.75
Solyc06g0	2.91	2.85	3.15	7.20	6.44	6.38	0.44	-1.17
Solyc11g0	0.03	0.03	0.04	0.07	0.18	0.19	0.23	-2.12
Solyc01g1	0.24	0.64	0.46	0.11	0	0	11.69	3.55
Solyc12g0	0	0	0.10	0.71	0.45	0.54	0.06	-4.10
Solyc12g0	28.19	28.79	30.41	3.23	0.26	0.05	24.66	4.62
Solyc05g0	1.83	2.50	1.82	1.23	0.59	0.72	2.43	1.28
Solyc11g0	0	0	0	0.57	1.16	0.25	0.00	-12.68
Solyc08g0	0.50	0.41	0.45	0.87	1.42	1.06	0.41	-1.30
Solyc04g0	0.21	0.11	0.05	0.75	0.59	0.45	0.21	-2.27
Solyc06g0	0	0.03	0	0.27	0.20	0.19	0.04	-4.64
Solyc12g0	0	0	0	0.48	0.37	0.43	0.00	-12.05

Solyc03g0	6.09	7.13	5.02	13.82	12.37	13.61	0.46	-1.13
Solyc03g0	0.36	0.54	0.49	0.07	0.15	0.06	4.97	2.31
Solyc02g0	0.69	0.65	0.55	0.26	0.10	0.21	3.35	1.74
Solyc02g0	0.70	0.57	0.53	0.32	0.37	0.17	2.10	1.07
Solyc01g0	0.66	1.17	2.34	4.95	5.50	3.02	0.31	-1.69
Solyc02g0	1.74	1.61	1.37	0.22	0.11	0.56	5.26	2.40
Solyc03g1	0.05	0.13	0.05	0	0	0	734.02	9.52
Solyc07g0	0.33	0.58	0.32	0.10	0.09	0	6.57	2.72
Solyc05g0	0.03	0.18	0.21	0	0	0	1370.75	10.42
Solyc02g0	0.03	0.03	0.03	0	0	0	307.12	8.26
Solyc12g0	0.04	0.05	0.05	0	0	0	467.31	8.87
Solyc02g0	0.92	0.91	1.26	0.41	0.50	0.45	2.27	1.18
Solyc01g0	0.02	0.02	0.07	0.21	0.43	0.33	0.12	-3.06
Solyc10g0	1.89	1.55	1.31	0.67	0.82	0.80	2.07	1.05
Solyc04g0	0.85	0.64	1.42	1.89	2.59	2.56	0.41	-1.27
Solyc01g1	1.98	2.31	2.16	1.34	0.96	0.80	2.08	1.06
Solyc01g0	0.15	0.12	0.19	0.03	0	0	14.28	3.84
Solyc02g0	0.35	0.41	0.40	1.91	1.87	1.85	0.21	-2.28
Solyc08g0	1.17	0.78	1.00	0.22	0.17	0	7.54	2.91
Solyc02g0	0.45	0.51	0.60	0.22	0.07	0.19	3.18	1.67
Solyc12g0	0.55	0.40	0.26	0.94	0.98	1.17	0.39	-1.35
Solyc01g0	0	0.09	0.14	0.71	0.66	0.49	0.12	-3.03
Solyc01g1	0.40	0.50	0.66	1.15	1.06	1.13	0.47	-1.09
Solyc03g0	0	0	0	0.34	0.09	0.27	0.00	-11.19
Solyc07g0	1.89	1.20	1.03	0.66	0.48	0.57	2.42	1.27
Solyc11g0	19.05	19.41	19.97	0.45	0	0	131.23	7.04
Solyc05g0	0.41	0.25	0.28	0.75	0.75	0.57	0.46	-1.13
Solyc07g0	4.23	4.17	2.43	0.25	0	0	42.77	5.42
Solyc08g0	1.06	0.97	1.14	0.53	0.44	0.55	2.09	1.06
Solyc03g1	0.17	0.32	0	0.95	0.85	1.53	0.15	-2.75
Solyc06g0	0.56	0.44	0.40	0.05	0	0.15	6.96	2.80
Solyc02g0	0.23	0.36	0.16	0.04	0	0	19.14	4.26
Solyc07g0	0.29	0.54	0.58	1.41	1.03	1.12	0.40	-1.33
Solyc10g0	0.08	0.04	0.08	0	0	0	683.10	9.42
Solyc03g1	0.22	0.11	0.18	0.02	0.06	0	6.23	2.64
Solyc03g0	0.30	0.28	0.21	0	0	0	2638.78	11.37
Solyc09g0	0	0	0	0.09	0.08	0.10	0.00	-9.78
Solyc08g0	0.06	0.03	0.04	0	0	0	451.28	8.82
Solyc03g1	0	0	0.16	1.44	2.65	1.41	0.03	-5.12
Solyc01g1	3.08	3.46	2.78	1.92	0.87	1.66	2.09	1.06
Solyc01g0	1.18	1.95	1.76	0.85	0.98	0.51	2.10	1.07
Solyc07g0	0.65	0.53	0.67	0.34	0.10	0.30	2.49	1.32
Solyc03g0	0.06	0.01	0.06	0.38	0.45	0.24	0.12	-3.11
Solyc11g0	2.01	2.11	1.75	5.38	3.23	3.70	0.48	-1.07
Solyc05g0	0	0	0	0.09	0.49	0.26	0.00	-11.45
Solyc01g1	0.09	0.12	0.15	0	0	0	1200.14	10.23
Solyc02g0	1.42	1.78	1.69	5.06	3.07	3.22	0.43	-1.21
Solyc01g0	0.60	0.26	0.23	1.26	1.08	1.06	0.32	-1.65
Solyc10g0	0.19	0.08	0.25	0.64	0.61	0.79	0.25	-1.99
Solyc03g0	0.79	0.82	1.15	2.32	2.35	1.56	0.44	-1.17
Solyc02g0	0.21	0.12	0.10	0	0	0	1446.12	10.50

Solyc04g0	14.44	11.29	13.04	1.58	0	0.56	18.06	4.17
Solyc04g0	0.06	0.06	0	0.29	0.35	0.34	0.12	-3.03
Solyc03g1	0.02	0	0	0.11	0.15	0.09	0.06	-4.15
Solyc10g0	0	0	0.02	0.32	0.43	0.26	0.02	-5.93
Solyc08g0	0.59	0.75	0.44	0.15	0.13	0.27	3.18	1.67
Solyc07g0	0.17	0.12	0.17	0	0.03	0.02	9.07	3.18
Solyc06g0	0.85	0.82	0.98	0.09	0	0.09	15.51	3.95
Solyc12g0	0	0	0	1.01	0.92	0.90	0.00	-13.20
Solyc03g0	0.21	0.22	0.54	0	0	0	3218.95	11.65
Solyc01g0	0	0	0	0.04	0.19	0.10	0.00	-10.07
Solyc09g0	1.35	2.34	1.74	1.24	0.53	0.60	2.29	1.20
Solyc07g0	1.97	1.32	1.16	0.77	0.30	0.37	3.11	1.64
Solyc12g0	0.98	0.86	1.01	0.46	0.26	0.05	3.66	1.87
Solyc01g1	1.36	1.37	2.06	0.09	0.17	0	18.01	4.17
Solyc09g0	0.06	0.02	0.06	0	0	0	461.50	8.85
Solyc11g0	0	0	0	0.05	0.11	0.02	0.00	-9.24
ENSRNA(	13.14	10.73	13.38	8.24	4.69	3.84	2.22	1.15
Solyc11g0	1.80	1.38	1.37	3.35	3.98	3.66	0.41	-1.27
Solyc12g0	0	0	0	0.05	0.18	0.06	0.00	-9.92
Solyc01g0	0.11	0.18	0.12	0.03	0.03	0	6.82	2.77
Solyc01g1	0.38	0.65	0.63	0.19	0.22	0.10	3.24	1.69
Solyc07g0	0.07	0.06	0.09	0	0	0	714.98	9.48
Solyc06g0	0.12	0.23	0.23	0.46	0.80	0.49	0.32	-1.62
Solyc07g0	1.48	0.89	1.50	0.61	0.56	0.77	2.01	1.01
Solyc09g0	0.50	0.38	0.26	0.06	0.13	0	5.86	2.55
Solyc11g0	0.17	0.19	0	1.06	1.31	1.16	0.10	-3.29
Solyc03g0	1.26	0.91	0.89	6.75	2.28	4.13	0.23	-2.10
Solyc04g0	0.12	0.08	0.07	0.48	0.42	0.34	0.22	-2.21
Solyc02g0	0.04	0.06	0.05	0.26	0.32	0.35	0.17	-2.58
Solyc10g0	0.39	0.46	0.55	0.17	0.18	0.18	2.60	1.38
Solyc04g0	0.58	0.75	0.73	0.13	0.49	0.22	2.47	1.30
Solyc12g0	0.94	1.00	0.95	2.51	2.24	1.43	0.47	-1.09
Solyc10g0	1.99	1.91	1.90	1.34	0.62	0.69	2.19	1.13
Solyc09g0	0.73	0.90	0.97	0.35	0.15	0.41	2.86	1.51
Solyc08g0	0.13	0.17	0.32	0.39	0.80	0.60	0.34	-1.54
Solyc07g0	0.02	0.05	0.04	0	0	0	401.43	8.65
Solyc05g0	0.09	0.05	0.06	0	0	0	668.09	9.38
Solyc02g0	0.38	0.61	0.35	0.10	0	0	13.99	3.81
Solyc12g0	0	0.12	0	0.95	0.64	1.48	0.04	-4.72
Solyc01g1	0.24	0.44	0.16	0.74	1.01	0.91	0.32	-1.66
Solyc12g0	0.33	1.08	0.38	0	0	0	5955.60	12.54
Solyc04g0	0.49	0.66	0.87	0.30	0.26	0.29	2.38	1.25
Solyc04g0	0	0	0.02	0.10	0.11	0.18	0.06	-3.97
Solyc01g0	1.18	1.03	0.58	1.65	2.75	2.71	0.39	-1.36
Solyc04g0	0.09	0.10	0.18	0	0	0	1227.78	10.26
Solyc06g0	0.89	0.53	0.64	0.22	0	0	9.44	3.24
Solyc06g0	0	0.06	0	1.15	0.20	0.52	0.03	-4.93
ENSRNA(	13.17	12.20	12.16	24.39	21.59	30.06	0.49	-1.02
Solyc01g0	0.36	0.24	0.28	0.79	0.67	0.63	0.42	-1.24
Solyc12g0	0.60	0.47	0.67	0.06	0	0.08	12.70	3.67
Solyc09g0	0.46	0.55	0.53	0.04	0.21	0.22	3.37	1.75

Solyc08g0	0.44	0.48	0.35	0.23	0.04	0.10	3.40	1.77
ENSRNA(	2.15	1.43	1.70	0.67	0.82	0.91	2.19	1.13
Solyc07g0	0.24	0.22	0.32	0.11	0.12	0.10	2.39	1.26
Solyc09g0	0.66	0.62	0.90	1.30	1.53	1.56	0.50	-1.01
Solyc04g0	0	0	0	0.21	0.16	0.19	0.00	-10.87
Solyc05g0	0.30	0.21	0.26	0.08	0	0	9.14	3.19
Solyc08g0	0.51	0.68	0.64	0.12	0	0	14.97	3.90
Solyc11g0	3.70	3.56	3.23	0.16	0	0	64.14	6.00
Solyc01g0	0.12	0.05	0.26	0.98	0.48	0.63	0.21	-2.28
Solyc02g0	0	0.04	0	0.10	0.21	0.37	0.06	-4.15
ENSRNA(	2.22	1.92	1.81	4.03	6.96	5.90	0.35	-1.51
Solyc02g0	21.63	23.40	19.98	45.00	29.10	56.33	0.50	-1.00
Solyc11g0	0.16	0.32	0.27	0.05	0.05	0.05	5.06	2.34
Solyc01g1	0	0	0	0.11	0.03	0.03	0.00	-9.07
Solyc03g0	0	0	0	0.05	0.08	0.05	0.00	-9.17
Solyc08g0	1.40	1.46	1.17	2.81	2.80	3.74	0.43	-1.21
Solyc05g0	0.41	0.38	0.70	0.11	0.19	0.17	3.21	1.68
Solyc10g0	0	0	0.01	0.15	0.15	0.06	0.02	-5.79
Solyc02g0	0.78	0.97	0.54	0.34	0.28	0.43	2.19	1.13
Solyc11g0	0.46	0.40	0.14	0.96	1.88	2.35	0.19	-2.38
Solyc03g0	0	0	0	0.53	0.29	0.54	0.00	-12.14
Solyc06g0	0.40	0.52	0.32	0.08	0	0	15.65	3.97
Solyc04g0	0.54	0.81	0.42	1.56	1.75	1.26	0.39	-1.37
Solyc07g0	0.07	0.08	0.08	0.28	0.25	0.32	0.27	-1.87
Solyc01g1	0.52	0.36	0.39	1.40	3.30	2.55	0.17	-2.52
Solyc02g0	0.35	0.22	0.18	0	0	0	2517.91	11.30
Solyc06g0	0.36	0.21	0.43	0.05	0.11	0.15	3.28	1.71
Solyc06g0	2.73	2.98	2.23	1.20	0.91	0.66	2.87	1.52
Solyc01g0	0.05	0.05	0.09	0	0	0	626.38	9.29
ENSRNA(	1.90	1.43	2.13	0.77	0.32	0.48	3.47	1.79
Solyc01g0	0.04	0.12	0.11	0	0	0	902.52	9.82
Solyc01g0	2.11	2.39	2.52	4.55	6.31	4.52	0.46	-1.13
Solyc02g0	0.25	0.20	0.25	0	0.07	0.03	7.09	2.83
Solyc06g0	0	0	0	0.05	0.15	0.04	0.00	-9.64
Solyc01g1	0.45	0.33	0.42	0.18	0.21	0.11	2.41	1.27
Solyc07g0	1.26	0.66	1.74	2.30	4.16	3.23	0.38	-1.40
Solyc05g0	1.23	1.14	1.47	2.89	2.94	2.42	0.47	-1.10
Solyc01g0	0.24	0.19	0.09	0	0.04	0	13.05	3.71
Solyc11g0	2.06	1.78	2.05	0.13	0	0	45.49	5.51
Solyc09g0	0.08	0.13	0.11	0.36	0.32	0.22	0.35	-1.53
Solyc01g0	0.99	0.90	0.74	1.41	1.93	2.13	0.48	-1.06
Solyc05g0	0.51	0.88	1.19	0	0.11	0.15	9.58	3.26
Solyc06g0	1.22	1.19	0.96	2.70	2.44	2.32	0.45	-1.14
Solyc07g0	0	0	0	0.30	0.27	0.08	0.00	-11.10
Solyc06g0	0.15	0.13	0.11	0.04	0	0	8.85	3.15
Solyc02g0	1.04	0.27	0.50	0.16	0	0	11.00	3.46
Solyc02g0	1.36	0.99	1.00	2.59	2.36	3.25	0.41	-1.29
Solyc02g0	0.70	0.61	0.45	1.19	1.23	1.17	0.49	-1.02
Solyc07g0	0.98	1.25	1.10	2.75	2.30	2.56	0.44	-1.19
Solyc09g0	5.74	5.23	5.62	0.23	0	0	73.03	6.19
Solyc12g0	0.23	0.35	0.14	1.82	1.10	1.58	0.16	-2.65

Solyc05g0	0.74	0.57	0.26	0.11	0.24	0.07	3.77	1.92
Solyc06g0	0.61	0.23	0.45	0.82	1.36	1.25	0.38	-1.41
Solyc02g0	0	0.04	0	0.23	0.27	0.13	0.07	-3.88
Solyc03g0	2.15	1.38	1.84	1.14	0.75	0.57	2.19	1.13
Solyc06g0	0.59	0.99	0.71	1.49	1.88	1.73	0.45	-1.16
Solyc10g0	0.25	0.15	0.08	0.60	0.48	0.76	0.26	-1.94
Solyc06g0	0.03	0.03	0.07	0	0	0	458.70	8.84
Solyc09g0	0.32	0.72	0.35	1.31	1.73	1.60	0.30	-1.74
Solyc08g0	0.18	0.26	0.43	0	0.12	0.02	6.46	2.69
Solyc11g0	0.61	0.39	0.37	0.20	0.16	0.28	2.15	1.10
Solyc09g0	0	0	0.03	0.28	0.13	0.17	0.06	-4.10
Solyc08g0	0.42	0.14	0.58	0.09	0	0	13.23	3.73
Solyc11g0	1.12	1.11	1.46	2.65	3.52	3.40	0.39	-1.37
Solyc03g0	0	0	0	0.38	0.21	0.23	0.00	-11.43
Solyc08g0	0.18	0.18	0.30	0.07	0.09	0.10	2.56	1.36
Solyc02g0	0.10	0.45	0.63	0.07	0	0	15.79	3.98
Solyc10g0	0.41	0.37	0.10	0	0	0	2935.96	11.52
Solyc03g0	0	0	0	0.95	0.41	0.70	0.00	-12.74
Solyc07g0	1.35	0.81	0.84	1.65	2.00	2.41	0.50	-1.01
Solyc01g0	0	0	0	0.05	0.08	0.05	0.00	-9.25
Solyc11g0	0.40	0.62	0.75	0.07	0.13	0.06	6.88	2.78
Solyc07g0	0.19	0.24	0.27	0.08	0.04	0.13	2.77	1.47
Solyc04g0	0	0.16	0	0.72	0.70	0.59	0.08	-3.62
Solyc04g0	0.23	0.37	0.12	0	0.07	0	9.75	3.29
Solyc03g0	1.11	1.93	1.01	4.46	4.35	2.34	0.36	-1.46
Solyc01g0	0.09	0.16	0.15	0.30	0.32	0.33	0.42	-1.24
Solyc03g0	0.62	0.55	0.49	0.14	0.16	0.36	2.51	1.33
Solyc07g0	0	0	0	2.15	0.34	0.93	0.00	-13.48
Solyc08g0	0.08	0.03	0	0.22	0.35	0.32	0.13	-2.97
Solyc12g0	0	0	0	0.04	0.12	0.13	0.00	-9.95
Solyc07g0	0.15	0.23	0.27	0	0.03	0.08	5.84	2.55
Solyc07g0	1.62	1.68	1.46	3.84	3.08	3.54	0.46	-1.13
Solyc02g0	0.15	0.06	0.24	0	0	0	1506.28	10.56
Solyc12g0	0.71	0.77	0.60	0.09	0.37	0.19	3.20	1.68
Solyc01g0	0.08	0.10	0.04	0.23	0.25	0.18	0.34	-1.54
Solyc03g0	0.02	0.10	0.07	0	0	0	627.68	9.29
Solyc11g0	0.12	0.04	0	0.59	0.47	0.73	0.09	-3.49
Solyc09g0	1.88	1.23	2.16	1.02	0.58	0.94	2.07	1.05
Solyc08g0	0.63	0.55	1.04	0.27	0.46	0.34	2.07	1.05
Solyc11g0	0.02	0	0	0.37	0.27	0.19	0.03	-5.20
Solyc06g0	0.63	0.72	0.43	0.08	0.07	0.15	5.98	2.58
Solyc05g0	0.04	0.05	0	0.18	0.23	0.19	0.14	-2.79
Solyc06g0	0.67	0.84	0.39	0.06	0	0	32.34	5.02
Solyc07g0	0.42	0.49	0.55	0.12	0.30	0.19	2.40	1.26
Solyc02g0	0.32	0.22	0.29	0.56	0.54	0.63	0.48	-1.07
Solyc08g0	0.10	0.07	0.05	0.02	0	0	12.03	3.59
Solyc06g0	0.28	0.13	0.21	0	0.06	0	10.22	3.35
Solyc01g0	2.17	2.41	1.37	4.86	4.91	5.99	0.38	-1.41
Solyc04g0	1.21	1.33	1.59	0	0.41	0	10.10	3.34
Solyc04g0	1.24	0.96	0.73	0.42	0.15	0	5.19	2.37
Solyc03g0	0.41	0.31	0.34	0	0.05	0.05	11.56	3.53

Solyc02g0	0	0	0	0.52	0.67	0.43	0.00	-12.40
Solyc05g0	0.37	0.60	0.56	1.12	1.09	0.87	0.50	-1.01
Solyc09g0	0.20	0.10	0.27	0	0	0	1910.72	10.90
Solyc07g0	2.01	1.47	2.07	3.24	5.14	2.96	0.49	-1.03
Solyc08g0	0.04	0.11	0.14	0.01	0.02	0	9.19	3.20
Solyc04g0	0.10	0.26	0.17	0.05	0.02	0	7.72	2.95
Solyc08g0	0.85	0.85	0.66	0.23	0.14	0.34	3.32	1.73
Solyc12g0	0.07	0.04	0	0	0	0	369.30	8.53
Solyc11g0	0.15	0	0	1.58	0.50	0.93	0.05	-4.33
Solyc09g0	0	0	0	0.01	0.08	0.05	0.00	-8.88
Solyc03g1	1.04	0.50	1.33	3.14	2.12	3.78	0.32	-1.65
Solyc02g0	1.75	1.93	1.50	4.99	3.13	6.40	0.36	-1.49
Solyc01g0	4.51	3.83	3.75	7.60	5.92	11.86	0.48	-1.07
Solyc11g0	0.18	0.21	0.54	1.02	2.08	0.77	0.24	-2.07
Solyc02g0	0.08	0.04	0.05	0	0	0	574.97	9.17
Solyc01g0	0.21	0.15	0.11	0	0	0	1566.92	10.61
Solyc09g0	0.22	0.17	0.07	0.61	0.76	0.92	0.20	-2.33
Solyc03g1	3.37	3.04	3.32	2.17	1.52	0.97	2.09	1.06
Solyc08g0	0.10	0.27	0.08	0	0	0	1537.41	10.59
Solyc02g0	0.77	1.36	1.11	0.09	0	0	34.22	5.10
Solyc09g0	0.19	0.12	0.12	0.40	0.54	0.38	0.34	-1.58
Solyc02g0	0.26	0.11	0.14	0.05	0.02	0.04	4.91	2.30
Solyc08g0	0.35	0.34	0.34	0	0.08	0.12	5.12	2.36
Solyc01g0	0.05	0.06	0.06	0	0	0	550.66	9.11
Solyc07g0	1.62	1.09	1.63	3.52	3.30	2.63	0.46	-1.12
Solyc11g0	0	0	0	0.75	0.70	0.53	0.00	-12.69
Solyc11g0	0.09	0.14	0.09	0.27	0.51	0.71	0.22	-2.19
Solyc11g0	14.14	12.86	13.99	0.35	0	0	116.23	6.86
Solyc07g0	0.04	0.01	0.06	0	0	0	383.57	8.58
Solyc09g0	0.34	0.19	0.29	0.78	1.29	0.40	0.34	-1.57
Solyc01g0	0	0.09	0	0.29	0.31	0.43	0.08	-3.58
Solyc06g0	1.28	1.32	1.19	0.92	0.23	0.37	2.48	1.31
Solyc03g1	0.05	0.04	0.02	0	0	0	356.83	8.48
Solyc09g0	0.18	0.04	0.19	0	0	0	1380.85	10.43
Solyc02g0	0.59	0.66	0.50	0.18	0.25	0.34	2.27	1.18
Solyc12g0	0.21	0.12	0	1.64	0.35	1.39	0.10	-3.34
Solyc03g0	2.08	2.22	2.18	4.32	6.01	3.88	0.46	-1.13
Solyc03g1	0.35	0.53	0.39	0	0	0	4226.00	12.05
Solyc12g0	0	0	0	0.38	0.36	0.15	0.00	-11.51
Solyc03g1	0.59	0.91	0.70	0.20	0.05	0.13	5.76	2.53
Solyc03g0	0.27	0.26	0.47	0.09	0	0	11.28	3.50
Solyc02g0	0.72	0.72	0.67	0	0.19	0.30	4.31	2.11
Solyc09g0	0	0	0	0.22	0.17	0.36	0.00	-11.29
Solyc12g0	0.40	0.43	0	2.03	0.97	1.95	0.17	-2.58
Solyc01g0	4.05	3.85	4.16	0.54	0	0.09	19.06	4.25
Solyc10g0	0.18	0.17	0.03	0.91	1.55	0.64	0.12	-3.03
Solyc04g0	2.40	1.41	1.60	0.16	0	0	32.87	5.04
Solyc11g0	0.09	0.05	0.16	0.50	0.44	0.33	0.23	-2.09
Solyc11g0	0.84	0.81	0.62	1.31	1.91	1.62	0.47	-1.09
Solyc06g0	0	0	0.02	0.06	0.04	0.16	0.07	-3.77
Solyc09g0	0.46	0.48	0.31	0.78	0.81	0.97	0.49	-1.03

Solyc06g0	1.18	1.01	0.88	2.73	1.79	2.70	0.43	-1.23
Solyc02g0	0.78	0.77	0.63	1.15	1.57	1.75	0.49	-1.03
Solyc04g0	0	0.27	0.06	0.89	1.43	0.75	0.10	-3.25
Solyc03g0	0.23	0	0.09	0.51	0.76	1.24	0.13	-3.00
Solyc06g0	0.02	0.08	0.09	0	0	0	659.59	9.37
Solyc01g1	0.78	0.49	1.08	0.28	0.38	0.19	2.76	1.46
Solyc02g0	0.44	0.20	0.21	0.55	1.02	0.83	0.35	-1.51
Solyc03g0	0.02	0.03	0.03	0	0	0	250.09	7.97
Solyc08g0	0.61	0.39	0.22	0.16	0.10	0.10	3.29	1.72
Solyc09g0	0.08	0	0.08	0	0	0	529.32	9.05
Solyc07g0	0.01	0	0.01	0.05	0.08	0.06	0.11	-3.12
Solyc06g0	1.23	0.66	1.07	2.24	2.24	1.60	0.49	-1.03
Solyc07g0	0.08	0.09	0.11	0.03	0	0	11.18	3.48
Solyc07g0	0.12	0.12	0.15	0.27	0.58	0.39	0.31	-1.68
Solyc12g0	0.14	0.11	0.07	0	0	0	1055.85	10.04
Solyc03g0	2.88	1.98	2.15	0.29	0.61	0.79	4.12	2.04
Solyc10g0	0	0.06	0	0.25	0.19	0.36	0.07	-3.77
Solyc06g0	0	0	0	0.37	0	0.21	0.00	-10.90
Solyc03g0	0.80	1.03	1.32	0.57	0.65	0.34	2.01	1.01
Solyc02g0	0.02	0.05	0.02	0	0	0	301.93	8.24
Solyc07g0	0.01	0.02	0.02	0	0	0	159.10	7.31
Solyc08g0	0.32	0.30	0.42	0.60	0.81	0.98	0.44	-1.20
Solyc06g0	0.39	0.38	0.33	0.99	0.68	0.70	0.46	-1.11
Solyc01g0	0.47	0.24	0.29	0.58	0.93	1.20	0.37	-1.43
Solyc07g0	0.10	0.09	0	0.31	0.21	0.48	0.19	-2.41
Solyc03g0	0	0	0	0.11	0	0.33	0.00	-10.51
Solyc02g0	0.25	0.13	0.11	0.34	0.54	0.53	0.35	-1.51
Solyc05g0	0	0	0	0.08	0.06	0.15	0.00	-9.92
Solyc10g0	0	0.12	0	0.42	0.32	0.48	0.10	-3.35
Solyc06g0	0.81	0.91	0.63	1.71	1.74	1.77	0.45	-1.15
Solyc08g0	0	0.18	0	0.89	0.74	0.80	0.07	-3.74
Solyc08g0	0.04	0	0.04	0.49	0.18	0.52	0.06	-3.96
Solyc03g0	0.14	0.08	0	0	0	0	748.45	9.55
Solyc02g0	1.48	1.78	0.66	2.38	3.77	3.24	0.42	-1.26
Solyc05g0	0.91	0.99	1.24	0.41	0	0	7.65	2.94
Solyc11g0	0.03	0	0.02	0.08	0.37	0.17	0.09	-3.52
Solyc02g0	0	0	0	0.21	0.55	0.43	0.00	-11.95
Solyc11g0	1.03	0.61	0.59	2.00	1.64	1.20	0.46	-1.12
Solyc01g0	0.02	0.02	0.08	0	0	0	392.54	8.62
Solyc01g1	0	0	0.07	0.27	0.25	0.41	0.08	-3.63
Solyc09g0	0	0	0	0.25	0.26	0.15	0.00	-11.10
Solyc01g0	0.31	0.10	0.08	0.65	0.58	0.82	0.24	-2.09
Solyc01g0	0	0	0	0	0.19	0.07	0.00	-9.77
Solyc02g0	0.30	0.03	0.12	0	0	0	1487.90	10.54
ENSRNA(	0.15	0	0.21	1.11	1.78	1.14	0.09	-3.50
Solyc08g0	0.06	0.06	0.07	0	0	0	599.38	9.23
Solyc02g0	0.40	0.45	0.51	1.00	1.24	0.77	0.45	-1.16
Solyc06g0	0.10	0.12	0.05	0.02	0	0	13.67	3.77
Solyc10g0	0.07	0	0	0.49	0.37	0.46	0.06	-4.16
Solyc11g0	0.67	1.37	0.76	0.18	0	0	15.49	3.95
Solyc09g0	2.26	1.83	2.23	5.11	3.43	5.13	0.46	-1.11

Solyc04g0	0.07	0.13	0.16	0.37	0.35	0.35	0.34	-1.57
Solyc07g0	0.07	0.08	0.08	0	0	0	770.05	9.59
Solyc01g0	0	0	0.04	0.16	0.15	0.22	0.08	-3.62
Solyc01g0	0.14	0.30	0.26	0	0	0	2335.77	11.19
Solyc12g0	0.12	0.21	0.12	0.06	0.03	0	4.99	2.32
Solyc12g1	0	0	0	0.09	0.06	0.13	0.00	-9.86
Solyc02g0	0.35	0.23	0.28	0.10	0.18	0.07	2.45	1.30
Solyc11g0	0	0	0	0.23	0.23	0.09	0.00	-10.82
Solyc03g0	0.39	0.32	0.34	0.75	0.76	1.18	0.39	-1.35
Solyc02g0	0.05	0.05	0.02	0	0	0	412.01	8.69
Solyc08g0	0.21	0.16	0.29	0.06	0.09	0	4.19	2.07
Solyc04g0	0.23	0	0.51	0	0	0	2448.95	11.26
Solyc04g0	0.47	0.21	0.57	0.16	0.23	0.15	2.31	1.21
Solyc05g0	0.62	0.75	0.44	0.29	0.14	0.31	2.44	1.28
Solyc09g0	5.60	4.79	7.60	1.14	0.21	0	13.30	3.73
Solyc06g0	0.15	0.11	0.16	0.39	0.22	0.60	0.35	-1.52
Solyc01g1	0.04	0	0	0.18	0.13	0.22	0.08	-3.66
Solyc02g0	1.37	1.58	1.21	1.92	3.89	4.47	0.40	-1.31
Solyc06g0	0	0.44	0.35	1.47	1.42	1.34	0.19	-2.41
Solyc02g0	0.06	0	0.06	0	0	0	394.05	8.62
Solyc02g0	1.22	1.24	2.03	0.86	0.36	0.56	2.53	1.34
Solyc07g0	0.77	0.89	0.73	0.17	0	0.07	9.83	3.30
Solyc01g1	0.33	0.36	0.54	0.15	0.10	0.25	2.45	1.29
Solyc06g0	0.40	0.50	0.24	0.06	0	0	17.92	4.16
Solyc03g0	0.27	0.18	0.27	0.41	0.58	0.69	0.43	-1.23
Solyc12g0	0.13	0.22	0.14	0.03	0.05	0	6.07	2.60
Solyc01g0	0	0	0.11	0.51	0.57	0.25	0.08	-3.59
Solyc03g0	0	0	0	0	0.11	0.19	0.00	-9.99
Solyc12g0	1.33	1.16	1.16	0.57	0.43	0.59	2.29	1.20
Solyc02g0	0	0.02	0	0.19	0.07	0.06	0.07	-3.78
Solyc01g0	0	0.09	0	0.62	0.54	0.84	0.05	-4.42
Solyc09g0	1.54	1.09	1.30	0.22	0	0.17	10.24	3.36
Solyc11g0	0.15	0.05	0.05	0	0	0	814.09	9.67
Solyc05g0	0.11	0.12	0.11	0.25	0.23	0.33	0.41	-1.28
Solyc10g0	0.78	0.74	0.76	0.32	0.45	0.28	2.16	1.11
Solyc03g1	0.38	0.24	0.22	0.67	0.58	0.60	0.45	-1.15
Solyc03g0	0.06	0.23	0.20	0.34	0.40	0.61	0.36	-1.46
Solyc05g0	0.09	0	0	0.36	0.36	0.23	0.09	-3.46
Solyc02g0	0.14	0.34	0.32	0.09	0	0	8.45	3.08
Solyc01g0	0.29	0.29	0.57	0	0	0	3846.31	11.91
Solyc02g0	0.24	0	0.18	0	0	0	1403.48	10.45
Solyc08g0	0.13	0.25	0.30	0.12	0.09	0.06	2.51	1.33
Solyc02g0	0.45	0.06	0.23	0.03	0	0.07	6.79	2.76
Solyc09g0	0	0	0	0.92	0.47	0.38	0.00	-12.54
Solyc09g0	0	0	0	0.03	0.02	0.04	0.00	-8.11
Solyc01g1	0.59	0.35	0.48	1.17	0.93	0.88	0.47	-1.07
Solyc12g0	0	0	0	0.04	0.03	0.01	0.00	-8.12
Solyc02g0	0	0	0	0.23	0.01	0.15	0.00	-10.36
Solyc07g0	0	0	0	0.03	0.02	0.01	0.01	-7.50
Solyc04g0	0.38	0.35	0.24	0.10	0.10	0.13	2.90	1.54
Solyc08g0	0.47	0.61	0.28	1.08	0.89	1.24	0.42	-1.24

Solyc01g0	0.39	0.33	0.86	0.18	0.10	0.21	3.28	1.71
Solyc06g0	0.53	0.19	0.59	0.16	0	0.08	5.59	2.48
Solyc06g0	0.46	0.69	0.85	1.16	1.74	1.57	0.45	-1.16
Solyc06g0	0.25	0.44	0.34	0.62	0.84	0.86	0.44	-1.17
Solyc06g0	0.67	0.66	0.64	0.19	0	0	10.58	3.40
Solyc06g0	0.32	0.12	0.15	0	0	0.04	13.74	3.78
Solyc06g0	0	0	0	0.04	0.10	0.07	0.00	-9.45
Solyc02g0	0	0.06	0	0.28	0.15	0.56	0.06	-4.03
Solyc05g0	0.13	0	0.11	0	0	0	768.68	9.59
Solyc02g0	0	15.25	17.02	0	0	0	107563.96	16.71
Solyc05g0	4.69	4.10	5.10	0.58	0.09	0	20.56	4.36
Solyc07g0	0.65	0.75	0.44	0.27	0.08	0.28	2.88	1.52
Solyc03g0	1.27	1.39	1.77	4.13	2.89	3.91	0.40	-1.31
Solyc09g0	0.13	0	0.11	0	0	0	783.34	9.61
Solyc07g0	1.48	1.67	0.81	2.78	2.89	3.20	0.45	-1.16
Solyc10g0	0	0	0	0	0.13	0.14	0.00	-9.79
Solyc06g0	0.57	0.71	0.91	1.38	2.21	1.48	0.43	-1.22
Solyc02g0	0.19	0.13	0.27	0.63	0.41	0.59	0.37	-1.45
Solyc01g1	0	0	0	0.05	0.06	0.04	0.00	-8.99
Solyc10g0	2.05	1.01	1.00	3.12	6.00	3.11	0.33	-1.59
Solyc05g0	1.43	1.13	1.01	0.17	0	0	20.96	4.39
Solyc03g0	0.12	0.06	0	0	0	0	604.27	9.24
Solyc03g0	0.49	0.29	0.71	1.38	0.86	1.15	0.44	-1.19
Solyc03g1	0.82	0.65	0.51	0	0.12	0.04	12.11	3.60
Solyc06g0	0.96	0.92	0.47	0.08	0.24	0.08	5.82	2.54
Solyc02g0	2.01	2.52	2.34	0.25	0	0	27.11	4.76
Solyc11g0	0.26	0.34	0.07	0.81	0.73	0.82	0.28	-1.82
Solyc11g0	0.38	0.54	0.40	0.66	1.43	0.94	0.43	-1.20
Solyc05g0	0.10	0.16	0.13	0.43	0.38	0.30	0.36	-1.48
Solyc06g0	1.39	1.58	1.63	0.31	0	0.05	12.77	3.67
Solyc06g0	0.09	0	0.09	0	0	0	587.73	9.20
Solyc03g0	0	0	0	0.22	0.39	0.30	0.00	-11.56
Solyc05g0	3.45	3.13	2.59	6.08	5.94	7.60	0.47	-1.10
Solyc08g0	0.74	0.43	0.42	1.21	1.06	1.58	0.41	-1.27
Solyc02g0	0.82	0.57	0.64	0.27	0.36	0.18	2.49	1.31
Solyc04g0	0.55	0.50	0.62	0.28	0.26	0.04	2.84	1.51
Solyc01g1	0.88	1.05	0.88	2.34	2.10	1.68	0.46	-1.12
Solyc10g0	0	0	0	0	0.90	0.77	0.00	-12.44
Solyc01g0	1.97	1.75	1.90	4.68	2.33	4.53	0.49	-1.04
Solyc11g0	0	0.30	0.40	0	0	0	2317.83	11.18
Solyc02g0	0.08	0.12	0.12	0.05	0.04	0.04	2.47	1.31
Solyc12g0	0.05	0.15	0.54	0.05	0	0	13.87	3.79
Solyc02g0	0.39	0.40	0.64	0.32	0.11	0.24	2.16	1.11
Solyc02g0	0.05	0	0.10	0	0	0	517.85	9.02
Solyc04g0	0.15	0.12	0	0	0	0	883.93	9.79
Solyc10g0	0	0	0	0.03	0.08	0.05	0.00	-9.02
Solyc12g0	0.47	1.01	0.36	0.20	0	0	8.97	3.17
Solyc11g0	0.03	0.02	0.04	0	0	0	281.82	8.14
Solyc08g0	0.27	0.29	0.18	0.51	0.52	0.48	0.49	-1.04
Solyc08g0	1.92	1.92	1.80	4.86	3.10	7.13	0.37	-1.42
Solyc09g0	0.51	0.63	0.59	0.28	0	0.12	4.42	2.14

Solyc07g0	0.81	0.68	1.01	0.24	0.65	0.25	2.18	1.13
Solyc05g0	0.13	0.11	0.19	0.27	0.54	0.56	0.31	-1.68
Solyc06g0	0.19	0.25	0.07	0.59	0.60	0.36	0.33	-1.59
Solyc11g0	0	0	0	0.19	0.04	0.08	0.00	-10.02
Solyc06g0	0.55	0.47	0.58	0.09	0.23	0.13	3.49	1.80
Solyc09g0	0.13	0.13	0.11	0.04	0.02	0.05	3.40	1.76
Solyc09g0	0.03	0.05	0.03	0	0	0	362.34	8.50
Solyc06g0	0.34	0.52	0.21	0	0	0	3600.66	11.81
ENSRNA(	3.31	3.00	2.31	5.91	8.02	5.93	0.43	-1.20
Solyc03g0	0	0	0	0.27	0.20	0.25	0.00	-11.24
Solyc01g0	0.32	0.28	0.30	0.56	0.61	0.72	0.48	-1.06
Solyc06g0	1.26	1.37	1.55	0	0.63	0	6.64	2.73
Solyc03g0	0.70	0.39	0.74	1.44	1.12	1.43	0.46	-1.12
Solyc03g0	2.41	2.00	2.07	0.35	0	0.03	17.00	4.09
Solyc08g0	0.33	0.26	0.18	0.11	0.10	0.05	2.98	1.58
Solyc01g0	1.11	1.39	0.81	2.79	1.89	2.61	0.45	-1.14
Solyc07g0	0.04	0.01	0.02	0	0	0	255.70	8.00
Solyc09g0	0.16	0.23	0.20	0.40	0.51	0.65	0.38	-1.39
Solyc01g0	0.46	0.09	0.16	0	0	0	2354.26	11.20
Solyc08g0	0	0.14	0	0.40	0.66	0.47	0.09	-3.48
Solyc01g0	1.34	1.14	1.97	0.18	0	0	25.34	4.66
Solyc02g0	0	0.07	0.06	0.36	0.25	0.17	0.17	-2.56
Solyc01g0	0.04	0.08	0.12	0.36	0.29	0.32	0.25	-1.98
Solyc03g0	0.05	0	0.08	0	0	0	420.75	8.72
Solyc08g0	0	0.05	0	0.18	0.11	0.15	0.11	-3.25
Solyc08g0	0.48	0.47	0.69	1.66	0.63	3.03	0.31	-1.70
Solyc04g0	0	0	0	1.32	0	0.60	0.00	-12.65
Solyc01g1	0.45	0.45	0.55	0.11	0.27	0.31	2.13	1.09
Solyc12g0	0.16	0.16	0.41	0.04	0.09	0.05	4.02	2.01
Solyc01g0	0.03	0.03	0.04	0	0	0	312.22	8.29
Solyc03g1	0.12	0	0	0.78	0.25	0.59	0.07	-3.78
Solyc01g0	0.23	0	0	0.41	1.02	0.82	0.10	-3.31
Solyc11g0	0.21	0.14	0.12	0.07	0.04	0.06	2.74	1.46
Solyc03g0	0.81	0.87	0.57	1.97	1.29	1.44	0.48	-1.06
Solyc03g0	146.74	131.21	129.07	2.52	0	0	161.83	7.34
Solyc06g0	4.27	3.73	4.19	0.34	0	0	35.97	5.17
Solyc01g1	0.22	0.16	0.08	0.04	0.06	0	4.58	2.20
Solyc06g0	3.15	3.80	3.00	6.90	5.34	9.28	0.46	-1.11
Solyc02g0	0	0	0	0.09	0	0.37	0.00	-10.57
Solyc04g0	0.39	0.26	0.36	0.10	0	0.09	5.43	2.44
Solyc08g0	0.04	0.06	0.04	0	0	0	474.28	8.89
Solyc06g0	0.23	0.16	0.08	0	0	0.05	9.89	3.31
Solyc06g0	0.27	0.31	0.37	0.06	0.09	0.06	4.49	2.17
Solyc08g0	0	0	0.05	0.11	0.24	0.15	0.10	-3.29
Solyc02g0	0.10	0.18	0	0	0	0	960.34	9.91
Solyc01g0	0	0	0	0.38	0.56	0	0.00	-11.62
Solyc01g1	0	0	0	0.01	0.05	0.17	0.00	-9.56
Solyc12g0	0.21	0.32	0.19	0	0	0	2361.68	11.21
ENSRNA(	2.91	1.36	2.65	1.14	0.91	1.30	2.06	1.05
Solyc07g0	0.09	0.17	0.13	0.26	0.49	0.24	0.39	-1.35
Solyc06g0	0.50	1.13	0.48	0.30	0.28	0.22	2.66	1.41

Solyc04g0	0.07	0.17	0.16	0.04	0	0	9.52	3.25
Solyc02g0	0.02	0.10	0.06	0.02	0	0	11.78	3.56
Solyc03g1	0.22	0.51	0.37	0.22	0.12	0.16	2.21	1.14
Solyc01g1	0	0	0	0.13	0.13	0.08	0.00	-10.11
Solyc02g0	0.06	0.05	0.06	0.24	0.36	0.10	0.23	-2.09
Solyc03g0	0.69	1.10	0.51	0.15	0.20	0	6.49	2.70
Solyc08g0	3.60	2.83	3.03	0.70	0.12	0	11.44	3.52
Solyc03g0	0.10	0.13	0.07	0.18	0.16	0.42	0.39	-1.37
Solyc11g0	0.60	0.55	0.25	1.40	2.00	1.18	0.30	-1.71
Solyc11g0	0.15	0.17	0.16	0.10	0.06	0.03	2.48	1.31
Solyc12g0	0	0	0	0.18	0.12	0.08	0.00	-10.33
Solyc04g0	0.28	0.26	0.29	0.95	0.55	0.46	0.42	-1.24
Solyc02g0	0.30	0.38	0.31	0.68	0.96	0.79	0.41	-1.29
Solyc08g0	0.44	0.63	0.78	1.62	1.87	1.66	0.36	-1.48
Solyc05g0	0.10	0.04	0.04	0	0	0	620.98	9.28
Solyc03g1	0	0	0	0.03	0.05	0.06	0.00	-8.90
Solyc04g0	0	0.11	0.06	0	0	0	553.61	9.11
Solyc11g0	1.52	0.71	2.28	3.93	4.50	3.50	0.38	-1.40
Solyc03g0	0	0	0	0.16	0	0.14	0.00	-9.94
Solyc01g0	0.41	0.25	0.18	0.06	0.15	0.13	2.50	1.32
Solyc02g0	0.39	1.38	2.22	0.17	0.45	0.59	3.31	1.73
Solyc06g0	0.01	0.51	0	0.70	1.57	2.20	0.12	-3.11
Solyc08g0	0	0	0	0.19	0.05	0.11	0.00	-10.17
Solyc11g0	0.32	0.27	0.18	0.42	0.56	0.72	0.46	-1.12
Solyc03g0	0.07	0	0	0.09	0.18	0.35	0.11	-3.20
Solyc11g0	0.59	0.70	0.07	1.93	1.32	1.71	0.27	-1.87
Solyc02g0	5.19	5.27	5.61	0.79	0.04	0	19.25	4.27
Solyc10g0	0.02	0.15	0.04	0	0	0	687.65	9.43
Solyc05g0	0.64	0.67	0.53	0.31	0.20	0.30	2.28	1.19
Solyc08g0	0.74	0.73	0.62	0.10	0	0	20.90	4.39
Solyc04g0	0.29	0.10	0.07	0	0	0	1528.10	10.58
Solyc03g0	0.38	0.56	0.38	0.24	0	0.09	4.00	2.00
Solyc05g0	0.31	0.38	0.22	0	0	0	3034.84	11.57
Solyc02g0	0.54	0.46	0.48	0.20	0	0.22	3.52	1.82
Solyc10g0	0.33	0.35	0.34	0.67	0.77	0.79	0.46	-1.13
Solyc02g0	0.54	0.46	0.59	0.30	0.22	0.24	2.09	1.07
Solyc07g0	0.81	0.73	0.69	0.45	0.21	0.19	2.61	1.38
Solyc11g0	0.42	0.36	0.32	0	0.06	0	18.97	4.25
ENSRNA(	1.26	1.42	1.68	0.32	0.33	0	6.74	2.75
Solyc04g0	0.78	0.43	0.37	0.13	0.14	0.21	3.28	1.72
Solyc09g0	0.36	0.28	0.12	0.13	0.02	0	5.06	2.34
Solyc11g0	0.46	0.34	0.65	0	0	0.14	10.33	3.37
Solyc12g0	0	0	0	0.12	0.15	0.08	0.00	-10.19
Solyc04g0	0.97	0.96	0.93	0.40	0.67	0.15	2.34	1.23
Solyc09g0	0	0	0	0.06	0.04	0.16	0.00	-9.75
Solyc12g0	0.04	0.02	0.02	0.01	0	0	11.23	3.49
Solyc10g0	0.20	0.26	0.20	0.13	0.05	0.09	2.42	1.27
Solyc04g0	0	0	0.70	2.51	1.28	1.83	0.12	-3.01
Solyc08g0	0.24	0.31	0	0.75	0.85	1.04	0.21	-2.27
Solyc07g0	0.30	0.07	0.27	0.05	0.04	0	7.27	2.86
Solyc06g0	1.02	1.01	0.85	0	0.30	0.07	7.72	2.95

Solyc01g1	0.43	0.20	0.33	0.86	1.17	0.54	0.37	-1.43
Solyc04g0	0.15	0	0.07	0	0	0	732.58	9.52
Solyc05g0	0.13	0.20	0.03	0.03	0.03	0	6.61	2.73
Solyc11g0	0.24	0.18	0.18	0.06	0	0	9.85	3.30
Solyc03g0	0	0	0.02	0.06	0.07	0.05	0.10	-3.29
Solyc02g0	0.23	0.39	0.20	0.43	0.63	0.62	0.49	-1.03
Solyc11g0	0.28	0.16	0.56	0.60	0.91	0.82	0.43	-1.23
Solyc03g0	0	0	0	0.83	0.45	0	0.00	-12.06
Solyc03g0	2.16	1.61	2.40	0.20	0	0	31.13	4.96
Solyc08g0	0.51	0.57	0.64	0.14	0	0.18	5.35	2.42
Solyc03g1	0.47	0.29	0.57	0.95	1.00	1.20	0.42	-1.24
Solyc11g0	0.02	0.02	0.05	0	0	0	292.92	8.19
Solyc11g0	0.19	0	0.44	1.01	1.17	0.72	0.22	-2.20
Solyc05g0	0	0	0	0.09	0.22	0.09	0.00	-10.39
Solyc06g0	0	0	0.04	0.22	0.19	0.02	0.08	-3.60
Solyc09g0	0.39	0.16	0.06	0.74	0.94	0.75	0.25	-2.00
Solyc06g0	0.20	0.09	0.42	0.50	0.92	0.87	0.31	-1.70
Solyc07g0	0.24	0.23	0.13	0.42	0.57	0.51	0.40	-1.31
Solyc09g0	0.12	0.14	0	0.39	0.33	0.49	0.22	-2.22
Solyc07g0	0.07	0.07	0	0	0	0	470.92	8.88
Solyc02g0	0.10	0.16	0.17	0.36	0.43	0.31	0.39	-1.34
Solyc01g0	0	0	0.12	1.01	1.41	1.40	0.03	-4.98
Solyc01g0	0.11	0.04	0.07	0	0.01	0	23.29	4.54
Solyc05g0	0.51	0.37	0.23	0.12	0.08	0.04	4.80	2.26
Solyc03g0	0.81	0.50	0.86	0.18	0.19	0.15	4.16	2.06
Solyc06g0	0.08	0	0.05	0	0	0	444.70	8.80
Solyc11g0	0	0	0	0.06	0.12	0	0.00	-9.22
Solyc09g0	0.06	0.16	0	0	0	0	740.17	9.53
Solyc09g0	0.32	0.47	0.28	0.78	1.02	0.65	0.44	-1.19
Solyc02g0	0	0	0	0.08	0	0.32	0.00	-10.40
Solyc04g0	1.66	0.60	0.39	0	0.13	0.18	8.56	3.10
Solyc02g0	0.48	0.77	0.61	0.25	0	0	7.52	2.91
Solyc03g0	0.17	0.15	0.10	0.27	0.36	0.43	0.41	-1.29
Solyc04g0	1.36	1.80	2.09	0.20	0	0	26.73	4.74
Solyc07g0	0.25	0.42	0.49	0.21	0.15	0.04	2.87	1.52
Solyc08g0	0	0	0	0	0.13	0.13	0.00	-9.72
Solyc04g0	0	0	0	0	0.10	0.10	0.00	-9.36
Solyc11g0	2.18	1.13	2.17	5.61	3.78	4.16	0.40	-1.31

pval	qval	regulation
0	0	up
0	0	up
0	0	down
0	0	up
0	0	up
0	0	up
0	0	down
0	0	up
0	0	up
0	0	down
0	0	down
0	0	down
0	0	up
0	0	up
0	0	down
0	0	down
0.00	0.00	up
0.00	0.00	up
0.00	0.00	down
0.00	0.00	up
0.00	0.00	up
0.00	0.00	up
0.00	0.00	up
0.00	0.00	down
0.00	0.00	up
0.00	0.00	up
0.00	0.00	up
0.00	0.00	down
0.00	0.00	down
0.00	0.00	up
0.00	0.00	up
0.00	0.00	up
0.00	0.00	down
0.00	0.00	down
0.00	0.00	down
0.00	0.00	up
0.00	0.00	down
0.00	0.00	up
0.00	0.00	up
0.00	0.00	up
0.00	0.00	up
0.00	0.00	up
0.00	0.00	up
0.00	0.00	up
0.00	0.00	up
0.00	0.00	down
0.00	0.00	down
0.00	0.00	up
0.00	0.00	down

0.00	0.00	up
0.00	0.00	down
0.00	0.00	up
0.00	0.00	up
0.00	0.00	down
0.00	0.00	down
0.00	0.00	up
0.00	0.00	up
0.00	0.00	up
0.00	0.00	up
0.00	0.00	up
0.00	0.00	up
0.00	0.00	up
0.00	0.00	up
0.00	0.00	down
0.00	0.00	up
0.00	0.00	up
0.00	0.00	down
0.00	0.00	down
0.00	0.00	down
0.00	0.00	down
0.00	0.00	down
0.00	0.00	up
0.00	0.00	up
0.00	0.00	up
0.00	0.00	up
0.00	0.00	up
0.00	0.00	up
0.00	0.00	up
0.00	0.00	down
0.00	0.00	down
0.00	0.00	up
0.00	0.00	up
0.00	0.00	up
0.00	0.00	up
0.00	0.00	down
0.00	0.00	up
0.00	0.00	up
0.00	0.00	up
0.00	0.00	down
0.00	0.00	up
0.00	0.00	up
0.00	0.00	down
0.00	0.00	up
0.00	0.00	up
0.00	0.00	down
0.00	0.00	down
0.00	0.00	down
0.00	0.00	up
0.00	0.00	down
0.00	0.00	up

0.00	0.00	up
0.00	0.00	up
0.00	0.00	up
0.00	0.00	up
0.00	0.00	up
0.00	0.00	down
0.00	0.00	up
0.00	0.00	up
0.00	0.00	down
0.00	0.00	up
0.00	0.00	down
0.00	0.00	up
0.00	0.00	down
0.00	0.00	up
0.00	0.00	down
0.00	0.00	down
0.00	0.00	down
0.00	0.00	up
0.00	0.00	up
0.00	0.00	up
0.00	0.00	up
0.00	0.00	up
0.00	0.00	down
0.00	0.00	down
0.00	0.00	up
0.00	0.00	up
0.00	0.00	down
0.00	0.00	down
0.00	0.00	up
0.00	0.00	up
0.00	0.00	up
0.00	0.00	up
0.00	0.00	up
0.00	0.00	up
0.00	0.00	up
0.00	0.00	up
0.00	0.00	up
0.00	0.00	up
0.00	0.00	up
0.00	0.00	up
0.00	0.00	up
0.00	0.00	up
0.00	0.00	up
0.00	0.00	up
0.00	0.00	up
0.00	0.00	up
0.00	0.00	up
0.00	0.00	down
0.00	0.00	up
0.00	0.00	up
0.00	0.00	up

0.00	0.00	up
0.00	0.00	down
0.00	0.00	up
0.00	0.00	up
0.00	0.00	up
0.00	0.00	down
0.00	0.00	up
0.00	0.00	up
0.00	0.00	down
0.00	0.00	up
0.00	0.00	up
0.00	0.00	up
0.00	0.00	down
0.00	0.00	down
0.00	0.00	up
0.00	0.00	up
0.00	0.00	up
0.00	0.00	up
0.00	0.00	down
0.00	0.00	down
0.00	0.00	up
0.00	0.00	down
0.00	0.00	up
0.00	0.00	up
0.00	0.00	down
0.00	0.00	up
0.00	0.00	up
0.00	0.00	up
0.00	0.00	up
0.00	0.00	down
0.00	0.00	down
0.00	0.00	up
0.00	0.00	up
0.00	0.00	up
0.00	0.00	down
0.00	0.00	up
0.00	0.00	up
0.00	0.00	down
0.00	0.00	down
0.00	0.00	up
0.00	0.00	up
0.00	0.00	down
0.00	0.00	up
0.00	0.00	down
0.00	0.00	up
0.00	0.00	up
0.00	0.00	up
0.00	0.00	up
0.00	0.00	down
0.00	0.00	up
0.00	0.00	up

0.00	0.00	down
0.00	0.00	up
0.00	0.00	up
0.00	0.00	up
0.00	0.00	down
0.00	0.00	up
0.00	0.00	up
0.00	0.00	up
0.00	0.00	down
0.00	0.00	down
0.00	0.00	down
0.00	0.00	up
0.00	0.00	up
0.00	0.00	down
0.00	0.00	down
0.00	0.00	up
0.00	0.00	down
0.00	0.00	down
0.00	0.00	up
0.00	0.00	down
0.00	0.00	down
0.00	0.00	up
0.00	0.00	up
0.00	0.00	down
0.00	0.00	up
0.00	0.00	up
0.00	0.00	down
0.00	0.00	down
0.00	0.00	down
0.00	0.00	up
0.00	0.00	up
0.00	0.00	up
0.00	0.00	up
0.00	0.00	up
0.00	0.00	down
0.00	0.00	up
0.00	0.00	up
0.00	0.00	up
0.00	0.00	down
0.00	0.00	down
0.00	0.00	up
0.00	0.00	down
0.00	0.00	up
0.00	0.00	down
0.00	0.00	up
0.00	0.00	up
0.00	0.00	down
0.00	0.00	down
0.00	0.00	up
0.00	0.00	up
0.00	0.00	up

0.00	0.00	up
0.00	0.00	down
0.00	0.00	up
0.00	0.00	down
0.00	0.00	down
0.00	0.00	up
0.00	0.00	down
0.00	0.00	up
0.00	0.00	down
0.00	0.00	down
0.00	0.00	down
0.00	0.00	up
0.00	0.00	down
0.00	0.00	up
0.00	0.00	up
0.00	0.00	up
0.00	0.00	up
0.00	0.00	up
0.00	0.00	up
0.00	0.00	up
0.00	0.00	down
0.00	0.00	up
0.00	0.00	up
0.00	0.00	up
0.00	0.00	up
0.00	0.00	down
0.00	0.00	up
0.00	0.00	up
0.00	0.00	up
0.00	0.00	up
0.00	0.00	up
0.00	0.00	up
0.00	0.00	down
0.00	0.00	up
0.00	0.00	up
0.00	0.00	up
0.00	0.00	down
0.00	0.00	up
0.00	0.00	up
0.00	0.00	up
0.00	0.00	down
0.00	0.00	up
0.00	0.00	up
0.00	0.00	down
0.00	0.00	down
0.00	0.00	down
0.00	0.00	up
0.00	0.00	down
0.00	0.00	up
0.00	0.00	up
0.00	0.00	up

0.00	0.00	up
0.00	0.00	up
0.00	0.00	down
0.00	0.00	down
0.00	0.00	down
0.00	0.00	up
0.00	0.00	down
0.00	0.00	up
0.00	0.00	up
0.00	0.00	down
0.00	0.00	down
0.00	0.00	down
0.00	0.00	down
0.00	0.00	up
0.00	0.00	up
0.00	0.00	up
0.00	0.00	down
0.00	0.00	up
0.00	0.00	up
0.00	0.00	down
0.00	0.00	up
0.00	0.00	up
0.00	0.00	down
0.00	0.00	up
0.00	0.00	up
0.00	0.00	up
0.00	0.00	down
0.00	0.00	up
0.00	0.00	down
0.00	0.00	up
0.00	0.00	up
0.00	0.00	down
0.00	0.00	down
0.00	0.00	up
0.00	0.00	up
0.00	0.00	up
0.00	0.00	up
0.00	0.00	up
0.00	0.00	up
0.00	0.00	up
0.00	0.00	down
0.00	0.00	down
0.00	0.00	up
0.00	0.00	down
0.00	0.00	up
0.00	0.00	down
0.00	0.00	up
0.00	0.00	up
0.00	0.00	up
0.00	0.00	down
0.00	0.00	up

0.00	0.00	down
0.00	0.00	down
0.00	0.00	up
0.00	0.00	down
0.00	0.00	up
0.00	0.00	up
0.00	0.00	down
0.00	0.00	up
0.00	0.00	up
0.00	0.00	up
0.00	0.00	down
0.00	0.00	up
0.00	0.00	down
0.00	0.00	down
0.00	0.00	up
0.00	0.00	up
0.00	0.00	up
0.00	0.00	up
0.00	0.00	up
0.00	0.00	down
0.00	0.00	down
0.00	0.00	down
0.00	0.00	down
0.00	0.00	up
0.00	0.00	up
0.00	0.00	up
0.00	0.00	up
0.00	0.00	down
0.00	0.00	down
0.00	0.00	down
0.00	0.00	down
0.00	0.00	up
0.00	0.00	up
0.00	0.00	down
0.00	0.00	down
0.00	0.00	up
0.00	0.00	up
0.00	0.00	down
0.00	0.00	up
0.00	0.00	up
0.00	0.00	up
0.00	0.00	up
0.00	0.00	down
0.00	0.00	up
0.00	0.00	down
0.00	0.00	up
0.00	0.00	down
0.00	0.00	down
0.00	0.00	up
0.00	0.00	up
0.00	0.00	up

0.00	0.00	up
0.00	0.00	up
0.00	0.00	down
0.00	0.00	down
0.00	0.00	down
0.00	0.00	up
0.00	0.00	down
0.00	0.00	down
0.00	0.00	up
0.00	0.00	up
0.00	0.00	down
0.00	0.00	down
0.00	0.00	down
0.00	0.00	up
0.00	0.00	down
0.00	0.00	down
0.00	0.00	up
0.00	0.00	up
0.00	0.00	up
0.00	0.00	up
0.00	0.00	up
0.00	0.00	up
0.00	0.00	down
0.00	0.00	down
0.00	0.00	down
0.00	0.00	up
0.00	0.00	down
0.00	0.00	up
0.00	0.00	down
0.00	0.00	down
0.00	0.00	up
0.00	0.00	down
0.00	0.00	up
0.00	0.00	down
0.00	0.00	up
0.00	0.00	up
0.00	0.00	up
0.00	0.00	down
0.00	0.00	down
0.00	0.00	up
0.00	0.00	up
0.00	0.00	up
0.00	0.00	down
0.00	0.00	down
0.00	0.00	up
0.00	0.00	up
0.00	0.00	up
0.00	0.00	down
0.00	0.00	down
0.00	0.00	down
0.00	0.00	up

0.00	0.00	down
0.00	0.00	down
0.00	0.00	up
0.00	0.00	up
0.00	0.00	up
0.00	0.00	down
0.00	0.00	down
0.00	0.00	up
0.00	0.00	up
0.00	0.00	down
0.00	0.00	down
0.00	0.00	down
0.00	0.00	up
0.00	0.00	down
0.00	0.00	down
0.00	0.00	down
0.00	0.00	up
0.00	0.00	up
0.00	0.00	up
0.00	0.00	up
0.00	0.00	down
0.00	0.00	down
0.00	0.00	up
0.00	0.00	up
0.00	0.00	down
0.00	0.00	down
0.00	0.00	down
0.00	0.00	down
0.00	0.00	down
0.00	0.00	down
0.00	0.00	up
0.00	0.00	down
0.00	0.00	up
0.00	0.00	up
0.00	0.00	up
0.00	0.00	up
0.00	0.00	down
0.00	0.00	down
0.00	0.00	up
0.00	0.00	up
0.00	0.00	down
0.00	0.00	up
0.00	0.00	down
0.00	0.00	down
0.00	0.00	down
0.00	0.00	up
0.00	0.00	up
0.00	0.00	down
0.00	0.00	down
0.00	0.00	down
0.00	0.00	down

0.00	0.00	up
0.00	0.00	down
0.00	0.00	up
0.00	0.00	down
0.00	0.00	up
0.00	0.00	down
0.00	0.00	up
0.00	0.00	up
0.00	0.00	up
0.00	0.00	up
0.00	0.00	down
0.00	0.00	up
0.00	0.00	up
0.00	0.00	up
0.00	0.00	down
0.00	0.00	up
0.00	0.00	down
0.00	0.00	up
0.00	0.00	down
0.00	0.00	up
0.00	0.00	down
0.00	0.00	up
0.00	0.00	up
0.00	0.00	down
0.00	0.00	up
0.00	0.00	up
0.00	0.00	up
0.00	0.00	down
0.00	0.00	down
0.00	0.00	down
0.00	0.00	down
0.00	0.00	up
0.00	0.00	up
0.00	0.00	up
0.00	0.00	up
0.00	0.00	up
0.00	0.00	down
0.00	0.00	up
0.00	0.00	up
0.00	0.00	up
0.00	0.00	up
0.00	0.00	up
0.00	0.00	down
0.00	0.00	down
0.00	0.00	down
0.00	0.00	up
0.00	0.00	up
0.00	0.00	up
0.00	0.00	down
0.00	0.00	up
0.00	0.00	down

0.00	0.00	up
0.00	0.00	up
0.00	0.00	up
0.00	0.00	up
0.00	0.00	down
0.00	0.00	down
0.00	0.00	down
0.00	0.00	up
0.00	0.00	up
0.00	0.00	up
0.00	0.00	up
0.00	0.00	up
0.00	0.00	down
0.00	0.00	up
0.00	0.00	up
0.00	0.00	up
0.00	0.00	up
0.00	0.00	down
0.00	0.00	down
0.00	0.00	up
0.00	0.00	up
0.00	0.00	down
0.00	0.00	up
0.00	0.00	down
0.00	0.00	down
0.00	0.00	down
0.00	0.00	up
0.00	0.00	up
0.00	0.00	down
0.00	0.00	up
0.00	0.00	down
0.00	0.00	down
0.00	0.00	down
0.00	0.00	down
0.00	0.00	down
0.00	0.00	down
0.00	0.00	up
0.00	0.00	up
0.00	0.00	down
0.00	0.00	down
0.00	0.00	up
0.00	0.00	down
0.00	0.00	down
0.00	0.00	up
0.00	0.00	down
0.00	0.00	up
0.00	0.00	down
0.00	0.00	up
0.00	0.00	up
0.00	0.00	up
0.00	0.00	down

0.00	0.00	up
0.00	0.00	down
0.00	0.00	down
0.00	0.00	up
0.00	0.00	up
0.00	0.00	down
0.00	0.00	down
0.00	0.00	up
0.00	0.00	down
0.00	0.00	down
0.00	0.00	down
0.00	0.00	down
0.00	0.00	up
0.00	0.00	up
0.00	0.00	up
0.00	0.00	up
0.00	0.00	down
0.00	0.00	down
0.00	0.00	up
0.00	0.00	down
0.00	0.00	up
0.00	0.00	down
0.00	0.00	down
0.00	0.00	up
0.00	0.00	up
0.00	0.00	up
0.00	0.00	down
0.00	0.00	up
0.00	0.00	up
0.00	0.00	up
0.00	0.00	down
0.00	0.00	up
0.00	0.00	down
0.00	0.00	up
0.00	0.00	down
0.00	0.00	down
0.00	0.00	down
0.00	0.00	down
0.00	0.00	up
0.00	0.00	down
0.00	0.00	down
0.00	0.00	up
0.00	0.00	up
0.00	0.00	up
0.00	0.00	up
0.00	0.00	up
0.00	0.00	down
0.00	0.00	up
0.00	0.00	down
0.00	0.00	up
0.00	0.00	down

0.00	0.00	up
0.00	0.00	up
0.00	0.00	up
0.00	0.00	up
0.00	0.00	up
0.00	0.00	down
0.00	0.00	down
0.00	0.00	up
0.00	0.00	up
0.00	0.00	up
0.00	0.00	down
0.00	0.00	down
0.00	0.00	down
0.00	0.00	down
0.00	0.00	down
0.00	0.00	down
0.00	0.00	up
0.00	0.00	up
0.00	0.00	down
0.00	0.00	up
0.00	0.00	up
0.00	0.00	down
0.00	0.00	up
0.00	0.00	up
0.00	0.00	up
0.00	0.00	up
0.00	0.00	up
0.00	0.00	up
0.00	0.00	down
0.00	0.00	down
0.00	0.00	up
0.00	0.00	up
0.00	0.00	up
0.00	0.00	down
0.00	0.00	up
0.00	0.00	up
0.00	0.00	up
0.00	0.00	up
0.00	0.00	up
0.00	0.00	up
0.00	0.00	down
0.00	0.00	up
0.00	0.00	up
0.00	0.00	up
0.00	0.00	up
0.00	0.00	up
0.00	0.00	up
0.00	0.00	down
0.00	0.00	down
0.00	0.00	down
0.00	0.00	down

0.00	0.00	up
0.00	0.00	up
0.00	0.00	up
0.00	0.00	up
0.00	0.00	down
0.00	0.00	up
0.00	0.00	down
0.00	0.00	up
0.00	0.00	down
0.00	0.00	up
0.00	0.00	down
0.00	0.00	up
0.00	0.00	up
0.00	0.00	down
0.00	0.00	down
0.00	0.00	down
0.00	0.00	up
0.00	0.00	up
0.00	0.00	down
0.00	0.00	down
0.00	0.00	up
0.00	0.00	up
0.00	0.00	up
0.00	0.00	down
0.00	0.00	up
0.00	0.00	down
0.00	0.00	down
0.00	0.00	down
0.00	0.00	down
0.00	0.00	down
0.00	0.00	up
0.00	0.00	up
0.00	0.00	up
0.00	0.00	up
0.00	0.00	up
0.00	0.00	up
0.00	0.00	up
0.00	0.00	down
0.00	0.00	up
0.00	0.00	down
0.00	0.00	down
0.00	0.00	up
0.00	0.00	up
0.00	0.00	up
0.00	0.00	down
0.00	0.00	up
0.00	0.00	down
0.00	0.00	up
0.00	0.00	up
0.00	0.00	down
0.00	0.00	up

0.00	0.00	up
0.00	0.00	up
0.00	0.00	up
0.00	0.00	up
0.00	0.00	up
0.00	0.00	up
0.00	0.00	up
0.00	0.00	up
0.00	0.00	up
0.00	0.00	up
0.00	0.00	up
0.00	0.00	down
0.00	0.00	up
0.00	0.00	up
0.00	0.00	up
0.00	0.00	down
0.00	0.00	up
0.00	0.00	down
0.00	0.00	down
0.00	0.00	down
0.00	0.00	up
0.00	0.00	down
0.00	0.00	down
0.00	0.00	down
0.00	0.00	up
0.00	0.00	down
0.00	0.00	up
0.00	0.00	up
0.00	0.00	down
0.00	0.00	down
0.00	0.00	up
0.00	0.00	up
0.00	0.00	down
0.00	0.00	down
0.00	0.00	down
0.00	0.00	up
0.00	0.00	down
0.00	0.00	up
0.00	0.00	up
0.00	0.00	up
0.00	0.00	up
0.00	0.00	up
0.00	0.00	up
0.00	0.00	up
0.00	0.00	down
0.00	0.00	down
0.00	0.00	up
0.00	0.00	down
0.00	0.00	down
0.00	0.00	up
0.00	0.00	up

0.00	0.00	up
0.00	0.00	up
0.00	0.00	down
0.00	0.00	up
0.00	0.00	up
0.00	0.00	up
0.00	0.00	up
0.00	0.00	up
0.00	0.00	up
0.00	0.00	up
0.00	0.00	down
0.00	0.00	up
0.00	0.00	up
0.00	0.00	up
0.00	0.00	up
0.00	0.00	up
0.00	0.00	up
0.00	0.00	down
0.00	0.00	up
0.00	0.00	up
0.00	0.00	up
0.00	0.00	up
0.00	0.00	down
0.00	0.00	down
0.00	0.00	up
0.00	0.00	up
0.00	0.00	down
0.00	0.00	down
0.00	0.00	up
0.00	0.00	up
0.00	0.00	up
0.00	0.00	down
0.00	0.00	up
0.00	0.00	down
0.00	0.00	up
0.00	0.00	up
0.00	0.00	up
0.00	0.00	down
0.00	0.00	down
0.00	0.00	down
0.00	0.00	up
0.00	0.00	down
0.00	0.00	up
0.00	0.00	up
0.00	0.00	down
0.00	0.00	up
0.00	0.00	down
0.00	0.00	down
0.00	0.00	up
0.00	0.00	up
0.00	0.00	down

0.00	0.00	up
0.00	0.00	down
0.00	0.00	up
0.00	0.00	up
0.00	0.00	up
0.00	0.00	down
0.00	0.00	up
0.00	0.00	down
0.00	0.00	down
0.00	0.00	up
0.00	0.00	down
0.00	0.00	down
0.00	0.00	down
0.00	0.00	up
0.00	0.00	down
0.00	0.00	up
0.00	0.00	up
0.00	0.00	down
0.00	0.00	up
0.00	0.00	up
0.00	0.00	up
0.00	0.00	up
0.00	0.00	down
0.00	0.00	up
0.00	0.00	up
0.00	0.00	down
0.00	0.00	up
0.00	0.00	up
0.00	0.00	up
0.00	0.00	up
0.00	0.00	up
0.00	0.00	down
0.00	0.00	up
0.00	0.00	down
0.00	0.00	down
0.00	0.00	up
0.00	0.00	up
0.00	0.00	up
0.00	0.00	up
0.00	0.00	down
0.00	0.00	down
0.00	0.00	down
0.00	0.00	up
0.00	0.00	up
0.00	0.00	up
0.00	0.00	up
0.00	0.00	up
0.00	0.00	up
0.00	0.00	up
0.00	0.00	down
0.00	0.00	up

0.00	0.00	up
0.00	0.00	up
0.00	0.00	up
0.00	0.00	down
0.00	0.00	down
0.00	0.00	up
0.00	0.00	up
0.00	0.00	down
0.00	0.00	up
0.00	0.00	up
0.00	0.00	up
0.00	0.00	down
0.00	0.00	down
0.00	0.00	up
0.00	0.00	up
0.00	0.00	up
0.00	0.00	up
0.00	0.00	up
0.00	0.00	down
0.00	0.00	down
0.00	0.00	up
0.00	0.00	down
0.00	0.00	down
0.00	0.00	up
0.00	0.00	down
0.00	0.00	up
0.00	0.00	down
0.00	0.00	up
0.00	0.00	up
0.00	0.00	down
0.00	0.00	down
0.00	0.00	up
0.00	0.00	down
0.00	0.00	up
0.00	0.00	down
0.00	0.00	down
0.00	0.00	up
0.00	0.00	up
0.00	0.00	up
0.00	0.00	down
0.00	0.00	up
0.00	0.00	up
0.00	0.00	down
0.00	0.00	down
0.00	0.00	down
0.00	0.00	down
0.00	0.00	up
0.00	0.00	up
0.00	0.00	up
0.00	0.00	down
0.00	0.00	up

0.00	0.00	down
0.00	0.00	up
0.00	0.00	down
0.00	0.00	up
0.00	0.00	down
0.00	0.00	up
0.00	0.00	up
0.00	0.00	up
0.00	0.00	down
0.00	0.00	down
0.00	0.00	up
0.00	0.00	up
0.00	0.00	up
0.00	0.00	down
0.00	0.00	up
0.00	0.00	up
0.00	0.00	up
0.00	0.00	up
0.00	0.00	up
0.00	0.00	up
0.00	0.00	down
0.00	0.00	down
0.00	0.00	down
0.00	0.00	down
0.00	0.00	up
0.00	0.00	up
0.00	0.00	up
0.00	0.00	down
0.00	0.00	up
0.00	0.00	up
0.00	0.00	up
0.00	0.00	up
0.00	0.00	down
0.00	0.00	down
0.00	0.00	up
0.00	0.00	down
0.00	0.00	down
0.00	0.00	down
0.00	0.00	down
0.00	0.00	up
0.00	0.00	down
0.00	0.00	up
0.00	0.00	up
0.00	0.00	up
0.00	0.00	up
0.00	0.00	down
0.00	0.00	up
0.00	0.00	up
0.00	0.00	up
0.00	0.00	up
0.00	0.00	up

0.00	0.00	up
0.00	0.00	up
0.00	0.00	down
0.00	0.00	down
0.00	0.00	up
0.00	0.00	up
0.00	0.00	up
0.00	0.00	down
0.00	0.00	down
0.00	0.00	down
0.00	0.00	down
0.00	0.00	up
0.00	0.00	down
0.00	0.00	down
0.00	0.00	down
0.00	0.00	down
0.00	0.00	up
0.00	0.00	up
0.00	0.00	down
0.00	0.00	down
0.00	0.00	up
0.00	0.00	up
0.00	0.00	down
0.00	0.00	down
0.00	0.00	up
0.00	0.00	down
0.00	0.00	down
0.00	0.00	up
0.00	0.00	up
0.00	0.00	down
0.00	0.00	up
0.00	0.00	down
0.00	0.00	up
0.00	0.00	down
0.00	0.00	down
0.00	0.00	down
0.00	0.00	up
0.00	0.00	down
0.00	0.00	up
0.00	0.00	down
0.00	0.00	down
0.00	0.00	up
0.00	0.00	up
0.00	0.00	up
0.00	0.00	up
0.00	0.00	up
0.00	0.00	down
0.00	0.00	up
0.00	0.00	up
0.00	0.00	up
0.00	0.00	up

0.00	0.00	up
0.00	0.00	down
0.00	0.00	up
0.00	0.00	down
0.00	0.00	up
0.00	0.00	down
0.00	0.00	up
0.00	0.00	down
0.00	0.00	up
0.00	0.00	down
0.00	0.00	up
0.00	0.00	up
0.00	0.00	up
0.00	0.00	down
0.00	0.00	up
0.00	0.00	up
0.00	0.00	up
0.00	0.00	up
0.00	0.00	down
0.00	0.00	up
0.00	0.00	up
0.00	0.00	up
0.00	0.00	down
0.00	0.00	down
0.00	0.00	up
0.00	0.00	down
0.00	0.00	down
0.00	0.00	down
0.00	0.00	down
0.00	0.00	down
0.00	0.00	up
0.00	0.00	up
0.00	0.00	down
0.00	0.00	down
0.00	0.00	down
0.00	0.00	up
0.00	0.00	up
0.00	0.00	up
0.00	0.00	down
0.00	0.00	down
0.00	0.00	up
0.00	0.00	up
0.00	0.00	up
0.00	0.00	up
0.00	0.00	down
0.00	0.00	down
0.00	0.00	up
0.00	0.00	down
0.00	0.00	down
0.00	0.00	down
0.00	0.00	down

0.00	0.00	up
0.00	0.00	down
0.00	0.00	up
0.00	0.00	up
0.00	0.00	up
0.00	0.00	up
0.00	0.00	down
0.00	0.00	up
0.00	0.00	down
0.00	0.00	down
0.00	0.00	down
0.00	0.00	down
0.00	0.00	down
0.00	0.00	down
0.00	0.00	up
0.00	0.00	up
0.00	0.00	down
0.00	0.00	up
0.00	0.00	down
0.00	0.00	up
0.00	0.00	up
0.00	0.00	up
0.00	0.00	up
0.00	0.00	up
0.00	0.00	down
0.00	0.00	down
0.00	0.00	up
0.00	0.00	up
0.00	0.00	up
0.00	0.00	up
0.00	0.00	up
0.00	0.00	up
0.00	0.00	up
0.00	0.00	down
0.00	0.00	down
0.00	0.00	up
0.00	0.00	down
0.00	0.00	up
0.00	0.00	up
0.00	0.00	down
0.00	0.00	up
0.00	0.00	down
0.00	0.00	up
0.00	0.00	down
0.00	0.00	up
0.00	0.00	up
0.00	0.00	up
0.00	0.00	up
0.00	0.00	down
0.00	0.00	up
0.00	0.00	down

0.00	0.00	up
0.00	0.00	down
0.00	0.00	down
0.00	0.00	up
0.00	0.00	up
0.00	0.00	up
0.00	0.00	down
0.00	0.00	down
0.00	0.00	up
0.00	0.00	up
0.00	0.00	up
0.00	0.00	down
0.00	0.00	up
0.00	0.00	up
0.00	0.00	down
0.00	0.00	up
0.00	0.00	down
0.00	0.00	down
0.00	0.00	down
0.00	0.00	down
0.00	0.00	up
0.00	0.00	up
0.00	0.00	up
0.00	0.00	up
0.00	0.00	up
0.00	0.00	up
0.00	0.00	down
0.00	0.00	up
0.00	0.00	up
0.00	0.00	up
0.00	0.00	up
0.00	0.00	up
0.00	0.00	down
0.00	0.00	up
0.00	0.00	up
0.00	0.00	up
0.00	0.00	down
0.00	0.00	up
0.00	0.00	up
0.00	0.00	down
0.00	0.00	up
0.00	0.00	down
0.00	0.00	down
0.00	0.00	down
0.00	0.00	down
0.00	0.00	down
0.00	0.00	up
0.00	0.00	down
0.00	0.00	up
0.00	0.00	down
0.00	0.00	down

0.00	0.00	up
0.00	0.00	up
0.00	0.00	down
0.00	0.00	up
0.00	0.00	up
0.00	0.00	up
0.00	0.00	up
0.00	0.00	up
0.00	0.00	down
0.00	0.00	up
0.00	0.00	up
0.00	0.00	down
0.00	0.00	up
0.00	0.00	up
0.00	0.00	up
0.00	0.00	down
0.00	0.00	up
0.00	0.00	down
0.00	0.00	up
0.00	0.00	up
0.00	0.00	up
0.00	0.00	down
0.00	0.00	up
0.00	0.00	up
0.00	0.00	down
0.00	0.00	down
0.00	0.00	down
0.00	0.00	down
0.00	0.00	down
0.00	0.00	down
0.00	0.00	down
0.00	0.00	up
0.00	0.00	down
0.00	0.00	up
0.00	0.00	up
0.00	0.00	up
0.00	0.00	up
0.00	0.00	down
0.00	0.00	up
0.00	0.00	down
0.00	0.00	down
0.00	0.00	down
0.00	0.00	up
0.00	0.00	up
0.00	0.00	up
0.00	0.00	up
0.00	0.00	down
0.00	0.00	up
0.00	0.00	up
0.00	0.00	down
0.00	0.00	up

0.00	0.00	up
0.00	0.00	down
0.00	0.00	down
0.00	0.00	down
0.00	0.00	down
0.00	0.00	down
0.00	0.00	up
0.00	0.00	down
0.00	0.00	down
0.00	0.00	up
0.00	0.00	down
0.00	0.00	up
0.00	0.00	up
0.00	0.00	up
0.00	0.00	up
0.00	0.00	down
0.00	0.00	up
0.00	0.00	up
0.00	0.00	down
0.00	0.00	down
0.00	0.00	down
0.00	0.00	down
0.00	0.00	down
0.00	0.00	down
0.00	0.00	up
0.00	0.00	up
0.00	0.00	up
0.00	0.00	down
0.00	0.00	down
0.00	0.00	down
0.00	0.00	down
0.00	0.00	down
0.00	0.00	up
0.00	0.00	down
0.00	0.00	up
0.00	0.00	up
0.00	0.00	up
0.00	0.00	down
0.00	0.00	down
0.00	0.00	down
0.00	0.00	up
0.00	0.00	up
0.00	0.00	up
0.00	0.00	down
0.00	0.00	up
0.00	0.00	up
0.00	0.00	up
0.00	0.00	down
0.00	0.00	up
0.00	0.00	down
0.00	0.00	down

0.00	0.00	up
0.00	0.00	up
0.00	0.00	down
0.00	0.00	down
0.00	0.00	up
0.00	0.00	down
0.00	0.00	down
0.00	0.00	down
0.00	0.00	up
0.00	0.00	down
0.00	0.00	up
0.00	0.00	down
0.00	0.00	down
0.00	0.00	up
0.00	0.00	down
0.00	0.00	up
0.00	0.00	down
0.00	0.00	up
0.00	0.00	up
0.00	0.00	down
0.00	0.00	up
0.00	0.00	up
0.00	0.00	down
0.00	0.00	up
0.00	0.00	down
0.00	0.00	up
0.00	0.00	up
0.00	0.00	down
0.00	0.00	up
0.00	0.00	down
0.00	0.00	up
0.00	0.00	up
0.00	0.00	down
0.00	0.00	down
0.00	0.00	down
0.00	0.00	up
0.00	0.00	up
0.00	0.00	up
0.00	0.00	down
0.00	0.00	up
0.00	0.00	up
0.00	0.00	down
0.00	0.00	up
0.00	0.00	up
0.00	0.00	up
0.00	0.00	up
0.00	0.00	down
0.00	0.00	up
0.00	0.00	up
0.00	0.00	up
0.00	0.00	up

0.00	0.00	up
0.00	0.00	down
0.00	0.00	down
0.00	0.00	up
0.00	0.00	down
0.00	0.00	down
0.00	0.00	up
0.00	0.00	up
0.00	0.00	down
0.00	0.00	down
0.00	0.00	up
0.00	0.00	down
0.00	0.00	up
0.00	0.00	up
0.00	0.00	up
0.00	0.00	up
0.00	0.00	up
0.00	0.00	up
0.00	0.00	up
0.00	0.00	up
0.00	0.00	down
0.00	0.00	up
0.00	0.00	up
0.00	0.00	down
0.00	0.00	up
0.00	0.00	down
0.00	0.00	up
0.00	0.00	down
0.00	0.00	up
0.00	0.00	down
0.00	0.00	up
0.00	0.00	up
0.00	0.00	down
0.00	0.00	up
0.00	0.00	up
0.00	0.00	up
0.00	0.00	down
0.00	0.00	up
0.00	0.00	up
0.00	0.00	down
0.00	0.00	up
0.00	0.00	down
0.00	0.00	up
0.00	0.00	down
0.00	0.00	down
0.00	0.00	down
0.00	0.00	down
0.00	0.00	up
0.00	0.00	down
0.00	0.00	up
0.00	0.00	up

0.00	0.00	up
0.00	0.00	up
0.00	0.00	up
0.00	0.00	up
0.00	0.00	down
0.00	0.00	down
0.00	0.00	up
0.00	0.00	up
0.00	0.00	down
0.00	0.00	down
0.00	0.00	down
0.00	0.00	up
0.00	0.00	up
0.00	0.00	down
0.00	0.00	up
0.00	0.00	down
0.00	0.00	down
0.00	0.00	up
0.00	0.00	down
0.00	0.00	up
0.00	0.00	down
0.00	0.00	up
0.00	0.00	up
0.00	0.00	up
0.00	0.00	up
0.00	0.00	up
0.00	0.00	up
0.00	0.00	up
0.00	0.00	up
0.00	0.00	down
0.00	0.00	up
0.00	0.00	up
0.00	0.00	down
0.00	0.00	up
0.00	0.00	down
0.00	0.00	down
0.00	0.00	down
0.00	0.00	up
0.00	0.00	down
0.00	0.00	up
0.00	0.00	down
0.00	0.00	down
0.00	0.00	up
0.00	0.00	up
0.00	0.00	up
0.00	0.00	down
0.00	0.00	down
0.00	0.00	up
0.00	0.00	up
0.00	0.00	up
0.00	0.00	up

0.00	0.00	up
0.00	0.00	up
0.00	0.00	up
0.00	0.00	up
0.00	0.00	down
0.00	0.00	up
0.00	0.00	up
0.00	0.00	down
0.00	0.00	down
0.00	0.00	down
0.00	0.00	up
0.00	0.00	up
0.00	0.00	down
0.00	0.00	down
0.00	0.00	up
0.00	0.00	down
0.00	0.00	up
0.00	0.00	up
0.00	0.00	up
0.00	0.00	up
0.00	0.00	up
0.00	0.00	up
0.00	0.00	up
0.00	0.00	down
0.00	0.00	up
0.00	0.00	up
0.00	0.00	up
0.00	0.00	up
0.00	0.00	down
0.00	0.00	down
0.00	0.00	down
0.00	0.00	up
0.00	0.00	down
0.00	0.00	up
0.00	0.00	down
0.00	0.00	down
0.00	0.00	up
0.00	0.00	up
0.00	0.00	up
0.00	0.00	up
0.00	0.00	up
0.00	0.00	up
0.00	0.00	up
0.00	0.00	up
0.00	0.00	down
0.00	0.00	up
0.00	0.00	up
0.00	0.00	up
0.00	0.00	down
0.00	0.00	down
0.00	0.00	down

0.00	0.00	up
0.00	0.00	up
0.00	0.00	down
0.00	0.00	up
0.00	0.00	down
0.00	0.00	down
0.00	0.00	down
0.00	0.00	down
0.00	0.00	down
0.00	0.00	down
0.00	0.00	down
0.00	0.00	up
0.00	0.00	up
0.00	0.00	up
0.00	0.00	down
0.00	0.00	down
0.00	0.00	up
0.00	0.00	up
0.00	0.00	up
0.00	0.00	up
0.00	0.00	down
0.00	0.00	up
0.00	0.00	up
0.00	0.00	down
0.00	0.00	up
0.00	0.00	down
0.00	0.00	up
0.00	0.00	up
0.00	0.00	down
0.00	0.00	up
0.00	0.00	up
0.00	0.00	up
0.00	0.00	up
0.00	0.00	down
0.00	0.00	up
0.00	0.00	up
0.00	0.00	down
0.00	0.00	down
0.00	0.00	down
0.00	0.00	down
0.00	0.00	up
0.00	0.00	down
0.00	0.00	up
0.00	0.00	down
0.00	0.00	down
0.00	0.00	up
0.00	0.00	down
0.00	0.00	down
0.00	0.00	down
0.00	0.00	down
0.00	0.00	down

0.00	0.00	down
0.00	0.00	up
0.00	0.00	down
0.00	0.00	down
0.00	0.00	up
0.00	0.00	down
0.00	0.00	up
0.00	0.00	down
0.00	0.00	up
0.00	0.00	up
0.00	0.00	down
0.00	0.00	up
0.00	0.00	up
0.00	0.00	down
0.00	0.00	up
0.00	0.00	down
0.00	0.00	up
0.00	0.00	down
0.00	0.00	up
0.00	0.00	up
0.00	0.00	down
0.00	0.00	up
0.00	0.00	up
0.00	0.00	up
0.00	0.00	up
0.00	0.00	down
0.00	0.00	down
0.00	0.00	down
0.00	0.00	down
0.00	0.00	up
0.00	0.00	up
0.00	0.00	up
0.00	0.00	up
0.00	0.00	up
0.00	0.00	up
0.00	0.00	down
0.00	0.00	down
0.00	0.00	up
0.00	0.00	down
0.00	0.00	up
0.00	0.00	down
0.00	0.00	down
0.00	0.00	up
0.00	0.00	down
0.00	0.00	up
0.00	0.00	up
0.00	0.00	down
0.00	0.00	down
0.00	0.00	down
0.00	0.00	down
0.00	0.00	down

0.00	0.00	down
0.00	0.00	up
0.00	0.00	up
0.00	0.00	up
0.00	0.00	down
0.00	0.00	up
0.00	0.00	up
0.00	0.00	up
0.00	0.00	up
0.00	0.00	up
0.00	0.00	up
0.00	0.00	up
0.00	0.00	down
0.00	0.00	up
0.00	0.00	down
0.00	0.00	up
0.00	0.00	up
0.00	0.00	down
0.00	0.00	up
0.00	0.00	up
0.00	0.00	down
0.00	0.00	down
0.00	0.00	down
0.00	0.00	down
0.00	0.00	up
0.00	0.00	up
0.00	0.00	down
0.00	0.00	up
0.00	0.00	up
0.00	0.00	down
0.00	0.00	up
0.00	0.00	up
0.00	0.00	down
0.00	0.00	up
0.00	0.00	up
0.00	0.00	up
0.00	0.00	down
0.00	0.00	up
0.00	0.00	down
0.00	0.00	up
0.00	0.00	up
0.00	0.00	up
0.00	0.00	down
0.00	0.00	down
0.00	0.00	down
0.00	0.00	up
0.00	0.00	down
0.00	0.00	down
0.00	0.00	down
0.00	0.00	down
0.00	0.00	up

0.00	0.00	up
0.00	0.00	down
0.00	0.00	down
0.00	0.00	down
0.00	0.00	up
0.00	0.00	up
0.00	0.00	up
0.00	0.00	down
0.00	0.00	up
0.00	0.00	down
0.00	0.00	up
0.00	0.00	up
0.00	0.00	up
0.00	0.00	up
0.00	0.00	up
0.00	0.00	down
0.00	0.00	up
0.00	0.00	down
0.00	0.00	down
0.00	0.00	up
0.00	0.00	up
0.00	0.00	up
0.00	0.00	down
0.00	0.00	up
0.00	0.00	up
0.00	0.00	down
0.00	0.00	down
0.00	0.00	down
0.00	0.00	down
0.00	0.00	up
0.00	0.00	up
0.00	0.00	down
0.00	0.00	up
0.00	0.00	up
0.00	0.00	down
0.00	0.00	up
0.00	0.00	up
0.00	0.00	up
0.00	0.00	down
0.00	0.00	down
0.00	0.00	up
0.00	0.00	up
0.00	0.00	down
0.00	0.00	down
0.00	0.00	up
0.00	0.00	up
0.00	0.00	down
0.00	0.00	down
0.00	0.00	down
0.00	0.00	up
0.00	0.00	up

0.00	0.01	up
0.00	0.01	up
0.00	0.01	up
0.00	0.01	down
0.00	0.01	down
0.00	0.01	up
0.00	0.01	up
0.00	0.01	up
0.00	0.01	down
0.00	0.01	down
0.00	0.01	down
0.00	0.01	down
0.00	0.01	up
0.00	0.01	down
0.00	0.01	down
0.00	0.01	down
0.00	0.01	up
0.00	0.01	down
0.00	0.01	up
0.00	0.01	down
0.00	0.01	down
0.00	0.01	up
0.00	0.01	down
0.00	0.01	down
0.00	0.01	down
0.00	0.01	up
0.00	0.01	up
0.00	0.01	up
0.00	0.01	up
0.00	0.01	up
0.00	0.01	up
0.00	0.01	down
0.00	0.01	up
0.00	0.01	down
0.00	0.01	up
0.00	0.01	down
0.00	0.01	down
0.00	0.01	up
0.00	0.01	up
0.00	0.01	down
0.00	0.01	down
0.00	0.01	up
0.00	0.01	down
0.00	0.01	down
0.00	0.01	up
0.00	0.01	up
0.00	0.01	down
0.00	0.01	down
0.00	0.01	down
0.00	0.01	up
0.00	0.01	down

0.00	0.01	up
0.00	0.01	down
0.00	0.01	down
0.00	0.01	up
0.00	0.01	down
0.00	0.01	down
0.00	0.01	up
0.00	0.01	down
0.00	0.01	up
0.00	0.01	up
0.00	0.01	down
0.00	0.01	up
0.00	0.01	down
0.00	0.01	down
0.00	0.01	up
0.00	0.01	up
0.00	0.01	up
0.00	0.01	down
0.00	0.01	down
0.00	0.01	down
0.00	0.01	up
0.00	0.01	up
0.00	0.01	down
0.00	0.01	up
0.00	0.01	down
0.00	0.01	down
0.00	0.01	up
0.00	0.01	down
0.00	0.01	down
0.00	0.01	down
0.00	0.01	up
0.00	0.01	down
0.00	0.01	up
0.00	0.01	up
0.00	0.01	down
0.00	0.01	up
0.00	0.01	down
0.00	0.01	up
0.00	0.01	up
0.00	0.01	down
0.00	0.01	up
0.00	0.01	down
0.00	0.01	up
0.00	0.01	up
0.00	0.01	down
0.00	0.01	up
0.00	0.01	up
0.00	0.01	down
0.00	0.01	up
0.00	0.01	up
0.00	0.01	up

0.00	0.01	down
0.00	0.01	down
0.00	0.01	up
0.00	0.01	down
0.00	0.01	up
0.00	0.01	up
0.00	0.01	up
0.00	0.01	up
0.00	0.01	down
0.00	0.01	down
0.00	0.01	down
0.00	0.01	down
0.00	0.01	down
0.00	0.01	down
0.00	0.01	up
0.00	0.01	up
0.00	0.01	down
0.00	0.01	up
0.00	0.01	up
0.00	0.01	up
0.00	0.01	down
0.00	0.01	up
0.00	0.01	up
0.00	0.01	up
0.00	0.01	down
0.00	0.01	down
0.00	0.01	down
0.00	0.01	up
0.00	0.01	up
0.00	0.01	down
0.00	0.01	down
0.00	0.01	up
0.00	0.01	up
0.00	0.01	up
0.00	0.01	up
0.00	0.01	down
0.00	0.01	down
0.00	0.01	up
0.00	0.01	down
0.00	0.01	up
0.00	0.01	up
0.00	0.01	up
0.00	0.01	down
0.00	0.01	down
0.00	0.01	up
0.00	0.01	down
0.00	0.01	up
0.00	0.01	down
0.00	0.01	down
0.00	0.01	down
0.00	0.01	down

0.00	0.01	down
0.00	0.01	down
0.00	0.01	down
0.00	0.01	down
0.00	0.01	up
0.00	0.01	up
0.00	0.01	down
0.00	0.01	up
0.00	0.01	up
0.00	0.01	up
0.00	0.01	down
0.00	0.01	down
0.00	0.01	up
0.00	0.01	down
0.00	0.01	up
0.00	0.01	up
0.00	0.01	down
0.00	0.01	down
0.00	0.01	up
0.00	0.01	up
0.00	0.01	up
0.00	0.01	down
0.00	0.01	down
0.00	0.01	down
0.00	0.01	down
0.00	0.01	down
0.00	0.01	down
0.00	0.01	down
0.00	0.01	down
0.00	0.01	down
0.00	0.01	down
0.00	0.01	down
0.00	0.01	up
0.00	0.01	down
0.00	0.01	up
0.00	0.02	down
0.00	0.02	down
0.00	0.02	down
0.00	0.02	up
0.00	0.02	down
0.00	0.02	down
0.00	0.02	down
0.00	0.02	down
0.00	0.02	up
0.00	0.02	down
0.00	0.02	up
0.01	0.02	down
0.01	0.02	up
0.01	0.02	down
0.01	0.02	up
0.01	0.02	down

0.01	0.02	down
0.01	0.02	up
0.01	0.02	down
0.01	0.02	up
0.01	0.02	up
0.01	0.02	down
0.01	0.02	up
0.01	0.02	down
0.01	0.02	down
0.01	0.02	up
0.01	0.02	up
0.01	0.02	up
0.01	0.02	up
0.01	0.02	up
0.01	0.02	up
0.01	0.02	down
0.01	0.02	down
0.01	0.02	down
0.01	0.02	down
0.01	0.02	up
0.01	0.02	up
0.01	0.02	up
0.01	0.02	up
0.01	0.02	up
0.01	0.02	down
0.01	0.02	up
0.01	0.02	down
0.01	0.02	down
0.01	0.02	up
0.01	0.02	down
0.01	0.02	down
0.01	0.02	up
0.01	0.02	up
0.01	0.02	down
0.01	0.02	up
0.01	0.02	down
0.01	0.02	down
0.01	0.02	down
0.01	0.02	up
0.01	0.02	up
0.01	0.02	up
0.01	0.02	up
0.01	0.02	up
0.01	0.02	down
0.01	0.02	down
0.01	0.02	down
0.01	0.02	down
0.01	0.02	down
0.01	0.02	down
0.01	0.02	up
0.01	0.02	down

0.01	0.02	up
0.01	0.02	up
0.01	0.02	down
0.01	0.02	down
0.01	0.02	up
0.01	0.02	up
0.01	0.02	down
0.01	0.02	down
0.01	0.02	up
0.01	0.02	up
0.01	0.02	up
0.01	0.02	up
0.01	0.02	down
0.01	0.02	up
0.01	0.02	down
0.01	0.02	down
0.01	0.02	down
0.01	0.02	down
0.01	0.02	down
0.01	0.02	down
0.01	0.02	up
0.01	0.02	up
0.01	0.02	down
0.01	0.02	up
0.01	0.02	up
0.01	0.02	up
0.01	0.02	down
0.01	0.02	down
0.01	0.02	down
0.01	0.02	up
0.01	0.02	up
0.01	0.02	down
0.01	0.02	down
0.01	0.02	down
0.01	0.02	up
0.01	0.02	up
0.01	0.03	down
0.01	0.03	down
0.01	0.03	down
0.01	0.03	up
0.01	0.03	up
0.01	0.03	up
0.01	0.03	up
0.01	0.03	up
0.01	0.03	up
0.01	0.03	down
0.01	0.03	up
0.01	0.03	up
0.01	0.03	down
0.01	0.03	down
0.01	0.03	up

0.01	0.03	up
0.01	0.03	down
0.01	0.03	down
0.01	0.03	down
0.01	0.03	up
0.01	0.03	up
0.01	0.03	up
0.01	0.03	up
0.01	0.03	down
0.01	0.03	down
0.01	0.03	down
0.01	0.03	up
0.01	0.03	down
0.01	0.03	up
0.01	0.03	up
0.01	0.03	down
0.01	0.03	up
0.01	0.03	down
0.01	0.03	up
0.01	0.03	down
0.01	0.03	up
0.01	0.03	down
0.01	0.03	down
0.01	0.03	up
0.01	0.03	down
0.01	0.03	down
0.01	0.03	down
0.01	0.03	up
0.01	0.03	up
0.01	0.03	up
0.01	0.03	down
0.01	0.03	down
0.01	0.03	up
0.01	0.03	down
0.01	0.03	up
0.01	0.03	up
0.01	0.03	up
0.01	0.03	down
0.01	0.03	down
0.01	0.03	up
0.01	0.03	up
0.01	0.03	up
0.01	0.03	up
0.01	0.03	down
0.01	0.03	up
0.01	0.03	down
0.01	0.03	down
0.01	0.03	up
0.01	0.03	up
0.01	0.03	down
0.01	0.03	up

0.01	0.03	up
0.01	0.03	up
0.01	0.03	up
0.01	0.03	down
0.01	0.03	down
0.01	0.03	up
0.01	0.03	up
0.01	0.03	down
0.01	0.04	down
0.01	0.04	up
0.01	0.04	down
0.01	0.04	down
0.01	0.04	down
0.01	0.04	down
0.01	0.04	up
0.01	0.04	down
0.01	0.04	up
0.01	0.04	down
0.01	0.04	down
0.01	0.04	up
0.01	0.04	up
0.01	0.04	down
0.01	0.04	down
0.01	0.04	down
0.01	0.04	down
0.01	0.04	down
0.01	0.04	up
0.01	0.04	up
0.01	0.04	up
0.01	0.04	up
0.01	0.04	up
0.01	0.04	up
0.01	0.04	up
0.01	0.04	up
0.01	0.04	down
0.02	0.04	up
0.02	0.04	up
0.02	0.04	up
0.02	0.04	up
0.02	0.04	up
0.02	0.04	up
0.02	0.04	up
0.02	0.04	down
0.02	0.04	up
0.02	0.04	down
0.02	0.04	up
0.02	0.04	up
0.02	0.04	down
0.02	0.04	down
0.02	0.04	up
0.02	0.04	up

0.02	0.04	down
0.02	0.04	up
0.02	0.04	up
0.02	0.04	up
0.02	0.04	down
0.02	0.05	down
0.02	0.05	down
0.02	0.05	down
0.02	0.05	up
0.02	0.05	up
0.02	0.05	down
0.02	0.05	up
0.02	0.05	down
0.02	0.05	down
0.02	0.05	down
0.02	0.05	down
0.02	0.05	down
0.02	0.05	down
0.02	0.05	down
0.02	0.05	up
0.02	0.05	down
0.02	0.05	down
0.02	0.05	up
0.02	0.05	up
0.02	0.05	up
0.02	0.05	up
0.02	0.05	down
0.02	0.05	up
0.02	0.05	down
0.02	0.05	down
0.02	0.05	up
0.02	0.05	up
0.02	0.05	down
0.02	0.05	up
0.02	0.05	up
0.02	0.05	down
0.02	0.05	down
0.02	0.05	down
