# Supplementary material for: Transcriptome reveals differential expression of flavor and color in closely related strains of tomato (Solanum lycopersicum)
Source: PeerJ. 2025 Oct 7;13:e20113. doi: 10.7717/peerj.20113 (PMC12513376; doi:10.7717/peerj.20113)
Supplement: Supplemental Information 7 [file peerj-13-20113-s007.pdf]

Table S5: Analysis of GO enrichment for DEGs in Br19-vs-Br20

GO_ID	GO_Term	GO_Categ	P.value	Q.value	gene_id	gene_nam	fc	log2(fc)
GO:00055	iron ion bi	Molecular	0.00	0.00	Solyc11g0	AOS2	0.12	-3.01
GO:00055	iron ion bi	Molecular	0.00	0.00	Solyc03g0	Solyc03g0	2.69	1.43
GO:00055	iron ion bi	Molecular	0.00	0.00	Solyc03g1	Solyc03g1	0.11	-3.23
GO:00055	iron ion bi	Molecular	0.00	0.00	Solyc05g0	Solyc05g0	0.22	-2.15
GO:00055	iron ion bi	Molecular	0.00	0.00	Solyc09g0	Solyc09g0	8.90	3.15
GO:00055	iron ion bi	Molecular	0.00	0.00	Solyc08g0	CYP707A	0.26	-1.93
GO:00055	iron ion bi	Molecular	0.00	0.00	Solyc04g0	Solyc04g0	0.45	-1.15
GO:00055	iron ion bi	Molecular	0.00	0.00	Solyc12g0	Solyc12g0	0.20	-2.32
GO:00055	iron ion bi	Molecular	0.00	0.00	Solyc10g0	Solyc10g0	0.45	-1.16
GO:00055	iron ion bi	Molecular	0.00	0.00	Solyc06g0	Solyc06g0	7.07	2.82
GO:00055	iron ion bi	Molecular	0.00	0.00	Solyc07g0	Solyc07g0	0.30	-1.76
GO:00055	iron ion bi	Molecular	0.00	0.00	Solyc06g0	Solyc06g0	0.40	-1.33
GO:00055	iron ion bi	Molecular	0.00	0.00	Solyc02g0	Solyc02g0	0.36	-1.49
GO:00055	iron ion bi	Molecular	0.00	0.00	Solyc11g0	Solyc11g0	9.10	3.19
GO:00055	iron ion bi	Molecular	0.00	0.00	Solyc12g0	Solyc12g0	0.49	-1.03
GO:00055	iron ion bi	Molecular	0.00	0.00	Solyc08g0	Solyc08g0	0.37	-1.43
GO:00055	iron ion bi	Molecular	0.00	0.00	Solyc02g0	Solyc02g0	0.10	-3.35
GO:00055	iron ion bi	Molecular	0.00	0.00	Solyc08g0	Solyc08g0	0.47	-1.09
GO:00055	iron ion bi	Molecular	0.00	0.00	Solyc01g0	Solyc01g0	0.29	-1.77
GO:00055	iron ion bi	Molecular	0.00	0.00	Solyc01g0	Solyc01g0	0.38	-1.39
GO:00055	iron ion bi	Molecular	0.00	0.00	Solyc01g0	Solyc01g0	6.15	2.62
GO:00055	iron ion bi	Molecular	0.00	0.00	Solyc08g0	Solyc08g0	0.36	-1.48
GO:00055	iron ion bi	Molecular	0.00	0.00	Solyc02g0	Solyc02g0	0.36	-1.49
GO:00055	iron ion bi	Molecular	0.00	0.00	Solyc02g0	Solyc02g0	0.00	-15.35
GO:00055	iron ion bi	Molecular	0.00	0.00	Solyc02g0	Solyc02g0	0.23	-2.14
GO:00055	iron ion bi	Molecular	0.00	0.00	Solyc02g0	Solyc02g0	2133.31	11.06
GO:00055	iron ion bi	Molecular	0.00	0.00	Solyc01g1	Solyc01g1	0.20	-2.33
GO:00055	iron ion bi	Molecular	0.00	0.00	Solyc04g0	Solyc04g0	0.16	-2.68
GO:00055	iron ion bi	Molecular	0.00	0.00	Solyc06g0	Solyc06g0	0.46	-1.12
GO:00055	iron ion bi	Molecular	0.00	0.00	Solyc08g0	Solyc08g0	2.17	1.12
GO:00055	iron ion bi	Molecular	0.00	0.00	Solyc06g0	Solyc06g0	5.75	2.52
GO:00055	iron ion bi	Molecular	0.00	0.00	Solyc04g0	Solyc04g0	0.25	-2.03
GO:00055	iron ion bi	Molecular	0.00	0.00	Solyc01g0	Solyc01g0	0.00	-10.07
GO:00055	iron ion bi	Molecular	0.00	0.00	Solyc07g0	Solyc07g0	2.89	1.53
GO:00055	iron ion bi	Molecular	0.00	0.00	Solyc10g0	Solyc10g0	0.15	-2.71
GO:00055	iron ion bi	Molecular	0.00	0.00	Solyc10g0	Solyc10g0	0.00	-11.52
GO:00055	iron ion bi	Molecular	0.00	0.00	Solyc03g1	Solyc03g1	0.10	-3.33
GO:00055	iron ion bi	Molecular	0.00	0.00	Solyc07g0	Solyc07g0	0.40	-1.31
GO:00055	iron ion bi	Molecular	0.00	0.00	Solyc01g0	Solyc01g0	2.28	1.19
GO:00055	iron ion bi	Molecular	0.00	0.00	Solyc08g0	Solyc08g0	29.09	4.86
GO:00055	iron ion bi	Molecular	0.00	0.00	Solyc11g0	Solyc11g0	0.00	-8.36
GO:00055	iron ion bi	Molecular	0.00	0.00	Solyc09g0	Solyc09g0	0.13	-2.94
GO:00055	iron ion bi	Molecular	0.00	0.00	Solyc10g0	Solyc10g0	0.08	-3.56
GO:00055	iron ion bi	Molecular	0.00	0.00	Solyc10g0	Solyc10g0	0.24	-2.04
GO:00055	iron ion bi	Molecular	0.00	0.00	Solyc03g0	Solyc03g0	0.34	-1.57
GO:00055	iron ion bi	Molecular	0.00	0.00	Solyc09g0	Solyc09g0	203.60	7.67
GO:00055	iron ion bi	Molecular	0.00	0.00	Solyc08g0	Solyc08g0	0.00	-9.01
GO:00055	iron ion bi	Molecular	0.00	0.00	Solyc06g0	Solyc06g0	0.00	-8.39
GO:00055	iron ion bi	Molecular	0.00	0.00	Solyc05g0	Solyc05g0	0.00	-8.79

GO:00055	extracellul	Cellular C	0.00	0.00	Solyc05g0	Solyc05g0	58.00	5.86
GO:00055	extracellul	Cellular C	0.00	0.00	Solyc08g0	Solyc08g0	0.19	-2.36
GO:00055	extracellul	Cellular C	0.00	0.00	Solyc09g0	Solyc09g0	0.01	-7.14
GO:00055	extracellul	Cellular C	0.00	0.00	Solyc01g1	Solyc01g1	2.32	1.21
GO:00055	extracellul	Cellular C	0.00	0.00	Solyc02g0	CHI3	0.24	-2.08
GO:00055	extracellul	Cellular C	0.00	0.00	Solyc01g0	Solyc01g0	2.53	1.34
GO:00055	extracellul	Cellular C	0.00	0.00	Solyc04g0	Solyc04g0	2.56	1.36
GO:00055	extracellul	Cellular C	0.00	0.00	Solyc01g0	Solyc01g0	0.33	-1.59
GO:00055	extracellul	Cellular C	0.00	0.00	Solyc08g0	Solyc08g0	0.16	-2.60
GO:00055	extracellul	Cellular C	0.00	0.00	Solyc03g1	Solyc03g1	0.37	-1.42
GO:00055	extracellul	Cellular C	0.00	0.00	Solyc01g0	XTH1	0.31	-1.69
GO:00055	extracellul	Cellular C	0.00	0.00	Solyc12g0	Solyc12g0	4.32	2.11
GO:00055	extracellul	Cellular C	0.00	0.00	Solyc02g0	Solyc02g0	2.19	1.13
GO:00055	extracellul	Cellular C	0.00	0.00	Solyc01g1	Solyc01g1	0.41	-1.30
GO:00055	extracellul	Cellular C	0.00	0.00	Solyc03g0	Solyc03g0	4.24	2.08
GO:00055	extracellul	Cellular C	0.00	0.00	Solyc05g0	Solyc05g0	0.26	-1.92
GO:00055	extracellul	Cellular C	0.00	0.00	Solyc10g0	CHI9	0.27	-1.88
GO:00055	extracellul	Cellular C	0.00	0.00	Solyc05g0	Solyc05g0	0.33	-1.59
GO:00055	extracellul	Cellular C	0.00	0.00	Solyc03g0	Solyc03g0	2.00	1.00
GO:00055	extracellul	Cellular C	0.00	0.00	Solyc01g0	Solyc01g0	0.18	-2.51
GO:00055	extracellul	Cellular C	0.00	0.00	Solyc04g0	Solyc04g0	0.30	-1.74
GO:00055	extracellul	Cellular C	0.00	0.00	Solyc07g0	Solyc07g0	0.45	-1.16
GO:00055	extracellul	Cellular C	0.00	0.00	Solyc01g0	Solyc01g0	4.89	2.29
GO:00055	extracellul	Cellular C	0.00	0.00	Solyc06g0	Solyc06g0	0.18	-2.48
GO:00055	extracellul	Cellular C	0.00	0.00	Solyc09g0	Solyc09g0	33.35	5.06
GO:00055	extracellul	Cellular C	0.00	0.00	Solyc09g0	Solyc09g0	2.43	1.28
GO:00055	extracellul	Cellular C	0.00	0.00	Solyc01g1	Solyc01g1	2.95	1.56
GO:00055	extracellul	Cellular C	0.00	0.00	Solyc05g0	Solyc05g0	0.48	-1.07
GO:00055	extracellul	Cellular C	0.00	0.00	Solyc03g0	Solyc03g0	2.14	1.10
GO:00055	extracellul	Cellular C	0.00	0.00	Solyc06g0	Solyc06g0	9.98	3.32
GO:00055	extracellul	Cellular C	0.00	0.00	Solyc10g0	Solyc10g0	2.57	1.36
GO:00055	extracellul	Cellular C	0.00	0.00	Solyc02g0	Solyc02g0	2.29	1.19
GO:00055	extracellul	Cellular C	0.00	0.00	Solyc02g0	Solyc02g0	0.10	-3.34
GO:00055	extracellul	Cellular C	0.00	0.00	Solyc03g1	Solyc03g1	0.10	-3.35
GO:00055	extracellul	Cellular C	0.00	0.00	Solyc02g0	Solyc02g0	0.20	-2.30
GO:00167	hydrolase a	Molecular	0.00	0.00	Solyc04g0	Solyc04g0	0.31	-1.70
GO:00167	hydrolase a	Molecular	0.00	0.00	Solyc01g1	Solyc01g1	2.32	1.21
GO:00167	hydrolase a	Molecular	0.00	0.00	Solyc03g1	Solyc03g1	2.21	1.14
GO:00167	hydrolase a	Molecular	0.00	0.00	Solyc02g0	CHI3	0.24	-2.08
GO:00167	hydrolase a	Molecular	0.00	0.00	Solyc09g0	Solyc09g0	0.15	-2.74
GO:00167	hydrolase a	Molecular	0.00	0.00	Solyc01g0	Solyc01g0	2.53	1.34
GO:00167	hydrolase a	Molecular	0.00	0.00	Solyc04g0	Solyc04g0	2.56	1.36
GO:00167	hydrolase a	Molecular	0.00	0.00	Solyc01g0	Solyc01g0	0.04	-4.82
GO:00167	hydrolase a	Molecular	0.00	0.00	Solyc03g1	Solyc03g1	0.41	-1.27
GO:00167	hydrolase a	Molecular	0.00	0.00	Solyc03g0	Solyc03g0	2.58	1.37
GO:00167	hydrolase a	Molecular	0.00	0.00	Solyc01g0	XTH1	0.31	-1.69
GO:00167	hydrolase a	Molecular	0.00	0.00	Solyc12g0	Solyc12g0	4.32	2.11
GO:00167	hydrolase a	Molecular	0.00	0.00	Solyc08g0	Solyc08g0	2.07	1.05
GO:00167	hydrolase a	Molecular	0.00	0.00	Solyc03g0	Solyc03g0	4.24	2.08
GO:00167	hydrolase a	Molecular	0.00	0.00	Solyc10g0	CHI9	0.27	-1.88
GO:00167	hydrolase a	Molecular	0.00	0.00	Solyc04g0	Solyc04g0	4.34	2.12

GO:00167	hydrolase a	Molecular	0.00	0.00	Solyc10g0	Solyc10g0	0.41	-1.27
GO:00167	hydrolase a	Molecular	0.00	0.00	Solyc06g0	Solyc06g0	0.18	-2.48
GO:00167	hydrolase a	Molecular	0.00	0.00	Solyc02g0	Solyc02g0	0.20	-2.30
GO:00095	photosyste	Cellular Co	0.00	0.00	Solyc03g0	Solyc03g0	0.18	-2.46
GO:00095	photosyste	Cellular Co	0.00	0.00	Solyc10g0	Solyc10g0	0.43	-1.23
GO:00095	photosyste	Cellular Co	0.00	0.00	Solyc06g0	psaD	0.43	-1.22
GO:00095	photosyste	Cellular Co	0.00	0.00	Solyc06g0	Solyc06g0	0.20	-2.31
GO:00095	photosyste	Cellular Co	0.00	0.00	Solyc05g0	Solyc05g0	0.15	-2.75
GO:00095	photosyste	Cellular Co	0.00	0.00	Solyc10g0	Solyc10g0	0.49	-1.02
GO:00095	photosyste	Cellular Co	0.00	0.00	Solyc08g0	Solyc08g0	0.24	-2.04
GO:00095	photosyste	Cellular Co	0.00	0.00	Solyc06g0	Solyc06g0	0.24	-2.07
GO:00095	photosyste	Cellular Co	0.00	0.00	Solyc08g0	Solyc08g0	0.21	-2.23
GO:00095	photosyste	Cellular Co	0.00	0.00	Solyc03g0	Solyc03g0	0.45	-1.15
GO:00095	photosyste	Cellular Co	0.00	0.00	Solyc05g0	Solyc05g0	0.00	-11.15
GO:00095	photosyste	Cellular Co	0.00	0.00	Solyc06g0	Solyc06g0	0.06	-4.04
GO:00095	photosyste	Cellular Co	0.00	0.00	Solyc05g0	Solyc05g0	0.46	-1.12
GO:00095	photosyste	Cellular Co	0.00	0.00	Solyc02g0	Solyc02g0	0.43	-1.20
GO:00044	monooxyg	Molecular	0.00	0.00	Solyc11g0	AOS2	0.12	-3.01
GO:00044	monooxyg	Molecular	0.00	0.00	Solyc03g1	Solyc03g1	0.11	-3.23
GO:00044	monooxyg	Molecular	0.00	0.00	Solyc05g0	Solyc05g0	0.22	-2.15
GO:00044	monooxyg	Molecular	0.00	0.00	Solyc09g0	Solyc09g0	8.90	3.15
GO:00044	monooxyg	Molecular	0.00	0.00	Solyc12g0	Solyc12g0	0.47	-1.08
GO:00044	monooxyg	Molecular	0.00	0.00	Solyc03g0	RBCS-2A	0.34	-1.58
GO:00044	monooxyg	Molecular	0.00	0.00	Solyc08g0	CYP707A	0.26	-1.93
GO:00044	monooxyg	Molecular	0.00	0.00	Solyc04g0	Solyc04g0	0.45	-1.15
GO:00044	monooxyg	Molecular	0.00	0.00	Solyc12g0	Solyc12g0	0.20	-2.32
GO:00044	monooxyg	Molecular	0.00	0.00	Solyc10g0	Solyc10g0	0.45	-1.16
GO:00044	monooxyg	Molecular	0.00	0.00	Solyc06g0	Solyc06g0	7.07	2.82
GO:00044	monooxyg	Molecular	0.00	0.00	Solyc07g0	Solyc07g0	0.30	-1.76
GO:00044	monooxyg	Molecular	0.00	0.00	Solyc06g0	Solyc06g0	0.40	-1.33
GO:00044	monooxyg	Molecular	0.00	0.00	Solyc02g0	Solyc02g0	0.36	-1.49
GO:00044	monooxyg	Molecular	0.00	0.00	Solyc12g0	Solyc12g0	0.49	-1.03
GO:00044	monooxyg	Molecular	0.00	0.00	Solyc08g0	Solyc08g0	0.37	-1.43
GO:00044	monooxyg	Molecular	0.00	0.00	Solyc02g0	Solyc02g0	0.10	-3.35
GO:00044	monooxyg	Molecular	0.00	0.00	Solyc08g0	Solyc08g0	0.47	-1.09
GO:00044	monooxyg	Molecular	0.00	0.00	Solyc01g0	Solyc01g0	0.29	-1.77
GO:00044	monooxyg	Molecular	0.00	0.00	Solyc01g0	Solyc01g0	6.15	2.62
GO:00044	monooxyg	Molecular	0.00	0.00	Solyc08g0	Solyc08g0	0.36	-1.48
GO:00044	monooxyg	Molecular	0.00	0.00	Solyc06g0	Solyc06g0	2.01	1.01
GO:00044	monooxyg	Molecular	0.00	0.00	Solyc02g0	Solyc02g0	0.00	-15.35
GO:00044	monooxyg	Molecular	0.00	0.00	Solyc02g0	Solyc02g0	0.23	-2.14
GO:00044	monooxyg	Molecular	0.00	0.00	Solyc01g1	Solyc01g1	0.20	-2.33
GO:00044	monooxyg	Molecular	0.00	0.00	Solyc04g0	Solyc04g0	0.16	-2.68
GO:00044	monooxyg	Molecular	0.00	0.00	Solyc06g0	Solyc06g0	0.46	-1.12
GO:00044	monooxyg	Molecular	0.00	0.00	Solyc06g0	Solyc06g0	5.75	2.52
GO:00044	monooxyg	Molecular	0.00	0.00	Solyc04g0	Solyc04g0	0.25	-2.03
GO:00044	monooxyg	Molecular	0.00	0.00	Solyc01g0	Solyc01g0	0.00	-10.07
GO:00044	monooxyg	Molecular	0.00	0.00	Solyc10g0	Solyc10g0	0.15	-2.71
GO:00044	monooxyg	Molecular	0.00	0.00	Solyc10g0	Solyc10g0	0.00	-11.52
GO:00044	monooxyg	Molecular	0.00	0.00	Solyc03g1	Solyc03g1	0.10	-3.33
GO:00044	monooxyg	Molecular	0.00	0.00	Solyc07g0	Solyc07g0	0.40	-1.31

GO:00044	monooxyg	Molecular	0.00	0.00	Solyc01g0	Solyc01g0	2.28	1.19
GO:00044	monooxyg	Molecular	0.00	0.00	Solyc08g0	Solyc08g0	29.09	4.86
GO:00044	monooxyg	Molecular	0.00	0.00	Solyc11g0	Solyc11g0	0.00	-8.36
GO:00044	monooxyg	Molecular	0.00	0.00	Solyc09g0	Solyc09g0	0.13	-2.94
GO:00044	monooxyg	Molecular	0.00	0.00	Solyc10g0	Solyc10g0	0.08	-3.56
GO:00044	monooxyg	Molecular	0.00	0.00	Solyc09g0	Solyc09g0	203.60	7.67
GO:00044	monooxyg	Molecular	0.00	0.00	Solyc08g0	Solyc08g0	0.00	-9.01
GO:00044	monooxyg	Molecular	0.00	0.00	Solyc06g0	Solyc06g0	0.00	-8.39
GO:00044	monooxyg	Molecular	0.00	0.00	Solyc05g0	Solyc05g0	0.00	-8.79
GO:00167	oxidoreduc	Molecular	0.00	0.00	Solyc11g0	AOS2	0.12	-3.01
GO:00167	oxidoreduc	Molecular	0.00	0.00	Solyc03g1	Solyc03g1	0.11	-3.23
GO:00167	oxidoreduc	Molecular	0.00	0.00	Solyc05g0	Solyc05g0	0.22	-2.15
GO:00167	oxidoreduc	Molecular	0.00	0.00	Solyc09g0	Solyc09g0	8.90	3.15
GO:00167	oxidoreduc	Molecular	0.00	0.00	Solyc08g0	CYP707A	0.26	-1.93
GO:00167	oxidoreduc	Molecular	0.00	0.00	Solyc04g0	Solyc04g0	0.45	-1.15
GO:00167	oxidoreduc	Molecular	0.00	0.00	Solyc12g0	Solyc12g0	0.20	-2.32
GO:00167	oxidoreduc	Molecular	0.00	0.00	Solyc10g0	Solyc10g0	0.45	-1.16
GO:00167	oxidoreduc	Molecular	0.00	0.00	Solyc06g0	Solyc06g0	7.07	2.82
GO:00167	oxidoreduc	Molecular	0.00	0.00	Solyc07g0	Solyc07g0	0.30	-1.76
GO:00167	oxidoreduc	Molecular	0.00	0.00	Solyc06g0	Solyc06g0	0.40	-1.33
GO:00167	oxidoreduc	Molecular	0.00	0.00	Solyc02g0	Solyc02g0	0.36	-1.49
GO:00167	oxidoreduc	Molecular	0.00	0.00	Solyc12g0	Solyc12g0	0.49	-1.03
GO:00167	oxidoreduc	Molecular	0.00	0.00	Solyc08g0	Solyc08g0	0.37	-1.43
GO:00167	oxidoreduc	Molecular	0.00	0.00	Solyc02g0	Solyc02g0	0.10	-3.35
GO:00167	oxidoreduc	Molecular	0.00	0.00	Solyc08g0	Solyc08g0	0.47	-1.09
GO:00167	oxidoreduc	Molecular	0.00	0.00	Solyc01g0	Solyc01g0	0.29	-1.77
GO:00167	oxidoreduc	Molecular	0.00	0.00	Solyc01g0	Solyc01g0	6.15	2.62
GO:00167	oxidoreduc	Molecular	0.00	0.00	Solyc08g0	Solyc08g0	0.36	-1.48
GO:00167	oxidoreduc	Molecular	0.00	0.00	Solyc02g0	Solyc02g0	0.00	-15.35
GO:00167	oxidoreduc	Molecular	0.00	0.00	Solyc02g0	Solyc02g0	0.23	-2.14
GO:00167	oxidoreduc	Molecular	0.00	0.00	Solyc02g0	Solyc02g0	2133.31	11.06
GO:00167	oxidoreduc	Molecular	0.00	0.00	Solyc01g1	Solyc01g1	0.20	-2.33
GO:00167	oxidoreduc	Molecular	0.00	0.00	Solyc04g0	Solyc04g0	0.16	-2.68
GO:00167	oxidoreduc	Molecular	0.00	0.00	Solyc06g0	Solyc06g0	0.46	-1.12
GO:00167	oxidoreduc	Molecular	0.00	0.00	Solyc06g0	Solyc06g0	5.75	2.52
GO:00167	oxidoreduc	Molecular	0.00	0.00	Solyc04g0	Solyc04g0	0.25	-2.03
GO:00167	oxidoreduc	Molecular	0.00	0.00	Solyc01g0	Solyc01g0	0.00	-10.07
GO:00167	oxidoreduc	Molecular	0.00	0.00	Solyc10g0	Solyc10g0	0.15	-2.71
GO:00167	oxidoreduc	Molecular	0.00	0.00	Solyc10g0	Solyc10g0	0.00	-11.52
GO:00167	oxidoreduc	Molecular	0.00	0.00	Solyc03g1	Solyc03g1	0.10	-3.33
GO:00167	oxidoreduc	Molecular	0.00	0.00	Solyc07g0	Solyc07g0	0.40	-1.31
GO:00167	oxidoreduc	Molecular	0.00	0.00	Solyc01g0	Solyc01g0	2.28	1.19
GO:00167	oxidoreduc	Molecular	0.00	0.00	Solyc08g0	Solyc08g0	29.09	4.86
GO:00167	oxidoreduc	Molecular	0.00	0.00	Solyc11g0	Solyc11g0	0.00	-8.36
GO:00167	oxidoreduc	Molecular	0.00	0.00	Solyc09g0	Solyc09g0	0.13	-2.94
GO:00167	oxidoreduc	Molecular	0.00	0.00	Solyc10g0	Solyc10g0	0.08	-3.56
GO:00167	oxidoreduc	Molecular	0.00	0.00	Solyc10g0	Solyc10g0	0.28	-1.85
GO:00167	oxidoreduc	Molecular	0.00	0.00	Solyc09g0	Solyc09g0	203.60	7.67
GO:00167	oxidoreduc	Molecular	0.00	0.00	Solyc08g0	Solyc08g0	0.00	-9.01
GO:00167	oxidoreduc	Molecular	0.00	0.00	Solyc06g0	Solyc06g0	0.00	-8.39
GO:00167	oxidoreduc	Molecular	0.00	0.00	Solyc05g0	Solyc05g0	0.00	-8.79

GO:00200	heme bind	Molecular	0.00	0.00	Solyc12g0	Solyc12g0	0.28	-1.84
GO:00200	heme bind	Molecular	0.00	0.00	Solyc11g0	AOS2	0.12	-3.01
GO:00200	heme bind	Molecular	0.00	0.00	Solyc03g1	Solyc03g1	0.11	-3.23
GO:00200	heme bind	Molecular	0.00	0.00	Solyc05g0	Solyc05g0	0.22	-2.15
GO:00200	heme bind	Molecular	0.00	0.00	Solyc09g0	Solyc09g0	8.90	3.15
GO:00200	heme bind	Molecular	0.00	0.00	Solyc09g0	Solyc09g0	0.44	-1.17
GO:00200	heme bind	Molecular	0.00	0.00	Solyc08g0	CYP707A	0.26	-1.93
GO:00200	heme bind	Molecular	0.00	0.00	Solyc04g0	Solyc04g0	0.45	-1.15
GO:00200	heme bind	Molecular	0.00	0.00	Solyc12g0	Solyc12g0	0.20	-2.32
GO:00200	heme bind	Molecular	0.00	0.00	Solyc10g0	Solyc10g0	0.45	-1.16
GO:00200	heme bind	Molecular	0.00	0.00	Solyc06g0	Solyc06g0	7.07	2.82
GO:00200	heme bind	Molecular	0.00	0.00	Solyc07g0	Solyc07g0	0.30	-1.76
GO:00200	heme bind	Molecular	0.00	0.00	Solyc06g0	Solyc06g0	0.40	-1.33
GO:00200	heme bind	Molecular	0.00	0.00	Solyc02g0	Solyc02g0	0.36	-1.49
GO:00200	heme bind	Molecular	0.00	0.00	Solyc12g0	Solyc12g0	0.49	-1.03
GO:00200	heme bind	Molecular	0.00	0.00	Solyc08g0	Solyc08g0	0.37	-1.43
GO:00200	heme bind	Molecular	0.00	0.00	Solyc02g0	Solyc02g0	0.10	-3.35
GO:00200	heme bind	Molecular	0.00	0.00	Solyc08g0	Solyc08g0	0.47	-1.09
GO:00200	heme bind	Molecular	0.00	0.00	Solyc03g0	Solyc03g0	0.29	-1.79
GO:00200	heme bind	Molecular	0.00	0.00	Solyc01g0	Solyc01g0	0.29	-1.77
GO:00200	heme bind	Molecular	0.00	0.00	Solyc04g0	Solyc04g0	0.31	-1.69
GO:00200	heme bind	Molecular	0.00	0.00	Solyc01g0	Solyc01g0	6.15	2.62
GO:00200	heme bind	Molecular	0.00	0.00	Solyc08g0	Solyc08g0	0.36	-1.48
GO:00200	heme bind	Molecular	0.00	0.00	Solyc02g0	Solyc02g0	2.89	1.53
GO:00200	heme bind	Molecular	0.00	0.00	Solyc02g0	Solyc02g0	0.00	-11.78
GO:00200	heme bind	Molecular	0.00	0.00	Solyc02g0	Solyc02g0	0.00	-15.35
GO:00200	heme bind	Molecular	0.00	0.00	Solyc02g0	Solyc02g0	0.23	-2.14
GO:00200	heme bind	Molecular	0.00	0.00	Solyc01g1	Solyc01g1	0.20	-2.33
GO:00200	heme bind	Molecular	0.00	0.00	Solyc04g0	Solyc04g0	0.16	-2.68
GO:00200	heme bind	Molecular	0.00	0.00	Solyc06g0	Solyc06g0	0.46	-1.12
GO:00200	heme bind	Molecular	0.00	0.00	Solyc09g0	Solyc09g0	2.43	1.28
GO:00200	heme bind	Molecular	0.00	0.00	Solyc06g0	Solyc06g0	5.75	2.52
GO:00200	heme bind	Molecular	0.00	0.00	Solyc04g0	Solyc04g0	0.25	-2.03
GO:00200	heme bind	Molecular	0.00	0.00	Solyc01g0	Solyc01g0	0.00	-10.07
GO:00200	heme bind	Molecular	0.00	0.00	Solyc10g0	Solyc10g0	0.15	-2.71
GO:00200	heme bind	Molecular	0.00	0.00	Solyc01g1	Solyc01g1	2.95	1.56
GO:00200	heme bind	Molecular	0.00	0.00	Solyc10g0	Solyc10g0	0.00	-11.52
GO:00200	heme bind	Molecular	0.00	0.00	Solyc03g0	Solyc03g0	0.39	-1.34
GO:00200	heme bind	Molecular	0.00	0.00	Solyc03g1	Solyc03g1	0.10	-3.33
GO:00200	heme bind	Molecular	0.00	0.00	Solyc07g0	Solyc07g0	0.40	-1.31
GO:00200	heme bind	Molecular	0.00	0.00	Solyc03g0	Solyc03g0	2.14	1.10
GO:00200	heme bind	Molecular	0.00	0.00	Solyc06g0	Solyc06g0	9.98	3.32
GO:00200	heme bind	Molecular	0.00	0.00	Solyc02g0	Solyc02g0	2.29	1.19
GO:00200	heme bind	Molecular	0.00	0.00	Solyc01g0	Solyc01g0	2.28	1.19
GO:00200	heme bind	Molecular	0.00	0.00	Solyc08g0	Solyc08g0	29.09	4.86
GO:00200	heme bind	Molecular	0.00	0.00	Solyc11g0	Solyc11g0	0.00	-8.36
GO:00200	heme bind	Molecular	0.00	0.00	Solyc09g0	Solyc09g0	0.13	-2.94
GO:00200	heme bind	Molecular	0.00	0.00	Solyc10g0	Solyc10g0	0.08	-3.56
GO:00200	heme bind	Molecular	0.00	0.00	Solyc02g0	Solyc02g0	0.10	-3.34
GO:00200	heme bind	Molecular	0.00	0.00	Solyc09g0	Solyc09g0	203.60	7.67
GO:00200	heme bind	Molecular	0.00	0.00	Solyc08g0	Solyc08g0	0.00	-9.01

GO:00200	heme bindi	Molecular	0.00	0.00	Solyc04g0	Solyc04g0	0.49	-1.04
GO:00200	heme bindi	Molecular	0.00	0.00	Solyc06g0	Solyc06g0	0.00	-8.39
GO:00200	heme bindi	Molecular	0.00	0.00	Solyc01g1	Solyc01g1	0.00	-9.56
GO:00200	heme bindi	Molecular	0.00	0.00	Solyc05g0	Solyc05g0	0.00	-8.79
GO:00081	metabolic	Biological	0.00	0.00	Solyc04g0	Solyc04g0	0.31	-1.70
GO:00081	metabolic	Biological	0.00	0.00	Solyc01g1	Solyc01g1	2.32	1.21
GO:00081	metabolic	Biological	0.00	0.00	Solyc03g1	Solyc03g1	2.21	1.14
GO:00081	metabolic	Biological	0.00	0.00	Solyc02g0	CHI3	0.24	-2.08
GO:00081	metabolic	Biological	0.00	0.00	Solyc09g0	Solyc09g0	0.15	-2.74
GO:00081	metabolic	Biological	0.00	0.00	Solyc01g0	Solyc01g0	2.53	1.34
GO:00081	metabolic	Biological	0.00	0.00	Solyc04g0	Solyc04g0	2.56	1.36
GO:00081	metabolic	Biological	0.00	0.00	Solyc01g0	Solyc01g0	0.04	-4.82
GO:00081	metabolic	Biological	0.00	0.00	Solyc03g0	Solyc03g0	2.58	1.37
GO:00081	metabolic	Biological	0.00	0.00	Solyc01g0	XTH1	0.31	-1.69
GO:00081	metabolic	Biological	0.00	0.00	Solyc12g0	Solyc12g0	4.32	2.11
GO:00081	metabolic	Biological	0.00	0.00	Solyc08g0	Solyc08g0	2.07	1.05
GO:00081	metabolic	Biological	0.00	0.00	Solyc03g0	Solyc03g0	4.24	2.08
GO:00081	metabolic	Biological	0.00	0.00	Solyc10g0	CHI9	0.27	-1.88
GO:00081	metabolic	Biological	0.00	0.00	Solyc04g0	Solyc04g0	4.34	2.12
GO:00081	metabolic	Biological	0.00	0.00	Solyc06g0	Solyc06g0	0.18	-2.48
GO:00081	metabolic	Biological	0.00	0.00	Solyc02g0	Solyc02g0	0.20	-2.30
GO:00167	hydrolase	Molecular	0.00	0.00	Solyc02g0	Solyc02g0	2.65	1.40
GO:00167	hydrolase	Molecular	0.00	0.00	Solyc06g0	Solyc06g0	3.24	1.69
GO:00167	hydrolase	Molecular	0.00	0.00	Solyc04g0	Solyc04g0	0.31	-1.70
GO:00167	hydrolase	Molecular	0.00	0.00	Solyc01g1	Solyc01g1	0.45	-1.16
GO:00167	hydrolase	Molecular	0.00	0.00	Solyc01g1	Solyc01g1	2.32	1.21
GO:00167	hydrolase	Molecular	0.00	0.00	Solyc03g1	Solyc03g1	2.21	1.14
GO:00167	hydrolase	Molecular	0.00	0.00	Solyc02g0	CHI3	0.24	-2.08
GO:00167	hydrolase	Molecular	0.00	0.00	Solyc09g0	Solyc09g0	0.15	-2.74
GO:00167	hydrolase	Molecular	0.00	0.00	Solyc03g0	Solyc03g0	0.15	-2.71
GO:00167	hydrolase	Molecular	0.00	0.00	Solyc08g0	Solyc08g0	0.26	-1.95
GO:00167	hydrolase	Molecular	0.00	0.00	Solyc01g0	Solyc01g0	2.53	1.34
GO:00167	hydrolase	Molecular	0.00	0.00	Solyc07g0	Solyc07g0	0.08	-3.60
GO:00167	hydrolase	Molecular	0.00	0.00	Solyc04g0	Solyc04g0	2.56	1.36
GO:00167	hydrolase	Molecular	0.00	0.00	Solyc03g0	Solyc03g0	2.03	1.02
GO:00167	hydrolase	Molecular	0.00	0.00	Solyc01g0	Solyc01g0	2.60	1.38
GO:00167	hydrolase	Molecular	0.00	0.00	Solyc01g0	Solyc01g0	0.04	-4.82
GO:00167	hydrolase	Molecular	0.00	0.00	Solyc03g1	Solyc03g1	0.37	-1.42
GO:00167	hydrolase	Molecular	0.00	0.00	Solyc03g0	Solyc03g0	2.58	1.37
GO:00167	hydrolase	Molecular	0.00	0.00	Solyc09g0	Solyc09g0	0.34	-1.55
GO:00167	hydrolase	Molecular	0.00	0.00	Solyc01g0	XTH1	0.31	-1.69
GO:00167	hydrolase	Molecular	0.00	0.00	Solyc12g0	Solyc12g0	0.48	-1.06
GO:00167	hydrolase	Molecular	0.00	0.00	Solyc12g0	Solyc12g0	4.32	2.11
GO:00167	hydrolase	Molecular	0.00	0.00	Solyc01g0	Solyc01g0	0.30	-1.74
GO:00167	hydrolase	Molecular	0.00	0.00	Solyc08g0	Solyc08g0	2.07	1.05
GO:00167	hydrolase	Molecular	0.00	0.00	Solyc01g1	Solyc01g1	0.41	-1.30
GO:00167	hydrolase	Molecular	0.00	0.00	Solyc03g0	Solyc03g0	4.24	2.08
GO:00167	hydrolase	Molecular	0.00	0.00	Solyc10g0	CHI9	0.27	-1.88
GO:00167	hydrolase	Molecular	0.00	0.00	Solyc05g0	Solyc05g0	0.40	-1.31
GO:00167	hydrolase	Molecular	0.00	0.00	Solyc04g0	Solyc04g0	0.24	-2.08
GO:00167	hydrolase	Molecular	0.00	0.00	Solyc04g0	Solyc04g0	4.34	2.12

GO:00167	hydrolase a	Molecular	0.00	0.00	Solyc08g0	Solyc08g0	0.47	-1.07
GO:00167	hydrolase a	Molecular	0.00	0.00	Solyc12g0	Solyc12g0	0.35	-1.50
GO:00167	hydrolase a	Molecular	0.00	0.00	Solyc06g0	Solyc06g0	2.69	1.43
GO:00167	hydrolase a	Molecular	0.00	0.00	Solyc03g0	Solyc03g0	0.08	-3.70
GO:00167	hydrolase a	Molecular	0.00	0.00	Solyc10g0	Solyc10g0	0.00	-12.54
GO:00167	hydrolase a	Molecular	0.00	0.00	Solyc04g0	Solyc04g0	0.30	-1.74
GO:00167	hydrolase a	Molecular	0.00	0.00	Solyc06g0	Solyc06g0	0.18	-2.48
GO:00167	hydrolase a	Molecular	0.00	0.00	Solyc01g0	Solyc01g0	0.50	-1.01
GO:00167	hydrolase a	Molecular	0.00	0.00	Solyc08g0	Solyc08g0	0.11	-3.22
GO:00167	hydrolase a	Molecular	0.00	0.00	Solyc08g0	Solyc08g0	0.37	-1.42
GO:00167	hydrolase a	Molecular	0.00	0.00	Solyc05g0	Solyc05g0	0.48	-1.07
GO:00167	hydrolase a	Molecular	0.00	0.00	Solyc01g0	Solyc01g0	0.31	-1.67
GO:00167	hydrolase a	Molecular	0.00	0.00	Solyc07g0	Solyc07g0	0.18	-2.47
GO:00167	hydrolase a	Molecular	0.00	0.00	Solyc07g0	Solyc07g0	0.47	-1.08
GO:00167	hydrolase a	Molecular	0.00	0.00	Solyc02g0	Solyc02g0	0.12	-3.05
GO:00167	hydrolase a	Molecular	0.00	0.00	Solyc09g0	Solyc09g0	0.47	-1.09
GO:00167	hydrolase a	Molecular	0.00	0.00	Solyc05g0	Solyc05g0	0.34	-1.55
GO:00167	hydrolase a	Molecular	0.00	0.00	Solyc07g0	Solyc07g0	0.00	-9.63
GO:00167	hydrolase a	Molecular	0.00	0.00	Solyc08g0	Solyc08g0	311.75	8.28
GO:00167	hydrolase a	Molecular	0.00	0.00	Solyc02g0	Solyc02g0	0.20	-2.30
GO:00485	recognition	Biological	0.00	0.00	Solyc05g0	Solyc05g0	0.46	-1.12
GO:00485	recognition	Biological	0.00	0.00	Solyc02g0	Solyc02g0	0.12	-3.00
GO:00485	recognition	Biological	0.00	0.00	Solyc10g0	Solyc10g0	0.06	-4.05
GO:00485	recognition	Biological	0.00	0.00	Solyc02g0	Solyc02g0	0.10	-3.33
GO:00485	recognition	Biological	0.00	0.00	Solyc02g0	Solyc02g0	0.02	-5.39
GO:00485	recognition	Biological	0.00	0.00	Solyc07g0	Solyc07g0	0.46	-1.11
GO:00485	recognition	Biological	0.00	0.00	Solyc02g0	Solyc02g0	2.71	1.44
GO:00485	recognition	Biological	0.00	0.00	Solyc02g0	Solyc02g0	0.06	-3.96
GO:00485	recognition	Biological	0.00	0.00	Solyc07g0	Solyc07g0	0.29	-1.79
GO:00485	recognition	Biological	0.00	0.00	Solyc09g0	Solyc09g0	0.38	-1.40
GO:00485	recognition	Biological	0.00	0.00	Solyc09g0	Solyc09g0	0.08	-3.73
GO:00485	recognition	Biological	0.00	0.00	Solyc04g0	Solyc04g0	0.00	-8.72
GO:00485	recognition	Biological	0.00	0.00	Solyc01g0	Solyc01g0	0.33	-1.61
GO:00094	response to	Biological	0.00	0.00	Solyc07g0	Solyc07g0	0.17	-2.54
GO:00094	response to	Biological	0.00	0.00	Solyc11g0	Solyc11g0	0.11	-3.13
GO:00094	response to	Biological	0.00	0.00	Solyc03g0	Solyc03g0	0.18	-2.46
GO:00094	response to	Biological	0.00	0.00	Solyc10g0	Solyc10g0	0.43	-1.23
GO:00094	response to	Biological	0.00	0.00	Solyc05g0	Solyc05g0	0.15	-2.75
GO:00094	response to	Biological	0.00	0.00	Solyc10g0	Solyc10g0	0.49	-1.02
GO:00094	response to	Biological	0.00	0.00	Solyc03g0	Solyc03g0	0.45	-1.15
GO:00094	response to	Biological	0.00	0.00	Solyc05g0	Solyc05g0	0.00	-11.15
GO:00094	response to	Biological	0.00	0.00	Solyc02g0	Solyc02g0	0.45	-1.16
GO:00094	response to	Biological	0.00	0.00	Solyc10g0	Solyc10g0	0.45	-1.15
GO:00094	response to	Biological	0.00	0.00	Solyc01g1	CAP10A	0.35	-1.51
GO:00094	response to	Biological	0.00	0.00	Solyc07g0	Solyc07g0	0.18	-2.49
GO:00045	hydrolase a	Molecular	0.00	0.00	Solyc04g0	Solyc04g0	0.31	-1.70
GO:00045	hydrolase a	Molecular	0.00	0.00	Solyc01g1	Solyc01g1	2.32	1.21
GO:00045	hydrolase a	Molecular	0.00	0.00	Solyc03g1	Solyc03g1	2.21	1.14
GO:00045	hydrolase a	Molecular	0.00	0.00	Solyc09g0	Solyc09g0	0.15	-2.74
GO:00045	hydrolase a	Molecular	0.00	0.00	Solyc01g0	Solyc01g0	2.53	1.34
GO:00045	hydrolase a	Molecular	0.00	0.00	Solyc07g0	Solyc07g0	0.03	-5.23

GO:00045	hydrolase a	Molecular	0.00	0.00	Solyc04g0	Solyc04g0	2.56	1.36
GO:00045	hydrolase a	Molecular	0.00	0.00	Solyc01g0	Solyc01g0	0.04	-4.82
GO:00045	hydrolase a	Molecular	0.00	0.00	Solyc05g0	Solyc05g0	0.37	-1.42
GO:00045	hydrolase a	Molecular	0.00	0.00	Solyc03g0	Solyc03g0	2.58	1.37
GO:00045	hydrolase a	Molecular	0.00	0.00	Solyc01g0	XTH1	0.31	-1.69
GO:00045	hydrolase a	Molecular	0.00	0.00	Solyc12g0	Solyc12g0	4.32	2.11
GO:00045	hydrolase a	Molecular	0.00	0.00	Solyc09g0	Solyc09g0	0.39	-1.37
GO:00045	hydrolase a	Molecular	0.00	0.00	Solyc05g0	Solyc05g0	3.55	1.83
GO:00045	hydrolase a	Molecular	0.00	0.00	Solyc08g0	Solyc08g0	2.07	1.05
GO:00045	hydrolase a	Molecular	0.00	0.00	Solyc12g0	Solyc12g0	0.44	-1.19
GO:00045	hydrolase a	Molecular	0.00	0.00	Solyc03g0	Solyc03g0	4.24	2.08
GO:00045	hydrolase a	Molecular	0.00	0.00	Solyc05g0	Solyc05g0	0.26	-1.92
GO:00045	hydrolase a	Molecular	0.00	0.00	Solyc12g0	Solyc12g0	3.12	1.64
GO:00045	hydrolase a	Molecular	0.00	0.00	Solyc03g0	Solyc03g0	3.51	1.81
GO:00045	hydrolase a	Molecular	0.00	0.00	Solyc11g0	Solyc11g0	2.41	1.27
GO:00045	hydrolase a	Molecular	0.00	0.00	Solyc11g0	Solyc11g0	2.71	1.44
GO:00045	hydrolase a	Molecular	0.00	0.00	Solyc01g0	Solyc01g0	0.02	-5.46
GO:00045	hydrolase a	Molecular	0.00	0.00	Solyc06g0	Solyc06g0	0.18	-2.48
GO:00045	hydrolase a	Molecular	0.00	0.00	Solyc12g0	Solyc12g0	0.00	-9.73
GO:00045	hydrolase a	Molecular	0.00	0.00	Solyc11g0	Solyc11g0	0.04	-4.78
GO:00045	hydrolase a	Molecular	0.00	0.00	Solyc11g0	Solyc11g0	0.42	-1.25
GO:00045	hydrolase a	Molecular	0.00	0.00	Solyc11g0	Solyc11g0	482.30	8.91
GO:00045	hydrolase a	Molecular	0.00	0.00	Solyc03g0	Solyc03g0	5.49	2.46
GO:00045	hydrolase a	Molecular	0.00	0.00	Solyc09g0	Solyc09g0	0.39	-1.34
GO:00045	hydrolase a	Molecular	0.00	0.00	Solyc02g0	Solyc02g0	0.20	-2.30
GO:00164	oxidoreduc	Molecular	0.00	0.00	Solyc10g0	Solyc10g0	0.03	-5.15
GO:00164	oxidoreduc	Molecular	0.00	0.00	Solyc07g0	Solyc07g0	0.17	-2.54
GO:00164	oxidoreduc	Molecular	0.00	0.00	Solyc02g0	Solyc02g0	0.23	-2.13
GO:00164	oxidoreduc	Molecular	0.00	0.00	Solyc12g0	Solyc12g0	0.28	-1.84
GO:00164	oxidoreduc	Molecular	0.00	0.00	Solyc06g0	Solyc06g0	16.95	4.08
GO:00164	oxidoreduc	Molecular	0.00	0.00	Solyc02g0	Solyc02g0	0.02	-5.62
GO:00164	oxidoreduc	Molecular	0.00	0.00	Solyc01g0	Solyc01g0	0.44	-1.18
GO:00164	oxidoreduc	Molecular	0.00	0.00	Solyc11g0	AOS2	0.12	-3.01
GO:00164	oxidoreduc	Molecular	0.00	0.00	Solyc03g0	Solyc03g0	2.69	1.43
GO:00164	oxidoreduc	Molecular	0.00	0.00	Solyc01g0	Solyc01g0	4.36	2.12
GO:00164	oxidoreduc	Molecular	0.00	0.00	Solyc01g0	Solyc01g0	2.27	1.18
GO:00164	oxidoreduc	Molecular	0.00	0.00	Solyc01g1	Solyc01g1	3.04	1.60
GO:00164	oxidoreduc	Molecular	0.00	0.00	Solyc07g0	NCED1	0.30	-1.72
GO:00164	oxidoreduc	Molecular	0.00	0.00	Solyc02g0	Solyc02g0	2.02	1.01
GO:00164	oxidoreduc	Molecular	0.00	0.00	Solyc03g0	Solyc03g0	0.36	-1.49
GO:00164	oxidoreduc	Molecular	0.00	0.00	Solyc07g0	Solyc07g0	0.16	-2.62
GO:00164	oxidoreduc	Molecular	0.00	0.00	Solyc12g0	Solyc12g0	2.13	1.09
GO:00164	oxidoreduc	Molecular	0.00	0.00	Solyc03g1	Solyc03g1	0.11	-3.23
GO:00164	oxidoreduc	Molecular	0.00	0.00	Solyc07g0	Solyc07g0	2.37	1.24
GO:00164	oxidoreduc	Molecular	0.00	0.00	Solyc08g0	Solyc08g0	0.21	-2.27
GO:00164	oxidoreduc	Molecular	0.00	0.00	Solyc04g0	Solyc04g0	0.48	-1.06
GO:00164	oxidoreduc	Molecular	0.00	0.00	Solyc08g0	Solyc08g0	0.39	-1.35
GO:00164	oxidoreduc	Molecular	0.00	0.00	Solyc08g0	Solyc08g0	0.19	-2.37
GO:00164	oxidoreduc	Molecular	0.00	0.00	Solyc11g0	Solyc11g0	0.16	-2.69
GO:00164	oxidoreduc	Molecular	0.00	0.00	Solyc08g0	CYP707A	0.26	-1.93
GO:00164	oxidoreduc	Molecular	0.00	0.00	Solyc01g0	Solyc01g0	0.26	-1.92

GO:00164	oxidoreduc	Molecular	0.00	0.00	Solyc06g0	Solyc06g0	0.47	-1.09
GO:00164	oxidoreduc	Molecular	0.00	0.00	Solyc08g0	Solyc08g0	2.04	1.03
GO:00164	oxidoreduc	Molecular	0.00	0.00	Solyc08g0	Solyc08g0	0.34	-1.55
GO:00164	oxidoreduc	Molecular	0.00	0.00	Solyc07g0	Solyc07g0	0.44	-1.19
GO:00164	oxidoreduc	Molecular	0.00	0.00	Solyc03g0	Solyc03g0	0.46	-1.11
GO:00164	oxidoreduc	Molecular	0.00	0.00	Solyc09g0	Solyc09g0	0.40	-1.33
GO:00164	oxidoreduc	Molecular	0.00	0.00	Solyc04g0	Solyc04g0	0.41	-1.27
GO:00164	oxidoreduc	Molecular	0.00	0.00	Solyc01g0	Solyc01g0	0.41	-1.30
GO:00164	oxidoreduc	Molecular	0.00	0.00	Solyc06g0	Solyc06g0	0.40	-1.33
GO:00164	oxidoreduc	Molecular	0.00	0.00	Solyc11g0	Solyc11g0	9.10	3.19
GO:00164	oxidoreduc	Molecular	0.00	0.00	Solyc08g0	Solyc08g0	0.35	-1.51
GO:00164	oxidoreduc	Molecular	0.00	0.00	Solyc02g0	Solyc02g0	0.50	-1.01
GO:00164	oxidoreduc	Molecular	0.00	0.00	Solyc04g0	Solyc04g0	0.19	-2.40
GO:00164	oxidoreduc	Molecular	0.00	0.00	Solyc11g0	Solyc11g0	3.98	1.99
GO:00164	oxidoreduc	Molecular	0.00	0.00	Solyc03g0	Solyc03g0	0.00	-13.23
GO:00164	oxidoreduc	Molecular	0.00	0.00	Solyc01g0	Solyc01g0	0.38	-1.39
GO:00164	oxidoreduc	Molecular	0.00	0.00	Solyc03g1	Solyc03g1	0.46	-1.12
GO:00164	oxidoreduc	Molecular	0.00	0.00	Solyc07g0	OPR3	0.47	-1.10
GO:00164	oxidoreduc	Molecular	0.00	0.00	Solyc02g0	Solyc02g0	6.42	2.68
GO:00164	oxidoreduc	Molecular	0.00	0.00	Solyc12g0	Solyc12g0	10.12	3.34
GO:00164	oxidoreduc	Molecular	0.00	0.00	Solyc02g0	Solyc02g0	5.84	2.55
GO:00164	oxidoreduc	Molecular	0.00	0.00	Solyc01g0	Solyc01g0	0.18	-2.51
GO:00164	oxidoreduc	Molecular	0.00	0.00	Solyc06g0	Solyc06g0	2.01	1.01
GO:00164	oxidoreduc	Molecular	0.00	0.00	Solyc02g0	Solyc02g0	0.36	-1.49
GO:00164	oxidoreduc	Molecular	0.00	0.00	Solyc02g0	Solyc02g0	0.00	-11.78
GO:00164	oxidoreduc	Molecular	0.00	0.00	Solyc05g0	Solyc05g0	0.31	-1.67
GO:00164	oxidoreduc	Molecular	0.00	0.00	Solyc01g0	Solyc01g0	0.32	-1.64
GO:00164	oxidoreduc	Molecular	0.00	0.00	Solyc08g0	Solyc08g0	0.44	-1.20
GO:00164	oxidoreduc	Molecular	0.00	0.00	Solyc02g0	Solyc02g0	2.79	1.48
GO:00164	oxidoreduc	Molecular	0.00	0.00	Solyc06g0	Solyc06g0	0.39	-1.35
GO:00164	oxidoreduc	Molecular	0.00	0.00	Solyc03g1	Solyc03g1	0.43	-1.23
GO:00164	oxidoreduc	Molecular	0.00	0.00	Solyc09g0	Solyc09g0	0.45	-1.14
GO:00164	oxidoreduc	Molecular	0.00	0.00	Solyc06g0	Solyc06g0	0.30	-1.75
GO:00164	oxidoreduc	Molecular	0.00	0.00	Solyc11g0	Solyc11g0	0.06	-4.06
GO:00164	oxidoreduc	Molecular	0.00	0.00	Solyc08g0	Solyc08g0	2.17	1.12
GO:00164	oxidoreduc	Molecular	0.00	0.00	Solyc09g0	Solyc09g0	4.39	2.13
GO:00164	oxidoreduc	Molecular	0.00	0.00	Solyc02g0	Solyc02g0	2.43	1.28
GO:00164	oxidoreduc	Molecular	0.00	0.00	Solyc12g0	Solyc12g0	0.13	-2.93
GO:00164	oxidoreduc	Molecular	0.00	0.00	Solyc01g1	Solyc01g1	2.95	1.56
GO:00164	oxidoreduc	Molecular	0.00	0.00	Solyc09g0	Solyc09g0	0.29	-1.78
GO:00164	oxidoreduc	Molecular	0.00	0.00	Solyc09g0	Solyc09g0	3.02	1.59
GO:00164	oxidoreduc	Molecular	0.00	0.00	Solyc02g0	Solyc02g0	0.00	-8.25
GO:00164	oxidoreduc	Molecular	0.00	0.00	Solyc01g0	Solyc01g0	3.58	1.84
GO:00097	photosynth	Biological	0.00	0.00	Solyc03g0	Solyc03g0	0.18	-2.46
GO:00097	photosynth	Biological	0.00	0.00	Solyc10g0	Solyc10g0	0.43	-1.23
GO:00097	photosynth	Biological	0.00	0.00	Solyc05g0	Solyc05g0	0.15	-2.75
GO:00097	photosynth	Biological	0.00	0.00	Solyc10g0	Solyc10g0	0.49	-1.02
GO:00097	photosynth	Biological	0.00	0.00	Solyc03g0	Solyc03g0	0.45	-1.15
GO:00097	photosynth	Biological	0.00	0.00	Solyc05g0	Solyc05g0	0.00	-11.15
GO:00097	photosynth	Biological	0.00	0.00	Solyc01g1	CAP10A	0.35	-1.51
GO:00167	oxidoreduc	Molecular	0.00	0.00	Solyc05g0	Solyc05g0	0.22	-2.15

GO:00167	oxidoreduc	Molecular	0.00	0.00	Solyc08g0	CYP707A	0.26	-1.93
GO:00167	oxidoreduc	Molecular	0.00	0.00	Solyc07g0	Solyc07g0	0.30	-1.76
GO:00167	oxidoreduc	Molecular	0.00	0.00	Solyc02g0	Solyc02g0	0.36	-1.49
GO:00167	oxidoreduc	Molecular	0.00	0.00	Solyc08g0	Solyc08g0	0.37	-1.43
GO:00167	oxidoreduc	Molecular	0.00	0.00	Solyc08g0	Solyc08g0	0.47	-1.09
GO:00167	oxidoreduc	Molecular	0.00	0.00	Solyc08g0	Solyc08g0	0.36	-1.48
GO:00167	oxidoreduc	Molecular	0.00	0.00	Solyc02g0	Solyc02g0	0.23	-2.14
GO:00167	oxidoreduc	Molecular	0.00	0.00	Solyc04g0	Solyc04g0	0.16	-2.68
GO:00167	oxidoreduc	Molecular	0.00	0.00	Solyc04g0	Solyc04g0	0.25	-2.03
GO:00167	oxidoreduc	Molecular	0.00	0.00	Solyc01g0	Solyc01g0	0.00	-10.07
GO:00167	oxidoreduc	Molecular	0.00	0.00	Solyc03g1	Solyc03g1	0.10	-3.33
GO:00167	oxidoreduc	Molecular	0.00	0.00	Solyc01g0	Solyc01g0	2.28	1.19
GO:00167	oxidoreduc	Molecular	0.00	0.00	Solyc11g0	Solyc11g0	0.00	-8.36
GO:00167	oxidoreduc	Molecular	0.00	0.00	Solyc09g0	Solyc09g0	0.13	-2.94
GO:00167	oxidoreduc	Molecular	0.00	0.00	Solyc08g0	Solyc08g0	0.00	-9.01
GO:00097	photosynth	Biological	0.00	0.00	Solyc03g0	Solyc03g0	0.18	-2.46
GO:00097	photosynth	Biological	0.00	0.00	Solyc10g0	Solyc10g0	0.43	-1.23
GO:00097	photosynth	Biological	0.00	0.00	Solyc05g0	Solyc05g0	0.15	-2.75
GO:00097	photosynth	Biological	0.00	0.00	Solyc10g0	Solyc10g0	0.49	-1.02
GO:00097	photosynth	Biological	0.00	0.00	Solyc03g0	Solyc03g0	0.45	-1.15
GO:00097	photosynth	Biological	0.00	0.00	Solyc07g0	Solyc07g0	0.32	-1.65
GO:00097	photosynth	Biological	0.00	0.00	Solyc05g0	Solyc05g0	0.00	-11.15
GO:00097	photosynth	Biological	0.00	0.00	Solyc01g1	CAP10A	0.35	-1.51
GO:00480	apoplast	Cellular C	0.00	0.00	Solyc09g0	Solyc09g0	0.15	-2.74
GO:00480	apoplast	Cellular C	0.00	0.00	Solyc04g0	Solyc04g0	2.56	1.36
GO:00480	apoplast	Cellular C	0.00	0.00	Solyc09g0	Solyc09g0	0.21	-2.26
GO:00480	apoplast	Cellular C	0.00	0.00	Solyc01g0	XTH1	0.31	-1.69
GO:00480	apoplast	Cellular C	0.00	0.00	Solyc12g0	Solyc12g0	4.32	2.11
GO:00480	apoplast	Cellular C	0.00	0.00	Solyc05g0	Solyc05g0	3.55	1.83
GO:00480	apoplast	Cellular C	0.00	0.00	Solyc03g0	Solyc03g0	4.24	2.08
GO:00480	apoplast	Cellular C	0.00	0.00	Solyc05g0	Solyc05g0	0.33	-1.59
GO:00480	apoplast	Cellular C	0.00	0.00	Solyc05g0	Solyc05g0	0.31	-1.67
GO:00480	apoplast	Cellular C	0.00	0.00	Solyc03g0	Solyc03g0	3.51	1.81
GO:00480	apoplast	Cellular C	0.00	0.00	Solyc02g0	Solyc02g0	0.46	-1.12
GO:00480	apoplast	Cellular C	0.00	0.00	Solyc08g0	Solyc08g0	0.11	-3.22
GO:00480	apoplast	Cellular C	0.00	0.00	Solyc12g0	Solyc12g0	0.13	-2.93
GO:00480	apoplast	Cellular C	0.00	0.00	Solyc02g0	Solyc02g0	0.00	-8.25
GO:00480	apoplast	Cellular C	0.00	0.00	Solyc03g1	Solyc03g1	0.10	-3.35
GO:00480	apoplast	Cellular C	0.00	0.00	Solyc09g0	Solyc09g0	0.49	-1.04
GO:00480	apoplast	Cellular C	0.00	0.00	Solyc09g0	Solyc09g0	0.39	-1.34
GO:00480	apoplast	Cellular C	0.00	0.00	Solyc02g0	Solyc02g0	0.20	-2.30
GO:00095	photosyste	Cellular C	0.00	0.00	Solyc09g0	Solyc09g0	2.11	1.07
GO:00095	photosyste	Cellular C	0.00	0.00	Solyc02g0	PSBO	0.48	-1.07
GO:00095	photosyste	Cellular C	0.00	0.00	Solyc02g0	Solyc02g0	0.35	-1.53
GO:00095	photosyste	Cellular C	0.00	0.00	Solyc03g0	Solyc03g0	0.18	-2.46
GO:00095	photosyste	Cellular C	0.00	0.00	Solyc10g0	Solyc10g0	0.43	-1.23
GO:00095	photosyste	Cellular C	0.00	0.00	Solyc09g0	Solyc09g0	0.43	-1.22
GO:00095	photosyste	Cellular C	0.00	0.00	Solyc05g0	Solyc05g0	0.15	-2.75
GO:00095	photosyste	Cellular C	0.00	0.00	Solyc10g0	Solyc10g0	0.49	-1.02
GO:00095	photosyste	Cellular C	0.00	0.00	Solyc10g0	Solyc10g0	0.00	-11.65
GO:00095	photosyste	Cellular C	0.00	0.00	Solyc03g0	Solyc03g0	0.45	-1.15

GO:00095	photosyste	Cellular Co	0.00	0.00	Solyc05g0	Solyc05g0	0.00	-11.15
GO:00095	photosyste	Cellular Co	0.00	0.00	Solyc06g0	Solyc06g0	0.31	-1.71
GO:00095	photosyste	Cellular Co	0.00	0.00	Solyc01g1	CAP10A	0.35	-1.51
GO:00512	dioxygenas	Molecular	0.00	0.00	Solyc07g0	Solyc07g0	0.17	-2.54
GO:00512	dioxygenas	Molecular	0.00	0.00	Solyc02g0	Solyc02g0	0.23	-2.13
GO:00512	dioxygenas	Molecular	0.00	0.00	Solyc02g0	Solyc02g0	0.02	-5.62
GO:00512	dioxygenas	Molecular	0.00	0.00	Solyc01g0	Solyc01g0	4.36	2.12
GO:00512	dioxygenas	Molecular	0.00	0.00	Solyc11g0	Solyc11g0	0.11	-3.13
GO:00512	dioxygenas	Molecular	0.00	0.00	Solyc07g0	NCED1	0.30	-1.72
GO:00512	dioxygenas	Molecular	0.00	0.00	Solyc07g0	Solyc07g0	0.16	-2.62
GO:00512	dioxygenas	Molecular	0.00	0.00	Solyc06g0	Solyc06g0	6.98	2.80
GO:00512	dioxygenas	Molecular	0.00	0.00	Solyc06g0	Solyc06g0	2.82	1.49
GO:00512	dioxygenas	Molecular	0.00	0.00	Solyc09g0	Solyc09g0	0.19	-2.37
GO:00512	dioxygenas	Molecular	0.00	0.00	Solyc12g0	Solyc12g0	10.12	3.34
GO:00512	dioxygenas	Molecular	0.00	0.00	Solyc10g0	Solyc10g0	0.46	-1.12
GO:00512	dioxygenas	Molecular	0.00	0.00	Solyc12g0	Solyc12g0	7.30	2.87
GO:00512	dioxygenas	Molecular	0.00	0.00	Solyc10g0	Solyc10g0	0.46	-1.13
GO:00512	dioxygenas	Molecular	0.00	0.00	Solyc09g0	Solyc09g0	0.11	-3.22
GO:00512	dioxygenas	Molecular	0.00	0.00	Solyc02g0	Solyc02g0	5.03	2.33
GO:00060	cellular glu	Biological	0.00	0.00	Solyc09g0	Solyc09g0	0.15	-2.74
GO:00060	cellular glu	Biological	0.00	0.00	Solyc04g0	Solyc04g0	2.56	1.36
GO:00060	cellular glu	Biological	0.00	0.00	Solyc01g0	XTH1	0.31	-1.69
GO:00060	cellular glu	Biological	0.00	0.00	Solyc12g0	Solyc12g0	4.32	2.11
GO:00060	cellular glu	Biological	0.00	0.00	Solyc05g0	Solyc05g0	3.55	1.83
GO:00060	cellular glu	Biological	0.00	0.00	Solyc03g0	Solyc03g0	4.24	2.08
GO:00060	cellular glu	Biological	0.00	0.00	Solyc03g0	Solyc03g0	3.51	1.81
GO:00060	cellular glu	Biological	0.00	0.00	Solyc09g0	Solyc09g0	0.39	-1.34
GO:00060	cellular glu	Biological	0.00	0.00	Solyc02g0	Solyc02g0	0.20	-2.30
GO:00104	xyloglucan	Biological	0.00	0.00	Solyc09g0	Solyc09g0	0.15	-2.74
GO:00104	xyloglucan	Biological	0.00	0.00	Solyc04g0	Solyc04g0	2.56	1.36
GO:00104	xyloglucan	Biological	0.00	0.00	Solyc01g0	XTH1	0.31	-1.69
GO:00104	xyloglucan	Biological	0.00	0.00	Solyc12g0	Solyc12g0	4.32	2.11
GO:00104	xyloglucan	Biological	0.00	0.00	Solyc05g0	Solyc05g0	3.55	1.83
GO:00104	xyloglucan	Biological	0.00	0.00	Solyc03g0	Solyc03g0	4.24	2.08
GO:00104	xyloglucan	Biological	0.00	0.00	Solyc03g0	Solyc03g0	3.51	1.81
GO:00104	xyloglucan	Biological	0.00	0.00	Solyc09g0	Solyc09g0	0.39	-1.34
GO:00104	xyloglucan	Biological	0.00	0.00	Solyc02g0	Solyc02g0	0.20	-2.30
GO:00167	xyloglucan	Molecular	0.00	0.00	Solyc09g0	Solyc09g0	0.15	-2.74
GO:00167	xyloglucan	Molecular	0.00	0.00	Solyc04g0	Solyc04g0	2.56	1.36
GO:00167	xyloglucan	Molecular	0.00	0.00	Solyc01g0	XTH1	0.31	-1.69
GO:00167	xyloglucan	Molecular	0.00	0.00	Solyc12g0	Solyc12g0	4.32	2.11
GO:00167	xyloglucan	Molecular	0.00	0.00	Solyc05g0	Solyc05g0	3.55	1.83
GO:00167	xyloglucan	Molecular	0.00	0.00	Solyc03g0	Solyc03g0	4.24	2.08
GO:00167	xyloglucan	Molecular	0.00	0.00	Solyc03g0	Solyc03g0	3.51	1.81
GO:00167	xyloglucan	Molecular	0.00	0.00	Solyc09g0	Solyc09g0	0.39	-1.34
GO:00167	xyloglucan	Molecular	0.00	0.00	Solyc02g0	Solyc02g0	0.20	-2.30
GO:00159	photosynth	Biological	0.00	0.00	Solyc02g0	PSBO	0.48	-1.07
GO:00159	photosynth	Biological	0.00	0.00	Solyc02g0	Solyc02g0	0.35	-1.53
GO:00159	photosynth	Biological	0.00	0.00	Solyc03g0	Solyc03g0	0.18	-2.46
GO:00159	photosynth	Biological	0.00	0.00	Solyc03g0	RBCS-2A	0.34	-1.58
GO:00159	photosynth	Biological	0.00	0.00	Solyc10g0	Solyc10g0	0.43	-1.23

GO:00159	photosynth	Biological	0.00	0.00	Solyc06g0	psaD	0.43	-1.22
GO:00159	photosynth	Biological	0.00	0.00	Solyc09g0	Solyc09g0	0.43	-1.22
GO:00159	photosynth	Biological	0.00	0.00	Solyc06g0	Solyc06g0	0.20	-2.31
GO:00159	photosynth	Biological	0.00	0.00	Solyc05g0	Solyc05g0	0.15	-2.75
GO:00159	photosynth	Biological	0.00	0.00	Solyc10g0	Solyc10g0	0.49	-1.02
GO:00159	photosynth	Biological	0.00	0.00	Solyc08g0	Solyc08g0	0.24	-2.04
GO:00159	photosynth	Biological	0.00	0.00	Solyc06g0	Solyc06g0	0.24	-2.07
GO:00159	photosynth	Biological	0.00	0.00	Solyc10g0	Solyc10g0	0.00	-11.65
GO:00159	photosynth	Biological	0.00	0.00	Solyc08g0	Solyc08g0	0.21	-2.23
GO:00159	photosynth	Biological	0.00	0.00	Solyc03g0	Solyc03g0	0.45	-1.15
GO:00159	photosynth	Biological	0.00	0.00	Solyc06g0	Solyc06g0	0.31	-1.71
GO:00159	photosynth	Biological	0.00	0.00	Solyc01g1	CAP10A	0.35	-1.51
GO:00159	photosynth	Biological	0.00	0.00	Solyc06g0	Solyc06g0	0.06	-4.04
GO:00159	photosynth	Biological	0.00	0.00	Solyc05g0	Solyc05g0	0.46	-1.12
GO:00159	photosynth	Biological	0.00	0.00	Solyc02g0	Solyc02g0	0.43	-1.20
GO:00109	negative re	Biological	0.00	0.00	Solyc08g0	Solyc08g0	0.19	-2.36
GO:00109	negative re	Biological	0.00	0.00	Solyc03g0	Solyc03g0	2.00	1.00
GO:00109	negative re	Biological	0.00	0.00	Solyc11g0	Solyc11g0	4.18	2.06
GO:00109	negative re	Biological	0.00	0.00	Solyc11g0	Solyc11g0	0.46	-1.12
GO:00109	negative re	Biological	0.00	0.00	Solyc09g0	Solyc09g0	33.35	5.06
GO:00109	negative re	Biological	0.00	0.00	Solyc07g0	Solyc07g0	2.42	1.28
GO:00095	chloroplast	Cellular Co	0.00	0.00	Solyc09g0	Solyc09g0	2.11	1.07
GO:00095	chloroplast	Cellular Co	0.00	0.00	Solyc11g0	AOS2	0.12	-3.01
GO:00095	chloroplast	Cellular Co	0.00	0.00	Solyc07g0	NCED1	0.30	-1.72
GO:00095	chloroplast	Cellular Co	0.00	0.00	Solyc02g0	PSBO	0.48	-1.07
GO:00095	chloroplast	Cellular Co	0.00	0.00	Solyc03g0	Solyc03g0	0.18	-2.46
GO:00095	chloroplast	Cellular Co	0.00	0.00	Solyc10g0	Solyc10g0	0.43	-1.23
GO:00095	chloroplast	Cellular Co	0.00	0.00	Solyc06g0	psaD	0.43	-1.22
GO:00095	chloroplast	Cellular Co	0.00	0.00	Solyc06g0	Solyc06g0	0.20	-2.31
GO:00095	chloroplast	Cellular Co	0.00	0.00	Solyc05g0	Solyc05g0	0.15	-2.75
GO:00095	chloroplast	Cellular Co	0.00	0.00	Solyc10g0	Solyc10g0	0.49	-1.02
GO:00095	chloroplast	Cellular Co	0.00	0.00	Solyc05g0	Solyc05g0	0.31	-1.69
GO:00095	chloroplast	Cellular Co	0.00	0.00	Solyc10g0	Solyc10g0	0.00	-11.65
GO:00095	chloroplast	Cellular Co	0.00	0.00	Solyc03g0	Solyc03g0	0.45	-1.15
GO:00095	chloroplast	Cellular Co	0.00	0.00	Solyc05g0	Solyc05g0	0.42	-1.25
GO:00095	chloroplast	Cellular Co	0.00	0.00	Solyc05g0	Solyc05g0	0.00	-11.15
GO:00095	chloroplast	Cellular Co	0.00	0.00	Solyc11g0	Solyc11g0	0.33	-1.60
GO:00095	chloroplast	Cellular Co	0.00	0.00	Solyc06g0	Solyc06g0	0.31	-1.71
GO:00095	chloroplast	Cellular Co	0.00	0.00	Solyc01g1	CAP10A	0.35	-1.51
GO:00095	chloroplast	Cellular Co	0.00	0.00	Solyc10g0	Solyc10g0	0.48	-1.05
GO:00095	chloroplast	Cellular Co	0.00	0.00	Solyc06g0	Solyc06g0	0.06	-4.04
GO:00095	chloroplast	Cellular Co	0.00	0.00	Solyc03g0	Solyc03g0	0.34	-1.57
GO:00095	chloroplast	Cellular Co	0.00	0.00	Solyc09g0	Solyc09g0	0.24	-2.04
GO:00095	chloroplast	Cellular Co	0.00	0.00	Solyc12g0	Solyc12g0	0.00	-10.26
GO:00059	carbohydra	Biological	0.00	0.00	Solyc05g0	Solyc05g0	58.00	5.86
GO:00059	carbohydra	Biological	0.00	0.00	Solyc04g0	Solyc04g0	0.31	-1.70
GO:00059	carbohydra	Biological	0.00	0.00	Solyc01g1	Solyc01g1	2.32	1.21
GO:00059	carbohydra	Biological	0.00	0.00	Solyc03g1	Solyc03g1	2.21	1.14
GO:00059	carbohydra	Biological	0.00	0.00	Solyc10g0	Solyc10g0	0.06	-4.12
GO:00059	carbohydra	Biological	0.00	0.00	Solyc02g0	CHI3	0.24	-2.08
GO:00059	carbohydra	Biological	0.00	0.00	Solyc07g0	Solyc07g0	0.48	-1.07

GO:00059	carbohydra	Biological	0.00	0.00	Solyc09g0	Solyc09g0	0.15	-2.74
GO:00059	carbohydra	Biological	0.00	0.00	Solyc07g0	Solyc07g0	0.49	-1.04
GO:00059	carbohydra	Biological	0.00	0.00	Solyc05g0	Solyc05g0	0.37	-1.44
GO:00059	carbohydra	Biological	0.00	0.00	Solyc07g0	Solyc07g0	0.03	-5.23
GO:00059	carbohydra	Biological	0.00	0.00	Solyc04g0	Solyc04g0	2.56	1.36
GO:00059	carbohydra	Biological	0.00	0.00	Solyc01g0	Solyc01g0	0.04	-4.82
GO:00059	carbohydra	Biological	0.00	0.00	Solyc11g0	Solyc11g0	2.10	1.07
GO:00059	carbohydra	Biological	0.00	0.00	Solyc05g0	Solyc05g0	0.37	-1.42
GO:00059	carbohydra	Biological	0.00	0.00	Solyc03g0	Solyc03g0	2.58	1.37
GO:00059	carbohydra	Biological	0.00	0.00	Solyc07g0	Solyc07g0	2.29	1.19
GO:00059	carbohydra	Biological	0.00	0.00	Solyc01g0	XTH1	0.31	-1.69
GO:00059	carbohydra	Biological	0.00	0.00	Solyc12g0	Solyc12g0	4.32	2.11
GO:00059	carbohydra	Biological	0.00	0.00	Solyc09g0	Solyc09g0	0.39	-1.37
GO:00059	carbohydra	Biological	0.00	0.00	Solyc05g0	Solyc05g0	3.55	1.83
GO:00059	carbohydra	Biological	0.00	0.00	Solyc08g0	Solyc08g0	2.07	1.05
GO:00059	carbohydra	Biological	0.00	0.00	Solyc11g0	Solyc11g0	0.33	-1.59
GO:00059	carbohydra	Biological	0.00	0.00	Solyc12g0	Solyc12g0	0.44	-1.19
GO:00059	carbohydra	Biological	0.00	0.00	Solyc03g0	Solyc03g0	4.24	2.08
GO:00059	carbohydra	Biological	0.00	0.00	Solyc05g0	Solyc05g0	0.26	-1.92
GO:00059	carbohydra	Biological	0.00	0.00	Solyc10g0	CHI9	0.27	-1.88
GO:00059	carbohydra	Biological	0.00	0.00	Solyc04g0	Solyc04g0	4.34	2.12
GO:00059	carbohydra	Biological	0.00	0.00	Solyc12g0	Solyc12g0	3.12	1.64
GO:00059	carbohydra	Biological	0.00	0.00	Solyc03g0	Solyc03g0	3.51	1.81
GO:00059	carbohydra	Biological	0.00	0.00	Solyc11g0	Solyc11g0	2.41	1.27
GO:00059	carbohydra	Biological	0.00	0.00	Solyc11g0	Solyc11g0	2.71	1.44
GO:00059	carbohydra	Biological	0.00	0.00	Solyc11g0	Solyc11g0	0.33	-1.61
GO:00059	carbohydra	Biological	0.00	0.00	Solyc01g0	Solyc01g0	0.02	-5.46
GO:00059	carbohydra	Biological	0.00	0.00	Solyc02g0	Solyc02g0	0.45	-1.14
GO:00059	carbohydra	Biological	0.00	0.00	Solyc07g0	Solyc07g0	0.45	-1.16
GO:00059	carbohydra	Biological	0.00	0.00	Solyc12g0	Solyc12g0	0.00	-9.73
GO:00059	carbohydra	Biological	0.00	0.00	Solyc11g0	Solyc11g0	0.04	-4.78
GO:00059	carbohydra	Biological	0.00	0.00	Solyc03g1	Solyc03g1	0.34	-1.57
GO:00059	carbohydra	Biological	0.00	0.00	Solyc11g0	Solyc11g0	0.42	-1.25
GO:00059	carbohydra	Biological	0.00	0.00	Solyc11g0	Solyc11g0	482.30	8.91
GO:00059	carbohydra	Biological	0.00	0.00	Solyc03g0	Solyc03g0	5.49	2.46
GO:00059	carbohydra	Biological	0.00	0.00	Solyc09g0	Solyc09g0	0.39	-1.34
GO:00059	carbohydra	Biological	0.00	0.00	Solyc02g0	Solyc02g0	0.20	-2.30
GO:00425	cell wall b	Biological	0.00	0.00	Solyc09g0	Solyc09g0	0.15	-2.74
GO:00425	cell wall b	Biological	0.00	0.00	Solyc04g0	Solyc04g0	2.56	1.36
GO:00425	cell wall b	Biological	0.00	0.00	Solyc01g0	XTH1	0.31	-1.69
GO:00425	cell wall b	Biological	0.00	0.00	Solyc12g0	Solyc12g0	4.32	2.11
GO:00425	cell wall b	Biological	0.00	0.00	Solyc05g0	Solyc05g0	3.55	1.83
GO:00425	cell wall b	Biological	0.00	0.00	Solyc03g0	Solyc03g0	4.24	2.08
GO:00425	cell wall b	Biological	0.00	0.00	Solyc03g0	Solyc03g0	3.51	1.81
GO:00425	cell wall b	Biological	0.00	0.00	Solyc09g0	Solyc09g0	0.39	-1.34
GO:00425	cell wall b	Biological	0.00	0.00	Solyc02g0	Solyc02g0	0.20	-2.30
GO:00467	protein aut	Biological	0.00	0.00	Solyc02g0	Solyc02g0	0.47	-1.10
GO:00467	protein aut	Biological	0.00	0.00	Solyc02g0	Solyc02g0	0.42	-1.24
GO:00467	protein aut	Biological	0.00	0.00	Solyc01g1	Solyc01g1	3.45	1.79
GO:00467	protein aut	Biological	0.00	0.00	Solyc03g1	Solyc03g1	0.39	-1.35
GO:00467	protein aut	Biological	0.00	0.00	Solyc11g0	Solyc11g0	0.48	-1.06

GO:00467	protein aut	Biological	0.00	0.00	Solyc03g0	Solyc03g0	3.70	1.89
GO:00467	protein aut	Biological	0.00	0.00	Solyc08g0	Solyc08g0	0.39	-1.34
GO:00467	protein aut	Biological	0.00	0.00	Solyc06g0	Solyc06g0	0.40	-1.31
GO:00467	protein aut	Biological	0.00	0.00	Solyc09g0	Solyc09g0	0.39	-1.35
GO:00467	protein aut	Biological	0.00	0.00	Solyc11g0	Solyc11g0	0.46	-1.11
GO:00467	protein aut	Biological	0.00	0.00	Solyc07g0	Solyc07g0	0.25	-1.97
GO:00467	protein aut	Biological	0.00	0.00	Solyc08g0	Solyc08g0	2.48	1.31
GO:00467	protein aut	Biological	0.00	0.00	Solyc12g0	Solyc12g0	0.09	-3.42
GO:00467	protein aut	Biological	0.00	0.00	Solyc02g0	Solyc02g0	0.45	-1.15
GO:00467	protein aut	Biological	0.00	0.00	Solyc06g0	Solyc06g0	0.26	-1.94
GO:00467	protein aut	Biological	0.00	0.00	Solyc06g0	Solyc06g0	0.41	-1.27
GO:00305	structural c	Molecular	0.00	0.00	Solyc01g0	Solyc01g0	3.68	1.88
GO:00305	structural c	Molecular	0.00	0.00	Solyc01g0	Solyc01g0	2.65	1.41
GO:00305	structural c	Molecular	0.00	0.00	Solyc04g0	Solyc04g0	2.55	1.35
GO:00305	structural c	Molecular	0.00	0.00	Solyc11g0	Solyc11g0	2.07	1.05
GO:00305	structural c	Molecular	0.00	0.00	Solyc06g0	Solyc06g0	2.05	1.04
GO:00305	structural c	Molecular	0.00	0.00	Solyc01g0	Solyc01g0	2.64	1.40
GO:00305	structural c	Molecular	0.00	0.00	Solyc05g0	Solyc05g0	2.73	1.45
GO:00305	structural c	Molecular	0.00	0.00	Solyc01g0	Solyc01g0	2.88	1.52
GO:00063	nucleosom	Biological	0.00	0.00	Solyc02g0	Solyc02g0	11.83	3.56
GO:00063	nucleosom	Biological	0.00	0.00	Solyc01g0	Solyc01g0	2.65	1.41
GO:00063	nucleosom	Biological	0.00	0.00	Solyc04g0	Solyc04g0	2.55	1.35
GO:00063	nucleosom	Biological	0.00	0.00	Solyc11g0	Solyc11g0	2.07	1.05
GO:00063	nucleosom	Biological	0.00	0.00	Solyc01g0	Solyc01g0	2.23	1.16
GO:00063	nucleosom	Biological	0.00	0.00	Solyc06g0	Solyc06g0	2.05	1.04
GO:00063	nucleosom	Biological	0.00	0.00	Solyc01g0	Solyc01g0	2.64	1.40
GO:00063	nucleosom	Biological	0.00	0.00	Solyc11g0	Solyc11g0	2.46	1.30
GO:00063	nucleosom	Biological	0.00	0.00	Solyc05g0	Solyc05g0	2.73	1.45
GO:00063	nucleosom	Biological	0.00	0.00	Solyc01g0	Solyc01g0	2.88	1.52
GO:00063	nucleosom	Biological	0.00	0.00	Solyc12g0	Solyc12g0	2.57	1.36
GO:00095	photosyste	Cellular Co	0.00	0.01	Solyc06g0	psaD	0.43	-1.22
GO:00095	photosyste	Cellular Co	0.00	0.01	Solyc06g0	Solyc06g0	0.20	-2.31
GO:00095	photosyste	Cellular Co	0.00	0.01	Solyc06g0	Solyc06g0	0.24	-2.07
GO:00095	photosyste	Cellular Co	0.00	0.01	Solyc06g0	Solyc06g0	0.06	-4.04
GO:00095	photosyste	Cellular Co	0.00	0.01	Solyc02g0	Solyc02g0	0.43	-1.20
GO:00468	metal ion b	Molecular	0.00	0.01	Solyc07g0	Solyc07g0	0.17	-2.54
GO:00468	metal ion b	Molecular	0.00	0.01	Solyc02g0	Solyc02g0	0.23	-2.13
GO:00468	metal ion b	Molecular	0.00	0.01	Solyc12g0	Solyc12g0	0.28	-1.84
GO:00468	metal ion b	Molecular	0.00	0.01	Solyc02g0	Solyc02g0	0.02	-5.62
GO:00468	metal ion b	Molecular	0.00	0.01	Solyc11g0	AOS2	0.12	-3.01
GO:00468	metal ion b	Molecular	0.00	0.01	Solyc01g0	Solyc01g0	4.36	2.12
GO:00468	metal ion b	Molecular	0.00	0.01	Solyc11g0	Solyc11g0	0.11	-3.13
GO:00468	metal ion b	Molecular	0.00	0.01	Solyc09g0	Solyc09g0	0.27	-1.88
GO:00468	metal ion b	Molecular	0.00	0.01	Solyc01g0	Solyc01g0	2.27	1.18
GO:00468	metal ion b	Molecular	0.00	0.01	Solyc07g0	Solyc07g0	0.47	-1.09
GO:00468	metal ion b	Molecular	0.00	0.01	Solyc07g0	NCED1	0.30	-1.72
GO:00468	metal ion b	Molecular	0.00	0.01	Solyc06g0	Solyc06g0	0.24	-2.05
GO:00468	metal ion b	Molecular	0.00	0.01	Solyc06g0	Solyc06g0	25.52	4.67
GO:00468	metal ion b	Molecular	0.00	0.01	Solyc07g0	Solyc07g0	0.16	-2.62
GO:00468	metal ion b	Molecular	0.00	0.01	Solyc03g1	Solyc03g1	0.11	-3.23
GO:00468	metal ion b	Molecular	0.00	0.01	Solyc01g1	Solyc01g1	0.46	-1.13

GO:00468	metal ion t	Molecular	0.00	0.01	Solyc03g0	Solyc03g0	0.18	-2.46
GO:00468	metal ion t	Molecular	0.00	0.01	Solyc08g0	Solyc08g0	0.19	-2.37
GO:00468	metal ion t	Molecular	0.00	0.01	Solyc10g0	Solyc10g0	0.43	-1.23
GO:00468	metal ion t	Molecular	0.00	0.01	Solyc07g0	Solyc07g0	0.08	-3.60
GO:00468	metal ion t	Molecular	0.00	0.01	Solyc04g0	Solyc04g0	0.27	-1.90
GO:00468	metal ion t	Molecular	0.00	0.01	Solyc08g0	CYP707A1	0.26	-1.93
GO:00468	metal ion t	Molecular	0.00	0.01	Solyc07g0	Solyc07g0	0.41	-1.28
GO:00468	metal ion t	Molecular	0.00	0.01	Solyc01g0	Solyc01g0	0.25	-1.99
GO:00468	metal ion t	Molecular	0.00	0.01	Solyc02g0	Solyc02g0	0.37	-1.43
GO:00468	metal ion t	Molecular	0.00	0.01	Solyc12g0	Solyc12g0	2.53	1.34
GO:00468	metal ion t	Molecular	0.00	0.01	Solyc06g0	Solyc06g0	0.22	-2.19
GO:00468	metal ion t	Molecular	0.00	0.01	Solyc11g0	Solyc11g0	9.10	3.19
GO:00468	metal ion t	Molecular	0.00	0.01	Solyc06g0	Solyc06g0	0.49	-1.03
GO:00468	metal ion t	Molecular	0.00	0.01	Solyc06g0	Solyc06g0	2.82	1.49
GO:00468	metal ion t	Molecular	0.00	0.01	Solyc09g0	Solyc09g0	0.19	-2.37
GO:00468	metal ion t	Molecular	0.00	0.01	Solyc05g0	Solyc05g0	0.15	-2.75
GO:00468	metal ion t	Molecular	0.00	0.01	Solyc06g0	Solyc06g0	2.41	1.27
GO:00468	metal ion t	Molecular	0.00	0.01	Solyc03g0	Solyc03g0	0.29	-1.79
GO:00468	metal ion t	Molecular	0.00	0.01	Solyc07g0	Solyc07g0	3.56	1.83
GO:00468	metal ion t	Molecular	0.00	0.01	Solyc04g0	MTA	0.42	-1.24
GO:00468	metal ion t	Molecular	0.00	0.01	Solyc10g0	Solyc10g0	0.43	-1.22
GO:00468	metal ion t	Molecular	0.00	0.01	Solyc01g0	Solyc01g0	0.38	-1.39
GO:00468	metal ion t	Molecular	0.00	0.01	Solyc10g0	Solyc10g0	0.49	-1.02
GO:00468	metal ion t	Molecular	0.00	0.01	Solyc11g0	Solyc11g0	10.65	3.41
GO:00468	metal ion t	Molecular	0.00	0.01	Solyc05g0	Solyc05g0	0.26	-1.96
GO:00468	metal ion t	Molecular	0.00	0.01	Solyc06g0	Solyc06g0	0.18	-2.46
GO:00468	metal ion t	Molecular	0.00	0.01	Solyc05g0	Solyc05g0	0.31	-1.69
GO:00468	metal ion t	Molecular	0.00	0.01	Solyc12g0	Solyc12g0	10.12	3.34
GO:00468	metal ion t	Molecular	0.00	0.01	Solyc12g0	Solyc12g0	5.61	2.49
GO:00468	metal ion t	Molecular	0.00	0.01	Solyc05g0	Solyc05g0	2.08	1.06
GO:00468	metal ion t	Molecular	0.00	0.01	Solyc05g0	Solyc05g0	2.31	1.21
GO:00468	metal ion t	Molecular	0.00	0.01	Solyc03g0	Solyc03g0	2.30	1.20
GO:00468	metal ion t	Molecular	0.00	0.01	Solyc12g0	Solyc12g0	0.35	-1.50
GO:00468	metal ion t	Molecular	0.00	0.01	Solyc12g0	Solyc12g0	2112.44	11.04
GO:00468	metal ion t	Molecular	0.00	0.01	Solyc04g0	Solyc04g0	0.30	-1.74
GO:00468	metal ion t	Molecular	0.00	0.01	Solyc10g0	Solyc10g0	0.32	-1.63
GO:00468	metal ion t	Molecular	0.00	0.01	Solyc06g0	Solyc06g0	0.49	-1.02
GO:00468	metal ion t	Molecular	0.00	0.01	Solyc02g0	Solyc02g0	3.01	1.59
GO:00468	metal ion t	Molecular	0.00	0.01	Solyc10g0	Solyc10g0	0.46	-1.12
GO:00468	metal ion t	Molecular	0.00	0.01	Solyc05g0	Solyc05g0	49.27	5.62
GO:00468	metal ion t	Molecular	0.00	0.01	Solyc03g0	Solyc03g0	0.45	-1.15
GO:00468	metal ion t	Molecular	0.00	0.01	Solyc09g0	Solyc09g0	2.43	1.28
GO:00468	metal ion t	Molecular	0.00	0.01	Solyc10g0	Solyc10g0	0.46	-1.13
GO:00468	metal ion t	Molecular	0.00	0.01	Solyc10g0	Solyc10g0	0.40	-1.31
GO:00468	metal ion t	Molecular	0.00	0.01	Solyc04g0	Solyc04g0	0.13	-2.98
GO:00468	metal ion t	Molecular	0.00	0.01	Solyc01g1	Solyc01g1	2.95	1.56
GO:00468	metal ion t	Molecular	0.00	0.01	Solyc07g0	Solyc07g0	0.18	-2.47
GO:00468	metal ion t	Molecular	0.00	0.01	Solyc07g0	Solyc07g0	0.47	-1.08
GO:00468	metal ion t	Molecular	0.00	0.01	Solyc06g0	Solyc06g0	896.80	9.81
GO:00468	metal ion t	Molecular	0.00	0.01	Solyc02g0	Solyc02g0	2.19	1.13
GO:00468	metal ion t	Molecular	0.00	0.01	Solyc03g0	Solyc03g0	2.14	1.10

GO:00468	metal ion t	Molecular	0.00	0.01	Solyc06g0	Solyc06g0	9.98	3.32
GO:00468	metal ion t	Molecular	0.00	0.01	Solyc02g0	Solyc02g0	2.29	1.19
GO:00468	metal ion t	Molecular	0.00	0.01	Solyc05g0	Solyc05g0	0.43	-1.23
GO:00468	metal ion t	Molecular	0.00	0.01	Solyc02g0	Solyc02g0	0.10	-3.34
GO:00468	metal ion t	Molecular	0.00	0.01	Solyc09g0	Solyc09g0	0.11	-3.22
GO:00468	metal ion t	Molecular	0.00	0.01	Solyc08g0	Solyc08g0	0.15	-2.74
GO:00468	metal ion t	Molecular	0.00	0.01	Solyc03g0	Solyc03g0	0.34	-1.57
GO:00468	metal ion t	Molecular	0.00	0.01	Solyc02g0	Solyc02g0	5.03	2.33
GO:00468	metal ion t	Molecular	0.00	0.01	Solyc09g0	Solyc09g0	0.00	-8.38
GO:00468	metal ion t	Molecular	0.00	0.01	Solyc01g0	Solyc01g0	3.58	1.84
GO:00468	metal ion t	Molecular	0.00	0.01	Solyc12g0	Solyc12g0	0.00	-10.45
GO:00167	oxidoreduc	Molecular	0.00	0.01	Solyc01g0	Solyc01g0	0.27	-1.87
GO:00167	oxidoreduc	Molecular	0.00	0.01	Solyc12g0	Solyc12g0	0.31	-1.70
GO:00167	oxidoreduc	Molecular	0.00	0.01	Solyc04g0	Solyc04g0	0.00	-10.62
GO:00167	oxidoreduc	Molecular	0.00	0.01	Solyc12g1	Solyc12g1	0.00	-10.37
GO:00167	oxidoreduc	Molecular	0.00	0.01	Solyc12g0	Solyc12g0	0.00	-9.93
GO:00056	cell wall	Cellular Co	0.00	0.01	Solyc03g1	Solyc03g1	2.21	1.14
GO:00056	cell wall	Cellular Co	0.00	0.01	Solyc09g0	Solyc09g0	0.15	-2.74
GO:00056	cell wall	Cellular Co	0.00	0.01	Solyc04g0	Solyc04g0	2.56	1.36
GO:00056	cell wall	Cellular Co	0.00	0.01	Solyc01g0	Solyc01g0	0.33	-1.59
GO:00056	cell wall	Cellular Co	0.00	0.01	Solyc03g0	Solyc03g0	2.58	1.37
GO:00056	cell wall	Cellular Co	0.00	0.01	Solyc09g0	Solyc09g0	4.75	2.25
GO:00056	cell wall	Cellular Co	0.00	0.01	Solyc01g0	XTH1	0.31	-1.69
GO:00056	cell wall	Cellular Co	0.00	0.01	Solyc02g0	Solyc02g0	2.19	1.13
GO:00056	cell wall	Cellular Co	0.00	0.01	Solyc05g0	Solyc05g0	3.55	1.83
GO:00056	cell wall	Cellular Co	0.00	0.01	Solyc03g0	Solyc03g0	4.24	2.08
GO:00056	cell wall	Cellular Co	0.00	0.01	Solyc10g0	CHI9	0.27	-1.88
GO:00056	cell wall	Cellular Co	0.00	0.01	Solyc03g0	Solyc03g0	0.28	-1.83
GO:00056	cell wall	Cellular Co	0.00	0.01	Solyc03g0	Solyc03g0	3.51	1.81
GO:00056	cell wall	Cellular Co	0.00	0.01	Solyc01g0	Solyc01g0	4.89	2.29
GO:00056	cell wall	Cellular Co	0.00	0.01	Solyc09g0	Solyc09g0	0.39	-1.34
GO:00056	cell wall	Cellular Co	0.00	0.01	Solyc07g0	Solyc07g0	2.40	1.27
GO:00056	cell wall	Cellular Co	0.00	0.01	Solyc02g0	Solyc02g0	0.20	-2.30
GO:00064	protein pho	Biological	0.00	0.01	Solyc02g0	Solyc02g0	0.14	-2.87
GO:00064	protein pho	Biological	0.00	0.01	Solyc06g0	Solyc06g0	2.42	1.28
GO:00064	protein pho	Biological	0.00	0.01	Solyc01g1	Solyc01g1	0.31	-1.68
GO:00064	protein pho	Biological	0.00	0.01	Solyc06g0	Solyc06g0	0.44	-1.19
GO:00064	protein pho	Biological	0.00	0.01	Solyc11g0	Solyc11g0	0.13	-3.00
GO:00064	protein pho	Biological	0.00	0.01	Solyc02g0	Solyc02g0	0.47	-1.10
GO:00064	protein pho	Biological	0.00	0.01	Solyc08g0	Solyc08g0	0.31	-1.70
GO:00064	protein pho	Biological	0.00	0.01	Solyc03g0	Solyc03g0	0.46	-1.13
GO:00064	protein pho	Biological	0.00	0.01	Solyc02g0	Solyc02g0	0.42	-1.24
GO:00064	protein pho	Biological	0.00	0.01	Solyc02g0	Solyc02g0	0.41	-1.29
GO:00064	protein pho	Biological	0.00	0.01	Solyc02g0	Solyc02g0	0.15	-2.72
GO:00064	protein pho	Biological	0.00	0.01	Solyc01g1	Solyc01g1	3.45	1.79
GO:00064	protein pho	Biological	0.00	0.01	Solyc03g1	Solyc03g1	0.39	-1.35
GO:00064	protein pho	Biological	0.00	0.01	Solyc09g0	Solyc09g0	2.10	1.07
GO:00064	protein pho	Biological	0.00	0.01	Solyc04g0	Solyc04g0	0.39	-1.35
GO:00064	protein pho	Biological	0.00	0.01	Solyc12g0	Solyc12g0	0.33	-1.60
GO:00064	protein pho	Biological	0.00	0.01	Solyc05g0	Solyc05g0	0.46	-1.11
GO:00064	protein pho	Biological	0.00	0.01	Solyc11g0	Solyc11g0	0.48	-1.06

GO:00064	protein ph	Biological	0.00	0.01	Solyc11g0	Solyc11g0	0.49	-1.03
GO:00064	protein ph	Biological	0.00	0.01	Solyc03g0	Solyc03g0	3.70	1.89
GO:00064	protein ph	Biological	0.00	0.01	Solyc04g0	Solyc04g0	0.48	-1.06
GO:00064	protein ph	Biological	0.00	0.01	Solyc03g0	Solyc03g0	2.33	1.22
GO:00064	protein ph	Biological	0.00	0.01	Solyc08g0	Solyc08g0	0.39	-1.34
GO:00064	protein ph	Biological	0.00	0.01	Solyc05g0	Solyc05g0	0.46	-1.12
GO:00064	protein ph	Biological	0.00	0.01	Solyc06g0	Solyc06g0	0.40	-1.31
GO:00064	protein ph	Biological	0.00	0.01	Solyc09g0	Solyc09g0	0.39	-1.35
GO:00064	protein ph	Biological	0.00	0.01	Solyc11g0	Solyc11g0	0.46	-1.11
GO:00064	protein ph	Biological	0.00	0.01	Solyc02g0	Solyc02g0	0.12	-3.00
GO:00064	protein ph	Biological	0.00	0.01	Solyc07g0	Solyc07g0	0.25	-1.97
GO:00064	protein ph	Biological	0.00	0.01	Solyc08g0	Solyc08g0	2.48	1.31
GO:00064	protein ph	Biological	0.00	0.01	Solyc07g0	Solyc07g0	0.02	-5.66
GO:00064	protein ph	Biological	0.00	0.01	Solyc11g0	Solyc11g0	5992.64	12.55
GO:00064	protein ph	Biological	0.00	0.01	Solyc06g0	Solyc06g0	3.73	1.90
GO:00064	protein ph	Biological	0.00	0.01	Solyc11g0	Solyc11g0	2.74	1.45
GO:00064	protein ph	Biological	0.00	0.01	Solyc10g0	Solyc10g0	0.06	-4.05
GO:00064	protein ph	Biological	0.00	0.01	Solyc02g0	Solyc02g0	0.10	-3.33
GO:00064	protein ph	Biological	0.00	0.01	Solyc01g0	Solyc01g0	0.00	-12.11
GO:00064	protein ph	Biological	0.00	0.01	Solyc01g1	Solyc01g1	0.20	-2.34
GO:00064	protein ph	Biological	0.00	0.01	Solyc12g0	Solyc12g0	0.33	-1.58
GO:00064	protein ph	Biological	0.00	0.01	Solyc02g0	Solyc02g0	0.02	-5.39
GO:00064	protein ph	Biological	0.00	0.01	Solyc04g0	Solyc04g0	0.21	-2.26
GO:00064	protein ph	Biological	0.00	0.01	Solyc12g0	Solyc12g0	2.48	1.31
GO:00064	protein ph	Biological	0.00	0.01	Solyc01g0	Solyc01g0	1525.65	10.58
GO:00064	protein ph	Biological	0.00	0.01	Solyc05g0	Solyc05g0	3.41	1.77
GO:00064	protein ph	Biological	0.00	0.01	Solyc07g0	Solyc07g0	0.46	-1.11
GO:00064	protein ph	Biological	0.00	0.01	Solyc02g0	Solyc02g0	0.36	-1.48
GO:00064	protein ph	Biological	0.00	0.01	Solyc02g0	Solyc02g0	8.65	3.11
GO:00064	protein ph	Biological	0.00	0.01	Solyc04g0	Solyc04g0	0.35	-1.51
GO:00064	protein ph	Biological	0.00	0.01	Solyc04g0	Solyc04g0	2.93	1.55
GO:00064	protein ph	Biological	0.00	0.01	Solyc04g0	Solyc04g0	0.48	-1.06
GO:00064	protein ph	Biological	0.00	0.01	Solyc09g0	Solyc09g0	0.33	-1.59
GO:00064	protein ph	Biological	0.00	0.01	Solyc06g0	Solyc06g0	2.93	1.55
GO:00064	protein ph	Biological	0.00	0.01	Solyc07g0	Solyc07g0	2.61	1.38
GO:00064	protein ph	Biological	0.00	0.01	Solyc03g0	Solyc03g0	0.48	-1.05
GO:00064	protein ph	Biological	0.00	0.01	Solyc02g0	Solyc02g0	0.16	-2.63
GO:00064	protein ph	Biological	0.00	0.01	Solyc11g0	Solyc11g0	0.12	-3.04
GO:00064	protein ph	Biological	0.00	0.01	Solyc05g0	Solyc05g0	0.47	-1.10
GO:00064	protein ph	Biological	0.00	0.01	Solyc02g0	Solyc02g0	0.06	-3.96
GO:00064	protein ph	Biological	0.00	0.01	Solyc04g0	Solyc04g0	2.18	1.13
GO:00064	protein ph	Biological	0.00	0.01	Solyc06g0	Solyc06g0	0.48	-1.07
GO:00064	protein ph	Biological	0.00	0.01	Solyc02g0	Solyc02g0	0.45	-1.15
GO:00064	protein ph	Biological	0.00	0.01	Solyc07g0	Solyc07g0	0.29	-1.79
GO:00064	protein ph	Biological	0.00	0.01	Solyc08g0	Solyc08g0	0.18	-2.44
GO:00064	protein ph	Biological	0.00	0.01	Solyc02g0	Solyc02g0	0.50	-1.01
GO:00064	protein ph	Biological	0.00	0.01	Solyc05g0	Solyc05g0	2.16	1.11
GO:00064	protein ph	Biological	0.00	0.01	Solyc08g0	Solyc08g0	0.19	-2.38
GO:00064	protein ph	Biological	0.00	0.01	Solyc03g0	Solyc03g0	0.00	-8.79
GO:00064	protein ph	Biological	0.00	0.01	Solyc03g0	Solyc03g0	0.01	-7.23
GO:00064	protein ph	Biological	0.00	0.01	Solyc01g0	Solyc01g0	2.66	1.41

GO:00064	protein ph	Biological	0.00	0.01	Solyc10g0	Solyc10g0	0.50	-1.01
GO:00064	protein ph	Biological	0.00	0.01	Solyc02g0	Solyc02g0	0.49	-1.03
GO:00064	protein ph	Biological	0.00	0.01	Solyc12g0	Solyc12g0	0.00	-11.47
GO:00064	protein ph	Biological	0.00	0.01	Solyc09g0	Solyc09g0	0.38	-1.40
GO:00064	protein ph	Biological	0.00	0.01	Solyc08g0	Solyc08g0	0.07	-3.76
GO:00064	protein ph	Biological	0.00	0.01	Solyc09g0	Solyc09g0	0.08	-3.73
GO:00064	protein ph	Biological	0.00	0.01	Solyc06g0	Solyc06g0	0.26	-1.94
GO:00064	protein ph	Biological	0.00	0.01	Solyc01g0	Solyc01g0	2.61	1.39
GO:00064	protein ph	Biological	0.00	0.01	Solyc09g0	Solyc09g0	2.30	1.20
GO:00064	protein ph	Biological	0.00	0.01	Solyc10g0	Solyc10g0	0.42	-1.27
GO:00064	protein ph	Biological	0.00	0.01	Solyc11g0	Solyc11g0	0.00	-9.30
GO:00064	protein ph	Biological	0.00	0.01	Solyc12g0	Solyc12g0	0.21	-2.24
GO:00064	protein ph	Biological	0.00	0.01	Solyc02g0	Solyc02g0	0.44	-1.18
GO:00064	protein ph	Biological	0.00	0.01	Solyc05g0	Solyc05g0	0.00	-8.91
GO:00064	protein ph	Biological	0.00	0.01	Solyc04g0	Solyc04g0	0.00	-8.72
GO:00064	protein ph	Biological	0.00	0.01	Solyc06g0	Solyc06g0	0.41	-1.27
GO:00064	protein ph	Biological	0.00	0.01	Solyc01g0	Solyc01g0	0.33	-1.61
GO:00064	protein ph	Biological	0.00	0.01	Solyc09g0	Solyc09g0	0.22	-2.21
GO:00098	flavonoid l	Biological	0.00	0.01	Solyc09g0	Solyc09g0	0.01	-7.13
GO:00098	flavonoid l	Biological	0.00	0.01	Solyc02g0	Solyc02g0	0.02	-5.62
GO:00098	flavonoid l	Biological	0.00	0.01	Solyc05g0	Solyc05g0	0.01	-6.95
GO:00098	flavonoid l	Biological	0.00	0.01	Solyc11g0	Solyc11g0	0.11	-3.13
GO:00036	DNA repli	Molecular	0.00	0.01	Solyc06g0	Solyc06g0	2.52	1.33
GO:00036	DNA repli	Molecular	0.00	0.01	Solyc05g0	Solyc05g0	2.55	1.35
GO:00036	DNA repli	Molecular	0.00	0.01	Solyc09g0	Solyc09g0	2.25	1.17
GO:00036	DNA repli	Molecular	0.00	0.01	Solyc01g1	Solyc01g1	2.41	1.27
GO:00056	nucleus	Cellular Co	0.00	0.01	Solyc09g0	Solyc09g0	0.07	-3.81
GO:00056	nucleus	Cellular Co	0.00	0.01	Solyc09g0	Solyc09g0	2.30	1.20
GO:00056	nucleus	Cellular Co	0.00	0.01	Solyc11g0	Solyc11g0	0.43	-1.23
GO:00056	nucleus	Cellular Co	0.00	0.01	Solyc01g1	Solyc01g1	0.41	-1.30
GO:00056	nucleus	Cellular Co	0.00	0.01	Solyc06g0	Solyc06g0	0.02	-5.35
GO:00056	nucleus	Cellular Co	0.00	0.01	Solyc01g0	Solyc01g0	0.04	-4.49
GO:00056	nucleus	Cellular Co	0.00	0.01	Solyc01g0	Solyc01g0	3.68	1.88
GO:00056	nucleus	Cellular Co	0.00	0.01	Solyc02g0	Solyc02g0	0.48	-1.05
GO:00056	nucleus	Cellular Co	0.00	0.01	Solyc11g0	Solyc11g0	0.44	-1.18
GO:00056	nucleus	Cellular Co	0.00	0.01	Solyc06g0	Solyc06g0	0.44	-1.19
GO:00056	nucleus	Cellular Co	0.00	0.01	Solyc09g0	Solyc09g0	0.31	-1.67
GO:00056	nucleus	Cellular Co	0.00	0.01	Solyc02g0	Solyc02g0	11.83	3.56
GO:00056	nucleus	Cellular Co	0.00	0.01	Solyc03g1	Solyc03g1	0.30	-1.72
GO:00056	nucleus	Cellular Co	0.00	0.01	Solyc04g0	Solyc04g0	2.92	1.55
GO:00056	nucleus	Cellular Co	0.00	0.01	Solyc04g0	Solyc04g0	0.42	-1.24
GO:00056	nucleus	Cellular Co	0.00	0.01	Solyc01g0	Solyc01g0	2.65	1.41
GO:00056	nucleus	Cellular Co	0.00	0.01	Solyc04g0	Solyc04g0	0.45	-1.14
GO:00056	nucleus	Cellular Co	0.00	0.01	Solyc04g0	Solyc04g0	2.55	1.35
GO:00056	nucleus	Cellular Co	0.00	0.01	Solyc08g0	Solyc08g0	0.50	-1.01
GO:00056	nucleus	Cellular Co	0.00	0.01	Solyc05g0	Solyc05g0	0.08	-3.67
GO:00056	nucleus	Cellular Co	0.00	0.01	Solyc11g0	Solyc11g0	0.19	-2.40
GO:00056	nucleus	Cellular Co	0.00	0.01	Solyc01g0	Solyc01g0	0.25	-1.99
GO:00056	nucleus	Cellular Co	0.00	0.01	Solyc03g1	Solyc03g1	0.04	-4.59
GO:00056	nucleus	Cellular Co	0.00	0.01	Solyc08g0	Solyc08g0	0.50	-1.01
GO:00056	nucleus	Cellular Co	0.00	0.01	Solyc12g0	Solyc12g0	0.44	-1.20

GO:00056	nucleus	Cellular Co	0.00	0.01	Solyc07g0	Solyc07g0	0.40	-1.31
GO:00056	nucleus	Cellular Co	0.00	0.01	Solyc12g0	Solyc12g0	0.32	-1.65
GO:00056	nucleus	Cellular Co	0.00	0.01	Solyc06g0	Solyc06g0	0.34	-1.54
GO:00056	nucleus	Cellular Co	0.00	0.01	Solyc04g0	Solyc04g0	0.13	-2.91
GO:00056	nucleus	Cellular Co	0.00	0.01	Solyc06g0	Solyc06g0	0.21	-2.28
GO:00056	nucleus	Cellular Co	0.00	0.01	Solyc09g0	Solyc09g0	0.48	-1.06
GO:00056	nucleus	Cellular Co	0.00	0.01	Solyc11g0	Solyc11g0	2.07	1.05
GO:00056	nucleus	Cellular Co	0.00	0.01	Solyc01g0	Solyc01g0	2.23	1.16
GO:00056	nucleus	Cellular Co	0.00	0.01	Solyc02g0	Solyc02g0	3.87	1.95
GO:00056	nucleus	Cellular Co	0.00	0.01	Solyc06g0	Solyc06g0	2.52	1.33
GO:00056	nucleus	Cellular Co	0.00	0.01	Solyc05g0	Solyc05g0	3.62	1.86
GO:00056	nucleus	Cellular Co	0.00	0.01	Solyc01g0	Solyc01g0	0.35	-1.51
GO:00056	nucleus	Cellular Co	0.00	0.01	Solyc04g0	Solyc04g0	0.47	-1.09
GO:00056	nucleus	Cellular Co	0.00	0.01	Solyc05g0	Solyc05g0	0.40	-1.33
GO:00056	nucleus	Cellular Co	0.00	0.01	Solyc03g1	Solyc03g1	2.29	1.19
GO:00056	nucleus	Cellular Co	0.00	0.01	Solyc10g0	Solyc10g0	2.92	1.54
GO:00056	nucleus	Cellular Co	0.00	0.01	Solyc06g0	Solyc06g0	2.05	1.04
GO:00056	nucleus	Cellular Co	0.00	0.01	Solyc02g0	Solyc02g0	0.33	-1.60
GO:00056	nucleus	Cellular Co	0.00	0.01	Solyc01g0	Solyc01g0	2.64	1.40
GO:00056	nucleus	Cellular Co	0.00	0.01	Solyc03g0	Solyc03g0	2.59	1.37
GO:00056	nucleus	Cellular Co	0.00	0.01	Solyc01g0	Solyc01g0	0.11	-3.13
GO:00056	nucleus	Cellular Co	0.00	0.01	Solyc06g0	Solyc06g0	0.41	-1.28
GO:00056	nucleus	Cellular Co	0.00	0.01	Solyc08g0	Solyc08g0	0.48	-1.04
GO:00056	nucleus	Cellular Co	0.00	0.01	Solyc01g0	Solyc01g0	0.34	-1.55
GO:00056	nucleus	Cellular Co	0.00	0.01	Solyc11g0	Solyc11g0	2.74	1.45
GO:00056	nucleus	Cellular Co	0.00	0.01	Solyc12g0	Solyc12g0	0.13	-2.97
GO:00056	nucleus	Cellular Co	0.00	0.01	Solyc01g1	Solyc01g1	2.11	1.08
GO:00056	nucleus	Cellular Co	0.00	0.01	Solyc01g0	Solyc01g0	0.00	-12.11
GO:00056	nucleus	Cellular Co	0.00	0.01	Solyc04g0	Solyc04g0	0.46	-1.11
GO:00056	nucleus	Cellular Co	0.00	0.01	Solyc11g0	Solyc11g0	0.48	-1.05
GO:00056	nucleus	Cellular Co	0.00	0.01	Solyc10g0	Solyc10g0	0.00	-13.06
GO:00056	nucleus	Cellular Co	0.00	0.01	Solyc01g1	Solyc01g1	0.44	-1.20
GO:00056	nucleus	Cellular Co	0.00	0.01	Solyc12g0	Solyc12g0	0.35	-1.50
GO:00056	nucleus	Cellular Co	0.00	0.01	Solyc03g0	Solyc03g0	0.40	-1.33
GO:00056	nucleus	Cellular Co	0.00	0.01	Solyc11g0	Solyc11g0	2.46	1.30
GO:00056	nucleus	Cellular Co	0.00	0.01	Solyc01g1	Solyc01g1	3.62	1.85
GO:00056	nucleus	Cellular Co	0.00	0.01	Solyc02g0	Solyc02g0	0.19	-2.41
GO:00056	nucleus	Cellular Co	0.00	0.01	Solyc10g0	Solyc10g0	0.23	-2.10
GO:00056	nucleus	Cellular Co	0.00	0.01	Solyc07g0	Solyc07g0	0.24	-2.07
GO:00056	nucleus	Cellular Co	0.00	0.01	Solyc05g0	Solyc05g0	0.24	-2.05
GO:00056	nucleus	Cellular Co	0.00	0.01	Solyc06g0	Solyc06g0	0.39	-1.36
GO:00056	nucleus	Cellular Co	0.00	0.01	Solyc08g0	Solyc08g0	0.28	-1.83
GO:00056	nucleus	Cellular Co	0.00	0.01	Solyc05g0	Solyc05g0	0.29	-1.78
GO:00056	nucleus	Cellular Co	0.00	0.01	Solyc07g0	Solyc07g0	0.07	-3.80
GO:00056	nucleus	Cellular Co	0.00	0.01	Solyc03g1	Solyc03g1	2.13	1.09
GO:00056	nucleus	Cellular Co	0.00	0.01	Solyc01g0	Solyc01g0	0.35	-1.51
GO:00056	nucleus	Cellular Co	0.00	0.01	Solyc10g0	Solyc10g0	0.32	-1.63
GO:00056	nucleus	Cellular Co	0.00	0.01	Solyc01g1	Solyc01g1	2.41	1.27
GO:00056	nucleus	Cellular Co	0.00	0.01	Solyc05g0	Solyc05g0	0.49	-1.03
GO:00056	nucleus	Cellular Co	0.00	0.01	Solyc05g0	Solyc05g0	2.73	1.45
GO:00056	nucleus	Cellular Co	0.00	0.01	Solyc08g0	Solyc08g0	2.07	1.05

GO:00056	nucleus	Cellular Co	0.00	0.01	Solyc02g0	Solyc02g0	8.65	3.11
GO:00056	nucleus	Cellular Co	0.00	0.01	Solyc01g0	Solyc01g0	2.88	1.52
GO:00056	nucleus	Cellular Co	0.00	0.01	Solyc01g0	Solyc01g0	208.08	7.70
GO:00056	nucleus	Cellular Co	0.00	0.01	Solyc01g0	Solyc01g0	0.50	-1.01
GO:00056	nucleus	Cellular Co	0.00	0.01	Solyc09g0	Solyc09g0	3.39	1.76
GO:00056	nucleus	Cellular Co	0.00	0.01	Solyc03g0	Solyc03g0	0.06	-4.13
GO:00056	nucleus	Cellular Co	0.00	0.01	Solyc12g0	Solyc12g0	2.57	1.36
GO:00056	nucleus	Cellular Co	0.00	0.01	Solyc05g0	Solyc05g0	0.00	-11.04
GO:00056	nucleus	Cellular Co	0.00	0.01	Solyc04g0	Solyc04g0	2.93	1.55
GO:00056	nucleus	Cellular Co	0.00	0.01	Solyc06g0	Solyc06g0	3.49	1.81
GO:00056	nucleus	Cellular Co	0.00	0.01	Solyc10g0	Solyc10g0	0.35	-1.50
GO:00056	nucleus	Cellular Co	0.00	0.01	Solyc03g1	Solyc03g1	0.37	-1.42
GO:00056	nucleus	Cellular Co	0.00	0.01	Solyc03g1	Solyc03g1	0.09	-3.43
GO:00056	nucleus	Cellular Co	0.00	0.01	Solyc08g0	Solyc08g0	0.35	-1.53
GO:00056	nucleus	Cellular Co	0.00	0.01	Solyc05g0	Solyc05g0	2.08	1.06
GO:00056	nucleus	Cellular Co	0.00	0.01	Solyc02g0	Solyc02g0	2.03	1.02
GO:00056	nucleus	Cellular Co	0.00	0.01	Solyc03g1	Solyc03g1	0.44	-1.19
GO:00056	nucleus	Cellular Co	0.00	0.01	Solyc04g0	Solyc04g0	2.19	1.13
GO:00056	nucleus	Cellular Co	0.00	0.01	Solyc06g0	Solyc06g0	0.38	-1.41
GO:00056	nucleus	Cellular Co	0.00	0.01	Solyc08g0	Solyc08g0	9.73	3.28
GO:00056	nucleus	Cellular Co	0.00	0.01	Solyc03g0	Solyc03g0	0.48	-1.05
GO:00056	nucleus	Cellular Co	0.00	0.01	Solyc08g0	Solyc08g0	0.38	-1.40
GO:00056	nucleus	Cellular Co	0.00	0.01	Solyc02g0	Solyc02g0	0.42	-1.25
GO:00056	nucleus	Cellular Co	0.00	0.01	Solyc04g0	Solyc04g0	2.18	1.13
GO:00056	nucleus	Cellular Co	0.00	0.01	Solyc09g0	Solyc09g0	0.37	-1.42
GO:00056	nucleus	Cellular Co	0.00	0.01	Solyc06g0	Solyc06g0	0.48	-1.07
GO:00056	nucleus	Cellular Co	0.00	0.01	Solyc03g0	Solyc03g0	0.29	-1.76
GO:00056	nucleus	Cellular Co	0.00	0.01	Solyc04g0	Solyc04g0	4.79	2.26
GO:00056	nucleus	Cellular Co	0.00	0.01	Solyc10g0	Solyc10g0	0.45	-1.15
GO:00056	nucleus	Cellular Co	0.00	0.01	Solyc12g0	Solyc12g0	0.40	-1.34
GO:00056	nucleus	Cellular Co	0.00	0.01	Solyc02g0	Solyc02g0	0.34	-1.57
GO:00056	nucleus	Cellular Co	0.00	0.01	Solyc02g0	Solyc02g0	0.00	-11.41
GO:00056	nucleus	Cellular Co	0.00	0.01	Solyc07g0	Solyc07g0	0.47	-1.08
GO:00056	nucleus	Cellular Co	0.00	0.01	Solyc11g0	Solyc11g0	5.14	2.36
GO:00056	nucleus	Cellular Co	0.00	0.01	Solyc02g0	Solyc02g0	2.15	1.10
GO:00056	nucleus	Cellular Co	0.00	0.01	Solyc03g1	Solyc03g1	0.08	-3.61
GO:00056	nucleus	Cellular Co	0.00	0.01	Solyc05g0	Solyc05g0	0.00	-8.91
GO:00056	nucleus	Cellular Co	0.00	0.01	Solyc03g0	Solyc03g0	11.59	3.53
GO:00056	nucleus	Cellular Co	0.00	0.01	Solyc10g0	Solyc10g0	0.33	-1.62
GO:00056	nucleus	Cellular Co	0.00	0.01	Solyc01g1	Solyc01g1	0.15	-2.78
GO:00056	nucleus	Cellular Co	0.00	0.01	Solyc10g0	Solyc10g0	2.49	1.31
GO:00056	nucleus	Cellular Co	0.00	0.01	Solyc01g0	Solyc01g0	0.48	-1.06
GO:00056	nucleus	Cellular Co	0.00	0.01	Solyc01g0	Solyc01g0	2.66	1.41
GO:00056	nucleus	Cellular Co	0.00	0.01	Solyc02g0	Solyc02g0	2.19	1.13
GO:00056	nucleus	Cellular Co	0.00	0.01	Solyc10g0	Solyc10g0	0.16	-2.65
GO:00056	nucleus	Cellular Co	0.00	0.01	Solyc10g0	Solyc10g0	1745.96	10.77
GO:00056	nucleus	Cellular Co	0.00	0.01	Solyc02g0	Solyc02g0	0.49	-1.03
GO:00056	nucleus	Cellular Co	0.00	0.01	Solyc04g0	Solyc04g0	0.37	-1.43
GO:00056	nucleus	Cellular Co	0.00	0.01	Solyc03g1	Solyc03g1	0.45	-1.14
GO:00056	nucleus	Cellular Co	0.00	0.01	Solyc05g0	Solyc05g0	0.42	-1.26
GO:00056	nucleus	Cellular Co	0.00	0.01	Solyc05g0	Solyc05g0	0.43	-1.23

GO:00056	nucleus	Cellular Co	0.00	0.01	Solyc06g0	Solyc06g0	0.22	-2.19
GO:00056	nucleus	Cellular Co	0.00	0.01	Solyc02g0	PTI5	0.07	-3.77
GO:00056	nucleus	Cellular Co	0.00	0.01	Solyc06g0	Solyc06g0	0.39	-1.36
GO:00056	nucleus	Cellular Co	0.00	0.01	Solyc03g1	Solyc03g1	0.46	-1.13
GO:00056	nucleus	Cellular Co	0.00	0.01	Solyc05g0	Solyc05g0	0.46	-1.13
GO:00056	nucleus	Cellular Co	0.00	0.01	Solyc10g0	Solyc10g0	0.15	-2.72
GO:00056	nucleus	Cellular Co	0.00	0.01	Solyc04g0	Solyc04g0	0.34	-1.54
GO:00056	nucleus	Cellular Co	0.00	0.01	Solyc06g0	Solyc06g0	0.42	-1.25
GO:00056	nucleus	Cellular Co	0.00	0.01	Solyc04g0	Solyc04g0	0.48	-1.05
GO:00056	nucleus	Cellular Co	0.00	0.01	Solyc06g0	Solyc06g0	3.20	1.68
GO:00056	nucleus	Cellular Co	0.00	0.01	Solyc05g0	Solyc05g0	0.00	-8.91
GO:00056	nucleus	Cellular Co	0.00	0.01	Solyc06g0	Solyc06g0	0.40	-1.30
GO:00056	nucleus	Cellular Co	0.00	0.01	Solyc03g1	Solyc03g1	0.40	-1.33
GO:00056	nucleus	Cellular Co	0.00	0.01	Solyc03g1	Solyc03g1	0.24	-2.05
GO:00056	nucleus	Cellular Co	0.00	0.01	Solyc09g0	Solyc09g0	0.49	-1.04
GO:00056	nucleus	Cellular Co	0.00	0.01	Solyc08g0	Solyc08g0	0.45	-1.17
GO:00056	nucleus	Cellular Co	0.00	0.01	Solyc10g0	Solyc10g0	2.30	1.20
GO:00056	nucleus	Cellular Co	0.00	0.01	Solyc10g0	Solyc10g0	0.35	-1.51
GO:00056	nucleus	Cellular Co	0.00	0.01	Solyc11g0	Solyc11g0	0.00	-9.45
GO:00056	nucleus	Cellular Co	0.00	0.01	Solyc02g0	Solyc02g0	0.23	-2.09
GO:00056	nucleus	Cellular Co	0.00	0.01	Solyc02g0	Solyc02g0	0.00	-9.32
GO:00056	nucleus	Cellular Co	0.00	0.01	Solyc01g0	Solyc01g0	0.42	-1.24
GO:00007	nucleosom	Cellular Co	0.00	0.02	Solyc01g0	Solyc01g0	3.68	1.88
GO:00007	nucleosom	Cellular Co	0.00	0.02	Solyc01g0	Solyc01g0	2.65	1.41
GO:00007	nucleosom	Cellular Co	0.00	0.02	Solyc04g0	Solyc04g0	2.55	1.35
GO:00007	nucleosom	Cellular Co	0.00	0.02	Solyc11g0	Solyc11g0	2.07	1.05
GO:00007	nucleosom	Cellular Co	0.00	0.02	Solyc01g0	Solyc01g0	2.23	1.16
GO:00007	nucleosom	Cellular Co	0.00	0.02	Solyc06g0	Solyc06g0	2.05	1.04
GO:00007	nucleosom	Cellular Co	0.00	0.02	Solyc01g0	Solyc01g0	2.64	1.40
GO:00007	nucleosom	Cellular Co	0.00	0.02	Solyc01g0	Solyc01g0	2.38	1.25
GO:00007	nucleosom	Cellular Co	0.00	0.02	Solyc11g0	Solyc11g0	2.46	1.30
GO:00007	nucleosom	Cellular Co	0.00	0.02	Solyc05g0	Solyc05g0	2.73	1.45
GO:00007	nucleosom	Cellular Co	0.00	0.02	Solyc01g0	Solyc01g0	2.88	1.52
GO:00007	nucleosom	Cellular Co	0.00	0.02	Solyc12g0	Solyc12g0	2.57	1.36
GO:00007	nucleosom	Cellular Co	0.00	0.02	Solyc02g0	Solyc02g0	2.15	1.10
GO:00081	UDP-glyco	Molecular	0.00	0.02	Solyc09g0	Solyc09g0	0.07	-3.81
GO:00081	UDP-glyco	Molecular	0.00	0.02	Solyc09g0	Solyc09g0	0.07	-3.84
GO:00081	UDP-glyco	Molecular	0.00	0.02	Solyc03g0	Solyc03g0	0.47	-1.10
GO:00081	UDP-glyco	Molecular	0.00	0.02	Solyc01g1	Solyc01g1	2.27	1.18
GO:00081	UDP-glyco	Molecular	0.00	0.02	Solyc04g0	Solyc04g0	0.49	-1.04
GO:00081	UDP-glyco	Molecular	0.00	0.02	Solyc04g0	Solyc04g0	0.21	-2.22
GO:00081	UDP-glyco	Molecular	0.00	0.02	Solyc10g0	Solyc10g0	0.37	-1.43
GO:00081	UDP-glyco	Molecular	0.00	0.02	Solyc09g0	Solyc09g0	0.00	-11.83
GO:00081	UDP-glyco	Molecular	0.00	0.02	Solyc12g0	Solyc12g0	2.94	1.56
GO:00081	UDP-glyco	Molecular	0.00	0.02	Solyc03g0	Solyc03g0	0.28	-1.84
GO:00081	UDP-glyco	Molecular	0.00	0.02	Solyc11g0	Solyc11g0	0.38	-1.39
GO:00081	UDP-glyco	Molecular	0.00	0.02	Solyc04g0	Solyc04g0	0.43	-1.23
GO:00081	UDP-glyco	Molecular	0.00	0.02	Solyc03g0	Solyc03g0	0.25	-2.02
GO:00081	UDP-glyco	Molecular	0.00	0.02	Solyc02g0	Solyc02g0	0.11	-3.14
GO:00081	UDP-glyco	Molecular	0.00	0.02	Solyc06g0	Solyc06g0	0.08	-3.70
GO:00081	UDP-glyco	Molecular	0.00	0.02	Solyc01g1	Solyc01g1	0.39	-1.36

GO:00081	UDP-glyc	Molecular	0.00	0.02	Solyc11g0	Solyc11g0	0.49	-1.03
GO:00081	UDP-glyc	Molecular	0.00	0.02	Solyc08g0	Solyc08g0	0.32	-1.65
GO:00081	UDP-glyc	Molecular	0.00	0.02	Solyc10g0	Solyc10g0	3.52	1.82
GO:00097	response t	Biological	0.00	0.02	Solyc02g0	Solyc02g0	0.44	-1.18
GO:00097	response t	Biological	0.00	0.02	Solyc09g0	Solyc09g0	0.07	-3.81
GO:00097	response t	Biological	0.00	0.02	Solyc07g0	NCED1	0.30	-1.72
GO:00097	response t	Biological	0.00	0.02	Solyc06g0	Solyc06g0	0.26	-1.93
GO:00097	response t	Biological	0.00	0.02	Solyc01g1	Solyc01g1	0.47	-1.09
GO:00097	response t	Biological	0.00	0.02	Solyc09g0	Solyc09g0	0.44	-1.17
GO:00097	response t	Biological	0.00	0.02	Solyc03g0	Solyc03g0	0.45	-1.16
GO:00097	response t	Biological	0.00	0.02	Solyc01g0	Solyc01g0	0.42	-1.26
GO:00097	response t	Biological	0.00	0.02	Solyc08g0	CYP707A	0.26	-1.93
GO:00097	response t	Biological	0.00	0.02	Solyc01g0	Solyc01g0	0.45	-1.15
GO:00097	response t	Biological	0.00	0.02	Solyc06g0	Solyc06g0	5812.44	12.50
GO:00097	response t	Biological	0.00	0.02	Solyc03g1	Solyc03g1	0.46	-1.13
GO:00038	catalytic a	Molecular	0.00	0.02	Solyc09g0	Solyc09g0	0.08	-3.59
GO:00038	catalytic a	Molecular	0.00	0.02	Solyc10g0	Solyc10g0	0.03	-5.15
GO:00038	catalytic a	Molecular	0.00	0.02	Solyc05g0	Solyc05g0	0.29	-1.78
GO:00038	catalytic a	Molecular	0.00	0.02	Solyc09g0	Solyc09g0	0.09	-3.50
GO:00038	catalytic a	Molecular	0.00	0.02	Solyc03g0	Solyc03g0	2.69	1.43
GO:00038	catalytic a	Molecular	0.00	0.02	Solyc04g0	Solyc04g0	0.34	-1.56
GO:00038	catalytic a	Molecular	0.00	0.02	Solyc02g0	Solyc02g0	0.45	-1.16
GO:00038	catalytic a	Molecular	0.00	0.02	Solyc07g0	Solyc07g0	0.48	-1.07
GO:00038	catalytic a	Molecular	0.00	0.02	Solyc08g0	Solyc08g0	0.21	-2.27
GO:00038	catalytic a	Molecular	0.00	0.02	Solyc12g0	Solyc12g0	2.21	1.14
GO:00038	catalytic a	Molecular	0.00	0.02	Solyc01g0	Solyc01g0	0.29	-1.78
GO:00038	catalytic a	Molecular	0.00	0.02	Solyc01g0	Solyc01g0	0.47	-1.10
GO:00038	catalytic a	Molecular	0.00	0.02	Solyc08g0	Solyc08g0	0.50	-1.01
GO:00038	catalytic a	Molecular	0.00	0.02	Solyc11g0	Solyc11g0	2.10	1.07
GO:00038	catalytic a	Molecular	0.00	0.02	Solyc09g0	Solyc09g0	0.21	-2.26
GO:00038	catalytic a	Molecular	0.00	0.02	Solyc09g0	Solyc09g0	0.49	-1.02
GO:00038	catalytic a	Molecular	0.00	0.02	Solyc03g0	Solyc03g0	0.46	-1.11
GO:00038	catalytic a	Molecular	0.00	0.02	Solyc09g0	Solyc09g0	0.40	-1.33
GO:00038	catalytic a	Molecular	0.00	0.02	Solyc03g0	Solyc03g0	0.38	-1.38
GO:00038	catalytic a	Molecular	0.00	0.02	Solyc01g0	Solyc01g0	0.31	-1.70
GO:00038	catalytic a	Molecular	0.00	0.02	Solyc11g0	Solyc11g0	0.33	-1.60
GO:00038	catalytic a	Molecular	0.00	0.02	Solyc01g0	Solyc01g0	0.41	-1.28
GO:00038	catalytic a	Molecular	0.00	0.02	Solyc01g1	Solyc01g1	0.31	-1.69
GO:00038	catalytic a	Molecular	0.00	0.02	Solyc10g0	Solyc10g0	0.38	-1.40
GO:00038	catalytic a	Molecular	0.00	0.02	Solyc01g0	Solyc01g0	4.01	2.00
GO:00038	catalytic a	Molecular	0.00	0.02	Solyc07g0	OPR3	0.47	-1.10
GO:00038	catalytic a	Molecular	0.00	0.02	Solyc05g0	Solyc05g0	0.40	-1.31
GO:00038	catalytic a	Molecular	0.00	0.02	Solyc08g0	Solyc08g0	0.11	-3.24
GO:00038	catalytic a	Molecular	0.00	0.02	Solyc12g0	Solyc12g0	0.48	-1.05
GO:00038	catalytic a	Molecular	0.00	0.02	Solyc03g0	Solyc03g0	2.49	1.31
GO:00038	catalytic a	Molecular	0.00	0.02	Solyc02g0	Solyc02g0	40.96	5.36
GO:00038	catalytic a	Molecular	0.00	0.02	Solyc08g0	Solyc08g0	0.47	-1.07
GO:00038	catalytic a	Molecular	0.00	0.02	Solyc12g0	Solyc12g0	0.49	-1.03
GO:00038	catalytic a	Molecular	0.00	0.02	Solyc02g0	Solyc02g0	9.14	3.19
GO:00038	catalytic a	Molecular	0.00	0.02	Solyc01g0	Solyc01g0	7223.16	12.82
GO:00038	catalytic a	Molecular	0.00	0.02	Solyc07g0	Solyc07g0	4.55	2.19

GO:00038	catalytic a	Molecular	0.00	0.02	Solyc07g0	Solyc07g0	3.41	1.77
GO:00038	catalytic a	Molecular	0.00	0.02	Solyc03g0	Solyc03g0	0.00	-11.21
GO:00038	catalytic a	Molecular	0.00	0.02	Solyc02g0	Solyc02g0	0.45	-1.14
GO:00038	catalytic a	Molecular	0.00	0.02	Solyc01g1	Solyc01g1	2.42	1.28
GO:00038	catalytic a	Molecular	0.00	0.02	Solyc12g0	Solyc12g0	0.30	-1.74
GO:00038	catalytic a	Molecular	0.00	0.02	Solyc08g0	Solyc08g0	0.00	-10.99
GO:00038	catalytic a	Molecular	0.00	0.02	Solyc08g0	Solyc08g0	0.05	-4.43
GO:00038	catalytic a	Molecular	0.00	0.02	Solyc01g1	Solyc01g1	0.48	-1.05
GO:00038	catalytic a	Molecular	0.00	0.02	Solyc03g0	Solyc03g0	0.27	-1.86
GO:00038	catalytic a	Molecular	0.00	0.02	Solyc11g0	Solyc11g0	0.38	-1.39
GO:00038	catalytic a	Molecular	0.00	0.02	Solyc08g0	Solyc08g0	799.01	9.64
GO:00038	catalytic a	Molecular	0.00	0.02	Solyc02g0	Solyc02g0	2.19	1.13
GO:00038	catalytic a	Molecular	0.00	0.02	Solyc08g0	Solyc08g0	0.07	-3.82
GO:00038	catalytic a	Molecular	0.00	0.02	Solyc02g0	Solyc02g0	7.41	2.89
GO:00038	catalytic a	Molecular	0.00	0.02	Solyc08g0	Solyc08g0	0.17	-2.53
GO:00038	catalytic a	Molecular	0.00	0.02	Solyc04g0	Solyc04g0	0.43	-1.23
GO:00038	catalytic a	Molecular	0.00	0.02	Solyc06g0	Solyc06g0	0.00	-9.59
GO:00038	catalytic a	Molecular	0.00	0.02	Solyc03g0	Solyc03g0	5.49	2.46
GO:00038	catalytic a	Molecular	0.00	0.02	Solyc08g0	Solyc08g0	2.74	1.45
GO:00038	catalytic a	Molecular	0.00	0.02	Solyc01g0	Solyc01g0	0.23	-2.09
GO:00038	catalytic a	Molecular	0.00	0.02	Solyc01g0	Solyc01g0	0.43	-1.23
GO:00038	catalytic a	Molecular	0.00	0.02	Solyc04g0	Solyc04g0	0.28	-1.84
GO:00038	catalytic a	Molecular	0.00	0.02	Solyc06g0	Solyc06g0	379.31	8.57
GO:00038	catalytic a	Molecular	0.00	0.02	Solyc01g1	Solyc01g1	0.47	-1.09
GO:00062	DNA repli	Biological	0.00	0.02	Solyc06g0	Solyc06g0	2.52	1.33
GO:00062	DNA repli	Biological	0.00	0.02	Solyc08g0	Solyc08g0	2.48	1.31
GO:00062	DNA repli	Biological	0.00	0.02	Solyc05g0	Solyc05g0	2.55	1.35
GO:00062	DNA repli	Biological	0.00	0.02	Solyc09g0	Solyc09g0	2.25	1.17
GO:00062	DNA repli	Biological	0.00	0.02	Solyc01g1	Solyc01g1	2.41	1.27
GO:00036	DNA bind	Molecular	0.00	0.02	Solyc06g0	Solyc06g0	3.24	1.69
GO:00036	DNA bind	Molecular	0.00	0.02	Solyc01g0	Solyc01g0	3.68	1.88
GO:00036	DNA bind	Molecular	0.00	0.02	Solyc02g0	Solyc02g0	0.48	-1.05
GO:00036	DNA bind	Molecular	0.00	0.02	Solyc09g0	Solyc09g0	0.31	-1.67
GO:00036	DNA bind	Molecular	0.00	0.02	Solyc04g0	Solyc04g0	2.92	1.55
GO:00036	DNA bind	Molecular	0.00	0.02	Solyc09g0	Solyc09g0	3.07	1.62
GO:00036	DNA bind	Molecular	0.00	0.02	Solyc04g0	Solyc04g0	0.42	-1.24
GO:00036	DNA bind	Molecular	0.00	0.02	Solyc01g0	Solyc01g0	2.65	1.41
GO:00036	DNA bind	Molecular	0.00	0.02	Solyc04g0	Solyc04g0	0.45	-1.14
GO:00036	DNA bind	Molecular	0.00	0.02	Solyc04g0	Solyc04g0	2.55	1.35
GO:00036	DNA bind	Molecular	0.00	0.02	Solyc11g0	Solyc11g0	0.19	-2.40
GO:00036	DNA bind	Molecular	0.00	0.02	Solyc09g0	Solyc09g0	0.39	-1.37
GO:00036	DNA bind	Molecular	0.00	0.02	Solyc03g1	Solyc03g1	0.04	-4.59
GO:00036	DNA bind	Molecular	0.00	0.02	Solyc07g0	Solyc07g0	0.40	-1.31
GO:00036	DNA bind	Molecular	0.00	0.02	Solyc12g0	Solyc12g0	0.32	-1.65
GO:00036	DNA bind	Molecular	0.00	0.02	Solyc04g0	Solyc04g0	0.13	-2.91
GO:00036	DNA bind	Molecular	0.00	0.02	Solyc06g0	Solyc06g0	0.21	-2.28
GO:00036	DNA bind	Molecular	0.00	0.02	Solyc09g0	Solyc09g0	0.48	-1.06
GO:00036	DNA bind	Molecular	0.00	0.02	Solyc11g0	Solyc11g0	2.07	1.05
GO:00036	DNA bind	Molecular	0.00	0.02	Solyc09g0	Solyc09g0	2.92	1.55
GO:00036	DNA bind	Molecular	0.00	0.02	Solyc01g0	Solyc01g0	2.23	1.16
GO:00036	DNA bind	Molecular	0.00	0.02	Solyc02g0	Solyc02g0	3.87	1.95

GO:00036	DNA bind	Molecular	0.00	0.02	Solyc05g0	Solyc05g0	4.03	2.01
GO:00036	DNA bind	Molecular	0.00	0.02	Solyc01g0	Solyc01g0	0.35	-1.51
GO:00036	DNA bind	Molecular	0.00	0.02	Solyc04g0	Solyc04g0	0.47	-1.09
GO:00036	DNA bind	Molecular	0.00	0.02	Solyc05g0	Solyc05g0	0.40	-1.33
GO:00036	DNA bind	Molecular	0.00	0.02	Solyc03g1	Solyc03g1	2.29	1.19
GO:00036	DNA bind	Molecular	0.00	0.02	Solyc06g0	Solyc06g0	2.05	1.04
GO:00036	DNA bind	Molecular	0.00	0.02	Solyc02g0	Solyc02g0	0.33	-1.60
GO:00036	DNA bind	Molecular	0.00	0.02	Solyc01g0	Solyc01g0	2.64	1.40
GO:00036	DNA bind	Molecular	0.00	0.02	Solyc01g0	Solyc01g0	0.11	-3.13
GO:00036	DNA bind	Molecular	0.00	0.02	Solyc01g0	Solyc01g0	0.34	-1.55
GO:00036	DNA bind	Molecular	0.00	0.02	Solyc04g0	Solyc04g0	0.42	-1.26
GO:00036	DNA bind	Molecular	0.00	0.02	Solyc12g0	Solyc12g0	5.61	2.49
GO:00036	DNA bind	Molecular	0.00	0.02	Solyc12g0	Solyc12g0	0.13	-2.97
GO:00036	DNA bind	Molecular	0.00	0.02	Solyc01g0	Solyc01g0	2.38	1.25
GO:00036	DNA bind	Molecular	0.00	0.02	Solyc11g0	Solyc11g0	0.48	-1.05
GO:00036	DNA bind	Molecular	0.00	0.02	Solyc10g0	Solyc10g0	0.00	-13.06
GO:00036	DNA bind	Molecular	0.00	0.02	Solyc05g0	Solyc05g0	2.55	1.35
GO:00036	DNA bind	Molecular	0.00	0.02	Solyc03g0	Solyc03g0	0.40	-1.33
GO:00036	DNA bind	Molecular	0.00	0.02	Solyc11g0	Solyc11g0	2.46	1.30
GO:00036	DNA bind	Molecular	0.00	0.02	Solyc05g0	Solyc05g0	0.49	-1.03
GO:00036	DNA bind	Molecular	0.00	0.02	Solyc01g1	Solyc01g1	3.62	1.85
GO:00036	DNA bind	Molecular	0.00	0.02	Solyc02g0	Solyc02g0	0.19	-2.41
GO:00036	DNA bind	Molecular	0.00	0.02	Solyc10g0	Solyc10g0	0.23	-2.10
GO:00036	DNA bind	Molecular	0.00	0.02	Solyc08g0	Solyc08g0	0.38	-1.41
GO:00036	DNA bind	Molecular	0.00	0.02	Solyc05g0	Solyc05g0	0.48	-1.04
GO:00036	DNA bind	Molecular	0.00	0.02	Solyc03g1	Solyc03g1	2.13	1.09
GO:00036	DNA bind	Molecular	0.00	0.02	Solyc01g0	Solyc01g0	0.35	-1.51
GO:00036	DNA bind	Molecular	0.00	0.02	Solyc10g0	Solyc10g0	0.32	-1.63
GO:00036	DNA bind	Molecular	0.00	0.02	Solyc05g0	Solyc05g0	0.49	-1.03
GO:00036	DNA bind	Molecular	0.00	0.02	Solyc05g0	Solyc05g0	2.73	1.45
GO:00036	DNA bind	Molecular	0.00	0.02	Solyc08g0	Solyc08g0	2.07	1.05
GO:00036	DNA bind	Molecular	0.00	0.02	Solyc01g0	Solyc01g0	2.88	1.52
GO:00036	DNA bind	Molecular	0.00	0.02	Solyc01g0	Solyc01g0	208.08	7.70
GO:00036	DNA bind	Molecular	0.00	0.02	Solyc09g0	Solyc09g0	3.39	1.76
GO:00036	DNA bind	Molecular	0.00	0.02	Solyc03g0	Solyc03g0	0.06	-4.13
GO:00036	DNA bind	Molecular	0.00	0.02	Solyc12g0	Solyc12g0	2.57	1.36
GO:00036	DNA bind	Molecular	0.00	0.02	Solyc06g0	Solyc06g0	3.49	1.81
GO:00036	DNA bind	Molecular	0.00	0.02	Solyc10g0	Solyc10g0	0.35	-1.50
GO:00036	DNA bind	Molecular	0.00	0.02	Solyc08g0	Solyc08g0	0.35	-1.53
GO:00036	DNA bind	Molecular	0.00	0.02	Solyc05g0	Solyc05g0	2.08	1.06
GO:00036	DNA bind	Molecular	0.00	0.02	Solyc02g0	Solyc02g0	2.03	1.02
GO:00036	DNA bind	Molecular	0.00	0.02	Solyc03g1	Solyc03g1	0.44	-1.19
GO:00036	DNA bind	Molecular	0.00	0.02	Solyc04g0	Solyc04g0	2.19	1.13
GO:00036	DNA bind	Molecular	0.00	0.02	Solyc06g0	Solyc06g0	0.38	-1.41
GO:00036	DNA bind	Molecular	0.00	0.02	Solyc08g0	Solyc08g0	9.73	3.28
GO:00036	DNA bind	Molecular	0.00	0.02	Solyc02g0	Solyc02g0	0.45	-1.17
GO:00036	DNA bind	Molecular	0.00	0.02	Solyc02g0	Solyc02g0	0.42	-1.25
GO:00036	DNA bind	Molecular	0.00	0.02	Solyc08g0	Solyc08g0	2.25	1.17
GO:00036	DNA bind	Molecular	0.00	0.02	Solyc03g0	Solyc03g0	0.29	-1.76
GO:00036	DNA bind	Molecular	0.00	0.02	Solyc12g0	Solyc12g0	0.40	-1.34
GO:00036	DNA bind	Molecular	0.00	0.02	Solyc02g0	Solyc02g0	0.34	-1.57

GO:00036	DNA bind	Molecular	0.00	0.02	Solyc02g0	Solyc02g0	0.00	-11.41
GO:00036	DNA bind	Molecular	0.00	0.02	Solyc06g0	Solyc06g0	4.54	2.18
GO:00036	DNA bind	Molecular	0.00	0.02	Solyc10g0	Solyc10g0	12.09	3.60
GO:00036	DNA bind	Molecular	0.00	0.02	Solyc11g0	Solyc11g0	0.00	-9.30
GO:00036	DNA bind	Molecular	0.00	0.02	Solyc01g1	Solyc01g1	0.45	-1.15
GO:00036	DNA bind	Molecular	0.00	0.02	Solyc11g0	Solyc11g0	5.14	2.36
GO:00036	DNA bind	Molecular	0.00	0.02	Solyc02g0	Solyc02g0	2.15	1.10
GO:00036	DNA bind	Molecular	0.00	0.02	Solyc03g1	Solyc03g1	0.08	-3.61
GO:00036	DNA bind	Molecular	0.00	0.02	Solyc03g1	Solyc03g1	0.47	-1.08
GO:00036	DNA bind	Molecular	0.00	0.02	Solyc05g0	Solyc05g0	0.00	-8.91
GO:00036	DNA bind	Molecular	0.00	0.02	Solyc03g0	Solyc03g0	11.59	3.53
GO:00036	DNA bind	Molecular	0.00	0.02	Solyc10g0	Solyc10g0	0.33	-1.62
GO:00036	DNA bind	Molecular	0.00	0.02	Solyc01g1	Solyc01g1	0.15	-2.78
GO:00036	DNA bind	Molecular	0.00	0.02	Solyc02g0	Solyc02g0	2.19	1.13
GO:00036	DNA bind	Molecular	0.00	0.02	Solyc10g0	Solyc10g0	1745.96	10.77
GO:00036	DNA bind	Molecular	0.00	0.02	Solyc04g0	Solyc04g0	0.37	-1.43
GO:00036	DNA bind	Molecular	0.00	0.02	Solyc03g1	Solyc03g1	0.45	-1.14
GO:00036	DNA bind	Molecular	0.00	0.02	Solyc01g1	Solyc01g1	0.00	-9.51
GO:00036	DNA bind	Molecular	0.00	0.02	Solyc05g0	Solyc05g0	0.43	-1.23
GO:00036	DNA bind	Molecular	0.00	0.02	Solyc02g0	PTI5	0.07	-3.77
GO:00036	DNA bind	Molecular	0.00	0.02	Solyc03g0	Solyc03g0	0.39	-1.37
GO:00036	DNA bind	Molecular	0.00	0.02	Solyc01g0	Solyc01g0	6.71	2.75
GO:00036	DNA bind	Molecular	0.00	0.02	Solyc02g0	Solyc02g0	0.00	-8.58
GO:00036	DNA bind	Molecular	0.00	0.02	Solyc10g0	Solyc10g0	0.15	-2.72
GO:00036	DNA bind	Molecular	0.00	0.02	Solyc04g0	Solyc04g0	0.34	-1.54
GO:00036	DNA bind	Molecular	0.00	0.02	Solyc06g0	Solyc06g0	0.42	-1.25
GO:00036	DNA bind	Molecular	0.00	0.02	Solyc04g0	Solyc04g0	0.48	-1.05
GO:00036	DNA bind	Molecular	0.00	0.02	Solyc03g1	Solyc03g1	0.40	-1.33
GO:00036	DNA bind	Molecular	0.00	0.02	Solyc09g0	Solyc09g0	0.49	-1.04
GO:00036	DNA bind	Molecular	0.00	0.02	Solyc08g0	Solyc08g0	0.45	-1.17
GO:00036	DNA bind	Molecular	0.00	0.02	Solyc10g0	Solyc10g0	2.30	1.20
GO:00036	DNA bind	Molecular	0.00	0.02	Solyc04g0	Solyc04g0	0.20	-2.32
GO:00036	DNA bind	Molecular	0.00	0.02	Solyc02g0	Solyc02g0	0.23	-2.09
GO:00037	DNA-bind	Molecular	0.00	0.02	Solyc09g0	Solyc09g0	2.30	1.20
GO:00037	DNA-bind	Molecular	0.00	0.02	Solyc02g0	Solyc02g0	0.48	-1.05
GO:00037	DNA-bind	Molecular	0.00	0.02	Solyc11g0	Solyc11g0	0.44	-1.18
GO:00037	DNA-bind	Molecular	0.00	0.02	Solyc09g0	Solyc09g0	0.31	-1.67
GO:00037	DNA-bind	Molecular	0.00	0.02	Solyc03g0	Solyc03g0	2.41	1.27
GO:00037	DNA-bind	Molecular	0.00	0.02	Solyc04g0	Solyc04g0	2.92	1.55
GO:00037	DNA-bind	Molecular	0.00	0.02	Solyc05g0	Solyc05g0	0.08	-3.67
GO:00037	DNA-bind	Molecular	0.00	0.02	Solyc07g0	Solyc07g0	0.39	-1.37
GO:00037	DNA-bind	Molecular	0.00	0.02	Solyc01g0	Solyc01g0	0.25	-1.99
GO:00037	DNA-bind	Molecular	0.00	0.02	Solyc03g1	Solyc03g1	0.04	-4.59
GO:00037	DNA-bind	Molecular	0.00	0.02	Solyc07g0	Solyc07g0	0.40	-1.31
GO:00037	DNA-bind	Molecular	0.00	0.02	Solyc04g0	Solyc04g0	0.13	-2.91
GO:00037	DNA-bind	Molecular	0.00	0.02	Solyc06g0	Solyc06g0	0.21	-2.28
GO:00037	DNA-bind	Molecular	0.00	0.02	Solyc09g0	Solyc09g0	0.48	-1.06
GO:00037	DNA-bind	Molecular	0.00	0.02	Solyc02g0	Solyc02g0	3.87	1.95
GO:00037	DNA-bind	Molecular	0.00	0.02	Solyc05g0	Solyc05g0	3.62	1.86
GO:00037	DNA-bind	Molecular	0.00	0.02	Solyc01g0	Solyc01g0	0.35	-1.51
GO:00037	DNA-bind	Molecular	0.00	0.02	Solyc04g0	Solyc04g0	0.47	-1.09

GO:00037	DNA-bind	Molecular	0.00	0.02	Solyc05g0	Solyc05g0	0.40	-1.33
GO:00037	DNA-bind	Molecular	0.00	0.02	Solyc02g0	Solyc02g0	0.33	-1.60
GO:00037	DNA-bind	Molecular	0.00	0.02	Solyc06g0	Solyc06g0	2.04	1.03
GO:00037	DNA-bind	Molecular	0.00	0.02	Solyc01g0	Solyc01g0	0.11	-3.13
GO:00037	DNA-bind	Molecular	0.00	0.02	Solyc01g0	Solyc01g0	0.34	-1.55
GO:00037	DNA-bind	Molecular	0.00	0.02	Solyc05g0	Solyc05g0	0.49	-1.03
GO:00037	DNA-bind	Molecular	0.00	0.02	Solyc02g0	Solyc02g0	0.19	-2.41
GO:00037	DNA-bind	Molecular	0.00	0.02	Solyc05g0	Solyc05g0	0.48	-1.04
GO:00037	DNA-bind	Molecular	0.00	0.02	Solyc09g0	Solyc09g0	0.00	-11.89
GO:00037	DNA-bind	Molecular	0.00	0.02	Solyc08g0	Solyc08g0	0.28	-1.83
GO:00037	DNA-bind	Molecular	0.00	0.02	Solyc05g0	Solyc05g0	0.41	-1.29
GO:00037	DNA-bind	Molecular	0.00	0.02	Solyc07g0	Solyc07g0	0.07	-3.80
GO:00037	DNA-bind	Molecular	0.00	0.02	Solyc01g0	Solyc01g0	0.35	-1.51
GO:00037	DNA-bind	Molecular	0.00	0.02	Solyc10g0	Solyc10g0	0.32	-1.63
GO:00037	DNA-bind	Molecular	0.00	0.02	Solyc10g0	Solyc10g0	0.32	-1.64
GO:00037	DNA-bind	Molecular	0.00	0.02	Solyc03g0	Solyc03g0	0.06	-4.13
GO:00037	DNA-bind	Molecular	0.00	0.02	Solyc05g0	Solyc05g0	0.00	-11.04
GO:00037	DNA-bind	Molecular	0.00	0.02	Solyc02g0	Solyc02g0	0.15	-2.75
GO:00037	DNA-bind	Molecular	0.00	0.02	Solyc06g0	Solyc06g0	3.49	1.81
GO:00037	DNA-bind	Molecular	0.00	0.02	Solyc10g0	Solyc10g0	0.35	-1.50
GO:00037	DNA-bind	Molecular	0.00	0.02	Solyc03g1	Solyc03g1	0.37	-1.42
GO:00037	DNA-bind	Molecular	0.00	0.02	Solyc05g0	Solyc05g0	2.08	1.06
GO:00037	DNA-bind	Molecular	0.00	0.02	Solyc03g1	Solyc03g1	0.44	-1.19
GO:00037	DNA-bind	Molecular	0.00	0.02	Solyc06g0	Solyc06g0	0.38	-1.41
GO:00037	DNA-bind	Molecular	0.00	0.02	Solyc02g0	Solyc02g0	0.42	-1.25
GO:00037	DNA-bind	Molecular	0.00	0.02	Solyc09g0	Solyc09g0	0.37	-1.42
GO:00037	DNA-bind	Molecular	0.00	0.02	Solyc03g0	Solyc03g0	0.29	-1.76
GO:00037	DNA-bind	Molecular	0.00	0.02	Solyc02g0	Solyc02g0	0.34	-1.57
GO:00037	DNA-bind	Molecular	0.00	0.02	Solyc02g0	Solyc02g0	0.00	-11.41
GO:00037	DNA-bind	Molecular	0.00	0.02	Solyc11g0	Solyc11g0	5.14	2.36
GO:00037	DNA-bind	Molecular	0.00	0.02	Solyc02g0	Solyc02g0	0.34	-1.56
GO:00037	DNA-bind	Molecular	0.00	0.02	Solyc03g0	Solyc03g0	11.59	3.53
GO:00037	DNA-bind	Molecular	0.00	0.02	Solyc10g0	Solyc10g0	0.33	-1.62
GO:00037	DNA-bind	Molecular	0.00	0.02	Solyc10g0	Solyc10g0	0.16	-2.65
GO:00037	DNA-bind	Molecular	0.00	0.02	Solyc10g0	Solyc10g0	1745.96	10.77
GO:00037	DNA-bind	Molecular	0.00	0.02	Solyc04g0	Solyc04g0	0.37	-1.43
GO:00037	DNA-bind	Molecular	0.00	0.02	Solyc03g1	Solyc03g1	0.45	-1.14
GO:00037	DNA-bind	Molecular	0.00	0.02	Solyc10g0	Solyc10g0	2.87	1.52
GO:00037	DNA-bind	Molecular	0.00	0.02	Solyc06g0	Solyc06g0	0.22	-2.19
GO:00037	DNA-bind	Molecular	0.00	0.02	Solyc02g0	PTI5	0.07	-3.77
GO:00037	DNA-bind	Molecular	0.00	0.02	Solyc03g0	Solyc03g0	0.39	-1.37
GO:00037	DNA-bind	Molecular	0.00	0.02	Solyc03g0	Solyc03g0	0.21	-2.26
GO:00037	DNA-bind	Molecular	0.00	0.02	Solyc05g0	Solyc05g0	0.46	-1.13
GO:00037	DNA-bind	Molecular	0.00	0.02	Solyc10g0	Solyc10g0	0.15	-2.72
GO:00037	DNA-bind	Molecular	0.00	0.02	Solyc04g0	Solyc04g0	0.34	-1.54
GO:00037	DNA-bind	Molecular	0.00	0.02	Solyc06g0	Solyc06g0	0.42	-1.25
GO:00037	DNA-bind	Molecular	0.00	0.02	Solyc04g0	Solyc04g0	0.48	-1.05
GO:00037	DNA-bind	Molecular	0.00	0.02	Solyc03g1	Solyc03g1	0.40	-1.33
GO:00037	DNA-bind	Molecular	0.00	0.02	Solyc09g0	Solyc09g0	0.49	-1.04
GO:00037	DNA-bind	Molecular	0.00	0.02	Solyc08g0	Solyc08g0	0.45	-1.17
GO:00037	DNA-bind	Molecular	0.00	0.02	Solyc03g0	Solyc03g0	16.58	4.05

GO:00037	DNA-bind	Molecular	0.00	0.02	Solyc11g0	Solyc11g0	0.00	-9.45
GO:00037	DNA-bind	Molecular	0.00	0.02	Solyc08g0	Solyc08g0	0.00	-9.51
GO:00037	DNA-bind	Molecular	0.00	0.02	Solyc01g0	Solyc01g0	0.42	-1.24
GO:00508	defense res	Biological	0.00	0.02	Solyc08g0	Solyc08g0	0.27	-1.87
GO:00508	defense res	Biological	0.00	0.02	Solyc09g0	Solyc09g0	0.01	-7.14
GO:00508	defense res	Biological	0.00	0.02	Solyc06g0	Solyc06g0	0.08	-3.62
GO:00508	defense res	Biological	0.00	0.02	Solyc02g0	CHI3	0.24	-2.08
GO:00508	defense res	Biological	0.00	0.02	Solyc01g0	Solyc01g0	0.33	-1.59
GO:00508	defense res	Biological	0.00	0.02	Solyc07g0	EIX1	0.33	-1.59
GO:00508	defense res	Biological	0.00	0.02	Solyc08g0	Solyc08g0	0.39	-1.34
GO:00508	defense res	Biological	0.00	0.02	Solyc10g0	CHI9	0.27	-1.88
GO:00508	defense res	Biological	0.00	0.02	Solyc09g0	Solyc09g0	2.43	1.28
GO:00508	defense res	Biological	0.00	0.02	Solyc06g0	Solyc06g0	0.31	-1.70
GO:00508	defense res	Biological	0.00	0.02	Solyc06g0	Solyc06g0	0.48	-1.07
GO:00046	protein ser	Molecular	0.00	0.02	Solyc02g0	Solyc02g0	0.47	-1.10
GO:00046	protein ser	Molecular	0.00	0.02	Solyc08g0	Solyc08g0	0.31	-1.70
GO:00046	protein ser	Molecular	0.00	0.02	Solyc02g0	Solyc02g0	0.42	-1.24
GO:00046	protein ser	Molecular	0.00	0.02	Solyc03g1	Solyc03g1	0.39	-1.35
GO:00046	protein ser	Molecular	0.00	0.02	Solyc11g0	Solyc11g0	0.48	-1.06
GO:00046	protein ser	Molecular	0.00	0.02	Solyc03g0	Solyc03g0	2.33	1.22
GO:00046	protein ser	Molecular	0.00	0.02	Solyc08g0	Solyc08g0	0.39	-1.34
GO:00046	protein ser	Molecular	0.00	0.02	Solyc05g0	Solyc05g0	0.46	-1.12
GO:00046	protein ser	Molecular	0.00	0.02	Solyc06g0	Solyc06g0	0.40	-1.31
GO:00046	protein ser	Molecular	0.00	0.02	Solyc02g0	Solyc02g0	0.12	-3.00
GO:00046	protein ser	Molecular	0.00	0.02	Solyc07g0	Solyc07g0	0.25	-1.97
GO:00046	protein ser	Molecular	0.00	0.02	Solyc08g0	Solyc08g0	2.48	1.31
GO:00046	protein ser	Molecular	0.00	0.02	Solyc07g0	Solyc07g0	0.02	-5.66
GO:00046	protein ser	Molecular	0.00	0.02	Solyc11g0	Solyc11g0	2.74	1.45
GO:00046	protein ser	Molecular	0.00	0.02	Solyc10g0	Solyc10g0	0.06	-4.05
GO:00046	protein ser	Molecular	0.00	0.02	Solyc02g0	Solyc02g0	0.10	-3.33
GO:00046	protein ser	Molecular	0.00	0.02	Solyc02g0	Solyc02g0	0.02	-5.39
GO:00046	protein ser	Molecular	0.00	0.02	Solyc12g0	Solyc12g0	2.48	1.31
GO:00046	protein ser	Molecular	0.00	0.02	Solyc07g0	Solyc07g0	0.46	-1.11
GO:00046	protein ser	Molecular	0.00	0.02	Solyc02g0	Solyc02g0	0.36	-1.48
GO:00046	protein ser	Molecular	0.00	0.02	Solyc02g0	Solyc02g0	0.16	-2.63
GO:00046	protein ser	Molecular	0.00	0.02	Solyc02g0	Solyc02g0	0.06	-3.96
GO:00046	protein ser	Molecular	0.00	0.02	Solyc06g0	Solyc06g0	0.48	-1.07
GO:00046	protein ser	Molecular	0.00	0.02	Solyc02g0	Solyc02g0	0.45	-1.15
GO:00046	protein ser	Molecular	0.00	0.02	Solyc07g0	Solyc07g0	0.29	-1.79
GO:00046	protein ser	Molecular	0.00	0.02	Solyc09g0	Solyc09g0	0.38	-1.40
GO:00046	protein ser	Molecular	0.00	0.02	Solyc09g0	Solyc09g0	0.08	-3.73
GO:00046	protein ser	Molecular	0.00	0.02	Solyc06g0	Solyc06g0	0.26	-1.94
GO:00046	protein ser	Molecular	0.00	0.02	Solyc11g0	Solyc11g0	0.00	-9.30
GO:00046	protein ser	Molecular	0.00	0.02	Solyc02g0	Solyc02g0	0.44	-1.18
GO:00046	protein ser	Molecular	0.00	0.02	Solyc05g0	Solyc05g0	0.00	-8.91
GO:00046	protein ser	Molecular	0.00	0.02	Solyc04g0	Solyc04g0	0.00	-8.72
GO:00550	transmemb	Biological	0.00	0.02	Solyc03g0	Solyc03g0	0.35	-1.50
GO:00550	transmemb	Biological	0.00	0.02	Solyc09g0	Solyc09g0	0.14	-2.81
GO:00550	transmemb	Biological	0.00	0.02	Solyc05g0	Solyc05g0	0.18	-2.51
GO:00550	transmemb	Biological	0.00	0.02	Solyc06g0	Solyc06g0	0.37	-1.44
GO:00550	transmemb	Biological	0.00	0.02	Solyc08g0	Solyc08g0	2.26	1.18

GO:00550	transmemb	Biological	0.00	0.02	Solyc09g0	Solyc09g0	0.43	-1.22
GO:00550	transmemb	Biological	0.00	0.02	Solyc07g0	Solyc07g0	0.03	-5.23
GO:00550	transmemb	Biological	0.00	0.02	Solyc12g0	Solyc12g0	2.45	1.29
GO:00550	transmemb	Biological	0.00	0.02	Solyc03g0	Solyc03g0	0.33	-1.60
GO:00550	transmemb	Biological	0.00	0.02	Solyc01g1	Solyc01g1	0.19	-2.38
GO:00550	transmemb	Biological	0.00	0.02	Solyc08g0	Solyc08g0	0.26	-1.92
GO:00550	transmemb	Biological	0.00	0.02	Solyc01g1	Solyc01g1	0.43	-1.23
GO:00550	transmemb	Biological	0.00	0.02	Solyc09g0	Solyc09g0	0.18	-2.47
GO:00550	transmemb	Biological	0.00	0.02	Solyc01g0	Solyc01g0	0.50	-1.01
GO:00550	transmemb	Biological	0.00	0.02	Solyc10g0	Solyc10g0	2.72	1.45
GO:00550	transmemb	Biological	0.00	0.02	Solyc11g0	Solyc11g0	0.36	-1.49
GO:00550	transmemb	Biological	0.00	0.02	Solyc05g0	Solyc05g0	0.09	-3.40
GO:00550	transmemb	Biological	0.00	0.02	Solyc04g0	Solyc04g0	0.19	-2.40
GO:00550	transmemb	Biological	0.00	0.02	Solyc08g0	Solyc08g0	0.47	-1.09
GO:00550	transmemb	Biological	0.00	0.02	Solyc02g0	Solyc02g0	0.34	-1.57
GO:00550	transmemb	Biological	0.00	0.02	Solyc01g0	Solyc01g0	0.44	-1.18
GO:00550	transmemb	Biological	0.00	0.02	Solyc09g0	Solyc09g0	0.13	-2.95
GO:00550	transmemb	Biological	0.00	0.02	Solyc08g0	Solyc08g0	0.00	-11.90
GO:00550	transmemb	Biological	0.00	0.02	Solyc05g0	Solyc05g0	0.41	-1.29
GO:00550	transmemb	Biological	0.00	0.02	Solyc03g1	Solyc03g1	0.40	-1.31
GO:00550	transmemb	Biological	0.00	0.02	Solyc02g0	Solyc02g0	0.19	-2.42
GO:00550	transmemb	Biological	0.00	0.02	Solyc01g0	Solyc01g0	2099.54	11.04
GO:00550	transmemb	Biological	0.00	0.02	Solyc03g1	Solyc03g1	18.12	4.18
GO:00550	transmemb	Biological	0.00	0.02	Solyc05g0	Solyc05g0	0.32	-1.63
GO:00550	transmemb	Biological	0.00	0.02	Solyc08g0	Solyc08g0	0.06	-4.18
GO:00550	transmemb	Biological	0.00	0.02	Solyc05g0	Solyc05g0	0.33	-1.60
GO:00550	transmemb	Biological	0.00	0.02	Solyc03g1	Solyc03g1	0.47	-1.08
GO:00550	transmemb	Biological	0.00	0.02	Solyc06g0	Solyc06g0	0.26	-1.94
GO:00550	transmemb	Biological	0.00	0.02	Solyc11g0	Solyc11g0	2.48	1.31
GO:00550	transmemb	Biological	0.00	0.02	Solyc03g1	Solyc03g1	0.48	-1.05
GO:00550	transmemb	Biological	0.00	0.02	Solyc02g0	Solyc02g0	0.31	-1.67
GO:00550	transmemb	Biological	0.00	0.02	Solyc02g0	Solyc02g0	0.33	-1.61
GO:00550	transmemb	Biological	0.00	0.02	Solyc08g0	Solyc08g0	0.40	-1.32
GO:00550	transmemb	Biological	0.00	0.02	Solyc04g0	Solyc04g0	2.01	1.01
GO:00550	transmemb	Biological	0.00	0.02	Solyc02g0	Solyc02g0	0.45	-1.16
GO:00550	transmemb	Biological	0.00	0.02	Solyc05g0	Solyc05g0	0.40	-1.34
GO:00550	transmemb	Biological	0.00	0.02	Solyc03g0	Solyc03g0	0.00	-10.59
GO:00550	transmemb	Biological	0.00	0.02	Solyc05g0	Solyc05g0	0.06	-4.14
GO:00550	transmemb	Biological	0.00	0.02	Solyc12g0	Solyc12g0	0.48	-1.07
GO:00550	transmemb	Biological	0.00	0.02	Solyc06g0	Solyc06g0	0.37	-1.45
GO:00550	transmemb	Biological	0.00	0.02	Solyc12g0	Solyc12g0	1405.23	10.46
GO:00550	transmemb	Biological	0.00	0.02	Solyc12g0	Solyc12g0	0.46	-1.12
GO:00550	transmemb	Biological	0.00	0.02	Solyc06g0	Solyc06g0	0.04	-4.72
GO:00550	transmemb	Biological	0.00	0.02	Solyc10g0	Solyc10g0	0.38	-1.38
GO:00550	transmemb	Biological	0.00	0.02	Solyc06g0	Solyc06g0	2.48	1.31
GO:00550	transmemb	Biological	0.00	0.02	Solyc03g0	Solyc03g0	27.60	4.79
GO:00550	transmemb	Biological	0.00	0.02	Solyc09g0	Solyc09g0	460.47	8.85
GO:00550	transmemb	Biological	0.00	0.02	Solyc12g0	Solyc12g0	0.12	-3.02
GO:00550	transmemb	Biological	0.00	0.02	Solyc09g0	Solyc09g0	0.42	-1.26
GO:00550	transmemb	Biological	0.00	0.02	Solyc07g0	Solyc07g0	0.00	-9.97
GO:00550	transmemb	Biological	0.00	0.02	Solyc04g0	Solyc04g0	0.49	-1.04

GO:00550	transmemb	Biological	0.00	0.02	Solyc08g0	Solyc08g0	3.30	1.72
GO:00550	transmemb	Biological	0.00	0.02	Solyc05g0	Solyc05g0	0.46	-1.13
GO:00066	lipid metal	Biological	0.00	0.02	Solyc11g0	AOS2	0.12	-3.01
GO:00066	lipid metal	Biological	0.00	0.02	Solyc03g0	Solyc03g0	2.46	1.30
GO:00066	lipid metal	Biological	0.00	0.02	Solyc01g0	Solyc01g0	0.27	-1.87
GO:00066	lipid metal	Biological	0.00	0.02	Solyc01g0	Solyc01g0	4.36	2.12
GO:00066	lipid metal	Biological	0.00	0.02	Solyc01g1	Solyc01g1	0.45	-1.16
GO:00066	lipid metal	Biological	0.00	0.02	Solyc07g0	Solyc07g0	0.46	-1.12
GO:00066	lipid metal	Biological	0.00	0.02	Solyc05g0	Solyc05g0	0.45	-1.15
GO:00066	lipid metal	Biological	0.00	0.02	Solyc03g0	Solyc03g0	2.03	1.02
GO:00066	lipid metal	Biological	0.00	0.02	Solyc01g0	Solyc01g0	2.60	1.38
GO:00066	lipid metal	Biological	0.00	0.02	Solyc12g0	Solyc12g0	0.31	-1.70
GO:00066	lipid metal	Biological	0.00	0.02	Solyc11g0	Solyc11g0	3.98	1.99
GO:00066	lipid metal	Biological	0.00	0.02	Solyc07g0	OPR3	0.47	-1.10
GO:00066	lipid metal	Biological	0.00	0.02	Solyc12g0	Solyc12g0	10.12	3.34
GO:00066	lipid metal	Biological	0.00	0.02	Solyc06g0	Solyc06g0	2.69	1.43
GO:00066	lipid metal	Biological	0.00	0.02	Solyc09g0	Solyc09g0	4.04	2.01
GO:00066	lipid metal	Biological	0.00	0.02	Solyc02g0	Solyc02g0	0.46	-1.13
GO:00066	lipid metal	Biological	0.00	0.02	Solyc04g0	Solyc04g0	0.00	-10.62
GO:00066	lipid metal	Biological	0.00	0.02	Solyc12g1	Solyc12g1	0.00	-10.37
GO:00066	lipid metal	Biological	0.00	0.02	Solyc09g0	Solyc09g0	0.47	-1.09
GO:00066	lipid metal	Biological	0.00	0.02	Solyc12g0	Solyc12g0	0.00	-9.93
GO:00066	lipid metal	Biological	0.00	0.02	Solyc06g0	Solyc06g0	0.19	-2.40
GO:00046	protein kin	Molecular	0.00	0.03	Solyc02g0	Solyc02g0	0.14	-2.87
GO:00046	protein kin	Molecular	0.00	0.03	Solyc06g0	Solyc06g0	2.42	1.28
GO:00046	protein kin	Molecular	0.00	0.03	Solyc01g1	Solyc01g1	0.31	-1.68
GO:00046	protein kin	Molecular	0.00	0.03	Solyc11g0	Solyc11g0	0.13	-3.00
GO:00046	protein kin	Molecular	0.00	0.03	Solyc02g0	Solyc02g0	0.47	-1.10
GO:00046	protein kin	Molecular	0.00	0.03	Solyc08g0	Solyc08g0	0.31	-1.70
GO:00046	protein kin	Molecular	0.00	0.03	Solyc03g0	Solyc03g0	0.46	-1.13
GO:00046	protein kin	Molecular	0.00	0.03	Solyc02g0	Solyc02g0	0.42	-1.24
GO:00046	protein kin	Molecular	0.00	0.03	Solyc02g0	Solyc02g0	0.41	-1.29
GO:00046	protein kin	Molecular	0.00	0.03	Solyc02g0	Solyc02g0	0.15	-2.72
GO:00046	protein kin	Molecular	0.00	0.03	Solyc01g1	Solyc01g1	3.45	1.79
GO:00046	protein kin	Molecular	0.00	0.03	Solyc03g1	Solyc03g1	0.39	-1.35
GO:00046	protein kin	Molecular	0.00	0.03	Solyc09g0	Solyc09g0	2.10	1.07
GO:00046	protein kin	Molecular	0.00	0.03	Solyc04g0	Solyc04g0	0.39	-1.35
GO:00046	protein kin	Molecular	0.00	0.03	Solyc12g0	Solyc12g0	0.33	-1.60
GO:00046	protein kin	Molecular	0.00	0.03	Solyc05g0	Solyc05g0	0.46	-1.11
GO:00046	protein kin	Molecular	0.00	0.03	Solyc11g0	Solyc11g0	0.48	-1.06
GO:00046	protein kin	Molecular	0.00	0.03	Solyc11g0	Solyc11g0	0.49	-1.03
GO:00046	protein kin	Molecular	0.00	0.03	Solyc03g0	Solyc03g0	3.70	1.89
GO:00046	protein kin	Molecular	0.00	0.03	Solyc04g0	Solyc04g0	0.48	-1.06
GO:00046	protein kin	Molecular	0.00	0.03	Solyc03g0	Solyc03g0	2.33	1.22
GO:00046	protein kin	Molecular	0.00	0.03	Solyc08g0	Solyc08g0	0.39	-1.34
GO:00046	protein kin	Molecular	0.00	0.03	Solyc05g0	Solyc05g0	0.46	-1.12
GO:00046	protein kin	Molecular	0.00	0.03	Solyc06g0	Solyc06g0	0.40	-1.31
GO:00046	protein kin	Molecular	0.00	0.03	Solyc09g0	Solyc09g0	0.39	-1.35
GO:00046	protein kin	Molecular	0.00	0.03	Solyc11g0	Solyc11g0	0.46	-1.11
GO:00046	protein kin	Molecular	0.00	0.03	Solyc02g0	Solyc02g0	0.12	-3.00
GO:00046	protein kin	Molecular	0.00	0.03	Solyc07g0	Solyc07g0	0.25	-1.97

GO:00046	protein kin	Molecular	0.00	0.03	Solyc08g0	Solyc08g0	2.48	1.31
GO:00046	protein kin	Molecular	0.00	0.03	Solyc07g0	Solyc07g0	0.02	-5.66
GO:00046	protein kin	Molecular	0.00	0.03	Solyc11g0	Solyc11g0	5992.64	12.55
GO:00046	protein kin	Molecular	0.00	0.03	Solyc06g0	Solyc06g0	3.73	1.90
GO:00046	protein kin	Molecular	0.00	0.03	Solyc11g0	Solyc11g0	2.74	1.45
GO:00046	protein kin	Molecular	0.00	0.03	Solyc10g0	Solyc10g0	0.06	-4.05
GO:00046	protein kin	Molecular	0.00	0.03	Solyc02g0	Solyc02g0	0.10	-3.33
GO:00046	protein kin	Molecular	0.00	0.03	Solyc01g0	Solyc01g0	0.00	-12.11
GO:00046	protein kin	Molecular	0.00	0.03	Solyc01g1	Solyc01g1	0.20	-2.34
GO:00046	protein kin	Molecular	0.00	0.03	Solyc12g0	Solyc12g0	0.33	-1.58
GO:00046	protein kin	Molecular	0.00	0.03	Solyc02g0	Solyc02g0	0.02	-5.39
GO:00046	protein kin	Molecular	0.00	0.03	Solyc04g0	Solyc04g0	0.21	-2.26
GO:00046	protein kin	Molecular	0.00	0.03	Solyc12g0	Solyc12g0	2.48	1.31
GO:00046	protein kin	Molecular	0.00	0.03	Solyc01g0	Solyc01g0	1525.65	10.58
GO:00046	protein kin	Molecular	0.00	0.03	Solyc05g0	Solyc05g0	3.41	1.77
GO:00046	protein kin	Molecular	0.00	0.03	Solyc07g0	Solyc07g0	0.46	-1.11
GO:00046	protein kin	Molecular	0.00	0.03	Solyc02g0	Solyc02g0	0.36	-1.48
GO:00046	protein kin	Molecular	0.00	0.03	Solyc04g0	Solyc04g0	0.35	-1.51
GO:00046	protein kin	Molecular	0.00	0.03	Solyc04g0	Solyc04g0	0.48	-1.06
GO:00046	protein kin	Molecular	0.00	0.03	Solyc09g0	Solyc09g0	0.33	-1.59
GO:00046	protein kin	Molecular	0.00	0.03	Solyc06g0	Solyc06g0	2.93	1.55
GO:00046	protein kin	Molecular	0.00	0.03	Solyc07g0	Solyc07g0	2.61	1.38
GO:00046	protein kin	Molecular	0.00	0.03	Solyc12g0	Solyc12g0	0.09	-3.42
GO:00046	protein kin	Molecular	0.00	0.03	Solyc03g0	Solyc03g0	0.48	-1.05
GO:00046	protein kin	Molecular	0.00	0.03	Solyc02g0	Solyc02g0	0.16	-2.63
GO:00046	protein kin	Molecular	0.00	0.03	Solyc11g0	Solyc11g0	0.12	-3.04
GO:00046	protein kin	Molecular	0.00	0.03	Solyc05g0	Solyc05g0	0.47	-1.10
GO:00046	protein kin	Molecular	0.00	0.03	Solyc02g0	Solyc02g0	0.06	-3.96
GO:00046	protein kin	Molecular	0.00	0.03	Solyc06g0	Solyc06g0	0.48	-1.07
GO:00046	protein kin	Molecular	0.00	0.03	Solyc02g0	Solyc02g0	0.45	-1.15
GO:00046	protein kin	Molecular	0.00	0.03	Solyc07g0	Solyc07g0	0.29	-1.79
GO:00046	protein kin	Molecular	0.00	0.03	Solyc08g0	Solyc08g0	0.18	-2.44
GO:00046	protein kin	Molecular	0.00	0.03	Solyc02g0	Solyc02g0	0.50	-1.01
GO:00046	protein kin	Molecular	0.00	0.03	Solyc05g0	Solyc05g0	2.16	1.11
GO:00046	protein kin	Molecular	0.00	0.03	Solyc08g0	Solyc08g0	0.19	-2.38
GO:00046	protein kin	Molecular	0.00	0.03	Solyc03g0	Solyc03g0	0.00	-8.79
GO:00046	protein kin	Molecular	0.00	0.03	Solyc03g0	Solyc03g0	0.01	-7.23
GO:00046	protein kin	Molecular	0.00	0.03	Solyc01g0	Solyc01g0	2.66	1.41
GO:00046	protein kin	Molecular	0.00	0.03	Solyc10g0	Solyc10g0	0.50	-1.01
GO:00046	protein kin	Molecular	0.00	0.03	Solyc02g0	Solyc02g0	0.49	-1.03
GO:00046	protein kin	Molecular	0.00	0.03	Solyc12g0	Solyc12g0	0.00	-11.47
GO:00046	protein kin	Molecular	0.00	0.03	Solyc09g0	Solyc09g0	0.38	-1.40
GO:00046	protein kin	Molecular	0.00	0.03	Solyc08g0	Solyc08g0	0.07	-3.76
GO:00046	protein kin	Molecular	0.00	0.03	Solyc09g0	Solyc09g0	0.08	-3.73
GO:00046	protein kin	Molecular	0.00	0.03	Solyc06g0	Solyc06g0	0.26	-1.94
GO:00046	protein kin	Molecular	0.00	0.03	Solyc01g0	Solyc01g0	2.61	1.39
GO:00046	protein kin	Molecular	0.00	0.03	Solyc09g0	Solyc09g0	2.30	1.20
GO:00046	protein kin	Molecular	0.00	0.03	Solyc10g0	Solyc10g0	0.42	-1.27
GO:00046	protein kin	Molecular	0.00	0.03	Solyc11g0	Solyc11g0	0.00	-9.30
GO:00046	protein kin	Molecular	0.00	0.03	Solyc12g0	Solyc12g0	0.21	-2.24
GO:00046	protein kin	Molecular	0.00	0.03	Solyc02g0	Solyc02g0	0.44	-1.18

GO:00046	protein kin	Molecular	0.00	0.03	Solyc05g0	Solyc05g0	0.00	-8.91
GO:00046	protein kin	Molecular	0.00	0.03	Solyc04g0	Solyc04g0	0.00	-8.72
GO:00046	protein kin	Molecular	0.00	0.03	Solyc06g0	Solyc06g0	0.41	-1.27
GO:00046	protein kin	Molecular	0.00	0.03	Solyc01g0	Solyc01g0	0.33	-1.61
GO:00046	protein kin	Molecular	0.00	0.03	Solyc09g0	Solyc09g0	0.22	-2.21
GO:00104	negative re	Biological	0.00	0.03	Solyc08g0	Solyc08g0	0.19	-2.36
GO:00104	negative re	Biological	0.00	0.03	Solyc03g0	Solyc03g0	2.00	1.00
GO:00104	negative re	Biological	0.00	0.03	Solyc11g0	Solyc11g0	4.18	2.06
GO:00104	negative re	Biological	0.00	0.03	Solyc09g0	Solyc09g0	33.35	5.06
GO:00304	peptidase i	Molecular	0.00	0.03	Solyc08g0	Solyc08g0	0.19	-2.36
GO:00304	peptidase i	Molecular	0.00	0.03	Solyc03g0	Solyc03g0	2.00	1.00
GO:00304	peptidase i	Molecular	0.00	0.03	Solyc11g0	Solyc11g0	4.18	2.06
GO:00304	peptidase i	Molecular	0.00	0.03	Solyc09g0	Solyc09g0	33.35	5.06
GO:00096	response tc	Biological	0.00	0.03	Solyc08g0	Solyc08g0	0.27	-1.87
GO:00096	response tc	Biological	0.00	0.03	Solyc09g0	Solyc09g0	0.01	-7.14
GO:00096	response tc	Biological	0.00	0.03	Solyc09g0	Solyc09g0	0.37	-1.43
GO:00096	response tc	Biological	0.00	0.03	Solyc02g0	CHI3	0.24	-2.08
GO:00096	response tc	Biological	0.00	0.03	Solyc01g0	Solyc01g0	0.33	-1.59
GO:00096	response tc	Biological	0.00	0.03	Solyc06g0	Solyc06g0	0.07	-3.75
GO:00160	membrane	Cellular Co	0.00	0.03	Solyc09g0	Solyc09g0	2.11	1.07
GO:00160	membrane	Cellular Co	0.00	0.03	Solyc11g0	AOS2	0.12	-3.01
GO:00160	membrane	Cellular Co	0.00	0.03	Solyc09g0	Solyc09g0	0.14	-2.81
GO:00160	membrane	Cellular Co	0.00	0.03	Solyc06g0	Solyc06g0	3.24	1.69
GO:00160	membrane	Cellular Co	0.00	0.03	Solyc01g1	Solyc01g1	3.04	1.60
GO:00160	membrane	Cellular Co	0.00	0.03	Solyc02g0	Solyc02g0	2.02	1.01
GO:00160	membrane	Cellular Co	0.00	0.03	Solyc03g1	Solyc03g1	0.11	-3.23
GO:00160	membrane	Cellular Co	0.00	0.03	Solyc05g0	Solyc05g0	0.18	-2.51
GO:00160	membrane	Cellular Co	0.00	0.03	Solyc05g0	Solyc05g0	0.22	-2.15
GO:00160	membrane	Cellular Co	0.00	0.03	Solyc06g0	Solyc06g0	0.08	-3.62
GO:00160	membrane	Cellular Co	0.00	0.03	Solyc02g0	PSBO	0.48	-1.07
GO:00160	membrane	Cellular Co	0.00	0.03	Solyc06g0	Solyc06g0	0.37	-1.44
GO:00160	membrane	Cellular Co	0.00	0.03	Solyc05g0	Solyc05g0	2.02	1.01
GO:00160	membrane	Cellular Co	0.00	0.03	Solyc09g0	Solyc09g0	0.15	-2.74
GO:00160	membrane	Cellular Co	0.00	0.03	Solyc03g0	Solyc03g0	0.18	-2.46
GO:00160	membrane	Cellular Co	0.00	0.03	Solyc08g0	Solyc08g0	0.19	-2.37
GO:00160	membrane	Cellular Co	0.00	0.03	Solyc10g0	Solyc10g0	0.43	-1.23
GO:00160	membrane	Cellular Co	0.00	0.03	Solyc09g0	Solyc09g0	0.43	-1.22
GO:00160	membrane	Cellular Co	0.00	0.03	Solyc02g0	Solyc02g0	0.47	-1.10
GO:00160	membrane	Cellular Co	0.00	0.03	Solyc04g0	Solyc04g0	0.17	-2.54
GO:00160	membrane	Cellular Co	0.00	0.03	Solyc07g0	Solyc07g0	0.03	-5.23
GO:00160	membrane	Cellular Co	0.00	0.03	Solyc08g0	CYP707A	0.26	-1.93
GO:00160	membrane	Cellular Co	0.00	0.03	Solyc01g0	Solyc01g0	0.29	-1.78
GO:00160	membrane	Cellular Co	0.00	0.03	Solyc12g0	Solyc12g0	2.45	1.29
GO:00160	membrane	Cellular Co	0.00	0.03	Solyc01g1	Solyc01g1	0.19	-2.38
GO:00160	membrane	Cellular Co	0.00	0.03	Solyc07g0	EIX1	0.33	-1.59
GO:00160	membrane	Cellular Co	0.00	0.03	Solyc03g1	Solyc03g1	0.41	-1.27
GO:00160	membrane	Cellular Co	0.00	0.03	Solyc03g1	Solyc03g1	0.37	-1.42
GO:00160	membrane	Cellular Co	0.00	0.03	Solyc01g1	Solyc01g1	0.43	-1.23
GO:00160	membrane	Cellular Co	0.00	0.03	Solyc07g0	Solyc07g0	0.30	-1.76
GO:00160	membrane	Cellular Co	0.00	0.03	Solyc12g0	Solyc12g0	0.31	-1.70
GO:00160	membrane	Cellular Co	0.00	0.03	Solyc09g0	Solyc09g0	0.18	-2.47

GO:00160	membrane	Cellular C	0.00	0.03	Solyc06g0	psaD	0.43	-1.22
GO:00160	membrane	Cellular C	0.00	0.03	Solyc10g0	Solyc10g0	2.72	1.45
GO:00160	membrane	Cellular C	0.00	0.03	Solyc02g0	Solyc02g0	2.19	1.13
GO:00160	membrane	Cellular C	0.00	0.03	Solyc08g0	Solyc08g0	0.37	-1.43
GO:00160	membrane	Cellular C	0.00	0.03	Solyc11g0	Solyc11g0	0.36	-1.49
GO:00160	membrane	Cellular C	0.00	0.03	Solyc09g0	Solyc09g0	2.86	1.51
GO:00160	membrane	Cellular C	0.00	0.03	Solyc08g0	Solyc08g0	0.47	-1.09
GO:00160	membrane	Cellular C	0.00	0.03	Solyc05g0	Solyc05g0	0.15	-2.75
GO:00160	membrane	Cellular C	0.00	0.03	Solyc10g0	Solyc10g0	0.49	-1.02
GO:00160	membrane	Cellular C	0.00	0.03	Solyc04g0	Solyc04g0	0.19	-2.40
GO:00160	membrane	Cellular C	0.00	0.03	Solyc08g0	Solyc08g0	0.47	-1.09
GO:00160	membrane	Cellular C	0.00	0.03	Solyc11g0	Solyc11g0	0.47	-1.08
GO:00160	membrane	Cellular C	0.00	0.03	Solyc06g0	Solyc06g0	0.41	-1.28
GO:00160	membrane	Cellular C	0.00	0.03	Solyc08g0	Solyc08g0	0.24	-2.04
GO:00160	membrane	Cellular C	0.00	0.03	Solyc02g0	Solyc02g0	0.34	-1.57
GO:00160	membrane	Cellular C	0.00	0.03	Solyc01g0	Solyc01g0	0.44	-1.18
GO:00160	membrane	Cellular C	0.00	0.03	Solyc08g0	Solyc08g0	0.36	-1.48
GO:00160	membrane	Cellular C	0.00	0.03	Solyc03g0	Solyc03g0	2.00	1.00
GO:00160	membrane	Cellular C	0.00	0.03	Solyc06g0	Solyc06g0	2.11	1.07
GO:00160	membrane	Cellular C	0.00	0.03	Solyc09g0	Solyc09g0	0.13	-2.95
GO:00160	membrane	Cellular C	0.00	0.03	Solyc08g0	Solyc08g0	0.47	-1.07
GO:00160	membrane	Cellular C	0.00	0.03	Solyc03g1	Solyc03g1	0.47	-1.08
GO:00160	membrane	Cellular C	0.00	0.03	Solyc06g0	Solyc06g0	2.69	1.43
GO:00160	membrane	Cellular C	0.00	0.03	Solyc12g0	Solyc12g0	2112.44	11.04
GO:00160	membrane	Cellular C	0.00	0.03	Solyc02g0	Solyc02g0	0.23	-2.14
GO:00160	membrane	Cellular C	0.00	0.03	Solyc06g0	Solyc06g0	0.07	-3.75
GO:00160	membrane	Cellular C	0.00	0.03	Solyc01g0	Solyc01g0	0.32	-1.64
GO:00160	membrane	Cellular C	0.00	0.03	Solyc10g0	Solyc10g0	0.41	-1.27
GO:00160	membrane	Cellular C	0.00	0.03	Solyc04g0	Solyc04g0	0.30	-1.74
GO:00160	membrane	Cellular C	0.00	0.03	Solyc03g1	Solyc03g1	0.40	-1.31
GO:00160	membrane	Cellular C	0.00	0.03	Solyc02g0	Solyc02g0	0.19	-2.42
GO:00160	membrane	Cellular C	0.00	0.03	Solyc09g0	Solyc09g0	0.34	-1.55
GO:00160	membrane	Cellular C	0.00	0.03	Solyc04g0	Solyc04g0	0.16	-2.68
GO:00160	membrane	Cellular C	0.00	0.03	Solyc06g0	Solyc06g0	0.08	-3.58
GO:00160	membrane	Cellular C	0.00	0.03	Solyc08g0	Solyc08g0	0.20	-2.32
GO:00160	membrane	Cellular C	0.00	0.03	Solyc05g0	Solyc05g0	2.03	1.02
GO:00160	membrane	Cellular C	0.00	0.03	Solyc09g0	Solyc09g0	8.52	3.09
GO:00160	membrane	Cellular C	0.00	0.03	Solyc08g0	Solyc08g0	0.48	-1.05
GO:00160	membrane	Cellular C	0.00	0.03	Solyc08g0	Solyc08g0	0.22	-2.16
GO:00160	membrane	Cellular C	0.00	0.03	Solyc05g0	Solyc05g0	0.32	-1.63
GO:00160	membrane	Cellular C	0.00	0.03	Solyc03g1	Solyc03g1	0.47	-1.08
GO:00160	membrane	Cellular C	0.00	0.03	Solyc03g0	Solyc03g0	0.45	-1.15
GO:00160	membrane	Cellular C	0.00	0.03	Solyc07g0	Solyc07g0	0.32	-1.65
GO:00160	membrane	Cellular C	0.00	0.03	Solyc07g0	Solyc07g0	0.50	-1.01
GO:00160	membrane	Cellular C	0.00	0.03	Solyc05g0	Solyc05g0	0.00	-11.15
GO:00160	membrane	Cellular C	0.00	0.03	Solyc04g0	Solyc04g0	0.25	-2.03
GO:00160	membrane	Cellular C	0.00	0.03	Solyc02g0	Solyc02g0	0.16	-2.63
GO:00160	membrane	Cellular C	0.00	0.03	Solyc06g0	Solyc06g0	0.31	-1.70
GO:00160	membrane	Cellular C	0.00	0.03	Solyc01g0	Solyc01g0	0.00	-10.07
GO:00160	membrane	Cellular C	0.00	0.03	Solyc11g0	Solyc11g0	2.48	1.31
GO:00160	membrane	Cellular C	0.00	0.03	Solyc11g0	Solyc11g0	0.33	-1.60

GO:00160	membrane	Cellular C	0.00	0.03	Solyc03g1	Solyc03g1	0.48	-1.05
GO:00160	membrane	Cellular C	0.00	0.03	Solyc02g0	Solyc02g0	0.31	-1.67
GO:00160	membrane	Cellular C	0.00	0.03	Solyc01g1	Solyc01g1	2.95	1.56
GO:00160	membrane	Cellular C	0.00	0.03	Solyc02g0	Solyc02g0	0.33	-1.61
GO:00160	membrane	Cellular C	0.00	0.03	Solyc08g0	Solyc08g0	0.40	-1.32
GO:00160	membrane	Cellular C	0.00	0.03	Solyc04g0	Solyc04g0	2.01	1.01
GO:00160	membrane	Cellular C	0.00	0.03	Solyc05g0	Solyc05g0	0.40	-1.34
GO:00160	membrane	Cellular C	0.00	0.03	Solyc03g0	Solyc03g0	0.00	-10.59
GO:00160	membrane	Cellular C	0.00	0.03	Solyc05g0	Solyc05g0	0.06	-4.14
GO:00160	membrane	Cellular C	0.00	0.03	Solyc12g0	Solyc12g0	0.48	-1.07
GO:00160	membrane	Cellular C	0.00	0.03	Solyc07g0	Solyc07g0	0.44	-1.18
GO:00160	membrane	Cellular C	0.00	0.03	Solyc11g0	Solyc11g0	0.16	-2.61
GO:00160	membrane	Cellular C	0.00	0.03	Solyc06g0	Solyc06g0	0.37	-1.45
GO:00160	membrane	Cellular C	0.00	0.03	Solyc03g1	Solyc03g1	0.10	-3.33
GO:00160	membrane	Cellular C	0.00	0.03	Solyc12g0	Solyc12g0	1405.23	10.46
GO:00160	membrane	Cellular C	0.00	0.03	Solyc10g0	Solyc10g0	2.57	1.36
GO:00160	membrane	Cellular C	0.00	0.03	Solyc06g0	Solyc06g0	366.52	8.52
GO:00160	membrane	Cellular C	0.00	0.03	Solyc01g1	CAP10A	0.35	-1.51
GO:00160	membrane	Cellular C	0.00	0.03	Solyc02g0	Solyc02g0	0.43	-1.23
GO:00160	membrane	Cellular C	0.00	0.03	Solyc10g0	Solyc10g0	0.00	-11.12
GO:00160	membrane	Cellular C	0.00	0.03	Solyc02g0	Solyc02g0	7.41	2.89
GO:00160	membrane	Cellular C	0.00	0.03	Solyc10g0	Solyc10g0	0.48	-1.05
GO:00160	membrane	Cellular C	0.00	0.03	Solyc01g0	Solyc01g0	2.28	1.19
GO:00160	membrane	Cellular C	0.00	0.03	Solyc06g0	Solyc06g0	0.26	-1.94
GO:00160	membrane	Cellular C	0.00	0.03	Solyc03g1	Solyc03g1	668.93	9.39
GO:00160	membrane	Cellular C	0.00	0.03	Solyc11g0	Solyc11g0	0.00	-8.36
GO:00160	membrane	Cellular C	0.00	0.03	Solyc09g0	Solyc09g0	0.13	-2.94
GO:00160	membrane	Cellular C	0.00	0.03	Solyc07g0	Solyc07g0	638.37	9.32
GO:00160	membrane	Cellular C	0.00	0.03	Solyc06g0	Solyc06g0	0.04	-4.72
GO:00160	membrane	Cellular C	0.00	0.03	Solyc04g0	Solyc04g0	0.43	-1.23
GO:00160	membrane	Cellular C	0.00	0.03	Solyc10g0	Solyc10g0	0.38	-1.38
GO:00160	membrane	Cellular C	0.00	0.03	Solyc11g0	Solyc11g0	0.24	-2.07
GO:00160	membrane	Cellular C	0.00	0.03	Solyc08g0	Solyc08g0	0.00	-9.52
GO:00160	membrane	Cellular C	0.00	0.03	Solyc03g0	Solyc03g0	0.34	-1.57
GO:00160	membrane	Cellular C	0.00	0.03	Solyc09g0	Solyc09g0	0.42	-1.26
GO:00160	membrane	Cellular C	0.00	0.03	Solyc02g0	Solyc02g0	0.43	-1.20
GO:00160	membrane	Cellular C	0.00	0.03	Solyc07g0	Solyc07g0	0.00	-9.97
GO:00160	membrane	Cellular C	0.00	0.03	Solyc08g0	Solyc08g0	0.00	-9.01
GO:00160	membrane	Cellular C	0.00	0.03	Solyc01g0	Solyc01g0	0.19	-2.40
GO:00160	membrane	Cellular C	0.00	0.03	Solyc06g0	Solyc06g0	0.00	-8.61
GO:00160	membrane	Cellular C	0.00	0.03	Solyc08g0	Solyc08g0	3.30	1.72
GO:00160	membrane	Cellular C	0.00	0.03	Solyc11g0	Solyc11g0	4.59	2.20
GO:00160	membrane	Cellular C	0.00	0.03	Solyc05g0	Solyc05g0	0.46	-1.13
GO:00080	chitin bind	Molecular	0.00	0.03	Solyc10g0	Solyc10g0	0.05	-4.23
GO:00080	chitin bind	Molecular	0.00	0.03	Solyc10g0	Solyc10g0	0.02	-5.52
GO:00080	chitin bind	Molecular	0.00	0.03	Solyc10g0	Solyc10g0	0.06	-4.12
GO:00080	chitin bind	Molecular	0.00	0.03	Solyc07g0	Solyc07g0	0.01	-6.20
GO:00080	chitin bind	Molecular	0.00	0.03	Solyc10g0	CHI9	0.27	-1.88
GO:00080	chitin bind	Molecular	0.00	0.03	Solyc07g0	Solyc07g0	0.45	-1.16
GO:00314	oxylipin bi	Biological	0.00	0.04	Solyc11g0	AOS2	0.12	-3.01
GO:00314	oxylipin bi	Biological	0.00	0.04	Solyc01g0	Solyc01g0	4.36	2.12

GO:00314	oxylipin bi	Biological	0.00	0.04	Solyc07g0	OPR3	0.47	-1.10
GO:00314	oxylipin bi	Biological	0.00	0.04	Solyc12g0	Solyc12g0	10.12	3.34
GO:00314	oxylipin bi	Biological	0.00	0.04	Solyc01g0	Solyc01g0	3.58	1.84
GO:00715	cell wall o	Biological	0.00	0.04	Solyc05g0	Solyc05g0	58.00	5.86
GO:00715	cell wall o	Biological	0.00	0.04	Solyc09g0	Solyc09g0	0.15	-2.74
GO:00715	cell wall o	Biological	0.00	0.04	Solyc04g0	Solyc04g0	2.56	1.36
GO:00715	cell wall o	Biological	0.00	0.04	Solyc03g1	Solyc03g1	0.37	-1.42
GO:00715	cell wall o	Biological	0.00	0.04	Solyc01g0	XTH1	0.31	-1.69
GO:00715	cell wall o	Biological	0.00	0.04	Solyc12g0	Solyc12g0	4.32	2.11
GO:00715	cell wall o	Biological	0.00	0.04	Solyc01g1	Solyc01g1	0.41	-1.30
GO:00715	cell wall o	Biological	0.00	0.04	Solyc03g0	Solyc03g0	4.24	2.08
GO:00715	cell wall o	Biological	0.00	0.04	Solyc05g0	Solyc05g0	0.33	-1.59
GO:00715	cell wall o	Biological	0.00	0.04	Solyc07g0	Solyc07g0	0.44	-1.18
GO:00715	cell wall o	Biological	0.00	0.04	Solyc10g0	Solyc10g0	2.57	1.36
GO:00715	cell wall o	Biological	0.00	0.04	Solyc09g0	Solyc09g0	0.39	-1.34
GO:00715	cell wall o	Biological	0.00	0.04	Solyc02g0	Solyc02g0	0.20	-2.30
GO:19015	organonitr	Biological	0.00	0.05	Solyc08g0	Solyc08g0	0.11	-3.24
GO:19015	organonitr	Biological	0.00	0.05	Solyc03g0	Solyc03g0	0.00	-11.21
GO:19015	organonitr	Biological	0.00	0.05	Solyc12g0	Solyc12g0	0.30	-1.74
GO:19015	organonitr	Biological	0.00	0.05	Solyc08g0	Solyc08g0	0.00	-10.99
GO:19015	organonitr	Biological	0.00	0.05	Solyc08g0	Solyc08g0	799.01	9.64
GO:19015	organonitr	Biological	0.00	0.05	Solyc08g0	Solyc08g0	0.17	-2.53
GO:00162	naringenin	Molecular	0.00	0.05	Solyc09g0	Solyc09g0	0.01	-7.13
GO:00162	naringenin	Molecular	0.00	0.05	Solyc05g0	Solyc05g0	0.01	-6.95
GO:01021	chalcone s	Molecular	0.00	0.05	Solyc09g0	Solyc09g0	0.01	-7.13
GO:01021	chalcone s	Molecular	0.00	0.05	Solyc05g0	Solyc05g0	0.01	-6.95
GO:00316	killing of c	Biological	0.00	0.05	Solyc08g0	Solyc08g0	0.27	-1.87
GO:00316	killing of c	Biological	0.00	0.05	Solyc09g0	Solyc09g0	0.01	-7.14
GO:00313	Ctf18 RFC	Cellular C	0.00	0.05	Solyc01g0	Solyc01g0	2.42	1.27
GO:00313	Ctf18 RFC	Cellular C	0.00	0.05	Solyc06g0	Solyc06g0	0.32	-1.65
GO:00484	stamen de	Biological	0.00	0.05	Solyc02g0	Solyc02g0	0.36	-1.49
GO:00484	stamen de	Biological	0.00	0.05	Solyc07g0	OPR3	0.47	-1.10
GO:00314	nucleosom	Molecular	0.00	0.06	Solyc01g0	Solyc01g0	2.65	1.41
GO:00314	nucleosom	Molecular	0.00	0.06	Solyc01g0	Solyc01g0	2.23	1.16
GO:00314	nucleosom	Molecular	0.00	0.06	Solyc01g0	Solyc01g0	2.64	1.40
GO:00314	nucleosom	Molecular	0.00	0.06	Solyc01g0	Solyc01g0	2.88	1.52
GO:00314	nucleosom	Molecular	0.00	0.06	Solyc12g0	Solyc12g0	2.57	1.36
GO:00063	transcripti	Biological	0.00	0.06	Solyc11g0	Solyc11g0	0.44	-1.18
GO:00063	transcripti	Biological	0.00	0.06	Solyc04g0	Solyc04g0	2.92	1.55
GO:00063	transcripti	Biological	0.00	0.06	Solyc07g0	Solyc07g0	0.40	-1.31
GO:00063	transcripti	Biological	0.00	0.06	Solyc04g0	Solyc04g0	0.13	-2.91
GO:00063	transcripti	Biological	0.00	0.06	Solyc04g0	Solyc04g0	0.47	-1.09
GO:00063	transcripti	Biological	0.00	0.06	Solyc08g0	Solyc08g0	0.48	-1.04
GO:00063	transcripti	Biological	0.00	0.06	Solyc07g0	Solyc07g0	0.26	-1.96
GO:00063	transcripti	Biological	0.00	0.06	Solyc12g0	Solyc12g0	5.61	2.49
GO:00063	transcripti	Biological	0.00	0.06	Solyc07g0	Solyc07g0	0.24	-2.07
GO:00063	transcripti	Biological	0.00	0.06	Solyc01g0	Solyc01g0	7223.16	12.82
GO:00063	transcripti	Biological	0.00	0.06	Solyc05g0	Solyc05g0	0.41	-1.29
GO:00063	transcripti	Biological	0.00	0.06	Solyc03g1	Solyc03g1	2.13	1.09
GO:00063	transcripti	Biological	0.00	0.06	Solyc10g0	Solyc10g0	0.32	-1.63
GO:00063	transcripti	Biological	0.00	0.06	Solyc03g0	Solyc03g0	0.06	-4.13

GO:00063	transcripti	Biological	0.00	0.06	Solyc06g0	Solyc06g0	3.49	1.81
GO:00063	transcripti	Biological	0.00	0.06	Solyc08g0	Solyc08g0	0.35	-1.53
GO:00063	transcripti	Biological	0.00	0.06	Solyc02g0	Solyc02g0	2.03	1.02
GO:00063	transcripti	Biological	0.00	0.06	Solyc08g0	Solyc08g0	0.38	-1.40
GO:00063	transcripti	Biological	0.00	0.06	Solyc02g0	Solyc02g0	0.42	-1.25
GO:00063	transcripti	Biological	0.00	0.06	Solyc09g0	Solyc09g0	0.37	-1.42
GO:00063	transcripti	Biological	0.00	0.06	Solyc03g0	Solyc03g0	0.29	-1.76
GO:00063	transcripti	Biological	0.00	0.06	Solyc02g0	Solyc02g0	0.34	-1.57
GO:00063	transcripti	Biological	0.00	0.06	Solyc11g0	Solyc11g0	5.14	2.36
GO:00063	transcripti	Biological	0.00	0.06	Solyc03g1	Solyc03g1	0.08	-3.61
GO:00063	transcripti	Biological	0.00	0.06	Solyc01g1	Solyc01g1	0.15	-2.78
GO:00063	transcripti	Biological	0.00	0.06	Solyc10g0	Solyc10g0	2.49	1.31
GO:00063	transcripti	Biological	0.00	0.06	Solyc01g0	Solyc01g0	0.48	-1.06
GO:00063	transcripti	Biological	0.00	0.06	Solyc12g0	Solyc12g0	0.39	-1.35
GO:00063	transcripti	Biological	0.00	0.06	Solyc04g0	Solyc04g0	0.37	-1.43
GO:00063	transcripti	Biological	0.00	0.06	Solyc03g1	Solyc03g1	0.45	-1.14
GO:00063	transcripti	Biological	0.00	0.06	Solyc04g0	Solyc04g0	0.48	-1.05
GO:00063	transcripti	Biological	0.00	0.06	Solyc03g1	Solyc03g1	0.40	-1.33
GO:00063	transcripti	Biological	0.00	0.06	Solyc09g0	Solyc09g0	0.49	-1.04
GO:00063	transcripti	Biological	0.00	0.06	Solyc08g0	Solyc08g0	0.45	-1.17
GO:00063	transcripti	Biological	0.00	0.06	Solyc01g0	Solyc01g0	0.42	-1.24
GO:00182	protein-ch	Biological	0.00	0.06	Solyc03g0	Solyc03g0	0.18	-2.46
GO:00182	protein-ch	Biological	0.00	0.06	Solyc10g0	Solyc10g0	0.43	-1.23
GO:00182	protein-ch	Biological	0.00	0.06	Solyc12g0	Solyc12g0	0.32	-1.65
GO:00182	protein-ch	Biological	0.00	0.06	Solyc05g0	Solyc05g0	0.15	-2.75
GO:00182	protein-ch	Biological	0.00	0.06	Solyc10g0	Solyc10g0	0.49	-1.02
GO:00182	protein-ch	Biological	0.00	0.06	Solyc03g0	Solyc03g0	0.45	-1.15
GO:00182	protein-ch	Biological	0.00	0.06	Solyc05g0	Solyc05g0	0.00	-11.15
GO:00182	protein-ch	Biological	0.00	0.06	Solyc01g1	CAP10A	0.35	-1.51
GO:00160	cellular res	Biological	0.00	0.06	Solyc08g0	Solyc08g0	0.49	-1.04
GO:00160	cellular res	Biological	0.00	0.06	Solyc12g0	Solyc12g0	0.39	-1.37
GO:00160	cellular res	Biological	0.00	0.06	Solyc01g0	Solyc01g0	0.11	-3.16
GO:00160	cellular res	Biological	0.00	0.06	Solyc04g0	Solyc04g0	0.24	-2.08
GO:00102	plastoglob	Cellular C	0.00	0.07	Solyc03g0	Solyc03g0	0.18	-2.46
GO:00102	plastoglob	Cellular C	0.00	0.07	Solyc05g0	Solyc05g0	0.15	-2.75
GO:00102	plastoglob	Cellular C	0.00	0.07	Solyc10g0	Solyc10g0	0.49	-1.02
GO:00102	plastoglob	Cellular C	0.00	0.07	Solyc05g0	Solyc05g0	0.00	-11.15
GO:00102	plastoglob	Cellular C	0.00	0.07	Solyc01g1	CAP10A	0.35	-1.51
GO:00312	replication	Cellular C	0.00	0.07	Solyc09g0	Solyc09g0	2.25	1.17
GO:00312	replication	Cellular C	0.00	0.07	Solyc01g1	Solyc01g1	2.41	1.27
GO:00312	replication	Cellular C	0.00	0.07	Solyc04g0	Solyc04g0	2.19	1.13
GO:00454	pectin cata	Biological	0.00	0.07	Solyc09g0	Solyc09g0	0.27	-1.88
GO:00454	pectin cata	Biological	0.00	0.07	Solyc03g1	Solyc03g1	0.37	-1.42
GO:00454	pectin cata	Biological	0.00	0.07	Solyc09g0	Solyc09g0	4.75	2.25
GO:00454	pectin cata	Biological	0.00	0.07	Solyc05g0	Solyc05g0	0.26	-1.96
GO:00454	pectin cata	Biological	0.00	0.07	Solyc07g0	Solyc07g0	0.25	-1.97
GO:00454	pectin cata	Biological	0.00	0.07	Solyc03g0	Solyc03g0	0.28	-1.83
GO:00454	pectin cata	Biological	0.00	0.07	Solyc01g0	Solyc01g0	4.89	2.29
GO:00454	pectin cata	Biological	0.00	0.07	Solyc07g0	Solyc07g0	2.40	1.27
GO:00356	oligopepti	Molecular	0.00	0.07	Solyc03g0	Solyc03g0	0.33	-1.60
GO:00356	oligopepti	Molecular	0.00	0.07	Solyc09g0	Solyc09g0	0.13	-2.95

GO:00356	oligopeptid	Molecular	0.00	0.07	Solyc03g0	Solyc03g0	0.48	-1.05
GO:00356	oligopeptid	Molecular	0.00	0.07	Solyc03g1	Solyc03g1	0.40	-1.31
GO:00356	oligopeptid	Molecular	0.00	0.07	Solyc05g0	Solyc05g0	0.40	-1.34
GO:00356	oligopeptid	Molecular	0.00	0.07	Solyc06g0	Solyc06g0	0.04	-4.72
GO:00356	oligopeptid	Molecular	0.00	0.07	Solyc04g0	Solyc04g0	0.49	-1.04
GO:00356	oligopeptid	Molecular	0.00	0.07	Solyc05g0	Solyc05g0	0.46	-1.13
GO:00712	cellular res	Biological	0.01	0.08	Solyc02g0	Solyc02g0	0.23	-2.13
GO:00712	cellular res	Biological	0.01	0.08	Solyc07g0	Solyc07g0	0.16	-2.62
GO:00712	cellular res	Biological	0.01	0.08	Solyc01g0	Solyc01g0	0.35	-1.51
GO:00712	cellular res	Biological	0.01	0.08	Solyc04g0	Solyc04g0	0.34	-1.54
GO:00046	phospholip	Molecular	0.01	0.08	Solyc08g0	Solyc08g0	0.47	-1.07
GO:00046	phospholip	Molecular	0.01	0.08	Solyc01g1	Solyc01g1	0.48	-1.05
GO:00046	phospholip	Molecular	0.01	0.08	Solyc02g0	Solyc02g0	7.41	2.89
GO:00046	phospholip	Molecular	0.01	0.08	Solyc04g0	Solyc04g0	0.43	-1.23
GO:00161	chlorophyl	Molecular	0.01	0.09	Solyc03g0	Solyc03g0	0.18	-2.46
GO:00161	chlorophyl	Molecular	0.01	0.09	Solyc10g0	Solyc10g0	0.43	-1.23
GO:00161	chlorophyl	Molecular	0.01	0.09	Solyc05g0	Solyc05g0	0.15	-2.75
GO:00161	chlorophyl	Molecular	0.01	0.09	Solyc10g0	Solyc10g0	0.49	-1.02
GO:00161	chlorophyl	Molecular	0.01	0.09	Solyc03g0	Solyc03g0	0.45	-1.15
GO:00161	chlorophyl	Molecular	0.01	0.09	Solyc05g0	Solyc05g0	0.00	-11.15
GO:00161	chlorophyl	Molecular	0.01	0.09	Solyc01g1	CAP10A	0.35	-1.51
GO:00058	plasma me	Cellular Co	0.01	0.10	Solyc12g0	Solyc12g0	0.28	-1.84
GO:00058	plasma me	Cellular Co	0.01	0.10	Solyc05g0	Solyc05g0	0.18	-2.51
GO:00058	plasma me	Cellular Co	0.01	0.10	Solyc06g0	Solyc06g0	0.08	-3.62
GO:00058	plasma me	Cellular Co	0.01	0.10	Solyc07g0	Solyc07g0	0.48	-1.07
GO:00058	plasma me	Cellular Co	0.01	0.10	Solyc02g0	Solyc02g0	0.47	-1.10
GO:00058	plasma me	Cellular Co	0.01	0.10	Solyc04g0	Solyc04g0	0.17	-2.54
GO:00058	plasma me	Cellular Co	0.01	0.10	Solyc09g0	Solyc09g0	0.48	-1.06
GO:00058	plasma me	Cellular Co	0.01	0.10	Solyc01g0	Solyc01g0	0.29	-1.78
GO:00058	plasma me	Cellular Co	0.01	0.10	Solyc02g0	Solyc02g0	0.42	-1.24
GO:00058	plasma me	Cellular Co	0.01	0.10	Solyc01g1	Solyc01g1	3.45	1.79
GO:00058	plasma me	Cellular Co	0.01	0.10	Solyc07g0	EIX1	0.33	-1.59
GO:00058	plasma me	Cellular Co	0.01	0.10	Solyc06g0	Solyc06g0	0.39	-1.35
GO:00058	plasma me	Cellular Co	0.01	0.10	Solyc09g0	Solyc09g0	2.10	1.07
GO:00058	plasma me	Cellular Co	0.01	0.10	Solyc05g0	Solyc05g0	0.46	-1.11
GO:00058	plasma me	Cellular Co	0.01	0.10	Solyc10g0	Solyc10g0	2.72	1.45
GO:00058	plasma me	Cellular Co	0.01	0.10	Solyc06g0	Solyc06g0	0.08	-3.69
GO:00058	plasma me	Cellular Co	0.01	0.10	Solyc11g0	Solyc11g0	0.40	-1.31
GO:00058	plasma me	Cellular Co	0.01	0.10	Solyc01g1	Solyc01g1	0.26	-1.97
GO:00058	plasma me	Cellular Co	0.01	0.10	Solyc06g0	Solyc06g0	2.41	1.27
GO:00058	plasma me	Cellular Co	0.01	0.10	Solyc08g0	Solyc08g0	0.39	-1.34
GO:00058	plasma me	Cellular Co	0.01	0.10	Solyc05g0	Solyc05g0	0.46	-1.12
GO:00058	plasma me	Cellular Co	0.01	0.10	Solyc11g0	Solyc11g0	0.47	-1.08
GO:00058	plasma me	Cellular Co	0.01	0.10	Solyc04g0	Solyc04g0	0.23	-2.11
GO:00058	plasma me	Cellular Co	0.01	0.10	Solyc06g0	Solyc06g0	0.41	-1.28
GO:00058	plasma me	Cellular Co	0.01	0.10	Solyc02g0	Solyc02g0	0.12	-3.00
GO:00058	plasma me	Cellular Co	0.01	0.10	Solyc07g0	Solyc07g0	0.25	-1.97
GO:00058	plasma me	Cellular Co	0.01	0.10	Solyc07g0	Solyc07g0	0.02	-5.66
GO:00058	plasma me	Cellular Co	0.01	0.10	Solyc11g0	Solyc11g0	2.74	1.45
GO:00058	plasma me	Cellular Co	0.01	0.10	Solyc06g0	Solyc06g0	2.69	1.43
GO:00058	plasma me	Cellular Co	0.01	0.10	Solyc12g0	Solyc12g0	2112.44	11.04

GO:00058	plasma me	Cellular Co	0.01	0.10	Solyc04g0	Solyc04g0	0.18	-2.51
GO:00058	plasma me	Cellular Co	0.01	0.10	Solyc03g1	Solyc03g1	0.40	-1.31
GO:00058	plasma me	Cellular Co	0.01	0.10	Solyc02g0	Solyc02g0	0.19	-2.42
GO:00058	plasma me	Cellular Co	0.01	0.10	Solyc06g0	Solyc06g0	0.08	-3.58
GO:00058	plasma me	Cellular Co	0.01	0.10	Solyc01g0	Solyc01g0	0.37	-1.43
GO:00058	plasma me	Cellular Co	0.01	0.10	Solyc07g0	Solyc07g0	0.46	-1.11
GO:00058	plasma me	Cellular Co	0.01	0.10	Solyc08g0	Solyc08g0	0.22	-2.16
GO:00058	plasma me	Cellular Co	0.01	0.10	Solyc04g0	Solyc04g0	0.46	-1.13
GO:00058	plasma me	Cellular Co	0.01	0.10	Solyc06g0	Solyc06g0	0.31	-1.70
GO:00058	plasma me	Cellular Co	0.01	0.10	Solyc02g0	Solyc02g0	0.06	-3.96
GO:00058	plasma me	Cellular Co	0.01	0.10	Solyc03g1	Solyc03g1	0.48	-1.05
GO:00058	plasma me	Cellular Co	0.01	0.10	Solyc09g0	Solyc09g0	0.43	-1.22
GO:00058	plasma me	Cellular Co	0.01	0.10	Solyc04g0	Solyc04g0	2.01	1.01
GO:00058	plasma me	Cellular Co	0.01	0.10	Solyc05g0	Solyc05g0	0.40	-1.34
GO:00058	plasma me	Cellular Co	0.01	0.10	Solyc11g0	Solyc11g0	2.39	1.25
GO:00058	plasma me	Cellular Co	0.01	0.10	Solyc07g0	Solyc07g0	0.44	-1.18
GO:00058	plasma me	Cellular Co	0.01	0.10	Solyc11g0	Solyc11g0	0.16	-2.61
GO:00058	plasma me	Cellular Co	0.01	0.10	Solyc01g0	Solyc01g0	0.12	-3.07
GO:00058	plasma me	Cellular Co	0.01	0.10	Solyc09g0	Solyc09g0	0.38	-1.40
GO:00058	plasma me	Cellular Co	0.01	0.10	Solyc09g0	Solyc09g0	0.08	-3.73
GO:00058	plasma me	Cellular Co	0.01	0.10	Solyc06g0	Solyc06g0	0.26	-1.94
GO:00058	plasma me	Cellular Co	0.01	0.10	Solyc03g1	Solyc03g1	668.93	9.39
GO:00058	plasma me	Cellular Co	0.01	0.10	Solyc10g0	Solyc10g0	0.42	-1.27
GO:00058	plasma me	Cellular Co	0.01	0.10	Solyc04g0	Solyc04g0	0.00	-8.72
GO:00058	plasma me	Cellular Co	0.01	0.10	Solyc01g0	Solyc01g0	0.19	-2.40
GO:00058	plasma me	Cellular Co	0.01	0.10	Solyc05g0	Solyc05g0	0.46	-1.13
GO:00098	fruit ripeni	Biological	0.01	0.10	Solyc09g0	Solyc09g0	0.07	-3.81
GO:00098	fruit ripeni	Biological	0.01	0.10	Solyc07g0	NCED1	0.30	-1.72
GO:00098	fruit ripeni	Biological	0.01	0.10	Solyc08g0	CYP707A	0.26	-1.93
GO:00098	fruit ripeni	Biological	0.01	0.10	Solyc05g0	Solyc05g0	0.33	-1.59
GO:00089	phosphoen	Molecular	0.01	0.10	Solyc04g0	Solyc04g0	0.34	-1.56
GO:00089	phosphoen	Molecular	0.01	0.10	Solyc09g0	Solyc09g0	0.21	-2.26
GO:00089	phosphoen	Molecular	0.01	0.10	Solyc11g0	Solyc11g0	0.33	-1.60
GO:00428	identical p	Molecular	0.01	0.10	Solyc06g0	Solyc06g0	0.40	-1.33
GO:00428	identical p	Molecular	0.01	0.10	Solyc07g0	OPR3	0.47	-1.10
GO:00428	identical p	Molecular	0.01	0.10	Solyc11g0	Solyc11g0	2.55	1.35
GO:00428	identical p	Molecular	0.01	0.10	Solyc11g0	Solyc11g0	2.39	1.25
GO:00428	identical p	Molecular	0.01	0.10	Solyc09g0	TD2	4.53	2.18
GO:00428	identical p	Molecular	0.01	0.10	Solyc12g0	Solyc12g0	0.48	-1.07
GO:00096	phenylpro	Biological	0.01	0.11	Solyc09g0	Solyc09g0	0.08	-3.59
GO:00096	phenylpro	Biological	0.01	0.11	Solyc06g0	Solyc06g0	0.40	-1.33
GO:00306	polyketide	Biological	0.01	0.11	Solyc09g0	Solyc09g0	0.01	-7.13
GO:00306	polyketide	Biological	0.01	0.11	Solyc05g0	Solyc05g0	0.01	-6.95
GO:00007	heterochro	Cellular Co	0.01	0.11	Solyc01g0	Solyc01g0	3.68	1.88
GO:00007	heterochro	Cellular Co	0.01	0.11	Solyc05g0	Solyc05g0	2.31	1.21
GO:20000	regulation	Biological	0.01	0.11	Solyc04g0	Solyc04g0	0.48	-1.06
GO:20000	regulation	Biological	0.01	0.11	Solyc06g0	Solyc06g0	0.08	-3.58
GO:00513	regulation	Biological	0.01	0.11	Solyc08g0	Solyc08g0	0.39	-1.34
GO:00513	regulation	Biological	0.01	0.11	Solyc06g0	Solyc06g0	0.08	-3.58
GO:00007	double-stra	Biological	0.01	0.11	Solyc08g0	Solyc08g0	2.48	1.31
GO:00007	double-stra	Biological	0.01	0.11	Solyc09g0	Solyc09g0	2.25	1.17

GO:00510	positive re	Biological	0.01	0.11	Solyc10g0	Solyc10g0	0.14	-2.88
GO:00510	positive re	Biological	0.01	0.11	Solyc01g0	Solyc01g0	0.10	-3.33
GO:00469	protein het	Molecular	0.01	0.11	Solyc01g0	Solyc01g0	3.68	1.88
GO:00469	protein het	Molecular	0.01	0.11	Solyc01g0	Solyc01g0	2.65	1.41
GO:00469	protein het	Molecular	0.01	0.11	Solyc04g0	Solyc04g0	2.55	1.35
GO:00469	protein het	Molecular	0.01	0.11	Solyc11g0	Solyc11g0	2.07	1.05
GO:00469	protein het	Molecular	0.01	0.11	Solyc01g0	Solyc01g0	2.23	1.16
GO:00469	protein het	Molecular	0.01	0.11	Solyc06g0	Solyc06g0	2.05	1.04
GO:00469	protein het	Molecular	0.01	0.11	Solyc01g0	Solyc01g0	2.64	1.40
GO:00469	protein het	Molecular	0.01	0.11	Solyc01g0	Solyc01g0	2.38	1.25
GO:00469	protein het	Molecular	0.01	0.11	Solyc11g0	Solyc11g0	2.46	1.30
GO:00469	protein het	Molecular	0.01	0.11	Solyc05g0	Solyc05g0	0.29	-1.78
GO:00469	protein het	Molecular	0.01	0.11	Solyc05g0	Solyc05g0	2.73	1.45
GO:00469	protein het	Molecular	0.01	0.11	Solyc01g0	Solyc01g0	2.88	1.52
GO:00469	protein het	Molecular	0.01	0.11	Solyc12g0	Solyc12g0	2.57	1.36
GO:00469	protein het	Molecular	0.01	0.11	Solyc02g0	Solyc02g0	2.15	1.10
GO:00469	protein het	Molecular	0.01	0.11	Solyc05g0	Solyc05g0	0.42	-1.26
GO:00469	protein het	Molecular	0.01	0.11	Solyc05g0	Solyc05g0	1995.76	10.96
GO:00045	chitinase a	Molecular	0.01	0.12	Solyc10g0	Solyc10g0	0.06	-4.12
GO:00045	chitinase a	Molecular	0.01	0.12	Solyc02g0	CHI3	0.24	-2.08
GO:00045	chitinase a	Molecular	0.01	0.12	Solyc05g0	Solyc05g0	0.26	-1.92
GO:00045	chitinase a	Molecular	0.01	0.12	Solyc10g0	CHI9	0.27	-1.88
GO:00045	chitinase a	Molecular	0.01	0.12	Solyc07g0	Solyc07g0	0.45	-1.16
GO:00427	hydrogen p	Biological	0.01	0.14	Solyc12g0	Solyc12g0	0.28	-1.84
GO:00427	hydrogen p	Biological	0.01	0.14	Solyc04g0	Solyc04g0	0.31	-1.69
GO:00427	hydrogen p	Biological	0.01	0.14	Solyc02g0	Solyc02g0	2.89	1.53
GO:00427	hydrogen p	Biological	0.01	0.14	Solyc09g0	Solyc09g0	2.43	1.28
GO:00427	hydrogen p	Biological	0.01	0.14	Solyc01g1	Solyc01g1	2.95	1.56
GO:00427	hydrogen p	Biological	0.01	0.14	Solyc03g0	Solyc03g0	0.39	-1.34
GO:00427	hydrogen p	Biological	0.01	0.14	Solyc03g0	Solyc03g0	2.14	1.10
GO:00427	hydrogen p	Biological	0.01	0.14	Solyc06g0	Solyc06g0	9.98	3.32
GO:00427	hydrogen p	Biological	0.01	0.14	Solyc02g0	Solyc02g0	2.29	1.19
GO:00427	hydrogen p	Biological	0.01	0.14	Solyc02g0	Solyc02g0	0.10	-3.34
GO:00427	hydrogen p	Biological	0.01	0.14	Solyc04g0	Solyc04g0	0.49	-1.04
GO:00427	hydrogen p	Biological	0.01	0.14	Solyc01g1	Solyc01g1	0.00	-9.56
GO:00159	carbon fixa	Biological	0.01	0.14	Solyc04g0	Solyc04g0	0.34	-1.56
GO:00159	carbon fixa	Biological	0.01	0.14	Solyc03g0	RBCS-2A	0.34	-1.58
GO:00159	carbon fixa	Biological	0.01	0.14	Solyc09g0	Solyc09g0	0.21	-2.26
GO:00159	carbon fixa	Biological	0.01	0.14	Solyc11g0	Solyc11g0	0.33	-1.60
GO:00063	regulation	Biological	0.01	0.15	Solyc06g0	Solyc06g0	3.24	1.69
GO:00063	regulation	Biological	0.01	0.15	Solyc09g0	Solyc09g0	2.30	1.20
GO:00063	regulation	Biological	0.01	0.15	Solyc03g0	Solyc03g0	3.36	1.75
GO:00063	regulation	Biological	0.01	0.15	Solyc02g0	Solyc02g0	0.48	-1.05
GO:00063	regulation	Biological	0.01	0.15	Solyc11g0	Solyc11g0	0.44	-1.18
GO:00063	regulation	Biological	0.01	0.15	Solyc09g0	Solyc09g0	0.31	-1.67
GO:00063	regulation	Biological	0.01	0.15	Solyc03g0	Solyc03g0	2.41	1.27
GO:00063	regulation	Biological	0.01	0.15	Solyc04g0	Solyc04g0	2.92	1.55
GO:00063	regulation	Biological	0.01	0.15	Solyc04g0	Solyc04g0	0.42	-1.24
GO:00063	regulation	Biological	0.01	0.15	Solyc08g0	Solyc08g0	0.50	-1.01
GO:00063	regulation	Biological	0.01	0.15	Solyc05g0	Solyc05g0	0.08	-3.67
GO:00063	regulation	Biological	0.01	0.15	Solyc03g1	Solyc03g1	0.04	-4.59

GO:00063	regulation	Biological	0.01	0.15	Solyc12g0	Solyc12g0	0.44	-1.20
GO:00063	regulation	Biological	0.01	0.15	Solyc07g0	Solyc07g0	0.40	-1.31
GO:00063	regulation	Biological	0.01	0.15	Solyc09g0	Solyc09g0	0.47	-1.10
GO:00063	regulation	Biological	0.01	0.15	Solyc04g0	Solyc04g0	0.13	-2.91
GO:00063	regulation	Biological	0.01	0.15	Solyc06g0	Solyc06g0	0.21	-2.28
GO:00063	regulation	Biological	0.01	0.15	Solyc09g0	Solyc09g0	0.48	-1.06
GO:00063	regulation	Biological	0.01	0.15	Solyc02g0	Solyc02g0	3.87	1.95
GO:00063	regulation	Biological	0.01	0.15	Solyc05g0	Solyc05g0	3.62	1.86
GO:00063	regulation	Biological	0.01	0.15	Solyc01g0	Solyc01g0	0.35	-1.51
GO:00063	regulation	Biological	0.01	0.15	Solyc04g0	Solyc04g0	0.47	-1.09
GO:00063	regulation	Biological	0.01	0.15	Solyc05g0	Solyc05g0	0.40	-1.33
GO:00063	regulation	Biological	0.01	0.15	Solyc02g0	Solyc02g0	0.33	-1.60
GO:00063	regulation	Biological	0.01	0.15	Solyc06g0	Solyc06g0	2.04	1.03
GO:00063	regulation	Biological	0.01	0.15	Solyc01g0	Solyc01g0	0.11	-3.13
GO:00063	regulation	Biological	0.01	0.15	Solyc08g0	Solyc08g0	0.48	-1.04
GO:00063	regulation	Biological	0.01	0.15	Solyc01g0	Solyc01g0	0.34	-1.55
GO:00063	regulation	Biological	0.01	0.15	Solyc11g0	Solyc11g0	0.48	-1.05
GO:00063	regulation	Biological	0.01	0.15	Solyc10g0	Solyc10g0	0.00	-13.06
GO:00063	regulation	Biological	0.01	0.15	Solyc03g0	Solyc03g0	0.40	-1.33
GO:00063	regulation	Biological	0.01	0.15	Solyc04g0	Solyc04g0	2.02	1.01
GO:00063	regulation	Biological	0.01	0.15	Solyc01g1	Solyc01g1	3.62	1.85
GO:00063	regulation	Biological	0.01	0.15	Solyc02g0	Solyc02g0	0.19	-2.41
GO:00063	regulation	Biological	0.01	0.15	Solyc10g0	Solyc10g0	0.23	-2.10
GO:00063	regulation	Biological	0.01	0.15	Solyc07g0	Solyc07g0	0.24	-2.07
GO:00063	regulation	Biological	0.01	0.15	Solyc05g0	Solyc05g0	0.48	-1.04
GO:00063	regulation	Biological	0.01	0.15	Solyc09g0	Solyc09g0	0.00	-11.89
GO:00063	regulation	Biological	0.01	0.15	Solyc08g0	Solyc08g0	0.28	-1.83
GO:00063	regulation	Biological	0.01	0.15	Solyc01g0	Solyc01g0	7223.16	12.82
GO:00063	regulation	Biological	0.01	0.15	Solyc05g0	Solyc05g0	0.29	-1.78
GO:00063	regulation	Biological	0.01	0.15	Solyc05g0	Solyc05g0	0.41	-1.29
GO:00063	regulation	Biological	0.01	0.15	Solyc07g0	Solyc07g0	0.07	-3.80
GO:00063	regulation	Biological	0.01	0.15	Solyc03g1	Solyc03g1	2.13	1.09
GO:00063	regulation	Biological	0.01	0.15	Solyc10g0	Solyc10g0	0.32	-1.64
GO:00063	regulation	Biological	0.01	0.15	Solyc03g0	Solyc03g0	0.06	-4.13
GO:00063	regulation	Biological	0.01	0.15	Solyc05g0	Solyc05g0	0.00	-11.04
GO:00063	regulation	Biological	0.01	0.15	Solyc02g0	Solyc02g0	0.15	-2.75
GO:00063	regulation	Biological	0.01	0.15	Solyc06g0	Solyc06g0	3.49	1.81
GO:00063	regulation	Biological	0.01	0.15	Solyc08g0	Solyc08g0	0.35	-1.53
GO:00063	regulation	Biological	0.01	0.15	Solyc05g0	Solyc05g0	2.08	1.06
GO:00063	regulation	Biological	0.01	0.15	Solyc02g0	Solyc02g0	2.03	1.02
GO:00063	regulation	Biological	0.01	0.15	Solyc03g1	Solyc03g1	0.44	-1.19
GO:00063	regulation	Biological	0.01	0.15	Solyc06g0	Solyc06g0	0.38	-1.41
GO:00063	regulation	Biological	0.01	0.15	Solyc02g0	Solyc02g0	0.45	-1.17
GO:00063	regulation	Biological	0.01	0.15	Solyc08g0	Solyc08g0	0.38	-1.40
GO:00063	regulation	Biological	0.01	0.15	Solyc03g1	Solyc03g1	2.90	1.54
GO:00063	regulation	Biological	0.01	0.15	Solyc02g0	Solyc02g0	0.42	-1.25
GO:00063	regulation	Biological	0.01	0.15	Solyc08g0	Solyc08g0	2.25	1.17
GO:00063	regulation	Biological	0.01	0.15	Solyc03g0	Solyc03g0	0.29	-1.76
GO:00063	regulation	Biological	0.01	0.15	Solyc02g0	Solyc02g0	0.34	-1.57
GO:00063	regulation	Biological	0.01	0.15	Solyc02g0	Solyc02g0	0.00	-11.41
GO:00063	regulation	Biological	0.01	0.15	Solyc06g0	Solyc06g0	4.54	2.18

GO:00063	regulation	Biological	0.01	0.15	Solyc10g0	Solyc10g0	12.09	3.60
GO:00063	regulation	Biological	0.01	0.15	Solyc11g0	Solyc11g0	0.00	-9.30
GO:00063	regulation	Biological	0.01	0.15	Solyc11g0	Solyc11g0	5.14	2.36
GO:00063	regulation	Biological	0.01	0.15	Solyc03g1	Solyc03g1	0.08	-3.61
GO:00063	regulation	Biological	0.01	0.15	Solyc02g0	Solyc02g0	0.34	-1.56
GO:00063	regulation	Biological	0.01	0.15	Solyc05g0	Solyc05g0	0.00	-8.91
GO:00063	regulation	Biological	0.01	0.15	Solyc03g0	Solyc03g0	11.59	3.53
GO:00063	regulation	Biological	0.01	0.15	Solyc10g0	Solyc10g0	0.33	-1.62
GO:00063	regulation	Biological	0.01	0.15	Solyc10g0	Solyc10g0	2.49	1.31
GO:00063	regulation	Biological	0.01	0.15	Solyc10g0	Solyc10g0	0.16	-2.65
GO:00063	regulation	Biological	0.01	0.15	Solyc10g0	Solyc10g0	1745.96	10.77
GO:00063	regulation	Biological	0.01	0.15	Solyc04g0	Solyc04g0	0.37	-1.43
GO:00063	regulation	Biological	0.01	0.15	Solyc03g1	Solyc03g1	0.45	-1.14
GO:00063	regulation	Biological	0.01	0.15	Solyc05g0	Solyc05g0	0.42	-1.26
GO:00063	regulation	Biological	0.01	0.15	Solyc06g0	Solyc06g0	0.22	-2.19
GO:00063	regulation	Biological	0.01	0.15	Solyc02g0	PTI5	0.07	-3.77
GO:00063	regulation	Biological	0.01	0.15	Solyc03g0	Solyc03g0	0.39	-1.37
GO:00063	regulation	Biological	0.01	0.15	Solyc03g0	Solyc03g0	0.21	-2.26
GO:00063	regulation	Biological	0.01	0.15	Solyc05g0	Solyc05g0	1995.76	10.96
GO:00063	regulation	Biological	0.01	0.15	Solyc05g0	Solyc05g0	0.46	-1.13
GO:00063	regulation	Biological	0.01	0.15	Solyc02g0	Solyc02g0	0.00	-8.58
GO:00063	regulation	Biological	0.01	0.15	Solyc04g0	Solyc04g0	0.48	-1.05
GO:00063	regulation	Biological	0.01	0.15	Solyc03g1	Solyc03g1	0.40	-1.33
GO:00063	regulation	Biological	0.01	0.15	Solyc03g1	Solyc03g1	0.24	-2.05
GO:00063	regulation	Biological	0.01	0.15	Solyc09g0	Solyc09g0	0.49	-1.04
GO:00063	regulation	Biological	0.01	0.15	Solyc08g0	Solyc08g0	0.45	-1.17
GO:00063	regulation	Biological	0.01	0.15	Solyc08g0	Solyc08g0	0.00	-9.51
GO:00435	sequence-s	Molecular	0.01	0.15	Solyc09g0	Solyc09g0	2.30	1.20
GO:00435	sequence-s	Molecular	0.01	0.15	Solyc01g0	Solyc01g0	0.04	-4.49
GO:00435	sequence-s	Molecular	0.01	0.15	Solyc02g0	Solyc02g0	0.48	-1.05
GO:00435	sequence-s	Molecular	0.01	0.15	Solyc11g0	Solyc11g0	0.44	-1.18
GO:00435	sequence-s	Molecular	0.01	0.15	Solyc09g0	Solyc09g0	0.31	-1.67
GO:00435	sequence-s	Molecular	0.01	0.15	Solyc04g0	Solyc04g0	2.92	1.55
GO:00435	sequence-s	Molecular	0.01	0.15	Solyc05g0	Solyc05g0	0.08	-3.67
GO:00435	sequence-s	Molecular	0.01	0.15	Solyc03g1	Solyc03g1	0.04	-4.59
GO:00435	sequence-s	Molecular	0.01	0.15	Solyc06g0	Solyc06g0	0.21	-2.28
GO:00435	sequence-s	Molecular	0.01	0.15	Solyc09g0	Solyc09g0	0.48	-1.06
GO:00435	sequence-s	Molecular	0.01	0.15	Solyc02g0	Solyc02g0	3.87	1.95
GO:00435	sequence-s	Molecular	0.01	0.15	Solyc05g0	Solyc05g0	3.62	1.86
GO:00435	sequence-s	Molecular	0.01	0.15	Solyc01g0	Solyc01g0	0.35	-1.51
GO:00435	sequence-s	Molecular	0.01	0.15	Solyc04g0	Solyc04g0	0.47	-1.09
GO:00435	sequence-s	Molecular	0.01	0.15	Solyc02g0	Solyc02g0	0.33	-1.60
GO:00435	sequence-s	Molecular	0.01	0.15	Solyc01g0	Solyc01g0	0.11	-3.13
GO:00435	sequence-s	Molecular	0.01	0.15	Solyc01g0	Solyc01g0	0.34	-1.55
GO:00435	sequence-s	Molecular	0.01	0.15	Solyc07g0	Solyc07g0	0.26	-1.96
GO:00435	sequence-s	Molecular	0.01	0.15	Solyc04g0	Solyc04g0	2.02	1.01
GO:00435	sequence-s	Molecular	0.01	0.15	Solyc02g0	Solyc02g0	0.19	-2.41
GO:00435	sequence-s	Molecular	0.01	0.15	Solyc06g0	Solyc06g0	0.39	-1.36
GO:00435	sequence-s	Molecular	0.01	0.15	Solyc09g0	Solyc09g0	0.00	-11.89
GO:00435	sequence-s	Molecular	0.01	0.15	Solyc08g0	Solyc08g0	0.28	-1.83
GO:00435	sequence-s	Molecular	0.01	0.15	Solyc05g0	Solyc05g0	0.41	-1.29

GO:00435	sequence-s	Molecular	0.01	0.15	Solyc07g0	Solyc07g0	0.07	-3.80
GO:00435	sequence-s	Molecular	0.01	0.15	Solyc05g0	Solyc05g0	0.00	-11.04
GO:00435	sequence-s	Molecular	0.01	0.15	Solyc03g1	Solyc03g1	0.37	-1.42
GO:00435	sequence-s	Molecular	0.01	0.15	Solyc05g0	Solyc05g0	2.08	1.06
GO:00435	sequence-s	Molecular	0.01	0.15	Solyc03g1	Solyc03g1	0.44	-1.19
GO:00435	sequence-s	Molecular	0.01	0.15	Solyc02g0	Solyc02g0	0.45	-1.17
GO:00435	sequence-s	Molecular	0.01	0.15	Solyc03g1	Solyc03g1	2.90	1.54
GO:00435	sequence-s	Molecular	0.01	0.15	Solyc05g0	Solyc05g0	0.00	-8.91
GO:00435	sequence-s	Molecular	0.01	0.15	Solyc03g0	Solyc03g0	11.59	3.53
GO:00435	sequence-s	Molecular	0.01	0.15	Solyc01g1	Solyc01g1	0.15	-2.78
GO:00435	sequence-s	Molecular	0.01	0.15	Solyc12g0	Solyc12g0	0.39	-1.35
GO:00435	sequence-s	Molecular	0.01	0.15	Solyc10g0	Solyc10g0	0.16	-2.65
GO:00435	sequence-s	Molecular	0.01	0.15	Solyc06g0	Solyc06g0	0.22	-2.19
GO:00435	sequence-s	Molecular	0.01	0.15	Solyc05g0	Solyc05g0	0.46	-1.13
GO:00435	sequence-s	Molecular	0.01	0.15	Solyc11g0	Solyc11g0	0.00	-9.45
GO:00435	sequence-s	Molecular	0.01	0.15	Solyc08g0	Solyc08g0	0.00	-9.51
GO:00435	sequence-s	Molecular	0.01	0.15	Solyc02g0	Solyc02g0	0.00	-9.32
GO:00153	peptide:pr	Molecular	0.01	0.15	Solyc09g0	Solyc09g0	0.13	-2.95
GO:00153	peptide:pr	Molecular	0.01	0.15	Solyc03g1	Solyc03g1	0.40	-1.31
GO:00153	peptide:pr	Molecular	0.01	0.15	Solyc05g0	Solyc05g0	0.40	-1.34
GO:00153	peptide:pr	Molecular	0.01	0.15	Solyc06g0	Solyc06g0	0.04	-4.72
GO:00153	peptide:pr	Molecular	0.01	0.15	Solyc05g0	Solyc05g0	0.46	-1.13
GO:19046	peptide tra	Molecular	0.01	0.15	Solyc09g0	Solyc09g0	0.13	-2.95
GO:19046	peptide tra	Molecular	0.01	0.15	Solyc03g1	Solyc03g1	0.40	-1.31
GO:19046	peptide tra	Molecular	0.01	0.15	Solyc05g0	Solyc05g0	0.40	-1.34
GO:19046	peptide tra	Molecular	0.01	0.15	Solyc06g0	Solyc06g0	0.04	-4.72
GO:19046	peptide tra	Molecular	0.01	0.15	Solyc05g0	Solyc05g0	0.46	-1.13
GO:00125	monolayer	Cellular C	0.01	0.16	Solyc07g0	Solyc07g0	0.45	-1.16
GO:00125	monolayer	Cellular C	0.01	0.16	Solyc03g1	Solyc03g1	0.44	-1.18
GO:00125	monolayer	Cellular C	0.01	0.16	Solyc06g0	Solyc06g0	0.38	-1.40
GO:00094	response t	Biological	0.01	0.16	Solyc02g0	Solyc02g0	0.44	-1.18
GO:00094	response t	Biological	0.01	0.16	Solyc07g0	NCED1	0.30	-1.72
GO:00094	response t	Biological	0.01	0.16	Solyc01g1	Solyc01g1	0.47	-1.09
GO:00094	response t	Biological	0.01	0.16	Solyc04g0	Solyc04g0	0.48	-1.06
GO:00094	response t	Biological	0.01	0.16	Solyc08g0	CYP707A	0.26	-1.93
GO:00094	response t	Biological	0.01	0.16	Solyc01g0	Solyc01g0	0.45	-1.15
GO:00094	response t	Biological	0.01	0.16	Solyc06g0	Solyc06g0	0.48	-1.07
GO:00094	response t	Biological	0.01	0.16	Solyc12g0	Solyc12g0	0.48	-1.07
GO:00068	xenobiotic	Biological	0.02	0.17	Solyc04g0	Solyc04g0	0.19	-2.40
GO:00068	xenobiotic	Biological	0.02	0.17	Solyc03g1	Solyc03g1	0.47	-1.08
GO:00068	xenobiotic	Biological	0.02	0.17	Solyc02g0	Solyc02g0	0.33	-1.61
GO:00068	xenobiotic	Biological	0.02	0.17	Solyc07g0	Solyc07g0	0.00	-9.97
GO:00068	xenobiotic	Biological	0.02	0.17	Solyc08g0	Solyc08g0	3.30	1.72
GO:00455	9-cis-epox	Molecular	0.02	0.18	Solyc07g0	NCED1	0.30	-1.72
GO:00455	9-cis-epox	Molecular	0.02	0.18	Solyc05g0	Solyc05g0	0.31	-1.69
GO:00066	fatty acid r	Biological	0.02	0.20	Solyc11g0	AOS2	0.12	-3.01
GO:00066	fatty acid r	Biological	0.02	0.20	Solyc01g0	Solyc01g0	4.36	2.12
GO:00066	fatty acid r	Biological	0.02	0.20	Solyc08g0	Solyc08g0	0.21	-2.27
GO:00066	fatty acid r	Biological	0.02	0.20	Solyc07g0	OPR3	0.47	-1.10
GO:00066	fatty acid r	Biological	0.02	0.20	Solyc12g0	Solyc12g0	10.12	3.34
GO:00066	fatty acid r	Biological	0.02	0.20	Solyc02g0	Solyc02g0	1436.80	10.49

GO:00066	fatty acid r	Biological	0.02	0.20	Solyc01g1	Solyc01g1	0.50	-1.01
GO:00095	plant-type	Cellular C	0.02	0.20	Solyc01g1	Solyc01g1	2.32	1.21
GO:00095	plant-type	Cellular C	0.02	0.20	Solyc03g1	Solyc03g1	0.41	-1.27
GO:00095	plant-type	Cellular C	0.02	0.20	Solyc03g0	Solyc03g0	2.58	1.37
GO:00095	plant-type	Cellular C	0.02	0.20	Solyc01g1	Solyc01g1	0.41	-1.30
GO:00095	plant-type	Cellular C	0.02	0.20	Solyc08g0	Solyc08g0	0.48	-1.06
GO:00095	plant-type	Cellular C	0.02	0.20	Solyc02g0	Solyc02g0	6.42	2.68
GO:00095	plant-type	Cellular C	0.02	0.20	Solyc01g0	Solyc01g0	0.18	-2.51
GO:00095	plant-type	Cellular C	0.02	0.20	Solyc09g0	Solyc09g0	2.43	1.28
GO:00095	plant-type	Cellular C	0.02	0.20	Solyc01g1	Solyc01g1	2.95	1.56
GO:00425	photosyste	Biological	0.02	0.20	Solyc02g0	PSBO	0.48	-1.07
GO:00425	photosyste	Biological	0.02	0.20	Solyc07g0	Solyc07g0	0.41	-1.28
GO:00425	photosyste	Biological	0.02	0.20	Solyc06g0	Solyc06g0	0.31	-1.71
GO:00069	response to	Biological	0.02	0.20	Solyc12g0	Solyc12g0	0.32	-1.65
GO:00069	response to	Biological	0.02	0.20	Solyc01g0	Solyc01g0	0.23	-2.14
GO:00069	response to	Biological	0.02	0.20	Solyc03g0	Solyc03g0	4.19	2.07
GO:00169	cell wall m	Biological	0.02	0.21	Solyc10g0	Solyc10g0	0.06	-4.12
GO:00169	cell wall m	Biological	0.02	0.21	Solyc02g0	CHI3	0.24	-2.08
GO:00169	cell wall m	Biological	0.02	0.21	Solyc10g0	CHI9	0.27	-1.88
GO:00169	cell wall m	Biological	0.02	0.21	Solyc09g0	Solyc09g0	0.39	-1.34
GO:00052	voltage-ga	Molecular	0.02	0.21	Solyc01g1	Solyc01g1	0.43	-1.23
GO:00052	voltage-ga	Molecular	0.02	0.21	Solyc05g0	Solyc05g0	11.71	3.55
GO:00052	voltage-ga	Molecular	0.02	0.21	Solyc08g0	Solyc08g0	0.40	-1.32
GO:00052	voltage-ga	Molecular	0.02	0.21	Solyc12g0	Solyc12g0	0.48	-1.07
GO:00160	integral co	Cellular C	0.02	0.23	Solyc09g0	Solyc09g0	2.11	1.07
GO:00160	integral co	Cellular C	0.02	0.23	Solyc03g0	Solyc03g0	0.35	-1.50
GO:00160	integral co	Cellular C	0.02	0.23	Solyc09g0	Solyc09g0	0.14	-2.81
GO:00160	integral co	Cellular C	0.02	0.23	Solyc06g0	Solyc06g0	3.24	1.69
GO:00160	integral co	Cellular C	0.02	0.23	Solyc01g0	Solyc01g0	0.27	-1.87
GO:00160	integral co	Cellular C	0.02	0.23	Solyc01g1	Solyc01g1	4.02	2.01
GO:00160	integral co	Cellular C	0.02	0.23	Solyc01g1	Solyc01g1	3.04	1.60
GO:00160	integral co	Cellular C	0.02	0.23	Solyc02g0	Solyc02g0	2.02	1.01
GO:00160	integral co	Cellular C	0.02	0.23	Solyc02g0	Solyc02g0	0.46	-1.12
GO:00160	integral co	Cellular C	0.02	0.23	Solyc11g0	Solyc11g0	0.31	-1.71
GO:00160	integral co	Cellular C	0.02	0.23	Solyc03g0	Solyc03g0	0.36	-1.49
GO:00160	integral co	Cellular C	0.02	0.23	Solyc03g1	Solyc03g1	0.11	-3.23
GO:00160	integral co	Cellular C	0.02	0.23	Solyc05g0	Solyc05g0	0.18	-2.51
GO:00160	integral co	Cellular C	0.02	0.23	Solyc05g0	Solyc05g0	0.22	-2.15
GO:00160	integral co	Cellular C	0.02	0.23	Solyc06g0	Solyc06g0	0.08	-3.62
GO:00160	integral co	Cellular C	0.02	0.23	Solyc03g0	Solyc03g0	0.48	-1.07
GO:00160	integral co	Cellular C	0.02	0.23	Solyc09g0	Solyc09g0	2.64	1.40
GO:00160	integral co	Cellular C	0.02	0.23	Solyc06g0	Solyc06g0	0.37	-1.44
GO:00160	integral co	Cellular C	0.02	0.23	Solyc02g0	Solyc02g0	0.43	-1.20
GO:00160	integral co	Cellular C	0.02	0.23	Solyc05g0	Solyc05g0	0.46	-1.12
GO:00160	integral co	Cellular C	0.02	0.23	Solyc03g1	Solyc03g1	0.40	-1.32
GO:00160	integral co	Cellular C	0.02	0.23	Solyc03g0	Solyc03g0	0.11	-3.22
GO:00160	integral co	Cellular C	0.02	0.23	Solyc09g0	Solyc09g0	0.15	-2.74
GO:00160	integral co	Cellular C	0.02	0.23	Solyc09g0	Solyc09g0	0.44	-1.17
GO:00160	integral co	Cellular C	0.02	0.23	Solyc12g0	Solyc12g0	0.47	-1.08
GO:00160	integral co	Cellular C	0.02	0.23	Solyc03g0	Solyc03g0	0.18	-2.46
GO:00160	integral co	Cellular C	0.02	0.23	Solyc09g0	Solyc09g0	2.87	1.52

GO:00160	integral co	Cellular C	0.02	0.23	Solyc08g0	Solyc08g0	0.19	-2.37
GO:00160	integral co	Cellular C	0.02	0.23	Solyc10g0	Solyc10g0	0.43	-1.23
GO:00160	integral co	Cellular C	0.02	0.23	Solyc09g0	Solyc09g0	0.43	-1.22
GO:00160	integral co	Cellular C	0.02	0.23	Solyc07g0	Solyc07g0	0.45	-1.16
GO:00160	integral co	Cellular C	0.02	0.23	Solyc02g0	Solyc02g0	0.47	-1.10
GO:00160	integral co	Cellular C	0.02	0.23	Solyc04g0	Solyc04g0	0.17	-2.54
GO:00160	integral co	Cellular C	0.02	0.23	Solyc08g0	Solyc08g0	0.30	-1.75
GO:00160	integral co	Cellular C	0.02	0.23	Solyc08g0	CYP707A	0.26	-1.93
GO:00160	integral co	Cellular C	0.02	0.23	Solyc04g0	Solyc04g0	0.45	-1.15
GO:00160	integral co	Cellular C	0.02	0.23	Solyc12g0	Solyc12g0	2.45	1.29
GO:00160	integral co	Cellular C	0.02	0.23	Solyc01g1	Solyc01g1	3.45	1.79
GO:00160	integral co	Cellular C	0.02	0.23	Solyc06g0	Solyc06g0	0.47	-1.10
GO:00160	integral co	Cellular C	0.02	0.23	Solyc01g0	Solyc01g0	0.46	-1.13
GO:00160	integral co	Cellular C	0.02	0.23	Solyc08g0	Solyc08g0	0.50	-1.01
GO:00160	integral co	Cellular C	0.02	0.23	Solyc11g0	Solyc11g0	2.10	1.07
GO:00160	integral co	Cellular C	0.02	0.23	Solyc01g0	Solyc01g0	0.50	-1.01
GO:00160	integral co	Cellular C	0.02	0.23	Solyc03g1	Solyc03g1	0.39	-1.35
GO:00160	integral co	Cellular C	0.02	0.23	Solyc08g0	Solyc08g0	0.26	-1.92
GO:00160	integral co	Cellular C	0.02	0.23	Solyc07g0	EIX1	0.33	-1.59
GO:00160	integral co	Cellular C	0.02	0.23	Solyc03g1	Solyc03g1	0.37	-1.42
GO:00160	integral co	Cellular C	0.02	0.23	Solyc07g0	Solyc07g0	0.44	-1.19
GO:00160	integral co	Cellular C	0.02	0.23	Solyc05g0	Solyc05g0	0.37	-1.42
GO:00160	integral co	Cellular C	0.02	0.23	Solyc06g0	Solyc06g0	0.39	-1.35
GO:00160	integral co	Cellular C	0.02	0.23	Solyc09g0	Solyc09g0	2.28	1.19
GO:00160	integral co	Cellular C	0.02	0.23	Solyc07g0	Solyc07g0	2.29	1.19
GO:00160	integral co	Cellular C	0.02	0.23	Solyc01g1	Solyc01g1	0.43	-1.23
GO:00160	integral co	Cellular C	0.02	0.23	Solyc07g0	Solyc07g0	0.30	-1.76
GO:00160	integral co	Cellular C	0.02	0.23	Solyc12g0	Solyc12g0	0.31	-1.70
GO:00160	integral co	Cellular C	0.02	0.23	Solyc03g1	Solyc03g1	0.44	-1.18
GO:00160	integral co	Cellular C	0.02	0.23	Solyc09g0	Solyc09g0	0.18	-2.47
GO:00160	integral co	Cellular C	0.02	0.23	Solyc05g0	Solyc05g0	0.38	-1.40
GO:00160	integral co	Cellular C	0.02	0.23	Solyc09g0	Solyc09g0	2.10	1.07
GO:00160	integral co	Cellular C	0.02	0.23	Solyc05g0	Solyc05g0	0.47	-1.08
GO:00160	integral co	Cellular C	0.02	0.23	Solyc12g0	Solyc12g0	0.33	-1.60
GO:00160	integral co	Cellular C	0.02	0.23	Solyc05g0	Solyc05g0	0.46	-1.11
GO:00160	integral co	Cellular C	0.02	0.23	Solyc01g0	Solyc01g0	0.50	-1.01
GO:00160	integral co	Cellular C	0.02	0.23	Solyc02g0	Solyc02g0	0.41	-1.30
GO:00160	integral co	Cellular C	0.02	0.23	Solyc10g0	Solyc10g0	2.72	1.45
GO:00160	integral co	Cellular C	0.02	0.23	Solyc02g0	Solyc02g0	0.36	-1.49
GO:00160	integral co	Cellular C	0.02	0.23	Solyc11g0	Solyc11g0	0.48	-1.06
GO:00160	integral co	Cellular C	0.02	0.23	Solyc09g0	Solyc09g0	0.20	-2.32
GO:00160	integral co	Cellular C	0.02	0.23	Solyc06g0	Solyc06g0	0.08	-3.69
GO:00160	integral co	Cellular C	0.02	0.23	Solyc11g0	Solyc11g0	0.36	-1.49
GO:00160	integral co	Cellular C	0.02	0.23	Solyc01g1	Solyc01g1	0.26	-1.97
GO:00160	integral co	Cellular C	0.02	0.23	Solyc04g0	Solyc04g0	0.48	-1.07
GO:00160	integral co	Cellular C	0.02	0.23	Solyc06g0	Solyc06g0	0.20	-2.31
GO:00160	integral co	Cellular C	0.02	0.23	Solyc03g0	Solyc03g0	3.70	1.89
GO:00160	integral co	Cellular C	0.02	0.23	Solyc08g0	Solyc08g0	0.47	-1.09
GO:00160	integral co	Cellular C	0.02	0.23	Solyc05g0	Solyc05g0	0.15	-2.75
GO:00160	integral co	Cellular C	0.02	0.23	Solyc11g0	Solyc11g0	3.98	1.99
GO:00160	integral co	Cellular C	0.02	0.23	Solyc08g0	Solyc08g0	0.39	-1.34

GO:00160	integral co	Cellular C	0.02	0.23	Solyc05g0	Solyc05g0	0.09	-3.40
GO:00160	integral co	Cellular C	0.02	0.23	Solyc01g0	Solyc01g0	0.29	-1.77
GO:00160	integral co	Cellular C	0.02	0.23	Solyc11g0	Solyc11g0	0.14	-2.88
GO:00160	integral co	Cellular C	0.02	0.23	Solyc03g0	Solyc03g0	2.72	1.44
GO:00160	integral co	Cellular C	0.02	0.23	Solyc11g0	Solyc11g0	6.34	2.66
GO:00160	integral co	Cellular C	0.02	0.23	Solyc12g0	Solyc12g0	0.35	-1.51
GO:00160	integral co	Cellular C	0.02	0.23	Solyc05g0	Solyc05g0	0.46	-1.12
GO:00160	integral co	Cellular C	0.02	0.23	Solyc10g0	Solyc10g0	0.49	-1.02
GO:00160	integral co	Cellular C	0.02	0.23	Solyc04g0	Solyc04g0	0.19	-2.40
GO:00160	integral co	Cellular C	0.02	0.23	Solyc04g0	Solyc04g0	0.41	-1.29
GO:00160	integral co	Cellular C	0.02	0.23	Solyc06g0	Solyc06g0	0.40	-1.31
GO:00160	integral co	Cellular C	0.02	0.23	Solyc08g0	Solyc08g0	0.47	-1.09
GO:00160	integral co	Cellular C	0.02	0.23	Solyc11g0	Solyc11g0	0.47	-1.08
GO:00160	integral co	Cellular C	0.02	0.23	Solyc01g0	Solyc01g0	0.43	-1.21
GO:00160	integral co	Cellular C	0.02	0.23	Solyc02g0	Solyc02g0	0.06	-4.14
GO:00160	integral co	Cellular C	0.02	0.23	Solyc09g0	Solyc09g0	0.39	-1.35
GO:00160	integral co	Cellular C	0.02	0.23	Solyc06g0	Solyc06g0	0.41	-1.28
GO:00160	integral co	Cellular C	0.02	0.23	Solyc11g0	Solyc11g0	0.46	-1.11
GO:00160	integral co	Cellular C	0.02	0.23	Solyc02g0	Solyc02g0	0.34	-1.57
GO:00160	integral co	Cellular C	0.02	0.23	Solyc02g0	Solyc02g0	0.43	-1.23
GO:00160	integral co	Cellular C	0.02	0.23	Solyc01g0	Solyc01g0	0.44	-1.18
GO:00160	integral co	Cellular C	0.02	0.23	Solyc08g0	Solyc08g0	0.36	-1.48
GO:00160	integral co	Cellular C	0.02	0.23	Solyc02g0	Solyc02g0	0.12	-3.00
GO:00160	integral co	Cellular C	0.02	0.23	Solyc02g0	Solyc02g0	0.35	-1.49
GO:00160	integral co	Cellular C	0.02	0.23	Solyc07g0	Solyc07g0	0.25	-1.97
GO:00160	integral co	Cellular C	0.02	0.23	Solyc03g0	Solyc03g0	2.00	1.00
GO:00160	integral co	Cellular C	0.02	0.23	Solyc03g0	Solyc03g0	0.44	-1.17
GO:00160	integral co	Cellular C	0.02	0.23	Solyc09g0	Solyc09g0	0.13	-2.95
GO:00160	integral co	Cellular C	0.02	0.23	Solyc04g0	Solyc04g0	0.17	-2.52
GO:00160	integral co	Cellular C	0.02	0.23	Solyc12g0	Solyc12g0	0.48	-1.05
GO:00160	integral co	Cellular C	0.02	0.23	Solyc03g0	Solyc03g0	0.28	-1.83
GO:00160	integral co	Cellular C	0.02	0.23	Solyc11g0	Solyc11g0	5992.64	12.55
GO:00160	integral co	Cellular C	0.02	0.23	Solyc01g1	Solyc01g1	0.22	-2.16
GO:00160	integral co	Cellular C	0.02	0.23	Solyc04g0	Solyc04g0	0.48	-1.07
GO:00160	integral co	Cellular C	0.02	0.23	Solyc02g0	Solyc02g0	2.89	1.53
GO:00160	integral co	Cellular C	0.02	0.23	Solyc02g0	Solyc02g0	40.96	5.36
GO:00160	integral co	Cellular C	0.02	0.23	Solyc06g0	Solyc06g0	0.47	-1.09
GO:00160	integral co	Cellular C	0.02	0.23	Solyc01g0	Solyc01g0	0.50	-1.01
GO:00160	integral co	Cellular C	0.02	0.23	Solyc02g0	Solyc02g0	0.37	-1.44
GO:00160	integral co	Cellular C	0.02	0.23	Solyc06g0	Solyc06g0	0.38	-1.40
GO:00160	integral co	Cellular C	0.02	0.23	Solyc02g0	Solyc02g0	0.36	-1.49
GO:00160	integral co	Cellular C	0.02	0.23	Solyc11g0	Solyc11g0	0.48	-1.05
GO:00160	integral co	Cellular C	0.02	0.23	Solyc12g0	Solyc12g0	2112.44	11.04
GO:00160	integral co	Cellular C	0.02	0.23	Solyc02g0	Solyc02g0	0.23	-2.14
GO:00160	integral co	Cellular C	0.02	0.23	Solyc06g0	Solyc06g0	0.07	-3.75
GO:00160	integral co	Cellular C	0.02	0.23	Solyc01g0	Solyc01g0	0.32	-1.64
GO:00160	integral co	Cellular C	0.02	0.23	Solyc11g0	Solyc11g0	2.71	1.44
GO:00160	integral co	Cellular C	0.02	0.23	Solyc01g0	Solyc01g0	2.66	1.41
GO:00160	integral co	Cellular C	0.02	0.23	Solyc08g0	Solyc08g0	0.00	-11.90
GO:00160	integral co	Cellular C	0.02	0.23	Solyc02g0	Solyc02g0	0.08	-3.65
GO:00160	integral co	Cellular C	0.02	0.23	Solyc03g1	Solyc03g1	0.03	-5.30

GO:00160	integral co	Cellular C	0.02	0.23	Solyc02g0	Solyc02g0	0.27	-1.90
GO:00160	integral co	Cellular C	0.02	0.23	Solyc05g0	Solyc05g0	0.01	-6.96
GO:00160	integral co	Cellular C	0.02	0.23	Solyc04g0	Solyc04g0	0.30	-1.74
GO:00160	integral co	Cellular C	0.02	0.23	Solyc03g1	Solyc03g1	0.40	-1.31
GO:00160	integral co	Cellular C	0.02	0.23	Solyc02g0	Solyc02g0	0.19	-2.42
GO:00160	integral co	Cellular C	0.02	0.23	Solyc04g0	Solyc04g0	0.16	-2.68
GO:00160	integral co	Cellular C	0.02	0.23	Solyc07g0	Solyc07g0	0.46	-1.11
GO:00160	integral co	Cellular C	0.02	0.23	Solyc09g0	Solyc09g0	8.52	3.09
GO:00160	integral co	Cellular C	0.02	0.23	Solyc08g0	Solyc08g0	0.22	-2.16
GO:00160	integral co	Cellular C	0.02	0.23	Solyc08g0	Solyc08g0	0.12	-3.09
GO:00160	integral co	Cellular C	0.02	0.23	Solyc08g0	Solyc08g0	0.26	-1.95
GO:00160	integral co	Cellular C	0.02	0.23	Solyc01g0	Solyc01g0	0.11	-3.14
GO:00160	integral co	Cellular C	0.02	0.23	Solyc08g0	Solyc08g0	0.06	-4.18
GO:00160	integral co	Cellular C	0.02	0.23	Solyc03g0	Solyc03g0	0.10	-3.33
GO:00160	integral co	Cellular C	0.02	0.23	Solyc11g0	Solyc11g0	0.11	-3.17
GO:00160	integral co	Cellular C	0.02	0.23	Solyc03g1	Solyc03g1	0.45	-1.16
GO:00160	integral co	Cellular C	0.02	0.23	Solyc08g0	Solyc08g0	0.29	-1.80
GO:00160	integral co	Cellular C	0.02	0.23	Solyc03g1	Solyc03g1	0.47	-1.08
GO:00160	integral co	Cellular C	0.02	0.23	Solyc03g0	Solyc03g0	0.45	-1.15
GO:00160	integral co	Cellular C	0.02	0.23	Solyc01g0	Solyc01g0	778.53	9.60
GO:00160	integral co	Cellular C	0.02	0.23	Solyc07g0	Solyc07g0	0.50	-1.01
GO:00160	integral co	Cellular C	0.02	0.23	Solyc06g0	Solyc06g0	0.26	-1.94
GO:00160	integral co	Cellular C	0.02	0.23	Solyc05g0	Solyc05g0	0.00	-11.15
GO:00160	integral co	Cellular C	0.02	0.23	Solyc02g0	Solyc02g0	0.16	-2.63
GO:00160	integral co	Cellular C	0.02	0.23	Solyc12g0	Solyc12g0	0.44	-1.17
GO:00160	integral co	Cellular C	0.02	0.23	Solyc08g0	Solyc08g0	0.37	-1.42
GO:00160	integral co	Cellular C	0.02	0.23	Solyc06g0	Solyc06g0	0.31	-1.70
GO:00160	integral co	Cellular C	0.02	0.23	Solyc02g0	Solyc02g0	0.06	-3.96
GO:00160	integral co	Cellular C	0.02	0.23	Solyc07g0	Solyc07g0	0.00	-10.38
GO:00160	integral co	Cellular C	0.02	0.23	Solyc11g0	Solyc11g0	2.48	1.31
GO:00160	integral co	Cellular C	0.02	0.23	Solyc03g1	Solyc03g1	0.48	-1.05
GO:00160	integral co	Cellular C	0.02	0.23	Solyc02g0	Solyc02g0	0.31	-1.67
GO:00160	integral co	Cellular C	0.02	0.23	Solyc01g1	Solyc01g1	2.95	1.56
GO:00160	integral co	Cellular C	0.02	0.23	Solyc02g0	Solyc02g0	0.45	-1.15
GO:00160	integral co	Cellular C	0.02	0.23	Solyc04g0	Solyc04g0	0.00	-10.62
GO:00160	integral co	Cellular C	0.02	0.23	Solyc02g0	Solyc02g0	0.33	-1.61
GO:00160	integral co	Cellular C	0.02	0.23	Solyc06g0	Solyc06g0	0.31	-1.71
GO:00160	integral co	Cellular C	0.02	0.23	Solyc10g0	Solyc10g0	0.49	-1.03
GO:00160	integral co	Cellular C	0.02	0.23	Solyc10g0	Solyc10g0	2.27	1.18
GO:00160	integral co	Cellular C	0.02	0.23	Solyc08g0	Solyc08g0	0.40	-1.32
GO:00160	integral co	Cellular C	0.02	0.23	Solyc04g0	Solyc04g0	2.01	1.01
GO:00160	integral co	Cellular C	0.02	0.23	Solyc09g0	Solyc09g0	0.48	-1.06
GO:00160	integral co	Cellular C	0.02	0.23	Solyc02g0	Solyc02g0	0.45	-1.16
GO:00160	integral co	Cellular C	0.02	0.23	Solyc03g1	Solyc03g1	0.48	-1.06
GO:00160	integral co	Cellular C	0.02	0.23	Solyc05g0	Solyc05g0	0.40	-1.34
GO:00160	integral co	Cellular C	0.02	0.23	Solyc03g0	Solyc03g0	0.00	-10.59
GO:00160	integral co	Cellular C	0.02	0.23	Solyc11g0	Solyc11g0	2.39	1.25
GO:00160	integral co	Cellular C	0.02	0.23	Solyc12g0	Solyc12g0	0.48	-1.07
GO:00160	integral co	Cellular C	0.02	0.23	Solyc12g0	Solyc12g0	0.45	-1.16
GO:00160	integral co	Cellular C	0.02	0.23	Solyc07g0	Solyc07g0	0.44	-1.18
GO:00160	integral co	Cellular C	0.02	0.23	Solyc11g0	Solyc11g0	0.16	-2.61

GO:00160	integral co	Cellular C	0.02	0.23	Solyc12g0	Solyc12g0	0.30	-1.76
GO:00160	integral co	Cellular C	0.02	0.23	Solyc02g0	Solyc02g0	0.46	-1.12
GO:00160	integral co	Cellular C	0.02	0.23	Solyc06g0	Solyc06g0	0.37	-1.45
GO:00160	integral co	Cellular C	0.02	0.23	Solyc03g1	Solyc03g1	0.10	-3.33
GO:00160	integral co	Cellular C	0.02	0.23	Solyc07g0	Solyc07g0	0.40	-1.31
GO:00160	integral co	Cellular C	0.02	0.23	Solyc10g0	Solyc10g0	14.83	3.89
GO:00160	integral co	Cellular C	0.02	0.23	Solyc01g0	Solyc01g0	2.13	1.09
GO:00160	integral co	Cellular C	0.02	0.23	Solyc04g0	Solyc04g0	0.44	-1.19
GO:00160	integral co	Cellular C	0.02	0.23	Solyc12g0	Solyc12g0	1405.23	10.46
GO:00160	integral co	Cellular C	0.02	0.23	Solyc01g1	CAP10A	0.35	-1.51
GO:00160	integral co	Cellular C	0.02	0.23	Solyc10g0	Solyc10g0	0.00	-11.12
GO:00160	integral co	Cellular C	0.02	0.23	Solyc06g0	Solyc06g0	0.00	-12.74
GO:00160	integral co	Cellular C	0.02	0.23	Solyc06g0	Solyc06g0	0.00	-10.57
GO:00160	integral co	Cellular C	0.02	0.23	Solyc01g0	Solyc01g0	0.12	-3.07
GO:00160	integral co	Cellular C	0.02	0.23	Solyc10g0	Solyc10g0	0.48	-1.05
GO:00160	integral co	Cellular C	0.02	0.23	Solyc01g0	Solyc01g0	2.28	1.19
GO:00160	integral co	Cellular C	0.02	0.23	Solyc01g1	Solyc01g1	0.29	-1.76
GO:00160	integral co	Cellular C	0.02	0.23	Solyc08g0	Solyc08g0	0.18	-2.51
GO:00160	integral co	Cellular C	0.02	0.23	Solyc04g0	Solyc04g0	0.45	-1.14
GO:00160	integral co	Cellular C	0.02	0.23	Solyc01g0	Solyc01g0	3.95	1.98
GO:00160	integral co	Cellular C	0.02	0.23	Solyc02g0	Solyc02g0	0.17	-2.55
GO:00160	integral co	Cellular C	0.02	0.23	Solyc09g0	Solyc09g0	0.08	-3.73
GO:00160	integral co	Cellular C	0.02	0.23	Solyc06g0	Solyc06g0	0.26	-1.94
GO:00160	integral co	Cellular C	0.02	0.23	Solyc12g0	Solyc12g0	0.00	-9.93
GO:00160	integral co	Cellular C	0.02	0.23	Solyc03g0	Solyc03g0	0.21	-2.28
GO:00160	integral co	Cellular C	0.02	0.23	Solyc05g0	Solyc05g0	0.46	-1.12
GO:00160	integral co	Cellular C	0.02	0.23	Solyc06g0	Solyc06g0	0.04	-4.72
GO:00160	integral co	Cellular C	0.02	0.23	Solyc10g0	Solyc10g0	0.38	-1.38
GO:00160	integral co	Cellular C	0.02	0.23	Solyc02g0	Solyc02g0	313.46	8.29
GO:00160	integral co	Cellular C	0.02	0.23	Solyc08g0	Solyc08g0	0.32	-1.65
GO:00160	integral co	Cellular C	0.02	0.23	Solyc11g0	Solyc11g0	0.24	-2.07
GO:00160	integral co	Cellular C	0.02	0.23	Solyc08g0	Solyc08g0	0.38	-1.38
GO:00160	integral co	Cellular C	0.02	0.23	Solyc06g0	Solyc06g0	2.48	1.31
GO:00160	integral co	Cellular C	0.02	0.23	Solyc03g0	Solyc03g0	27.60	4.79
GO:00160	integral co	Cellular C	0.02	0.23	Solyc08g0	Solyc08g0	0.00	-9.52
GO:00160	integral co	Cellular C	0.02	0.23	Solyc02g0	Solyc02g0	4.24	2.09
GO:00160	integral co	Cellular C	0.02	0.23	Solyc06g0	Solyc06g0	0.19	-2.40
GO:00160	integral co	Cellular C	0.02	0.23	Solyc09g0	Solyc09g0	0.49	-1.04
GO:00160	integral co	Cellular C	0.02	0.23	Solyc09g0	Solyc09g0	460.47	8.85
GO:00160	integral co	Cellular C	0.02	0.23	Solyc12g0	Solyc12g0	0.12	-3.02
GO:00160	integral co	Cellular C	0.02	0.23	Solyc01g1	Solyc01g1	0.40	-1.31
GO:00160	integral co	Cellular C	0.02	0.23	Solyc04g0	Solyc04g0	0.00	-8.72
GO:00160	integral co	Cellular C	0.02	0.23	Solyc11g0	Solyc11g0	2.70	1.43
GO:00160	integral co	Cellular C	0.02	0.23	Solyc06g0	Solyc06g0	0.41	-1.27
GO:00160	integral co	Cellular C	0.02	0.23	Solyc02g0	Solyc02g0	0.43	-1.20
GO:00160	integral co	Cellular C	0.02	0.23	Solyc07g0	Solyc07g0	0.00	-9.97
GO:00160	integral co	Cellular C	0.02	0.23	Solyc04g0	Solyc04g0	0.48	-1.06
GO:00160	integral co	Cellular C	0.02	0.23	Solyc08g0	Solyc08g0	0.00	-9.01
GO:00160	integral co	Cellular C	0.02	0.23	Solyc01g0	Solyc01g0	0.19	-2.40
GO:00160	integral co	Cellular C	0.02	0.23	Solyc01g0	Solyc01g0	0.45	-1.14
GO:00160	integral co	Cellular C	0.02	0.23	Solyc06g0	Solyc06g0	0.08	-3.56

GO:00160	integral co	Cellular Co	0.02	0.23	Solyc08g0	Solyc08g0	3.30	1.72
GO:00160	integral co	Cellular Co	0.02	0.23	Solyc11g0	Solyc11g0	4.59	2.20
GO:00160	integral co	Cellular Co	0.02	0.23	Solyc05g0	Solyc05g0	0.46	-1.13
GO:00160	lipid catab	Biological	0.02	0.23	Solyc03g0	Solyc03g0	2.46	1.30
GO:00160	lipid catab	Biological	0.02	0.23	Solyc03g0	Solyc03g0	2.03	1.02
GO:00160	lipid catab	Biological	0.02	0.23	Solyc08g0	Solyc08g0	0.47	-1.07
GO:00160	lipid catab	Biological	0.02	0.23	Solyc06g0	Solyc06g0	2.69	1.43
GO:00160	lipid catab	Biological	0.02	0.23	Solyc09g0	Solyc09g0	0.47	-1.09
GO:00099	auxin pola	Biological	0.02	0.23	Solyc05g0	Solyc05g0	0.18	-2.51
GO:00099	auxin pola	Biological	0.02	0.23	Solyc11g0	Solyc11g0	0.47	-1.08
GO:00099	auxin pola	Biological	0.02	0.23	Solyc03g1	Solyc03g1	0.48	-1.05
GO:00081	ferrous iro	Molecular	0.02	0.23	Solyc03g0	Solyc03g0	0.15	-2.71
GO:00081	ferrous iro	Molecular	0.02	0.23	Solyc04g0	Solyc04g0	0.30	-1.74
GO:00081	ferrous iro	Molecular	0.02	0.23	Solyc07g0	Solyc07g0	2.89	1.53
GO:00464	phosphatid	Biological	0.02	0.23	Solyc08g0	Solyc08g0	0.47	-1.07
GO:00464	phosphatid	Biological	0.02	0.23	Solyc02g0	Solyc02g0	7.41	2.89
GO:00464	phosphatid	Biological	0.02	0.23	Solyc04g0	Solyc04g0	0.43	-1.23
GO:00095	pollen dev	Biological	0.02	0.23	Solyc07g0	NCED1	0.30	-1.72
GO:00095	pollen dev	Biological	0.02	0.23	Solyc06g0	Solyc06g0	0.40	-1.33
GO:00095	pollen dev	Biological	0.02	0.23	Solyc05g0	Solyc05g0	2.55	1.35
GO:00095	pollen dev	Biological	0.02	0.23	Solyc11g0	Solyc11g0	2.39	1.25
GO:00104	carotenoid	Molecular	0.03	0.24	Solyc07g0	NCED1	0.30	-1.72
GO:00104	carotenoid	Molecular	0.03	0.24	Solyc05g0	Solyc05g0	0.31	-1.69
GO:00161	carotene ca	Biological	0.03	0.24	Solyc07g0	NCED1	0.30	-1.72
GO:00161	carotene ca	Biological	0.03	0.24	Solyc05g0	Solyc05g0	0.31	-1.69
GO:00333	mitotic DN	Biological	0.03	0.24	Solyc06g0	Solyc06g0	2.52	1.33
GO:00333	mitotic DN	Biological	0.03	0.24	Solyc03g0	Solyc03g0	2.59	1.37
GO:00336	receptor se	Molecular	0.03	0.24	Solyc08g0	Solyc08g0	0.39	-1.34
GO:00336	receptor se	Molecular	0.03	0.24	Solyc09g0	Solyc09g0	0.49	-1.04
GO:01200	proton exp	Biological	0.03	0.24	Solyc12g0	Solyc12g0	2112.44	11.04
GO:01200	proton exp	Biological	0.03	0.24	Solyc08g0	Solyc08g0	0.37	-1.42
GO:00047	transmemb	Molecular	0.03	0.24	Solyc02g0	Solyc02g0	0.47	-1.10
GO:00047	transmemb	Molecular	0.03	0.24	Solyc01g1	Solyc01g1	3.45	1.79
GO:00047	transmemb	Molecular	0.03	0.24	Solyc04g0	Solyc04g0	0.21	-2.26
GO:00047	transmemb	Molecular	0.03	0.24	Solyc02g0	Solyc02g0	0.16	-2.63
GO:00047	transmemb	Molecular	0.03	0.24	Solyc05g0	Solyc05g0	0.47	-1.10
GO:00047	transmemb	Molecular	0.03	0.24	Solyc02g0	Solyc02g0	0.50	-1.01
GO:00097	abscisic ac	Biological	0.03	0.24	Solyc09g0	Solyc09g0	0.07	-3.81
GO:00097	abscisic ac	Biological	0.03	0.24	Solyc07g0	NCED1	0.30	-1.72
GO:00097	abscisic ac	Biological	0.03	0.24	Solyc09g0	Solyc09g0	0.37	-1.43
GO:00097	abscisic ac	Biological	0.03	0.24	Solyc06g0	Solyc06g0	0.41	-1.28
GO:00097	abscisic ac	Biological	0.03	0.24	Solyc01g0	Solyc01g0	0.00	-12.11
GO:00097	abscisic ac	Biological	0.03	0.24	Solyc01g0	Solyc01g0	2.66	1.41
GO:00097	abscisic ac	Biological	0.03	0.24	Solyc02g0	Solyc02g0	0.49	-1.03
GO:00097	abscisic ac	Biological	0.03	0.24	Solyc06g0	Solyc06g0	0.39	-1.36
GO:00095	plasmodes	Cellular Co	0.03	0.25	Solyc03g0	RBCS-2A	0.34	-1.58
GO:00095	plasmodes	Cellular Co	0.03	0.25	Solyc04g0	Solyc04g0	0.48	-1.06
GO:00095	plasmodes	Cellular Co	0.03	0.25	Solyc02g0	Solyc02g0	0.47	-1.10
GO:00095	plasmodes	Cellular Co	0.03	0.25	Solyc01g1	Solyc01g1	3.45	1.79
GO:00095	plasmodes	Cellular Co	0.03	0.25	Solyc03g1	Solyc03g1	0.39	-1.35
GO:00095	plasmodes	Cellular Co	0.03	0.25	Solyc06g0	Solyc06g0	0.39	-1.35

GO:00095	plasmodes	Cellular C	0.03	0.25	Solyc11g0	Solyc11g0	0.48	-1.06
GO:00095	plasmodes	Cellular C	0.03	0.25	Solyc03g0	Solyc03g0	3.70	1.89
GO:00095	plasmodes	Cellular C	0.03	0.25	Solyc08g0	Solyc08g0	0.39	-1.34
GO:00095	plasmodes	Cellular C	0.03	0.25	Solyc06g0	Solyc06g0	0.40	-1.31
GO:00095	plasmodes	Cellular C	0.03	0.25	Solyc09g0	Solyc09g0	0.39	-1.35
GO:00095	plasmodes	Cellular C	0.03	0.25	Solyc11g0	Solyc11g0	0.46	-1.11
GO:00095	plasmodes	Cellular C	0.03	0.25	Solyc02g0	Solyc02g0	6.42	2.68
GO:00095	plasmodes	Cellular C	0.03	0.25	Solyc07g0	Solyc07g0	0.25	-1.97
GO:00095	plasmodes	Cellular C	0.03	0.25	Solyc01g0	Solyc01g0	0.18	-2.51
GO:00095	plasmodes	Cellular C	0.03	0.25	Solyc09g0	Solyc09g0	2.43	1.28
GO:00095	plasmodes	Cellular C	0.03	0.25	Solyc12g0	Solyc12g0	0.09	-3.42
GO:00095	plasmodes	Cellular C	0.03	0.25	Solyc01g1	Solyc01g1	2.95	1.56
GO:00095	plasmodes	Cellular C	0.03	0.25	Solyc02g0	Solyc02g0	0.45	-1.15
GO:00095	plasmodes	Cellular C	0.03	0.25	Solyc06g0	Solyc06g0	0.41	-1.27
GO:00060	chitin catal	Biological	0.03	0.25	Solyc10g0	Solyc10g0	0.06	-4.12
GO:00060	chitin catal	Biological	0.03	0.25	Solyc02g0	CHI3	0.24	-2.08
GO:00060	chitin catal	Biological	0.03	0.25	Solyc10g0	CHI9	0.27	-1.88
GO:00060	chitin catal	Biological	0.03	0.25	Solyc07g0	Solyc07g0	0.45	-1.16
GO:00458	positive re	Biological	0.03	0.25	Solyc04g0	Solyc04g0	0.47	-1.09
GO:00458	positive re	Biological	0.03	0.25	Solyc01g0	Solyc01g0	0.34	-1.55
GO:00458	positive re	Biological	0.03	0.25	Solyc04g0	Solyc04g0	0.46	-1.11
GO:00458	positive re	Biological	0.03	0.25	Solyc05g0	Solyc05g0	0.29	-1.78
GO:00458	positive re	Biological	0.03	0.25	Solyc10g0	Solyc10g0	0.32	-1.64
GO:00458	positive re	Biological	0.03	0.25	Solyc02g0	Solyc02g0	0.15	-2.75
GO:00458	positive re	Biological	0.03	0.25	Solyc03g1	Solyc03g1	0.44	-1.19
GO:00458	positive re	Biological	0.03	0.25	Solyc05g0	Solyc05g0	0.42	-1.26
GO:00458	positive re	Biological	0.03	0.25	Solyc10g0	Solyc10g0	0.15	-2.72
GO:00458	positive re	Biological	0.03	0.25	Solyc06g0	Solyc06g0	0.42	-1.25
GO:00458	positive re	Biological	0.03	0.25	Solyc07g0	Solyc07g0	0.18	-2.49
GO:00053	inorganic p	Molecular	0.03	0.25	Solyc09g0	Solyc09g0	0.14	-2.81
GO:00053	inorganic p	Molecular	0.03	0.25	Solyc09g0	Solyc09g0	0.18	-2.47
GO:00053	inorganic p	Molecular	0.03	0.25	Solyc03g1	Solyc03g1	0.47	-1.08
GO:00102	auxin hom	Biological	0.03	0.25	Solyc05g0	Solyc05g0	0.18	-2.51
GO:00102	auxin hom	Biological	0.03	0.25	Solyc03g1	Solyc03g1	0.48	-1.05
GO:00102	auxin hom	Biological	0.03	0.25	Solyc02g0	Solyc02g0	5.03	2.33
GO:00039	acid phosp	Molecular	0.03	0.25	Solyc03g0	Solyc03g0	0.15	-2.71
GO:00039	acid phosp	Molecular	0.03	0.25	Solyc07g0	Solyc07g0	0.08	-3.60
GO:00039	acid phosp	Molecular	0.03	0.25	Solyc04g0	Solyc04g0	0.24	-2.08
GO:00039	acid phosp	Molecular	0.03	0.25	Solyc04g0	Solyc04g0	0.30	-1.74
GO:00039	acid phosp	Molecular	0.03	0.25	Solyc07g0	Solyc07g0	0.18	-2.47
GO:00325	developme	Biological	0.03	0.25	Solyc11g0	Solyc11g0	0.19	-2.40
GO:00325	developme	Biological	0.03	0.25	Solyc06g0	Solyc06g0	0.40	-1.33
GO:00325	developme	Biological	0.03	0.25	Solyc08g0	Solyc08g0	0.48	-1.04
GO:00325	developme	Biological	0.03	0.25	Solyc07g0	Solyc07g0	0.24	-2.07
GO:00325	developme	Biological	0.03	0.25	Solyc10g0	Solyc10g0	2.49	1.31
GO:00325	developme	Biological	0.03	0.25	Solyc01g0	Solyc01g0	0.48	-1.06
GO:00045	nuclease a	Molecular	0.03	0.25	Solyc04g0	Solyc04g0	0.42	-1.26
GO:00045	nuclease a	Molecular	0.03	0.25	Solyc06g0	Solyc06g0	4.39	2.14
GO:00045	nuclease a	Molecular	0.03	0.25	Solyc09g0	Solyc09g0	0.04	-4.53
GO:00045	nuclease a	Molecular	0.03	0.25	Solyc11g0	Solyc11g0	3.60	1.85
GO:00045	nuclease a	Molecular	0.03	0.25	Solyc05g0	Solyc05g0	0.48	-1.07

GO:00045	nuclease a	Molecular	0.03	0.25	Solyc07g0	Solyc07g0	0.47	-1.08
GO:00045	nuclease a	Molecular	0.03	0.25	Solyc02g0	Solyc02g0	2.19	1.13
GO:00046	peroxidase	Molecular	0.03	0.25	Solyc12g0	Solyc12g0	0.28	-1.84
GO:00046	peroxidase	Molecular	0.03	0.25	Solyc04g0	Solyc04g0	0.31	-1.69
GO:00046	peroxidase	Molecular	0.03	0.25	Solyc02g0	Solyc02g0	2.89	1.53
GO:00046	peroxidase	Molecular	0.03	0.25	Solyc02g0	Solyc02g0	0.00	-11.78
GO:00046	peroxidase	Molecular	0.03	0.25	Solyc09g0	Solyc09g0	2.43	1.28
GO:00046	peroxidase	Molecular	0.03	0.25	Solyc01g1	Solyc01g1	2.95	1.56
GO:00046	peroxidase	Molecular	0.03	0.25	Solyc03g0	Solyc03g0	0.39	-1.34
GO:00046	peroxidase	Molecular	0.03	0.25	Solyc03g0	Solyc03g0	2.14	1.10
GO:00046	peroxidase	Molecular	0.03	0.25	Solyc06g0	Solyc06g0	9.98	3.32
GO:00046	peroxidase	Molecular	0.03	0.25	Solyc02g0	Solyc02g0	2.29	1.19
GO:00046	peroxidase	Molecular	0.03	0.25	Solyc02g0	Solyc02g0	0.10	-3.34
GO:00046	peroxidase	Molecular	0.03	0.25	Solyc04g0	Solyc04g0	0.49	-1.04
GO:00046	peroxidase	Molecular	0.03	0.25	Solyc01g1	Solyc01g1	0.00	-9.56
GO:00515	iron-sulfur	Molecular	0.03	0.25	Solyc03g0	Solyc03g0	2.69	1.43
GO:00515	iron-sulfur	Molecular	0.03	0.25	Solyc01g1	Solyc01g1	0.46	-1.13
GO:00515	iron-sulfur	Molecular	0.03	0.25	Solyc04g0	Solyc04g0	0.41	-1.27
GO:00515	iron-sulfur	Molecular	0.03	0.25	Solyc11g0	Solyc11g0	9.10	3.19
GO:00515	iron-sulfur	Molecular	0.03	0.25	Solyc01g0	Solyc01g0	0.38	-1.39
GO:00515	iron-sulfur	Molecular	0.03	0.25	Solyc07g0	Solyc07g0	2.89	1.53
GO:00515	iron-sulfur	Molecular	0.03	0.25	Solyc10g0	Solyc10g0	0.24	-2.04
GO:00515	iron-sulfur	Molecular	0.03	0.25	Solyc03g0	Solyc03g0	0.34	-1.57
GO:00228	transmemb	Molecular	0.03	0.25	Solyc03g0	Solyc03g0	0.35	-1.50
GO:00228	transmemb	Molecular	0.03	0.25	Solyc09g0	Solyc09g0	0.14	-2.81
GO:00228	transmemb	Molecular	0.03	0.25	Solyc08g0	Solyc08g0	2.26	1.18
GO:00228	transmemb	Molecular	0.03	0.25	Solyc07g0	Solyc07g0	0.03	-5.23
GO:00228	transmemb	Molecular	0.03	0.25	Solyc12g0	Solyc12g0	2.45	1.29
GO:00228	transmemb	Molecular	0.03	0.25	Solyc01g1	Solyc01g1	0.19	-2.38
GO:00228	transmemb	Molecular	0.03	0.25	Solyc09g0	Solyc09g0	0.18	-2.47
GO:00228	transmemb	Molecular	0.03	0.25	Solyc10g0	Solyc10g0	2.72	1.45
GO:00228	transmemb	Molecular	0.03	0.25	Solyc11g0	Solyc11g0	0.36	-1.49
GO:00228	transmemb	Molecular	0.03	0.25	Solyc05g0	Solyc05g0	0.09	-3.40
GO:00228	transmemb	Molecular	0.03	0.25	Solyc08g0	Solyc08g0	0.47	-1.09
GO:00228	transmemb	Molecular	0.03	0.25	Solyc01g0	Solyc01g0	0.44	-1.18
GO:00228	transmemb	Molecular	0.03	0.25	Solyc09g0	Solyc09g0	0.13	-2.95
GO:00228	transmemb	Molecular	0.03	0.25	Solyc04g0	Solyc04g0	0.48	-1.07
GO:00228	transmemb	Molecular	0.03	0.25	Solyc05g0	Solyc05g0	0.41	-1.29
GO:00228	transmemb	Molecular	0.03	0.25	Solyc03g1	Solyc03g1	0.40	-1.31
GO:00228	transmemb	Molecular	0.03	0.25	Solyc01g0	Solyc01g0	2099.54	11.04
GO:00228	transmemb	Molecular	0.03	0.25	Solyc05g0	Solyc05g0	0.32	-1.63
GO:00228	transmemb	Molecular	0.03	0.25	Solyc05g0	Solyc05g0	0.33	-1.60
GO:00228	transmemb	Molecular	0.03	0.25	Solyc05g0	Solyc05g0	0.40	-1.34
GO:00228	transmemb	Molecular	0.03	0.25	Solyc11g0	Solyc11g0	0.16	-2.61
GO:00228	transmemb	Molecular	0.03	0.25	Solyc06g0	Solyc06g0	0.37	-1.45
GO:00228	transmemb	Molecular	0.03	0.25	Solyc06g0	Solyc06g0	0.04	-4.72
GO:00228	transmemb	Molecular	0.03	0.25	Solyc10g0	Solyc10g0	0.38	-1.38
GO:00228	transmemb	Molecular	0.03	0.25	Solyc09g0	Solyc09g0	0.42	-1.26
GO:00228	transmemb	Molecular	0.03	0.25	Solyc05g0	Solyc05g0	0.46	-1.13
GO:00199	lipid storag	Biological	0.04	0.25	Solyc04g0	Solyc04g0	0.46	-1.13
GO:00199	lipid storag	Biological	0.04	0.25	Solyc03g1	Solyc03g1	0.44	-1.18

GO:00199	lipid stora	Biological	0.04	0.25	Solyc06g0	Solyc06g0	0.38	-1.40
GO:00022	response t	Biological	0.04	0.25	Solyc07g0	Solyc07g0	0.17	-2.54
GO:00022	response t	Biological	0.04	0.25	Solyc02g0	Solyc02g0	0.23	-2.13
GO:00022	response t	Biological	0.04	0.25	Solyc02g0	Solyc02g0	0.02	-5.62
GO:00022	response t	Biological	0.04	0.25	Solyc07g0	Solyc07g0	0.16	-2.62
GO:00096	photosyste	Cellular C	0.04	0.25	Solyc02g0	PSBO	0.48	-1.07
GO:00096	photosyste	Cellular C	0.04	0.25	Solyc02g0	Solyc02g0	0.35	-1.53
GO:00096	photosyste	Cellular C	0.04	0.25	Solyc07g0	Solyc07g0	0.41	-1.28
GO:00096	photosyste	Cellular C	0.04	0.25	Solyc10g0	Solyc10g0	0.00	-11.65
GO:00430	negative re	Biological	0.04	0.25	Solyc09g0	Solyc09g0	0.37	-1.43
GO:00430	negative re	Biological	0.04	0.25	Solyc03g1	Solyc03g1	0.37	-1.42
GO:00430	negative re	Biological	0.04	0.25	Solyc06g0	Solyc06g0	0.41	-1.28
GO:00430	negative re	Biological	0.04	0.25	Solyc01g0	Solyc01g0	2.08	1.05
GO:00430	negative re	Biological	0.04	0.25	Solyc07g0	Solyc07g0	2.42	1.28
GO:00095	thylakoid	Cellular C	0.04	0.25	Solyc02g0	PSBO	0.48	-1.07
GO:00095	thylakoid	Cellular C	0.04	0.25	Solyc02g0	Solyc02g0	0.35	-1.53
GO:00095	thylakoid	Cellular C	0.04	0.25	Solyc03g0	Solyc03g0	0.18	-2.46
GO:00095	thylakoid	Cellular C	0.04	0.25	Solyc10g0	Solyc10g0	0.43	-1.23
GO:00095	thylakoid	Cellular C	0.04	0.25	Solyc06g0	psaD	0.43	-1.22
GO:00095	thylakoid	Cellular C	0.04	0.25	Solyc05g0	Solyc05g0	0.15	-2.75
GO:00095	thylakoid	Cellular C	0.04	0.25	Solyc10g0	Solyc10g0	0.49	-1.02
GO:00095	thylakoid	Cellular C	0.04	0.25	Solyc03g0	Solyc03g0	0.45	-1.15
GO:00095	thylakoid	Cellular C	0.04	0.25	Solyc11g0	Solyc11g0	0.33	-1.60
GO:00095	thylakoid	Cellular C	0.04	0.25	Solyc01g1	CAP10A	0.35	-1.51
GO:00095	thylakoid	Cellular C	0.04	0.25	Solyc05g0	Solyc05g0	0.46	-1.12
GO:00095	thylakoid	Cellular C	0.04	0.25	Solyc03g0	Solyc03g0	0.34	-1.57
GO:00301	pyridoxal	Molecular	0.04	0.25	Solyc05g0	Solyc05g0	0.37	-1.44
GO:00301	pyridoxal	Molecular	0.04	0.25	Solyc01g0	Solyc01g0	0.47	-1.10
GO:00301	pyridoxal	Molecular	0.04	0.25	Solyc08g0	Solyc08g0	0.11	-3.24
GO:00301	pyridoxal	Molecular	0.04	0.25	Solyc03g0	Solyc03g0	0.00	-11.21
GO:00301	pyridoxal	Molecular	0.04	0.25	Solyc01g1	Solyc01g1	2.42	1.28
GO:00301	pyridoxal	Molecular	0.04	0.25	Solyc08g0	Solyc08g0	0.00	-10.99
GO:00301	pyridoxal	Molecular	0.04	0.25	Solyc08g0	Solyc08g0	0.05	-4.43
GO:00301	pyridoxal	Molecular	0.04	0.25	Solyc09g0	TD2	4.53	2.18
GO:00301	pyridoxal	Molecular	0.04	0.25	Solyc08g0	Solyc08g0	799.01	9.64
GO:00301	pyridoxal	Molecular	0.04	0.25	Solyc08g0	Solyc08g0	0.07	-3.82
GO:00301	pyridoxal	Molecular	0.04	0.25	Solyc08g0	Solyc08g0	0.17	-2.53
GO:00301	pyridoxal	Molecular	0.04	0.25	Solyc06g0	Solyc06g0	0.00	-9.59
GO:00098	1-aminocy	Molecular	0.04	0.25	Solyc02g0	Solyc02g0	0.23	-2.13
GO:00098	1-aminocy	Molecular	0.04	0.25	Solyc07g0	Solyc07g0	0.16	-2.62
GO:00713	cellular res	Biological	0.04	0.25	Solyc02g0	Solyc02g0	0.23	-2.13
GO:00713	cellular res	Biological	0.04	0.25	Solyc07g0	Solyc07g0	0.16	-2.62
GO:00069	immune re	Biological	0.04	0.25	Solyc09g0	Solyc09g0	0.48	-1.06
GO:00069	immune re	Biological	0.04	0.25	Solyc01g0	Solyc01g0	0.37	-1.43
GO:00429	tripeptide t	Molecular	0.04	0.25	Solyc09g0	Solyc09g0	0.13	-2.95
GO:00429	tripeptide t	Molecular	0.04	0.25	Solyc06g0	Solyc06g0	0.04	-4.72
GO:00429	dipeptide t	Biological	0.04	0.25	Solyc09g0	Solyc09g0	0.13	-2.95
GO:00429	dipeptide t	Biological	0.04	0.25	Solyc06g0	Solyc06g0	0.04	-4.72
GO:00719	dipeptide t	Molecular	0.04	0.25	Solyc09g0	Solyc09g0	0.13	-2.95
GO:00719	dipeptide t	Molecular	0.04	0.25	Solyc06g0	Solyc06g0	0.04	-4.72
GO:00070	mitotic spi	Biological	0.04	0.25	Solyc07g0	Solyc07g0	2.61	1.38

GO:00070	mitotic spi	Biological	0.04	0.25	Solyc05g0	Solyc05g0	2.25	1.17
GO:00053	nucleoside	Molecular	0.04	0.25	Solyc07g0	Solyc07g0	0.00	-10.38
GO:00053	nucleoside	Molecular	0.04	0.25	Solyc02g0	Solyc02g0	313.46	8.29
GO:19016	nucleoside	Biological	0.04	0.25	Solyc07g0	Solyc07g0	0.00	-10.38
GO:19016	nucleoside	Biological	0.04	0.25	Solyc02g0	Solyc02g0	313.46	8.29
GO:00056	chromosor	Cellular C	0.04	0.25	Solyc01g0	Solyc01g0	3.68	1.88
GO:00056	chromosor	Cellular C	0.04	0.25	Solyc05g0	Solyc05g0	2.04	1.03
GO:00056	chromosor	Cellular C	0.04	0.25	Solyc04g0	Solyc04g0	2.55	1.35
GO:00056	chromosor	Cellular C	0.04	0.25	Solyc11g0	Solyc11g0	2.07	1.05
GO:00056	chromosor	Cellular C	0.04	0.25	Solyc06g0	Solyc06g0	2.05	1.04
GO:00056	chromosor	Cellular C	0.04	0.25	Solyc05g0	Solyc05g0	2.73	1.45
GO:00166	oxidoreduc	Molecular	0.04	0.25	Solyc01g0	Solyc01g0	0.41	-1.30
GO:00166	oxidoreduc	Molecular	0.04	0.25	Solyc03g1	Solyc03g1	0.46	-1.12
GO:00166	oxidoreduc	Molecular	0.04	0.25	Solyc02g0	Solyc02g0	0.46	-1.12
GO:00166	oxidoreduc	Molecular	0.04	0.25	Solyc02g0	Solyc02g0	2.79	1.48
GO:00166	oxidoreduc	Molecular	0.04	0.25	Solyc12g0	Solyc12g0	0.13	-2.93
GO:00167	2-oxogluta	Molecular	0.04	0.25	Solyc07g0	Solyc07g0	0.17	-2.54
GO:00167	2-oxogluta	Molecular	0.04	0.25	Solyc02g0	Solyc02g0	0.23	-2.13
GO:00167	2-oxogluta	Molecular	0.04	0.25	Solyc02g0	Solyc02g0	0.02	-5.62
GO:00167	2-oxogluta	Molecular	0.04	0.25	Solyc07g0	Solyc07g0	0.16	-2.62
GO:00453	aspartyl es	Molecular	0.05	0.25	Solyc03g1	Solyc03g1	0.37	-1.42
GO:00453	aspartyl es	Molecular	0.05	0.25	Solyc09g0	Solyc09g0	4.75	2.25
GO:00453	aspartyl es	Molecular	0.05	0.25	Solyc03g0	Solyc03g0	0.28	-1.83
GO:00453	aspartyl es	Molecular	0.05	0.25	Solyc01g0	Solyc01g0	4.89	2.29
GO:00453	aspartyl es	Molecular	0.05	0.25	Solyc07g0	Solyc07g0	2.40	1.27
GO:00432	intracellul	Cellular C	0.05	0.25	Solyc10g0	Solyc10g0	0.37	-1.43
GO:00432	intracellul	Cellular C	0.05	0.25	Solyc12g0	Solyc12g0	2.94	1.56
GO:00432	intracellul	Cellular C	0.05	0.25	Solyc11g0	Solyc11g0	0.33	-1.60
GO:00432	intracellul	Cellular C	0.05	0.25	Solyc03g0	Solyc03g0	0.25	-2.02
GO:00432	intracellul	Cellular C	0.05	0.25	Solyc02g0	Solyc02g0	0.11	-3.14
GO:00432	intracellul	Cellular C	0.05	0.25	Solyc09g0	Solyc09g0	6.02	2.59
GO:00432	intracellul	Cellular C	0.05	0.25	Solyc01g1	Solyc01g1	0.39	-1.36
GO:00432	intracellul	Cellular C	0.05	0.25	Solyc07g0	Solyc07g0	638.37	9.32
GO:00432	intracellul	Cellular C	0.05	0.25	Solyc08g0	Solyc08g0	0.32	-1.65
GO:00432	intracellul	Cellular C	0.05	0.25	Solyc05g0	Solyc05g0	0.00	-11.22
GO:00432	intracellul	Cellular C	0.05	0.25	Solyc10g0	Solyc10g0	3.52	1.82
GO:00095	plastid	Cellular C	0.05	0.25	Solyc09g0	Solyc09g0	2.11	1.07
GO:00095	plastid	Cellular C	0.05	0.25	Solyc11g0	AOS2	0.12	-3.01
GO:00095	plastid	Cellular C	0.05	0.25	Solyc07g0	NCED1	0.30	-1.72
GO:00095	plastid	Cellular C	0.05	0.25	Solyc02g0	PSBO	0.48	-1.07
GO:00095	plastid	Cellular C	0.05	0.25	Solyc03g0	Solyc03g0	0.18	-2.46
GO:00095	plastid	Cellular C	0.05	0.25	Solyc03g0	RBCS-2A	0.34	-1.58
GO:00095	plastid	Cellular C	0.05	0.25	Solyc10g0	Solyc10g0	0.43	-1.23
GO:00095	plastid	Cellular C	0.05	0.25	Solyc03g0	Solyc03g0	0.46	-1.11
GO:00095	plastid	Cellular C	0.05	0.25	Solyc06g0	psaD	0.43	-1.22
GO:00095	plastid	Cellular C	0.05	0.25	Solyc11g0	Solyc11g0	9.10	3.19
GO:00095	plastid	Cellular C	0.05	0.25	Solyc05g0	Solyc05g0	0.15	-2.75
GO:00095	plastid	Cellular C	0.05	0.25	Solyc01g0	Solyc01g0	0.38	-1.39
GO:00095	plastid	Cellular C	0.05	0.25	Solyc10g0	Solyc10g0	0.49	-1.02
GO:00095	plastid	Cellular C	0.05	0.25	Solyc03g0	Solyc03g0	0.45	-1.15
GO:00095	plastid	Cellular C	0.05	0.25	Solyc11g0	Solyc11g0	0.33	-1.60

GO:00095	plastid	Cellular Co	0.05	0.25	Solyc09g0	TD2	4.53	2.18
GO:00095	plastid	Cellular Co	0.05	0.25	Solyc01g1	CAP10A	0.35	-1.51
GO:00095	plastid	Cellular Co	0.05	0.25	Solyc11g0	Solyc11g0	0.24	-2.07
GO:00095	plastid	Cellular Co	0.05	0.25	Solyc05g0	Solyc05g0	0.00	-11.22
GO:00095	plastid	Cellular Co	0.05	0.25	Solyc03g0	Solyc03g0	0.34	-1.57
GO:00167	oxidoreduc	Molecular	0.05	0.25	Solyc01g0	Solyc01g0	4.36	2.12
GO:00167	oxidoreduc	Molecular	0.05	0.25	Solyc01g0	Solyc01g0	2.27	1.18
GO:00167	oxidoreduc	Molecular	0.05	0.25	Solyc07g0	NCED1	0.30	-1.72
GO:00167	oxidoreduc	Molecular	0.05	0.25	Solyc05g0	Solyc05g0	0.31	-1.69
GO:00167	oxidoreduc	Molecular	0.05	0.25	Solyc12g0	Solyc12g0	10.12	3.34
GO:00167	oxidoreduc	Molecular	0.05	0.25	Solyc01g0	Solyc01g0	3.58	1.84
GO:00719	FAD bindi	Molecular	0.05	0.25	Solyc02g0	Solyc02g0	0.36	-1.46
GO:00719	FAD bindi	Molecular	0.05	0.25	Solyc12g0	Solyc12g0	0.32	-1.65
GO:00719	FAD bindi	Molecular	0.05	0.25	Solyc11g0	Solyc11g0	9.10	3.19
GO:00719	FAD bindi	Molecular	0.05	0.25	Solyc04g0	Solyc04g0	0.09	-3.42
GO:00719	FAD bindi	Molecular	0.05	0.25	Solyc01g0	Solyc01g0	0.38	-1.39
GO:00719	FAD bindi	Molecular	0.05	0.25	Solyc02g0	Solyc02g0	5.84	2.55
GO:00719	FAD bindi	Molecular	0.05	0.25	Solyc09g0	Solyc09g0	3.02	1.59
GO:00094	response tc	Biological	0.05	0.25	Solyc02g0	Solyc02g0	0.44	-1.18
GO:00094	response tc	Biological	0.05	0.25	Solyc01g1	Solyc01g1	0.47	-1.09
GO:00168	intramolec	Molecular	0.05	0.25	Solyc05g0	Solyc05g0	0.05	-4.31
GO:00168	intramolec	Molecular	0.05	0.25	Solyc02g0	Solyc02g0	1436.80	10.49
GO:00096	jasmonic a	Biological	0.05	0.25	Solyc11g0	AOS2	0.12	-3.01
GO:00096	jasmonic a	Biological	0.05	0.25	Solyc07g0	OPR3	0.47	-1.10
GO:00429	glucan end	Molecular	0.05	0.25	Solyc04g0	Solyc04g0	0.31	-1.70
GO:00429	glucan end	Molecular	0.05	0.25	Solyc01g0	Solyc01g0	0.04	-4.82
GO:00092	thiamine b	Biological	0.05	0.25	Solyc07g0	Solyc07g0	0.47	-1.09
GO:00092	thiamine b	Biological	0.05	0.25	Solyc11g0	Solyc11g0	0.38	-1.39
GO:00081	ferric iron	Molecular	0.05	0.25	Solyc03g0	Solyc03g0	0.15	-2.71
GO:00081	ferric iron	Molecular	0.05	0.25	Solyc04g0	Solyc04g0	0.30	-1.74
GO:00347	regulation	Biological	0.05	0.25	Solyc01g1	Solyc01g1	0.43	-1.23
GO:00347	regulation	Biological	0.05	0.25	Solyc12g0	Solyc12g0	0.48	-1.07
GO:00061	ferredoxin	Biological	0.05	0.25	Solyc11g0	Solyc11g0	9.10	3.19
GO:00061	ferredoxin	Biological	0.05	0.25	Solyc01g0	Solyc01g0	0.38	-1.39
GO:00157	nitrate tran	Biological	0.05	0.25	Solyc02g0	Solyc02g0	0.31	-1.67
GO:00157	nitrate tran	Biological	0.05	0.25	Solyc06g0	Solyc06g0	0.37	-1.45
GO:00040	allantoinas	Molecular	0.05	0.25	Solyc02g0	Solyc02g0	2.65	1.40
GO:00454	naringenin	Molecular	0.05	0.25	Solyc02g0	Solyc02g0	0.02	-5.62
GO:00472	indole-3-ac	Molecular	0.05	0.25	Solyc09g0	Solyc09g0	0.07	-3.81
GO:00801	negative re	Biological	0.05	0.25	Solyc09g0	Solyc09g0	0.07	-3.81
GO:00160	glutamate s	Molecular	0.05	0.25	Solyc03g0	Solyc03g0	2.69	1.43
GO:00454	flavonol sy	Molecular	0.05	0.25	Solyc11g0	Solyc11g0	0.11	-3.13
GO:00702	protein pol	Biological	0.05	0.25	Solyc11g0	Solyc11g0	0.43	-1.23
GO:19904	protein AD	Molecular	0.05	0.25	Solyc11g0	Solyc11g0	0.43	-1.23
GO:00101	pollen tube	Biological	0.05	0.25	Solyc01g1	Solyc01g1	0.41	-1.30
GO:00360	mediator c	Molecular	0.05	0.25	Solyc01g1	Solyc01g1	0.41	-1.30
GO:00528	thiazole bi	Biological	0.05	0.25	Solyc07g0	Solyc07g0	0.47	-1.09
GO:19018	beta-carote	Biological	0.05	0.25	Solyc07g0	NCED1	0.30	-1.72
GO:00335	cellular res	Biological	0.05	0.25	Solyc08g0	Solyc08g0	0.49	-1.04
GO:00067	porphyrin-	Biological	0.05	0.25	Solyc09g0	Solyc09g0	0.44	-1.17
GO:00364	microtubul	Cellular Co	0.05	0.25	Solyc04g0	Solyc04g0	0.48	-1.06

GO:00515	response to	Biological	0.05	0.25	Solyc04g0	Solyc04g0	0.48	-1.06
GO:00726	protein loc	Biological	0.05	0.25	Solyc04g0	Solyc04g0	0.48	-1.06
GO:00340	maintenan	Biological	0.05	0.25	Solyc01g0	Solyc01g0	2.42	1.27
GO:00096	abscisic ac	Biological	0.05	0.25	Solyc08g0	CYP707A	0.26	-1.93
GO:00041	dihydroorc	Molecular	0.05	0.25	Solyc01g0	Solyc01g0	0.29	-1.78
GO:00503	tocopherol	Molecular	0.05	0.25	Solyc08g0	Solyc08g0	2.10	1.07
GO:00088	dTDP-4-de	Molecular	0.05	0.25	Solyc08g0	Solyc08g0	0.34	-1.55
GO:00088	dTDP-4-de	Molecular	0.05	0.25	Solyc08g0	Solyc08g0	0.34	-1.55
GO:00102	UDP-rham	Biological	0.05	0.25	Solyc08g0	Solyc08g0	0.34	-1.55
GO:00193	dTDP-rhar	Biological	0.05	0.25	Solyc08g0	Solyc08g0	0.34	-1.55
GO:00088	cyanate hy	Molecular	0.05	0.25	Solyc09g0	Solyc09g0	0.39	-1.37
GO:00094	cyanate me	Biological	0.05	0.25	Solyc09g0	Solyc09g0	0.39	-1.37
GO:00333	chlorophyl	Biological	0.05	0.25	Solyc09g0	Solyc09g0	0.40	-1.33
GO:00904	7-hydroxy	Molecular	0.05	0.25	Solyc09g0	Solyc09g0	0.40	-1.33
GO:00167	trans-cinna	Molecular	0.05	0.25	Solyc06g0	Solyc06g0	0.40	-1.33
GO:00428	monopolar	Biological	0.05	0.25	Solyc02g0	Solyc02g0	0.36	-1.49
GO:00484	petal devel	Biological	0.05	0.25	Solyc02g0	Solyc02g0	0.36	-1.49
GO:00101	stomatal co	Biological	0.05	0.25	Solyc08g0	Solyc08g0	0.39	-1.34
GO:00101	transpiratio	Biological	0.05	0.25	Solyc08g0	Solyc08g0	0.39	-1.34
GO:00301	regulation	Biological	0.05	0.25	Solyc08g0	Solyc08g0	0.39	-1.34
GO:00482	inflorescer	Biological	0.05	0.25	Solyc08g0	Solyc08g0	0.39	-1.34
GO:19054	regulation	Biological	0.05	0.25	Solyc08g0	Solyc08g0	0.39	-1.34
GO:19046	negative re	Biological	0.05	0.25	Solyc10g0	Solyc10g0	2.92	1.54
GO:00084	selenium b	Molecular	0.05	0.25	Solyc09g0	Solyc09g0	0.44	-1.20
GO:00103	auxin influ	Molecular	0.05	0.25	Solyc11g0	Solyc11g0	0.47	-1.08
GO:00102	response to	Biological	0.05	0.25	Solyc03g0	Solyc03g0	2.59	1.37
GO:00098	auxin bios	Biological	0.05	0.25	Solyc06g0	Solyc06g0	2.01	1.01
GO:00513	meiotic ch	Biological	0.05	0.25	Solyc01g1	Solyc01g1	2.11	1.08
GO:00461	thymidine	Biological	0.05	0.25	Solyc11g0	Solyc11g0	2.55	1.35
GO:00002	C-5 sterol	Molecular	0.05	0.25	Solyc02g0	Solyc02g0	0.36	-1.49
GO:00904	pollen tube	Cellular Co	0.05	0.25	Solyc01g1	Solyc01g1	0.44	-1.20
GO:00062	dGTP cata	Biological	0.05	0.25	Solyc03g0	Solyc03g0	0.08	-3.70
GO:00355	8-oxo-7,8-	Molecular	0.05	0.25	Solyc03g0	Solyc03g0	0.08	-3.70
GO:00312	DNA repli	Cellular Co	0.05	0.25	Solyc09g0	Solyc09g0	2.25	1.17
GO:00319	obsolete re	Biological	0.05	0.25	Solyc09g0	Solyc09g0	2.25	1.17
GO:19000	positive re	Biological	0.05	0.25	Solyc09g0	Solyc09g0	2.25	1.17
GO:19029	mitotic DN	Biological	0.05	0.25	Solyc09g0	Solyc09g0	2.25	1.17
GO:00018	selenium c	Biological	0.05	0.25	Solyc01g1	Solyc01g1	2.42	1.28
GO:00307	methionine	Molecular	0.05	0.25	Solyc01g1	Solyc01g1	2.42	1.28
GO:00512	protein pol	Biological	0.05	0.25	Solyc06g0	Solyc06g0	0.08	-3.58
GO:00907	specificatio	Biological	0.05	0.25	Solyc06g0	Solyc06g0	0.08	-3.58
GO:00047	serine-pyr	Molecular	0.05	0.25	Solyc12g0	Solyc12g0	0.30	-1.74
GO:00084	alanine-gly	Molecular	0.05	0.25	Solyc12g0	Solyc12g0	0.30	-1.74
GO:00192	glycine bio	Biological	0.05	0.25	Solyc12g0	Solyc12g0	0.30	-1.74
GO:00090	homoserin	Biological	0.05	0.25	Solyc06g0	Solyc06g0	0.30	-1.75
GO:19004	negative re	Biological	0.05	0.25	Solyc09g0	Solyc09g0	0.37	-1.42
GO:00450	protein im	Biological	0.05	0.25	Solyc02g0	Solyc02g0	0.45	-1.16
GO:00800	signal reco	Cellular Co	0.05	0.25	Solyc02g0	Solyc02g0	0.45	-1.16
GO:00043	glycerol ki	Molecular	0.05	0.25	Solyc03g1	Solyc03g1	0.34	-1.57
GO:00066	triglycerid	Biological	0.05	0.25	Solyc03g1	Solyc03g1	0.34	-1.57
GO:00461	glycerol-3-	Biological	0.05	0.25	Solyc03g1	Solyc03g1	0.34	-1.57

GO:00313	positive re	Biological	0.05	0.25	Solyc09g0	TD2	4.53	2.18
GO:00052	inward rec	Molecular	0.05	0.25	Solyc12g0	Solyc12g0	0.48	-1.07
GO:00041	cystathioni	Molecular	0.05	0.25	Solyc08g0	Solyc08g0	0.07	-3.82
GO:00193	cysteine bi	Biological	0.05	0.25	Solyc08g0	Solyc08g0	0.07	-3.82
GO:00320	negative re	Biological	0.05	0.25	Solyc10g0	Solyc10g0	0.48	-1.05
GO:00037	telomerase	Molecular	0.05	0.25	Solyc01g0	Solyc01g0	6.71	2.75
GO:00726	mitochond	Biological	0.05	0.25	Solyc10g0	Solyc10g0	476.24	8.90
GO:00098	auxin catal	Biological	0.05	0.25	Solyc02g0	Solyc02g0	5.03	2.33
GO:00503	indole-3-ac	Molecular	0.05	0.25	Solyc02g0	Solyc02g0	5.03	2.33
GO:00166	oxidoreduc	Molecular	0.05	0.25	Solyc02g0	Solyc02g0	0.14	-2.87
GO:00166	oxidoreduc	Molecular	0.05	0.25	Solyc02g0	Solyc02g0	2.02	1.01
GO:00166	oxidoreduc	Molecular	0.05	0.25	Solyc08g0	Solyc08g0	0.21	-2.27
GO:00166	oxidoreduc	Molecular	0.05	0.25	Solyc11g0	Solyc11g0	0.16	-2.69
GO:00166	oxidoreduc	Molecular	0.05	0.25	Solyc01g0	Solyc01g0	0.26	-1.92
GO:00166	oxidoreduc	Molecular	0.05	0.25	Solyc12g0	Solyc12g0	0.34	-1.56
GO:00166	oxidoreduc	Molecular	0.05	0.25	Solyc08g0	Solyc08g0	0.34	-1.55
GO:00166	oxidoreduc	Molecular	0.05	0.25	Solyc03g0	Solyc03g0	0.46	-1.11
GO:00166	oxidoreduc	Molecular	0.05	0.25	Solyc08g0	Solyc08g0	0.35	-1.51
GO:00069	response to	Biological	0.05	0.25	Solyc12g0	Solyc12g0	0.28	-1.84
GO:00069	response to	Biological	0.05	0.25	Solyc04g0	Solyc04g0	0.31	-1.69
GO:00069	response to	Biological	0.05	0.25	Solyc02g0	Solyc02g0	2.89	1.53
GO:00069	response to	Biological	0.05	0.25	Solyc02g0	Solyc02g0	0.00	-11.78
GO:00069	response to	Biological	0.05	0.25	Solyc09g0	Solyc09g0	2.43	1.28
GO:00069	response to	Biological	0.05	0.25	Solyc01g1	Solyc01g1	2.95	1.56
GO:00069	response to	Biological	0.05	0.25	Solyc03g0	Solyc03g0	0.39	-1.34
GO:00069	response to	Biological	0.05	0.25	Solyc03g0	Solyc03g0	2.14	1.10
GO:00069	response to	Biological	0.05	0.25	Solyc06g0	Solyc06g0	9.98	3.32
GO:00069	response to	Biological	0.05	0.25	Solyc02g0	Solyc02g0	2.29	1.19
GO:00069	response to	Biological	0.05	0.25	Solyc02g0	Solyc02g0	0.10	-3.34
GO:00069	response to	Biological	0.05	0.25	Solyc09g0	Solyc09g0	0.24	-2.04
GO:00069	response to	Biological	0.05	0.25	Solyc04g0	Solyc04g0	0.49	-1.04
GO:00069	response to	Biological	0.05	0.25	Solyc01g1	Solyc01g1	0.00	-9.56
GO:00314	L-ascorbic	Molecular	0.05	0.25	Solyc07g0	Solyc07g0	0.17	-2.54
GO:00314	L-ascorbic	Molecular	0.05	0.25	Solyc02g0	Solyc02g0	0.23	-2.13
GO:00314	L-ascorbic	Molecular	0.05	0.25	Solyc02g0	Solyc02g0	0.02	-5.62
GO:00314	L-ascorbic	Molecular	0.05	0.25	Solyc07g0	Solyc07g0	0.16	-2.62
GO:00314	L-ascorbic	Molecular	0.05	0.25	Solyc02g0	Solyc02g0	2133.31	11.06
GO:00048	serine-type	Molecular	0.05	0.25	Solyc08g0	Solyc08g0	0.19	-2.36
GO:00048	serine-type	Molecular	0.05	0.25	Solyc09g0	Solyc09g0	0.00	-15.42
GO:00048	serine-type	Molecular	0.05	0.25	Solyc03g0	Solyc03g0	2.00	1.00
GO:00048	serine-type	Molecular	0.05	0.25	Solyc11g0	Solyc11g0	0.46	-1.12
GO:00048	serine-type	Molecular	0.05	0.25	Solyc09g0	Solyc09g0	33.35	5.06
GO:00096	plant-type	Biological	0.05	0.25	Solyc04g0	Solyc04g0	0.38	-1.41
GO:00096	plant-type	Biological	0.05	0.25	Solyc04g0	Solyc04g0	0.13	-2.97
GO:00096	plant-type	Biological	0.05	0.25	Solyc02g0	Solyc02g0	2.19	1.13
GO:00096	plant-type	Biological	0.05	0.25	Solyc08g0	Solyc08g0	0.39	-1.34
GO:00096	plant-type	Biological	0.05	0.25	Solyc10g0	Solyc10g0	2.57	1.36
GO:00551	obsolete o	Biological	0.06	0.26	Solyc11g0	AOS2	0.12	-3.01
GO:00551	obsolete o	Biological	0.06	0.26	Solyc11g0	Solyc11g0	0.16	-2.69
GO:00551	obsolete o	Biological	0.06	0.26	Solyc08g0	CYP707A	0.26	-1.93
GO:00551	obsolete o	Biological	0.06	0.26	Solyc05g0	Solyc05g0	0.21	-2.26

GO:00551	obsolete o:	Biological	0.06	0.26	Solyc02g0	Solyc02g0	0.36	-1.49
GO:00551	obsolete o:	Biological	0.06	0.26	Solyc01g0	Solyc01g0	0.29	-1.77
GO:00041	cysteine-ty	Molecular	0.06	0.26	Solyc11g0	Solyc11g0	0.46	-1.12
GO:00041	cysteine-ty	Molecular	0.06	0.26	Solyc08g0	Solyc08g0	0.45	-1.14
GO:00041	cysteine-ty	Molecular	0.06	0.26	Solyc01g1	Solyc01g1	2.11	1.08
GO:00041	cysteine-ty	Molecular	0.06	0.26	Solyc12g0	Solyc12g0	0.35	-1.50
GO:00041	cysteine-ty	Molecular	0.06	0.26	Solyc03g0	Solyc03g0	0.48	-1.07
GO:00041	cysteine-ty	Molecular	0.06	0.26	Solyc08g0	Solyc08g0	311.75	8.28
GO:00167	acyltransfe	Molecular	0.06	0.26	Solyc09g0	Solyc09g0	0.01	-7.13
GO:00167	acyltransfe	Molecular	0.06	0.26	Solyc05g0	Solyc05g0	0.01	-6.95
GO:00167	acyltransfe	Molecular	0.06	0.26	Solyc02g0	Solyc02g0	3.03	1.60
GO:00167	acyltransfe	Molecular	0.06	0.26	Solyc06g0	Solyc06g0	2.65	1.41
GO:00167	acyltransfe	Molecular	0.06	0.26	Solyc08g0	Solyc08g0	5.08	2.34
GO:00167	acyltransfe	Molecular	0.06	0.26	Solyc04g0	Solyc04g0	0.49	-1.04
GO:00167	acyltransfe	Molecular	0.06	0.26	Solyc05g0	Solyc05g0	2.98	1.58
GO:00167	acyltransfe	Molecular	0.06	0.26	Solyc01g0	Solyc01g0	2.13	1.09
GO:00167	acyltransfe	Molecular	0.06	0.26	Solyc09g0	Solyc09g0	8.52	3.09
GO:00167	acyltransfe	Molecular	0.06	0.26	Solyc09g0	Solyc09g0	0.06	-4.02
GO:00167	acyltransfe	Molecular	0.06	0.26	Solyc09g0	Solyc09g0	0.00	-11.98
GO:00167	acyltransfe	Molecular	0.06	0.26	Solyc01g1	Solyc01g1	0.00	-9.69
GO:00167	acyltransfe	Molecular	0.06	0.26	Solyc07g0	Solyc07g0	2.22	1.15
GO:00098	coumarin b	Biological	0.06	0.27	Solyc07g0	Solyc07g0	0.17	-2.54
GO:00098	coumarin b	Biological	0.06	0.27	Solyc02g0	Solyc02g0	0.23	-2.13
GO:00098	coumarin b	Biological	0.06	0.27	Solyc02g0	Solyc02g0	0.02	-5.62
GO:00098	coumarin b	Biological	0.06	0.27	Solyc07g0	Solyc07g0	0.16	-2.62
GO:00161	sterol meta	Biological	0.06	0.27	Solyc11g0	AOS2	0.12	-3.01
GO:00161	sterol meta	Biological	0.06	0.27	Solyc08g0	CYP707A:	0.26	-1.93
GO:00161	sterol meta	Biological	0.06	0.27	Solyc02g0	Solyc02g0	0.36	-1.49
GO:00161	sterol meta	Biological	0.06	0.27	Solyc01g0	Solyc01g0	0.29	-1.77
GO:00071	cell surfac	Biological	0.06	0.27	Solyc02g0	Solyc02g0	0.41	-1.28
GO:00071	cell surfac	Biological	0.06	0.27	Solyc09g0	Solyc09g0	0.44	-1.19
GO:00071	cell surfac	Biological	0.06	0.27	Solyc01g1	Solyc01g1	0.44	-1.20
GO:00071	cell surfac	Biological	0.06	0.27	Solyc12g0	Solyc12g0	0.18	-2.45
GO:00071	cell surfac	Biological	0.06	0.27	Solyc09g0	Solyc09g0	2.30	1.20
GO:00069	defense re:	Biological	0.06	0.27	Solyc11g0	AOS2	0.12	-3.01
GO:00069	defense re:	Biological	0.06	0.27	Solyc08g0	Solyc08g0	0.27	-1.87
GO:00069	defense re:	Biological	0.06	0.27	Solyc04g0	Solyc04g0	0.31	-1.70
GO:00069	defense re:	Biological	0.06	0.27	Solyc09g0	Solyc09g0	0.01	-7.14
GO:00069	defense re:	Biological	0.06	0.27	Solyc01g1	Solyc01g1	0.08	-3.71
GO:00069	defense re:	Biological	0.06	0.27	Solyc09g0	Solyc09g0	0.37	-1.43
GO:00069	defense re:	Biological	0.06	0.27	Solyc02g0	CHI3	0.24	-2.08
GO:00069	defense re:	Biological	0.06	0.27	Solyc04g0	Solyc04g0	2.92	1.55
GO:00069	defense re:	Biological	0.06	0.27	Solyc05g0	Solyc05g0	2.15	1.10
GO:00069	defense re:	Biological	0.06	0.27	Solyc09g0	Solyc09g0	0.48	-1.06
GO:00069	defense re:	Biological	0.06	0.27	Solyc01g0	Solyc01g0	0.33	-1.59
GO:00069	defense re:	Biological	0.06	0.27	Solyc01g0	Solyc01g0	0.04	-4.82
GO:00069	defense re:	Biological	0.06	0.27	Solyc07g0	EIX1	0.33	-1.59
GO:00069	defense re:	Biological	0.06	0.27	Solyc04g0	Solyc04g0	0.13	-2.91
GO:00069	defense re:	Biological	0.06	0.27	Solyc05g0	Solyc05g0	0.36	-1.49
GO:00069	defense re:	Biological	0.06	0.27	Solyc09g0	Solyc09g0	0.35	-1.51
GO:00069	defense re:	Biological	0.06	0.27	Solyc10g0	CHI9	0.27	-1.88

GO:00069	defense res	Biological	0.06	0.27	Solyc11g0	Solyc11g0	0.23	-2.13
GO:00069	defense res	Biological	0.06	0.27	Solyc06g0	Solyc06g0	0.07	-3.75
GO:00069	defense res	Biological	0.06	0.27	Solyc04g0	Solyc04g0	0.29	-1.80
GO:00069	defense res	Biological	0.06	0.27	Solyc01g0	Solyc01g0	0.37	-1.43
GO:00069	defense res	Biological	0.06	0.27	Solyc07g0	Solyc07g0	0.49	-1.04
GO:00069	defense res	Biological	0.06	0.27	Solyc05g0	Solyc05g0	0.13	-3.00
GO:00069	defense res	Biological	0.06	0.27	Solyc06g0	Solyc06g0	3.49	1.81
GO:00069	defense res	Biological	0.06	0.27	Solyc11g0	Solyc11g0	2.28	1.19
GO:00069	defense res	Biological	0.06	0.27	Solyc09g0	Solyc09g0	0.00	-9.29
GO:00069	defense res	Biological	0.06	0.27	Solyc07g0	Solyc07g0	5.85	2.55
GO:00069	defense res	Biological	0.06	0.27	Solyc04g0	Solyc04g0	607.22	9.25
GO:00069	defense res	Biological	0.06	0.27	Solyc02g0	Solyc02g0	0.42	-1.25
GO:00069	defense res	Biological	0.06	0.27	Solyc09g0	TD2	4.53	2.18
GO:00069	defense res	Biological	0.06	0.27	Solyc07g0	Solyc07g0	4.97	2.31
GO:00069	defense res	Biological	0.06	0.27	Solyc03g1	Solyc03g1	0.45	-1.14
GO:00069	defense res	Biological	0.06	0.27	Solyc02g0	PTI5	0.07	-3.77
GO:00080	N-acetyltra	Molecular	0.06	0.27	Solyc09g0	Solyc09g0	0.49	-1.02
GO:00080	N-acetyltra	Molecular	0.06	0.27	Solyc08g0	Solyc08g0	0.34	-1.54
GO:00080	N-acetyltra	Molecular	0.06	0.27	Solyc09g0	Solyc09g0	0.06	-4.02
GO:00080	N-acetyltra	Molecular	0.06	0.27	Solyc05g0	Solyc05g0	2.31	1.21
GO:00080	N-acetyltra	Molecular	0.06	0.27	Solyc09g0	Solyc09g0	0.00	-11.98
GO:00080	N-acetyltra	Molecular	0.06	0.27	Solyc08g0	Solyc08g0	3.05	1.61
GO:00168	ammonia-l	Molecular	0.06	0.27	Solyc09g0	Solyc09g0	0.08	-3.59
GO:00168	ammonia-l	Molecular	0.06	0.27	Solyc09g0	Solyc09g0	0.09	-3.50
GO:00100	positive re	Biological	0.06	0.27	Solyc02g0	Solyc02g0	0.23	-2.13
GO:00100	positive re	Biological	0.06	0.27	Solyc03g1	Solyc03g1	0.46	-1.13
GO:00717	cellular res	Biological	0.06	0.27	Solyc02g0	Solyc02g0	0.23	-2.13
GO:00717	cellular res	Biological	0.06	0.27	Solyc07g0	Solyc07g0	0.16	-2.62
GO:00517	cellular res	Biological	0.06	0.27	Solyc12g0	Solyc12g0	0.28	-1.84
GO:00517	cellular res	Biological	0.06	0.27	Solyc12g0	Solyc12g0	0.32	-1.65
GO:00103	auxin efflu	Biological	0.06	0.27	Solyc05g0	Solyc05g0	0.18	-2.51
GO:00103	auxin efflu	Biological	0.06	0.27	Solyc03g1	Solyc03g1	0.48	-1.05
GO:00103	auxin efflu	Molecular	0.06	0.27	Solyc05g0	Solyc05g0	0.18	-2.51
GO:00103	auxin efflu	Molecular	0.06	0.27	Solyc03g1	Solyc03g1	0.48	-1.05
GO:00043	glyceraldel	Molecular	0.06	0.27	Solyc02g0	Solyc02g0	0.46	-1.12
GO:00043	glyceraldel	Molecular	0.06	0.27	Solyc12g0	Solyc12g0	0.13	-2.93
GO:00039	RNA-direc	Molecular	0.06	0.27	Solyc09g0	Solyc09g0	6.02	2.59
GO:00039	RNA-direc	Molecular	0.06	0.27	Solyc01g0	Solyc01g0	6.71	2.75
GO:00100	phloem de	Biological	0.06	0.27	Solyc03g1	Solyc03g1	4.07	2.02
GO:00100	phloem de	Biological	0.06	0.27	Solyc05g0	Solyc05g0	2.79	1.48
GO:00801	intracellula	Biological	0.06	0.27	Solyc03g0	Solyc03g0	27.60	4.79
GO:00801	intracellula	Biological	0.06	0.27	Solyc12g0	Solyc12g0	0.12	-3.02
GO:00101	FMN bind	Molecular	0.07	0.28	Solyc10g0	Solyc10g0	0.03	-5.15
GO:00101	FMN bind	Molecular	0.07	0.28	Solyc03g0	Solyc03g0	2.69	1.43
GO:00101	FMN bind	Molecular	0.07	0.28	Solyc12g0	Solyc12g0	0.34	-1.57
GO:00101	FMN bind	Molecular	0.07	0.28	Solyc02g0	Solyc02g0	0.50	-1.01
GO:00101	FMN bind	Molecular	0.07	0.28	Solyc07g0	OPR3	0.47	-1.10
GO:00167	acyltransfe	Molecular	0.07	0.28	Solyc09g0	Solyc09g0	0.01	-7.13
GO:00167	acyltransfe	Molecular	0.07	0.28	Solyc05g0	Solyc05g0	0.01	-6.95
GO:00167	acyltransfe	Molecular	0.07	0.28	Solyc05g0	Solyc05g0	2.02	1.01
GO:00167	acyltransfe	Molecular	0.07	0.28	Solyc08g0	Solyc08g0	0.50	-1.01

GO:00167	acyltransfe	Molecular	0.07	0.28	Solyc11g0	Solyc11g0	6.34	2.66
GO:00167	acyltransfe	Molecular	0.07	0.28	Solyc12g0	Solyc12g0	0.43	-1.20
GO:00167	acyltransfe	Molecular	0.07	0.28	Solyc02g0	Solyc02g0	2133.31	11.06
GO:00167	acyltransfe	Molecular	0.07	0.28	Solyc01g0	Solyc01g0	7223.16	12.82
GO:00167	acyltransfe	Molecular	0.07	0.28	Solyc09g0	Solyc09g0	8.52	3.09
GO:00041	aspartic-ty	Molecular	0.07	0.29	Solyc01g1	Solyc01g1	0.45	-1.16
GO:00041	aspartic-ty	Molecular	0.07	0.29	Solyc08g0	Solyc08g0	2.25	1.17
GO:00041	aspartic-ty	Molecular	0.07	0.29	Solyc01g0	Solyc01g0	0.46	-1.12
GO:00041	aspartic-ty	Molecular	0.07	0.29	Solyc06g0	Solyc06g0	0.49	-1.04
GO:00041	aspartic-ty	Molecular	0.07	0.29	Solyc08g0	Solyc08g0	0.48	-1.06
GO:00041	aspartic-ty	Molecular	0.07	0.29	Solyc01g0	Solyc01g0	0.24	-2.04
GO:00041	aspartic-ty	Molecular	0.07	0.29	Solyc09g0	Solyc09g0	4.04	2.01
GO:00041	aspartic-ty	Molecular	0.07	0.29	Solyc02g0	Solyc02g0	0.46	-1.13
GO:00041	aspartic-ty	Molecular	0.07	0.29	Solyc03g0	Solyc03g0	2.33	1.22
GO:00041	aspartic-ty	Molecular	0.07	0.29	Solyc01g0	Solyc01g0	0.18	-2.50
GO:00168	carboxy-ly	Molecular	0.07	0.30	Solyc10g0	Solyc10g0	0.38	-1.40
GO:00168	carboxy-ly	Molecular	0.07	0.30	Solyc08g0	Solyc08g0	0.11	-3.24
GO:00168	carboxy-ly	Molecular	0.07	0.30	Solyc08g0	Solyc08g0	0.00	-10.99
GO:00168	carboxy-ly	Molecular	0.07	0.30	Solyc08g0	Solyc08g0	0.05	-4.43
GO:00168	carboxy-ly	Molecular	0.07	0.30	Solyc08g0	Solyc08g0	799.01	9.64
GO:00510	actin filam	Molecular	0.07	0.30	Solyc04g0	Solyc04g0	0.27	-1.90
GO:00510	actin filam	Molecular	0.07	0.30	Solyc06g0	Solyc06g0	0.47	-1.09
GO:00510	actin filam	Molecular	0.07	0.30	Solyc01g0	Solyc01g0	0.11	-3.14
GO:00510	actin filam	Molecular	0.07	0.30	Solyc11g0	Solyc11g0	0.04	-4.78
GO:00510	actin filam	Molecular	0.07	0.30	Solyc06g0	Solyc06g0	0.36	-1.46
GO:00510	actin filam	Molecular	0.07	0.30	Solyc12g0	Solyc12g0	0.00	-10.45
GO:00081	O-methyltı	Molecular	0.08	0.31	Solyc03g0	Solyc03g0	3.27	1.71
GO:00081	O-methyltı	Molecular	0.08	0.31	Solyc06g0	Solyc06g0	2.55	1.35
GO:00081	O-methyltı	Molecular	0.08	0.31	Solyc02g0	Solyc02g0	0.49	-1.02
GO:00081	O-methyltı	Molecular	0.08	0.31	Solyc02g0	Solyc02g0	0.24	-2.05
GO:00081	O-methyltı	Molecular	0.08	0.31	Solyc04g0	Solyc04g0	0.27	-1.87
GO:00081	O-methyltı	Molecular	0.08	0.31	Solyc03g0	Solyc03g0	0.04	-4.71
GO:00060	tricarboxyl	Biological	0.08	0.31	Solyc04g0	Solyc04g0	0.34	-1.56
GO:00060	tricarboxyl	Biological	0.08	0.31	Solyc07g0	Solyc07g0	0.49	-1.04
GO:00060	tricarboxyl	Biological	0.08	0.31	Solyc09g0	Solyc09g0	0.21	-2.26
GO:00060	tricarboxyl	Biological	0.08	0.31	Solyc04g0	Solyc04g0	0.41	-1.27
GO:00060	tricarboxyl	Biological	0.08	0.31	Solyc11g0	Solyc11g0	0.33	-1.60
GO:00097	response tc	Biological	0.08	0.31	Solyc07g0	NCED1	0.30	-1.72
GO:00097	response tc	Biological	0.08	0.31	Solyc12g0	Solyc12g0	0.40	-1.34
GO:00097	response tc	Biological	0.08	0.31	Solyc03g0	Solyc03g0	16.58	4.05
GO:00096	response tc	Biological	0.08	0.31	Solyc02g0	Solyc02g0	0.23	-2.13
GO:00096	response tc	Biological	0.08	0.31	Solyc07g0	OPR3	0.47	-1.10
GO:00102	oxygen ev	Molecular	0.08	0.31	Solyc02g0	PSBO	0.48	-1.07
GO:00102	oxygen ev	Molecular	0.08	0.31	Solyc07g0	Solyc07g0	0.41	-1.28
GO:00069	response tc	Biological	0.08	0.31	Solyc10g0	Solyc10g0	0.47	-1.09
GO:00069	response tc	Biological	0.08	0.31	Solyc03g1	Solyc03g1	0.00	-11.76
GO:00101	response tc	Biological	0.08	0.31	Solyc10g0	Solyc10g0	2.72	1.45
GO:00101	response tc	Biological	0.08	0.31	Solyc02g0	Solyc02g0	0.31	-1.67
GO:00099	leaf morph	Biological	0.08	0.31	Solyc02g0	Solyc02g0	0.36	-1.49
GO:00099	leaf morph	Biological	0.08	0.31	Solyc08g0	Solyc08g0	0.39	-1.34
GO:00168	hydrolase a	Molecular	0.08	0.31	Solyc03g0	Solyc03g0	2.30	1.20

GO:00168	hydrolase a	Molecular	0.08	0.31	Solyc10g0	Solyc10g0	2.30	1.20
GO:00085	P-type prot	Molecular	0.08	0.31	Solyc12g0	Solyc12g0	2112.44	11.04
GO:00085	P-type prot	Molecular	0.08	0.31	Solyc08g0	Solyc08g0	0.37	-1.42
GO:00457	nutrient res	Molecular	0.08	0.31	Solyc02g0	Solyc02g0	0.46	-1.12
GO:00457	nutrient res	Molecular	0.08	0.31	Solyc11g0	Solyc11g0	0.49	-1.04
GO:00457	nutrient res	Molecular	0.08	0.31	Solyc09g0	Solyc09g0	0.49	-1.04
GO:00457	nutrient res	Molecular	0.08	0.31	Solyc09g0	Solyc09g0	0.35	-1.50
GO:00168	carbon-car	Molecular	0.08	0.31	Solyc08g0	Solyc08g0	0.11	-3.24
GO:00168	carbon-car	Molecular	0.08	0.31	Solyc08g0	Solyc08g0	0.00	-10.99
GO:00168	carbon-car	Molecular	0.08	0.31	Solyc08g0	Solyc08g0	0.05	-4.43
GO:00168	carbon-car	Molecular	0.08	0.31	Solyc08g0	Solyc08g0	799.01	9.64
GO:00062	DNA repli	Biological	0.09	0.31	Solyc03g1	Solyc03g1	2.29	1.19
GO:00062	DNA repli	Biological	0.09	0.31	Solyc03g0	Solyc03g0	2.59	1.37
GO:00062	DNA repli	Biological	0.09	0.31	Solyc05g0	Solyc05g0	2.55	1.35
GO:00062	DNA repli	Biological	0.09	0.31	Solyc01g1	Solyc01g1	2.41	1.27
GO:00062	DNA repli	Biological	0.09	0.31	Solyc01g0	Solyc01g0	208.08	7.70
GO:00062	DNA repli	Biological	0.09	0.31	Solyc09g0	Solyc09g0	3.39	1.76
GO:00062	DNA repli	Biological	0.09	0.31	Solyc08g0	Solyc08g0	9.73	3.28
GO:00062	DNA repli	Biological	0.09	0.31	Solyc10g0	Solyc10g0	2.30	1.20
GO:00062	DNA repli	Biological	0.09	0.31	Solyc02g0	Solyc02g0	0.23	-2.09
GO:00516	proteolysis	Biological	0.09	0.31	Solyc11g0	Solyc11g0	0.46	-1.12
GO:00516	proteolysis	Biological	0.09	0.31	Solyc08g0	Solyc08g0	0.45	-1.14
GO:00516	proteolysis	Biological	0.09	0.31	Solyc12g0	Solyc12g0	0.43	-1.22
GO:00516	proteolysis	Biological	0.09	0.31	Solyc11g0	Solyc11g0	3.66	1.87
GO:00516	proteolysis	Biological	0.09	0.31	Solyc03g0	Solyc03g0	25578.61	14.64
GO:00516	proteolysis	Biological	0.09	0.31	Solyc08g0	Solyc08g0	311.75	8.28
GO:00098	ethylene-ac	Biological	0.09	0.31	Solyc01g0	Solyc01g0	0.35	-1.51
GO:00098	ethylene-ac	Biological	0.09	0.31	Solyc04g0	Solyc04g0	0.37	-1.43
GO:00098	ethylene-ac	Biological	0.09	0.31	Solyc02g0	PTI5	0.07	-3.77
GO:00098	ethylene-ac	Biological	0.09	0.31	Solyc03g0	Solyc03g0	0.39	-1.37
GO:00098	ethylene-ac	Biological	0.09	0.31	Solyc04g0	Solyc04g0	0.34	-1.54
GO:00098	ethylene-ac	Biological	0.09	0.31	Solyc09g0	Solyc09g0	0.49	-1.04
GO:00082	isoprenoid	Biological	0.09	0.31	Solyc02g0	Solyc02g0	2.02	1.01
GO:00082	isoprenoid	Biological	0.09	0.31	Solyc04g0	Solyc04g0	2.15	1.11
GO:00082	isoprenoid	Biological	0.09	0.31	Solyc12g0	Solyc12g0	0.43	-1.20
GO:00082	isoprenoid	Biological	0.09	0.31	Solyc11g0	Solyc11g0	0.38	-1.39
GO:00052	ion channe	Molecular	0.09	0.31	Solyc01g1	Solyc01g1	0.43	-1.23
GO:00052	ion channe	Molecular	0.09	0.31	Solyc11g0	Solyc11g0	2.48	1.31
GO:00052	ion channe	Molecular	0.09	0.31	Solyc08g0	Solyc08g0	0.40	-1.32
GO:00052	ion channe	Molecular	0.09	0.31	Solyc12g0	Solyc12g0	0.48	-1.07
GO:00300	metal ion t	Biological	0.09	0.31	Solyc02g0	Solyc02g0	0.37	-1.43
GO:00300	metal ion t	Biological	0.09	0.31	Solyc06g0	Solyc06g0	0.49	-1.03
GO:00300	metal ion t	Biological	0.09	0.31	Solyc06g0	Solyc06g0	0.18	-2.46
GO:00300	metal ion t	Biological	0.09	0.31	Solyc02g0	Solyc02g0	0.19	-2.42
GO:00300	metal ion t	Biological	0.09	0.31	Solyc10g0	Solyc10g0	0.40	-1.31
GO:00300	metal ion t	Biological	0.09	0.31	Solyc06g0	Solyc06g0	896.80	9.81
GO:00300	metal ion t	Biological	0.09	0.31	Solyc08g0	Solyc08g0	0.15	-2.74
GO:00057	vacuole	Cellular Co	0.09	0.31	Solyc03g1	Solyc03g1	2.21	1.14
GO:00057	vacuole	Cellular Co	0.09	0.31	Solyc06g0	Solyc06g0	0.37	-1.44
GO:00057	vacuole	Cellular Co	0.09	0.31	Solyc01g0	Solyc01g0	0.04	-4.82
GO:00057	vacuole	Cellular Co	0.09	0.31	Solyc03g0	Solyc03g0	2.58	1.37

GO:00057	vacuole	Cellular C	0.09	0.31	Solyc06g0	Solyc06g0	0.32	-1.65
GO:00057	vacuole	Cellular C	0.09	0.31	Solyc10g0	CHI9	0.27	-1.88
GO:00057	vacuole	Cellular C	0.09	0.31	Solyc11g0	Solyc11g0	3.66	1.87
GO:00057	vacuole	Cellular C	0.09	0.31	Solyc04g0	Solyc04g0	0.46	-1.13
GO:00096	cold acclin	Biological	0.10	0.31	Solyc02g0	Solyc02g0	0.44	-1.18
GO:00096	cold acclin	Biological	0.10	0.31	Solyc01g1	Solyc01g1	0.47	-1.09
GO:00043	fructose-bi	Molecular	0.10	0.31	Solyc02g0	Solyc02g0	0.45	-1.16
GO:00043	fructose-bi	Molecular	0.10	0.31	Solyc01g1	Solyc01g1	0.31	-1.69
GO:00517	misfolded	Molecular	0.10	0.31	Solyc10g0	Solyc10g0	0.47	-1.09
GO:00517	misfolded	Molecular	0.10	0.31	Solyc03g1	Solyc03g1	0.00	-11.76
GO:00051	structural c	Molecular	0.10	0.31	Solyc04g0	Solyc04g0	0.38	-1.41
GO:00051	structural c	Molecular	0.10	0.31	Solyc04g0	Solyc04g0	0.13	-2.97
GO:00169	mannan en	Molecular	0.10	0.31	Solyc01g0	Solyc01g0	2.53	1.34
GO:00169	mannan en	Molecular	0.10	0.31	Solyc06g0	Solyc06g0	0.18	-2.48
GO:00171	Golgi trans	Cellular C	0.10	0.31	Solyc08g0	Solyc08g0	2.03	1.02
GO:00171	Golgi trans	Cellular C	0.10	0.31	Solyc04g0	Solyc04g0	0.19	-2.40
GO:00060	cellular alc	Biological	0.10	0.31	Solyc01g0	Solyc01g0	0.41	-1.30
GO:00060	cellular alc	Biological	0.10	0.31	Solyc02g0	Solyc02g0	2.79	1.48
GO:00065	ornithine n	Biological	0.10	0.31	Solyc08g0	Solyc08g0	0.34	-1.54
GO:00065	ornithine n	Biological	0.10	0.31	Solyc08g0	Solyc08g0	3.05	1.61
GO:00515	2 iron, 2 su	Molecular	0.10	0.31	Solyc01g1	Solyc01g1	0.46	-1.13
GO:00515	2 iron, 2 su	Molecular	0.10	0.31	Solyc04g0	Solyc04g0	0.41	-1.27
GO:00515	2 iron, 2 su	Molecular	0.10	0.31	Solyc11g0	Solyc11g0	9.10	3.19
GO:00515	2 iron, 2 su	Molecular	0.10	0.31	Solyc01g0	Solyc01g0	0.38	-1.39
GO:00515	2 iron, 2 su	Molecular	0.10	0.31	Solyc07g0	Solyc07g0	2.89	1.53
GO:00161	sterol bios	Biological	0.10	0.31	Solyc02g0	Solyc02g0	2.02	1.01
GO:00161	sterol bios	Biological	0.10	0.31	Solyc12g0	Solyc12g0	0.43	-1.20
GO:00161	sterol bios	Biological	0.10	0.31	Solyc02g0	Solyc02g0	0.36	-1.49
GO:00181	peptidyl-se	Biological	0.10	0.31	Solyc11g0	Solyc11g0	0.49	-1.03
GO:00181	peptidyl-se	Biological	0.10	0.31	Solyc08g0	Solyc08g0	2.48	1.31
GO:00181	peptidyl-se	Biological	0.10	0.31	Solyc01g0	Solyc01g0	0.00	-12.11
GO:00181	peptidyl-se	Biological	0.10	0.31	Solyc12g0	Solyc12g0	2.48	1.31
GO:00181	peptidyl-se	Biological	0.10	0.31	Solyc01g0	Solyc01g0	2.66	1.41
GO:00181	peptidyl-se	Biological	0.10	0.31	Solyc02g0	Solyc02g0	0.49	-1.03
GO:00002	allantoin c	Biological	0.10	0.31	Solyc02g0	Solyc02g0	2.65	1.40
GO:00102	abscisic ac	Molecular	0.10	0.31	Solyc09g0	Solyc09g0	0.07	-3.81
GO:00098	pollination	Biological	0.10	0.31	Solyc07g0	NCED1	0.30	-1.72
GO:19011	lycopene b	Biological	0.10	0.31	Solyc07g0	NCED1	0.30	-1.72
GO:00040	alcohol del	Molecular	0.10	0.31	Solyc12g0	Solyc12g0	2.13	1.09
GO:00038	3-isopropy	Molecular	0.10	0.31	Solyc09g0	Solyc09g0	0.01	-6.18
GO:00162	4-coumara	Molecular	0.10	0.31	Solyc03g0	Solyc03g0	0.11	-3.22
GO:00047	dihydrolip	Molecular	0.10	0.31	Solyc05g0	Solyc05g0	2.02	1.01
GO:00315	nucleotide-	Cellular C	0.10	0.31	Solyc06g0	Solyc06g0	0.44	-1.19
GO:00421	cellular res	Biological	0.10	0.31	Solyc06g0	Solyc06g0	0.44	-1.19
GO:00098	cytoplasmi	Cellular C	0.10	0.31	Solyc04g0	Solyc04g0	0.48	-1.06
GO:00102	pollen wal	Biological	0.10	0.31	Solyc04g0	Solyc04g0	0.48	-1.06
GO:00550	cortical mi	Cellular C	0.10	0.31	Solyc04g0	Solyc04g0	0.48	-1.06
GO:00705	regulation	Biological	0.10	0.31	Solyc04g0	Solyc04g0	0.48	-1.06
GO:00700	oligopeptic	Molecular	0.10	0.31	Solyc04g0	Solyc04g0	0.18	-2.44
GO:00102	(+)-abscisi	Molecular	0.10	0.31	Solyc08g0	CYP707A	0.26	-1.93
GO:00463	abscisic ac	Biological	0.10	0.31	Solyc08g0	CYP707A	0.26	-1.93

GO:00092	pyrimidine	Biological	0.10	0.31	Solyc01g0	Solyc01g0	0.29	-1.78
GO:00046	proline del	Molecular	0.10	0.31	Solyc02g0	Solyc02g0	0.36	-1.46
GO:00065	proline cat	Biological	0.10	0.31	Solyc02g0	Solyc02g0	0.36	-1.46
GO:00101	proline cat	Biological	0.10	0.31	Solyc02g0	Solyc02g0	0.36	-1.46
GO:00465	malate deh	Molecular	0.10	0.31	Solyc03g0	Solyc03g0	0.46	-1.11
GO:00329	circadian r	Biological	0.10	0.31	Solyc12g0	Solyc12g0	0.32	-1.65
GO:00431	entrainmer	Biological	0.10	0.31	Solyc12g0	Solyc12g0	0.32	-1.65
GO:00098	lignin meta	Biological	0.10	0.31	Solyc06g0	Solyc06g0	0.40	-1.33
GO:00040	aldehyde o	Molecular	0.10	0.31	Solyc11g0	Solyc11g0	9.10	3.19
GO:00157	myo-inosit	Biological	0.10	0.31	Solyc11g0	Solyc11g0	0.36	-1.49
GO:00098	plant-type	Biological	0.10	0.31	Solyc05g0	Solyc05g0	3.55	1.83
GO:00422	peptide bir	Molecular	0.10	0.31	Solyc08g0	Solyc08g0	0.39	-1.34
GO:00087	arginine de	Molecular	0.10	0.31	Solyc10g0	Solyc10g0	0.38	-1.40
GO:00517	regulation	Biological	0.10	0.31	Solyc10g0	Solyc10g0	2.92	1.54
GO:00488	root cap de	Biological	0.10	0.31	Solyc11g0	Solyc11g0	0.47	-1.08
GO:00166	12-oxophy	Molecular	0.10	0.31	Solyc07g0	OPR3	0.47	-1.10
GO:00344	ubiquitin-u	Molecular	0.10	0.31	Solyc06g0	Solyc06g0	6.40	2.68
GO:00101	lateral root	Biological	0.10	0.31	Solyc07g0	Solyc07g0	0.25	-1.97
GO:00102	floral orga	Biological	0.10	0.31	Solyc07g0	Solyc07g0	0.25	-1.97
GO:00508	defense res	Biological	0.10	0.31	Solyc07g0	Solyc07g0	0.25	-1.97
GO:00608	leaf abscis	Biological	0.10	0.31	Solyc07g0	Solyc07g0	0.25	-1.97
GO:00168	racemase a	Molecular	0.10	0.31	Solyc03g1	Solyc03g1	0.38	-1.40
GO:00726	mitotic spi	Cellular Co	0.10	0.31	Solyc01g1	Solyc01g1	2.11	1.08
GO:00091	deoxyribon	Biological	0.10	0.31	Solyc11g0	Solyc11g0	2.55	1.35
GO:00102	response to	Biological	0.10	0.31	Solyc11g0	Solyc11g0	2.55	1.35
GO:00084	8-oxo-7,8-	Molecular	0.10	0.31	Solyc03g0	Solyc03g0	0.08	-3.70
GO:00191	dihydroneo	Molecular	0.10	0.31	Solyc03g0	Solyc03g0	0.08	-3.70
GO:00068	regulation	Biological	0.10	0.31	Solyc01g0	Solyc01g0	7223.16	12.82
GO:00151	L-cystine t	Molecular	0.10	0.31	Solyc02g0	Solyc02g0	0.27	-1.90
GO:00158	L-cystine t	Biological	0.10	0.31	Solyc02g0	Solyc02g0	0.27	-1.90
GO:00041	chorismate	Molecular	0.10	0.31	Solyc11g0	Solyc11g0	0.43	-1.20
GO:00464	chorismate	Biological	0.10	0.31	Solyc11g0	Solyc11g0	0.43	-1.20
GO:00056	nuclear pre	Cellular Co	0.10	0.31	Solyc09g0	Solyc09g0	2.25	1.17
GO:00062	pre-replica	Biological	0.10	0.31	Solyc09g0	Solyc09g0	2.25	1.17
GO:00465	S-adenosyl	Biological	0.10	0.31	Solyc01g1	Solyc01g1	2.42	1.28
GO:00103	gibberellin	Molecular	0.10	0.31	Solyc01g0	Solyc01g0	0.50	-1.01
GO:00484	floral orga	Biological	0.10	0.31	Solyc01g0	Solyc01g0	0.50	-1.01
GO:00901	nucleoid o	Biological	0.10	0.31	Solyc08g0	Solyc08g0	0.48	-1.05
GO:00480	inositol lip	Biological	0.10	0.31	Solyc01g1	Solyc01g1	0.48	-1.05
GO:00166	glutaminyl	Molecular	0.10	0.31	Solyc03g0	Solyc03g0	0.15	-2.72
GO:00171	peptidyl-py	Biological	0.10	0.31	Solyc03g0	Solyc03g0	0.15	-2.72
GO:00517	meiotic sis	Biological	0.10	0.31	Solyc07g0	Solyc07g0	2.61	1.38
GO:00049	G protein-c	Molecular	0.10	0.31	Solyc07g0	Solyc07g0	0.50	-1.01
GO:01200	jasmonic a	Molecular	0.10	0.31	Solyc10g0	Solyc10g0	0.46	-1.13
GO:00364	iron-sulfur	Molecular	0.10	0.31	Solyc07g0	Solyc07g0	2.89	1.53
GO:00328	negative re	Biological	0.10	0.31	Solyc03g1	Solyc03g1	2.90	1.54
GO:00458	negative re	Biological	0.10	0.31	Solyc09g0	Solyc09g0	0.37	-1.42
GO:19010	positive re	Biological	0.10	0.31	Solyc06g0	Solyc06g0	0.48	-1.07
GO:00096	nitrate:pro	Molecular	0.10	0.31	Solyc02g0	Solyc02g0	0.31	-1.67
GO:00528	1-deoxy-D	Biological	0.10	0.31	Solyc11g0	Solyc11g0	0.38	-1.39
GO:00022	defense res	Biological	0.10	0.31	Solyc09g0	TD2	4.53	2.18

GO:00039	L-serine ar	Molecular	0.10	0.31	Solyc09g0	TD2	4.53	2.18
GO:00512	protein hoi	Biological	0.10	0.31	Solyc09g0	TD2	4.53	2.18
GO:00039	cystathioni	Molecular	0.10	0.31	Solyc08g0	Solyc08g0	0.07	-3.82
GO:00712	'de novo' L	Biological	0.10	0.31	Solyc08g0	Solyc08g0	0.07	-3.82
GO:00152	thiamine tr	Molecular	0.10	0.31	Solyc12g0	Solyc12g0	0.46	-1.12
GO:00309	thiamine p	Biological	0.10	0.31	Solyc12g0	Solyc12g0	0.46	-1.12
GO:00059	caveola	Cellular C	0.10	0.31	Solyc03g1	Solyc03g1	668.93	9.39
GO:00427	tRNA 3'-tr	Biological	0.10	0.31	Solyc10g0	Solyc10g0	476.24	8.90
GO:00009	DNA-bind	Molecular	0.11	0.32	Solyc06g0	Solyc06g0	0.21	-2.28
GO:00009	DNA-bind	Molecular	0.11	0.32	Solyc02g0	Solyc02g0	3.87	1.95
GO:00009	DNA-bind	Molecular	0.11	0.32	Solyc01g0	Solyc01g0	0.35	-1.51
GO:00009	DNA-bind	Molecular	0.11	0.32	Solyc10g0	Solyc10g0	0.00	-13.06
GO:00009	DNA-bind	Molecular	0.11	0.32	Solyc04g0	Solyc04g0	2.02	1.01
GO:00009	DNA-bind	Molecular	0.11	0.32	Solyc02g0	Solyc02g0	0.19	-2.41
GO:00009	DNA-bind	Molecular	0.11	0.32	Solyc02g0	Solyc02g0	0.45	-1.17
GO:00009	DNA-bind	Molecular	0.11	0.32	Solyc03g1	Solyc03g1	2.90	1.54
GO:00009	DNA-bind	Molecular	0.11	0.32	Solyc05g0	Solyc05g0	0.00	-8.91
GO:00009	DNA-bind	Molecular	0.11	0.32	Solyc03g0	Solyc03g0	11.59	3.53
GO:00009	DNA-bind	Molecular	0.11	0.32	Solyc01g1	Solyc01g1	0.15	-2.78
GO:00082	peptidase a	Molecular	0.11	0.32	Solyc01g1	Solyc01g1	0.45	-1.16
GO:00082	peptidase a	Molecular	0.11	0.32	Solyc08g0	Solyc08g0	0.45	-1.14
GO:00082	peptidase a	Molecular	0.11	0.32	Solyc01g0	Solyc01g0	0.30	-1.74
GO:00082	peptidase a	Molecular	0.11	0.32	Solyc03g0	Solyc03g0	2.00	1.00
GO:00082	peptidase a	Molecular	0.11	0.32	Solyc12g0	Solyc12g0	0.35	-1.50
GO:00082	peptidase a	Molecular	0.11	0.32	Solyc08g0	Solyc08g0	0.11	-3.22
GO:00082	peptidase a	Molecular	0.11	0.32	Solyc01g0	Solyc01g0	0.31	-1.67
GO:00082	peptidase a	Molecular	0.11	0.32	Solyc08g0	Solyc08g0	311.75	8.28
GO:00104	abscisic ac	Molecular	0.11	0.32	Solyc09g0	Solyc09g0	0.37	-1.43
GO:00104	abscisic ac	Molecular	0.11	0.32	Solyc06g0	Solyc06g0	0.41	-1.28
GO:00104	abscisic ac	Molecular	0.11	0.32	Solyc06g0	Solyc06g0	0.39	-1.36
GO:00002	polysaccha	Biological	0.11	0.32	Solyc02g0	CHI3	0.24	-2.08
GO:00002	polysaccha	Biological	0.11	0.32	Solyc08g0	Solyc08g0	2.07	1.05
GO:00002	polysaccha	Biological	0.11	0.32	Solyc10g0	CHI9	0.27	-1.88
GO:00082	serine-type	Molecular	0.11	0.32	Solyc05g0	Solyc05g0	0.24	-2.03
GO:00082	serine-type	Molecular	0.11	0.32	Solyc04g0	Solyc04g0	0.18	-2.44
GO:00082	serine-type	Molecular	0.11	0.32	Solyc10g0	Solyc10g0	0.09	-3.52
GO:00082	serine-type	Molecular	0.11	0.32	Solyc01g0	Solyc01g0	0.30	-1.74
GO:00082	serine-type	Molecular	0.11	0.32	Solyc09g0	Solyc09g0	3.21	1.68
GO:00082	serine-type	Molecular	0.11	0.32	Solyc08g0	Solyc08g0	0.11	-3.22
GO:00082	serine-type	Molecular	0.11	0.32	Solyc01g0	Solyc01g0	0.31	-1.67
GO:00082	serine-type	Molecular	0.11	0.32	Solyc02g0	Solyc02g0	722.84	9.50
GO:00082	serine-type	Molecular	0.11	0.32	Solyc10g0	Solyc10g0	0.30	-1.75
GO:00065	L-phenylal	Biological	0.11	0.32	Solyc09g0	Solyc09g0	0.08	-3.59
GO:00065	L-phenylal	Biological	0.11	0.32	Solyc09g0	Solyc09g0	0.09	-3.50
GO:00801	response to	Biological	0.11	0.32	Solyc11g0	Solyc11g0	0.11	-3.13
GO:00801	response to	Biological	0.11	0.32	Solyc03g0	Solyc03g0	2.58	1.37
GO:00063	double-stra	Biological	0.11	0.32	Solyc11g0	Solyc11g0	0.43	-1.23
GO:00063	double-stra	Biological	0.11	0.32	Solyc11g0	Solyc11g0	2.55	1.35
GO:00609	auxin trans	Biological	0.11	0.32	Solyc05g0	Solyc05g0	0.18	-2.51
GO:00609	auxin trans	Biological	0.11	0.32	Solyc03g1	Solyc03g1	0.48	-1.05
GO:00102	photosyste	Biological	0.11	0.32	Solyc02g0	PSBO	0.48	-1.07

GO:00102	photosyste	Biological	0.11	0.32	Solyc07g0	Solyc07g0	0.41	-1.28
GO:00346	cellular res	Biological	0.11	0.32	Solyc10g0	Solyc10g0	0.47	-1.09
GO:00346	cellular res	Biological	0.11	0.32	Solyc03g1	Solyc03g1	0.00	-11.76
GO:00436	cortical mi	Biological	0.11	0.32	Solyc02g0	Solyc02g0	0.47	-1.09
GO:00436	cortical mi	Biological	0.11	0.32	Solyc04g0	Solyc04g0	0.48	-1.06
GO:00125	programm	Biological	0.11	0.32	Solyc09g0	Solyc09g0	0.48	-1.06
GO:00125	programm	Biological	0.11	0.32	Solyc01g0	Solyc01g0	0.37	-1.43
GO:00099	chloroplas	Biological	0.11	0.32	Solyc06g0	Solyc06g0	5.58	2.48
GO:00099	chloroplas	Biological	0.11	0.32	Solyc12g0	Solyc12g0	9.14	3.19
GO:00338	ribonuclea	Molecular	0.11	0.32	Solyc07g0	Solyc07g0	6759.25	12.72
GO:00338	ribonuclea	Molecular	0.11	0.32	Solyc05g0	Solyc05g0	0.48	-1.07
GO:00168	1-aminocy	Molecular	0.11	0.32	Solyc03g0	Solyc03g0	0.00	-11.21
GO:00168	1-aminocy	Molecular	0.11	0.32	Solyc08g0	Solyc08g0	0.17	-2.53
GO:00422	1-aminocy	Biological	0.11	0.32	Solyc03g0	Solyc03g0	0.00	-11.21
GO:00422	1-aminocy	Biological	0.11	0.32	Solyc08g0	Solyc08g0	0.17	-2.53
GO:00346	cellular res	Biological	0.11	0.33	Solyc10g0	Solyc10g0	0.47	-1.09
GO:00346	cellular res	Biological	0.11	0.33	Solyc09g0	Solyc09g0	0.48	-1.06
GO:00346	cellular res	Biological	0.11	0.33	Solyc03g1	Solyc03g1	0.00	-11.76
GO:00346	cellular res	Biological	0.11	0.33	Solyc07g0	Solyc07g0	0.07	-3.80
GO:00043	glutathion	Molecular	0.12	0.34	Solyc01g0	Solyc01g0	0.31	-1.69
GO:00043	glutathion	Molecular	0.12	0.34	Solyc01g0	Solyc01g0	0.24	-2.03
GO:00043	glutathion	Molecular	0.12	0.34	Solyc09g0	Solyc09g0	0.47	-1.08
GO:00043	glutathion	Molecular	0.12	0.34	Solyc05g0	Solyc05g0	0.32	-1.65
GO:00043	glutathion	Molecular	0.12	0.34	Solyc09g0	Solyc09g0	0.34	-1.56
GO:19909	xenobiotic	Biological	0.12	0.34	Solyc04g0	Solyc04g0	0.19	-2.40
GO:19909	xenobiotic	Biological	0.12	0.34	Solyc03g1	Solyc03g1	0.47	-1.08
GO:19909	xenobiotic	Biological	0.12	0.34	Solyc02g0	Solyc02g0	0.33	-1.61
GO:19909	xenobiotic	Biological	0.12	0.34	Solyc05g0	Solyc05g0	0.06	-4.14
GO:19909	xenobiotic	Biological	0.12	0.34	Solyc08g0	Solyc08g0	3.30	1.72
GO:00065	proteolysis	Biological	0.12	0.34	Solyc01g1	Solyc01g1	0.45	-1.16
GO:00065	proteolysis	Biological	0.12	0.34	Solyc08g0	Solyc08g0	2.25	1.17
GO:00065	proteolysis	Biological	0.12	0.34	Solyc11g0	Solyc11g0	0.46	-1.12
GO:00065	proteolysis	Biological	0.12	0.34	Solyc08g0	Solyc08g0	0.45	-1.14
GO:00065	proteolysis	Biological	0.12	0.34	Solyc05g0	Solyc05g0	0.24	-2.03
GO:00065	proteolysis	Biological	0.12	0.34	Solyc01g0	Solyc01g0	0.46	-1.12
GO:00065	proteolysis	Biological	0.12	0.34	Solyc04g0	Solyc04g0	0.18	-2.44
GO:00065	proteolysis	Biological	0.12	0.34	Solyc12g0	Solyc12g0	0.43	-1.22
GO:00065	proteolysis	Biological	0.12	0.34	Solyc04g0	Solyc04g0	0.40	-1.33
GO:00065	proteolysis	Biological	0.12	0.34	Solyc10g0	Solyc10g0	0.09	-3.52
GO:00065	proteolysis	Biological	0.12	0.34	Solyc01g0	Solyc01g0	0.30	-1.74
GO:00065	proteolysis	Biological	0.12	0.34	Solyc06g0	Solyc06g0	0.49	-1.04
GO:00065	proteolysis	Biological	0.12	0.34	Solyc05g0	Solyc05g0	2.08	1.06
GO:00065	proteolysis	Biological	0.12	0.34	Solyc08g0	Solyc08g0	0.48	-1.06
GO:00065	proteolysis	Biological	0.12	0.34	Solyc03g0	Solyc03g0	2.00	1.00
GO:00065	proteolysis	Biological	0.12	0.34	Solyc01g1	Solyc01g1	2.11	1.08
GO:00065	proteolysis	Biological	0.12	0.34	Solyc11g0	Solyc11g0	3.66	1.87
GO:00065	proteolysis	Biological	0.12	0.34	Solyc01g0	Solyc01g0	0.24	-2.04
GO:00065	proteolysis	Biological	0.12	0.34	Solyc12g0	Solyc12g0	0.35	-1.50
GO:00065	proteolysis	Biological	0.12	0.34	Solyc09g0	Solyc09g0	4.04	2.01
GO:00065	proteolysis	Biological	0.12	0.34	Solyc12g0	Solyc12g0	2321.45	11.18
GO:00065	proteolysis	Biological	0.12	0.34	Solyc02g0	Solyc02g0	0.46	-1.13

GO:00065	proteolysis	Biological	0.12	0.34	Solyc09g0	Solyc09g0	3.21	1.68
GO:00065	proteolysis	Biological	0.12	0.34	Solyc08g0	Solyc08g0	0.11	-3.22
GO:00065	proteolysis	Biological	0.12	0.34	Solyc03g0	Solyc03g0	2.33	1.22
GO:00065	proteolysis	Biological	0.12	0.34	Solyc01g0	Solyc01g0	0.31	-1.67
GO:00065	proteolysis	Biological	0.12	0.34	Solyc01g0	Solyc01g0	0.18	-2.50
GO:00065	proteolysis	Biological	0.12	0.34	Solyc02g0	Solyc02g0	722.84	9.50
GO:00065	proteolysis	Biological	0.12	0.34	Solyc10g0	Solyc10g0	0.30	-1.75
GO:00065	proteolysis	Biological	0.12	0.34	Solyc08g0	Solyc08g0	311.75	8.28
GO:00068	potassium	Biological	0.12	0.35	Solyc01g1	Solyc01g1	0.43	-1.23
GO:00068	potassium	Biological	0.12	0.35	Solyc05g0	Solyc05g0	11.71	3.55
GO:00068	potassium	Biological	0.12	0.35	Solyc08g0	Solyc08g0	0.40	-1.32
GO:00068	potassium	Biological	0.12	0.35	Solyc12g0	Solyc12g0	0.48	-1.07
GO:00082	lipid bindi	Molecular	0.13	0.36	Solyc09g0	Solyc09g0	2.72	1.44
GO:00082	lipid bindi	Molecular	0.13	0.36	Solyc05g0	Solyc05g0	3.29	1.72
GO:00082	lipid bindi	Molecular	0.13	0.36	Solyc10g0	Solyc10g0	0.01	-7.08
GO:00082	lipid bindi	Molecular	0.13	0.36	Solyc01g0	Solyc01g0	0.42	-1.26
GO:00082	lipid bindi	Molecular	0.13	0.36	Solyc01g1	Solyc01g1	7.06	2.82
GO:00082	lipid bindi	Molecular	0.13	0.36	Solyc01g0	Solyc01g0	2.38	1.25
GO:00082	lipid bindi	Molecular	0.13	0.36	Solyc03g0	Solyc03g0	0.40	-1.33
GO:00082	lipid bindi	Molecular	0.13	0.36	Solyc08g0	Solyc08g0	2.07	1.05
GO:00082	lipid bindi	Molecular	0.13	0.36	Solyc09g0	Solyc09g0	0.18	-2.45
GO:00082	lipid bindi	Molecular	0.13	0.36	Solyc07g0	Solyc07g0	2.43	1.28
GO:00082	lipid bindi	Molecular	0.13	0.36	Solyc07g0	Solyc07g0	638.37	9.32
GO:00168	lyase activ	Molecular	0.13	0.36	Solyc09g0	Solyc09g0	0.08	-3.59
GO:00168	lyase activ	Molecular	0.13	0.36	Solyc11g0	AOS2	0.12	-3.01
GO:00168	lyase activ	Molecular	0.13	0.36	Solyc10g0	Solyc10g0	0.38	-1.40
GO:00168	lyase activ	Molecular	0.13	0.36	Solyc08g0	Solyc08g0	0.11	-3.24
GO:00168	lyase activ	Molecular	0.13	0.36	Solyc03g0	Solyc03g0	0.00	-11.21
GO:00168	lyase activ	Molecular	0.13	0.36	Solyc08g0	Solyc08g0	0.00	-10.99
GO:00168	lyase activ	Molecular	0.13	0.36	Solyc05g0	Solyc05g0	0.48	-1.07
GO:00168	lyase activ	Molecular	0.13	0.36	Solyc09g0	TD2	4.53	2.18
GO:00168	lyase activ	Molecular	0.13	0.36	Solyc08g0	Solyc08g0	799.01	9.64
GO:00168	lyase activ	Molecular	0.13	0.36	Solyc08g0	Solyc08g0	0.17	-2.53
GO:00095	plastid inn	Cellular C	0.13	0.36	Solyc11g0	AOS2	0.12	-3.01
GO:00095	plastid inn	Cellular C	0.13	0.36	Solyc11g0	Solyc11g0	0.24	-2.07
GO:00442	cellular lip	Biological	0.13	0.36	Solyc03g0	Solyc03g0	2.46	1.30
GO:00442	cellular lip	Biological	0.13	0.36	Solyc01g1	Solyc01g1	0.40	-1.31
GO:00045	beta-galact	Molecular	0.13	0.36	Solyc03g1	Solyc03g1	2.21	1.14
GO:00045	beta-galact	Molecular	0.13	0.36	Solyc03g0	Solyc03g0	2.58	1.37
GO:00066	phospholip	Biological	0.13	0.36	Solyc01g0	Solyc01g0	2.06	1.04
GO:00066	phospholip	Biological	0.13	0.36	Solyc02g0	Solyc02g0	0.42	-1.27
GO:20000	regulation	Biological	0.13	0.36	Solyc09g0	Solyc09g0	0.48	-1.06
GO:20000	regulation	Biological	0.13	0.36	Solyc01g0	Solyc01g0	0.37	-1.43
GO:00067	glutathione	Biological	0.13	0.36	Solyc01g0	Solyc01g0	0.31	-1.69
GO:00067	glutathione	Biological	0.13	0.36	Solyc01g0	Solyc01g0	0.24	-2.03
GO:00067	glutathione	Biological	0.13	0.36	Solyc09g0	Solyc09g0	0.47	-1.08
GO:00067	glutathione	Biological	0.13	0.36	Solyc05g0	Solyc05g0	0.32	-1.65
GO:00067	glutathione	Biological	0.13	0.36	Solyc09g0	Solyc09g0	0.34	-1.56
GO:00067	glutathione	Biological	0.13	0.36	Solyc01g0	Solyc01g0	0.23	-2.09
GO:00063	DNA reco	Biological	0.13	0.36	Solyc03g1	Solyc03g1	2.29	1.19
GO:00063	DNA reco	Biological	0.13	0.36	Solyc01g0	Solyc01g0	208.08	7.70

GO:00063	DNA recoı	Biological	0.13	0.36	Solyc09g0	Solyc09g0	3.39	1.76
GO:00063	DNA recoı	Biological	0.13	0.36	Solyc08g0	Solyc08g0	9.73	3.28
GO:00063	DNA recoı	Biological	0.13	0.36	Solyc10g0	Solyc10g0	2.30	1.20
GO:00063	DNA recoı	Biological	0.13	0.36	Solyc02g0	Solyc02g0	0.23	-2.09
GO:00425	response tc	Biological	0.13	0.36	Solyc12g0	Solyc12g0	0.28	-1.84
GO:00425	response tc	Biological	0.13	0.36	Solyc04g0	Solyc04g0	0.49	-1.03
GO:00425	response tc	Biological	0.13	0.36	Solyc01g0	Solyc01g0	0.45	-1.15
GO:00048	protein pho	Molecular	0.13	0.36	Solyc09g0	Solyc09g0	0.37	-1.43
GO:00048	protein pho	Molecular	0.13	0.36	Solyc06g0	Solyc06g0	0.41	-1.28
GO:00048	protein pho	Molecular	0.13	0.36	Solyc06g0	Solyc06g0	0.39	-1.36
GO:00096	response tc	Biological	0.13	0.36	Solyc02g0	Solyc02g0	0.23	-2.13
GO:00096	response tc	Biological	0.13	0.36	Solyc07g0	NCED1	0.30	-1.72
GO:00096	response tc	Biological	0.13	0.36	Solyc04g0	Solyc04g0	0.49	-1.03
GO:00096	response tc	Biological	0.13	0.36	Solyc09g0	Solyc09g0	0.44	-1.17
GO:00096	response tc	Biological	0.13	0.36	Solyc01g0	Solyc01g0	0.45	-1.15
GO:00096	response tc	Biological	0.13	0.36	Solyc12g0	Solyc12g0	0.40	-1.34
GO:00096	response tc	Biological	0.13	0.36	Solyc12g0	Solyc12g0	0.48	-1.07
GO:00167	hexosyltra	Molecular	0.13	0.36	Solyc09g0	Solyc09g0	0.07	-3.81
GO:00167	hexosyltra	Molecular	0.13	0.36	Solyc12g0	Solyc12g0	2.94	1.56
GO:00167	hexosyltra	Molecular	0.13	0.36	Solyc07g0	Solyc07g0	0.34	-1.55
GO:00167	hexosyltra	Molecular	0.13	0.36	Solyc03g0	Solyc03g0	0.25	-2.02
GO:00167	hexosyltra	Molecular	0.13	0.36	Solyc02g0	Solyc02g0	0.11	-3.14
GO:00167	hexosyltra	Molecular	0.13	0.36	Solyc01g1	Solyc01g1	0.39	-1.36
GO:00167	hexosyltra	Molecular	0.13	0.36	Solyc10g0	Solyc10g0	3.52	1.82
GO:00151	amino acid	Molecular	0.14	0.36	Solyc03g0	Solyc03g0	0.48	-1.07
GO:00151	amino acid	Molecular	0.14	0.36	Solyc04g0	Solyc04g0	0.17	-2.54
GO:00151	amino acid	Molecular	0.14	0.36	Solyc01g1	Solyc01g1	0.26	-1.97
GO:00151	amino acid	Molecular	0.14	0.36	Solyc11g0	Solyc11g0	0.47	-1.08
GO:00069	nucleocyto	Biological	0.15	0.36	Solyc11g0	Solyc11g0	8.11	3.02
GO:00069	nucleocyto	Biological	0.15	0.36	Solyc02g0	Solyc02g0	4.79	2.26
GO:00069	nucleocyto	Biological	0.15	0.36	Solyc02g0	Solyc02g0	0.25	-1.99
GO:00070	mitotic sist	Biological	0.15	0.36	Solyc03g1	Solyc03g1	0.30	-1.72
GO:00070	mitotic sist	Biological	0.15	0.36	Solyc01g0	Solyc01g0	2.42	1.27
GO:00070	mitotic sist	Biological	0.15	0.36	Solyc06g0	Solyc06g0	0.32	-1.65
GO:00717	organic sul	Biological	0.15	0.36	Solyc01g0	Solyc01g0	2.53	1.34
GO:00717	organic sul	Biological	0.15	0.36	Solyc06g0	Solyc06g0	0.18	-2.48
GO:00717	organic sul	Biological	0.15	0.36	Solyc11g0	Solyc11g0	0.04	-4.78
GO:00511	sugar trans	Molecular	0.15	0.36	Solyc04g0	Solyc04g0	0.48	-1.07
GO:00511	sugar trans	Molecular	0.15	0.36	Solyc01g0	Solyc01g0	0.44	-1.18
GO:00511	sugar trans	Molecular	0.15	0.36	Solyc01g0	Solyc01g0	0.12	-3.07
GO:00151	nitrate tran	Molecular	0.15	0.36	Solyc10g0	Solyc10g0	2.72	1.45
GO:00151	nitrate tran	Molecular	0.15	0.36	Solyc06g0	Solyc06g0	0.37	-1.45
GO:00181	peptidyl-th	Biological	0.15	0.36	Solyc11g0	Solyc11g0	0.49	-1.03
GO:00181	peptidyl-th	Biological	0.15	0.36	Solyc08g0	Solyc08g0	2.48	1.31
GO:00012	DNA-bind	Molecular	0.15	0.36	Solyc04g0	Solyc04g0	2.02	1.01
GO:00012	DNA-bind	Molecular	0.15	0.36	Solyc03g1	Solyc03g1	2.90	1.54
GO:00427	defense res	Biological	0.15	0.36	Solyc10g0	Solyc10g0	0.03	-5.15
GO:00427	defense res	Biological	0.15	0.36	Solyc01g0	Solyc01g0	0.33	-1.59
GO:00427	defense res	Biological	0.15	0.36	Solyc08g0	Solyc08g0	0.39	-1.34
GO:00427	defense res	Biological	0.15	0.36	Solyc09g0	Solyc09g0	0.37	-1.42
GO:00427	defense res	Biological	0.15	0.36	Solyc06g0	Solyc06g0	0.48	-1.07

GO:00098	cinnamic a	Biological	0.15	0.36	Solyc09g0	Solyc09g0	0.08	-3.59
GO:00506	hydrogen p	Biological	0.15	0.36	Solyc10g0	Solyc10g0	0.03	-5.15
GO:00099	allene oxid	Molecular	0.15	0.36	Solyc11g0	AOS2	0.12	-3.01
GO:00159	glutamate :	Molecular	0.15	0.36	Solyc03g0	Solyc03g0	2.69	1.43
GO:00312	arabinan ca	Biological	0.15	0.36	Solyc01g1	Solyc01g1	2.32	1.21
GO:00044	hydroxyme	Molecular	0.15	0.36	Solyc02g0	Solyc02g0	2.02	1.01
GO:00159	coenzyme	Biological	0.15	0.36	Solyc02g0	Solyc02g0	2.02	1.01
GO:00090	anaerobic r	Biological	0.15	0.36	Solyc02g0	Solyc02g0	4.39	2.13
GO:00513	cellular res	Biological	0.15	0.36	Solyc07g0	Solyc07g0	0.16	-2.62
GO:00504	arachidoni	Biological	0.15	0.36	Solyc01g0	Solyc01g0	2.06	1.04
GO:00038	ATP citrat	Molecular	0.15	0.36	Solyc05g0	Solyc05g0	2.01	1.01
GO:00060	acetyl-CoA	Biological	0.15	0.36	Solyc05g0	Solyc05g0	2.01	1.01
GO:00093	ATP-indep	Cellular Co	0.15	0.36	Solyc05g0	Solyc05g0	2.01	1.01
GO:00038	3-hydroxya	Molecular	0.15	0.36	Solyc08g0	Solyc08g0	0.21	-2.27
GO:00162	AMP bindi	Molecular	0.15	0.36	Solyc06g0	Solyc06g0	0.44	-1.19
GO:00300	cell junctic	Cellular Co	0.15	0.36	Solyc03g0	RBCS-2A	0.34	-1.58
GO:00041	citrate (Si)	Molecular	0.15	0.36	Solyc07g0	Solyc07g0	0.49	-1.04
GO:00484	gynoecium	Biological	0.15	0.36	Solyc04g0	Solyc04g0	0.48	-1.06
GO:00512	anisotropic	Biological	0.15	0.36	Solyc04g0	Solyc04g0	0.48	-1.06
GO:20010	regulation	Biological	0.15	0.36	Solyc04g0	Solyc04g0	0.48	-1.06
GO:00059	glycogen c	Biological	0.15	0.36	Solyc05g0	Solyc05g0	0.37	-1.44
GO:01022	linear malt	Molecular	0.15	0.36	Solyc05g0	Solyc05g0	0.37	-1.44
GO:01024	SHG alpha	Molecular	0.15	0.36	Solyc05g0	Solyc05g0	0.37	-1.44
GO:00062	regulation	Biological	0.15	0.36	Solyc01g0	Solyc01g0	2.42	1.27
GO:00442	'de novo' U	Biological	0.15	0.36	Solyc01g0	Solyc01g0	0.29	-1.78
GO:00045	beta-glucu	Molecular	0.15	0.36	Solyc03g1	Solyc03g1	0.41	-1.27
GO:00971	organic cy	Molecular	0.15	0.36	Solyc12g0	Solyc12g0	0.32	-1.65
GO:19013	heterocycli	Molecular	0.15	0.36	Solyc12g0	Solyc12g0	0.32	-1.65
GO:00053	myo-inosit	Molecular	0.15	0.36	Solyc11g0	Solyc11g0	0.36	-1.49
GO:00314	BRCA1-B.	Cellular Co	0.15	0.36	Solyc06g0	Solyc06g0	2.41	1.27
GO:00015	regulation	Biological	0.15	0.36	Solyc08g0	Solyc08g0	0.39	-1.34
GO:00703	cellular he	Biological	0.15	0.36	Solyc08g0	Solyc08g0	0.39	-1.34
GO:00065	arginine ca	Biological	0.15	0.36	Solyc10g0	Solyc10g0	0.38	-1.40
GO:00087	D-alanine-	Molecular	0.15	0.36	Solyc10g0	Solyc10g0	0.43	-1.22
GO:00197	NEDD8-sp	Molecular	0.15	0.36	Solyc05g0	Solyc05g0	2.08	1.06
GO:00101	response to	Biological	0.15	0.36	Solyc07g0	OPR3	0.47	-1.10
GO:00047	thymidine	Molecular	0.15	0.36	Solyc11g0	Solyc11g0	2.55	1.35
GO:00702	peptidyl-se	Biological	0.15	0.36	Solyc06g0	Solyc06g0	0.33	-1.61
GO:00602	regulation	Biological	0.15	0.36	Solyc06g0	Solyc06g0	4.37	2.13
GO:00515	regulation	Biological	0.15	0.36	Solyc02g0	Solyc02g0	0.46	-1.11
GO:00099	positive re	Biological	0.15	0.36	Solyc01g0	Solyc01g0	0.50	-1.01
GO:00007	condensin	Cellular Co	0.15	0.36	Solyc07g0	Solyc07g0	2.25	1.17
GO:00066	phosphatid	Biological	0.15	0.36	Solyc01g1	Solyc01g1	0.48	-1.05
GO:00044	homoserin	Molecular	0.15	0.36	Solyc06g0	Solyc06g0	0.30	-1.75
GO:00090	aspartate fa	Biological	0.15	0.36	Solyc06g0	Solyc06g0	0.30	-1.75
GO:00300	chloroplast	Cellular Co	0.15	0.36	Solyc08g0	Solyc08g0	0.21	-2.23
GO:00005	glycosylph	Cellular Co	0.15	0.36	Solyc03g0	Solyc03g0	2.48	1.31
GO:00484	replication	Biological	0.15	0.36	Solyc04g0	Solyc04g0	2.19	1.13
GO:00325	adenyl ribo	Molecular	0.15	0.36	Solyc11g0	Solyc11g0	0.33	-1.60
GO:00086	1-deoxy-D	Molecular	0.15	0.36	Solyc11g0	Solyc11g0	0.38	-1.39
GO:00060	glycerol m	Biological	0.15	0.36	Solyc03g1	Solyc03g1	0.34	-1.57

GO:00800	response to	Biological	0.15	0.36	Solyc09g0	TD2	4.53	2.18
GO:19905	potassium	Biological	0.15	0.36	Solyc12g0	Solyc12g0	0.48	-1.07
GO:00353	5'-3' exode	Molecular	0.15	0.36	Solyc02g0	Solyc02g0	2.19	1.13
GO:00084	dTDP-gluc	Molecular	0.15	0.36	Solyc07g0	Solyc07g0	0.37	-1.43
GO:00092	nucleotide-	Biological	0.15	0.36	Solyc07g0	Solyc07g0	0.37	-1.43
GO:00104	thermospe	Molecular	0.15	0.36	Solyc08g0	Solyc08g0	2.74	1.45
GO:00086	cellular an	Biological	0.16	0.37	Solyc09g0	Solyc09g0	0.01	-6.18
GO:00086	cellular an	Biological	0.16	0.37	Solyc06g0	Solyc06g0	0.30	-1.75
GO:00086	cellular an	Biological	0.16	0.37	Solyc09g0	TD2	4.53	2.18
GO:00167	phosphotra	Molecular	0.16	0.37	Solyc11g0	Solyc11g0	0.33	-1.59
GO:00167	phosphotra	Molecular	0.16	0.37	Solyc11g0	Solyc11g0	0.33	-1.61
GO:00167	phosphotra	Molecular	0.16	0.37	Solyc03g1	Solyc03g1	0.34	-1.57
GO:00152	purine nuc	Molecular	0.16	0.37	Solyc12g0	Solyc12g0	0.45	-1.16
GO:00152	purine nuc	Molecular	0.16	0.37	Solyc04g0	Solyc04g0	0.44	-1.19
GO:00152	purine nuc	Molecular	0.16	0.37	Solyc06g0	Solyc06g0	0.08	-3.56
GO:00057	peroxisom	Cellular Co	0.16	0.37	Solyc10g0	Solyc10g0	0.03	-5.15
GO:00057	peroxisom	Cellular Co	0.16	0.37	Solyc12g0	Solyc12g0	0.28	-1.84
GO:00057	peroxisom	Cellular Co	0.16	0.37	Solyc08g0	Solyc08g0	0.21	-2.27
GO:00057	peroxisom	Cellular Co	0.16	0.37	Solyc07g0	OPR3	0.47	-1.10
GO:00057	peroxisom	Cellular Co	0.16	0.37	Solyc12g0	Solyc12g0	0.30	-1.74
GO:00466	anchored c	Cellular Co	0.16	0.37	Solyc01g0	Solyc01g0	0.04	-4.82
GO:00466	anchored c	Cellular Co	0.16	0.37	Solyc12g0	Solyc12g0	0.44	-1.19
GO:00466	anchored c	Cellular Co	0.16	0.37	Solyc12g0	Solyc12g0	3.12	1.64
GO:00466	anchored c	Cellular Co	0.16	0.37	Solyc11g0	Solyc11g0	2.71	1.44
GO:00466	anchored c	Cellular Co	0.16	0.37	Solyc03g1	Solyc03g1	0.00	-12.84
GO:00466	anchored c	Cellular Co	0.16	0.37	Solyc07g0	Solyc07g0	0.12	-3.04
GO:00466	anchored c	Cellular Co	0.16	0.37	Solyc07g0	Solyc07g0	0.44	-1.19
GO:00090	biosyntheti	Biological	0.16	0.37	Solyc09g0	Solyc09g0	0.01	-7.13
GO:00090	biosyntheti	Biological	0.16	0.37	Solyc05g0	Solyc05g0	0.01	-6.95
GO:00090	biosyntheti	Biological	0.16	0.37	Solyc01g0	Solyc01g0	0.47	-1.10
GO:00090	biosyntheti	Biological	0.16	0.37	Solyc11g0	Solyc11g0	0.32	-1.65
GO:00090	biosyntheti	Biological	0.16	0.37	Solyc03g0	Solyc03g0	0.00	-11.21
GO:00090	biosyntheti	Biological	0.16	0.37	Solyc01g1	Solyc01g1	2.42	1.28
GO:00090	biosyntheti	Biological	0.16	0.37	Solyc08g0	Solyc08g0	0.17	-2.53
GO:00090	biosyntheti	Biological	0.16	0.37	Solyc06g0	Solyc06g0	0.00	-9.59
GO:00090	biosyntheti	Biological	0.16	0.37	Solyc06g0	Solyc06g0	0.00	-9.26
GO:00105	green leaf	Biological	0.16	0.38	Solyc01g0	Solyc01g0	4.36	2.12
GO:00105	green leaf	Biological	0.16	0.38	Solyc01g0	Solyc01g0	0.04	-4.49
GO:00105	green leaf	Biological	0.16	0.38	Solyc10g0	Solyc10g0	0.00	-13.06
GO:00105	green leaf	Biological	0.16	0.38	Solyc02g0	Solyc02g0	0.00	-9.32
GO:00197	carboxylic	Biological	0.16	0.38	Solyc08g0	Solyc08g0	0.11	-3.24
GO:00197	carboxylic	Biological	0.16	0.38	Solyc08g0	Solyc08g0	0.00	-10.99
GO:00197	carboxylic	Biological	0.16	0.38	Solyc08g0	Solyc08g0	0.05	-4.43
GO:00197	carboxylic	Biological	0.16	0.38	Solyc08g0	Solyc08g0	799.01	9.64
GO:00068	phosphate	Biological	0.17	0.38	Solyc09g0	Solyc09g0	0.14	-2.81
GO:00068	phosphate	Biological	0.17	0.38	Solyc12g0	Solyc12g0	2.45	1.29
GO:00068	phosphate	Biological	0.17	0.38	Solyc09g0	Solyc09g0	0.18	-2.47
GO:00068	phosphate	Biological	0.17	0.38	Solyc11g0	Solyc11g0	0.36	-1.49
GO:00068	phosphate	Biological	0.17	0.38	Solyc06g0	Solyc06g0	0.37	-1.45
GO:00002	mitotic cel	Biological	0.17	0.38	Solyc06g0	Solyc06g0	2.52	1.33
GO:00002	mitotic cel	Biological	0.17	0.38	Solyc06g0	Solyc06g0	0.33	-1.61

GO:00002	mitotic cel	Biological	0.17	0.38	Solyc02g0	Solyc02g0	8.65	3.11
GO:00002	mitotic cel	Biological	0.17	0.38	Solyc04g0	Solyc04g0	2.93	1.55
GO:00002	mitotic cel	Biological	0.17	0.38	Solyc04g0	Solyc04g0	2.18	1.13
GO:00701	large ribos	Molecular	0.17	0.38	Solyc09g0	Solyc09g0	3.66	1.87
GO:00701	large ribos	Molecular	0.17	0.38	Solyc09g0	Solyc09g0	6.55	2.71
GO:00096	plant-type	Biological	0.17	0.38	Solyc09g0	Solyc09g0	0.48	-1.06
GO:00096	plant-type	Biological	0.17	0.38	Solyc01g0	Solyc01g0	0.37	-1.43
GO:00152	symporter	Molecular	0.17	0.38	Solyc12g0	Solyc12g0	2.45	1.29
GO:00152	symporter	Molecular	0.17	0.38	Solyc01g0	Solyc01g0	0.50	-1.01
GO:00349	response tc	Biological	0.17	0.38	Solyc06g0	Solyc06g0	0.06	-4.15
GO:00349	response tc	Biological	0.17	0.38	Solyc06g0	Solyc06g0	0.07	-3.94
GO:00088	cellulase a	Molecular	0.17	0.38	Solyc05g0	Solyc05g0	0.37	-1.42
GO:00088	cellulase a	Molecular	0.17	0.38	Solyc08g0	Solyc08g0	2.07	1.05
GO:00302	cellulose c	Biological	0.17	0.38	Solyc05g0	Solyc05g0	0.37	-1.42
GO:00302	cellulose c	Biological	0.17	0.38	Solyc08g0	Solyc08g0	2.07	1.05
GO:00067	ubiquinone	Biological	0.17	0.38	Solyc06g0	Solyc06g0	0.40	-1.33
GO:00067	ubiquinone	Biological	0.17	0.38	Solyc10g0	Solyc10g0	0.28	-1.85
GO:00056	transcriptic	Cellular Co	0.17	0.38	Solyc04g0	Solyc04g0	2.02	1.01
GO:00056	transcriptic	Cellular Co	0.17	0.38	Solyc03g1	Solyc03g1	2.90	1.54
GO:00903	nucleic aci	Biological	0.17	0.38	Solyc05g0	Solyc05g0	0.48	-1.07
GO:00903	nucleic aci	Biological	0.17	0.38	Solyc07g0	Solyc07g0	0.47	-1.08
GO:00380	signaling r	Molecular	0.17	0.38	Solyc09g0	Solyc09g0	0.37	-1.43
GO:00380	signaling r	Molecular	0.17	0.38	Solyc06g0	Solyc06g0	0.41	-1.28
GO:00380	signaling r	Molecular	0.17	0.38	Solyc06g0	Solyc06g0	0.39	-1.36
GO:00003	response tc	Biological	0.17	0.38	Solyc07g0	Solyc07g0	0.46	-1.12
GO:00003	response tc	Biological	0.17	0.38	Solyc04g0	Solyc04g0	0.49	-1.03
GO:00003	response tc	Biological	0.17	0.38	Solyc02g0	Solyc02g0	2.89	1.53
GO:00053	carbohydra	Molecular	0.17	0.38	Solyc12g0	Solyc12g0	2.45	1.29
GO:00053	carbohydra	Molecular	0.17	0.38	Solyc11g0	Solyc11g0	0.36	-1.49
GO:00053	carbohydra	Molecular	0.17	0.38	Solyc01g0	Solyc01g0	0.44	-1.18
GO:00053	glucose tra	Molecular	0.17	0.38	Solyc12g0	Solyc12g0	2.45	1.29
GO:00053	glucose tra	Molecular	0.17	0.38	Solyc11g0	Solyc11g0	0.36	-1.49
GO:00053	glucose tra	Molecular	0.17	0.38	Solyc01g0	Solyc01g0	0.44	-1.18
GO:00070	regulation	Biological	0.17	0.38	Solyc02g0	Solyc02g0	8.65	3.11
GO:00070	regulation	Biological	0.17	0.38	Solyc04g0	Solyc04g0	2.93	1.55
GO:00070	regulation	Biological	0.17	0.38	Solyc04g0	Solyc04g0	2.18	1.13
GO:00429	xenobiotic	Molecular	0.18	0.39	Solyc04g0	Solyc04g0	0.19	-2.40
GO:00429	xenobiotic	Molecular	0.18	0.39	Solyc03g1	Solyc03g1	0.47	-1.08
GO:00429	xenobiotic	Molecular	0.18	0.39	Solyc02g0	Solyc02g0	0.33	-1.61
GO:00429	xenobiotic	Molecular	0.18	0.39	Solyc05g0	Solyc05g0	0.06	-4.14
GO:00429	xenobiotic	Molecular	0.18	0.39	Solyc07g0	Solyc07g0	0.00	-9.97
GO:00429	xenobiotic	Molecular	0.18	0.39	Solyc08g0	Solyc08g0	3.30	1.72
GO:00012	DNA-bind	Molecular	0.18	0.39	Solyc04g0	Solyc04g0	2.02	1.01
GO:00012	DNA-bind	Molecular	0.18	0.39	Solyc05g0	Solyc05g0	0.29	-1.78
GO:00012	DNA-bind	Molecular	0.18	0.39	Solyc03g1	Solyc03g1	2.90	1.54
GO:00012	DNA-bind	Molecular	0.18	0.39	Solyc05g0	Solyc05g0	0.42	-1.26
GO:00012	DNA-bind	Molecular	0.18	0.39	Solyc05g0	Solyc05g0	1995.76	10.96
GO:00033	amino acid	Biological	0.18	0.39	Solyc03g0	Solyc03g0	0.48	-1.07
GO:00033	amino acid	Biological	0.18	0.39	Solyc04g0	Solyc04g0	0.17	-2.54
GO:00033	amino acid	Biological	0.18	0.39	Solyc01g1	Solyc01g1	0.26	-1.97
GO:00033	amino acid	Biological	0.18	0.39	Solyc11g0	Solyc11g0	0.47	-1.08

GO:00041	serine-type	Molecular	0.18	0.39	Solyc05g0	Solyc05g0	0.24	-2.03
GO:00041	serine-type	Molecular	0.18	0.39	Solyc12g0	Solyc12g0	0.43	-1.22
GO:00041	serine-type	Molecular	0.18	0.39	Solyc04g0	Solyc04g0	0.40	-1.33
GO:00041	serine-type	Molecular	0.18	0.39	Solyc11g0	Solyc11g0	3.66	1.87
GO:00086	hexose tra	Biological	0.19	0.39	Solyc12g0	Solyc12g0	2.45	1.29
GO:00086	hexose tra	Biological	0.19	0.39	Solyc11g0	Solyc11g0	0.36	-1.49
GO:00086	hexose tra	Biological	0.19	0.39	Solyc01g0	Solyc01g0	0.44	-1.18
GO:00069	cellular res	Biological	0.19	0.39	Solyc06g0	Solyc06g0	2.41	1.27
GO:00069	cellular res	Biological	0.19	0.39	Solyc03g0	Solyc03g0	0.08	-3.70
GO:00069	cellular res	Biological	0.19	0.39	Solyc04g0	Solyc04g0	2.19	1.13
GO:00457	positive re	Biological	0.19	0.39	Solyc02g0	Solyc02g0	8.65	3.11
GO:00457	positive re	Biological	0.19	0.39	Solyc04g0	Solyc04g0	2.93	1.55
GO:00457	positive re	Biological	0.19	0.39	Solyc04g0	Solyc04g0	2.18	1.13
GO:00422	response to	Biological	0.19	0.39	Solyc12g0	Solyc12g0	0.28	-1.84
GO:00422	response to	Biological	0.19	0.39	Solyc01g0	Solyc01g0	0.31	-1.69
GO:00039	NAD+ AD	Molecular	0.19	0.39	Solyc11g0	Solyc11g0	0.43	-1.23
GO:00039	NAD+ AD	Molecular	0.19	0.39	Solyc05g0	Solyc05g0	2.10	1.07
GO:00181	peptidyl-ty	Biological	0.19	0.39	Solyc02g0	Solyc02g0	0.47	-1.10
GO:00181	peptidyl-ty	Biological	0.19	0.39	Solyc02g0	Solyc02g0	0.16	-2.63
GO:00517	regulation	Biological	0.19	0.39	Solyc04g0	Solyc04g0	2.02	1.01
GO:00517	regulation	Biological	0.19	0.39	Solyc03g1	Solyc03g1	2.90	1.54
GO:00905	RNA poly	Cellular C	0.19	0.39	Solyc04g0	Solyc04g0	2.02	1.01
GO:00905	RNA poly	Cellular C	0.19	0.39	Solyc03g1	Solyc03g1	2.90	1.54
GO:00083	voltage-ga	Molecular	0.19	0.39	Solyc03g1	Solyc03g1	18.12	4.18
GO:00083	voltage-ga	Molecular	0.19	0.39	Solyc09g0	Solyc09g0	460.47	8.85
GO:00055	calcium io	Molecular	0.19	0.39	Solyc02g0	Solyc02g0	0.35	-1.53
GO:00055	calcium io	Molecular	0.19	0.39	Solyc12g0	Solyc12g0	0.47	-1.08
GO:00055	calcium io	Molecular	0.19	0.39	Solyc01g0	Solyc01g0	0.23	-2.14
GO:00055	calcium io	Molecular	0.19	0.39	Solyc11g0	Solyc11g0	10.65	3.41
GO:00055	calcium io	Molecular	0.19	0.39	Solyc08g0	Solyc08g0	0.47	-1.07
GO:00055	calcium io	Molecular	0.19	0.39	Solyc01g0	Solyc01g0	0.00	-12.11
GO:00055	calcium io	Molecular	0.19	0.39	Solyc06g0	Solyc06g0	2.19	1.13
GO:00055	calcium io	Molecular	0.19	0.39	Solyc10g0	Solyc10g0	0.00	-11.65
GO:00055	calcium io	Molecular	0.19	0.39	Solyc02g0	Solyc02g0	0.45	-1.16
GO:00055	calcium io	Molecular	0.19	0.39	Solyc01g0	Solyc01g0	2.66	1.41
GO:00055	calcium io	Molecular	0.19	0.39	Solyc02g0	Solyc02g0	7.41	2.89
GO:00055	calcium io	Molecular	0.19	0.39	Solyc02g0	Solyc02g0	0.49	-1.03
GO:00055	calcium io	Molecular	0.19	0.39	Solyc09g0	Solyc09g0	2.30	1.20
GO:00055	calcium io	Molecular	0.19	0.39	Solyc04g0	Solyc04g0	0.43	-1.23
GO:00055	calcium io	Molecular	0.19	0.39	Solyc03g0	Solyc03g0	4.23	2.08
GO:00055	calcium io	Molecular	0.19	0.39	Solyc06g0	Solyc06g0	3.20	1.68
GO:00055	calcium io	Molecular	0.19	0.39	Solyc01g0	Solyc01g0	0.20	-2.35
GO:00455	phenylalan	Molecular	0.20	0.39	Solyc09g0	Solyc09g0	0.08	-3.59
GO:00097	regulation	Biological	0.20	0.39	Solyc09g0	Solyc09g0	0.07	-3.81
GO:00065	glutamate	Biological	0.20	0.39	Solyc03g0	Solyc03g0	2.69	1.43
GO:00166	oxidoreduc	Molecular	0.20	0.39	Solyc03g0	Solyc03g0	2.69	1.43
GO:00064	protein AD	Biological	0.20	0.39	Solyc11g0	Solyc11g0	0.43	-1.23
GO:00096	abscisic ac	Biological	0.20	0.39	Solyc07g0	NCED1	0.30	-1.72
GO:00486	anther dev	Biological	0.20	0.39	Solyc07g0	NCED1	0.30	-1.72
GO:00046	phospholip	Molecular	0.20	0.39	Solyc01g0	Solyc01g0	2.06	1.04
GO:00226	cytosolic r	Cellular C	0.20	0.39	Solyc05g0	Solyc05g0	2.02	1.01

GO:00452	pyruvate d	Cellular Co	0.20	0.39	Solyc05g0	Solyc05g0	2.02	1.01
GO:00088	crossover j	Molecular	0.20	0.39	Solyc01g0	Solyc01g0	2.93	1.55
GO:00057	Golgi stacl	Cellular Co	0.20	0.39	Solyc09g0	Solyc09g0	0.44	-1.17
GO:00099	alternative	Molecular	0.20	0.39	Solyc08g0	Solyc08g0	0.19	-2.37
GO:01027	ubiquinol:(	Molecular	0.20	0.39	Solyc08g0	Solyc08g0	0.19	-2.37
GO:00099	anther deh	Biological	0.20	0.39	Solyc04g0	Solyc04g0	0.48	-1.06
GO:00103	cellulose s	Cellular Co	0.20	0.39	Solyc04g0	Solyc04g0	0.48	-1.06
GO:00488	pollen tube	Biological	0.20	0.39	Solyc04g0	Solyc04g0	0.48	-1.06
GO:00046	1,4-alpha-(	Molecular	0.20	0.39	Solyc05g0	Solyc05g0	0.37	-1.44
GO:00081	glycogen p	Molecular	0.20	0.39	Solyc05g0	Solyc05g0	0.37	-1.44
GO:00700	serine-type	Molecular	0.20	0.39	Solyc04g0	Solyc04g0	0.18	-2.44
GO:00045	inositol-3-]	Molecular	0.20	0.39	Solyc05g0	Solyc05g0	0.45	-1.15
GO:00101	vitamin E l	Biological	0.20	0.39	Solyc08g0	Solyc08g0	2.10	1.07
GO:00040	3-chloroall	Molecular	0.20	0.39	Solyc01g0	Solyc01g0	0.41	-1.30
GO:00350	positive re	Biological	0.20	0.39	Solyc06g0	Solyc06g0	2.41	1.27
GO:00350	negative re	Biological	0.20	0.39	Solyc06g0	Solyc06g0	2.41	1.27
GO:00047	ribose-5-pl	Molecular	0.20	0.39	Solyc05g0	Solyc05g0	0.44	-1.18
GO:00517	ent-kauren	Molecular	0.20	0.39	Solyc01g0	Solyc01g0	0.29	-1.77
GO:00038	1-acylglyc	Molecular	0.20	0.39	Solyc11g0	Solyc11g0	6.34	2.66
GO:00084	3'(2'),5'-bis	Molecular	0.20	0.39	Solyc02g0	Solyc02g0	0.49	-1.03
GO:00044	hydroxyme	Molecular	0.20	0.39	Solyc12g0	Solyc12g0	0.43	-1.20
GO:00060	acetyl-CoA	Biological	0.20	0.39	Solyc12g0	Solyc12g0	0.43	-1.20
GO:00472	inositol 3-a	Molecular	0.20	0.39	Solyc01g0	Solyc01g0	0.45	-1.15
GO:00717	nitrogen co	Biological	0.20	0.39	Solyc04g0	Solyc04g0	0.48	-1.07
GO:00056	nuclear ori	Cellular Co	0.20	0.39	Solyc05g0	Solyc05g0	2.55	1.35
GO:00312	extrinsic co	Cellular Co	0.20	0.39	Solyc06g0	Solyc06g0	0.08	-3.58
GO:00103	raffinose fa	Biological	0.20	0.39	Solyc01g0	Solyc01g0	0.50	-1.01
GO:00070	mitotic chr	Biological	0.20	0.39	Solyc07g0	Solyc07g0	2.25	1.17
GO:00040	aspartate k	Molecular	0.20	0.39	Solyc06g0	Solyc06g0	0.30	-1.75
GO:00000	DNA repli	Biological	0.20	0.39	Solyc04g0	Solyc04g0	2.19	1.13
GO:00431	replication	Biological	0.20	0.39	Solyc04g0	Solyc04g0	2.19	1.13
GO:00095	embryo sac	Biological	0.20	0.39	Solyc11g0	Solyc11g0	2.39	1.25
GO:00047	L-threonin	Molecular	0.20	0.39	Solyc09g0	TD2	4.53	2.18
GO:00065	threonine c	Biological	0.20	0.39	Solyc09g0	TD2	4.53	2.18
GO:00421	nitrate assi	Biological	0.20	0.39	Solyc06g0	Solyc06g0	0.37	-1.45
GO:00451	single-stra	Molecular	0.20	0.39	Solyc02g0	Solyc02g0	2.19	1.13
GO:00482	flap endon	Molecular	0.20	0.39	Solyc02g0	Solyc02g0	2.19	1.13
GO:00519	double-stra	Molecular	0.20	0.39	Solyc02g0	Solyc02g0	2.19	1.13
GO:00170	structural c	Molecular	0.20	0.40	Solyc11g0	Solyc11g0	8.11	3.02
GO:00170	structural c	Molecular	0.20	0.40	Solyc02g0	Solyc02g0	4.79	2.26
GO:00170	structural c	Molecular	0.20	0.40	Solyc02g0	Solyc02g0	0.25	-1.99
GO:00090	electron tra	Molecular	0.21	0.41	Solyc01g1	Solyc01g1	0.46	-1.13
GO:00090	electron tra	Molecular	0.21	0.41	Solyc04g0	Solyc04g0	0.41	-1.27
GO:00090	electron tra	Molecular	0.21	0.41	Solyc11g0	Solyc11g0	9.10	3.19
GO:00090	electron tra	Molecular	0.21	0.41	Solyc11g0	Solyc11g0	0.28	-1.82
GO:00090	electron tra	Molecular	0.21	0.41	Solyc07g0	Solyc07g0	7.98	3.00
GO:00090	electron tra	Molecular	0.21	0.41	Solyc01g0	Solyc01g0	0.38	-1.39
GO:00090	electron tra	Molecular	0.21	0.41	Solyc03g1	Solyc03g1	0.00	-12.84
GO:00090	electron tra	Molecular	0.21	0.41	Solyc10g0	Solyc10g0	0.46	-1.12
GO:00090	electron tra	Molecular	0.21	0.41	Solyc08g0	Solyc08g0	2.04	1.03
GO:00090	electron tra	Molecular	0.21	0.41	Solyc07g0	Solyc07g0	0.12	-3.04

GO:00090	electron tra	Molecular	0.21	0.41	Solyc07g0	Solyc07g0	1759.77	10.78
GO:00507	regulation	Biological	0.21	0.41	Solyc06g0	Solyc06g0	0.44	-1.19
GO:00507	regulation	Biological	0.21	0.41	Solyc03g1	Solyc03g1	2479.17	11.28
GO:00096	cytokinin b	Biological	0.21	0.41	Solyc08g0	Solyc08g0	0.50	-1.01
GO:00096	cytokinin b	Biological	0.21	0.41	Solyc01g0	Solyc01g0	0.42	-1.26
GO:00066	protein targ	Biological	0.21	0.41	Solyc05g0	Solyc05g0	0.38	-1.40
GO:00066	protein targ	Biological	0.21	0.41	Solyc02g0	Solyc02g0	0.41	-1.30
GO:00182	peptidyl-L	Biological	0.21	0.41	Solyc05g0	Solyc05g0	0.38	-1.40
GO:00182	peptidyl-L	Biological	0.21	0.41	Solyc02g0	Solyc02g0	0.41	-1.30
GO:00452	proton-tran	Cellular Co	0.21	0.41	Solyc06g0	Solyc06g0	3.71	1.89
GO:00452	proton-tran	Cellular Co	0.21	0.41	Solyc11g0	Solyc11g0	0.33	-1.60
GO:00450	actin nucle	Biological	0.21	0.41	Solyc06g0	Solyc06g0	0.47	-1.09
GO:00450	actin nucle	Biological	0.21	0.41	Solyc01g0	Solyc01g0	0.11	-3.14
GO:00047	protein tyro	Molecular	0.21	0.41	Solyc04g0	Solyc04g0	0.21	-2.26
GO:00047	protein tyro	Molecular	0.21	0.41	Solyc04g0	Solyc04g0	0.35	-1.51
GO:00152	antiporter a	Molecular	0.21	0.41	Solyc04g0	Solyc04g0	0.19	-2.40
GO:00152	antiporter a	Molecular	0.21	0.41	Solyc03g1	Solyc03g1	0.47	-1.08
GO:00152	antiporter a	Molecular	0.21	0.41	Solyc02g0	Solyc02g0	0.33	-1.61
GO:00152	antiporter a	Molecular	0.21	0.41	Solyc05g0	Solyc05g0	0.06	-4.14
GO:00152	antiporter a	Molecular	0.21	0.41	Solyc07g0	Solyc07g0	0.00	-9.97
GO:00152	antiporter a	Molecular	0.21	0.41	Solyc08g0	Solyc08g0	3.30	1.72
GO:00056	nuclear po	Cellular Co	0.21	0.41	Solyc02g0	Solyc02g0	4.79	2.26
GO:00056	nuclear po	Cellular Co	0.21	0.41	Solyc02g0	Solyc02g0	0.25	-1.99
GO:00056	nuclear po	Cellular Co	0.21	0.41	Solyc02g0	Solyc02g0	5.07	2.34
GO:00300	actin cytos	Biological	0.21	0.41	Solyc06g0	Solyc06g0	0.47	-1.09
GO:00300	actin cytos	Biological	0.21	0.41	Solyc01g1	Solyc01g1	0.44	-1.20
GO:00300	actin cytos	Biological	0.21	0.41	Solyc01g0	Solyc01g0	0.11	-3.14
GO:00355	intracellula	Biological	0.22	0.43	Solyc01g0	Solyc01g0	0.00	-12.11
GO:00355	intracellula	Biological	0.22	0.43	Solyc06g0	Solyc06g0	2.69	1.43
GO:00355	intracellula	Biological	0.22	0.43	Solyc12g0	Solyc12g0	2.48	1.31
GO:00355	intracellula	Biological	0.22	0.43	Solyc06g0	Solyc06g0	0.48	-1.07
GO:00355	intracellula	Biological	0.22	0.43	Solyc01g0	Solyc01g0	2.66	1.41
GO:00355	intracellula	Biological	0.22	0.43	Solyc02g0	Solyc02g0	0.49	-1.03
GO:00355	intracellula	Biological	0.22	0.43	Solyc05g0	Solyc05g0	0.00	-8.91
GO:00086	carbohydra	Biological	0.23	0.44	Solyc12g0	Solyc12g0	2.45	1.29
GO:00086	carbohydra	Biological	0.23	0.44	Solyc01g0	Solyc01g0	0.44	-1.18
GO:00086	carbohydra	Biological	0.23	0.44	Solyc01g0	Solyc01g0	0.50	-1.01
GO:00167	transferase	Molecular	0.23	0.44	Solyc09g0	Solyc09g0	0.01	-7.13
GO:00167	transferase	Molecular	0.23	0.44	Solyc05g0	Solyc05g0	0.01	-6.95
GO:00167	transferase	Molecular	0.23	0.44	Solyc09g0	Solyc09g0	0.07	-3.81
GO:00167	transferase	Molecular	0.23	0.44	Solyc01g0	Solyc01g0	0.31	-1.69
GO:00167	transferase	Molecular	0.23	0.44	Solyc12g0	Solyc12g0	0.40	-1.33
GO:00167	transferase	Molecular	0.23	0.44	Solyc02g0	Solyc02g0	0.47	-1.10
GO:00167	transferase	Molecular	0.23	0.44	Solyc04g0	Solyc04g0	2.15	1.11
GO:00167	transferase	Molecular	0.23	0.44	Solyc04g0	Solyc04g0	2.56	1.36
GO:00167	transferase	Molecular	0.23	0.44	Solyc08g0	Solyc08g0	2.10	1.07
GO:00167	transferase	Molecular	0.23	0.44	Solyc01g0	XTH1	0.31	-1.69
GO:00167	transferase	Molecular	0.23	0.44	Solyc12g0	Solyc12g0	4.32	2.11
GO:00167	transferase	Molecular	0.23	0.44	Solyc01g0	Solyc01g0	4.27	2.09
GO:00167	transferase	Molecular	0.23	0.44	Solyc06g0	Solyc06g0	0.49	-1.02
GO:00167	transferase	Molecular	0.23	0.44	Solyc07g0	Solyc07g0	2.24	1.16

GO:00167	transferase	Molecular	0.23	0.44	Solyc03g0	Solyc03g0	4.24	2.08
GO:00167	transferase	Molecular	0.23	0.44	Solyc07g0	Solyc07g0	0.02	-5.66
GO:00167	transferase	Molecular	0.23	0.44	Solyc12g0	Solyc12g0	5.61	2.49
GO:00167	transferase	Molecular	0.23	0.44	Solyc11g0	Solyc11g0	2.55	1.35
GO:00167	transferase	Molecular	0.23	0.44	Solyc01g0	Solyc01g0	0.42	-1.26
GO:00167	transferase	Molecular	0.23	0.44	Solyc01g0	Solyc01g0	2.66	1.41
GO:00167	transferase	Molecular	0.23	0.44	Solyc07g0	Solyc07g0	2.18	1.12
GO:00167	transferase	Molecular	0.23	0.44	Solyc07g0	Solyc07g0	0.04	-4.66
GO:00167	transferase	Molecular	0.23	0.44	Solyc02g0	Solyc02g0	0.16	-2.63
GO:00167	transferase	Molecular	0.23	0.44	Solyc06g0	Solyc06g0	0.48	-1.07
GO:00167	transferase	Molecular	0.23	0.44	Solyc11g0	Solyc11g0	0.38	-1.39
GO:00167	transferase	Molecular	0.23	0.44	Solyc08g0	Solyc08g0	3.05	1.61
GO:00167	transferase	Molecular	0.23	0.44	Solyc06g0	Solyc06g0	0.00	-9.59
GO:00167	transferase	Molecular	0.23	0.44	Solyc08g0	Solyc08g0	2.74	1.45
GO:00167	transferase	Molecular	0.23	0.44	Solyc09g0	Solyc09g0	0.00	-8.38
GO:00167	transferase	Molecular	0.23	0.44	Solyc02g0	Solyc02g0	0.20	-2.30
GO:00068	amino acid	Biological	0.23	0.44	Solyc04g0	Solyc04g0	0.17	-2.54
GO:00068	amino acid	Biological	0.23	0.44	Solyc11g0	Solyc11g0	0.47	-1.08
GO:00197	protein-cys	Molecular	0.23	0.44	Solyc05g0	Solyc05g0	0.38	-1.40
GO:00197	protein-cys	Molecular	0.23	0.44	Solyc02g0	Solyc02g0	0.41	-1.30
GO:00801	regulation	Biological	0.23	0.44	Solyc06g0	Solyc06g0	0.41	-1.28
GO:00801	regulation	Biological	0.23	0.44	Solyc06g0	Solyc06g0	0.39	-1.36
GO:00304	ubiquitin-c	Biological	0.23	0.44	Solyc06g0	Solyc06g0	6.40	2.68
GO:00304	ubiquitin-c	Biological	0.23	0.44	Solyc01g0	Solyc01g0	0.32	-1.64
GO:00097	detection c	Biological	0.24	0.44	Solyc02g0	Solyc02g0	0.23	-2.13
GO:00040	arylformar	Molecular	0.24	0.44	Solyc10g0	Solyc10g0	0.34	-1.55
GO:00194	tryptophan	Biological	0.24	0.44	Solyc10g0	Solyc10g0	0.34	-1.55
GO:00098	blue light p	Molecular	0.24	0.44	Solyc12g0	Solyc12g0	0.32	-1.65
GO:00091	nucleoside	Biological	0.24	0.44	Solyc09g0	Solyc09g0	3.87	1.95
GO:00457	negative re	Biological	0.24	0.44	Solyc06g0	Solyc06g0	2.41	1.27
GO:00198	oxygen bir	Molecular	0.24	0.44	Solyc03g0	Solyc03g0	0.29	-1.79
GO:00090	pentose-ph	Biological	0.24	0.44	Solyc05g0	Solyc05g0	0.44	-1.18
GO:00067	sulfur com	Biological	0.24	0.44	Solyc02g0	Solyc02g0	0.49	-1.03
GO:00102	maintenan	Biological	0.24	0.44	Solyc05g0	Solyc05g0	2.31	1.21
GO:00192	kinase inhi	Molecular	0.24	0.44	Solyc04g0	Solyc04g0	0.18	-2.51
GO:00801	regulation	Biological	0.24	0.44	Solyc03g1	Solyc03g1	0.37	-1.42
GO:00325	plastid trar	Biological	0.24	0.44	Solyc05g0	Solyc05g0	0.42	-1.25
GO:00420	DNA endo	Biological	0.24	0.44	Solyc03g1	Solyc03g1	2.90	1.54
GO:19024	chloride tr	Biological	0.24	0.44	Solyc02g0	Solyc02g0	0.31	-1.67
GO:00150	molybdate	Molecular	0.24	0.44	Solyc03g1	Solyc03g1	0.48	-1.06
GO:00156	molybdate	Biological	0.24	0.44	Solyc03g1	Solyc03g1	0.48	-1.06
GO:00096	response to	Biological	0.24	0.44	Solyc09g0	TD2	4.53	2.18
GO:00171	5'-flap end	Molecular	0.24	0.44	Solyc02g0	Solyc02g0	2.19	1.13
GO:00062	RNA-depe	Biological	0.24	0.44	Solyc09g0	Solyc09g0	6.02	2.59
GO:00152	sterol trans	Molecular	0.24	0.44	Solyc07g0	Solyc07g0	638.37	9.32
GO:00329	sterol bind	Molecular	0.24	0.44	Solyc07g0	Solyc07g0	638.37	9.32
GO:00100	xylem dev	Biological	0.24	0.44	Solyc09g0	Solyc09g0	0.49	-1.04
GO:00056	extracellul	Cellular C	0.24	0.44	Solyc09g0	Solyc09g0	0.01	-7.14
GO:00056	extracellul	Cellular C	0.24	0.44	Solyc11g0	Solyc11g0	0.46	-1.12
GO:00056	extracellul	Cellular C	0.24	0.44	Solyc02g0	CHI3	0.24	-2.08
GO:00056	extracellul	Cellular C	0.24	0.44	Solyc02g0	Solyc02g0	2.11	1.08

GO:00199	protein kin	Molecular	0.24	0.44	Solyc06g0	Solyc06g0	0.44	-1.19
GO:00199	protein kin	Molecular	0.24	0.44	Solyc02g0	Solyc02g0	8.65	3.11
GO:00199	protein kin	Molecular	0.24	0.44	Solyc04g0	Solyc04g0	2.93	1.55
GO:00199	protein kin	Molecular	0.24	0.44	Solyc04g0	Solyc04g0	2.18	1.13
GO:00508	response to	Biological	0.24	0.45	Solyc09g0	Solyc09g0	0.14	-2.81
GO:00508	response to	Biological	0.24	0.45	Solyc12g0	Solyc12g0	2.45	1.29
GO:00508	response to	Biological	0.24	0.45	Solyc09g0	Solyc09g0	0.18	-2.47
GO:00508	response to	Biological	0.24	0.45	Solyc11g0	Solyc11g0	0.36	-1.49
GO:00508	response to	Biological	0.24	0.45	Solyc06g0	Solyc06g0	0.37	-1.45
GO:00441	protein fol	Molecular	0.25	0.45	Solyc10g0	Solyc10g0	0.47	-1.09
GO:00441	protein fol	Molecular	0.25	0.45	Solyc03g1	Solyc03g1	0.00	-11.76
GO:00462	lignin catal	Biological	0.25	0.45	Solyc05g0	Solyc05g0	0.31	-1.67
GO:00462	lignin catal	Biological	0.25	0.45	Solyc02g0	Solyc02g0	0.00	-8.25
GO:00527	hydroquino	Molecular	0.25	0.45	Solyc05g0	Solyc05g0	0.31	-1.67
GO:00527	hydroquino	Molecular	0.25	0.45	Solyc02g0	Solyc02g0	0.00	-8.25
GO:00152	solute:prot	Molecular	0.26	0.47	Solyc08g0	Solyc08g0	0.26	-1.92
GO:00152	solute:prot	Molecular	0.26	0.47	Solyc08g0	Solyc08g0	0.00	-11.90
GO:00152	solute:prot	Molecular	0.26	0.47	Solyc08g0	Solyc08g0	0.06	-4.18
GO:00082	positive re	Biological	0.26	0.47	Solyc02g0	Solyc02g0	8.65	3.11
GO:00082	positive re	Biological	0.26	0.47	Solyc04g0	Solyc04g0	2.93	1.55
GO:00082	positive re	Biological	0.26	0.47	Solyc04g0	Solyc04g0	2.18	1.13
GO:00068	ion transpo	Biological	0.26	0.47	Solyc01g1	Solyc01g1	0.43	-1.23
GO:00068	ion transpo	Biological	0.26	0.47	Solyc07g0	Solyc07g0	0.50	-1.01
GO:00068	ion transpo	Biological	0.26	0.47	Solyc11g0	Solyc11g0	2.48	1.31
GO:00068	ion transpo	Biological	0.26	0.47	Solyc11g0	Solyc11g0	0.33	-1.60
GO:00068	ion transpo	Biological	0.26	0.47	Solyc08g0	Solyc08g0	0.40	-1.32
GO:00068	ion transpo	Biological	0.26	0.47	Solyc12g0	Solyc12g0	0.48	-1.07
GO:00704	NAD+ bin	Molecular	0.27	0.47	Solyc08g0	Solyc08g0	0.21	-2.27
GO:00704	NAD+ bin	Molecular	0.27	0.47	Solyc09g0	Solyc09g0	2.89	1.53
GO:00310	heat shock	Molecular	0.27	0.47	Solyc10g0	Solyc10g0	0.47	-1.09
GO:00310	heat shock	Molecular	0.27	0.47	Solyc03g1	Solyc03g1	0.00	-11.76
GO:00166	oxidoreduc	Molecular	0.27	0.47	Solyc01g0	Solyc01g0	0.32	-1.62
GO:00166	oxidoreduc	Molecular	0.27	0.47	Solyc05g0	Solyc05g0	0.21	-2.26
GO:00048	endopeptic	Molecular	0.27	0.47	Solyc11g0	Solyc11g0	4.18	2.06
GO:00048	endopeptic	Molecular	0.27	0.47	Solyc07g0	Solyc07g0	2.42	1.28
GO:00302	enzyme reg	Molecular	0.27	0.47	Solyc01g0	Solyc01g0	7223.16	12.82
GO:00302	enzyme reg	Molecular	0.27	0.47	Solyc06g0	Solyc06g0	3.20	1.68
GO:00046	calmodulin	Molecular	0.27	0.47	Solyc01g0	Solyc01g0	0.00	-12.11
GO:00046	calmodulin	Molecular	0.27	0.47	Solyc01g0	Solyc01g0	2.66	1.41
GO:00046	calmodulin	Molecular	0.27	0.47	Solyc02g0	Solyc02g0	0.49	-1.03
GO:00099	calcium-de	Molecular	0.27	0.47	Solyc01g0	Solyc01g0	0.00	-12.11
GO:00099	calcium-de	Molecular	0.27	0.47	Solyc01g0	Solyc01g0	2.66	1.41
GO:00099	calcium-de	Molecular	0.27	0.47	Solyc02g0	Solyc02g0	0.49	-1.03
GO:00166	CCAAT-b	Cellular Co	0.27	0.47	Solyc05g0	Solyc05g0	0.29	-1.78
GO:00166	CCAAT-b	Cellular Co	0.27	0.47	Solyc05g0	Solyc05g0	0.42	-1.26
GO:00166	CCAAT-b	Cellular Co	0.27	0.47	Solyc05g0	Solyc05g0	1995.76	10.96
GO:00310	gene silenc	Biological	0.27	0.47	Solyc01g0	Solyc01g0	0.00	-10.39
GO:00310	gene silenc	Biological	0.27	0.47	Solyc06g0	Solyc06g0	4.37	2.13
GO:00310	gene silenc	Biological	0.27	0.47	Solyc01g0	Solyc01g0	4163.59	12.02
GO:00009	RNA polym	Molecular	0.28	0.47	Solyc09g0	Solyc09g0	0.48	-1.06
GO:00009	RNA polym	Molecular	0.28	0.47	Solyc10g0	Solyc10g0	0.00	-13.06

GO:00009	RNA poly	Molecular	0.28	0.47	Solyc04g0	Solyc04g0	2.02	1.01
GO:00009	RNA poly	Molecular	0.28	0.47	Solyc07g0	Solyc07g0	0.07	-3.80
GO:00009	RNA poly	Molecular	0.28	0.47	Solyc03g1	Solyc03g1	2.90	1.54
GO:00801	gene silenc	Biological	0.28	0.47	Solyc06g0	Solyc06g0	4.08	2.03
GO:00801	gene silenc	Biological	0.28	0.47	Solyc01g0	Solyc01g0	3.11	1.64
GO:00801	gene silenc	Biological	0.28	0.47	Solyc06g0	Solyc06g0	4.37	2.13
GO:00801	gene silenc	Biological	0.28	0.47	Solyc01g0	Solyc01g0	4163.59	12.02
GO:00454	gibberellin	Biological	0.28	0.47	Solyc07g0	Solyc07g0	0.17	-2.54
GO:00526	C-19 gibbe	Molecular	0.28	0.47	Solyc07g0	Solyc07g0	0.17	-2.54
GO:00166	oxidoreduc	Molecular	0.28	0.47	Solyc03g0	Solyc03g0	2.69	1.43
GO:00465	alpha-L-ar	Molecular	0.28	0.47	Solyc01g1	Solyc01g1	2.32	1.21
GO:00425	hyperosmo	Biological	0.28	0.47	Solyc07g0	NCED1	0.30	-1.72
GO:00168	hydro-lyas	Molecular	0.28	0.47	Solyc09g0	Solyc09g0	0.01	-6.18
GO:00000	ribosomal	Biological	0.28	0.47	Solyc11g0	Solyc11g0	8.11	3.02
GO:00343	lipid dropl	Biological	0.28	0.47	Solyc04g0	Solyc04g0	0.46	-1.13
GO:00512	chromosor	Biological	0.28	0.47	Solyc05g0	Solyc05g0	2.04	1.03
GO:00166	hydroxypy	Molecular	0.28	0.47	Solyc12g0	Solyc12g0	0.34	-1.56
GO:00302	glyoxylate	Molecular	0.28	0.47	Solyc12g0	Solyc12g0	0.34	-1.56
GO:00705	BRCA1-A	Cellular C	0.28	0.47	Solyc06g0	Solyc06g0	2.41	1.27
GO:19015	organonitr	Biological	0.28	0.47	Solyc10g0	Solyc10g0	0.38	-1.40
GO:00016	cellular gl	Biological	0.28	0.47	Solyc11g0	Solyc11g0	0.33	-1.59
GO:00043	glucokinas	Molecular	0.28	0.47	Solyc11g0	Solyc11g0	0.33	-1.59
GO:00043	hexokinase	Molecular	0.28	0.47	Solyc11g0	Solyc11g0	0.33	-1.59
GO:00055	glucose bi	Molecular	0.28	0.47	Solyc11g0	Solyc11g0	0.33	-1.59
GO:00191	mannokina	Molecular	0.28	0.47	Solyc11g0	Solyc11g0	0.33	-1.59
GO:00103	lateral root	Biological	0.28	0.47	Solyc11g0	Solyc11g0	0.47	-1.08
GO:00101	farnesyl di	Biological	0.28	0.47	Solyc12g0	Solyc12g0	0.43	-1.20
GO:00040	branched-c	Molecular	0.28	0.47	Solyc03g0	Solyc03g0	2.49	1.31
GO:00090	branched-c	Biological	0.28	0.47	Solyc03g0	Solyc03g0	2.49	1.31
GO:00168	carbon-sul	Molecular	0.28	0.47	Solyc02g0	Solyc02g0	40.96	5.36
GO:00702	N-acylpho	Molecular	0.28	0.47	Solyc08g0	Solyc08g0	0.47	-1.07
GO:00354	phosphate	Biological	0.28	0.47	Solyc03g1	Solyc03g1	0.47	-1.08
GO:00477	chlorophyl	Molecular	0.28	0.47	Solyc09g0	Solyc09g0	2049.83	11.00
GO:00060	glycerol-3-	Biological	0.28	0.47	Solyc03g1	Solyc03g1	0.34	-1.57
GO:00903	regulation	Biological	0.28	0.47	Solyc12g0	Solyc12g0	0.48	-1.07
GO:00096	response to	Biological	0.28	0.47	Solyc06g0	Solyc06g0	0.04	-4.72
GO:00513	spindle pol	Biological	0.28	0.47	Solyc06g0	Solyc06g0	3.20	1.68
GO:00096	entrainmer	Biological	0.28	0.47	Solyc06g0	Solyc06g0	0.40	-1.30
GO:00090	aromatic a	Biological	0.28	0.47	Solyc01g0	Solyc01g0	0.23	-2.09
GO:00324	demethyla	Molecular	0.28	0.47	Solyc06g0	Solyc06g0	0.45	-1.14
GO:00469	pectinester	Molecular	0.29	0.48	Solyc03g1	Solyc03g1	0.37	-1.42
GO:00469	pectinester	Molecular	0.29	0.48	Solyc03g0	Solyc03g0	0.28	-1.83
GO:00469	pectinester	Molecular	0.29	0.48	Solyc01g0	Solyc01g0	4.89	2.29
GO:00000	regulation	Biological	0.29	0.48	Solyc02g0	Solyc02g0	8.65	3.11
GO:00000	regulation	Biological	0.29	0.48	Solyc04g0	Solyc04g0	2.93	1.55
GO:00000	regulation	Biological	0.29	0.48	Solyc04g0	Solyc04g0	2.18	1.13
GO:00003	cyclin-dep	Cellular C	0.29	0.48	Solyc02g0	Solyc02g0	8.65	3.11
GO:00003	cyclin-dep	Cellular C	0.29	0.48	Solyc04g0	Solyc04g0	2.93	1.55
GO:00003	cyclin-dep	Cellular C	0.29	0.48	Solyc04g0	Solyc04g0	2.18	1.13
GO:00165	cyclin-dep	Molecular	0.29	0.48	Solyc02g0	Solyc02g0	8.65	3.11
GO:00165	cyclin-dep	Molecular	0.29	0.48	Solyc04g0	Solyc04g0	2.93	1.55

GO:00165	cyclin-dep	Molecular	0.29	0.48	Solyc04g0	Solyc04g0	2.18	1.13
GO:00001	negative re	Biological	0.29	0.49	Solyc04g0	Solyc04g0	2.02	1.01
GO:00001	negative re	Biological	0.29	0.49	Solyc03g1	Solyc03g1	2.90	1.54
GO:00515	4 iron, 4 su	Molecular	0.30	0.50	Solyc09g0	Solyc09g0	0.01	-6.18
GO:00515	4 iron, 4 su	Molecular	0.30	0.50	Solyc12g0	Solyc12g0	0.34	-1.57
GO:00515	4 iron, 4 su	Molecular	0.30	0.50	Solyc06g0	Solyc06g0	0.08	-3.69
GO:00515	4 iron, 4 su	Molecular	0.30	0.50	Solyc07g0	Solyc07g0	2.89	1.53
GO:00515	4 iron, 4 su	Molecular	0.30	0.50	Solyc03g0	Solyc03g0	0.34	-1.57
GO:00066	steroid bio	Biological	0.31	0.51	Solyc02g0	Solyc02g0	0.14	-2.87
GO:00066	steroid bio	Biological	0.31	0.51	Solyc01g1	Solyc01g1	0.28	-1.84
GO:00045	endonucle	Molecular	0.31	0.51	Solyc02g0	Solyc02g0	2.65	1.40
GO:00045	endonucle	Molecular	0.31	0.51	Solyc05g0	Solyc05g0	0.48	-1.07
GO:00198	extrinsic c	Cellular C	0.31	0.51	Solyc02g0	Solyc02g0	0.35	-1.53
GO:00198	extrinsic c	Cellular C	0.31	0.51	Solyc10g0	Solyc10g0	0.00	-11.65
GO:00098	plant-type	Biological	0.31	0.51	Solyc04g0	Solyc04g0	0.48	-1.06
GO:00098	plant-type	Biological	0.31	0.51	Solyc07g0	Solyc07g0	0.44	-1.18
GO:00102	cellulose n	Biological	0.31	0.51	Solyc04g0	Solyc04g0	0.48	-1.06
GO:00102	cellulose n	Biological	0.31	0.51	Solyc03g1	Solyc03g1	0.00	-12.22
GO:00102	brassinoste	Biological	0.31	0.51	Solyc02g0	Solyc02g0	0.36	-1.49
GO:00102	brassinoste	Biological	0.31	0.51	Solyc01g0	Solyc01g0	0.29	-1.77
GO:00060	glucose me	Biological	0.31	0.51	Solyc02g0	Solyc02g0	0.46	-1.12
GO:00060	glucose me	Biological	0.31	0.51	Solyc12g0	Solyc12g0	0.13	-2.93
GO:00055	copper ion	Molecular	0.31	0.51	Solyc02g0	Solyc02g0	0.23	-2.13
GO:00055	copper ion	Molecular	0.31	0.51	Solyc08g0	Solyc08g0	2.04	1.03
GO:00055	copper ion	Molecular	0.31	0.51	Solyc02g0	Solyc02g0	6.42	2.68
GO:00055	copper ion	Molecular	0.31	0.51	Solyc01g0	Solyc01g0	0.18	-2.51
GO:00055	copper ion	Molecular	0.31	0.51	Solyc05g0	Solyc05g0	0.31	-1.67
GO:00055	copper ion	Molecular	0.31	0.51	Solyc02g0	Solyc02g0	0.00	-8.25
GO:00066	fatty acid b	Biological	0.31	0.51	Solyc11g0	AOS2	0.12	-3.01
GO:00066	fatty acid b	Biological	0.31	0.51	Solyc01g0	Solyc01g0	4.36	2.12
GO:00066	fatty acid b	Biological	0.31	0.51	Solyc05g0	Solyc05g0	2.01	1.01
GO:00066	fatty acid b	Biological	0.31	0.51	Solyc07g0	OPR3	0.47	-1.10
GO:00066	fatty acid b	Biological	0.31	0.51	Solyc12g0	Solyc12g0	10.12	3.34
GO:00066	fatty acid b	Biological	0.31	0.51	Solyc09g0	Solyc09g0	8.52	3.09
GO:00168	hydrolase a	Molecular	0.32	0.51	Solyc02g0	Solyc02g0	2.65	1.40
GO:00098	auxin meta	Biological	0.32	0.51	Solyc06g0	Solyc06g0	3.24	1.69
GO:00090	xylan 1,4-b	Molecular	0.32	0.51	Solyc01g1	Solyc01g1	2.32	1.21
GO:00161	xanthophy	Biological	0.32	0.51	Solyc07g0	NCED1	0.30	-1.72
GO:00044	malic enzy	Molecular	0.32	0.51	Solyc12g0	Solyc12g0	0.42	-1.25
GO:00044	malate deh	Molecular	0.32	0.51	Solyc12g0	Solyc12g0	0.42	-1.25
GO:00000	ribosomal	Biological	0.32	0.51	Solyc11g0	Solyc11g0	8.11	3.02
GO:01406	ATP-depen	Molecular	0.32	0.51	Solyc10g0	Solyc10g0	0.47	-1.09
GO:00060	inositol bio	Biological	0.32	0.51	Solyc05g0	Solyc05g0	0.45	-1.15
GO:00062	'de novo' p	Biological	0.32	0.51	Solyc01g0	Solyc01g0	0.29	-1.78
GO:00001	3'-5'-exorib	Molecular	0.32	0.51	Solyc06g0	Solyc06g0	0.30	-1.75
GO:00474	nucleoside	Molecular	0.32	0.51	Solyc09g0	Solyc09g0	3.87	1.95
GO:00099	polarity sp	Biological	0.32	0.51	Solyc08g0	Solyc08g0	0.39	-1.34
GO:00527	pectin acet	Molecular	0.32	0.51	Solyc01g1	Solyc01g1	0.41	-1.30
GO:00044	phosphatid	Molecular	0.32	0.51	Solyc06g0	Solyc06g0	2.69	1.43
GO:00063	DNA-temp	Biological	0.32	0.51	Solyc04g0	Solyc04g0	0.48	-1.05
GO:00092	glycolipid	Biological	0.32	0.51	Solyc07g0	Solyc07g0	0.34	-1.55

GO:00068	cellular iro	Biological	0.32	0.51	Solyc07g0	Solyc07g0	2.89	1.53
GO:00058	cytoplasmi	Cellular C	0.32	0.51	Solyc03g1	Solyc03g1	0.35	-1.51
GO:00455	dynein inte	Molecular	0.32	0.51	Solyc03g1	Solyc03g1	0.35	-1.51
GO:00519	dynein ligh	Molecular	0.32	0.51	Solyc03g1	Solyc03g1	0.35	-1.51
GO:20005	obsolete p	Biological	0.32	0.51	Solyc03g1	Solyc03g1	0.35	-1.51
GO:00487	root hair el	Biological	0.32	0.51	Solyc12g0	Solyc12g0	0.48	-1.07
GO:00063	homing of	Biological	0.32	0.51	Solyc09g0	Solyc09g0	6.02	2.59
GO:00168	endoribonu	Molecular	0.32	0.51	Solyc10g0	Solyc10g0	476.24	8.90
GO:00068	cellular ion	Biological	0.32	0.51	Solyc09g0	Solyc09g0	460.47	8.85
GO:00311	mRNA 3'-e	Biological	0.32	0.51	Solyc09g0	Solyc09g0	0.10	-3.31
GO:00048	enzyme inh	Molecular	0.32	0.51	Solyc03g1	Solyc03g1	0.37	-1.42
GO:00048	enzyme inh	Molecular	0.32	0.51	Solyc03g0	Solyc03g0	0.28	-1.83
GO:00048	enzyme inh	Molecular	0.32	0.51	Solyc01g0	Solyc01g0	4.89	2.29
GO:00048	enzyme inh	Molecular	0.32	0.51	Solyc01g0	Solyc01g0	2.08	1.05
GO:00048	enzyme inh	Molecular	0.32	0.51	Solyc07g0	Solyc07g0	0.42	-1.26
GO:00048	enzyme inh	Molecular	0.32	0.51	Solyc07g0	Solyc07g0	2.42	1.28
GO:00420	protein ref	Biological	0.33	0.52	Solyc10g0	Solyc10g0	0.47	-1.09
GO:00420	protein ref	Biological	0.33	0.52	Solyc03g1	Solyc03g1	0.00	-11.76
GO:00510	chaperone	Biological	0.33	0.52	Solyc10g0	Solyc10g0	0.47	-1.09
GO:00510	chaperone	Biological	0.33	0.52	Solyc03g1	Solyc03g1	0.00	-11.76
GO:00007	chromatin	Cellular C	0.33	0.52	Solyc03g1	Solyc03g1	0.30	-1.72
GO:00007	chromatin	Cellular C	0.33	0.52	Solyc01g0	Solyc01g0	2.42	1.27
GO:00042	serine-type	Molecular	0.33	0.52	Solyc04g0	Solyc04g0	0.18	-2.44
GO:00042	serine-type	Molecular	0.33	0.52	Solyc10g0	Solyc10g0	0.09	-3.52
GO:00042	serine-type	Molecular	0.33	0.52	Solyc01g0	Solyc01g0	0.30	-1.74
GO:00042	serine-type	Molecular	0.33	0.52	Solyc02g0	Solyc02g0	0.06	-4.14
GO:00042	serine-type	Molecular	0.33	0.52	Solyc09g0	Solyc09g0	3.21	1.68
GO:00042	serine-type	Molecular	0.33	0.52	Solyc08g0	Solyc08g0	0.11	-3.22
GO:00042	serine-type	Molecular	0.33	0.52	Solyc01g0	Solyc01g0	0.31	-1.67
GO:00042	serine-type	Molecular	0.33	0.52	Solyc02g0	Solyc02g0	722.84	9.50
GO:00042	serine-type	Molecular	0.33	0.52	Solyc10g0	Solyc10g0	0.30	-1.75
GO:00167	hydrolase a	Molecular	0.33	0.53	Solyc03g0	Solyc03g0	2.46	1.30
GO:00167	hydrolase a	Molecular	0.33	0.53	Solyc01g1	Solyc01g1	3.32	1.73
GO:00167	hydrolase a	Molecular	0.33	0.53	Solyc01g0	Solyc01g0	4.27	2.09
GO:00167	hydrolase a	Molecular	0.33	0.53	Solyc01g0	Solyc01g0	4.01	2.00
GO:00167	hydrolase a	Molecular	0.33	0.53	Solyc05g0	Solyc05g0	12.80	3.68
GO:00167	hydrolase a	Molecular	0.33	0.53	Solyc02g0	Solyc02g0	2.27	1.18
GO:00167	hydrolase a	Molecular	0.33	0.53	Solyc02g0	Solyc02g0	2.19	1.13
GO:00167	hydrolase a	Molecular	0.33	0.53	Solyc02g0	Solyc02g0	6.38	2.67
GO:00459	positive re	Biological	0.34	0.53	Solyc06g0	Solyc06g0	2.41	1.27
GO:00459	positive re	Biological	0.34	0.53	Solyc04g0	Solyc04g0	0.46	-1.11
GO:00459	positive re	Biological	0.34	0.53	Solyc04g0	Solyc04g0	2.02	1.01
GO:00459	positive re	Biological	0.34	0.53	Solyc03g1	Solyc03g1	2.90	1.54
GO:00459	positive re	Biological	0.34	0.53	Solyc02g0	Solyc02g0	0.00	-11.41
GO:00459	positive re	Biological	0.34	0.53	Solyc01g1	Solyc01g1	0.15	-2.78
GO:00459	positive re	Biological	0.34	0.53	Solyc10g0	Solyc10g0	1745.96	10.77
GO:00800	quercetin 3	Molecular	0.35	0.54	Solyc09g0	Solyc09g0	0.07	-3.81
GO:00800	quercetin 3	Molecular	0.35	0.54	Solyc10g0	Solyc10g0	0.37	-1.43
GO:00800	quercetin 3	Molecular	0.35	0.54	Solyc08g0	Solyc08g0	0.32	-1.65
GO:00800	quercetin 7	Molecular	0.35	0.54	Solyc09g0	Solyc09g0	0.07	-3.81
GO:00800	quercetin 7	Molecular	0.35	0.54	Solyc10g0	Solyc10g0	0.37	-1.43

GO:00800	quercetin 7	Molecular	0.35	0.54	Solyc08g0	Solyc08g0	0.32	-1.65
GO:00302	cellulose b	Biological	0.35	0.54	Solyc04g0	Solyc04g0	0.48	-1.06
GO:00302	cellulose b	Biological	0.35	0.54	Solyc06g0	Solyc06g0	5812.44	12.50
GO:00302	cellulose b	Biological	0.35	0.54	Solyc07g0	Solyc07g0	0.44	-1.18
GO:00036	minor groc	Molecular	0.35	0.54	Solyc01g0	Solyc01g0	0.40	-1.33
GO:00036	minor groc	Molecular	0.35	0.54	Solyc09g0	Solyc09g0	0.37	-1.42
GO:00036	minor groc	Molecular	0.35	0.54	Solyc01g0	Solyc01g0	0.42	-1.24
GO:00007	double-stra	Biological	0.35	0.54	Solyc05g0	Solyc05g0	2.04	1.03
GO:00007	double-stra	Biological	0.35	0.54	Solyc06g0	Solyc06g0	2.41	1.27
GO:00161	brassinoste	Biological	0.35	0.54	Solyc02g0	Solyc02g0	0.36	-1.49
GO:00161	brassinoste	Biological	0.35	0.54	Solyc01g0	Solyc01g0	0.29	-1.77
GO:00518	Hsp90 pro	Molecular	0.36	0.54	Solyc08g0	Solyc08g0	0.49	-1.02
GO:00102	response tc	Biological	0.36	0.54	Solyc02g0	Solyc02g0	0.02	-5.62
GO:00454	xylan catal	Biological	0.36	0.54	Solyc01g1	Solyc01g1	2.32	1.21
GO:00329	membrane	Molecular	0.36	0.54	Solyc11g0	Solyc11g0	0.31	-1.71
GO:00192	reductive p	Biological	0.36	0.54	Solyc03g0	RBCS-2A	0.34	-1.58
GO:00309	Smc5-Smc	Cellular Co	0.36	0.54	Solyc05g0	Solyc05g0	2.04	1.03
GO:00451	cell fate co	Biological	0.36	0.54	Solyc11g0	Solyc11g0	0.19	-2.40
GO:00166	malate deh	Molecular	0.36	0.54	Solyc03g0	Solyc03g0	0.46	-1.11
GO:00169	poly(A)+ r	Biological	0.36	0.54	Solyc12g0	Solyc12g0	2.53	1.34
GO:00096	response tc	Biological	0.36	0.54	Solyc01g0	Solyc01g0	0.45	-1.15
GO:00718	DNA biosy	Biological	0.36	0.54	Solyc11g0	Solyc11g0	2.55	1.35
GO:00452	respiratory	Cellular Co	0.36	0.54	Solyc08g0	Solyc08g0	0.48	-1.06
GO:00801	regulation	Biological	0.36	0.54	Solyc02g0	Solyc02g0	0.08	-3.65
GO:00431	3'-5' DNA	Molecular	0.36	0.54	Solyc09g0	Solyc09g0	2.25	1.17
GO:00095	phragmopl	Cellular Co	0.36	0.54	Solyc05g0	Solyc05g0	2.46	1.30
GO:00085	plus-end-d	Molecular	0.36	0.54	Solyc10g0	Solyc10g0	2.44	1.29
GO:00044	long-chain	Molecular	0.36	0.54	Solyc01g1	Solyc01g1	0.50	-1.01
GO:00320	regulation	Biological	0.36	0.54	Solyc02g0	Solyc02g0	2.27	1.18
GO:00065	L-serine ca	Biological	0.36	0.54	Solyc09g0	TD2	4.53	2.18
GO:00090	isoleucine	Biological	0.36	0.54	Solyc09g0	TD2	4.53	2.18
GO:00906	mitochond	Biological	0.36	0.54	Solyc09g0	Solyc09g0	6.02	2.59
GO:00105	NAD(P)H	Cellular Co	0.36	0.54	Solyc12g0	Solyc12g0	0.00	-10.26
GO:00039	NAD+ nuc	Molecular	0.36	0.54	Solyc01g1	Solyc01g1	0.08	-3.71
GO:00039	NAD+ nuc	Molecular	0.36	0.54	Solyc05g0	Solyc05g0	2.15	1.10
GO:00039	NAD+ nuc	Molecular	0.36	0.54	Solyc09g0	Solyc09g0	0.00	-9.29
GO:00301	protein cat	Biological	0.36	0.54	Solyc01g0	Solyc01g0	0.46	-1.12
GO:00301	protein cat	Biological	0.36	0.54	Solyc08g0	Solyc08g0	0.48	-1.06
GO:00301	protein cat	Biological	0.36	0.54	Solyc01g0	Solyc01g0	0.24	-2.04
GO:00301	protein cat	Biological	0.36	0.54	Solyc09g0	Solyc09g0	4.04	2.01
GO:00301	protein cat	Biological	0.36	0.54	Solyc01g0	Solyc01g0	0.18	-2.50
GO:00305	pectate lya	Molecular	0.37	0.55	Solyc09g0	Solyc09g0	0.27	-1.88
GO:00305	pectate lya	Molecular	0.37	0.55	Solyc05g0	Solyc05g0	0.26	-1.96
GO:00451	electron tra	Molecular	0.37	0.55	Solyc02g0	Solyc02g0	0.35	-1.53
GO:00451	electron tra	Molecular	0.37	0.55	Solyc05g0	Solyc05g0	0.46	-1.12
GO:00164	palmitoyltı	Molecular	0.37	0.55	Solyc05g0	Solyc05g0	0.38	-1.40
GO:00164	palmitoyltı	Molecular	0.37	0.55	Solyc02g0	Solyc02g0	0.41	-1.30
GO:00229	electron tra	Biological	0.37	0.56	Solyc01g1	Solyc01g1	0.46	-1.13
GO:00229	electron tra	Biological	0.37	0.56	Solyc11g0	Solyc11g0	9.10	3.19
GO:00229	electron tra	Biological	0.37	0.56	Solyc01g0	Solyc01g0	0.38	-1.39
GO:00229	electron tra	Biological	0.37	0.56	Solyc03g0	Solyc03g0	0.34	-1.57

GO:00506	NADP bin	Molecular	0.37	0.56	Solyc06g0	Solyc06g0	2.01	1.01
GO:00506	NADP bin	Molecular	0.37	0.56	Solyc02g0	Solyc02g0	0.46	-1.12
GO:00506	NADP bin	Molecular	0.37	0.56	Solyc06g0	Solyc06g0	0.30	-1.75
GO:00506	NADP bin	Molecular	0.37	0.56	Solyc12g0	Solyc12g0	0.13	-2.93
GO:00062	DNA repai	Biological	0.38	0.57	Solyc03g1	Solyc03g1	0.30	-1.72
GO:00062	DNA repai	Biological	0.38	0.57	Solyc05g0	Solyc05g0	2.04	1.03
GO:00062	DNA repai	Biological	0.38	0.57	Solyc06g0	Solyc06g0	2.41	1.27
GO:00062	DNA repai	Biological	0.38	0.57	Solyc03g1	Solyc03g1	2.29	1.19
GO:00062	DNA repai	Biological	0.38	0.57	Solyc01g0	Solyc01g0	208.08	7.70
GO:00062	DNA repai	Biological	0.38	0.57	Solyc09g0	Solyc09g0	3.39	1.76
GO:00062	DNA repai	Biological	0.38	0.57	Solyc01g0	Solyc01g0	2.16	1.11
GO:00062	DNA repai	Biological	0.38	0.57	Solyc08g0	Solyc08g0	9.73	3.28
GO:00062	DNA repai	Biological	0.38	0.57	Solyc02g0	Solyc02g0	2.19	1.13
GO:00062	DNA repai	Biological	0.38	0.57	Solyc10g0	Solyc10g0	2.30	1.20
GO:00062	DNA repai	Biological	0.38	0.57	Solyc02g0	Solyc02g0	0.23	-2.09
GO:00009	transcriptio	Molecular	0.38	0.57	Solyc01g0	Solyc01g0	0.04	-4.49
GO:00009	transcriptio	Molecular	0.38	0.57	Solyc02g0	Solyc02g0	0.33	-1.60
GO:00009	transcriptio	Molecular	0.38	0.57	Solyc01g0	Solyc01g0	0.11	-3.13
GO:00009	transcriptio	Molecular	0.38	0.57	Solyc10g0	Solyc10g0	0.00	-13.06
GO:00009	transcriptio	Molecular	0.38	0.57	Solyc02g0	Solyc02g0	0.00	-9.32
GO:00065	GPI ancho	Biological	0.39	0.57	Solyc08g0	Solyc08g0	0.50	-1.01
GO:00065	GPI ancho	Biological	0.39	0.57	Solyc03g0	Solyc03g0	2.48	1.31
GO:00080	tRNA proc	Biological	0.39	0.57	Solyc05g0	Solyc05g0	0.00	-11.22
GO:00080	tRNA proc	Biological	0.39	0.57	Solyc10g0	Solyc10g0	476.24	8.90
GO:00040	catalase ac	Molecular	0.39	0.57	Solyc12g0	Solyc12g0	0.28	-1.84
GO:00313	translation	Molecular	0.39	0.57	Solyc06g0	Solyc06g0	2.11	1.07
GO:00105	pollen exir	Biological	0.39	0.57	Solyc03g0	Solyc03g0	0.11	-3.22
GO:00469	acyltransfe	Molecular	0.39	0.57	Solyc07g0	Solyc07g0	0.49	-1.04
GO:00007	chromosor	Cellular Co	0.39	0.57	Solyc01g0	Solyc01g0	2.42	1.27
GO:00168	racemase a	Molecular	0.39	0.57	Solyc07g0	Solyc07g0	2.29	1.19
GO:00097	blue light s	Biological	0.39	0.57	Solyc12g0	Solyc12g0	0.32	-1.65
GO:00100	phloem or	Biological	0.39	0.57	Solyc08g0	Solyc08g0	0.39	-1.34
GO:00082	spermidine	Biological	0.39	0.57	Solyc10g0	Solyc10g0	0.38	-1.40
GO:00099	chloroplast	Biological	0.39	0.57	Solyc06g0	Solyc06g0	5.58	2.48
GO:00040	aldehyde d	Molecular	0.39	0.57	Solyc03g1	Solyc03g1	0.46	-1.12
GO:00430	alpha-tubu	Molecular	0.39	0.57	Solyc05g0	Solyc05g0	0.13	-3.00
GO:00986	anion trans	Biological	0.39	0.57	Solyc03g1	Solyc03g1	18.12	4.18
GO:00159	chlorophyl	Biological	0.39	0.57	Solyc09g0	Solyc09g0	2049.83	11.00
GO:00160	RNA meta	Biological	0.39	0.57	Solyc05g0	Solyc05g0	0.48	-1.07
GO:00092	mRNA tra	Biological	0.39	0.57	Solyc10g0	Solyc10g0	0.45	-1.15
GO:00097	response to	Biological	0.39	0.57	Solyc12g0	Solyc12g0	0.40	-1.34
GO:00709	protein loc	Biological	0.39	0.57	Solyc04g0	Solyc04g0	0.45	-1.14
GO:00427	positive re	Biological	0.39	0.57	Solyc06g0	Solyc06g0	0.40	-1.30
GO:00096	response to	Biological	0.39	0.57	Solyc08g0	Solyc08g0	0.19	-2.36
GO:00096	response to	Biological	0.39	0.57	Solyc09g0	Solyc09g0	0.00	-15.42
GO:00096	response to	Biological	0.39	0.57	Solyc09g0	Solyc09g0	33.35	5.06
GO:00054	SNAP rece	Molecular	0.39	0.57	Solyc09g0	Solyc09g0	2.28	1.19
GO:00054	SNAP rece	Molecular	0.39	0.57	Solyc06g0	Solyc06g0	2.11	1.07
GO:00054	SNAP rece	Molecular	0.39	0.57	Solyc01g0	Solyc01g0	0.19	-2.40
GO:00080	microtubul	Molecular	0.39	0.57	Solyc04g0	Solyc04g0	0.48	-1.06
GO:00080	microtubul	Molecular	0.39	0.57	Solyc02g0	Solyc02g0	2.64	1.40

GO:00080	microtubul	Molecular	0.39	0.57	Solyc05g0	Solyc05g0	2.46	1.30
GO:00080	microtubul	Molecular	0.39	0.57	Solyc10g0	Solyc10g0	2.44	1.29
GO:00080	microtubul	Molecular	0.39	0.57	Solyc12g0	Solyc12g0	9.14	3.19
GO:00080	microtubul	Molecular	0.39	0.57	Solyc04g0	Solyc04g0	3.15	1.66
GO:00080	microtubul	Molecular	0.39	0.57	Solyc09g0	Solyc09g0	0.00	-9.38
GO:00080	microtubul	Molecular	0.39	0.57	Solyc03g0	Solyc03g0	0.00	-8.17
GO:00095	chloroplas	Cellular C	0.40	0.57	Solyc11g0	AOS2	0.12	-3.01
GO:00095	chloroplas	Cellular C	0.40	0.57	Solyc01g0	Solyc01g0	4.36	2.12
GO:00095	chloroplas	Cellular C	0.40	0.57	Solyc07g0	NCED1	0.30	-1.72
GO:00095	chloroplas	Cellular C	0.40	0.57	Solyc01g1	Solyc01g1	0.46	-1.13
GO:00095	chloroplas	Cellular C	0.40	0.57	Solyc02g0	PSBO	0.48	-1.07
GO:00095	chloroplas	Cellular C	0.40	0.57	Solyc02g0	Solyc02g0	0.35	-1.53
GO:00095	chloroplas	Cellular C	0.40	0.57	Solyc03g0	Solyc03g0	0.18	-2.46
GO:00095	chloroplas	Cellular C	0.40	0.57	Solyc03g0	RBCS-2A	0.34	-1.58
GO:00095	chloroplas	Cellular C	0.40	0.57	Solyc10g0	Solyc10g0	0.43	-1.23
GO:00095	chloroplas	Cellular C	0.40	0.57	Solyc09g0	Solyc09g0	0.21	-2.26
GO:00095	chloroplas	Cellular C	0.40	0.57	Solyc03g0	Solyc03g0	0.46	-1.11
GO:00095	chloroplas	Cellular C	0.40	0.57	Solyc09g0	Solyc09g0	0.40	-1.33
GO:00095	chloroplas	Cellular C	0.40	0.57	Solyc06g0	Solyc06g0	0.23	-2.10
GO:00095	chloroplas	Cellular C	0.40	0.57	Solyc06g0	psaD	0.43	-1.22
GO:00095	chloroplas	Cellular C	0.40	0.57	Solyc09g0	Solyc09g0	0.43	-1.22
GO:00095	chloroplas	Cellular C	0.40	0.57	Solyc11g0	Solyc11g0	9.10	3.19
GO:00095	chloroplas	Cellular C	0.40	0.57	Solyc05g0	Solyc05g0	0.15	-2.75
GO:00095	chloroplas	Cellular C	0.40	0.57	Solyc01g0	Solyc01g0	0.38	-1.39
GO:00095	chloroplas	Cellular C	0.40	0.57	Solyc10g0	Solyc10g0	0.49	-1.02
GO:00095	chloroplas	Cellular C	0.40	0.57	Solyc05g0	Solyc05g0	0.31	-1.69
GO:00095	chloroplas	Cellular C	0.40	0.57	Solyc01g0	Solyc01g0	7223.16	12.82
GO:00095	chloroplas	Cellular C	0.40	0.57	Solyc02g0	Solyc02g0	0.46	-1.12
GO:00095	chloroplas	Cellular C	0.40	0.57	Solyc08g0	Solyc08g0	0.48	-1.05
GO:00095	chloroplas	Cellular C	0.40	0.57	Solyc03g0	Solyc03g0	0.45	-1.15
GO:00095	chloroplas	Cellular C	0.40	0.57	Solyc12g0	Solyc12g0	0.13	-2.93
GO:00095	chloroplas	Cellular C	0.40	0.57	Solyc11g0	Solyc11g0	0.33	-1.60
GO:00095	chloroplas	Cellular C	0.40	0.57	Solyc02g0	Solyc02g0	0.45	-1.16
GO:00095	chloroplas	Cellular C	0.40	0.57	Solyc06g0	Solyc06g0	0.31	-1.71
GO:00095	chloroplas	Cellular C	0.40	0.57	Solyc09g0	TD2	4.53	2.18
GO:00095	chloroplas	Cellular C	0.40	0.57	Solyc12g0	Solyc12g0	0.30	-1.76
GO:00095	chloroplas	Cellular C	0.40	0.57	Solyc01g1	CAP10A	0.35	-1.51
GO:00095	chloroplas	Cellular C	0.40	0.57	Solyc11g0	Solyc11g0	0.24	-2.07
GO:00095	chloroplas	Cellular C	0.40	0.57	Solyc05g0	Solyc05g0	0.00	-11.22
GO:00095	chloroplas	Cellular C	0.40	0.57	Solyc03g0	Solyc03g0	0.34	-1.57
GO:00095	chloroplas	Cellular C	0.40	0.57	Solyc09g0	Solyc09g0	0.24	-2.04
GO:00097	auxin-acti	Biological	0.40	0.58	Solyc05g0	Solyc05g0	0.18	-2.51
GO:00097	auxin-acti	Biological	0.40	0.58	Solyc06g0	Solyc06g0	0.39	-1.35
GO:00097	auxin-acti	Biological	0.40	0.58	Solyc08g0	Solyc08g0	0.38	-1.40
GO:00097	auxin-acti	Biological	0.40	0.58	Solyc03g1	Solyc03g1	0.48	-1.05
GO:00097	auxin-acti	Biological	0.40	0.58	Solyc03g1	Solyc03g1	0.08	-3.61
GO:00302	polysaccha	Molecular	0.40	0.58	Solyc02g0	Solyc02g0	0.15	-2.72
GO:00302	polysaccha	Molecular	0.40	0.58	Solyc01g0	XTH1	0.31	-1.69
GO:00302	polysaccha	Molecular	0.40	0.58	Solyc09g0	Solyc09g0	2.30	1.20
GO:00166	oxidoreduc	Molecular	0.41	0.58	Solyc01g0	Solyc01g0	0.29	-1.78
GO:00166	oxidoreduc	Molecular	0.41	0.58	Solyc11g0	Solyc11g0	3.98	1.99

GO:00469	proton-trar	Molecular	0.41	0.58	Solyc06g0	Solyc06g0	3.71	1.89
GO:00469	proton-trar	Molecular	0.41	0.58	Solyc11g0	Solyc11g0	0.33	-1.60
GO:00167	oxidoreduc	Molecular	0.41	0.58	Solyc02g0	Solyc02g0	6.42	2.68
GO:00167	oxidoreduc	Molecular	0.41	0.58	Solyc01g0	Solyc01g0	0.18	-2.51
GO:00063	regulation	Biological	0.41	0.58	Solyc09g0	Solyc09g0	0.48	-1.06
GO:00063	regulation	Biological	0.41	0.58	Solyc01g0	Solyc01g0	0.35	-1.51
GO:00063	regulation	Biological	0.41	0.58	Solyc10g0	Solyc10g0	0.00	-13.06
GO:00063	regulation	Biological	0.41	0.58	Solyc04g0	Solyc04g0	2.02	1.01
GO:00063	regulation	Biological	0.41	0.58	Solyc12g0	Solyc12g0	0.00	-10.69
GO:00063	regulation	Biological	0.41	0.58	Solyc03g1	Solyc03g1	2.90	1.54
GO:00063	regulation	Biological	0.41	0.58	Solyc05g0	Solyc05g0	0.00	-8.91
GO:00063	regulation	Biological	0.41	0.58	Solyc10g0	Solyc10g0	2.87	1.52
GO:00063	regulation	Biological	0.41	0.58	Solyc04g0	Solyc04g0	0.20	-2.32
GO:00425	cell wall m	Biological	0.41	0.59	Solyc03g1	Solyc03g1	0.37	-1.42
GO:00425	cell wall m	Biological	0.41	0.59	Solyc09g0	Solyc09g0	4.75	2.25
GO:00425	cell wall m	Biological	0.41	0.59	Solyc03g0	Solyc03g0	0.28	-1.83
GO:00425	cell wall m	Biological	0.41	0.59	Solyc01g0	Solyc01g0	4.89	2.29
GO:00425	cell wall m	Biological	0.41	0.59	Solyc07g0	Solyc07g0	2.40	1.27
GO:00055	ATP bindi	Molecular	0.42	0.59	Solyc02g0	Solyc02g0	0.14	-2.87
GO:00055	ATP bindi	Molecular	0.42	0.59	Solyc02g0	Solyc02g0	2.65	1.40
GO:00055	ATP bindi	Molecular	0.42	0.59	Solyc06g0	Solyc06g0	2.42	1.28
GO:00055	ATP bindi	Molecular	0.42	0.59	Solyc05g0	Solyc05g0	2.01	1.01
GO:00055	ATP bindi	Molecular	0.42	0.59	Solyc01g1	Solyc01g1	0.31	-1.68
GO:00055	ATP bindi	Molecular	0.42	0.59	Solyc10g0	Solyc10g0	0.47	-1.09
GO:00055	ATP bindi	Molecular	0.42	0.59	Solyc11g0	Solyc11g0	0.13	-3.00
GO:00055	ATP bindi	Molecular	0.42	0.59	Solyc05g0	Solyc05g0	2.04	1.03
GO:00055	ATP bindi	Molecular	0.42	0.59	Solyc09g0	Solyc09g0	0.43	-1.22
GO:00055	ATP bindi	Molecular	0.42	0.59	Solyc02g0	Solyc02g0	0.47	-1.10
GO:00055	ATP bindi	Molecular	0.42	0.59	Solyc08g0	Solyc08g0	0.31	-1.70
GO:00055	ATP bindi	Molecular	0.42	0.59	Solyc03g0	Solyc03g0	0.46	-1.13
GO:00055	ATP bindi	Molecular	0.42	0.59	Solyc02g0	Solyc02g0	0.42	-1.24
GO:00055	ATP bindi	Molecular	0.42	0.59	Solyc02g0	Solyc02g0	0.41	-1.29
GO:00055	ATP bindi	Molecular	0.42	0.59	Solyc05g0	Solyc05g0	2.27	1.18
GO:00055	ATP bindi	Molecular	0.42	0.59	Solyc02g0	Solyc02g0	0.15	-2.72
GO:00055	ATP bindi	Molecular	0.42	0.59	Solyc01g1	Solyc01g1	3.45	1.79
GO:00055	ATP bindi	Molecular	0.42	0.59	Solyc03g1	Solyc03g1	0.39	-1.35
GO:00055	ATP bindi	Molecular	0.42	0.59	Solyc02g0	Solyc02g0	2.64	1.40
GO:00055	ATP bindi	Molecular	0.42	0.59	Solyc09g0	Solyc09g0	2.10	1.07
GO:00055	ATP bindi	Molecular	0.42	0.59	Solyc04g0	Solyc04g0	0.39	-1.35
GO:00055	ATP bindi	Molecular	0.42	0.59	Solyc12g0	Solyc12g0	0.33	-1.60
GO:00055	ATP bindi	Molecular	0.42	0.59	Solyc05g0	Solyc05g0	0.46	-1.11
GO:00055	ATP bindi	Molecular	0.42	0.59	Solyc11g0	Solyc11g0	0.48	-1.06
GO:00055	ATP bindi	Molecular	0.42	0.59	Solyc03g0	Solyc03g0	4.75	2.25
GO:00055	ATP bindi	Molecular	0.42	0.59	Solyc11g0	Solyc11g0	0.49	-1.03
GO:00055	ATP bindi	Molecular	0.42	0.59	Solyc09g0	Solyc09g0	2.86	1.51
GO:00055	ATP bindi	Molecular	0.42	0.59	Solyc03g0	Solyc03g0	3.70	1.89
GO:00055	ATP bindi	Molecular	0.42	0.59	Solyc04g0	Solyc04g0	0.48	-1.06
GO:00055	ATP bindi	Molecular	0.42	0.59	Solyc03g0	Solyc03g0	2.33	1.22
GO:00055	ATP bindi	Molecular	0.42	0.59	Solyc08g0	Solyc08g0	0.39	-1.34
GO:00055	ATP bindi	Molecular	0.42	0.59	Solyc10g0	Solyc10g0	0.43	-1.22
GO:00055	ATP bindi	Molecular	0.42	0.59	Solyc06g0	Solyc06g0	0.49	-1.02

GO:00055	ATP bindi	Molecular	0.42	0.59	Solyc11g0	Solyc11g0	0.33	-1.59
GO:00055	ATP bindi	Molecular	0.42	0.59	Solyc05g0	Solyc05g0	0.46	-1.12
GO:00055	ATP bindi	Molecular	0.42	0.59	Solyc06g0	Solyc06g0	0.40	-1.31
GO:00055	ATP bindi	Molecular	0.42	0.59	Solyc09g0	Solyc09g0	0.39	-1.35
GO:00055	ATP bindi	Molecular	0.42	0.59	Solyc08g0	Solyc08g0	0.48	-1.04
GO:00055	ATP bindi	Molecular	0.42	0.59	Solyc11g0	Solyc11g0	0.46	-1.11
GO:00055	ATP bindi	Molecular	0.42	0.59	Solyc02g0	Solyc02g0	0.34	-1.57
GO:00055	ATP bindi	Molecular	0.42	0.59	Solyc02g0	Solyc02g0	0.12	-3.00
GO:00055	ATP bindi	Molecular	0.42	0.59	Solyc01g0	Solyc01g0	3.11	1.64
GO:00055	ATP bindi	Molecular	0.42	0.59	Solyc07g0	Solyc07g0	0.25	-1.97
GO:00055	ATP bindi	Molecular	0.42	0.59	Solyc08g0	Solyc08g0	2.48	1.31
GO:00055	ATP bindi	Molecular	0.42	0.59	Solyc07g0	Solyc07g0	0.02	-5.66
GO:00055	ATP bindi	Molecular	0.42	0.59	Solyc11g0	Solyc11g0	5992.64	12.55
GO:00055	ATP bindi	Molecular	0.42	0.59	Solyc06g0	Solyc06g0	3.73	1.90
GO:00055	ATP bindi	Molecular	0.42	0.59	Solyc11g0	Solyc11g0	2.74	1.45
GO:00055	ATP bindi	Molecular	0.42	0.59	Solyc10g0	Solyc10g0	0.06	-4.05
GO:00055	ATP bindi	Molecular	0.42	0.59	Solyc02g0	Solyc02g0	0.10	-3.33
GO:00055	ATP bindi	Molecular	0.42	0.59	Solyc11g0	Solyc11g0	2.55	1.35
GO:00055	ATP bindi	Molecular	0.42	0.59	Solyc01g0	Solyc01g0	0.00	-12.11
GO:00055	ATP bindi	Molecular	0.42	0.59	Solyc03g0	Solyc03g0	2.30	1.20
GO:00055	ATP bindi	Molecular	0.42	0.59	Solyc10g0	Solyc10g0	2.30	1.20
GO:00055	ATP bindi	Molecular	0.42	0.59	Solyc01g1	Solyc01g1	0.20	-2.34
GO:00055	ATP bindi	Molecular	0.42	0.59	Solyc12g0	Solyc12g0	0.33	-1.58
GO:00055	ATP bindi	Molecular	0.42	0.59	Solyc03g1	Solyc03g1	0.47	-1.08
GO:00055	ATP bindi	Molecular	0.42	0.59	Solyc12g0	Solyc12g0	2112.44	11.04
GO:00055	ATP bindi	Molecular	0.42	0.59	Solyc02g0	Solyc02g0	0.02	-5.39
GO:00055	ATP bindi	Molecular	0.42	0.59	Solyc04g0	Solyc04g0	0.21	-2.26
GO:00055	ATP bindi	Molecular	0.42	0.59	Solyc07g0	Solyc07g0	0.24	-2.07
GO:00055	ATP bindi	Molecular	0.42	0.59	Solyc12g0	Solyc12g0	2.48	1.31
GO:00055	ATP bindi	Molecular	0.42	0.59	Solyc03g1	Solyc03g1	0.00	-11.76
GO:00055	ATP bindi	Molecular	0.42	0.59	Solyc01g0	Solyc01g0	1525.65	10.58
GO:00055	ATP bindi	Molecular	0.42	0.59	Solyc05g0	Solyc05g0	3.41	1.77
GO:00055	ATP bindi	Molecular	0.42	0.59	Solyc07g0	Solyc07g0	0.46	-1.11
GO:00055	ATP bindi	Molecular	0.42	0.59	Solyc02g0	Solyc02g0	0.36	-1.48
GO:00055	ATP bindi	Molecular	0.42	0.59	Solyc08g0	Solyc08g0	0.20	-2.32
GO:00055	ATP bindi	Molecular	0.42	0.59	Solyc04g0	Solyc04g0	0.35	-1.51
GO:00055	ATP bindi	Molecular	0.42	0.59	Solyc04g0	Solyc04g0	0.48	-1.06
GO:00055	ATP bindi	Molecular	0.42	0.59	Solyc09g0	Solyc09g0	0.33	-1.59
GO:00055	ATP bindi	Molecular	0.42	0.59	Solyc11g0	Solyc11g0	3.86	1.95
GO:00055	ATP bindi	Molecular	0.42	0.59	Solyc04g0	Solyc04g0	2.12	1.08
GO:00055	ATP bindi	Molecular	0.42	0.59	Solyc06g0	Solyc06g0	2.93	1.55
GO:00055	ATP bindi	Molecular	0.42	0.59	Solyc03g0	Solyc03g0	4.19	2.07
GO:00055	ATP bindi	Molecular	0.42	0.59	Solyc10g0	Solyc10g0	2.44	1.29
GO:00055	ATP bindi	Molecular	0.42	0.59	Solyc07g0	Solyc07g0	2.61	1.38
GO:00055	ATP bindi	Molecular	0.42	0.59	Solyc12g0	Solyc12g0	9.14	3.19
GO:00055	ATP bindi	Molecular	0.42	0.59	Solyc03g0	Solyc03g0	0.48	-1.05
GO:00055	ATP bindi	Molecular	0.42	0.59	Solyc02g0	Solyc02g0	0.16	-2.63
GO:00055	ATP bindi	Molecular	0.42	0.59	Solyc11g0	Solyc11g0	0.12	-3.04
GO:00055	ATP bindi	Molecular	0.42	0.59	Solyc08g0	Solyc08g0	0.37	-1.42
GO:00055	ATP bindi	Molecular	0.42	0.59	Solyc05g0	Solyc05g0	0.47	-1.10
GO:00055	ATP bindi	Molecular	0.42	0.59	Solyc02g0	Solyc02g0	0.06	-3.96

GO:00055	ATP bindi	Molecular	0.42	0.59	Solyc11g0	Solyc11g0	0.33	-1.60
GO:00055	ATP bindi	Molecular	0.42	0.59	Solyc06g0	Solyc06g0	0.48	-1.07
GO:00055	ATP bindi	Molecular	0.42	0.59	Solyc02g0	Solyc02g0	0.45	-1.15
GO:00055	ATP bindi	Molecular	0.42	0.59	Solyc07g0	Solyc07g0	0.29	-1.79
GO:00055	ATP bindi	Molecular	0.42	0.59	Solyc08g0	Solyc08g0	0.18	-2.44
GO:00055	ATP bindi	Molecular	0.42	0.59	Solyc04g0	Solyc04g0	2.01	1.01
GO:00055	ATP bindi	Molecular	0.42	0.59	Solyc02g0	Solyc02g0	0.50	-1.01
GO:00055	ATP bindi	Molecular	0.42	0.59	Solyc05g0	Solyc05g0	2.16	1.11
GO:00055	ATP bindi	Molecular	0.42	0.59	Solyc08g0	Solyc08g0	0.19	-2.38
GO:00055	ATP bindi	Molecular	0.42	0.59	Solyc03g0	Solyc03g0	0.00	-8.79
GO:00055	ATP bindi	Molecular	0.42	0.59	Solyc06g0	Solyc06g0	3.53	1.82
GO:00055	ATP bindi	Molecular	0.42	0.59	Solyc03g0	Solyc03g0	0.01	-7.23
GO:00055	ATP bindi	Molecular	0.42	0.59	Solyc10g0	Solyc10g0	2.49	1.31
GO:00055	ATP bindi	Molecular	0.42	0.59	Solyc01g0	Solyc01g0	2.66	1.41
GO:00055	ATP bindi	Molecular	0.42	0.59	Solyc10g0	Solyc10g0	0.50	-1.01
GO:00055	ATP bindi	Molecular	0.42	0.59	Solyc02g0	Solyc02g0	0.49	-1.03
GO:00055	ATP bindi	Molecular	0.42	0.59	Solyc12g0	Solyc12g0	0.00	-11.47
GO:00055	ATP bindi	Molecular	0.42	0.59	Solyc09g0	Solyc09g0	0.38	-1.40
GO:00055	ATP bindi	Molecular	0.42	0.59	Solyc08g0	Solyc08g0	0.07	-3.76
GO:00055	ATP bindi	Molecular	0.42	0.59	Solyc09g0	Solyc09g0	0.08	-3.73
GO:00055	ATP bindi	Molecular	0.42	0.59	Solyc06g0	Solyc06g0	0.26	-1.94
GO:00055	ATP bindi	Molecular	0.42	0.59	Solyc03g0	Solyc03g0	0.00	-8.17
GO:00055	ATP bindi	Molecular	0.42	0.59	Solyc01g0	Solyc01g0	2.61	1.39
GO:00055	ATP bindi	Molecular	0.42	0.59	Solyc09g0	Solyc09g0	2.30	1.20
GO:00055	ATP bindi	Molecular	0.42	0.59	Solyc10g0	Solyc10g0	0.42	-1.27
GO:00055	ATP bindi	Molecular	0.42	0.59	Solyc10g0	Solyc10g0	0.35	-1.51
GO:00055	ATP bindi	Molecular	0.42	0.59	Solyc06g0	Solyc06g0	2.48	1.31
GO:00055	ATP bindi	Molecular	0.42	0.59	Solyc11g0	Solyc11g0	0.00	-9.30
GO:00055	ATP bindi	Molecular	0.42	0.59	Solyc02g0	Solyc02g0	2.46	1.30
GO:00055	ATP bindi	Molecular	0.42	0.59	Solyc12g0	Solyc12g0	0.21	-2.24
GO:00055	ATP bindi	Molecular	0.42	0.59	Solyc02g0	Solyc02g0	0.44	-1.18
GO:00055	ATP bindi	Molecular	0.42	0.59	Solyc05g0	Solyc05g0	0.00	-8.91
GO:00055	ATP bindi	Molecular	0.42	0.59	Solyc04g0	Solyc04g0	0.00	-8.72
GO:00055	ATP bindi	Molecular	0.42	0.59	Solyc06g0	Solyc06g0	0.41	-1.27
GO:00055	ATP bindi	Molecular	0.42	0.59	Solyc01g0	Solyc01g0	0.33	-1.61
GO:00055	ATP bindi	Molecular	0.42	0.59	Solyc09g0	Solyc09g0	0.22	-2.21
GO:00152	channel ac	Molecular	0.42	0.59	Solyc06g0	Solyc06g0	0.37	-1.44
GO:00152	channel ac	Molecular	0.42	0.59	Solyc03g0	Solyc03g0	0.00	-10.59
GO:00152	channel ac	Molecular	0.42	0.59	Solyc12g0	Solyc12g0	1405.23	10.46
GO:00061	nucleobase	Biological	0.42	0.59	Solyc09g0	Solyc09g0	5.78	2.53
GO:00061	nucleobase	Biological	0.42	0.59	Solyc12g0	Solyc12g0	0.32	-1.65
GO:00061	nucleobase	Biological	0.42	0.59	Solyc10g0	Solyc10g0	2.30	1.20
GO:00096	ethylene bi	Biological	0.42	0.59	Solyc02g0	Solyc02g0	0.23	-2.13
GO:00038	3-beta-hyd	Molecular	0.42	0.59	Solyc02g0	Solyc02g0	0.14	-2.87
GO:00161	sucrose sy	Molecular	0.42	0.59	Solyc12g0	Solyc12g0	0.40	-1.33
GO:00162	antioxidan	Molecular	0.42	0.59	Solyc07g0	Solyc07g0	2.37	1.24
GO:00198	protein kin	Molecular	0.42	0.59	Solyc06g0	Solyc06g0	0.44	-1.19
GO:00043	farnesyltra	Molecular	0.42	0.59	Solyc04g0	Solyc04g0	2.15	1.11
GO:00423	indole gluc	Biological	0.42	0.59	Solyc07g0	Solyc07g0	0.30	-1.76
GO:00039	NAD(P)H	Molecular	0.42	0.59	Solyc02g0	Solyc02g0	0.50	-1.01
GO:00480	UDP-gluc	Molecular	0.42	0.59	Solyc09g0	Solyc09g0	2.89	1.53

GO:00300	cellular ma	Biological	0.42	0.59	Solyc04g0	Solyc04g0	0.48	-1.05
GO:00071	G protein-	Biological	0.42	0.59	Solyc07g0	Solyc07g0	0.50	-1.01
GO:00152	ligand-gate	Molecular	0.42	0.59	Solyc07g0	Solyc07g0	0.50	-1.01
GO:00302	dynein con	Cellular C	0.42	0.59	Solyc03g1	Solyc03g1	0.35	-1.51
GO:00052	voltage-ga	Molecular	0.42	0.59	Solyc02g0	Solyc02g0	0.31	-1.67
GO:00068	chloride tr	Biological	0.42	0.59	Solyc02g0	Solyc02g0	0.31	-1.67
GO:00193	transsulfur	Biological	0.42	0.59	Solyc08g0	Solyc08g0	0.07	-3.82
GO:00800	fatty-acyl-	Molecular	0.42	0.59	Solyc11g0	Solyc11g0	0.00	-8.21
GO:00083	asymmetri	Biological	0.42	0.59	Solyc09g0	Solyc09g0	0.39	-1.37
GO:00450	glycerolipi	Biological	0.42	0.59	Solyc12g0	Solyc12g0	0.00	-9.16
GO:00100	trichome d	Biological	0.42	0.59	Solyc03g0	Solyc03g0	16.58	4.05
GO:00048	trehalose-p	Molecular	0.42	0.59	Solyc06g0	Solyc06g0	379.31	8.57
GO:00097	chloroplas	Cellular C	0.43	0.59	Solyc11g0	AOS2	0.12	-3.01
GO:00097	chloroplas	Cellular C	0.43	0.59	Solyc11g0	Solyc11g0	0.24	-2.07
GO:00516	defense re	Biological	0.43	0.59	Solyc03g0	RBCS-2A	0.34	-1.58
GO:00516	defense re	Biological	0.43	0.59	Solyc01g0	Solyc01g0	0.00	-10.39
GO:00057	mitochond	Cellular C	0.43	0.59	Solyc07g0	Solyc07g0	0.49	-1.04
GO:00057	mitochond	Cellular C	0.43	0.59	Solyc07g0	Solyc07g0	2.89	1.53
GO:00483	leaf develo	Biological	0.43	0.59	Solyc09g0	Solyc09g0	0.21	-2.26
GO:00483	leaf develo	Biological	0.43	0.59	Solyc04g0	Solyc04g0	0.46	-1.11
GO:00097	embryo de	Biological	0.43	0.60	Solyc10g0	LE25	0.47	-1.10
GO:00097	embryo de	Biological	0.43	0.60	Solyc06g0	Solyc06g0	0.44	-1.19
GO:00097	embryo de	Biological	0.43	0.60	Solyc11g0	Solyc11g0	2.39	1.25
GO:00718	potassium	Biological	0.43	0.60	Solyc01g1	Solyc01g1	0.43	-1.23
GO:00718	potassium	Biological	0.43	0.60	Solyc12g0	Solyc12g0	0.48	-1.07
GO:00718	potassium	Biological	0.43	0.60	Solyc06g0	Solyc06g0	366.52	8.52
GO:00055	calmodulin	Molecular	0.44	0.60	Solyc02g0	Solyc02g0	0.33	-1.60
GO:00055	calmodulin	Molecular	0.44	0.60	Solyc06g0	Solyc06g0	0.07	-3.75
GO:00055	calmodulin	Molecular	0.44	0.60	Solyc03g1	Solyc03g1	0.37	-1.42
GO:00055	calmodulin	Molecular	0.44	0.60	Solyc01g0	Solyc01g0	0.28	-1.86
GO:00342	ion transm	Biological	0.44	0.61	Solyc07g0	Solyc07g0	0.50	-1.01
GO:00342	ion transm	Biological	0.44	0.61	Solyc12g0	Solyc12g0	0.48	-1.07
GO:00162	iron-sulfur	Biological	0.44	0.61	Solyc07g0	Solyc07g0	2.89	1.53
GO:00162	iron-sulfur	Biological	0.44	0.61	Solyc10g0	Solyc10g0	0.24	-2.04
GO:00305	pectinester	Molecular	0.45	0.61	Solyc03g1	Solyc03g1	0.37	-1.42
GO:00305	pectinester	Molecular	0.45	0.61	Solyc09g0	Solyc09g0	4.75	2.25
GO:00305	pectinester	Molecular	0.45	0.61	Solyc03g0	Solyc03g0	0.28	-1.83
GO:00305	pectinester	Molecular	0.45	0.61	Solyc01g0	Solyc01g0	4.89	2.29
GO:00305	pectinester	Molecular	0.45	0.61	Solyc07g0	Solyc07g0	2.40	1.27
GO:00068	lipid trans	Biological	0.45	0.61	Solyc10g0	Solyc10g0	0.01	-7.08
GO:00068	lipid trans	Biological	0.45	0.61	Solyc01g0	Solyc01g0	0.42	-1.26
GO:00068	lipid trans	Biological	0.45	0.61	Solyc01g0	Solyc01g0	2.38	1.25
GO:00068	lipid trans	Biological	0.45	0.61	Solyc03g0	Solyc03g0	0.10	-3.33
GO:00036	chromatin	Molecular	0.45	0.61	Solyc01g0	Solyc01g0	3.68	1.88
GO:00036	chromatin	Molecular	0.45	0.61	Solyc01g0	Solyc01g0	2.19	1.13
GO:00036	chromatin	Molecular	0.45	0.61	Solyc09g0	Solyc09g0	2.25	1.17
GO:00426	ATPase-co	Molecular	0.45	0.61	Solyc09g0	Solyc09g0	0.43	-1.22
GO:00426	ATPase-co	Molecular	0.45	0.61	Solyc02g0	Solyc02g0	0.34	-1.57
GO:00426	ATPase-co	Molecular	0.45	0.61	Solyc04g0	Solyc04g0	2.01	1.01
GO:00097	response t	Biological	0.45	0.61	Solyc07g0	NCED1	0.30	-1.72
GO:00017	formation	Biological	0.45	0.61	Solyc06g0	Solyc06g0	2.11	1.07

GO:00162	eukaryotic	Cellular Co	0.45	0.61	Solyc06g0	Solyc06g0	2.11	1.07
GO:00098	photorespi	Biological	0.45	0.61	Solyc03g0	RBCS-2A	0.34	-1.58
GO:00003	plant-type	Cellular Co	0.45	0.61	Solyc09g0	Solyc09g0	0.43	-1.22
GO:00086	phospholip	Biological	0.45	0.61	Solyc05g0	Solyc05g0	0.45	-1.15
GO:00167	hydrolase a	Molecular	0.45	0.61	Solyc08g0	Solyc08g0	0.50	-1.01
GO:00457	negative re	Biological	0.45	0.61	Solyc06g0	Solyc06g0	0.34	-1.54
GO:00102	response to	Biological	0.45	0.61	Solyc02g0	Solyc02g0	0.33	-1.60
GO:00514	regulation	Biological	0.45	0.61	Solyc12g0	Solyc12g0	2112.44	11.04
GO:00100	chloroplast	Biological	0.45	0.61	Solyc08g0	Solyc08g0	0.48	-1.05
GO:00070	cytoskeleto	Biological	0.45	0.61	Solyc04g0	Solyc04g0	3.15	1.66
GO:00071	reciprocal	Biological	0.45	0.61	Solyc03g0	Solyc03g0	2.49	1.31
GO:00161	carotenoid	Biological	0.45	0.61	Solyc10g0	Solyc10g0	0.28	-1.85
GO:00041	diacylglyco	Molecular	0.45	0.61	Solyc12g0	Solyc12g0	0.00	-9.16
GO:00058	integral co	Cellular Co	0.46	0.61	Solyc12g0	Solyc12g0	2.45	1.29
GO:00058	integral co	Cellular Co	0.46	0.61	Solyc11g0	Solyc11g0	0.36	-1.49
GO:00058	integral co	Cellular Co	0.46	0.61	Solyc05g0	Solyc05g0	3.55	1.83
GO:00058	integral co	Cellular Co	0.46	0.61	Solyc01g0	Solyc01g0	0.44	-1.18
GO:00058	integral co	Cellular Co	0.46	0.61	Solyc04g0	Solyc04g0	0.45	-1.14
GO:00068	cation tran	Biological	0.46	0.61	Solyc08g0	Solyc08g0	0.26	-1.92
GO:00068	cation tran	Biological	0.46	0.61	Solyc01g0	Solyc01g0	0.50	-1.01
GO:00068	cation tran	Biological	0.46	0.61	Solyc08g0	Solyc08g0	0.00	-11.90
GO:00068	cation tran	Biological	0.46	0.61	Solyc08g0	Solyc08g0	0.06	-4.18
GO:00988	cellular ox	Biological	0.46	0.62	Solyc12g0	Solyc12g0	0.28	-1.84
GO:00988	cellular ox	Biological	0.46	0.62	Solyc02g0	Solyc02g0	0.00	-11.78
GO:00988	cellular ox	Biological	0.46	0.62	Solyc01g1	Solyc01g1	2.95	1.56
GO:00086	lipid biosy	Biological	0.46	0.62	Solyc02g0	Solyc02g0	0.36	-1.49
GO:00086	lipid biosy	Biological	0.46	0.62	Solyc08g0	Solyc08g0	2.17	1.12
GO:00312	anchored c	Cellular Co	0.46	0.62	Solyc03g1	Solyc03g1	0.00	-12.22
GO:00312	anchored c	Cellular Co	0.46	0.62	Solyc08g0	Solyc08g0	0.22	-2.16
GO:00050	guanyl-nuc	Molecular	0.46	0.62	Solyc03g1	Solyc03g1	2479.17	11.28
GO:00050	guanyl-nuc	Molecular	0.46	0.62	Solyc02g0	Solyc02g0	2.27	1.18
GO:00302	carbohydra	Molecular	0.46	0.62	Solyc03g1	Solyc03g1	2.21	1.14
GO:00302	carbohydra	Molecular	0.46	0.62	Solyc11g0	Solyc11g0	2.10	1.07
GO:00302	carbohydra	Molecular	0.46	0.62	Solyc03g0	Solyc03g0	2.58	1.37
GO:00302	carbohydra	Molecular	0.46	0.62	Solyc02g0	Solyc02g0	0.45	-1.14
GO:00302	carbohydra	Molecular	0.46	0.62	Solyc12g0	Solyc12g0	0.00	-9.73
GO:00302	carbohydra	Molecular	0.46	0.62	Solyc03g0	Solyc03g0	5.49	2.46
GO:00052	transporter	Molecular	0.47	0.63	Solyc07g0	Solyc07g0	0.46	-1.12
GO:00052	transporter	Molecular	0.47	0.63	Solyc12g0	Solyc12g0	2112.44	11.04
GO:00052	transporter	Molecular	0.47	0.63	Solyc08g0	Solyc08g0	0.37	-1.42
GO:00052	transporter	Molecular	0.47	0.63	Solyc12g0	Solyc12g0	0.45	-1.16
GO:00070	microtubul	Biological	0.47	0.63	Solyc02g0	Solyc02g0	2.64	1.40
GO:00070	microtubul	Biological	0.47	0.63	Solyc10g0	Solyc10g0	2.44	1.29
GO:00070	microtubul	Biological	0.47	0.63	Solyc12g0	Solyc12g0	9.14	3.19
GO:00070	microtubul	Biological	0.47	0.63	Solyc03g0	Solyc03g0	0.00	-8.17
GO:00068	oligopeptic	Biological	0.48	0.63	Solyc05g0	Solyc05g0	0.40	-1.34
GO:00068	oligopeptic	Biological	0.48	0.63	Solyc09g0	Solyc09g0	0.42	-1.26
GO:00329	protein-cor	Cellular Co	0.48	0.63	Solyc09g0	Solyc09g0	0.08	-3.59
GO:00162	lipase activ	Molecular	0.48	0.63	Solyc03g0	Solyc03g0	2.46	1.30
GO:00332	eukaryotic	Cellular Co	0.48	0.63	Solyc06g0	Solyc06g0	2.11	1.07
GO:00169	ribulose-bi	Molecular	0.48	0.63	Solyc03g0	RBCS-2A	0.34	-1.58

GO:00168	strictosidir	Molecular	0.48	0.63	Solyc11g0	Solyc11g0	0.32	-1.65
GO:00048	cysteine-ty	Molecular	0.48	0.63	Solyc12g0	Solyc12g0	0.25	-2.02
GO:00458	negative re	Biological	0.48	0.63	Solyc06g0	Solyc06g0	0.34	-1.54
GO:00101	response tc	Biological	0.48	0.63	Solyc05g0	Solyc05g0	0.31	-1.69
GO:00427	D-xylose n	Biological	0.48	0.63	Solyc09g0	Solyc09g0	2.89	1.53
GO:00523	tRNA dim	Molecular	0.48	0.63	Solyc01g0	Solyc01g0	0.42	-1.26
GO:00715	zinc ion tra	Biological	0.48	0.63	Solyc02g0	Solyc02g0	0.19	-2.42
GO:00001	MAPK cas	Biological	0.48	0.63	Solyc06g0	Solyc06g0	0.48	-1.07
GO:00097	photosynth	Biological	0.48	0.63	Solyc12g0	Solyc12g0	0.00	-10.26
GO:00037	microtubul	Molecular	0.48	0.63	Solyc02g0	Solyc02g0	2.64	1.40
GO:00037	microtubul	Molecular	0.48	0.63	Solyc10g0	Solyc10g0	2.44	1.29
GO:00037	microtubul	Molecular	0.48	0.63	Solyc12g0	Solyc12g0	9.14	3.19
GO:00037	microtubul	Molecular	0.48	0.63	Solyc03g0	Solyc03g0	0.00	-8.17
GO:00459	negative re	Biological	0.51	0.65	Solyc02g0	Solyc02g0	2.65	1.40
GO:00508	cobalt ion	Molecular	0.51	0.65	Solyc02g0	Solyc02g0	2.65	1.40
GO:00305	Hsp70 pro	Molecular	0.51	0.65	Solyc08g0	Solyc08g0	0.49	-1.02
GO:00098	seed germi	Biological	0.51	0.65	Solyc09g0	Solyc09g0	0.07	-3.81
GO:00057	peroxisom	Cellular Co	0.51	0.65	Solyc02g0	Solyc02g0	2.02	1.01
GO:00060	acetyl-CoA	Biological	0.51	0.65	Solyc05g0	Solyc05g0	2.02	1.01
GO:00342	carbohydra	Biological	0.51	0.65	Solyc12g0	Solyc12g0	2.45	1.29
GO:00088	fructokinas	Molecular	0.51	0.65	Solyc11g0	Solyc11g0	0.33	-1.59
GO:00098	pollen tube	Biological	0.51	0.65	Solyc01g1	Solyc01g1	0.44	-1.20
GO:00096	salicylic ac	Biological	0.51	0.65	Solyc02g0	Solyc02g0	8.70	3.12
GO:00800	methyl ind	Molecular	0.51	0.65	Solyc02g0	Solyc02g0	8.70	3.12
GO:00800	methyl sali	Molecular	0.51	0.65	Solyc02g0	Solyc02g0	8.70	3.12
GO:00800	methyl jasi	Molecular	0.51	0.65	Solyc02g0	Solyc02g0	8.70	3.12
GO:00058	spindle	Cellular Co	0.51	0.65	Solyc05g0	Solyc05g0	2.46	1.30
GO:00351	post-transc	Biological	0.51	0.65	Solyc01g0	Solyc01g0	0.00	-10.39
GO:00450	innate imn	Biological	0.51	0.65	Solyc08g0	Solyc08g0	0.11	-3.22
GO:00055	fatty acid b	Molecular	0.51	0.65	Solyc02g0	Solyc02g0	1436.80	10.49
GO:00067	ATP biosy	Biological	0.51	0.65	Solyc11g0	Solyc11g0	0.33	-1.60
GO:00052	calcium ac	Molecular	0.51	0.65	Solyc02g0	Solyc02g0	0.43	-1.23
GO:00098	lignin bios	Biological	0.51	0.65	Solyc01g0	Solyc01g0	2.28	1.19
GO:00065	cellular an	Biological	0.52	0.66	Solyc03g0	Solyc03g0	0.00	-11.21
GO:00065	cellular an	Biological	0.52	0.66	Solyc06g0	Solyc06g0	0.30	-1.75
GO:00065	cellular an	Biological	0.52	0.66	Solyc09g0	TD2	4.53	2.18
GO:00512	NAD bindi	Molecular	0.53	0.67	Solyc12g0	Solyc12g0	0.34	-1.57
GO:00512	NAD bindi	Molecular	0.53	0.67	Solyc12g0	Solyc12g0	0.42	-1.25
GO:00512	NAD bindi	Molecular	0.53	0.67	Solyc12g0	Solyc12g0	0.34	-1.56
GO:00512	NAD bindi	Molecular	0.53	0.67	Solyc02g0	Solyc02g0	0.46	-1.12
GO:00512	NAD bindi	Molecular	0.53	0.67	Solyc12g0	Solyc12g0	0.13	-2.93
GO:00190	SCF ubiqu	Cellular Co	0.53	0.67	Solyc04g0	Solyc04g0	0.45	-1.15
GO:00190	SCF ubiqu	Cellular Co	0.53	0.67	Solyc03g0	Solyc03g0	4.40	2.14
GO:00081	sulfotransf	Molecular	0.53	0.67	Solyc12g0	Solyc12g0	0.40	-1.33
GO:00081	sulfotransf	Molecular	0.53	0.67	Solyc03g1	Solyc03g1	0.16	-2.67
GO:00309	mismatche	Molecular	0.54	0.67	Solyc02g0	Solyc02g0	2.65	1.40
GO:00069	response tc	Biological	0.54	0.67	Solyc07g0	NCED1	0.30	-1.72
GO:00089	phospholip	Molecular	0.54	0.67	Solyc01g0	Solyc01g0	2.60	1.38
GO:00061	malate met	Biological	0.54	0.67	Solyc03g0	Solyc03g0	0.46	-1.11
GO:00096	photomorp	Biological	0.54	0.67	Solyc12g0	Solyc12g0	0.44	-1.20
GO:00062	DNA meta	Biological	0.54	0.67	Solyc11g0	Solyc11g0	2.55	1.35

GO:00053	zinc ion tra	Molecular	0.54	0.67	Solyc02g0	Solyc02g0	0.19	-2.42
GO:00057	mitochond	Cellular Co	0.54	0.67	Solyc03g1	Solyc03g1	18.12	4.18
GO:00053	manganese	Molecular	0.54	0.67	Solyc04g0	Solyc04g0	0.48	-1.05
GO:00065	polyamine	Biological	0.54	0.67	Solyc08g0	Solyc08g0	2.74	1.45
GO:01403	ABC-type	Molecular	0.54	0.67	Solyc09g0	Solyc09g0	0.43	-1.22
GO:01403	ABC-type	Molecular	0.54	0.67	Solyc09g0	Solyc09g0	2.86	1.51
GO:01403	ABC-type	Molecular	0.54	0.67	Solyc02g0	Solyc02g0	0.34	-1.57
GO:01403	ABC-type	Molecular	0.54	0.67	Solyc08g0	Solyc08g0	0.20	-2.32
GO:01403	ABC-type	Molecular	0.54	0.67	Solyc06g0	Solyc06g0	2.48	1.31
GO:00056	obsolete ce	Cellular Co	0.54	0.67	Solyc07g0	Solyc07g0	2.37	1.24
GO:00056	obsolete ce	Cellular Co	0.54	0.67	Solyc11g0	Solyc11g0	0.28	-1.82
GO:00056	obsolete ce	Cellular Co	0.54	0.67	Solyc02g0	Solyc02g0	2.01	1.01
GO:00056	obsolete ce	Cellular Co	0.54	0.67	Solyc08g0	Solyc08g0	2.04	1.03
GO:00322	methylatio	Biological	0.54	0.68	Solyc08g0	Solyc08g0	2.10	1.07
GO:00322	methylatio	Biological	0.54	0.68	Solyc01g1	Solyc01g1	0.28	-1.84
GO:00322	methylatio	Biological	0.54	0.68	Solyc09g0	Solyc09g0	0.00	-8.38
GO:00166	oxidoreduc	Molecular	0.55	0.68	Solyc12g0	Solyc12g0	0.34	-1.57
GO:00166	oxidoreduc	Molecular	0.55	0.68	Solyc03g0	Solyc03g0	0.34	-1.57
GO:00036	single-stra	Molecular	0.55	0.68	Solyc09g0	Solyc09g0	2.25	1.17
GO:00036	single-stra	Molecular	0.55	0.68	Solyc01g1	Solyc01g1	2.41	1.27
GO:00301	manganese	Molecular	0.55	0.68	Solyc07g0	Solyc07g0	2.14	1.10
GO:00301	manganese	Molecular	0.55	0.68	Solyc03g1	Solyc03g1	0.11	-3.19
GO:00083	O-acyltran	Molecular	0.55	0.68	Solyc06g0	Solyc06g0	0.19	-2.40
GO:00083	O-acyltran	Molecular	0.55	0.68	Solyc12g0	Solyc12g0	0.00	-9.16
GO:00048	ubiquitin-p	Molecular	0.55	0.68	Solyc04g0	Solyc04g0	0.45	-1.15
GO:00048	ubiquitin-p	Molecular	0.55	0.68	Solyc06g0	Solyc06g0	2.41	1.27
GO:00048	ubiquitin-p	Molecular	0.55	0.68	Solyc06g0	Solyc06g0	0.49	-1.02
GO:00048	ubiquitin-p	Molecular	0.55	0.68	Solyc03g0	Solyc03g0	0.29	-1.76
GO:00048	ubiquitin-p	Molecular	0.55	0.68	Solyc06g0	Solyc06g0	6.40	2.68
GO:00048	ubiquitin-p	Molecular	0.55	0.68	Solyc01g1	Solyc01g1	0.42	-1.24
GO:00048	ubiquitin-p	Molecular	0.55	0.68	Solyc09g0	Solyc09g0	0.44	-1.19
GO:00048	ubiquitin-p	Molecular	0.55	0.68	Solyc03g1	Solyc03g1	0.09	-3.43
GO:00048	ubiquitin-p	Molecular	0.55	0.68	Solyc12g0	Solyc12g0	0.18	-2.45
GO:00099	chloroplast	Cellular Co	0.55	0.69	Solyc11g0	AOS2	0.12	-3.01
GO:00099	chloroplast	Cellular Co	0.55	0.69	Solyc05g0	Solyc05g0	2.02	1.01
GO:00099	chloroplast	Cellular Co	0.55	0.69	Solyc03g0	Solyc03g0	0.18	-2.46
GO:00099	chloroplast	Cellular Co	0.55	0.69	Solyc05g0	Solyc05g0	0.15	-2.75
GO:00099	chloroplast	Cellular Co	0.55	0.69	Solyc05g0	Solyc05g0	0.00	-11.15
GO:00099	chloroplast	Cellular Co	0.55	0.69	Solyc01g1	CAP10A	0.35	-1.51
GO:00168	isomerase	Molecular	0.56	0.69	Solyc05g0	Solyc05g0	0.45	-1.15
GO:00168	isomerase	Molecular	0.56	0.69	Solyc02g0	Solyc02g0	0.45	-1.14
GO:00168	isomerase	Molecular	0.56	0.69	Solyc02g0	Solyc02g0	1436.80	10.49
GO:00512	protein cor	Biological	0.56	0.69	Solyc04g0	Solyc04g0	0.49	-1.03
GO:00229	respiratory	Biological	0.56	0.69	Solyc04g0	Solyc04g0	0.41	-1.27
GO:00328	regulation	Biological	0.56	0.69	Solyc06g0	Solyc06g0	0.34	-1.54
GO:00060	galactose r	Biological	0.56	0.69	Solyc01g0	Solyc01g0	0.45	-1.15
GO:00325	ribonucleo	Molecular	0.56	0.69	Solyc12g0	Solyc12g0	5.61	2.49
GO:00096	jasmonic a	Biological	0.56	0.69	Solyc02g0	Solyc02g0	8.70	3.12
GO:00045	endoribonu	Molecular	0.56	0.69	Solyc05g0	Solyc05g0	0.48	-1.07
GO:00090	branched-c	Biological	0.56	0.69	Solyc09g0	TD2	4.53	2.18
GO:00482	vesicle doc	Biological	0.56	0.69	Solyc01g0	Solyc01g0	0.19	-2.40

GO:00063	DNA-temp	Biological	0.56	0.69	Solyc03g0	Solyc03g0	2.41	1.27
GO:00063	DNA-temp	Biological	0.56	0.69	Solyc06g0	Solyc06g0	2.04	1.03
GO:00094	response to	Biological	0.56	0.69	Solyc01g0	Solyc01g0	0.45	-1.15
GO:00094	response to	Biological	0.56	0.69	Solyc06g0	Solyc06g0	0.48	-1.07
GO:00087	S-adenosyl	Molecular	0.57	0.69	Solyc09g0	Solyc09g0	2.87	1.52
GO:00087	S-adenosyl	Molecular	0.57	0.69	Solyc04g0	Solyc04g0	0.27	-1.87
GO:00087	S-adenosyl	Molecular	0.57	0.69	Solyc03g0	Solyc03g0	0.04	-4.71
GO:00002	microtubul	Biological	0.58	0.71	Solyc05g0	Solyc05g0	2.46	1.30
GO:00002	microtubul	Biological	0.58	0.71	Solyc09g0	Solyc09g0	0.00	-9.38
GO:00059	sucrose me	Biological	0.58	0.71	Solyc12g0	Solyc12g0	0.40	-1.33
GO:00082	cell popula	Biological	0.58	0.71	Solyc04g0	Solyc04g0	0.46	-1.11
GO:00090	aromatic a	Biological	0.58	0.71	Solyc11g0	Solyc11g0	0.43	-1.20
GO:00157	malate tran	Biological	0.58	0.71	Solyc06g0	Solyc06g0	0.26	-1.93
GO:20000	regulation	Biological	0.58	0.71	Solyc10g0	Solyc10g0	0.46	-1.13
GO:00064	RNA catab	Biological	0.58	0.71	Solyc05g0	Solyc05g0	0.48	-1.07
GO:00167	cellulose s	Molecular	0.58	0.71	Solyc07g0	Solyc07g0	0.44	-1.18
GO:00009	cis-regulat	Molecular	0.58	0.71	Solyc01g1	Solyc01g1	0.15	-2.78
GO:00313	integral co	Cellular Co	0.58	0.71	Solyc12g0	Solyc12g0	0.46	-1.12
GO:00097	response to	Biological	0.59	0.72	Solyc02g0	Solyc02g0	0.23	-2.13
GO:00097	response to	Biological	0.59	0.72	Solyc01g0	Solyc01g0	0.42	-1.26
GO:00096	gibberellin	Biological	0.61	0.73	Solyc07g0	Solyc07g0	0.17	-2.54
GO:00057	lysosome	Cellular Co	0.61	0.73	Solyc11g0	Solyc11g0	0.46	-1.12
GO:00442	cellular me	Biological	0.61	0.73	Solyc09g0	Solyc09g0	3.19	1.67
GO:20003	regulation	Biological	0.61	0.73	Solyc04g0	Solyc04g0	0.34	-1.54
GO:00103	stomatal co	Biological	0.61	0.73	Solyc04g0	Solyc04g0	2.27	1.18
GO:00046	cyclin-dep	Molecular	0.61	0.73	Solyc03g0	Solyc03g0	0.48	-1.05
GO:00096	anatomical	Biological	0.61	0.73	Solyc10g0	Solyc10g0	2.57	1.36
GO:00100	trichome n	Biological	0.61	0.73	Solyc03g0	Solyc03g0	16.58	4.05
GO:00301	integral co	Cellular Co	0.61	0.73	Solyc04g0	Solyc04g0	0.46	-1.13
GO:00301	integral co	Cellular Co	0.61	0.73	Solyc01g0	Solyc01g0	0.32	-1.64
GO:00311	SCF-deper	Biological	0.61	0.73	Solyc04g0	Solyc04g0	0.45	-1.15
GO:00311	SCF-deper	Biological	0.61	0.73	Solyc03g0	Solyc03g0	4.40	2.14
GO:00081	methyltran	Molecular	0.61	0.73	Solyc03g0	Solyc03g0	3.27	1.71
GO:00081	methyltran	Molecular	0.61	0.73	Solyc06g0	Solyc06g0	2.55	1.35
GO:00081	methyltran	Molecular	0.61	0.73	Solyc09g0	Solyc09g0	2.87	1.52
GO:00081	methyltran	Molecular	0.61	0.73	Solyc03g0	Solyc03g0	0.33	-1.60
GO:00081	methyltran	Molecular	0.61	0.73	Solyc08g0	Solyc08g0	2.10	1.07
GO:00081	methyltran	Molecular	0.61	0.73	Solyc01g1	Solyc01g1	0.28	-1.84
GO:00081	methyltran	Molecular	0.61	0.73	Solyc02g0	Solyc02g0	0.49	-1.02
GO:00081	methyltran	Molecular	0.61	0.73	Solyc01g0	Solyc01g0	2.19	1.13
GO:00081	methyltran	Molecular	0.61	0.73	Solyc01g1	Solyc01g1	2.42	1.28
GO:00081	methyltran	Molecular	0.61	0.73	Solyc09g0	Solyc09g0	0.00	-8.38
GO:00159	ATP synth	Biological	0.62	0.74	Solyc06g0	Solyc06g0	3.71	1.89
GO:00159	ATP synth	Biological	0.62	0.74	Solyc11g0	Solyc11g0	0.33	-1.60
GO:00015	RNA meth	Biological	0.63	0.74	Solyc03g0	Solyc03g0	0.33	-1.60
GO:00101	abaxial cel	Biological	0.63	0.74	Solyc11g0	Solyc11g0	0.19	-2.40
GO:00055	calcium-de	Molecular	0.63	0.74	Solyc01g0	Solyc01g0	0.23	-2.14
GO:00460	ATP metal	Biological	0.63	0.74	Solyc11g0	Solyc11g0	0.33	-1.60
GO:00047	MAP kinas	Molecular	0.63	0.74	Solyc06g0	Solyc06g0	0.48	-1.07
GO:00166	oxidoreduc	Molecular	0.63	0.74	Solyc03g0	Solyc03g0	0.34	-1.57
GO:00985	defense res	Biological	0.63	0.75	Solyc09g0	Solyc09g0	0.35	-1.51

GO:00985	defense re:	Biological	0.63	0.75	Solyc11g0	Solyc11g0	0.23	-2.13
GO:00985	defense re:	Biological	0.63	0.75	Solyc10g0	Solyc10g0	0.49	-1.03
GO:00985	defense re:	Biological	0.63	0.75	Solyc05g0	Solyc05g0	0.13	-3.00
GO:00985	defense re:	Biological	0.63	0.75	Solyc01g0	Solyc01g0	0.00	-11.90
GO:00985	defense re:	Biological	0.63	0.75	Solyc06g0	Solyc06g0	0.26	-1.94
GO:00985	defense re:	Biological	0.63	0.75	Solyc11g0	Solyc11g0	4.59	2.20
GO:00082	cysteine-ty	Molecular	0.63	0.75	Solyc11g0	Solyc11g0	0.46	-1.12
GO:00082	cysteine-ty	Molecular	0.63	0.75	Solyc05g0	Solyc05g0	2.08	1.06
GO:00082	cysteine-ty	Molecular	0.63	0.75	Solyc12g0	Solyc12g0	0.35	-1.50
GO:00082	cysteine-ty	Molecular	0.63	0.75	Solyc12g0	Solyc12g0	2321.45	11.18
GO:00506	flavin ader	Molecular	0.64	0.75	Solyc03g0	Solyc03g0	2.69	1.43
GO:00506	flavin ader	Molecular	0.64	0.75	Solyc11g0	Solyc11g0	9.10	3.19
GO:00506	flavin ader	Molecular	0.64	0.75	Solyc01g0	Solyc01g0	0.38	-1.39
GO:00506	flavin ader	Molecular	0.64	0.75	Solyc02g0	Solyc02g0	5.84	2.55
GO:00506	flavin ader	Molecular	0.64	0.75	Solyc06g0	Solyc06g0	2.01	1.01
GO:00506	flavin ader	Molecular	0.64	0.75	Solyc09g0	Solyc09g0	3.02	1.59
GO:00163	phosphory	Biological	0.65	0.76	Solyc02g0	Solyc02g0	0.47	-1.10
GO:00163	phosphory	Biological	0.65	0.76	Solyc07g0	Solyc07g0	0.02	-5.66
GO:00163	phosphory	Biological	0.65	0.76	Solyc11g0	Solyc11g0	2.55	1.35
GO:00163	phosphory	Biological	0.65	0.76	Solyc11g0	Solyc11g0	0.33	-1.61
GO:00163	phosphory	Biological	0.65	0.76	Solyc03g1	Solyc03g1	2479.17	11.28
GO:00163	phosphory	Biological	0.65	0.76	Solyc02g0	Solyc02g0	0.16	-2.63
GO:00163	phosphory	Biological	0.65	0.76	Solyc06g0	Solyc06g0	0.48	-1.07
GO:00163	phosphory	Biological	0.65	0.76	Solyc03g1	Solyc03g1	0.34	-1.57
GO:00163	phosphory	Biological	0.65	0.76	Solyc06g0	Solyc06g0	0.26	-1.94
GO:00095	chloroplast	Cellular C	0.65	0.76	Solyc05g0	Solyc05g0	2.02	1.01
GO:00704	respirasom	Cellular C	0.65	0.76	Solyc08g0	Solyc08g0	0.19	-2.37
GO:00468	phosphatid	Biological	0.65	0.76	Solyc02g0	Solyc02g0	0.49	-1.03
GO:00044	N,N-dimet	Molecular	0.65	0.76	Solyc06g0	Solyc06g0	2.01	1.01
GO:00001	protein ph	Cellular C	0.65	0.76	Solyc06g0	Solyc06g0	0.33	-1.61
GO:00150	glucuronos	Molecular	0.65	0.76	Solyc09g0	Solyc09g0	0.34	-1.55
GO:00097	response tc	Biological	0.65	0.76	Solyc12g0	Solyc12g0	0.40	-1.34
GO:00084	transamina	Molecular	0.65	0.76	Solyc01g0	Solyc01g0	0.47	-1.10
GO:00084	transamina	Molecular	0.65	0.76	Solyc03g0	Solyc03g0	0.00	-11.21
GO:00097	cytokinin-a	Biological	0.66	0.77	Solyc06g0	Solyc06g0	2.00	1.00
GO:00097	cytokinin-a	Biological	0.66	0.77	Solyc05g0	Solyc05g0	0.49	-1.03
GO:00525	cell wall p	Biological	0.67	0.77	Solyc09g0	Solyc09g0	2.87	1.52
GO:00055	phospholip	Molecular	0.67	0.77	Solyc04g0	Solyc04g0	0.34	-1.54
GO:00482	clathrin co	Biological	0.67	0.77	Solyc04g0	Solyc04g0	0.34	-1.54
GO:00068	endocytosi	Biological	0.67	0.77	Solyc11g0	Solyc11g0	0.49	-1.03
GO:00070	actin filam	Biological	0.67	0.77	Solyc11g0	Solyc11g0	0.04	-4.78
GO:01101	cellular an	Cellular C	0.67	0.77	Solyc08g0	Solyc08g0	311.75	8.28
GO:00060	glycolytic	Biological	0.67	0.78	Solyc02g0	Solyc02g0	0.45	-1.16
GO:00060	glycolytic	Biological	0.67	0.78	Solyc01g1	Solyc01g1	0.31	-1.69
GO:00060	glycolytic	Biological	0.67	0.78	Solyc11g0	Solyc11g0	0.33	-1.59
GO:01406	ATP-deper	Molecular	0.67	0.78	Solyc01g0	Solyc01g0	3.11	1.64
GO:01406	ATP-deper	Molecular	0.67	0.78	Solyc03g0	Solyc03g0	2.30	1.20
GO:01406	ATP-deper	Molecular	0.67	0.78	Solyc04g0	Solyc04g0	2.12	1.08
GO:00037	actin bindi	Molecular	0.68	0.78	Solyc06g0	Solyc06g0	0.47	-1.09
GO:00037	actin bindi	Molecular	0.68	0.78	Solyc01g0	Solyc01g0	0.11	-3.14
GO:00037	actin bindi	Molecular	0.68	0.78	Solyc06g0	Solyc06g0	0.36	-1.46

GO:00001	phosphorel	Biological	0.68	0.78	Solyc06g0	Solyc06g0	2.00	1.00
GO:00001	phosphorel	Biological	0.68	0.78	Solyc05g0	Solyc05g0	0.49	-1.03
GO:00001	phosphorel	Biological	0.68	0.78	Solyc06g0	Solyc06g0	0.42	-1.25
GO:00058	eukaryotic	Cellular Co	0.68	0.78	Solyc06g0	Solyc06g0	2.11	1.07
GO:00066	fatty acid b	Biological	0.68	0.78	Solyc08g0	Solyc08g0	0.21	-2.27
GO:00041	endopeptic	Molecular	0.68	0.78	Solyc04g0	Solyc04g0	0.18	-2.44
GO:00001	ubiquitin li	Cellular Co	0.68	0.78	Solyc06g0	Solyc06g0	6.40	2.68
GO:00058	proteasom	Cellular Co	0.68	0.78	Solyc03g0	Solyc03g0	25578.61	14.64
GO:00614	positive re	Biological	0.68	0.78	Solyc07g0	Solyc07g0	0.07	-3.80
GO:00099	flower dev	Biological	0.68	0.78	Solyc10g0	Solyc10g0	0.32	-1.63
GO:00069	vesicle fus	Biological	0.68	0.78	Solyc01g0	Solyc01g0	0.19	-2.40
GO:00059	trehalose b	Biological	0.68	0.78	Solyc06g0	Solyc06g0	379.31	8.57
GO:00104	regulation	Biological	0.69	0.79	Solyc07g0	Solyc07g0	0.25	-1.97
GO:00104	regulation	Biological	0.69	0.79	Solyc06g0	Solyc06g0	0.48	-1.07
GO:00081	transcriptio	Molecular	0.69	0.79	Solyc04g0	Solyc04g0	2.02	1.01
GO:00081	transcriptio	Molecular	0.69	0.79	Solyc03g1	Solyc03g1	2.90	1.54
GO:00081	transcriptio	Molecular	0.69	0.79	Solyc01g1	Solyc01g1	0.15	-2.78
GO:00324	Rab protei	Biological	0.70	0.79	Solyc09g0	Solyc09g0	0.33	-1.60
GO:00324	Rab protei	Biological	0.70	0.79	Solyc05g0	Solyc05g0	0.43	-1.23
GO:00062	mismatch r	Biological	0.70	0.79	Solyc02g0	Solyc02g0	2.65	1.40
GO:00045	ribonuclea	Molecular	0.70	0.79	Solyc01g0	Solyc01g0	0.33	-1.59
GO:00066	glycerol et	Biological	0.70	0.79	Solyc02g0	Solyc02g0	2.01	1.01
GO:00004	DNA-direc	Cellular Co	0.70	0.79	Solyc12g0	Solyc12g0	5.61	2.49
GO:00058	centrosom	Cellular Co	0.70	0.79	Solyc01g1	Solyc01g1	2.11	1.08
GO:00159	phospholip	Biological	0.70	0.79	Solyc01g1	Solyc01g1	7.06	2.82
GO:00080	phosphoric	Molecular	0.70	0.79	Solyc06g0	Solyc06g0	2.69	1.43
GO:00198	protein pho	Molecular	0.70	0.79	Solyc06g0	Solyc06g0	0.33	-1.61
GO:00099	regulation	Biological	0.70	0.79	Solyc01g0	Solyc01g0	0.12	-3.05
GO:00097	photosynth	Biological	0.70	0.79	Solyc05g0	Solyc05g0	0.46	-1.12
GO:00102	vegetative	Biological	0.70	0.79	Solyc01g0	Solyc01g0	0.42	-1.24
GO:00064	mRNA cat	Biological	0.72	0.81	Solyc06g0	Solyc06g0	0.45	-1.14
GO:00094	response to	Biological	0.72	0.81	Solyc04g0	Solyc04g0	0.49	-1.03
GO:00094	response to	Biological	0.72	0.81	Solyc01g0	Solyc01g0	0.45	-1.15
GO:00068	exocytosis	Biological	0.72	0.81	Solyc06g0	Solyc06g0	0.14	-2.87
GO:00068	exocytosis	Biological	0.72	0.81	Solyc01g0	Solyc01g0	0.19	-2.40
GO:00310	stress-activ	Biological	0.72	0.81	Solyc02g0	Solyc02g0	0.36	-1.48
GO:00310	stress-activ	Biological	0.72	0.81	Solyc02g0	Solyc02g0	0.44	-1.18
GO:00196	photosynth	Biological	0.72	0.81	Solyc05g0	Solyc05g0	0.46	-1.12
GO:00196	photosynth	Biological	0.72	0.81	Solyc03g0	Solyc03g0	0.34	-1.57
GO:00072	multicellul	Biological	0.73	0.82	Solyc11g0	Solyc11g0	0.19	-2.40
GO:00072	multicellul	Biological	0.73	0.82	Solyc02g0	Solyc02g0	0.36	-1.49
GO:00072	multicellul	Biological	0.73	0.82	Solyc01g0	Solyc01g0	0.29	-1.77
GO:00072	multicellul	Biological	0.73	0.82	Solyc01g1	Solyc01g1	0.15	-2.78
GO:00436	protein sel	Molecular	0.73	0.82	Solyc04g0	Solyc04g0	0.49	-1.03
GO:00056	nucleoplas	Cellular Co	0.73	0.82	Solyc01g0	Solyc01g0	2.42	1.27
GO:00064	tRNA mod	Biological	0.73	0.82	Solyc01g0	Solyc01g0	0.42	-1.26
GO:00083	RNA splic	Biological	0.73	0.82	Solyc05g0	Solyc05g0	0.00	-11.22
GO:00165	protein ubi	Biological	0.74	0.82	Solyc06g0	Solyc06g0	0.49	-1.02
GO:00165	protein ubi	Biological	0.74	0.82	Solyc03g0	Solyc03g0	0.29	-1.76
GO:00165	protein ubi	Biological	0.74	0.82	Solyc06g0	Solyc06g0	6.40	2.68
GO:00165	protein ubi	Biological	0.74	0.82	Solyc01g1	Solyc01g1	0.42	-1.24

GO:00165	protein ubi	Biological	0.74	0.82	Solyc09g0	Solyc09g0	0.44	-1.19
GO:00165	protein ubi	Biological	0.74	0.82	Solyc05g0	Solyc05g0	2.31	1.21
GO:00165	protein ubi	Biological	0.74	0.82	Solyc03g1	Solyc03g1	0.09	-3.43
GO:00165	protein ubi	Biological	0.74	0.82	Solyc02g0	Solyc02g0	7.32	2.87
GO:00165	protein ubi	Biological	0.74	0.82	Solyc12g0	Solyc12g0	0.18	-2.45
GO:00165	protein ubi	Biological	0.74	0.82	Solyc03g0	Solyc03g0	4.40	2.14
GO:00165	protein ubi	Biological	0.74	0.82	Solyc11g0	Solyc11g0	0.00	-9.98
GO:00095	chloroplas	Cellular C	0.74	0.83	Solyc07g0	Solyc07g0	0.47	-1.09
GO:00095	chloroplas	Cellular C	0.74	0.83	Solyc07g0	NCED1	0.30	-1.72
GO:00095	chloroplas	Cellular C	0.74	0.83	Solyc05g0	Solyc05g0	2.02	1.01
GO:00095	chloroplas	Cellular C	0.74	0.83	Solyc05g0	Solyc05g0	0.31	-1.69
GO:00095	chloroplas	Cellular C	0.74	0.83	Solyc05g0	Solyc05g0	0.42	-1.25
GO:00095	chloroplas	Cellular C	0.74	0.83	Solyc02g0	Solyc02g0	1436.80	10.49
GO:00095	chloroplas	Cellular C	0.74	0.83	Solyc10g0	Solyc10g0	0.24	-2.04
GO:00152	water chan	Molecular	0.75	0.83	Solyc06g0	Solyc06g0	0.37	-1.44
GO:00097	photosynth	Biological	0.75	0.83	Solyc02g0	Solyc02g0	0.35	-1.53
GO:00083	cation tran	Molecular	0.75	0.83	Solyc01g0	Solyc01g0	0.50	-1.01
GO:00161	terpenoid b	Biological	0.75	0.83	Solyc11g0	Solyc11g0	0.38	-1.39
GO:00150	potassium	Molecular	0.75	0.83	Solyc06g0	Solyc06g0	366.52	8.52
GO:00150	DNA integ	Biological	0.76	0.84	Solyc02g0	Solyc02g0	4.29	2.10
GO:00068	intra-Golgi	Biological	0.76	0.84	Solyc08g0	Solyc08g0	2.03	1.02
GO:00905	RNA phos	Biological	0.76	0.84	Solyc01g0	Solyc01g0	0.33	-1.59
GO:00345	cellular res	Biological	0.76	0.84	Solyc02g0	Solyc02g0	2.89	1.53
GO:00058	kinesin cor	Cellular C	0.76	0.84	Solyc10g0	Solyc10g0	2.44	1.29
GO:00057	vacuolar m	Cellular C	0.76	0.84	Solyc03g0	Solyc03g0	2.26	1.17
GO:00057	vacuolar m	Cellular C	0.76	0.84	Solyc09g0	Solyc09g0	2.87	1.52
GO:00057	vacuolar m	Cellular C	0.76	0.84	Solyc09g0	Solyc09g0	0.43	-1.22
GO:00057	vacuolar m	Cellular C	0.76	0.84	Solyc02g0	Solyc02g0	0.27	-1.90
GO:00056	intracellula	Cellular C	0.76	0.84	Solyc12g0	Solyc12g0	2.53	1.34
GO:00056	intracellula	Cellular C	0.76	0.84	Solyc09g0	Solyc09g0	0.33	-1.60
GO:00056	intracellula	Cellular C	0.76	0.84	Solyc06g0	Solyc06g0	2.00	1.00
GO:00056	intracellula	Cellular C	0.76	0.84	Solyc12g0	Solyc12g0	2.48	1.31
GO:00056	intracellula	Cellular C	0.76	0.84	Solyc05g0	Solyc05g0	0.43	-1.23
GO:00037	translation	Molecular	0.77	0.85	Solyc11g0	Solyc11g0	0.13	-2.91
GO:00468	inositol ph	Biological	0.77	0.85	Solyc02g0	Solyc02g0	0.49	-1.03
GO:00150	protein-dis	Molecular	0.78	0.85	Solyc11g0	Solyc11g0	0.28	-1.82
GO:00150	protein-dis	Molecular	0.78	0.85	Solyc02g0	Solyc02g0	2.01	1.01
GO:00150	protein-dis	Molecular	0.78	0.85	Solyc08g0	Solyc08g0	2.04	1.03
GO:00151	carbohydra	Molecular	0.78	0.86	Solyc12g0	Solyc12g0	2.45	1.29
GO:00037	transcriptio	Molecular	0.78	0.86	Solyc04g0	Solyc04g0	0.46	-1.11
GO:00466	response to	Biological	0.78	0.86	Solyc12g0	Solyc12g0	0.40	-1.34
GO:00001	SNARE bi	Molecular	0.78	0.86	Solyc01g0	Solyc01g0	0.19	-2.40
GO:00125	endomemb	Cellular C	0.78	0.86	Solyc01g0	Solyc01g0	0.19	-2.40
GO:00454	cell redox	Biological	0.79	0.86	Solyc07g0	Solyc07g0	2.37	1.24
GO:00454	cell redox	Biological	0.79	0.86	Solyc11g0	Solyc11g0	0.28	-1.82
GO:00454	cell redox	Biological	0.79	0.86	Solyc02g0	Solyc02g0	2.01	1.01
GO:00454	cell redox	Biological	0.79	0.86	Solyc08g0	Solyc08g0	2.04	1.03
GO:00301	cell differe	Biological	0.79	0.86	Solyc01g0	Solyc01g0	0.04	-4.49
GO:00301	cell differe	Biological	0.79	0.86	Solyc06g0	Solyc06g0	0.39	-1.36
GO:00301	cell differe	Biological	0.79	0.86	Solyc02g0	Solyc02g0	0.00	-9.32
GO:00435	ADP bindi	Molecular	0.79	0.86	Solyc01g1	Solyc01g1	0.08	-3.71

GO:00435	ADP bindi	Molecular	0.79	0.86	Solyc05g0	Solyc05g0	2.15	1.10
GO:00435	ADP bindi	Molecular	0.79	0.86	Solyc09g0	Solyc09g0	0.35	-1.51
GO:00435	ADP bindi	Molecular	0.79	0.86	Solyc11g0	Solyc11g0	0.23	-2.13
GO:00435	ADP bindi	Molecular	0.79	0.86	Solyc04g0	Solyc04g0	0.17	-2.57
GO:00435	ADP bindi	Molecular	0.79	0.86	Solyc05g0	Solyc05g0	0.13	-3.00
GO:00435	ADP bindi	Molecular	0.79	0.86	Solyc09g0	Solyc09g0	0.00	-9.29
GO:00435	ADP bindi	Molecular	0.79	0.86	Solyc11g0	Solyc11g0	0.33	-1.60
GO:00435	ADP bindi	Molecular	0.79	0.86	Solyc07g0	Solyc07g0	5.85	2.55
GO:00435	ADP bindi	Molecular	0.79	0.86	Solyc04g0	Solyc04g0	607.22	9.25
GO:00435	ADP bindi	Molecular	0.79	0.86	Solyc02g0	Solyc02g0	0.42	-1.25
GO:00068	nitrogen cc	Biological	0.80	0.86	Solyc03g0	Solyc03g0	2.69	1.43
GO:00064	translation	Biological	0.80	0.86	Solyc11g0	Solyc11g0	0.13	-2.91
GO:00084	3'-5' exonu	Molecular	0.80	0.86	Solyc09g0	Solyc09g0	5.78	2.53
GO:00422	ribosome b	Biological	0.81	0.88	Solyc09g0	Solyc09g0	6.55	2.71
GO:00063	mRNA prc	Biological	0.81	0.88	Solyc09g0	Solyc09g0	6.02	2.59
GO:00063	mRNA prc	Biological	0.81	0.88	Solyc05g0	Solyc05g0	0.00	-11.22
GO:00097	response tc	Biological	0.81	0.88	Solyc11g0	Solyc11g0	0.11	-3.13
GO:00097	response tc	Biological	0.81	0.88	Solyc07g0	NCED1	0.30	-1.72
GO:00097	response tc	Biological	0.81	0.88	Solyc11g0	Solyc11g0	0.47	-1.08
GO:00097	response tc	Biological	0.81	0.88	Solyc01g0	Solyc01g0	0.42	-1.26
GO:00097	response tc	Biological	0.81	0.88	Solyc12g0	Solyc12g0	0.40	-1.34
GO:00046	polygalacti	Molecular	0.82	0.88	Solyc05g0	Solyc05g0	58.00	5.86
GO:00046	polygalacti	Molecular	0.82	0.88	Solyc04g0	Solyc04g0	4.34	2.12
GO:00469	protein din	Molecular	0.82	0.88	Solyc03g0	Solyc03g0	3.27	1.71
GO:00469	protein din	Molecular	0.82	0.88	Solyc06g0	Solyc06g0	2.55	1.35
GO:00469	protein din	Molecular	0.82	0.88	Solyc08g0	Solyc08g0	0.50	-1.01
GO:00469	protein din	Molecular	0.82	0.88	Solyc10g0	Solyc10g0	0.45	-1.16
GO:00469	protein din	Molecular	0.82	0.88	Solyc09g0	Solyc09g0	0.47	-1.10
GO:00469	protein din	Molecular	0.82	0.88	Solyc09g0	Solyc09g0	2.92	1.55
GO:00469	protein din	Molecular	0.82	0.88	Solyc02g0	Solyc02g0	0.49	-1.02
GO:00469	protein din	Molecular	0.82	0.88	Solyc04g0	Solyc04g0	2.02	1.01
GO:00469	protein din	Molecular	0.82	0.88	Solyc05g0	Solyc05g0	0.00	-11.04
GO:00469	protein din	Molecular	0.82	0.88	Solyc10g0	Solyc10g0	13.20	3.72
GO:00469	protein din	Molecular	0.82	0.88	Solyc02g0	Solyc02g0	0.00	-11.41
GO:00469	protein din	Molecular	0.82	0.88	Solyc11g0	Solyc11g0	0.13	-2.94
GO:00469	protein din	Molecular	0.82	0.88	Solyc02g0	Solyc02g0	0.34	-1.56
GO:00469	protein din	Molecular	0.82	0.88	Solyc03g1	Solyc03g1	0.47	-1.08
GO:00469	protein din	Molecular	0.82	0.88	Solyc01g1	Solyc01g1	0.15	-2.78
GO:00469	protein din	Molecular	0.82	0.88	Solyc08g0	Solyc08g0	3.83	1.94
GO:00469	protein din	Molecular	0.82	0.88	Solyc10g0	Solyc10g0	1745.96	10.77
GO:00469	protein din	Molecular	0.82	0.88	Solyc10g0	Solyc10g0	2.87	1.52
GO:00469	protein din	Molecular	0.82	0.88	Solyc03g0	Solyc03g0	0.21	-2.26
GO:00469	protein din	Molecular	0.82	0.88	Solyc01g0	Solyc01g0	12.75	3.67
GO:00469	protein din	Molecular	0.82	0.88	Solyc09g0	Solyc09g0	2.52	1.33
GO:00469	protein din	Molecular	0.82	0.88	Solyc12g0	Solyc12g0	485.70	8.92
GO:00469	protein din	Molecular	0.82	0.88	Solyc10g0	Solyc10g0	0.35	-1.51
GO:00021	cytoplasmi	Biological	0.82	0.88	Solyc09g0	Solyc09g0	6.55	2.71
GO:00057	endoplasm	Cellular Co	0.82	0.89	Solyc02g0	Solyc02g0	2.02	1.01
GO:00057	endoplasm	Cellular Co	0.82	0.89	Solyc01g0	Solyc01g0	0.43	-1.21
GO:00057	endoplasm	Cellular Co	0.82	0.89	Solyc02g0	Solyc02g0	0.36	-1.49
GO:00057	endoplasm	Cellular Co	0.82	0.89	Solyc04g0	Solyc04g0	0.45	-1.14

GO:00057	endoplasm	Cellular C	0.82	0.89	Solyc07g0	Solyc07g0	0.20	-2.33
GO:00513	cell divisic	Biological	0.83	0.89	Solyc06g0	Solyc06g0	2.52	1.33
GO:00097	response tc	Biological	0.83	0.89	Solyc03g1	Solyc03g1	0.08	-3.61
GO:00036	double-stra	Molecular	0.83	0.89	Solyc01g1	Solyc01g1	2.41	1.27
GO:00036	double-stra	Molecular	0.83	0.89	Solyc10g0	Solyc10g0	0.15	-2.72
GO:00321	activation	Biological	0.83	0.89	Solyc02g0	Solyc02g0	0.36	-1.48
GO:00321	activation	Biological	0.83	0.89	Solyc02g0	Solyc02g0	0.44	-1.18
GO:00057	cytoplasm	Cellular C	0.83	0.89	Solyc09g0	Solyc09g0	0.08	-3.59
GO:00057	cytoplasm	Cellular C	0.83	0.89	Solyc12g0	Solyc12g0	0.28	-1.84
GO:00057	cytoplasm	Cellular C	0.83	0.89	Solyc09g0	Solyc09g0	0.07	-3.81
GO:00057	cytoplasm	Cellular C	0.83	0.89	Solyc09g0	Solyc09g0	0.09	-3.50
GO:00057	cytoplasm	Cellular C	0.83	0.89	Solyc08g0	Solyc08g0	0.27	-1.87
GO:00057	cytoplasm	Cellular C	0.83	0.89	Solyc11g0	Solyc11g0	0.13	-2.91
GO:00057	cytoplasm	Cellular C	0.83	0.89	Solyc06g0	Solyc06g0	0.02	-5.35
GO:00057	cytoplasm	Cellular C	0.83	0.89	Solyc01g0	Solyc01g0	0.31	-1.69
GO:00057	cytoplasm	Cellular C	0.83	0.89	Solyc07g0	Solyc07g0	0.46	-1.12
GO:00057	cytoplasm	Cellular C	0.83	0.89	Solyc06g0	Solyc06g0	0.44	-1.19
GO:00057	cytoplasm	Cellular C	0.83	0.89	Solyc10g0	Solyc10g0	0.47	-1.09
GO:00057	cytoplasm	Cellular C	0.83	0.89	Solyc05g0	Solyc05g0	0.37	-1.44
GO:00057	cytoplasm	Cellular C	0.83	0.89	Solyc04g0	Solyc04g0	0.18	-2.44
GO:00057	cytoplasm	Cellular C	0.83	0.89	Solyc05g0	Solyc05g0	0.45	-1.15
GO:00057	cytoplasm	Cellular C	0.83	0.89	Solyc01g0	Solyc01g0	0.29	-1.78
GO:00057	cytoplasm	Cellular C	0.83	0.89	Solyc12g0	Solyc12g0	0.32	-1.65
GO:00057	cytoplasm	Cellular C	0.83	0.89	Solyc04g0	Solyc04g0	0.45	-1.15
GO:00057	cytoplasm	Cellular C	0.83	0.89	Solyc11g0	Solyc11g0	3.98	1.99
GO:00057	cytoplasm	Cellular C	0.83	0.89	Solyc06g0	Solyc06g0	0.41	-1.28
GO:00057	cytoplasm	Cellular C	0.83	0.89	Solyc02g0	Solyc02g0	0.49	-1.03
GO:00057	cytoplasm	Cellular C	0.83	0.89	Solyc09g0	Solyc09g0	2.89	1.53
GO:00057	cytoplasm	Cellular C	0.83	0.89	Solyc11g0	Solyc11g0	2.74	1.45
GO:00057	cytoplasm	Cellular C	0.83	0.89	Solyc01g1	Solyc01g1	2.11	1.08
GO:00057	cytoplasm	Cellular C	0.83	0.89	Solyc01g0	Solyc01g0	0.00	-12.11
GO:00057	cytoplasm	Cellular C	0.83	0.89	Solyc01g1	Solyc01g1	0.44	-1.20
GO:00057	cytoplasm	Cellular C	0.83	0.89	Solyc11g0	Solyc11g0	0.33	-1.61
GO:00057	cytoplasm	Cellular C	0.83	0.89	Solyc03g1	Solyc03g1	0.00	-11.76
GO:00057	cytoplasm	Cellular C	0.83	0.89	Solyc09g0	Solyc09g0	0.34	-1.56
GO:00057	cytoplasm	Cellular C	0.83	0.89	Solyc01g0	Solyc01g0	0.00	-10.39
GO:00057	cytoplasm	Cellular C	0.83	0.89	Solyc02g0	Solyc02g0	0.36	-1.48
GO:00057	cytoplasm	Cellular C	0.83	0.89	Solyc02g0	Solyc02g0	8.65	3.11
GO:00057	cytoplasm	Cellular C	0.83	0.89	Solyc01g0	Solyc01g0	0.50	-1.01
GO:00057	cytoplasm	Cellular C	0.83	0.89	Solyc04g0	Solyc04g0	2.93	1.55
GO:00057	cytoplasm	Cellular C	0.83	0.89	Solyc03g1	Solyc03g1	0.09	-3.43
GO:00057	cytoplasm	Cellular C	0.83	0.89	Solyc07g0	Solyc07g0	2.89	1.53
GO:00057	cytoplasm	Cellular C	0.83	0.89	Solyc04g0	Solyc04g0	2.18	1.13
GO:00057	cytoplasm	Cellular C	0.83	0.89	Solyc06g0	Solyc06g0	0.48	-1.07
GO:00057	cytoplasm	Cellular C	0.83	0.89	Solyc04g0	Solyc04g0	4.79	2.26
GO:00057	cytoplasm	Cellular C	0.83	0.89	Solyc05g0	Solyc05g0	0.48	-1.07
GO:00057	cytoplasm	Cellular C	0.83	0.89	Solyc03g1	Solyc03g1	0.34	-1.57
GO:00057	cytoplasm	Cellular C	0.83	0.89	Solyc01g0	Solyc01g0	2.66	1.41
GO:00057	cytoplasm	Cellular C	0.83	0.89	Solyc08g0	Solyc08g0	0.07	-3.82
GO:00057	cytoplasm	Cellular C	0.83	0.89	Solyc09g0	Solyc09g0	6.02	2.59
GO:00057	cytoplasm	Cellular C	0.83	0.89	Solyc02g0	Solyc02g0	0.49	-1.03

GO:00057	cytoplasm	Cellular C	0.83	0.89	Solyc06g0	Solyc06g0	0.39	-1.36
GO:00057	cytoplasm	Cellular C	0.83	0.89	Solyc05g0	Solyc05g0	0.00	-11.22
GO:00057	cytoplasm	Cellular C	0.83	0.89	Solyc02g0	Solyc02g0	0.44	-1.18
GO:00057	cytoplasm	Cellular C	0.83	0.89	Solyc11g0	Solyc11g0	0.00	-9.98
GO:00057	cytoplasm	Cellular C	0.83	0.89	Solyc05g0	Solyc05g0	0.00	-8.91
GO:00057	cytoplasm	Cellular C	0.83	0.89	Solyc01g0	Solyc01g0	0.23	-2.09
GO:00058	microtubul	Cellular C	0.83	0.89	Solyc05g0	Solyc05g0	2.46	1.30
GO:00058	microtubul	Cellular C	0.83	0.89	Solyc10g0	Solyc10g0	2.44	1.29
GO:00058	microtubul	Cellular C	0.83	0.89	Solyc12g0	Solyc12g0	9.14	3.19
GO:00063	chromatin	Biological	0.84	0.90	Solyc01g0	Solyc01g0	3.68	1.88
GO:00090	aerobic res	Biological	0.85	0.90	Solyc04g0	Solyc04g0	0.41	-1.27
GO:00616	ubiquitin c	Molecular	0.85	0.90	Solyc06g0	Solyc06g0	0.49	-1.02
GO:00163	dephospho	Biological	0.85	0.90	Solyc02g0	Solyc02g0	0.49	-1.03
GO:00164	O-acetyltra	Molecular	0.85	0.90	Solyc01g0	Solyc01g0	2.66	1.41
GO:00168	ligase acti	Molecular	0.85	0.91	Solyc03g0	Solyc03g0	0.29	-1.76
GO:00167	cellulose s	Molecular	0.85	0.91	Solyc07g0	Solyc07g0	0.44	-1.18
GO:00001	exocyst	Cellular C	0.86	0.91	Solyc06g0	Solyc06g0	0.14	-2.87
GO:00905	RNA phos	Biological	0.86	0.91	Solyc05g0	Solyc05g0	0.48	-1.07
GO:00197	calcium-m	Biological	0.86	0.91	Solyc06g0	Solyc06g0	3.20	1.68
GO:00312	SNARE cc	Cellular C	0.86	0.91	Solyc01g0	Solyc01g0	0.19	-2.40
GO:00009	RNA poly	Molecular	0.86	0.91	Solyc02g0	Solyc02g0	0.00	-11.41
GO:00009	RNA poly	Molecular	0.86	0.91	Solyc10g0	Solyc10g0	1745.96	10.77
GO:00068	retrograde	Biological	0.87	0.92	Solyc09g0	Solyc09g0	2.28	1.19
GO:00468	metal ion t	Molecular	0.87	0.92	Solyc02g0	Solyc02g0	0.19	-2.42
GO:00469	proton-trar	Molecular	0.87	0.92	Solyc11g0	Solyc11g0	0.33	-1.60
GO:00097	plant-type	Cellular C	0.87	0.92	Solyc02g0	Solyc02g0	0.31	-1.67
GO:00064	translation	Biological	0.88	0.92	Solyc06g0	Solyc06g0	2.11	1.07
GO:00070	microtubul	Biological	0.88	0.92	Solyc03g1	Solyc03g1	0.35	-1.51
GO:00168	ATP hydrc	Molecular	0.88	0.93	Solyc02g0	Solyc02g0	2.65	1.40
GO:00168	ATP hydrc	Molecular	0.88	0.93	Solyc10g0	Solyc10g0	0.47	-1.09
GO:00168	ATP hydrc	Molecular	0.88	0.93	Solyc05g0	Solyc05g0	2.04	1.03
GO:00168	ATP hydrc	Molecular	0.88	0.93	Solyc03g0	Solyc03g0	4.75	2.25
GO:00168	ATP hydrc	Molecular	0.88	0.93	Solyc03g1	Solyc03g1	0.47	-1.08
GO:00168	ATP hydrc	Molecular	0.88	0.93	Solyc12g0	Solyc12g0	2112.44	11.04
GO:00168	ATP hydrc	Molecular	0.88	0.93	Solyc03g1	Solyc03g1	0.00	-11.76
GO:00168	ATP hydrc	Molecular	0.88	0.93	Solyc03g0	Solyc03g0	4.19	2.07
GO:00168	ATP hydrc	Molecular	0.88	0.93	Solyc10g0	Solyc10g0	2.44	1.29
GO:00168	ATP hydrc	Molecular	0.88	0.93	Solyc08g0	Solyc08g0	0.37	-1.42
GO:00168	ATP hydrc	Molecular	0.88	0.93	Solyc06g0	Solyc06g0	3.53	1.82
GO:00168	ATP hydrc	Molecular	0.88	0.93	Solyc10g0	Solyc10g0	0.35	-1.51
GO:00168	ATP hydrc	Molecular	0.88	0.93	Solyc02g0	Solyc02g0	2.46	1.30
GO:00000	ribosomal	Biological	0.89	0.94	Solyc09g0	Solyc09g0	6.55	2.71
GO:00071	signal tran	Biological	0.91	0.95	Solyc01g1	Solyc01g1	0.08	-3.71
GO:00071	signal tran	Biological	0.91	0.95	Solyc06g0	Solyc06g0	2.42	1.28
GO:00071	signal tran	Biological	0.91	0.95	Solyc05g0	Solyc05g0	2.15	1.10
GO:00071	signal tran	Biological	0.91	0.95	Solyc06g0	Solyc06g0	3.73	1.90
GO:00071	signal tran	Biological	0.91	0.95	Solyc06g0	Solyc06g0	2.69	1.43
GO:00071	signal tran	Biological	0.91	0.95	Solyc02g0	Solyc02g0	0.36	-1.48
GO:00071	signal tran	Biological	0.91	0.95	Solyc09g0	Solyc09g0	0.00	-9.29
GO:00071	signal tran	Biological	0.91	0.95	Solyc02g0	Solyc02g0	0.44	-1.18
GO:00071	signal tran	Biological	0.91	0.95	Solyc05g0	Solyc05g0	0.00	-8.91

GO:00058	trans-Golg	Cellular C	0.91	0.95	Solyc02g0	Solyc02g0	4.39	2.13
GO:00058	trans-Golg	Cellular C	0.91	0.95	Solyc09g0	Solyc09g0	2.87	1.52
GO:00058	trans-Golg	Cellular C	0.91	0.95	Solyc07g0	Solyc07g0	0.44	-1.18
GO:19026	proton tran	Biological	0.91	0.96	Solyc12g0	Solyc12g0	2112.44	11.04
GO:19026	proton tran	Biological	0.91	0.96	Solyc11g0	Solyc11g0	0.33	-1.60
GO:00167	glycosyltra	Molecular	0.92	0.96	Solyc09g0	Solyc09g0	0.07	-3.81
GO:00167	glycosyltra	Molecular	0.92	0.96	Solyc12g0	Solyc12g0	0.40	-1.33
GO:00167	glycosyltra	Molecular	0.92	0.96	Solyc02g0	Solyc02g0	0.33	-1.59
GO:00167	glycosyltra	Molecular	0.92	0.96	Solyc01g0	Solyc01g0	0.45	-1.15
GO:00167	glycosyltra	Molecular	0.92	0.96	Solyc05g0	Solyc05g0	0.00	-11.28
GO:00167	glycosyltra	Molecular	0.92	0.96	Solyc09g0	Solyc09g0	0.34	-1.55
GO:00167	glycosyltra	Molecular	0.92	0.96	Solyc05g0	Solyc05g0	2.03	1.02
GO:00167	glycosyltra	Molecular	0.92	0.96	Solyc01g0	Solyc01g0	4.29	2.10
GO:00167	glycosyltra	Molecular	0.92	0.96	Solyc07g0	Solyc07g0	0.30	-1.72
GO:00043	helicase ac	Molecular	0.92	0.96	Solyc01g0	Solyc01g0	2.13	1.09
GO:00043	helicase ac	Molecular	0.92	0.96	Solyc10g0	Solyc10g0	2.30	1.20
GO:00058	cytosol	Cellular C	0.92	0.96	Solyc02g0	Solyc02g0	0.44	-1.18
GO:00058	cytosol	Cellular C	0.92	0.96	Solyc01g1	Solyc01g1	0.41	-1.30
GO:00058	cytosol	Cellular C	0.92	0.96	Solyc07g0	Solyc07g0	0.47	-1.09
GO:00058	cytosol	Cellular C	0.92	0.96	Solyc06g0	Solyc06g0	0.26	-1.93
GO:00058	cytosol	Cellular C	0.92	0.96	Solyc01g1	Solyc01g1	0.47	-1.09
GO:00058	cytosol	Cellular C	0.92	0.96	Solyc05g0	Solyc05g0	2.01	1.01
GO:00058	cytosol	Cellular C	0.92	0.96	Solyc04g0	Solyc04g0	0.18	-2.44
GO:00058	cytosol	Cellular C	0.92	0.96	Solyc12g0	Solyc12g0	0.34	-1.56
GO:00058	cytosol	Cellular C	0.92	0.96	Solyc09g0	Solyc09g0	0.21	-2.26
GO:00058	cytosol	Cellular C	0.92	0.96	Solyc08g0	Solyc08g0	0.50	-1.01
GO:00058	cytosol	Cellular C	0.92	0.96	Solyc11g0	Solyc11g0	0.49	-1.03
GO:00058	cytosol	Cellular C	0.92	0.96	Solyc06g0	Solyc06g0	5.58	2.48
GO:00058	cytosol	Cellular C	0.92	0.96	Solyc11g0	Solyc11g0	0.33	-1.59
GO:00058	cytosol	Cellular C	0.92	0.96	Solyc12g0	Solyc12g0	0.35	-1.50
GO:00058	cytosol	Cellular C	0.92	0.96	Solyc05g0	Solyc05g0	0.24	-2.05
GO:00058	cytosol	Cellular C	0.92	0.96	Solyc06g0	Solyc06g0	0.33	-1.61
GO:00058	cytosol	Cellular C	0.92	0.96	Solyc03g0	Solyc03g0	0.08	-3.70
GO:00058	cytosol	Cellular C	0.92	0.96	Solyc01g1	Solyc01g1	2.42	1.28
GO:00058	cytosol	Cellular C	0.92	0.96	Solyc03g0	Solyc03g0	2.48	1.31
GO:00058	cytosol	Cellular C	0.92	0.96	Solyc09g0	Solyc09g0	2.43	1.28
GO:00058	cytosol	Cellular C	0.92	0.96	Solyc07g0	Solyc07g0	638.37	9.32
GO:00058	cytosol	Cellular C	0.92	0.96	Solyc11g0	Solyc11g0	0.47	-1.08
GO:00483	root develo	Biological	0.92	0.97	Solyc03g0	Solyc03g0	2979.20	11.54
GO:00510	unfolded p	Molecular	0.93	0.97	Solyc04g0	Solyc04g0	0.49	-1.03
GO:00510	unfolded p	Molecular	0.93	0.97	Solyc10g0	Solyc10g0	0.47	-1.09
GO:00510	unfolded p	Molecular	0.93	0.97	Solyc03g1	Solyc03g1	0.00	-11.76
GO:00045	ribonuclea	Molecular	0.93	0.97	Solyc03g0	Solyc03g0	5.64	2.50
GO:00431	proteasom	Biological	0.93	0.97	Solyc05g0	Solyc05g0	0.24	-2.05
GO:00480	quinone bi	Molecular	0.93	0.97	Solyc03g0	Solyc03g0	0.34	-1.57
GO:00039	GTPase ac	Molecular	0.94	0.97	Solyc11g0	Solyc11g0	0.13	-2.91
GO:00039	GTPase ac	Molecular	0.94	0.97	Solyc03g0	Solyc03g0	2.26	1.17
GO:00039	GTPase ac	Molecular	0.94	0.97	Solyc09g0	Solyc09g0	0.33	-1.60
GO:00039	GTPase ac	Molecular	0.94	0.97	Solyc05g0	Solyc05g0	2266.47	11.15
GO:00039	GTPase ac	Molecular	0.94	0.97	Solyc01g0	Solyc01g0	208.08	7.70
GO:00039	GTPase ac	Molecular	0.94	0.97	Solyc05g0	Solyc05g0	0.43	-1.23

GO:00039	GTPase ac	Molecular	0.94	0.97	Solyc08g0	Solyc08g0	9.73	3.28
GO:00057	endosome	Cellular C	0.94	0.97	Solyc02g0	Solyc02g0	4.39	2.13
GO:00057	endosome	Cellular C	0.94	0.97	Solyc09g0	Solyc09g0	2.87	1.52
GO:00316	ubiquitin p	Molecular	0.94	0.98	Solyc07g0	Solyc07g0	0.02	-5.66
GO:00165	protein de	Biological	0.94	0.98	Solyc12g0	Solyc12g0	0.35	-1.50
GO:00048	thiol-deper	Molecular	0.95	0.98	Solyc12g0	Solyc12g0	0.35	-1.50
GO:00068	endoplasm	Biological	0.95	0.98	Solyc04g0	Solyc04g0	0.45	-1.14
GO:00068	endoplasm	Biological	0.95	0.98	Solyc07g0	Solyc07g0	0.20	-2.33
GO:00046	transmemb	Molecular	0.95	0.98	Solyc05g0	Solyc05g0	0.46	-1.11
GO:00046	transmemb	Molecular	0.95	0.98	Solyc10g0	Solyc10g0	0.42	-1.27
GO:00057	mitochond	Cellular C	0.95	0.98	Solyc01g0	Solyc01g0	0.29	-1.78
GO:00975	glutathione	Molecular	0.95	0.98	Solyc08g0	Solyc08g0	2.04	1.03
GO:00167	nucleotidy	Molecular	0.95	0.98	Solyc12g0	Solyc12g0	5.61	2.49
GO:00057	nucleolus	Cellular C	0.95	0.98	Solyc11g0	Solyc11g0	0.43	-1.23
GO:00057	nucleolus	Cellular C	0.95	0.98	Solyc01g0	Solyc01g0	3.68	1.88
GO:00057	endoplasm	Cellular C	0.96	0.99	Solyc02g0	Solyc02g0	2.02	1.01
GO:00057	endoplasm	Cellular C	0.96	0.99	Solyc05g0	Solyc05g0	0.18	-2.51
GO:00057	endoplasm	Cellular C	0.96	0.99	Solyc09g0	Solyc09g0	0.44	-1.17
GO:00057	endoplasm	Cellular C	0.96	0.99	Solyc05g0	Solyc05g0	0.38	-1.40
GO:00057	endoplasm	Cellular C	0.96	0.99	Solyc02g0	Solyc02g0	0.41	-1.30
GO:00057	endoplasm	Cellular C	0.96	0.99	Solyc06g0	Solyc06g0	0.40	-1.33
GO:00057	endoplasm	Cellular C	0.96	0.99	Solyc01g0	Solyc01g0	0.29	-1.77
GO:00057	endoplasm	Cellular C	0.96	0.99	Solyc03g1	Solyc03g1	0.48	-1.05
GO:00057	endoplasm	Cellular C	0.96	0.99	Solyc04g0	Solyc04g0	0.45	-1.14
GO:00163	kinase acti	Molecular	0.96	0.99	Solyc02g0	Solyc02g0	0.47	-1.10
GO:00163	kinase acti	Molecular	0.96	0.99	Solyc07g0	Solyc07g0	0.02	-5.66
GO:00163	kinase acti	Molecular	0.96	0.99	Solyc11g0	Solyc11g0	2.55	1.35
GO:00163	kinase acti	Molecular	0.96	0.99	Solyc03g1	Solyc03g1	2479.17	11.28
GO:00163	kinase acti	Molecular	0.96	0.99	Solyc02g0	Solyc02g0	0.16	-2.63
GO:00163	kinase acti	Molecular	0.96	0.99	Solyc06g0	Solyc06g0	0.48	-1.07
GO:00163	kinase acti	Molecular	0.96	0.99	Solyc06g0	Solyc06g0	0.26	-1.94
GO:00064	protein fol	Biological	0.97	1.00	Solyc08g0	Solyc08g0	0.49	-1.02
GO:00064	protein fol	Biological	0.97	1.00	Solyc04g0	Solyc04g0	0.49	-1.03
GO:00064	protein fol	Biological	0.97	1.00	Solyc10g0	Solyc10g0	0.47	-1.09
GO:00064	protein fol	Biological	0.97	1.00	Solyc05g0	Solyc05g0	0.13	-3.00
GO:00037	translation	Molecular	0.97	1.00	Solyc06g0	Solyc06g0	2.11	1.07
GO:00167	phosphatas	Molecular	0.97	1.00	Solyc06g0	Solyc06g0	0.24	-2.05
GO:00167	phosphatas	Molecular	0.97	1.00	Solyc06g0	Solyc06g0	0.22	-2.19
GO:00167	phosphatas	Molecular	0.97	1.00	Solyc01g0	Solyc01g0	0.45	-1.16
GO:00167	phosphatas	Molecular	0.97	1.00	Solyc09g0	Solyc09g0	0.06	-4.18
GO:00167	phosphatas	Molecular	0.97	1.00	Solyc03g1	Solyc03g1	0.44	-1.19
GO:00150	protein tra	Biological	0.97	1.00	Solyc03g0	Solyc03g0	2.26	1.17
GO:00150	protein tra	Biological	0.97	1.00	Solyc06g0	Solyc06g0	0.14	-2.87
GO:00150	protein tra	Biological	0.97	1.00	Solyc11g0	Solyc11g0	0.24	-2.07
GO:00047	protein ser	Molecular	0.98	1.00	Solyc09g0	Solyc09g0	0.06	-4.18
GO:00081	NADH del	Molecular	0.98	1.00	Solyc03g0	Solyc03g0	0.34	-1.57
GO:00055	GTP bindi	Molecular	0.99	1.00	Solyc11g0	Solyc11g0	0.13	-2.91
GO:00055	GTP bindi	Molecular	0.99	1.00	Solyc03g0	Solyc03g0	2.26	1.17
GO:00055	GTP bindi	Molecular	0.99	1.00	Solyc09g0	Solyc09g0	0.33	-1.60
GO:00055	GTP bindi	Molecular	0.99	1.00	Solyc05g0	Solyc05g0	2266.47	11.15
GO:00055	GTP bindi	Molecular	0.99	1.00	Solyc01g0	Solyc01g0	208.08	7.70

GO:00055	GTP bindi	Molecular	0.99	1.00	Solyc05g0	Solyc05g0	0.43	-1.23
GO:00055	GTP bindi	Molecular	0.99	1.00	Solyc08g0	Solyc08g0	9.73	3.28
GO:00226	cytosolic l	Cellular C	0.99	1.00	Solyc09g0	Solyc09g0	3.66	1.87
GO:00226	cytosolic l	Cellular C	0.99	1.00	Solyc09g0	Solyc09g0	6.55	2.71
GO:00038	DNA-direc	Molecular	0.99	1.00	Solyc12g0	Solyc12g0	5.61	2.49
GO:00001	nucleotide	Molecular	0.99	1.00	Solyc10g0	Solyc10g0	0.47	-1.09
GO:00001	nucleotide	Molecular	0.99	1.00	Solyc02g0	Solyc02g0	0.47	-1.10
GO:00001	nucleotide	Molecular	0.99	1.00	Solyc06g0	Solyc06g0	0.49	-1.02
GO:00001	nucleotide	Molecular	0.99	1.00	Solyc02g0	Solyc02g0	0.34	-1.57
GO:00001	nucleotide	Molecular	0.99	1.00	Solyc07g0	Solyc07g0	0.02	-5.66
GO:00001	nucleotide	Molecular	0.99	1.00	Solyc11g0	Solyc11g0	2.55	1.35
GO:00001	nucleotide	Molecular	0.99	1.00	Solyc12g0	Solyc12g0	2112.44	11.04
GO:00001	nucleotide	Molecular	0.99	1.00	Solyc12g0	Solyc12g0	9.14	3.19
GO:00001	nucleotide	Molecular	0.99	1.00	Solyc02g0	Solyc02g0	0.16	-2.63
GO:00001	nucleotide	Molecular	0.99	1.00	Solyc08g0	Solyc08g0	0.37	-1.42
GO:00001	nucleotide	Molecular	0.99	1.00	Solyc11g0	Solyc11g0	0.33	-1.60
GO:00001	nucleotide	Molecular	0.99	1.00	Solyc06g0	Solyc06g0	0.48	-1.07
GO:00458	negative re	Biological	0.99	1.00	Solyc03g0	Solyc03g0	0.23	-2.14
GO:00616	ubiquitin p	Molecular	0.99	1.00	Solyc01g1	Solyc01g1	0.42	-1.24
GO:00616	ubiquitin p	Molecular	0.99	1.00	Solyc05g0	Solyc05g0	2.31	1.21
GO:00616	ubiquitin p	Molecular	0.99	1.00	Solyc11g0	Solyc11g0	0.00	-9.98
GO:00485	meristem c	Biological	0.99	1.00	Solyc07g0	Solyc07g0	0.00	-9.40
GO:00068	intracellul	Biological	0.99	1.00	Solyc06g0	Solyc06g0	2.11	1.07
GO:00068	intracellul	Biological	0.99	1.00	Solyc09g0	Solyc09g0	0.33	-1.60
GO:00068	intracellul	Biological	0.99	1.00	Solyc05g0	Solyc05g0	0.43	-1.23
GO:00068	intracellul	Biological	0.99	1.00	Solyc04g0	Solyc04g0	0.45	-1.14
GO:00068	intracellul	Biological	0.99	1.00	Solyc01g0	Solyc01g0	0.19	-2.40
GO:00161	vesicle-me	Biological	0.99	1.00	Solyc06g0	Solyc06g0	2.11	1.07
GO:00161	vesicle-me	Biological	0.99	1.00	Solyc01g0	Solyc01g0	0.19	-2.40
GO:00100	meristem r	Biological	0.99	1.00	Solyc07g0	Solyc07g0	0.00	-9.40
GO:00064	protein gly	Biological	0.99	1.00	Solyc07g0	Solyc07g0	0.30	-1.72
GO:00055	protein bin	Molecular	1.00	1.00	Solyc08g0	Solyc08g0	0.49	-1.02
GO:00055	protein bin	Molecular	1.00	1.00	Solyc01g0	Solyc01g0	4.36	2.12
GO:00055	protein bin	Molecular	1.00	1.00	Solyc01g1	Solyc01g1	0.08	-3.71
GO:00055	protein bin	Molecular	1.00	1.00	Solyc01g0	Solyc01g0	2.27	1.18
GO:00055	protein bin	Molecular	1.00	1.00	Solyc02g0	Solyc02g0	2.02	1.01
GO:00055	protein bin	Molecular	1.00	1.00	Solyc02g0	Solyc02g0	4.39	2.13
GO:00055	protein bin	Molecular	1.00	1.00	Solyc01g0	Solyc01g0	0.31	-1.69
GO:00055	protein bin	Molecular	1.00	1.00	Solyc06g0	Solyc06g0	0.08	-3.62
GO:00055	protein bin	Molecular	1.00	1.00	Solyc05g0	Solyc05g0	0.03	-5.26
GO:00055	protein bin	Molecular	1.00	1.00	Solyc10g0	Solyc10g0	2.09	1.06
GO:00055	protein bin	Molecular	1.00	1.00	Solyc01g1	Solyc01g1	0.31	-1.68
GO:00055	protein bin	Molecular	1.00	1.00	Solyc11g0	Solyc11g0	3.00	1.58
GO:00055	protein bin	Molecular	1.00	1.00	Solyc09g0	Solyc09g0	0.03	-4.88
GO:00055	protein bin	Molecular	1.00	1.00	Solyc05g0	Solyc05g0	2.04	1.03
GO:00055	protein bin	Molecular	1.00	1.00	Solyc04g0	Solyc04g0	0.48	-1.06
GO:00055	protein bin	Molecular	1.00	1.00	Solyc03g0	Solyc03g0	0.46	-1.13
GO:00055	protein bin	Molecular	1.00	1.00	Solyc02g0	Solyc02g0	0.42	-1.24
GO:00055	protein bin	Molecular	1.00	1.00	Solyc10g0	Solyc10g0	0.45	-1.16
GO:00055	protein bin	Molecular	1.00	1.00	Solyc09g0	Solyc09g0	0.49	-1.03
GO:00055	protein bin	Molecular	1.00	1.00	Solyc03g1	Solyc03g1	0.39	-1.35

GO:00055	protein bin	Molecular	1.00	1.00	Solyc09g0	Solyc09g0	0.45	-1.17
GO:00055	protein bin	Molecular	1.00	1.00	Solyc07g0	EIX1	0.33	-1.59
GO:00055	protein bin	Molecular	1.00	1.00	Solyc01g1	Solyc01g1	0.43	-1.23
GO:00055	protein bin	Molecular	1.00	1.00	Solyc09g0	Solyc09g0	2.63	1.39
GO:00055	protein bin	Molecular	1.00	1.00	Solyc09g0	Solyc09g0	2.10	1.07
GO:00055	protein bin	Molecular	1.00	1.00	Solyc11g0	Solyc11g0	0.33	-1.60
GO:00055	protein bin	Molecular	1.00	1.00	Solyc04g0	Solyc04g0	0.45	-1.15
GO:00055	protein bin	Molecular	1.00	1.00	Solyc05g0	Solyc05g0	0.46	-1.11
GO:00055	protein bin	Molecular	1.00	1.00	Solyc06g0	Solyc06g0	0.34	-1.55
GO:00055	protein bin	Molecular	1.00	1.00	Solyc11g0	Solyc11g0	0.48	-1.06
GO:00055	protein bin	Molecular	1.00	1.00	Solyc02g0	Solyc02g0	30.37	4.92
GO:00055	protein bin	Molecular	1.00	1.00	Solyc05g0	Solyc05g0	34721.47	15.08
GO:00055	protein bin	Molecular	1.00	1.00	Solyc01g0	Solyc01g0	0.24	-2.03
GO:00055	protein bin	Molecular	1.00	1.00	Solyc03g0	Solyc03g0	3.70	1.89
GO:00055	protein bin	Molecular	1.00	1.00	Solyc04g0	Solyc04g0	0.48	-1.06
GO:00055	protein bin	Molecular	1.00	1.00	Solyc06g0	Solyc06g0	3.34	1.74
GO:00055	protein bin	Molecular	1.00	1.00	Solyc09g0	Solyc09g0	91.78	6.52
GO:00055	protein bin	Molecular	1.00	1.00	Solyc02g0	Solyc02g0	0.41	-1.28
GO:00055	protein bin	Molecular	1.00	1.00	Solyc03g0	Solyc03g0	2.33	1.22
GO:00055	protein bin	Molecular	1.00	1.00	Solyc08g0	Solyc08g0	0.39	-1.34
GO:00055	protein bin	Molecular	1.00	1.00	Solyc12g0	Solyc12g0	0.42	-1.25
GO:00055	protein bin	Molecular	1.00	1.00	Solyc06g0	Solyc06g0	0.49	-1.02
GO:00055	protein bin	Molecular	1.00	1.00	Solyc03g0	Solyc03g0	0.29	-1.76
GO:00055	protein bin	Molecular	1.00	1.00	Solyc11g0	Solyc11g0	2.17	1.12
GO:00055	protein bin	Molecular	1.00	1.00	Solyc06g0	Solyc06g0	0.40	-1.31
GO:00055	protein bin	Molecular	1.00	1.00	Solyc06g0	Solyc06g0	0.07	-3.94
GO:00055	protein bin	Molecular	1.00	1.00	Solyc09g0	Solyc09g0	0.47	-1.08
GO:00055	protein bin	Molecular	1.00	1.00	Solyc09g0	Solyc09g0	0.39	-1.35
GO:00055	protein bin	Molecular	1.00	1.00	Solyc05g0	Solyc05g0	0.38	-1.41
GO:00055	protein bin	Molecular	1.00	1.00	Solyc11g0	Solyc11g0	0.46	-1.11
GO:00055	protein bin	Molecular	1.00	1.00	Solyc05g0	Solyc05g0	0.32	-1.65
GO:00055	protein bin	Molecular	1.00	1.00	Solyc07g0	Solyc07g0	0.25	-1.97
GO:00055	protein bin	Molecular	1.00	1.00	Solyc04g0	Solyc04g0	0.42	-1.26
GO:00055	protein bin	Molecular	1.00	1.00	Solyc11g0	Solyc11g0	5992.64	12.55
GO:00055	protein bin	Molecular	1.00	1.00	Solyc09g0	Solyc09g0	0.44	-1.19
GO:00055	protein bin	Molecular	1.00	1.00	Solyc10g0	Solyc10g0	0.31	-1.69
GO:00055	protein bin	Molecular	1.00	1.00	Solyc11g0	Solyc11g0	0.47	-1.10
GO:00055	protein bin	Molecular	1.00	1.00	Solyc01g1	Solyc01g1	0.44	-1.20
GO:00055	protein bin	Molecular	1.00	1.00	Solyc12g0	Solyc12g0	0.33	-1.58
GO:00055	protein bin	Molecular	1.00	1.00	Solyc05g0	Solyc05g0	0.24	-2.05
GO:00055	protein bin	Molecular	1.00	1.00	Solyc06g0	Solyc06g0	0.33	-1.61
GO:00055	protein bin	Molecular	1.00	1.00	Solyc03g0	Solyc03g0	0.00	-11.91
GO:00055	protein bin	Molecular	1.00	1.00	Solyc02g0	Solyc02g0	4951.62	12.27
GO:00055	protein bin	Molecular	1.00	1.00	Solyc04g0	Solyc04g0	0.27	-1.91
GO:00055	protein bin	Molecular	1.00	1.00	Solyc02g0	Solyc02g0	0.23	-2.10
GO:00055	protein bin	Molecular	1.00	1.00	Solyc01g0	Solyc01g0	0.00	-11.38
GO:00055	protein bin	Molecular	1.00	1.00	Solyc09g0	Solyc09g0	0.34	-1.56
GO:00055	protein bin	Molecular	1.00	1.00	Solyc01g0	Solyc01g0	0.00	-10.39
GO:00055	protein bin	Molecular	1.00	1.00	Solyc08g0	Solyc08g0	2.14	1.10
GO:00055	protein bin	Molecular	1.00	1.00	Solyc02g0	Solyc02g0	0.28	-1.85
GO:00055	protein bin	Molecular	1.00	1.00	Solyc04g0	Solyc04g0	0.35	-1.51

GO:00055	protein bin	Molecular	1.00	1.00	Solyc02g0	Solyc02g0	2.20	1.14
GO:00055	protein bin	Molecular	1.00	1.00	Solyc08g0	Solyc08g0	0.12	-3.09
GO:00055	protein bin	Molecular	1.00	1.00	Solyc08g0	Solyc08g0	0.26	-1.95
GO:00055	protein bin	Molecular	1.00	1.00	Solyc03g1	Solyc03g1	0.09	-3.43
GO:00055	protein bin	Molecular	1.00	1.00	Solyc12g0	Solyc12g0	9.14	3.19
GO:00055	protein bin	Molecular	1.00	1.00	Solyc12g0	Solyc12g0	0.09	-3.42
GO:00055	protein bin	Molecular	1.00	1.00	Solyc08g0	Solyc08g0	0.38	-1.40
GO:00055	protein bin	Molecular	1.00	1.00	Solyc06g0	Solyc06g0	0.31	-1.70
GO:00055	protein bin	Molecular	1.00	1.00	Solyc09g0	Solyc09g0	3.90	1.96
GO:00055	protein bin	Molecular	1.00	1.00	Solyc01g1	Solyc01g1	0.20	-2.34
GO:00055	protein bin	Molecular	1.00	1.00	Solyc02g0	Solyc02g0	7.32	2.87
GO:00055	protein bin	Molecular	1.00	1.00	Solyc02g0	Solyc02g0	0.45	-1.16
GO:00055	protein bin	Molecular	1.00	1.00	Solyc12g0	Solyc12g0	0.41	-1.30
GO:00055	protein bin	Molecular	1.00	1.00	Solyc02g0	Solyc02g0	0.45	-1.15
GO:00055	protein bin	Molecular	1.00	1.00	Solyc07g0	Solyc07g0	558.88	9.13
GO:00055	protein bin	Molecular	1.00	1.00	Solyc08g0	Solyc08g0	0.18	-2.44
GO:00055	protein bin	Molecular	1.00	1.00	Solyc09g0	Solyc09g0	0.48	-1.06
GO:00055	protein bin	Molecular	1.00	1.00	Solyc02g0	Solyc02g0	0.50	-1.01
GO:00055	protein bin	Molecular	1.00	1.00	Solyc05g0	Solyc05g0	2.16	1.11
GO:00055	protein bin	Molecular	1.00	1.00	Solyc03g0	Solyc03g0	0.00	-8.79
GO:00055	protein bin	Molecular	1.00	1.00	Solyc12g0	Solyc12g0	0.48	-1.07
GO:00055	protein bin	Molecular	1.00	1.00	Solyc03g1	Solyc03g1	0.08	-3.61
GO:00055	protein bin	Molecular	1.00	1.00	Solyc03g0	Solyc03g0	0.01	-7.23
GO:00055	protein bin	Molecular	1.00	1.00	Solyc12g0	Solyc12g0	0.18	-2.45
GO:00055	protein bin	Molecular	1.00	1.00	Solyc08g0	Solyc08g0	745.09	9.54
GO:00055	protein bin	Molecular	1.00	1.00	Solyc09g0	Solyc09g0	0.33	-1.59
GO:00055	protein bin	Molecular	1.00	1.00	Solyc10g0	Solyc10g0	0.50	-1.01
GO:00055	protein bin	Molecular	1.00	1.00	Solyc03g0	Solyc03g0	4.40	2.14
GO:00055	protein bin	Molecular	1.00	1.00	Solyc08g0	Solyc08g0	0.07	-3.76
GO:00055	protein bin	Molecular	1.00	1.00	Solyc03g0	Solyc03g0	0.00	-8.17
GO:00055	protein bin	Molecular	1.00	1.00	Solyc07g0	Solyc07g0	0.00	-8.72
GO:00055	protein bin	Molecular	1.00	1.00	Solyc08g0	Solyc08g0	11.48	3.52
GO:00055	protein bin	Molecular	1.00	1.00	Solyc06g0	Solyc06g0	11.24	3.49
GO:00055	protein bin	Molecular	1.00	1.00	Solyc08g0	Solyc08g0	0.00	-9.52
GO:00055	protein bin	Molecular	1.00	1.00	Solyc01g0	Solyc01g0	2748.30	11.42
GO:00055	protein bin	Molecular	1.00	1.00	Solyc09g0	Solyc09g0	0.10	-3.32
GO:00055	protein bin	Molecular	1.00	1.00	Solyc03g1	Solyc03g1	0.24	-2.05
GO:00055	protein bin	Molecular	1.00	1.00	Solyc01g0	Solyc01g0	0.23	-2.09
GO:00055	protein bin	Molecular	1.00	1.00	Solyc06g0	Solyc06g0	0.41	-1.27
GO:00055	protein bin	Molecular	1.00	1.00	Solyc06g0	Solyc06g0	0.00	-8.89
GO:00055	protein bin	Molecular	1.00	1.00	Solyc06g0	Solyc06g0	0.37	-1.42
GO:00055	protein bin	Molecular	1.00	1.00	Solyc09g0	Solyc09g0	0.38	-1.39
GO:00055	protein bin	Molecular	1.00	1.00	Solyc08g0	Solyc08g0	0.39	-1.34
GO:00055	protein bin	Molecular	1.00	1.00	Solyc01g0	Solyc01g0	3.58	1.84
GO:00055	protein bin	Molecular	1.00	1.00	Solyc09g0	Solyc09g0	0.22	-2.21
GO:00055	protein bin	Molecular	1.00	1.00	Solyc09g0	Solyc09g0	0.00	-11.54
GO:00055	protein bin	Molecular	1.00	1.00	Solyc07g0	Solyc07g0	314.95	8.30
GO:00055	protein bin	Molecular	1.00	1.00	Solyc08g0	Solyc08g0	2.33	1.22
GO:00055	protein bin	Molecular	1.00	1.00	Solyc04g0	Solyc04g0	0.43	-1.22
GO:00037	mRNA bin	Molecular	1.00	1.00	Solyc12g0	Solyc12g0	2.53	1.34
GO:00037	mRNA bin	Molecular	1.00	1.00	Solyc06g0	Solyc06g0	0.45	-1.14

GO:00057	Golgi appa	Cellular C	1.00	1.00	Solyc04g0	Solyc04g0	0.48	-1.06
GO:00057	Golgi appa	Cellular C	1.00	1.00	Solyc05g0	Solyc05g0	0.38	-1.40
GO:00057	Golgi appa	Cellular C	1.00	1.00	Solyc02g0	Solyc02g0	0.41	-1.30
GO:00057	Golgi appa	Cellular C	1.00	1.00	Solyc01g0	Solyc01g0	2.66	1.41
GO:00057	Golgi appa	Cellular C	1.00	1.00	Solyc07g0	Solyc07g0	0.44	-1.18
GO:00082	zinc ion bi	Molecular	1.00	1.00	Solyc02g0	Solyc02g0	2.65	1.40
GO:00082	zinc ion bi	Molecular	1.00	1.00	Solyc03g0	Solyc03g0	3.36	1.75
GO:00082	zinc ion bi	Molecular	1.00	1.00	Solyc04g0	Solyc04g0	0.48	-1.06
GO:00082	zinc ion bi	Molecular	1.00	1.00	Solyc12g0	Solyc12g0	0.44	-1.20
GO:00082	zinc ion bi	Molecular	1.00	1.00	Solyc01g0	Solyc01g0	0.34	-1.55
GO:00082	zinc ion bi	Molecular	1.00	1.00	Solyc04g0	Solyc04g0	0.42	-1.26
GO:00082	zinc ion bi	Molecular	1.00	1.00	Solyc02g0	Solyc02g0	0.10	-3.33
GO:00082	zinc ion bi	Molecular	1.00	1.00	Solyc03g0	Solyc03g0	2.30	1.20
GO:00082	zinc ion bi	Molecular	1.00	1.00	Solyc03g0	Solyc03g0	0.00	-11.91
GO:00082	zinc ion bi	Molecular	1.00	1.00	Solyc06g0	Solyc06g0	0.15	-2.69
GO:00082	zinc ion bi	Molecular	1.00	1.00	Solyc08g0	Solyc08g0	0.44	-1.20
GO:00082	zinc ion bi	Molecular	1.00	1.00	Solyc06g0	Solyc06g0	0.39	-1.35
GO:00082	zinc ion bi	Molecular	1.00	1.00	Solyc02g0	Solyc02g0	3.01	1.59
GO:00082	zinc ion bi	Molecular	1.00	1.00	Solyc03g1	Solyc03g1	0.44	-1.19
GO:00082	zinc ion bi	Molecular	1.00	1.00	Solyc04g0	Solyc04g0	2.19	1.13
GO:00082	zinc ion bi	Molecular	1.00	1.00	Solyc12g0	Solyc12g0	0.40	-1.34
GO:00082	zinc ion bi	Molecular	1.00	1.00	Solyc01g0	Solyc01g0	2.01	1.01
GO:00082	zinc ion bi	Molecular	1.00	1.00	Solyc01g0	Solyc01g0	0.32	-1.64
GO:00082	zinc ion bi	Molecular	1.00	1.00	Solyc02g0	Solyc02g0	0.34	-1.54
GO:00082	zinc ion bi	Molecular	1.00	1.00	Solyc11g0	Solyc11g0	0.00	-9.98
GO:00082	zinc ion bi	Molecular	1.00	1.00	Solyc03g1	Solyc03g1	0.24	-2.05
GO:00063	RNA proc	Biological	1.00	1.00	Solyc03g0	Solyc03g0	5.64	2.50
GO:00057	mitochond	Cellular C	1.00	1.00	Solyc01g0	Solyc01g0	0.29	-1.78
GO:00057	mitochond	Cellular C	1.00	1.00	Solyc02g0	Solyc02g0	0.36	-1.46
GO:00057	mitochond	Cellular C	1.00	1.00	Solyc04g0	Solyc04g0	0.41	-1.27
GO:00057	mitochond	Cellular C	1.00	1.00	Solyc08g0	Solyc08g0	0.39	-1.34
GO:00057	mitochond	Cellular C	1.00	1.00	Solyc08g0	Solyc08g0	0.48	-1.06
GO:00057	mitochond	Cellular C	1.00	1.00	Solyc01g0	Solyc01g0	0.42	-1.26
GO:00057	mitochond	Cellular C	1.00	1.00	Solyc11g0	Solyc11g0	0.33	-1.60
GO:00057	mitochond	Cellular C	1.00	1.00	Solyc03g1	Solyc03g1	0.34	-1.57
GO:00057	mitochond	Cellular C	1.00	1.00	Solyc09g0	Solyc09g0	6.02	2.59
GO:00057	mitochond	Cellular C	1.00	1.00	Solyc09g0	Solyc09g0	0.11	-3.24
GO:00057	mitochond	Cellular C	1.00	1.00	Solyc10g0	Solyc10g0	476.24	8.90
GO:00065	ubiquitin-c	Biological	1.00	1.00	Solyc06g0	Solyc06g0	0.49	-1.02
GO:00065	ubiquitin-c	Biological	1.00	1.00	Solyc06g0	Solyc06g0	6.40	2.68
GO:00065	ubiquitin-c	Biological	1.00	1.00	Solyc03g0	Solyc03g0	4.40	2.14
GO:00094	RNA modi	Biological	1.00	1.00	Solyc04g0	Solyc04g0	0.42	-1.26
GO:00058	ribosome	Cellular C	1.00	1.00	Solyc09g0	Solyc09g0	3.66	1.87
GO:00058	ribosome	Cellular C	1.00	1.00	Solyc11g0	Solyc11g0	5.73	2.52
GO:00058	ribosome	Cellular C	1.00	1.00	Solyc06g0	Solyc06g0	0.34	-1.55
GO:00058	ribosome	Cellular C	1.00	1.00	Solyc02g0	Solyc02g0	0.35	-1.49
GO:00058	ribosome	Cellular C	1.00	1.00	Solyc05g0	Solyc05g0	0.42	-1.25
GO:00058	ribosome	Cellular C	1.00	1.00	Solyc09g0	Solyc09g0	0.11	-3.24
GO:00037	structural c	Molecular	1.00	1.00	Solyc09g0	Solyc09g0	3.66	1.87
GO:00037	structural c	Molecular	1.00	1.00	Solyc09g0	Solyc09g0	6.55	2.71
GO:00037	structural c	Molecular	1.00	1.00	Solyc11g0	Solyc11g0	5.73	2.52

GO:00037	structural c	Molecular	1.00	1.00	Solyc06g0	Solyc06g0	0.34	-1.55
GO:00037	structural c	Molecular	1.00	1.00	Solyc02g0	Solyc02g0	0.35	-1.49
GO:00037	structural c	Molecular	1.00	1.00	Solyc05g0	Solyc05g0	0.42	-1.25
GO:00037	structural c	Molecular	1.00	1.00	Solyc09g0	Solyc09g0	0.11	-3.24
GO:00064	translation	Biological	1.00	1.00	Solyc11g0	Solyc11g0	0.13	-2.91
GO:00064	translation	Biological	1.00	1.00	Solyc09g0	Solyc09g0	3.66	1.87
GO:00064	translation	Biological	1.00	1.00	Solyc11g0	Solyc11g0	5.73	2.52
GO:00064	translation	Biological	1.00	1.00	Solyc06g0	Solyc06g0	0.34	-1.55
GO:00064	translation	Biological	1.00	1.00	Solyc02g0	Solyc02g0	0.35	-1.49
GO:00064	translation	Biological	1.00	1.00	Solyc05g0	Solyc05g0	0.42	-1.25
GO:00064	translation	Biological	1.00	1.00	Solyc09g0	Solyc09g0	0.11	-3.24
GO:00036	nucleic aci	Molecular	1.00	1.00	Solyc02g0	Solyc02g0	4.29	2.10
GO:00036	nucleic aci	Molecular	1.00	1.00	Solyc05g0	Solyc05g0	2.27	1.18
GO:00036	nucleic aci	Molecular	1.00	1.00	Solyc09g0	Solyc09g0	5.78	2.53
GO:00036	nucleic aci	Molecular	1.00	1.00	Solyc01g0	Solyc01g0	0.25	-1.99
GO:00036	nucleic aci	Molecular	1.00	1.00	Solyc11g0	Solyc11g0	2.38	1.25
GO:00036	nucleic aci	Molecular	1.00	1.00	Solyc06g0	Solyc06g0	0.30	-1.75
GO:00036	nucleic aci	Molecular	1.00	1.00	Solyc09g0	Solyc09g0	0.32	-1.62
GO:00036	nucleic aci	Molecular	1.00	1.00	Solyc03g1	Solyc03g1	2.29	1.19
GO:00036	nucleic aci	Molecular	1.00	1.00	Solyc03g0	Solyc03g0	2.30	1.20
GO:00036	nucleic aci	Molecular	1.00	1.00	Solyc10g0	Solyc10g0	2.30	1.20
GO:00036	nucleic aci	Molecular	1.00	1.00	Solyc01g0	Solyc01g0	0.00	-10.39
GO:00036	nucleic aci	Molecular	1.00	1.00	Solyc04g0	Solyc04g0	2.19	1.13
GO:00036	nucleic aci	Molecular	1.00	1.00	Solyc12g0	Solyc12g0	0.40	-1.34
GO:00036	nucleic aci	Molecular	1.00	1.00	Solyc01g0	Solyc01g0	2.01	1.01
GO:00036	nucleic aci	Molecular	1.00	1.00	Solyc05g0	Solyc05g0	0.14	-2.83
GO:00036	nucleic aci	Molecular	1.00	1.00	Solyc06g0	Solyc06g0	1647.59	10.69
GO:00037	RNA bindi	Molecular	1.00	1.00	Solyc06g0	Solyc06g0	2.11	1.07
GO:00037	RNA bindi	Molecular	1.00	1.00	Solyc11g0	Solyc11g0	2.38	1.25
GO:00037	RNA bindi	Molecular	1.00	1.00	Solyc09g0	Solyc09g0	0.32	-1.62
GO:00037	RNA bindi	Molecular	1.00	1.00	Solyc09g0	Solyc09g0	2.35	1.24
GO:00037	RNA bindi	Molecular	1.00	1.00	Solyc04g0	Solyc04g0	0.42	-1.26
GO:00037	RNA bindi	Molecular	1.00	1.00	Solyc07g0	Solyc07g0	6759.25	12.72
GO:00037	RNA bindi	Molecular	1.00	1.00	Solyc01g0	Solyc01g0	0.00	-10.39
GO:00037	RNA bindi	Molecular	1.00	1.00	Solyc05g0	Solyc05g0	0.48	-1.07
GO:00037	RNA bindi	Molecular	1.00	1.00	Solyc09g0	Solyc09g0	0.11	-3.24
GO:00037	RNA bindi	Molecular	1.00	1.00	Solyc05g0	Solyc05g0	0.00	-11.22

pval	qval	regulation
0	0	down
0	0	up
0.00	0.00	down
0.00	0.00	down
0.00	0.00	up
0.00	0.00	down
0.00	0.00	down
0.00	0.00	down
0.00	0.00	down
0.00	0.00	up
0.00	0.00	down
0.00	0.00	down
0.00	0.00	down
0.00	0.00	up
0.00	0.00	down
0.00	0.00	down
0.00	0.00	down
0.00	0.00	down
0.00	0.00	down
0.00	0.00	down
0.00	0.00	up
0.00	0.00	down
0.00	0.00	down
0.00	0.00	down
0.00	0.00	down
0.00	0.00	up
0.00	0.00	down
0.00	0.00	down
0.00	0.00	down
0.00	0.00	up
0.00	0.00	up
0.00	0.00	down
0.00	0.00	down
0.00	0.00	up
0.00	0.00	down
0.00	0.00	down
0.00	0.00	down
0.00	0.00	down
0.00	0.01	up
0.00	0.01	up
0.00	0.01	down
0.00	0.01	down
0.00	0.01	down
0.01	0.03	down
0.01	0.03	down
0.01	0.03	up
0.01	0.04	down
0.01	0.04	down
0.01	0.05	down

0	0	up
0	0	down
0.00	0.00	down
0.00	0.00	up
0.00	0.00	down
0.00	0.00	up
0.00	0.00	up
0.00	0.00	down
0.00	0.00	down
0.00	0.00	down
0.00	0.00	down
0.00	0.00	up
0.00	0.00	up
0.00	0.00	down
0.00	0.00	up
0.00	0.00	down
0.00	0.00	down
0.00	0.00	down
0.00	0.00	up
0.00	0.00	down
0.00	0.00	down
0.00	0.00	down
0.00	0.00	up
0.00	0.00	down
0.00	0.00	up
0.00	0.00	up
0.00	0.00	up
0.00	0.00	down
0.00	0.00	up
0.00	0.00	up
0.00	0.01	up
0.00	0.01	up
0.00	0.02	down
0.00	0.02	down
0.02	0.05	down
0	0	down
0.00	0.00	up
0.00	0.00	up
0.00	0.00	down
0.00	0.00	down
0.00	0.00	up
0.00	0.00	up
0.00	0.00	down
0.00	0.00	down
0.00	0.00	up
0.00	0.00	down
0.00	0.00	up
0.00	0.00	up
0.00	0.00	up
0.00	0.00	down
0.00	0.00	up

0.00	0.00	down
0.00	0.00	down
0.02	0.05	down
0.00	0.00	down
0.00	0.00	down
0.00	0.00	down
0.00	0.00	down
0.00	0.00	down
0.00	0.00	down
0.00	0.00	down
0.00	0.00	down
0.00	0.00	down
0.00	0.00	down
0.00	0.00	down
0.00	0.01	down
0.00	0.01	down
0.00	0.01	down
0	0	down
0.00	0.00	down
0.00	0.00	down
0.00	0.00	up
0.00	0.00	down
0.00	0.00	down
0.00	0.00	down
0.00	0.00	down
0.00	0.00	down
0.00	0.00	down
0.00	0.00	up
0.00	0.00	down
0.00	0.00	down
0.00	0.00	down
0.00	0.00	down
0.00	0.00	down
0.00	0.00	down
0.00	0.00	down
0.00	0.00	down
0.00	0.00	up
0.00	0.00	down
0.00	0.00	up
0.00	0.00	down
0.00	0.00	down
0.00	0.00	down
0.00	0.00	down
0.00	0.00	down
0.00	0.00	up
0.00	0.00	down
0.00	0.00	down
0.00	0.00	down
0.00	0.00	down
0.00	0.00	down
0.00	0.00	down

0.00	0.01	up
0.00	0.01	up
0.00	0.01	down
0.00	0.01	down
0.00	0.01	down
0.01	0.03	up
0.01	0.04	down
0.01	0.04	down
0.01	0.05	down
0	0	down
0.00	0.00	down
0.00	0.00	down
0.00	0.00	up
0.00	0.00	down
0.00	0.00	down
0.00	0.00	down
0.00	0.00	down
0.00	0.00	up
0.00	0.00	down
0.00	0.00	down
0.00	0.00	down
0.00	0.00	down
0.00	0.00	down
0.00	0.00	down
0.00	0.00	down
0.00	0.00	down
0.00	0.00	up
0.00	0.00	down
0.00	0.00	down
0.00	0.00	down
0.00	0.00	up
0.00	0.00	down
0.00	0.00	down
0.00	0.00	down
0.00	0.00	up
0.00	0.00	down
0.00	0.00	down
0.00	0.00	down
0.00	0.00	down
0.00	0.00	down
0.00	0.00	down
0.00	0.01	up
0.00	0.01	up
0.00	0.01	down
0.00	0.01	down
0.00	0.01	down
0.01	0.02	down
0.01	0.03	up
0.01	0.04	down
0.01	0.04	down
0.01	0.05	down

0	0	down
0	0	down
0.00	0.00	down
0.00	0.00	down
0.00	0.00	up
0.00	0.00	down
0.00	0.00	down
0.00	0.00	down
0.00	0.00	down
0.00	0.00	down
0.00	0.00	up
0.00	0.00	down
0.00	0.00	down
0.00	0.00	down
0.00	0.00	down
0.00	0.00	down
0.00	0.00	down
0.00	0.00	down
0.00	0.00	down
0.00	0.00	down
0.00	0.00	down
0.00	0.00	up
0.00	0.00	down
0.00	0.00	up
0.00	0.00	down
0.00	0.00	down
0.00	0.00	down
0.00	0.00	down
0.00	0.00	down
0.00	0.00	down
0.00	0.00	up
0.00	0.00	up
0.00	0.00	down
0.00	0.00	down
0.00	0.00	down
0.00	0.00	up
0.00	0.00	down
0.00	0.00	down
0.00	0.00	down
0.00	0.00	down
0.00	0.00	up
0.00	0.00	up
0.00	0.01	up
0.00	0.01	up
0.00	0.01	up
0.00	0.01	down
0.00	0.01	down
0.00	0.01	down
0.00	0.02	down
0.01	0.03	up
0.01	0.04	down

0.01	0.04	down
0.01	0.04	down
0.01	0.04	down
0.01	0.05	down
0	0	down
0.00	0.00	up
0.00	0.00	up
0.00	0.00	down
0.00	0.00	down
0.00	0.00	up
0.00	0.00	up
0.00	0.00	down
0.00	0.00	up
0.00	0.00	down
0.00	0.00	up
0.00	0.00	up
0.00	0.00	up
0.00	0.00	down
0.00	0.00	up
0.00	0.00	down
0.02	0.05	down
0	0	up
0	0	up
0	0	down
0.00	0.00	down
0.00	0.00	up
0.00	0.00	up
0.00	0.00	down
0.00	0.00	down
0.00	0.00	down
0.00	0.00	down
0.00	0.00	up
0.00	0.00	down
0.00	0.00	up
0.00	0.00	up
0.00	0.00	up
0.00	0.00	down
0.00	0.00	down
0.00	0.00	up
0.00	0.00	down
0.00	0.00	down
0.00	0.00	down
0.00	0.00	up
0.00	0.00	down
0.00	0.00	up
0.00	0.00	down
0.00	0.00	up
0.00	0.00	down
0.00	0.00	down
0.00	0.00	down
0.00	0.00	up

0.00	0.00	down
0.00	0.00	down
0.00	0.00	up
0.00	0.00	down
0.00	0.00	down
0.00	0.00	down
0.00	0.00	down
0.00	0.00	down
0.00	0.00	down
0.00	0.00	down
0.00	0.00	down
0.00	0.00	down
0.00	0.00	down
0.00	0.00	down
0.00	0.01	down
0.00	0.01	down
0.00	0.02	down
0.01	0.04	down
0.02	0.05	up
0.02	0.05	down
0.00	0.00	down
0.00	0.00	down
0.00	0.00	down
0.00	0.00	down
0.00	0.00	down
0.00	0.00	down
0.00	0.00	up
0.00	0.00	down
0.00	0.00	down
0.00	0.01	down
0.00	0.01	down
0.01	0.03	down
0.01	0.03	down
0	0	down
0	0	down
0.00	0.00	down
0.00	0.00	down
0.00	0.00	down
0.00	0.00	down
0.00	0.00	down
0.00	0.00	down
0.00	0.00	down
0.00	0.00	down
0.00	0.01	down
0.01	0.03	down
0	0	down
0.00	0.00	up
0.00	0.00	up
0.00	0.00	down
0.00	0.00	up
0.00	0.00	down

0.00	0.00	up
0.00	0.00	down
0.00	0.00	down
0.00	0.00	up
0.00	0.00	down
0.00	0.00	up
0.00	0.00	down
0.00	0.00	up
0.00	0.00	up
0.00	0.00	down
0.00	0.00	up
0.00	0.00	down
0.00	0.00	up
0.00	0.00	up
0.00	0.00	up
0.00	0.00	up
0.00	0.00	down
0.00	0.00	down
0.00	0.00	down
0.00	0.00	down
0.01	0.02	down
0.01	0.02	up
0.01	0.02	up
0.01	0.03	down
0.02	0.05	down
0	0	down
0	0	down
0	0	down
0	0	down
0	0	up
0	0	down
0	0	down
0	0	down
0	0	up
0	0	up
0.00	0.00	up
0.00	0.00	up
0.00	0.00	down
0.00	0.00	up
0.00	0.00	down
0.00	0.00	down
0.00	0.00	up
0.00	0.00	down
0.00	0.00	up
0.00	0.00	down
0.00	0.00	down
0.00	0.00	down
0.00	0.00	down
0.00	0.00	down
0.00	0.00	down
0.00	0.00	down

0.00	0.00	down
0.00	0.00	up
0.00	0.00	down
0.00	0.00	down
0.00	0.00	down
0.00	0.00	down
0.00	0.00	down
0.00	0.00	down
0.00	0.00	down
0.00	0.00	up
0.00	0.00	down
0.00	0.00	down
0.00	0.00	down
0.00	0.00	up
0.00	0.00	down
0.00	0.00	down
0.00	0.00	down
0.00	0.00	down
0.00	0.00	up
0.00	0.00	up
0.00	0.00	up
0.00	0.00	down
0.00	0.00	up
0.00	0.00	down
0.00	0.00	down
0.00	0.00	down
0.00	0.00	down
0.00	0.00	down
0.00	0.00	up
0.00	0.00	down
0.00	0.00	down
0.00	0.00	down
0.00	0.00	down
0.00	0.00	down
0.00	0.00	up
0.00	0.00	up
0.00	0.00	up
0.00	0.00	down
0.00	0.00	up
0.00	0.01	down
0.00	0.01	up
0.00	0.01	down
0.01	0.04	up
0.00	0.00	down
0.00	0.00	down
0.00	0.00	down
0.00	0.00	down
0.00	0.00	down
0.00	0.00	down
0.00	0.01	down
0.00	0.00	down

0.00	0.00	down
0.00	0.00	down
0.00	0.00	down
0.00	0.00	down
0.00	0.00	down
0.00	0.00	down
0.00	0.00	down
0.00	0.00	down
0.00	0.00	down
0.00	0.00	down
0.00	0.00	down
0.00	0.01	up
0.00	0.01	down
0.00	0.01	down
0.01	0.04	down
0.00	0.00	down
0.00	0.00	down
0.00	0.00	down
0.00	0.00	down
0.00	0.00	down
0.00	0.00	down
0.00	0.00	down
0.00	0.01	down
0.00	0.00	down
0.00	0.00	up
0.00	0.00	down
0.00	0.00	down
0.00	0.00	up
0.00	0.00	up
0.00	0.00	up
0.00	0.00	down
0.00	0.00	down
0.00	0.00	up
0.00	0.00	down
0.00	0.00	down
0.00	0.00	down
0.00	0.01	down
0.00	0.02	down
0.01	0.02	down
0.01	0.03	down
0.02	0.05	down
0	0	up
0.00	0.00	down
0.00	0.00	down
0.00	0.00	down
0.00	0.00	down
0.00	0.00	down
0.00	0.00	down
0.00	0.00	down
0.00	0.00	down
0.00	0.00	down

0.00	0.00	down
0.00	0.00	down
0.00	0.01	down
0	0	down
0	0	down
0	0	down
0	0	up
0	0	down
0.00	0.00	down
0.00	0.00	down
0.00	0.00	up
0.00	0.00	up
0.00	0.00	down
0.00	0.00	up
0.00	0.00	down
0.00	0.00	up
0.00	0.00	down
0.00	0.02	down
0.01	0.04	up
0.00	0.00	down
0.00	0.00	up
0.00	0.00	down
0.00	0.00	up
0.00	0.00	up
0.00	0.00	up
0.00	0.00	up
0.01	0.03	down
0.02	0.05	down
0.00	0.00	down
0.00	0.00	up
0.00	0.00	down
0.00	0.00	up
0.00	0.00	up
0.00	0.00	up
0.00	0.00	up
0.01	0.03	down
0.02	0.05	down
0.00	0.00	down
0.00	0.00	up
0.00	0.00	down
0.00	0.00	up
0.00	0.00	up
0.00	0.00	up
0.00	0.00	up
0.01	0.03	down
0.02	0.05	down
0.00	0.00	down
0.00	0.00	down
0.00	0.00	down
0.00	0.00	down
0.00	0.00	down

0.00	0.00	down
0.00	0.00	down
0.00	0.00	down
0.00	0.00	down
0.00	0.00	down
0.00	0.00	down
0.00	0.00	down
0.00	0.00	down
0.00	0.00	down
0.00	0.00	down
0.00	0.00	down
0.00	0.01	down
0.00	0.01	down
0.00	0.01	down
0.00	0.01	down
0	0	down
0.00	0.00	up
0.00	0.00	up
0.00	0.00	down
0.00	0.00	up
0.00	0.00	up
0	0	up
0	0	down
0.00	0.00	down
0.00	0.00	down
0.00	0.00	down
0.00	0.00	down
0.00	0.00	down
0.00	0.00	down
0.00	0.00	down
0.00	0.00	down
0.00	0.00	down
0.00	0.00	down
0.00	0.00	down
0.00	0.00	down
0.00	0.00	down
0.00	0.00	down
0.00	0.00	down
0.00	0.01	down
0.00	0.01	down
0.00	0.01	down
0.01	0.03	down
0.01	0.03	down
0.01	0.04	down
0	0	up
0	0	down
0.00	0.00	up
0.00	0.00	up
0.00	0.00	down
0.00	0.00	down
0.00	0.00	down

0.00	0.00	down
0.00	0.00	down
0.00	0.00	down
0.00	0.00	down
0.00	0.00	up
0.00	0.00	down
0.00	0.00	up
0.00	0.00	down
0.00	0.00	up
0.00	0.00	up
0.00	0.00	down
0.00	0.00	up
0.00	0.00	down
0.00	0.00	up
0.00	0.00	up
0.00	0.00	down
0.00	0.00	down
0.00	0.00	up
0.00	0.00	down
0.00	0.00	down
0.00	0.00	up
0.00	0.00	up
0.00	0.00	up
0.00	0.00	up
0.00	0.00	up
0.00	0.00	down
0.00	0.00	down
0.00	0.00	down
0.00	0.00	down
0.00	0.00	down
0.00	0.00	down
0.00	0.00	down
0.01	0.02	down
0.01	0.02	up
0.01	0.02	up
0.01	0.03	down
0.02	0.05	down
0.00	0.00	down
0.00	0.00	up
0.00	0.00	down
0.00	0.00	up
0.00	0.00	up
0.00	0.00	up
0.00	0.00	up
0.01	0.03	down
0.02	0.05	down
0.00	0.00	down
0.00	0.00	down
0.00	0.00	up
0.00	0.00	down
0.00	0.00	down

0.00	0.00	up
0.00	0.00	down
0.00	0.00	down
0.00	0.00	down
0.00	0.00	down
0.00	0.00	down
0.00	0.00	up
0.00	0.00	down
0.00	0.00	down
0.00	0.01	down
0.01	0.03	down
0.00	0.00	up
0.00	0.00	up
0.00	0.00	up
0.00	0.00	up
0.00	0.00	up
0.00	0.00	up
0.00	0.00	up
0.00	0.00	up
0.00	0.00	up
0.00	0.00	up
0.00	0.00	up
0.00	0.00	up
0.00	0.00	up
0.00	0.00	up
0.00	0.00	up
0.00	0.00	up
0.00	0.00	up
0.00	0.00	up
0.00	0.00	up
0.00	0.00	down
0.00	0.00	down
0.00	0.00	down
0.00	0.01	down
0.00	0.01	down
0	0	down
0	0	down
0	0	down
0	0	down
0	0	down
0	0	up
0	0	down
0.00	0.00	down
0.00	0.00	up
0.00	0.00	down
0.00	0.00	down
0.00	0.00	down
0.00	0.00	up
0.00	0.00	down
0.00	0.00	down
0.00	0.00	down

0.00	0.00	down
0.00	0.00	down
0.00	0.00	down
0.00	0.00	down
0.00	0.00	down
0.00	0.00	down
0.00	0.00	down
0.00	0.00	down
0.00	0.00	down
0.00	0.00	up
0.00	0.00	down
0.00	0.00	up
0.00	0.00	down
0.00	0.00	up
0.00	0.00	down
0.00	0.00	down
0.00	0.00	up
0.00	0.00	down
0.00	0.00	up
0.00	0.00	down
0.00	0.00	down
0.00	0.00	down
0.00	0.00	down
0.00	0.00	up
0.00	0.00	down
0.00	0.00	down
0.00	0.00	down
0.00	0.00	up
0.00	0.00	up
0.00	0.00	up
0.00	0.00	up
0.00	0.00	up
0.00	0.00	down
0.00	0.00	up
0.00	0.00	down
0.00	0.00	down
0.00	0.00	down
0.00	0.00	up
0.00	0.00	down
0.00	0.00	up
0.00	0.00	down
0.00	0.00	up
0.00	0.00	down
0.00	0.00	down
0.00	0.00	down
0.00	0.00	up
0.00	0.00	down
0.00	0.00	down
0.00	0.00	up
0.00	0.00	up
0.00	0.00	up

0.00	0.00	up
0.00	0.01	up
0.00	0.01	down
0.00	0.02	down
0.00	0.02	down
0.01	0.02	down
0.01	0.03	down
0.01	0.04	up
0.01	0.04	down
0.01	0.04	up
0.01	0.05	down
0	0	down
0.00	0.00	down
0.00	0.00	down
0.00	0.00	down
0.00	0.01	down
0.00	0.00	up
0.00	0.00	down
0.00	0.00	up
0.00	0.00	down
0.00	0.00	up
0.00	0.00	up
0.00	0.00	down
0.00	0.00	up
0.00	0.00	up
0.00	0.00	up
0.00	0.00	down
0.00	0.00	down
0.00	0.00	up
0.00	0.00	up
0.01	0.03	down
0.01	0.04	up
0.02	0.05	down
0	0	down
0.00	0.00	up
0.00	0.00	down
0.00	0.00	down
0.00	0.00	down
0.00	0.00	down
0.00	0.00	down
0.00	0.00	down
0.00	0.00	down
0.00	0.00	down
0.00	0.00	down
0.00	0.00	up
0.00	0.00	down
0.00	0.00	up
0.00	0.00	down
0.00	0.00	down
0.00	0.00	down
0.00	0.00	down

0.00	0.00	down
0.00	0.00	up
0.00	0.00	down
0.00	0.00	up
0.00	0.00	down
0.00	0.00	down
0.00	0.00	down
0.00	0.00	down
0.00	0.00	down
0.00	0.00	down
0.00	0.00	down
0.00	0.00	up
0.00	0.00	down
0.00	0.00	up
0.00	0.00	up
0.00	0.00	up
0.00	0.00	down
0.00	0.00	down
0.00	0.00	down
0.00	0.00	down
0.00	0.00	down
0.00	0.00	down
0.00	0.00	down
0.00	0.00	up
0.00	0.00	up
0.00	0.00	up
0.00	0.00	down
0.00	0.00	down
0.00	0.00	up
0.00	0.00	down
0.00	0.00	up
0.00	0.00	down
0.00	0.00	down
0.00	0.00	up
0.00	0.00	up
0.00	0.00	down
0.00	0.00	down
0.00	0.00	down
0.00	0.00	down
0.00	0.00	down
0.00	0.00	up
0.00	0.00	down
0.00	0.00	down
0.00	0.00	down
0.00	0.00	down
0.00	0.00	down
0.00	0.00	up
0.00	0.00	down
0.00	0.00	down
0.00	0.00	down
0.00	0.00	up

0.00	0.01	down
0.00	0.01	down
0.00	0.01	down
0.00	0.01	down
0.00	0.01	down
0.00	0.01	down
0.00	0.01	down
0.00	0.01	up
0.00	0.01	up
0.00	0.01	down
0.01	0.02	down
0.01	0.02	down
0.01	0.03	down
0.01	0.03	down
0.01	0.03	down
0.01	0.03	down
0.01	0.03	down
0.01	0.04	down
0	0	down
0	0	down
0	0	down
0	0	down
0.00	0.00	up
0.00	0.00	up
0.00	0.00	up
0.00	0.00	up
0	0	down
0.00	0.00	up
0.00	0.00	down
0.00	0.00	down
0.00	0.00	down
0.00	0.00	down
0.00	0.00	up
0.00	0.00	down
0.00	0.00	down
0.00	0.00	down
0.00	0.00	down
0.00	0.00	up
0.00	0.00	down
0.00	0.00	up
0.00	0.00	down
0.00	0.00	up
0.00	0.00	down
0.00	0.00	up
0.00	0.00	down
0.00	0.00	down
0.00	0.00	down
0.00	0.00	down
0.00	0.00	down
0.00	0.00	down
0.00	0.00	down

0.00	0.00	down
0.00	0.00	down
0.00	0.00	down
0.00	0.00	down
0.00	0.00	down
0.00	0.00	down
0.00	0.00	up
0.00	0.00	up
0.00	0.00	up
0.00	0.00	up
0.00	0.00	up
0.00	0.00	down
0.00	0.00	down
0.00	0.00	down
0.00	0.00	up
0.00	0.00	up
0.00	0.00	up
0.00	0.00	down
0.00	0.00	up
0.00	0.00	up
0.00	0.00	down
0.00	0.00	down
0.00	0.00	down
0.00	0.00	down
0.00	0.00	up
0.00	0.00	down
0.00	0.00	up
0.00	0.00	down
0.00	0.00	down
0.00	0.00	down
0.00	0.00	down
0.00	0.00	down
0.00	0.00	down
0.00	0.00	down
0.00	0.00	up
0.00	0.00	up
0.00	0.00	down
0.00	0.00	down
0.00	0.00	down
0.00	0.00	down
0.00	0.00	down
0.00	0.00	down
0.00	0.00	down
0.00	0.00	down
0.00	0.00	up
0.00	0.00	down
0.00	0.00	down
0.00	0.00	up
0.00	0.00	down
0.00	0.00	up
0.00	0.00	up

0.00	0.00	up
0.00	0.00	up
0.00	0.00	up
0.00	0.00	down
0.00	0.00	up
0.00	0.00	down
0.00	0.00	up
0.00	0.00	down
0.00	0.00	up
0.00	0.00	up
0.00	0.00	down
0.00	0.00	down
0.00	0.00	down
0.00	0.00	down
0.00	0.00	up
0.00	0.00	up
0.00	0.00	down
0.00	0.00	up
0.00	0.00	down
0.00	0.00	up
0.00	0.00	down
0.00	0.00	down
0.00	0.00	down
0.00	0.00	up
0.00	0.00	down
0.00	0.00	down
0.00	0.00	down
0.00	0.00	up
0.00	0.00	down
0.00	0.00	down
0.00	0.00	down
0.00	0.00	down
0.00	0.00	down
0.00	0.00	up
0.00	0.00	up
0.00	0.00	down
0.00	0.00	down
0.00	0.00	up
0.00	0.00	down
0.00	0.00	down
0.00	0.00	up
0.00	0.00	down
0.00	0.00	up
0.00	0.00	up
0.00	0.01	down
0.00	0.01	up
0.00	0.01	down
0.00	0.01	down
0.00	0.01	down
0.00	0.01	down
0.00	0.01	down

0.00	0.01	down
0.00	0.01	down
0.00	0.01	down
0.00	0.02	down
0.01	0.02	down
0.01	0.02	down
0.01	0.02	down
0.01	0.02	down
0.01	0.03	down
0.01	0.03	up
0.01	0.03	down
0.01	0.03	down
0.01	0.03	down
0.01	0.03	down
0.01	0.03	down
0.01	0.03	down
0.01	0.04	up
0.01	0.04	down
0.01	0.04	down
0.01	0.04	down
0.01	0.05	down
0.02	0.05	down
0.00	0.00	up
0.00	0.00	up
0.00	0.00	up
0.00	0.00	up
0.00	0.00	up
0.00	0.00	up
0.00	0.00	up
0.00	0.00	up
0.00	0.00	up
0.00	0.00	up
0.00	0.00	up
0.00	0.00	up
0.00	0.00	up
0	0	down
0.00	0.00	down
0.00	0.00	down
0.00	0.00	up
0.00	0.00	down
0.00	0.00	down
0.00	0.00	down
0.00	0.00	down
0.00	0.00	up
0.00	0.00	down
0.00	0.00	down
0.00	0.00	down
0.00	0.00	down
0.00	0.00	down
0.00	0.00	down
0.00	0.01	down

0.00	0.01	down
0.00	0.02	down
0.02	0.05	up
0	0	down
0	0	down
0.00	0.00	down
0.00	0.00	down
0.00	0.00	down
0.00	0.00	down
0.00	0.00	down
0.00	0.00	down
0.00	0.00	down
0.00	0.00	down
0.00	0.00	up
0.00	0.02	down
0	0	down
0	0	down
0	0	down
0	0	down
0	0	up
0.00	0.00	down
0.00	0.00	down
0.00	0.00	down
0.00	0.00	down
0.00	0.00	up
0.00	0.00	down
0.00	0.00	down
0.00	0.00	down
0.00	0.00	up
0.00	0.00	down
0.00	0.00	down
0.00	0.00	down
0.00	0.00	down
0.00	0.00	down
0.00	0.00	down
0.00	0.00	down
0.00	0.00	down
0.00	0.00	down
0.00	0.00	down
0.00	0.00	up
0.00	0.00	down
0.00	0.00	down
0.00	0.00	down
0.00	0.00	down
0.00	0.00	up
0.00	0.00	up
0.00	0.00	down
0.00	0.00	down
0.00	0.00	up
0.00	0.00	up
0.00	0.00	up

0.00	0.00	up
0.00	0.00	down
0.00	0.00	down
0.00	0.00	up
0.00	0.00	down
0.00	0.00	down
0.00	0.00	down
0.00	0.00	down
0.00	0.00	down
0.00	0.00	down
0.00	0.00	up
0.00	0.00	up
0.00	0.01	down
0.00	0.01	up
0.00	0.01	down
0.00	0.02	down
0.01	0.02	down
0.01	0.02	up
0.01	0.03	up
0.01	0.03	down
0.01	0.03	down
0.01	0.04	down
0.01	0.04	up
0.01	0.05	down
0.00	0.00	up
0.00	0.00	up
0.00	0.00	up
0.00	0.00	up
0.00	0.00	up
0	0	up
0.00	0.00	up
0.00	0.00	down
0.00	0.00	down
0.00	0.00	up
0.00	0.00	up
0.00	0.00	down
0.00	0.00	up
0.00	0.00	down
0.00	0.00	up
0.00	0.00	down
0.00	0.00	down
0.00	0.00	down
0.00	0.00	down
0.00	0.00	down
0.00	0.00	down
0.00	0.00	down
0.00	0.00	down
0.00	0.00	up
0.00	0.00	up
0.00	0.00	up
0.00	0.00	up

0.00	0.00	up
0.00	0.00	down
0.00	0.00	down
0.00	0.00	down
0.00	0.00	up
0.00	0.00	up
0.00	0.00	down
0.00	0.00	up
0.00	0.00	down
0.00	0.00	down
0.00	0.00	down
0.00	0.00	up
0.00	0.00	down
0.00	0.00	up
0.00	0.00	down
0.00	0.00	down
0.00	0.00	up
0.00	0.00	down
0.00	0.00	up
0.00	0.00	down
0.00	0.00	up
0.00	0.00	down
0.00	0.00	down
0.00	0.00	down
0.00	0.00	down
0.00	0.00	up
0.00	0.00	down
0.00	0.00	down
0.00	0.00	down
0.00	0.00	up
0.00	0.00	up
0.00	0.00	up
0.00	0.00	up
0.00	0.00	up
0.00	0.00	down
0.00	0.00	up
0.00	0.00	up
0.00	0.00	down
0.00	0.00	down
0.00	0.00	up
0.00	0.00	up
0.00	0.00	down
0.00	0.00	up
0.00	0.00	down
0.00	0.00	up
0.00	0.00	down
0.00	0.00	down
0.00	0.00	up
0.00	0.00	down
0.00	0.00	down
0.00	0.00	down

0.00	0.00	down
0.00	0.00	up
0.00	0.00	up
0.00	0.00	down
0.00	0.00	down
0.00	0.00	up
0.00	0.00	up
0.00	0.00	down
0.00	0.00	down
0.00	0.00	down
0.00	0.00	up
0.00	0.00	down
0.00	0.00	down
0.00	0.00	up
0.00	0.01	up
0.00	0.01	down
0.00	0.01	down
0.00	0.01	down
0.00	0.01	down
0.00	0.01	down
0.00	0.01	down
0.00	0.02	up
0.01	0.02	down
0.01	0.02	down
0.01	0.02	down
0.01	0.02	down
0.01	0.03	down
0.01	0.03	down
0.01	0.03	down
0.01	0.03	down
0.01	0.04	up
0.01	0.04	down
0.01	0.04	down
0.00	0.00	up
0.00	0.00	down
0.00	0.00	down
0.00	0.00	down
0.00	0.00	up
0.00	0.00	up
0.00	0.00	down
0.00	0.00	down
0.00	0.00	down
0.00	0.00	down
0.00	0.00	down
0.00	0.00	down
0.00	0.00	down
0.00	0.00	down
0.00	0.00	up
0.00	0.00	up
0.00	0.00	down
0.00	0.00	down

0.00	0.00	down
0.00	0.00	down
0.00	0.00	up
0.00	0.00	down
0.00	0.00	down
0.00	0.00	down
0.00	0.00	down
0.00	0.00	down
0.00	0.00	down
0.00	0.00	down
0.00	0.00	down
0.00	0.00	down
0.00	0.00	down
0.00	0.00	down
0.00	0.00	down
0.00	0.00	down
0.00	0.00	down
0.00	0.00	down
0.00	0.00	up
0.00	0.00	down
0.00	0.00	down
0.00	0.00	up
0.00	0.00	down
0.00	0.00	down
0.00	0.00	down
0.00	0.00	down
0.00	0.00	down
0.00	0.00	down
0.00	0.00	down
0.00	0.00	up
0.00	0.00	down
0.00	0.00	up
0.00	0.00	down
0.00	0.01	down
0.00	0.01	up
0.00	0.01	down
0.00	0.01	down
0.00	0.01	up
0.00	0.01	down
0.00	0.01	down
0.00	0.01	down
0.00	0.02	down
0.01	0.02	down
0.01	0.02	down
0.01	0.02	down
0.01	0.02	down
0.01	0.03	down
0.01	0.03	down
0.01	0.03	down
0.01	0.03	down
0.01	0.04	up

0.01	0.04	down
0.01	0.04	down
0.02	0.05	down
0	0	down
0.00	0.00	down
0.00	0.00	down
0.00	0.00	down
0.00	0.00	down
0.00	0.00	down
0.00	0.00	down
0.00	0.00	down
0.00	0.00	up
0.00	0.00	down
0.00	0.00	down
0.00	0.00	down
0.00	0.00	down
0.00	0.00	down
0.00	0.00	down
0.00	0.00	down
0.00	0.00	up
0.00	0.00	down
0.00	0.00	down
0.00	0.00	down
0.00	0.00	down
0.00	0.00	down
0.00	0.00	up
0.00	0.00	down
0.00	0.00	up
0.00	0.00	down
0.00	0.00	down
0.00	0.00	down
0.00	0.00	up
0.00	0.00	down
0.00	0.00	down
0.00	0.00	down
0.00	0.00	down
0.00	0.00	down
0.00	0.00	down
0.00	0.00	down
0.00	0.01	down
0.00	0.01	down
0.00	0.01	down
0.01	0.02	down
0.01	0.03	down
0.01	0.03	down
0.01	0.03	down
0	0	down
0	0	down
0.00	0.00	down
0.00	0.00	down
0.00	0.00	up

0.00	0.00	down
0.00	0.00	down
0.00	0.00	up
0.00	0.00	down
0.00	0.00	down
0.00	0.00	down
0.00	0.00	down
0.00	0.00	down
0.00	0.00	down
0.00	0.00	up
0.00	0.00	down
0.00	0.00	down
0.00	0.00	down
0.00	0.00	down
0.00	0.00	down
0.00	0.00	down
0.00	0.00	down
0.00	0.00	down
0.00	0.00	down
0.00	0.00	down
0.00	0.00	down
0.00	0.00	up
0.00	0.00	up
0.00	0.00	down
0.00	0.00	down
0.00	0.00	down
0.00	0.00	down
0.00	0.00	down
0.00	0.00	up
0.00	0.00	down
0.00	0.00	down
0.00	0.00	down
0.00	0.00	down
0.00	0.00	up
0.00	0.00	down
0.00	0.00	down
0.00	0.00	down
0.00	0.00	down
0.00	0.00	down
0.00	0.00	down
0.00	0.01	up
0.00	0.01	down
0.00	0.01	down
0.00	0.02	down
0.01	0.02	up
0.01	0.02	up
0.01	0.02	up
0.01	0.02	down
0.01	0.03	down
0.01	0.04	down
0.01	0.04	down

0.01	0.05	up
0.02	0.05	down
0	0	down
0	0	up
0	0	down
0	0	up
0.00	0.00	down
0.00	0.00	down
0.00	0.00	down
0.00	0.00	up
0.00	0.00	up
0.00	0.00	down
0.00	0.00	up
0.00	0.00	down
0.00	0.00	up
0.00	0.00	up
0.00	0.00	up
0.00	0.00	down
0.00	0.00	down
0.00	0.00	down
0.00	0.01	down
0.00	0.01	down
0.01	0.02	down
0	0	down
0.00	0.00	up
0.00	0.00	down
0.00	0.00	down
0.00	0.00	down
0.00	0.00	down
0.00	0.00	down
0.00	0.00	down
0.00	0.00	down
0.00	0.00	down
0.00	0.00	up
0.00	0.00	down
0.00	0.00	up
0.00	0.00	down
0.00	0.00	down
0.00	0.00	down
0.00	0.00	down
0.00	0.00	down
0.00	0.00	up
0.00	0.00	down
0.00	0.00	up
0.00	0.00	down
0.00	0.00	down
0.00	0.00	down
0.00	0.00	down
0.00	0.00	down
0.00	0.00	down
0.00	0.00	down

0.00	0.00	up
0.00	0.00	down
0.00	0.00	up
0.00	0.00	up
0.00	0.00	up
0.00	0.00	down
0.00	0.00	down
0.00	0.00	down
0.00	0.00	down
0.00	0.00	down
0.00	0.00	down
0.00	0.00	down
0.00	0.00	up
0.00	0.00	up
0.00	0.00	up
0.00	0.00	down
0.00	0.00	down
0.00	0.00	down
0.00	0.00	down
0.00	0.00	down
0.00	0.00	up
0.00	0.00	up
0.00	0.00	down
0.00	0.00	down
0.00	0.00	down
0.00	0.00	down
0.00	0.00	down
0.00	0.00	down
0.00	0.00	down
0.00	0.00	down
0.00	0.00	down
0.00	0.00	down
0.00	0.00	down
0.00	0.00	up
0.00	0.00	down
0.00	0.00	down
0.00	0.00	down
0.00	0.00	up
0.00	0.01	down
0.00	0.01	down
0.00	0.01	down
0.00	0.01	down
0.00	0.01	down
0.00	0.01	down
0.00	0.01	down
0.00	0.01	up
0.00	0.01	up
0.00	0.01	down
0.01	0.02	down
0.01	0.02	down
0.01	0.03	down

0.01	0.03	down
0.01	0.03	down
0.01	0.03	down
0.01	0.03	down
0.01	0.04	down
0	0	down
0.00	0.00	up
0.00	0.00	up
0.00	0.00	up
0	0	down
0.00	0.00	up
0.00	0.00	up
0.00	0.00	up
0	0	down
0.00	0.00	down
0.00	0.00	down
0.00	0.00	down
0.00	0.00	down
0.00	0.00	down
0	0	up
0	0	down
0	0	down
0	0	up
0.00	0.00	up
0.00	0.00	up
0.00	0.00	down
0.00	0.00	down
0.00	0.00	down
0.00	0.00	down
0.00	0.00	down
0.00	0.00	down
0.00	0.00	up
0.00	0.00	down
0.00	0.00	down
0.00	0.00	down
0.00	0.00	down
0.00	0.00	down
0.00	0.00	down
0.00	0.00	down
0.00	0.00	down
0.00	0.00	down
0.00	0.00	down
0.00	0.00	up
0.00	0.00	down
0.00	0.00	down
0.00	0.00	down
0.00	0.00	down
0.00	0.00	down
0.00	0.00	down
0.00	0.00	down
0.00	0.00	down

0.00	0.00	down
0.00	0.00	up
0.00	0.00	up
0.00	0.00	down
0.00	0.00	down
0.00	0.00	up
0.00	0.00	down
0.00	0.00	down
0.00	0.00	down
0.00	0.00	down
0.00	0.00	down
0.00	0.00	down
0.00	0.00	down
0.00	0.00	down
0.00	0.00	down
0.00	0.00	down
0.00	0.00	down
0.00	0.00	up
0.00	0.00	up
0.00	0.00	down
0.00	0.00	down
0.00	0.00	down
0.00	0.00	up
0.00	0.00	up
0.00	0.00	down
0.00	0.00	down
0.00	0.00	down
0.00	0.00	down
0.00	0.00	down
0.00	0.00	down
0.00	0.00	down
0.00	0.00	down
0.00	0.00	down
0.00	0.00	down
0.00	0.00	down
0.00	0.00	up
0.00	0.00	up
0.00	0.00	down
0.00	0.00	down
0.00	0.00	down
0.00	0.00	down
0.00	0.00	down
0.00	0.00	down
0.00	0.00	down
0.00	0.00	down
0.00	0.00	down
0.00	0.00	down
0.00	0.00	down
0.00	0.00	down
0.00	0.00	up
0.00	0.00	down

0.00	0.00	down
0.00	0.00	down
0.00	0.00	up
0.00	0.00	down
0.00	0.00	down
0.00	0.00	up
0.00	0.00	down
0.00	0.00	down
0.00	0.00	down
0.00	0.00	down
0.00	0.00	down
0.00	0.00	down
0.00	0.00	down
0.00	0.00	down
0.00	0.01	up
0.00	0.01	up
0.00	0.01	up
0.00	0.01	down
0.00	0.01	down
0.00	0.01	down
0.00	0.01	up
0.00	0.01	down
0.00	0.01	up
0.00	0.01	down
0.00	0.01	up
0.00	0.01	down
0.00	0.01	down
0.00	0.01	up
0.00	0.01	down
0.00	0.02	down
0.00	0.02	down
0.01	0.02	down
0.01	0.02	down
0.01	0.03	down
0.01	0.03	down
0.01	0.03	down
0.01	0.04	down
0.01	0.04	down
0.01	0.04	down
0.01	0.04	down
0.01	0.05	up
0.01	0.05	up
0.02	0.05	down
0	0	down
0.00	0.00	down
0.00	0.00	down
0.00	0.00	down
0.00	0.00	down
0.00	0.00	down
0	0	down
0	0	up

0.00	0.00	down
0.00	0.00	up
0.01	0.04	up
0	0	up
0.00	0.00	down
0.00	0.00	up
0.00	0.00	down
0.00	0.00	down
0.00	0.00	up
0.00	0.00	down
0.00	0.00	up
0.00	0.00	down
0.00	0.00	down
0.00	0.01	up
0.01	0.03	down
0.02	0.05	down
0.00	0.00	down
0.00	0.00	down
0.00	0.00	down
0.00	0.00	down
0.00	0.00	up
0.00	0.01	down
0	0	down
0	0	down
0	0	down
0	0	down
0	0	down
0.00	0.00	down
0.00	0.00	up
0.00	0.00	down
0.00	0.00	down
0.00	0.00	down
0.00	0.00	up
0.00	0.00	up
0.00	0.00	up
0.00	0.00	up
0.00	0.00	up
0.00	0.00	down
0.00	0.00	up
0.00	0.00	down
0.00	0.00	down
0.00	0.00	down
0.00	0.00	down
0.00	0.00	down
0.00	0.00	up
0.00	0.00	down
0.00	0.00	up
0.00	0.00	down
0.00	0.00	up
0.00	0.00	down
0.00	0.00	down

0.00	0.00	up
0.00	0.00	down
0.00	0.00	up
0.00	0.00	down
0.00	0.00	down
0.00	0.00	down
0.00	0.00	down
0.00	0.00	down
0.00	0.00	up
0.00	0.00	down
0.00	0.00	down
0.00	0.00	up
0.00	0.00	down
0.00	0.00	down
0.00	0.01	down
0.00	0.01	down
0.01	0.03	down
0.01	0.03	down
0.01	0.03	down
0.01	0.03	down
0.02	0.05	down
0.00	0.00	down
0.00	0.00	down
0.00	0.00	down
0.00	0.00	down
0.00	0.00	down
0.00	0.00	down
0.00	0.00	down
0.00	0.01	down
0.00	0.00	down
0.00	0.00	down
0.00	0.00	down
0.00	0.00	down
0.00	0.00	down
0.00	0.00	down
0.00	0.00	down
0.00	0.00	down
0.00	0.01	down
0.00	0.00	up
0.00	0.00	up
0.00	0.00	up
0.00	0.00	down
0.00	0.00	down
0.00	0.00	up
0.00	0.00	down
0.00	0.00	down
0.00	0.00	down
0.00	0.00	up
0.01	0.04	up
0.00	0.00	down
0.00	0.00	down

0.00	0.00	down
0.00	0.00	down
0.00	0.00	down
0.00	0.01	down
0.01	0.04	down
0.02	0.05	down
0	0	down
0.00	0.00	down
0.00	0.00	down
0.01	0.02	down
0.00	0.00	down
0.00	0.00	down
0.00	0.01	up
0.00	0.02	down
0.00	0.00	down
0.00	0.00	down
0.00	0.00	down
0.00	0.00	down
0.00	0.00	down
0.00	0.00	down
0.00	0.01	down
0	0	down
0.00	0.00	down
0.00	0.00	down
0.00	0.00	down
0.00	0.00	down
0.00	0.00	down
0.00	0.00	down
0.00	0.00	down
0.00	0.00	down
0.00	0.00	up
0.00	0.00	down
0.00	0.00	down
0.00	0.00	up
0.00	0.00	down
0.00	0.00	up
0.00	0.00	down
0.00	0.00	down
0.00	0.00	down
0.00	0.00	up
0.00	0.00	down
0.00	0.00	down
0.00	0.00	down
0.00	0.00	down
0.00	0.00	down
0.00	0.00	down
0.00	0.00	down
0.00	0.00	down
0.00	0.00	up
0.00	0.00	up
0.00	0.00	up

0.00	0.00	down
0.00	0.00	down
0.00	0.00	down
0.00	0.00	down
0.00	0.00	down
0.00	0.00	down
0.00	0.00	down
0.00	0.00	down
0.00	0.00	down
0.00	0.00	down
0.00	0.00	down
0.00	0.00	down
0.00	0.00	up
0.00	0.00	down
0.00	0.00	up
0.00	0.00	down
0.00	0.00	down
0.00	0.01	down
0.00	0.01	down
0.00	0.01	down
0.00	0.01	down
0.00	0.01	up
0.00	0.01	down
0.01	0.03	down
0.01	0.04	down
0.02	0.05	down
0	0	down
0.00	0.00	down
0.00	0.00	down
0.00	0.00	down
0.00	0.00	down
0.00	0.00	down
0.00	0.00	down
0.00	0.00	down
0.00	0.00	down
0.00	0.00	up
0.00	0.00	up
0.00	0.00	up
0.00	0.00	down
0	0	down
0.00	0.00	down
0	0	down
0	0	down
0.00	0.00	up
0.00	0.00	up
0.00	0.00	down
0.00	0.00	down
0.00	0.00	down
0.00	0.00	down
0.00	0.00	up
0.00	0.00	up

0.00	0.00	down
0.01	0.02	down
0.00	0.00	up
0.00	0.00	up
0.00	0.00	up
0.00	0.00	up
0.00	0.00	up
0.00	0.00	up
0.00	0.00	up
0.00	0.00	up
0.00	0.00	up
0.00	0.00	down
0.00	0.00	up
0.00	0.00	up
0.00	0.00	up
0.00	0.00	up
0.00	0.01	down
0.01	0.02	up
0.00	0.00	down
0.00	0.00	down
0.00	0.00	down
0.00	0.00	down
0.00	0.00	down
0	0	down
0.00	0.00	down
0.00	0.00	up
0.00	0.00	up
0.00	0.00	up
0.00	0.00	down
0.00	0.00	up
0.00	0.00	up
0.00	0.01	up
0.00	0.02	down
0.01	0.04	down
0.01	0.04	down
0.00	0.00	down
0.00	0.00	down
0.00	0.00	down
0.00	0.00	down
0	0	up
0.00	0.00	up
0.00	0.00	up
0.00	0.00	down
0.00	0.00	down
0.00	0.00	down
0.00	0.00	up
0.00	0.00	up
0.00	0.00	down
0.00	0.00	down
0.00	0.00	down
0.00	0.00	down

0.00	0.00	down
0.00	0.00	down
0.00	0.00	down
0.00	0.00	down
0.00	0.00	down
0.00	0.00	down
0.00	0.00	up
0.00	0.00	up
0.00	0.00	down
0.00	0.00	down
0.00	0.00	down
0.00	0.00	down
0.00	0.00	up
0.00	0.00	down
0.00	0.00	down
0.00	0.00	down
0.00	0.00	down
0.00	0.00	down
0.00	0.00	down
0.00	0.00	up
0.00	0.00	up
0.00	0.00	down
0.00	0.00	down
0.00	0.00	down
0.00	0.00	down
0.00	0.00	down
0.00	0.00	down
0.00	0.00	up
0.00	0.00	down
0.00	0.00	down
0.00	0.00	down
0.00	0.00	up
0.00	0.00	down
0.00	0.00	down
0.00	0.00	down
0.00	0.00	down
0.00	0.00	up
0.00	0.00	down
0.00	0.00	up
0.00	0.00	up
0.00	0.00	down
0.00	0.00	down
0.00	0.00	down
0.00	0.00	down
0.00	0.00	up
0.00	0.00	down
0.00	0.00	up
0.00	0.00	down
0.00	0.00	down
0.00	0.00	down
0.00	0.00	up

0.00	0.00	up
0.00	0.00	down
0.00	0.00	up
0.00	0.00	down
0.00	0.00	down
0.00	0.00	down
0.00	0.00	up
0.00	0.00	down
0.00	0.00	up
0.00	0.01	down
0.00	0.01	up
0.00	0.01	down
0.00	0.01	down
0.00	0.01	down
0.00	0.01	down
0.00	0.01	down
0.00	0.01	down
0.00	0.02	down
0.01	0.02	up
0.01	0.02	down
0.01	0.02	down
0.01	0.03	down
0.01	0.03	down
0.01	0.03	down
0.01	0.03	down
0.01	0.03	down
0.01	0.04	down
0.00	0.00	up
0.00	0.00	down
0.00	0.00	down
0.00	0.00	down
0.00	0.00	down
0.00	0.00	up
0.00	0.00	down
0.00	0.00	down
0.00	0.00	down
0.00	0.00	down
0.00	0.00	up
0.00	0.00	up
0.00	0.00	down
0.00	0.00	down
0.00	0.00	down
0.00	0.00	down
0.00	0.00	down
0.00	0.00	down
0.00	0.00	up
0.00	0.00	down
0.00	0.00	down
0.00	0.00	down
0.00	0.00	down
0.00	0.00	down

0.00	0.00	down
0.00	0.00	down
0.00	0.00	down
0.00	0.00	up
0.00	0.00	down
0.00	0.00	down
0.00	0.00	up
0.00	0.00	down
0.00	0.00	up
0.00	0.00	down
0.00	0.00	down
0.00	0.01	down
0.00	0.01	down
0.01	0.02	down
0.01	0.04	down
0.01	0.04	down
0.01	0.05	down
0.00	0.00	down
0.00	0.00	down
0.00	0.00	down
0.00	0.01	down
0.02	0.05	down
0.00	0.00	down
0.00	0.00	down
0.00	0.00	down
0.00	0.01	down
0.02	0.05	down
0.00	0.00	down
0.00	0.00	down
0.00	0.00	down
0	0	down
0.00	0.00	down
0.00	0.00	down
0.00	0.00	down
0.00	0.00	down
0.00	0.00	down
0.00	0.00	down
0.00	0.00	down
0.00	0.00	down
0.00	0.00	down
0.00	0.00	down
0.01	0.04	down
0.01	0.05	up
0.00	0.00	down
0.00	0.00	down
0	0	down
0	0	up
0.00	0.00	down
0.00	0.00	down
0.00	0.00	up
0.00	0.00	up

0.00	0.00	down
0.00	0.00	up
0.00	0.00	down
0.00	0.00	up
0.00	0.00	down
0.00	0.00	down
0.00	0.00	up
0.00	0.00	down
0.00	0.00	up
0.00	0.00	up
0.00	0.00	down
0.00	0.00	down
0.00	0.00	down
0.00	0.00	down
0.00	0.00	down
0.00	0.00	up
0.00	0.00	down
0.00	0.00	down
0.00	0.00	down
0.01	0.03	down
0.00	0.00	down
0.00	0.00	up
0.00	0.00	down
0.00	0.00	down
0	0	up
0	0	down
0	0	down
0	0	up
0	0	down
0.00	0.00	up
0.00	0.00	up
0.00	0.00	up
0.00	0.00	down
0.00	0.00	down
0.00	0.00	down
0.00	0.00	down
0.00	0.00	down
0.00	0.00	down
0.00	0.00	down
0.00	0.00	down
0.00	0.00	up
0.00	0.00	down
0.00	0.00	down
0.00	0.00	down
0.00	0.00	down
0.00	0.00	down
0.00	0.00	down
0.00	0.00	down
0.00	0.00	down
0.00	0.00	down
0.00	0.00	up

0.00	0.00	down
0.00	0.00	down
0.00	0.00	down
0.00	0.00	down
0.00	0.00	down
0.00	0.00	down
0.00	0.00	down
0.00	0.00	down
0.00	0.00	down
0.00	0.00	up
0.00	0.00	up
0.00	0.00	down
0.00	0.00	down
0.00	0.00	down
0.00	0.00	up
0.00	0.00	down
0.00	0.00	down
0.00	0.00	down
0.00	0.00	down
0.00	0.00	down
0.00	0.00	down
0.00	0.00	down
0.00	0.00	down
0.00	0.00	up
0.00	0.00	up
0.00	0.00	down
0.00	0.00	down
0.00	0.00	down
0.00	0.00	down
0.00	0.00	down
0.00	0.00	down
0.00	0.00	up
0.00	0.00	down
0.00	0.00	down
0.00	0.00	down
0.00	0.00	down
0.00	0.00	down
0.00	0.00	up
0.00	0.00	down
0.00	0.00	down
0.00	0.00	down
0.00	0.00	down
0.00	0.00	down
0.00	0.00	down
0.00	0.00	down
0.00	0.00	down
0.00	0.00	up
0.00	0.00	down
0.00	0.00	down
0.00	0.00	up
0.00	0.00	down

0.00	0.00	down
0.00	0.00	down
0.00	0.00	down
0.00	0.00	up
0.00	0.00	up
0.00	0.00	down
0.00	0.00	down
0.00	0.00	down
0.00	0.00	down
0.00	0.00	down
0.00	0.00	down
0.00	0.00	down
0.00	0.00	down
0.00	0.00	down
0.00	0.00	down
0.00	0.00	down
0.00	0.00	down
0.00	0.00	down
0.00	0.00	down
0.00	0.00	down
0.00	0.00	down
0.00	0.00	down
0.00	0.00	down
0.00	0.00	down
0.00	0.00	down
0.00	0.00	up
0.00	0.00	down
0.00	0.00	down
0.00	0.00	down
0.00	0.00	down
0.00	0.00	down
0.00	0.00	up
0.00	0.00	down
0.00	0.00	down
0.00	0.00	up
0.00	0.00	up
0.00	0.00	down
0.00	0.00	down
0.00	0.00	down
0.00	0.00	down
0.00	0.00	down
0.00	0.00	down
0.00	0.00	up
0.00	0.00	down
0.00	0.00	down
0.00	0.00	down
0.00	0.00	up
0.00	0.00	up
0.00	0.00	down
0.00	0.00	down
0.00	0.00	down

0.00	0.00	down
0.00	0.00	down
0.00	0.00	down
0.00	0.00	down
0.00	0.00	down
0.00	0.00	down
0.00	0.00	down
0.00	0.00	up
0.00	0.00	down
0.00	0.00	down
0.00	0.00	down
0.00	0.00	down
0.00	0.00	down
0.00	0.00	down
0.00	0.00	down
0.00	0.00	down
0.00	0.00	down
0.00	0.00	down
0.00	0.00	down
0.00	0.00	up
0.00	0.00	down
0.00	0.00	down
0.00	0.00	down
0.00	0.00	down
0.00	0.00	down
0.00	0.00	down
0.00	0.00	down
0.00	0.00	down
0.00	0.00	down
0.00	0.00	up
0.00	0.00	down
0.00	0.00	down
0.00	0.00	up
0.00	0.00	down
0.00	0.00	down
0.00	0.00	down
0.00	0.00	down
0.00	0.00	down
0.00	0.00	up
0.00	0.00	down
0.00	0.00	up
0.00	0.00	down
0.00	0.00	down
0.00	0.00	down
0.00	0.00	down
0.00	0.00	down
0.00	0.00	up
0.00	0.00	down
0.00	0.00	down
0.00	0.00	down
0.00	0.00	down

0.00	0.00	down
0.00	0.00	down
0.00	0.00	down
0.00	0.00	down
0.00	0.00	down
0.00	0.00	up
0.00	0.00	up
0.00	0.01	down
0.00	0.01	up
0.00	0.01	down
0.00	0.01	down
0.00	0.01	down
0.00	0.01	down
0.00	0.01	down
0.00	0.01	down
0.00	0.01	up
0.00	0.01	down
0.00	0.01	down
0.00	0.01	down
0.00	0.01	up
0.00	0.01	down
0.00	0.01	down
0.00	0.01	down
0.00	0.01	down
0.00	0.01	down
0.00	0.01	down
0.00	0.01	down
0.00	0.02	down
0.00	0.02	up
0.00	0.02	down
0.01	0.02	down
0.01	0.02	down
0.01	0.02	up
0.01	0.02	up
0.01	0.02	down
0.01	0.02	up
0.01	0.02	down
0.01	0.02	down
0.01	0.02	up
0.01	0.02	down
0.01	0.03	down
0.01	0.03	down
0.01	0.03	up
0.01	0.03	down
0.01	0.03	down
0.01	0.04	down
0.01	0.04	down
0.01	0.04	down
0.01	0.04	down
0.01	0.04	down
0.01	0.04	down

0.01	0.05	up
0.01	0.05	up
0.02	0.05	down
0	0	up
0.00	0.00	up
0.00	0.00	down
0.00	0.00	up
0.00	0.01	down
0.00	0.00	down
0.00	0.00	down
0.00	0.00	down
0.00	0.00	down
0.00	0.00	down
0.00	0.00	up
0.00	0.00	down
0.00	0.01	up
0.00	0.02	down
0.00	0.00	down
0.00	0.00	down
0.00	0.00	up
0.00	0.00	up
0.00	0.00	down
0.00	0.00	down
0.00	0.00	down
0.00	0.00	down
0.00	0.00	up
0.00	0.00	up
0.00	0.00	down
0.01	0.02	down
0.00	0.00	up
0.00	0.00	down
0.00	0.00	down
0.00	0.00	up
0.00	0.00	down
0.00	0.00	down
0.00	0.00	down
0.00	0.00	down
0	0	down
0.00	0.00	down
0.00	0.00	down
0.00	0.00	down
0.00	0.00	down
0.00	0.00	up
0.00	0.01	down
0.00	0.01	down
0.00	0.00	down
0.00	0.00	down
0.00	0.00	down
0.00	0.00	up
0.00	0.00	down
0.00	0.00	down

0.00	0.00	down
0.00	0.00	up
0.00	0.00	down
0.00	0.00	down
0.00	0.00	down
0.00	0.00	down
0.00	0.00	up
0.00	0.00	down
0.00	0.00	down
0.00	0.00	up
0.00	0.00	down
0.00	0.00	up
0.00	0.00	down
0.01	0.03	down
0.00	0.00	down
0.00	0.00	down
0.00	0.00	down
0.00	0.00	down
0.00	0.00	down
0.00	0.00	down
0.00	0.00	down
0.00	0.00	down
0.00	0.00	down
0.00	0.00	down
0.00	0.00	down
0.00	0.01	down
0.01	0.02	down
0.01	0.02	down
0.01	0.03	down
0	0	down
0.00	0.00	down
0.00	0.00	down
0.00	0.00	down
0.00	0.00	down
0.01	0.04	up
0.00	0.00	down
0.00	0.00	down
0.00	0.00	down
0.00	0.00	down
0.00	0.00	down
0.00	0.00	down
0.00	0.00	down
0.00	0.00	down
0.00	0.00	down
0.00	0.00	up
0.00	0.00	down
0.00	0.00	down
0.00	0.00	up
0.00	0.00	down
0.00	0.00	up
0.00	0.00	down

0.00	0.00	down
0.00	0.00	up
0	0	down
0.00	0.00	down
0.00	0.00	up
0.00	0.00	down
0.00	0.00	up
0.00	0.00	up
0.00	0.00	down
0.00	0.00	up
0.00	0.00	up
0.00	0.01	up
0.00	0.02	down
0.01	0.04	down
0.01	0.04	down
0	0	up
0.00	0.00	down
0.00	0.00	down
0.00	0.00	up
0.00	0.00	down
0.00	0.00	up
0.01	0.03	down
0.01	0.03	down
0	0	down
0	0	down
0.00	0.00	up
0.00	0.00	down
0.00	0.00	up
0.00	0.00	down
0.00	0.00	down
0.00	0.00	up
0.00	0.00	down
0.00	0.00	down
0.00	0.00	down
0.00	0.00	down
0.00	0.00	down
0.00	0.00	down
0.00	0.00	down
0.00	0.00	down
0.00	0.00	up
0.00	0.00	down
0.00	0.00	down
0.00	0.00	down
0.00	0.00	down
0.00	0.00	down
0.00	0.01	down
0.00	0.02	down
0.01	0.03	down
0.02	0.05	down
0.00	0.00	down
0.00	0.00	down

0.00	0.00	down
0	0	down
0	0	down
0	0	down
0.00	0.00	down
0.00	0.00	down
0.00	0.00	down
0.00	0.00	down
0.00	0.00	down
0.00	0.00	down
0.00	0.00	down
0.00	0.00	down
0.00	0.00	up
0.00	0.00	up
0.00	0.00	down
0.00	0.00	down
0.00	0.00	down
0.00	0.00	down
0.00	0.00	down
0.00	0.00	down
0.00	0.00	down
0.00	0.00	down
0.00	0.00	down
0.00	0.01	down
0.00	0.01	down
0.01	0.03	down
0.00	0.00	down
0.00	0.00	down
0.00	0.00	down
0.00	0.00	down
0.00	0.00	up
0.00	0.00	down
0.00	0.00	down
0.00	0.00	up
0.00	0.00	up
0.00	0.01	down
0.00	0.01	down
0.01	0.02	down
0	0	down
0.00	0.00	down
0	0	down
0.00	0.00	down
0.00	0.00	down
0.00	0.00	down
0.00	0.00	down
0.00	0.01	down
0.00	0.00	down
0.00	0.01	down
0.00	0.00	down
0.00	0.01	down
0.00	0.00	up

0.00	0.00	up
0.00	0.00	down
0.00	0.02	up
0.00	0.00	down
0.00	0.02	up
0.00	0.00	up
0.00	0.00	up
0.00	0.00	up
0.00	0.00	up
0.00	0.00	up
0.00	0.00	up
0.00	0.00	down
0.00	0.00	down
0.00	0.00	down
0.00	0.00	up
0.00	0.00	down
0	0	down
0	0	down
0	0	down
0.00	0.00	down
0.00	0.00	down
0.00	0.00	up
0.00	0.00	down
0.00	0.00	up
0.01	0.04	up
0.00	0.00	down
0.00	0.00	up
0.00	0.00	down
0.00	0.00	down
0.00	0.00	down
0.00	0.01	up
0.00	0.01	down
0.00	0.01	up
0.00	0.02	down
0.01	0.02	down
0.02	0.05	up
0	0	up
0	0	down
0.00	0.00	down
0.00	0.00	down
0.00	0.00	down
0.00	0.00	down
0.00	0.00	down
0.00	0.00	down
0.00	0.00	down
0.00	0.00	up
0.00	0.00	down
0.00	0.00	down
0.00	0.00	down
0.00	0.00	down
0.00	0.00	down

0.00	0.00	up
0.00	0.01	down
0.01	0.02	down
0.01	0.02	down
0.01	0.03	down
0	0	up
0.00	0.00	up
0.00	0.00	down
0.00	0.00	down
0.00	0.00	up
0.01	0.04	up
0.00	0.00	down
0.00	0.00	down
0.00	0.00	up
0.00	0.00	down
0.00	0.00	down
0.00	0.00	up
0.00	0.01	up
0	0	down
0.00	0.00	down
0	0	down
0.00	0.00	up
0	0	down
0.00	0.00	down
0	0	down
0.00	0.00	down
0.00	0.00	down
0.00	0.00	down
0.00	0.00	down
0.00	0.00	down
0.00	0.00	down
0.00	0.00	down
0.00	0.00	up
0.00	0.00	down
0.00	0.00	down
0.00	0.00	down
0	0	up
0	0	down
0	0	down
0	0	down
0	0	up
0	0	down
0.00	0.00	down
0.00	0.00	down
0.00	0.00	down
0.00	0.00	down
0.00	0.00	down
0.00	0.00	down
0.00	0.00	down
0.00	0.00	down
0.00	0.00	down

0.00	0.00	down
0.00	0.00	down
0.00	0.00	up
0.00	0.00	down
0.00	0.00	down
0.00	0.00	up
0.00	0.00	down
0.00	0.00	down
0.00	0.00	down
0.00	0.00	down
0.00	0.00	down
0.00	0.00	down
0.00	0.00	down
0.00	0.00	down
0.00	0.00	down
0.00	0.00	down
0.00	0.00	down
0.00	0.00	down
0.00	0.00	down
0.00	0.00	down
0.00	0.00	down
0.00	0.00	down
0.00	0.00	up
0.00	0.00	down
0.00	0.00	down
0.00	0.00	up
0.00	0.00	up
0.00	0.00	up
0.00	0.00	up
0.00	0.00	down
0.00	0.00	down
0.00	0.00	down
0.00	0.00	down
0.00	0.00	up
0.00	0.00	up
0.00	0.00	up
0.00	0.00	up
0.00	0.00	up
0.00	0.00	up
0.00	0.00	down
0.00	0.00	down
0.00	0.00	down
0.00	0.00	down
0.00	0.00	down
0.00	0.00	down
0.00	0.00	down
0.00	0.00	down
0.00	0.00	down
0.00	0.00	down
0.00	0.00	down
0.00	0.00	down

0.00	0.00	up
0.00	0.00	down
0.00	0.01	down
0.00	0.01	down
0.00	0.01	down
0.00	0.02	up
0.01	0.02	up
0.01	0.04	up
0.01	0.04	up
0	0	down
0.00	0.00	up
0.00	0.00	down
0.00	0.00	down
0.00	0.00	down
0.00	0.00	down
0.00	0.00	down
0.00	0.00	down
0.00	0.00	down
0	0	down
0.00	0.00	down
0.00	0.00	up
0.00	0.00	down
0.00	0.00	up
0.00	0.00	up
0.00	0.00	down
0.00	0.00	up
0.00	0.00	up
0.00	0.01	up
0.00	0.02	down
0.01	0.03	down
0.01	0.04	down
0.01	0.04	down
0	0	down
0	0	down
0	0	down
0.00	0.00	down
0.00	0.00	up
0	0	down
0.00	0.00	down
0.00	0.00	up
0.00	0.00	down
0.00	0.00	up
0.00	0.00	down
0.00	0.00	down
0.00	0.00	up
0.00	0.00	down
0.00	0.01	up
0	0	down
0.00	0.00	down
0.00	0.00	down
0.00	0.00	down

0.00	0.00	down
0.00	0.00	down
0.00	0.00	down
0.00	0.00	down
0.00	0.00	up
0.00	0.00	down
0.00	0.00	down
0.02	0.05	up
0	0	down
0	0	down
0.00	0.00	up
0.00	0.00	up
0.00	0.00	up
0.00	0.00	down
0.00	0.00	up
0.00	0.00	up
0.00	0.00	up
0.00	0.00	down
0.00	0.01	down
0.00	0.01	down
0.01	0.04	up
0	0	down
0	0	down
0	0	down
0.00	0.00	down
0	0	down
0.00	0.00	down
0.00	0.00	down
0.00	0.00	down
0.00	0.00	down
0.00	0.00	down
0.00	0.00	down
0.00	0.00	down
0.00	0.01	up
0	0	down
0	0	down
0	0	down
0.00	0.00	down
0.00	0.00	down
0.00	0.00	down
0.00	0.00	down
0.00	0.00	up
0.00	0.00	up
0.00	0.00	down
0.00	0.00	down
0.00	0.00	down
0.00	0.00	down
0.00	0.00	down
0.00	0.00	down
0.00	0.00	down
0.00	0.00	down

0.00	0.00	down
0.00	0.00	down
0.00	0.00	down
0.00	0.00	down
0.00	0.00	down
0.00	0.00	down
0.00	0.00	up
0.00	0.00	up
0.00	0.00	down
0.00	0.00	up
0.00	0.00	up
0.00	0.00	down
0.00	0.00	up
0.00	0.01	up
0.00	0.01	down
0.00	0.01	down
0.00	0.00	down
0.00	0.00	down
0.00	0.00	down
0.00	0.00	up
0.00	0.01	down
0.01	0.02	up
0	0	down
0	0	down
0	0	down
0.00	0.02	down
0	0	down
0.00	0.00	down
0	0	down
0.00	0.00	down
0.00	0.00	down
0.00	0.00	down
0.00	0.00	down
0.00	0.00	down
0.00	0.00	down
0.00	0.00	down
0.00	0.01	up
0.00	0.02	up
0.00	0.01	up
0.00	0.01	up
0.01	0.02	up
0.01	0.02	down
0	0	down
0	0	up
0.00	0.00	down
0.00	0.00	down
0.00	0.00	down
0	0	down
0	0	down
0.00	0.00	up
0.00	0.00	down

0.00	0.00	up
0.00	0.00	down
0.00	0.00	up
0.00	0.00	up
0.00	0.00	up
0.00	0.00	down
0.00	0.00	up
0.00	0.00	down
0.00	0.00	down
0.00	0.00	down
0.00	0.00	down
0.00	0.00	up
0.00	0.00	down
0.00	0.00	up
0.00	0.00	down
0.00	0.00	down
0.00	0.00	down
0.00	0.00	down
0.00	0.00	down
0.00	0.00	up
0.00	0.00	down
0.00	0.00	down
0.00	0.00	down
0.00	0.00	down
0.00	0.01	down
0.01	0.05	down
0	0	up
0.00	0.00	up
0.00	0.00	down
0.00	0.00	down
0.00	0.01	down
0.00	0.01	down
0.00	0.00	down
0.00	0.00	down
0.00	0.00	down
0.00	0.00	down
0.00	0.00	down
0.00	0.00	down
0.00	0.00	down
0.01	0.04	up
0	0	down
0.00	0.00	down
0.00	0.00	down
0.00	0.00	down
0.00	0.00	down
0.00	0.00	down
0.00	0.00	up
0.00	0.00	down
0.00	0.00	down
0.00	0.00	down
0.00	0.00	up

0.00	0.00	up
0.00	0.00	up
0.00	0.00	down
0.00	0.00	down
0.00	0.00	down
0.00	0.00	down
0.00	0.00	down
0.00	0.00	down
0.00	0.00	down
0.00	0.00	down
0.00	0.00	up
0.00	0.00	up
0.00	0.00	up
0.00	0.00	up
0.00	0.00	up
0.00	0.00	up
0.00	0.00	up
0.00	0.00	up
0.01	0.04	up
0.01	0.04	down
0.00	0.00	down
0.00	0.00	down
0.00	0.00	down
0.00	0.00	up
0.00	0.00	up
0.02	0.05	up
0.00	0.00	down
0.00	0.01	down
0.00	0.01	down
0.00	0.01	down
0.01	0.02	down
0.01	0.03	down
0.00	0.00	up
0.00	0.00	up
0.00	0.00	down
0.00	0.00	down
0.00	0.00	down
0.00	0.00	up
0.00	0.00	down
0.00	0.00	down
0.00	0.00	down
0.00	0.00	down
0.00	0.00	down
0.00	0.00	down
0.00	0.00	down
0.00	0.00	up
0.01	0.02	down
0.00	0.00	up
0.00	0.00	down
0.00	0.00	down
0.00	0.00	up

0.00	0.00	down
0.00	0.00	down
0.00	0.00	up
0.00	0.00	down
0	0	down
0.00	0.00	down
0.00	0.00	down
0.00	0.00	down
0.00	0.00	down
0.00	0.00	down
0.00	0.00	down
0.00	0.00	down
0.00	0.00	up
0.00	0.00	down
0.00	0.00	up
0.00	0.00	down
0.00	0.00	down
0.00	0.00	up
0.00	0.00	down
0.01	0.02	up
0.00	0.00	down
0.00	0.00	down
0.00	0.00	up
0.00	0.00	down
0.00	0.00	up
0.00	0.00	up
0.00	0.00	down
0.00	0.00	down
0.00	0.00	down
0.00	0.00	up
0.00	0.00	down
0.00	0.00	up
0.00	0.00	up
0.00	0.01	down
0	0	up
0	0	down
0.00	0.00	down
0.00	0.00	down
0.00	0.00	up
0.00	0.00	down
0.00	0.00	down
0.00	0.00	up
0.00	0.00	down
0.00	0.00	down
0.00	0.00	down
0.00	0.00	down
0.00	0.00	down
0.00	0.00	down
0.00	0.00	down
0.00	0.00	down
0.00	0.00	down

0.00	0.00	down
0.00	0.00	down
0.00	0.00	down
0.00	0.00	down
0.00	0.00	down
0.00	0.00	down
0.00	0.00	down
0.00	0.00	down
0.00	0.00	up
0.00	0.00	down
0.00	0.00	up
0.00	0.00	down
0.00	0.00	down
0.00	0.00	up
0.00	0.00	down
0.00	0.00	down
0.00	0.00	up
0.00	0.00	down
0.00	0.00	down
0.00	0.00	down
0.00	0.00	down
0.00	0.00	down
0.00	0.00	up
0.00	0.00	up
0.00	0.00	up
0.00	0.00	down
0.00	0.00	down
0.00	0.00	up
0.00	0.00	down
0.00	0.00	down
0.00	0.00	down
0.00	0.00	down
0.00	0.00	up
0.00	0.00	up
0.00	0.00	up
0.00	0.00	down
0.00	0.00	down
0.00	0.00	down
0.00	0.00	down
0.00	0.00	down
0.00	0.00	down
0.00	0.00	up
0.00	0.00	down
0.00	0.00	down
0.00	0.00	up
0.00	0.00	up
0.00	0.00	down
0.00	0.00	down
0.00	0.00	down
0.00	0.00	down
0.00	0.00	up

0.00	0.00	up
0.00	0.00	up
0.00	0.01	down
0.00	0.01	down
0.00	0.01	down
0.00	0.01	down
0.00	0.01	up
0.01	0.02	up
0.00	0.00	down
0.00	0.00	up
0.00	0.00	down
0.00	0.00	down
0.00	0.00	up
0.00	0.00	down
0.00	0.00	down
0.00	0.00	up
0.00	0.00	down
0.00	0.00	up
0.00	0.00	down
0.00	0.00	down
0.00	0.00	down
0.00	0.00	down
0.00	0.00	up
0.00	0.00	down
0.00	0.00	down
0.00	0.00	down
0.02	0.05	up
0.00	0.00	down
0.00	0.00	down
0.00	0.01	down
0.00	0.00	down
0.00	0.00	up
0.00	0.00	down
0.00	0.00	down
0.00	0.00	down
0.00	0.00	down
0.00	0.00	down
0.00	0.00	up
0.00	0.00	down
0.00	0.00	down
0.00	0.01	up
0.01	0.04	down
0	0	down
0	0	down
0	0	down
0.00	0.00	up
0.00	0.00	down
0.00	0.00	up
0.00	0.00	down
0.00	0.00	down
0.00	0.00	down

0.00	0.00	down
0.00	0.00	down
0.00	0.00	down
0.00	0.00	down
0.00	0.00	down
0.00	0.00	down
0.00	0.00	down
0.00	0.00	up
0.00	0.00	up
0.00	0.00	up
0.00	0.00	down
0.00	0.00	down
0.00	0.01	down
0.00	0.00	down
0.00	0.01	down
0.00	0.00	down
0.00	0.00	down
0.00	0.00	down
0.00	0.00	down
0.00	0.00	down
0.00	0.00	down
0.00	0.00	down
0.00	0.00	down
0.00	0.00	down
0.00	0.00	down
0.00	0.00	down
0.00	0.00	down
0.00	0.00	down
0.01	0.05	up
0.00	0.00	down
0.00	0.00	up
0.00	0.00	down
0.00	0.00	down
0.00	0.00	down
0.00	0.00	down
0.00	0.00	down
0.00	0.00	down
0.00	0.00	down
0.00	0.00	down
0.00	0.00	down
0.00	0.00	down
0.00	0.00	up
0.00	0.00	down
0.00	0.00	up
0.00	0.00	up
0.00	0.00	up
0.00	0.00	down
0.00	0.00	down
0.00	0.00	up
0.00	0.00	up
0.00	0.00	down

0.00	0.00	up
0.00	0.00	down
0.00	0.00	up
0.00	0.00	down
0.00	0.00	down
0.00	0.01	up
0.01	0.04	down
0.02	0.05	up
0.00	0.00	down
0.00	0.00	up
0.00	0.00	down
0.00	0.00	down
0.00	0.00	up
0.00	0.00	up
0.00	0.00	down
0.00	0.00	down
0.00	0.00	up
0.00	0.00	up
0.00	0.00	down
0.00	0.00	up
0.00	0.00	down
0.00	0.00	up
0.00	0.01	up
0	0	down
0	0	down
0.00	0.00	down
0.00	0.00	down
0.00	0.00	down
0.00	0.00	down
0.00	0.00	down
0.00	0.00	up
0.00	0.00	up
0.00	0.01	down
0	0	down
0.01	0.02	down
0	0	up
0.01	0.03	down
0.00	0.00	up
0.00	0.00	up
0.00	0.00	up
0.00	0.00	down
0.00	0.00	down
0.00	0.00	down
0.00	0.00	down
0.00	0.00	down
0.00	0.00	down
0.00	0.00	down
0.00	0.00	down
0.01	0.03	down
0.00	0.00	up
0.00	0.00	up

0.00	0.00	up
0.00	0.00	up
0.01	0.04	up
0.01	0.04	down
0	0	down
0.00	0.00	down
0.00	0.00	down
0.00	0.00	down
0.00	0.00	down
0.00	0.01	down
0	0	down
0.00	0.00	down
0.00	0.00	down
0.00	0.00	down
0.00	0.00	down
0.00	0.00	down
0.00	0.00	down
0	0	down
0.00	0.00	up
0.00	0.00	down
0.00	0.00	down
0.00	0.00	down
0.00	0.01	down
0.02	0.05	up
0.00	0.00	down
0.00	0.00	down
0.00	0.00	down
0.00	0.00	down
0.00	0.00	up
0.00	0.00	up
0.00	0.00	down
0.00	0.00	down
0.00	0.00	up
0.00	0.00	down
0.00	0.00	up
0.00	0.00	down
0.00	0.00	down
0.00	0.00	down
0.00	0.00	down
0.00	0.01	down
0.00	0.00	up
0.00	0.00	down
0.00	0.00	down
0.00	0.00	up
0.00	0.00	up
0.00	0.00	up
0	0	down
0.00	0.00	down
0.00	0.00	down
0.00	0.00	down
0.00	0.00	down

0	0	down
0	0	down
0	0	down
0	0	up
0.00	0.00	up
0.00	0.00	up
0.00	0.00	up
0.00	0.00	up
0.00	0.00	down
0.00	0.00	up
0.00	0.00	up
0.00	0.00	up
0.00	0.00	up
0.00	0.00	down
0.00	0.00	down
0.00	0.00	down
0.00	0.00	down
0.00	0.00	down
0.00	0.00	down
0.00	0.00	down
0.00	0.00	down
0.00	0.00	down
0.00	0.00	down
0.00	0.00	up
0.00	0.00	down
0.00	0.00	down
0.00	0.00	down
0.00	0.00	down
0.00	0.00	down
0.00	0.00	up
0.00	0.00	down
0.00	0.00	down
0.00	0.00	down
0.00	0.00	down
0.00	0.00	up
0.00	0.00	down
0.00	0.00	up
0.00	0.00	down
0.00	0.00	up
0.00	0.00	down
0.00	0.00	down
0.00	0.00	up
0.00	0.00	down
0.00	0.00	down
0.00	0.00	down
0.00	0.00	down
0.00	0.00	up
0.00	0.00	up
0.00	0.00	down
0.00	0.00	down
0.00	0.00	down

0.00	0.00	up
0.00	0.00	down
0.00	0.00	up
0.00	0.01	down
0.00	0.01	down
0.01	0.03	up
0.00	0.00	down
0.00	0.00	down
0.00	0.00	up
0.00	0.00	down
0.00	0.00	down
0.00	0.00	down
0.00	0.00	down
0.00	0.01	down
0.01	0.04	down
0	0	down
0	0	down
0.00	0.00	down
0.00	0.00	down
0.00	0.00	down
0.00	0.00	down
0.00	0.00	down
0.00	0.00	up
0.00	0.00	up
0.00	0.00	down
0.00	0.00	down
0.01	0.02	down
0	0	down
0	0	down
0.00	0.00	down
0.00	0.00	down
0.00	0.00	down
0.00	0.00	up
0.00	0.01	down
0.01	0.02	down
0.01	0.02	down
0	0	up
0.00	0.00	down
0.00	0.00	down
0.01	0.05	down
0.00	0.00	down
0.00	0.00	down
0.00	0.00	down
0.00	0.00	up
0	0	down
0.00	0.00	up
0.00	0.00	down
0.00	0.00	down
0.00	0.00	down
0.00	0.00	up
0.00	0.00	down

0.00	0.00	up
0.00	0.00	up
0.00	0.00	up
0.00	0.00	up
0.00	0.00	up
0.00	0.00	down
0.00	0.00	down
0.00	0.00	up
0.00	0.00	down
0.00	0.00	down
0.00	0.00	down
0.00	0.00	down
0.00	0.00	up
0.00	0.00	down
0.00	0.00	up
0.00	0.00	down
0.01	0.02	down
0.00	0.00	up
0.00	0.00	up
0.00	0.00	down
0.00	0.00	down
0.00	0.00	down
0.00	0.00	down
0.00	0.01	down
0.00	0.00	down
0.00	0.00	down
0.00	0.00	up
0.00	0.00	up
0.00	0.00	down
0.00	0.00	down
0.00	0.00	up
0.00	0.00	down
0.00	0.00	down
0.00	0.00	up
0.00	0.00	up
0.00	0.00	up
0.00	0.00	down
0.00	0.00	down
0.00	0.00	down
0.00	0.00	down
0.01	0.04	down
0.01	0.05	up
0.00	0.00	up
0.00	0.00	down
0.00	0.00	up
0.00	0.01	down
0.01	0.02	up
0.00	0.00	down
0.00	0.00	down
0.00	0.00	down
0.00	0.00	down

0.00	0.00	down
0.00	0.00	down
0.00	0.00	down
0.00	0.00	up
0.00	0.00	up
0.00	0.00	down
0.00	0.00	down
0.00	0.00	up
0.00	0.00	down
0.00	0.00	up
0.00	0.00	up
0.00	0.00	up
0.00	0.00	up
0	0	down
0.00	0.00	down
0.00	0.00	down
0.00	0.00	up
0.00	0.00	down
0.00	0.00	down
0.00	0.00	up
0.00	0.00	up
0.00	0.00	up
0.00	0.00	up
0.00	0.00	up
0.01	0.02	up
0.00	0.00	down
0.00	0.00	down
0.00	0.00	down
0.00	0.00	up
0.00	0.00	down
0.00	0.00	down
0.00	0.00	up
0.00	0.00	down
0.00	0.00	down
0.00	0.00	up
0.00	0.01	up
0.00	0.01	down
0.00	0.01	up
0.00	0.02	down
0.01	0.03	up
0.01	0.03	up
0.01	0.03	down
0	0	down
0	0	down
0	0	up
0	0	up
0.00	0.00	down
0.00	0.00	down
0.00	0.00	down
0.00	0.00	up
0.00	0.00	up

0.00	0.00	up
0.00	0.00	up
0.00	0.00	down
0.00	0.00	down
0.00	0.00	down
0.00	0.00	down
0.00	0.00	down
0.00	0.00	down
0.00	0.00	down
0.00	0.00	down
0.00	0.00	down
0.00	0.00	down
0.00	0.00	up
0.00	0.00	down
0.00	0.00	up
0.00	0.00	up
0.00	0.00	down
0.00	0.00	down
0.00	0.00	up
0.00	0.00	down
0.00	0.00	down
0.00	0.00	down
0.00	0.00	down
0.00	0.00	down
0.00	0.00	up
0.00	0.00	down
0.00	0.00	down
0.00	0.00	up
0.00	0.00	down
0.00	0.00	up
0.00	0.00	up
0.00	0.00	up
0.00	0.00	up
0.00	0.00	up
0.00	0.00	down
0.00	0.00	up
0.00	0.00	up
0.00	0.00	up
0.00	0.00	up
0.00	0.00	up
0.00	0.00	down
0.00	0.00	down
0.00	0.00	down
0.00	0.00	up
0.00	0.00	down
0.00	0.00	up
0.00	0.00	down
0.00	0.00	down
0.00	0.00	down
0.00	0.00	up
0.00	0.00	down

0.01	0.03	up
0.00	0.00	down
0.00	0.00	up
0.00	0.00	down
0.00	0.00	down
0.00	0.00	down
0.00	0.00	down
0.00	0.00	down
0.00	0.00	down
0.00	0.00	up
0.00	0.00	down
0.00	0.00	down
0.00	0.00	down
0.00	0.00	down
0.00	0.00	down
0.00	0.00	down
0.00	0.00	down
0.00	0.00	down
0.00	0.00	down
0.01	0.04	down
0.01	0.05	up
0.00	0.00	up
0.00	0.00	down
0.00	0.00	up
0.00	0.00	down
0.00	0.00	down
0.00	0.00	down
0.00	0.00	down
0.00	0.00	up
0.00	0.00	up
0.00	0.00	down
0.00	0.00	up
0.00	0.01	down
0.01	0.03	down
0.00	0.00	up
0.00	0.00	down
0.00	0.00	down
0	0	down
0	0	down
0	0	down
0.00	0.00	down
0.00	0.00	down
0.00	0.00	down
0.00	0.00	up
0.00	0.00	up
0.00	0.00	up
0.00	0.00	down
0.00	0.00	up
0.00	0.00	up
0.00	0.00	down
0.00	0.00	up

0.00	0.00	up
0.00	0.00	down
0.00	0.00	up
0.00	0.00	up
0.00	0.00	down
0.00	0.00	up
0.00	0.00	up
0.00	0.00	down
0.00	0.00	down
0.00	0.00	down
0.00	0.00	down
0.01	0.02	up
0.01	0.02	down
0.01	0.03	up
0.01	0.04	down
0.02	0.05	down
0.00	0.00	down
0.00	0.00	down
0.00	0.00	down
0.00	0.00	down
0.00	0.00	down
0.00	0.01	down
0.00	0.00	up
0.00	0.01	down
0	0	down
0.00	0.00	down
0.00	0.00	down
0.00	0.00	down
0.00	0.00	up
0.00	0.00	up
0.00	0.00	down
0.00	0.00	down
0.00	0.00	down
0.00	0.00	up
0.00	0.00	down
0.00	0.00	down
0.00	0.00	down
0.00	0.00	up
0.00	0.00	down
0.00	0.00	down
0.00	0.00	down
0.00	0.00	up
0.00	0.00	up
0.00	0.01	up
0.00	0.01	up
0.00	0.01	up
0.01	0.02	down
0.00	0.00	down
0.00	0.00	down
0.00	0.00	down
0.01	0.04	up

0.00	0.00	down
0.00	0.00	up
0.00	0.00	up
0.00	0.00	up
0	0	down
0.00	0.00	up
0.00	0.00	down
0.00	0.00	down
0.00	0.00	down
0.00	0.00	down
0.00	0.00	down
0.00	0.00	down
0.00	0.01	down
0.00	0.00	down
0.00	0.01	down
0.00	0.00	down
0.00	0.00	down
0.00	0.00	down
0.00	0.00	up
0.00	0.00	up
0.00	0.00	up
0.00	0.00	down
0.00	0.00	down
0.00	0.00	up
0.00	0.00	down
0.00	0.00	down
0.00	0.00	down
0.00	0.00	down
0.00	0.00	up
0.00	0.00	down
0.00	0.00	down
0.00	0.00	down
0.00	0.00	down
0.00	0.00	up
0.00	0.00	up
0.00	0.00	up
0.01	0.03	up
0.00	0.00	down
0.00	0.00	up
0.00	0.01	down
0.00	0.00	down
0.00	0.00	up
0.00	0.01	down
0.00	0.00	down
0.00	0.01	down
0.01	0.02	up
0.00	0.00	down
0.00	0.00	up
0.00	0.00	up
0.00	0.00	down
0.00	0.00	down

0.00	0.00	up
0.00	0.00	down
0.00	0.00	up
0.00	0.00	up
0.00	0.00	up
0.00	0.00	up
0.00	0.00	up
0	0	down
0	0	down
0	0	up
0.00	0.00	up
0.00	0.00	down
0.00	0.00	down
0.00	0.00	up
0.00	0.00	down
0.00	0.00	up
0.00	0.00	down
0.00	0.00	down
0.00	0.00	up
0.00	0.00	down
0.00	0.00	down
0.00	0.00	down
0.00	0.00	down
0.00	0.00	down
0.00	0.00	down
0.00	0.00	down
0.00	0.00	down
0.00	0.00	up
0.00	0.00	up
0.00	0.00	up
0.00	0.00	down
0.00	0.00	down
0.00	0.00	up
0.00	0.00	down
0.00	0.00	down
0.00	0.01	down
0.01	0.03	up
0.01	0.03	down
0.01	0.03	down
0.01	0.04	down
0.00	0.00	down
0.00	0.00	down
0.00	0.00	up
0.00	0.00	up
0.00	0.00	up
0.00	0.00	up
0.00	0.00	up
0.00	0.00	up
0.00	0.00	up
0.00	0.00	up
0.00	0.00	up

0.00	0.00	up
0.00	0.00	up
0.00	0.00	up
0.00	0.00	down
0.00	0.00	down
0.00	0.00	down
0.00	0.00	up
0.01	0.03	down
0	0	down
0.00	0.00	down
0	0	up
0.00	0.00	down
0.00	0.00	down
0.00	0.00	down
0.00	0.00	down
0.00	0.00	down
0.00	0.00	down
0.00	0.00	down
0.00	0.00	down
0.00	0.00	down
0.00	0.00	down
0.00	0.00	down
0	0	down
0.00	0.00	up
0.00	0.00	up
0.00	0.00	down
0.00	0.00	down
0.00	0.01	down
0	0	down
0	0	up
0.00	0.00	up
0.00	0.00	down
0.00	0.00	up
0.00	0.00	up
0	0	up
0	0	up
0.00	0.00	up
0.00	0.00	down
0.00	0.00	down
0.00	0.00	down
0.00	0.00	up
0.00	0.00	down
0.00	0.00	down
0.00	0.00	down
0.00	0.00	down
0.00	0.00	up
0.00	0.00	down
0.00	0.00	down
0.00	0.00	up
0.00	0.00	down
0.00	0.00	down

0.00	0.00	up
0.00	0.00	down
0.00	0.00	down
0.00	0.00	down
0.00	0.00	down
0.00	0.00	down
0.00	0.01	up
0.01	0.02	up
0.01	0.02	up
0.01	0.02	down
0.00	0.00	down
0.00	0.00	down
0.00	0.00	up
0.00	0.00	up
0.00	0.00	down
0.00	0.00	up
0.00	0.00	down
0.00	0.00	down
0.00	0.00	down
0.00	0.00	down
0.00	0.00	down
0.00	0.00	up
0.00	0.00	down
0.00	0.00	down
0.00	0.00	down
0.00	0.00	down
0.00	0.00	up
0.00	0.00	down
0.00	0.00	down
0.00	0.01	up
0.01	0.04	down
0	0	up
0.00	0.00	up
0.00	0.00	up
0.00	0.00	up
0.00	0.00	up
0.00	0.00	up
0.00	0.00	up
0.02	0.05	up
0.00	0.00	up
0.00	0.00	down
0.00	0.00	up
0.00	0.00	up
0.00	0.00	down
0.00	0.00	down
0.00	0.01	up
0	0	down
0.00	0.00	down
0.00	0.02	down
0	0	down
0.00	0.00	down

0.00	0.02	down
0.00	0.00	down
0.00	0.00	up
0.00	0.00	down
0.00	0.00	down
0.00	0.00	down
0.02	0.05	down
0.00	0.00	up
0.00	0.00	up
0.00	0.00	down
0.00	0.00	down
0	0	down
0	0	down
0.00	0.00	up
0.00	0.00	down
0.00	0.00	down
0.00	0.00	up
0.00	0.00	down
0.00	0.00	down
0.00	0.00	up
0.00	0.00	down
0.00	0.00	up
0.00	0.00	down
0.00	0.00	down
0.00	0.00	up
0.00	0.00	up
0.00	0.00	up
0.00	0.00	down
0.00	0.00	up
0.00	0.00	up
0.00	0.00	up
0.00	0.01	up
0.01	0.04	down
0.00	0.00	down
0.00	0.00	up
0.00	0.00	down
0.00	0.00	down
0.00	0.00	down
0.00	0.00	down
0.00	0.00	up
0.00	0.00	down
0.00	0.00	down
0.00	0.00	down
0.00	0.00	down
0.00	0.01	down
0.00	0.00	down
0.00	0.00	down
0.00	0.00	down
0.00	0.00	up
0.00	0.00	down
0.01	0.03	down

0.00	0.00	up
0.00	0.00	down
0.00	0.00	down
0.00	0.00	down
0.00	0.00	down
0.00	0.00	up
0.00	0.00	up
0.00	0.00	up
0.00	0.00	up
0.00	0.00	up
0.00	0.00	up
0.00	0.00	up
0.00	0.00	up
0.01	0.04	up
0.01	0.04	down
0.00	0.00	down
0.00	0.00	down
0.00	0.00	down
0.00	0.00	down
0.01	0.05	down
0.00	0.00	down
0.00	0.00	up
0.01	0.02	down
0.01	0.02	up
0	0	down
0.00	0.00	up
0.00	0.00	down
0.00	0.00	down
0.00	0.00	up
0.00	0.00	up
0.00	0.00	down
0.00	0.00	down
0.00	0.00	down
0.00	0.00	up
0.00	0.00	down
0.00	0.00	down
0.00	0.00	up
0.00	0.00	up
0.00	0.00	down
0.00	0.00	down
0.00	0.00	down
0.00	0.01	down
0.01	0.03	down
0	0	down
0.00	0.00	down
0.00	0.00	up
0.00	0.00	up
0.00	0.00	up
0.01	0.04	down
0.00	0.00	down
0.00	0.00	up

0.00	0.00	up
0.00	0.00	up
0.00	0.00	up
0.00	0.00	up
0.00	0.00	down
0.00	0.01	down
0	0	down
0	0	up
0.00	0.00	down
0.00	0.00	down
0.00	0.00	down
0.00	0.00	down
0.00	0.00	down
0.00	0.00	down
0.00	0.00	down
0.00	0.00	down
0.00	0.00	down
0.00	0.00	down
0.00	0.00	down
0.00	0.00	down
0.00	0.00	down
0.00	0.00	up
0.00	0.00	down
0.00	0.00	down
0.00	0.00	down
0.00	0.00	down
0.00	0.00	up
0.00	0.00	down
0.00	0.00	down
0.00	0.00	down
0.00	0.00	down
0.00	0.00	down
0.00	0.00	down
0.00	0.00	down
0.00	0.00	up
0.00	0.00	down
0.00	0.01	down
0.01	0.02	down
0.01	0.02	down
0.01	0.03	down
0.01	0.03	down
0.00	0.00	down
0.00	0.00	down
0.00	0.00	down
0.00	0.00	down
0.00	0.00	down
0.00	0.00	down
0.00	0.00	down
0.00	0.01	up
0.00	0.00	down
0.00	0.00	up

0.00	0.00	up
0.00	0.00	down
0.00	0.00	up
0.00	0.00	down
0.00	0.00	down
0.00	0.00	down
0.00	0.00	down
0.00	0.00	up
0.00	0.00	down
0.00	0.00	up
0.00	0.00	down
0.00	0.01	up
0.01	0.04	down
0.00	0.00	down
0.00	0.00	up
0.00	0.00	down
0.00	0.00	up
0.01	0.04	up
0	0	down
0	0	up
0.00	0.00	up
0.00	0.00	up
0.00	0.00	down
0.00	0.00	down
0.00	0.00	down
0.00	0.00	up
0.00	0.00	down
0.00	0.00	down
0.00	0.00	down
0.00	0.00	down
0.00	0.00	down
0.00	0.00	down
0.00	0.00	up
0.00	0.00	down
0.00	0.00	up
0.00	0.00	down
0.00	0.00	up
0.00	0.00	up
0.00	0.00	down
0.00	0.00	down
0.00	0.00	down
0.00	0.00	down
0.00	0.00	up
0.00	0.00	down
0.00	0.00	up
0.00	0.00	up
0.00	0.00	down
0.00	0.00	up
0.00	0.00	down
0.00	0.00	down
0.00	0.00	down

0.00	0.00	down
0.00	0.00	down
0.00	0.00	down
0.00	0.00	down
0.00	0.00	down
0.00	0.00	down
0.00	0.00	down
0.00	0.00	down
0.00	0.00	up
0.00	0.00	down
0.00	0.00	up
0.00	0.00	down
0.00	0.00	up
0.00	0.00	up
0.00	0.00	up
0.00	0.00	down
0.00	0.00	down
0.00	0.00	up
0.00	0.00	down
0.00	0.00	up
0.00	0.00	up
0.00	0.00	down
0.00	0.00	down
0.00	0.00	down
0.00	0.00	up
0.00	0.00	down
0.00	0.00	down
0.00	0.00	down
0.00	0.00	up
0.00	0.00	down
0.00	0.00	up
0.00	0.00	up
0.00	0.00	down
0.00	0.00	down
0.00	0.00	down
0.00	0.00	down
0.00	0.00	down
0.00	0.00	down
0.00	0.00	up
0.00	0.00	up
0.00	0.00	up
0.00	0.00	up
0.00	0.00	up
0.00	0.00	up
0.00	0.00	up
0.00	0.00	down
0.00	0.00	down
0.00	0.00	down
0.00	0.00	down
0.00	0.00	down
0.00	0.00	down

0.00	0.00	down
0.00	0.00	down
0.00	0.00	down
0.00	0.00	down
0.00	0.00	down
0.00	0.00	up
0.00	0.00	down
0.00	0.00	up
0.00	0.00	down
0.00	0.00	down
0.00	0.00	up
0.00	0.00	down
0.00	0.00	up
0.00	0.00	up
0.00	0.01	down
0.00	0.01	down
0.00	0.01	down
0.00	0.01	down
0.00	0.01	down
0.00	0.01	down
0.00	0.01	down
0.00	0.01	down
0.00	0.01	up
0.00	0.01	up
0.00	0.01	down
0.00	0.02	down
0.01	0.02	up
0.01	0.02	down
0.01	0.02	up
0.01	0.02	down
0.01	0.03	down
0.01	0.03	down
0.01	0.03	down
0.01	0.03	down
0.01	0.03	down
0.01	0.04	down
0.00	0.00	down
0.00	0.00	down
0.00	0.01	up
0.00	0.00	up
0.00	0.00	down
0.00	0.00	up
0	0	down
0	0	down
0.00	0.00	down
0.00	0.00	up
0.00	0.00	down
0.00	0.00	up
0.00	0.00	down
0.00	0.00	down
0.00	0.00	up

0.00	0.00	down
0.00	0.00	down
0.00	0.00	down
0.00	0.00	down
0.00	0.00	down
0.00	0.00	down
0.00	0.01	down
0.00	0.02	down
0.01	0.02	down
0.01	0.02	down
0.01	0.04	up
0.01	0.04	up
0	0	down
0.01	0.02	down
0.00	0.00	down
0.00	0.00	down
0.00	0.00	down
0.00	0.00	up
0.00	0.00	down
0.00	0.00	down
0.00	0.00	down
0.00	0.00	down
0.00	0.00	up
0.00	0.00	down
0.00	0.00	down
0.00	0.01	up
0.00	0.00	down
0.00	0.00	down
0.00	0.00	down
0.00	0.00	down
0.00	0.00	down
0.00	0.00	down
0.00	0.00	up
0.01	0.03	down
0.00	0.00	down
0.00	0.00	up
0.00	0.00	down
0.00	0.00	up
0.01	0.04	up
0.00	0.00	down
0.00	0.00	down
0.00	0.00	up
0.00	0.00	down
0.00	0.00	up
0.00	0.00	up
0.00	0.00	up
0.00	0.00	down
0.00	0.00	down
0.00	0.00	up
0.00	0.00	down
0.00	0.00	up

0.00	0.00	up
0.00	0.00	down
0.00	0.00	down
0.00	0.00	down
0.00	0.00	down
0.00	0.00	down
0.00	0.00	down
0.00	0.00	up
0.00	0.00	down
0.00	0.00	up
0.00	0.00	up
0.01	0.02	down
0.01	0.02	down
0.00	0.00	up
0.00	0.00	down
0.00	0.00	up
0.00	0.00	down
0.00	0.01	down
0.00	0.00	down
0.00	0.00	down
0.00	0.00	down
0.00	0.00	down
0	0	down
0.00	0.00	down
0.00	0.00	up
0.00	0.00	down
0.00	0.00	up
0.00	0.00	down
0.00	0.00	down
0.00	0.00	up
0.00	0.00	up
0.00	0.00	up
0.00	0.00	up
0.00	0.00	up
0.00	0.00	down
0.00	0.00	down
0.01	0.02	up
0.00	0.00	down
0.00	0.00	up
0.00	0.00	down
0.00	0.00	down
0.00	0.00	up
0.00	0.00	up
0.00	0.00	up
0.00	0.01	down
0.00	0.00	down
0.01	0.03	down
0	0	down
0	0	up
0.00	0.00	up
0.00	0.00	down

0.00	0.00	down
0.00	0.00	down
0.00	0.00	down
0.00	0.00	down
0.00	0.00	up
0.00	0.00	down
0.00	0.00	down
0.00	0.00	down
0.01	0.04	down
0.00	0.00	up
0.00	0.00	up
0.00	0.00	up
0.00	0.01	down
0	0	up
0	0	up
0	0	down
0	0	down
0.00	0.00	up
0.00	0.00	up
0.00	0.00	up
0.00	0.00	down
0.00	0.00	down
0.00	0.00	up
0.00	0.00	up
0.00	0.00	up
0.00	0.00	up
0.00	0.00	up
0.00	0.00	down
0.00	0.00	down
0.00	0.00	up
0.00	0.00	down
0.00	0.01	down
0.00	0.01	up
0.00	0.00	down
0.00	0.00	down
0.00	0.00	up
0.00	0.00	down
0.00	0.00	down
0.00	0.00	down
0.00	0.00	down
0.00	0.00	down
0.00	0.00	down
0.00	0.01	up
0.00	0.00	down
0.01	0.02	down
0	0	up
0.00	0.00	down
0.00	0.00	up
0.00	0.00	down
0.00	0.00	down
0.00	0.00	up

0.00	0.00	down
0.00	0.00	up
0.00	0.00	down
0.01	0.03	up
0.00	0.00	down
0.00	0.00	up
0.00	0.00	down
0.00	0.00	down
0.01	0.02	up
0.00	0.00	up
0.00	0.00	down
0.00	0.00	up
0.00	0.00	up
0.00	0.00	up
0.00	0.00	down
0.01	0.04	down
0.00	0.00	down
0.01	0.03	down
0.00	0.00	up
0.00	0.00	up
0.00	0.00	up
0.01	0.02	down
0.01	0.02	down
0.01	0.02	down
0.00	0.00	down
0.00	0.00	up
0.00	0.00	down
0.00	0.00	down
0.00	0.00	up
0.00	0.00	down
0.00	0.00	down
0.00	0.00	down
0.00	0.00	down
0	0	down
0.00	0.00	up
0.00	0.00	down
0.00	0.00	down
0.00	0.00	down
0.00	0.01	down
0.00	0.00	down
0.00	0.00	down
0.00	0.00	up
0.00	0.00	down
0.00	0.00	down
0.00	0.00	down
0.00	0.00	down
0.00	0.00	up
0.00	0.00	up
0.00	0.00	down
0.00	0.00	up
0.01	0.04	down

0.00	0.00	up
0.00	0.00	up
0.00	0.00	down
0.00	0.00	down
0.00	0.00	up
0.00	0.01	down
0.00	0.01	down
0.00	0.00	up
0.00	0.00	down
0.00	0.00	down
0.00	0.00	down
0.00	0.00	down
0.00	0.00	down
0.00	0.00	down
0.00	0.00	down
0.00	0.00	down
0.00	0.00	down
0.00	0.01	down
0	0	down
0.00	0.00	down
0	0	down
0.00	0.00	down
0.00	0.00	up
0.00	0.00	down
0.00	0.00	up
0.00	0.00	down
0.00	0.01	up
0.01	0.04	up
0.00	0.00	down
0.00	0.01	down
0.00	0.00	down
0.00	0.01	up
0	0	up
0.00	0.00	up
0.00	0.00	up
0.00	0.00	down
0.00	0.00	up
0.00	0.00	down
0.00	0.00	down
0.00	0.00	up
0.00	0.00	up
0.01	0.04	down
0.00	0.00	up
0.00	0.00	down
0.00	0.00	down
0.00	0.00	down
0.00	0.00	down
0.00	0.00	down
0.00	0.00	down
0.01	0.03	down
0.00	0.00	down

0.00	0.00	down
0.00	0.00	down
0.00	0.00	down
0.00	0.00	down
0.00	0.01	down
0.01	0.05	up
0.00	0.00	down
0.00	0.00	up
0.00	0.00	down
0.00	0.00	up
0	0	up
0.00	0.00	up
0.00	0.00	down
0.00	0.00	up
0.00	0.00	up
0.00	0.01	up
0.00	0.00	down
0.00	0.00	down
0.00	0.00	up
0.00	0.00	down
0.00	0.00	up
0.00	0.00	down
0.00	0.00	down
0.00	0.00	down
0.00	0.01	down
0.00	0.00	up
0.00	0.00	down
0.00	0.00	down
0.00	0.00	up
0.00	0.00	down
0.00	0.00	down
0.00	0.00	down
0.00	0.00	down
0.00	0.00	down
0.00	0.00	up
0.00	0.00	down
0.00	0.00	up
0.00	0.00	down
0.00	0.00	down
0.00	0.00	down
0.00	0.00	down
0.02	0.05	up
0.00	0.00	down
0.00	0.00	down
0.00	0.00	down
0.00	0.00	up
0.00	0.00	up
0.00	0.00	up
0.00	0.00	down
0.00	0.00	down
0.00	0.01	down

0.00	0.00	up
0.00	0.00	down
0.01	0.02	down
0.00	0.00	up
0.00	0.00	down
0.00	0.00	down
0.00	0.00	up
0.00	0.00	up
0.00	0.00	down
0.00	0.00	down
0.01	0.04	down
0.01	0.04	up
0.00	0.00	down
0.00	0.00	down
0.00	0.00	up
0.00	0.00	up
0.00	0.00	down
0.00	0.00	down
0.00	0.00	down
0	0	up
0.00	0.00	down
0.00	0.00	up
0.00	0.00	up
0.00	0.00	up
0.00	0.00	up
0.00	0.00	up
0.00	0.00	down
0.00	0.01	down
0.00	0.01	down
0.02	0.05	down
0.01	0.04	down
0.00	0.00	down
0.00	0.00	down
0.00	0.00	down
0.01	0.04	down
0.00	0.00	down
0.01	0.03	down
0.00	0.01	down
0.01	0.03	down
0.00	0.00	down
0.00	0.00	down
0.00	0.00	down
0.00	0.00	down
0.00	0.00	down
0.00	0.00	up
0.00	0.00	down
0.01	0.02	down
0.00	0.00	down
0.00	0.00	down
0.00	0.00	up
0.00	0.00	down

0.00	0.00	down
0.00	0.00	up
0.00	0.00	down
0.00	0.00	up
0.00	0.00	down
0.00	0.01	up
0.01	0.03	down
0.00	0.00	down
0.00	0.00	down
0.00	0.00	up
0.00	0.00	down
0.00	0.00	down
0.00	0.00	up
0.01	0.03	down
0.00	0.00	down
0.00	0.00	down
0.00	0.00	down
0.00	0.00	down
0.00	0.01	up
0	0	up
0.00	0.00	up
0.00	0.00	down
0.00	0.00	up
0.00	0.00	up
0.00	0.00	up
0.00	0.00	up
0.00	0.00	down
0.00	0.00	down
0.00	0.00	up
0.00	0.00	down
0.00	0.00	up
0.00	0.00	up
0.00	0.00	down
0.00	0.00	down
0.00	0.00	down
0.00	0.00	down
0.00	0.00	up
0.00	0.00	up
0.00	0.00	up
0.00	0.00	down
0.00	0.00	down
0.01	0.04	down
0.01	0.04	down
0.00	0.00	up
0.00	0.00	down
0.00	0.00	up
0.00	0.00	up
0.00	0.00	down
0.00	0.00	down
0.01	0.05	down
0.00	0.00	down

0.00	0.00	up
0.00	0.00	down
0.00	0.00	down
0.00	0.00	down
0.00	0.00	down
0.00	0.00	down
0.00	0.00	down
0.00	0.00	up
0.00	0.00	up
0.00	0.00	down
0	0	up
0.00	0.00	down
0.00	0.00	up
0.00	0.00	up
0.00	0.01	up
0.01	0.02	down
0	0	down
0.00	0.00	down
0.00	0.00	down
0.00	0.00	down
0.00	0.00	down
0	0	up
0.00	0.00	up
0	0	up
0.00	0.00	up
0.00	0.00	down
0.00	0.00	down
0.00	0.00	down
0.00	0.00	up
0.00	0.00	down
0.00	0.00	up
0.00	0.00	down
0.00	0.00	up
0.00	0.00	down
0.00	0.00	down
0.00	0.00	down
0.00	0.00	down
0.00	0.00	down
0.00	0.01	up
0.00	0.01	up
0.00	0.01	up
0.00	0.02	down
0.01	0.03	up
0.01	0.03	up
0.01	0.04	up
0.01	0.04	down
0.00	0.00	up
0.00	0.00	up
0.00	0.00	down
0.00	0.00	down
0.00	0.01	down

0.01	0.02	down
0.00	0.00	up
0.00	0.00	down
0.00	0.00	up
0.01	0.02	down
0.00	0.00	down
0.01	0.03	down
0	0	down
0	0	down
0	0	down
0	0	down
0	0	down
0.00	0.00	down
0.00	0.00	down
0.00	0.00	down
0.00	0.00	down
0.00	0.00	down
0.00	0.00	down
0.00	0.00	down
0.00	0.00	down
0.00	0.00	down
0.00	0.00	down
0.00	0.00	down
0.00	0.00	down
0.00	0.00	up
0.00	0.00	down
0.00	0.00	down
0.00	0.00	up
0.00	0.00	up
0.00	0.00	up
0.00	0.00	down
0.00	0.00	down
0.00	0.00	down
0.00	0.00	down
0.00	0.00	down
0.00	0.00	down
0.00	0.00	down
0.00	0.00	up
0.00	0.00	down
0.00	0.00	up
0.00	0.00	down
0.00	0.00	up
0.00	0.00	up
0.00	0.00	down
0.00	0.00	up
0.00	0.00	down
0.00	0.00	down
0.00	0.00	up
0.00	0.01	down
0.00	0.01	up
0.00	0.01	down

0.00	0.01	down
0.01	0.02	down
0.01	0.03	down
0.01	0.03	down
0.01	0.03	down
0.01	0.03	down
0.00	0.00	up
0.00	0.00	up
0.00	0.00	up
0.00	0.00	up
0.00	0.00	down
0.00	0.00	down
0.00	0.00	down
0.00	0.00	up
0.00	0.00	down
0.00	0.00	down
0.00	0.00	down
0.00	0.00	down
0.01	0.03	up
0.01	0.04	down
0.00	0.00	down
0.00	0.01	up
0.00	0.00	up
0.00	0.00	down
0.00	0.00	down
0.00	0.00	down
0.00	0.00	up
0.00	0.00	down
0	0	up
0.00	0.00	down
0.00	0.00	up
0.00	0.00	up
0.00	0.00	down
0.00	0.00	up
0.00	0.00	down
0.00	0.00	up
0.00	0.00	up
0.00	0.00	down
0.00	0.00	up
0.00	0.02	down
0.01	0.02	up
0.00	0.00	up
0.00	0.00	down
0.00	0.00	up
0.00	0.00	up
0.00	0.00	up
0.00	0.00	up
0.00	0.00	down
0.00	0.00	down
0.01	0.03	down
0.01	0.03	down

0.00	0.00	up
0.00	0.00	up
0.00	0.00	down
0.00	0.00	up
0.00	0.00	down
0	0	down
0.00	0.00	down
0.00	0.00	down
0.00	0.00	down
0.00	0.00	down
0.00	0.00	down
0.00	0.00	up
0.01	0.02	up
0.01	0.02	down
0.00	0.00	up
0.00	0.00	up
0	0	down
0.00	0.00	down
0.00	0.00	down
0.00	0.00	down
0.00	0.00	down
0.00	0.00	up
0.00	0.00	down
0.00	0.00	down
0.00	0.00	down
0.00	0.00	down
0.00	0.00	down
0.00	0.00	up
0.00	0.00	down
0.00	0.00	down
0.00	0.00	down
0.00	0.00	down
0.00	0.00	down
0.00	0.00	up
0.00	0.00	up
0.00	0.00	up
0.00	0.01	up
0.00	0.02	down
0.01	0.02	up
0.00	0.00	down
0.00	0.00	down
0.00	0.00	down
0.00	0.00	up
0.00	0.00	down
0.01	0.03	down
0.00	0.00	down
0.00	0.00	up
0.00	0.00	down
0.00	0.00	up
0.00	0.00	up
0.00	0.00	down

0.00	0.00	up
0.00	0.00	up
0.00	0.00	up
0.00	0.00	down
0.00	0.00	down
0.00	0.00	down
0.00	0.01	down
0.01	0.02	down
0.00	0.00	down
0.00	0.01	down
0.00	0.00	down
0.00	0.00	up
0.00	0.00	up
0.00	0.00	down
0.00	0.00	up
0.00	0.00	up
0.00	0.00	down
0.00	0.00	down
0.00	0.00	down
0.00	0.00	down
0.00	0.00	down
0.00	0.00	down
0.00	0.00	down
0.00	0.01	down
0.00	0.00	down
0.00	0.00	down
0.00	0.00	up
0.00	0.00	up
0.00	0.00	down
0.00	0.00	down
0.00	0.01	down
0	0	down
0.00	0.00	down
0.00	0.00	down
0.00	0.00	down
0.00	0.00	up
0.00	0.00	down
0.00	0.00	down
0.00	0.00	down
0.00	0.00	down
0.01	0.04	down
0.00	0.00	up
0.00	0.00	down
0.01	0.02	down
0.00	0.00	down
0.01	0.03	down
0.00	0.00	down
0.00	0.00	up
0.00	0.00	down
0.00	0.00	up
0.00	0.00	up

0.00	0.00	down
0.00	0.00	up
0.00	0.00	up
0.00	0.00	up
0.00	0.00	up
0.00	0.00	down
0.00	0.00	down
0.00	0.00	down
0.00	0.00	down
0.00	0.00	down
0.00	0.00	up
0.00	0.00	up
0.00	0.00	up
0.00	0.00	down
0.00	0.00	down
0.00	0.00	down
0.00	0.00	down
0.00	0.00	down
0.00	0.00	down
0.00	0.00	up
0.01	0.03	down
0.00	0.02	down
0.00	0.00	up
0.00	0.00	down
0.00	0.00	down
0.00	0.01	down
0.01	0.04	down
0.00	0.00	up
0.01	0.04	down
0.00	0.02	down
0.01	0.02	down
0	0	down
0	0	up
0.00	0.00	down
0.00	0.00	up
0.00	0.00	up
0.00	0.00	up
0.00	0.00	down
0.00	0.00	down
0.00	0.00	down
0.00	0.00	up
0.00	0.00	down
0.00	0.00	up
0.00	0.00	down
0.00	0.00	up
0.00	0.00	down
0.00	0.00	down
0.00	0.00	down
0.00	0.00	down
0.00	0.00	down
0.00	0.00	down

0.00	0.00	down
0.00	0.00	down
0.00	0.00	down
0.00	0.00	up
0.00	0.00	up
0.00	0.00	down
0.00	0.00	down
0.00	0.00	down
0.00	0.00	down
0.00	0.00	down
0.00	0.00	up
0.00	0.00	up
0.00	0.00	down
0.00	0.00	up
0.00	0.00	down
0.00	0.00	up
0.00	0.00	up
0.00	0.00	down
0.00	0.00	up
0.00	0.00	down
0.00	0.00	down
0.00	0.00	down
0.00	0.00	down
0.00	0.00	up
0.00	0.00	down
0.00	0.00	down
0.00	0.00	down
0.00	0.00	down
0.00	0.00	down
0.00	0.00	down
0.00	0.00	down
0.00	0.00	down
0.00	0.00	down
0.00	0.00	up
0.00	0.00	down
0.00	0.00	down
0.00	0.00	down
0.00	0.00	down
0.00	0.00	down
0.00	0.00	down
0.00	0.00	down
0.00	0.00	down
0.00	0.00	up
0.00	0.00	down
0.00	0.00	down
0.00	0.00	down
0.00	0.00	down
0.00	0.00	down
0.00	0.00	up
0.00	0.00	down
0.00	0.00	down

0.00	0.00	up
0.00	0.00	down
0.00	0.00	down
0.00	0.00	down
0.00	0.00	up
0.00	0.00	down
0.00	0.00	down
0.00	0.00	down
0.00	0.00	up
0.00	0.00	down
0.00	0.00	up
0.00	0.00	down
0.00	0.00	down
0.00	0.00	down
0.00	0.00	up
0.00	0.00	down
0.00	0.00	down
0.00	0.00	down
0.00	0.00	up
0.00	0.00	down
0.00	0.00	down
0.00	0.00	down
0.00	0.00	down
0.00	0.00	down
0.00	0.00	up
0.00	0.01	down
0.00	0.01	down
0.00	0.01	up
0.00	0.01	down
0.00	0.01	down
0.00	0.01	down
0.00	0.02	up
0.00	0.02	up
0.01	0.02	down
0.01	0.02	up
0.01	0.02	down
0.01	0.03	down
0.01	0.03	down
0.01	0.03	down
0.01	0.03	down
0.01	0.03	down
0.01	0.03	down
0.01	0.04	down
0.01	0.04	up
0.01	0.04	down
0.01	0.04	down
0.02	0.05	up
0.02	0.05	up
0.02	0.05	down
0.00	0.00	up
0.01	0.04	down

0.00	0.00	down
0.00	0.00	down
0.00	0.00	down
0.00	0.00	up
0.00	0.00	down
0	0	up
0.00	0.00	up
0.00	0.00	down
0.00	0.00	down
0.00	0.00	down
0.00	0.00	down
0.00	0.00	down
0.00	0.00	up
0.00	0.00	down
0.00	0.00	down
0.00	0.00	down
0.00	0.00	down
0.00	0.00	up
0.00	0.00	down
0.00	0.00	up
0.00	0.00	down
0.00	0.01	up
0.00	0.01	down
0.01	0.02	down
0.01	0.03	down
0.01	0.03	down
0.00	0.00	up
0.00	0.00	down
0.00	0.00	down
0.00	0.00	down
0.00	0.00	down
0.00	0.00	down
0.00	0.00	down
0.00	0.00	down
0.00	0.00	down
0.00	0.01	up
0.01	0.02	down
0.01	0.02	up
0.00	0.00	down
0.00	0.00	up
0.00	0.01	up
0.00	0.00	down
0.00	0.00	up
0.00	0.00	up
0.00	0.00	down
0.00	0.00	down
0.00	0.00	down
0.01	0.02	down
0.00	0.00	up
0.00	0.00	up
0.00	0.00	up

0.00	0.00	down
0.00	0.00	down
0.00	0.00	down
0.01	0.02	down
0.00	0.00	down
0.00	0.00	up
0.00	0.00	up
0.00	0.00	down
0.00	0.00	down
0.00	0.00	down
0.01	0.02	down
0	0	up
0.00	0.00	up
0.00	0.00	up
0.00	0.00	down
0.00	0.00	up
0.00	0.00	down
0.00	0.00	down
0.00	0.00	up
0.00	0.00	up
0.00	0.00	up
0.00	0.00	down
0.00	0.00	up
0.00	0.00	down
0.00	0.01	up
0.01	0.04	down
0.02	0.05	up
0.00	0.00	up
0.00	0.00	up
0.00	0.00	down
0.00	0.00	up
0.00	0.00	down
0.00	0.00	up
0.00	0.00	down
0.00	0.00	down
0.01	0.02	down
0.01	0.02	down
