# Supplementary material for: Transcriptome reveals differential expression of flavor and color in closely related strains of tomato (Solanum lycopersicum)
Source: PeerJ. 2025 Oct 7;13:e20113. doi: 10.7717/peerj.20113 (PMC12513376; doi:10.7717/peerj.20113)
Supplement: Supplemental Information 8 [file peerj-13-20113-s008.pdf]

Table S6: Analysis of GO enrichment for DEGs in MF19-vs-MF20

GO_ID	GO_Term	GO_Cate	P.value	Q.value	gene_id	gene_nam	fc	log2(fc)
GO:00095	plastid	Cellular C	0.00	0.00	Solyc11g0	Solyc11g0	8.62	3.11
GO:00095	plastid	Cellular C	0.00	0.00	Solyc09g0	Solyc09g0	2.22	1.15
GO:00095	plastid	Cellular C	0.00	0.00	Solyc11g0	AOS2	3.20	1.68
GO:00095	plastid	Cellular C	0.00	0.00	Solyc04g0	Solyc04g0	3.78	1.92
GO:00095	plastid	Cellular C	0.00	0.00	Solyc11g0	Solyc11g0	4.12	2.04
GO:00095	plastid	Cellular C	0.00	0.00	Solyc01g0	Solyc01g0	0.03	-5.12
GO:00095	plastid	Cellular C	0.00	0.00	Solyc07g0	Solyc07g0	4.02	2.01
GO:00095	plastid	Cellular C	0.00	0.00	Solyc04g0	PETE	2.69	1.43
GO:00095	plastid	Cellular C	0.00	0.00	Solyc05g0	Solyc05g0	0.09	-3.44
GO:00095	plastid	Cellular C	0.00	0.00	Solyc02g0	Solyc02g0	0.45	-1.14
GO:00095	plastid	Cellular C	0.00	0.00	Solyc11g0	Solyc11g0	0.02	-5.38
GO:00095	plastid	Cellular C	0.00	0.00	Solyc03g0	Solyc03g0	2.79	1.48
GO:00095	plastid	Cellular C	0.00	0.00	Solyc07g0	PDF1A	4.20	2.07
GO:00095	plastid	Cellular C	0.00	0.00	Solyc05g0	Solyc05g0	0.08	-3.58
GO:00095	plastid	Cellular C	0.00	0.00	Solyc03g1	Solyc03g1	4.55	2.18
GO:00095	plastid	Cellular C	0.00	0.00	Solyc02g0	PSBO	5.02	2.33
GO:00095	plastid	Cellular C	0.00	0.00	Solyc09g0	Solyc09g0	0.18	-2.50
GO:00095	plastid	Cellular C	0.00	0.00	Solyc09g0	Solyc09g0	0.05	-4.37
GO:00095	plastid	Cellular C	0.00	0.00	Solyc09g0	Solyc09g0	0.12	-3.08
GO:00095	plastid	Cellular C	0.00	0.00	Solyc11g0	Solyc11g0	0.32	-1.67
GO:00095	plastid	Cellular C	0.00	0.00	Solyc07g0	PSBR	3.05	1.61
GO:00095	plastid	Cellular C	0.00	0.00	Solyc10g0	Solyc10g0	4.66	2.22
GO:00095	plastid	Cellular C	0.00	0.00	Solyc11g0	Solyc11g0	2.20	1.14
GO:00095	plastid	Cellular C	0.00	0.00	Solyc04g0	Solyc04g0	0.00	-11.98
GO:00095	plastid	Cellular C	0.00	0.00	Solyc04g0	Solyc04g0	0.00	-13.91
GO:00095	plastid	Cellular C	0.00	0.00	Solyc11g0	Solyc11g0	0.00	-13.30
GO:00095	plastid	Cellular C	0.00	0.00	Solyc10g0	Solyc10g0	0.04	-4.70
GO:00095	plastid	Cellular C	0.00	0.00	Solyc08g0	Solyc08g0	0.00	-12.86
GO:00095	plastid	Cellular C	0.00	0.00	Solyc10g0	Solyc10g0	0.00	-13.39
GO:00095	plastid	Cellular C	0.00	0.00	Solyc09g0	rpl33	0.05	-4.19
GO:00095	plastid	Cellular C	0.00	0.00	Solyc05g0	Solyc05g0	2.32	1.21
GO:00095	plastid	Cellular C	0.00	0.00	Solyc03g0	Solyc03g0	0.04	-4.63
GO:00095	plastid	Cellular C	0.00	0.00	Solyc12g0	Solyc12g0	0.00	-11.26
GO:00095	plastid	Cellular C	0.00	0.00	Solyc04g0	Solyc04g0	0.00	-13.79
GO:00095	plastid	Cellular C	0.00	0.00	Solyc03g0	Solyc03g0	0.11	-3.15
GO:00095	plastid	Cellular C	0.00	0.00	Solyc05g0	Solyc05g0	0.03	-5.29
GO:00095	plastid	Cellular C	0.00	0.00	Solyc10g0	Solyc10g0	0.00	-13.73
GO:00095	plastid	Cellular C	0.00	0.00	Solyc09g0	Solyc09g0	0.00	-13.14
GO:00095	plastid	Cellular C	0.00	0.00	Solyc10g0	Solyc10g0	0.00	-12.76
GO:00095	plastid	Cellular C	0.00	0.00	Solyc11g0	Solyc11g0	0.00	-11.62
GO:00095	plastid	Cellular C	0.00	0.00	Solyc03g0	RBCS-2A	2.71	1.44
GO:00095	plastid	Cellular C	0.00	0.00	Solyc06g0	Solyc06g0	0.05	-4.46
GO:00095	plastid	Cellular C	0.00	0.00	Solyc08g0	Solyc08g0	0.03	-5.23
GO:00095	plastid	Cellular C	0.00	0.00	Solyc10g0	Solyc10g0	2.07	1.05
GO:00095	plastid	Cellular C	0.00	0.00	Solyc01g0	Solyc01g0	0.02	-5.72
GO:00095	plastid	Cellular C	0.00	0.00	Solyc12g0	Solyc12g0	1818.26	10.83
GO:00095	plastid	Cellular C	0.00	0.00	Solyc06g0	PSBS	2.99	1.58
GO:00095	plastid	Cellular C	0.00	0.00	Solyc08g0	Solyc08g0	8.90	3.15
GO:00095	plastid	Cellular C	0.00	0.00	Solyc03g0	Solyc03g0	2.60	1.38

GO:00095	plastid	Cellular C	0.00	0.00	Solyc10g0	Solyc10g0	0.03	-5.05
GO:00095	plastid	Cellular C	0.00	0.00	Solyc11g0	Solyc11g0	0.00	-11.68
GO:00095	plastid	Cellular C	0.00	0.00	Solyc03g0	Solyc03g0	3.69	1.88
GO:00095	plastid	Cellular C	0.00	0.00	Solyc03g1	Solyc03g1	0.10	-3.25
GO:00095	plastid	Cellular C	0.00	0.00	Solyc02g0	Solyc02g0	0.19	-2.37
GO:00095	plastid	Cellular C	0.00	0.00	Solyc01g0	Solyc01g0	0.00	-11.99
GO:00095	plastid	Cellular C	0.00	0.00	Solyc06g0	Solyc06g0	15.51	3.95
GO:00095	plastid	Cellular C	0.00	0.00	Solyc04g0	Solyc04g0	0.06	-3.97
GO:00095	plastid	Cellular C	0.00	0.00	Solyc01g1	psbZ	0.17	-2.52
GO:00095	plastid	Cellular C	0.00	0.00	Solyc12g0	Solyc12g0	0.16	-2.65
GO:00095	plastid	Cellular C	0.00	0.00	Solyc11g0	Solyc11g0	0.09	-3.49
GO:00095	plastid	Cellular C	0.00	0.00	Solyc11g0	Solyc11g0	0.03	-5.20
GO:00095	plastid	Cellular C	0.00	0.00	Solyc01g0	Solyc01g0	550.66	9.11
GO:00095	plastid	Cellular C	0.00	0.00	Solyc11g0	Solyc11g0	0.22	-2.19
GO:00095	plastid	Cellular C	0.00	0.00	Solyc01g0	Solyc01g0	0.08	-3.58
GO:00095	plastid	Cellular C	0.00	0.00	Solyc12g0	Solyc12g0	6.07	2.60
GO:00095	plastid	Cellular C	0.00	0.00	Solyc01g0	Solyc01g0	0.08	-3.59
GO:00095	plastid	Cellular C	0.00	0.00	Solyc06g0	Solyc06g0	12.77	3.67
GO:00095	plastid	Cellular C	0.00	0.00	Solyc01g0	Solyc01g0	0.00	-11.62
GO:00095	plastid	Cellular C	0.00	0.00	Solyc09g0	Solyc09g0	0.25	-2.00
GO:00095	chloroplast	Cellular C	0.00	0.00	Solyc09g0	Solyc09g0	2.22	1.15
GO:00095	chloroplast	Cellular C	0.00	0.00	Solyc11g0	AOS2	3.20	1.68
GO:00095	chloroplast	Cellular C	0.00	0.00	Solyc07g0	Solyc07g0	4.02	2.01
GO:00095	chloroplast	Cellular C	0.00	0.00	Solyc12g0	Solyc12g0	2.97	1.57
GO:00095	chloroplast	Cellular C	0.00	0.00	Solyc12g0	Solyc12g0	2.48	1.31
GO:00095	chloroplast	Cellular C	0.00	0.00	Solyc04g0	PETE	2.69	1.43
GO:00095	chloroplast	Cellular C	0.00	0.00	Solyc09g0	Solyc09g0	7.18	2.84
GO:00095	chloroplast	Cellular C	0.00	0.00	Solyc09g0	Solyc09g0	5.85	2.55
GO:00095	chloroplast	Cellular C	0.00	0.00	Solyc12g0	Solyc12g0	0.01	-7.31
GO:00095	chloroplast	Cellular C	0.00	0.00	Solyc02g0	Solyc02g0	2.31	1.21
GO:00095	chloroplast	Cellular C	0.00	0.00	Solyc08g0	Solyc08g0	2.79	1.48
GO:00095	chloroplast	Cellular C	0.00	0.00	Solyc12g0	Solyc12g0	2.10	1.07
GO:00095	chloroplast	Cellular C	0.00	0.00	Solyc04g0	Solyc04g0	2.22	1.15
GO:00095	chloroplast	Cellular C	0.00	0.00	Solyc02g0	Solyc02g0	2.62	1.39
GO:00095	chloroplast	Cellular C	0.00	0.00	Solyc05g0	Solyc05g0	0.08	-3.58
GO:00095	chloroplast	Cellular C	0.00	0.00	Solyc02g0	PSBO	5.02	2.33
GO:00095	chloroplast	Cellular C	0.00	0.00	Solyc09g0	Solyc09g0	0.18	-2.50
GO:00095	chloroplast	Cellular C	0.00	0.00	Solyc07g0	PSBR	3.05	1.61
GO:00095	chloroplast	Cellular C	0.00	0.00	Solyc10g0	Solyc10g0	4.66	2.22
GO:00095	chloroplast	Cellular C	0.00	0.00	Solyc10g0	Solyc10g0	15621.48	13.93
GO:00095	chloroplast	Cellular C	0.00	0.00	Solyc11g0	Solyc11g0	0.00	-13.30
GO:00095	chloroplast	Cellular C	0.00	0.00	Solyc05g0	Solyc05g0	6.90	2.79
GO:00095	chloroplast	Cellular C	0.00	0.00	Solyc03g0	Solyc03g0	0.04	-4.63
GO:00095	chloroplast	Cellular C	0.00	0.00	Solyc01g0	Solyc01g0	34.25	5.10
GO:00095	chloroplast	Cellular C	0.00	0.00	Solyc12g0	Solyc12g0	0.00	-11.26
GO:00095	chloroplast	Cellular C	0.00	0.00	Solyc04g0	Solyc04g0	0.00	-13.79
GO:00095	chloroplast	Cellular C	0.00	0.00	Solyc03g0	Solyc03g0	0.11	-3.15
GO:00095	chloroplast	Cellular C	0.00	0.00	Solyc10g0	Solyc10g0	0.00	-13.73
GO:00095	chloroplast	Cellular C	0.00	0.00	Solyc05g0	Solyc05g0	2.46	1.30
GO:00095	chloroplast	Cellular C	0.00	0.00	Solyc09g0	Solyc09g0	0.00	-13.14
GO:00095	chloroplast	Cellular C	0.00	0.00	Solyc05g0	Solyc05g0	39.90	5.32

GO:00095	chloroplast	Cellular Co	0.00	0.00	Solyc06g0	Solyc06g0	3.33	1.73
GO:00095	chloroplast	Cellular Co	0.00	0.00	Solyc06g0	Solyc06g0	0.05	-4.46
GO:00095	chloroplast	Cellular Co	0.00	0.00	Solyc10g0	Solyc10g0	2.07	1.05
GO:00095	chloroplast	Cellular Co	0.00	0.00	Solyc01g0	Solyc01g0	0.02	-5.72
GO:00095	chloroplast	Cellular Co	0.00	0.00	Solyc06g0	PSBS	2.99	1.58
GO:00095	chloroplast	Cellular Co	0.00	0.00	Solyc03g0	Solyc03g0	2.60	1.38
GO:00095	chloroplast	Cellular Co	0.00	0.00	Solyc10g0	Solyc10g0	0.03	-5.05
GO:00095	chloroplast	Cellular Co	0.00	0.00	Solyc03g0	Solyc03g0	3.69	1.88
GO:00095	chloroplast	Cellular Co	0.00	0.00	Solyc03g1	Solyc03g1	0.10	-3.25
GO:00095	chloroplast	Cellular Co	0.00	0.00	Solyc05g0	Solyc05g0	10.06	3.33
GO:00095	chloroplast	Cellular Co	0.00	0.00	Solyc02g0	Solyc02g0	0.19	-2.37
GO:00095	chloroplast	Cellular Co	0.00	0.00	Solyc12g0	Solyc12g0	0.25	-2.01
GO:00095	chloroplast	Cellular Co	0.00	0.00	Solyc07g0	Solyc07g0	2.00	1.00
GO:00095	chloroplast	Cellular Co	0.00	0.00	Solyc09g0	Solyc09g0	2.86	1.51
GO:00095	chloroplast	Cellular Co	0.00	0.00	Solyc01g1	psbZ	0.17	-2.52
GO:00095	chloroplast	Cellular Co	0.00	0.00	Solyc01g0	Solyc01g0	550.66	9.11
GO:00095	chloroplast	Cellular Co	0.00	0.00	Solyc11g0	Solyc11g0	0.00	-12.69
GO:00095	chloroplast	Cellular Co	0.00	0.00	Solyc11g0	Solyc11g0	0.22	-2.19
GO:00095	chloroplast	Cellular Co	0.00	0.00	Solyc01g0	Solyc01g0	0.08	-3.58
GO:00095	chloroplast	Cellular Co	0.00	0.00	Solyc01g0	Solyc01g0	0.00	-11.62
GO:00095	chloroplast	Cellular Co	0.00	0.00	Solyc06g0	Solyc06g0	2.66	1.41
GO:00095	chloroplast	Cellular Co	0.00	0.00	Solyc01g1	Solyc01g1	5.77	2.53
GO:00095	chloroplast	Cellular Co	0.00	0.00	Solyc01g0	Solyc01g0	2.18	1.13
GO:00095	chloroplast	Cellular Co	0.00	0.00	Solyc02g0	Solyc02g0	3.57	1.83
GO:00095	chloroplast	Cellular Co	0.00	0.00	Solyc11g0	Solyc11g0	8.62	3.11
GO:00095	chloroplast	Cellular Co	0.00	0.00	Solyc11g0	AOS2	3.20	1.68
GO:00095	chloroplast	Cellular Co	0.00	0.00	Solyc04g0	Solyc04g0	3.78	1.92
GO:00095	chloroplast	Cellular Co	0.00	0.00	Solyc11g0	Solyc11g0	4.12	2.04
GO:00095	chloroplast	Cellular Co	0.00	0.00	Solyc01g0	Solyc01g0	0.03	-5.12
GO:00095	chloroplast	Cellular Co	0.00	0.00	Solyc04g0	Solyc04g0	3.50	1.81
GO:00095	chloroplast	Cellular Co	0.00	0.00	Solyc07g0	Solyc07g0	4.02	2.01
GO:00095	chloroplast	Cellular Co	0.00	0.00	Solyc04g0	Solyc04g0	8.55	3.10
GO:00095	chloroplast	Cellular Co	0.00	0.00	Solyc12g0	Solyc12g0	2.20	1.14
GO:00095	chloroplast	Cellular Co	0.00	0.00	Solyc04g0	PETE	2.69	1.43
GO:00095	chloroplast	Cellular Co	0.00	0.00	Solyc05g0	Solyc05g0	0.09	-3.44
GO:00095	chloroplast	Cellular Co	0.00	0.00	Solyc05g0	Solyc05g0	0.49	-1.03
GO:00095	chloroplast	Cellular Co	0.00	0.00	Solyc05g0	Solyc05g0	4.51	2.17
GO:00095	chloroplast	Cellular Co	0.00	0.00	Solyc02g0	Solyc02g0	0.45	-1.14
GO:00095	chloroplast	Cellular Co	0.00	0.00	Solyc01g0	Solyc01g0	2.45	1.29
GO:00095	chloroplast	Cellular Co	0.00	0.00	Solyc11g0	Solyc11g0	0.02	-5.38
GO:00095	chloroplast	Cellular Co	0.00	0.00	Solyc09g0	Solyc09g0	0.36	-1.46
GO:00095	chloroplast	Cellular Co	0.00	0.00	Solyc03g0	Solyc03g0	2.79	1.48
GO:00095	chloroplast	Cellular Co	0.00	0.00	Solyc07g0	PDF1A	4.20	2.07
GO:00095	chloroplast	Cellular Co	0.00	0.00	Solyc05g0	Solyc05g0	0.08	-3.58
GO:00095	chloroplast	Cellular Co	0.00	0.00	Solyc03g1	Solyc03g1	4.55	2.18
GO:00095	chloroplast	Cellular Co	0.00	0.00	Solyc02g0	PSBO	5.02	2.33
GO:00095	chloroplast	Cellular Co	0.00	0.00	Solyc06g0	Solyc06g0	0.34	-1.57
GO:00095	chloroplast	Cellular Co	0.00	0.00	Solyc09g0	Solyc09g0	0.18	-2.50
GO:00095	chloroplast	Cellular Co	0.00	0.00	Solyc09g0	Solyc09g0	0.05	-4.37
GO:00095	chloroplast	Cellular Co	0.00	0.00	Solyc09g0	Solyc09g0	0.12	-3.08
GO:00095	chloroplast	Cellular Co	0.00	0.00	Solyc11g0	Solyc11g0	0.32	-1.67

GO:00095	chloroplast	Cellular C	0.00	0.00	Solyc07g0	PSBR	3.05	1.61
GO:00095	chloroplast	Cellular C	0.00	0.00	Solyc10g0	Solyc10g0	4.66	2.22
GO:00095	chloroplast	Cellular C	0.00	0.00	Solyc07g0	Solyc07g0	2.00	1.00
GO:00095	chloroplast	Cellular C	0.00	0.00	Solyc11g0	Solyc11g0	2.20	1.14
GO:00095	chloroplast	Cellular C	0.00	0.00	Solyc04g0	Solyc04g0	0.00	-11.98
GO:00095	chloroplast	Cellular C	0.00	0.00	Solyc04g0	Solyc04g0	0.00	-13.91
GO:00095	chloroplast	Cellular C	0.00	0.00	Solyc11g0	Solyc11g0	0.00	-13.30
GO:00095	chloroplast	Cellular C	0.00	0.00	Solyc10g0	Solyc10g0	0.04	-4.70
GO:00095	chloroplast	Cellular C	0.00	0.00	Solyc08g0	Solyc08g0	0.00	-12.86
GO:00095	chloroplast	Cellular C	0.00	0.00	Solyc10g0	Solyc10g0	0.00	-13.39
GO:00095	chloroplast	Cellular C	0.00	0.00	Solyc09g0	rpl33	0.05	-4.19
GO:00095	chloroplast	Cellular C	0.00	0.00	Solyc09g0	Solyc09g0	0.00	-13.71
GO:00095	chloroplast	Cellular C	0.00	0.00	Solyc03g0	Solyc03g0	2.06	1.04
GO:00095	chloroplast	Cellular C	0.00	0.00	Solyc12g0	Solyc12g0	0.00	-13.90
GO:00095	chloroplast	Cellular C	0.00	0.00	Solyc05g0	Solyc05g0	2.32	1.21
GO:00095	chloroplast	Cellular C	0.00	0.00	Solyc03g0	Solyc03g0	0.04	-4.63
GO:00095	chloroplast	Cellular C	0.00	0.00	Solyc09g0	Solyc09g0	4.92	2.30
GO:00095	chloroplast	Cellular C	0.00	0.00	Solyc09g0	Solyc09g0	0.03	-4.88
GO:00095	chloroplast	Cellular C	0.00	0.00	Solyc12g0	Solyc12g0	0.00	-11.26
GO:00095	chloroplast	Cellular C	0.00	0.00	Solyc02g0	Solyc02g0	3.41	1.77
GO:00095	chloroplast	Cellular C	0.00	0.00	Solyc05g0	Solyc05g0	0.00	-14.18
GO:00095	chloroplast	Cellular C	0.00	0.00	Solyc04g0	Solyc04g0	0.00	-13.79
GO:00095	chloroplast	Cellular C	0.00	0.00	Solyc03g0	Solyc03g0	0.11	-3.15
GO:00095	chloroplast	Cellular C	0.00	0.00	Solyc04g0	Solyc04g0	0.49	-1.01
GO:00095	chloroplast	Cellular C	0.00	0.00	Solyc05g0	Solyc05g0	0.03	-5.29
GO:00095	chloroplast	Cellular C	0.00	0.00	Solyc04g0	Solyc04g0	2.74	1.46
GO:00095	chloroplast	Cellular C	0.00	0.00	Solyc10g0	Solyc10g0	0.00	-13.73
GO:00095	chloroplast	Cellular C	0.00	0.00	Solyc08g0	Solyc08g0	0.02	-6.06
GO:00095	chloroplast	Cellular C	0.00	0.00	Solyc09g0	Solyc09g0	0.00	-13.84
GO:00095	chloroplast	Cellular C	0.00	0.00	Solyc09g0	Solyc09g0	0.00	-13.14
GO:00095	chloroplast	Cellular C	0.00	0.00	Solyc05g0	Solyc05g0	39.90	5.32
GO:00095	chloroplast	Cellular C	0.00	0.00	Solyc10g0	Solyc10g0	0.00	-12.76
GO:00095	chloroplast	Cellular C	0.00	0.00	Solyc06g0	Solyc06g0	3.33	1.73
GO:00095	chloroplast	Cellular C	0.00	0.00	Solyc11g0	Solyc11g0	0.00	-11.62
GO:00095	chloroplast	Cellular C	0.00	0.00	Solyc03g0	RBCS-2A	2.71	1.44
GO:00095	chloroplast	Cellular C	0.00	0.00	Solyc06g0	Solyc06g0	0.05	-4.46
GO:00095	chloroplast	Cellular C	0.00	0.00	Solyc08g0	Solyc08g0	0.03	-5.23
GO:00095	chloroplast	Cellular C	0.00	0.00	Solyc09g0	Solyc09g0	8.04	3.01
GO:00095	chloroplast	Cellular C	0.00	0.00	Solyc10g0	Solyc10g0	0.03	-5.30
GO:00095	chloroplast	Cellular C	0.00	0.00	Solyc03g0	Solyc03g0	0.00	-12.50
GO:00095	chloroplast	Cellular C	0.00	0.00	Solyc10g0	Solyc10g0	2.07	1.05
GO:00095	chloroplast	Cellular C	0.00	0.00	Solyc08g0	Solyc08g0	2.43	1.28
GO:00095	chloroplast	Cellular C	0.00	0.00	Solyc09g0	Solyc09g0	0.07	-3.94
GO:00095	chloroplast	Cellular C	0.00	0.00	Solyc01g0	Solyc01g0	0.02	-5.72
GO:00095	chloroplast	Cellular C	0.00	0.00	Solyc12g0	Solyc12g0	1818.26	10.83
GO:00095	chloroplast	Cellular C	0.00	0.00	Solyc06g0	PSBS	2.99	1.58
GO:00095	chloroplast	Cellular C	0.00	0.00	Solyc08g0	Solyc08g0	8.90	3.15
GO:00095	chloroplast	Cellular C	0.00	0.00	Solyc03g1	Solyc03g1	0.02	-5.50
GO:00095	chloroplast	Cellular C	0.00	0.00	Solyc03g0	Solyc03g0	2.60	1.38
GO:00095	chloroplast	Cellular C	0.00	0.00	Solyc10g0	Solyc10g0	0.03	-5.05
GO:00095	chloroplast	Cellular C	0.00	0.00	Solyc11g0	Solyc11g0	0.00	-11.68

GO:00095	chloroplas	Cellular Co	0.00	0.00	Solyc03g0	Solyc03g0	3.69	1.88
GO:00095	chloroplas	Cellular Co	0.00	0.00	Solyc09g0	Solyc09g0	0.41	-1.30
GO:00095	chloroplas	Cellular Co	0.00	0.00	Solyc03g1	Solyc03g1	0.10	-3.25
GO:00095	chloroplas	Cellular Co	0.00	0.00	Solyc02g0	Solyc02g0	0.19	-2.37
GO:00095	chloroplas	Cellular Co	0.00	0.00	Solyc01g0	Solyc01g0	0.00	-11.99
GO:00095	chloroplas	Cellular Co	0.00	0.00	Solyc07g0	Solyc07g0	2.00	1.00
GO:00095	chloroplas	Cellular Co	0.00	0.00	Solyc11g0	Solyc11g0	0.00	-12.68
GO:00095	chloroplas	Cellular Co	0.00	0.00	Solyc06g0	Solyc06g0	15.51	3.95
GO:00095	chloroplas	Cellular Co	0.00	0.00	Solyc04g0	Solyc04g0	0.06	-3.97
GO:00095	chloroplas	Cellular Co	0.00	0.00	Solyc06g0	Solyc06g0	0.03	-4.93
GO:00095	chloroplas	Cellular Co	0.00	0.00	Solyc01g1	psbZ	0.17	-2.52
GO:00095	chloroplas	Cellular Co	0.00	0.00	Solyc12g0	Solyc12g0	0.16	-2.65
GO:00095	chloroplas	Cellular Co	0.00	0.00	Solyc11g0	Solyc11g0	0.09	-3.49
GO:00095	chloroplas	Cellular Co	0.00	0.00	Solyc11g0	Solyc11g0	0.03	-5.20
GO:00095	chloroplas	Cellular Co	0.00	0.00	Solyc02g0	Solyc02g0	574.97	9.17
GO:00095	chloroplas	Cellular Co	0.00	0.00	Solyc01g0	Solyc01g0	550.66	9.11
GO:00095	chloroplas	Cellular Co	0.00	0.00	Solyc11g0	Solyc11g0	0.22	-2.19
GO:00095	chloroplas	Cellular Co	0.00	0.00	Solyc01g0	Solyc01g0	0.08	-3.58
GO:00095	chloroplas	Cellular Co	0.00	0.00	Solyc02g0	Solyc02g0	2.27	1.18
GO:00095	chloroplas	Cellular Co	0.00	0.00	Solyc03g1	Solyc03g1	5.76	2.53
GO:00095	chloroplas	Cellular Co	0.00	0.00	Solyc09g0	Solyc09g0	0.49	-1.03
GO:00095	chloroplas	Cellular Co	0.00	0.00	Solyc12g0	Solyc12g0	6.07	2.60
GO:00095	chloroplas	Cellular Co	0.00	0.00	Solyc01g0	Solyc01g0	0.08	-3.59
GO:00095	chloroplas	Cellular Co	0.00	0.00	Solyc06g0	Solyc06g0	12.77	3.67
GO:00095	chloroplas	Cellular Co	0.00	0.00	Solyc01g0	Solyc01g0	0.00	-11.62
GO:00095	chloroplas	Cellular Co	0.00	0.00	Solyc06g0	Solyc06g0	2.66	1.41
GO:00095	chloroplas	Cellular Co	0.00	0.00	Solyc01g1	Solyc01g1	0.37	-1.43
GO:00095	chloroplas	Cellular Co	0.00	0.00	Solyc09g0	Solyc09g0	0.25	-2.00
GO:00095	thylakoid	Cellular Co	0.00	0.00	Solyc05g0	Solyc05g0	0.01	-6.09
GO:00095	thylakoid	Cellular Co	0.00	0.00	Solyc04g0	Solyc04g0	2.70	1.43
GO:00095	thylakoid	Cellular Co	0.00	0.00	Solyc07g0	Solyc07g0	4.02	2.01
GO:00095	thylakoid	Cellular Co	0.00	0.00	Solyc04g0	PETE	2.69	1.43
GO:00095	thylakoid	Cellular Co	0.00	0.00	Solyc09g0	Solyc09g0	7.18	2.84
GO:00095	thylakoid	Cellular Co	0.00	0.00	Solyc02g0	Solyc02g0	2.31	1.21
GO:00095	thylakoid	Cellular Co	0.00	0.00	Solyc05g0	Solyc05g0	0.08	-3.58
GO:00095	thylakoid	Cellular Co	0.00	0.00	Solyc02g0	PSBO	5.02	2.33
GO:00095	thylakoid	Cellular Co	0.00	0.00	Solyc09g0	Solyc09g0	0.18	-2.50
GO:00095	thylakoid	Cellular Co	0.00	0.00	Solyc07g0	PSBR	3.05	1.61
GO:00095	thylakoid	Cellular Co	0.00	0.00	Solyc10g0	Solyc10g0	4.66	2.22
GO:00095	thylakoid	Cellular Co	0.00	0.00	Solyc11g0	Solyc11g0	0.00	-13.30
GO:00095	thylakoid	Cellular Co	0.00	0.00	Solyc03g0	Solyc03g0	0.04	-4.63
GO:00095	thylakoid	Cellular Co	0.00	0.00	Solyc12g0	Solyc12g0	0.00	-11.26
GO:00095	thylakoid	Cellular Co	0.00	0.00	Solyc02g0	Solyc02g0	3.41	1.77
GO:00095	thylakoid	Cellular Co	0.00	0.00	Solyc05g0	Solyc05g0	0.00	-13.36
GO:00095	thylakoid	Cellular Co	0.00	0.00	Solyc04g0	Solyc04g0	0.00	-13.79
GO:00095	thylakoid	Cellular Co	0.00	0.00	Solyc03g0	Solyc03g0	0.11	-3.15
GO:00095	thylakoid	Cellular Co	0.00	0.00	Solyc10g0	Solyc10g0	0.00	-13.68
GO:00095	thylakoid	Cellular Co	0.00	0.00	Solyc10g0	Solyc10g0	0.00	-13.73
GO:00095	thylakoid	Cellular Co	0.00	0.00	Solyc09g0	Solyc09g0	0.00	-13.14
GO:00095	thylakoid	Cellular Co	0.00	0.00	Solyc02g0	Solyc02g0	0.03	-4.99
GO:00095	thylakoid	Cellular Co	0.00	0.00	Solyc06g0	Solyc06g0	0.05	-4.46

GO:00095	thylakoid	Cellular C	0.00	0.00	Solyc12g0	Solyc12g0	0.00	-10.94
GO:00095	thylakoid	Cellular C	0.00	0.00	Solyc10g0	Solyc10g0	0.03	-5.30
GO:00095	thylakoid	Cellular C	0.00	0.00	Solyc10g0	Solyc10g0	2.07	1.05
GO:00095	thylakoid	Cellular C	0.00	0.00	Solyc01g0	Solyc01g0	0.02	-5.72
GO:00095	thylakoid	Cellular C	0.00	0.00	Solyc06g0	PSBS	2.99	1.58
GO:00095	thylakoid	Cellular C	0.00	0.00	Solyc03g0	Solyc03g0	2.60	1.38
GO:00095	thylakoid	Cellular C	0.00	0.00	Solyc10g0	Solyc10g0	0.03	-5.05
GO:00095	thylakoid	Cellular C	0.00	0.00	Solyc11g0	Solyc11g0	0.00	-11.68
GO:00095	thylakoid	Cellular C	0.00	0.00	Solyc03g0	Solyc03g0	3.69	1.88
GO:00095	thylakoid	Cellular C	0.00	0.00	Solyc03g1	Solyc03g1	0.10	-3.25
GO:00095	thylakoid	Cellular C	0.00	0.00	Solyc02g0	Solyc02g0	0.19	-2.37
GO:00095	thylakoid	Cellular C	0.00	0.00	Solyc01g0	Solyc01g0	0.00	-11.99
GO:00095	thylakoid	Cellular C	0.00	0.00	Solyc01g1	psbZ	0.17	-2.52
GO:00095	thylakoid	Cellular C	0.00	0.00	Solyc11g0	Solyc11g0	0.22	-2.19
GO:00095	thylakoid	Cellular C	0.00	0.00	Solyc01g0	Solyc01g0	0.08	-3.58
GO:00095	thylakoid	Cellular C	0.00	0.00	Solyc04g0	Solyc04g0	0.10	-3.25
GO:00095	thylakoid	Cellular C	0.00	0.00	Solyc01g0	Solyc01g0	0.05	-4.42
GO:00095	thylakoid	Cellular C	0.00	0.00	Solyc01g0	Solyc01g0	0.00	-11.62
GO:00095	thylakoid	Cellular C	0.00	0.00	Solyc06g0	Solyc06g0	0.12	-3.11
GO:00159	photosynth	Biological	0.00	0.00	Solyc05g0	Solyc05g0	0.01	-6.09
GO:00159	photosynth	Biological	0.00	0.00	Solyc01g0	Solyc01g0	0.03	-5.12
GO:00159	photosynth	Biological	0.00	0.00	Solyc07g0	Solyc07g0	4.02	2.01
GO:00159	photosynth	Biological	0.00	0.00	Solyc10g0	Solyc10g0	2.93	1.55
GO:00159	photosynth	Biological	0.00	0.00	Solyc09g0	Solyc09g0	7.18	2.84
GO:00159	photosynth	Biological	0.00	0.00	Solyc02g0	Solyc02g0	2.31	1.21
GO:00159	photosynth	Biological	0.00	0.00	Solyc12g0	Solyc12g0	2.10	1.07
GO:00159	photosynth	Biological	0.00	0.00	Solyc05g0	Solyc05g0	0.08	-3.58
GO:00159	photosynth	Biological	0.00	0.00	Solyc02g0	PSBO	5.02	2.33
GO:00159	photosynth	Biological	0.00	0.00	Solyc07g0	PSBR	3.05	1.61
GO:00159	photosynth	Biological	0.00	0.00	Solyc10g0	Solyc10g0	4.66	2.22
GO:00159	photosynth	Biological	0.00	0.00	Solyc09g0	Solyc09g0	4.92	2.30
GO:00159	photosynth	Biological	0.00	0.00	Solyc12g0	Solyc12g0	0.00	-11.26
GO:00159	photosynth	Biological	0.00	0.00	Solyc02g0	Solyc02g0	3.41	1.77
GO:00159	photosynth	Biological	0.00	0.00	Solyc05g0	Solyc05g0	0.00	-13.36
GO:00159	photosynth	Biological	0.00	0.00	Solyc04g0	Solyc04g0	0.00	-13.79
GO:00159	photosynth	Biological	0.00	0.00	Solyc10g0	Solyc10g0	0.00	-13.68
GO:00159	photosynth	Biological	0.00	0.00	Solyc10g0	Solyc10g0	0.00	-12.09
GO:00159	photosynth	Biological	0.00	0.00	Solyc09g0	Solyc09g0	0.00	-13.14
GO:00159	photosynth	Biological	0.00	0.00	Solyc02g0	Solyc02g0	0.03	-4.99
GO:00159	photosynth	Biological	0.00	0.00	Solyc03g0	RBCS-2A	2.71	1.44
GO:00159	photosynth	Biological	0.00	0.00	Solyc11g0	Solyc11g0	0.00	-13.08
GO:00159	photosynth	Biological	0.00	0.00	Solyc09g0	Solyc09g0	0.00	-13.63
GO:00159	photosynth	Biological	0.00	0.00	Solyc12g0	Solyc12g0	0.00	-10.94
GO:00159	photosynth	Biological	0.00	0.00	Solyc10g0	Solyc10g0	0.03	-5.30
GO:00159	photosynth	Biological	0.00	0.00	Solyc10g0	Solyc10g0	2.07	1.05
GO:00159	photosynth	Biological	0.00	0.00	Solyc01g0	Solyc01g0	0.02	-5.72
GO:00159	photosynth	Biological	0.00	0.00	Solyc06g0	PSBS	2.99	1.58
GO:00159	photosynth	Biological	0.00	0.00	Solyc07g0	Solyc07g0	2.47	1.30
GO:00159	photosynth	Biological	0.00	0.00	Solyc03g0	Solyc03g0	2.60	1.38
GO:00159	photosynth	Biological	0.00	0.00	Solyc03g0	Solyc03g0	3.69	1.88
GO:00159	photosynth	Biological	0.00	0.00	Solyc03g1	Solyc03g1	0.10	-3.25

GO:00159	photosynth	Biological	0.00	0.00	Solyc01g1	psbZ	0.17	-2.52
GO:00159	photosynth	Biological	0.00	0.00	Solyc07g0	Solyc07g0	0.00	-13.48
GO:00159	photosynth	Biological	0.00	0.00	Solyc04g0	Solyc04g0	5.19	2.37
GO:00159	photosynth	Biological	0.00	0.00	Solyc08g0	Solyc08g0	1537.41	10.59
GO:00159	photosynth	Biological	0.00	0.00	Solyc04g0	Solyc04g0	0.10	-3.25
GO:00159	photosynth	Biological	0.00	0.00	Solyc12g0	Solyc12g0	6.07	2.60
GO:00159	photosynth	Biological	0.00	0.00	Solyc01g0	Solyc01g0	0.05	-4.42
GO:00159	photosynth	Biological	0.00	0.00	Solyc06g0	Solyc06g0	4.49	2.17
GO:00159	photosynth	Biological	0.00	0.00	Solyc03g1	Solyc03g1	2.21	1.14
GO:00159	photosynth	Biological	0.00	0.00	Solyc06g0	Solyc06g0	0.12	-3.11
GO:00164	oxidoreduc	Molecular	0.00	0.00	Solyc07g0	ACO1	2.82	1.49
GO:00164	oxidoreduc	Molecular	0.00	0.00	Solyc10g0	Solyc10g0	4.23	2.08
GO:00164	oxidoreduc	Molecular	0.00	0.00	Solyc03g0	Solyc03g0	7.65	2.93
GO:00164	oxidoreduc	Molecular	0.00	0.00	Solyc07g0	Solyc07g0	9.18	3.20
GO:00164	oxidoreduc	Molecular	0.00	0.00	Solyc08g0	Solyc08g0	0.14	-2.87
GO:00164	oxidoreduc	Molecular	0.00	0.00	Solyc02g0	Solyc02g0	0.08	-3.73
GO:00164	oxidoreduc	Molecular	0.00	0.00	Solyc01g0	Solyc01g0	2.18	1.13
GO:00164	oxidoreduc	Molecular	0.00	0.00	Solyc10g0	Solyc10g0	2.09	1.06
GO:00164	oxidoreduc	Molecular	0.00	0.00	Solyc02g0	Solyc02g0	3.57	1.83
GO:00164	oxidoreduc	Molecular	0.00	0.00	Solyc11g0	Solyc11g0	3.25	1.70
GO:00164	oxidoreduc	Molecular	0.00	0.00	Solyc12g0	Solyc12g0	2.82	1.50
GO:00164	oxidoreduc	Molecular	0.00	0.00	Solyc11g0	Solyc11g0	8.62	3.11
GO:00164	oxidoreduc	Molecular	0.00	0.00	Solyc08g0	Solyc08g0	4.51	2.17
GO:00164	oxidoreduc	Molecular	0.00	0.00	Solyc06g0	Solyc06g0	2.22	1.15
GO:00164	oxidoreduc	Molecular	0.00	0.00	Solyc01g0	Solyc01g0	0.31	-1.67
GO:00164	oxidoreduc	Molecular	0.00	0.00	Solyc03g0	Solyc03g0	5.30	2.41
GO:00164	oxidoreduc	Molecular	0.00	0.00	Solyc11g0	AOS2	3.20	1.68
GO:00164	oxidoreduc	Molecular	0.00	0.00	Solyc02g0	Solyc02g0	0.35	-1.50
GO:00164	oxidoreduc	Molecular	0.00	0.00	Solyc10g0	Solyc10g0	6.94	2.80
GO:00164	oxidoreduc	Molecular	0.00	0.00	Solyc01g1	Solyc01g1	4.98	2.32
GO:00164	oxidoreduc	Molecular	0.00	0.00	Solyc11g0	Solyc11g0	4.12	2.04
GO:00164	oxidoreduc	Molecular	0.00	0.00	Solyc01g0	Solyc01g0	0.03	-5.12
GO:00164	oxidoreduc	Molecular	0.00	0.00	Solyc04g0	Solyc04g0	3.50	1.81
GO:00164	oxidoreduc	Molecular	0.00	0.00	Solyc03g0	Solyc03g0	5.61	2.49
GO:00164	oxidoreduc	Molecular	0.00	0.00	Solyc08g0	LOX1.1	2.60	1.38
GO:00164	oxidoreduc	Molecular	0.00	0.00	Solyc10g0	Solyc10g0	3.13	1.64
GO:00164	oxidoreduc	Molecular	0.00	0.00	Solyc07g0	Solyc07g0	3.00	1.59
GO:00164	oxidoreduc	Molecular	0.00	0.00	Solyc05g0	Solyc05g0	2.65	1.41
GO:00164	oxidoreduc	Molecular	0.00	0.00	Solyc10g0	Solyc10g0	2.48	1.31
GO:00164	oxidoreduc	Molecular	0.00	0.00	Solyc03g0	Solyc03g0	2.10	1.07
GO:00164	oxidoreduc	Molecular	0.00	0.00	Solyc01g0	Solyc01g0	0.22	-2.18
GO:00164	oxidoreduc	Molecular	0.00	0.00	Solyc03g1	E4	2.38	1.25
GO:00164	oxidoreduc	Molecular	0.00	0.00	Solyc02g0	Solyc02g0	0.50	-1.01
GO:00164	oxidoreduc	Molecular	0.00	0.00	Solyc01g1	Solyc01g1	2.47	1.30
GO:00164	oxidoreduc	Molecular	0.00	0.00	Solyc05g0	Solyc05g0	4.77	2.25
GO:00164	oxidoreduc	Molecular	0.00	0.00	Solyc07g0	Solyc07g0	0.26	-1.95
GO:00164	oxidoreduc	Molecular	0.00	0.00	Solyc08g0	Solyc08g0	2.85	1.51
GO:00164	oxidoreduc	Molecular	0.00	0.00	Solyc02g0	Solyc02g0	0.16	-2.68
GO:00164	oxidoreduc	Molecular	0.00	0.00	Solyc04g0	Solyc04g0	2.29	1.19
GO:00164	oxidoreduc	Molecular	0.00	0.00	Solyc11g0	Solyc11g0	2.05	1.04
GO:00164	oxidoreduc	Molecular	0.00	0.00	Solyc08g0	Solyc08g0	3.99	2.00

GO:00164	oxidoreduc	Molecular	0.00	0.00	Solyc03g1	Solyc03g1	0.30	-1.75
GO:00164	oxidoreduc	Molecular	0.00	0.00	Solyc05g0	Solyc05g0	2.11	1.08
GO:00164	oxidoreduc	Molecular	0.00	0.00	Solyc07g0	Solyc07g0	2.96	1.57
GO:00164	oxidoreduc	Molecular	0.00	0.00	Solyc02g0	Solyc02g0	0.45	-1.14
GO:00164	oxidoreduc	Molecular	0.00	0.00	Solyc07g0	Solyc07g0	2.20	1.14
GO:00164	oxidoreduc	Molecular	0.00	0.00	Solyc09g0	Solyc09g0	0.36	-1.46
GO:00164	oxidoreduc	Molecular	0.00	0.00	Solyc03g0	Solyc03g0	2.79	1.48
GO:00164	oxidoreduc	Molecular	0.00	0.00	Solyc04g0	Solyc04g0	4.57	2.19
GO:00164	oxidoreduc	Molecular	0.00	0.00	Solyc01g0	Solyc01g0	2.26	1.18
GO:00164	oxidoreduc	Molecular	0.00	0.00	Solyc02g0	Solyc02g0	14.41	3.85
GO:00164	oxidoreduc	Molecular	0.00	0.00	Solyc05g0	Solyc05g0	3.22	1.69
GO:00164	oxidoreduc	Molecular	0.00	0.00	Solyc06g0	Solyc06g0	0.15	-2.75
GO:00164	oxidoreduc	Molecular	0.00	0.00	Solyc02g0	Solyc02g0	2.56	1.36
GO:00164	oxidoreduc	Molecular	0.00	0.00	Solyc11g0	Solyc11g0	0.32	-1.67
GO:00164	oxidoreduc	Molecular	0.00	0.00	Solyc06g0	Solyc06g0	3.53	1.82
GO:00164	oxidoreduc	Molecular	0.00	0.00	Solyc06g0	Solyc06g0	3.25	1.70
GO:00164	oxidoreduc	Molecular	0.00	0.00	Solyc01g0	Solyc01g0	0.06	-3.97
GO:00164	oxidoreduc	Molecular	0.00	0.00	Solyc12g0	Solyc12g0	4.01	2.00
GO:00164	oxidoreduc	Molecular	0.00	0.00	Solyc11g0	Solyc11g0	5.99	2.58
GO:00164	oxidoreduc	Molecular	0.00	0.00	Solyc12g0	Solyc12g0	0.00	-11.26
GO:00164	oxidoreduc	Molecular	0.00	0.00	Solyc03g1	Solyc03g1	0.48	-1.07
GO:00164	oxidoreduc	Molecular	0.00	0.00	Solyc11g0	Solyc11g0	3.46	1.79
GO:00164	oxidoreduc	Molecular	0.00	0.00	Solyc09g0	Solyc09g0	0.47	-1.10
GO:00164	oxidoreduc	Molecular	0.00	0.00	Solyc07g0	Solyc07g0	4.00	2.00
GO:00164	oxidoreduc	Molecular	0.00	0.00	Solyc01g1	Solyc01g1	37.32	5.22
GO:00164	oxidoreduc	Molecular	0.00	0.00	Solyc12g0	Solyc12g0	2.73	1.45
GO:00164	oxidoreduc	Molecular	0.00	0.00	Solyc02g0	Solyc02g0	2.01	1.01
GO:00164	oxidoreduc	Molecular	0.00	0.00	Solyc01g1	Solyc01g1	4.37	2.13
GO:00164	oxidoreduc	Molecular	0.00	0.00	Solyc09g0	Solyc09g0	0.00	-13.14
GO:00164	oxidoreduc	Molecular	0.00	0.00	Solyc01g0	Solyc01g0	3.53	1.82
GO:00164	oxidoreduc	Molecular	0.00	0.00	Solyc02g0	Solyc02g0	5.76	2.53
GO:00164	oxidoreduc	Molecular	0.00	0.00	Solyc06g0	Solyc06g0	2169.21	11.08
GO:00164	oxidoreduc	Molecular	0.00	0.00	Solyc05g0	Solyc05g0	0.18	-2.44
GO:00164	oxidoreduc	Molecular	0.00	0.00	Solyc07g0	Solyc07g0	29.42	4.88
GO:00164	oxidoreduc	Molecular	0.00	0.00	Solyc09g0	Solyc09g0	767.92	9.58
GO:00164	oxidoreduc	Molecular	0.00	0.00	Solyc01g0	Solyc01g0	0.02	-5.72
GO:00164	oxidoreduc	Molecular	0.00	0.00	Solyc10g0	Solyc10g0	2.03	1.02
GO:00164	oxidoreduc	Molecular	0.00	0.00	Solyc03g1	Solyc03g1	2.05	1.04
GO:00164	oxidoreduc	Molecular	0.00	0.00	Solyc02g0	Solyc02g0	0.07	-3.82
GO:00164	oxidoreduc	Molecular	0.00	0.00	Solyc08g0	Solyc08g0	1235.43	10.27
GO:00164	oxidoreduc	Molecular	0.00	0.00	Solyc08g0	Solyc08g0	0.46	-1.13
GO:00164	oxidoreduc	Molecular	0.00	0.00	Solyc12g1	Solyc12g1	0.00	-10.11
GO:00164	oxidoreduc	Molecular	0.00	0.00	Solyc02g0	Solyc02g0	0.22	-2.21
GO:00164	oxidoreduc	Molecular	0.00	0.00	Solyc01g0	Solyc01g0	2.27	1.18
GO:00164	oxidoreduc	Molecular	0.00	0.00	Solyc01g0	Solyc01g0	36.75	5.20
GO:00164	oxidoreduc	Molecular	0.00	0.00	Solyc03g1	Solyc03g1	0.10	-3.25
GO:00164	oxidoreduc	Molecular	0.00	0.00	Solyc04g0	Solyc04g0	15.69	3.97
GO:00164	oxidoreduc	Molecular	0.00	0.00	Solyc04g0	Solyc04g0	39.10	5.29
GO:00164	oxidoreduc	Molecular	0.00	0.00	Solyc01g0	Solyc01g0	0.12	-3.03
GO:00164	oxidoreduc	Molecular	0.00	0.00	Solyc08g0	Solyc08g0	2.09	1.06
GO:00164	oxidoreduc	Molecular	0.00	0.00	Solyc07g0	Solyc07g0	9.07	3.18

GO:00164	oxidoreduc	Molecular	0.00	0.00	Solyc08g0	Solyc08g0	0.34	-1.54
GO:00164	oxidoreduc	Molecular	0.00	0.00	Solyc09g0	Solyc09g0	0.50	-1.01
GO:00164	oxidoreduc	Molecular	0.00	0.00	Solyc11g0	Solyc11g0	5.06	2.34
GO:00164	oxidoreduc	Molecular	0.00	0.00	Solyc09g0	Solyc09g0	0.06	-4.10
GO:00164	oxidoreduc	Molecular	0.00	0.00	Solyc08g0	Solyc08g0	5.12	2.36
GO:00164	oxidoreduc	Molecular	0.00	0.00	Solyc04g0	Solyc04g0	32.87	5.04
GO:00164	oxidoreduc	Molecular	0.00	0.00	Solyc04g0	Solyc04g0	2.31	1.21
GO:00164	oxidoreduc	Molecular	0.00	0.00	Solyc12g0	Solyc12g0	6.07	2.60
GO:00164	oxidoreduc	Molecular	0.00	0.00	Solyc02g0	Solyc02g0	8.45	3.08
GO:00164	oxidoreduc	Molecular	0.00	0.00	Solyc02g0	Solyc02g0	6.79	2.76
GO:00164	oxidoreduc	Molecular	0.00	0.00	Solyc01g0	Solyc01g0	3.28	1.71
GO:00164	oxidoreduc	Molecular	0.00	0.00	Solyc02g0	Solyc02g0	0.37	-1.45
GO:00164	oxidoreduc	Molecular	0.00	0.00	Solyc03g0	Solyc03g0	0.44	-1.19
GO:00164	oxidoreduc	Molecular	0.00	0.00	Solyc11g0	Solyc11g0	281.82	8.14
GO:00164	oxidoreduc	Molecular	0.00	0.00	Solyc01g1	Solyc01g1	4.58	2.20
GO:00164	oxidoreduc	Molecular	0.00	0.00	Solyc11g0	Solyc11g0	0.43	-1.23
GO:00164	oxidoreduc	Molecular	0.00	0.00	Solyc01g0	Solyc01g0	0.03	-4.98
GO:00164	oxidoreduc	Molecular	0.00	0.00	Solyc06g0	Solyc06g0	444.70	8.80
GO:00160	membrane	Cellular Co	0.00	0.00	Solyc04g0	Solyc04g0	0.18	-2.50
GO:00160	membrane	Cellular Co	0.00	0.00	Solyc08g0	Solyc08g0	0.14	-2.87
GO:00160	membrane	Cellular Co	0.00	0.00	Solyc07g0	Solyc07g0	4.14	2.05
GO:00160	membrane	Cellular Co	0.00	0.00	Solyc01g1	Solyc01g1	5.77	2.53
GO:00160	membrane	Cellular Co	0.00	0.00	Solyc04g0	Solyc04g0	2.70	1.43
GO:00160	membrane	Cellular Co	0.00	0.00	Solyc04g0	Solyc04g0	2.19	1.13
GO:00160	membrane	Cellular Co	0.00	0.00	Solyc11g0	Solyc11g0	8.62	3.11
GO:00160	membrane	Cellular Co	0.00	0.00	Solyc08g0	Solyc08g0	4.51	2.17
GO:00160	membrane	Cellular Co	0.00	0.00	Solyc12g0	Solyc12g0	4.94	2.30
GO:00160	membrane	Cellular Co	0.00	0.00	Solyc03g1	Solyc03g1	3.43	1.78
GO:00160	membrane	Cellular Co	0.00	0.00	Solyc04g0	Solyc04g0	0.35	-1.50
GO:00160	membrane	Cellular Co	0.00	0.00	Solyc01g0	Solyc01g0	2.01	1.01
GO:00160	membrane	Cellular Co	0.00	0.00	Solyc09g0	Solyc09g0	2.22	1.15
GO:00160	membrane	Cellular Co	0.00	0.00	Solyc11g0	AOS2	3.20	1.68
GO:00160	membrane	Cellular Co	0.00	0.00	Solyc03g1	Solyc03g1	2.44	1.28
GO:00160	membrane	Cellular Co	0.00	0.00	Solyc02g0	Solyc02g0	2.09	1.06
GO:00160	membrane	Cellular Co	0.00	0.00	Solyc01g1	Solyc01g1	0.29	-1.80
GO:00160	membrane	Cellular Co	0.00	0.00	Solyc07g0	Solyc07g0	4.02	2.01
GO:00160	membrane	Cellular Co	0.00	0.00	Solyc11g0	Solyc11g0	2.28	1.19
GO:00160	membrane	Cellular Co	0.00	0.00	Solyc02g0	Solyc02g0	0.34	-1.57
GO:00160	membrane	Cellular Co	0.00	0.00	Solyc02g0	Solyc02g0	0.50	-1.01
GO:00160	membrane	Cellular Co	0.00	0.00	Solyc04g0	Solyc04g0	2.89	1.53
GO:00160	membrane	Cellular Co	0.00	0.00	Solyc01g1	Solyc01g1	2.47	1.30
GO:00160	membrane	Cellular Co	0.00	0.00	Solyc05g0	Solyc05g0	4.77	2.25
GO:00160	membrane	Cellular Co	0.00	0.00	Solyc12g0	Solyc12g0	2.97	1.57
GO:00160	membrane	Cellular Co	0.00	0.00	Solyc06g0	Solyc06g0	2.40	1.26
GO:00160	membrane	Cellular Co	0.00	0.00	Solyc04g0	PETE	2.69	1.43
GO:00160	membrane	Cellular Co	0.00	0.00	Solyc09g0	Solyc09g0	4.52	2.18
GO:00160	membrane	Cellular Co	0.00	0.00	Solyc01g1	Solyc01g1	2.90	1.54
GO:00160	membrane	Cellular Co	0.00	0.00	Solyc07g0	Solyc07g0	2.63	1.40
GO:00160	membrane	Cellular Co	0.00	0.00	Solyc06g0	Solyc06g0	2.47	1.31
GO:00160	membrane	Cellular Co	0.00	0.00	Solyc06g0	Solyc06g0	16.51	4.04
GO:00160	membrane	Cellular Co	0.00	0.00	Solyc09g0	Solyc09g0	7.18	2.84

GO:00160	membrane	Cellular C	0.00	0.00	Solyc05g0	Solyc05g0	4.78	2.26
GO:00160	membrane	Cellular C	0.00	0.00	Solyc09g0	Solyc09g0	5.85	2.55
GO:00160	membrane	Cellular C	0.00	0.00	Solyc01g1	Solyc01g1	0.24	-2.06
GO:00160	membrane	Cellular C	0.00	0.00	Solyc03g1	Solyc03g1	0.30	-1.75
GO:00160	membrane	Cellular C	0.00	0.00	Solyc01g0	Solyc01g0	0.32	-1.65
GO:00160	membrane	Cellular C	0.00	0.00	Solyc05g0	Solyc05g0	2.41	1.27
GO:00160	membrane	Cellular C	0.00	0.00	Solyc03g0	Solyc03g0	0.31	-1.67
GO:00160	membrane	Cellular C	0.00	0.00	Solyc02g0	Solyc02g0	2.85	1.51
GO:00160	membrane	Cellular C	0.00	0.00	Solyc05g0	Solyc05g0	0.49	-1.03
GO:00160	membrane	Cellular C	0.00	0.00	Solyc10g0	Solyc10g0	3.31	1.73
GO:00160	membrane	Cellular C	0.00	0.00	Solyc02g0	Solyc02g0	0.44	-1.19
GO:00160	membrane	Cellular C	0.00	0.00	Solyc11g0	Solyc11g0	0.35	-1.51
GO:00160	membrane	Cellular C	0.00	0.00	Solyc12g0	Solyc12g0	5.32	2.41
GO:00160	membrane	Cellular C	0.00	0.00	Solyc02g0	Solyc02g0	0.45	-1.14
GO:00160	membrane	Cellular C	0.00	0.00	Solyc11g0	Solyc11g0	0.02	-5.38
GO:00160	membrane	Cellular C	0.00	0.00	Solyc04g0	Solyc04g0	0.38	-1.41
GO:00160	membrane	Cellular C	0.00	0.00	Solyc06g0	Solyc06g0	2.02	1.02
GO:00160	membrane	Cellular C	0.00	0.00	Solyc01g1	Solyc01g1	3.01	1.59
GO:00160	membrane	Cellular C	0.00	0.00	Solyc06g0	Solyc06g0	0.41	-1.30
GO:00160	membrane	Cellular C	0.00	0.00	Solyc12g0	Solyc12g0	2.10	1.07
GO:00160	membrane	Cellular C	0.00	0.00	Solyc03g1	Solyc03g1	0.25	-2.02
GO:00160	membrane	Cellular C	0.00	0.00	Solyc10g0	Solyc10g0	2.60	1.38
GO:00160	membrane	Cellular C	0.00	0.00	Solyc06g0	Solyc06g0	0.40	-1.31
GO:00160	membrane	Cellular C	0.00	0.00	Solyc10g0	Solyc10g0	2.56	1.36
GO:00160	membrane	Cellular C	0.00	0.00	Solyc05g0	Solyc05g0	0.08	-3.58
GO:00160	membrane	Cellular C	0.00	0.00	Solyc06g0	Solyc06g0	0.46	-1.11
GO:00160	membrane	Cellular C	0.00	0.00	Solyc07g0	Solyc07g0	3.79	1.92
GO:00160	membrane	Cellular C	0.00	0.00	Solyc11g0	Solyc11g0	0.24	-2.04
GO:00160	membrane	Cellular C	0.00	0.00	Solyc02g0	PSBO	5.02	2.33
GO:00160	membrane	Cellular C	0.00	0.00	Solyc11g0	Solyc11g0	0.49	-1.04
GO:00160	membrane	Cellular C	0.00	0.00	Solyc04g0	Solyc04g0	7.43	2.89
GO:00160	membrane	Cellular C	0.00	0.00	Solyc01g0	Solyc01g0	0.49	-1.04
GO:00160	membrane	Cellular C	0.00	0.00	Solyc10g0	Solyc10g0	2.36	1.24
GO:00160	membrane	Cellular C	0.00	0.00	Solyc09g0	Solyc09g0	0.18	-2.50
GO:00160	membrane	Cellular C	0.00	0.00	Solyc01g0	Solyc01g0	0.42	-1.26
GO:00160	membrane	Cellular C	0.00	0.00	Solyc09g0	Solyc09g0	0.36	-1.46
GO:00160	membrane	Cellular C	0.00	0.00	Solyc09g0	Solyc09g0	0.12	-3.08
GO:00160	membrane	Cellular C	0.00	0.00	Solyc07g0	PSBR	3.05	1.61
GO:00160	membrane	Cellular C	0.00	0.00	Solyc01g1	Solyc01g1	14.96	3.90
GO:00160	membrane	Cellular C	0.00	0.00	Solyc05g0	Solyc05g0	3.13	1.65
GO:00160	membrane	Cellular C	0.00	0.00	Solyc02g0	Solyc02g0	0.38	-1.39
GO:00160	membrane	Cellular C	0.00	0.00	Solyc09g0	Solyc09g0	0.47	-1.07
GO:00160	membrane	Cellular C	0.00	0.00	Solyc03g1	Solyc03g1	0.39	-1.35
GO:00160	membrane	Cellular C	0.00	0.00	Solyc10g0	Solyc10g0	4.66	2.22
GO:00160	membrane	Cellular C	0.00	0.00	Solyc10g0	Solyc10g0	40.50	5.34
GO:00160	membrane	Cellular C	0.00	0.00	Solyc04g0	Solyc04g0	0.26	-1.94
GO:00160	membrane	Cellular C	0.00	0.00	Solyc10g0	Solyc10g0	15621.48	13.93
GO:00160	membrane	Cellular C	0.00	0.00	Solyc08g0	Solyc08g0	4598.17	12.17
GO:00160	membrane	Cellular C	0.00	0.00	Solyc04g0	Solyc04g0	0.00	-11.98
GO:00160	membrane	Cellular C	0.00	0.00	Solyc12g0	Solyc12g0	2.35	1.23
GO:00160	membrane	Cellular C	0.00	0.00	Solyc10g0	Solyc10g0	2.02	1.01

GO:00160	membrane	Cellular C	0.00	0.00	Solyc11g0	Solyc11g0	0.00	-13.30
GO:00160	membrane	Cellular C	0.00	0.00	Solyc01g0	Solyc01g0	3528.16	11.78
GO:00160	membrane	Cellular C	0.00	0.00	Solyc03g1	Solyc03g1	0.44	-1.20
GO:00160	membrane	Cellular C	0.00	0.00	Solyc10g0	Solyc10g0	0.04	-4.70
GO:00160	membrane	Cellular C	0.00	0.00	Solyc03g1	Solyc03g1	0.33	-1.59
GO:00160	membrane	Cellular C	0.00	0.00	Solyc08g0	Solyc08g0	0.42	-1.27
GO:00160	membrane	Cellular C	0.00	0.00	Solyc09g0	Solyc09g0	51.95	5.70
GO:00160	membrane	Cellular C	0.00	0.00	Solyc01g0	Solyc01g0	6.50	2.70
GO:00160	membrane	Cellular C	0.00	0.00	Solyc03g0	Solyc03g0	0.04	-4.63
GO:00160	membrane	Cellular C	0.00	0.00	Solyc12g0	Solyc12g0	3.10	1.63
GO:00160	membrane	Cellular C	0.00	0.00	Solyc12g0	Solyc12g0	0.00	-11.26
GO:00160	membrane	Cellular C	0.00	0.00	Solyc03g1	Solyc03g1	0.48	-1.07
GO:00160	membrane	Cellular C	0.00	0.00	Solyc12g0	Solyc12g0	0.37	-1.44
GO:00160	membrane	Cellular C	0.00	0.00	Solyc12g0	Solyc12g0	0.05	-4.20
GO:00160	membrane	Cellular C	0.00	0.00	Solyc06g0	Solyc06g0	0.11	-3.25
GO:00160	membrane	Cellular C	0.00	0.00	Solyc02g0	Solyc02g0	4.67	2.22
GO:00160	membrane	Cellular C	0.00	0.00	Solyc04g0	Solyc04g0	0.00	-13.79
GO:00160	membrane	Cellular C	0.00	0.00	Solyc01g1	Solyc01g1	3.40	1.77
GO:00160	membrane	Cellular C	0.00	0.00	Solyc03g0	Solyc03g0	0.11	-3.15
GO:00160	membrane	Cellular C	0.00	0.00	Solyc10g0	Solyc10g0	0.00	-13.73
GO:00160	membrane	Cellular C	0.00	0.00	Solyc09g0	Solyc09g0	0.49	-1.04
GO:00160	membrane	Cellular C	0.00	0.00	Solyc04g0	Solyc04g0	0.41	-1.29
GO:00160	membrane	Cellular C	0.00	0.00	Solyc09g0	Solyc09g0	3.57	1.84
GO:00160	membrane	Cellular C	0.00	0.00	Solyc12g0	Solyc12g0	3.03	1.60
GO:00160	membrane	Cellular C	0.00	0.00	Solyc10g0	Solyc10g0	0.00	-12.09
GO:00160	membrane	Cellular C	0.00	0.00	Solyc12g0	Solyc12g0	2.66	1.41
GO:00160	membrane	Cellular C	0.00	0.00	Solyc02g0	Solyc02g0	2.28	1.19
GO:00160	membrane	Cellular C	0.00	0.00	Solyc01g0	Solyc01g0	1991.27	10.96
GO:00160	membrane	Cellular C	0.00	0.00	Solyc01g1	Solyc01g1	4.37	2.13
GO:00160	membrane	Cellular C	0.00	0.00	Solyc09g0	Solyc09g0	0.00	-13.14
GO:00160	membrane	Cellular C	0.00	0.00	Solyc01g0	Solyc01g0	3.53	1.82
GO:00160	membrane	Cellular C	0.00	0.00	Solyc01g1	Solyc01g1	2829.11	11.47
GO:00160	membrane	Cellular C	0.00	0.00	Solyc10g0	Solyc10g0	0.00	-12.76
GO:00160	membrane	Cellular C	0.00	0.00	Solyc01g1	Solyc01g1	0.20	-2.35
GO:00160	membrane	Cellular C	0.00	0.00	Solyc06g0	Solyc06g0	0.05	-4.46
GO:00160	membrane	Cellular C	0.00	0.00	Solyc11g0	Solyc11g0	0.00	-13.08
GO:00160	membrane	Cellular C	0.00	0.00	Solyc09g0	Solyc09g0	0.00	-13.63
GO:00160	membrane	Cellular C	0.00	0.00	Solyc01g0	Solyc01g0	3.64	1.87
GO:00160	membrane	Cellular C	0.00	0.00	Solyc10g0	Solyc10g0	0.03	-5.30
GO:00160	membrane	Cellular C	0.00	0.00	Solyc06g0	Solyc06g0	0.00	-9.49
GO:00160	membrane	Cellular C	0.00	0.00	Solyc06g0	Solyc06g0	0.00	-10.22
GO:00160	membrane	Cellular C	0.00	0.00	Solyc08g0	Solyc08g0	0.29	-1.80
GO:00160	membrane	Cellular C	0.00	0.00	Solyc10g0	Solyc10g0	2.07	1.05
GO:00160	membrane	Cellular C	0.00	0.00	Solyc08g0	Solyc08g0	0.47	-1.08
GO:00160	membrane	Cellular C	0.00	0.00	Solyc10g0	Solyc10g0	4.22	2.08
GO:00160	membrane	Cellular C	0.00	0.00	Solyc03g0	Solyc03g0	1336.08	10.38
GO:00160	membrane	Cellular C	0.00	0.00	Solyc01g0	Solyc01g0	0.02	-5.72
GO:00160	membrane	Cellular C	0.00	0.00	Solyc06g0	PSBS	2.99	1.58
GO:00160	membrane	Cellular C	0.00	0.00	Solyc01g0	Solyc01g0	0.19	-2.40
GO:00160	membrane	Cellular C	0.00	0.00	Solyc08g0	Solyc08g0	1235.43	10.27
GO:00160	membrane	Cellular C	0.00	0.00	Solyc10g0	Solyc10g0	0.37	-1.43

GO:00160	membrane	Cellular Co	0.00	0.00	Solyc04g0	Solyc04g0	3.05	1.61
GO:00160	membrane	Cellular Co	0.00	0.00	Solyc07g0	Solyc07g0	2.47	1.30
GO:00160	membrane	Cellular Co	0.00	0.00	Solyc12g1	Solyc12g1	249.94	7.97
GO:00160	membrane	Cellular Co	0.00	0.00	Solyc03g0	Solyc03g0	2.60	1.38
GO:00160	membrane	Cellular Co	0.00	0.00	Solyc01g0	Solyc01g0	36.75	5.20
GO:00160	membrane	Cellular Co	0.00	0.00	Solyc04g0	Solyc04g0	900.23	9.81
GO:00160	membrane	Cellular Co	0.00	0.00	Solyc12g0	Solyc12g0	0.47	-1.10
GO:00160	membrane	Cellular Co	0.00	0.00	Solyc06g0	Solyc06g0	17.36	4.12
GO:00160	membrane	Cellular Co	0.00	0.00	Solyc02g0	Solyc02g0	19.78	4.31
GO:00160	membrane	Cellular Co	0.00	0.00	Solyc12g0	Solyc12g0	0.45	-1.17
GO:00160	membrane	Cellular Co	0.00	0.00	Solyc10g0	Solyc10g0	0.03	-5.05
GO:00160	membrane	Cellular Co	0.00	0.00	Solyc11g0	Solyc11g0	0.00	-11.68
GO:00160	membrane	Cellular Co	0.00	0.00	Solyc09g0	Solyc09g0	2.82	1.50
GO:00160	membrane	Cellular Co	0.00	0.00	Solyc03g0	Solyc03g0	2.40	1.26
GO:00160	membrane	Cellular Co	0.00	0.00	Solyc03g0	Solyc03g0	3.69	1.88
GO:00160	membrane	Cellular Co	0.00	0.00	Solyc01g0	Solyc01g0	894.88	9.81
GO:00160	membrane	Cellular Co	0.00	0.00	Solyc03g1	Solyc03g1	0.10	-3.25
GO:00160	membrane	Cellular Co	0.00	0.00	Solyc07g0	Solyc07g0	3.48	1.80
GO:00160	membrane	Cellular Co	0.00	0.00	Solyc10g0	Solyc10g0	0.43	-1.23
GO:00160	membrane	Cellular Co	0.00	0.00	Solyc02g0	Solyc02g0	0.19	-2.37
GO:00160	membrane	Cellular Co	0.00	0.00	Solyc12g0	Solyc12g0	0.25	-2.01
GO:00160	membrane	Cellular Co	0.00	0.00	Solyc05g0	Solyc05g0	2.42	1.27
GO:00160	membrane	Cellular Co	0.00	0.00	Solyc01g0	Solyc01g0	0.00	-11.99
GO:00160	membrane	Cellular Co	0.00	0.00	Solyc09g0	Solyc09g0	429.36	8.75
GO:00160	membrane	Cellular Co	0.00	0.00	Solyc11g0	Solyc11g0	0.23	-2.12
GO:00160	membrane	Cellular Co	0.00	0.00	Solyc05g0	Solyc05g0	1370.75	10.42
GO:00160	membrane	Cellular Co	0.00	0.00	Solyc02g0	Solyc02g0	307.12	8.26
GO:00160	membrane	Cellular Co	0.00	0.00	Solyc02g0	Solyc02g0	3.18	1.67
GO:00160	membrane	Cellular Co	0.00	0.00	Solyc03g1	Solyc03g1	0.15	-2.75
GO:00160	membrane	Cellular Co	0.00	0.00	Solyc07g0	Solyc07g0	0.40	-1.33
GO:00160	membrane	Cellular Co	0.00	0.00	Solyc02g0	Solyc02g0	0.43	-1.21
GO:00160	membrane	Cellular Co	0.00	0.00	Solyc07g0	Solyc07g0	9.07	3.18
GO:00160	membrane	Cellular Co	0.00	0.00	Solyc11g0	Solyc11g0	0.00	-9.24
GO:00160	membrane	Cellular Co	0.00	0.00	Solyc12g0	Solyc12g0	0.00	-9.92
GO:00160	membrane	Cellular Co	0.00	0.00	Solyc07g0	Solyc07g0	714.98	9.48
GO:00160	membrane	Cellular Co	0.00	0.00	Solyc06g0	Solyc06g0	0.32	-1.62
GO:00160	membrane	Cellular Co	0.00	0.00	Solyc11g0	Solyc11g0	0.10	-3.29
GO:00160	membrane	Cellular Co	0.00	0.00	Solyc02g0	Solyc02g0	0.17	-2.58
GO:00160	membrane	Cellular Co	0.00	0.00	Solyc08g0	Solyc08g0	0.34	-1.54
GO:00160	membrane	Cellular Co	0.00	0.00	Solyc07g0	Solyc07g0	401.43	8.65
GO:00160	membrane	Cellular Co	0.00	0.00	Solyc04g0	Solyc04g0	0.06	-3.97
GO:00160	membrane	Cellular Co	0.00	0.00	Solyc09g0	Solyc09g0	3.37	1.75
GO:00160	membrane	Cellular Co	0.00	0.00	Solyc07g0	Solyc07g0	2.39	1.26
GO:00160	membrane	Cellular Co	0.00	0.00	Solyc05g0	Solyc05g0	9.14	3.19
GO:00160	membrane	Cellular Co	0.00	0.00	Solyc01g1	psbZ	0.17	-2.52
GO:00160	membrane	Cellular Co	0.00	0.00	Solyc08g0	Solyc08g0	13.23	3.73
GO:00160	membrane	Cellular Co	0.00	0.00	Solyc11g0	Solyc11g0	0.09	-3.49
GO:00160	membrane	Cellular Co	0.00	0.00	Solyc11g0	Solyc11g0	0.03	-5.20
GO:00160	membrane	Cellular Co	0.00	0.00	Solyc04g0	Solyc04g0	7.72	2.95
GO:00160	membrane	Cellular Co	0.00	0.00	Solyc12g0	Solyc12g0	369.30	8.53
GO:00160	membrane	Cellular Co	0.00	0.00	Solyc11g0	Solyc11g0	0.22	-2.19

GO:00160	membrane	Cellular Co	0.00	0.00	Solyc01g0	Solyc01g0	0.08	-3.58
GO:00160	membrane	Cellular Co	0.00	0.00	Solyc03g1	Solyc03g1	356.83	8.48
GO:00160	membrane	Cellular Co	0.00	0.00	Solyc03g0	Solyc03g0	11.28	3.50
GO:00160	membrane	Cellular Co	0.00	0.00	Solyc09g0	Solyc09g0	0.49	-1.03
GO:00160	membrane	Cellular Co	0.00	0.00	Solyc11g0	Solyc11g0	0.09	-3.52
GO:00160	membrane	Cellular Co	0.00	0.00	Solyc08g0	Solyc08g0	599.38	9.23
GO:00160	membrane	Cellular Co	0.00	0.00	Solyc06g0	Solyc06g0	13.67	3.77
GO:00160	membrane	Cellular Co	0.00	0.00	Solyc10g0	Solyc10g0	0.06	-4.16
GO:00160	membrane	Cellular Co	0.00	0.00	Solyc12g0	Solyc12g0	4.99	2.32
GO:00160	membrane	Cellular Co	0.00	0.00	Solyc11g0	Solyc11g0	0.00	-10.82
GO:00160	membrane	Cellular Co	0.00	0.00	Solyc03g0	Solyc03g0	0.00	-9.99
GO:00160	membrane	Cellular Co	0.00	0.00	Solyc08g0	Solyc08g0	2.51	1.33
GO:00160	membrane	Cellular Co	0.00	0.00	Solyc09g0	Solyc09g0	0.00	-8.11
GO:00160	membrane	Cellular Co	0.00	0.00	Solyc06g0	Solyc06g0	0.44	-1.17
GO:00160	membrane	Cellular Co	0.00	0.00	Solyc10g0	Solyc10g0	0.00	-9.79
GO:00160	membrane	Cellular Co	0.00	0.00	Solyc11g0	Solyc11g0	0.43	-1.20
GO:00160	membrane	Cellular Co	0.00	0.00	Solyc09g0	Solyc09g0	0.38	-1.39
GO:00160	membrane	Cellular Co	0.00	0.00	Solyc01g0	Solyc01g0	0.00	-11.62
GO:00160	membrane	Cellular Co	0.00	0.00	Solyc11g0	Solyc11g0	0.30	-1.71
GO:00160	membrane	Cellular Co	0.00	0.00	Solyc03g1	Solyc03g1	0.00	-8.90
GO:00160	membrane	Cellular Co	0.00	0.00	Solyc03g0	Solyc03g0	4.00	2.00
GO:00160	membrane	Cellular Co	0.00	0.00	Solyc11g0	Solyc11g0	292.92	8.19
GO:00160	membrane	Cellular Co	0.00	0.00	Solyc09g0	Solyc09g0	0.44	-1.19
GO:00095	photosyste	Cellular Co	0.00	0.00	Solyc09g0	Solyc09g0	2.22	1.15
GO:00095	photosyste	Cellular Co	0.00	0.00	Solyc07g0	Solyc07g0	4.02	2.01
GO:00095	photosyste	Cellular Co	0.00	0.00	Solyc10g0	Solyc10g0	2.93	1.55
GO:00095	photosyste	Cellular Co	0.00	0.00	Solyc09g0	Solyc09g0	7.18	2.84
GO:00095	photosyste	Cellular Co	0.00	0.00	Solyc09g0	Solyc09g0	5.85	2.55
GO:00095	photosyste	Cellular Co	0.00	0.00	Solyc02g0	PSBO	5.02	2.33
GO:00095	photosyste	Cellular Co	0.00	0.00	Solyc07g0	PSBR	3.05	1.61
GO:00095	photosyste	Cellular Co	0.00	0.00	Solyc10g0	Solyc10g0	4.66	2.22
GO:00095	photosyste	Cellular Co	0.00	0.00	Solyc09g0	Solyc09g0	4.23	2.08
GO:00095	photosyste	Cellular Co	0.00	0.00	Solyc09g0	Solyc09g0	4.92	2.30
GO:00095	photosyste	Cellular Co	0.00	0.00	Solyc02g0	Solyc02g0	3.41	1.77
GO:00095	photosyste	Cellular Co	0.00	0.00	Solyc09g0	Solyc09g0	0.00	-13.14
GO:00095	photosyste	Cellular Co	0.00	0.00	Solyc09g0	Solyc09g0	0.00	-13.63
GO:00095	photosyste	Cellular Co	0.00	0.00	Solyc10g0	Solyc10g0	2.07	1.05
GO:00095	photosyste	Cellular Co	0.00	0.00	Solyc06g0	PSBS	2.99	1.58
GO:00095	photosyste	Cellular Co	0.00	0.00	Solyc03g0	Solyc03g0	2.60	1.38
GO:00095	photosyste	Cellular Co	0.00	0.00	Solyc03g0	Solyc03g0	3.69	1.88
GO:00095	photosyste	Cellular Co	0.00	0.00	Solyc12g0	Solyc12g0	0.25	-2.01
GO:00095	photosyste	Cellular Co	0.00	0.00	Solyc01g1	psbZ	0.17	-2.52
GO:00095	photosyste	Cellular Co	0.00	0.00	Solyc04g0	Solyc04g0	5.19	2.37
GO:00095	photosyste	Cellular Co	0.00	0.00	Solyc03g1	Solyc03g1	2.21	1.14
GO:00095	photosyste	Cellular Co	0.00	0.00	Solyc05g0	Solyc05g0	0.01	-6.09
GO:00095	photosyste	Cellular Co	0.00	0.00	Solyc09g0	Solyc09g0	5.85	2.55
GO:00095	photosyste	Cellular Co	0.00	0.00	Solyc02g0	Solyc02g0	2.31	1.21
GO:00095	photosyste	Cellular Co	0.00	0.00	Solyc10g0	Solyc10g0	4.66	2.22
GO:00095	photosyste	Cellular Co	0.00	0.00	Solyc12g0	Solyc12g0	0.00	-11.26
GO:00095	photosyste	Cellular Co	0.00	0.00	Solyc05g0	Solyc05g0	0.00	-13.36
GO:00095	photosyste	Cellular Co	0.00	0.00	Solyc04g0	Solyc04g0	0.00	-13.79

GO:00095	photosyste	Cellular Co	0.00	0.00	Solyc02g0	Solyc02g0	0.03	-4.99
GO:00095	photosyste	Cellular Co	0.00	0.00	Solyc10g0	Solyc10g0	0.03	-5.30
GO:00095	photosyste	Cellular Co	0.00	0.00	Solyc10g0	Solyc10g0	2.07	1.05
GO:00095	photosyste	Cellular Co	0.00	0.00	Solyc07g0	Solyc07g0	2.47	1.30
GO:00095	photosyste	Cellular Co	0.00	0.00	Solyc03g0	Solyc03g0	2.60	1.38
GO:00095	photosyste	Cellular Co	0.00	0.00	Solyc03g0	Solyc03g0	3.69	1.88
GO:00095	photosyste	Cellular Co	0.00	0.00	Solyc12g0	Solyc12g0	0.25	-2.01
GO:00095	photosyste	Cellular Co	0.00	0.00	Solyc07g0	Solyc07g0	0.00	-13.48
GO:00095	photosyste	Cellular Co	0.00	0.00	Solyc08g0	Solyc08g0	1537.41	10.59
GO:00095	photosyste	Cellular Co	0.00	0.00	Solyc06g0	Solyc06g0	4.49	2.17
GO:00095	photosyste	Cellular Co	0.00	0.00	Solyc06g0	Solyc06g0	0.12	-3.11
GO:00196	photosynth	Biological	0.00	0.00	Solyc05g0	Solyc05g0	0.01	-6.09
GO:00196	photosynth	Biological	0.00	0.00	Solyc01g0	Solyc01g0	0.01	-6.32
GO:00196	photosynth	Biological	0.00	0.00	Solyc12g0	Solyc12g0	0.01	-7.31
GO:00196	photosynth	Biological	0.00	0.00	Solyc02g0	Solyc02g0	0.01	-6.27
GO:00196	photosynth	Biological	0.00	0.00	Solyc09g0	Solyc09g0	0.18	-2.50
GO:00196	photosynth	Biological	0.00	0.00	Solyc12g0	Solyc12g0	0.05	-4.20
GO:00196	photosynth	Biological	0.00	0.00	Solyc03g0	Solyc03g0	0.11	-3.15
GO:00196	photosynth	Biological	0.00	0.00	Solyc10g0	Solyc10g0	0.00	-12.09
GO:00196	photosynth	Biological	0.00	0.00	Solyc09g0	Solyc09g0	0.00	-13.14
GO:00196	photosynth	Biological	0.00	0.00	Solyc06g0	Solyc06g0	0.05	-4.46
GO:00196	photosynth	Biological	0.00	0.00	Solyc11g0	Solyc11g0	0.00	-13.08
GO:00196	photosynth	Biological	0.00	0.00	Solyc09g0	Solyc09g0	0.00	-13.63
GO:00196	photosynth	Biological	0.00	0.00	Solyc02g0	Solyc02g0	0.19	-2.37
GO:00196	photosynth	Biological	0.00	0.00	Solyc05g0	Solyc05g0	0.00	-12.71
GO:00196	photosynth	Biological	0.00	0.00	Solyc02g0	Solyc02g0	0.43	-1.21
GO:00196	photosynth	Biological	0.00	0.00	Solyc11g0	Solyc11g0	0.00	-12.69
GO:00196	photosynth	Biological	0.00	0.00	Solyc01g0	Solyc01g0	0.00	-11.62
GO:00468	metal ion t	Molecular	0.00	0.00	Solyc07g0	ACO1	2.82	1.49
GO:00468	metal ion t	Molecular	0.00	0.00	Solyc08g0	Solyc08g0	0.14	-2.87
GO:00468	metal ion t	Molecular	0.00	0.00	Solyc07g0	Solyc07g0	0.35	-1.51
GO:00468	metal ion t	Molecular	0.00	0.00	Solyc02g0	Solyc02g0	0.08	-3.73
GO:00468	metal ion t	Molecular	0.00	0.00	Solyc01g0	Solyc01g0	2.18	1.13
GO:00468	metal ion t	Molecular	0.00	0.00	Solyc11g0	Solyc11g0	3.25	1.70
GO:00468	metal ion t	Molecular	0.00	0.00	Solyc11g0	Solyc11g0	0.28	-1.84
GO:00468	metal ion t	Molecular	0.00	0.00	Solyc10g0	Solyc10g0	65.27	6.03
GO:00468	metal ion t	Molecular	0.00	0.00	Solyc01g0	Solyc01g0	0.31	-1.67
GO:00468	metal ion t	Molecular	0.00	0.00	Solyc11g0	AOS2	3.20	1.68
GO:00468	metal ion t	Molecular	0.00	0.00	Solyc03g0	Solyc03g0	22.76	4.51
GO:00468	metal ion t	Molecular	0.00	0.00	Solyc01g0	Solyc01g0	0.03	-5.12
GO:00468	metal ion t	Molecular	0.00	0.00	Solyc06g0	Solyc06g0	0.29	-1.78
GO:00468	metal ion t	Molecular	0.00	0.00	Solyc03g1	Solyc03g1	0.35	-1.54
GO:00468	metal ion t	Molecular	0.00	0.00	Solyc08g0	LOX1.1	2.60	1.38
GO:00468	metal ion t	Molecular	0.00	0.00	Solyc07g0	Solyc07g0	2.20	1.14
GO:00468	metal ion t	Molecular	0.00	0.00	Solyc05g0	Solyc05g0	0.46	-1.12
GO:00468	metal ion t	Molecular	0.00	0.00	Solyc08g0	Solyc08g0	3.48	1.80
GO:00468	metal ion t	Molecular	0.00	0.00	Solyc03g0	Solyc03g0	0.42	-1.27
GO:00468	metal ion t	Molecular	0.00	0.00	Solyc03g1	Solyc03g1	2.22	1.15
GO:00468	metal ion t	Molecular	0.00	0.00	Solyc04g0	PETE	2.69	1.43
GO:00468	metal ion t	Molecular	0.00	0.00	Solyc09g0	Solyc09g0	3.61	1.85
GO:00468	metal ion t	Molecular	0.00	0.00	Solyc06g0	Solyc06g0	0.18	-2.44

GO:00468	metal ion t	Molecular	0.00	0.00	Solyc12g0	Solyc12g0	2.04	1.03
GO:00468	metal ion t	Molecular	0.00	0.00	Solyc03g1	Solyc03g1	0.30	-1.75
GO:00468	metal ion t	Molecular	0.00	0.00	Solyc12g0	Solyc12g0	0.37	-1.43
GO:00468	metal ion t	Molecular	0.00	0.00	Solyc03g1	Solyc03g1	0.46	-1.13
GO:00468	metal ion t	Molecular	0.00	0.00	Solyc07g0	Solyc07g0	2.20	1.14
GO:00468	metal ion t	Molecular	0.00	0.00	Solyc06g0	Solyc06g0	19.10	4.26
GO:00468	metal ion t	Molecular	0.00	0.00	Solyc07g0	PDF1A	4.20	2.07
GO:00468	metal ion t	Molecular	0.00	0.00	Solyc10g0	Solyc10g0	2.91	1.54
GO:00468	metal ion t	Molecular	0.00	0.00	Solyc05g0	Solyc05g0	3.20	1.68
GO:00468	metal ion t	Molecular	0.00	0.00	Solyc05g0	Solyc05g0	0.08	-3.58
GO:00468	metal ion t	Molecular	0.00	0.00	Solyc06g0	Solyc06g0	0.46	-1.11
GO:00468	metal ion t	Molecular	0.00	0.00	Solyc05g0	Solyc05g0	2.24	1.16
GO:00468	metal ion t	Molecular	0.00	0.00	Solyc03g0	Solyc03g0	3.90	1.96
GO:00468	metal ion t	Molecular	0.00	0.00	Solyc10g0	Solyc10g0	0.43	-1.22
GO:00468	metal ion t	Molecular	0.00	0.00	Solyc06g0	Solyc06g0	0.15	-2.75
GO:00468	metal ion t	Molecular	0.00	0.00	Solyc02g0	Solyc02g0	2.56	1.36
GO:00468	metal ion t	Molecular	0.00	0.00	Solyc06g0	Solyc06g0	4.45	2.15
GO:00468	metal ion t	Molecular	0.00	0.00	Solyc11g0	Solyc11g0	0.32	-1.67
GO:00468	metal ion t	Molecular	0.00	0.00	Solyc07g0	Solyc07g0	2.09	1.06
GO:00468	metal ion t	Molecular	0.00	0.00	Solyc10g0	Solyc10g0	4.66	2.22
GO:00468	metal ion t	Molecular	0.00	0.00	Solyc09g0	Solyc09g0	0.20	-2.33
GO:00468	metal ion t	Molecular	0.00	0.00	Solyc11g0	Solyc11g0	2.20	1.14
GO:00468	metal ion t	Molecular	0.00	0.00	Solyc04g0	Solyc04g0	0.00	-13.91
GO:00468	metal ion t	Molecular	0.00	0.00	Solyc06g0	Solyc06g0	0.14	-2.82
GO:00468	metal ion t	Molecular	0.00	0.00	Solyc06g0	Solyc06g0	2.54	1.35
GO:00468	metal ion t	Molecular	0.00	0.00	Solyc11g0	Solyc11g0	5.04	2.33
GO:00468	metal ion t	Molecular	0.00	0.00	Solyc08g0	Solyc08g0	0.00	-12.86
GO:00468	metal ion t	Molecular	0.00	0.00	Solyc02g0	Solyc02g0	4235.90	12.05
GO:00468	metal ion t	Molecular	0.00	0.00	Solyc06g0	Solyc06g0	3.98	1.99
GO:00468	metal ion t	Molecular	0.00	0.00	Solyc03g0	Solyc03g0	5511.05	12.43
GO:00468	metal ion t	Molecular	0.00	0.00	Solyc07g0	Solyc07g0	40.33	5.33
GO:00468	metal ion t	Molecular	0.00	0.00	Solyc12g0	Solyc12g0	0.00	-11.26
GO:00468	metal ion t	Molecular	0.00	0.00	Solyc03g1	Solyc03g1	0.48	-1.07
GO:00468	metal ion t	Molecular	0.00	0.00	Solyc08g0	Solyc08g0	2.67	1.41
GO:00468	metal ion t	Molecular	0.00	0.00	Solyc01g1	Solyc01g1	2993.63	11.55
GO:00468	metal ion t	Molecular	0.00	0.00	Solyc09g0	Solyc09g0	0.47	-1.10
GO:00468	metal ion t	Molecular	0.00	0.00	Solyc06g0	Solyc06g0	2266.68	11.15
GO:00468	metal ion t	Molecular	0.00	0.00	Solyc11g0	Solyc11g0	3143.94	11.62
GO:00468	metal ion t	Molecular	0.00	0.00	Solyc07g0	Solyc07g0	4.00	2.00
GO:00468	metal ion t	Molecular	0.00	0.00	Solyc04g0	Solyc04g0	0.41	-1.30
GO:00468	metal ion t	Molecular	0.00	0.00	Solyc03g0	Solyc03g0	0.11	-3.15
GO:00468	metal ion t	Molecular	0.00	0.00	Solyc10g0	Solyc10g0	0.00	-13.68
GO:00468	metal ion t	Molecular	0.00	0.00	Solyc12g0	Solyc12g0	2.73	1.45
GO:00468	metal ion t	Molecular	0.00	0.00	Solyc07g0	Solyc07g0	2.37	1.24
GO:00468	metal ion t	Molecular	0.00	0.00	Solyc07g0	Solyc07g0	4.69	2.23
GO:00468	metal ion t	Molecular	0.00	0.00	Solyc10g0	Solyc10g0	2907.35	11.51
GO:00468	metal ion t	Molecular	0.00	0.00	Solyc01g1	Solyc01g1	4.37	2.13
GO:00468	metal ion t	Molecular	0.00	0.00	Solyc09g0	Solyc09g0	0.00	-13.14
GO:00468	metal ion t	Molecular	0.00	0.00	Solyc05g0	Solyc05g0	39.90	5.32
GO:00468	metal ion t	Molecular	0.00	0.00	Solyc06g0	Solyc06g0	3.27	1.71
GO:00468	metal ion t	Molecular	0.00	0.00	Solyc06g0	Solyc06g0	0.05	-4.46

GO:00468	metal ion b	Molecular	0.00	0.00	Solyc08g0	Solyc08g0	0.03	-5.23
GO:00468	metal ion b	Molecular	0.00	0.00	Solyc10g0	Solyc10g0	0.03	-5.30
GO:00468	metal ion b	Molecular	0.00	0.00	Solyc03g0	Solyc03g0	3.99	2.00
GO:00468	metal ion b	Molecular	0.00	0.00	Solyc10g0	Solyc10g0	2.07	1.05
GO:00468	metal ion b	Molecular	0.00	0.00	Solyc07g0	Solyc07g0	29.42	4.88
GO:00468	metal ion b	Molecular	0.00	0.00	Solyc01g0	Solyc01g0	0.02	-5.72
GO:00468	metal ion b	Molecular	0.00	0.00	Solyc12g0	Solyc12g0	1818.26	10.83
GO:00468	metal ion b	Molecular	0.00	0.00	Solyc08g0	Solyc08g0	1235.43	10.27
GO:00468	metal ion b	Molecular	0.00	0.00	Solyc08g0	Solyc08g0	8.90	3.15
GO:00468	metal ion b	Molecular	0.00	0.00	Solyc03g0	Solyc03g0	2.60	1.38
GO:00468	metal ion b	Molecular	0.00	0.00	Solyc09g0	Solyc09g0	1048.85	10.03
GO:00468	metal ion b	Molecular	0.00	0.00	Solyc03g0	Solyc03g0	3.69	1.88
GO:00468	metal ion b	Molecular	0.00	0.00	Solyc04g0	Solyc04g0	39.10	5.29
GO:00468	metal ion b	Molecular	0.00	0.00	Solyc11g0	Solyc11g0	856.16	9.74
GO:00468	metal ion b	Molecular	0.00	0.00	Solyc06g0	Solyc06g0	0.25	-2.01
GO:00468	metal ion b	Molecular	0.00	0.00	Solyc04g0	Solyc04g0	2.37	1.25
GO:00468	metal ion b	Molecular	0.00	0.00	Solyc12g0	Solyc12g0	0.39	-1.35
GO:00468	metal ion b	Molecular	0.00	0.00	Solyc07g0	Solyc07g0	9.07	3.18
GO:00468	metal ion b	Molecular	0.00	0.00	Solyc08g0	Solyc08g0	0.34	-1.54
GO:00468	metal ion b	Molecular	0.00	0.00	Solyc12g0	Solyc12g0	12.70	3.67
GO:00468	metal ion b	Molecular	0.00	0.00	Solyc09g0	Solyc09g0	0.50	-1.01
GO:00468	metal ion b	Molecular	0.00	0.00	Solyc04g0	Solyc04g0	0.39	-1.37
GO:00468	metal ion b	Molecular	0.00	0.00	Solyc01g0	Solyc01g0	902.52	9.82
GO:00468	metal ion b	Molecular	0.00	0.00	Solyc06g0	Solyc06g0	0.45	-1.14
GO:00468	metal ion b	Molecular	0.00	0.00	Solyc03g0	Solyc03g0	2.19	1.13
GO:00468	metal ion b	Molecular	0.00	0.00	Solyc09g0	Solyc09g0	529.32	9.05
GO:00468	metal ion b	Molecular	0.00	0.00	Solyc07g0	Solyc07g0	11.18	3.48
GO:00468	metal ion b	Molecular	0.00	0.00	Solyc11g0	Solyc11g0	0.09	-3.52
GO:00468	metal ion b	Molecular	0.00	0.00	Solyc12g0	Solyc12g0	4.99	2.32
GO:00468	metal ion b	Molecular	0.00	0.00	Solyc06g0	Solyc06g0	17.92	4.16
GO:00468	metal ion b	Molecular	0.00	0.00	Solyc01g0	Solyc01g0	0.05	-4.42
GO:00468	metal ion b	Molecular	0.00	0.00	Solyc02g0	Solyc02g0	27.11	4.76
GO:00468	metal ion b	Molecular	0.00	0.00	Solyc06g0	Solyc06g0	12.77	3.67
GO:00468	metal ion b	Molecular	0.00	0.00	Solyc06g0	Solyc06g0	0.12	-3.11
GO:00468	metal ion b	Molecular	0.00	0.00	Solyc07g0	Solyc07g0	470.92	8.88
GO:00468	metal ion b	Molecular	0.00	0.00	Solyc06g0	Solyc06g0	444.70	8.80
GO:00468	metal ion b	Molecular	0.00	0.00	Solyc07g0	Solyc07g0	2.87	1.52
GO:00055	iron ion bi	Molecular	0.00	0.00	Solyc03g0	Solyc03g0	7.65	2.93
GO:00055	iron ion bi	Molecular	0.00	0.00	Solyc11g0	AOS2	3.20	1.68
GO:00055	iron ion bi	Molecular	0.00	0.00	Solyc04g0	Solyc04g0	2.89	1.53
GO:00055	iron ion bi	Molecular	0.00	0.00	Solyc07g0	Solyc07g0	0.32	-1.63
GO:00055	iron ion bi	Molecular	0.00	0.00	Solyc12g0	Solyc12g0	2.35	1.23
GO:00055	iron ion bi	Molecular	0.00	0.00	Solyc10g0	Solyc10g0	0.40	-1.34
GO:00055	iron ion bi	Molecular	0.00	0.00	Solyc03g1	Solyc03g1	0.30	-1.75
GO:00055	iron ion bi	Molecular	0.00	0.00	Solyc06g0	Solyc06g0	3.21	1.68
GO:00055	iron ion bi	Molecular	0.00	0.00	Solyc06g0	Solyc06g0	7.48	2.90
GO:00055	iron ion bi	Molecular	0.00	0.00	Solyc03g1	Solyc03g1	4.61	2.21
GO:00055	iron ion bi	Molecular	0.00	0.00	Solyc04g0	Solyc04g0	6.45	2.69
GO:00055	iron ion bi	Molecular	0.00	0.00	Solyc01g1	Solyc01g1	0.03	-5.02
GO:00055	iron ion bi	Molecular	0.00	0.00	Solyc01g0	Solyc01g0	2.45	1.29
GO:00055	iron ion bi	Molecular	0.00	0.00	Solyc05g0	Solyc05g0	2.81	1.49

GO:00055	iron ion bi	Molecular	0.00	0.00	Solyc05g0	Solyc05g0	0.08	-3.58
GO:00055	iron ion bi	Molecular	0.00	0.00	Solyc08g0	Solyc08g0	2.39	1.25
GO:00055	iron ion bi	Molecular	0.00	0.00	Solyc11g0	Solyc11g0	0.32	-1.67
GO:00055	iron ion bi	Molecular	0.00	0.00	Solyc01g0	Solyc01g0	5.13	2.36
GO:00055	iron ion bi	Molecular	0.00	0.00	Solyc08g0	Solyc08g0	4598.17	12.17
GO:00055	iron ion bi	Molecular	0.00	0.00	Solyc01g0	Solyc01g0	3528.16	11.78
GO:00055	iron ion bi	Molecular	0.00	0.00	Solyc09g0	Solyc09g0	51.95	5.70
GO:00055	iron ion bi	Molecular	0.00	0.00	Solyc08g0	Solyc08g0	4039.84	11.98
GO:00055	iron ion bi	Molecular	0.00	0.00	Solyc05g0	Solyc05g0	2.96	1.57
GO:00055	iron ion bi	Molecular	0.00	0.00	Solyc03g0	Solyc03g0	0.11	-3.15
GO:00055	iron ion bi	Molecular	0.00	0.00	Solyc01g0	Solyc01g0	3.21	1.68
GO:00055	iron ion bi	Molecular	0.00	0.00	Solyc09g0	Solyc09g0	0.00	-13.14
GO:00055	iron ion bi	Molecular	0.00	0.00	Solyc02g0	Solyc02g0	0.48	-1.05
GO:00055	iron ion bi	Molecular	0.00	0.00	Solyc04g0	Solyc04g0	0.45	-1.16
GO:00055	iron ion bi	Molecular	0.00	0.00	Solyc06g0	Solyc06g0	0.05	-4.46
GO:00055	iron ion bi	Molecular	0.00	0.00	Solyc06g0	Solyc06g0	0.00	-10.22
GO:00055	iron ion bi	Molecular	0.00	0.00	Solyc01g0	Solyc01g0	0.02	-5.72
GO:00055	iron ion bi	Molecular	0.00	0.00	Solyc07g0	Solyc07g0	2.07	1.05
GO:00055	iron ion bi	Molecular	0.00	0.00	Solyc08g0	Solyc08g0	1235.43	10.27
GO:00055	iron ion bi	Molecular	0.00	0.00	Solyc12g1	Solyc12g1	0.00	-10.11
GO:00055	iron ion bi	Molecular	0.00	0.00	Solyc09g0	Solyc09g0	54.33	5.76
GO:00055	iron ion bi	Molecular	0.00	0.00	Solyc04g0	Solyc04g0	900.23	9.81
GO:00055	iron ion bi	Molecular	0.00	0.00	Solyc06g0	Solyc06g0	17.36	4.12
GO:00055	iron ion bi	Molecular	0.00	0.00	Solyc02g0	Solyc02g0	0.28	-1.83
GO:00055	iron ion bi	Molecular	0.00	0.00	Solyc05g0	Solyc05g0	2.42	1.27
GO:00055	iron ion bi	Molecular	0.00	0.00	Solyc12g0	Solyc12g0	1446.11	10.50
GO:00055	iron ion bi	Molecular	0.00	0.00	Solyc02g0	Solyc02g0	3.18	1.67
GO:00055	iron ion bi	Molecular	0.00	0.00	Solyc01g1	Solyc01g1	0.47	-1.09
GO:00055	iron ion bi	Molecular	0.00	0.00	Solyc07g0	Solyc07g0	9.07	3.18
GO:00055	iron ion bi	Molecular	0.00	0.00	Solyc11g0	Solyc11g0	0.00	-9.24
GO:00055	iron ion bi	Molecular	0.00	0.00	Solyc12g0	Solyc12g0	0.00	-9.92
GO:00055	iron ion bi	Molecular	0.00	0.00	Solyc08g0	Solyc08g0	0.34	-1.54
GO:00055	iron ion bi	Molecular	0.00	0.00	Solyc01g0	Solyc01g0	13.05	3.71
GO:00055	iron ion bi	Molecular	0.00	0.00	Solyc07g0	Solyc07g0	0.50	-1.01
GO:00055	iron ion bi	Molecular	0.00	0.00	Solyc04g0	Solyc04g0	9.75	3.29
GO:00055	iron ion bi	Molecular	0.00	0.00	Solyc07g0	Solyc07g0	159.10	7.31
GO:00055	iron ion bi	Molecular	0.00	0.00	Solyc08g0	Solyc08g0	599.38	9.23
GO:00055	iron ion bi	Molecular	0.00	0.00	Solyc02g0	Solyc02g0	8.45	3.08
GO:00055	iron ion bi	Molecular	0.00	0.00	Solyc10g0	Solyc10g0	0.00	-9.79
GO:00055	iron ion bi	Molecular	0.00	0.00	Solyc04g0	Solyc04g0	26.73	4.74
GO:00160	integral co	Cellular C	0.00	0.00	Solyc05g0	Solyc05g0	0.01	-6.09
GO:00160	integral co	Cellular C	0.00	0.00	Solyc01g0	Solyc01g0	0.09	-3.43
GO:00160	integral co	Cellular C	0.00	0.00	Solyc04g0	Solyc04g0	0.18	-2.50
GO:00160	integral co	Cellular C	0.00	0.00	Solyc08g0	Solyc08g0	0.14	-2.87
GO:00160	integral co	Cellular C	0.00	0.00	Solyc01g1	Solyc01g1	5.77	2.53
GO:00160	integral co	Cellular C	0.00	0.00	Solyc03g0	Solyc03g0	0.24	-2.04
GO:00160	integral co	Cellular C	0.00	0.00	Solyc05g0	Solyc05g0	4.45	2.16
GO:00160	integral co	Cellular C	0.00	0.00	Solyc07g0	Solyc07g0	3.16	1.66
GO:00160	integral co	Cellular C	0.00	0.00	Solyc11g0	Solyc11g0	8.62	3.11
GO:00160	integral co	Cellular C	0.00	0.00	Solyc08g0	Solyc08g0	4.51	2.17
GO:00160	integral co	Cellular C	0.00	0.00	Solyc12g0	Solyc12g0	4.94	2.30

GO:00160	integral co	Cellular C	0.00	0.00	Solyc12g0	Solyc12g0	0.25	-1.99
GO:00160	integral co	Cellular C	0.00	0.00	Solyc04g0	Solyc04g0	0.35	-1.50
GO:00160	integral co	Cellular C	0.00	0.00	Solyc02g0	Solyc02g0	0.21	-2.23
GO:00160	integral co	Cellular C	0.00	0.00	Solyc09g0	Solyc09g0	2.22	1.15
GO:00160	integral co	Cellular C	0.00	0.00	Solyc03g0	Solyc03g0	0.38	-1.38
GO:00160	integral co	Cellular C	0.00	0.00	Solyc01g1	Solyc01g1	0.29	-1.80
GO:00160	integral co	Cellular C	0.00	0.00	Solyc12g0	Solyc12g0	0.30	-1.75
GO:00160	integral co	Cellular C	0.00	0.00	Solyc02g0	Solyc02g0	0.34	-1.57
GO:00160	integral co	Cellular C	0.00	0.00	Solyc01g0	Solyc01g0	0.33	-1.61
GO:00160	integral co	Cellular C	0.00	0.00	Solyc02g0	Solyc02g0	2.41	1.27
GO:00160	integral co	Cellular C	0.00	0.00	Solyc02g0	Solyc02g0	0.50	-1.01
GO:00160	integral co	Cellular C	0.00	0.00	Solyc03g0	Solyc03g0	4.02	2.01
GO:00160	integral co	Cellular C	0.00	0.00	Solyc01g1	Solyc01g1	2.47	1.30
GO:00160	integral co	Cellular C	0.00	0.00	Solyc04g0	Solyc04g0	2.83	1.50
GO:00160	integral co	Cellular C	0.00	0.00	Solyc06g0	Solyc06g0	12.59	3.65
GO:00160	integral co	Cellular C	0.00	0.00	Solyc01g0	Solyc01g0	0.46	-1.13
GO:00160	integral co	Cellular C	0.00	0.00	Solyc09g0	Solyc09g0	2.40	1.26
GO:00160	integral co	Cellular C	0.00	0.00	Solyc09g0	Solyc09g0	0.25	-2.01
GO:00160	integral co	Cellular C	0.00	0.00	Solyc01g0	Solyc01g0	0.36	-1.49
GO:00160	integral co	Cellular C	0.00	0.00	Solyc01g1	Solyc01g1	72.82	6.19
GO:00160	integral co	Cellular C	0.00	0.00	Solyc10g0	Solyc10g0	2.93	1.55
GO:00160	integral co	Cellular C	0.00	0.00	Solyc01g0	Solyc01g0	0.35	-1.51
GO:00160	integral co	Cellular C	0.00	0.00	Solyc07g0	Solyc07g0	2.63	1.40
GO:00160	integral co	Cellular C	0.00	0.00	Solyc11g0	Solyc11g0	2.05	1.04
GO:00160	integral co	Cellular C	0.00	0.00	Solyc10g0	Solyc10g0	2.91	1.54
GO:00160	integral co	Cellular C	0.00	0.00	Solyc09g0	Solyc09g0	5.85	2.55
GO:00160	integral co	Cellular C	0.00	0.00	Solyc03g1	Solyc03g1	0.30	-1.75
GO:00160	integral co	Cellular C	0.00	0.00	Solyc10g0	Solyc10g0	0.39	-1.37
GO:00160	integral co	Cellular C	0.00	0.00	Solyc03g0	Solyc03g0	3.72	1.89
GO:00160	integral co	Cellular C	0.00	0.00	Solyc08g0	Solyc08g0	0.29	-1.78
GO:00160	integral co	Cellular C	0.00	0.00	Solyc03g0	Solyc03g0	2.16	1.11
GO:00160	integral co	Cellular C	0.00	0.00	Solyc06g0	Solyc06g0	2.09	1.06
GO:00160	integral co	Cellular C	0.00	0.00	Solyc03g0	Solyc03g0	7.56	2.92
GO:00160	integral co	Cellular C	0.00	0.00	Solyc03g1	Solyc03g1	2.42	1.27
GO:00160	integral co	Cellular C	0.00	0.00	Solyc05g0	Solyc05g0	2.41	1.27
GO:00160	integral co	Cellular C	0.00	0.00	Solyc12g0	Solyc12g0	0.01	-7.31
GO:00160	integral co	Cellular C	0.00	0.00	Solyc06g0	Solyc06g0	0.33	-1.58
GO:00160	integral co	Cellular C	0.00	0.00	Solyc05g0	Solyc05g0	0.49	-1.03
GO:00160	integral co	Cellular C	0.00	0.00	Solyc10g0	Solyc10g0	3.31	1.73
GO:00160	integral co	Cellular C	0.00	0.00	Solyc02g0	Solyc02g0	0.44	-1.19
GO:00160	integral co	Cellular C	0.00	0.00	Solyc11g0	Solyc11g0	0.35	-1.51
GO:00160	integral co	Cellular C	0.00	0.00	Solyc10g0	Solyc10g0	3.83	1.94
GO:00160	integral co	Cellular C	0.00	0.00	Solyc05g0	Solyc05g0	4.51	2.17
GO:00160	integral co	Cellular C	0.00	0.00	Solyc12g0	Solyc12g0	5.32	2.41
GO:00160	integral co	Cellular C	0.00	0.00	Solyc02g0	Solyc02g0	0.38	-1.41
GO:00160	integral co	Cellular C	0.00	0.00	Solyc10g0	Solyc10g0	2.38	1.25
GO:00160	integral co	Cellular C	0.00	0.00	Solyc01g0	Solyc01g0	2.45	1.29
GO:00160	integral co	Cellular C	0.00	0.00	Solyc11g0	Solyc11g0	0.02	-5.38
GO:00160	integral co	Cellular C	0.00	0.00	Solyc02g0	Solyc02g0	2.31	1.21
GO:00160	integral co	Cellular C	0.00	0.00	Solyc06g0	Solyc06g0	2.35	1.23
GO:00160	integral co	Cellular C	0.00	0.00	Solyc04g0	Solyc04g0	0.41	-1.29

GO:00160	integral co	Cellular Co	0.00	0.00	Solyc04g0	Solyc04g0	0.38	-1.41
GO:00160	integral co	Cellular Co	0.00	0.00	Solyc06g0	Solyc06g0	2.02	1.02
GO:00160	integral co	Cellular Co	0.00	0.00	Solyc04g0	Solyc04g0	0.41	-1.28
GO:00160	integral co	Cellular Co	0.00	0.00	Solyc02g0	Solyc02g0	0.50	-1.00
GO:00160	integral co	Cellular Co	0.00	0.00	Solyc06g0	Solyc06g0	0.41	-1.30
GO:00160	integral co	Cellular Co	0.00	0.00	Solyc12g0	Solyc12g0	2.10	1.07
GO:00160	integral co	Cellular Co	0.00	0.00	Solyc03g1	Solyc03g1	0.25	-2.02
GO:00160	integral co	Cellular Co	0.00	0.00	Solyc04g0	Solyc04g0	2.22	1.15
GO:00160	integral co	Cellular Co	0.00	0.00	Solyc02g0	Solyc02g0	3.55	1.83
GO:00160	integral co	Cellular Co	0.00	0.00	Solyc10g0	Solyc10g0	2.60	1.38
GO:00160	integral co	Cellular Co	0.00	0.00	Solyc05g0	Solyc05g0	0.27	-1.87
GO:00160	integral co	Cellular Co	0.00	0.00	Solyc06g0	Solyc06g0	0.40	-1.31
GO:00160	integral co	Cellular Co	0.00	0.00	Solyc03g0	Solyc03g0	2.09	1.07
GO:00160	integral co	Cellular Co	0.00	0.00	Solyc10g0	Solyc10g0	2.56	1.36
GO:00160	integral co	Cellular Co	0.00	0.00	Solyc09g0	Solyc09g0	2.01	1.01
GO:00160	integral co	Cellular Co	0.00	0.00	Solyc03g0	Solyc03g0	0.18	-2.45
GO:00160	integral co	Cellular Co	0.00	0.00	Solyc05g0	Solyc05g0	0.08	-3.58
GO:00160	integral co	Cellular Co	0.00	0.00	Solyc10g0	Solyc10g0	3.33	1.74
GO:00160	integral co	Cellular Co	0.00	0.00	Solyc06g0	Solyc06g0	0.46	-1.11
GO:00160	integral co	Cellular Co	0.00	0.00	Solyc09g0	Solyc09g0	3.41	1.77
GO:00160	integral co	Cellular Co	0.00	0.00	Solyc01g0	Solyc01g0	0.46	-1.12
GO:00160	integral co	Cellular Co	0.00	0.00	Solyc05g0	Solyc05g0	0.28	-1.82
GO:00160	integral co	Cellular Co	0.00	0.00	Solyc11g0	Solyc11g0	0.24	-2.04
GO:00160	integral co	Cellular Co	0.00	0.00	Solyc08g0	Solyc08g0	0.12	-3.05
GO:00160	integral co	Cellular Co	0.00	0.00	Solyc11g0	Solyc11g0	0.49	-1.04
GO:00160	integral co	Cellular Co	0.00	0.00	Solyc05g0	Solyc05g0	0.50	-1.00
GO:00160	integral co	Cellular Co	0.00	0.00	Solyc04g0	Solyc04g0	7.43	2.89
GO:00160	integral co	Cellular Co	0.00	0.00	Solyc01g0	Solyc01g0	0.49	-1.04
GO:00160	integral co	Cellular Co	0.00	0.00	Solyc10g0	Solyc10g0	2.36	1.24
GO:00160	integral co	Cellular Co	0.00	0.00	Solyc02g0	Solyc02g0	0.34	-1.56
GO:00160	integral co	Cellular Co	0.00	0.00	Solyc02g0	Solyc02g0	2.52	1.33
GO:00160	integral co	Cellular Co	0.00	0.00	Solyc09g0	Solyc09g0	0.18	-2.50
GO:00160	integral co	Cellular Co	0.00	0.00	Solyc01g0	Solyc01g0	0.42	-1.26
GO:00160	integral co	Cellular Co	0.00	0.00	Solyc02g0	Solyc02g0	2.54	1.34
GO:00160	integral co	Cellular Co	0.00	0.00	Solyc12g0	Solyc12g0	0.17	-2.52
GO:00160	integral co	Cellular Co	0.00	0.00	Solyc07g0	Solyc07g0	5.25	2.39
GO:00160	integral co	Cellular Co	0.00	0.00	Solyc09g0	Solyc09g0	0.36	-1.46
GO:00160	integral co	Cellular Co	0.00	0.00	Solyc11g0	Solyc11g0	6.63	2.73
GO:00160	integral co	Cellular Co	0.00	0.00	Solyc09g0	Solyc09g0	0.12	-3.08
GO:00160	integral co	Cellular Co	0.00	0.00	Solyc01g1	Solyc01g1	14.96	3.90
GO:00160	integral co	Cellular Co	0.00	0.00	Solyc04g0	Solyc04g0	0.30	-1.76
GO:00160	integral co	Cellular Co	0.00	0.00	Solyc09g0	Solyc09g0	3.19	1.68
GO:00160	integral co	Cellular Co	0.00	0.00	Solyc11g0	Solyc11g0	3.29	1.72
GO:00160	integral co	Cellular Co	0.00	0.00	Solyc05g0	Solyc05g0	0.44	-1.19
GO:00160	integral co	Cellular Co	0.00	0.00	Solyc09g0	Solyc09g0	0.47	-1.07
GO:00160	integral co	Cellular Co	0.00	0.00	Solyc05g0	Solyc05g0	0.43	-1.22
GO:00160	integral co	Cellular Co	0.00	0.00	Solyc02g0	Solyc02g0	2.50	1.32
GO:00160	integral co	Cellular Co	0.00	0.00	Solyc03g1	Solyc03g1	0.39	-1.35
GO:00160	integral co	Cellular Co	0.00	0.00	Solyc10g0	Solyc10g0	4.66	2.22
GO:00160	integral co	Cellular Co	0.00	0.00	Solyc03g0	Solyc03g0	15.63	3.97
GO:00160	integral co	Cellular Co	0.00	0.00	Solyc04g0	Solyc04g0	0.26	-1.94

GO:00160	integral co	Cellular Co	0.00	0.00	Solyc10g0	Solyc10g0	15621.48	13.93
GO:00160	integral co	Cellular Co	0.00	0.00	Solyc06g0	Solyc06g0	3.13	1.64
GO:00160	integral co	Cellular Co	0.00	0.00	Solyc01g0	Solyc01g0	2.56	1.36
GO:00160	integral co	Cellular Co	0.00	0.00	Solyc10g0	Solyc10g0	17.76	4.15
GO:00160	integral co	Cellular Co	0.00	0.00	Solyc04g0	Solyc04g0	0.00	-11.98
GO:00160	integral co	Cellular Co	0.00	0.00	Solyc02g0	Solyc02g0	0.27	-1.89
GO:00160	integral co	Cellular Co	0.00	0.00	Solyc03g1	Solyc03g1	0.49	-1.04
GO:00160	integral co	Cellular Co	0.00	0.00	Solyc10g0	Solyc10g0	2.02	1.01
GO:00160	integral co	Cellular Co	0.00	0.00	Solyc11g0	Solyc11g0	0.00	-13.30
GO:00160	integral co	Cellular Co	0.00	0.00	Solyc06g0	Solyc06g0	0.43	-1.22
GO:00160	integral co	Cellular Co	0.00	0.00	Solyc02g0	Solyc02g0	0.18	-2.49
GO:00160	integral co	Cellular Co	0.00	0.00	Solyc04g0	Solyc04g0	2.36	1.24
GO:00160	integral co	Cellular Co	0.00	0.00	Solyc01g0	Solyc01g0	3528.16	11.78
GO:00160	integral co	Cellular Co	0.00	0.00	Solyc03g1	Solyc03g1	2.14	1.09
GO:00160	integral co	Cellular Co	0.00	0.00	Solyc03g1	Solyc03g1	0.44	-1.20
GO:00160	integral co	Cellular Co	0.00	0.00	Solyc05g0	Solyc05g0	6.90	2.79
GO:00160	integral co	Cellular Co	0.00	0.00	Solyc11g0	Solyc11g0	5096.34	12.32
GO:00160	integral co	Cellular Co	0.00	0.00	Solyc10g0	Solyc10g0	0.04	-4.70
GO:00160	integral co	Cellular Co	0.00	0.00	Solyc03g1	Solyc03g1	10.69	3.42
GO:00160	integral co	Cellular Co	0.00	0.00	Solyc03g1	Solyc03g1	0.33	-1.59
GO:00160	integral co	Cellular Co	0.00	0.00	Solyc03g1	Solyc03g1	2.04	1.03
GO:00160	integral co	Cellular Co	0.00	0.00	Solyc08g0	Solyc08g0	0.42	-1.27
GO:00160	integral co	Cellular Co	0.00	0.00	Solyc10g0	Solyc10g0	47.10	5.56
GO:00160	integral co	Cellular Co	0.00	0.00	Solyc09g0	Solyc09g0	51.95	5.70
GO:00160	integral co	Cellular Co	0.00	0.00	Solyc03g0	Solyc03g0	0.49	-1.02
GO:00160	integral co	Cellular Co	0.00	0.00	Solyc03g0	Solyc03g0	2.06	1.04
GO:00160	integral co	Cellular Co	0.00	0.00	Solyc10g0	Solyc10g0	1973.43	10.95
GO:00160	integral co	Cellular Co	0.00	0.00	Solyc01g0	Solyc01g0	6.50	2.70
GO:00160	integral co	Cellular Co	0.00	0.00	Solyc05g0	Solyc05g0	6.84	2.77
GO:00160	integral co	Cellular Co	0.00	0.00	Solyc03g0	Solyc03g0	2.05	1.04
GO:00160	integral co	Cellular Co	0.00	0.00	Solyc03g0	Solyc03g0	0.04	-4.63
GO:00160	integral co	Cellular Co	0.00	0.00	Solyc05g0	Solyc05g0	21240.81	14.37
GO:00160	integral co	Cellular Co	0.00	0.00	Solyc12g0	Solyc12g0	3.10	1.63
GO:00160	integral co	Cellular Co	0.00	0.00	Solyc01g0	Solyc01g0	2.42	1.27
GO:00160	integral co	Cellular Co	0.00	0.00	Solyc12g0	Solyc12g0	0.00	-11.26
GO:00160	integral co	Cellular Co	0.00	0.00	Solyc02g0	Solyc02g0	2381.72	11.22
GO:00160	integral co	Cellular Co	0.00	0.00	Solyc04g0	Solyc04g0	2.09	1.06
GO:00160	integral co	Cellular Co	0.00	0.00	Solyc03g1	Solyc03g1	0.48	-1.07
GO:00160	integral co	Cellular Co	0.00	0.00	Solyc12g0	Solyc12g0	0.00	-13.60
GO:00160	integral co	Cellular Co	0.00	0.00	Solyc12g0	Solyc12g0	0.37	-1.44
GO:00160	integral co	Cellular Co	0.00	0.00	Solyc12g0	Solyc12g0	0.05	-4.20
GO:00160	integral co	Cellular Co	0.00	0.00	Solyc08g0	Solyc08g0	0.24	-2.05
GO:00160	integral co	Cellular Co	0.00	0.00	Solyc08g0	Solyc08g0	2.67	1.41
GO:00160	integral co	Cellular Co	0.00	0.00	Solyc03g0	Solyc03g0	2.16	1.11
GO:00160	integral co	Cellular Co	0.00	0.00	Solyc05g0	Solyc05g0	0.00	-13.36
GO:00160	integral co	Cellular Co	0.00	0.00	Solyc04g0	Solyc04g0	0.00	-13.79
GO:00160	integral co	Cellular Co	0.00	0.00	Solyc05g0	Solyc05g0	0.01	-6.23
GO:00160	integral co	Cellular Co	0.00	0.00	Solyc10g0	Solyc10g0	0.00	-13.68
GO:00160	integral co	Cellular Co	0.00	0.00	Solyc10g0	Solyc10g0	0.00	-13.73
GO:00160	integral co	Cellular Co	0.00	0.00	Solyc11g0	Solyc11g0	0.38	-1.41
GO:00160	integral co	Cellular Co	0.00	0.00	Solyc03g1	Solyc03g1	13.81	3.79

GO:00160	integral co	Cellular C	0.00	0.00	Solyc04g0	Solyc04g0	9579.06	13.23
GO:00160	integral co	Cellular C	0.00	0.00	Solyc05g0	Solyc05g0	2.46	1.30
GO:00160	integral co	Cellular C	0.00	0.00	Solyc09g0	Solyc09g0	0.49	-1.04
GO:00160	integral co	Cellular C	0.00	0.00	Solyc04g0	Solyc04g0	0.41	-1.29
GO:00160	integral co	Cellular C	0.00	0.00	Solyc09g0	Solyc09g0	3.57	1.84
GO:00160	integral co	Cellular C	0.00	0.00	Solyc12g0	Solyc12g0	44.33	5.47
GO:00160	integral co	Cellular C	0.00	0.00	Solyc09g0	Solyc09g0	0.26	-1.97
GO:00160	integral co	Cellular C	0.00	0.00	Solyc10g0	Solyc10g0	0.38	-1.41
GO:00160	integral co	Cellular C	0.00	0.00	Solyc06g0	Solyc06g0	2059.41	11.01
GO:00160	integral co	Cellular C	0.00	0.00	Solyc12g0	Solyc12g0	3.03	1.60
GO:00160	integral co	Cellular C	0.00	0.00	Solyc05g0	Solyc05g0	2.29	1.20
GO:00160	integral co	Cellular C	0.00	0.00	Solyc09g0	Solyc09g0	0.49	-1.04
GO:00160	integral co	Cellular C	0.00	0.00	Solyc08g0	Solyc08g0	3738.24	11.87
GO:00160	integral co	Cellular C	0.00	0.00	Solyc10g0	Solyc10g0	0.00	-12.09
GO:00160	integral co	Cellular C	0.00	0.00	Solyc03g0	Solyc03g0	0.31	-1.69
GO:00160	integral co	Cellular C	0.00	0.00	Solyc01g1	Solyc01g1	4.37	2.13
GO:00160	integral co	Cellular C	0.00	0.00	Solyc09g0	Solyc09g0	0.00	-13.14
GO:00160	integral co	Cellular C	0.00	0.00	Solyc02g0	Solyc02g0	0.48	-1.05
GO:00160	integral co	Cellular C	0.00	0.00	Solyc01g0	Solyc01g0	3.53	1.82
GO:00160	integral co	Cellular C	0.00	0.00	Solyc01g1	Solyc01g1	2829.11	11.47
GO:00160	integral co	Cellular C	0.00	0.00	Solyc10g0	Solyc10g0	0.00	-12.76
GO:00160	integral co	Cellular C	0.00	0.00	Solyc03g0	Solyc03g0	3.43	1.78
GO:00160	integral co	Cellular C	0.00	0.00	Solyc12g0	Solyc12g0	3.03	1.60
GO:00160	integral co	Cellular C	0.00	0.00	Solyc02g0	Solyc02g0	0.03	-4.99
GO:00160	integral co	Cellular C	0.00	0.00	Solyc01g1	Solyc01g1	0.20	-2.35
GO:00160	integral co	Cellular C	0.00	0.00	Solyc06g0	Solyc06g0	0.05	-4.46
GO:00160	integral co	Cellular C	0.00	0.00	Solyc11g0	Solyc11g0	0.00	-13.08
GO:00160	integral co	Cellular C	0.00	0.00	Solyc09g0	Solyc09g0	0.00	-13.63
GO:00160	integral co	Cellular C	0.00	0.00	Solyc06g0	Solyc06g0	0.42	-1.25
GO:00160	integral co	Cellular C	0.00	0.00	Solyc12g0	Solyc12g0	0.00	-10.94
GO:00160	integral co	Cellular C	0.00	0.00	Solyc04g0	Solyc04g0	15.77	3.98
GO:00160	integral co	Cellular C	0.00	0.00	Solyc10g0	Solyc10g0	0.03	-5.30
GO:00160	integral co	Cellular C	0.00	0.00	Solyc04g0	Solyc04g0	0.46	-1.11
GO:00160	integral co	Cellular C	0.00	0.00	Solyc06g0	Solyc06g0	0.00	-10.22
GO:00160	integral co	Cellular C	0.00	0.00	Solyc08g0	Solyc08g0	0.50	-1.00
GO:00160	integral co	Cellular C	0.00	0.00	Solyc08g0	Solyc08g0	0.29	-1.80
GO:00160	integral co	Cellular C	0.00	0.00	Solyc10g0	Solyc10g0	2.07	1.05
GO:00160	integral co	Cellular C	0.00	0.00	Solyc06g0	Solyc06g0	18.67	4.22
GO:00160	integral co	Cellular C	0.00	0.00	Solyc08g0	Solyc08g0	0.47	-1.08
GO:00160	integral co	Cellular C	0.00	0.00	Solyc02g0	Solyc02g0	3.38	1.76
GO:00160	integral co	Cellular C	0.00	0.00	Solyc02g0	Solyc02g0	0.40	-1.31
GO:00160	integral co	Cellular C	0.00	0.00	Solyc10g0	Solyc10g0	4.22	2.08
GO:00160	integral co	Cellular C	0.00	0.00	Solyc01g1	Solyc01g1	0.24	-2.07
GO:00160	integral co	Cellular C	0.00	0.00	Solyc04g0	Solyc04g0	0.39	-1.37
GO:00160	integral co	Cellular C	0.00	0.00	Solyc03g0	Solyc03g0	0.34	-1.57
GO:00160	integral co	Cellular C	0.00	0.00	Solyc04g0	Solyc04g0	1191.32	10.22
GO:00160	integral co	Cellular C	0.00	0.00	Solyc08g0	Solyc08g0	21.56	4.43
GO:00160	integral co	Cellular C	0.00	0.00	Solyc01g0	Solyc01g0	0.02	-5.72
GO:00160	integral co	Cellular C	0.00	0.00	Solyc06g0	Solyc06g0	2.08	1.06
GO:00160	integral co	Cellular C	0.00	0.00	Solyc04g0	Solyc04g0	5759.74	12.49
GO:00160	integral co	Cellular C	0.00	0.00	Solyc06g0	PSBS	2.99	1.58

GO:00160	integral co	Cellular C	0.00	0.00	Solyc01g0	Solyc01g0	0.19	-2.40
GO:00160	integral co	Cellular C	0.00	0.00	Solyc07g0	Solyc07g0	2.77	1.47
GO:00160	integral co	Cellular C	0.00	0.00	Solyc05g0	Solyc05g0	0.05	-4.24
GO:00160	integral co	Cellular C	0.00	0.00	Solyc06g0	Solyc06g0	2.13	1.09
GO:00160	integral co	Cellular C	0.00	0.00	Solyc08g0	Solyc08g0	1235.43	10.27
GO:00160	integral co	Cellular C	0.00	0.00	Solyc12g1	Solyc12g1	0.00	-10.11
GO:00160	integral co	Cellular C	0.00	0.00	Solyc10g0	Solyc10g0	0.37	-1.43
GO:00160	integral co	Cellular C	0.00	0.00	Solyc04g0	Solyc04g0	3.05	1.61
GO:00160	integral co	Cellular C	0.00	0.00	Solyc02g0	Solyc02g0	7.75	2.95
GO:00160	integral co	Cellular C	0.00	0.00	Solyc03g0	Solyc03g0	0.49	-1.03
GO:00160	integral co	Cellular C	0.00	0.00	Solyc02g0	Solyc02g0	2.29	1.20
GO:00160	integral co	Cellular C	0.00	0.00	Solyc07g0	Solyc07g0	2.47	1.30
GO:00160	integral co	Cellular C	0.00	0.00	Solyc03g0	Solyc03g0	2.60	1.38
GO:00160	integral co	Cellular C	0.00	0.00	Solyc01g0	Solyc01g0	36.75	5.20
GO:00160	integral co	Cellular C	0.00	0.00	Solyc04g0	Solyc04g0	1523.79	10.57
GO:00160	integral co	Cellular C	0.00	0.00	Solyc12g0	Solyc12g0	0.47	-1.10
GO:00160	integral co	Cellular C	0.00	0.00	Solyc02g0	Solyc02g0	19.78	4.31
GO:00160	integral co	Cellular C	0.00	0.00	Solyc10g0	Solyc10g0	0.03	-5.05
GO:00160	integral co	Cellular C	0.00	0.00	Solyc11g0	Solyc11g0	0.00	-11.68
GO:00160	integral co	Cellular C	0.00	0.00	Solyc07g0	Solyc07g0	14.86	3.89
GO:00160	integral co	Cellular C	0.00	0.00	Solyc03g0	Solyc03g0	2.40	1.26
GO:00160	integral co	Cellular C	0.00	0.00	Solyc03g0	Solyc03g0	3.69	1.88
GO:00160	integral co	Cellular C	0.00	0.00	Solyc01g0	Solyc01g0	894.88	9.81
GO:00160	integral co	Cellular C	0.00	0.00	Solyc03g1	Solyc03g1	0.10	-3.25
GO:00160	integral co	Cellular C	0.00	0.00	Solyc08g0	Solyc08g0	629.04	9.30
GO:00160	integral co	Cellular C	0.00	0.00	Solyc09g0	Solyc09g0	6.94	2.79
GO:00160	integral co	Cellular C	0.00	0.00	Solyc06g0	Solyc06g0	0.15	-2.76
GO:00160	integral co	Cellular C	0.00	0.00	Solyc10g0	Solyc10g0	0.43	-1.23
GO:00160	integral co	Cellular C	0.00	0.00	Solyc02g0	Solyc02g0	0.19	-2.37
GO:00160	integral co	Cellular C	0.00	0.00	Solyc06g0	Solyc06g0	2.09	1.06
GO:00160	integral co	Cellular C	0.00	0.00	Solyc03g0	Solyc03g0	4.00	2.00
GO:00160	integral co	Cellular C	0.00	0.00	Solyc12g0	Solyc12g0	0.25	-2.01
GO:00160	integral co	Cellular C	0.00	0.00	Solyc10g0	Solyc10g0	0.04	-4.58
GO:00160	integral co	Cellular C	0.00	0.00	Solyc01g0	Solyc01g0	0.00	-11.99
GO:00160	integral co	Cellular C	0.00	0.00	Solyc07g0	Solyc07g0	2.00	1.00
GO:00160	integral co	Cellular C	0.00	0.00	Solyc12g0	Solyc12g0	14.44	3.85
GO:00160	integral co	Cellular C	0.00	0.00	Solyc01g0	Solyc01g0	10.72	3.42
GO:00160	integral co	Cellular C	0.00	0.00	Solyc02g0	Solyc02g0	307.12	8.26
GO:00160	integral co	Cellular C	0.00	0.00	Solyc02g0	Solyc02g0	0.21	-2.28
GO:00160	integral co	Cellular C	0.00	0.00	Solyc02g0	Solyc02g0	3.18	1.67
GO:00160	integral co	Cellular C	0.00	0.00	Solyc06g0	Solyc06g0	6.96	2.80
GO:00160	integral co	Cellular C	0.00	0.00	Solyc07g0	Solyc07g0	0.40	-1.33
GO:00160	integral co	Cellular C	0.00	0.00	Solyc08g0	Solyc08g0	451.28	8.82
GO:00160	integral co	Cellular C	0.00	0.00	Solyc03g1	Solyc03g1	0.06	-4.15
GO:00160	integral co	Cellular C	0.00	0.00	Solyc07g0	Solyc07g0	9.07	3.18
GO:00160	integral co	Cellular C	0.00	0.00	Solyc07g0	Solyc07g0	714.98	9.48
GO:00160	integral co	Cellular C	0.00	0.00	Solyc06g0	Solyc06g0	0.32	-1.62
GO:00160	integral co	Cellular C	0.00	0.00	Solyc07g0	Solyc07g0	2.01	1.01
GO:00160	integral co	Cellular C	0.00	0.00	Solyc12g0	Solyc12g0	0.47	-1.09
GO:00160	integral co	Cellular C	0.00	0.00	Solyc08g0	Solyc08g0	0.34	-1.54
GO:00160	integral co	Cellular C	0.00	0.00	Solyc04g0	Solyc04g0	0.06	-3.97

GO:00160	integral co	Cellular C	0.00	0.00	Solyc09g0	Solyc09g0	3.37	1.75
GO:00160	integral co	Cellular C	0.00	0.00	Solyc07g0	Solyc07g0	2.39	1.26
GO:00160	integral co	Cellular C	0.00	0.00	Solyc05g0	Solyc05g0	9.14	3.19
GO:00160	integral co	Cellular C	0.00	0.00	Solyc01g1	Solyc01g1	0.00	-9.07
GO:00160	integral co	Cellular C	0.00	0.00	Solyc02g0	Solyc02g0	2.19	1.13
GO:00160	integral co	Cellular C	0.00	0.00	Solyc01g1	psbZ	0.17	-2.52
GO:00160	integral co	Cellular C	0.00	0.00	Solyc06g0	Solyc06g0	3.28	1.71
GO:00160	integral co	Cellular C	0.00	0.00	Solyc08g0	Solyc08g0	13.23	3.73
GO:00160	integral co	Cellular C	0.00	0.00	Solyc08g0	Solyc08g0	2.56	1.36
GO:00160	integral co	Cellular C	0.00	0.00	Solyc07g0	Solyc07g0	0.50	-1.01
GO:00160	integral co	Cellular C	0.00	0.00	Solyc11g0	Solyc11g0	0.09	-3.49
GO:00160	integral co	Cellular C	0.00	0.00	Solyc11g0	Solyc11g0	0.03	-5.20
GO:00160	integral co	Cellular C	0.00	0.00	Solyc05g0	Solyc05g0	0.14	-2.79
GO:00160	integral co	Cellular C	0.00	0.00	Solyc08g0	Solyc08g0	12.03	3.59
GO:00160	integral co	Cellular C	0.00	0.00	Solyc04g0	Solyc04g0	5.19	2.37
GO:00160	integral co	Cellular C	0.00	0.00	Solyc04g0	Solyc04g0	7.72	2.95
GO:00160	integral co	Cellular C	0.00	0.00	Solyc12g0	Solyc12g0	369.30	8.53
GO:00160	integral co	Cellular C	0.00	0.00	Solyc11g0	Solyc11g0	0.24	-2.07
GO:00160	integral co	Cellular C	0.00	0.00	Solyc09g0	Solyc09g0	0.20	-2.33
GO:00160	integral co	Cellular C	0.00	0.00	Solyc01g0	Solyc01g0	0.08	-3.58
GO:00160	integral co	Cellular C	0.00	0.00	Solyc06g0	Solyc06g0	2.48	1.31
GO:00160	integral co	Cellular C	0.00	0.00	Solyc03g1	Solyc03g1	356.83	8.48
GO:00160	integral co	Cellular C	0.00	0.00	Solyc12g0	Solyc12g0	0.10	-3.34
GO:00160	integral co	Cellular C	0.00	0.00	Solyc03g1	Solyc03g1	5.76	2.53
GO:00160	integral co	Cellular C	0.00	0.00	Solyc03g0	Solyc03g0	11.28	3.50
GO:00160	integral co	Cellular C	0.00	0.00	Solyc11g0	Solyc11g0	0.23	-2.09
GO:00160	integral co	Cellular C	0.00	0.00	Solyc09g0	Solyc09g0	0.49	-1.03
GO:00160	integral co	Cellular C	0.00	0.00	Solyc02g0	Solyc02g0	0.49	-1.03
GO:00160	integral co	Cellular C	0.00	0.00	Solyc04g0	Solyc04g0	0.10	-3.25
GO:00160	integral co	Cellular C	0.00	0.00	Solyc03g0	Solyc03g0	0.13	-3.00
GO:00160	integral co	Cellular C	0.00	0.00	Solyc03g0	Solyc03g0	2.01	1.01
GO:00160	integral co	Cellular C	0.00	0.00	Solyc08g0	Solyc08g0	0.07	-3.74
GO:00160	integral co	Cellular C	0.00	0.00	Solyc05g0	Solyc05g0	7.65	2.94
GO:00160	integral co	Cellular C	0.00	0.00	Solyc11g0	Solyc11g0	0.09	-3.52
GO:00160	integral co	Cellular C	0.00	0.00	Solyc06g0	Solyc06g0	13.67	3.77
GO:00160	integral co	Cellular C	0.00	0.00	Solyc10g0	Solyc10g0	0.06	-4.16
GO:00160	integral co	Cellular C	0.00	0.00	Solyc11g0	Solyc11g0	15.49	3.95
GO:00160	integral co	Cellular C	0.00	0.00	Solyc01g0	Solyc01g0	2335.77	11.19
GO:00160	integral co	Cellular C	0.00	0.00	Solyc12g0	Solyc12g0	4.99	2.32
GO:00160	integral co	Cellular C	0.00	0.00	Solyc02g0	Solyc02g0	412.01	8.69
GO:00160	integral co	Cellular C	0.00	0.00	Solyc08g0	Solyc08g0	4.19	2.07
GO:00160	integral co	Cellular C	0.00	0.00	Solyc04g0	Solyc04g0	2.31	1.21
GO:00160	integral co	Cellular C	0.00	0.00	Solyc01g1	Solyc01g1	0.08	-3.66
GO:00160	integral co	Cellular C	0.00	0.00	Solyc02g0	Solyc02g0	2.53	1.34
GO:00160	integral co	Cellular C	0.00	0.00	Solyc03g0	Solyc03g0	0.43	-1.23
GO:00160	integral co	Cellular C	0.00	0.00	Solyc03g0	Solyc03g0	0.00	-9.99
GO:00160	integral co	Cellular C	0.00	0.00	Solyc12g0	Solyc12g0	2.29	1.20
GO:00160	integral co	Cellular C	0.00	0.00	Solyc01g0	Solyc01g0	0.05	-4.42
GO:00160	integral co	Cellular C	0.00	0.00	Solyc10g0	Solyc10g0	2.16	1.11
GO:00160	integral co	Cellular C	0.00	0.00	Solyc03g0	Solyc03g0	0.36	-1.46
GO:00160	integral co	Cellular C	0.00	0.00	Solyc02g0	Solyc02g0	8.45	3.08

GO:00160	integral co	Cellular C	0.00	0.00	Solyc09g0	Solyc09g0	0.00	-8.11
GO:00160	integral co	Cellular C	0.00	0.00	Solyc07g0	Solyc07g0	0.01	-7.50
GO:00160	integral co	Cellular C	0.00	0.00	Solyc06g0	Solyc06g0	0.44	-1.17
GO:00160	integral co	Cellular C	0.00	0.00	Solyc05g0	Solyc05g0	20.56	4.36
GO:00160	integral co	Cellular C	0.00	0.00	Solyc10g0	Solyc10g0	0.33	-1.59
GO:00160	integral co	Cellular C	0.00	0.00	Solyc11g0	Solyc11g0	0.43	-1.20
GO:00160	integral co	Cellular C	0.00	0.00	Solyc06g0	Solyc06g0	587.73	9.20
GO:00160	integral co	Cellular C	0.00	0.00	Solyc02g0	Solyc02g0	517.85	9.02
GO:00160	integral co	Cellular C	0.00	0.00	Solyc03g0	Solyc03g0	0.00	-11.24
GO:00160	integral co	Cellular C	0.00	0.00	Solyc09g0	Solyc09g0	0.38	-1.39
GO:00160	integral co	Cellular C	0.00	0.00	Solyc03g1	Solyc03g1	0.07	-3.78
GO:00160	integral co	Cellular C	0.00	0.00	Solyc07g0	Solyc07g0	0.39	-1.35
GO:00160	integral co	Cellular C	0.00	0.00	Solyc06g0	Solyc06g0	2.66	1.41
GO:00160	integral co	Cellular C	0.00	0.00	Solyc04g0	Solyc04g0	9.52	3.25
GO:00160	integral co	Cellular C	0.00	0.00	Solyc08g0	Solyc08g0	11.44	3.52
GO:00160	integral co	Cellular C	0.00	0.00	Solyc03g0	Solyc03g0	0.39	-1.37
GO:00160	integral co	Cellular C	0.00	0.00	Solyc11g0	Solyc11g0	0.30	-1.71
GO:00160	integral co	Cellular C	0.00	0.00	Solyc03g1	Solyc03g1	0.00	-8.90
GO:00160	integral co	Cellular C	0.00	0.00	Solyc06g0	Solyc06g0	0.12	-3.11
GO:00160	integral co	Cellular C	0.00	0.00	Solyc02g0	Solyc02g0	19.25	4.27
GO:00160	integral co	Cellular C	0.00	0.00	Solyc03g0	Solyc03g0	4.00	2.00
GO:00160	integral co	Cellular C	0.00	0.00	Solyc11g0	Solyc11g0	18.97	4.25
GO:00160	integral co	Cellular C	0.00	0.00	Solyc03g0	Solyc03g0	31.13	4.96
GO:00160	integral co	Cellular C	0.00	0.00	Solyc02g0	Solyc02g0	0.39	-1.34
GO:00160	integral co	Cellular C	0.00	0.00	Solyc04g0	Solyc04g0	26.73	4.74
GO:00160	integral co	Cellular C	0.00	0.00	Solyc04g0	Solyc04g0	0.00	-9.36
GO:00160	integral co	Cellular C	0.00	0.00	Solyc11g0	Solyc11g0	0.40	-1.31
GO:00200	heme bindi	Molecular	0.00	0.00	Solyc07g0	Solyc07g0	6.73	2.75
GO:00200	heme bindi	Molecular	0.00	0.00	Solyc11g0	Solyc11g0	8.62	3.11
GO:00200	heme bindi	Molecular	0.00	0.00	Solyc01g0	Solyc01g0	14.73	3.88
GO:00200	heme bindi	Molecular	0.00	0.00	Solyc11g0	AOS2	3.20	1.68
GO:00200	heme bindi	Molecular	0.00	0.00	Solyc04g0	Solyc04g0	2.89	1.53
GO:00200	heme bindi	Molecular	0.00	0.00	Solyc07g0	Solyc07g0	0.32	-1.63
GO:00200	heme bindi	Molecular	0.00	0.00	Solyc02g0	Solyc02g0	8.87	3.15
GO:00200	heme bindi	Molecular	0.00	0.00	Solyc10g0	Solyc10g0	0.40	-1.34
GO:00200	heme bindi	Molecular	0.00	0.00	Solyc03g1	Solyc03g1	0.30	-1.75
GO:00200	heme bindi	Molecular	0.00	0.00	Solyc06g0	Solyc06g0	7.48	2.90
GO:00200	heme bindi	Molecular	0.00	0.00	Solyc03g1	Solyc03g1	4.61	2.21
GO:00200	heme bindi	Molecular	0.00	0.00	Solyc04g0	Solyc04g0	6.45	2.69
GO:00200	heme bindi	Molecular	0.00	0.00	Solyc01g1	Solyc01g1	0.03	-5.02
GO:00200	heme bindi	Molecular	0.00	0.00	Solyc05g0	Solyc05g0	2.81	1.49
GO:00200	heme bindi	Molecular	0.00	0.00	Solyc12g0	Solyc12g0	4.31	2.11
GO:00200	heme bindi	Molecular	0.00	0.00	Solyc05g0	Solyc05g0	0.08	-3.58
GO:00200	heme bindi	Molecular	0.00	0.00	Solyc09g0	Solyc09g0	3.41	1.77
GO:00200	heme bindi	Molecular	0.00	0.00	Solyc02g0	Solyc02g0	2.54	1.34
GO:00200	heme bindi	Molecular	0.00	0.00	Solyc08g0	Solyc08g0	2.39	1.25
GO:00200	heme bindi	Molecular	0.00	0.00	Solyc01g0	Solyc01g0	5.13	2.36
GO:00200	heme bindi	Molecular	0.00	0.00	Solyc08g0	Solyc08g0	4598.17	12.17
GO:00200	heme bindi	Molecular	0.00	0.00	Solyc11g0	Solyc11g0	0.00	-13.30
GO:00200	heme bindi	Molecular	0.00	0.00	Solyc01g0	Solyc01g0	3528.16	11.78
GO:00200	heme bindi	Molecular	0.00	0.00	Solyc09g0	Solyc09g0	51.95	5.70

GO:00200	heme bind	Molecular	0.00	0.00	Solyc08g0	Solyc08g0	4039.84	11.98
GO:00200	heme bind	Molecular	0.00	0.00	Solyc03g0	Solyc03g0	5511.05	12.43
GO:00200	heme bind	Molecular	0.00	0.00	Solyc07g0	Solyc07g0	40.33	5.33
GO:00200	heme bind	Molecular	0.00	0.00	Solyc10g0	Solyc10g0	1604.71	10.65
GO:00200	heme bind	Molecular	0.00	0.00	Solyc01g0	Solyc01g0	3.21	1.68
GO:00200	heme bind	Molecular	0.00	0.00	Solyc01g1	Solyc01g1	4.37	2.13
GO:00200	heme bind	Molecular	0.00	0.00	Solyc02g0	Solyc02g0	0.48	-1.05
GO:00200	heme bind	Molecular	0.00	0.00	Solyc04g0	Solyc04g0	0.45	-1.16
GO:00200	heme bind	Molecular	0.00	0.00	Solyc02g0	Solyc02g0	0.17	-2.56
GO:00200	heme bind	Molecular	0.00	0.00	Solyc06g0	Solyc06g0	0.00	-10.22
GO:00200	heme bind	Molecular	0.00	0.00	Solyc01g0	Solyc01g0	0.02	-5.72
GO:00200	heme bind	Molecular	0.00	0.00	Solyc03g0	Solyc03g0	0.41	-1.30
GO:00200	heme bind	Molecular	0.00	0.00	Solyc08g0	Solyc08g0	1235.43	10.27
GO:00200	heme bind	Molecular	0.00	0.00	Solyc09g0	Solyc09g0	54.33	5.76
GO:00200	heme bind	Molecular	0.00	0.00	Solyc01g0	Solyc01g0	3.59	1.84
GO:00200	heme bind	Molecular	0.00	0.00	Solyc04g0	Solyc04g0	900.23	9.81
GO:00200	heme bind	Molecular	0.00	0.00	Solyc06g0	Solyc06g0	17.36	4.12
GO:00200	heme bind	Molecular	0.00	0.00	Solyc07g0	Solyc07g0	2327.11	11.18
GO:00200	heme bind	Molecular	0.00	0.00	Solyc02g0	Solyc02g0	0.28	-1.83
GO:00200	heme bind	Molecular	0.00	0.00	Solyc04g0	Solyc04g0	0.48	-1.06
GO:00200	heme bind	Molecular	0.00	0.00	Solyc06g0	Solyc06g0	0.25	-2.01
GO:00200	heme bind	Molecular	0.00	0.00	Solyc05g0	Solyc05g0	2.42	1.27
GO:00200	heme bind	Molecular	0.00	0.00	Solyc12g0	Solyc12g0	1446.11	10.50
GO:00200	heme bind	Molecular	0.00	0.00	Solyc02g0	Solyc02g0	3.18	1.67
GO:00200	heme bind	Molecular	0.00	0.00	Solyc01g1	Solyc01g1	0.47	-1.09
GO:00200	heme bind	Molecular	0.00	0.00	Solyc07g0	Solyc07g0	9.07	3.18
GO:00200	heme bind	Molecular	0.00	0.00	Solyc11g0	Solyc11g0	0.00	-9.24
GO:00200	heme bind	Molecular	0.00	0.00	Solyc12g0	Solyc12g0	0.00	-9.92
GO:00200	heme bind	Molecular	0.00	0.00	Solyc08g0	Solyc08g0	0.34	-1.54
GO:00200	heme bind	Molecular	0.00	0.00	Solyc01g0	Solyc01g0	13.05	3.71
GO:00200	heme bind	Molecular	0.00	0.00	Solyc07g0	Solyc07g0	0.50	-1.01
GO:00200	heme bind	Molecular	0.00	0.00	Solyc04g0	Solyc04g0	9.75	3.29
GO:00200	heme bind	Molecular	0.00	0.00	Solyc01g0	Solyc01g0	0.48	-1.07
GO:00200	heme bind	Molecular	0.00	0.00	Solyc07g0	Solyc07g0	159.10	7.31
GO:00200	heme bind	Molecular	0.00	0.00	Solyc11g0	Solyc11g0	0.09	-3.52
GO:00200	heme bind	Molecular	0.00	0.00	Solyc08g0	Solyc08g0	599.38	9.23
GO:00200	heme bind	Molecular	0.00	0.00	Solyc10g0	Solyc10g0	0.00	-9.79
GO:00200	heme bind	Molecular	0.00	0.00	Solyc01g1	Solyc01g1	4.58	2.20
GO:00200	heme bind	Molecular	0.00	0.00	Solyc04g0	Solyc04g0	26.73	4.74
GO:00200	heme bind	Molecular	0.00	0.00	Solyc07g0	Solyc07g0	2.87	1.52
GO:00055	extracellul	Cellular C	0.00	0.00	Solyc10g0	PG2	9.48	3.25
GO:00055	extracellul	Cellular C	0.00	0.00	Solyc01g1	Solyc01g1	4.83	2.27
GO:00055	extracellul	Cellular C	0.00	0.00	Solyc07g0	Solyc07g0	6.72	2.75
GO:00055	extracellul	Cellular C	0.00	0.00	Solyc10g0	CHI9	0.28	-1.84
GO:00055	extracellul	Cellular C	0.00	0.00	Solyc01g1	Solyc01g1	3.25	1.70
GO:00055	extracellul	Cellular C	0.00	0.00	Solyc04g0	Solyc04g0	3.38	1.76
GO:00055	extracellul	Cellular C	0.00	0.00	Solyc01g0	Solyc01g0	15.46	3.95
GO:00055	extracellul	Cellular C	0.00	0.00	Solyc06g0	Solyc06g0	12.59	3.65
GO:00055	extracellul	Cellular C	0.00	0.00	Solyc12g0	Solyc12g0	2.75	1.46
GO:00055	extracellul	Cellular C	0.00	0.00	Solyc01g1	Solyc01g1	2.90	1.54
GO:00055	extracellul	Cellular C	0.00	0.00	Solyc02g0	Solyc02g0	2.85	1.51

GO:00055	extracellul	Cellular Co	0.00	0.00	Solyc01g0	XTH1	5.24	2.39
GO:00055	extracellul	Cellular Co	0.00	0.00	Solyc08g0	Solyc08g0	2.18	1.12
GO:00055	extracellul	Cellular Co	0.00	0.00	Solyc12g0	Solyc12g0	2.15	1.10
GO:00055	extracellul	Cellular Co	0.00	0.00	Solyc02g0	Solyc02g0	0.33	-1.59
GO:00055	extracellul	Cellular Co	0.00	0.00	Solyc01g0	Solyc01g0	0.06	-3.97
GO:00055	extracellul	Cellular Co	0.00	0.00	Solyc05g0	Solyc05g0	0.35	-1.52
GO:00055	extracellul	Cellular Co	0.00	0.00	Solyc09g0	Solyc09g0	10879.94	13.41
GO:00055	extracellul	Cellular Co	0.00	0.00	Solyc03g1	Solyc03g1	0.44	-1.20
GO:00055	extracellul	Cellular Co	0.00	0.00	Solyc02g0	Solyc02g0	4235.90	12.05
GO:00055	extracellul	Cellular Co	0.00	0.00	Solyc03g0	Solyc03g0	5511.05	12.43
GO:00055	extracellul	Cellular Co	0.00	0.00	Solyc06g0	Solyc06g0	0.11	-3.25
GO:00055	extracellul	Cellular Co	0.00	0.00	Solyc01g1	Solyc01g1	4.37	2.13
GO:00055	extracellul	Cellular Co	0.00	0.00	Solyc02g0	GAST1	0.35	-1.53
GO:00055	extracellul	Cellular Co	0.00	0.00	Solyc06g0	Solyc06g0	0.25	-2.01
GO:00055	extracellul	Cellular Co	0.00	0.00	Solyc01g0	Solyc01g0	0.31	-1.69
GO:00055	extracellul	Cellular Co	0.00	0.00	Solyc03g1	Solyc03g1	0.15	-2.75
GO:00055	extracellul	Cellular Co	0.00	0.00	Solyc02g0	Solyc02g0	13.99	3.81
GO:00055	extracellul	Cellular Co	0.00	0.00	Solyc06g0	Solyc06g0	32.34	5.02
GO:00055	extracellul	Cellular Co	0.00	0.00	Solyc07g0	Solyc07g0	2.40	1.26
GO:00055	extracellul	Cellular Co	0.00	0.00	Solyc03g0	Solyc03g0	11.28	3.50
GO:00055	extracellul	Cellular Co	0.00	0.00	Solyc05g0	Solyc05g0	20.96	4.39
GO:00055	extracellul	Cellular Co	0.00	0.00	Solyc11g0	Solyc11g0	2317.83	11.18
GO:00055	extracellul	Cellular Co	0.00	0.00	Solyc08g0	Solyc08g0	11.44	3.52
GO:00055	extracellul	Cellular Co	0.00	0.00	Solyc11g0	Solyc11g0	0.43	-1.23
GO:00055	extracellul	Cellular Co	0.00	0.00	Solyc07g0	Solyc07g0	2.87	1.52
GO:00167	hydrolase a	Molecular	0.00	0.00	Solyc10g0	PG2	9.48	3.25
GO:00167	hydrolase a	Molecular	0.00	0.00	Solyc01g1	Solyc01g1	4.83	2.27
GO:00167	hydrolase a	Molecular	0.00	0.00	Solyc04g0	Solyc04g0	6.77	2.76
GO:00167	hydrolase a	Molecular	0.00	0.00	Solyc09g0	Solyc09g0	0.34	-1.54
GO:00167	hydrolase a	Molecular	0.00	0.00	Solyc08g0	Solyc08g0	3.70	1.89
GO:00167	hydrolase a	Molecular	0.00	0.00	Solyc01g0	Solyc01g0	2.01	1.01
GO:00167	hydrolase a	Molecular	0.00	0.00	Solyc03g1	Solyc03g1	2.16	1.11
GO:00167	hydrolase a	Molecular	0.00	0.00	Solyc01g1	Solyc01g1	0.44	-1.20
GO:00167	hydrolase a	Molecular	0.00	0.00	Solyc03g1	Solyc03g1	2.20	1.14
GO:00167	hydrolase a	Molecular	0.00	0.00	Solyc02g0	Solyc02g0	0.34	-1.57
GO:00167	hydrolase a	Molecular	0.00	0.00	Solyc10g0	CHI9	0.28	-1.84
GO:00167	hydrolase a	Molecular	0.00	0.00	Solyc01g1	Solyc01g1	3.25	1.70
GO:00167	hydrolase a	Molecular	0.00	0.00	Solyc04g0	Solyc04g0	3.38	1.76
GO:00167	hydrolase a	Molecular	0.00	0.00	Solyc01g0	Solyc01g0	15.46	3.95
GO:00167	hydrolase a	Molecular	0.00	0.00	Solyc06g0	Solyc06g0	2.40	1.26
GO:00167	hydrolase a	Molecular	0.00	0.00	Solyc04g0	Solyc04g0	2.29	1.19
GO:00167	hydrolase a	Molecular	0.00	0.00	Solyc01g0	Solyc01g0	2.16	1.11
GO:00167	hydrolase a	Molecular	0.00	0.00	Solyc06g0	Solyc06g0	0.26	-1.97
GO:00167	hydrolase a	Molecular	0.00	0.00	Solyc08g0	Solyc08g0	6.25	2.64
GO:00167	hydrolase a	Molecular	0.00	0.00	Solyc08g0	Solyc08g0	0.41	-1.27
GO:00167	hydrolase a	Molecular	0.00	0.00	Solyc01g0	XTH1	5.24	2.39
GO:00167	hydrolase a	Molecular	0.00	0.00	Solyc11g0	Solyc11g0	2.36	1.24
GO:00167	hydrolase a	Molecular	0.00	0.00	Solyc08g0	Solyc08g0	2.18	1.12
GO:00167	hydrolase a	Molecular	0.00	0.00	Solyc03g0	Solyc03g0	2.12	1.09
GO:00167	hydrolase a	Molecular	0.00	0.00	Solyc07g0	PDF1A	4.20	2.07
GO:00167	hydrolase a	Molecular	0.00	0.00	Solyc05g0	Solyc05g0	3.20	1.68

GO:00167	hydrolase a	Molecular	0.00	0.00	Solyc02g0	Solyc02g0	0.33	-1.59
GO:00167	hydrolase a	Molecular	0.00	0.00	Solyc02g0	Solyc02g0	19.98	4.32
GO:00167	hydrolase a	Molecular	0.00	0.00	Solyc08g0	Solyc08g0	0.43	-1.21
GO:00167	hydrolase a	Molecular	0.00	0.00	Solyc03g0	Solyc03g0	0.22	-2.16
GO:00167	hydrolase a	Molecular	0.00	0.00	Solyc09g0	Solyc09g0	0.36	-1.46
GO:00167	hydrolase a	Molecular	0.00	0.00	Solyc09g0	Solyc09g0	0.12	-3.08
GO:00167	hydrolase a	Molecular	0.00	0.00	Solyc01g0	Solyc01g0	2.38	1.25
GO:00167	hydrolase a	Molecular	0.00	0.00	Solyc07g0	Solyc07g0	2.09	1.06
GO:00167	hydrolase a	Molecular	0.00	0.00	Solyc02g0	Solyc02g0	2.28	1.19
GO:00167	hydrolase a	Molecular	0.00	0.00	Solyc03g0	Solyc03g0	3.84	1.94
GO:00167	hydrolase a	Molecular	0.00	0.00	Solyc10g0	Solyc10g0	11.34	3.50
GO:00167	hydrolase a	Molecular	0.00	0.00	Solyc03g1	Solyc03g1	0.44	-1.20
GO:00167	hydrolase a	Molecular	0.00	0.00	Solyc04g0	Solyc04g0	0.41	-1.30
GO:00167	hydrolase a	Molecular	0.00	0.00	Solyc09g0	Solyc09g0	2.35	1.23
GO:00167	hydrolase a	Molecular	0.00	0.00	Solyc04g0	Solyc04g0	0.41	-1.30
GO:00167	hydrolase a	Molecular	0.00	0.00	Solyc01g0	Solyc01g0	0.48	-1.06
GO:00167	hydrolase a	Molecular	0.00	0.00	Solyc03g0	Solyc03g0	0.02	-5.67
GO:00167	hydrolase a	Molecular	0.00	0.00	Solyc07g0	Solyc07g0	4.69	2.23
GO:00167	hydrolase a	Molecular	0.00	0.00	Solyc03g0	Solyc03g0	1566.99	10.61
GO:00167	hydrolase a	Molecular	0.00	0.00	Solyc09g0	Solyc09g0	617.25	9.27
GO:00167	hydrolase a	Molecular	0.00	0.00	Solyc04g0	Solyc04g0	8.84	3.14
GO:00167	hydrolase a	Molecular	0.00	0.00	Solyc12g0	Solyc12g0	1818.26	10.83
GO:00167	hydrolase a	Molecular	0.00	0.00	Solyc08g0	Solyc08g0	2.80	1.49
GO:00167	hydrolase a	Molecular	0.00	0.00	Solyc12g0	Solyc12g0	0.39	-1.35
GO:00167	hydrolase a	Molecular	0.00	0.00	Solyc06g0	Solyc06g0	15.51	3.95
GO:00167	hydrolase a	Molecular	0.00	0.00	Solyc06g0	Solyc06g0	0.38	-1.41
GO:00167	hydrolase a	Molecular	0.00	0.00	Solyc06g0	Solyc06g0	32.34	5.02
GO:00167	hydrolase a	Molecular	0.00	0.00	Solyc07g0	Solyc07g0	2.40	1.26
GO:00167	hydrolase a	Molecular	0.00	0.00	Solyc07g0	Solyc07g0	11.18	3.48
GO:00167	hydrolase a	Molecular	0.00	0.00	Solyc05g0	Solyc05g0	20.96	4.39
GO:00167	hydrolase a	Molecular	0.00	0.00	Solyc01g0	Solyc01g0	0.48	-1.06
GO:00167	hydrolase a	Molecular	0.00	0.00	Solyc07g0	Solyc07g0	470.92	8.88
GO:00167	hydrolase a	Molecular	0.00	0.00	Solyc05g0	Solyc05g0	4.80	2.26
GO:00125	monolayer	Cellular Co	0.00	0.00	Solyc06g0	Solyc06g0	12.59	3.65
GO:00125	monolayer	Cellular Co	0.00	0.00	Solyc07g0	Solyc07g0	5.25	2.39
GO:00125	monolayer	Cellular Co	0.00	0.00	Solyc03g1	Solyc03g1	10.69	3.42
GO:00125	monolayer	Cellular Co	0.00	0.00	Solyc06g0	Solyc06g0	18.67	4.22
GO:00125	monolayer	Cellular Co	0.00	0.00	Solyc12g0	Solyc12g0	14.44	3.85
GO:00125	monolayer	Cellular Co	0.00	0.00	Solyc08g0	Solyc08g0	11.44	3.52
GO:00182	protein-chr	Biological	0.00	0.00	Solyc09g0	Solyc09g0	0.40	-1.34
GO:00182	protein-chr	Biological	0.00	0.00	Solyc09g0	Solyc09g0	5.85	2.55
GO:00182	protein-chr	Biological	0.00	0.00	Solyc10g0	Solyc10g0	4.66	2.22
GO:00182	protein-chr	Biological	0.00	0.00	Solyc12g0	Solyc12g0	0.00	-11.26
GO:00182	protein-chr	Biological	0.00	0.00	Solyc12g0	Solyc12g0	0.05	-4.20
GO:00182	protein-chr	Biological	0.00	0.00	Solyc10g0	Solyc10g0	0.00	-12.09
GO:00182	protein-chr	Biological	0.00	0.00	Solyc09g0	Solyc09g0	0.00	-13.14
GO:00182	protein-chr	Biological	0.00	0.00	Solyc12g0	Solyc12g0	2.29	1.20
GO:00182	protein-chr	Biological	0.00	0.00	Solyc11g0	Solyc11g0	0.00	-13.08
GO:00182	protein-chr	Biological	0.00	0.00	Solyc10g0	Solyc10g0	2.07	1.05
GO:00182	protein-chr	Biological	0.00	0.00	Solyc03g0	Solyc03g0	2.60	1.38
GO:00182	protein-chr	Biological	0.00	0.00	Solyc03g0	Solyc03g0	3.69	1.88

GO:00182	protein-ch	Biological	0.00	0.00	Solyc12g0	Solyc12g0	0.25	-2.01
GO:00512	dioxygena	Molecular	0.00	0.00	Solyc07g0	ACO1	2.82	1.49
GO:00512	dioxygena	Molecular	0.00	0.00	Solyc02g0	Solyc02g0	0.08	-3.73
GO:00512	dioxygena	Molecular	0.00	0.00	Solyc01g0	Solyc01g0	2.18	1.13
GO:00512	dioxygena	Molecular	0.00	0.00	Solyc11g0	Solyc11g0	0.28	-1.84
GO:00512	dioxygena	Molecular	0.00	0.00	Solyc10g0	Solyc10g0	65.27	6.03
GO:00512	dioxygena	Molecular	0.00	0.00	Solyc06g0	Solyc06g0	6.89	2.78
GO:00512	dioxygena	Molecular	0.00	0.00	Solyc08g0	LOX1.1	2.60	1.38
GO:00512	dioxygena	Molecular	0.00	0.00	Solyc07g0	Solyc07g0	2.20	1.14
GO:00512	dioxygena	Molecular	0.00	0.00	Solyc03g0	Solyc03g0	3.90	1.96
GO:00512	dioxygena	Molecular	0.00	0.00	Solyc02g0	Solyc02g0	2.56	1.36
GO:00512	dioxygena	Molecular	0.00	0.00	Solyc09g0	Solyc09g0	0.20	-2.33
GO:00512	dioxygena	Molecular	0.00	0.00	Solyc07g0	Solyc07g0	4.00	2.00
GO:00512	dioxygena	Molecular	0.00	0.00	Solyc12g0	Solyc12g0	2.73	1.45
GO:00512	dioxygena	Molecular	0.00	0.00	Solyc10g0	Solyc10g0	2907.35	11.51
GO:00512	dioxygena	Molecular	0.00	0.00	Solyc06g0	Solyc06g0	3.27	1.71
GO:00512	dioxygena	Molecular	0.00	0.00	Solyc07g0	Solyc07g0	29.42	4.88
GO:00512	dioxygena	Molecular	0.00	0.00	Solyc09g0	Solyc09g0	1048.85	10.03
GO:00512	dioxygena	Molecular	0.00	0.00	Solyc12g0	Solyc12g0	3.66	1.87
GO:00512	dioxygena	Molecular	0.00	0.00	Solyc01g0	Solyc01g0	902.52	9.82
GO:00451	electron tr	Molecular	0.00	0.00	Solyc05g0	Solyc05g0	0.01	-6.09
GO:00451	electron tr	Molecular	0.00	0.00	Solyc01g0	Solyc01g0	0.01	-6.32
GO:00451	electron tr	Molecular	0.00	0.00	Solyc12g0	Solyc12g0	0.01	-7.31
GO:00451	electron tr	Molecular	0.00	0.00	Solyc02g0	Solyc02g0	0.01	-6.27
GO:00451	electron tr	Molecular	0.00	0.00	Solyc02g0	Solyc02g0	3.41	1.77
GO:00451	electron tr	Molecular	0.00	0.00	Solyc09g0	Solyc09g0	0.00	-13.14
GO:00451	electron tr	Molecular	0.00	0.00	Solyc03g1	Solyc03g1	0.10	-3.25
GO:00451	electron tr	Molecular	0.00	0.00	Solyc05g0	Solyc05g0	0.00	-12.71
GO:00451	electron tr	Molecular	0.00	0.00	Solyc11g0	Solyc11g0	0.00	-12.69
GO:00161	chlorophyl	Molecular	0.00	0.00	Solyc09g0	Solyc09g0	5.85	2.55
GO:00161	chlorophyl	Molecular	0.00	0.00	Solyc10g0	Solyc10g0	4.66	2.22
GO:00161	chlorophyl	Molecular	0.00	0.00	Solyc12g0	Solyc12g0	0.00	-11.26
GO:00161	chlorophyl	Molecular	0.00	0.00	Solyc12g0	Solyc12g0	0.05	-4.20
GO:00161	chlorophyl	Molecular	0.00	0.00	Solyc10g0	Solyc10g0	0.00	-12.09
GO:00161	chlorophyl	Molecular	0.00	0.00	Solyc09g0	Solyc09g0	0.00	-13.14
GO:00161	chlorophyl	Molecular	0.00	0.00	Solyc11g0	Solyc11g0	0.00	-13.08
GO:00161	chlorophyl	Molecular	0.00	0.00	Solyc10g0	Solyc10g0	2.07	1.05
GO:00161	chlorophyl	Molecular	0.00	0.00	Solyc03g0	Solyc03g0	2.60	1.38
GO:00161	chlorophyl	Molecular	0.00	0.00	Solyc03g0	Solyc03g0	3.69	1.88
GO:00161	chlorophyl	Molecular	0.00	0.00	Solyc12g0	Solyc12g0	0.25	-2.01
GO:00161	chlorophyl	Molecular	0.00	0.00	Solyc02g0	Solyc02g0	0.43	-1.21
GO:00167	hydrolase	Molecular	0.00	0.00	Solyc10g0	PG2	9.48	3.25
GO:00167	hydrolase	Molecular	0.00	0.00	Solyc01g1	Solyc01g1	4.83	2.27
GO:00167	hydrolase	Molecular	0.00	0.00	Solyc09g0	Solyc09g0	0.34	-1.54
GO:00167	hydrolase	Molecular	0.00	0.00	Solyc01g0	Solyc01g0	2.01	1.01
GO:00167	hydrolase	Molecular	0.00	0.00	Solyc03g1	Solyc03g1	2.20	1.14
GO:00167	hydrolase	Molecular	0.00	0.00	Solyc10g0	CHI9	0.28	-1.84
GO:00167	hydrolase	Molecular	0.00	0.00	Solyc04g0	Solyc04g0	3.38	1.76
GO:00167	hydrolase	Molecular	0.00	0.00	Solyc01g0	Solyc01g0	15.46	3.95
GO:00167	hydrolase	Molecular	0.00	0.00	Solyc06g0	Solyc06g0	2.47	1.31
GO:00167	hydrolase	Molecular	0.00	0.00	Solyc08g0	Solyc08g0	6.25	2.64

GO:00167	hydrolase a	Molecular	0.00	0.00	Solyc01g0	XTH1	5.24	2.39
GO:00167	hydrolase a	Molecular	0.00	0.00	Solyc08g0	Solyc08g0	2.18	1.12
GO:00167	hydrolase a	Molecular	0.00	0.00	Solyc02g0	Solyc02g0	0.33	-1.59
GO:00167	hydrolase a	Molecular	0.00	0.00	Solyc09g0	Solyc09g0	0.36	-1.46
GO:00167	hydrolase a	Molecular	0.00	0.00	Solyc03g0	Solyc03g0	3.84	1.94
GO:00167	hydrolase a	Molecular	0.00	0.00	Solyc09g0	Solyc09g0	2.14	1.10
GO:00167	hydrolase a	Molecular	0.00	0.00	Solyc04g0	Solyc04g0	8.84	3.14
GO:00167	hydrolase a	Molecular	0.00	0.00	Solyc06g0	Solyc06g0	32.34	5.02
GO:00044	monooxyg	Molecular	0.00	0.00	Solyc01g1	Solyc01g1	11.39	3.51
GO:00044	monooxyg	Molecular	0.00	0.00	Solyc11g0	AOS2	3.20	1.68
GO:00044	monooxyg	Molecular	0.00	0.00	Solyc01g0	Solyc01g0	0.03	-5.12
GO:00044	monooxyg	Molecular	0.00	0.00	Solyc04g0	Solyc04g0	2.89	1.53
GO:00044	monooxyg	Molecular	0.00	0.00	Solyc07g0	Solyc07g0	0.32	-1.63
GO:00044	monooxyg	Molecular	0.00	0.00	Solyc10g0	Solyc10g0	0.40	-1.34
GO:00044	monooxyg	Molecular	0.00	0.00	Solyc03g1	Solyc03g1	0.30	-1.75
GO:00044	monooxyg	Molecular	0.00	0.00	Solyc06g0	Solyc06g0	7.48	2.90
GO:00044	monooxyg	Molecular	0.00	0.00	Solyc03g1	Solyc03g1	4.61	2.21
GO:00044	monooxyg	Molecular	0.00	0.00	Solyc04g0	Solyc04g0	6.45	2.69
GO:00044	monooxyg	Molecular	0.00	0.00	Solyc01g1	Solyc01g1	0.03	-5.02
GO:00044	monooxyg	Molecular	0.00	0.00	Solyc05g0	Solyc05g0	2.81	1.49
GO:00044	monooxyg	Molecular	0.00	0.00	Solyc08g0	Solyc08g0	2.39	1.25
GO:00044	monooxyg	Molecular	0.00	0.00	Solyc01g0	Solyc01g0	5.13	2.36
GO:00044	monooxyg	Molecular	0.00	0.00	Solyc06g0	Solyc06g0	3.53	1.82
GO:00044	monooxyg	Molecular	0.00	0.00	Solyc01g0	Solyc01g0	6.67	2.74
GO:00044	monooxyg	Molecular	0.00	0.00	Solyc08g0	Solyc08g0	4598.17	12.17
GO:00044	monooxyg	Molecular	0.00	0.00	Solyc01g0	Solyc01g0	3528.16	11.78
GO:00044	monooxyg	Molecular	0.00	0.00	Solyc09g0	Solyc09g0	51.95	5.70
GO:00044	monooxyg	Molecular	0.00	0.00	Solyc08g0	Solyc08g0	4039.84	11.98
GO:00044	monooxyg	Molecular	0.00	0.00	Solyc01g0	Solyc01g0	3.21	1.68
GO:00044	monooxyg	Molecular	0.00	0.00	Solyc02g0	Solyc02g0	0.48	-1.05
GO:00044	monooxyg	Molecular	0.00	0.00	Solyc04g0	Solyc04g0	0.45	-1.16
GO:00044	monooxyg	Molecular	0.00	0.00	Solyc03g0	RBCS-2A	2.71	1.44
GO:00044	monooxyg	Molecular	0.00	0.00	Solyc06g0	Solyc06g0	0.00	-10.22
GO:00044	monooxyg	Molecular	0.00	0.00	Solyc08g0	Solyc08g0	1235.43	10.27
GO:00044	monooxyg	Molecular	0.00	0.00	Solyc09g0	Solyc09g0	54.33	5.76
GO:00044	monooxyg	Molecular	0.00	0.00	Solyc04g0	Solyc04g0	900.23	9.81
GO:00044	monooxyg	Molecular	0.00	0.00	Solyc06g0	Solyc06g0	17.36	4.12
GO:00044	monooxyg	Molecular	0.00	0.00	Solyc02g0	Solyc02g0	0.28	-1.83
GO:00044	monooxyg	Molecular	0.00	0.00	Solyc05g0	Solyc05g0	2.42	1.27
GO:00044	monooxyg	Molecular	0.00	0.00	Solyc12g0	Solyc12g0	1446.11	10.50
GO:00044	monooxyg	Molecular	0.00	0.00	Solyc02g0	Solyc02g0	3.18	1.67
GO:00044	monooxyg	Molecular	0.00	0.00	Solyc01g1	Solyc01g1	0.47	-1.09
GO:00044	monooxyg	Molecular	0.00	0.00	Solyc07g0	Solyc07g0	9.07	3.18
GO:00044	monooxyg	Molecular	0.00	0.00	Solyc11g0	Solyc11g0	0.00	-9.24
GO:00044	monooxyg	Molecular	0.00	0.00	Solyc12g0	Solyc12g0	0.00	-9.92
GO:00044	monooxyg	Molecular	0.00	0.00	Solyc08g0	Solyc08g0	0.34	-1.54
GO:00044	monooxyg	Molecular	0.00	0.00	Solyc01g0	Solyc01g0	13.05	3.71
GO:00044	monooxyg	Molecular	0.00	0.00	Solyc07g0	Solyc07g0	0.50	-1.01
GO:00044	monooxyg	Molecular	0.00	0.00	Solyc04g0	Solyc04g0	9.75	3.29
GO:00044	monooxyg	Molecular	0.00	0.00	Solyc01g0	Solyc01g0	1566.92	10.61
GO:00044	monooxyg	Molecular	0.00	0.00	Solyc07g0	Solyc07g0	159.10	7.31

GO:00044	monooxyg	Molecular	0.00	0.00	Solyc08g0	Solyc08g0	599.38	9.23
GO:00044	monooxyg	Molecular	0.00	0.00	Solyc10g0	Solyc10g0	0.00	-9.79
GO:00044	monooxyg	Molecular	0.00	0.00	Solyc04g0	Solyc04g0	26.73	4.74
GO:00066	lipid metal	Biological	0.00	0.00	Solyc01g0	Solyc01g0	0.09	-3.43
GO:00066	lipid metal	Biological	0.00	0.00	Solyc12g0	Solyc12g0	0.32	-1.65
GO:00066	lipid metal	Biological	0.00	0.00	Solyc01g0	Solyc01g0	2.18	1.13
GO:00066	lipid metal	Biological	0.00	0.00	Solyc07g0	Solyc07g0	2.77	1.47
GO:00066	lipid metal	Biological	0.00	0.00	Solyc08g0	Solyc08g0	4.51	2.17
GO:00066	lipid metal	Biological	0.00	0.00	Solyc11g0	AOS2	3.20	1.68
GO:00066	lipid metal	Biological	0.00	0.00	Solyc01g1	Solyc01g1	0.44	-1.20
GO:00066	lipid metal	Biological	0.00	0.00	Solyc08g0	LOX1.1	2.60	1.38
GO:00066	lipid metal	Biological	0.00	0.00	Solyc06g0	Solyc06g0	2.40	1.26
GO:00066	lipid metal	Biological	0.00	0.00	Solyc11g0	Solyc11g0	2.05	1.04
GO:00066	lipid metal	Biological	0.00	0.00	Solyc03g1	Solyc03g1	2.42	1.27
GO:00066	lipid metal	Biological	0.00	0.00	Solyc06g0	Solyc06g0	0.41	-1.30
GO:00066	lipid metal	Biological	0.00	0.00	Solyc05g0	Solyc05g0	5.36	2.42
GO:00066	lipid metal	Biological	0.00	0.00	Solyc06g0	Solyc06g0	0.40	-1.31
GO:00066	lipid metal	Biological	0.00	0.00	Solyc03g1	Solyc03g1	4.55	2.18
GO:00066	lipid metal	Biological	0.00	0.00	Solyc01g0	Solyc01g0	2.38	1.25
GO:00066	lipid metal	Biological	0.00	0.00	Solyc04g0	Solyc04g0	0.00	-13.91
GO:00066	lipid metal	Biological	0.00	0.00	Solyc10g0	Solyc10g0	11.34	3.50
GO:00066	lipid metal	Biological	0.00	0.00	Solyc02g0	Solyc02g0	2.24	1.17
GO:00066	lipid metal	Biological	0.00	0.00	Solyc12g0	Solyc12g0	2.04	1.03
GO:00066	lipid metal	Biological	0.00	0.00	Solyc12g0	Solyc12g0	2.73	1.45
GO:00066	lipid metal	Biological	0.00	0.00	Solyc10g0	Solyc10g0	2.03	1.02
GO:00066	lipid metal	Biological	0.00	0.00	Solyc06g0	Solyc06g0	2.13	1.09
GO:00066	lipid metal	Biological	0.00	0.00	Solyc12g0	Solyc12g0	0.47	-1.10
GO:00066	lipid metal	Biological	0.00	0.00	Solyc03g0	Solyc03g0	627.68	9.29
GO:00066	lipid metal	Biological	0.00	0.00	Solyc02g0	Solyc02g0	0.35	-1.51
GO:00066	lipid metal	Biological	0.00	0.00	Solyc12g1	Solyc12g1	0.00	-9.86
GO:00066	lipid metal	Biological	0.00	0.00	Solyc04g0	Solyc04g0	9.52	3.25
GO:00066	lipid metal	Biological	0.00	0.00	Solyc09g0	Solyc09g0	5.06	2.34
GO:00097	photosynth	Biological	0.00	0.00	Solyc05g0	Solyc05g0	0.01	-6.09
GO:00097	photosynth	Biological	0.00	0.00	Solyc01g0	Solyc01g0	0.01	-6.32
GO:00097	photosynth	Biological	0.00	0.00	Solyc12g0	Solyc12g0	2.97	1.57
GO:00097	photosynth	Biological	0.00	0.00	Solyc12g0	Solyc12g0	0.01	-7.31
GO:00097	photosynth	Biological	0.00	0.00	Solyc02g0	Solyc02g0	0.01	-6.27
GO:00097	photosynth	Biological	0.00	0.00	Solyc09g0	Solyc09g0	0.00	-13.14
GO:00097	photosynth	Biological	0.00	0.00	Solyc05g0	Solyc05g0	0.00	-12.71
GO:00097	photosynth	Biological	0.00	0.00	Solyc11g0	Solyc11g0	0.00	-12.69
GO:00168	ammonia-l	Molecular	0.00	0.00	Solyc10g0	Solyc10g0	4.26	2.09
GO:00168	ammonia-l	Molecular	0.00	0.00	Solyc09g0	Solyc09g0	0.27	-1.91
GO:00168	ammonia-l	Molecular	0.00	0.00	Solyc09g0	Solyc09g0	0.34	-1.54
GO:00168	ammonia-l	Molecular	0.00	0.00	Solyc10g0	Solyc10g0	2.14	1.09
GO:00168	ammonia-l	Molecular	0.00	0.00	Solyc03g0	Solyc03g0	794.30	9.63
GO:00098	flavonoid l	Biological	0.00	0.00	Solyc02g0	Solyc02g0	0.08	-3.73
GO:00098	flavonoid l	Biological	0.00	0.00	Solyc05g0	Solyc05g0	0.06	-4.07
GO:00098	flavonoid l	Biological	0.00	0.00	Solyc11g0	Solyc11g0	0.28	-1.84
GO:00098	flavonoid l	Biological	0.00	0.00	Solyc09g0	Solyc09g0	0.06	-4.05
GO:00098	flavonoid l	Biological	0.00	0.00	Solyc05g0	Solyc05g0	3.96	1.99
GO:00305	structural c	Molecular	0.00	0.01	Solyc01g0	Solyc01g0	2.68	1.42

GO:00305	structural c	Molecular	0.00	0.01	Solyc01g0	Solyc01g0	3.72	1.90
GO:00305	structural c	Molecular	0.00	0.01	Solyc02g0	Solyc02g0	2.01	1.01
GO:00305	structural c	Molecular	0.00	0.01	Solyc04g0	Solyc04g0	2.47	1.30
GO:00305	structural c	Molecular	0.00	0.01	Solyc11g0	Solyc11g0	2.31	1.21
GO:00305	structural c	Molecular	0.00	0.01	Solyc06g0	Solyc06g0	2.48	1.31
GO:00305	structural c	Molecular	0.00	0.01	Solyc01g0	Solyc01g0	3.17	1.66
GO:00305	structural c	Molecular	0.00	0.01	Solyc01g0	Solyc01g0	2.30	1.20
GO:00305	structural c	Molecular	0.00	0.01	Solyc05g0	Solyc05g0	2.76	1.47
GO:00095	chloroplast	Cellular Co	0.00	0.01	Solyc06g0	Solyc06g0	2.52	1.33
GO:00095	chloroplast	Cellular Co	0.00	0.01	Solyc04g0	Solyc04g0	2.70	1.43
GO:00095	chloroplast	Cellular Co	0.00	0.01	Solyc11g0	Solyc11g0	3.25	1.70
GO:00095	chloroplast	Cellular Co	0.00	0.01	Solyc09g0	Solyc09g0	7.18	2.84
GO:00095	chloroplast	Cellular Co	0.00	0.01	Solyc02g0	Solyc02g0	2.62	1.39
GO:00095	chloroplast	Cellular Co	0.00	0.01	Solyc07g0	PDF1A	4.20	2.07
GO:00095	chloroplast	Cellular Co	0.00	0.01	Solyc10g0	Solyc10g0	2.18	1.13
GO:00095	chloroplast	Cellular Co	0.00	0.01	Solyc03g1	Solyc03g1	4.55	2.18
GO:00095	chloroplast	Cellular Co	0.00	0.01	Solyc06g0	Solyc06g0	2.19	1.13
GO:00095	chloroplast	Cellular Co	0.00	0.01	Solyc09g0	Solyc09g0	0.12	-3.08
GO:00095	chloroplast	Cellular Co	0.00	0.01	Solyc04g0	Solyc04g0	0.00	-11.98
GO:00095	chloroplast	Cellular Co	0.00	0.01	Solyc04g0	Solyc04g0	0.00	-13.91
GO:00095	chloroplast	Cellular Co	0.00	0.01	Solyc10g0	Solyc10g0	0.04	-4.70
GO:00095	chloroplast	Cellular Co	0.00	0.01	Solyc05g0	Solyc05g0	2.32	1.21
GO:00095	chloroplast	Cellular Co	0.00	0.01	Solyc01g0	Solyc01g0	34.25	5.10
GO:00095	chloroplast	Cellular Co	0.00	0.01	Solyc05g0	Solyc05g0	2.96	1.57
GO:00095	chloroplast	Cellular Co	0.00	0.01	Solyc04g0	Solyc04g0	2.74	1.46
GO:00095	chloroplast	Cellular Co	0.00	0.01	Solyc05g0	Solyc05g0	39.90	5.32
GO:00095	chloroplast	Cellular Co	0.00	0.01	Solyc10g0	Solyc10g0	0.00	-12.76
GO:00095	chloroplast	Cellular Co	0.00	0.01	Solyc09g0	Solyc09g0	25.41	4.67
GO:00095	chloroplast	Cellular Co	0.00	0.01	Solyc05g0	Solyc05g0	10.06	3.33
GO:00095	chloroplast	Cellular Co	0.00	0.01	Solyc10g0	Solyc10g0	2.07	1.05
GO:00095	chloroplast	Cellular Co	0.00	0.01	Solyc04g0	Solyc04g0	0.06	-3.97
GO:00095	chloroplast	Cellular Co	0.00	0.01	Solyc02g0	Solyc02g0	574.97	9.17
GO:00095	chloroplast	Cellular Co	0.00	0.01	Solyc01g0	Solyc01g0	550.66	9.11
GO:00166	oxidoreduc	Molecular	0.00	0.01	Solyc09g0	Solyc09g0	0.18	-2.50
GO:00166	oxidoreduc	Molecular	0.00	0.01	Solyc05g0	Solyc05g0	6.90	2.79
GO:00166	oxidoreduc	Molecular	0.00	0.01	Solyc03g0	Solyc03g0	0.11	-3.15
GO:00166	oxidoreduc	Molecular	0.00	0.01	Solyc10g0	Solyc10g0	0.00	-13.73
GO:00166	oxidoreduc	Molecular	0.00	0.01	Solyc06g0	Solyc06g0	0.05	-4.46
GO:00166	oxidoreduc	Molecular	0.00	0.01	Solyc02g0	Solyc02g0	0.19	-2.37
GO:00166	oxidoreduc	Molecular	0.00	0.01	Solyc01g0	Solyc01g0	0.00	-11.62
GO:00022	response to	Biological	0.00	0.01	Solyc07g0	ACO1	2.82	1.49
GO:00022	response to	Biological	0.00	0.01	Solyc02g0	Solyc02g0	0.08	-3.73
GO:00022	response to	Biological	0.00	0.01	Solyc07g0	Solyc07g0	2.20	1.14
GO:00022	response to	Biological	0.00	0.01	Solyc06g0	Solyc06g0	0.15	-2.75
GO:00022	response to	Biological	0.00	0.01	Solyc02g0	Solyc02g0	2.56	1.36
GO:00022	response to	Biological	0.00	0.01	Solyc07g0	Solyc07g0	4.00	2.00
GO:00022	response to	Biological	0.00	0.01	Solyc07g0	Solyc07g0	29.42	4.88
GO:00022	response to	Biological	0.00	0.01	Solyc06g0	Solyc06g0	444.70	8.80
GO:00096	photosyste	Cellular Co	0.00	0.01	Solyc07g0	Solyc07g0	4.02	2.01
GO:00096	photosyste	Cellular Co	0.00	0.01	Solyc09g0	Solyc09g0	7.18	2.84
GO:00096	photosyste	Cellular Co	0.00	0.01	Solyc07g0	Solyc07g0	3.28	1.71

GO:00096	photosyste	Cellular C	0.00	0.01	Solyc02g0	PSBO	5.02	2.33
GO:00096	photosyste	Cellular C	0.00	0.01	Solyc07g0	PSBR	3.05	1.61
GO:00096	photosyste	Cellular C	0.00	0.01	Solyc02g0	Solyc02g0	3.41	1.77
GO:00096	photosyste	Cellular C	0.00	0.01	Solyc04g0	Solyc04g0	5.19	2.37
GO:00096	photosyste	Cellular C	0.00	0.01	Solyc03g1	Solyc03g1	2.21	1.14
GO:00229	electron tr	Biological	0.00	0.01	Solyc04g0	PETE	2.69	1.43
GO:00229	electron tr	Biological	0.00	0.01	Solyc11g0	Solyc11g0	2.92	1.55
GO:00229	electron tr	Biological	0.00	0.01	Solyc05g0	Solyc05g0	0.08	-3.58
GO:00229	electron tr	Biological	0.00	0.01	Solyc11g0	Solyc11g0	0.32	-1.67
GO:00229	electron tr	Biological	0.00	0.01	Solyc11g0	Solyc11g0	2.20	1.14
GO:00229	electron tr	Biological	0.00	0.01	Solyc03g0	Solyc03g0	0.04	-4.63
GO:00229	electron tr	Biological	0.00	0.01	Solyc12g0	Solyc12g0	0.00	-11.26
GO:00229	electron tr	Biological	0.00	0.01	Solyc03g0	Solyc03g0	0.11	-3.15
GO:00229	electron tr	Biological	0.00	0.01	Solyc06g0	Solyc06g0	0.05	-4.46
GO:00229	electron tr	Biological	0.00	0.01	Solyc02g0	Solyc02g0	0.19	-2.37
GO:00229	electron tr	Biological	0.00	0.01	Solyc11g0	Solyc11g0	0.09	-3.52
GO:00229	electron tr	Biological	0.00	0.01	Solyc04g0	Solyc04g0	883.93	9.79
GO:00229	electron tr	Biological	0.00	0.01	Solyc01g0	Solyc01g0	0.00	-11.62
GO:00081	metabolic	Biological	0.00	0.01	Solyc10g0	PG2	9.48	3.25
GO:00081	metabolic	Biological	0.00	0.01	Solyc01g1	Solyc01g1	4.83	2.27
GO:00081	metabolic	Biological	0.00	0.01	Solyc09g0	Solyc09g0	0.34	-1.54
GO:00081	metabolic	Biological	0.00	0.01	Solyc01g0	Solyc01g0	2.01	1.01
GO:00081	metabolic	Biological	0.00	0.01	Solyc03g1	Solyc03g1	2.20	1.14
GO:00081	metabolic	Biological	0.00	0.01	Solyc10g0	CHI9	0.28	-1.84
GO:00081	metabolic	Biological	0.00	0.01	Solyc04g0	Solyc04g0	3.38	1.76
GO:00081	metabolic	Biological	0.00	0.01	Solyc01g0	Solyc01g0	15.46	3.95
GO:00081	metabolic	Biological	0.00	0.01	Solyc08g0	Solyc08g0	6.25	2.64
GO:00081	metabolic	Biological	0.00	0.01	Solyc01g0	XTH1	5.24	2.39
GO:00081	metabolic	Biological	0.00	0.01	Solyc08g0	Solyc08g0	2.18	1.12
GO:00081	metabolic	Biological	0.00	0.01	Solyc02g0	Solyc02g0	0.33	-1.59
GO:00081	metabolic	Biological	0.00	0.01	Solyc09g0	Solyc09g0	0.36	-1.46
GO:00081	metabolic	Biological	0.00	0.01	Solyc03g0	Solyc03g0	3.84	1.94
GO:00081	metabolic	Biological	0.00	0.01	Solyc04g0	Solyc04g0	8.84	3.14
GO:00081	metabolic	Biological	0.00	0.01	Solyc06g0	Solyc06g0	32.34	5.02
GO:00550	transmemb	Biological	0.00	0.01	Solyc04g0	Solyc04g0	0.18	-2.50
GO:00550	transmemb	Biological	0.00	0.01	Solyc01g1	Solyc01g1	5.77	2.53
GO:00550	transmemb	Biological	0.00	0.01	Solyc04g0	Solyc04g0	2.19	1.13
GO:00550	transmemb	Biological	0.00	0.01	Solyc12g0	Solyc12g0	4.94	2.30
GO:00550	transmemb	Biological	0.00	0.01	Solyc03g1	Solyc03g1	3.43	1.78
GO:00550	transmemb	Biological	0.00	0.01	Solyc02g0	Solyc02g0	2.09	1.06
GO:00550	transmemb	Biological	0.00	0.01	Solyc01g1	Solyc01g1	0.29	-1.80
GO:00550	transmemb	Biological	0.00	0.01	Solyc01g0	Solyc01g0	0.33	-1.61
GO:00550	transmemb	Biological	0.00	0.01	Solyc03g0	Solyc03g0	0.28	-1.86
GO:00550	transmemb	Biological	0.00	0.01	Solyc07g0	Solyc07g0	2.63	1.40
GO:00550	transmemb	Biological	0.00	0.01	Solyc05g0	Solyc05g0	2.41	1.27
GO:00550	transmemb	Biological	0.00	0.01	Solyc03g0	Solyc03g0	0.31	-1.67
GO:00550	transmemb	Biological	0.00	0.01	Solyc06g0	Solyc06g0	0.33	-1.58
GO:00550	transmemb	Biological	0.00	0.01	Solyc11g0	Solyc11g0	0.35	-1.51
GO:00550	transmemb	Biological	0.00	0.01	Solyc12g0	Solyc12g0	5.32	2.41
GO:00550	transmemb	Biological	0.00	0.01	Solyc06g0	Solyc06g0	2.35	1.23
GO:00550	transmemb	Biological	0.00	0.01	Solyc06g0	Solyc06g0	2.02	1.02

GO:00550	transmemb	Biological	0.00	0.01	Solyc04g0	Solyc04g0	0.41	-1.28
GO:00550	transmemb	Biological	0.00	0.01	Solyc01g1	Solyc01g1	3.01	1.59
GO:00550	transmemb	Biological	0.00	0.01	Solyc03g1	Solyc03g1	0.25	-2.02
GO:00550	transmemb	Biological	0.00	0.01	Solyc10g0	Solyc10g0	3.46	1.79
GO:00550	transmemb	Biological	0.00	0.01	Solyc10g0	Solyc10g0	2.60	1.38
GO:00550	transmemb	Biological	0.00	0.01	Solyc10g0	Solyc10g0	2.56	1.36
GO:00550	transmemb	Biological	0.00	0.01	Solyc07g0	Solyc07g0	3.79	1.92
GO:00550	transmemb	Biological	0.00	0.01	Solyc05g0	Solyc05g0	0.28	-1.82
GO:00550	transmemb	Biological	0.00	0.01	Solyc11g0	Solyc11g0	0.24	-2.04
GO:00550	transmemb	Biological	0.00	0.01	Solyc01g0	Solyc01g0	0.49	-1.04
GO:00550	transmemb	Biological	0.00	0.01	Solyc01g0	Solyc01g0	0.42	-1.26
GO:00550	transmemb	Biological	0.00	0.01	Solyc05g0	Solyc05g0	3.13	1.65
GO:00550	transmemb	Biological	0.00	0.01	Solyc02g0	Solyc02g0	0.38	-1.39
GO:00550	transmemb	Biological	0.00	0.01	Solyc03g0	Solyc03g0	15.63	3.97
GO:00550	transmemb	Biological	0.00	0.01	Solyc10g0	Solyc10g0	40.50	5.34
GO:00550	transmemb	Biological	0.00	0.01	Solyc04g0	Solyc04g0	0.26	-1.94
GO:00550	transmemb	Biological	0.00	0.01	Solyc10g0	Solyc10g0	17.76	4.15
GO:00550	transmemb	Biological	0.00	0.01	Solyc10g0	Solyc10g0	2.02	1.01
GO:00550	transmemb	Biological	0.00	0.01	Solyc03g1	Solyc03g1	0.33	-1.59
GO:00550	transmemb	Biological	0.00	0.01	Solyc01g0	Solyc01g0	6.50	2.70
GO:00550	transmemb	Biological	0.00	0.01	Solyc03g0	Solyc03g0	2.05	1.04
GO:00550	transmemb	Biological	0.00	0.01	Solyc12g0	Solyc12g0	0.37	-1.44
GO:00550	transmemb	Biological	0.00	0.01	Solyc02g0	Solyc02g0	4.67	2.22
GO:00550	transmemb	Biological	0.00	0.01	Solyc03g0	Solyc03g0	2.16	1.11
GO:00550	transmemb	Biological	0.00	0.01	Solyc01g1	Solyc01g1	3.40	1.77
GO:00550	transmemb	Biological	0.00	0.01	Solyc04g0	Solyc04g0	0.41	-1.29
GO:00550	transmemb	Biological	0.00	0.01	Solyc10g0	Solyc10g0	0.38	-1.41
GO:00550	transmemb	Biological	0.00	0.01	Solyc06g0	Solyc06g0	2059.41	11.01
GO:00550	transmemb	Biological	0.00	0.01	Solyc02g0	Solyc02g0	2.28	1.19
GO:00550	transmemb	Biological	0.00	0.01	Solyc01g0	Solyc01g0	1991.27	10.96
GO:00550	transmemb	Biological	0.00	0.01	Solyc01g1	Solyc01g1	2829.11	11.47
GO:00550	transmemb	Biological	0.00	0.01	Solyc06g0	Solyc06g0	0.42	-1.25
GO:00550	transmemb	Biological	0.00	0.01	Solyc08g0	Solyc08g0	0.29	-1.80
GO:00550	transmemb	Biological	0.00	0.01	Solyc08g0	Solyc08g0	0.47	-1.08
GO:00550	transmemb	Biological	0.00	0.01	Solyc02g0	Solyc02g0	0.40	-1.31
GO:00550	transmemb	Biological	0.00	0.01	Solyc03g0	Solyc03g0	1336.08	10.38
GO:00550	transmemb	Biological	0.00	0.01	Solyc03g1	Solyc03g1	2773.71	11.44
GO:00550	transmemb	Biological	0.00	0.01	Solyc10g0	Solyc10g0	0.37	-1.43
GO:00550	transmemb	Biological	0.00	0.01	Solyc05g0	Solyc05g0	1020.85	10.00
GO:00550	transmemb	Biological	0.00	0.01	Solyc02g0	Solyc02g0	19.78	4.31
GO:00550	transmemb	Biological	0.00	0.01	Solyc12g0	Solyc12g0	0.45	-1.17
GO:00550	transmemb	Biological	0.00	0.01	Solyc01g0	Solyc01g0	894.88	9.81
GO:00550	transmemb	Biological	0.00	0.01	Solyc02g0	Solyc02g0	7.78	2.96
GO:00550	transmemb	Biological	0.00	0.01	Solyc09g0	Solyc09g0	429.36	8.75
GO:00550	transmemb	Biological	0.00	0.01	Solyc07g0	Solyc07g0	0.40	-1.33
GO:00550	transmemb	Biological	0.00	0.01	Solyc06g0	Solyc06g0	0.32	-1.62
GO:00550	transmemb	Biological	0.00	0.01	Solyc07g0	Solyc07g0	401.43	8.65
GO:00550	transmemb	Biological	0.00	0.01	Solyc05g0	Solyc05g0	9.14	3.19
GO:00550	transmemb	Biological	0.00	0.01	Solyc02g0	Solyc02g0	2.19	1.13
GO:00550	transmemb	Biological	0.00	0.01	Solyc06g0	Solyc06g0	3.28	1.71
GO:00550	transmemb	Biological	0.00	0.01	Solyc08g0	Solyc08g0	6.46	2.69

GO:00550	transmemb	Biological	0.00	0.01	Solyc04g0	Solyc04g0	7.72	2.95
GO:00550	transmemb	Biological	0.00	0.01	Solyc12g0	Solyc12g0	369.30	8.53
GO:00550	transmemb	Biological	0.00	0.01	Solyc09g0	Solyc09g0	0.49	-1.03
GO:00550	transmemb	Biological	0.00	0.01	Solyc02g0	Solyc02g0	412.01	8.69
GO:00550	transmemb	Biological	0.00	0.01	Solyc03g0	Solyc03g0	0.00	-9.99
GO:00550	transmemb	Biological	0.00	0.01	Solyc08g0	Solyc08g0	2.51	1.33
GO:00550	transmemb	Biological	0.00	0.01	Solyc06g0	Solyc06g0	0.44	-1.17
GO:00550	transmemb	Biological	0.00	0.01	Solyc09g0	Solyc09g0	0.38	-1.39
GO:00550	transmemb	Biological	0.00	0.01	Solyc03g1	Solyc03g1	0.00	-8.90
GO:00480	apoplast	Cellular C	0.00	0.01	Solyc10g0	PG2	9.48	3.25
GO:00480	apoplast	Cellular C	0.00	0.01	Solyc04g0	Solyc04g0	2.70	1.43
GO:00480	apoplast	Cellular C	0.00	0.01	Solyc12g0	Solyc12g0	24.67	4.62
GO:00480	apoplast	Cellular C	0.00	0.01	Solyc04g0	Solyc04g0	8.55	3.10
GO:00480	apoplast	Cellular C	0.00	0.01	Solyc04g0	Solyc04g0	3.38	1.76
GO:00480	apoplast	Cellular C	0.00	0.01	Solyc08g0	Solyc08g0	4.36	2.13
GO:00480	apoplast	Cellular C	0.00	0.01	Solyc01g0	XTH1	5.24	2.39
GO:00480	apoplast	Cellular C	0.00	0.01	Solyc09g0	Solyc09g0	0.36	-1.46
GO:00480	apoplast	Cellular C	0.00	0.01	Solyc05g0	Solyc05g0	0.35	-1.52
GO:00480	apoplast	Cellular C	0.00	0.01	Solyc03g0	Solyc03g0	3.84	1.94
GO:00480	apoplast	Cellular C	0.00	0.01	Solyc09g0	Solyc09g0	8.04	3.01
GO:00480	apoplast	Cellular C	0.00	0.01	Solyc05g0	Solyc05g0	0.18	-2.44
GO:00480	apoplast	Cellular C	0.00	0.01	Solyc09g0	Solyc09g0	25.41	4.67
GO:00480	apoplast	Cellular C	0.00	0.01	Solyc02g0	Solyc02g0	0.22	-2.21
GO:00480	apoplast	Cellular C	0.00	0.01	Solyc01g0	Solyc01g0	550.66	9.11
GO:00480	apoplast	Cellular C	0.00	0.01	Solyc07g0	Solyc07g0	0.46	-1.12
GO:00480	apoplast	Cellular C	0.00	0.01	Solyc02g0	Solyc02g0	2.27	1.18
GO:00480	apoplast	Cellular C	0.00	0.01	Solyc02g0	Solyc02g0	0.35	-1.51
GO:00480	apoplast	Cellular C	0.00	0.01	Solyc03g0	Solyc03g0	0.46	-1.12
GO:00199	lipid storag	Biological	0.00	0.01	Solyc06g0	Solyc06g0	12.59	3.65
GO:00199	lipid storag	Biological	0.00	0.01	Solyc04g0	Solyc04g0	4.53	2.18
GO:00199	lipid storag	Biological	0.00	0.01	Solyc03g1	Solyc03g1	10.69	3.42
GO:00199	lipid storag	Biological	0.00	0.01	Solyc06g0	Solyc06g0	18.67	4.22
GO:00199	lipid storag	Biological	0.00	0.01	Solyc12g0	Solyc12g0	14.44	3.85
GO:00199	lipid storag	Biological	0.00	0.01	Solyc08g0	Solyc08g0	11.44	3.52
GO:00167	2-oxogluta	Molecular	0.00	0.01	Solyc07g0	ACO1	2.82	1.49
GO:00167	2-oxogluta	Molecular	0.00	0.01	Solyc02g0	Solyc02g0	0.08	-3.73
GO:00167	2-oxogluta	Molecular	0.00	0.01	Solyc07g0	Solyc07g0	2.20	1.14
GO:00167	2-oxogluta	Molecular	0.00	0.01	Solyc06g0	Solyc06g0	0.15	-2.75
GO:00167	2-oxogluta	Molecular	0.00	0.01	Solyc02g0	Solyc02g0	2.56	1.36
GO:00167	2-oxogluta	Molecular	0.00	0.01	Solyc07g0	Solyc07g0	4.00	2.00
GO:00167	2-oxogluta	Molecular	0.00	0.01	Solyc07g0	Solyc07g0	29.42	4.88
GO:00167	2-oxogluta	Molecular	0.00	0.01	Solyc06g0	Solyc06g0	444.70	8.80
GO:00095	chromopla	Cellular C	0.00	0.01	Solyc04g0	Solyc04g0	0.00	-11.98
GO:00095	chromopla	Cellular C	0.00	0.01	Solyc04g0	Solyc04g0	0.00	-13.91
GO:00095	chromopla	Cellular C	0.00	0.01	Solyc10g0	Solyc10g0	0.04	-4.70
GO:00095	chromopla	Cellular C	0.00	0.01	Solyc10g0	Solyc10g0	0.00	-12.76
GO:00095	chromopla	Cellular C	0.00	0.01	Solyc04g0	Solyc04g0	0.06	-3.97
GO:00097	photosynth	Biological	0.00	0.01	Solyc09g0	Solyc09g0	5.85	2.55
GO:00097	photosynth	Biological	0.00	0.01	Solyc10g0	Solyc10g0	4.66	2.22
GO:00097	photosynth	Biological	0.00	0.01	Solyc10g0	Solyc10g0	2.07	1.05
GO:00097	photosynth	Biological	0.00	0.01	Solyc03g0	Solyc03g0	2.60	1.38

GO:00097	photosynth	Biological	0.00	0.01	Solyc03g0	Solyc03g0	3.69	1.88
GO:00097	photosynth	Biological	0.00	0.01	Solyc12g0	Solyc12g0	0.25	-2.01
GO:00515	iron-sulfur	Molecular	0.00	0.01	Solyc03g0	Solyc03g0	7.65	2.93
GO:00515	iron-sulfur	Molecular	0.00	0.01	Solyc11g0	Solyc11g0	2.92	1.55
GO:00515	iron-sulfur	Molecular	0.00	0.01	Solyc11g0	Solyc11g0	0.32	-1.67
GO:00515	iron-sulfur	Molecular	0.00	0.01	Solyc11g0	Solyc11g0	2.20	1.14
GO:00515	iron-sulfur	Molecular	0.00	0.01	Solyc12g0	Solyc12g0	0.00	-11.26
GO:00515	iron-sulfur	Molecular	0.00	0.01	Solyc05g0	Solyc05g0	2.96	1.57
GO:00515	iron-sulfur	Molecular	0.00	0.01	Solyc01g1	Solyc01g1	2993.63	11.55
GO:00515	iron-sulfur	Molecular	0.00	0.01	Solyc12g0	Solyc12g0	2.58	1.37
GO:00515	iron-sulfur	Molecular	0.00	0.01	Solyc03g0	Solyc03g0	0.11	-3.15
GO:00515	iron-sulfur	Molecular	0.00	0.01	Solyc06g0	Solyc06g0	0.05	-4.46
GO:00515	iron-sulfur	Molecular	0.00	0.01	Solyc04g0	Solyc04g0	39.10	5.29
GO:00515	iron-sulfur	Molecular	0.00	0.01	Solyc04g0	Solyc04g0	32.87	5.04
GO:00515	iron-sulfur	Molecular	0.00	0.01	Solyc04g0	Solyc04g0	883.93	9.79
GO:00515	iron-sulfur	Molecular	0.00	0.01	Solyc01g1	Solyc01g1	4.58	2.20
GO:00045	hydrolase a	Molecular	0.00	0.02	Solyc01g1	Solyc01g1	4.83	2.27
GO:00045	hydrolase a	Molecular	0.00	0.02	Solyc11g0	Solyc11g0	2.27	1.18
GO:00045	hydrolase a	Molecular	0.00	0.02	Solyc12g0	Solyc12g0	24.67	4.62
GO:00045	hydrolase a	Molecular	0.00	0.02	Solyc01g0	Solyc01g0	2.01	1.01
GO:00045	hydrolase a	Molecular	0.00	0.02	Solyc03g1	Solyc03g1	2.20	1.14
GO:00045	hydrolase a	Molecular	0.00	0.02	Solyc04g0	Solyc04g0	3.38	1.76
GO:00045	hydrolase a	Molecular	0.00	0.02	Solyc01g0	Solyc01g0	15.46	3.95
GO:00045	hydrolase a	Molecular	0.00	0.02	Solyc11g0	Solyc11g0	3.16	1.66
GO:00045	hydrolase a	Molecular	0.00	0.02	Solyc06g0	Solyc06g0	3.62	1.85
GO:00045	hydrolase a	Molecular	0.00	0.02	Solyc10g0	Solyc10g0	2.91	1.54
GO:00045	hydrolase a	Molecular	0.00	0.02	Solyc08g0	Solyc08g0	6.25	2.64
GO:00045	hydrolase a	Molecular	0.00	0.02	Solyc07g0	Solyc07g0	0.48	-1.05
GO:00045	hydrolase a	Molecular	0.00	0.02	Solyc02g0	Solyc02g0	0.15	-2.74
GO:00045	hydrolase a	Molecular	0.00	0.02	Solyc08g0	Solyc08g0	4.36	2.13
GO:00045	hydrolase a	Molecular	0.00	0.02	Solyc11g0	Solyc11g0	0.25	-1.98
GO:00045	hydrolase a	Molecular	0.00	0.02	Solyc01g0	XTH1	5.24	2.39
GO:00045	hydrolase a	Molecular	0.00	0.02	Solyc08g0	Solyc08g0	2.18	1.12
GO:00045	hydrolase a	Molecular	0.00	0.02	Solyc05g0	Solyc05g0	0.27	-1.87
GO:00045	hydrolase a	Molecular	0.00	0.02	Solyc05g0	Solyc05g0	2.18	1.12
GO:00045	hydrolase a	Molecular	0.00	0.02	Solyc09g0	Solyc09g0	0.36	-1.46
GO:00045	hydrolase a	Molecular	0.00	0.02	Solyc03g0	Solyc03g0	3.84	1.94
GO:00045	hydrolase a	Molecular	0.00	0.02	Solyc04g0	Solyc04g0	2.36	1.24
GO:00045	hydrolase a	Molecular	0.00	0.02	Solyc11g0	Solyc11g0	4049.02	11.98
GO:00045	hydrolase a	Molecular	0.00	0.02	Solyc11g0	Solyc11g0	4.22	2.08
GO:00045	hydrolase a	Molecular	0.00	0.02	Solyc07g0	Solyc07g0	8.50	3.09
GO:00045	hydrolase a	Molecular	0.00	0.02	Solyc06g0	Solyc06g0	2.25	1.17
GO:00045	hydrolase a	Molecular	0.00	0.02	Solyc07g0	Solyc07g0	14.86	3.89
GO:00045	hydrolase a	Molecular	0.00	0.02	Solyc07g0	Solyc07g0	401.43	8.65
GO:00045	hydrolase a	Molecular	0.00	0.02	Solyc06g0	Solyc06g0	32.34	5.02
GO:00045	hydrolase a	Molecular	0.00	0.02	Solyc02g0	Solyc02g0	0.35	-1.51
GO:00045	hydrolase a	Molecular	0.00	0.02	Solyc03g0	Solyc03g0	0.46	-1.12
GO:00045	hydrolase a	Molecular	0.00	0.02	Solyc11g0	Solyc11g0	2.74	1.46
GO:00098	coumarin b	Biological	0.00	0.02	Solyc07g0	ACO1	2.82	1.49
GO:00098	coumarin b	Biological	0.00	0.02	Solyc02g0	Solyc02g0	0.08	-3.73
GO:00098	coumarin b	Biological	0.00	0.02	Solyc07g0	Solyc07g0	2.20	1.14

GO:00098	coumarin b	Biological	0.00	0.02	Solyc06g0	Solyc06g0	0.15	-2.75
GO:00098	coumarin b	Biological	0.00	0.02	Solyc02g0	Solyc02g0	2.56	1.36
GO:00098	coumarin b	Biological	0.00	0.02	Solyc07g0	Solyc07g0	4.00	2.00
GO:00098	coumarin b	Biological	0.00	0.02	Solyc07g0	Solyc07g0	29.42	4.88
GO:00098	coumarin b	Biological	0.00	0.02	Solyc06g0	Solyc06g0	444.70	8.80
GO:00065	L-phenylal	Biological	0.00	0.02	Solyc10g0	Solyc10g0	4.26	2.09
GO:00065	L-phenylal	Biological	0.00	0.02	Solyc09g0	Solyc09g0	0.27	-1.91
GO:00065	L-phenylal	Biological	0.00	0.02	Solyc09g0	Solyc09g0	0.34	-1.54
GO:00065	L-phenylal	Biological	0.00	0.02	Solyc10g0	Solyc10g0	2.14	1.09
GO:00065	L-phenylal	Biological	0.00	0.02	Solyc03g0	Solyc03g0	794.30	9.63
GO:00059	carbohydra	Biological	0.00	0.02	Solyc10g0	PG2	9.48	3.25
GO:00059	carbohydra	Biological	0.00	0.02	Solyc01g1	Solyc01g1	4.83	2.27
GO:00059	carbohydra	Biological	0.00	0.02	Solyc11g0	Solyc11g0	2.27	1.18
GO:00059	carbohydra	Biological	0.00	0.02	Solyc09g0	Solyc09g0	0.34	-1.54
GO:00059	carbohydra	Biological	0.00	0.02	Solyc12g0	Solyc12g0	24.67	4.62
GO:00059	carbohydra	Biological	0.00	0.02	Solyc12g0	Solyc12g0	0.25	-1.99
GO:00059	carbohydra	Biological	0.00	0.02	Solyc01g0	Solyc01g0	2.01	1.01
GO:00059	carbohydra	Biological	0.00	0.02	Solyc11g0	Solyc11g0	4.12	2.04
GO:00059	carbohydra	Biological	0.00	0.02	Solyc07g0	Solyc07g0	7.24	2.86
GO:00059	carbohydra	Biological	0.00	0.02	Solyc03g1	Solyc03g1	2.20	1.14
GO:00059	carbohydra	Biological	0.00	0.02	Solyc10g0	CHI9	0.28	-1.84
GO:00059	carbohydra	Biological	0.00	0.02	Solyc04g0	Solyc04g0	3.38	1.76
GO:00059	carbohydra	Biological	0.00	0.02	Solyc07g0	Solyc07g0	3.93	1.98
GO:00059	carbohydra	Biological	0.00	0.02	Solyc11g0	Solyc11g0	3.16	1.66
GO:00059	carbohydra	Biological	0.00	0.02	Solyc06g0	Solyc06g0	3.62	1.85
GO:00059	carbohydra	Biological	0.00	0.02	Solyc08g0	Solyc08g0	6.25	2.64
GO:00059	carbohydra	Biological	0.00	0.02	Solyc07g0	Solyc07g0	0.48	-1.05
GO:00059	carbohydra	Biological	0.00	0.02	Solyc02g0	Solyc02g0	0.15	-2.74
GO:00059	carbohydra	Biological	0.00	0.02	Solyc08g0	Solyc08g0	4.36	2.13
GO:00059	carbohydra	Biological	0.00	0.02	Solyc11g0	Solyc11g0	0.25	-1.98
GO:00059	carbohydra	Biological	0.00	0.02	Solyc01g0	XTH1	5.24	2.39
GO:00059	carbohydra	Biological	0.00	0.02	Solyc08g0	Solyc08g0	2.18	1.12
GO:00059	carbohydra	Biological	0.00	0.02	Solyc11g0	Solyc11g0	2.94	1.56
GO:00059	carbohydra	Biological	0.00	0.02	Solyc05g0	Solyc05g0	0.27	-1.87
GO:00059	carbohydra	Biological	0.00	0.02	Solyc05g0	Solyc05g0	3.20	1.68
GO:00059	carbohydra	Biological	0.00	0.02	Solyc02g0	Solyc02g0	0.33	-1.59
GO:00059	carbohydra	Biological	0.00	0.02	Solyc10g0	Solyc10g0	2.23	1.16
GO:00059	carbohydra	Biological	0.00	0.02	Solyc09g0	Solyc09g0	0.36	-1.46
GO:00059	carbohydra	Biological	0.00	0.02	Solyc02g0	Solyc02g0	2.97	1.57
GO:00059	carbohydra	Biological	0.00	0.02	Solyc04g0	Solyc04g0	0.37	-1.44
GO:00059	carbohydra	Biological	0.00	0.02	Solyc05g0	Solyc05g0	2.50	1.32
GO:00059	carbohydra	Biological	0.00	0.02	Solyc03g0	Solyc03g0	3.84	1.94
GO:00059	carbohydra	Biological	0.00	0.02	Solyc04g0	Solyc04g0	2.36	1.24
GO:00059	carbohydra	Biological	0.00	0.02	Solyc11g0	Solyc11g0	4049.02	11.98
GO:00059	carbohydra	Biological	0.00	0.02	Solyc07g0	Solyc07g0	8.50	3.09
GO:00059	carbohydra	Biological	0.00	0.02	Solyc06g0	Solyc06g0	2.25	1.17
GO:00059	carbohydra	Biological	0.00	0.02	Solyc04g0	Solyc04g0	8.84	3.14
GO:00059	carbohydra	Biological	0.00	0.02	Solyc08g0	Solyc08g0	2.43	1.28
GO:00059	carbohydra	Biological	0.00	0.02	Solyc09g0	Solyc09g0	25.41	4.67
GO:00059	carbohydra	Biological	0.00	0.02	Solyc12g0	Solyc12g0	3.17	1.66
GO:00059	carbohydra	Biological	0.00	0.02	Solyc11g0	Solyc11g0	2.98	1.58

GO:00059	carbohydra	Biological	0.00	0.02	Solyc04g0	Solyc04g0	1564.01	10.61
GO:00059	carbohydra	Biological	0.00	0.02	Solyc07g0	Solyc07g0	14.86	3.89
GO:00059	carbohydra	Biological	0.00	0.02	Solyc07g0	Solyc07g0	401.43	8.65
GO:00059	carbohydra	Biological	0.00	0.02	Solyc03g0	Solyc03g0	11.56	3.53
GO:00059	carbohydra	Biological	0.00	0.02	Solyc02g0	Solyc02g0	0.35	-1.51
GO:00059	carbohydra	Biological	0.00	0.02	Solyc06g0	Solyc06g0	13.74	3.78
GO:00059	carbohydra	Biological	0.00	0.02	Solyc01g1	Solyc01g1	0.00	-8.99
GO:00059	carbohydra	Biological	0.00	0.02	Solyc10g0	Solyc10g0	0.00	-9.02
GO:00059	carbohydra	Biological	0.00	0.02	Solyc03g0	Solyc03g0	0.46	-1.12
GO:00059	carbohydra	Biological	0.00	0.02	Solyc11g0	Solyc11g0	2.74	1.46
GO:00167	oxidoreduc	Molecular	0.00	0.02	Solyc04g0	Solyc04g0	2.89	1.53
GO:00167	oxidoreduc	Molecular	0.00	0.02	Solyc08g0	Solyc08g0	4598.17	12.17
GO:00167	oxidoreduc	Molecular	0.00	0.02	Solyc01g0	Solyc01g0	3528.16	11.78
GO:00167	oxidoreduc	Molecular	0.00	0.02	Solyc09g0	Solyc09g0	51.95	5.70
GO:00167	oxidoreduc	Molecular	0.00	0.02	Solyc02g0	Solyc02g0	0.48	-1.05
GO:00167	oxidoreduc	Molecular	0.00	0.02	Solyc06g0	Solyc06g0	0.00	-10.22
GO:00167	oxidoreduc	Molecular	0.00	0.02	Solyc08g0	Solyc08g0	1235.43	10.27
GO:00167	oxidoreduc	Molecular	0.00	0.02	Solyc04g0	Solyc04g0	900.23	9.81
GO:00167	oxidoreduc	Molecular	0.00	0.02	Solyc06g0	Solyc06g0	17.36	4.12
GO:00167	oxidoreduc	Molecular	0.00	0.02	Solyc05g0	Solyc05g0	2.42	1.27
GO:00167	oxidoreduc	Molecular	0.00	0.02	Solyc02g0	Solyc02g0	3.18	1.67
GO:00167	oxidoreduc	Molecular	0.00	0.02	Solyc11g0	Solyc11g0	0.00	-9.24
GO:00167	oxidoreduc	Molecular	0.00	0.02	Solyc12g0	Solyc12g0	0.00	-9.92
GO:00167	oxidoreduc	Molecular	0.00	0.02	Solyc08g0	Solyc08g0	599.38	9.23
GO:00167	oxidoreduc	Molecular	0.00	0.02	Solyc10g0	Solyc10g0	0.00	-9.79
GO:00167	oxidoreduc	Molecular	0.00	0.03	Solyc01g0	Solyc01g0	0.09	-3.43
GO:00167	oxidoreduc	Molecular	0.00	0.03	Solyc08g0	Solyc08g0	4.51	2.17
GO:00167	oxidoreduc	Molecular	0.00	0.03	Solyc06g0	Solyc06g0	0.40	-1.31
GO:00167	oxidoreduc	Molecular	0.00	0.03	Solyc12g0	Solyc12g0	0.47	-1.10
GO:00167	oxidoreduc	Molecular	0.00	0.03	Solyc12g1	Solyc12g1	0.00	-9.86
GO:00095	plastid inn	Cellular C	0.00	0.03	Solyc11g0	AOS2	3.20	1.68
GO:00095	plastid inn	Cellular C	0.00	0.03	Solyc11g0	Solyc11g0	0.02	-5.38
GO:00095	plastid inn	Cellular C	0.00	0.03	Solyc11g0	Solyc11g0	0.09	-3.49
GO:00095	plastid inn	Cellular C	0.00	0.03	Solyc11g0	Solyc11g0	0.03	-5.20
GO:00095	plastid inn	Cellular C	0.00	0.03	Solyc10g0	Solyc10g0	0.06	-4.16
GO:00167	acyltransfe	Molecular	0.00	0.03	Solyc05g0	Solyc05g0	0.06	-4.07
GO:00167	acyltransfe	Molecular	0.00	0.03	Solyc09g0	Solyc09g0	0.06	-4.05
GO:00167	acyltransfe	Molecular	0.00	0.03	Solyc04g0	Solyc04g0	0.35	-1.50
GO:00167	acyltransfe	Molecular	0.00	0.03	Solyc05g0	Solyc05g0	3.02	1.60
GO:00167	acyltransfe	Molecular	0.00	0.03	Solyc03g1	Solyc03g1	2.44	1.28
GO:00167	acyltransfe	Molecular	0.00	0.03	Solyc07g0	Solyc07g0	0.49	-1.02
GO:00167	acyltransfe	Molecular	0.00	0.03	Solyc02g0	Solyc02g0	0.46	-1.11
GO:00167	acyltransfe	Molecular	0.00	0.03	Solyc05g0	Solyc05g0	3.86	1.95
GO:00167	acyltransfe	Molecular	0.00	0.03	Solyc12g0	Solyc12g0	2.35	1.23
GO:00167	acyltransfe	Molecular	0.00	0.03	Solyc09g0	Solyc09g0	0.00	-14.79
GO:00167	acyltransfe	Molecular	0.00	0.03	Solyc01g0	Solyc01g0	2.52	1.33
GO:00167	acyltransfe	Molecular	0.00	0.03	Solyc09g0	Solyc09g0	0.00	-11.98
GO:00167	acyltransfe	Molecular	0.00	0.03	Solyc08g0	Solyc08g0	2.54	1.34
GO:00167	acyltransfe	Molecular	0.00	0.03	Solyc07g0	Solyc07g0	1180.56	10.21
GO:00167	acyltransfe	Molecular	0.00	0.03	Solyc12g0	Solyc12g0	2.62	1.39
GO:00167	acyltransfe	Molecular	0.00	0.03	Solyc06g0	Solyc06g0	2.03	1.02

GO:00167	acyltransfe	Molecular	0.00	0.03	Solyc07g0	Solyc07g0	2.71	1.44
GO:00167	acyltransfe	Molecular	0.00	0.03	Solyc02g0	Solyc02g0	44.26	5.47
GO:00167	acyltransfe	Molecular	0.00	0.03	Solyc09g0	Solyc09g0	0.30	-1.74
GO:00167	acyltransfe	Molecular	0.00	0.03	Solyc03g0	Solyc03g0	2.51	1.33
GO:00167	acyltransfe	Molecular	0.00	0.03	Solyc04g0	Solyc04g0	1528.10	10.58
GO:00167	acyltransfe	Molecular	0.00	0.03	Solyc11g0	Solyc11g0	292.92	8.19
GO:00167	oxidoreduc	Molecular	0.00	0.03	Solyc11g0	AOS2	3.20	1.68
GO:00167	oxidoreduc	Molecular	0.00	0.03	Solyc04g0	Solyc04g0	2.89	1.53
GO:00167	oxidoreduc	Molecular	0.00	0.03	Solyc07g0	Solyc07g0	0.32	-1.63
GO:00167	oxidoreduc	Molecular	0.00	0.03	Solyc10g0	Solyc10g0	0.40	-1.34
GO:00167	oxidoreduc	Molecular	0.00	0.03	Solyc03g1	Solyc03g1	0.30	-1.75
GO:00167	oxidoreduc	Molecular	0.00	0.03	Solyc06g0	Solyc06g0	7.48	2.90
GO:00167	oxidoreduc	Molecular	0.00	0.03	Solyc03g1	Solyc03g1	4.61	2.21
GO:00167	oxidoreduc	Molecular	0.00	0.03	Solyc04g0	Solyc04g0	6.45	2.69
GO:00167	oxidoreduc	Molecular	0.00	0.03	Solyc01g1	Solyc01g1	0.03	-5.02
GO:00167	oxidoreduc	Molecular	0.00	0.03	Solyc05g0	Solyc05g0	2.81	1.49
GO:00167	oxidoreduc	Molecular	0.00	0.03	Solyc08g0	Solyc08g0	2.39	1.25
GO:00167	oxidoreduc	Molecular	0.00	0.03	Solyc01g0	Solyc01g0	5.13	2.36
GO:00167	oxidoreduc	Molecular	0.00	0.03	Solyc08g0	Solyc08g0	4598.17	12.17
GO:00167	oxidoreduc	Molecular	0.00	0.03	Solyc01g0	Solyc01g0	3528.16	11.78
GO:00167	oxidoreduc	Molecular	0.00	0.03	Solyc09g0	Solyc09g0	51.95	5.70
GO:00167	oxidoreduc	Molecular	0.00	0.03	Solyc08g0	Solyc08g0	4039.84	11.98
GO:00167	oxidoreduc	Molecular	0.00	0.03	Solyc01g0	Solyc01g0	3.21	1.68
GO:00167	oxidoreduc	Molecular	0.00	0.03	Solyc02g0	Solyc02g0	0.48	-1.05
GO:00167	oxidoreduc	Molecular	0.00	0.03	Solyc04g0	Solyc04g0	0.45	-1.16
GO:00167	oxidoreduc	Molecular	0.00	0.03	Solyc06g0	Solyc06g0	0.00	-10.22
GO:00167	oxidoreduc	Molecular	0.00	0.03	Solyc08g0	Solyc08g0	1235.43	10.27
GO:00167	oxidoreduc	Molecular	0.00	0.03	Solyc09g0	Solyc09g0	54.33	5.76
GO:00167	oxidoreduc	Molecular	0.00	0.03	Solyc04g0	Solyc04g0	900.23	9.81
GO:00167	oxidoreduc	Molecular	0.00	0.03	Solyc06g0	Solyc06g0	17.36	4.12
GO:00167	oxidoreduc	Molecular	0.00	0.03	Solyc02g0	Solyc02g0	0.28	-1.83
GO:00167	oxidoreduc	Molecular	0.00	0.03	Solyc05g0	Solyc05g0	2.42	1.27
GO:00167	oxidoreduc	Molecular	0.00	0.03	Solyc12g0	Solyc12g0	1446.11	10.50
GO:00167	oxidoreduc	Molecular	0.00	0.03	Solyc02g0	Solyc02g0	3.18	1.67
GO:00167	oxidoreduc	Molecular	0.00	0.03	Solyc01g1	Solyc01g1	0.47	-1.09
GO:00167	oxidoreduc	Molecular	0.00	0.03	Solyc07g0	Solyc07g0	9.07	3.18
GO:00167	oxidoreduc	Molecular	0.00	0.03	Solyc11g0	Solyc11g0	0.00	-9.24
GO:00167	oxidoreduc	Molecular	0.00	0.03	Solyc12g0	Solyc12g0	0.00	-9.92
GO:00167	oxidoreduc	Molecular	0.00	0.03	Solyc08g0	Solyc08g0	0.34	-1.54
GO:00167	oxidoreduc	Molecular	0.00	0.03	Solyc01g0	Solyc01g0	13.05	3.71
GO:00167	oxidoreduc	Molecular	0.00	0.03	Solyc07g0	Solyc07g0	0.50	-1.01
GO:00167	oxidoreduc	Molecular	0.00	0.03	Solyc04g0	Solyc04g0	9.75	3.29
GO:00167	oxidoreduc	Molecular	0.00	0.03	Solyc07g0	Solyc07g0	159.10	7.31
GO:00167	oxidoreduc	Molecular	0.00	0.03	Solyc08g0	Solyc08g0	599.38	9.23
GO:00167	oxidoreduc	Molecular	0.00	0.03	Solyc10g0	Solyc10g0	0.00	-9.79
GO:00167	oxidoreduc	Molecular	0.00	0.03	Solyc04g0	Solyc04g0	26.73	4.74
GO:00094	response to	Biological	0.00	0.04	Solyc11g0	Solyc11g0	0.28	-1.84
GO:00094	response to	Biological	0.00	0.04	Solyc09g0	Solyc09g0	5.85	2.55
GO:00094	response to	Biological	0.00	0.04	Solyc07g0	Solyc07g0	2.20	1.14
GO:00094	response to	Biological	0.00	0.04	Solyc03g1	Solyc03g1	4.55	2.18
GO:00094	response to	Biological	0.00	0.04	Solyc10g0	Solyc10g0	4.66	2.22

GO:00094	response to	Biological	0.00	0.04	Solyc10g0	Solyc10g0	2.07	1.05
GO:00094	response to	Biological	0.00	0.04	Solyc07g0	Solyc07g0	29.42	4.88
GO:00094	response to	Biological	0.00	0.04	Solyc03g0	Solyc03g0	2.60	1.38
GO:00094	response to	Biological	0.00	0.04	Solyc03g0	Solyc03g0	3.69	1.88
GO:00094	response to	Biological	0.00	0.04	Solyc12g0	Solyc12g0	0.25	-2.01
GO:00166	oxidoreduc	Molecular	0.00	0.04	Solyc03g0	Solyc03g0	7.65	2.93
GO:00166	oxidoreduc	Molecular	0.00	0.04	Solyc03g1	Solyc03g1	2.22	1.15
GO:00166	oxidoreduc	Molecular	0.00	0.04	Solyc08g0	Solyc08g0	14.34	3.84
GO:00451	electron tra	Molecular	0.00	0.04	Solyc12g0	Solyc12g0	2.10	1.07
GO:00451	electron tra	Molecular	0.00	0.04	Solyc01g0	Solyc01g0	0.02	-5.72
GO:00451	electron tra	Molecular	0.00	0.04	Solyc03g1	Solyc03g1	0.10	-3.25
GO:00080	chitin bind	Molecular	0.00	0.04	Solyc01g1	Solyc01g1	14.06	3.81
GO:00080	chitin bind	Molecular	0.00	0.04	Solyc10g0	CHI9	0.28	-1.84
GO:00080	chitin bind	Molecular	0.00	0.04	Solyc07g0	Solyc07g0	0.02	-5.77
GO:00080	chitin bind	Molecular	0.00	0.04	Solyc03g1	Solyc03g1	12.24	3.61
GO:00080	chitin bind	Molecular	0.00	0.04	Solyc03g1	Solyc03g1	49.39	5.63
GO:00080	chitin bind	Molecular	0.00	0.04	Solyc06g0	Solyc06g0	13.74	3.78
GO:00080	chitin bind	Molecular	0.00	0.04	Solyc10g0	Solyc10g0	0.00	-9.02
GO:00095	plasmodes	Cellular Co	0.00	0.04	Solyc04g0	Solyc04g0	2.70	1.43
GO:00095	plasmodes	Cellular Co	0.00	0.04	Solyc01g1	Solyc01g1	14.06	3.81
GO:00095	plasmodes	Cellular Co	0.00	0.04	Solyc12g0	Solyc12g0	9.07	3.18
GO:00095	plasmodes	Cellular Co	0.00	0.04	Solyc01g1	Solyc01g1	72.82	6.19
GO:00095	plasmodes	Cellular Co	0.00	0.04	Solyc01g1	Solyc01g1	2.90	1.54
GO:00095	plasmodes	Cellular Co	0.00	0.04	Solyc04g0	Solyc04g0	2.29	1.19
GO:00095	plasmodes	Cellular Co	0.00	0.04	Solyc03g0	Solyc03g0	7.56	2.92
GO:00095	plasmodes	Cellular Co	0.00	0.04	Solyc02g0	Solyc02g0	0.44	-1.19
GO:00095	plasmodes	Cellular Co	0.00	0.04	Solyc10g0	Solyc10g0	3.83	1.94
GO:00095	plasmodes	Cellular Co	0.00	0.04	Solyc01g1	Solyc01g1	2.32	1.22
GO:00095	plasmodes	Cellular Co	0.00	0.04	Solyc04g0	Solyc04g0	0.41	-1.29
GO:00095	plasmodes	Cellular Co	0.00	0.04	Solyc06g0	Solyc06g0	2.02	1.02
GO:00095	plasmodes	Cellular Co	0.00	0.04	Solyc02g0	Solyc02g0	14.41	3.85
GO:00095	plasmodes	Cellular Co	0.00	0.04	Solyc11g0	Solyc11g0	3.29	1.72
GO:00095	plasmodes	Cellular Co	0.00	0.04	Solyc01g0	Solyc01g0	0.06	-3.97
GO:00095	plasmodes	Cellular Co	0.00	0.04	Solyc01g1	Solyc01g1	37.32	5.22
GO:00095	plasmodes	Cellular Co	0.00	0.04	Solyc09g0	Solyc09g0	0.49	-1.04
GO:00095	plasmodes	Cellular Co	0.00	0.04	Solyc01g1	Solyc01g1	4.37	2.13
GO:00095	plasmodes	Cellular Co	0.00	0.04	Solyc03g0	RBCS-2A	2.71	1.44
GO:00095	plasmodes	Cellular Co	0.00	0.04	Solyc04g0	Solyc04g0	0.46	-1.11
GO:00095	plasmodes	Cellular Co	0.00	0.04	Solyc08g0	Solyc08g0	0.50	-1.00
GO:00095	plasmodes	Cellular Co	0.00	0.04	Solyc04g0	Solyc04g0	0.39	-1.37
GO:00095	plasmodes	Cellular Co	0.00	0.04	Solyc03g0	Solyc03g0	0.34	-1.57
GO:00095	plasmodes	Cellular Co	0.00	0.04	Solyc11g0	Solyc11g0	0.23	-2.09
GO:00095	plasmodes	Cellular Co	0.00	0.04	Solyc08g0	Solyc08g0	4.19	2.07
GO:00095	plasmodes	Cellular Co	0.00	0.04	Solyc03g0	Solyc03g0	0.36	-1.46
GO:00095	plasmodes	Cellular Co	0.00	0.04	Solyc05g0	Solyc05g0	0.31	-1.68
GO:00095	plasmodes	Cellular Co	0.00	0.04	Solyc07g0	Solyc07g0	0.39	-1.35
GO:00095	plasmodes	Cellular Co	0.00	0.04	Solyc03g0	Solyc03g0	0.39	-1.37
GO:00095	plasmodes	Cellular Co	0.00	0.04	Solyc11g0	Solyc11g0	0.43	-1.23
GO:00052	ion channe	Molecular	0.00	0.04	Solyc01g1	Solyc01g1	0.29	-1.80
GO:00052	ion channe	Molecular	0.00	0.04	Solyc03g0	Solyc03g0	0.31	-1.67
GO:00052	ion channe	Molecular	0.00	0.04	Solyc11g0	Solyc11g0	0.24	-2.04

GO:00052	ion channe	Molecular	0.00	0.04	Solyc10g0	Solyc10g0	40.50	5.34
GO:00052	ion channe	Molecular	0.00	0.04	Solyc02g0	Solyc02g0	4.67	2.22
GO:00052	ion channe	Molecular	0.00	0.04	Solyc01g0	Solyc01g0	894.88	9.81
GO:00052	ion channe	Molecular	0.00	0.04	Solyc12g0	Solyc12g0	369.30	8.53
GO:00052	ion channe	Molecular	0.00	0.04	Solyc08g0	Solyc08g0	2.51	1.33
GO:00480	quinone bi	Molecular	0.00	0.05	Solyc09g0	Solyc09g0	0.18	-2.50
GO:00480	quinone bi	Molecular	0.00	0.05	Solyc03g0	Solyc03g0	0.04	-4.63
GO:00480	quinone bi	Molecular	0.00	0.05	Solyc03g0	Solyc03g0	0.11	-3.15
GO:00480	quinone bi	Molecular	0.00	0.05	Solyc10g0	Solyc10g0	0.00	-13.73
GO:00480	quinone bi	Molecular	0.00	0.05	Solyc10g0	Solyc10g0	0.00	-12.09
GO:00480	quinone bi	Molecular	0.00	0.05	Solyc06g0	Solyc06g0	0.05	-4.46
GO:00480	quinone bi	Molecular	0.00	0.05	Solyc01g0	Solyc01g0	0.12	-3.04
GO:00480	quinone bi	Molecular	0.00	0.05	Solyc02g0	Solyc02g0	0.19	-2.37
GO:00480	quinone bi	Molecular	0.00	0.05	Solyc01g0	Solyc01g0	0.08	-3.58
GO:00480	quinone bi	Molecular	0.00	0.05	Solyc01g0	Solyc01g0	0.00	-11.62
GO:00314	nucleosom	Molecular	0.00	0.05	Solyc01g0	Solyc01g0	2.68	1.42
GO:00314	nucleosom	Molecular	0.00	0.05	Solyc02g0	Solyc02g0	2.01	1.01
GO:00314	nucleosom	Molecular	0.00	0.05	Solyc01g0	Solyc01g0	3.17	1.66
GO:00314	nucleosom	Molecular	0.00	0.05	Solyc01g0	Solyc01g0	2.30	1.20
GO:00314	nucleosom	Molecular	0.00	0.05	Solyc12g0	Solyc12g0	2.41	1.27
GO:00314	nucleosom	Molecular	0.00	0.05	Solyc01g0	Solyc01g0	2.57	1.36
GO:00088	cellulase a	Molecular	0.00	0.05	Solyc08g0	Solyc08g0	6.25	2.64
GO:00088	cellulase a	Molecular	0.00	0.05	Solyc08g0	Solyc08g0	2.18	1.12
GO:00088	cellulase a	Molecular	0.00	0.05	Solyc05g0	Solyc05g0	0.27	-1.87
GO:00088	cellulase a	Molecular	0.00	0.05	Solyc04g0	Solyc04g0	2.36	1.24
GO:00088	cellulase a	Molecular	0.00	0.05	Solyc07g0	Solyc07g0	14.86	3.89
GO:00302	cellulose c	Biological	0.00	0.05	Solyc08g0	Solyc08g0	6.25	2.64
GO:00302	cellulose c	Biological	0.00	0.05	Solyc08g0	Solyc08g0	2.18	1.12
GO:00302	cellulose c	Biological	0.00	0.05	Solyc05g0	Solyc05g0	0.27	-1.87
GO:00302	cellulose c	Biological	0.00	0.05	Solyc04g0	Solyc04g0	2.36	1.24
GO:00302	cellulose c	Biological	0.00	0.05	Solyc07g0	Solyc07g0	14.86	3.89
GO:00095	chromopla	Cellular C	0.00	0.05	Solyc04g0	Solyc04g0	0.00	-11.98
GO:00095	chromopla	Cellular C	0.00	0.05	Solyc04g0	Solyc04g0	0.00	-13.91
GO:00095	chromopla	Cellular C	0.00	0.05	Solyc10g0	Solyc10g0	0.04	-4.70
GO:00095	chromopla	Cellular C	0.00	0.05	Solyc10g0	Solyc10g0	0.00	-12.76
GO:00095	chromopla	Cellular C	0.00	0.05	Solyc04g0	Solyc04g0	0.06	-3.97
GO:00095	plant-type	Cellular C	0.00	0.05	Solyc01g1	Solyc01g1	4.83	2.27
GO:00095	plant-type	Cellular C	0.00	0.05	Solyc01g1	Solyc01g1	3.25	1.70
GO:00095	plant-type	Cellular C	0.00	0.05	Solyc01g1	Solyc01g1	2.90	1.54
GO:00095	plant-type	Cellular C	0.00	0.05	Solyc04g0	Solyc04g0	2.29	1.19
GO:00095	plant-type	Cellular C	0.00	0.05	Solyc06g0	Solyc06g0	2.47	1.31
GO:00095	plant-type	Cellular C	0.00	0.05	Solyc06g0	Solyc06g0	3.62	1.85
GO:00095	plant-type	Cellular C	0.00	0.05	Solyc07g0	PDF1A	4.20	2.07
GO:00095	plant-type	Cellular C	0.00	0.05	Solyc02g0	Solyc02g0	14.41	3.85
GO:00095	plant-type	Cellular C	0.00	0.05	Solyc01g0	Solyc01g0	0.06	-3.97
GO:00095	plant-type	Cellular C	0.00	0.05	Solyc03g0	Solyc03g0	3.84	1.94
GO:00095	plant-type	Cellular C	0.00	0.05	Solyc01g1	Solyc01g1	37.32	5.22
GO:00095	plant-type	Cellular C	0.00	0.05	Solyc01g1	Solyc01g1	4.37	2.13
GO:00095	plant-type	Cellular C	0.00	0.05	Solyc11g0	Solyc11g0	0.43	-1.23
GO:00102	plastoglob	Cellular C	0.00	0.06	Solyc12g0	Solyc12g0	2.97	1.57
GO:00102	plastoglob	Cellular C	0.00	0.06	Solyc09g0	Solyc09g0	5.85	2.55

GO:00102	plastoglob	Cellular C	0.00	0.06	Solyc03g1	Solyc03g1	2.16	1.11
GO:00102	plastoglob	Cellular C	0.00	0.06	Solyc10g0	Solyc10g0	2.07	1.05
GO:00102	plastoglob	Cellular C	0.00	0.06	Solyc03g0	Solyc03g0	2.60	1.38
GO:00102	plastoglob	Cellular C	0.00	0.06	Solyc12g0	Solyc12g0	0.25	-2.01
GO:00055	copper ion	Molecular	0.00	0.06	Solyc04g0	Solyc04g0	2.70	1.43
GO:00055	copper ion	Molecular	0.00	0.06	Solyc04g0	Solyc04g0	3.53	1.82
GO:00055	copper ion	Molecular	0.00	0.06	Solyc08g0	Solyc08g0	2.85	1.51
GO:00055	copper ion	Molecular	0.00	0.06	Solyc04g0	PETE	2.69	1.43
GO:00055	copper ion	Molecular	0.00	0.06	Solyc04g0	Solyc04g0	2.29	1.19
GO:00055	copper ion	Molecular	0.00	0.06	Solyc02g0	Solyc02g0	14.41	3.85
GO:00055	copper ion	Molecular	0.00	0.06	Solyc02g0	Solyc02g0	2.56	1.36
GO:00055	copper ion	Molecular	0.00	0.06	Solyc01g0	Solyc01g0	0.06	-3.97
GO:00055	copper ion	Molecular	0.00	0.06	Solyc01g1	Solyc01g1	37.32	5.22
GO:00055	copper ion	Molecular	0.00	0.06	Solyc05g0	Solyc05g0	0.18	-2.44
GO:00055	copper ion	Molecular	0.00	0.06	Solyc04g0	Solyc04g0	1191.32	10.22
GO:00055	copper ion	Molecular	0.00	0.06	Solyc02g0	Solyc02g0	0.22	-2.21
GO:00055	copper ion	Molecular	0.00	0.06	Solyc11g0	Solyc11g0	0.00	-10.82
GO:00055	copper ion	Molecular	0.00	0.06	Solyc11g0	Solyc11g0	0.43	-1.23
GO:00082	secondary	Molecular	0.00	0.06	Solyc04g0	Solyc04g0	0.18	-2.50
GO:00082	secondary	Molecular	0.00	0.06	Solyc12g0	Solyc12g0	0.37	-1.44
GO:00082	secondary	Molecular	0.00	0.06	Solyc04g0	Solyc04g0	0.41	-1.29
GO:00082	secondary	Molecular	0.00	0.06	Solyc05g0	Solyc05g0	9.14	3.19
GO:00082	secondary	Molecular	0.00	0.06	Solyc09g0	Solyc09g0	0.49	-1.03
GO:00325	ribonucleo	Molecular	0.00	0.06	Solyc08g0	Solyc08g0	4.60	2.20
GO:00325	ribonucleo	Molecular	0.00	0.06	Solyc10g0	Solyc10g0	0.00	-13.39
GO:00325	ribonucleo	Molecular	0.00	0.06	Solyc12g0	Solyc12g0	0.00	-12.05
GO:00325	ribonucleo	Molecular	0.00	0.06	Solyc12g0	Solyc12g0	12.70	3.67
GO:00325	ribonucleo	Molecular	0.00	0.06	Solyc02g0	Solyc02g0	0.00	-12.40
GO:00063	transcripti	Biological	0.00	0.08	Solyc04g0	Solyc04g0	2.73	1.45
GO:00063	transcripti	Biological	0.00	0.08	Solyc08g0	Solyc08g0	4.60	2.20
GO:00063	transcripti	Biological	0.00	0.08	Solyc09g0	Solyc09g0	0.34	-1.58
GO:00063	transcripti	Biological	0.00	0.08	Solyc11g0	Solyc11g0	2.29	1.19
GO:00063	transcripti	Biological	0.00	0.08	Solyc08g0	Solyc08g0	0.20	-2.35
GO:00063	transcripti	Biological	0.00	0.08	Solyc08g0	Solyc08g0	3.77	1.91
GO:00063	transcripti	Biological	0.00	0.08	Solyc04g0	Solyc04g0	0.21	-2.27
GO:00063	transcripti	Biological	0.00	0.08	Solyc10g0	Solyc10g0	0.03	-5.19
GO:00063	transcripti	Biological	0.00	0.08	Solyc08g0	Solyc08g0	0.00	-12.86
GO:00063	transcripti	Biological	0.00	0.08	Solyc10g0	Solyc10g0	0.00	-13.39
GO:00063	transcripti	Biological	0.00	0.08	Solyc10g0	Solyc10g0	0.00	-9.03
GO:00063	transcripti	Biological	0.00	0.08	Solyc09g0	Solyc09g0	0.25	-1.98
GO:00063	transcripti	Biological	0.00	0.08	Solyc10g0	Solyc10g0	1081.58	10.08
GO:00063	transcripti	Biological	0.00	0.08	Solyc06g0	Solyc06g0	0.39	-1.37
GO:00063	transcripti	Biological	0.00	0.08	Solyc03g0	Solyc03g0	2300.48	11.17
GO:00063	transcripti	Biological	0.00	0.08	Solyc08g0	Solyc08g0	0.03	-5.23
GO:00063	transcripti	Biological	0.00	0.08	Solyc12g0	Solyc12g0	2529.18	11.30
GO:00063	transcripti	Biological	0.00	0.08	Solyc01g0	Solyc01g0	1296.75	10.34
GO:00063	transcripti	Biological	0.00	0.08	Solyc12g0	Solyc12g0	0.09	-3.53
GO:00063	transcripti	Biological	0.00	0.08	Solyc09g0	Solyc09g0	2135.62	11.06
GO:00063	transcripti	Biological	0.00	0.08	Solyc03g1	Solyc03g1	2361.02	11.21
GO:00063	transcripti	Biological	0.00	0.08	Solyc12g0	Solyc12g0	0.00	-12.05
GO:00063	transcripti	Biological	0.00	0.08	Solyc07g0	Solyc07g0	42.77	5.42

GO:00063	transcriptio	Biological	0.00	0.08	Solyc10g0	Solyc10g0	2.60	1.38
GO:00063	transcriptio	Biological	0.00	0.08	Solyc12g0	Solyc12g0	12.70	3.67
GO:00063	transcriptio	Biological	0.00	0.08	Solyc06g0	Solyc06g0	15.65	3.97
GO:00063	transcriptio	Biological	0.00	0.08	Solyc06g0	Solyc06g0	8.85	3.15
GO:00063	transcriptio	Biological	0.00	0.08	Solyc02g0	Solyc02g0	11.00	3.46
GO:00063	transcriptio	Biological	0.00	0.08	Solyc06g0	Solyc06g0	0.45	-1.16
GO:00063	transcriptio	Biological	0.00	0.08	Solyc02g0	Solyc02g0	15.79	3.98
GO:00063	transcriptio	Biological	0.00	0.08	Solyc02g0	Solyc02g0	1506.28	10.56
GO:00063	transcriptio	Biological	0.00	0.08	Solyc08g0	Solyc08g0	2.07	1.05
GO:00063	transcriptio	Biological	0.00	0.08	Solyc02g0	Solyc02g0	0.00	-12.40
GO:00063	transcriptio	Biological	0.00	0.08	Solyc11g0	Solyc11g0	0.47	-1.09
GO:00063	transcriptio	Biological	0.00	0.08	Solyc08g0	Solyc08g0	3.29	1.72
GO:00063	transcriptio	Biological	0.00	0.08	Solyc01g0	Solyc01g0	0.24	-2.09
GO:00063	transcriptio	Biological	0.00	0.08	Solyc06g0	Solyc06g0	0.45	-1.16
GO:00063	transcriptio	Biological	0.00	0.08	Solyc02g0	Solyc02g0	0.06	-4.03
GO:00063	transcriptio	Biological	0.00	0.08	Solyc01g0	Solyc01g0	0.00	-11.62
GO:00063	transcriptio	Biological	0.00	0.08	Solyc10g0	Solyc10g0	2.42	1.27
GO:00063	transcriptio	Biological	0.00	0.08	Solyc05g0	Solyc05g0	0.00	-10.39
GO:00063	transcriptio	Biological	0.00	0.08	Solyc09g0	Solyc09g0	0.22	-2.22
GO:00063	transcriptio	Biological	0.00	0.08	Solyc11g0	Solyc11g0	0.00	-9.22
GO:00063	transcriptio	Biological	0.00	0.08	Solyc02g0	Solyc02g0	7.52	2.91
GO:00715	cell wall or	Biological	0.00	0.08	Solyc10g0	PG2	9.48	3.25
GO:00715	cell wall or	Biological	0.00	0.08	Solyc12g0	Solyc12g0	24.67	4.62
GO:00715	cell wall or	Biological	0.00	0.08	Solyc01g1	Solyc01g1	3.25	1.70
GO:00715	cell wall or	Biological	0.00	0.08	Solyc04g0	Solyc04g0	3.38	1.76
GO:00715	cell wall or	Biological	0.00	0.08	Solyc08g0	Solyc08g0	4.36	2.13
GO:00715	cell wall or	Biological	0.00	0.08	Solyc01g0	XTH1	5.24	2.39
GO:00715	cell wall or	Biological	0.00	0.08	Solyc09g0	Solyc09g0	0.36	-1.46
GO:00715	cell wall or	Biological	0.00	0.08	Solyc05g0	Solyc05g0	0.35	-1.52
GO:00715	cell wall or	Biological	0.00	0.08	Solyc03g1	Solyc03g1	0.44	-1.20
GO:00715	cell wall or	Biological	0.00	0.08	Solyc06g0	Solyc06g0	0.11	-3.25
GO:00715	cell wall or	Biological	0.00	0.08	Solyc12g0	Solyc12g0	3.03	1.60
GO:00715	cell wall or	Biological	0.00	0.08	Solyc01g0	Solyc01g0	0.19	-2.40
GO:00715	cell wall or	Biological	0.00	0.08	Solyc03g1	Solyc03g1	0.15	-2.75
GO:00715	cell wall or	Biological	0.00	0.08	Solyc02g0	Solyc02g0	0.35	-1.51
GO:00715	cell wall or	Biological	0.00	0.08	Solyc03g0	Solyc03g0	0.46	-1.12
GO:00082	sulfate trar	Biological	0.00	0.08	Solyc04g0	Solyc04g0	0.18	-2.50
GO:00082	sulfate trar	Biological	0.00	0.08	Solyc12g0	Solyc12g0	0.37	-1.44
GO:00082	sulfate trar	Biological	0.00	0.08	Solyc04g0	Solyc04g0	0.41	-1.29
GO:00082	sulfate trar	Biological	0.00	0.08	Solyc05g0	Solyc05g0	9.14	3.19
GO:00082	sulfate trar	Biological	0.00	0.08	Solyc09g0	Solyc09g0	0.49	-1.03
GO:00151	sulfate trar	Molecular	0.00	0.08	Solyc04g0	Solyc04g0	0.18	-2.50
GO:00151	sulfate trar	Molecular	0.00	0.08	Solyc12g0	Solyc12g0	0.37	-1.44
GO:00151	sulfate trar	Molecular	0.00	0.08	Solyc04g0	Solyc04g0	0.41	-1.29
GO:00151	sulfate trar	Molecular	0.00	0.08	Solyc05g0	Solyc05g0	9.14	3.19
GO:00151	sulfate trar	Molecular	0.00	0.08	Solyc09g0	Solyc09g0	0.49	-1.03
GO:00038	catalytic ac	Molecular	0.00	0.08	Solyc03g0	Solyc03g0	7.65	2.93
GO:00038	catalytic ac	Molecular	0.00	0.08	Solyc08g0	Solyc08g0	2.65	1.41
GO:00038	catalytic ac	Molecular	0.00	0.08	Solyc03g0	Solyc03g0	4.05	2.02
GO:00038	catalytic ac	Molecular	0.00	0.08	Solyc10g0	Solyc10g0	4.26	2.09
GO:00038	catalytic ac	Molecular	0.00	0.08	Solyc07g0	Solyc07g0	0.35	-1.51

GO:00038	catalytic ac	Molecular	0.00	0.08	Solyc09g0	Solyc09g0	0.27	-1.91
GO:00038	catalytic ac	Molecular	0.00	0.08	Solyc08g0	Solyc08g0	2.53	1.34
GO:00038	catalytic ac	Molecular	0.00	0.08	Solyc04g0	Solyc04g0	4.06	2.02
GO:00038	catalytic ac	Molecular	0.00	0.08	Solyc06g0	Solyc06g0	2.22	1.15
GO:00038	catalytic ac	Molecular	0.00	0.08	Solyc04g0	Solyc04g0	3.78	1.92
GO:00038	catalytic ac	Molecular	0.00	0.08	Solyc09g0	Solyc09g0	2.88	1.53
GO:00038	catalytic ac	Molecular	0.00	0.08	Solyc11g0	Solyc11g0	4.12	2.04
GO:00038	catalytic ac	Molecular	0.00	0.08	Solyc07g0	Solyc07g0	7.24	2.86
GO:00038	catalytic ac	Molecular	0.00	0.08	Solyc12g0	Solyc12g0	2.01	1.00
GO:00038	catalytic ac	Molecular	0.00	0.08	Solyc09g0	Solyc09g0	0.37	-1.45
GO:00038	catalytic ac	Molecular	0.00	0.08	Solyc10g0	Solyc10g0	3.13	1.64
GO:00038	catalytic ac	Molecular	0.00	0.08	Solyc04g0	Solyc04g0	0.40	-1.31
GO:00038	catalytic ac	Molecular	0.00	0.08	Solyc11g0	Solyc11g0	20.23	4.34
GO:00038	catalytic ac	Molecular	0.00	0.08	Solyc09g0	Solyc09g0	0.34	-1.54
GO:00038	catalytic ac	Molecular	0.00	0.08	Solyc09g0	Solyc09g0	0.35	-1.50
GO:00038	catalytic ac	Molecular	0.00	0.08	Solyc06g0	Solyc06g0	3.62	1.85
GO:00038	catalytic ac	Molecular	0.00	0.08	Solyc04g0	Solyc04g0	2.89	1.53
GO:00038	catalytic ac	Molecular	0.00	0.08	Solyc01g1	Solyc01g1	0.24	-2.06
GO:00038	catalytic ac	Molecular	0.00	0.08	Solyc11g0	Solyc11g0	0.50	-1.01
GO:00038	catalytic ac	Molecular	0.00	0.08	Solyc01g0	Solyc01g0	0.32	-1.65
GO:00038	catalytic ac	Molecular	0.00	0.08	Solyc12g0	Solyc12g0	7.81	2.96
GO:00038	catalytic ac	Molecular	0.00	0.08	Solyc07g0	Solyc07g0	0.48	-1.05
GO:00038	catalytic ac	Molecular	0.00	0.08	Solyc03g0	Solyc03g0	4.53	2.18
GO:00038	catalytic ac	Molecular	0.00	0.08	Solyc09g0	Solyc09g0	0.47	-1.07
GO:00038	catalytic ac	Molecular	0.00	0.08	Solyc07g0	Solyc07g0	6.21	2.63
GO:00038	catalytic ac	Molecular	0.00	0.08	Solyc09g0	Solyc09g0	0.36	-1.46
GO:00038	catalytic ac	Molecular	0.00	0.08	Solyc01g0	Solyc01g0	3.54	1.82
GO:00038	catalytic ac	Molecular	0.00	0.08	Solyc03g0	Solyc03g0	2.79	1.48
GO:00038	catalytic ac	Molecular	0.00	0.08	Solyc03g1	Solyc03g1	0.38	-1.38
GO:00038	catalytic ac	Molecular	0.00	0.08	Solyc02g0	Solyc02g0	2.97	1.57
GO:00038	catalytic ac	Molecular	0.00	0.08	Solyc04g0	Solyc04g0	0.37	-1.44
GO:00038	catalytic ac	Molecular	0.00	0.08	Solyc09g0	Solyc09g0	3.37	1.75
GO:00038	catalytic ac	Molecular	0.00	0.08	Solyc12g0	Solyc12g0	13681.82	13.74
GO:00038	catalytic ac	Molecular	0.00	0.08	Solyc02g0	Solyc02g0	3.02	1.60
GO:00038	catalytic ac	Molecular	0.00	0.08	Solyc03g0	Solyc03g0	3.84	1.94
GO:00038	catalytic ac	Molecular	0.00	0.08	Solyc01g0	Solyc01g0	3.26	1.71
GO:00038	catalytic ac	Molecular	0.00	0.08	Solyc08g0	Solyc08g0	14.34	3.84
GO:00038	catalytic ac	Molecular	0.00	0.08	Solyc08g0	Solyc08g0	3785.25	11.89
GO:00038	catalytic ac	Molecular	0.00	0.08	Solyc09g0	Solyc09g0	2.14	1.10
GO:00038	catalytic ac	Molecular	0.00	0.08	Solyc02g0	Solyc02g0	2381.72	11.22
GO:00038	catalytic ac	Molecular	0.00	0.08	Solyc10g0	Solyc10g0	0.35	-1.51
GO:00038	catalytic ac	Molecular	0.00	0.08	Solyc01g0	Solyc01g0	0.41	-1.29
GO:00038	catalytic ac	Molecular	0.00	0.08	Solyc05g0	Solyc05g0	4.12	2.04
GO:00038	catalytic ac	Molecular	0.00	0.08	Solyc12g0	Solyc12g0	3.03	1.60
GO:00038	catalytic ac	Molecular	0.00	0.08	Solyc04g0	Solyc04g0	1711.14	10.74
GO:00038	catalytic ac	Molecular	0.00	0.08	Solyc07g0	Solyc07g0	8.04	3.01
GO:00038	catalytic ac	Molecular	0.00	0.08	Solyc09g0	Solyc09g0	8.04	3.01
GO:00038	catalytic ac	Molecular	0.00	0.08	Solyc06g0	Solyc06g0	0.11	-3.23
GO:00038	catalytic ac	Molecular	0.00	0.08	Solyc02g0	Solyc02g0	3056.62	11.58
GO:00038	catalytic ac	Molecular	0.00	0.08	Solyc10g0	Solyc10g0	2.14	1.09
GO:00038	catalytic ac	Molecular	0.00	0.08	Solyc05g0	Solyc05g0	2.69	1.43

GO:00038	catalytic ac	Molecular	0.00	0.08	Solyc03g0	Solyc03g0	794.30	9.63
GO:00038	catalytic ac	Molecular	0.00	0.08	Solyc04g0	Solyc04g0	1564.01	10.61
GO:00038	catalytic ac	Molecular	0.00	0.08	Solyc03g1	Solyc03g1	6.29	2.65
GO:00038	catalytic ac	Molecular	0.00	0.08	Solyc09g0	Solyc09g0	2.09	1.07
GO:00038	catalytic ac	Molecular	0.00	0.08	Solyc11g0	Solyc11g0	3.46	1.79
GO:00038	catalytic ac	Molecular	0.00	0.08	Solyc04g0	Solyc04g0	1185.03	10.21
GO:00038	catalytic ac	Molecular	0.00	0.08	Solyc03g1	Solyc03g1	6.23	2.64
GO:00038	catalytic ac	Molecular	0.00	0.08	Solyc09g0	Solyc09g0	2.29	1.20
GO:00038	catalytic ac	Molecular	0.00	0.08	Solyc08g0	Solyc08g0	0.13	-2.97
GO:00038	catalytic ac	Molecular	0.00	0.08	Solyc07g0	Solyc07g0	5.84	2.55
GO:00038	catalytic ac	Molecular	0.00	0.08	Solyc01g0	Solyc01g0	550.66	9.11
GO:00038	catalytic ac	Molecular	0.00	0.08	Solyc07g0	Solyc07g0	9.83	3.30
GO:00038	catalytic ac	Molecular	0.00	0.08	Solyc06g0	Solyc06g0	0.00	-9.45
GO:00038	catalytic ac	Molecular	0.00	0.08	Solyc03g0	Solyc03g0	604.27	9.24
GO:00038	catalytic ac	Molecular	0.00	0.08	Solyc02g0	Solyc02g0	3.52	1.82
GO:00038	catalytic ac	Molecular	0.00	0.08	Solyc08g0	Solyc08g0	0.00	-9.72
GO:00063	nucleosom	Biological	0.00	0.08	Solyc01g0	Solyc01g0	2.68	1.42
GO:00063	nucleosom	Biological	0.00	0.08	Solyc02g0	Solyc02g0	2.01	1.01
GO:00063	nucleosom	Biological	0.00	0.08	Solyc04g0	Solyc04g0	2.47	1.30
GO:00063	nucleosom	Biological	0.00	0.08	Solyc11g0	Solyc11g0	2.31	1.21
GO:00063	nucleosom	Biological	0.00	0.08	Solyc06g0	Solyc06g0	2.48	1.31
GO:00063	nucleosom	Biological	0.00	0.08	Solyc01g0	Solyc01g0	3.17	1.66
GO:00063	nucleosom	Biological	0.00	0.08	Solyc01g0	Solyc01g0	2.30	1.20
GO:00063	nucleosom	Biological	0.00	0.08	Solyc12g0	Solyc12g0	2.41	1.27
GO:00063	nucleosom	Biological	0.00	0.08	Solyc05g0	Solyc05g0	2.76	1.47
GO:00063	nucleosom	Biological	0.00	0.08	Solyc01g0	Solyc01g0	2.57	1.36
GO:00097	photosynth	Biological	0.00	0.08	Solyc09g0	Solyc09g0	5.85	2.55
GO:00097	photosynth	Biological	0.00	0.08	Solyc10g0	Solyc10g0	4.66	2.22
GO:00097	photosynth	Biological	0.00	0.08	Solyc10g0	Solyc10g0	2.07	1.05
GO:00097	photosynth	Biological	0.00	0.08	Solyc03g0	Solyc03g0	2.60	1.38
GO:00097	photosynth	Biological	0.00	0.08	Solyc03g0	Solyc03g0	3.69	1.88
GO:00097	photosynth	Biological	0.00	0.08	Solyc12g0	Solyc12g0	0.25	-2.01
GO:00162	naringenin	Molecular	0.01	0.08	Solyc05g0	Solyc05g0	0.06	-4.07
GO:00162	naringenin	Molecular	0.01	0.08	Solyc09g0	Solyc09g0	0.06	-4.05
GO:01021	chalcone s	Molecular	0.01	0.08	Solyc05g0	Solyc05g0	0.06	-4.07
GO:01021	chalcone s	Molecular	0.01	0.08	Solyc09g0	Solyc09g0	0.06	-4.05
GO:01200	jasmonic a	Molecular	0.01	0.08	Solyc10g0	Solyc10g0	65.27	6.03
GO:01200	jasmonic a	Molecular	0.01	0.08	Solyc03g0	Solyc03g0	3.90	1.96
GO:00099	positive re	Biological	0.01	0.08	Solyc03g1	Solyc03g1	2.44	1.28
GO:00099	positive re	Biological	0.01	0.08	Solyc08g0	Solyc08g0	0.34	-1.54
GO:00472	quinate O-	Molecular	0.01	0.08	Solyc03g1	Solyc03g1	2.44	1.28
GO:00472	quinate O-	Molecular	0.01	0.08	Solyc07g0	Solyc07g0	0.49	-1.02
GO:00465	malate deh	Molecular	0.01	0.08	Solyc11g0	Solyc11g0	4.12	2.04
GO:00465	malate deh	Molecular	0.01	0.08	Solyc03g0	Solyc03g0	2.79	1.48
GO:00313	Ctf18 RFC	Cellular Co	0.01	0.08	Solyc01g0	Solyc01g0	2.56	1.36
GO:00313	Ctf18 RFC	Cellular Co	0.01	0.08	Solyc06g0	Solyc06g0	0.31	-1.70
GO:00329	circadian r	Biological	0.01	0.08	Solyc09g0	Solyc09g0	0.40	-1.34
GO:00329	circadian r	Biological	0.01	0.08	Solyc12g0	Solyc12g0	2.29	1.20
GO:00431	entrainmer	Biological	0.01	0.08	Solyc09g0	Solyc09g0	0.40	-1.34
GO:00431	entrainmer	Biological	0.01	0.08	Solyc12g0	Solyc12g0	2.29	1.20
GO:00615	glucose 6-p	Molecular	0.01	0.08	Solyc06g0	Solyc06g0	0.33	-1.58

GO:00615	glucose 6-p	Molecular	0.01	0.08	Solyc06g0	Solyc06g0	2.35	1.23
GO:00100	phytochror	Biological	0.01	0.08	Solyc01g0	Solyc01g0	2.26	1.18
GO:00100	phytochror	Biological	0.01	0.08	Solyc01g0	Solyc01g0	2.27	1.18
GO:00166	oxidoreduc	Molecular	0.01	0.08	Solyc01g0	Solyc01g0	2.26	1.18
GO:00166	oxidoreduc	Molecular	0.01	0.08	Solyc01g0	Solyc01g0	2.27	1.18
GO:00506	phytochror	Molecular	0.01	0.08	Solyc01g0	Solyc01g0	2.26	1.18
GO:00506	phytochror	Molecular	0.01	0.08	Solyc01g0	Solyc01g0	2.27	1.18
GO:00081	NADH deh	Molecular	0.01	0.09	Solyc09g0	Solyc09g0	0.18	-2.50
GO:00081	NADH deh	Molecular	0.01	0.09	Solyc03g0	Solyc03g0	0.04	-4.63
GO:00081	NADH deh	Molecular	0.01	0.09	Solyc03g0	Solyc03g0	0.11	-3.15
GO:00081	NADH deh	Molecular	0.01	0.09	Solyc10g0	Solyc10g0	0.00	-13.73
GO:00081	NADH deh	Molecular	0.01	0.09	Solyc06g0	Solyc06g0	0.05	-4.46
GO:00081	NADH deh	Molecular	0.01	0.09	Solyc07g0	Solyc07g0	0.04	-4.69
GO:00081	NADH deh	Molecular	0.01	0.09	Solyc11g0	Solyc11g0	0.00	-11.68
GO:00081	NADH deh	Molecular	0.01	0.09	Solyc02g0	Solyc02g0	0.19	-2.37
GO:00081	NADH deh	Molecular	0.01	0.09	Solyc01g0	Solyc01g0	0.08	-3.58
GO:00081	NADH deh	Molecular	0.01	0.09	Solyc03g0	Solyc03g0	0.13	-3.00
GO:00081	NADH deh	Molecular	0.01	0.09	Solyc11g0	Solyc11g0	0.00	-10.02
GO:00081	NADH deh	Molecular	0.01	0.09	Solyc01g0	Solyc01g0	0.00	-11.62
GO:00314	L-ascorbic	Molecular	0.01	0.09	Solyc07g0	ACO1	2.82	1.49
GO:00314	L-ascorbic	Molecular	0.01	0.09	Solyc02g0	Solyc02g0	0.08	-3.73
GO:00314	L-ascorbic	Molecular	0.01	0.09	Solyc07g0	Solyc07g0	2.20	1.14
GO:00314	L-ascorbic	Molecular	0.01	0.09	Solyc06g0	Solyc06g0	0.15	-2.75
GO:00314	L-ascorbic	Molecular	0.01	0.09	Solyc02g0	Solyc02g0	2.56	1.36
GO:00314	L-ascorbic	Molecular	0.01	0.09	Solyc07g0	Solyc07g0	4.00	2.00
GO:00314	L-ascorbic	Molecular	0.01	0.09	Solyc07g0	Solyc07g0	29.42	4.88
GO:00314	L-ascorbic	Molecular	0.01	0.09	Solyc06g0	Solyc06g0	444.70	8.80
GO:00069	response to	Biological	0.01	0.09	Solyc04g0	Solyc04g0	2.70	1.43
GO:00069	response to	Biological	0.01	0.09	Solyc04g0	Solyc04g0	3.53	1.82
GO:00069	response to	Biological	0.01	0.09	Solyc09g0	Solyc09g0	0.40	-1.34
GO:00069	response to	Biological	0.01	0.09	Solyc12g0	Solyc12g0	2.29	1.20
GO:00102	photosyste	Biological	0.01	0.09	Solyc01g1	Solyc01g1	6.83	2.77
GO:00102	photosyste	Biological	0.01	0.09	Solyc07g0	Solyc07g0	3.28	1.71
GO:00102	photosyste	Biological	0.01	0.09	Solyc02g0	PSBO	5.02	2.33
GO:00102	photosyste	Biological	0.01	0.09	Solyc09g0	Solyc09g0	4.23	2.08
GO:00043	glutamate-	Molecular	0.01	0.09	Solyc04g0	Solyc04g0	0.40	-1.31
GO:00043	glutamate-	Molecular	0.01	0.09	Solyc12g0	Solyc12g0	13681.82	13.74
GO:00043	glutamate-	Molecular	0.01	0.09	Solyc12g0	Solyc12g0	12341.84	13.59
GO:00043	glutamate-	Molecular	0.01	0.09	Solyc01g0	Solyc01g0	550.66	9.11
GO:00038	CTP synth	Molecular	0.01	0.09	Solyc02g0	Solyc02g0	3.08	1.62
GO:00038	CTP synth	Molecular	0.01	0.09	Solyc02g0	Solyc02g0	0.42	-1.25
GO:00038	CTP synth	Molecular	0.01	0.09	Solyc02g0	Solyc02g0	0.36	-1.49
GO:00060	cellular gl	Biological	0.01	0.10	Solyc12g0	Solyc12g0	24.67	4.62
GO:00060	cellular gl	Biological	0.01	0.10	Solyc04g0	Solyc04g0	3.38	1.76
GO:00060	cellular gl	Biological	0.01	0.10	Solyc08g0	Solyc08g0	4.36	2.13
GO:00060	cellular gl	Biological	0.01	0.10	Solyc01g0	XTH1	5.24	2.39
GO:00060	cellular gl	Biological	0.01	0.10	Solyc09g0	Solyc09g0	0.36	-1.46
GO:00060	cellular gl	Biological	0.01	0.10	Solyc02g0	Solyc02g0	0.35	-1.51
GO:00060	cellular gl	Biological	0.01	0.10	Solyc03g0	Solyc03g0	0.46	-1.12
GO:00167	xyloglucan	Molecular	0.01	0.10	Solyc12g0	Solyc12g0	24.67	4.62
GO:00167	xyloglucan	Molecular	0.01	0.10	Solyc04g0	Solyc04g0	3.38	1.76

GO:00167	xyloglucan	Molecular	0.01	0.10	Solyc08g0	Solyc08g0	4.36	2.13
GO:00167	xyloglucan	Molecular	0.01	0.10	Solyc01g0	XTH1	5.24	2.39
GO:00167	xyloglucan	Molecular	0.01	0.10	Solyc09g0	Solyc09g0	0.36	-1.46
GO:00167	xyloglucan	Molecular	0.01	0.10	Solyc02g0	Solyc02g0	0.35	-1.51
GO:00167	xyloglucan	Molecular	0.01	0.10	Solyc03g0	Solyc03g0	0.46	-1.12
GO:00097	photosynth	Biological	0.01	0.11	Solyc02g0	Solyc02g0	3.41	1.77
GO:00097	photosynth	Biological	0.01	0.11	Solyc12g0	Solyc12g0	0.05	-4.20
GO:00097	photosynth	Biological	0.01	0.11	Solyc10g0	Solyc10g0	0.00	-12.09
GO:00097	photosynth	Biological	0.01	0.11	Solyc11g0	Solyc11g0	0.00	-13.08
GO:00097	photosynth	Biological	0.01	0.11	Solyc03g1	Solyc03g1	0.10	-3.25
GO:00097	photosynth	Biological	0.01	0.11	Solyc02g0	Solyc02g0	0.43	-1.21
GO:00097	photosynth	Biological	0.01	0.12	Solyc12g0	Solyc12g0	2.97	1.57
GO:00097	photosynth	Biological	0.01	0.12	Solyc08g0	Solyc08g0	2.79	1.48
GO:00097	photosynth	Biological	0.01	0.12	Solyc09g0	Solyc09g0	25.41	4.67
GO:00097	photosynth	Biological	0.01	0.12	Solyc07g0	Solyc07g0	2.47	1.30
GO:00167	oxidoreduc	Molecular	0.01	0.12	Solyc01g0	Solyc01g0	2.18	1.13
GO:00167	oxidoreduc	Molecular	0.01	0.12	Solyc01g0	Solyc01g0	0.31	-1.67
GO:00167	oxidoreduc	Molecular	0.01	0.12	Solyc08g0	LOX1.1	2.60	1.38
GO:00167	oxidoreduc	Molecular	0.01	0.12	Solyc02g0	Solyc02g0	2.27	1.18
GO:00167	oxidoreduc	Molecular	0.01	0.12	Solyc03g1	Solyc03g1	3.89	1.96
GO:00167	oxidoreduc	Molecular	0.01	0.12	Solyc09g0	Solyc09g0	0.47	-1.10
GO:00167	oxidoreduc	Molecular	0.01	0.12	Solyc12g0	Solyc12g0	2.73	1.45
GO:00167	oxidoreduc	Molecular	0.01	0.12	Solyc05g0	Solyc05g0	39.90	5.32
GO:00167	oxidoreduc	Molecular	0.01	0.12	Solyc09g0	Solyc09g0	0.50	-1.01
GO:00007	nucleosom	Cellular Co	0.01	0.12	Solyc01g0	Solyc01g0	2.68	1.42
GO:00007	nucleosom	Cellular Co	0.01	0.12	Solyc01g0	Solyc01g0	3.72	1.90
GO:00007	nucleosom	Cellular Co	0.01	0.12	Solyc02g0	Solyc02g0	2.01	1.01
GO:00007	nucleosom	Cellular Co	0.01	0.12	Solyc06g0	Solyc06g0	2.67	1.41
GO:00007	nucleosom	Cellular Co	0.01	0.12	Solyc04g0	Solyc04g0	2.47	1.30
GO:00007	nucleosom	Cellular Co	0.01	0.12	Solyc11g0	Solyc11g0	2.31	1.21
GO:00007	nucleosom	Cellular Co	0.01	0.12	Solyc01g0	Solyc01g0	2.37	1.24
GO:00007	nucleosom	Cellular Co	0.01	0.12	Solyc06g0	Solyc06g0	2.48	1.31
GO:00007	nucleosom	Cellular Co	0.01	0.12	Solyc01g0	Solyc01g0	3.17	1.66
GO:00007	nucleosom	Cellular Co	0.01	0.12	Solyc01g0	Solyc01g0	2.30	1.20
GO:00007	nucleosom	Cellular Co	0.01	0.12	Solyc12g0	Solyc12g0	2.41	1.27
GO:00007	nucleosom	Cellular Co	0.01	0.12	Solyc05g0	Solyc05g0	2.76	1.47
GO:00007	nucleosom	Cellular Co	0.01	0.12	Solyc01g0	Solyc01g0	2.57	1.36
GO:00469	proton-tran	Molecular	0.01	0.12	Solyc12g0	Solyc12g0	2.97	1.57
GO:00469	proton-tran	Molecular	0.01	0.12	Solyc06g0	Solyc06g0	2.29	1.20
GO:00469	proton-tran	Molecular	0.01	0.12	Solyc04g0	Solyc04g0	0.16	-2.66
GO:00469	proton-tran	Molecular	0.01	0.12	Solyc10g0	Solyc10g0	0.03	-5.05
GO:00469	proton-tran	Molecular	0.01	0.12	Solyc06g0	Solyc06g0	15.51	3.95
GO:00469	proton-tran	Molecular	0.01	0.12	Solyc11g0	Solyc11g0	0.22	-2.19
GO:00167	oxidoreduc	Molecular	0.01	0.12	Solyc04g0	Solyc04g0	2.29	1.19
GO:00167	oxidoreduc	Molecular	0.01	0.12	Solyc02g0	Solyc02g0	14.41	3.85
GO:00167	oxidoreduc	Molecular	0.01	0.12	Solyc01g0	Solyc01g0	0.06	-3.97
GO:00167	oxidoreduc	Molecular	0.01	0.12	Solyc01g1	Solyc01g1	37.32	5.22
GO:00167	oxidoreduc	Molecular	0.01	0.12	Solyc04g0	Solyc04g0	1191.32	10.22
GO:00167	oxidoreduc	Molecular	0.01	0.12	Solyc11g0	Solyc11g0	0.43	-1.23
GO:00168	intramolec	Molecular	0.01	0.14	Solyc05g0	Solyc05g0	0.12	-3.00
GO:00168	intramolec	Molecular	0.01	0.14	Solyc06g0	Solyc06g0	2.19	1.13

GO:00168	intramolec	Molecular	0.01	0.14	Solyc05g0	Solyc05g0	3.96	1.99
GO:00506	oxidoreduc	Molecular	0.01	0.14	Solyc05g0	Solyc05g0	4.33	2.11
GO:00506	oxidoreduc	Molecular	0.01	0.14	Solyc03g1	Solyc03g1	0.48	-1.07
GO:00506	oxidoreduc	Molecular	0.01	0.14	Solyc01g0	Solyc01g0	3.53	1.82
GO:00430	negative re	Biological	0.01	0.15	Solyc12g0	Solyc12g0	2.75	1.46
GO:00430	negative re	Biological	0.01	0.15	Solyc12g0	Solyc12g0	2.22	1.15
GO:00430	negative re	Biological	0.01	0.15	Solyc12g0	Solyc12g0	2.15	1.10
GO:00430	negative re	Biological	0.01	0.15	Solyc03g1	Solyc03g1	5.44	2.44
GO:00430	negative re	Biological	0.01	0.15	Solyc03g1	Solyc03g1	0.44	-1.20
GO:00430	negative re	Biological	0.01	0.15	Solyc09g0	Solyc09g0	8.11	3.02
GO:00430	negative re	Biological	0.01	0.15	Solyc07g0	Solyc07g0	3.98	1.99
GO:00041	aspartic-ty	Molecular	0.01	0.16	Solyc08g0	Solyc08g0	19.15	4.26
GO:00041	aspartic-ty	Molecular	0.01	0.16	Solyc05g0	Solyc05g0	0.36	-1.47
GO:00041	aspartic-ty	Molecular	0.01	0.16	Solyc01g1	Solyc01g1	0.44	-1.20
GO:00041	aspartic-ty	Molecular	0.01	0.16	Solyc11g0	Solyc11g0	0.29	-1.80
GO:00041	aspartic-ty	Molecular	0.01	0.16	Solyc10g0	Solyc10g0	2.35	1.23
GO:00041	aspartic-ty	Molecular	0.01	0.16	Solyc09g0	Solyc09g0	0.41	-1.27
GO:00041	aspartic-ty	Molecular	0.01	0.16	Solyc05g0	Solyc05g0	4.45	2.15
GO:00041	aspartic-ty	Molecular	0.01	0.16	Solyc01g0	Solyc01g0	0.46	-1.12
GO:00041	aspartic-ty	Molecular	0.01	0.16	Solyc03g1	Solyc03g1	4459.91	12.12
GO:00041	aspartic-ty	Molecular	0.01	0.16	Solyc03g0	Solyc03g0	3.13	1.65
GO:00041	aspartic-ty	Molecular	0.01	0.16	Solyc01g0	Solyc01g0	0.46	-1.12
GO:00041	aspartic-ty	Molecular	0.01	0.16	Solyc03g0	Solyc03g0	627.68	9.29
GO:00041	aspartic-ty	Molecular	0.01	0.16	Solyc01g0	Solyc01g0	19.06	4.25
GO:00041	aspartic-ty	Molecular	0.01	0.16	Solyc06g0	Solyc06g0	0.33	-1.59
GO:00041	aspartic-ty	Molecular	0.01	0.16	Solyc09g0	Solyc09g0	5.06	2.34
GO:00052	voltage-ga	Molecular	0.01	0.16	Solyc01g1	Solyc01g1	0.29	-1.80
GO:00052	voltage-ga	Molecular	0.01	0.16	Solyc03g0	Solyc03g0	0.31	-1.67
GO:00052	voltage-ga	Molecular	0.01	0.16	Solyc05g0	Solyc05g0	8.29	3.05
GO:00052	voltage-ga	Molecular	0.01	0.16	Solyc01g0	Solyc01g0	894.88	9.81
GO:00052	voltage-ga	Molecular	0.01	0.16	Solyc12g0	Solyc12g0	369.30	8.53
GO:00036	DNA bindi	Molecular	0.01	0.16	Solyc04g0	Solyc04g0	0.09	-3.54
GO:00036	DNA bindi	Molecular	0.01	0.16	Solyc06g0	Solyc06g0	7.71	2.95
GO:00036	DNA bindi	Molecular	0.01	0.16	Solyc06g0	Solyc06g0	3.14	1.65
GO:00036	DNA bindi	Molecular	0.01	0.16	Solyc11g0	Solyc11g0	0.36	-1.47
GO:00036	DNA bindi	Molecular	0.01	0.16	Solyc08g0	Solyc08g0	2.82	1.49
GO:00036	DNA bindi	Molecular	0.01	0.16	Solyc01g0	Solyc01g0	2.68	1.42
GO:00036	DNA bindi	Molecular	0.01	0.16	Solyc04g0	Solyc04g0	2.73	1.45
GO:00036	DNA bindi	Molecular	0.01	0.16	Solyc05g0	Solyc05g0	0.30	-1.72
GO:00036	DNA bindi	Molecular	0.01	0.16	Solyc01g0	Solyc01g0	3.72	1.90
GO:00036	DNA bindi	Molecular	0.01	0.16	Solyc02g0	Solyc02g0	2.17	1.12
GO:00036	DNA bindi	Molecular	0.01	0.16	Solyc09g0	Solyc09g0	0.18	-2.45
GO:00036	DNA bindi	Molecular	0.01	0.16	Solyc05g0	Solyc05g0	0.46	-1.12
GO:00036	DNA bindi	Molecular	0.01	0.16	Solyc09g0	Solyc09g0	0.40	-1.34
GO:00036	DNA bindi	Molecular	0.01	0.16	Solyc09g0	Solyc09g0	0.32	-1.63
GO:00036	DNA bindi	Molecular	0.01	0.16	Solyc06g0	Solyc06g0	0.48	-1.05
GO:00036	DNA bindi	Molecular	0.01	0.16	Solyc08g0	Solyc08g0	4.60	2.20
GO:00036	DNA bindi	Molecular	0.01	0.16	Solyc05g0	Solyc05g0	2.40	1.27
GO:00036	DNA bindi	Molecular	0.01	0.16	Solyc04g0	Solyc04g0	0.38	-1.40
GO:00036	DNA bindi	Molecular	0.01	0.16	Solyc11g0	Solyc11g0	2.29	1.19
GO:00036	DNA bindi	Molecular	0.01	0.16	Solyc06g0	Solyc06g0	0.46	-1.11

GO:00036	DNA bind	Molecular	0.01	0.16	Solyc01g1	Solyc01g1	2.32	1.22
GO:00036	DNA bind	Molecular	0.01	0.16	Solyc02g0	Solyc02g0	2.01	1.01
GO:00036	DNA bind	Molecular	0.01	0.16	Solyc10g0	Solyc10g0	0.07	-3.89
GO:00036	DNA bind	Molecular	0.01	0.16	Solyc01g0	Solyc01g0	0.44	-1.18
GO:00036	DNA bind	Molecular	0.01	0.16	Solyc06g0	Solyc06g0	0.49	-1.03
GO:00036	DNA bind	Molecular	0.01	0.16	Solyc06g0	Solyc06g0	2.67	1.41
GO:00036	DNA bind	Molecular	0.01	0.16	Solyc03g1	Solyc03g1	0.48	-1.06
GO:00036	DNA bind	Molecular	0.01	0.16	Solyc04g0	Solyc04g0	2.47	1.30
GO:00036	DNA bind	Molecular	0.01	0.16	Solyc09g0	Solyc09g0	0.35	-1.51
GO:00036	DNA bind	Molecular	0.01	0.16	Solyc02g0	Solyc02g0	0.49	-1.02
GO:00036	DNA bind	Molecular	0.01	0.16	Solyc11g0	Solyc11g0	2.31	1.21
GO:00036	DNA bind	Molecular	0.01	0.16	Solyc11g0	Solyc11g0	17863.20	14.12
GO:00036	DNA bind	Molecular	0.01	0.16	Solyc01g0	Solyc01g0	2.37	1.24
GO:00036	DNA bind	Molecular	0.01	0.16	Solyc09g0	Solyc09g0	0.44	-1.19
GO:00036	DNA bind	Molecular	0.01	0.16	Solyc07g0	Solyc07g0	4759.57	12.22
GO:00036	DNA bind	Molecular	0.01	0.16	Solyc06g0	Solyc06g0	2.48	1.31
GO:00036	DNA bind	Molecular	0.01	0.16	Solyc05g0	Solyc05g0	6.89	2.79
GO:00036	DNA bind	Molecular	0.01	0.16	Solyc11g0	Solyc11g0	18.46	4.21
GO:00036	DNA bind	Molecular	0.01	0.16	Solyc04g0	Solyc04g0	0.21	-2.27
GO:00036	DNA bind	Molecular	0.01	0.16	Solyc02g0	Solyc02g0	0.32	-1.64
GO:00036	DNA bind	Molecular	0.01	0.16	Solyc01g0	Solyc01g0	3.17	1.66
GO:00036	DNA bind	Molecular	0.01	0.16	Solyc01g0	Solyc01g0	2.30	1.20
GO:00036	DNA bind	Molecular	0.01	0.16	Solyc06g0	Solyc06g0	3.13	1.64
GO:00036	DNA bind	Molecular	0.01	0.16	Solyc01g0	Solyc01g0	0.39	-1.35
GO:00036	DNA bind	Molecular	0.01	0.16	Solyc10g0	Solyc10g0	0.03	-5.19
GO:00036	DNA bind	Molecular	0.01	0.16	Solyc06g0	Solyc06g0	0.35	-1.53
GO:00036	DNA bind	Molecular	0.01	0.16	Solyc08g0	Solyc08g0	0.00	-12.86
GO:00036	DNA bind	Molecular	0.01	0.16	Solyc10g0	Solyc10g0	5392.75	12.40
GO:00036	DNA bind	Molecular	0.01	0.16	Solyc05g0	Solyc05g0	2.54	1.35
GO:00036	DNA bind	Molecular	0.01	0.16	Solyc10g0	Solyc10g0	0.00	-13.39
GO:00036	DNA bind	Molecular	0.01	0.16	Solyc08g0	Solyc08g0	0.42	-1.25
GO:00036	DNA bind	Molecular	0.01	0.16	Solyc09g0	Solyc09g0	2.14	1.10
GO:00036	DNA bind	Molecular	0.01	0.16	Solyc05g0	Solyc05g0	0.00	-14.00
GO:00036	DNA bind	Molecular	0.01	0.16	Solyc08g0	Solyc08g0	4.49	2.17
GO:00036	DNA bind	Molecular	0.01	0.16	Solyc04g0	Solyc04g0	2.35	1.23
GO:00036	DNA bind	Molecular	0.01	0.16	Solyc10g0	Solyc10g0	0.00	-9.03
GO:00036	DNA bind	Molecular	0.01	0.16	Solyc12g0	Solyc12g0	2.41	1.27
GO:00036	DNA bind	Molecular	0.01	0.16	Solyc05g0	Solyc05g0	31.58	4.98
GO:00036	DNA bind	Molecular	0.01	0.16	Solyc08g0	Solyc08g0	0.38	-1.39
GO:00036	DNA bind	Molecular	0.01	0.16	Solyc09g0	Solyc09g0	0.25	-1.98
GO:00036	DNA bind	Molecular	0.01	0.16	Solyc11g0	Solyc11g0	2.43	1.28
GO:00036	DNA bind	Molecular	0.01	0.16	Solyc08g0	Solyc08g0	0.37	-1.44
GO:00036	DNA bind	Molecular	0.01	0.16	Solyc10g0	Solyc10g0	2.26	1.18
GO:00036	DNA bind	Molecular	0.01	0.16	Solyc06g0	Solyc06g0	2.01	1.01
GO:00036	DNA bind	Molecular	0.01	0.16	Solyc04g0	Solyc04g0	0.07	-3.79
GO:00036	DNA bind	Molecular	0.01	0.16	Solyc06g0	Solyc06g0	0.39	-1.37
GO:00036	DNA bind	Molecular	0.01	0.16	Solyc01g0	Solyc01g0	0.40	-1.32
GO:00036	DNA bind	Molecular	0.01	0.16	Solyc02g0	Solyc02g0	0.00	-11.29
GO:00036	DNA bind	Molecular	0.01	0.16	Solyc12g0	Solyc12g0	2.29	1.20
GO:00036	DNA bind	Molecular	0.01	0.16	Solyc12g0	Solyc12g0	0.33	-1.61
GO:00036	DNA bind	Molecular	0.01	0.16	Solyc07g0	Solyc07g0	2.33	1.22

GO:00036	DNA bind	Molecular	0.01	0.16	Solyc08g0	Solyc08g0	2486.58	11.28
GO:00036	DNA bind	Molecular	0.01	0.16	Solyc05g0	Solyc05g0	2.76	1.47
GO:00036	DNA bind	Molecular	0.01	0.16	Solyc08g0	Solyc08g0	0.03	-5.23
GO:00036	DNA bind	Molecular	0.01	0.16	Solyc02g0	Solyc02g0	2.66	1.41
GO:00036	DNA bind	Molecular	0.01	0.16	Solyc06g0	Solyc06g0	0.00	-10.94
GO:00036	DNA bind	Molecular	0.01	0.16	Solyc09g0	Solyc09g0	1363.92	10.41
GO:00036	DNA bind	Molecular	0.01	0.16	Solyc01g1	Solyc01g1	1067.51	10.06
GO:00036	DNA bind	Molecular	0.01	0.16	Solyc01g0	Solyc01g0	2.57	1.36
GO:00036	DNA bind	Molecular	0.01	0.16	Solyc03g0	Solyc03g0	20.56	4.36
GO:00036	DNA bind	Molecular	0.01	0.16	Solyc02g0	Solyc02g0	2.33	1.22
GO:00036	DNA bind	Molecular	0.01	0.16	Solyc10g0	Solyc10g0	0.27	-1.87
GO:00036	DNA bind	Molecular	0.01	0.16	Solyc06g0	Solyc06g0	2.85	1.51
GO:00036	DNA bind	Molecular	0.01	0.16	Solyc02g0	Solyc02g0	0.39	-1.37
GO:00036	DNA bind	Molecular	0.01	0.16	Solyc04g0	Solyc04g0	2071.20	11.02
GO:00036	DNA bind	Molecular	0.01	0.16	Solyc03g0	Solyc03g0	0.00	-11.07
GO:00036	DNA bind	Molecular	0.01	0.16	Solyc02g0	Solyc02g0	8.19	3.03
GO:00036	DNA bind	Molecular	0.01	0.16	Solyc09g0	Solyc09g0	2.32	1.21
GO:00036	DNA bind	Molecular	0.01	0.16	Solyc03g1	Solyc03g1	0.44	-1.20
GO:00036	DNA bind	Molecular	0.01	0.16	Solyc04g0	Solyc04g0	0.48	-1.06
GO:00036	DNA bind	Molecular	0.01	0.16	Solyc10g0	Solyc10g0	2.02	1.01
GO:00036	DNA bind	Molecular	0.01	0.16	Solyc12g0	Solyc12g0	0.09	-3.53
GO:00036	DNA bind	Molecular	0.01	0.16	Solyc03g1	Solyc03g1	2361.02	11.21
GO:00036	DNA bind	Molecular	0.01	0.16	Solyc04g0	Solyc04g0	1733.54	10.76
GO:00036	DNA bind	Molecular	0.01	0.16	Solyc10g0	Solyc10g0	0.29	-1.77
GO:00036	DNA bind	Molecular	0.01	0.16	Solyc12g0	Solyc12g0	0.00	-12.05
GO:00036	DNA bind	Molecular	0.01	0.16	Solyc03g1	Solyc03g1	734.02	9.52
GO:00036	DNA bind	Molecular	0.01	0.16	Solyc07g0	Solyc07g0	42.77	5.42
GO:00036	DNA bind	Molecular	0.01	0.16	Solyc02g0	Solyc02g0	1446.12	10.50
GO:00036	DNA bind	Molecular	0.01	0.16	Solyc09g0	Solyc09g0	2.29	1.20
GO:00036	DNA bind	Molecular	0.01	0.16	Solyc01g0	Solyc01g0	6.82	2.77
GO:00036	DNA bind	Molecular	0.01	0.16	Solyc01g1	Solyc01g1	3.24	1.69
GO:00036	DNA bind	Molecular	0.01	0.16	Solyc10g0	Solyc10g0	2.60	1.38
GO:00036	DNA bind	Molecular	0.01	0.16	Solyc12g0	Solyc12g0	12.70	3.67
GO:00036	DNA bind	Molecular	0.01	0.16	Solyc08g0	Solyc08g0	3.40	1.77
GO:00036	DNA bind	Molecular	0.01	0.16	Solyc06g0	Solyc06g0	15.65	3.97
GO:00036	DNA bind	Molecular	0.01	0.16	Solyc01g0	Solyc01g0	0.48	-1.06
GO:00036	DNA bind	Molecular	0.01	0.16	Solyc06g0	Solyc06g0	8.85	3.15
GO:00036	DNA bind	Molecular	0.01	0.16	Solyc01g0	Solyc01g0	0.42	-1.24
GO:00036	DNA bind	Molecular	0.01	0.16	Solyc02g0	Solyc02g0	1506.28	10.56
GO:00036	DNA bind	Molecular	0.01	0.16	Solyc02g0	Solyc02g0	0.00	-12.40
GO:00036	DNA bind	Molecular	0.01	0.16	Solyc11g0	Solyc11g0	116.23	6.86
GO:00036	DNA bind	Molecular	0.01	0.16	Solyc03g1	Solyc03g1	356.83	8.48
GO:00036	DNA bind	Molecular	0.01	0.16	Solyc03g0	Solyc03g0	0.46	-1.13
GO:00036	DNA bind	Molecular	0.01	0.16	Solyc11g0	Solyc11g0	0.47	-1.09
GO:00036	DNA bind	Molecular	0.01	0.16	Solyc08g0	Solyc08g0	3.29	1.72
GO:00036	DNA bind	Molecular	0.01	0.16	Solyc10g0	Solyc10g0	0.10	-3.35
GO:00036	DNA bind	Molecular	0.01	0.16	Solyc01g0	Solyc01g0	0.24	-2.09
GO:00036	DNA bind	Molecular	0.01	0.16	Solyc02g0	Solyc02g0	0.06	-4.03
GO:00036	DNA bind	Molecular	0.01	0.16	Solyc06g0	PTI6	0.43	-1.22
GO:00036	DNA bind	Molecular	0.01	0.16	Solyc08g0	Solyc08g0	2.98	1.58
GO:00036	DNA bind	Molecular	0.01	0.16	Solyc04g0	Solyc04g0	0.00	-12.65

GO:00036	DNA bindi	Molecular	0.01	0.16	Solyc01g0	Solyc01g0	312.22	8.29
GO:00036	DNA bindi	Molecular	0.01	0.16	Solyc10g0	Solyc10g0	2.42	1.27
GO:00036	DNA bindi	Molecular	0.01	0.16	Solyc04g0	Solyc04g0	732.58	9.52
GO:00036	DNA bindi	Molecular	0.01	0.16	Solyc05g0	Solyc05g0	0.00	-10.39
GO:00036	DNA bindi	Molecular	0.01	0.16	Solyc02g0	Solyc02g0	0.00	-10.40
GO:00036	DNA bindi	Molecular	0.01	0.16	Solyc02g0	Solyc02g0	7.52	2.91
GO:00080	N-acetyltra	Molecular	0.01	0.16	Solyc09g0	Solyc09g0	0.35	-1.50
GO:00080	N-acetyltra	Molecular	0.01	0.16	Solyc02g0	Solyc02g0	0.46	-1.11
GO:00080	N-acetyltra	Molecular	0.01	0.16	Solyc08g0	Solyc08g0	0.38	-1.38
GO:00080	N-acetyltra	Molecular	0.01	0.16	Solyc09g0	Solyc09g0	0.12	-3.00
GO:00080	N-acetyltra	Molecular	0.01	0.16	Solyc05g0	Solyc05g0	11629.84	13.51
GO:00080	N-acetyltra	Molecular	0.01	0.16	Solyc09g0	Solyc09g0	0.00	-14.79
GO:00080	N-acetyltra	Molecular	0.01	0.16	Solyc09g0	Solyc09g0	0.00	-11.98
GO:00080	N-acetyltra	Molecular	0.01	0.16	Solyc06g0	Solyc06g0	2.03	1.02
GO:00080	N-acetyltra	Molecular	0.01	0.16	Solyc09g0	Solyc09g0	0.30	-1.74
GO:00068	ion transpo	Biological	0.01	0.16	Solyc01g1	Solyc01g1	0.29	-1.80
GO:00068	ion transpo	Biological	0.01	0.16	Solyc03g0	Solyc03g0	0.31	-1.67
GO:00068	ion transpo	Biological	0.01	0.16	Solyc11g0	Solyc11g0	0.24	-2.04
GO:00068	ion transpo	Biological	0.01	0.16	Solyc10g0	Solyc10g0	40.50	5.34
GO:00068	ion transpo	Biological	0.01	0.16	Solyc08g0	Solyc08g0	0.42	-1.27
GO:00068	ion transpo	Biological	0.01	0.16	Solyc02g0	Solyc02g0	4.67	2.22
GO:00068	ion transpo	Biological	0.01	0.16	Solyc10g0	Solyc10g0	0.03	-5.05
GO:00068	ion transpo	Biological	0.01	0.16	Solyc01g0	Solyc01g0	894.88	9.81
GO:00068	ion transpo	Biological	0.01	0.16	Solyc06g0	Solyc06g0	15.51	3.95
GO:00068	ion transpo	Biological	0.01	0.16	Solyc12g0	Solyc12g0	369.30	8.53
GO:00068	ion transpo	Biological	0.01	0.16	Solyc11g0	Solyc11g0	0.22	-2.19
GO:00068	ion transpo	Biological	0.01	0.16	Solyc08g0	Solyc08g0	2.51	1.33
GO:00166	oxidoreduc	Molecular	0.01	0.16	Solyc06g0	Solyc06g0	2.22	1.15
GO:00166	oxidoreduc	Molecular	0.01	0.16	Solyc03g0	Solyc03g0	2.38	1.25
GO:00166	oxidoreduc	Molecular	0.01	0.16	Solyc03g0	Solyc03g0	5.30	2.41
GO:00166	oxidoreduc	Molecular	0.01	0.16	Solyc01g1	Solyc01g1	4.98	2.32
GO:00166	oxidoreduc	Molecular	0.01	0.16	Solyc11g0	Solyc11g0	4.12	2.04
GO:00166	oxidoreduc	Molecular	0.01	0.16	Solyc02g0	Solyc02g0	0.30	-1.71
GO:00166	oxidoreduc	Molecular	0.01	0.16	Solyc02g0	Solyc02g0	0.50	-1.01
GO:00166	oxidoreduc	Molecular	0.01	0.16	Solyc03g0	Solyc03g0	2.79	1.48
GO:00166	oxidoreduc	Molecular	0.01	0.16	Solyc12g0	Solyc12g0	0.45	-1.15
GO:00166	oxidoreduc	Molecular	0.01	0.16	Solyc11g0	Solyc11g0	3.46	1.79
GO:00166	oxidoreduc	Molecular	0.01	0.16	Solyc02g0	Solyc02g0	2.01	1.01
GO:00166	oxidoreduc	Molecular	0.01	0.16	Solyc08g0	Solyc08g0	0.46	-1.13
GO:00166	oxidoreduc	Molecular	0.01	0.16	Solyc04g0	Solyc04g0	2.31	1.21
GO:00068	xenobiotic	Biological	0.01	0.16	Solyc11g0	Solyc11g0	0.35	-1.51
GO:00068	xenobiotic	Biological	0.01	0.16	Solyc03g1	Solyc03g1	0.25	-2.02
GO:00068	xenobiotic	Biological	0.01	0.16	Solyc10g0	Solyc10g0	2.60	1.38
GO:00068	xenobiotic	Biological	0.01	0.16	Solyc04g0	Solyc04g0	0.26	-1.94
GO:00068	xenobiotic	Biological	0.01	0.16	Solyc08g0	Solyc08g0	0.47	-1.08
GO:00068	xenobiotic	Biological	0.01	0.16	Solyc07g0	Solyc07g0	0.40	-1.33
GO:00056	cell wall	Cellular Co	0.01	0.16	Solyc10g0	PG2	9.48	3.25
GO:00056	cell wall	Cellular Co	0.01	0.16	Solyc04g0	Solyc04g0	2.70	1.43
GO:00056	cell wall	Cellular Co	0.01	0.16	Solyc12g0	Solyc12g0	24.67	4.62
GO:00056	cell wall	Cellular Co	0.01	0.16	Solyc03g1	Solyc03g1	2.20	1.14
GO:00056	cell wall	Cellular Co	0.01	0.16	Solyc10g0	CHI9	0.28	-1.84

GO:00056	cell wall	Cellular C	0.01	0.16	Solyc04g0	Solyc04g0	3.38	1.76
GO:00056	cell wall	Cellular C	0.01	0.16	Solyc02g0	Solyc02g0	2.85	1.51
GO:00056	cell wall	Cellular C	0.01	0.16	Solyc08g0	Solyc08g0	4.36	2.13
GO:00056	cell wall	Cellular C	0.01	0.16	Solyc01g0	XTH1	5.24	2.39
GO:00056	cell wall	Cellular C	0.01	0.16	Solyc09g0	Solyc09g0	0.36	-1.46
GO:00056	cell wall	Cellular C	0.01	0.16	Solyc06g0	Solyc06g0	0.11	-3.25
GO:00056	cell wall	Cellular C	0.01	0.16	Solyc01g0	Solyc01g0	0.31	-1.69
GO:00056	cell wall	Cellular C	0.01	0.16	Solyc03g1	Solyc03g1	0.15	-2.75
GO:00056	cell wall	Cellular C	0.01	0.16	Solyc02g0	Solyc02g0	0.35	-1.51
GO:00056	cell wall	Cellular C	0.01	0.16	Solyc05g0	Solyc05g0	20.96	4.39
GO:00056	cell wall	Cellular C	0.01	0.16	Solyc03g0	Solyc03g0	0.46	-1.12
GO:00097	response to	Biological	0.01	0.16	Solyc07g0	ACO1	2.82	1.49
GO:00097	response to	Biological	0.01	0.16	Solyc12g0	Solyc12g0	2.97	1.57
GO:00097	response to	Biological	0.01	0.16	Solyc03g1	Solyc03g1	4.55	2.18
GO:00097	response to	Biological	0.01	0.16	Solyc02g0	Solyc02g0	2.56	1.36
GO:00097	response to	Biological	0.01	0.16	Solyc07g0	PSBR	3.05	1.61
GO:00097	response to	Biological	0.01	0.16	Solyc09g0	Solyc09g0	25.41	4.67
GO:00097	response to	Biological	0.01	0.16	Solyc01g0	Solyc01g0	550.66	9.11
GO:00712	cellular res	Biological	0.01	0.16	Solyc07g0	ACO1	2.82	1.49
GO:00712	cellular res	Biological	0.01	0.16	Solyc02g0	Solyc02g0	2.56	1.36
GO:00712	cellular res	Biological	0.01	0.16	Solyc01g0	Solyc01g0	0.42	-1.24
GO:00712	cellular res	Biological	0.01	0.16	Solyc04g0	Solyc04g0	732.58	9.52
GO:00109	negative re	Biological	0.01	0.16	Solyc11g0	Solyc11g0	0.07	-3.83
GO:00109	negative re	Biological	0.01	0.16	Solyc09g0	Solyc09g0	10879.94	13.41
GO:00109	negative re	Biological	0.01	0.16	Solyc07g0	Solyc07g0	3.98	1.99
GO:00109	negative re	Biological	0.01	0.16	Solyc03g0	Solyc03g0	11.28	3.50
GO:00159	glutamate	Molecular	0.01	0.16	Solyc03g0	Solyc03g0	7.65	2.93
GO:00159	glutamate	Molecular	0.01	0.16	Solyc08g0	Solyc08g0	14.34	3.84
GO:00098	cinnamic a	Biological	0.01	0.16	Solyc10g0	Solyc10g0	4.26	2.09
GO:00098	cinnamic a	Biological	0.01	0.16	Solyc09g0	Solyc09g0	0.27	-1.91
GO:00306	polyketide	Biological	0.01	0.16	Solyc05g0	Solyc05g0	0.06	-4.07
GO:00306	polyketide	Biological	0.01	0.16	Solyc09g0	Solyc09g0	0.06	-4.05
GO:00044	hydroxyme	Molecular	0.01	0.16	Solyc03g0	Solyc03g0	2.38	1.25
GO:00044	hydroxyme	Molecular	0.01	0.16	Solyc02g0	Solyc02g0	0.50	-1.01
GO:00159	coenzyme	Biological	0.01	0.16	Solyc03g0	Solyc03g0	2.38	1.25
GO:00159	coenzyme	Biological	0.01	0.16	Solyc02g0	Solyc02g0	0.50	-1.01
GO:00045	beta-N-ace	Molecular	0.01	0.16	Solyc01g0	Solyc01g0	2.01	1.01
GO:00045	beta-N-ace	Molecular	0.01	0.16	Solyc11g0	Solyc11g0	2.74	1.46
GO:00007	heterochro	Cellular C	0.01	0.16	Solyc01g0	Solyc01g0	3.72	1.90
GO:00007	heterochro	Cellular C	0.01	0.16	Solyc05g0	Solyc05g0	2.24	1.16
GO:00103	stromule	Cellular C	0.01	0.16	Solyc12g0	Solyc12g0	2.97	1.57
GO:00103	stromule	Cellular C	0.01	0.16	Solyc09g0	Solyc09g0	25.41	4.67
GO:00971	organic cy	Molecular	0.01	0.16	Solyc09g0	Solyc09g0	0.40	-1.34
GO:00971	organic cy	Molecular	0.01	0.16	Solyc12g0	Solyc12g0	2.29	1.20
GO:19013	heterocycli	Molecular	0.01	0.16	Solyc09g0	Solyc09g0	0.40	-1.34
GO:19013	heterocycli	Molecular	0.01	0.16	Solyc12g0	Solyc12g0	2.29	1.20
GO:00101	nonphotoc	Biological	0.01	0.16	Solyc12g0	Solyc12g0	2.10	1.07
GO:00101	nonphotoc	Biological	0.01	0.16	Solyc06g0	PSBS	2.99	1.58
GO:00300	chloroplast	Cellular C	0.01	0.16	Solyc07g0	Solyc07g0	2.47	1.30
GO:00300	chloroplast	Cellular C	0.01	0.16	Solyc08g0	Solyc08g0	1537.41	10.59
GO:00007	recombina	Biological	0.01	0.16	Solyc08g0	Solyc08g0	9.19	3.20

GO:00007	recombina	Biological	0.01	0.16	Solyc07g0	Solyc07g0	0.19	-2.41
GO:00068	nitrogen cc	Biological	0.02	0.17	Solyc03g0	Solyc03g0	7.65	2.93
GO:00068	nitrogen cc	Biological	0.02	0.17	Solyc03g0	Solyc03g0	2.10	1.07
GO:00068	nitrogen cc	Biological	0.02	0.17	Solyc04g0	Solyc04g0	0.40	-1.31
GO:00068	nitrogen cc	Biological	0.02	0.17	Solyc12g0	Solyc12g0	13681.82	13.74
GO:00068	nitrogen cc	Biological	0.02	0.17	Solyc12g0	Solyc12g0	12341.84	13.59
GO:00068	nitrogen cc	Biological	0.02	0.17	Solyc01g0	Solyc01g0	550.66	9.11
GO:00515	2 iron, 2 su	Molecular	0.02	0.17	Solyc11g0	Solyc11g0	2.92	1.55
GO:00515	2 iron, 2 su	Molecular	0.02	0.17	Solyc12g0	Solyc12g0	2.10	1.07
GO:00515	2 iron, 2 su	Molecular	0.02	0.17	Solyc11g0	Solyc11g0	0.32	-1.67
GO:00515	2 iron, 2 su	Molecular	0.02	0.17	Solyc11g0	Solyc11g0	2.20	1.14
GO:00515	2 iron, 2 su	Molecular	0.02	0.17	Solyc07g0	Solyc07g0	2.07	1.05
GO:00515	2 iron, 2 su	Molecular	0.02	0.17	Solyc04g0	Solyc04g0	39.10	5.29
GO:00515	2 iron, 2 su	Molecular	0.02	0.17	Solyc04g0	Solyc04g0	32.87	5.04
GO:00515	2 iron, 2 su	Molecular	0.02	0.17	Solyc04g0	Solyc04g0	883.93	9.79
GO:00065	glutamine	Biological	0.02	0.17	Solyc04g0	Solyc04g0	0.40	-1.31
GO:00065	glutamine	Biological	0.02	0.17	Solyc12g0	Solyc12g0	12341.84	13.59
GO:00065	glutamine	Biological	0.02	0.17	Solyc01g0	Solyc01g0	550.66	9.11
GO:00153	anion:anio	Molecular	0.02	0.17	Solyc12g0	Solyc12g0	0.37	-1.44
GO:00153	anion:anio	Molecular	0.02	0.17	Solyc04g0	Solyc04g0	0.41	-1.29
GO:00153	anion:anio	Molecular	0.02	0.17	Solyc09g0	Solyc09g0	0.49	-1.03
GO:00081	UDP-glycc	Molecular	0.02	0.18	Solyc09g0	Solyc09g0	0.05	-4.31
GO:00081	UDP-glycc	Molecular	0.02	0.18	Solyc01g1	Solyc01g1	4.75	2.25
GO:00081	UDP-glycc	Molecular	0.02	0.18	Solyc02g0	Solyc02g0	11.82	3.56
GO:00081	UDP-glycc	Molecular	0.02	0.18	Solyc09g0	Solyc09g0	0.23	-2.13
GO:00081	UDP-glycc	Molecular	0.02	0.18	Solyc04g0	Solyc04g0	0.10	-3.29
GO:00081	UDP-glycc	Molecular	0.02	0.18	Solyc01g1	Solyc01g1	2.62	1.39
GO:00081	UDP-glycc	Molecular	0.02	0.18	Solyc03g0	Solyc03g0	4.81	2.26
GO:00081	UDP-glycc	Molecular	0.02	0.18	Solyc12g0	Solyc12g0	2.38	1.25
GO:00081	UDP-glycc	Molecular	0.02	0.18	Solyc12g0	Solyc12g0	7107.62	12.80
GO:00081	UDP-glycc	Molecular	0.02	0.18	Solyc03g0	Solyc03g0	7.07	2.82
GO:00081	UDP-glycc	Molecular	0.02	0.18	Solyc11g0	Solyc11g0	0.25	-2.02
GO:00081	UDP-glycc	Molecular	0.02	0.18	Solyc07g0	Solyc07g0	6.57	2.72
GO:00081	UDP-glycc	Molecular	0.02	0.18	Solyc04g0	Solyc04g0	0.22	-2.21
GO:00081	UDP-glycc	Molecular	0.02	0.18	Solyc07g0	Solyc07g0	0.00	-11.10
GO:00081	UDP-glycc	Molecular	0.02	0.18	Solyc12g0	Solyc12g0	0.00	-9.95
GO:00081	UDP-glycc	Molecular	0.02	0.18	Solyc03g0	Solyc03g0	0.39	-1.35
GO:00081	UDP-glycc	Molecular	0.02	0.18	Solyc01g1	Solyc01g1	0.47	-1.07
GO:00081	UDP-glycc	Molecular	0.02	0.18	Solyc03g0	Solyc03g0	420.75	8.72
GO:00081	UDP-glycc	Molecular	0.02	0.18	Solyc01g1	Solyc01g1	2.13	1.09
GO:00152	antiporter a	Molecular	0.02	0.18	Solyc01g1	Solyc01g1	5.77	2.53
GO:00152	antiporter a	Molecular	0.02	0.18	Solyc04g0	Solyc04g0	2.19	1.13
GO:00152	antiporter a	Molecular	0.02	0.18	Solyc11g0	Solyc11g0	0.35	-1.51
GO:00152	antiporter a	Molecular	0.02	0.18	Solyc12g0	Solyc12g0	5.32	2.41
GO:00152	antiporter a	Molecular	0.02	0.18	Solyc03g1	Solyc03g1	0.25	-2.02
GO:00152	antiporter a	Molecular	0.02	0.18	Solyc10g0	Solyc10g0	2.60	1.38
GO:00152	antiporter a	Molecular	0.02	0.18	Solyc07g0	Solyc07g0	3.79	1.92
GO:00152	antiporter a	Molecular	0.02	0.18	Solyc04g0	Solyc04g0	0.26	-1.94
GO:00152	antiporter a	Molecular	0.02	0.18	Solyc01g0	Solyc01g0	1991.27	10.96
GO:00152	antiporter a	Molecular	0.02	0.18	Solyc08g0	Solyc08g0	0.47	-1.08
GO:00152	antiporter a	Molecular	0.02	0.18	Solyc07g0	Solyc07g0	0.40	-1.33

GO:00002	polysaccha	Biological	0.02	0.18	Solyc09g0	Solyc09g0	0.34	-1.54
GO:00002	polysaccha	Biological	0.02	0.18	Solyc10g0	CHI9	0.28	-1.84
GO:00002	polysaccha	Biological	0.02	0.18	Solyc08g0	Solyc08g0	6.25	2.64
GO:00002	polysaccha	Biological	0.02	0.18	Solyc08g0	Solyc08g0	2.18	1.12
GO:00002	polysaccha	Biological	0.02	0.18	Solyc02g0	Solyc02g0	0.33	-1.59
GO:00098	fruit ripeni	Biological	0.02	0.20	Solyc07g0	ACO1	2.82	1.49
GO:00098	fruit ripeni	Biological	0.02	0.20	Solyc10g0	PG2	9.48	3.25
GO:00098	fruit ripeni	Biological	0.02	0.20	Solyc09g0	Solyc09g0	0.05	-4.31
GO:00098	fruit ripeni	Biological	0.02	0.20	Solyc05g0	Solyc05g0	0.35	-1.52
GO:00046	peroxidase	Molecular	0.02	0.22	Solyc04g0	Solyc04g0	2.70	1.43
GO:00046	peroxidase	Molecular	0.02	0.22	Solyc04g0	Solyc04g0	3.53	1.82
GO:00046	peroxidase	Molecular	0.02	0.22	Solyc11g0	Solyc11g0	8.62	3.11
GO:00046	peroxidase	Molecular	0.02	0.22	Solyc01g0	Solyc01g0	14.73	3.88
GO:00046	peroxidase	Molecular	0.02	0.22	Solyc02g0	Solyc02g0	8.87	3.15
GO:00046	peroxidase	Molecular	0.02	0.22	Solyc05g0	Solyc05g0	4.33	2.11
GO:00046	peroxidase	Molecular	0.02	0.22	Solyc02g0	Solyc02g0	2.54	1.34
GO:00046	peroxidase	Molecular	0.02	0.22	Solyc03g0	Solyc03g0	5511.05	12.43
GO:00046	peroxidase	Molecular	0.02	0.22	Solyc03g1	Solyc03g1	0.48	-1.07
GO:00046	peroxidase	Molecular	0.02	0.22	Solyc10g0	Solyc10g0	1604.71	10.65
GO:00046	peroxidase	Molecular	0.02	0.22	Solyc01g1	Solyc01g1	4.37	2.13
GO:00046	peroxidase	Molecular	0.02	0.22	Solyc01g0	Solyc01g0	3.53	1.82
GO:00046	peroxidase	Molecular	0.02	0.22	Solyc02g0	Solyc02g0	0.17	-2.56
GO:00046	peroxidase	Molecular	0.02	0.22	Solyc03g0	Solyc03g0	0.41	-1.30
GO:00046	peroxidase	Molecular	0.02	0.22	Solyc04g0	Solyc04g0	0.48	-1.06
GO:00046	peroxidase	Molecular	0.02	0.22	Solyc06g0	Solyc06g0	0.25	-2.01
GO:00046	peroxidase	Molecular	0.02	0.22	Solyc07g0	Solyc07g0	2.87	1.52
GO:19909	xenobiotic	Biological	0.02	0.22	Solyc04g0	Solyc04g0	2.19	1.13
GO:19909	xenobiotic	Biological	0.02	0.22	Solyc11g0	Solyc11g0	0.35	-1.51
GO:19909	xenobiotic	Biological	0.02	0.22	Solyc03g1	Solyc03g1	0.25	-2.02
GO:19909	xenobiotic	Biological	0.02	0.22	Solyc10g0	Solyc10g0	2.60	1.38
GO:19909	xenobiotic	Biological	0.02	0.22	Solyc07g0	Solyc07g0	3.79	1.92
GO:19909	xenobiotic	Biological	0.02	0.22	Solyc04g0	Solyc04g0	0.26	-1.94
GO:19909	xenobiotic	Biological	0.02	0.22	Solyc08g0	Solyc08g0	0.47	-1.08
GO:19909	xenobiotic	Biological	0.02	0.22	Solyc07g0	Solyc07g0	0.40	-1.33
GO:00096	response to	Biological	0.02	0.22	Solyc07g0	ACO1	2.82	1.49
GO:00096	response to	Biological	0.02	0.22	Solyc02g0	Solyc02g0	2.56	1.36
GO:00096	response to	Biological	0.02	0.22	Solyc03g0	Solyc03g0	3.84	1.94
GO:00469	acyltransfe	Molecular	0.02	0.22	Solyc08g0	Solyc08g0	2.65	1.41
GO:00469	acyltransfe	Molecular	0.02	0.22	Solyc07g0	Solyc07g0	3.93	1.98
GO:00469	acyltransfe	Molecular	0.02	0.22	Solyc12g0	Solyc12g0	7.81	2.96
GO:00102	oxygen evo	Molecular	0.02	0.22	Solyc07g0	Solyc07g0	3.28	1.71
GO:00102	oxygen evo	Molecular	0.02	0.22	Solyc02g0	PSBO	5.02	2.33
GO:00102	oxygen evo	Molecular	0.02	0.22	Solyc09g0	Solyc09g0	0.00	-13.14
GO:00099	leaf morph	Biological	0.02	0.22	Solyc03g0	Solyc03g0	3.84	1.94
GO:00099	leaf morph	Biological	0.02	0.22	Solyc02g0	Solyc02g0	0.48	-1.05
GO:00099	leaf morph	Biological	0.02	0.22	Solyc08g0	Solyc08g0	4.19	2.07
GO:00060	fructose 6-	Biological	0.02	0.23	Solyc08g0	Solyc08g0	3.48	1.80
GO:00060	fructose 6-	Biological	0.02	0.23	Solyc10g0	Solyc10g0	2.23	1.16
GO:00060	fructose 6-	Biological	0.02	0.23	Solyc09g0	Solyc09g0	25.41	4.67
GO:00060	fructose 6-	Biological	0.02	0.23	Solyc04g0	Solyc04g0	0.41	-1.27
GO:00058	plasma me	Cellular Co	0.02	0.23	Solyc09g0	Solyc09g0	6.99	2.80

GO:00058	plasma me	Cellular C	0.02	0.23	Solyc07g0	Solyc07g0	4.14	2.05
GO:00058	plasma me	Cellular C	0.02	0.23	Solyc04g0	Solyc04g0	2.70	1.43
GO:00058	plasma me	Cellular C	0.02	0.23	Solyc01g0	Solyc01g0	2.01	1.01
GO:00058	plasma me	Cellular C	0.02	0.23	Solyc12g0	Solyc12g0	9.07	3.18
GO:00058	plasma me	Cellular C	0.02	0.23	Solyc07g0	Solyc07g0	7.24	2.86
GO:00058	plasma me	Cellular C	0.02	0.23	Solyc03g0	Solyc03g0	5.61	2.49
GO:00058	plasma me	Cellular C	0.02	0.23	Solyc09g0	Solyc09g0	2.40	1.26
GO:00058	plasma me	Cellular C	0.02	0.23	Solyc06g0	Solyc06g0	2.40	1.26
GO:00058	plasma me	Cellular C	0.02	0.23	Solyc01g1	Solyc01g1	72.82	6.19
GO:00058	plasma me	Cellular C	0.02	0.23	Solyc01g0	Solyc01g0	0.32	-1.65
GO:00058	plasma me	Cellular C	0.02	0.23	Solyc02g0	Solyc02g0	0.44	-1.19
GO:00058	plasma me	Cellular C	0.02	0.23	Solyc09g0	Solyc09g0	0.48	-1.06
GO:00058	plasma me	Cellular C	0.02	0.23	Solyc06g0	Solyc06g0	2.02	1.02
GO:00058	plasma me	Cellular C	0.02	0.23	Solyc02g0	Solyc02g0	6.74	2.75
GO:00058	plasma me	Cellular C	0.02	0.23	Solyc12g0	Solyc12g0	2.10	1.07
GO:00058	plasma me	Cellular C	0.02	0.23	Solyc06g0	Solyc06g0	0.37	-1.44
GO:00058	plasma me	Cellular C	0.02	0.23	Solyc05g0	Solyc05g0	0.50	-1.00
GO:00058	plasma me	Cellular C	0.02	0.23	Solyc10g0	Solyc10g0	2.36	1.24
GO:00058	plasma me	Cellular C	0.02	0.23	Solyc03g1	Solyc03g1	2.04	1.03
GO:00058	plasma me	Cellular C	0.02	0.23	Solyc11g0	Solyc11g0	6.63	2.73
GO:00058	plasma me	Cellular C	0.02	0.23	Solyc01g1	Solyc01g1	14.96	3.90
GO:00058	plasma me	Cellular C	0.02	0.23	Solyc11g0	Solyc11g0	3.29	1.72
GO:00058	plasma me	Cellular C	0.02	0.23	Solyc09g0	Solyc09g0	0.47	-1.07
GO:00058	plasma me	Cellular C	0.02	0.23	Solyc05g0	Solyc05g0	0.43	-1.22
GO:00058	plasma me	Cellular C	0.02	0.23	Solyc01g0	Solyc01g0	2.56	1.36
GO:00058	plasma me	Cellular C	0.02	0.23	Solyc03g1	Solyc03g1	0.49	-1.04
GO:00058	plasma me	Cellular C	0.02	0.23	Solyc10g0	Solyc10g0	2.02	1.01
GO:00058	plasma me	Cellular C	0.02	0.23	Solyc03g1	Solyc03g1	0.33	-1.59
GO:00058	plasma me	Cellular C	0.02	0.23	Solyc11g0	Solyc11g0	2.78	1.48
GO:00058	plasma me	Cellular C	0.02	0.23	Solyc12g0	Solyc12g0	0.37	-1.44
GO:00058	plasma me	Cellular C	0.02	0.23	Solyc04g0	Solyc04g0	0.29	-1.76
GO:00058	plasma me	Cellular C	0.02	0.23	Solyc04g0	Solyc04g0	2.84	1.50
GO:00058	plasma me	Cellular C	0.02	0.23	Solyc12g0	Solyc12g0	44.33	5.47
GO:00058	plasma me	Cellular C	0.02	0.23	Solyc12g0	Solyc12g0	3.03	1.60
GO:00058	plasma me	Cellular C	0.02	0.23	Solyc08g0	Solyc08g0	3738.24	11.87
GO:00058	plasma me	Cellular C	0.02	0.23	Solyc01g1	Solyc01g1	0.20	-2.35
GO:00058	plasma me	Cellular C	0.02	0.23	Solyc10g0	Solyc10g0	4.22	2.08
GO:00058	plasma me	Cellular C	0.02	0.23	Solyc01g0	Solyc01g0	0.19	-2.40
GO:00058	plasma me	Cellular C	0.02	0.23	Solyc05g0	Solyc05g0	2.50	1.32
GO:00058	plasma me	Cellular C	0.02	0.23	Solyc10g0	Solyc10g0	0.37	-1.43
GO:00058	plasma me	Cellular C	0.02	0.23	Solyc10g0	Solyc10g0	0.03	-5.05
GO:00058	plasma me	Cellular C	0.02	0.23	Solyc03g0	Solyc03g0	2.40	1.26
GO:00058	plasma me	Cellular C	0.02	0.23	Solyc02g0	Solyc02g0	0.20	-2.29
GO:00058	plasma me	Cellular C	0.02	0.23	Solyc01g0	Solyc01g0	0.43	-1.23
GO:00058	plasma me	Cellular C	0.02	0.23	Solyc09g0	Solyc09g0	6.94	2.79
GO:00058	plasma me	Cellular C	0.02	0.23	Solyc02g0	Solyc02g0	0.19	-2.37
GO:00058	plasma me	Cellular C	0.02	0.23	Solyc06g0	Solyc06g0	6.96	2.80
GO:00058	plasma me	Cellular C	0.02	0.23	Solyc03g1	Solyc03g1	0.06	-4.15
GO:00058	plasma me	Cellular C	0.02	0.23	Solyc06g0	Solyc06g0	15.51	3.95
GO:00058	plasma me	Cellular C	0.02	0.23	Solyc07g0	Solyc07g0	714.98	9.48
GO:00058	plasma me	Cellular C	0.02	0.23	Solyc02g0	Solyc02g0	0.17	-2.58

GO:00058	plasma me	Cellular C	0.02	0.23	Solyc07g0	Solyc07g0	2.39	1.26
GO:00058	plasma me	Cellular C	0.02	0.23	Solyc08g0	Solyc08g0	13.23	3.73
GO:00058	plasma me	Cellular C	0.02	0.23	Solyc08g0	Solyc08g0	2.56	1.36
GO:00058	plasma me	Cellular C	0.02	0.23	Solyc08g0	Solyc08g0	9.19	3.20
GO:00058	plasma me	Cellular C	0.02	0.23	Solyc04g0	Solyc04g0	7.72	2.95
GO:00058	plasma me	Cellular C	0.02	0.23	Solyc06g0	Solyc06g0	13.67	3.77
GO:00058	plasma me	Cellular C	0.02	0.23	Solyc12g0	Solyc12g0	4.99	2.32
GO:00058	plasma me	Cellular C	0.02	0.23	Solyc08g0	Solyc08g0	4.19	2.07
GO:00058	plasma me	Cellular C	0.02	0.23	Solyc02g0	Solyc02g0	0.07	-3.78
GO:00058	plasma me	Cellular C	0.02	0.23	Solyc10g0	Solyc10g0	2.16	1.11
GO:00058	plasma me	Cellular C	0.02	0.23	Solyc06g0	Solyc06g0	0.44	-1.17
GO:00058	plasma me	Cellular C	0.02	0.23	Solyc11g0	Solyc11g0	0.43	-1.20
GO:00058	plasma me	Cellular C	0.02	0.23	Solyc02g0	Solyc02g0	517.85	9.02
GO:00058	plasma me	Cellular C	0.02	0.23	Solyc07g0	Solyc07g0	0.39	-1.35
GO:00058	plasma me	Cellular C	0.02	0.23	Solyc03g1	Solyc03g1	0.00	-8.90
GO:00058	plasma me	Cellular C	0.02	0.23	Solyc11g0	Solyc11g0	18.97	4.25
GO:00228	transmemb	Molecular	0.02	0.23	Solyc12g0	Solyc12g0	4.94	2.30
GO:00228	transmemb	Molecular	0.02	0.23	Solyc03g1	Solyc03g1	3.43	1.78
GO:00228	transmemb	Molecular	0.02	0.23	Solyc02g0	Solyc02g0	2.09	1.06
GO:00228	transmemb	Molecular	0.02	0.23	Solyc07g0	Solyc07g0	2.63	1.40
GO:00228	transmemb	Molecular	0.02	0.23	Solyc05g0	Solyc05g0	2.41	1.27
GO:00228	transmemb	Molecular	0.02	0.23	Solyc06g0	Solyc06g0	0.33	-1.58
GO:00228	transmemb	Molecular	0.02	0.23	Solyc06g0	Solyc06g0	2.35	1.23
GO:00228	transmemb	Molecular	0.02	0.23	Solyc04g0	Solyc04g0	0.38	-1.41
GO:00228	transmemb	Molecular	0.02	0.23	Solyc01g1	Solyc01g1	3.01	1.59
GO:00228	transmemb	Molecular	0.02	0.23	Solyc05g0	Solyc05g0	0.28	-1.82
GO:00228	transmemb	Molecular	0.02	0.23	Solyc04g0	Solyc04g0	7.43	2.89
GO:00228	transmemb	Molecular	0.02	0.23	Solyc10g0	Solyc10g0	2.36	1.24
GO:00228	transmemb	Molecular	0.02	0.23	Solyc01g0	Solyc01g0	0.42	-1.26
GO:00228	transmemb	Molecular	0.02	0.23	Solyc01g1	Solyc01g1	14.96	3.90
GO:00228	transmemb	Molecular	0.02	0.23	Solyc01g0	Solyc01g0	5.13	2.36
GO:00228	transmemb	Molecular	0.02	0.23	Solyc05g0	Solyc05g0	3.13	1.65
GO:00228	transmemb	Molecular	0.02	0.23	Solyc02g0	Solyc02g0	0.38	-1.39
GO:00228	transmemb	Molecular	0.02	0.23	Solyc10g0	Solyc10g0	2.02	1.01
GO:00228	transmemb	Molecular	0.02	0.23	Solyc01g1	Solyc01g1	3.40	1.77
GO:00228	transmemb	Molecular	0.02	0.23	Solyc10g0	Solyc10g0	0.38	-1.41
GO:00228	transmemb	Molecular	0.02	0.23	Solyc02g0	Solyc02g0	2.28	1.19
GO:00228	transmemb	Molecular	0.02	0.23	Solyc03g0	Solyc03g0	1336.08	10.38
GO:00228	transmemb	Molecular	0.02	0.23	Solyc05g0	Solyc05g0	1020.85	10.00
GO:00228	transmemb	Molecular	0.02	0.23	Solyc12g0	Solyc12g0	0.45	-1.17
GO:00228	transmemb	Molecular	0.02	0.23	Solyc02g0	Solyc02g0	7.78	2.96
GO:00228	transmemb	Molecular	0.02	0.23	Solyc09g0	Solyc09g0	429.36	8.75
GO:00228	transmemb	Molecular	0.02	0.23	Solyc07g0	Solyc07g0	714.98	9.48
GO:00228	transmemb	Molecular	0.02	0.23	Solyc06g0	Solyc06g0	0.32	-1.62
GO:00228	transmemb	Molecular	0.02	0.23	Solyc07g0	Solyc07g0	401.43	8.65
GO:00228	transmemb	Molecular	0.02	0.23	Solyc06g0	Solyc06g0	3.28	1.71
GO:00228	transmemb	Molecular	0.02	0.23	Solyc06g0	Solyc06g0	0.44	-1.17
GO:00228	transmemb	Molecular	0.02	0.23	Solyc11g0	Solyc11g0	0.43	-1.20
GO:00228	transmemb	Molecular	0.02	0.23	Solyc09g0	Solyc09g0	0.38	-1.39
GO:00228	transmemb	Molecular	0.02	0.23	Solyc03g0	Solyc03g0	4.00	2.00
GO:00104	xyloglucan	Biological	0.02	0.23	Solyc12g0	Solyc12g0	24.67	4.62

GO:00104	xyloglucan	Biological	0.02	0.23	Solyc04g0	Solyc04g0	3.38	1.76
GO:00104	xyloglucan	Biological	0.02	0.23	Solyc01g0	XTH1	5.24	2.39
GO:00104	xyloglucan	Biological	0.02	0.23	Solyc09g0	Solyc09g0	0.36	-1.46
GO:00104	xyloglucan	Biological	0.02	0.23	Solyc02g0	Solyc02g0	0.35	-1.51
GO:00104	xyloglucan	Biological	0.02	0.23	Solyc03g0	Solyc03g0	0.46	-1.12
GO:00467	protein aut	Biological	0.03	0.25	Solyc01g1	Solyc01g1	72.82	6.19
GO:00467	protein aut	Biological	0.03	0.25	Solyc03g0	Solyc03g0	7.56	2.92
GO:00467	protein aut	Biological	0.03	0.25	Solyc02g0	Solyc02g0	0.44	-1.19
GO:00467	protein aut	Biological	0.03	0.25	Solyc04g0	Solyc04g0	0.41	-1.29
GO:00467	protein aut	Biological	0.03	0.25	Solyc04g0	Solyc04g0	0.46	-1.11
GO:00467	protein aut	Biological	0.03	0.25	Solyc08g0	Solyc08g0	0.50	-1.00
GO:00467	protein aut	Biological	0.03	0.25	Solyc04g0	Solyc04g0	0.39	-1.37
GO:00467	protein aut	Biological	0.03	0.25	Solyc03g0	Solyc03g0	2.40	1.26
GO:00467	protein aut	Biological	0.03	0.25	Solyc08g0	Solyc08g0	4.19	2.07
GO:00467	protein aut	Biological	0.03	0.25	Solyc03g0	Solyc03g0	0.36	-1.46
GO:00467	protein aut	Biological	0.03	0.25	Solyc05g0	Solyc05g0	0.31	-1.68
GO:00467	protein aut	Biological	0.03	0.25	Solyc07g0	Solyc07g0	0.39	-1.35
GO:00467	protein aut	Biological	0.03	0.25	Solyc03g0	Solyc03g0	0.39	-1.37
GO:00066	fatty acid r	Biological	0.03	0.25	Solyc01g0	Solyc01g0	2.18	1.13
GO:00066	fatty acid r	Biological	0.03	0.25	Solyc11g0	Solyc11g0	3.25	1.70
GO:00066	fatty acid r	Biological	0.03	0.25	Solyc11g0	AOS2	3.20	1.68
GO:00066	fatty acid r	Biological	0.03	0.25	Solyc08g0	LOX1.1	2.60	1.38
GO:00066	fatty acid r	Biological	0.03	0.25	Solyc03g1	Solyc03g1	4.55	2.18
GO:00066	fatty acid r	Biological	0.03	0.25	Solyc06g0	Solyc06g0	2.19	1.13
GO:00066	fatty acid r	Biological	0.03	0.25	Solyc04g0	Solyc04g0	0.00	-13.91
GO:00066	fatty acid r	Biological	0.03	0.25	Solyc12g0	Solyc12g0	2.73	1.45
GO:00506	flavin ader	Molecular	0.03	0.25	Solyc03g0	Solyc03g0	7.65	2.93
GO:00506	flavin ader	Molecular	0.03	0.25	Solyc03g0	Solyc03g0	4.05	2.02
GO:00506	flavin ader	Molecular	0.03	0.25	Solyc01g1	Solyc01g1	11.39	3.51
GO:00506	flavin ader	Molecular	0.03	0.25	Solyc10g0	Solyc10g0	3.13	1.64
GO:00506	flavin ader	Molecular	0.03	0.25	Solyc06g0	Solyc06g0	0.32	-1.66
GO:00506	flavin ader	Molecular	0.03	0.25	Solyc06g0	Solyc06g0	2.15	1.10
GO:00506	flavin ader	Molecular	0.03	0.25	Solyc04g0	Solyc04g0	2.83	1.50
GO:00506	flavin ader	Molecular	0.03	0.25	Solyc02g0	Solyc02g0	0.16	-2.68
GO:00506	flavin ader	Molecular	0.03	0.25	Solyc05g0	Solyc05g0	3.22	1.69
GO:00506	flavin ader	Molecular	0.03	0.25	Solyc11g0	Solyc11g0	0.32	-1.67
GO:00506	flavin ader	Molecular	0.03	0.25	Solyc06g0	Solyc06g0	3.53	1.82
GO:00506	flavin ader	Molecular	0.03	0.25	Solyc10g0	Solyc10g0	2.27	1.19
GO:00506	flavin ader	Molecular	0.03	0.25	Solyc06g0	Solyc06g0	0.11	-3.23
GO:00506	flavin ader	Molecular	0.03	0.25	Solyc02g0	Solyc02g0	6.79	2.76
GO:00506	flavin ader	Molecular	0.03	0.25	Solyc02g0	Solyc02g0	0.37	-1.45
GO:00039	acid phosp	Molecular	0.03	0.25	Solyc09g0	Solyc09g0	0.47	-1.07
GO:00039	acid phosp	Molecular	0.03	0.25	Solyc03g0	Solyc03g0	0.22	-2.16
GO:00039	acid phosp	Molecular	0.03	0.25	Solyc07g0	Solyc07g0	2.09	1.06
GO:00039	acid phosp	Molecular	0.03	0.25	Solyc07g0	Solyc07g0	4.69	2.23
GO:00039	acid phosp	Molecular	0.03	0.25	Solyc07g0	Solyc07g0	11.18	3.48
GO:00039	acid phosp	Molecular	0.03	0.25	Solyc07g0	Solyc07g0	470.92	8.88
GO:00065	glutamate	Biological	0.03	0.25	Solyc03g0	Solyc03g0	7.65	2.93
GO:00065	glutamate	Biological	0.03	0.25	Solyc08g0	Solyc08g0	14.34	3.84
GO:00455	phenylalan	Molecular	0.03	0.25	Solyc10g0	Solyc10g0	4.26	2.09
GO:00455	phenylalan	Molecular	0.03	0.25	Solyc09g0	Solyc09g0	0.27	-1.91

GO:00091	purine nuc	Biological	0.03	0.25	Solyc01g0	Solyc01g0	2.62	1.39
GO:00091	purine nuc	Biological	0.03	0.25	Solyc01g1	Solyc01g1	2993.63	11.55
GO:00352	UDP-gluc	Molecular	0.03	0.25	Solyc01g0	Solyc01g0	2.01	1.01
GO:00352	UDP-gluc	Molecular	0.03	0.25	Solyc09g0	Solyc09g0	2.71	1.44
GO:00101	vitamin E	Biological	0.03	0.25	Solyc08g0	Solyc08g0	2.64	1.40
GO:00101	vitamin E	Biological	0.03	0.25	Solyc03g0	Solyc03g0	2.06	1.04
GO:00526	raffinose a	Molecular	0.03	0.25	Solyc06g0	Solyc06g0	3.62	1.85
GO:00526	raffinose a	Molecular	0.03	0.25	Solyc03g0	Solyc03g0	3.84	1.94
GO:00303	fructose 1,	Biological	0.03	0.25	Solyc10g0	Solyc10g0	2.23	1.16
GO:00303	fructose 1,	Biological	0.03	0.25	Solyc09g0	Solyc09g0	25.41	4.67
GO:00429	xenobiotic	Molecular	0.03	0.25	Solyc01g1	Solyc01g1	5.77	2.53
GO:00429	xenobiotic	Molecular	0.03	0.25	Solyc04g0	Solyc04g0	2.19	1.13
GO:00429	xenobiotic	Molecular	0.03	0.25	Solyc11g0	Solyc11g0	0.35	-1.51
GO:00429	xenobiotic	Molecular	0.03	0.25	Solyc03g1	Solyc03g1	0.25	-2.02
GO:00429	xenobiotic	Molecular	0.03	0.25	Solyc10g0	Solyc10g0	2.60	1.38
GO:00429	xenobiotic	Molecular	0.03	0.25	Solyc07g0	Solyc07g0	3.79	1.92
GO:00429	xenobiotic	Molecular	0.03	0.25	Solyc04g0	Solyc04g0	0.26	-1.94
GO:00429	xenobiotic	Molecular	0.03	0.25	Solyc01g0	Solyc01g0	1991.27	10.96
GO:00429	xenobiotic	Molecular	0.03	0.25	Solyc08g0	Solyc08g0	0.47	-1.08
GO:00429	xenobiotic	Molecular	0.03	0.25	Solyc07g0	Solyc07g0	0.40	-1.33
GO:00159	carbon fix	Biological	0.03	0.25	Solyc01g0	Solyc01g0	0.03	-5.12
GO:00159	carbon fix	Biological	0.03	0.25	Solyc11g0	Solyc11g0	20.23	4.34
GO:00159	carbon fix	Biological	0.03	0.25	Solyc03g0	RBCS-2A	2.71	1.44
GO:00159	carbon fix	Biological	0.03	0.25	Solyc09g0	Solyc09g0	8.04	3.01
GO:00095	photosyste	Cellular C	0.03	0.25	Solyc12g0	Solyc12g0	0.05	-4.20
GO:00095	photosyste	Cellular C	0.03	0.25	Solyc10g0	Solyc10g0	0.00	-12.09
GO:00095	photosyste	Cellular C	0.03	0.25	Solyc11g0	Solyc11g0	0.00	-13.08
GO:00095	photosyste	Cellular C	0.03	0.25	Solyc02g0	Solyc02g0	0.43	-1.21
GO:00167	hydrolase	Molecular	0.03	0.25	Solyc03g1	Solyc03g1	2.49	1.32
GO:00167	hydrolase	Molecular	0.03	0.25	Solyc04g0	Solyc04g0	0.18	-2.48
GO:00167	hydrolase	Molecular	0.03	0.25	Solyc05g0	Solyc05g0	2.32	1.21
GO:00167	hydrolase	Molecular	0.03	0.25	Solyc09g0	Solyc09g0	0.47	-1.07
GO:00167	hydrolase	Molecular	0.03	0.25	Solyc10g0	Solyc10g0	10.66	3.41
GO:00167	hydrolase	Molecular	0.03	0.25	Solyc11g0	Solyc11g0	9351.68	13.19
GO:00167	hydrolase	Molecular	0.03	0.25	Solyc01g0	Solyc01g0	3.26	1.71
GO:00167	hydrolase	Molecular	0.03	0.25	Solyc11g0	Solyc11g0	5096.34	12.32
GO:00167	hydrolase	Molecular	0.03	0.25	Solyc01g0	Solyc01g0	3.07	1.62
GO:00167	hydrolase	Molecular	0.03	0.25	Solyc02g0	Solyc02g0	0.25	-1.99
GO:00167	hydrolase	Molecular	0.03	0.25	Solyc07g0	Solyc07g0	1462.99	10.51
GO:00167	hydrolase	Molecular	0.03	0.25	Solyc03g1	Solyc03g1	4.71	2.24
GO:00167	hydrolase	Molecular	0.03	0.25	Solyc03g0	Solyc03g0	23.80	4.57
GO:00167	hydrolase	Molecular	0.03	0.25	Solyc05g0	Solyc05g0	0.50	-1.01
GO:00167	hydrolase	Molecular	0.03	0.25	Solyc04g0	Solyc04g0	9.52	3.25
GO:00717	organic sul	Biological	0.03	0.25	Solyc01g0	Solyc01g0	15.46	3.95
GO:00717	organic sul	Biological	0.03	0.25	Solyc10g0	Solyc10g0	2.91	1.54
GO:00717	organic sul	Biological	0.03	0.25	Solyc05g0	Solyc05g0	2.18	1.12
GO:00717	organic sul	Biological	0.03	0.25	Solyc11g0	Solyc11g0	4.22	2.08
GO:00717	organic sul	Biological	0.03	0.25	Solyc06g0	Solyc06g0	32.34	5.02
GO:00159	electron tr	Biological	0.03	0.25	Solyc10g0	Solyc10g0	0.00	-13.73
GO:00159	electron tr	Biological	0.03	0.25	Solyc06g0	Solyc06g0	0.05	-4.46
GO:00159	electron tr	Biological	0.03	0.25	Solyc11g0	Solyc11g0	0.00	-11.68

GO:00159	electron tra	Biological	0.03	0.25	Solyc01g0	Solyc01g0	0.08	-3.58
GO:00159	electron tra	Biological	0.03	0.25	Solyc11g0	Solyc11g0	0.09	-3.52
GO:00051	structural c	Molecular	0.03	0.25	Solyc04g0	Solyc04g0	0.05	-4.33
GO:00051	structural c	Molecular	0.03	0.25	Solyc04g0	Solyc04g0	0.18	-2.45
GO:00051	structural c	Molecular	0.03	0.25	Solyc02g0	Solyc02g0	0.25	-2.00
GO:00048	trehalose-p	Molecular	0.03	0.25	Solyc04g0	Solyc04g0	4.06	2.02
GO:00048	trehalose-p	Molecular	0.03	0.25	Solyc04g0	Solyc04g0	2.89	1.53
GO:00048	trehalose-p	Molecular	0.03	0.25	Solyc03g0	Solyc03g0	604.27	9.24
GO:00169	mannan en	Molecular	0.03	0.25	Solyc01g0	Solyc01g0	15.46	3.95
GO:00169	mannan en	Molecular	0.03	0.25	Solyc10g0	Solyc10g0	2.91	1.54
GO:00169	mannan en	Molecular	0.03	0.25	Solyc06g0	Solyc06g0	32.34	5.02
GO:00059	sucrose bio	Biological	0.03	0.25	Solyc10g0	Solyc10g0	2.23	1.16
GO:00059	sucrose bio	Biological	0.03	0.25	Solyc09g0	Solyc09g0	2.71	1.44
GO:00059	sucrose bio	Biological	0.03	0.25	Solyc09g0	Solyc09g0	25.41	4.67
GO:00104	negative re	Biological	0.03	0.25	Solyc11g0	Solyc11g0	0.07	-3.83
GO:00104	negative re	Biological	0.03	0.25	Solyc09g0	Solyc09g0	10879.94	13.41
GO:00104	negative re	Biological	0.03	0.25	Solyc03g0	Solyc03g0	11.28	3.50
GO:00304	peptidase i	Molecular	0.03	0.25	Solyc11g0	Solyc11g0	0.07	-3.83
GO:00304	peptidase i	Molecular	0.03	0.25	Solyc09g0	Solyc09g0	10879.94	13.41
GO:00304	peptidase i	Molecular	0.03	0.25	Solyc03g0	Solyc03g0	11.28	3.50
GO:00038	DNA-direc	Molecular	0.03	0.26	Solyc08g0	Solyc08g0	4.60	2.20
GO:00038	DNA-direc	Molecular	0.03	0.26	Solyc10g0	Solyc10g0	0.03	-5.19
GO:00038	DNA-direc	Molecular	0.03	0.26	Solyc08g0	Solyc08g0	0.00	-12.86
GO:00038	DNA-direc	Molecular	0.03	0.26	Solyc10g0	Solyc10g0	0.00	-13.39
GO:00038	DNA-direc	Molecular	0.03	0.26	Solyc10g0	Solyc10g0	0.00	-9.03
GO:00038	DNA-direc	Molecular	0.03	0.26	Solyc08g0	Solyc08g0	0.03	-5.23
GO:00038	DNA-direc	Molecular	0.03	0.26	Solyc12g0	Solyc12g0	0.09	-3.53
GO:00038	DNA-direc	Molecular	0.03	0.26	Solyc12g0	Solyc12g0	0.00	-12.05
GO:00038	DNA-direc	Molecular	0.03	0.26	Solyc12g0	Solyc12g0	12.70	3.67
GO:00038	DNA-direc	Molecular	0.03	0.26	Solyc02g0	Solyc02g0	0.00	-12.40
GO:00038	DNA-direc	Molecular	0.03	0.26	Solyc01g0	Solyc01g0	0.00	-11.62
GO:00060	tricarboxyl	Biological	0.03	0.26	Solyc11g0	Solyc11g0	4.12	2.04
GO:00060	tricarboxyl	Biological	0.03	0.26	Solyc11g0	Solyc11g0	20.23	4.34
GO:00060	tricarboxyl	Biological	0.03	0.26	Solyc07g0	Solyc07g0	3.93	1.98
GO:00060	tricarboxyl	Biological	0.03	0.26	Solyc09g0	Solyc09g0	8.04	3.01
GO:00060	tricarboxyl	Biological	0.03	0.26	Solyc03g1	Solyc03g1	6.29	2.65
GO:00060	tricarboxyl	Biological	0.03	0.26	Solyc04g0	Solyc04g0	39.10	5.29
GO:00060	tricarboxyl	Biological	0.03	0.26	Solyc04g0	Solyc04g0	32.87	5.04
GO:00167	transferase	Molecular	0.03	0.26	Solyc09g0	Solyc09g0	0.05	-4.31
GO:00167	transferase	Molecular	0.03	0.26	Solyc08g0	Solyc08g0	2.65	1.41
GO:00167	transferase	Molecular	0.03	0.26	Solyc01g1	Solyc01g1	4.75	2.25
GO:00167	transferase	Molecular	0.03	0.26	Solyc05g0	Solyc05g0	0.06	-4.07
GO:00167	transferase	Molecular	0.03	0.26	Solyc09g0	Solyc09g0	0.06	-4.05
GO:00167	transferase	Molecular	0.03	0.26	Solyc04g0	Solyc04g0	2.62	1.39
GO:00167	transferase	Molecular	0.03	0.26	Solyc02g0	Solyc02g0	0.21	-2.23
GO:00167	transferase	Molecular	0.03	0.26	Solyc08g0	Solyc08g0	2.64	1.40
GO:00167	transferase	Molecular	0.03	0.26	Solyc04g0	Solyc04g0	3.38	1.76
GO:00167	transferase	Molecular	0.03	0.26	Solyc01g0	Solyc01g0	8.17	3.03
GO:00167	transferase	Molecular	0.03	0.26	Solyc07g0	Solyc07g0	0.49	-1.02
GO:00167	transferase	Molecular	0.03	0.26	Solyc05g0	Solyc05g0	0.09	-3.44
GO:00167	transferase	Molecular	0.03	0.26	Solyc02g0	Solyc02g0	0.44	-1.19

GO:00167	transferase	Molecular	0.03	0.26	Solyc01g0	XTH1	5.24	2.39
GO:00167	transferase	Molecular	0.03	0.26	Solyc09g0	Solyc09g0	2.71	1.44
GO:00167	transferase	Molecular	0.03	0.26	Solyc04g0	Solyc04g0	0.00	-13.91
GO:00167	transferase	Molecular	0.03	0.26	Solyc07g0	Solyc07g0	12.95	3.70
GO:00167	transferase	Molecular	0.03	0.26	Solyc08g0	Solyc08g0	0.00	-12.86
GO:00167	transferase	Molecular	0.03	0.26	Solyc01g0	Solyc01g0	3.07	1.62
GO:00167	transferase	Molecular	0.03	0.26	Solyc10g0	Solyc10g0	0.00	-13.39
GO:00167	transferase	Molecular	0.03	0.26	Solyc01g0	Solyc01g0	2.42	1.27
GO:00167	transferase	Molecular	0.03	0.26	Solyc06g0	Solyc06g0	0.15	-2.74
GO:00167	transferase	Molecular	0.03	0.26	Solyc09g0	Solyc09g0	0.00	-11.98
GO:00167	transferase	Molecular	0.03	0.26	Solyc07g0	Solyc07g0	3182.84	11.64
GO:00167	transferase	Molecular	0.03	0.26	Solyc09g0	Solyc09g0	0.49	-1.04
GO:00167	transferase	Molecular	0.03	0.26	Solyc08g0	Solyc08g0	0.03	-5.23
GO:00167	transferase	Molecular	0.03	0.26	Solyc02g0	Solyc02g0	3.38	1.76
GO:00167	transferase	Molecular	0.03	0.26	Solyc10g0	Solyc10g0	4.22	2.08
GO:00167	transferase	Molecular	0.03	0.26	Solyc12g0	Solyc12g0	3.17	1.66
GO:00167	transferase	Molecular	0.03	0.26	Solyc11g0	Solyc11g0	0.25	-2.02
GO:00167	transferase	Molecular	0.03	0.26	Solyc08g0	Solyc08g0	0.41	-1.30
GO:00167	transferase	Molecular	0.03	0.26	Solyc02g0	Solyc02g0	307.12	8.26
GO:00167	transferase	Molecular	0.03	0.26	Solyc07g0	Solyc07g0	2.42	1.27
GO:00167	transferase	Molecular	0.03	0.26	Solyc12g0	Solyc12g0	12.70	3.67
GO:00167	transferase	Molecular	0.03	0.26	Solyc07g0	Solyc07g0	2.39	1.26
GO:00167	transferase	Molecular	0.03	0.26	Solyc12g0	Solyc12g0	0.00	-9.95
GO:00167	transferase	Molecular	0.03	0.26	Solyc11g0	Solyc11g0	0.24	-2.07
GO:00167	transferase	Molecular	0.03	0.26	Solyc07g0	Solyc07g0	383.57	8.58
GO:00167	transferase	Molecular	0.03	0.26	Solyc03g1	Solyc03g1	356.83	8.48
GO:00167	transferase	Molecular	0.03	0.26	Solyc09g0	Solyc09g0	529.32	9.05
GO:00167	transferase	Molecular	0.03	0.26	Solyc02g0	Solyc02g0	2.45	1.30
GO:00167	transferase	Molecular	0.03	0.26	Solyc07g0	Solyc07g0	0.01	-7.50
GO:00167	transferase	Molecular	0.03	0.26	Solyc06g0	Solyc06g0	0.00	-9.45
GO:00167	transferase	Molecular	0.03	0.26	Solyc06g0	Solyc06g0	12.77	3.67
GO:00167	transferase	Molecular	0.03	0.26	Solyc11g0	Solyc11g0	0.46	-1.12
GO:00167	transferase	Molecular	0.03	0.26	Solyc08g0	Solyc08g0	0.00	-9.72
GO:00090	aerobic res	Biological	0.03	0.26	Solyc10g0	Solyc10g0	0.00	-13.73
GO:00090	aerobic res	Biological	0.03	0.26	Solyc06g0	Solyc06g0	0.05	-4.46
GO:00090	aerobic res	Biological	0.03	0.26	Solyc04g0	Solyc04g0	39.10	5.29
GO:00090	aerobic res	Biological	0.03	0.26	Solyc02g0	Solyc02g0	0.19	-2.37
GO:00090	aerobic res	Biological	0.03	0.26	Solyc04g0	Solyc04g0	32.87	5.04
GO:00090	aerobic res	Biological	0.03	0.26	Solyc11g0	Solyc11g0	0.09	-3.52
GO:00719	FAD bindi	Molecular	0.03	0.28	Solyc10g0	Solyc10g0	3.13	1.64
GO:00719	FAD bindi	Molecular	0.03	0.28	Solyc09g0	Solyc09g0	0.40	-1.34
GO:00719	FAD bindi	Molecular	0.03	0.28	Solyc02g0	Solyc02g0	0.16	-2.68
GO:00719	FAD bindi	Molecular	0.03	0.28	Solyc02g0	Solyc02g0	0.45	-1.14
GO:00719	FAD bindi	Molecular	0.03	0.28	Solyc02g0	Solyc02g0	3.06	1.62
GO:00719	FAD bindi	Molecular	0.03	0.28	Solyc11g0	Solyc11g0	0.32	-1.67
GO:00719	FAD bindi	Molecular	0.03	0.28	Solyc12g0	Solyc12g0	2.29	1.20
GO:00719	FAD bindi	Molecular	0.03	0.28	Solyc02g0	Solyc02g0	6.79	2.76
GO:00719	FAD bindi	Molecular	0.03	0.28	Solyc02g0	Solyc02g0	0.37	-1.45
GO:00166	oxidoreduc	Molecular	0.03	0.28	Solyc11g0	Solyc11g0	2.05	1.04
GO:00166	oxidoreduc	Molecular	0.03	0.28	Solyc03g1	Solyc03g1	2.42	1.27
GO:00166	oxidoreduc	Molecular	0.03	0.28	Solyc01g0	Solyc01g0	0.32	-1.65

GO:00166	oxidoreduc	Molecular	0.03	0.28	Solyc10g0	Solyc10g0	2.27	1.19
GO:00166	oxidoreduc	Molecular	0.03	0.28	Solyc06g0	Solyc06g0	2.13	1.09
GO:00314	oxylipin bi	Biological	0.04	0.29	Solyc01g0	Solyc01g0	2.18	1.13
GO:00314	oxylipin bi	Biological	0.04	0.29	Solyc11g0	AOS2	3.20	1.68
GO:00314	oxylipin bi	Biological	0.04	0.29	Solyc08g0	LOX1.1	2.60	1.38
GO:00314	oxylipin bi	Biological	0.04	0.29	Solyc12g0	Solyc12g0	2.73	1.45
GO:00425	cell wall bi	Biological	0.04	0.29	Solyc12g0	Solyc12g0	24.67	4.62
GO:00425	cell wall bi	Biological	0.04	0.29	Solyc04g0	Solyc04g0	3.38	1.76
GO:00425	cell wall bi	Biological	0.04	0.29	Solyc01g0	XTH1	5.24	2.39
GO:00425	cell wall bi	Biological	0.04	0.29	Solyc09g0	Solyc09g0	0.36	-1.46
GO:00425	cell wall bi	Biological	0.04	0.29	Solyc02g0	Solyc02g0	0.35	-1.51
GO:00425	cell wall bi	Biological	0.04	0.29	Solyc03g0	Solyc03g0	0.46	-1.12
GO:00055	calcium io	Molecular	0.04	0.30	Solyc04g0	Solyc04g0	2.70	1.43
GO:00055	calcium io	Molecular	0.04	0.30	Solyc04g0	Solyc04g0	3.53	1.82
GO:00055	calcium io	Molecular	0.04	0.30	Solyc03g1	Solyc03g1	8.55	3.10
GO:00055	calcium io	Molecular	0.04	0.30	Solyc07g0	Solyc07g0	4.02	2.01
GO:00055	calcium io	Molecular	0.04	0.30	Solyc01g0	Solyc01g0	0.34	-1.54
GO:00055	calcium io	Molecular	0.04	0.30	Solyc02g0	Solyc02g0	0.37	-1.42
GO:00055	calcium io	Molecular	0.04	0.30	Solyc01g1	Solyc01g1	0.24	-2.06
GO:00055	calcium io	Molecular	0.04	0.30	Solyc05g0	Solyc05g0	4.33	2.11
GO:00055	calcium io	Molecular	0.04	0.30	Solyc12g0	Solyc12g0	0.36	-1.46
GO:00055	calcium io	Molecular	0.04	0.30	Solyc01g0	Solyc01g0	6.67	2.74
GO:00055	calcium io	Molecular	0.04	0.30	Solyc03g1	Solyc03g1	0.48	-1.07
GO:00055	calcium io	Molecular	0.04	0.30	Solyc02g0	Solyc02g0	3.41	1.77
GO:00055	calcium io	Molecular	0.04	0.30	Solyc11g0	Solyc11g0	3143.94	11.62
GO:00055	calcium io	Molecular	0.04	0.30	Solyc01g0	Solyc01g0	3.53	1.82
GO:00055	calcium io	Molecular	0.04	0.30	Solyc11g0	Solyc11g0	2.24	1.16
GO:00055	calcium io	Molecular	0.04	0.30	Solyc02g0	Solyc02g0	0.40	-1.31
GO:00055	calcium io	Molecular	0.04	0.30	Solyc06g0	Solyc06g0	0.37	-1.44
GO:00055	calcium io	Molecular	0.04	0.30	Solyc04g0	Solyc04g0	1564.01	10.61
GO:00055	calcium io	Molecular	0.04	0.30	Solyc01g0	Solyc01g0	5.28	2.40
GO:00055	calcium io	Molecular	0.04	0.30	Solyc03g0	Solyc03g0	2638.78	11.37
GO:00055	calcium io	Molecular	0.04	0.30	Solyc10g0	Solyc10g0	0.25	-1.99
GO:00055	calcium io	Molecular	0.04	0.30	Solyc04g0	Solyc04g0	5.19	2.37
GO:00055	calcium io	Molecular	0.04	0.30	Solyc01g0	Solyc01g0	1566.92	10.61
GO:00055	calcium io	Molecular	0.04	0.30	Solyc01g0	Solyc01g0	0.00	-9.77
GO:00055	calcium io	Molecular	0.04	0.30	Solyc08g0	Solyc08g0	0.09	-3.48
GO:00055	calcium io	Molecular	0.04	0.30	Solyc03g1	Solyc03g1	2.21	1.14
GO:00801	response to	Biological	0.04	0.30	Solyc11g0	Solyc11g0	0.28	-1.84
GO:00801	response to	Biological	0.04	0.30	Solyc12g0	Solyc12g0	2.10	1.07
GO:00801	response to	Biological	0.04	0.30	Solyc02g0	Solyc02g0	3.42	1.77
GO:00199	sexual repr	Biological	0.04	0.30	Solyc06g0	Solyc06g0	12.59	3.65
GO:00199	sexual repr	Biological	0.04	0.30	Solyc01g1	Solyc01g1	2.90	1.54
GO:00199	sexual repr	Biological	0.04	0.30	Solyc08g0	Solyc08g0	11.44	3.52
GO:00125	programme	Biological	0.04	0.30	Solyc09g0	Solyc09g0	0.41	-1.27
GO:00125	programme	Biological	0.04	0.30	Solyc09g0	Solyc09g0	0.48	-1.06
GO:00125	programme	Biological	0.04	0.30	Solyc01g0	Solyc01g0	0.43	-1.23
GO:00425	photosyste	Biological	0.04	0.30	Solyc07g0	Solyc07g0	3.28	1.71
GO:00425	photosyste	Biological	0.04	0.30	Solyc02g0	PSBO	5.02	2.33
GO:00425	photosyste	Biological	0.04	0.30	Solyc01g1	psbZ	0.17	-2.52
GO:00609	auxin trans	Biological	0.04	0.30	Solyc03g1	Solyc03g1	0.33	-1.59

GO:00609	auxin trans	Biological	0.04	0.30	Solyc01g0	Solyc01g0	6.50	2.70
GO:00609	auxin trans	Biological	0.04	0.30	Solyc10g0	Solyc10g0	0.37	-1.43
GO:00099	chloroplas	Biological	0.04	0.30	Solyc06g0	Solyc06g0	3.04	1.60
GO:00099	chloroplas	Biological	0.04	0.30	Solyc02g0	Solyc02g0	2.66	1.41
GO:00099	chloroplas	Biological	0.04	0.30	Solyc12g0	Solyc12g0	467.31	8.87
GO:00325	developme	Biological	0.04	0.30	Solyc08g0	Solyc08g0	3.77	1.91
GO:00325	developme	Biological	0.04	0.30	Solyc11g0	Solyc11g0	18.46	4.21
GO:00325	developme	Biological	0.04	0.30	Solyc03g0	Solyc03g0	2300.48	11.17
GO:00325	developme	Biological	0.04	0.30	Solyc08g0	Solyc08g0	2486.58	11.28
GO:00325	developme	Biological	0.04	0.30	Solyc01g0	Solyc01g0	1296.75	10.34
GO:00325	developme	Biological	0.04	0.30	Solyc08g0	Solyc08g0	2.07	1.05
GO:00325	developme	Biological	0.04	0.30	Solyc01g1	Solyc01g1	0.37	-1.43
GO:00097	chloroplas	Cellular C	0.04	0.30	Solyc11g0	AOS2	3.20	1.68
GO:00097	chloroplas	Cellular C	0.04	0.30	Solyc11g0	Solyc11g0	0.02	-5.38
GO:00097	chloroplas	Cellular C	0.04	0.30	Solyc11g0	Solyc11g0	0.09	-3.49
GO:00097	chloroplas	Cellular C	0.04	0.30	Solyc11g0	Solyc11g0	0.03	-5.20
GO:00097	chloroplas	Cellular C	0.04	0.30	Solyc10g0	Solyc10g0	0.06	-4.16
GO:00068	potassium	Biological	0.04	0.31	Solyc01g1	Solyc01g1	0.29	-1.80
GO:00068	potassium	Biological	0.04	0.31	Solyc03g0	Solyc03g0	0.31	-1.67
GO:00068	potassium	Biological	0.04	0.31	Solyc05g0	Solyc05g0	0.49	-1.03
GO:00068	potassium	Biological	0.04	0.31	Solyc05g0	Solyc05g0	8.29	3.05
GO:00068	potassium	Biological	0.04	0.31	Solyc01g0	Solyc01g0	894.88	9.81
GO:00068	potassium	Biological	0.04	0.31	Solyc12g0	Solyc12g0	369.30	8.53
GO:00066	fatty acid	Biological	0.04	0.31	Solyc01g0	Solyc01g0	2.18	1.13
GO:00066	fatty acid	Biological	0.04	0.31	Solyc11g0	Solyc11g0	3.25	1.70
GO:00066	fatty acid	Biological	0.04	0.31	Solyc05g0	Solyc05g0	2.80	1.49
GO:00066	fatty acid	Biological	0.04	0.31	Solyc04g0	Solyc04g0	0.35	-1.50
GO:00066	fatty acid	Biological	0.04	0.31	Solyc11g0	AOS2	3.20	1.68
GO:00066	fatty acid	Biological	0.04	0.31	Solyc08g0	LOX1.1	2.60	1.38
GO:00066	fatty acid	Biological	0.04	0.31	Solyc03g1	Solyc03g1	4.55	2.18
GO:00066	fatty acid	Biological	0.04	0.31	Solyc04g0	Solyc04g0	0.00	-13.91
GO:00066	fatty acid	Biological	0.04	0.31	Solyc12g0	Solyc12g0	2.35	1.23
GO:00066	fatty acid	Biological	0.04	0.31	Solyc12g0	Solyc12g0	2.73	1.45
GO:00066	fatty acid	Biological	0.04	0.31	Solyc11g0	Solyc11g0	292.92	8.19
GO:00097	detection c	Biological	0.04	0.31	Solyc07g0	ACO1	2.82	1.49
GO:00097	detection c	Biological	0.04	0.31	Solyc02g0	Solyc02g0	2.56	1.36
GO:00059	starch met	Biological	0.04	0.31	Solyc06g0	Solyc06g0	2.52	1.33
GO:00059	starch met	Biological	0.04	0.31	Solyc08g0	Solyc08g0	0.36	-1.48
GO:00098	blue light	Molecular	0.04	0.31	Solyc09g0	Solyc09g0	0.40	-1.34
GO:00098	blue light	Molecular	0.04	0.31	Solyc12g0	Solyc12g0	2.29	1.20
GO:00043	glutamate	Molecular	0.04	0.31	Solyc11g0	Solyc11g0	0.50	-1.01
GO:00043	glutamate	Molecular	0.04	0.31	Solyc04g0	Solyc04g0	1711.14	10.74
GO:00065	glutamate	Biological	0.04	0.31	Solyc11g0	Solyc11g0	0.50	-1.01
GO:00065	glutamate	Biological	0.04	0.31	Solyc04g0	Solyc04g0	1711.14	10.74
GO:00065	glutamate	Biological	0.04	0.31	Solyc11g0	Solyc11g0	0.50	-1.01
GO:00065	glutamate	Biological	0.04	0.31	Solyc04g0	Solyc04g0	1711.14	10.74
GO:00325	plastid tra	Biological	0.04	0.31	Solyc05g0	Solyc05g0	2.32	1.21
GO:00325	plastid tra	Biological	0.04	0.31	Solyc05g0	Solyc05g0	10.06	3.33
GO:19023	sulfate tra	Biological	0.04	0.31	Solyc12g0	Solyc12g0	0.37	-1.44
GO:19023	sulfate tra	Biological	0.04	0.31	Solyc04g0	Solyc04g0	0.41	-1.29
GO:00070	microtubul	Biological	0.04	0.31	Solyc09g0	Solyc09g0	0.05	-4.40

GO:00070	microtubul	Biological	0.04	0.31	Solyc02g0	Solyc02g0	11.83	3.56
GO:00070	microtubul	Biological	0.04	0.31	Solyc09g0	Solyc09g0	0.07	-3.89
GO:00070	microtubul	Biological	0.04	0.31	Solyc06g0	Solyc06g0	0.34	-1.55
GO:00070	microtubul	Biological	0.04	0.31	Solyc06g0	Solyc06g0	0.34	-1.57
GO:00070	microtubul	Biological	0.04	0.31	Solyc12g0	Solyc12g0	467.31	8.87
GO:00070	microtubul	Biological	0.04	0.31	Solyc09g0	Solyc09g0	0.35	-1.53
GO:00070	microtubul	Biological	0.04	0.31	Solyc06g0	Solyc06g0	0.07	-3.77
GO:00070	microtubul	Biological	0.04	0.31	Solyc07g0	Solyc07g0	0.11	-3.12
GO:00056	extracellul	Cellular Co	0.05	0.31	Solyc03g1	Solyc03g1	2.16	1.11
GO:00056	extracellul	Cellular Co	0.05	0.31	Solyc04g0	Solyc04g0	2.20	1.14
GO:00056	extracellul	Cellular Co	0.05	0.31	Solyc02g0	Solyc02g0	0.33	-1.59
GO:00056	extracellul	Cellular Co	0.05	0.31	Solyc04g0	Solyc04g0	0.28	-1.84
GO:00056	extracellul	Cellular Co	0.05	0.31	Solyc02g0	Solyc02g0	2.07	1.05
GO:00056	extracellul	Cellular Co	0.05	0.31	Solyc07g0	Solyc07g0	2.40	1.26
GO:00056	extracellul	Cellular Co	0.05	0.31	Solyc05g0	Solyc05g0	20.96	4.39
GO:00037	microtubul	Molecular	0.05	0.31	Solyc09g0	Solyc09g0	0.05	-4.40
GO:00037	microtubul	Molecular	0.05	0.31	Solyc02g0	Solyc02g0	11.83	3.56
GO:00037	microtubul	Molecular	0.05	0.31	Solyc09g0	Solyc09g0	0.07	-3.89
GO:00037	microtubul	Molecular	0.05	0.31	Solyc06g0	Solyc06g0	0.34	-1.55
GO:00037	microtubul	Molecular	0.05	0.31	Solyc06g0	Solyc06g0	0.34	-1.57
GO:00037	microtubul	Molecular	0.05	0.31	Solyc12g0	Solyc12g0	467.31	8.87
GO:00037	microtubul	Molecular	0.05	0.31	Solyc09g0	Solyc09g0	0.35	-1.53
GO:00037	microtubul	Molecular	0.05	0.31	Solyc06g0	Solyc06g0	0.07	-3.77
GO:00037	microtubul	Molecular	0.05	0.31	Solyc07g0	Solyc07g0	0.11	-3.12
GO:00099	auxin pola	Biological	0.05	0.31	Solyc03g1	Solyc03g1	0.33	-1.59
GO:00099	auxin pola	Biological	0.05	0.31	Solyc10g0	Solyc10g0	0.37	-1.43
GO:00099	auxin pola	Biological	0.05	0.31	Solyc08g0	Solyc08g0	0.34	-1.54
GO:00169	cell wall m	Biological	0.05	0.31	Solyc10g0	CHI9	0.28	-1.84
GO:00169	cell wall m	Biological	0.05	0.31	Solyc02g0	Solyc02g0	0.33	-1.59
GO:00169	cell wall m	Biological	0.05	0.31	Solyc06g0	Solyc06g0	13.74	3.78
GO:00169	cell wall m	Biological	0.05	0.31	Solyc10g0	Solyc10g0	0.00	-9.02
GO:00151	amino acid	Molecular	0.05	0.31	Solyc03g0	Solyc03g0	0.24	-2.04
GO:00151	amino acid	Molecular	0.05	0.31	Solyc01g0	Solyc01g0	5.13	2.36
GO:00151	amino acid	Molecular	0.05	0.31	Solyc10g0	Solyc10g0	1973.43	10.95
GO:00151	amino acid	Molecular	0.05	0.31	Solyc03g0	Solyc03g0	0.31	-1.69
GO:00151	amino acid	Molecular	0.05	0.31	Solyc01g1	Solyc01g1	0.20	-2.35
GO:00151	amino acid	Molecular	0.05	0.31	Solyc06g0	Solyc06g0	0.15	-2.76
GO:00427	ATP synth	Biological	0.05	0.31	Solyc09g0	Solyc09g0	0.18	-2.50
GO:00427	ATP synth	Biological	0.05	0.31	Solyc10g0	Solyc10g0	0.00	-13.73
GO:00427	ATP synth	Biological	0.05	0.31	Solyc11g0	Solyc11g0	0.00	-11.68
GO:00427	ATP synth	Biological	0.05	0.31	Solyc01g0	Solyc01g0	0.08	-3.58
GO:00427	ATP synth	Biological	0.05	0.31	Solyc03g0	Solyc03g0	0.13	-3.00
GO:00427	ATP synth	Biological	0.05	0.31	Solyc11g0	Solyc11g0	0.00	-10.02
GO:00090	electron tra	Molecular	0.05	0.31	Solyc04g0	PETE	2.69	1.43
GO:00090	electron tra	Molecular	0.05	0.31	Solyc11g0	Solyc11g0	2.92	1.55
GO:00090	electron tra	Molecular	0.05	0.31	Solyc01g0	Solyc01g0	2.45	1.29
GO:00090	electron tra	Molecular	0.05	0.31	Solyc05g0	Solyc05g0	3.22	1.69
GO:00090	electron tra	Molecular	0.05	0.31	Solyc05g0	Solyc05g0	0.08	-3.58
GO:00090	electron tra	Molecular	0.05	0.31	Solyc11g0	Solyc11g0	0.32	-1.67
GO:00090	electron tra	Molecular	0.05	0.31	Solyc11g0	Solyc11g0	2.20	1.14
GO:00090	electron tra	Molecular	0.05	0.31	Solyc12g0	Solyc12g0	0.00	-11.26

GO:00090	electron tra	Molecular	0.05	0.31	Solyc09g0	Solyc09g0	0.00	-13.14
GO:00090	electron tra	Molecular	0.05	0.31	Solyc01g0	Solyc01g0	0.02	-5.72
GO:00090	electron tra	Molecular	0.05	0.31	Solyc03g1	Solyc03g1	0.10	-3.25
GO:00090	electron tra	Molecular	0.05	0.31	Solyc04g0	Solyc04g0	39.10	5.29
GO:00090	electron tra	Molecular	0.05	0.31	Solyc07g0	Solyc07g0	0.44	-1.19
GO:00090	electron tra	Molecular	0.05	0.31	Solyc04g0	Solyc04g0	32.87	5.04
GO:00090	electron tra	Molecular	0.05	0.31	Solyc01g1	Solyc01g1	0.08	-3.66
GO:00090	electron tra	Molecular	0.05	0.31	Solyc11g0	Solyc11g0	0.28	-1.82
GO:00090	electron tra	Molecular	0.05	0.31	Solyc04g0	Solyc04g0	883.93	9.79
GO:00096	plant-type	Biological	0.06	0.31	Solyc04g0	Solyc04g0	0.05	-4.33
GO:00096	plant-type	Biological	0.06	0.31	Solyc02g0	Solyc02g0	2.85	1.51
GO:00096	plant-type	Biological	0.06	0.31	Solyc04g0	Solyc04g0	0.18	-2.45
GO:00096	plant-type	Biological	0.06	0.31	Solyc06g0	Solyc06g0	0.11	-3.25
GO:00096	plant-type	Biological	0.06	0.31	Solyc03g1	Solyc03g1	0.15	-2.75
GO:00096	plant-type	Biological	0.06	0.31	Solyc08g0	Solyc08g0	4.19	2.07
GO:00059	trehalose b	Biological	0.06	0.31	Solyc04g0	Solyc04g0	4.06	2.02
GO:00059	trehalose b	Biological	0.06	0.31	Solyc04g0	Solyc04g0	2.89	1.53
GO:00059	trehalose b	Biological	0.06	0.31	Solyc02g0	Solyc02g0	3056.62	11.58
GO:00059	trehalose b	Biological	0.06	0.31	Solyc03g0	Solyc03g0	604.27	9.24
GO:00198	extrinsic co	Cellular Co	0.06	0.31	Solyc07g0	Solyc07g0	4.02	2.01
GO:00198	extrinsic co	Cellular Co	0.06	0.31	Solyc02g0	Solyc02g0	3.41	1.77
GO:00198	extrinsic co	Cellular Co	0.06	0.31	Solyc04g0	Solyc04g0	5.19	2.37
GO:00198	extrinsic co	Cellular Co	0.06	0.31	Solyc03g1	Solyc03g1	2.21	1.14
GO:00062	pyrimidine	Biological	0.06	0.31	Solyc02g0	Solyc02g0	3.08	1.62
GO:00062	pyrimidine	Biological	0.06	0.31	Solyc02g0	Solyc02g0	0.42	-1.25
GO:00062	pyrimidine	Biological	0.06	0.31	Solyc02g0	Solyc02g0	0.36	-1.49
GO:00065	glutamine	Biological	0.06	0.31	Solyc02g0	Solyc02g0	3.08	1.62
GO:00065	glutamine	Biological	0.06	0.31	Solyc02g0	Solyc02g0	19.98	4.32
GO:00065	glutamine	Biological	0.06	0.31	Solyc03g0	Solyc03g0	1566.99	10.61
GO:00098	lignin bios	Biological	0.06	0.31	Solyc03g1	Solyc03g1	2.44	1.28
GO:00098	lignin bios	Biological	0.06	0.31	Solyc01g0	Solyc01g0	3528.16	11.78
GO:00098	lignin bios	Biological	0.06	0.31	Solyc10g0	Solyc10g0	0.00	-9.79
GO:00102	auxin hom	Biological	0.06	0.31	Solyc03g1	Solyc03g1	2.44	1.28
GO:00102	auxin hom	Biological	0.06	0.31	Solyc03g1	Solyc03g1	0.33	-1.59
GO:00102	auxin hom	Biological	0.06	0.31	Solyc10g0	Solyc10g0	0.37	-1.43
GO:00151	nitrate tran	Molecular	0.06	0.31	Solyc10g0	Solyc10g0	2.02	1.01
GO:00151	nitrate tran	Molecular	0.06	0.31	Solyc02g0	Solyc02g0	7.78	2.96
GO:00151	nitrate tran	Molecular	0.06	0.31	Solyc06g0	Solyc06g0	0.32	-1.62
GO:00181	peptidyl-se	Biological	0.06	0.31	Solyc09g0	Solyc09g0	0.32	-1.64
GO:00181	peptidyl-se	Biological	0.06	0.31	Solyc01g0	Solyc01g0	0.34	-1.54
GO:00181	peptidyl-se	Biological	0.06	0.31	Solyc11g0	Solyc11g0	2.57	1.36
GO:00181	peptidyl-se	Biological	0.06	0.31	Solyc12g0	Solyc12g0	2.20	1.14
GO:00181	peptidyl-se	Biological	0.06	0.31	Solyc11g0	Solyc11g0	2.24	1.16
GO:00181	peptidyl-se	Biological	0.06	0.31	Solyc01g0	Solyc01g0	5.28	2.40
GO:00181	peptidyl-se	Biological	0.06	0.31	Solyc12g0	Solyc12g0	3.71	1.89
GO:00181	peptidyl-se	Biological	0.06	0.31	Solyc01g0	Solyc01g0	0.00	-9.77
GO:00098	1-aminocy	Molecular	0.06	0.31	Solyc07g0	ACO1	2.82	1.49
GO:00098	1-aminocy	Molecular	0.06	0.31	Solyc02g0	Solyc02g0	2.56	1.36
GO:00713	cellular res	Biological	0.06	0.31	Solyc07g0	ACO1	2.82	1.49
GO:00713	cellular res	Biological	0.06	0.31	Solyc02g0	Solyc02g0	2.56	1.36
GO:00166	oxidoreduc	Molecular	0.06	0.31	Solyc03g0	Solyc03g0	7.65	2.93

GO:00166	oxidoreduc	Molecular	0.06	0.31	Solyc03g0	Solyc03g0	0.44	-1.19
GO:00041	cysteine sy	Molecular	0.06	0.31	Solyc01g0	Solyc01g0	2.46	1.30
GO:00041	cysteine sy	Molecular	0.06	0.31	Solyc01g0	Solyc01g0	2.88	1.53
GO:00166	hydroxypy	Molecular	0.06	0.31	Solyc01g1	Solyc01g1	4.98	2.32
GO:00166	hydroxypy	Molecular	0.06	0.31	Solyc12g0	Solyc12g0	0.45	-1.15
GO:00302	glyoxylate	Molecular	0.06	0.31	Solyc01g1	Solyc01g1	4.98	2.32
GO:00302	glyoxylate	Molecular	0.06	0.31	Solyc12g0	Solyc12g0	0.45	-1.15
GO:00423	phylloquin	Biological	0.06	0.31	Solyc12g0	Solyc12g0	2.20	1.14
GO:00423	phylloquin	Biological	0.06	0.31	Solyc03g0	Solyc03g0	0.00	-11.43
GO:00040	branched-c	Molecular	0.06	0.31	Solyc03g0	Solyc03g0	4.53	2.18
GO:00040	branched-c	Molecular	0.06	0.31	Solyc04g0	Solyc04g0	1185.03	10.21
GO:00090	branched-c	Biological	0.06	0.31	Solyc03g0	Solyc03g0	4.53	2.18
GO:00090	branched-c	Biological	0.06	0.31	Solyc04g0	Solyc04g0	1185.03	10.21
GO:00069	immune re	Biological	0.06	0.31	Solyc09g0	Solyc09g0	0.48	-1.06
GO:00069	immune re	Biological	0.06	0.31	Solyc01g0	Solyc01g0	0.43	-1.23
GO:00454	gibberellin	Biological	0.06	0.31	Solyc07g0	Solyc07g0	2.20	1.14
GO:00454	gibberellin	Biological	0.06	0.31	Solyc07g0	Solyc07g0	29.42	4.88
GO:00526	C-19 gibbe	Molecular	0.06	0.31	Solyc07g0	Solyc07g0	2.20	1.14
GO:00526	C-19 gibbe	Molecular	0.06	0.31	Solyc07g0	Solyc07g0	29.42	4.88
GO:00421	fructose 1,	Molecular	0.06	0.31	Solyc10g0	Solyc10g0	2.23	1.16
GO:00421	fructose 1,	Molecular	0.06	0.31	Solyc09g0	Solyc09g0	25.41	4.67
GO:00168	carbon-sul	Molecular	0.06	0.31	Solyc02g0	Solyc02g0	2381.72	11.22
GO:00168	carbon-sul	Molecular	0.06	0.31	Solyc11g0	Solyc11g0	0.48	-1.07
GO:00060	chitin catal	Biological	0.07	0.31	Solyc10g0	CHI9	0.28	-1.84
GO:00060	chitin catal	Biological	0.07	0.31	Solyc02g0	Solyc02g0	0.33	-1.59
GO:00060	chitin catal	Biological	0.07	0.31	Solyc06g0	Solyc06g0	13.74	3.78
GO:00060	chitin catal	Biological	0.07	0.31	Solyc10g0	Solyc10g0	0.00	-9.02
GO:00004	DNA-direc	Cellular Co	0.07	0.31	Solyc08g0	Solyc08g0	0.00	-12.86
GO:00004	DNA-direc	Cellular Co	0.07	0.31	Solyc10g0	Solyc10g0	0.00	-13.39
GO:00004	DNA-direc	Cellular Co	0.07	0.31	Solyc08g0	Solyc08g0	0.03	-5.23
GO:00004	DNA-direc	Cellular Co	0.07	0.31	Solyc12g0	Solyc12g0	12.70	3.67
GO:00472	indole-3-ac	Molecular	0.07	0.31	Solyc09g0	Solyc09g0	0.05	-4.31
GO:00801	negative re	Biological	0.07	0.31	Solyc09g0	Solyc09g0	0.05	-4.31
GO:00160	glutamate s	Molecular	0.07	0.31	Solyc03g0	Solyc03g0	7.65	2.93
GO:00060	glucose ca	Biological	0.07	0.31	Solyc07g0	Solyc07g0	0.35	-1.51
GO:00101	pollen tube	Biological	0.07	0.31	Solyc01g1	Solyc01g1	5.46	2.45
GO:00360	mediator c	Molecular	0.07	0.31	Solyc01g1	Solyc01g1	5.46	2.45
GO:00454	naringenin	Molecular	0.07	0.31	Solyc02g0	Solyc02g0	0.08	-3.73
GO:00430	starch grai	Cellular Co	0.07	0.31	Solyc06g0	Solyc06g0	2.52	1.33
GO:00976	potassium	Biological	0.07	0.31	Solyc04g0	Solyc04g0	2.70	1.43
GO:01101	phloem suc	Biological	0.07	0.31	Solyc04g0	Solyc04g0	2.70	1.43
GO:00043	formate-tet	Molecular	0.07	0.31	Solyc01g0	Solyc01g0	2.62	1.39
GO:00092	10-formylt	Biological	0.07	0.31	Solyc01g0	Solyc01g0	2.62	1.39
GO:00043	ferredoxin-	Molecular	0.07	0.31	Solyc02g0	Solyc02g0	3.57	1.83
GO:00454	flavonol sy	Molecular	0.07	0.31	Solyc11g0	Solyc11g0	0.28	-1.84
GO:00302	glycosamii	Biological	0.07	0.31	Solyc01g0	Solyc01g0	2.01	1.01
GO:00471	shikimate (	Molecular	0.07	0.31	Solyc03g1	Solyc03g1	2.44	1.28
GO:00422	glutamate-	Molecular	0.07	0.31	Solyc04g0	Solyc04g0	3.78	1.92
GO:00067	NADH me	Biological	0.07	0.31	Solyc11g0	Solyc11g0	4.12	2.04
GO:00503	tocopherol	Molecular	0.07	0.31	Solyc08g0	Solyc08g0	2.64	1.40
GO:00340	maintenan	Biological	0.07	0.31	Solyc01g0	Solyc01g0	2.56	1.36

GO:00337	L-methion	Molecular	0.07	0.31	Solyc03g1	E4	2.38	1.25
GO:00065	protein mo	Biological	0.07	0.31	Solyc05g0	Solyc05g0	0.48	-1.05
GO:00192	phosphatas	Molecular	0.07	0.31	Solyc01g0	Solyc01g0	0.50	-1.00
GO:00526	2-phytyl-1,	Molecular	0.07	0.31	Solyc12g0	Solyc12g0	2.20	1.14
GO:00000	alpha-1,6-r	Molecular	0.07	0.31	Solyc01g0	Solyc01g0	0.35	-1.51
GO:00045	obsolete d	Molecular	0.07	0.31	Solyc01g0	Solyc01g0	0.35	-1.51
GO:00315	mannosylt	Cellular C	0.07	0.31	Solyc01g0	Solyc01g0	0.35	-1.51
GO:00039	acylphosph	Molecular	0.07	0.31	Solyc09g0	Solyc09g0	2.13	1.09
GO:00041	dihydroorc	Molecular	0.07	0.31	Solyc01g0	Solyc01g0	0.32	-1.65
GO:00086	4-amino-4-	Molecular	0.07	0.31	Solyc07g0	Solyc07g0	6.21	2.63
GO:00107	positive re	Biological	0.07	0.31	Solyc05g0	Solyc05g0	4.51	2.17
GO:00054	UDP-xylos	Molecular	0.07	0.31	Solyc12g0	Solyc12g0	5.32	2.41
GO:00157	UDP-xylos	Biological	0.07	0.31	Solyc12g0	Solyc12g0	5.32	2.41
GO:00159	nucleobase	Biological	0.07	0.31	Solyc12g0	Solyc12g0	5.32	2.41
GO:19012	carbohydra	Biological	0.07	0.31	Solyc12g0	Solyc12g0	5.32	2.41
GO:00526	zeaxanthin	Molecular	0.07	0.31	Solyc02g0	Solyc02g0	0.45	-1.14
GO:00434	alkane cata	Biological	0.07	0.31	Solyc01g0	Solyc01g0	2.45	1.29
GO:00100	regulation	Biological	0.07	0.31	Solyc04g0	Solyc04g0	0.41	-1.29
GO:00333	chlorophyl	Biological	0.07	0.31	Solyc09g0	Solyc09g0	0.36	-1.46
GO:00904	7-hydroxy	Molecular	0.07	0.31	Solyc09g0	Solyc09g0	0.36	-1.46
GO:00002	tRNA-intr	Molecular	0.07	0.31	Solyc06g0	Solyc06g0	0.40	-1.33
GO:00094	plastoquin	Molecular	0.07	0.31	Solyc12g0	Solyc12g0	2.10	1.07
GO:00985	stromal sid	Cellular C	0.07	0.31	Solyc02g0	Solyc02g0	2.62	1.39
GO:00313	N-terminal	Biological	0.07	0.31	Solyc07g0	PDF1A	4.20	2.07
GO:00436	co-translat	Biological	0.07	0.31	Solyc07g0	PDF1A	4.20	2.07
GO:00067	porphyrin-	Biological	0.07	0.31	Solyc09g0	Solyc09g0	3.41	1.77
GO:00513	meiotic ch	Biological	0.07	0.31	Solyc01g1	Solyc01g1	2.18	1.13
GO:00723	clathrin co	Biological	0.07	0.31	Solyc04g0	Solyc04g0	2.07	1.05
GO:19906	response to	Biological	0.07	0.31	Solyc02g0	Solyc02g0	2.52	1.33
GO:00975	stress resp	Biological	0.07	0.31	Solyc01g0	Solyc01g0	0.42	-1.26
GO:00098	auxin bios	Biological	0.07	0.31	Solyc06g0	Solyc06g0	3.53	1.82
GO:00041	dihydroorc	Molecular	0.07	0.31	Solyc02g0	Solyc02g0	2.28	1.19
GO:00168	hydrolase a	Molecular	0.07	0.31	Solyc02g0	Solyc02g0	2.28	1.19
GO:00198	pyrimidine	Biological	0.07	0.31	Solyc02g0	Solyc02g0	2.28	1.19
GO:00320	negative re	Biological	0.07	0.31	Solyc10g0	Solyc10g0	15621.48	13.93
GO:00045	alpha-gala	Molecular	0.07	0.31	Solyc03g0	Solyc03g0	3.84	1.94
GO:00159	galactosida	Molecular	0.07	0.31	Solyc03g0	Solyc03g0	3.84	1.94
GO:00447	mitotic DN	Biological	0.07	0.31	Solyc01g0	Solyc01g0	0.39	-1.35
GO:00323	MutLalpha	Cellular C	0.07	0.31	Solyc01g0	Solyc01g0	0.49	-1.04
GO:00102	NADH deh	Biological	0.07	0.31	Solyc05g0	Solyc05g0	6.90	2.79
GO:00095	plasmodes	Cellular C	0.07	0.31	Solyc03g0	Solyc03g0	0.49	-1.02
GO:00102	phytol kin	Molecular	0.07	0.31	Solyc03g0	Solyc03g0	2.06	1.04
GO:00330	regulation	Biological	0.07	0.31	Solyc11g0	Solyc11g0	6976.37	12.77
GO:00430	maintenan	Biological	0.07	0.31	Solyc11g0	Solyc11g0	6976.37	12.77
GO:20000	negative re	Biological	0.07	0.31	Solyc11g0	Solyc11g0	6976.37	12.77
GO:00007	purine-spe	Molecular	0.07	0.31	Solyc09g0	Solyc09g0	2.14	1.10
GO:00354	adenine/gu	Molecular	0.07	0.31	Solyc09g0	Solyc09g0	2.14	1.10
GO:00436	P-type div	Molecular	0.07	0.31	Solyc08g0	Solyc08g0	2.67	1.41
GO:00065	leucine me	Biological	0.07	0.31	Solyc02g0	Solyc02g0	2.01	1.01
GO:00084	3-hydroxy	Molecular	0.07	0.31	Solyc02g0	Solyc02g0	2.01	1.01
GO:00003	spliceosom	Biological	0.07	0.31	Solyc06g0	Solyc06g0	0.46	-1.11

GO:00062	dGTP cata	Biological	0.07	0.31	Solyc03g0	Solyc03g0	0.02	-5.67
GO:00355	8-oxo-7,8-	Molecular	0.07	0.31	Solyc03g0	Solyc03g0	0.02	-5.67
GO:00428	monopolar	Biological	0.07	0.31	Solyc02g0	Solyc02g0	0.48	-1.05
GO:00484	petal devel	Biological	0.07	0.31	Solyc02g0	Solyc02g0	0.48	-1.05
GO:00099	chloroplast	Biological	0.07	0.31	Solyc10g0	Solyc10g0	2.33	1.22
GO:00428	central vac	Cellular C	0.07	0.31	Solyc08g0	Solyc08g0	0.29	-1.80
GO:00089	phosphorib	Molecular	0.07	0.31	Solyc08g0	Solyc08g0	2.43	1.28
GO:00007	phosphatid	Molecular	0.07	0.31	Solyc06g0	Solyc06g0	2.08	1.06
GO:00046	phosphatid	Molecular	0.07	0.31	Solyc06g0	Solyc06g0	2.08	1.06
GO:00801	phosphatid	Molecular	0.07	0.31	Solyc06g0	Solyc06g0	2.08	1.06
GO:00089	1,4-dihydr	Molecular	0.07	0.31	Solyc05g0	Solyc05g0	2.69	1.43
GO:00423	beta-amyri	Molecular	0.07	0.31	Solyc12g0	Solyc12g0	2.17	1.12
GO:00097	photosynth	Biological	0.07	0.31	Solyc07g0	Solyc07g0	2.47	1.30
GO:00425	photosyste	Biological	0.07	0.31	Solyc07g0	Solyc07g0	2.47	1.30
GO:00427	plasma me	Biological	0.07	0.31	Solyc10g0	Solyc10g0	0.03	-5.05
GO:00052	inward rec	Molecular	0.07	0.31	Solyc12g0	Solyc12g0	369.30	8.53
GO:00972	mitochond	Biological	0.07	0.31	Solyc06g0	Solyc06g0	2.48	1.31
GO:00101	stomatal c	Biological	0.07	0.31	Solyc08g0	Solyc08g0	4.19	2.07
GO:00101	transpiratio	Biological	0.07	0.31	Solyc08g0	Solyc08g0	4.19	2.07
GO:00301	regulation	Biological	0.07	0.31	Solyc08g0	Solyc08g0	4.19	2.07
GO:00482	inflorescen	Biological	0.07	0.31	Solyc08g0	Solyc08g0	4.19	2.07
GO:19054	regulation	Biological	0.07	0.31	Solyc08g0	Solyc08g0	4.19	2.07
GO:00002	C-5 sterol	Molecular	0.07	0.31	Solyc02g0	Solyc02g0	8.45	3.08
GO:01020	2-cis,6-cis-	Molecular	0.07	0.31	Solyc06g0	Solyc06g0	12.77	3.67
GO:00421	thiamine p	Molecular	0.07	0.31	Solyc07g0	Solyc07g0	7.27	2.86
GO:00427	hydrogen p	Biological	0.08	0.33	Solyc10g0	Solyc10g0	4.23	2.08
GO:00427	hydrogen p	Biological	0.08	0.33	Solyc01g0	Solyc01g0	14.73	3.88
GO:00427	hydrogen p	Biological	0.08	0.33	Solyc02g0	Solyc02g0	8.87	3.15
GO:00427	hydrogen p	Biological	0.08	0.33	Solyc02g0	Solyc02g0	2.54	1.34
GO:00427	hydrogen p	Biological	0.08	0.33	Solyc03g0	Solyc03g0	5511.05	12.43
GO:00427	hydrogen p	Biological	0.08	0.33	Solyc10g0	Solyc10g0	1604.71	10.65
GO:00427	hydrogen p	Biological	0.08	0.33	Solyc01g1	Solyc01g1	4.37	2.13
GO:00427	hydrogen p	Biological	0.08	0.33	Solyc02g0	Solyc02g0	0.17	-2.56
GO:00427	hydrogen p	Biological	0.08	0.33	Solyc03g0	Solyc03g0	0.41	-1.30
GO:00427	hydrogen p	Biological	0.08	0.33	Solyc04g0	Solyc04g0	0.48	-1.06
GO:00427	hydrogen p	Biological	0.08	0.33	Solyc06g0	Solyc06g0	0.25	-2.01
GO:00427	hydrogen p	Biological	0.08	0.33	Solyc07g0	Solyc07g0	2.87	1.52
GO:00064	protein ph	Biological	0.08	0.33	Solyc06g0	Solyc06g0	7.83	2.97
GO:00064	protein ph	Biological	0.08	0.33	Solyc09g0	Solyc09g0	2.98	1.58
GO:00064	protein ph	Biological	0.08	0.33	Solyc09g0	Solyc09g0	0.32	-1.64
GO:00064	protein ph	Biological	0.08	0.33	Solyc09g0	Solyc09g0	0.23	-2.09
GO:00064	protein ph	Biological	0.08	0.33	Solyc02g0	Solyc02g0	0.30	-1.71
GO:00064	protein ph	Biological	0.08	0.33	Solyc01g0	Solyc01g0	3.08	1.62
GO:00064	protein ph	Biological	0.08	0.33	Solyc09g0	Solyc09g0	0.29	-1.78
GO:00064	protein ph	Biological	0.08	0.33	Solyc01g1	Solyc01g1	3.76	1.91
GO:00064	protein ph	Biological	0.08	0.33	Solyc09g0	Solyc09g0	7.25	2.86
GO:00064	protein ph	Biological	0.08	0.33	Solyc09g0	Solyc09g0	2.40	1.26
GO:00064	protein ph	Biological	0.08	0.33	Solyc09g0	Solyc09g0	0.25	-2.01
GO:00064	protein ph	Biological	0.08	0.33	Solyc09g0	Solyc09g0	0.24	-2.04
GO:00064	protein ph	Biological	0.08	0.33	Solyc01g0	Solyc01g0	0.34	-1.54
GO:00064	protein ph	Biological	0.08	0.33	Solyc01g1	Solyc01g1	72.82	6.19

GO:00064	protein ph	Biological	0.08	0.33	Solyc03g0	Solyc03g0	3.18	1.67
GO:00064	protein ph	Biological	0.08	0.33	Solyc03g0	Solyc03g0	2.16	1.11
GO:00064	protein ph	Biological	0.08	0.33	Solyc03g0	Solyc03g0	7.56	2.92
GO:00064	protein ph	Biological	0.08	0.33	Solyc02g0	Solyc02g0	0.44	-1.19
GO:00064	protein ph	Biological	0.08	0.33	Solyc02g0	Solyc02g0	0.38	-1.41
GO:00064	protein ph	Biological	0.08	0.33	Solyc04g0	Solyc04g0	0.43	-1.23
GO:00064	protein ph	Biological	0.08	0.33	Solyc04g0	Solyc04g0	0.41	-1.29
GO:00064	protein ph	Biological	0.08	0.33	Solyc06g0	Solyc06g0	4.81	2.27
GO:00064	protein ph	Biological	0.08	0.33	Solyc02g0	Solyc02g0	0.50	-1.00
GO:00064	protein ph	Biological	0.08	0.33	Solyc06g0	Solyc06g0	0.37	-1.44
GO:00064	protein ph	Biological	0.08	0.33	Solyc09g0	Solyc09g0	0.37	-1.43
GO:00064	protein ph	Biological	0.08	0.33	Solyc05g0	Solyc05g0	0.50	-1.00
GO:00064	protein ph	Biological	0.08	0.33	Solyc05g0	Solyc05g0	0.22	-2.15
GO:00064	protein ph	Biological	0.08	0.33	Solyc11g0	Solyc11g0	6.63	2.73
GO:00064	protein ph	Biological	0.08	0.33	Solyc06g0	Solyc06g0	3.42	1.77
GO:00064	protein ph	Biological	0.08	0.33	Solyc07g0	Solyc07g0	2.15	1.11
GO:00064	protein ph	Biological	0.08	0.33	Solyc05g0	Solyc05g0	0.43	-1.22
GO:00064	protein ph	Biological	0.08	0.33	Solyc01g0	Solyc01g0	2.56	1.36
GO:00064	protein ph	Biological	0.08	0.33	Solyc03g1	Solyc03g1	0.49	-1.04
GO:00064	protein ph	Biological	0.08	0.33	Solyc11g0	Solyc11g0	0.46	-1.13
GO:00064	protein ph	Biological	0.08	0.33	Solyc11g0	Solyc11g0	2.57	1.36
GO:00064	protein ph	Biological	0.08	0.33	Solyc04g0	Solyc04g0	0.41	-1.28
GO:00064	protein ph	Biological	0.08	0.33	Solyc11g0	Solyc11g0	2.78	1.48
GO:00064	protein ph	Biological	0.08	0.33	Solyc12g0	Solyc12g0	2.20	1.14
GO:00064	protein ph	Biological	0.08	0.33	Solyc10g0	Solyc10g0	0.08	-3.68
GO:00064	protein ph	Biological	0.08	0.33	Solyc06g0	Solyc06g0	0.15	-2.77
GO:00064	protein ph	Biological	0.08	0.33	Solyc03g0	Solyc03g0	4.18	2.06
GO:00064	protein ph	Biological	0.08	0.33	Solyc01g0	Solyc01g0	0.41	-1.30
GO:00064	protein ph	Biological	0.08	0.33	Solyc11g0	Solyc11g0	2.24	1.16
GO:00064	protein ph	Biological	0.08	0.33	Solyc04g0	Solyc04g0	3.70	1.89
GO:00064	protein ph	Biological	0.08	0.33	Solyc12g0	Solyc12g0	0.41	-1.28
GO:00064	protein ph	Biological	0.08	0.33	Solyc06g0	Solyc06g0	0.43	-1.23
GO:00064	protein ph	Biological	0.08	0.33	Solyc04g0	Solyc04g0	0.46	-1.11
GO:00064	protein ph	Biological	0.08	0.33	Solyc08g0	Solyc08g0	0.50	-1.00
GO:00064	protein ph	Biological	0.08	0.33	Solyc10g0	Solyc10g0	4.22	2.08
GO:00064	protein ph	Biological	0.08	0.33	Solyc04g0	Solyc04g0	0.39	-1.37
GO:00064	protein ph	Biological	0.08	0.33	Solyc08g0	Solyc08g0	0.00	-10.00
GO:00064	protein ph	Biological	0.08	0.33	Solyc07g0	Solyc07g0	1180.56	10.21
GO:00064	protein ph	Biological	0.08	0.33	Solyc06g0	Solyc06g0	0.37	-1.44
GO:00064	protein ph	Biological	0.08	0.33	Solyc04g0	Solyc04g0	0.44	-1.18
GO:00064	protein ph	Biological	0.08	0.33	Solyc03g0	Solyc03g0	0.49	-1.03
GO:00064	protein ph	Biological	0.08	0.33	Solyc03g0	Solyc03g0	2.40	1.26
GO:00064	protein ph	Biological	0.08	0.33	Solyc01g0	Solyc01g0	5.28	2.40
GO:00064	protein ph	Biological	0.08	0.33	Solyc07g0	Solyc07g0	0.47	-1.10
GO:00064	protein ph	Biological	0.08	0.33	Solyc12g0	Solyc12g0	3.71	1.89
GO:00064	protein ph	Biological	0.08	0.33	Solyc06g0	Solyc06g0	912.98	9.83
GO:00064	protein ph	Biological	0.08	0.33	Solyc04g0	Solyc04g0	0.18	-2.51
GO:00064	protein ph	Biological	0.08	0.33	Solyc08g0	Solyc08g0	0.41	-1.30
GO:00064	protein ph	Biological	0.08	0.33	Solyc02g0	Solyc02g0	307.12	8.26
GO:00064	protein ph	Biological	0.08	0.33	Solyc02g0	Solyc02g0	2.27	1.18
GO:00064	protein ph	Biological	0.08	0.33	Solyc05g0	Solyc05g0	0.46	-1.13

GO:00064	protein ph	Biological	0.08	0.33	Solyc03g1	Solyc03g1	0.06	-4.15
GO:00064	protein ph	Biological	0.08	0.33	Solyc01g1	Solyc01g1	18.01	4.17
GO:00064	protein ph	Biological	0.08	0.33	Solyc02g0	Solyc02g0	0.17	-2.58
GO:00064	protein ph	Biological	0.08	0.33	Solyc07g0	Solyc07g0	2.39	1.26
GO:00064	protein ph	Biological	0.08	0.33	Solyc08g0	Solyc08g0	2.56	1.36
GO:00064	protein ph	Biological	0.08	0.33	Solyc01g0	Solyc01g0	0.00	-9.25
GO:00064	protein ph	Biological	0.08	0.33	Solyc07g0	Solyc07g0	2.77	1.47
GO:00064	protein ph	Biological	0.08	0.33	Solyc09g0	Solyc09g0	0.34	-1.58
GO:00064	protein ph	Biological	0.08	0.33	Solyc02g0	Solyc02g0	4.91	2.30
GO:00064	protein ph	Biological	0.08	0.33	Solyc03g1	Solyc03g1	356.83	8.48
GO:00064	protein ph	Biological	0.08	0.33	Solyc12g0	Solyc12g0	0.00	-11.51
GO:00064	protein ph	Biological	0.08	0.33	Solyc01g0	Solyc01g0	0.00	-9.77
GO:00064	protein ph	Biological	0.08	0.33	Solyc07g0	Solyc07g0	770.05	9.59
GO:00064	protein ph	Biological	0.08	0.33	Solyc08g0	Solyc08g0	4.19	2.07
GO:00064	protein ph	Biological	0.08	0.33	Solyc02g0	Solyc02g0	394.05	8.62
GO:00064	protein ph	Biological	0.08	0.33	Solyc02g0	Solyc02g0	0.07	-3.78
GO:00064	protein ph	Biological	0.08	0.33	Solyc03g0	Solyc03g0	0.36	-1.46
GO:00064	protein ph	Biological	0.08	0.33	Solyc07g0	Solyc07g0	2.88	1.52
GO:00064	protein ph	Biological	0.08	0.33	Solyc02g0	Solyc02g0	517.85	9.02
GO:00064	protein ph	Biological	0.08	0.33	Solyc08g0	Solyc08g0	0.11	-3.25
GO:00064	protein ph	Biological	0.08	0.33	Solyc06g0	Solyc06g0	9.89	3.31
GO:00064	protein ph	Biological	0.08	0.33	Solyc02g0	Solyc02g0	960.34	9.91
GO:00064	protein ph	Biological	0.08	0.33	Solyc07g0	Solyc07g0	0.39	-1.35
GO:00064	protein ph	Biological	0.08	0.33	Solyc03g0	Solyc03g0	0.39	-1.37
GO:00064	protein ph	Biological	0.08	0.33	Solyc11g0	Solyc11g0	2.48	1.31
GO:00064	protein ph	Biological	0.08	0.33	Solyc01g0	Solyc01g0	2.50	1.32
GO:00064	protein ph	Biological	0.08	0.33	Solyc12g0	Solyc12g0	11.23	3.49
GO:00064	protein ph	Biological	0.08	0.33	Solyc03g0	Solyc03g0	0.10	-3.29
GO:00064	protein ph	Biological	0.08	0.33	Solyc11g0	Solyc11g0	0.22	-2.20
GO:00037	DNA-bind	Molecular	0.08	0.34	Solyc09g0	Solyc09g0	6.00	2.58
GO:00037	DNA-bind	Molecular	0.08	0.34	Solyc06g0	Solyc06g0	7.71	2.95
GO:00037	DNA-bind	Molecular	0.08	0.34	Solyc03g0	Solyc03g0	0.24	-2.06
GO:00037	DNA-bind	Molecular	0.08	0.34	Solyc11g0	Solyc11g0	0.36	-1.47
GO:00037	DNA-bind	Molecular	0.08	0.34	Solyc04g0	Solyc04g0	2.73	1.45
GO:00037	DNA-bind	Molecular	0.08	0.34	Solyc02g0	Solyc02g0	2.17	1.12
GO:00037	DNA-bind	Molecular	0.08	0.34	Solyc11g0	Solyc11g0	10.55	3.40
GO:00037	DNA-bind	Molecular	0.08	0.34	Solyc12g0	Solyc12g0	2.65	1.40
GO:00037	DNA-bind	Molecular	0.08	0.34	Solyc09g0	Solyc09g0	0.32	-1.63
GO:00037	DNA-bind	Molecular	0.08	0.34	Solyc05g0	Solyc05g0	2.40	1.27
GO:00037	DNA-bind	Molecular	0.08	0.34	Solyc04g0	Solyc04g0	0.38	-1.40
GO:00037	DNA-bind	Molecular	0.08	0.34	Solyc11g0	Solyc11g0	2.29	1.19
GO:00037	DNA-bind	Molecular	0.08	0.34	Solyc06g0	Solyc06g0	0.46	-1.11
GO:00037	DNA-bind	Molecular	0.08	0.34	Solyc10g0	Solyc10g0	0.07	-3.89
GO:00037	DNA-bind	Molecular	0.08	0.34	Solyc01g0	Solyc01g0	0.44	-1.18
GO:00037	DNA-bind	Molecular	0.08	0.34	Solyc06g0	Solyc06g0	3.01	1.59
GO:00037	DNA-bind	Molecular	0.08	0.34	Solyc06g0	Solyc06g0	0.49	-1.03
GO:00037	DNA-bind	Molecular	0.08	0.34	Solyc08g0	Solyc08g0	0.49	-1.02
GO:00037	DNA-bind	Molecular	0.08	0.34	Solyc03g1	Solyc03g1	0.48	-1.06
GO:00037	DNA-bind	Molecular	0.08	0.34	Solyc07g0	Solyc07g0	0.34	-1.57
GO:00037	DNA-bind	Molecular	0.08	0.34	Solyc06g0	Solyc06g0	0.46	-1.11
GO:00037	DNA-bind	Molecular	0.08	0.34	Solyc02g0	Solyc02g0	0.42	-1.24

GO:00037	DNA-bind	Molecular	0.08	0.34	Solyc09g0	Solyc09g0	0.44	-1.19
GO:00037	DNA-bind	Molecular	0.08	0.34	Solyc05g0	Solyc05g0	4.20	2.07
GO:00037	DNA-bind	Molecular	0.08	0.34	Solyc04g0	Solyc04g0	0.21	-2.27
GO:00037	DNA-bind	Molecular	0.08	0.34	Solyc02g0	Solyc02g0	0.32	-1.64
GO:00037	DNA-bind	Molecular	0.08	0.34	Solyc11g0	Solyc11g0	2.73	1.45
GO:00037	DNA-bind	Molecular	0.08	0.34	Solyc06g0	Solyc06g0	2.54	1.35
GO:00037	DNA-bind	Molecular	0.08	0.34	Solyc06g0	Solyc06g0	0.35	-1.53
GO:00037	DNA-bind	Molecular	0.08	0.34	Solyc10g0	Solyc10g0	5392.75	12.40
GO:00037	DNA-bind	Molecular	0.08	0.34	Solyc11g0	Solyc11g0	0.50	-1.00
GO:00037	DNA-bind	Molecular	0.08	0.34	Solyc08g0	Solyc08g0	4.49	2.17
GO:00037	DNA-bind	Molecular	0.08	0.34	Solyc02g0	Solyc02g0	0.42	-1.26
GO:00037	DNA-bind	Molecular	0.08	0.34	Solyc05g0	Solyc05g0	31.58	4.98
GO:00037	DNA-bind	Molecular	0.08	0.34	Solyc08g0	Solyc08g0	0.38	-1.39
GO:00037	DNA-bind	Molecular	0.08	0.34	Solyc09g0	Solyc09g0	0.25	-1.98
GO:00037	DNA-bind	Molecular	0.08	0.34	Solyc10g0	Solyc10g0	0.33	-1.59
GO:00037	DNA-bind	Molecular	0.08	0.34	Solyc08g0	Solyc08g0	0.37	-1.44
GO:00037	DNA-bind	Molecular	0.08	0.34	Solyc06g0	Solyc06g0	2.01	1.01
GO:00037	DNA-bind	Molecular	0.08	0.34	Solyc06g0	Solyc06g0	0.39	-1.37
GO:00037	DNA-bind	Molecular	0.08	0.34	Solyc12g0	Solyc12g0	0.33	-1.61
GO:00037	DNA-bind	Molecular	0.08	0.34	Solyc07g0	Solyc07g0	2.33	1.22
GO:00037	DNA-bind	Molecular	0.08	0.34	Solyc11g0	Solyc11g0	0.00	-10.83
GO:00037	DNA-bind	Molecular	0.08	0.34	Solyc02g0	Solyc02g0	25.47	4.67
GO:00037	DNA-bind	Molecular	0.08	0.34	Solyc02g0	Solyc02g0	2.66	1.41
GO:00037	DNA-bind	Molecular	0.08	0.34	Solyc03g1	Solyc03g1	0.06	-4.16
GO:00037	DNA-bind	Molecular	0.08	0.34	Solyc09g0	Solyc09g0	1363.92	10.41
GO:00037	DNA-bind	Molecular	0.08	0.34	Solyc10g0	Solyc10g0	0.27	-1.87
GO:00037	DNA-bind	Molecular	0.08	0.34	Solyc02g0	Solyc02g0	0.39	-1.37
GO:00037	DNA-bind	Molecular	0.08	0.34	Solyc02g0	Solyc02g0	8.19	3.03
GO:00037	DNA-bind	Molecular	0.08	0.34	Solyc02g0	Solyc02g0	0.38	-1.40
GO:00037	DNA-bind	Molecular	0.08	0.34	Solyc03g1	Solyc03g1	2361.02	11.21
GO:00037	DNA-bind	Molecular	0.08	0.34	Solyc04g0	Solyc04g0	0.00	-12.04
GO:00037	DNA-bind	Molecular	0.08	0.34	Solyc10g0	Solyc10g0	0.29	-1.77
GO:00037	DNA-bind	Molecular	0.08	0.34	Solyc03g1	Solyc03g1	734.02	9.52
GO:00037	DNA-bind	Molecular	0.08	0.34	Solyc07g0	Solyc07g0	42.77	5.42
GO:00037	DNA-bind	Molecular	0.08	0.34	Solyc10g0	Solyc10g0	683.10	9.42
GO:00037	DNA-bind	Molecular	0.08	0.34	Solyc03g0	Solyc03g0	3218.95	11.65
GO:00037	DNA-bind	Molecular	0.08	0.34	Solyc01g0	Solyc01g0	6.82	2.77
GO:00037	DNA-bind	Molecular	0.08	0.34	Solyc10g0	Solyc10g0	2.60	1.38
GO:00037	DNA-bind	Molecular	0.08	0.34	Solyc06g0	Solyc06g0	15.65	3.97
GO:00037	DNA-bind	Molecular	0.08	0.34	Solyc01g0	Solyc01g0	0.48	-1.06
GO:00037	DNA-bind	Molecular	0.08	0.34	Solyc06g0	Solyc06g0	8.85	3.15
GO:00037	DNA-bind	Molecular	0.08	0.34	Solyc05g0	Solyc05g0	3.77	1.92
GO:00037	DNA-bind	Molecular	0.08	0.34	Solyc01g0	Solyc01g0	0.42	-1.24
GO:00037	DNA-bind	Molecular	0.08	0.34	Solyc02g0	Solyc02g0	1506.28	10.56
GO:00037	DNA-bind	Molecular	0.08	0.34	Solyc03g0	Solyc03g0	0.46	-1.13
GO:00037	DNA-bind	Molecular	0.08	0.34	Solyc11g0	Solyc11g0	0.47	-1.09
GO:00037	DNA-bind	Molecular	0.08	0.34	Solyc01g0	Solyc01g0	0.24	-2.09
GO:00037	DNA-bind	Molecular	0.08	0.34	Solyc06g0	Solyc06g0	0.45	-1.16
GO:00037	DNA-bind	Molecular	0.08	0.34	Solyc06g0	PTI6	0.43	-1.22
GO:00037	DNA-bind	Molecular	0.08	0.34	Solyc08g0	Solyc08g0	0.41	-1.27
GO:00037	DNA-bind	Molecular	0.08	0.34	Solyc04g0	Solyc04g0	2.34	1.23

GO:00037	DNA-bind	Molecular	0.08	0.34	Solyc10g0	Solyc10g0	2.42	1.27
GO:00037	DNA-bind	Molecular	0.08	0.34	Solyc04g0	Solyc04g0	732.58	9.52
GO:00037	DNA-bind	Molecular	0.08	0.34	Solyc05g0	Solyc05g0	6.61	2.73
GO:00037	DNA-bind	Molecular	0.08	0.34	Solyc11g0	Solyc11g0	0.00	-9.22
GO:00037	DNA-bind	Molecular	0.08	0.34	Solyc02g0	Solyc02g0	7.52	2.91
GO:00080	microtubul	Molecular	0.08	0.35	Solyc09g0	Solyc09g0	0.05	-4.40
GO:00080	microtubul	Molecular	0.08	0.35	Solyc02g0	Solyc02g0	11.83	3.56
GO:00080	microtubul	Molecular	0.08	0.35	Solyc12g0	Solyc12g0	0.48	-1.07
GO:00080	microtubul	Molecular	0.08	0.35	Solyc07g0	Solyc07g0	8.41	3.07
GO:00080	microtubul	Molecular	0.08	0.35	Solyc02g0	Solyc02g0	2.08	1.06
GO:00080	microtubul	Molecular	0.08	0.35	Solyc09g0	Solyc09g0	0.07	-3.89
GO:00080	microtubul	Molecular	0.08	0.35	Solyc06g0	Solyc06g0	0.34	-1.55
GO:00080	microtubul	Molecular	0.08	0.35	Solyc06g0	Solyc06g0	0.34	-1.57
GO:00080	microtubul	Molecular	0.08	0.35	Solyc12g0	Solyc12g0	467.31	8.87
GO:00080	microtubul	Molecular	0.08	0.35	Solyc01g0	Solyc01g0	0.00	-10.07
GO:00080	microtubul	Molecular	0.08	0.35	Solyc09g0	Solyc09g0	0.35	-1.53
GO:00080	microtubul	Molecular	0.08	0.35	Solyc06g0	Solyc06g0	0.07	-3.77
GO:00080	microtubul	Molecular	0.08	0.35	Solyc07g0	Solyc07g0	0.11	-3.12
GO:00080	microtubul	Molecular	0.08	0.35	Solyc04g0	Solyc04g0	553.61	9.11
GO:00428	identical p	Molecular	0.08	0.35	Solyc09g0	Solyc09g0	0.34	-1.58
GO:00428	identical p	Molecular	0.08	0.35	Solyc11g0	Solyc11g0	5.99	2.58
GO:00428	identical p	Molecular	0.08	0.35	Solyc12g0	Solyc12g0	369.30	8.53
GO:00428	identical p	Molecular	0.08	0.35	Solyc01g0	Solyc01g0	0.45	-1.14
GO:00428	identical p	Molecular	0.08	0.35	Solyc11g0	Solyc11g0	18.97	4.25
GO:00347	regulation	Biological	0.08	0.35	Solyc01g1	Solyc01g1	0.29	-1.80
GO:00347	regulation	Biological	0.08	0.35	Solyc12g0	Solyc12g0	369.30	8.53
GO:00425	phosphoric	Molecular	0.08	0.35	Solyc05g0	Solyc05g0	3.20	1.68
GO:00425	phosphoric	Molecular	0.08	0.35	Solyc09g0	Solyc09g0	25.41	4.67
GO:00061	ferredoxin	Biological	0.08	0.35	Solyc11g0	Solyc11g0	0.32	-1.67
GO:00061	ferredoxin	Biological	0.08	0.35	Solyc11g0	Solyc11g0	2.20	1.14
GO:00058	cytoplasmi	Cellular C	0.08	0.35	Solyc07g0	Solyc07g0	3.21	1.68
GO:00058	cytoplasmi	Cellular C	0.08	0.35	Solyc03g1	Solyc03g1	2.39	1.26
GO:00455	dynein inte	Molecular	0.08	0.35	Solyc07g0	Solyc07g0	3.21	1.68
GO:00455	dynein inte	Molecular	0.08	0.35	Solyc03g1	Solyc03g1	2.39	1.26
GO:00519	dynein ligh	Molecular	0.08	0.35	Solyc07g0	Solyc07g0	3.21	1.68
GO:00519	dynein ligh	Molecular	0.08	0.35	Solyc03g1	Solyc03g1	2.39	1.26
GO:20005	obsolete p	Biological	0.08	0.35	Solyc07g0	Solyc07g0	3.21	1.68
GO:20005	obsolete p	Biological	0.08	0.35	Solyc03g1	Solyc03g1	2.39	1.26
GO:00099	polarity sp	Biological	0.08	0.35	Solyc08g0	Solyc08g0	2486.58	11.28
GO:00099	polarity sp	Biological	0.08	0.35	Solyc08g0	Solyc08g0	4.19	2.07
GO:00157	nitrate tran	Biological	0.08	0.35	Solyc02g0	Solyc02g0	19.78	4.31
GO:00157	nitrate tran	Biological	0.08	0.35	Solyc06g0	Solyc06g0	0.32	-1.62
GO:00090	branched-c	Biological	0.09	0.36	Solyc08g0	Solyc08g0	2.65	1.41
GO:00090	branched-c	Biological	0.09	0.36	Solyc03g0	Solyc03g0	4.05	2.02
GO:00090	branched-c	Biological	0.09	0.36	Solyc11g0	Solyc11g0	2.27	1.18
GO:00229	respiratory	Biological	0.09	0.36	Solyc01g0	Solyc01g0	0.02	-5.72
GO:00229	respiratory	Biological	0.09	0.36	Solyc04g0	Solyc04g0	39.10	5.29
GO:00229	respiratory	Biological	0.09	0.36	Solyc04g0	Solyc04g0	32.87	5.04
GO:00152	channel ac	Molecular	0.09	0.38	Solyc06g0	Solyc06g0	2.02	1.02
GO:00152	channel ac	Molecular	0.09	0.38	Solyc10g0	Solyc10g0	2.56	1.36
GO:00152	channel ac	Molecular	0.09	0.38	Solyc01g0	Solyc01g0	0.49	-1.04

GO:00152	channel ac	Molecular	0.09	0.38	Solyc01g1	Solyc01g1	2829.11	11.47
GO:00152	channel ac	Molecular	0.09	0.38	Solyc08g0	Solyc08g0	0.29	-1.80
GO:00152	channel ac	Molecular	0.09	0.38	Solyc03g0	Solyc03g0	0.00	-9.99
GO:00164	O-acetyltra	Molecular	0.09	0.38	Solyc02g0	Solyc02g0	0.21	-2.23
GO:00164	O-acetyltra	Molecular	0.09	0.38	Solyc01g0	Solyc01g0	2.42	1.27
GO:00164	O-acetyltra	Molecular	0.09	0.38	Solyc09g0	Solyc09g0	0.49	-1.04
GO:00164	O-acetyltra	Molecular	0.09	0.38	Solyc02g0	Solyc02g0	3.38	1.76
GO:00164	O-acetyltra	Molecular	0.09	0.38	Solyc07g0	Solyc07g0	0.01	-7.50
GO:00046	calmodulin	Molecular	0.09	0.38	Solyc01g0	Solyc01g0	0.34	-1.54
GO:00046	calmodulin	Molecular	0.09	0.38	Solyc11g0	Solyc11g0	2.24	1.16
GO:00046	calmodulin	Molecular	0.09	0.38	Solyc01g0	Solyc01g0	5.28	2.40
GO:00046	calmodulin	Molecular	0.09	0.38	Solyc12g0	Solyc12g0	3.71	1.89
GO:00046	calmodulin	Molecular	0.09	0.38	Solyc01g0	Solyc01g0	0.00	-9.77
GO:00099	calcium-de	Molecular	0.09	0.38	Solyc01g0	Solyc01g0	0.34	-1.54
GO:00099	calcium-de	Molecular	0.09	0.38	Solyc11g0	Solyc11g0	2.24	1.16
GO:00099	calcium-de	Molecular	0.09	0.38	Solyc01g0	Solyc01g0	5.28	2.40
GO:00099	calcium-de	Molecular	0.09	0.38	Solyc12g0	Solyc12g0	3.71	1.89
GO:00099	calcium-de	Molecular	0.09	0.38	Solyc01g0	Solyc01g0	0.00	-9.77
GO:00045	chitinase a	Molecular	0.10	0.39	Solyc10g0	CHI9	0.28	-1.84
GO:00045	chitinase a	Molecular	0.10	0.39	Solyc02g0	Solyc02g0	0.33	-1.59
GO:00045	chitinase a	Molecular	0.10	0.39	Solyc06g0	Solyc06g0	13.74	3.78
GO:00045	chitinase a	Molecular	0.10	0.39	Solyc10g0	Solyc10g0	0.00	-9.02
GO:00152	water chan	Molecular	0.10	0.39	Solyc06g0	Solyc06g0	2.02	1.02
GO:00152	water chan	Molecular	0.10	0.39	Solyc10g0	Solyc10g0	2.56	1.36
GO:00152	water chan	Molecular	0.10	0.39	Solyc08g0	Solyc08g0	0.29	-1.80
GO:00152	water chan	Molecular	0.10	0.39	Solyc10g0	Solyc10g0	5617.07	12.46
GO:20000	regulation	Biological	0.10	0.39	Solyc10g0	Solyc10g0	65.27	6.03
GO:20000	regulation	Biological	0.10	0.39	Solyc03g0	Solyc03g0	3.90	1.96
GO:20000	regulation	Biological	0.10	0.39	Solyc12g0	Solyc12g0	3.08	1.62
GO:00452	proton-trar	Cellular C	0.10	0.39	Solyc06g0	Solyc06g0	2.29	1.20
GO:00452	proton-trar	Cellular C	0.10	0.39	Solyc04g0	Solyc04g0	0.16	-2.66
GO:00452	proton-trar	Cellular C	0.10	0.39	Solyc11g0	Solyc11g0	0.22	-2.19
GO:00099	chloroplast	Cellular C	0.10	0.39	Solyc11g0	AOS2	3.20	1.68
GO:00099	chloroplast	Cellular C	0.10	0.39	Solyc12g0	Solyc12g0	2.97	1.57
GO:00099	chloroplast	Cellular C	0.10	0.39	Solyc09g0	Solyc09g0	5.85	2.55
GO:00099	chloroplast	Cellular C	0.10	0.39	Solyc12g0	Solyc12g0	2.10	1.07
GO:00099	chloroplast	Cellular C	0.10	0.39	Solyc02g0	Solyc02g0	2.62	1.39
GO:00099	chloroplast	Cellular C	0.10	0.39	Solyc03g1	Solyc03g1	4.55	2.18
GO:00099	chloroplast	Cellular C	0.10	0.39	Solyc01g0	Solyc01g0	2.02	1.02
GO:00099	chloroplast	Cellular C	0.10	0.39	Solyc05g0	Solyc05g0	2.32	1.21
GO:00099	chloroplast	Cellular C	0.10	0.39	Solyc06g0	Solyc06g0	3.33	1.73
GO:00099	chloroplast	Cellular C	0.10	0.39	Solyc07g0	Solyc07g0	2.47	1.30
GO:00099	chloroplast	Cellular C	0.10	0.39	Solyc03g0	Solyc03g0	2.60	1.38
GO:00099	chloroplast	Cellular C	0.10	0.39	Solyc01g0	Solyc01g0	550.66	9.11
GO:00097	auxin-activ	Biological	0.10	0.39	Solyc09g0	Solyc09g0	0.34	-1.58
GO:00097	auxin-activ	Biological	0.10	0.39	Solyc08g0	Solyc08g0	0.20	-2.35
GO:00097	auxin-activ	Biological	0.10	0.39	Solyc11g0	Solyc11g0	3.29	1.72
GO:00097	auxin-activ	Biological	0.10	0.39	Solyc03g1	Solyc03g1	0.33	-1.59
GO:00097	auxin-activ	Biological	0.10	0.39	Solyc01g0	Solyc01g0	6.50	2.70
GO:00097	auxin-activ	Biological	0.10	0.39	Solyc10g0	Solyc10g0	0.37	-1.43
GO:00097	auxin-activ	Biological	0.10	0.39	Solyc06g0	Solyc06g0	0.45	-1.16

GO:00097	auxin-acti	Biological	0.10	0.39	Solyc01g0	Solyc01g0	0.45	-1.14
GO:00097	auxin-acti	Biological	0.10	0.39	Solyc09g0	Solyc09g0	0.22	-2.22
GO:00097	abscisic ac	Biological	0.10	0.39	Solyc09g0	Solyc09g0	0.05	-4.31
GO:00097	abscisic ac	Biological	0.10	0.39	Solyc01g0	Solyc01g0	0.34	-1.54
GO:00097	abscisic ac	Biological	0.10	0.39	Solyc09g0	Solyc09g0	0.27	-1.90
GO:00097	abscisic ac	Biological	0.10	0.39	Solyc09g0	Solyc09g0	8.11	3.02
GO:00097	abscisic ac	Biological	0.10	0.39	Solyc11g0	Solyc11g0	2.24	1.16
GO:00097	abscisic ac	Biological	0.10	0.39	Solyc01g0	Solyc01g0	5.28	2.40
GO:00097	abscisic ac	Biological	0.10	0.39	Solyc12g0	Solyc12g0	3.71	1.89
GO:00097	abscisic ac	Biological	0.10	0.39	Solyc01g0	Solyc01g0	0.00	-9.77
GO:00100	positive re	Biological	0.11	0.39	Solyc07g0	ACO1	2.82	1.49
GO:00100	positive re	Biological	0.11	0.39	Solyc02g0	Solyc02g0	2.56	1.36
GO:00717	cellular res	Biological	0.11	0.39	Solyc07g0	ACO1	2.82	1.49
GO:00717	cellular res	Biological	0.11	0.39	Solyc02g0	Solyc02g0	2.56	1.36
GO:00801	regulation	Biological	0.11	0.39	Solyc07g0	Solyc07g0	3.16	1.66
GO:00801	regulation	Biological	0.11	0.39	Solyc02g0	Solyc02g0	0.21	-2.28
GO:00166	malate deh	Molecular	0.11	0.39	Solyc11g0	Solyc11g0	4.12	2.04
GO:00166	malate deh	Molecular	0.11	0.39	Solyc03g0	Solyc03g0	2.79	1.48
GO:00192	reductive p	Biological	0.11	0.39	Solyc01g0	Solyc01g0	0.03	-5.12
GO:00192	reductive p	Biological	0.11	0.39	Solyc03g0	RBCS-2A	2.71	1.44
GO:00043	glyceralde	Molecular	0.11	0.39	Solyc04g0	Solyc04g0	8.55	3.10
GO:00043	glyceralde	Molecular	0.11	0.39	Solyc02g0	Solyc02g0	2.27	1.18
GO:00089	phosphoen	Molecular	0.11	0.39	Solyc11g0	Solyc11g0	20.23	4.34
GO:00089	phosphoen	Molecular	0.11	0.39	Solyc09g0	Solyc09g0	8.04	3.01
GO:00517	cellular res	Biological	0.11	0.39	Solyc09g0	Solyc09g0	0.40	-1.34
GO:00517	cellular res	Biological	0.11	0.39	Solyc12g0	Solyc12g0	2.29	1.20
GO:00100	phloem de	Biological	0.11	0.39	Solyc03g1	Solyc03g1	0.17	-2.53
GO:00100	phloem de	Biological	0.11	0.39	Solyc05g0	Solyc05g0	0.34	-1.54
GO:00099	negative g	Biological	0.11	0.39	Solyc03g0	Solyc03g0	0.48	-1.05
GO:00099	negative g	Biological	0.11	0.39	Solyc02g0	Solyc02g0	2.58	1.37
GO:00060	fructose m	Biological	0.11	0.39	Solyc10g0	Solyc10g0	2.23	1.16
GO:00060	fructose m	Biological	0.11	0.39	Solyc09g0	Solyc09g0	25.41	4.67
GO:00451	cell fate co	Biological	0.11	0.39	Solyc11g0	Solyc11g0	18.46	4.21
GO:00451	cell fate co	Biological	0.11	0.39	Solyc08g0	Solyc08g0	2486.58	11.28
GO:00801	intracellul	Biological	0.11	0.39	Solyc03g0	Solyc03g0	15.63	3.97
GO:00801	intracellul	Biological	0.11	0.39	Solyc02g0	Solyc02g0	2.19	1.13
GO:00105	NAD(P)H	Cellular C	0.11	0.39	Solyc05g0	Solyc05g0	6.90	2.79
GO:00105	NAD(P)H	Cellular C	0.11	0.39	Solyc06g0	Solyc06g0	2.66	1.41
GO:00103	auxin efflu	Biological	0.11	0.39	Solyc03g1	Solyc03g1	0.33	-1.59
GO:00103	auxin efflu	Biological	0.11	0.39	Solyc10g0	Solyc10g0	0.37	-1.43
GO:00103	auxin efflu	Molecular	0.11	0.39	Solyc03g1	Solyc03g1	0.33	-1.59
GO:00103	auxin efflu	Molecular	0.11	0.39	Solyc10g0	Solyc10g0	0.37	-1.43
GO:00085	plus-end-d	Molecular	0.11	0.39	Solyc09g0	Solyc09g0	0.07	-3.89
GO:00085	plus-end-d	Molecular	0.11	0.39	Solyc09g0	Solyc09g0	0.35	-1.53
GO:00452	respiratory	Cellular C	0.11	0.39	Solyc08g0	Solyc08g0	8.58	3.10
GO:00452	respiratory	Cellular C	0.11	0.39	Solyc11g0	Solyc11g0	0.09	-3.52
GO:00345	cellular res	Biological	0.11	0.39	Solyc10g0	Solyc10g0	4.23	2.08
GO:00345	cellular res	Biological	0.11	0.39	Solyc11g0	Solyc11g0	8.62	3.11
GO:00345	cellular res	Biological	0.11	0.39	Solyc03g1	E4	2.38	1.25
GO:00345	cellular res	Biological	0.11	0.39	Solyc02g0	Solyc02g0	2.54	1.34
GO:00039	NADH del	Molecular	0.11	0.39	Solyc10g0	Solyc10g0	0.00	-13.73

GO:00039	NADH del	Molecular	0.11	0.39	Solyc11g0	Solyc11g0	0.00	-11.68
GO:00039	NADH del	Molecular	0.11	0.39	Solyc02g0	Solyc02g0	0.19	-2.37
GO:00039	NADH del	Molecular	0.11	0.39	Solyc01g0	Solyc01g0	0.08	-3.58
GO:00166	oxidoreduc	Molecular	0.11	0.39	Solyc02g0	Solyc02g0	0.35	-1.50
GO:00166	oxidoreduc	Molecular	0.11	0.39	Solyc04g0	Solyc04g0	8.55	3.10
GO:00166	oxidoreduc	Molecular	0.11	0.39	Solyc01g0	Solyc01g0	0.22	-2.18
GO:00166	oxidoreduc	Molecular	0.11	0.39	Solyc12g0	Solyc12g0	4.01	2.00
GO:00166	oxidoreduc	Molecular	0.11	0.39	Solyc02g0	Solyc02g0	2.27	1.18
GO:00094	response tc	Biological	0.12	0.39	Solyc04g0	Solyc04g0	2.70	1.43
GO:00094	response tc	Biological	0.12	0.39	Solyc10g0	Solyc10g0	0.09	-3.53
GO:00094	response tc	Biological	0.12	0.39	Solyc02g0	Solyc02g0	0.45	-1.14
GO:00094	response tc	Biological	0.12	0.39	Solyc02g0	Solyc02g0	2.46	1.30
GO:00094	response tc	Biological	0.12	0.39	Solyc05g0	Solyc05g0	4.68	2.23
GO:00094	response tc	Biological	0.12	0.39	Solyc01g0	Solyc01g0	3.53	1.82
GO:00056	chromosor	Cellular Co	0.12	0.39	Solyc01g0	Solyc01g0	3.72	1.90
GO:00056	chromosor	Cellular Co	0.12	0.39	Solyc01g1	Solyc01g1	2.32	1.22
GO:00056	chromosor	Cellular Co	0.12	0.39	Solyc04g0	Solyc04g0	2.47	1.30
GO:00056	chromosor	Cellular Co	0.12	0.39	Solyc11g0	Solyc11g0	2.31	1.21
GO:00056	chromosor	Cellular Co	0.12	0.39	Solyc06g0	Solyc06g0	2.48	1.31
GO:00056	chromosor	Cellular Co	0.12	0.39	Solyc05g0	Solyc05g0	2.76	1.47
GO:00199	protein kin	Molecular	0.12	0.39	Solyc06g0	Solyc06g0	4.81	2.27
GO:00199	protein kin	Molecular	0.12	0.39	Solyc02g0	Solyc02g0	6.74	2.75
GO:00199	protein kin	Molecular	0.12	0.39	Solyc05g0	Solyc05g0	0.22	-2.15
GO:00199	protein kin	Molecular	0.12	0.39	Solyc04g0	Solyc04g0	0.41	-1.28
GO:00199	protein kin	Molecular	0.12	0.39	Solyc03g0	Solyc03g0	4.18	2.06
GO:00199	protein kin	Molecular	0.12	0.39	Solyc02g0	Solyc02g0	4.91	2.30
GO:00442	cellular me	Biological	0.12	0.39	Solyc09g0	Solyc09g0	3.01	1.59
GO:00442	cellular me	Biological	0.12	0.39	Solyc01g1	Solyc01g1	2.32	1.22
GO:00442	cellular me	Biological	0.12	0.39	Solyc07g0	Solyc07g0	2.07	1.05
GO:00096	anatomical	Biological	0.12	0.39	Solyc01g1	Solyc01g1	2.90	1.54
GO:00096	anatomical	Biological	0.12	0.39	Solyc06g0	Solyc06g0	0.11	-3.25
GO:00096	anatomical	Biological	0.12	0.39	Solyc03g1	Solyc03g1	0.15	-2.75
GO:00096	gibberellin	Biological	0.12	0.39	Solyc07g0	Solyc07g0	2.20	1.14
GO:00096	gibberellin	Biological	0.12	0.39	Solyc07g0	Solyc07g0	29.42	4.88
GO:00096	gibberellin	Biological	0.12	0.39	Solyc08g0	Solyc08g0	8.90	3.15
GO:00097	plant-type	Cellular Co	0.12	0.39	Solyc03g1	Solyc03g1	0.39	-1.35
GO:00097	plant-type	Cellular Co	0.12	0.39	Solyc09g0	Solyc09g0	0.26	-1.97
GO:00097	plant-type	Cellular Co	0.12	0.39	Solyc06g0	Solyc06g0	2059.41	11.01
GO:00097	plant-type	Cellular Co	0.12	0.39	Solyc08g0	Solyc08g0	0.29	-1.80
GO:00097	plant-type	Cellular Co	0.12	0.39	Solyc02g0	Solyc02g0	19.78	4.31
GO:00003	response tc	Biological	0.12	0.39	Solyc07g0	Solyc07g0	2.77	1.47
GO:00003	response tc	Biological	0.12	0.39	Solyc10g0	Solyc10g0	0.09	-3.53
GO:00003	response tc	Biological	0.12	0.39	Solyc02g0	Solyc02g0	2.54	1.34
GO:00003	response tc	Biological	0.12	0.39	Solyc02g0	Solyc02g0	2.46	1.30
GO:00516	defense res	Biological	0.12	0.39	Solyc09g0	Solyc09g0	2.34	1.23
GO:00516	defense res	Biological	0.12	0.39	Solyc02g0	Solyc02g0	0.49	-1.04
GO:00516	defense res	Biological	0.12	0.39	Solyc06g0	Solyc06g0	2.07	1.05
GO:00516	defense res	Biological	0.12	0.39	Solyc03g0	RBCS-2A	2.71	1.44
GO:00070	regulation	Biological	0.12	0.39	Solyc05g0	Solyc05g0	0.22	-2.15
GO:00070	regulation	Biological	0.12	0.39	Solyc04g0	Solyc04g0	0.41	-1.28
GO:00070	regulation	Biological	0.12	0.39	Solyc03g0	Solyc03g0	4.18	2.06

GO:00070	regulation	Biological	0.12	0.39	Solyc02g0	Solyc02g0	4.91	2.30
GO:00432	intracellula	Cellular Co	0.13	0.39	Solyc02g0	Solyc02g0	11.82	3.56
GO:00432	intracellula	Cellular Co	0.13	0.39	Solyc04g0	Solyc04g0	2.07	1.05
GO:00432	intracellula	Cellular Co	0.13	0.39	Solyc09g0	Solyc09g0	0.05	-4.37
GO:00432	intracellula	Cellular Co	0.13	0.39	Solyc12g0	Solyc12g0	2.38	1.25
GO:00432	intracellula	Cellular Co	0.13	0.39	Solyc12g0	Solyc12g0	7107.62	12.80
GO:00432	intracellula	Cellular Co	0.13	0.39	Solyc12g0	Solyc12g0	0.00	-13.90
GO:00432	intracellula	Cellular Co	0.13	0.39	Solyc05g0	Solyc05g0	0.03	-5.29
GO:00432	intracellula	Cellular Co	0.13	0.39	Solyc11g0	Solyc11g0	0.25	-2.02
GO:00432	intracellula	Cellular Co	0.13	0.39	Solyc07g0	Solyc07g0	6.57	2.72
GO:00432	intracellula	Cellular Co	0.13	0.39	Solyc12g0	Solyc12g0	0.00	-9.95
GO:00432	intracellula	Cellular Co	0.13	0.39	Solyc11g0	Solyc11g0	0.22	-2.19
GO:00432	intracellula	Cellular Co	0.13	0.39	Solyc01g1	Solyc01g1	0.47	-1.07
GO:00097	blue light s	Biological	0.13	0.39	Solyc09g0	Solyc09g0	0.40	-1.34
GO:00097	blue light s	Biological	0.13	0.39	Solyc12g0	Solyc12g0	2.29	1.20
GO:00099	positive re	Biological	0.13	0.39	Solyc03g0	Solyc03g0	3.84	1.94
GO:00099	positive re	Biological	0.13	0.39	Solyc03g0	Solyc03g0	0.49	-1.02
GO:00101	response to	Biological	0.13	0.39	Solyc10g0	Solyc10g0	2.02	1.01
GO:00101	response to	Biological	0.13	0.39	Solyc02g0	Solyc02g0	19.78	4.31
GO:00432	intracellula	Cellular Co	0.13	0.39	Solyc09g0	rpl33	0.05	-4.19
GO:00432	intracellula	Cellular Co	0.13	0.39	Solyc09g0	Solyc09g0	0.00	-13.63
GO:00099	chloroplast	Biological	0.13	0.39	Solyc06g0	Solyc06g0	3.04	1.60
GO:00099	chloroplast	Biological	0.13	0.39	Solyc02g0	Solyc02g0	2.66	1.41
GO:00055	calcium-de	Molecular	0.13	0.39	Solyc04g0	Solyc04g0	2.70	1.43
GO:00055	calcium-de	Molecular	0.13	0.39	Solyc04g0	Solyc04g0	3.53	1.82
GO:00055	calcium-de	Molecular	0.13	0.39	Solyc10g0	Solyc10g0	0.25	-1.99
GO:00457	positive re	Biological	0.14	0.39	Solyc05g0	Solyc05g0	0.22	-2.15
GO:00457	positive re	Biological	0.14	0.39	Solyc04g0	Solyc04g0	0.41	-1.28
GO:00457	positive re	Biological	0.14	0.39	Solyc03g0	Solyc03g0	4.18	2.06
GO:00457	positive re	Biological	0.14	0.39	Solyc02g0	Solyc02g0	4.91	2.30
GO:00342	ion transm	Biological	0.14	0.39	Solyc01g0	Solyc01g0	5.13	2.36
GO:00342	ion transm	Biological	0.14	0.39	Solyc03g1	Solyc03g1	0.39	-1.35
GO:00342	ion transm	Biological	0.14	0.39	Solyc08g0	Solyc08g0	0.42	-1.27
GO:00342	ion transm	Biological	0.14	0.39	Solyc12g0	Solyc12g0	369.30	8.53
GO:00100	fruit dehisc	Biological	0.14	0.39	Solyc10g0	PG2	9.48	3.25
GO:00102	abscisic ac	Molecular	0.14	0.39	Solyc09g0	Solyc09g0	0.05	-4.31
GO:00040	asparagina	Molecular	0.14	0.39	Solyc04g0	Solyc04g0	6.77	2.76
GO:00087	beta-aspart	Molecular	0.14	0.39	Solyc04g0	Solyc04g0	6.77	2.76
GO:00333	asparagine	Biological	0.14	0.39	Solyc04g0	Solyc04g0	6.77	2.76
GO:00471	betaine-ho	Molecular	0.14	0.39	Solyc09g0	Solyc09g0	6.99	2.80
GO:00442	cellular ca	Biological	0.14	0.39	Solyc07g0	Solyc07g0	0.35	-1.51
GO:00465	2,3-bispho	Molecular	0.14	0.39	Solyc07g0	Solyc07g0	0.35	-1.51
GO:00356	root hair ti	Cellular Co	0.14	0.39	Solyc04g0	Solyc04g0	2.70	1.43
GO:00500	L-3-cyano	Molecular	0.14	0.39	Solyc01g0	Solyc01g0	2.46	1.30
GO:00442	'de novo' C	Biological	0.14	0.39	Solyc02g0	Solyc02g0	3.08	1.62
GO:00040	alcohol del	Molecular	0.14	0.39	Solyc12g0	Solyc12g0	2.82	1.50
GO:00166	cinnamoyl	Molecular	0.14	0.39	Solyc06g0	Solyc06g0	2.22	1.15
GO:01021	N-acetyl-b	Molecular	0.14	0.39	Solyc01g0	Solyc01g0	2.01	1.01
GO:00061	oxaloaceta	Biological	0.14	0.39	Solyc11g0	Solyc11g0	4.12	2.04
GO:00100	xanthophy	Biological	0.14	0.39	Solyc04g0	Solyc04g0	3.50	1.81
GO:00464	violaxanth	Molecular	0.14	0.39	Solyc04g0	Solyc04g0	3.50	1.81

GO:19901	linoleate 9	Molecular	0.14	0.39	Solyc08g0	LOX1.1	2.60	1.38
GO:00479	GDP-manr	Molecular	0.14	0.39	Solyc09g0	Solyc09g0	0.37	-1.45
GO:19902	RNA polyı	Molecular	0.14	0.39	Solyc09g0	Solyc09g0	0.18	-2.45
GO:00161	carotenoid	Biological	0.14	0.39	Solyc05g0	Solyc05g0	2.65	1.41
GO:00080	protein C-t	Molecular	0.14	0.39	Solyc05g0	Solyc05g0	0.48	-1.05
GO:19904	peroxisom	Cellular C	0.14	0.39	Solyc05g0	Solyc05g0	0.48	-1.05
GO:00047	pyridoxam	Molecular	0.14	0.39	Solyc03g1	Solyc03g1	2.22	1.15
GO:00528	NADHX e	Molecular	0.14	0.39	Solyc03g1	Solyc03g1	2.22	1.15
GO:00528	NADPHX	Molecular	0.14	0.39	Solyc03g1	Solyc03g1	2.22	1.15
GO:00098	pollen tube	Biological	0.14	0.39	Solyc05g0	Solyc05g0	2.38	1.25
GO:00092	pyrimidine	Biological	0.14	0.39	Solyc01g0	Solyc01g0	0.32	-1.65
GO:00053	nucleotide	Molecular	0.14	0.39	Solyc12g0	Solyc12g0	5.32	2.41
GO:00093	four-way j	Molecular	0.14	0.39	Solyc01g1	Solyc01g1	2.32	1.22
GO:00712	cellular res	Biological	0.14	0.39	Solyc01g1	Solyc01g1	2.32	1.22
GO:00488	specificati	Biological	0.14	0.39	Solyc04g0	Solyc04g0	0.41	-1.29
GO:00315	nucleotide	Cellular C	0.14	0.39	Solyc06g0	Solyc06g0	4.81	2.27
GO:00421	cellular res	Biological	0.14	0.39	Solyc06g0	Solyc06g0	4.81	2.27
GO:00046	phosphatid	Molecular	0.14	0.39	Solyc06g0	Solyc06g0	0.41	-1.30
GO:00088	formyltetra	Molecular	0.14	0.39	Solyc06g0	Solyc06g0	0.41	-1.30
GO:00165	protein aut	Biological	0.14	0.39	Solyc06g0	Solyc06g0	0.41	-1.30
GO:00182	peptidyl-m	Biological	0.14	0.39	Solyc07g0	PDF1A	4.20	2.07
GO:00425	peptide de	Molecular	0.14	0.39	Solyc07g0	PDF1A	4.20	2.07
GO:00324	regulation	Biological	0.14	0.39	Solyc08g0	Solyc08g0	5.40	2.43
GO:00001	DNA stran	Molecular	0.14	0.39	Solyc09g0	Solyc09g0	0.35	-1.51
GO:00162	4-coumara	Molecular	0.14	0.39	Solyc03g0	Solyc03g0	0.18	-2.45
GO:00313	integral co	Cellular C	0.14	0.39	Solyc05g0	Solyc05g0	0.08	-3.58
GO:00726	mitotic spi	Cellular C	0.14	0.39	Solyc01g1	Solyc01g1	2.18	1.13
GO:00046	proline del	Molecular	0.14	0.39	Solyc02g0	Solyc02g0	3.06	1.62
GO:00065	proline cat	Biological	0.14	0.39	Solyc02g0	Solyc02g0	3.06	1.62
GO:00101	proline cat	Biological	0.14	0.39	Solyc02g0	Solyc02g0	3.06	1.62
GO:00058	cytoplasmi	Cellular C	0.14	0.39	Solyc02g0	Solyc02g0	2.08	1.06
GO:00096	thigmotrop	Biological	0.14	0.39	Solyc02g0	Solyc02g0	2.08	1.06
GO:00311	regulation	Biological	0.14	0.39	Solyc02g0	Solyc02g0	2.08	1.06
GO:00353	microtubul	Cellular C	0.14	0.39	Solyc02g0	Solyc02g0	2.08	1.06
GO:00353	protein loc	Biological	0.14	0.39	Solyc02g0	Solyc02g0	2.08	1.06
GO:19048	protein loc	Biological	0.14	0.39	Solyc02g0	Solyc02g0	2.08	1.06
GO:00479	glutamine	Molecular	0.14	0.39	Solyc09g0	Solyc09g0	2.71	1.44
GO:00454	chalcone is	Molecular	0.14	0.39	Solyc05g0	Solyc05g0	3.96	1.99
GO:00040	aldehyde o	Molecular	0.14	0.39	Solyc11g0	Solyc11g0	0.32	-1.67
GO:00102	photosyste	Biological	0.14	0.39	Solyc07g0	PSBR	3.05	1.61
GO:00046	phosphom	Molecular	0.14	0.39	Solyc06g0	Solyc06g0	2.33	1.22
GO:00700	oligopeptic	Molecular	0.14	0.39	Solyc04g0	Solyc04g0	0.43	-1.23
GO:00700	rRNA (uri	Molecular	0.14	0.39	Solyc07g0	Solyc07g0	0.40	-1.31
GO:00168	racemase a	Molecular	0.14	0.39	Solyc03g1	Solyc03g1	0.42	-1.26
GO:20012	malonyl-C	Biological	0.14	0.39	Solyc04g0	Solyc04g0	0.00	-13.91
GO:19906	acetolactat	Molecular	0.14	0.39	Solyc11g0	Solyc11g0	2.27	1.18
GO:00485	long-day p	Biological	0.14	0.39	Solyc03g0	Solyc03g0	0.49	-1.02
GO:00430	peptide bic	Biological	0.14	0.39	Solyc10g0	Solyc10g0	2.10	1.07
GO:00340	8-oxo-7,8-	Molecular	0.14	0.39	Solyc09g0	Solyc09g0	2.14	1.10
GO:00087	arginine de	Molecular	0.14	0.39	Solyc10g0	Solyc10g0	0.35	-1.51
GO:00040	amidophos	Molecular	0.14	0.39	Solyc01g1	Solyc01g1	2993.63	11.55

GO:00084	8-oxo-7,8-	Molecular	0.14	0.39	Solyc03g0	Solyc03g0	0.02	-5.67
GO:00191	dihydrone	Molecular	0.14	0.39	Solyc03g0	Solyc03g0	0.02	-5.67
GO:00008	inositol he	Molecular	0.14	0.39	Solyc03g0	Solyc03g0	2.02	1.02
GO:00484	stamen de	Biological	0.14	0.39	Solyc02g0	Solyc02g0	0.48	-1.05
GO:00161	NAD(P)H	Molecular	0.14	0.39	Solyc01g0	Solyc01g0	3.53	1.82
GO:00327	RNA biosy	Biological	0.14	0.39	Solyc08g0	Solyc08g0	0.03	-5.23
GO:00009	6,7-dimeth	Molecular	0.14	0.39	Solyc08g0	Solyc08g0	2.11	1.08
GO:00093	riboflavin	Cellular C	0.14	0.39	Solyc08g0	Solyc08g0	2.11	1.08
GO:00099	regulation	Biological	0.14	0.39	Solyc07g0	Solyc07g0	5532.77	12.43
GO:00092	menaquinc	Biological	0.14	0.39	Solyc05g0	Solyc05g0	2.69	1.43
GO:00081	phosphatid	Molecular	0.14	0.39	Solyc04g0	Solyc04g0	1523.79	10.57
GO:00096	nitrate:pro	Molecular	0.14	0.39	Solyc02g0	Solyc02g0	19.78	4.31
GO:00515	3 iron, 4 s	Molecular	0.14	0.39	Solyc04g0	Solyc04g0	39.10	5.29
GO:00067	cellular ar	Biological	0.14	0.39	Solyc01g0	Solyc01g0	0.12	-3.03
GO:00058	microtubul	Cellular C	0.14	0.39	Solyc01g0	Solyc01g0	0.00	-10.07
GO:20006	regulation	Biological	0.14	0.39	Solyc01g0	Solyc01g0	0.00	-10.07
GO:00068	acyl carnit	Biological	0.14	0.39	Solyc06g0	Solyc06g0	3.28	1.71
GO:00469	3'-phospho	Molecular	0.14	0.39	Solyc08g0	Solyc08g0	6.46	2.69
GO:00162	CoA hydro	Molecular	0.14	0.39	Solyc03g0	Solyc03g0	0.00	-11.43
GO:00615	1,4-dihydr	Molecular	0.14	0.39	Solyc03g0	Solyc03g0	0.00	-11.43
GO:00469	tetrahydro	Biological	0.14	0.39	Solyc07g0	Solyc07g0	2.40	1.26
GO:00061	oxidative p	Biological	0.14	0.39	Solyc11g0	Solyc11g0	0.09	-3.52
GO:00422	peptide bir	Molecular	0.14	0.39	Solyc08g0	Solyc08g0	4.19	2.07
GO:00152	nucleobase	Molecular	0.14	0.39	Solyc06g0	Solyc06g0	0.44	-1.17
GO:00158	adenine tra	Biological	0.14	0.39	Solyc06g0	Solyc06g0	0.44	-1.17
GO:00158	guanine tra	Biological	0.14	0.39	Solyc06g0	Solyc06g0	0.44	-1.17
GO:00478	dimethylal	Molecular	0.14	0.39	Solyc06g0	Solyc06g0	12.77	3.67
GO:00062	regulation	Biological	0.14	0.39	Solyc07g0	Solyc07g0	2.18	1.13
GO:00101	lateral root	Biological	0.14	0.39	Solyc07g0	Solyc07g0	0.39	-1.35
GO:00102	floral orga	Biological	0.14	0.39	Solyc07g0	Solyc07g0	0.39	-1.35
GO:00508	defense res	Biological	0.14	0.39	Solyc07g0	Solyc07g0	0.39	-1.35
GO:00608	leaf absciss	Biological	0.14	0.39	Solyc07g0	Solyc07g0	0.39	-1.35
GO:00092	thiamine d	Biological	0.14	0.39	Solyc07g0	Solyc07g0	7.27	2.86
GO:00426	plastid nuc	Cellular C	0.14	0.39	Solyc01g1	Solyc01g1	0.37	-1.43
GO:00167	acyltransfe	Molecular	0.14	0.40	Solyc05g0	Solyc05g0	0.06	-4.07
GO:00167	acyltransfe	Molecular	0.14	0.40	Solyc09g0	Solyc09g0	0.06	-4.05
GO:00167	acyltransfe	Molecular	0.14	0.40	Solyc04g0	Solyc04g0	0.35	-1.50
GO:00167	acyltransfe	Molecular	0.14	0.40	Solyc07g0	Solyc07g0	0.49	-1.02
GO:00167	acyltransfe	Molecular	0.14	0.40	Solyc09g0	Solyc09g0	2.71	1.44
GO:00167	acyltransfe	Molecular	0.14	0.40	Solyc12g0	Solyc12g0	2.35	1.23
GO:00167	acyltransfe	Molecular	0.14	0.40	Solyc11g0	Solyc11g0	0.38	-1.41
GO:00167	acyltransfe	Molecular	0.14	0.40	Solyc09g0	Solyc09g0	3.37	1.75
GO:00167	acyltransfe	Molecular	0.14	0.40	Solyc07g0	Solyc07g0	0.31	-1.68
GO:00167	acyltransfe	Molecular	0.14	0.40	Solyc11g0	Solyc11g0	292.92	8.19
GO:00046	protein kin	Molecular	0.14	0.41	Solyc06g0	Solyc06g0	7.83	2.97
GO:00046	protein kin	Molecular	0.14	0.41	Solyc09g0	Solyc09g0	2.98	1.58
GO:00046	protein kin	Molecular	0.14	0.41	Solyc09g0	Solyc09g0	0.32	-1.64
GO:00046	protein kin	Molecular	0.14	0.41	Solyc09g0	Solyc09g0	0.23	-2.09
GO:00046	protein kin	Molecular	0.14	0.41	Solyc02g0	Solyc02g0	0.30	-1.71
GO:00046	protein kin	Molecular	0.14	0.41	Solyc01g0	Solyc01g0	3.08	1.62
GO:00046	protein kin	Molecular	0.14	0.41	Solyc09g0	Solyc09g0	0.29	-1.78

GO:00046	protein kin	Molecular	0.14	0.41	Solyc01g1	Solyc01g1	3.76	1.91
GO:00046	protein kin	Molecular	0.14	0.41	Solyc09g0	Solyc09g0	7.25	2.86
GO:00046	protein kin	Molecular	0.14	0.41	Solyc09g0	Solyc09g0	2.40	1.26
GO:00046	protein kin	Molecular	0.14	0.41	Solyc09g0	Solyc09g0	0.25	-2.01
GO:00046	protein kin	Molecular	0.14	0.41	Solyc09g0	Solyc09g0	0.24	-2.04
GO:00046	protein kin	Molecular	0.14	0.41	Solyc01g0	Solyc01g0	0.34	-1.54
GO:00046	protein kin	Molecular	0.14	0.41	Solyc01g1	Solyc01g1	72.82	6.19
GO:00046	protein kin	Molecular	0.14	0.41	Solyc03g0	Solyc03g0	3.18	1.67
GO:00046	protein kin	Molecular	0.14	0.41	Solyc03g0	Solyc03g0	2.16	1.11
GO:00046	protein kin	Molecular	0.14	0.41	Solyc03g0	Solyc03g0	7.56	2.92
GO:00046	protein kin	Molecular	0.14	0.41	Solyc02g0	Solyc02g0	0.44	-1.19
GO:00046	protein kin	Molecular	0.14	0.41	Solyc02g0	Solyc02g0	0.38	-1.41
GO:00046	protein kin	Molecular	0.14	0.41	Solyc04g0	Solyc04g0	0.43	-1.23
GO:00046	protein kin	Molecular	0.14	0.41	Solyc04g0	Solyc04g0	0.41	-1.29
GO:00046	protein kin	Molecular	0.14	0.41	Solyc02g0	Solyc02g0	0.50	-1.00
GO:00046	protein kin	Molecular	0.14	0.41	Solyc06g0	Solyc06g0	0.37	-1.44
GO:00046	protein kin	Molecular	0.14	0.41	Solyc09g0	Solyc09g0	0.37	-1.43
GO:00046	protein kin	Molecular	0.14	0.41	Solyc05g0	Solyc05g0	0.50	-1.00
GO:00046	protein kin	Molecular	0.14	0.41	Solyc11g0	Solyc11g0	6.63	2.73
GO:00046	protein kin	Molecular	0.14	0.41	Solyc06g0	Solyc06g0	3.42	1.77
GO:00046	protein kin	Molecular	0.14	0.41	Solyc07g0	Solyc07g0	2.15	1.11
GO:00046	protein kin	Molecular	0.14	0.41	Solyc05g0	Solyc05g0	0.43	-1.22
GO:00046	protein kin	Molecular	0.14	0.41	Solyc01g0	Solyc01g0	2.56	1.36
GO:00046	protein kin	Molecular	0.14	0.41	Solyc03g1	Solyc03g1	0.49	-1.04
GO:00046	protein kin	Molecular	0.14	0.41	Solyc11g0	Solyc11g0	0.46	-1.13
GO:00046	protein kin	Molecular	0.14	0.41	Solyc11g0	Solyc11g0	2.57	1.36
GO:00046	protein kin	Molecular	0.14	0.41	Solyc11g0	Solyc11g0	2.78	1.48
GO:00046	protein kin	Molecular	0.14	0.41	Solyc12g0	Solyc12g0	2.20	1.14
GO:00046	protein kin	Molecular	0.14	0.41	Solyc10g0	Solyc10g0	0.08	-3.68
GO:00046	protein kin	Molecular	0.14	0.41	Solyc06g0	Solyc06g0	0.15	-2.77
GO:00046	protein kin	Molecular	0.14	0.41	Solyc01g0	Solyc01g0	0.41	-1.30
GO:00046	protein kin	Molecular	0.14	0.41	Solyc11g0	Solyc11g0	2.24	1.16
GO:00046	protein kin	Molecular	0.14	0.41	Solyc04g0	Solyc04g0	3.70	1.89
GO:00046	protein kin	Molecular	0.14	0.41	Solyc12g0	Solyc12g0	0.41	-1.28
GO:00046	protein kin	Molecular	0.14	0.41	Solyc06g0	Solyc06g0	0.43	-1.23
GO:00046	protein kin	Molecular	0.14	0.41	Solyc04g0	Solyc04g0	0.46	-1.11
GO:00046	protein kin	Molecular	0.14	0.41	Solyc08g0	Solyc08g0	0.50	-1.00
GO:00046	protein kin	Molecular	0.14	0.41	Solyc10g0	Solyc10g0	4.22	2.08
GO:00046	protein kin	Molecular	0.14	0.41	Solyc04g0	Solyc04g0	0.39	-1.37
GO:00046	protein kin	Molecular	0.14	0.41	Solyc08g0	Solyc08g0	0.00	-10.00
GO:00046	protein kin	Molecular	0.14	0.41	Solyc07g0	Solyc07g0	1180.56	10.21
GO:00046	protein kin	Molecular	0.14	0.41	Solyc06g0	Solyc06g0	0.37	-1.44
GO:00046	protein kin	Molecular	0.14	0.41	Solyc04g0	Solyc04g0	0.44	-1.18
GO:00046	protein kin	Molecular	0.14	0.41	Solyc03g0	Solyc03g0	0.49	-1.03
GO:00046	protein kin	Molecular	0.14	0.41	Solyc03g0	Solyc03g0	2.40	1.26
GO:00046	protein kin	Molecular	0.14	0.41	Solyc01g0	Solyc01g0	5.28	2.40
GO:00046	protein kin	Molecular	0.14	0.41	Solyc07g0	Solyc07g0	0.47	-1.10
GO:00046	protein kin	Molecular	0.14	0.41	Solyc12g0	Solyc12g0	3.71	1.89
GO:00046	protein kin	Molecular	0.14	0.41	Solyc06g0	Solyc06g0	912.98	9.83
GO:00046	protein kin	Molecular	0.14	0.41	Solyc04g0	Solyc04g0	0.18	-2.51
GO:00046	protein kin	Molecular	0.14	0.41	Solyc08g0	Solyc08g0	0.41	-1.30

GO:00046	protein kin	Molecular	0.14	0.41	Solyc02g0	Solyc02g0	307.12	8.26
GO:00046	protein kin	Molecular	0.14	0.41	Solyc02g0	Solyc02g0	2.27	1.18
GO:00046	protein kin	Molecular	0.14	0.41	Solyc05g0	Solyc05g0	0.46	-1.13
GO:00046	protein kin	Molecular	0.14	0.41	Solyc03g1	Solyc03g1	0.06	-4.15
GO:00046	protein kin	Molecular	0.14	0.41	Solyc01g1	Solyc01g1	18.01	4.17
GO:00046	protein kin	Molecular	0.14	0.41	Solyc02g0	Solyc02g0	0.17	-2.58
GO:00046	protein kin	Molecular	0.14	0.41	Solyc07g0	Solyc07g0	2.39	1.26
GO:00046	protein kin	Molecular	0.14	0.41	Solyc08g0	Solyc08g0	2.56	1.36
GO:00046	protein kin	Molecular	0.14	0.41	Solyc01g0	Solyc01g0	0.00	-9.25
GO:00046	protein kin	Molecular	0.14	0.41	Solyc07g0	Solyc07g0	2.77	1.47
GO:00046	protein kin	Molecular	0.14	0.41	Solyc09g0	Solyc09g0	0.34	-1.58
GO:00046	protein kin	Molecular	0.14	0.41	Solyc03g1	Solyc03g1	356.83	8.48
GO:00046	protein kin	Molecular	0.14	0.41	Solyc12g0	Solyc12g0	0.00	-11.51
GO:00046	protein kin	Molecular	0.14	0.41	Solyc01g0	Solyc01g0	0.00	-9.77
GO:00046	protein kin	Molecular	0.14	0.41	Solyc07g0	Solyc07g0	770.05	9.59
GO:00046	protein kin	Molecular	0.14	0.41	Solyc08g0	Solyc08g0	4.19	2.07
GO:00046	protein kin	Molecular	0.14	0.41	Solyc02g0	Solyc02g0	394.05	8.62
GO:00046	protein kin	Molecular	0.14	0.41	Solyc02g0	Solyc02g0	0.07	-3.78
GO:00046	protein kin	Molecular	0.14	0.41	Solyc03g0	Solyc03g0	0.36	-1.46
GO:00046	protein kin	Molecular	0.14	0.41	Solyc07g0	Solyc07g0	2.88	1.52
GO:00046	protein kin	Molecular	0.14	0.41	Solyc02g0	Solyc02g0	517.85	9.02
GO:00046	protein kin	Molecular	0.14	0.41	Solyc05g0	Solyc05g0	0.31	-1.68
GO:00046	protein kin	Molecular	0.14	0.41	Solyc08g0	Solyc08g0	0.11	-3.25
GO:00046	protein kin	Molecular	0.14	0.41	Solyc06g0	Solyc06g0	9.89	3.31
GO:00046	protein kin	Molecular	0.14	0.41	Solyc02g0	Solyc02g0	960.34	9.91
GO:00046	protein kin	Molecular	0.14	0.41	Solyc07g0	Solyc07g0	0.39	-1.35
GO:00046	protein kin	Molecular	0.14	0.41	Solyc03g0	Solyc03g0	0.39	-1.37
GO:00046	protein kin	Molecular	0.14	0.41	Solyc11g0	Solyc11g0	2.48	1.31
GO:00046	protein kin	Molecular	0.14	0.41	Solyc01g0	Solyc01g0	2.50	1.32
GO:00046	protein kin	Molecular	0.14	0.41	Solyc12g0	Solyc12g0	11.23	3.49
GO:00046	protein kin	Molecular	0.14	0.41	Solyc03g0	Solyc03g0	0.10	-3.29
GO:00046	protein kin	Molecular	0.14	0.41	Solyc11g0	Solyc11g0	0.22	-2.20
GO:00159	ATP synth	Biological	0.14	0.41	Solyc12g0	Solyc12g0	2.97	1.57
GO:00159	ATP synth	Biological	0.14	0.41	Solyc06g0	Solyc06g0	2.29	1.20
GO:00159	ATP synth	Biological	0.14	0.41	Solyc10g0	Solyc10g0	0.03	-5.05
GO:00159	ATP synth	Biological	0.14	0.41	Solyc06g0	Solyc06g0	15.51	3.95
GO:00159	ATP synth	Biological	0.14	0.41	Solyc11g0	Solyc11g0	0.22	-2.19
GO:00057	vacuolar m	Cellular C	0.15	0.42	Solyc07g0	Solyc07g0	6.73	2.75
GO:00057	vacuolar m	Cellular C	0.15	0.42	Solyc04g0	Solyc04g0	2.70	1.43
GO:00057	vacuolar m	Cellular C	0.15	0.42	Solyc05g0	Solyc05g0	0.44	-1.19
GO:00057	vacuolar m	Cellular C	0.15	0.42	Solyc01g0	Solyc01g0	2.02	1.02
GO:00057	vacuolar m	Cellular C	0.15	0.42	Solyc10g0	Solyc10g0	1973.43	10.95
GO:00057	vacuolar m	Cellular C	0.15	0.42	Solyc03g0	Solyc03g0	0.31	-1.69
GO:00057	vacuolar m	Cellular C	0.15	0.42	Solyc04g0	Solyc04g0	3.05	1.61
GO:00057	vacuolar m	Cellular C	0.15	0.42	Solyc07g0	Solyc07g0	2327.11	11.18
GO:00057	vacuolar m	Cellular C	0.15	0.42	Solyc07g0	Solyc07g0	0.40	-1.33
GO:00057	vacuolar m	Cellular C	0.15	0.42	Solyc06g0	Solyc06g0	15.51	3.95
GO:00044	N,N-dimet	Molecular	0.15	0.42	Solyc01g1	Solyc01g1	11.39	3.51
GO:00044	N,N-dimet	Molecular	0.15	0.42	Solyc06g0	Solyc06g0	0.32	-1.66
GO:00044	N,N-dimet	Molecular	0.15	0.42	Solyc06g0	Solyc06g0	3.53	1.82
GO:00048	endopeptic	Molecular	0.15	0.42	Solyc03g0	Solyc03g0	3.54	1.82

GO:00048	endopeptic	Molecular	0.15	0.42	Solyc11g0	Solyc11g0	0.07	-3.83
GO:00048	endopeptic	Molecular	0.15	0.42	Solyc07g0	Solyc07g0	3.98	1.99
GO:00197	carboxylic	Biological	0.15	0.43	Solyc08g0	Solyc08g0	2.65	1.41
GO:00197	carboxylic	Biological	0.15	0.43	Solyc11g0	Solyc11g0	0.50	-1.01
GO:00197	carboxylic	Biological	0.15	0.43	Solyc08g0	Solyc08g0	3785.25	11.89
GO:00197	carboxylic	Biological	0.15	0.43	Solyc04g0	Solyc04g0	1711.14	10.74
GO:00197	carboxylic	Biological	0.15	0.43	Solyc08g0	Solyc08g0	0.13	-2.97
GO:00105	green leaf	Biological	0.15	0.43	Solyc01g0	Solyc01g0	2.18	1.13
GO:00105	green leaf	Biological	0.15	0.43	Solyc06g0	Solyc06g0	0.48	-1.05
GO:00105	green leaf	Biological	0.15	0.43	Solyc01g0	Solyc01g0	0.10	-3.35
GO:00105	green leaf	Biological	0.15	0.43	Solyc02g0	Solyc02g0	0.06	-4.15
GO:00105	green leaf	Biological	0.15	0.43	Solyc10g0	Solyc10g0	0.26	-1.94
GO:00096	ethylene bi	Biological	0.16	0.43	Solyc07g0	ACO1	2.82	1.49
GO:00096	ethylene bi	Biological	0.16	0.43	Solyc02g0	Solyc02g0	2.56	1.36
GO:00065	cysteine bi	Biological	0.16	0.43	Solyc01g0	Solyc01g0	2.46	1.30
GO:00065	cysteine bi	Biological	0.16	0.43	Solyc01g0	Solyc01g0	2.88	1.53
GO:00039	NAD(P)H	Molecular	0.16	0.43	Solyc03g0	Solyc03g0	5.61	2.49
GO:00039	NAD(P)H	Molecular	0.16	0.43	Solyc03g0	Solyc03g0	2.10	1.07
GO:00038	3-beta-hyd	Molecular	0.16	0.43	Solyc02g0	Solyc02g0	0.30	-1.71
GO:00038	3-beta-hyd	Molecular	0.16	0.43	Solyc04g0	Solyc04g0	2.31	1.21
GO:00339	glycopepti	Molecular	0.16	0.43	Solyc11g0	Solyc11g0	2.94	1.56
GO:00339	glycopepti	Molecular	0.16	0.43	Solyc01g1	Solyc01g1	0.00	-8.99
GO:00061	'de novo' I	Biological	0.16	0.43	Solyc06g0	Solyc06g0	0.41	-1.30
GO:00061	'de novo' I	Biological	0.16	0.43	Solyc01g1	Solyc01g1	2993.63	11.55
GO:00047	superoxide	Molecular	0.16	0.43	Solyc01g0	Solyc01g0	0.42	-1.26
GO:00047	superoxide	Molecular	0.16	0.43	Solyc06g0	Solyc06g0	17.92	4.16
GO:00426	thylakoid r	Cellular C	0.16	0.43	Solyc07g0	PSBR	3.05	1.61
GO:00426	thylakoid r	Cellular C	0.16	0.43	Solyc03g1	Solyc03g1	0.10	-3.25
GO:00302	dynein con	Cellular C	0.16	0.43	Solyc07g0	Solyc07g0	3.21	1.68
GO:00302	dynein con	Cellular C	0.16	0.43	Solyc03g1	Solyc03g1	2.39	1.26
GO:00300	cellular ma	Biological	0.16	0.43	Solyc04g0	Solyc04g0	0.33	-1.58
GO:00300	cellular ma	Biological	0.16	0.43	Solyc04g0	Solyc04g0	3.05	1.61
GO:00301	protein cat	Biological	0.16	0.43	Solyc05g0	Solyc05g0	0.36	-1.47
GO:00301	protein cat	Biological	0.16	0.43	Solyc11g0	Solyc11g0	0.29	-1.80
GO:00301	protein cat	Biological	0.16	0.43	Solyc10g0	Solyc10g0	2.35	1.23
GO:00301	protein cat	Biological	0.16	0.43	Solyc09g0	Solyc09g0	0.41	-1.27
GO:00301	protein cat	Biological	0.16	0.43	Solyc05g0	Solyc05g0	4.45	2.15
GO:00301	protein cat	Biological	0.16	0.43	Solyc01g0	Solyc01g0	0.46	-1.12
GO:00301	protein cat	Biological	0.16	0.43	Solyc01g0	Solyc01g0	19.06	4.25
GO:00301	protein cat	Biological	0.16	0.43	Solyc09g0	Solyc09g0	5.06	2.34
GO:00427	defense res	Biological	0.18	0.45	Solyc12g0	Solyc12g0	2.97	1.57
GO:00427	defense res	Biological	0.18	0.45	Solyc01g1	Solyc01g1	0.44	-1.20
GO:00427	defense res	Biological	0.18	0.45	Solyc12g0	Solyc12g0	2.10	1.07
GO:00427	defense res	Biological	0.18	0.45	Solyc10g0	Solyc10g0	4.22	2.08
GO:00427	defense res	Biological	0.18	0.45	Solyc01g0	Solyc01g0	0.31	-1.69
GO:00427	defense res	Biological	0.18	0.45	Solyc08g0	Solyc08g0	4.19	2.07
GO:00168	carbon-car	Molecular	0.18	0.45	Solyc11g0	Solyc11g0	0.50	-1.01
GO:00168	carbon-car	Molecular	0.18	0.45	Solyc08g0	Solyc08g0	3785.25	11.89
GO:00168	carbon-car	Molecular	0.18	0.45	Solyc04g0	Solyc04g0	1711.14	10.74
GO:00168	carbon-car	Molecular	0.18	0.45	Solyc08g0	Solyc08g0	0.13	-2.97
GO:00033	amino acid	Biological	0.18	0.45	Solyc03g0	Solyc03g0	0.24	-2.04

GO:00033	amino acid	Biological	0.18	0.45	Solyc10g0	Solyc10g0	1973.43	10.95
GO:00033	amino acid	Biological	0.18	0.45	Solyc03g0	Solyc03g0	0.31	-1.69
GO:00033	amino acid	Biological	0.18	0.45	Solyc01g1	Solyc01g1	0.20	-2.35
GO:00033	amino acid	Biological	0.18	0.45	Solyc06g0	Solyc06g0	0.15	-2.76
GO:00168	carboxy-ly	Molecular	0.18	0.45	Solyc11g0	Solyc11g0	0.50	-1.01
GO:00168	carboxy-ly	Molecular	0.18	0.45	Solyc06g0	Solyc06g0	0.41	-1.30
GO:00168	carboxy-ly	Molecular	0.18	0.45	Solyc08g0	Solyc08g0	3785.25	11.89
GO:00168	carboxy-ly	Molecular	0.18	0.45	Solyc10g0	Solyc10g0	0.35	-1.51
GO:00168	carboxy-ly	Molecular	0.18	0.45	Solyc08g0	Solyc08g0	0.13	-2.97
GO:00068	cation tran	Biological	0.18	0.45	Solyc01g0	Solyc01g0	0.33	-1.61
GO:00068	cation tran	Biological	0.18	0.45	Solyc04g0	Solyc04g0	0.41	-1.28
GO:00068	cation tran	Biological	0.18	0.45	Solyc10g0	Solyc10g0	17.76	4.15
GO:00068	cation tran	Biological	0.18	0.45	Solyc08g0	Solyc08g0	2.67	1.41
GO:00068	cation tran	Biological	0.18	0.45	Solyc03g0	Solyc03g0	2.16	1.11
GO:00068	cation tran	Biological	0.18	0.45	Solyc06g0	Solyc06g0	2059.41	11.01
GO:00068	cation tran	Biological	0.18	0.45	Solyc02g0	Solyc02g0	412.01	8.69
GO:00168	lyase activ	Molecular	0.18	0.45	Solyc10g0	Solyc10g0	4.26	2.09
GO:00168	lyase activ	Molecular	0.18	0.45	Solyc09g0	Solyc09g0	0.27	-1.91
GO:00168	lyase activ	Molecular	0.18	0.45	Solyc11g0	AOS2	3.20	1.68
GO:00168	lyase activ	Molecular	0.18	0.45	Solyc02g0	Solyc02g0	11.09	3.47
GO:00168	lyase activ	Molecular	0.18	0.45	Solyc01g0	Solyc01g0	0.03	-5.12
GO:00168	lyase activ	Molecular	0.18	0.45	Solyc11g0	Solyc11g0	0.50	-1.01
GO:00168	lyase activ	Molecular	0.18	0.45	Solyc06g0	Solyc06g0	0.41	-1.30
GO:00168	lyase activ	Molecular	0.18	0.45	Solyc02g0	Solyc02g0	4235.90	12.05
GO:00168	lyase activ	Molecular	0.18	0.45	Solyc10g0	Solyc10g0	0.35	-1.51
GO:00168	lyase activ	Molecular	0.18	0.45	Solyc08g0	Solyc08g0	8.90	3.15
GO:00168	lyase activ	Molecular	0.18	0.45	Solyc08g0	Solyc08g0	0.13	-2.97
GO:00168	lyase activ	Molecular	0.18	0.45	Solyc05g0	Solyc05g0	20.96	4.39
GO:00048	triglycerid	Molecular	0.18	0.45	Solyc12g0	Solyc12g0	0.32	-1.65
GO:00048	triglycerid	Molecular	0.18	0.45	Solyc02g0	Solyc02g0	2.24	1.17
GO:00338	ribonuclea	Molecular	0.18	0.45	Solyc07g0	Solyc07g0	6.72	2.75
GO:00338	ribonuclea	Molecular	0.18	0.45	Solyc05g0	Solyc05g0	20.96	4.39
GO:00098	photorespi	Biological	0.18	0.45	Solyc01g0	Solyc01g0	0.03	-5.12
GO:00098	photorespi	Biological	0.18	0.45	Solyc03g0	RBCS-2A	2.71	1.44
GO:00095	photosyste	Cellular C	0.18	0.45	Solyc02g0	Solyc02g0	2.31	1.21
GO:00095	photosyste	Cellular C	0.18	0.45	Solyc06g0	Solyc06g0	4.49	2.17
GO:00060	gluconeog	Biological	0.18	0.45	Solyc10g0	Solyc10g0	2.23	1.16
GO:00060	gluconeog	Biological	0.18	0.45	Solyc09g0	Solyc09g0	25.41	4.67
GO:00092	riboflavin	Biological	0.18	0.45	Solyc11g0	Solyc11g0	7.03	2.81
GO:00092	riboflavin	Biological	0.18	0.45	Solyc08g0	Solyc08g0	2.11	1.08
GO:00610	chaperone-	Biological	0.18	0.45	Solyc09g0	Solyc09g0	4.54	2.18
GO:00610	chaperone-	Biological	0.18	0.45	Solyc09g0	Solyc09g0	0.41	-1.30
GO:00614	positive re	Biological	0.19	0.45	Solyc03g0	Solyc03g0	0.24	-2.06
GO:00614	positive re	Biological	0.19	0.45	Solyc02g0	Solyc02g0	0.42	-1.24
GO:00614	positive re	Biological	0.19	0.45	Solyc11g0	Solyc11g0	0.00	-10.83
GO:00161	sterol bios	Biological	0.19	0.45	Solyc02g0	Solyc02g0	0.50	-1.01
GO:00161	sterol bios	Biological	0.19	0.45	Solyc04g0	Solyc04g0	2.83	1.50
GO:00161	sterol bios	Biological	0.19	0.45	Solyc02g0	Solyc02g0	8.45	3.08
GO:00095	pollen dev	Biological	0.19	0.45	Solyc05g0	Solyc05g0	2.54	1.35
GO:00095	pollen dev	Biological	0.19	0.45	Solyc11g0	Solyc11g0	6976.37	12.77
GO:00095	pollen dev	Biological	0.19	0.45	Solyc11g0	Solyc11g0	18.97	4.25

GO:00057	mitochond	Cellular C	0.19	0.45	Solyc01g0	Solyc01g0	0.32	-1.65
GO:00057	mitochond	Cellular C	0.19	0.45	Solyc06g0	Solyc06g0	0.41	-1.30
GO:00057	mitochond	Cellular C	0.19	0.45	Solyc12g0	Solyc12g0	0.36	-1.46
GO:00057	mitochond	Cellular C	0.19	0.45	Solyc04g0	Solyc04g0	39.10	5.29
GO:00057	mitochond	Cellular C	0.19	0.45	Solyc11g0	Solyc11g0	0.09	-3.52
GO:00057	mitochond	Cellular C	0.19	0.45	Solyc01g0	Solyc01g0	0.00	-11.62
GO:00047	transmemb	Molecular	0.19	0.45	Solyc01g1	Solyc01g1	72.82	6.19
GO:00047	transmemb	Molecular	0.19	0.45	Solyc02g0	Solyc02g0	0.44	-1.19
GO:00047	transmemb	Molecular	0.19	0.45	Solyc07g0	Solyc07g0	1180.56	10.21
GO:00047	transmemb	Molecular	0.19	0.45	Solyc03g0	Solyc03g0	0.49	-1.03
GO:00047	transmemb	Molecular	0.19	0.45	Solyc02g0	Solyc02g0	0.07	-3.78
GO:00082	isoprenoid	Biological	0.19	0.45	Solyc03g0	Solyc03g0	2.38	1.25
GO:00082	isoprenoid	Biological	0.19	0.45	Solyc04g0	Solyc04g0	2.62	1.39
GO:00082	isoprenoid	Biological	0.19	0.45	Solyc02g0	Solyc02g0	0.50	-1.01
GO:00082	isoprenoid	Biological	0.19	0.45	Solyc07g0	Solyc07g0	2.42	1.27
GO:00512	NAD bindi	Molecular	0.19	0.45	Solyc01g1	Solyc01g1	4.98	2.32
GO:00512	NAD bindi	Molecular	0.19	0.45	Solyc09g0	Solyc09g0	0.37	-1.45
GO:00512	NAD bindi	Molecular	0.19	0.45	Solyc04g0	Solyc04g0	8.55	3.10
GO:00512	NAD bindi	Molecular	0.19	0.45	Solyc12g0	Solyc12g0	2.01	1.01
GO:00512	NAD bindi	Molecular	0.19	0.45	Solyc12g0	Solyc12g0	0.45	-1.15
GO:00512	NAD bindi	Molecular	0.19	0.45	Solyc02g0	Solyc02g0	2.01	1.01
GO:00512	NAD bindi	Molecular	0.19	0.45	Solyc10g0	Solyc10g0	0.00	-12.09
GO:00512	NAD bindi	Molecular	0.19	0.45	Solyc01g0	Solyc01g0	0.12	-3.04
GO:00512	NAD bindi	Molecular	0.19	0.45	Solyc02g0	Solyc02g0	2.27	1.18
GO:00082	peptidase a	Molecular	0.19	0.45	Solyc03g1	Solyc03g1	2.16	1.11
GO:00082	peptidase a	Molecular	0.19	0.45	Solyc01g1	Solyc01g1	0.44	-1.20
GO:00082	peptidase a	Molecular	0.19	0.45	Solyc02g0	Solyc02g0	0.34	-1.57
GO:00082	peptidase a	Molecular	0.19	0.45	Solyc09g0	Solyc09g0	0.12	-3.08
GO:00082	peptidase a	Molecular	0.19	0.45	Solyc01g0	Solyc01g0	0.48	-1.06
GO:00082	peptidase a	Molecular	0.19	0.45	Solyc02g0	Solyc02g0	0.48	-1.05
GO:00082	peptidase a	Molecular	0.19	0.45	Solyc12g0	Solyc12g0	1818.26	10.83
GO:00082	peptidase a	Molecular	0.19	0.45	Solyc12g0	Solyc12g0	0.39	-1.35
GO:00082	peptidase a	Molecular	0.19	0.45	Solyc03g0	Solyc03g0	11.28	3.50
GO:00312	arabinan ca	Biological	0.20	0.45	Solyc01g1	Solyc01g1	4.83	2.27
GO:00038	2-isopropy	Molecular	0.20	0.45	Solyc08g0	Solyc08g0	2.65	1.41
GO:00039	acetolactat	Molecular	0.20	0.45	Solyc03g0	Solyc03g0	4.05	2.02
GO:00090	valine bios	Biological	0.20	0.45	Solyc03g0	Solyc03g0	4.05	2.02
GO:00046	phosphogly	Molecular	0.20	0.45	Solyc07g0	Solyc07g0	0.35	-1.51
GO:00800	primary ro	Biological	0.20	0.45	Solyc04g0	Solyc04g0	2.70	1.43
GO:00096	phenylprop	Biological	0.20	0.45	Solyc09g0	Solyc09g0	0.27	-1.91
GO:00044	methenylte	Molecular	0.20	0.45	Solyc01g0	Solyc01g0	2.62	1.39
GO:00194	cyanide me	Biological	0.20	0.45	Solyc01g0	Solyc01g0	2.46	1.30
GO:00038	ATP citrat	Molecular	0.20	0.45	Solyc05g0	Solyc05g0	2.80	1.49
GO:00060	acetyl-CoA	Biological	0.20	0.45	Solyc05g0	Solyc05g0	2.80	1.49
GO:00093	ATP-indep	Cellular C	0.20	0.45	Solyc05g0	Solyc05g0	2.80	1.49
GO:01022	amylopecti	Molecular	0.20	0.45	Solyc09g0	Solyc09g0	0.34	-1.54
GO:00099	allene oxid	Molecular	0.20	0.45	Solyc11g0	AOS2	3.20	1.68
GO:00344	lipid oxida	Biological	0.20	0.45	Solyc08g0	LOX1.1	2.60	1.38
GO:00090	anaerobic r	Biological	0.20	0.45	Solyc02g0	Solyc02g0	3.63	1.86
GO:00062	regulation	Biological	0.20	0.45	Solyc01g0	Solyc01g0	2.56	1.36
GO:00063	DNA catab	Biological	0.20	0.45	Solyc05g0	Solyc05g0	2.32	1.21

GO:00041	citrate (Si)	Molecular	0.20	0.45	Solyc07g0	Solyc07g0	3.93	1.98
GO:00430	ribosomal	Molecular	0.20	0.45	Solyc12g0	Solyc12g0	2.35	1.23
GO:00043	glycolipid	Molecular	0.20	0.45	Solyc01g0	Solyc01g0	0.35	-1.51
GO:00045	beta-glucu	Molecular	0.20	0.45	Solyc06g0	Solyc06g0	2.47	1.31
GO:00442	'de novo' U	Biological	0.20	0.45	Solyc01g0	Solyc01g0	0.32	-1.65
GO:00048	transketola	Molecular	0.20	0.45	Solyc01g0	Solyc01g0	3.54	1.82
GO:00162	AMP bind	Molecular	0.20	0.45	Solyc06g0	Solyc06g0	4.81	2.27
GO:00068	receptor-m	Biological	0.20	0.45	Solyc04g0	Solyc04g0	2.07	1.05
GO:00087	D-alanine-	Molecular	0.20	0.45	Solyc10g0	Solyc10g0	0.43	-1.22
GO:00510	microtubul	Molecular	0.20	0.45	Solyc02g0	Solyc02g0	2.08	1.06
GO:00512	spindle mi	Cellular Co	0.20	0.45	Solyc02g0	Solyc02g0	2.08	1.06
GO:19905	mitochond	Biological	0.20	0.45	Solyc01g0	Solyc01g0	0.42	-1.26
GO:00197	glucosinol	Biological	0.20	0.45	Solyc09g0	Solyc09g0	0.44	-1.19
GO:00045	alpha-man	Molecular	0.20	0.45	Solyc02g0	Solyc02g0	2.97	1.57
GO:00038	gamma-glu	Molecular	0.20	0.45	Solyc11g0	Solyc11g0	2.07	1.05
GO:00093	endopeptic	Cellular Co	0.20	0.45	Solyc09g0	Solyc09g0	0.12	-3.08
GO:00039	GTP cyclo	Molecular	0.20	0.45	Solyc11g0	Solyc11g0	7.03	2.81
GO:00086	3,4-dihydr	Molecular	0.20	0.45	Solyc11g0	Solyc11g0	7.03	2.81
GO:00059	glycogen c	Biological	0.20	0.45	Solyc05g0	Solyc05g0	2.50	1.32
GO:01022	linear malt	Molecular	0.20	0.45	Solyc05g0	Solyc05g0	2.50	1.32
GO:01024	SHG alpha	Molecular	0.20	0.45	Solyc05g0	Solyc05g0	2.50	1.32
GO:00007	condensin	Cellular Co	0.20	0.45	Solyc07g0	Solyc07g0	2.66	1.41
GO:00005	glycosylph	Cellular Co	0.20	0.45	Solyc03g0	Solyc03g0	6.17	2.63
GO:00341	acylglycer	Biological	0.20	0.45	Solyc01g0	Solyc01g0	2.02	1.02
GO:19900	ER to chlo	Biological	0.20	0.45	Solyc01g0	Solyc01g0	2.02	1.02
GO:00053	oxygen car	Molecular	0.20	0.45	Solyc07g0	Solyc07g0	40.33	5.33
GO:00156	oxygen tra	Biological	0.20	0.45	Solyc07g0	Solyc07g0	40.33	5.33
GO:00484	replication	Biological	0.20	0.45	Solyc04g0	Solyc04g0	2.35	1.23
GO:00065	arginine ca	Biological	0.20	0.45	Solyc10g0	Solyc10g0	0.35	-1.51
GO:00088	glyceropho	Molecular	0.20	0.45	Solyc12g0	Solyc12g0	2.04	1.03
GO:00060	inositol me	Biological	0.20	0.45	Solyc04g0	Solyc04g0	0.41	-1.30
GO:00528	inositol mo	Molecular	0.20	0.45	Solyc04g0	Solyc04g0	0.41	-1.30
GO:00528	inositol mo	Molecular	0.20	0.45	Solyc04g0	Solyc04g0	0.41	-1.30
GO:00528	inositol mo	Molecular	0.20	0.45	Solyc04g0	Solyc04g0	0.41	-1.30
GO:00065	valine cata	Biological	0.20	0.45	Solyc02g0	Solyc02g0	2.01	1.01
GO:00160	CDP-diacy	Biological	0.20	0.45	Solyc09g0	Solyc09g0	3.57	1.84
GO:00055	folic acid b	Molecular	0.20	0.45	Solyc07g0	Solyc07g0	3182.84	11.64
GO:00300	plasma me	Cellular Co	0.20	0.45	Solyc09g0	Solyc09g0	0.00	-13.14
GO:00100	xylem and	Biological	0.20	0.45	Solyc04g0	Solyc04g0	0.07	-3.79
GO:00041	dUTP diph	Molecular	0.20	0.45	Solyc01g0	Solyc01g0	2.02	1.02
GO:00062	dUMP bio	Biological	0.20	0.45	Solyc01g0	Solyc01g0	2.02	1.02
GO:00460	dUTP cata	Biological	0.20	0.45	Solyc01g0	Solyc01g0	2.02	1.02
GO:00711	DNA repli	Biological	0.20	0.45	Solyc01g0	Solyc01g0	0.40	-1.32
GO:19021	regulation	Biological	0.20	0.45	Solyc08g0	Solyc08g0	2486.58	11.28
GO:20000	regulation	Biological	0.20	0.45	Solyc08g0	Solyc08g0	2486.58	11.28
GO:00300	cell junctio	Cellular Co	0.20	0.45	Solyc03g0	RBCS-2A	2.71	1.44
GO:00342	protein he	Biological	0.20	0.45	Solyc12g0	Solyc12g0	1818.26	10.83
GO:00515	regulation	Biological	0.20	0.45	Solyc03g1	Solyc03g1	14.48	3.86
GO:00044	malate syn	Molecular	0.20	0.45	Solyc03g1	Solyc03g1	6.29	2.65
GO:00060	glyoxylate	Biological	0.20	0.45	Solyc03g1	Solyc03g1	6.29	2.65
GO:00084	dTDP-gluc	Molecular	0.20	0.45	Solyc07g0	Solyc07g0	0.14	-2.88

GO:00092	nucleotide-	Biological	0.20	0.45	Solyc07g0	Solyc07g0	0.14	-2.88
GO:00081	succinate c	Molecular	0.20	0.45	Solyc04g0	Solyc04g0	39.10	5.29
GO:00103	seed oilboc	Biological	0.20	0.45	Solyc12g0	Solyc12g0	14.44	3.85
GO:00331	proton-trar	Cellular Co	0.20	0.45	Solyc06g0	Solyc06g0	15.51	3.95
GO:00095	preprophas	Cellular Co	0.20	0.45	Solyc01g0	Solyc01g0	0.00	-10.07
GO:00099	regulation	Biological	0.20	0.45	Solyc08g0	Solyc08g0	0.34	-1.54
GO:00723	obsolete cl	Biological	0.20	0.45	Solyc03g0	Solyc03g0	2.19	1.13
GO:00082	omega pep	Molecular	0.20	0.45	Solyc07g0	Solyc07g0	2.40	1.26
GO:00347	gamma-glu	Molecular	0.20	0.45	Solyc07g0	Solyc07g0	2.40	1.26
GO:00702	peptidyl-se	Biological	0.20	0.45	Solyc06g0	Solyc06g0	10.22	3.35
GO:19905	potassium	Biological	0.20	0.45	Solyc12g0	Solyc12g0	369.30	8.53
GO:00477	arogenate (	Molecular	0.20	0.45	Solyc02g0	Solyc02g0	574.97	9.17
GO:00325	adenyl ribo	Molecular	0.20	0.45	Solyc11g0	Solyc11g0	0.22	-2.19
GO:00015	regulation	Biological	0.20	0.45	Solyc08g0	Solyc08g0	4.19	2.07
GO:00513	regulation	Biological	0.20	0.45	Solyc08g0	Solyc08g0	4.19	2.07
GO:00703	cellular he	Biological	0.20	0.45	Solyc08g0	Solyc08g0	4.19	2.07
GO:00166	protochloro	Molecular	0.20	0.45	Solyc12g0	Solyc12g0	6.07	2.60
GO:00482	Casparian	Cellular Co	0.20	0.45	Solyc10g0	Solyc10g0	2.16	1.11
GO:00104	thermosper	Molecular	0.20	0.45	Solyc08g0	Solyc08g0	0.00	-9.72
GO:00082	lipid bindi	Molecular	0.20	0.46	Solyc05g0	Solyc05g0	5.88	2.56
GO:00082	lipid bindi	Molecular	0.20	0.46	Solyc08g0	Solyc08g0	4.70	2.23
GO:00082	lipid bindi	Molecular	0.20	0.46	Solyc03g1	Solyc03g1	0.48	-1.06
GO:00082	lipid bindi	Molecular	0.20	0.46	Solyc01g0	Solyc01g0	44322.86	15.44
GO:00082	lipid bindi	Molecular	0.20	0.46	Solyc07g0	Solyc07g0	4759.57	12.22
GO:00082	lipid bindi	Molecular	0.20	0.46	Solyc08g0	Solyc08g0	0.38	-1.39
GO:00082	lipid bindi	Molecular	0.20	0.46	Solyc10g0	Solyc10g0	2.26	1.18
GO:00082	lipid bindi	Molecular	0.20	0.46	Solyc07g0	Solyc07g0	6.31	2.66
GO:00082	lipid bindi	Molecular	0.20	0.46	Solyc07g0	Solyc07g0	3.55	1.83
GO:00082	lipid bindi	Molecular	0.20	0.46	Solyc02g0	Solyc02g0	2.33	1.22
GO:00082	lipid bindi	Molecular	0.20	0.46	Solyc03g0	Solyc03g0	15.71	3.97
GO:00082	lipid bindi	Molecular	0.20	0.46	Solyc08g0	Solyc08g0	3.40	1.77
GO:00082	lipid bindi	Molecular	0.20	0.46	Solyc09g0	Solyc09g0	783.34	9.61
GO:00037	cytoskeleta	Molecular	0.21	0.46	Solyc10g0	Solyc10g0	2.78	1.48
GO:00037	cytoskeleta	Molecular	0.21	0.46	Solyc07g0	Solyc07g0	3.21	1.68
GO:00037	cytoskeleta	Molecular	0.21	0.46	Solyc03g1	Solyc03g1	2.39	1.26
GO:00099	regulation	Biological	0.21	0.46	Solyc07g0	Solyc07g0	2.02	1.02
GO:00099	regulation	Biological	0.21	0.46	Solyc01g0	Solyc01g0	0.21	-2.26
GO:00099	regulation	Biological	0.21	0.46	Solyc02g0	Solyc02g0	0.50	-1.00
GO:00152	solute:prot	Molecular	0.21	0.47	Solyc04g0	Solyc04g0	0.41	-1.28
GO:00152	solute:prot	Molecular	0.21	0.47	Solyc10g0	Solyc10g0	17.76	4.15
GO:00152	solute:prot	Molecular	0.21	0.47	Solyc03g0	Solyc03g0	2.16	1.11
GO:00152	solute:prot	Molecular	0.21	0.47	Solyc02g0	Solyc02g0	412.01	8.69
GO:00082	positive re	Biological	0.21	0.47	Solyc05g0	Solyc05g0	0.22	-2.15
GO:00082	positive re	Biological	0.21	0.47	Solyc04g0	Solyc04g0	0.41	-1.28
GO:00082	positive re	Biological	0.21	0.47	Solyc03g0	Solyc03g0	4.18	2.06
GO:00082	positive re	Biological	0.21	0.47	Solyc02g0	Solyc02g0	4.91	2.30
GO:00169	ribulose-bi	Molecular	0.21	0.47	Solyc01g0	Solyc01g0	0.03	-5.12
GO:00169	ribulose-bi	Molecular	0.21	0.47	Solyc03g0	RBCS-2A	2.71	1.44
GO:20000	regulation	Biological	0.21	0.47	Solyc09g0	Solyc09g0	0.48	-1.06
GO:20000	regulation	Biological	0.21	0.47	Solyc01g0	Solyc01g0	0.43	-1.23
GO:00101	response to	Biological	0.21	0.47	Solyc02g0	Solyc02g0	0.45	-1.14

GO:00101	response tc	Biological	0.21	0.47	Solyc05g0	Solyc05g0	39.90	5.32
GO:00081	ferrous iro	Molecular	0.21	0.47	Solyc03g0	Solyc03g0	0.22	-2.16
GO:00081	ferrous iro	Molecular	0.21	0.47	Solyc01g0	Solyc01g0	0.12	-3.03
GO:00191	DNA N-gl	Molecular	0.21	0.47	Solyc09g0	Solyc09g0	2.14	1.10
GO:00191	DNA N-gl	Molecular	0.21	0.47	Solyc09g0	Solyc09g0	2.29	1.20
GO:00066	phospholip	Biological	0.21	0.47	Solyc02g0	Solyc02g0	3.57	1.83
GO:00066	phospholip	Biological	0.21	0.47	Solyc04g0	Solyc04g0	1523.79	10.57
GO:00080	growth fac	Molecular	0.21	0.47	Solyc02g0	Solyc02g0	13.99	3.81
GO:00080	growth fac	Molecular	0.21	0.47	Solyc11g0	Solyc11g0	2317.83	11.18
GO:00072	multicellul	Biological	0.21	0.47	Solyc11g0	Solyc11g0	0.22	-2.21
GO:00072	multicellul	Biological	0.21	0.47	Solyc07g0	Solyc07g0	7.42	2.89
GO:00072	multicellul	Biological	0.21	0.47	Solyc05g0	Solyc05g0	2.81	1.49
GO:00072	multicellul	Biological	0.21	0.47	Solyc11g0	Solyc11g0	18.46	4.21
GO:00072	multicellul	Biological	0.21	0.47	Solyc02g0	Solyc02g0	0.48	-1.05
GO:00072	multicellul	Biological	0.21	0.47	Solyc08g0	Solyc08g0	2486.58	11.28
GO:00072	multicellul	Biological	0.21	0.47	Solyc03g0	Solyc03g0	794.30	9.63
GO:00072	multicellul	Biological	0.21	0.47	Solyc03g0	Solyc03g0	0.44	-1.17
GO:00072	multicellul	Biological	0.21	0.47	Solyc05g0	Solyc05g0	3.77	1.92
GO:00515	4 iron, 4 su	Molecular	0.21	0.47	Solyc12g0	Solyc12g0	0.00	-11.26
GO:00515	4 iron, 4 su	Molecular	0.21	0.47	Solyc05g0	Solyc05g0	2.96	1.57
GO:00515	4 iron, 4 su	Molecular	0.21	0.47	Solyc03g0	Solyc03g0	0.11	-3.15
GO:00515	4 iron, 4 su	Molecular	0.21	0.47	Solyc06g0	Solyc06g0	0.05	-4.46
GO:00515	4 iron, 4 su	Molecular	0.21	0.47	Solyc04g0	Solyc04g0	39.10	5.29
GO:00515	4 iron, 4 su	Molecular	0.21	0.47	Solyc09g0	Solyc09g0	2.29	1.20
GO:00515	4 iron, 4 su	Molecular	0.21	0.47	Solyc10g0	Solyc10g0	2.16	1.11
GO:00096	response tc	Biological	0.21	0.47	Solyc07g0	ACO1	2.82	1.49
GO:00096	response tc	Biological	0.21	0.47	Solyc04g0	Solyc04g0	2.70	1.43
GO:00096	response tc	Biological	0.21	0.47	Solyc10g0	Solyc10g0	0.09	-3.53
GO:00096	response tc	Biological	0.21	0.47	Solyc09g0	Solyc09g0	3.41	1.77
GO:00096	response tc	Biological	0.21	0.47	Solyc02g0	Solyc02g0	2.56	1.36
GO:00096	response tc	Biological	0.21	0.47	Solyc02g0	Solyc02g0	2.46	1.30
GO:00096	response tc	Biological	0.21	0.47	Solyc12g0	Solyc12g0	369.30	8.53
GO:00096	response tc	Biological	0.21	0.47	Solyc01g0	Solyc01g0	550.66	9.11
GO:00469	protein het	Molecular	0.22	0.48	Solyc01g0	Solyc01g0	2.68	1.42
GO:00469	protein het	Molecular	0.22	0.48	Solyc01g0	Solyc01g0	3.72	1.90
GO:00469	protein het	Molecular	0.22	0.48	Solyc02g0	Solyc02g0	2.01	1.01
GO:00469	protein het	Molecular	0.22	0.48	Solyc06g0	Solyc06g0	2.67	1.41
GO:00469	protein het	Molecular	0.22	0.48	Solyc04g0	Solyc04g0	2.47	1.30
GO:00469	protein het	Molecular	0.22	0.48	Solyc11g0	Solyc11g0	2.31	1.21
GO:00469	protein het	Molecular	0.22	0.48	Solyc01g0	Solyc01g0	2.37	1.24
GO:00469	protein het	Molecular	0.22	0.48	Solyc06g0	Solyc06g0	2.48	1.31
GO:00469	protein het	Molecular	0.22	0.48	Solyc01g0	Solyc01g0	3.17	1.66
GO:00469	protein het	Molecular	0.22	0.48	Solyc01g0	Solyc01g0	2.30	1.20
GO:00469	protein het	Molecular	0.22	0.48	Solyc12g0	Solyc12g0	2.41	1.27
GO:00469	protein het	Molecular	0.22	0.48	Solyc05g0	Solyc05g0	2.76	1.47
GO:00469	protein het	Molecular	0.22	0.48	Solyc01g0	Solyc01g0	2.57	1.36
GO:00469	protein het	Molecular	0.22	0.48	Solyc06g0	Solyc06g0	0.00	-12.03
GO:00082	cysteine-ty	Molecular	0.22	0.49	Solyc03g1	Solyc03g1	2.16	1.11
GO:00082	cysteine-ty	Molecular	0.22	0.49	Solyc04g0	Solyc04g0	2.20	1.14
GO:00082	cysteine-ty	Molecular	0.22	0.49	Solyc02g0	Solyc02g0	0.34	-1.57
GO:00082	cysteine-ty	Molecular	0.22	0.49	Solyc05g0	Solyc05g0	2.06	1.05

GO:00082	cysteine-ty	Molecular	0.22	0.49	Solyc12g0	Solyc12g0	2.97	1.57
GO:00082	cysteine-ty	Molecular	0.22	0.49	Solyc12g0	Solyc12g0	0.39	-1.35
GO:00082	cysteine-ty	Molecular	0.22	0.49	Solyc10g0	Solyc10g0	0.02	-5.79
GO:00082	cysteine-ty	Molecular	0.22	0.49	Solyc09g0	Solyc09g0	0.00	-11.10
GO:00508	defense res	Biological	0.22	0.49	Solyc10g0	CHI9	0.28	-1.84
GO:00508	defense res	Biological	0.22	0.49	Solyc02g0	Solyc02g0	0.33	-1.59
GO:00508	defense res	Biological	0.22	0.49	Solyc09g0	Solyc09g0	0.47	-1.07
GO:00508	defense res	Biological	0.22	0.49	Solyc02g0	Solyc02g0	0.43	-1.23
GO:00508	defense res	Biological	0.22	0.49	Solyc01g0	Solyc01g0	0.31	-1.69
GO:00508	defense res	Biological	0.22	0.49	Solyc07g0	Solyc07g0	2.39	1.26
GO:00508	defense res	Biological	0.22	0.49	Solyc08g0	Solyc08g0	4.19	2.07
GO:00166	oxidoreduc	Molecular	0.22	0.49	Solyc03g0	Solyc03g0	0.11	-3.15
GO:00166	oxidoreduc	Molecular	0.22	0.49	Solyc10g0	Solyc10g0	0.00	-12.09
GO:00166	oxidoreduc	Molecular	0.22	0.49	Solyc01g0	Solyc01g0	0.12	-3.04
GO:00166	oxidoreduc	Molecular	0.22	0.49	Solyc01g0	Solyc01g0	0.00	-11.62
GO:00045	nuclease a	Molecular	0.23	0.49	Solyc04g0	Solyc04g0	0.09	-3.54
GO:00045	nuclease a	Molecular	0.23	0.49	Solyc12g0	Solyc12g0	3.30	1.72
GO:00045	nuclease a	Molecular	0.23	0.49	Solyc06g0	Solyc06g0	7023.58	12.78
GO:00045	nuclease a	Molecular	0.23	0.49	Solyc01g1	Solyc01g1	0.47	-1.08
GO:00045	nuclease a	Molecular	0.23	0.49	Solyc09g0	Solyc09g0	0.00	-9.78
GO:00045	nuclease a	Molecular	0.23	0.49	Solyc05g0	Solyc05g0	20.96	4.39
GO:00506	NADP bin	Molecular	0.23	0.49	Solyc01g1	Solyc01g1	11.39	3.51
GO:00506	NADP bin	Molecular	0.23	0.49	Solyc06g0	Solyc06g0	0.32	-1.66
GO:00506	NADP bin	Molecular	0.23	0.49	Solyc04g0	Solyc04g0	8.55	3.10
GO:00506	NADP bin	Molecular	0.23	0.49	Solyc06g0	Solyc06g0	3.53	1.82
GO:00506	NADP bin	Molecular	0.23	0.49	Solyc02g0	Solyc02g0	2.01	1.01
GO:00506	NADP bin	Molecular	0.23	0.49	Solyc02g0	Solyc02g0	2.27	1.18
GO:00098	seed germi	Biological	0.24	0.49	Solyc09g0	Solyc09g0	0.05	-4.31
GO:00098	seed germi	Biological	0.24	0.49	Solyc01g0	Solyc01g0	3.53	1.82
GO:00351	post-transc	Biological	0.24	0.49	Solyc02g0	Solyc02g0	0.49	-1.04
GO:00351	post-transc	Biological	0.24	0.49	Solyc06g0	Solyc06g0	2.07	1.05
GO:00096	response to	Biological	0.24	0.49	Solyc03g0	Solyc03g0	0.48	-1.05
GO:00096	response to	Biological	0.24	0.49	Solyc02g0	Solyc02g0	2.58	1.37
GO:00055	fatty acid b	Molecular	0.24	0.49	Solyc03g0	Solyc03g0	0.24	-2.06
GO:00055	fatty acid b	Molecular	0.24	0.49	Solyc06g0	Solyc06g0	2.19	1.13
GO:00508	cobalt ion	Molecular	0.24	0.49	Solyc01g0	Solyc01g0	2.26	1.18
GO:00508	cobalt ion	Molecular	0.24	0.49	Solyc01g0	Solyc01g0	2.27	1.18
GO:00091	nucleoside	Biological	0.24	0.49	Solyc01g1	Solyc01g1	2993.63	11.55
GO:00091	nucleoside	Biological	0.24	0.49	Solyc11g0	Solyc11g0	3.46	1.79
GO:00067	ATP biosy	Biological	0.24	0.49	Solyc10g0	Solyc10g0	0.03	-5.05
GO:00067	ATP biosy	Biological	0.24	0.49	Solyc11g0	Solyc11g0	0.22	-2.19
GO:00346	cellular res	Biological	0.24	0.49	Solyc03g0	Solyc03g0	0.24	-2.06
GO:00346	cellular res	Biological	0.24	0.49	Solyc06g0	Solyc06g0	2.09	1.07
GO:00346	cellular res	Biological	0.24	0.49	Solyc02g0	Solyc02g0	0.42	-1.24
GO:00346	cellular res	Biological	0.24	0.49	Solyc11g0	Solyc11g0	0.00	-10.83
GO:00167	cellulose s	Molecular	0.24	0.49	Solyc11g0	Solyc11g0	2.28	1.19
GO:00167	cellulose s	Molecular	0.24	0.49	Solyc12g0	Solyc12g0	3.03	1.60
GO:00167	cellulose s	Molecular	0.24	0.49	Solyc12g0	Solyc12g0	2.66	1.41
GO:00167	cellulose s	Molecular	0.24	0.49	Solyc01g0	Solyc01g0	0.19	-2.40
GO:00000	regulation	Biological	0.24	0.49	Solyc05g0	Solyc05g0	0.22	-2.15
GO:00000	regulation	Biological	0.24	0.49	Solyc04g0	Solyc04g0	0.41	-1.28

GO:00000	regulation	Biological	0.24	0.49	Solyc03g0	Solyc03g0	4.18	2.06
GO:00000	regulation	Biological	0.24	0.49	Solyc02g0	Solyc02g0	4.91	2.30
GO:00003	cyclin-dep	Cellular C	0.24	0.49	Solyc05g0	Solyc05g0	0.22	-2.15
GO:00003	cyclin-dep	Cellular C	0.24	0.49	Solyc04g0	Solyc04g0	0.41	-1.28
GO:00003	cyclin-dep	Cellular C	0.24	0.49	Solyc03g0	Solyc03g0	4.18	2.06
GO:00003	cyclin-dep	Cellular C	0.24	0.49	Solyc02g0	Solyc02g0	4.91	2.30
GO:00165	cyclin-dep	Molecular	0.24	0.49	Solyc05g0	Solyc05g0	0.22	-2.15
GO:00165	cyclin-dep	Molecular	0.24	0.49	Solyc04g0	Solyc04g0	0.41	-1.28
GO:00165	cyclin-dep	Molecular	0.24	0.49	Solyc03g0	Solyc03g0	4.18	2.06
GO:00165	cyclin-dep	Molecular	0.24	0.49	Solyc02g0	Solyc02g0	4.91	2.30
GO:00988	cellular ox	Biological	0.24	0.49	Solyc04g0	Solyc04g0	2.70	1.43
GO:00988	cellular ox	Biological	0.24	0.49	Solyc11g0	Solyc11g0	8.62	3.11
GO:00988	cellular ox	Biological	0.24	0.49	Solyc03g1	Solyc03g1	0.48	-1.07
GO:00988	cellular ox	Biological	0.24	0.49	Solyc01g1	Solyc01g1	4.37	2.13
GO:00988	cellular ox	Biological	0.24	0.49	Solyc03g0	Solyc03g0	4.16	2.06
GO:00436	protein sel	Molecular	0.24	0.49	Solyc10g0	Solyc10g0	0.09	-3.53
GO:00436	protein sel	Molecular	0.24	0.49	Solyc02g0	Solyc02g0	2.46	1.30
GO:00436	protein sel	Molecular	0.24	0.49	Solyc02g0	Solyc02g0	2.01	1.01
GO:00305	pectate lya	Molecular	0.24	0.49	Solyc09g0	Solyc09g0	3.61	1.85
GO:00305	pectate lya	Molecular	0.24	0.49	Solyc02g0	Solyc02g0	0.25	-2.01
GO:00305	pectate lya	Molecular	0.24	0.49	Solyc02g0	Solyc02g0	4235.90	12.05
GO:00096	response to	Biological	0.24	0.49	Solyc10g0	Solyc10g0	3.31	1.73
GO:00096	response to	Biological	0.24	0.49	Solyc09g0	Solyc09g0	8.11	3.02
GO:00096	response to	Biological	0.24	0.49	Solyc01g0	Solyc01g0	0.31	-1.69
GO:00099	anther deh	Biological	0.26	0.49	Solyc10g0	PG2	9.48	3.25
GO:00097	regulation	Biological	0.26	0.49	Solyc09g0	Solyc09g0	0.05	-4.31
GO:00090	leucine bic	Biological	0.26	0.49	Solyc08g0	Solyc08g0	2.65	1.41
GO:00099	alternative	Molecular	0.26	0.49	Solyc08g0	Solyc08g0	0.14	-2.87
GO:01027	ubiquinol:	Molecular	0.26	0.49	Solyc08g0	Solyc08g0	0.14	-2.87
GO:00044	methylenet	Molecular	0.26	0.49	Solyc01g0	Solyc01g0	2.62	1.39
GO:00088	crossover j	Molecular	0.26	0.49	Solyc01g0	Solyc01g0	3.52	1.81
GO:00159	carbon util	Biological	0.26	0.49	Solyc02g0	Solyc02g0	11.09	3.47
GO:00067	protoporph	Biological	0.26	0.49	Solyc04g0	Solyc04g0	3.78	1.92
GO:00161	nickel cati	Molecular	0.26	0.49	Solyc03g0	Solyc03g0	2.10	1.07
GO:00040	3-chloroall	Molecular	0.26	0.49	Solyc01g0	Solyc01g0	0.22	-2.18
GO:00364	L-methion	Molecular	0.26	0.49	Solyc03g1	E4	2.38	1.25
GO:00165	protein im	Biological	0.26	0.49	Solyc05g0	Solyc05g0	0.48	-1.05
GO:00086	pyridoxine	Biological	0.26	0.49	Solyc03g1	Solyc03g1	2.22	1.15
GO:00045	squalene m	Molecular	0.26	0.49	Solyc04g0	Solyc04g0	2.83	1.50
GO:00040	aspartate k	Molecular	0.26	0.49	Solyc01g0	Solyc01g0	2.09	1.06
GO:00000	ribosomal	Biological	0.26	0.49	Solyc12g0	Solyc12g0	2.35	1.23
GO:00000	mannosylt	Molecular	0.26	0.49	Solyc01g0	Solyc01g0	0.35	-1.51
GO:00324	histone de	Molecular	0.26	0.49	Solyc08g0	Solyc08g0	2.38	1.25
GO:00346	histone H3	Molecular	0.26	0.49	Solyc08g0	Solyc08g0	2.38	1.25
GO:00350	histone me	Cellular C	0.26	0.49	Solyc08g0	Solyc08g0	2.38	1.25
GO:00466	folic acid b	Biological	0.26	0.49	Solyc07g0	Solyc07g0	6.21	2.63
GO:00466	negative re	Biological	0.26	0.49	Solyc03g0	Solyc03g0	2.92	1.54
GO:00096	abscisic ac	Biological	0.26	0.49	Solyc02g0	Solyc02g0	0.45	-1.14
GO:00016	G protein-	Molecular	0.26	0.49	Solyc01g1	Solyc01g1	0.44	-1.20
GO:00071	adenylate	Biological	0.26	0.49	Solyc01g1	Solyc01g1	0.44	-1.20
GO:00063	tRNA splic	Biological	0.26	0.49	Solyc06g0	Solyc06g0	0.40	-1.33

GO:00057	mitochond	Cellular C	0.26	0.49	Solyc11g0	Solyc11g0	2.53	1.34
GO:00167	hydroxyme	Molecular	0.26	0.49	Solyc06g0	Solyc06g0	0.41	-1.30
GO:00167	oxidoreduc	Molecular	0.26	0.49	Solyc08g0	Solyc08g0	2.79	1.48
GO:00059	glycine cle	Cellular C	0.26	0.49	Solyc06g0	Solyc06g0	2.01	1.01
GO:00057	Golgi stack	Cellular C	0.26	0.49	Solyc09g0	Solyc09g0	3.41	1.77
GO:00157	sucrose tra	Biological	0.26	0.49	Solyc11g0	Solyc11g0	0.49	-1.04
GO:00465	sucrose-ph	Molecular	0.26	0.49	Solyc09g0	Solyc09g0	2.71	1.44
GO:00046	1,4-alpha-	Molecular	0.26	0.49	Solyc05g0	Solyc05g0	2.50	1.32
GO:00081	glycogen p	Molecular	0.26	0.49	Solyc05g0	Solyc05g0	2.50	1.32
GO:00070	mitotic chr	Biological	0.26	0.49	Solyc07g0	Solyc07g0	2.66	1.41
GO:00313	integral co	Cellular C	0.26	0.49	Solyc01g0	Solyc01g0	5.13	2.36
GO:00700	serine-type	Molecular	0.26	0.49	Solyc04g0	Solyc04g0	0.43	-1.23
GO:00039	acyl-CoA (	Molecular	0.26	0.49	Solyc10g0	Solyc10g0	2.27	1.19
GO:00007	resolution	Biological	0.26	0.49	Solyc01g0	Solyc01g0	0.39	-1.35
GO:00008	sister chro	Biological	0.26	0.49	Solyc01g0	Solyc01g0	0.39	-1.35
GO:00056	nuclear ori	Cellular C	0.26	0.49	Solyc05g0	Solyc05g0	2.54	1.35
GO:00000	DNA repli	Biological	0.26	0.49	Solyc04g0	Solyc04g0	2.35	1.23
GO:00431	replication	Biological	0.26	0.49	Solyc04g0	Solyc04g0	2.35	1.23
GO:00089	inositol m	Molecular	0.26	0.49	Solyc04g0	Solyc04g0	0.41	-1.30
GO:00038	1-acylglyc	Molecular	0.26	0.49	Solyc11g0	Solyc11g0	0.38	-1.41
GO:00312	extrinsic c	Cellular C	0.26	0.49	Solyc04g0	Solyc04g0	2.84	1.50
GO:00046	phosphatid	Molecular	0.26	0.49	Solyc09g0	Solyc09g0	3.57	1.84
GO:00092	glucan cata	Biological	0.26	0.49	Solyc06g0	Solyc06g0	2.25	1.17
GO:00017	cell fate sp	Biological	0.26	0.49	Solyc04g0	Solyc04g0	0.07	-3.79
GO:00455	9-cis-epox	Molecular	0.26	0.49	Solyc05g0	Solyc05g0	39.90	5.32
GO:00101	fruit devel	Biological	0.26	0.49	Solyc08g0	Solyc08g0	2486.58	11.28
GO:00452	plasma me	Cellular C	0.26	0.49	Solyc06g0	Solyc06g0	0.05	-4.46
GO:00057	mitochond	Cellular C	0.26	0.49	Solyc04g0	Solyc04g0	0.16	-2.66
GO:00066	phosphatid	Biological	0.26	0.49	Solyc06g0	Solyc06g0	2.08	1.06
GO:00001	sphingosin	Molecular	0.26	0.49	Solyc12g1	Solyc12g1	0.00	-10.11
GO:00197	secondary	Biological	0.26	0.49	Solyc05g0	Solyc05g0	1063.88	10.06
GO:00038	beta-1,4-m	Molecular	0.26	0.49	Solyc07g0	Solyc07g0	3.48	1.80
GO:00508	response t	Biological	0.26	0.49	Solyc12g0	Solyc12g0	14.44	3.85
GO:00167	oxidoreduc	Molecular	0.26	0.49	Solyc01g0	Solyc01g0	0.12	-3.03
GO:00071	male meios	Biological	0.26	0.49	Solyc07g0	Solyc07g0	2.49	1.32
GO:00009	phragmopl	Biological	0.26	0.49	Solyc01g0	Solyc01g0	0.00	-10.07
GO:00062	base-excisi	Biological	0.26	0.49	Solyc09g0	Solyc09g0	2.29	1.20
GO:00421	nitrate assi	Biological	0.26	0.49	Solyc06g0	Solyc06g0	0.32	-1.62
GO:00046	prephenate	Molecular	0.26	0.49	Solyc02g0	Solyc02g0	574.97	9.17
GO:00226	cytosolic r	Cellular C	0.26	0.49	Solyc01g0	Solyc01g0	550.66	9.11
GO:00070	cell-cell ju	Biological	0.26	0.49	Solyc10g0	Solyc10g0	2.16	1.11
GO:00100	regulation	Biological	0.26	0.49	Solyc10g0	Solyc10g0	687.65	9.43
GO:00095	embryo sa	Biological	0.26	0.49	Solyc11g0	Solyc11g0	18.97	4.25
GO:00459	positive re	Biological	0.26	0.49	Solyc09g0	Solyc09g0	0.25	-2.00
GO:00065	polyamine	Biological	0.26	0.49	Solyc01g0	Solyc01g0	0.03	-4.98
GO:00094	response t	Biological	0.26	0.49	Solyc04g0	Solyc04g0	2.70	1.43
GO:00094	response t	Biological	0.26	0.49	Solyc09g0	Solyc09g0	0.34	-1.54
GO:00094	response t	Biological	0.26	0.49	Solyc02g0	Solyc02g0	0.45	-1.14
GO:00094	response t	Biological	0.26	0.49	Solyc06g0	Solyc06g0	2.02	1.02
GO:00094	response t	Biological	0.26	0.49	Solyc01g1	Solyc01g1	14.86	3.89
GO:00094	response t	Biological	0.26	0.49	Solyc12g0	Solyc12g0	369.30	8.53

GO:00511	sugar trans	Molecular	0.26	0.50	Solyc12g0	Solyc12g0	44.33	5.47
GO:00511	sugar trans	Molecular	0.26	0.50	Solyc09g0	Solyc09g0	6.94	2.79
GO:00511	sugar trans	Molecular	0.26	0.50	Solyc06g0	Solyc06g0	6.96	2.80
GO:00061	malate met	Biological	0.26	0.50	Solyc11g0	Solyc11g0	4.12	2.04
GO:00061	malate met	Biological	0.26	0.50	Solyc03g0	Solyc03g0	2.79	1.48
GO:00038	6-phospho	Molecular	0.26	0.50	Solyc08g0	Solyc08g0	3.48	1.80
GO:00038	6-phospho	Molecular	0.26	0.50	Solyc04g0	Solyc04g0	0.41	-1.27
GO:00701	large ribos	Molecular	0.26	0.50	Solyc09g0	Solyc09g0	4.17	2.06
GO:00701	large ribos	Molecular	0.26	0.50	Solyc09g0	Solyc09g0	2.84	1.51
GO:00096	plant-type	Biological	0.26	0.50	Solyc09g0	Solyc09g0	0.48	-1.06
GO:00096	plant-type	Biological	0.26	0.50	Solyc01g0	Solyc01g0	0.43	-1.23
GO:00062	DNA meta	Biological	0.26	0.50	Solyc09g0	Solyc09g0	0.35	-1.51
GO:00062	DNA meta	Biological	0.26	0.50	Solyc01g0	Solyc01g0	0.39	-1.35
GO:00053	manganese	Molecular	0.26	0.50	Solyc04g0	Solyc04g0	0.33	-1.58
GO:00053	manganese	Molecular	0.26	0.50	Solyc04g0	Solyc04g0	3.05	1.61
GO:00046	transmemb	Molecular	0.27	0.52	Solyc06g0	Solyc06g0	0.37	-1.44
GO:00046	transmemb	Molecular	0.27	0.52	Solyc05g0	Solyc05g0	0.50	-1.00
GO:00046	transmemb	Molecular	0.27	0.52	Solyc11g0	Solyc11g0	6.63	2.73
GO:00046	transmemb	Molecular	0.27	0.52	Solyc03g1	Solyc03g1	0.49	-1.04
GO:00046	transmemb	Molecular	0.27	0.52	Solyc11g0	Solyc11g0	2.78	1.48
GO:00046	transmemb	Molecular	0.27	0.52	Solyc10g0	Solyc10g0	4.22	2.08
GO:00046	transmemb	Molecular	0.27	0.52	Solyc02g0	Solyc02g0	0.17	-2.58
GO:00046	transmemb	Molecular	0.27	0.52	Solyc02g0	Solyc02g0	0.07	-3.78
GO:00058	kinesin co	Cellular C	0.28	0.54	Solyc09g0	Solyc09g0	0.05	-4.40
GO:00058	kinesin co	Cellular C	0.28	0.54	Solyc09g0	Solyc09g0	0.07	-3.89
GO:00058	kinesin co	Cellular C	0.28	0.54	Solyc09g0	Solyc09g0	0.35	-1.53
GO:00454	pectin cata	Biological	0.29	0.54	Solyc10g0	PG2	9.48	3.25
GO:00454	pectin cata	Biological	0.29	0.54	Solyc09g0	Solyc09g0	3.61	1.85
GO:00454	pectin cata	Biological	0.29	0.54	Solyc03g1	Solyc03g1	0.44	-1.20
GO:00454	pectin cata	Biological	0.29	0.54	Solyc02g0	Solyc02g0	4235.90	12.05
GO:00454	pectin cata	Biological	0.29	0.54	Solyc07g0	Solyc07g0	0.39	-1.35
GO:00045	endoribonu	Molecular	0.29	0.55	Solyc07g0	Solyc07g0	6.72	2.75
GO:00045	endoribonu	Molecular	0.29	0.55	Solyc05g0	Solyc05g0	20.96	4.39
GO:00512	protein co	Biological	0.29	0.55	Solyc10g0	Solyc10g0	0.09	-3.53
GO:00512	protein co	Biological	0.29	0.55	Solyc02g0	Solyc02g0	2.46	1.30
GO:00055	ATP bindi	Molecular	0.30	0.55	Solyc09g0	Solyc09g0	0.05	-4.40
GO:00055	ATP bindi	Molecular	0.30	0.55	Solyc04g0	Solyc04g0	2.70	1.43
GO:00055	ATP bindi	Molecular	0.30	0.55	Solyc01g0	Solyc01g0	2.62	1.39
GO:00055	ATP bindi	Molecular	0.30	0.55	Solyc06g0	Solyc06g0	9.47	3.24
GO:00055	ATP bindi	Molecular	0.30	0.55	Solyc02g0	Solyc02g0	3.08	1.62
GO:00055	ATP bindi	Molecular	0.30	0.55	Solyc05g0	Solyc05g0	4.45	2.16
GO:00055	ATP bindi	Molecular	0.30	0.55	Solyc06g0	Solyc06g0	7.83	2.97
GO:00055	ATP bindi	Molecular	0.30	0.55	Solyc05g0	Solyc05g0	2.80	1.49
GO:00055	ATP bindi	Molecular	0.30	0.55	Solyc09g0	Solyc09g0	2.98	1.58
GO:00055	ATP bindi	Molecular	0.30	0.55	Solyc09g0	Solyc09g0	0.32	-1.64
GO:00055	ATP bindi	Molecular	0.30	0.55	Solyc04g0	Solyc04g0	3.53	1.82
GO:00055	ATP bindi	Molecular	0.30	0.55	Solyc09g0	Solyc09g0	0.23	-2.09
GO:00055	ATP bindi	Molecular	0.30	0.55	Solyc02g0	Solyc02g0	11.83	3.56
GO:00055	ATP bindi	Molecular	0.30	0.55	Solyc02g0	Solyc02g0	0.30	-1.71
GO:00055	ATP bindi	Molecular	0.30	0.55	Solyc07g0	Solyc07g0	2.20	1.14
GO:00055	ATP bindi	Molecular	0.30	0.55	Solyc01g0	Solyc01g0	3.08	1.62

GO:00055	ATP bindi	Molecular	0.30	0.55	Solyc11g0	Solyc11g0	0.41	-1.28
GO:00055	ATP bindi	Molecular	0.30	0.55	Solyc04g0	Solyc04g0	0.40	-1.31
GO:00055	ATP bindi	Molecular	0.30	0.55	Solyc08g0	Solyc08g0	3.48	1.80
GO:00055	ATP bindi	Molecular	0.30	0.55	Solyc09g0	Solyc09g0	0.29	-1.78
GO:00055	ATP bindi	Molecular	0.30	0.55	Solyc01g1	Solyc01g1	3.76	1.91
GO:00055	ATP bindi	Molecular	0.30	0.55	Solyc09g0	Solyc09g0	7.25	2.86
GO:00055	ATP bindi	Molecular	0.30	0.55	Solyc09g0	Solyc09g0	2.40	1.26
GO:00055	ATP bindi	Molecular	0.30	0.55	Solyc09g0	Solyc09g0	0.25	-2.01
GO:00055	ATP bindi	Molecular	0.30	0.55	Solyc09g0	Solyc09g0	0.24	-2.04
GO:00055	ATP bindi	Molecular	0.30	0.55	Solyc01g0	Solyc01g0	0.34	-1.54
GO:00055	ATP bindi	Molecular	0.30	0.55	Solyc12g0	Solyc12g0	2.35	1.23
GO:00055	ATP bindi	Molecular	0.30	0.55	Solyc09g0	Solyc09g0	4.52	2.18
GO:00055	ATP bindi	Molecular	0.30	0.55	Solyc01g1	Solyc01g1	72.82	6.19
GO:00055	ATP bindi	Molecular	0.30	0.55	Solyc11g0	Solyc11g0	0.47	-1.10
GO:00055	ATP bindi	Molecular	0.30	0.55	Solyc03g0	Solyc03g0	3.18	1.67
GO:00055	ATP bindi	Molecular	0.30	0.55	Solyc03g0	Solyc03g0	2.16	1.11
GO:00055	ATP bindi	Molecular	0.30	0.55	Solyc03g0	Solyc03g0	7.56	2.92
GO:00055	ATP bindi	Molecular	0.30	0.55	Solyc05g0	Solyc05g0	0.19	-2.39
GO:00055	ATP bindi	Molecular	0.30	0.55	Solyc02g0	Solyc02g0	0.44	-1.19
GO:00055	ATP bindi	Molecular	0.30	0.55	Solyc07g0	Solyc07g0	2.19	1.13
GO:00055	ATP bindi	Molecular	0.30	0.55	Solyc01g1	Solyc01g1	2.32	1.22
GO:00055	ATP bindi	Molecular	0.30	0.55	Solyc02g0	Solyc02g0	0.38	-1.41
GO:00055	ATP bindi	Molecular	0.30	0.55	Solyc04g0	Solyc04g0	0.43	-1.23
GO:00055	ATP bindi	Molecular	0.30	0.55	Solyc04g0	Solyc04g0	0.41	-1.29
GO:00055	ATP bindi	Molecular	0.30	0.55	Solyc01g0	Solyc01g0	0.30	-1.75
GO:00055	ATP bindi	Molecular	0.30	0.55	Solyc02g0	Solyc02g0	0.50	-1.00
GO:00055	ATP bindi	Molecular	0.30	0.55	Solyc06g0	Solyc06g0	2.09	1.07
GO:00055	ATP bindi	Molecular	0.30	0.55	Solyc09g0	Solyc09g0	0.35	-1.51
GO:00055	ATP bindi	Molecular	0.30	0.55	Solyc06g0	Solyc06g0	0.37	-1.44
GO:00055	ATP bindi	Molecular	0.30	0.55	Solyc09g0	Solyc09g0	0.37	-1.43
GO:00055	ATP bindi	Molecular	0.30	0.55	Solyc10g0	Solyc10g0	2.78	1.48
GO:00055	ATP bindi	Molecular	0.30	0.55	Solyc05g0	Solyc05g0	0.50	-1.00
GO:00055	ATP bindi	Molecular	0.30	0.55	Solyc10g0	Solyc10g0	0.43	-1.22
GO:00055	ATP bindi	Molecular	0.30	0.55	Solyc08g0	Solyc08g0	3.77	1.91
GO:00055	ATP bindi	Molecular	0.30	0.55	Solyc11g0	Solyc11g0	6.63	2.73
GO:00055	ATP bindi	Molecular	0.30	0.55	Solyc06g0	Solyc06g0	3.42	1.77
GO:00055	ATP bindi	Molecular	0.30	0.55	Solyc06g0	Solyc06g0	2.33	1.22
GO:00055	ATP bindi	Molecular	0.30	0.55	Solyc07g0	Solyc07g0	2.15	1.11
GO:00055	ATP bindi	Molecular	0.30	0.55	Solyc05g0	Solyc05g0	0.43	-1.22
GO:00055	ATP bindi	Molecular	0.30	0.55	Solyc07g0	Solyc07g0	2.62	1.39
GO:00055	ATP bindi	Molecular	0.30	0.55	Solyc06g0	Solyc06g0	2513.21	11.30
GO:00055	ATP bindi	Molecular	0.30	0.55	Solyc01g0	Solyc01g0	2.56	1.36
GO:00055	ATP bindi	Molecular	0.30	0.55	Solyc04g0	Solyc04g0	0.00	-11.98
GO:00055	ATP bindi	Molecular	0.30	0.55	Solyc02g0	Solyc02g0	0.49	-1.02
GO:00055	ATP bindi	Molecular	0.30	0.55	Solyc04g0	Solyc04g0	0.00	-13.91
GO:00055	ATP bindi	Molecular	0.30	0.55	Solyc03g1	Solyc03g1	0.49	-1.04
GO:00055	ATP bindi	Molecular	0.30	0.55	Solyc01g0	Solyc01g0	0.39	-1.35
GO:00055	ATP bindi	Molecular	0.30	0.55	Solyc10g0	Solyc10g0	0.04	-4.70
GO:00055	ATP bindi	Molecular	0.30	0.55	Solyc11g0	Solyc11g0	0.46	-1.13
GO:00055	ATP bindi	Molecular	0.30	0.55	Solyc09g0	Solyc09g0	0.00	-13.71
GO:00055	ATP bindi	Molecular	0.30	0.55	Solyc11g0	Solyc11g0	2.57	1.36

GO:00055	ATP bindi	Molecular	0.30	0.55	Solyc12g0	Solyc12g0	0.00	-13.90
GO:00055	ATP bindi	Molecular	0.30	0.55	Solyc09g0	Solyc09g0	0.03	-4.88
GO:00055	ATP bindi	Molecular	0.30	0.55	Solyc11g0	Solyc11g0	2.78	1.48
GO:00055	ATP bindi	Molecular	0.30	0.55	Solyc12g0	Solyc12g0	2.20	1.14
GO:00055	ATP bindi	Molecular	0.30	0.55	Solyc05g0	Solyc05g0	0.00	-14.18
GO:00055	ATP bindi	Molecular	0.30	0.55	Solyc10g0	Solyc10g0	0.08	-3.68
GO:00055	ATP bindi	Molecular	0.30	0.55	Solyc08g0	Solyc08g0	2.67	1.41
GO:00055	ATP bindi	Molecular	0.30	0.55	Solyc12g0	Solyc12g0	2.58	1.37
GO:00055	ATP bindi	Molecular	0.30	0.55	Solyc06g0	Solyc06g0	0.15	-2.77
GO:00055	ATP bindi	Molecular	0.30	0.55	Solyc08g0	Solyc08g0	0.02	-6.06
GO:00055	ATP bindi	Molecular	0.30	0.55	Solyc06g0	Solyc06g0	0.46	-1.11
GO:00055	ATP bindi	Molecular	0.30	0.55	Solyc09g0	Solyc09g0	0.00	-13.84
GO:00055	ATP bindi	Molecular	0.30	0.55	Solyc01g0	Solyc01g0	2.62	1.39
GO:00055	ATP bindi	Molecular	0.30	0.55	Solyc08g0	Solyc08g0	2.38	1.25
GO:00055	ATP bindi	Molecular	0.30	0.55	Solyc01g0	Solyc01g0	0.41	-1.30
GO:00055	ATP bindi	Molecular	0.30	0.55	Solyc10g0	Solyc10g0	0.00	-12.76
GO:00055	ATP bindi	Molecular	0.30	0.55	Solyc03g0	Solyc03g0	2300.48	11.17
GO:00055	ATP bindi	Molecular	0.30	0.55	Solyc11g0	Solyc11g0	2.24	1.16
GO:00055	ATP bindi	Molecular	0.30	0.55	Solyc04g0	Solyc04g0	3.70	1.89
GO:00055	ATP bindi	Molecular	0.30	0.55	Solyc06g0	Solyc06g0	0.42	-1.25
GO:00055	ATP bindi	Molecular	0.30	0.55	Solyc12g0	Solyc12g0	0.41	-1.28
GO:00055	ATP bindi	Molecular	0.30	0.55	Solyc01g0	Solyc01g0	3.64	1.87
GO:00055	ATP bindi	Molecular	0.30	0.55	Solyc06g0	Solyc06g0	0.43	-1.23
GO:00055	ATP bindi	Molecular	0.30	0.55	Solyc04g0	Solyc04g0	0.46	-1.11
GO:00055	ATP bindi	Molecular	0.30	0.55	Solyc03g0	Solyc03g0	0.00	-12.50
GO:00055	ATP bindi	Molecular	0.30	0.55	Solyc06g0	Solyc06g0	0.00	-9.49
GO:00055	ATP bindi	Molecular	0.30	0.55	Solyc10g0	Solyc10g0	2.19	1.13
GO:00055	ATP bindi	Molecular	0.30	0.55	Solyc08g0	Solyc08g0	0.50	-1.00
GO:00055	ATP bindi	Molecular	0.30	0.55	Solyc08g0	Solyc08g0	2.43	1.28
GO:00055	ATP bindi	Molecular	0.30	0.55	Solyc09g0	Solyc09g0	0.07	-3.94
GO:00055	ATP bindi	Molecular	0.30	0.55	Solyc10g0	Solyc10g0	4.22	2.08
GO:00055	ATP bindi	Molecular	0.30	0.55	Solyc04g0	Solyc04g0	0.39	-1.37
GO:00055	ATP bindi	Molecular	0.30	0.55	Solyc04g0	Solyc04g0	0.16	-2.66
GO:00055	ATP bindi	Molecular	0.30	0.55	Solyc08g0	Solyc08g0	0.00	-10.00
GO:00055	ATP bindi	Molecular	0.30	0.55	Solyc07g0	Solyc07g0	1180.56	10.21
GO:00055	ATP bindi	Molecular	0.30	0.55	Solyc03g1	Solyc03g1	0.02	-5.50
GO:00055	ATP bindi	Molecular	0.30	0.55	Solyc12g0	Solyc12g0	3.17	1.66
GO:00055	ATP bindi	Molecular	0.30	0.55	Solyc06g0	Solyc06g0	0.37	-1.44
GO:00055	ATP bindi	Molecular	0.30	0.55	Solyc04g0	Solyc04g0	0.44	-1.18
GO:00055	ATP bindi	Molecular	0.30	0.55	Solyc12g1	Solyc12g1	249.94	7.97
GO:00055	ATP bindi	Molecular	0.30	0.55	Solyc03g0	Solyc03g0	0.49	-1.03
GO:00055	ATP bindi	Molecular	0.30	0.55	Solyc09g0	Solyc09g0	0.07	-3.89
GO:00055	ATP bindi	Molecular	0.30	0.55	Solyc03g0	Solyc03g0	2.40	1.26
GO:00055	ATP bindi	Molecular	0.30	0.55	Solyc01g0	Solyc01g0	5.28	2.40
GO:00055	ATP bindi	Molecular	0.30	0.55	Solyc07g0	Solyc07g0	0.47	-1.10
GO:00055	ATP bindi	Molecular	0.30	0.55	Solyc06g0	Solyc06g0	0.34	-1.55
GO:00055	ATP bindi	Molecular	0.30	0.55	Solyc12g0	Solyc12g0	3.71	1.89
GO:00055	ATP bindi	Molecular	0.30	0.55	Solyc06g0	Solyc06g0	912.98	9.83
GO:00055	ATP bindi	Molecular	0.30	0.55	Solyc04g0	Solyc04g0	0.18	-2.51
GO:00055	ATP bindi	Molecular	0.30	0.55	Solyc06g0	Solyc06g0	0.34	-1.57
GO:00055	ATP bindi	Molecular	0.30	0.55	Solyc11g0	Solyc11g0	0.00	-12.68

GO:00055	ATP bindi	Molecular	0.30	0.55	Solyc08g0	Solyc08g0	0.41	-1.30
GO:00055	ATP bindi	Molecular	0.30	0.55	Solyc02g0	Solyc02g0	307.12	8.26
GO:00055	ATP bindi	Molecular	0.30	0.55	Solyc12g0	Solyc12g0	467.31	8.87
GO:00055	ATP bindi	Molecular	0.30	0.55	Solyc02g0	Solyc02g0	2.27	1.18
GO:00055	ATP bindi	Molecular	0.30	0.55	Solyc04g0	Solyc04g0	0.41	-1.27
GO:00055	ATP bindi	Molecular	0.30	0.55	Solyc05g0	Solyc05g0	0.46	-1.13
GO:00055	ATP bindi	Molecular	0.30	0.55	Solyc03g1	Solyc03g1	0.06	-4.15
GO:00055	ATP bindi	Molecular	0.30	0.55	Solyc06g0	Solyc06g0	15.51	3.95
GO:00055	ATP bindi	Molecular	0.30	0.55	Solyc01g1	Solyc01g1	18.01	4.17
GO:00055	ATP bindi	Molecular	0.30	0.55	Solyc02g0	Solyc02g0	0.17	-2.58
GO:00055	ATP bindi	Molecular	0.30	0.55	Solyc04g0	Solyc04g0	0.06	-3.97
GO:00055	ATP bindi	Molecular	0.30	0.55	Solyc06g0	Solyc06g0	0.03	-4.93
GO:00055	ATP bindi	Molecular	0.30	0.55	Solyc07g0	Solyc07g0	2.39	1.26
GO:00055	ATP bindi	Molecular	0.30	0.55	Solyc09g0	Solyc09g0	0.35	-1.53
GO:00055	ATP bindi	Molecular	0.30	0.55	Solyc08g0	Solyc08g0	2.56	1.36
GO:00055	ATP bindi	Molecular	0.30	0.55	Solyc01g0	Solyc01g0	0.00	-9.25
GO:00055	ATP bindi	Molecular	0.30	0.55	Solyc07g0	Solyc07g0	2.77	1.47
GO:00055	ATP bindi	Molecular	0.30	0.55	Solyc08g0	Solyc08g0	2.07	1.05
GO:00055	ATP bindi	Molecular	0.30	0.55	Solyc04g0	Solyc04g0	7.72	2.95
GO:00055	ATP bindi	Molecular	0.30	0.55	Solyc09g0	Solyc09g0	0.34	-1.58
GO:00055	ATP bindi	Molecular	0.30	0.55	Solyc01g0	Solyc01g0	550.66	9.11
GO:00055	ATP bindi	Molecular	0.30	0.55	Solyc11g0	Solyc11g0	0.22	-2.19
GO:00055	ATP bindi	Molecular	0.30	0.55	Solyc03g1	Solyc03g1	356.83	8.48
GO:00055	ATP bindi	Molecular	0.30	0.55	Solyc12g0	Solyc12g0	0.00	-11.51
GO:00055	ATP bindi	Molecular	0.30	0.55	Solyc06g0	Solyc06g0	0.07	-3.77
GO:00055	ATP bindi	Molecular	0.30	0.55	Solyc07g0	Solyc07g0	0.11	-3.12
GO:00055	ATP bindi	Molecular	0.30	0.55	Solyc01g0	Solyc01g0	0.00	-9.77
GO:00055	ATP bindi	Molecular	0.30	0.55	Solyc07g0	Solyc07g0	770.05	9.59
GO:00055	ATP bindi	Molecular	0.30	0.55	Solyc12g0	Solyc12g0	4.99	2.32
GO:00055	ATP bindi	Molecular	0.30	0.55	Solyc08g0	Solyc08g0	4.19	2.07
GO:00055	ATP bindi	Molecular	0.30	0.55	Solyc02g0	Solyc02g0	394.05	8.62
GO:00055	ATP bindi	Molecular	0.30	0.55	Solyc02g0	Solyc02g0	0.07	-3.78
GO:00055	ATP bindi	Molecular	0.30	0.55	Solyc03g0	Solyc03g0	0.36	-1.46
GO:00055	ATP bindi	Molecular	0.30	0.55	Solyc07g0	Solyc07g0	2.88	1.52
GO:00055	ATP bindi	Molecular	0.30	0.55	Solyc02g0	Solyc02g0	517.85	9.02
GO:00055	ATP bindi	Molecular	0.30	0.55	Solyc08g0	Solyc08g0	0.11	-3.25
GO:00055	ATP bindi	Molecular	0.30	0.55	Solyc06g0	Solyc06g0	9.89	3.31
GO:00055	ATP bindi	Molecular	0.30	0.55	Solyc02g0	Solyc02g0	960.34	9.91
GO:00055	ATP bindi	Molecular	0.30	0.55	Solyc07g0	Solyc07g0	0.39	-1.35
GO:00055	ATP bindi	Molecular	0.30	0.55	Solyc03g0	Solyc03g0	0.39	-1.37
GO:00055	ATP bindi	Molecular	0.30	0.55	Solyc11g0	Solyc11g0	2.48	1.31
GO:00055	ATP bindi	Molecular	0.30	0.55	Solyc03g1	Solyc03g1	0.00	-8.90
GO:00055	ATP bindi	Molecular	0.30	0.55	Solyc01g0	Solyc01g0	2.50	1.32
GO:00055	ATP bindi	Molecular	0.30	0.55	Solyc12g0	Solyc12g0	11.23	3.49
GO:00055	ATP bindi	Molecular	0.30	0.55	Solyc03g0	Solyc03g0	0.10	-3.29
GO:00055	ATP bindi	Molecular	0.30	0.55	Solyc11g0	Solyc11g0	0.22	-2.20
GO:00009	transcripti	Molecular	0.30	0.55	Solyc06g0	Solyc06g0	0.48	-1.05
GO:00009	transcripti	Molecular	0.30	0.55	Solyc09g0	Solyc09g0	0.34	-1.58
GO:00009	transcripti	Molecular	0.30	0.55	Solyc01g0	Solyc01g0	0.10	-3.35
GO:00009	transcripti	Molecular	0.30	0.55	Solyc06g0	Solyc06g0	0.49	-1.03
GO:00009	transcripti	Molecular	0.30	0.55	Solyc02g0	Solyc02g0	0.06	-4.15

GO:00009	transcripti	Molecular	0.30	0.55	Solyc10g0	Solyc10g0	0.26	-1.94
GO:00009	transcripti	Molecular	0.30	0.55	Solyc01g0	Solyc01g0	0.45	-1.14
GO:00800	quercetin 3	Molecular	0.30	0.55	Solyc09g0	Solyc09g0	0.05	-4.31
GO:00800	quercetin 3	Molecular	0.30	0.55	Solyc02g0	Solyc02g0	11.82	3.56
GO:00800	quercetin 3	Molecular	0.30	0.55	Solyc12g0	Solyc12g0	2.38	1.25
GO:00800	quercetin 3	Molecular	0.30	0.55	Solyc12g0	Solyc12g0	0.00	-9.95
GO:00800	quercetin 7	Molecular	0.30	0.55	Solyc09g0	Solyc09g0	0.05	-4.31
GO:00800	quercetin 7	Molecular	0.30	0.55	Solyc02g0	Solyc02g0	11.82	3.56
GO:00800	quercetin 7	Molecular	0.30	0.55	Solyc12g0	Solyc12g0	2.38	1.25
GO:00800	quercetin 7	Molecular	0.30	0.55	Solyc12g0	Solyc12g0	0.00	-9.95
GO:00302	cellulose b	Biological	0.30	0.55	Solyc11g0	Solyc11g0	2.28	1.19
GO:00302	cellulose b	Biological	0.30	0.55	Solyc12g0	Solyc12g0	3.03	1.60
GO:00302	cellulose b	Biological	0.30	0.55	Solyc12g0	Solyc12g0	2.66	1.41
GO:00302	cellulose b	Biological	0.30	0.55	Solyc01g0	Solyc01g0	0.19	-2.40
GO:00048	serine-type	Molecular	0.30	0.55	Solyc04g0	Solyc04g0	0.28	-1.84
GO:00048	serine-type	Molecular	0.30	0.55	Solyc09g0	Solyc09g0	10879.94	13.41
GO:00048	serine-type	Molecular	0.30	0.55	Solyc03g0	Solyc03g0	0.00	-11.75
GO:00048	serine-type	Molecular	0.30	0.55	Solyc03g0	Solyc03g0	11.28	3.50
GO:00057	cytoplasm	Cellular C	0.31	0.55	Solyc10g0	Solyc10g0	4.23	2.08
GO:00057	cytoplasm	Cellular C	0.31	0.55	Solyc09g0	Solyc09g0	0.05	-4.31
GO:00057	cytoplasm	Cellular C	0.31	0.55	Solyc10g0	Solyc10g0	4.26	2.09
GO:00057	cytoplasm	Cellular C	0.31	0.55	Solyc04g0	Solyc04g0	6.77	2.76
GO:00057	cytoplasm	Cellular C	0.31	0.55	Solyc09g0	Solyc09g0	6.99	2.80
GO:00057	cytoplasm	Cellular C	0.31	0.55	Solyc07g0	Solyc07g0	0.35	-1.51
GO:00057	cytoplasm	Cellular C	0.31	0.55	Solyc04g0	Solyc04g0	2.70	1.43
GO:00057	cytoplasm	Cellular C	0.31	0.55	Solyc09g0	Solyc09g0	0.27	-1.91
GO:00057	cytoplasm	Cellular C	0.31	0.55	Solyc01g0	Solyc01g0	2.62	1.39
GO:00057	cytoplasm	Cellular C	0.31	0.55	Solyc11g0	Solyc11g0	0.10	-3.26
GO:00057	cytoplasm	Cellular C	0.31	0.55	Solyc01g0	Solyc01g0	2.46	1.30
GO:00057	cytoplasm	Cellular C	0.31	0.55	Solyc07g0	Solyc07g0	2.77	1.47
GO:00057	cytoplasm	Cellular C	0.31	0.55	Solyc09g0	Solyc09g0	2.88	1.53
GO:00057	cytoplasm	Cellular C	0.31	0.55	Solyc03g1	Solyc03g1	0.35	-1.54
GO:00057	cytoplasm	Cellular C	0.31	0.55	Solyc08g0	LOX1.1	2.60	1.38
GO:00057	cytoplasm	Cellular C	0.31	0.55	Solyc01g0	Solyc01g0	2.26	1.17
GO:00057	cytoplasm	Cellular C	0.31	0.55	Solyc04g0	Solyc04g0	2.35	1.23
GO:00057	cytoplasm	Cellular C	0.31	0.55	Solyc03g1	E4	2.38	1.25
GO:00057	cytoplasm	Cellular C	0.31	0.55	Solyc08g0	Solyc08g0	3.48	1.80
GO:00057	cytoplasm	Cellular C	0.31	0.55	Solyc09g0	Solyc09g0	0.34	-1.54
GO:00057	cytoplasm	Cellular C	0.31	0.55	Solyc07g0	Solyc07g0	2.17	1.12
GO:00057	cytoplasm	Cellular C	0.31	0.55	Solyc09g0	Solyc09g0	0.40	-1.34
GO:00057	cytoplasm	Cellular C	0.31	0.55	Solyc02g0	Solyc02g0	0.49	-1.04
GO:00057	cytoplasm	Cellular C	0.31	0.55	Solyc01g0	Solyc01g0	0.34	-1.54
GO:00057	cytoplasm	Cellular C	0.31	0.55	Solyc11g0	Solyc11g0	2.05	1.04
GO:00057	cytoplasm	Cellular C	0.31	0.55	Solyc09g0	Solyc09g0	4.17	2.06
GO:00057	cytoplasm	Cellular C	0.31	0.55	Solyc03g1	Solyc03g1	2.42	1.27
GO:00057	cytoplasm	Cellular C	0.31	0.55	Solyc01g0	Solyc01g0	0.32	-1.65
GO:00057	cytoplasm	Cellular C	0.31	0.55	Solyc06g0	Solyc06g0	3.21	1.68
GO:00057	cytoplasm	Cellular C	0.31	0.55	Solyc07g0	Solyc07g0	8.41	3.07
GO:00057	cytoplasm	Cellular C	0.31	0.55	Solyc06g0	Solyc06g0	0.11	-3.19
GO:00057	cytoplasm	Cellular C	0.31	0.55	Solyc09g0	Solyc09g0	0.27	-1.90
GO:00057	cytoplasm	Cellular C	0.31	0.55	Solyc01g1	Solyc01g1	2.32	1.22

GO:00057	cytoplasm	Cellular Co	0.31	0.55	Solyc06g0	Solyc06g0	4.81	2.27
GO:00057	cytoplasm	Cellular Co	0.31	0.55	Solyc02g0	Solyc02g0	6.74	2.75
GO:00057	cytoplasm	Cellular Co	0.31	0.55	Solyc06g0	Solyc06g0	2.09	1.07
GO:00057	cytoplasm	Cellular Co	0.31	0.55	Solyc07g0	Solyc07g0	0.46	-1.11
GO:00057	cytoplasm	Cellular Co	0.31	0.55	Solyc01g1	Solyc01g1	2.18	1.13
GO:00057	cytoplasm	Cellular Co	0.31	0.55	Solyc04g0	Solyc04g0	2.07	1.05
GO:00057	cytoplasm	Cellular Co	0.31	0.55	Solyc05g0	Solyc05g0	0.22	-2.15
GO:00057	cytoplasm	Cellular Co	0.31	0.55	Solyc09g0	Solyc09g0	0.05	-4.37
GO:00057	cytoplasm	Cellular Co	0.31	0.55	Solyc10g0	Solyc10g0	2.23	1.16
GO:00057	cytoplasm	Cellular Co	0.31	0.55	Solyc11g0	Solyc11g0	2.07	1.05
GO:00057	cytoplasm	Cellular Co	0.31	0.55	Solyc05g0	Solyc05g0	2.50	1.32
GO:00057	cytoplasm	Cellular Co	0.31	0.55	Solyc07g0	Solyc07g0	2.15	1.11
GO:00057	cytoplasm	Cellular Co	0.31	0.55	Solyc04g0	Solyc04g0	0.43	-1.23
GO:00057	cytoplasm	Cellular Co	0.31	0.55	Solyc12g0	Solyc12g0	3.54	1.82
GO:00057	cytoplasm	Cellular Co	0.31	0.55	Solyc07g0	Solyc07g0	3.21	1.68
GO:00057	cytoplasm	Cellular Co	0.31	0.55	Solyc04g0	Solyc04g0	6800.94	12.73
GO:00057	cytoplasm	Cellular Co	0.31	0.55	Solyc06g0	Solyc06g0	2.07	1.05
GO:00057	cytoplasm	Cellular Co	0.31	0.55	Solyc11g0	Solyc11g0	0.46	-1.13
GO:00057	cytoplasm	Cellular Co	0.31	0.55	Solyc09g0	rpl33	0.05	-4.19
GO:00057	cytoplasm	Cellular Co	0.31	0.55	Solyc10g0	Solyc10g0	2.10	1.07
GO:00057	cytoplasm	Cellular Co	0.31	0.55	Solyc03g1	Solyc03g1	2.39	1.26
GO:00057	cytoplasm	Cellular Co	0.31	0.55	Solyc12g0	Solyc12g0	0.00	-13.90
GO:00057	cytoplasm	Cellular Co	0.31	0.55	Solyc04g0	Solyc04g0	0.41	-1.28
GO:00057	cytoplasm	Cellular Co	0.31	0.55	Solyc06g0	Solyc06g0	0.15	-2.74
GO:00057	cytoplasm	Cellular Co	0.31	0.55	Solyc05g0	Solyc05g0	0.03	-5.29
GO:00057	cytoplasm	Cellular Co	0.31	0.55	Solyc03g0	Solyc03g0	4.18	2.06
GO:00057	cytoplasm	Cellular Co	0.31	0.55	Solyc09g0	Solyc09g0	4.54	2.18
GO:00057	cytoplasm	Cellular Co	0.31	0.55	Solyc12g0	Solyc12g0	2.29	1.20
GO:00057	cytoplasm	Cellular Co	0.31	0.55	Solyc11g0	Solyc11g0	2.24	1.16
GO:00057	cytoplasm	Cellular Co	0.31	0.55	Solyc09g0	Solyc09g0	0.00	-13.63
GO:00057	cytoplasm	Cellular Co	0.31	0.55	Solyc12g0	Solyc12g0	1818.26	10.83
GO:00057	cytoplasm	Cellular Co	0.31	0.55	Solyc10g0	Solyc10g0	2.14	1.09
GO:00057	cytoplasm	Cellular Co	0.31	0.55	Solyc06g0	Solyc06g0	2.13	1.09
GO:00057	cytoplasm	Cellular Co	0.31	0.55	Solyc09g0	Solyc09g0	25.41	4.67
GO:00057	cytoplasm	Cellular Co	0.31	0.55	Solyc12g0	Solyc12g0	3.17	1.66
GO:00057	cytoplasm	Cellular Co	0.31	0.55	Solyc11g0	Solyc11g0	2.98	1.58
GO:00057	cytoplasm	Cellular Co	0.31	0.55	Solyc03g0	Solyc03g0	794.30	9.63
GO:00057	cytoplasm	Cellular Co	0.31	0.55	Solyc03g1	Solyc03g1	6.29	2.65
GO:00057	cytoplasm	Cellular Co	0.31	0.55	Solyc01g0	Solyc01g0	5.28	2.40
GO:00057	cytoplasm	Cellular Co	0.31	0.55	Solyc12g0	Solyc12g0	3.71	1.89
GO:00057	cytoplasm	Cellular Co	0.31	0.55	Solyc02g0	Solyc02g0	2.27	1.18
GO:00057	cytoplasm	Cellular Co	0.31	0.55	Solyc03g0	Solyc03g0	0.44	-1.17
GO:00057	cytoplasm	Cellular Co	0.31	0.55	Solyc09g0	Solyc09g0	0.00	-8.88
GO:00057	cytoplasm	Cellular Co	0.31	0.55	Solyc02g0	Solyc02g0	4.91	2.30
GO:00057	cytoplasm	Cellular Co	0.31	0.55	Solyc01g0	Solyc01g0	0.00	-9.77
GO:00057	cytoplasm	Cellular Co	0.31	0.55	Solyc07g0	Solyc07g0	2.88	1.52
GO:00057	cytoplasm	Cellular Co	0.31	0.55	Solyc01g0	Solyc01g0	2.50	1.32
GO:00038	3-methyl-2	Molecular	0.31	0.55	Solyc04g0	Solyc04g0	6.11	2.61
GO:00059	mitochond	Cellular Co	0.31	0.55	Solyc04g0	Solyc04g0	6.11	2.61
GO:00090	branched-c	Biological	0.31	0.55	Solyc04g0	Solyc04g0	6.11	2.61
GO:00159	chlorophyl	Biological	0.31	0.55	Solyc04g0	Solyc04g0	3.50	1.81

GO:00057	mitochond	Cellular C	0.31	0.55	Solyc06g0	Solyc06g0	21.01	4.39
GO:00081	peptide-me	Molecular	0.31	0.55	Solyc03g1	E4	2.38	1.25
GO:00091	nucleoside	Biological	0.31	0.55	Solyc09g0	Solyc09g0	21.60	4.43
GO:00096	phototropi	Biological	0.31	0.55	Solyc07g0	Solyc07g0	2.17	1.12
GO:00480	phosphatid	Biological	0.31	0.55	Solyc06g0	Solyc06g0	2.40	1.26
GO:00193	inositol ca	Biological	0.31	0.55	Solyc06g0	Solyc06g0	3.21	1.68
GO:00501	inositol ox	Molecular	0.31	0.55	Solyc06g0	Solyc06g0	3.21	1.68
GO:00093	phospholip	Biological	0.31	0.55	Solyc09g0	Solyc09g0	0.47	-1.07
GO:00704	cellular res	Biological	0.31	0.55	Solyc01g1	Solyc01g1	2.32	1.22
GO:00058	heterotrim	Cellular C	0.31	0.55	Solyc01g1	Solyc01g1	0.44	-1.20
GO:00452	respiratory	Cellular C	0.31	0.55	Solyc11g0	Solyc11g0	2.53	1.34
GO:00066	phosphatid	Biological	0.31	0.55	Solyc06g0	Solyc06g0	0.41	-1.30
GO:00194	glycine de	Biological	0.31	0.55	Solyc06g0	Solyc06g0	2.01	1.01
GO:00102	maintenan	Biological	0.31	0.55	Solyc05g0	Solyc05g0	2.24	1.16
GO:00085	sucrose tra	Molecular	0.31	0.55	Solyc11g0	Solyc11g0	0.49	-1.04
GO:00725	clathrin-de	Biological	0.31	0.55	Solyc04g0	Solyc04g0	2.07	1.05
GO:00067	glutathione	Biological	0.31	0.55	Solyc11g0	Solyc11g0	2.07	1.05
GO:00095	plastid stro	Cellular C	0.31	0.55	Solyc09g0	Solyc09g0	0.12	-3.08
GO:00050	Rho GDP-	Molecular	0.31	0.55	Solyc12g0	Solyc12g0	3.54	1.82
GO:00167	carboxyl- (	Molecular	0.31	0.55	Solyc04g0	Solyc04g0	0.00	-13.91
GO:00198	oxygen bin	Molecular	0.31	0.55	Solyc07g0	Solyc07g0	40.33	5.33
GO:00100	xylem dev	Biological	0.31	0.55	Solyc04g0	Solyc04g0	0.07	-3.79
GO:00055	FK506 bin	Molecular	0.31	0.55	Solyc09g0	Solyc09g0	4.54	2.18
GO:00104	carotenoid	Molecular	0.31	0.55	Solyc05g0	Solyc05g0	39.90	5.32
GO:00161	carotene ca	Biological	0.31	0.55	Solyc05g0	Solyc05g0	39.90	5.32
GO:00301	regulation	Biological	0.31	0.55	Solyc01g0	Solyc01g0	0.40	-1.32
GO:00435	positive re	Biological	0.31	0.55	Solyc07g0	Solyc07g0	2.26	1.18
GO:00427	mitochond	Biological	0.31	0.55	Solyc04g0	Solyc04g0	0.16	-2.66
GO:00517	mannan sy	Molecular	0.31	0.55	Solyc01g0	Solyc01g0	0.19	-2.40
GO:00045	alpha-amy	Molecular	0.31	0.55	Solyc04g0	Solyc04g0	1564.01	10.61
GO:19024	chloride tr	Biological	0.31	0.55	Solyc02g0	Solyc02g0	19.78	4.31
GO:00102	secondary	Biological	0.31	0.55	Solyc08g0	Solyc08g0	0.34	-1.54
GO:00095	photosyste	Cellular C	0.31	0.55	Solyc01g1	psbZ	0.17	-2.52
GO:00082	nucleotida	Molecular	0.31	0.55	Solyc06g0	Solyc06g0	0.38	-1.41
GO:00180	peptidyl-ly	Biological	0.31	0.55	Solyc06g0	Solyc06g0	458.70	8.84
GO:01200	intermemb	Biological	0.31	0.55	Solyc09g0	Solyc09g0	0.00	-8.88
GO:01200	lipid transf	Molecular	0.31	0.55	Solyc09g0	Solyc09g0	0.00	-8.88
GO:00090	L-phenylal	Biological	0.31	0.55	Solyc02g0	Solyc02g0	574.97	9.17
GO:00526	carboxylic	Molecular	0.31	0.55	Solyc02g0	Solyc02g0	0.35	-1.51
GO:01200	proton exp	Biological	0.31	0.55	Solyc12g0	Solyc12g0	4.99	2.32
GO:00336	receptor se	Molecular	0.31	0.55	Solyc08g0	Solyc08g0	4.19	2.07
GO:00053	purine nuc	Molecular	0.31	0.55	Solyc06g0	Solyc06g0	0.44	-1.17
GO:00192	carbohydra	Molecular	0.31	0.55	Solyc08g0	Solyc08g0	0.36	-1.48
GO:00551	obsolete o	Biological	0.31	0.55	Solyc11g0	AOS2	3.20	1.68
GO:00551	obsolete o	Biological	0.31	0.55	Solyc03g0	Solyc03g0	5.61	2.49
GO:00551	obsolete o	Biological	0.31	0.55	Solyc05g0	Solyc05g0	2.81	1.49
GO:00551	obsolete o	Biological	0.31	0.55	Solyc11g0	Solyc11g0	3.46	1.79
GO:00551	obsolete o	Biological	0.31	0.55	Solyc02g0	Solyc02g0	0.48	-1.05
GO:00065	proteolysis	Biological	0.32	0.56	Solyc08g0	Solyc08g0	19.15	4.26
GO:00065	proteolysis	Biological	0.32	0.56	Solyc05g0	Solyc05g0	0.36	-1.47
GO:00065	proteolysis	Biological	0.32	0.56	Solyc03g1	Solyc03g1	2.16	1.11

GO:00065	proteolysis	Biological	0.32	0.56	Solyc04g0	Solyc04g0	2.20	1.14
GO:00065	proteolysis	Biological	0.32	0.56	Solyc01g1	Solyc01g1	0.44	-1.20
GO:00065	proteolysis	Biological	0.32	0.56	Solyc11g0	Solyc11g0	0.29	-1.80
GO:00065	proteolysis	Biological	0.32	0.56	Solyc01g1	Solyc01g1	0.44	-1.18
GO:00065	proteolysis	Biological	0.32	0.56	Solyc10g0	Solyc10g0	2.35	1.23
GO:00065	proteolysis	Biological	0.32	0.56	Solyc01g0	Solyc01g0	2.08	1.05
GO:00065	proteolysis	Biological	0.32	0.56	Solyc02g0	Solyc02g0	0.34	-1.57
GO:00065	proteolysis	Biological	0.32	0.56	Solyc12g0	Solyc12g0	4.50	2.17
GO:00065	proteolysis	Biological	0.32	0.56	Solyc09g0	Solyc09g0	0.41	-1.27
GO:00065	proteolysis	Biological	0.32	0.56	Solyc07g0	Solyc07g0	2.17	1.12
GO:00065	proteolysis	Biological	0.32	0.56	Solyc05g0	Solyc05g0	4.45	2.15
GO:00065	proteolysis	Biological	0.32	0.56	Solyc01g0	Solyc01g0	0.46	-1.12
GO:00065	proteolysis	Biological	0.32	0.56	Solyc01g1	Solyc01g1	2.18	1.13
GO:00065	proteolysis	Biological	0.32	0.56	Solyc09g0	Solyc09g0	0.12	-3.08
GO:00065	proteolysis	Biological	0.32	0.56	Solyc04g0	Solyc04g0	0.43	-1.23
GO:00065	proteolysis	Biological	0.32	0.56	Solyc03g1	Solyc03g1	4459.91	12.12
GO:00065	proteolysis	Biological	0.32	0.56	Solyc09g0	Solyc09g0	3042	11.57
GO:00065	proteolysis	Biological	0.32	0.56	Solyc05g0	Solyc05g0	2.06	1.05
GO:00065	proteolysis	Biological	0.32	0.56	Solyc01g0	Solyc01g0	0.48	-1.06
GO:00065	proteolysis	Biological	0.32	0.56	Solyc03g0	Solyc03g0	3.13	1.65
GO:00065	proteolysis	Biological	0.32	0.56	Solyc12g0	Solyc12g0	2.97	1.57
GO:00065	proteolysis	Biological	0.32	0.56	Solyc12g0	Solyc12g0	1818.26	10.83
GO:00065	proteolysis	Biological	0.32	0.56	Solyc05g0	Solyc05g0	1063.88	10.06
GO:00065	proteolysis	Biological	0.32	0.56	Solyc01g0	Solyc01g0	0.46	-1.12
GO:00065	proteolysis	Biological	0.32	0.56	Solyc12g0	Solyc12g0	0.39	-1.35
GO:00065	proteolysis	Biological	0.32	0.56	Solyc10g0	Solyc10g0	0.02	-5.79
GO:00065	proteolysis	Biological	0.32	0.56	Solyc03g0	Solyc03g0	627.68	9.29
GO:00065	proteolysis	Biological	0.32	0.56	Solyc03g0	Solyc03g0	11.28	3.50
GO:00065	proteolysis	Biological	0.32	0.56	Solyc01g0	Solyc01g0	19.06	4.25
GO:00065	proteolysis	Biological	0.32	0.56	Solyc09g0	Solyc09g0	0.00	-11.10
GO:00065	proteolysis	Biological	0.32	0.56	Solyc06g0	Solyc06g0	0.33	-1.59
GO:00065	proteolysis	Biological	0.32	0.56	Solyc09g0	Solyc09g0	5.06	2.34
GO:00064	RNA catab	Biological	0.32	0.56	Solyc07g0	Solyc07g0	6.72	2.75
GO:00064	RNA catab	Biological	0.32	0.56	Solyc05g0	Solyc05g0	20.96	4.39
GO:00159	chlorophyl	Biological	0.32	0.56	Solyc04g0	Solyc04g0	3.78	1.92
GO:00159	chlorophyl	Biological	0.32	0.56	Solyc12g0	Solyc12g0	6.07	2.60
GO:00313	integral co	Cellular C	0.32	0.56	Solyc06g0	Solyc06g0	21.01	4.39
GO:00313	integral co	Cellular C	0.32	0.56	Solyc06g0	Solyc06g0	0.41	-1.30
GO:00512	spindle ass	Biological	0.32	0.56	Solyc07g0	Solyc07g0	8.41	3.07
GO:00512	spindle ass	Biological	0.32	0.56	Solyc02g0	Solyc02g0	2.08	1.06
GO:00059	sucrose me	Biological	0.32	0.56	Solyc11g0	Solyc11g0	0.49	-1.04
GO:00059	sucrose me	Biological	0.32	0.56	Solyc09g0	Solyc09g0	2.71	1.44
GO:00157	malate trar	Biological	0.32	0.56	Solyc03g1	Solyc03g1	0.39	-1.35
GO:00157	malate trar	Biological	0.32	0.56	Solyc09g0	Solyc09g0	0.26	-1.97
GO:00450	actin nucle	Biological	0.32	0.56	Solyc08g0	Solyc08g0	21.56	4.43
GO:00450	actin nucle	Biological	0.32	0.56	Solyc07g0	Solyc07g0	0.45	-1.16
GO:00082	cell popula	Biological	0.32	0.56	Solyc02g0	Solyc02g0	13.99	3.81
GO:00082	cell popula	Biological	0.32	0.56	Solyc11g0	Solyc11g0	2317.83	11.18
GO:00039	NAD+ nuc	Molecular	0.32	0.56	Solyc02g0	Solyc02g0	0.47	-1.10
GO:00039	NAD+ nuc	Molecular	0.32	0.56	Solyc08g0	Solyc08g0	0.34	-1.57
GO:00039	NAD+ nuc	Molecular	0.32	0.56	Solyc08g0	Solyc08g0	9.19	3.20

GO:00039	NAD+ nuc	Molecular	0.32	0.56	Solyc12g0	Solyc12g0	0.00	-8.12
GO:00301	cell differe	Biological	0.32	0.56	Solyc01g0	Solyc01g0	0.10	-3.35
GO:00301	cell differe	Biological	0.32	0.56	Solyc02g0	Solyc02g0	13.99	3.81
GO:00301	cell differe	Biological	0.32	0.56	Solyc02g0	Solyc02g0	0.06	-4.15
GO:00301	cell differe	Biological	0.32	0.56	Solyc10g0	Solyc10g0	0.26	-1.94
GO:00301	cell differe	Biological	0.32	0.56	Solyc11g0	Solyc11g0	2317.83	11.18
GO:00301	cell differe	Biological	0.32	0.56	Solyc03g0	Solyc03g0	0.00	-9.94
GO:00301	cell differe	Biological	0.32	0.56	Solyc06g0	Solyc06g0	0.08	-3.60
GO:00161	sterol meta	Biological	0.32	0.56	Solyc11g0	AOS2	3.20	1.68
GO:00161	sterol meta	Biological	0.32	0.56	Solyc05g0	Solyc05g0	2.81	1.49
GO:00161	sterol meta	Biological	0.32	0.56	Solyc02g0	Solyc02g0	0.48	-1.05
GO:00009	RNA polyı	Molecular	0.33	0.58	Solyc03g0	Solyc03g0	0.24	-2.06
GO:00009	RNA polyı	Molecular	0.33	0.58	Solyc11g0	Solyc11g0	2.29	1.19
GO:00009	RNA polyı	Molecular	0.33	0.58	Solyc02g0	Solyc02g0	0.42	-1.24
GO:00009	RNA polyı	Molecular	0.33	0.58	Solyc04g0	Solyc04g0	0.07	-3.79
GO:00009	RNA polyı	Molecular	0.33	0.58	Solyc11g0	Solyc11g0	0.00	-10.83
GO:00009	RNA polyı	Molecular	0.33	0.58	Solyc04g0	Solyc04g0	1227.78	10.26
GO:00355	intracellul	Biological	0.34	0.58	Solyc09g0	Solyc09g0	0.32	-1.64
GO:00355	intracellul	Biological	0.34	0.58	Solyc06g0	Solyc06g0	2.40	1.26
GO:00355	intracellul	Biological	0.34	0.58	Solyc01g0	Solyc01g0	0.34	-1.54
GO:00355	intracellul	Biological	0.34	0.58	Solyc12g0	Solyc12g0	2.20	1.14
GO:00355	intracellul	Biological	0.34	0.58	Solyc11g0	Solyc11g0	2.24	1.16
GO:00355	intracellul	Biological	0.34	0.58	Solyc01g0	Solyc01g0	5.28	2.40
GO:00355	intracellul	Biological	0.34	0.58	Solyc12g0	Solyc12g0	3.71	1.89
GO:00355	intracellul	Biological	0.34	0.58	Solyc01g0	Solyc01g0	0.00	-9.77
GO:00101	FMN bind	Molecular	0.34	0.58	Solyc03g0	Solyc03g0	7.65	2.93
GO:00101	FMN bind	Molecular	0.34	0.58	Solyc03g0	Solyc03g0	5.61	2.49
GO:00101	FMN bind	Molecular	0.34	0.58	Solyc03g0	Solyc03g0	2.10	1.07
GO:00101	FMN bind	Molecular	0.34	0.58	Solyc03g1	Solyc03g1	2.22	1.15
GO:00057	lysosome	Cellular C	0.35	0.59	Solyc03g1	Solyc03g1	2.16	1.11
GO:00057	lysosome	Cellular C	0.35	0.59	Solyc04g0	Solyc04g0	2.20	1.14
GO:00316	ubiquitin c	Molecular	0.35	0.59	Solyc03g1	Solyc03g1	0.35	-1.54
GO:00316	ubiquitin c	Molecular	0.35	0.59	Solyc03g0	Solyc03g0	2.92	1.54
GO:00068	amino acid	Biological	0.35	0.59	Solyc05g0	Solyc05g0	2.41	1.27
GO:00068	amino acid	Biological	0.35	0.59	Solyc01g1	Solyc01g1	0.20	-2.35
GO:00168	hydrolase a	Molecular	0.35	0.59	Solyc02g0	Solyc02g0	19.98	4.32
GO:00168	hydrolase a	Molecular	0.35	0.59	Solyc03g0	Solyc03g0	1566.99	10.61
GO:00167	hexosyltra	Molecular	0.35	0.59	Solyc09g0	Solyc09g0	0.05	-4.31
GO:00167	hexosyltra	Molecular	0.35	0.59	Solyc01g1	Solyc01g1	4.75	2.25
GO:00167	hexosyltra	Molecular	0.35	0.59	Solyc12g0	Solyc12g0	7107.62	12.80
GO:00167	hexosyltra	Molecular	0.35	0.59	Solyc11g0	Solyc11g0	0.25	-2.02
GO:00167	hexosyltra	Molecular	0.35	0.59	Solyc07g0	Solyc07g0	6.57	2.72
GO:00167	hexosyltra	Molecular	0.35	0.59	Solyc12g0	Solyc12g0	0.00	-9.95
GO:00167	hexosyltra	Molecular	0.35	0.59	Solyc01g1	Solyc01g1	0.47	-1.07
GO:00167	nucleotidy	Molecular	0.36	0.59	Solyc11g0	Solyc11g0	0.46	-1.13
GO:00167	nucleotidy	Molecular	0.36	0.59	Solyc08g0	Solyc08g0	0.00	-12.86
GO:00167	nucleotidy	Molecular	0.36	0.59	Solyc10g0	Solyc10g0	0.00	-13.39
GO:00167	nucleotidy	Molecular	0.36	0.59	Solyc08g0	Solyc08g0	0.03	-5.23
GO:00167	nucleotidy	Molecular	0.36	0.59	Solyc12g0	Solyc12g0	12.70	3.67
GO:00002	mitotic cel	Biological	0.36	0.59	Solyc05g0	Solyc05g0	0.22	-2.15
GO:00002	mitotic cel	Biological	0.36	0.59	Solyc04g0	Solyc04g0	0.41	-1.28

GO:00002	mitotic cel	Biological	0.36	0.59	Solyc03g0	Solyc03g0	4.18	2.06
GO:00002	mitotic cel	Biological	0.36	0.59	Solyc06g0	Solyc06g0	10.22	3.35
GO:00002	mitotic cel	Biological	0.36	0.59	Solyc02g0	Solyc02g0	4.91	2.30
GO:00465	alpha-L-ar	Molecular	0.36	0.59	Solyc01g1	Solyc01g1	4.83	2.27
GO:00083	thioredoxi	Molecular	0.36	0.59	Solyc10g0	Solyc10g0	4.23	2.08
GO:00516	protein ma	Biological	0.36	0.59	Solyc04g0	Solyc04g0	6.77	2.76
GO:00000	ribosomal	Biological	0.36	0.59	Solyc11g0	Solyc11g0	0.05	-4.30
GO:00059	starch cata	Biological	0.36	0.59	Solyc09g0	Solyc09g0	0.34	-1.54
GO:00166	L-ascorbat	Molecular	0.36	0.59	Solyc11g0	Solyc11g0	8.62	3.11
GO:00328	cellular res	Biological	0.36	0.59	Solyc09g0	Solyc09g0	2.88	1.53
GO:00190	guanyl nuc	Molecular	0.36	0.59	Solyc01g1	Solyc01g1	0.44	-1.20
GO:00308	positive re	Biological	0.36	0.59	Solyc02g0	Solyc02g0	6.74	2.75
GO:00315	actin cytos	Biological	0.36	0.59	Solyc02g0	Solyc02g0	6.74	2.75
GO:00053	nucleoside	Molecular	0.36	0.59	Solyc02g0	Solyc02g0	3.55	1.83
GO:19016	nucleoside	Biological	0.36	0.59	Solyc02g0	Solyc02g0	3.55	1.83
GO:00166	oxidoreduc	Molecular	0.36	0.59	Solyc05g0	Solyc05g0	3.22	1.69
GO:00097	regulation	Biological	0.36	0.59	Solyc03g1	Solyc03g1	2.04	1.03
GO:00065	protein qua	Biological	0.36	0.59	Solyc09g0	Solyc09g0	0.12	-3.08
GO:00343	lipid dropl	Biological	0.36	0.59	Solyc04g0	Solyc04g0	4.53	2.18
GO:00101	farnesyl di	Biological	0.36	0.59	Solyc06g0	Solyc06g0	2.33	1.22
GO:00192	isopenteny	Biological	0.36	0.59	Solyc06g0	Solyc06g0	2.33	1.22
GO:00153	sodium:pro	Molecular	0.36	0.59	Solyc10g0	Solyc10g0	17.76	4.15
GO:00703	phosphatid	Molecular	0.36	0.59	Solyc01g0	Solyc01g0	2.02	1.02
GO:00323	oxidized p	Molecular	0.36	0.59	Solyc09g0	Solyc09g0	2.14	1.10
GO:19015	organonitr	Biological	0.36	0.59	Solyc10g0	Solyc10g0	0.35	-1.51
GO:00421	meiotic DN	Biological	0.36	0.59	Solyc03g1	Solyc03g1	0.47	-1.08
GO:00477	chlorophyl	Molecular	0.36	0.59	Solyc09g0	Solyc09g0	2647.84	11.37
GO:00096	response to	Biological	0.36	0.59	Solyc02g0	Solyc02g0	2.66	1.41
GO:00016	cellular glu	Biological	0.36	0.59	Solyc12g0	Solyc12g0	3.17	1.66
GO:00043	glucokinas	Molecular	0.36	0.59	Solyc12g0	Solyc12g0	3.17	1.66
GO:00043	hexokinase	Molecular	0.36	0.59	Solyc12g0	Solyc12g0	3.17	1.66
GO:00055	glucose bi	Molecular	0.36	0.59	Solyc12g0	Solyc12g0	3.17	1.66
GO:00191	mannokina	Molecular	0.36	0.59	Solyc12g0	Solyc12g0	3.17	1.66
GO:00193	hexose me	Biological	0.36	0.59	Solyc12g0	Solyc12g0	3.17	1.66
GO:00513	spindle pol	Biological	0.36	0.59	Solyc03g0	Solyc03g0	2638.78	11.37
GO:00904	glycerol-3-	Molecular	0.36	0.59	Solyc09g0	Solyc09g0	3.37	1.75
GO:00421	regulation	Biological	0.36	0.59	Solyc01g1	Solyc01g1	0.00	-9.07
GO:00903	regulation	Biological	0.36	0.59	Solyc12g0	Solyc12g0	369.30	8.53
GO:00324	demethyla	Molecular	0.36	0.59	Solyc06g0	Solyc06g0	659.59	9.37
GO:00714	cellular res	Biological	0.36	0.59	Solyc01g1	Solyc01g1	0.37	-1.43
GO:00068	oligopeptic	Biological	0.36	0.60	Solyc07g0	Solyc07g0	2.63	1.40
GO:00068	oligopeptic	Biological	0.36	0.60	Solyc05g0	Solyc05g0	3.13	1.65
GO:00068	oligopeptic	Biological	0.36	0.60	Solyc09g0	Solyc09g0	429.36	8.75
GO:00300	actin cytos	Biological	0.36	0.60	Solyc02g0	Solyc02g0	6.74	2.75
GO:00300	actin cytos	Biological	0.36	0.60	Solyc08g0	Solyc08g0	21.56	4.43
GO:00300	actin cytos	Biological	0.36	0.60	Solyc07g0	Solyc07g0	0.45	-1.16
GO:00081	O-methyltı	Molecular	0.37	0.60	Solyc03g0	Solyc03g0	2.53	1.34
GO:00081	O-methyltı	Molecular	0.37	0.60	Solyc06g0	Solyc06g0	2.05	1.03
GO:00081	O-methyltı	Molecular	0.37	0.60	Solyc10g0	Solyc10g0	2.14	1.10
GO:00081	O-methyltı	Molecular	0.37	0.60	Solyc03g0	Solyc03g0	0.39	-1.35
GO:00081	O-methyltı	Molecular	0.37	0.60	Solyc02g0	Solyc02g0	34.22	5.10

GO:00705	calcium io	Biological	0.37	0.60	Solyc04g0	Solyc04g0	2.70	1.43
GO:00705	calcium io	Biological	0.37	0.60	Solyc06g0	Solyc06g0	2059.41	11.01
GO:00506	obsolete co	Molecular	0.37	0.60	Solyc06g0	Solyc06g0	2.22	1.15
GO:00506	obsolete co	Molecular	0.37	0.60	Solyc09g0	Solyc09g0	3.37	1.75
GO:00101	abaxial cel	Biological	0.37	0.60	Solyc11g0	Solyc11g0	18.46	4.21
GO:00101	abaxial cel	Biological	0.37	0.60	Solyc08g0	Solyc08g0	2486.58	11.28
GO:00462	lignin cata	Biological	0.37	0.60	Solyc05g0	Solyc05g0	0.18	-2.44
GO:00462	lignin cata	Biological	0.37	0.60	Solyc02g0	Solyc02g0	0.22	-2.21
GO:00527	hydroquino	Molecular	0.37	0.60	Solyc05g0	Solyc05g0	0.18	-2.44
GO:00527	hydroquino	Molecular	0.37	0.60	Solyc02g0	Solyc02g0	0.22	-2.21
GO:00460	ATP metab	Biological	0.37	0.60	Solyc06g0	Solyc06g0	15.51	3.95
GO:00460	ATP metab	Biological	0.37	0.60	Solyc11g0	Solyc11g0	0.22	-2.19
GO:00071	signal tran	Biological	0.38	0.61	Solyc09g0	Solyc09g0	0.23	-2.09
GO:00071	signal tran	Biological	0.38	0.61	Solyc04g0	Solyc04g0	2.35	1.23
GO:00071	signal tran	Biological	0.38	0.61	Solyc02g0	Solyc02g0	0.47	-1.10
GO:00071	signal tran	Biological	0.38	0.61	Solyc06g0	Solyc06g0	2.40	1.26
GO:00071	signal tran	Biological	0.38	0.61	Solyc01g1	Solyc01g1	0.44	-1.20
GO:00071	signal tran	Biological	0.38	0.61	Solyc08g0	Solyc08g0	0.34	-1.57
GO:00071	signal tran	Biological	0.38	0.61	Solyc07g0	Solyc07g0	2.15	1.11
GO:00071	signal tran	Biological	0.38	0.61	Solyc11g0	Solyc11g0	0.46	-1.13
GO:00071	signal tran	Biological	0.38	0.61	Solyc04g0	Solyc04g0	0.41	-1.30
GO:00071	signal tran	Biological	0.38	0.61	Solyc06g0	Solyc06g0	0.43	-1.23
GO:00071	signal tran	Biological	0.38	0.61	Solyc02g0	Solyc02g0	2.27	1.18
GO:00071	signal tran	Biological	0.38	0.61	Solyc02g0	Solyc02g0	13.99	3.81
GO:00071	signal tran	Biological	0.38	0.61	Solyc08g0	Solyc08g0	9.19	3.20
GO:00071	signal tran	Biological	0.38	0.61	Solyc09g0	Solyc09g0	0.34	-1.58
GO:00071	signal tran	Biological	0.38	0.61	Solyc12g0	Solyc12g0	0.00	-8.12
GO:00071	signal tran	Biological	0.38	0.61	Solyc07g0	Solyc07g0	2.88	1.52
GO:00071	signal tran	Biological	0.38	0.61	Solyc11g0	Solyc11g0	2317.83	11.18
GO:00071	signal tran	Biological	0.38	0.61	Solyc06g0	Solyc06g0	9.89	3.31
GO:00071	signal tran	Biological	0.38	0.61	Solyc01g0	Solyc01g0	2.50	1.32
GO:00069	response to	Biological	0.38	0.62	Solyc11g0	Solyc11g0	8.62	3.11
GO:00069	response to	Biological	0.38	0.62	Solyc01g0	Solyc01g0	14.73	3.88
GO:00069	response to	Biological	0.38	0.62	Solyc02g0	Solyc02g0	8.87	3.15
GO:00069	response to	Biological	0.38	0.62	Solyc02g0	Solyc02g0	2.54	1.34
GO:00069	response to	Biological	0.38	0.62	Solyc03g0	Solyc03g0	5511.05	12.43
GO:00069	response to	Biological	0.38	0.62	Solyc10g0	Solyc10g0	1604.71	10.65
GO:00069	response to	Biological	0.38	0.62	Solyc01g1	Solyc01g1	4.37	2.13
GO:00069	response to	Biological	0.38	0.62	Solyc02g0	Solyc02g0	0.17	-2.56
GO:00069	response to	Biological	0.38	0.62	Solyc03g0	Solyc03g0	0.41	-1.30
GO:00069	response to	Biological	0.38	0.62	Solyc04g0	Solyc04g0	0.48	-1.06
GO:00069	response to	Biological	0.38	0.62	Solyc06g0	Solyc06g0	0.25	-2.01
GO:00069	response to	Biological	0.38	0.62	Solyc01g0	Solyc01g0	0.48	-1.07
GO:00069	response to	Biological	0.38	0.62	Solyc07g0	Solyc07g0	2.87	1.52
GO:00021	cytoplasmi	Biological	0.38	0.62	Solyc09g0	Solyc09g0	4.17	2.06
GO:00021	cytoplasmi	Biological	0.38	0.62	Solyc05g0	Solyc05g0	0.09	-3.44
GO:00021	cytoplasmi	Biological	0.38	0.62	Solyc09g0	Solyc09g0	0.47	-1.10
GO:00061	nucleobase	Biological	0.39	0.62	Solyc09g0	Solyc09g0	0.40	-1.34
GO:00061	nucleobase	Biological	0.39	0.62	Solyc09g0	Solyc09g0	6.17	2.63
GO:00061	nucleobase	Biological	0.39	0.62	Solyc01g1	Solyc01g1	2.98	1.58
GO:00061	nucleobase	Biological	0.39	0.62	Solyc12g0	Solyc12g0	2.29	1.20

GO:00058	microtubul	Cellular C	0.39	0.62	Solyc09g0	Solyc09g0	0.05	-4.40
GO:00058	microtubul	Cellular C	0.39	0.62	Solyc02g0	Solyc02g0	2.08	1.06
GO:00058	microtubul	Cellular C	0.39	0.62	Solyc07g0	Solyc07g0	3.21	1.68
GO:00058	microtubul	Cellular C	0.39	0.62	Solyc03g1	Solyc03g1	2.39	1.26
GO:00058	microtubul	Cellular C	0.39	0.62	Solyc09g0	Solyc09g0	0.07	-3.89
GO:00058	microtubul	Cellular C	0.39	0.62	Solyc12g0	Solyc12g0	467.31	8.87
GO:00058	microtubul	Cellular C	0.39	0.62	Solyc09g0	Solyc09g0	0.35	-1.53
GO:00704	respirasom	Cellular C	0.40	0.62	Solyc08g0	Solyc08g0	0.14	-2.87
GO:00704	respirasom	Cellular C	0.40	0.62	Solyc11g0	Solyc11g0	0.09	-3.52
GO:00166	oxidoreduc	Molecular	0.40	0.62	Solyc07g0	Solyc07g0	0.26	-1.95
GO:00166	oxidoreduc	Molecular	0.40	0.62	Solyc12g0	Solyc12g0	0.30	-1.74
GO:00022	defense res	Biological	0.40	0.62	Solyc05g0	Solyc05g0	4.51	2.17
GO:00022	defense res	Biological	0.40	0.62	Solyc10g0	Solyc10g0	4.22	2.08
GO:00302	enzyme reg	Molecular	0.40	0.62	Solyc03g0	Solyc03g0	2638.78	11.37
GO:00302	enzyme reg	Molecular	0.40	0.62	Solyc01g1	Solyc01g1	0.00	-9.07
GO:00090	xylan 1,4-t	Molecular	0.41	0.62	Solyc01g1	Solyc01g1	4.83	2.27
GO:20010	starch bind	Molecular	0.41	0.62	Solyc06g0	Solyc06g0	2.52	1.33
GO:00000	ribosomal	Biological	0.41	0.62	Solyc11g0	Solyc11g0	0.05	-4.30
GO:00096	jasmonic a	Biological	0.41	0.62	Solyc11g0	AOS2	3.20	1.68
GO:00067	porphyrin-	Biological	0.41	0.62	Solyc04g0	Solyc04g0	3.78	1.92
GO:00336	mitochond	Biological	0.41	0.62	Solyc06g0	Solyc06g0	21.01	4.39
GO:00191	cytokinin c	Molecular	0.41	0.62	Solyc10g0	Solyc10g0	3.13	1.64
GO:00165	histone mc	Biological	0.41	0.62	Solyc09g0	Solyc09g0	0.18	-2.45
GO:00165	Cdc73/Paf	Cellular C	0.41	0.62	Solyc09g0	Solyc09g0	0.18	-2.45
GO:00168	endoribonu	Molecular	0.41	0.62	Solyc11g0	Solyc11g0	0.41	-1.28
GO:00527	pectin acet	Molecular	0.41	0.62	Solyc01g1	Solyc01g1	3.25	1.70
GO:00069	organelle c	Biological	0.41	0.62	Solyc07g0	Solyc07g0	2.04	1.03
GO:00474	nucleoside	Molecular	0.41	0.62	Solyc09g0	Solyc09g0	21.60	4.43
GO:00044	phosphatid	Molecular	0.41	0.62	Solyc06g0	Solyc06g0	2.40	1.26
GO:00062	'de novo' p	Biological	0.41	0.62	Solyc01g0	Solyc01g0	0.32	-1.65
GO:00058	nuclear mi	Cellular C	0.41	0.62	Solyc07g0	Solyc07g0	8.41	3.07
GO:00070	centrosome	Biological	0.41	0.62	Solyc07g0	Solyc07g0	8.41	3.07
GO:00161	xanthophy	Biological	0.41	0.62	Solyc02g0	Solyc02g0	0.45	-1.14
GO:00316	G-protein l	Molecular	0.41	0.62	Solyc01g1	Solyc01g1	0.44	-1.20
GO:00044	malic enzy	Molecular	0.41	0.62	Solyc12g0	Solyc12g0	2.01	1.01
GO:00044	malate deh	Molecular	0.41	0.62	Solyc12g0	Solyc12g0	2.01	1.01
GO:01406	ATP-deper	Molecular	0.41	0.62	Solyc06g0	Solyc06g0	2.09	1.07
GO:00485	photosyste	Biological	0.41	0.62	Solyc02g0	Solyc02g0	2.62	1.39
GO:00001	3'-5'-exorit	Molecular	0.41	0.62	Solyc06g0	Solyc06g0	0.31	-1.69
GO:00098	pollen gern	Biological	0.41	0.62	Solyc01g0	Solyc01g0	0.49	-1.04
GO:00081	ferric iron	Molecular	0.41	0.62	Solyc03g0	Solyc03g0	0.22	-2.16
GO:00060	mannose n	Biological	0.41	0.62	Solyc02g0	Solyc02g0	2.97	1.57
GO:00094	response tc	Biological	0.41	0.62	Solyc01g1	Solyc01g1	14.86	3.89
GO:00312	replication	Cellular C	0.41	0.62	Solyc04g0	Solyc04g0	2.35	1.23
GO:00198	ATPase-cc	Molecular	0.41	0.62	Solyc08g0	Solyc08g0	2.67	1.41
GO:00060	inositol bic	Biological	0.41	0.62	Solyc04g0	Solyc04g0	0.41	-1.30
GO:00068	cellular cal	Biological	0.41	0.62	Solyc06g0	Solyc06g0	2059.41	11.01
GO:20000	regulation	Biological	0.41	0.62	Solyc03g1	Solyc03g1	0.06	-4.16
GO:00053	iron ion tra	Molecular	0.41	0.62	Solyc04g0	Solyc04g0	3.05	1.61
GO:00068	intracellula	Biological	0.41	0.62	Solyc04g0	Solyc04g0	3.05	1.61
GO:00508	protein sta	Biological	0.41	0.62	Solyc07g0	Solyc07g0	2.47	1.30

GO:00063	DNA-temp	Biological	0.41	0.62	Solyc04g0	Solyc04g0	0.34	-1.57
GO:00047	protein ser	Molecular	0.41	0.62	Solyc08g0	Solyc08g0	0.41	-1.30
GO:00039	RNA-direc	Molecular	0.41	0.62	Solyc08g0	Solyc08g0	3.18	1.67
GO:00039	DNA-(apu	Molecular	0.41	0.62	Solyc09g0	Solyc09g0	2.29	1.20
GO:00487	root hair el	Biological	0.41	0.62	Solyc12g0	Solyc12g0	369.30	8.53
GO:00097	negative re	Biological	0.41	0.62	Solyc01g0	Solyc01g0	0.37	-1.43
GO:00085	ammonium	Molecular	0.41	0.62	Solyc09g0	Solyc09g0	0.00	-8.11
GO:00724	ammonium	Biological	0.41	0.62	Solyc09g0	Solyc09g0	0.00	-8.11
GO:00070	actin filam	Biological	0.42	0.65	Solyc02g0	Solyc02g0	6.74	2.75
GO:00070	actin filam	Biological	0.42	0.65	Solyc10g0	Solyc10g0	2.14	1.10
GO:01101	cellular an	Cellular Co	0.42	0.65	Solyc11g0	Solyc11g0	3.66	1.87
GO:01101	cellular an	Cellular Co	0.42	0.65	Solyc07g0	Solyc07g0	2.40	1.26
GO:00048	enzyme inh	Molecular	0.43	0.66	Solyc12g0	Solyc12g0	2.75	1.46
GO:00048	enzyme inh	Molecular	0.43	0.66	Solyc12g0	Solyc12g0	2.22	1.15
GO:00048	enzyme inh	Molecular	0.43	0.66	Solyc12g0	Solyc12g0	2.15	1.10
GO:00048	enzyme inh	Molecular	0.43	0.66	Solyc03g1	Solyc03g1	5.44	2.44
GO:00048	enzyme inh	Molecular	0.43	0.66	Solyc03g1	Solyc03g1	0.44	-1.20
GO:00048	enzyme inh	Molecular	0.43	0.66	Solyc07g0	Solyc07g0	3.98	1.99
GO:00048	enzyme inh	Molecular	0.43	0.66	Solyc07g0	Solyc07g0	3.11	1.64
GO:00046	protein ser	Molecular	0.44	0.66	Solyc09g0	Solyc09g0	0.32	-1.64
GO:00046	protein ser	Molecular	0.44	0.66	Solyc03g0	Solyc03g0	3.18	1.67
GO:00046	protein ser	Molecular	0.44	0.66	Solyc02g0	Solyc02g0	0.44	-1.19
GO:00046	protein ser	Molecular	0.44	0.66	Solyc07g0	Solyc07g0	2.15	1.11
GO:00046	protein ser	Molecular	0.44	0.66	Solyc05g0	Solyc05g0	0.43	-1.22
GO:00046	protein ser	Molecular	0.44	0.66	Solyc01g0	Solyc01g0	2.56	1.36
GO:00046	protein ser	Molecular	0.44	0.66	Solyc11g0	Solyc11g0	0.46	-1.13
GO:00046	protein ser	Molecular	0.44	0.66	Solyc12g0	Solyc12g0	2.20	1.14
GO:00046	protein ser	Molecular	0.44	0.66	Solyc10g0	Solyc10g0	0.08	-3.68
GO:00046	protein ser	Molecular	0.44	0.66	Solyc04g0	Solyc04g0	0.46	-1.11
GO:00046	protein ser	Molecular	0.44	0.66	Solyc03g0	Solyc03g0	2.40	1.26
GO:00046	protein ser	Molecular	0.44	0.66	Solyc08g0	Solyc08g0	0.41	-1.30
GO:00046	protein ser	Molecular	0.44	0.66	Solyc02g0	Solyc02g0	307.12	8.26
GO:00046	protein ser	Molecular	0.44	0.66	Solyc02g0	Solyc02g0	2.27	1.18
GO:00046	protein ser	Molecular	0.44	0.66	Solyc03g1	Solyc03g1	0.06	-4.15
GO:00046	protein ser	Molecular	0.44	0.66	Solyc07g0	Solyc07g0	2.39	1.26
GO:00046	protein ser	Molecular	0.44	0.66	Solyc01g0	Solyc01g0	0.00	-9.25
GO:00046	protein ser	Molecular	0.44	0.66	Solyc08g0	Solyc08g0	4.19	2.07
GO:00046	protein ser	Molecular	0.44	0.66	Solyc02g0	Solyc02g0	394.05	8.62
GO:00046	protein ser	Molecular	0.44	0.66	Solyc03g0	Solyc03g0	0.36	-1.46
GO:00046	protein ser	Molecular	0.44	0.66	Solyc07g0	Solyc07g0	2.88	1.52
GO:00046	protein ser	Molecular	0.44	0.66	Solyc02g0	Solyc02g0	517.85	9.02
GO:00046	protein ser	Molecular	0.44	0.66	Solyc07g0	Solyc07g0	0.39	-1.35
GO:00046	protein ser	Molecular	0.44	0.66	Solyc03g0	Solyc03g0	0.39	-1.37
GO:00046	protein ser	Molecular	0.44	0.66	Solyc12g0	Solyc12g0	11.23	3.49
GO:00301	manganese	Molecular	0.44	0.66	Solyc07g0	Solyc07g0	0.35	-1.51
GO:00301	manganese	Molecular	0.44	0.66	Solyc10g0	Solyc10g0	2.93	1.55
GO:00301	manganese	Molecular	0.44	0.66	Solyc12g0	Solyc12g0	1818.26	10.83
GO:00310	gene silenc	Biological	0.44	0.66	Solyc09g0	Solyc09g0	2.34	1.23
GO:00310	gene silenc	Biological	0.44	0.66	Solyc02g0	Solyc02g0	0.49	-1.04
GO:00310	gene silenc	Biological	0.44	0.66	Solyc06g0	Solyc06g0	2.07	1.05
GO:00454	xylan catal	Biological	0.45	0.66	Solyc01g1	Solyc01g1	4.83	2.27

GO:00165	cytidine to	Biological	0.45	0.66	Solyc05g0	Solyc05g0	19.89	4.31
GO:00090	isoleucine	Biological	0.45	0.66	Solyc03g0	Solyc03g0	4.05	2.02
GO:00090	methionine	Biological	0.45	0.66	Solyc09g0	Solyc09g0	6.99	2.80
GO:00102	response to	Biological	0.45	0.66	Solyc02g0	Solyc02g0	0.08	-3.73
GO:00453	acyl-[acyl-	Molecular	0.45	0.66	Solyc11g0	Solyc11g0	3.25	1.70
GO:00300	L-malate d	Molecular	0.45	0.66	Solyc11g0	Solyc11g0	4.12	2.04
GO:00463	mannan ca	Biological	0.45	0.66	Solyc10g0	Solyc10g0	2.91	1.54
GO:00169	poly(A)+ r	Biological	0.45	0.66	Solyc12g0	Solyc12g0	2.04	1.03
GO:00350	methylated	Molecular	0.45	0.66	Solyc08g0	Solyc08g0	2.38	1.25
GO:00451	cell-cell si	Biological	0.45	0.66	Solyc07g0	Solyc07g0	11.30	3.50
GO:00431	3'-5' DNA	Molecular	0.45	0.66	Solyc01g1	Solyc01g1	2.32	1.22
GO:00191	transmemb	Molecular	0.45	0.66	Solyc02g0	Solyc02g0	0.38	-1.41
GO:00164	cell migrat	Biological	0.45	0.66	Solyc02g0	Solyc02g0	6.74	2.75
GO:00164	RISC comp	Cellular C	0.45	0.66	Solyc07g0	Solyc07g0	0.46	-1.11
GO:00058	microtubul	Cellular C	0.45	0.66	Solyc02g0	Solyc02g0	2.08	1.06
GO:00511	ATPase bi	Molecular	0.45	0.66	Solyc09g0	Solyc09g0	0.12	-3.08
GO:00093	acetyl-CoA	Cellular C	0.45	0.66	Solyc04g0	Solyc04g0	0.00	-13.91
GO:00102	photosyste	Biological	0.45	0.66	Solyc09g0	Solyc09g0	4.23	2.08
GO:00053	copper ion	Molecular	0.45	0.66	Solyc06g0	Solyc06g0	0.43	-1.22
GO:00354	copper ion	Biological	0.45	0.66	Solyc06g0	Solyc06g0	0.43	-1.22
GO:00036	DNA repli	Molecular	0.45	0.66	Solyc05g0	Solyc05g0	2.54	1.35
GO:00974	protein ma	Biological	0.45	0.66	Solyc05g0	Solyc05g0	2.96	1.57
GO:00153	calcium:pr	Molecular	0.45	0.66	Solyc06g0	Solyc06g0	2059.41	11.01
GO:00421	plastid me	Cellular C	0.45	0.66	Solyc09g0	Solyc09g0	0.00	-13.14
GO:00101	cutin biosy	Biological	0.45	0.66	Solyc09g0	Solyc09g0	3.37	1.75
GO:00162	protein-lys	Molecular	0.45	0.66	Solyc06g0	Solyc06g0	458.70	8.84
GO:00058	cis-Golgi r	Cellular C	0.45	0.66	Solyc01g0	Solyc01g0	2335.77	11.19
GO:00320	regulation	Biological	0.45	0.66	Solyc02g0	Solyc02g0	2.47	1.31
GO:00465	polyamine	Molecular	0.45	0.66	Solyc01g0	Solyc01g0	0.03	-4.98
GO:00066	steroid bio	Biological	0.45	0.66	Solyc02g0	Solyc02g0	0.30	-1.71
GO:00066	steroid bio	Biological	0.45	0.66	Solyc04g0	Solyc04g0	2.31	1.21
GO:00060	glucose me	Biological	0.45	0.66	Solyc04g0	Solyc04g0	8.55	3.10
GO:00060	glucose me	Biological	0.45	0.66	Solyc02g0	Solyc02g0	2.27	1.18
GO:00045	endonucle	Molecular	0.45	0.66	Solyc05g0	Solyc05g0	2.32	1.21
GO:00045	endonucle	Molecular	0.45	0.66	Solyc05g0	Solyc05g0	20.96	4.39
GO:00102	brassinoste	Biological	0.45	0.66	Solyc05g0	Solyc05g0	2.81	1.49
GO:00102	brassinoste	Biological	0.45	0.66	Solyc02g0	Solyc02g0	0.48	-1.05
GO:00098	plant-type	Biological	0.45	0.66	Solyc12g0	Solyc12g0	3.03	1.60
GO:00098	plant-type	Biological	0.45	0.66	Solyc01g0	Solyc01g0	0.19	-2.40
GO:00094	response to	Biological	0.46	0.68	Solyc04g0	Solyc04g0	2.70	1.43
GO:00094	response to	Biological	0.46	0.68	Solyc12g0	Solyc12g0	2.97	1.57
GO:00094	response to	Biological	0.46	0.68	Solyc09g0	Solyc09g0	25.41	4.67
GO:00043	glutathione	Molecular	0.47	0.68	Solyc09g0	Solyc09g0	2.37	1.24
GO:00043	glutathione	Molecular	0.47	0.68	Solyc09g0	Solyc09g0	2.07	1.05
GO:00043	glutathione	Molecular	0.47	0.68	Solyc06g0	Solyc06g0	0.15	-2.74
GO:00043	glutathione	Molecular	0.47	0.68	Solyc05g0	Solyc05g0	7.28	2.86
GO:00435	sequence-s	Molecular	0.47	0.68	Solyc09g0	Solyc09g0	6.00	2.58
GO:00435	sequence-s	Molecular	0.47	0.68	Solyc03g0	Solyc03g0	0.24	-2.06
GO:00435	sequence-s	Molecular	0.47	0.68	Solyc11g0	Solyc11g0	0.36	-1.47
GO:00435	sequence-s	Molecular	0.47	0.68	Solyc04g0	Solyc04g0	2.73	1.45
GO:00435	sequence-s	Molecular	0.47	0.68	Solyc02g0	Solyc02g0	2.17	1.12

GO:00435	sequence-s	Molecular	0.47	0.68	Solyc09g0	Solyc09g0	0.32	-1.63
GO:00435	sequence-s	Molecular	0.47	0.68	Solyc06g0	Solyc06g0	0.46	-1.11
GO:00435	sequence-s	Molecular	0.47	0.68	Solyc01g0	Solyc01g0	0.10	-3.35
GO:00435	sequence-s	Molecular	0.47	0.68	Solyc01g0	Solyc01g0	0.44	-1.18
GO:00435	sequence-s	Molecular	0.47	0.68	Solyc02g0	Solyc02g0	0.42	-1.24
GO:00435	sequence-s	Molecular	0.47	0.68	Solyc09g0	Solyc09g0	0.44	-1.19
GO:00435	sequence-s	Molecular	0.47	0.68	Solyc05g0	Solyc05g0	4.20	2.07
GO:00435	sequence-s	Molecular	0.47	0.68	Solyc02g0	Solyc02g0	0.32	-1.64
GO:00435	sequence-s	Molecular	0.47	0.68	Solyc06g0	Solyc06g0	0.35	-1.53
GO:00435	sequence-s	Molecular	0.47	0.68	Solyc02g0	Solyc02g0	0.42	-1.26
GO:00435	sequence-s	Molecular	0.47	0.68	Solyc10g0	Solyc10g0	1081.58	10.08
GO:00435	sequence-s	Molecular	0.47	0.68	Solyc06g0	Solyc06g0	2.01	1.01
GO:00435	sequence-s	Molecular	0.47	0.68	Solyc04g0	Solyc04g0	0.07	-3.79
GO:00435	sequence-s	Molecular	0.47	0.68	Solyc11g0	Solyc11g0	0.00	-10.83
GO:00435	sequence-s	Molecular	0.47	0.68	Solyc02g0	Solyc02g0	25.47	4.67
GO:00435	sequence-s	Molecular	0.47	0.68	Solyc02g0	Solyc02g0	2.66	1.41
GO:00435	sequence-s	Molecular	0.47	0.68	Solyc03g1	Solyc03g1	0.06	-4.16
GO:00435	sequence-s	Molecular	0.47	0.68	Solyc12g0	Solyc12g0	2529.18	11.30
GO:00435	sequence-s	Molecular	0.47	0.68	Solyc03g0	Solyc03g0	20.56	4.36
GO:00435	sequence-s	Molecular	0.47	0.68	Solyc06g0	Solyc06g0	0.00	-12.03
GO:00435	sequence-s	Molecular	0.47	0.68	Solyc02g0	Solyc02g0	8.19	3.03
GO:00435	sequence-s	Molecular	0.47	0.68	Solyc09g0	Solyc09g0	2135.62	11.06
GO:00435	sequence-s	Molecular	0.47	0.68	Solyc04g0	Solyc04g0	0.00	-12.04
GO:00435	sequence-s	Molecular	0.47	0.68	Solyc02g0	Solyc02g0	1446.12	10.50
GO:00435	sequence-s	Molecular	0.47	0.68	Solyc02g0	Solyc02g0	0.06	-4.15
GO:00435	sequence-s	Molecular	0.47	0.68	Solyc01g0	Solyc01g0	0.48	-1.06
GO:00435	sequence-s	Molecular	0.47	0.68	Solyc02g0	Solyc02g0	11.00	3.46
GO:00435	sequence-s	Molecular	0.47	0.68	Solyc10g0	Solyc10g0	0.26	-1.94
GO:00435	sequence-s	Molecular	0.47	0.68	Solyc02g0	Solyc02g0	15.79	3.98
GO:00435	sequence-s	Molecular	0.47	0.68	Solyc06g0	Solyc06g0	0.45	-1.16
GO:00435	sequence-s	Molecular	0.47	0.68	Solyc08g0	Solyc08g0	0.41	-1.27
GO:00435	sequence-s	Molecular	0.47	0.68	Solyc03g0	Solyc03g0	0.00	-9.94
GO:00435	sequence-s	Molecular	0.47	0.68	Solyc06g0	Solyc06g0	0.08	-3.60
GO:00435	sequence-s	Molecular	0.47	0.68	Solyc11g0	Solyc11g0	0.00	-9.22
GO:00056	intracellula	Cellular Co	0.47	0.68	Solyc09g0	Solyc09g0	0.32	-1.64
GO:00056	intracellula	Cellular Co	0.47	0.68	Solyc01g0	Solyc01g0	3.08	1.62
GO:00056	intracellula	Cellular Co	0.47	0.68	Solyc06g0	Solyc06g0	2.40	1.26
GO:00056	intracellula	Cellular Co	0.47	0.68	Solyc12g0	Solyc12g0	2.35	1.23
GO:00056	intracellula	Cellular Co	0.47	0.68	Solyc12g0	Solyc12g0	2.04	1.03
GO:00056	intracellula	Cellular Co	0.47	0.68	Solyc06g0	Solyc06g0	2.20	1.14
GO:00056	intracellula	Cellular Co	0.47	0.68	Solyc12g0	Solyc12g0	2.20	1.14
GO:00056	intracellula	Cellular Co	0.47	0.68	Solyc08g0	Solyc08g0	2.11	1.08
GO:00056	intracellula	Cellular Co	0.47	0.68	Solyc09g0	Solyc09g0	0.44	-1.20
GO:00055	calmodulin	Molecular	0.47	0.68	Solyc10g0	Solyc10g0	3.31	1.73
GO:00055	calmodulin	Molecular	0.47	0.68	Solyc01g1	Solyc01g1	0.45	-1.14
GO:00055	calmodulin	Molecular	0.47	0.68	Solyc09g0	Solyc09g0	0.44	-1.19
GO:00055	calmodulin	Molecular	0.47	0.68	Solyc10g0	Solyc10g0	0.40	-1.31
GO:00055	calmodulin	Molecular	0.47	0.68	Solyc12g0	Solyc12g0	4.02	2.01
GO:00066	glycerol et	Biological	0.47	0.68	Solyc04g0	Solyc04g0	2.43	1.28
GO:00066	glycerol et	Biological	0.47	0.68	Solyc10g0	Solyc10g0	2.07	1.05
GO:00080	phosphoric	Molecular	0.47	0.68	Solyc06g0	Solyc06g0	2.40	1.26

GO:00080	phosphoric	Molecular	0.47	0.68	Solyc12g0	Solyc12g0	2.04	1.03
GO:00058	centrosome	Cellular C	0.47	0.68	Solyc07g0	Solyc07g0	8.41	3.07
GO:00058	centrosome	Cellular C	0.47	0.68	Solyc01g1	Solyc01g1	2.18	1.13
GO:00104	abscisic ac	Molecular	0.47	0.68	Solyc09g0	Solyc09g0	0.27	-1.90
GO:00104	abscisic ac	Molecular	0.47	0.68	Solyc09g0	Solyc09g0	8.11	3.02
GO:00062	mismatch r	Biological	0.47	0.68	Solyc01g0	Solyc01g0	0.49	-1.04
GO:00062	mismatch r	Biological	0.47	0.68	Solyc09g0	Solyc09g0	2.14	1.10
GO:00159	small ribos	Cellular C	0.48	0.68	Solyc12g0	Solyc12g0	0.16	-2.65
GO:00159	small ribos	Cellular C	0.48	0.68	Solyc01g0	Solyc01g0	0.08	-3.59
GO:00159	small ribos	Cellular C	0.48	0.68	Solyc09g0	Solyc09g0	0.25	-2.00
GO:00069	defense res	Biological	0.48	0.68	Solyc11g0	AOS2	3.20	1.68
GO:00069	defense res	Biological	0.48	0.68	Solyc04g0	Solyc04g0	2.73	1.45
GO:00069	defense res	Biological	0.48	0.68	Solyc10g0	CHI9	0.28	-1.84
GO:00069	defense res	Biological	0.48	0.68	Solyc02g0	Solyc02g0	0.47	-1.10
GO:00069	defense res	Biological	0.48	0.68	Solyc05g0	Solyc05g0	2.40	1.27
GO:00069	defense res	Biological	0.48	0.68	Solyc10g0	Solyc10g0	0.39	-1.34
GO:00069	defense res	Biological	0.48	0.68	Solyc10g0	Solyc10g0	3.31	1.73
GO:00069	defense res	Biological	0.48	0.68	Solyc09g0	Solyc09g0	0.48	-1.06
GO:00069	defense res	Biological	0.48	0.68	Solyc05g0	Solyc05g0	4.51	2.17
GO:00069	defense res	Biological	0.48	0.68	Solyc10g0	Solyc10g0	0.39	-1.36
GO:00069	defense res	Biological	0.48	0.68	Solyc02g0	Solyc02g0	0.33	-1.59
GO:00069	defense res	Biological	0.48	0.68	Solyc08g0	Solyc08g0	0.34	-1.57
GO:00069	defense res	Biological	0.48	0.68	Solyc07g0	Solyc07g0	61.65	5.95
GO:00069	defense res	Biological	0.48	0.68	Solyc01g0	Solyc01g0	0.49	-1.03
GO:00069	defense res	Biological	0.48	0.68	Solyc08g0	Solyc08g0	0.35	-1.51
GO:00069	defense res	Biological	0.48	0.68	Solyc09g0	Solyc09g0	8.11	3.02
GO:00069	defense res	Biological	0.48	0.68	Solyc04g0	Solyc04g0	3.89	1.96
GO:00069	defense res	Biological	0.48	0.68	Solyc06g0	Solyc06g0	0.39	-1.37
GO:00069	defense res	Biological	0.48	0.68	Solyc12g0	Solyc12g0	0.33	-1.61
GO:00069	defense res	Biological	0.48	0.68	Solyc10g0	Solyc10g0	4.22	2.08
GO:00069	defense res	Biological	0.48	0.68	Solyc08g0	Solyc08g0	0.42	-1.25
GO:00069	defense res	Biological	0.48	0.68	Solyc03g1	Solyc03g1	2361.02	11.21
GO:00069	defense res	Biological	0.48	0.68	Solyc01g0	Solyc01g0	0.43	-1.23
GO:00069	defense res	Biological	0.48	0.68	Solyc01g0	Solyc01g0	0.31	-1.69
GO:00069	defense res	Biological	0.48	0.68	Solyc03g1	Solyc03g1	734.02	9.52
GO:00069	defense res	Biological	0.48	0.68	Solyc01g0	Solyc01g0	2.10	1.07
GO:00069	defense res	Biological	0.48	0.68	Solyc07g0	Solyc07g0	0.38	-1.40
GO:00069	defense res	Biological	0.48	0.68	Solyc02g0	Solyc02g0	0.35	-1.51
GO:00069	defense res	Biological	0.48	0.68	Solyc05g0	Solyc05g0	0.41	-1.28
GO:00069	defense res	Biological	0.48	0.68	Solyc12g0	Solyc12g0	0.00	-8.12
GO:00069	defense res	Biological	0.48	0.68	Solyc06g0	PTI6	0.43	-1.22
GO:00069	defense res	Biological	0.48	0.68	Solyc05g0	Solyc05g0	0.36	-1.48
GO:00069	defense res	Biological	0.48	0.68	Solyc09g0	Solyc09g0	362.34	8.50
GO:00069	defense res	Biological	0.48	0.68	Solyc12g0	Solyc12g0	0.00	-10.19
GO:00060	glycolytic	Biological	0.49	0.68	Solyc09g0	Solyc09g0	2.88	1.53
GO:00060	glycolytic	Biological	0.49	0.68	Solyc08g0	Solyc08g0	3.48	1.80
GO:00060	glycolytic	Biological	0.49	0.68	Solyc12g0	Solyc12g0	3.17	1.66
GO:00060	glycolytic	Biological	0.49	0.68	Solyc04g0	Solyc04g0	0.41	-1.27
GO:00060	glycolytic	Biological	0.49	0.68	Solyc02g0	Solyc02g0	3.52	1.82
GO:00068	lipid transp	Biological	0.49	0.68	Solyc02g0	Solyc02g0	9.57	3.26
GO:00068	lipid transp	Biological	0.49	0.68	Solyc01g0	Solyc01g0	44322.86	15.44

GO:00068	lipid trans	Biological	0.49	0.68	Solyc03g0	Solyc03g0	0.23	-2.14
GO:00068	lipid trans	Biological	0.49	0.68	Solyc06g0	Solyc06g0	0.29	-1.80
GO:00068	lipid trans	Biological	0.49	0.68	Solyc05g0	Solyc05g0	0.03	-5.01
GO:00062	CTP biosy	Biological	0.49	0.68	Solyc02g0	Solyc02g0	3.08	1.62
GO:00161	beta-amyla	Molecular	0.49	0.68	Solyc09g0	Solyc09g0	0.34	-1.54
GO:00168	racemase a	Molecular	0.49	0.68	Solyc12g0	Solyc12g0	0.25	-1.99
GO:00007	chromoson	Cellular C	0.49	0.68	Solyc01g0	Solyc01g0	2.56	1.36
GO:00057	integral co	Cellular C	0.49	0.68	Solyc05g0	Solyc05g0	0.48	-1.05
GO:00072	Rho protei	Biological	0.49	0.68	Solyc02g0	Solyc02g0	6.74	2.75
GO:00069	response t	Biological	0.49	0.68	Solyc06g0	Solyc06g0	2.09	1.07
GO:00105	pollen exir	Biological	0.49	0.68	Solyc03g0	Solyc03g0	0.18	-2.45
GO:00194	removal of	Biological	0.49	0.68	Solyc01g0	Solyc01g0	0.42	-1.26
GO:00040	aldehyde d	Molecular	0.49	0.68	Solyc12g0	Solyc12g0	4.01	2.00
GO:00039	acetyl-CoA	Molecular	0.49	0.68	Solyc04g0	Solyc04g0	0.00	-13.91
GO:00039	DNA topo	Molecular	0.49	0.68	Solyc01g0	Solyc01g0	0.39	-1.35
GO:00158	heme trans	Biological	0.49	0.68	Solyc11g0	Solyc11g0	0.00	-13.30
GO:00726	protein loc	Biological	0.49	0.68	Solyc12g0	Solyc12g0	3.10	1.63
GO:00082	spermidine	Biological	0.49	0.68	Solyc10g0	Solyc10g0	0.35	-1.51
GO:00480	ubiquinone	Molecular	0.49	0.68	Solyc10g0	Solyc10g0	0.00	-13.73
GO:00710	post-mRN	Cellular C	0.49	0.68	Solyc06g0	Solyc06g0	0.46	-1.11
GO:00163	1-phospha	Molecular	0.49	0.68	Solyc10g0	Solyc10g0	2.19	1.13
GO:00159	chlorophyl	Biological	0.49	0.68	Solyc09g0	Solyc09g0	2647.84	11.37
GO:00709	protein loc	Biological	0.49	0.68	Solyc04g0	Solyc04g0	5759.74	12.49
GO:00986	anion trans	Biological	0.49	0.68	Solyc03g1	Solyc03g1	2773.71	11.44
GO:00468	carbohydra	Biological	0.49	0.68	Solyc12g0	Solyc12g0	3.17	1.66
GO:00161	triterpenoi	Biological	0.49	0.68	Solyc12g0	Solyc12g0	2.17	1.12
GO:00040	catalase ac	Molecular	0.49	0.68	Solyc01g0	Solyc01g0	0.48	-1.07
GO:00085	P-type pro	Molecular	0.49	0.68	Solyc12g0	Solyc12g0	4.99	2.32
GO:00100	phloem or	Biological	0.49	0.68	Solyc08g0	Solyc08g0	4.19	2.07
GO:00068	superoxide	Biological	0.49	0.68	Solyc06g0	Solyc06g0	17.92	4.16
GO:00324	histone H3	Molecular	0.49	0.68	Solyc08g0	Solyc08g0	0.42	-1.24
GO:00331	histone H3	Biological	0.49	0.68	Solyc08g0	Solyc08g0	0.42	-1.24
GO:00160	RNA meta	Biological	0.49	0.68	Solyc05g0	Solyc05g0	20.96	4.39
GO:00109	magnesiun	Biological	0.49	0.68	Solyc06g0	Solyc06g0	587.73	9.20
GO:00161	brassinoste	Biological	0.50	0.69	Solyc05g0	Solyc05g0	2.81	1.49
GO:00161	brassinoste	Biological	0.50	0.69	Solyc02g0	Solyc02g0	0.48	-1.05
GO:00064	mRNA cat	Biological	0.50	0.69	Solyc06g0	Solyc06g0	659.59	9.37
GO:00064	mRNA cat	Biological	0.50	0.69	Solyc07g0	Solyc07g0	0.40	-1.31
GO:00063	DNA reco	Biological	0.50	0.70	Solyc01g1	Solyc01g1	2.32	1.22
GO:00063	DNA reco	Biological	0.50	0.70	Solyc03g0	Solyc03g0	0.00	-11.07
GO:00063	DNA reco	Biological	0.50	0.70	Solyc09g0	Solyc09g0	2.32	1.21
GO:00063	DNA reco	Biological	0.50	0.70	Solyc01g0	Solyc01g0	312.22	8.29
GO:00063	DNA reco	Biological	0.50	0.70	Solyc02g0	Solyc02g0	0.00	-10.40
GO:00458	positive re	Biological	0.50	0.70	Solyc10g0	Solyc10g0	5392.75	12.40
GO:00458	positive re	Biological	0.50	0.70	Solyc03g0	Solyc03g0	20.56	4.36
GO:00458	positive re	Biological	0.50	0.70	Solyc06g0	Solyc06g0	0.00	-12.03
GO:00458	positive re	Biological	0.50	0.70	Solyc01g0	Solyc01g0	4.12	2.04
GO:00458	positive re	Biological	0.50	0.70	Solyc02g0	Solyc02g0	0.39	-1.37
GO:00458	positive re	Biological	0.50	0.70	Solyc02g0	Solyc02g0	0.38	-1.40
GO:00458	positive re	Biological	0.50	0.70	Solyc10g0	Solyc10g0	683.10	9.42
GO:00458	positive re	Biological	0.50	0.70	Solyc02g0	Solyc02g0	1446.12	10.50

GO:00009	DNA-bind	Molecular	0.50	0.70	Solyc11g0	Solyc11g0	0.36	-1.47
GO:00009	DNA-bind	Molecular	0.50	0.70	Solyc11g0	Solyc11g0	2.29	1.19
GO:00009	DNA-bind	Molecular	0.50	0.70	Solyc01g0	Solyc01g0	0.44	-1.18
GO:00009	DNA-bind	Molecular	0.50	0.70	Solyc06g0	Solyc06g0	0.35	-1.53
GO:00009	DNA-bind	Molecular	0.50	0.70	Solyc06g0	Solyc06g0	2.01	1.01
GO:00009	DNA-bind	Molecular	0.50	0.70	Solyc04g0	Solyc04g0	0.07	-3.79
GO:00009	DNA-bind	Molecular	0.50	0.70	Solyc02g0	Solyc02g0	2.66	1.41
GO:00009	DNA-bind	Molecular	0.50	0.70	Solyc02g0	Solyc02g0	8.19	3.03
GO:00009	DNA-bind	Molecular	0.50	0.70	Solyc04g0	Solyc04g0	1227.78	10.26
GO:00009	DNA-bind	Molecular	0.50	0.70	Solyc01g0	Solyc01g0	0.48	-1.06
GO:00052	transporter	Molecular	0.51	0.70	Solyc07g0	Solyc07g0	2.77	1.47
GO:00052	transporter	Molecular	0.51	0.70	Solyc02g0	Solyc02g0	2.41	1.27
GO:00052	transporter	Molecular	0.51	0.70	Solyc08g0	Solyc08g0	2.67	1.41
GO:00052	transporter	Molecular	0.51	0.70	Solyc02g0	Solyc02g0	2.29	1.20
GO:00052	transporter	Molecular	0.51	0.70	Solyc12g0	Solyc12g0	4.99	2.32
GO:00041	cysteine-ty	Molecular	0.52	0.70	Solyc03g1	Solyc03g1	2.16	1.11
GO:00041	cysteine-ty	Molecular	0.52	0.70	Solyc04g0	Solyc04g0	2.20	1.14
GO:00041	cysteine-ty	Molecular	0.52	0.70	Solyc01g1	Solyc01g1	2.18	1.13
GO:00041	cysteine-ty	Molecular	0.52	0.70	Solyc12g0	Solyc12g0	0.39	-1.35
GO:00056	nucleoplas	Cellular C	0.52	0.70	Solyc01g0	Solyc01g0	2.56	1.36
GO:00056	nucleoplas	Cellular C	0.52	0.70	Solyc01g1	Solyc01g1	2.32	1.22
GO:00425	response to	Biological	0.52	0.70	Solyc10g0	Solyc10g0	0.09	-3.53
GO:00425	response to	Biological	0.52	0.70	Solyc02g0	Solyc02g0	2.46	1.30
GO:00048	protein pho	Molecular	0.52	0.70	Solyc09g0	Solyc09g0	0.27	-1.90
GO:00048	protein pho	Molecular	0.52	0.70	Solyc09g0	Solyc09g0	8.11	3.02
GO:00083	RNA splic	Biological	0.52	0.70	Solyc09g0	Solyc09g0	0.05	-4.37
GO:00083	RNA splic	Biological	0.52	0.70	Solyc05g0	Solyc05g0	0.03	-5.29
GO:00041	cytochrom	Molecular	0.52	0.70	Solyc11g0	Solyc11g0	0.09	-3.52
GO:00041	cytochrom	Molecular	0.52	0.70	Solyc11g0	Solyc11g0	0.00	-10.82
GO:00801	mitochond	Biological	0.52	0.70	Solyc05g0	Solyc05g0	19.89	4.31
GO:00162	antioxidan	Molecular	0.52	0.70	Solyc07g0	Solyc07g0	9.18	3.20
GO:00309	thiamine p	Molecular	0.52	0.70	Solyc03g0	Solyc03g0	4.05	2.02
GO:00043	farnesyltra	Molecular	0.52	0.70	Solyc04g0	Solyc04g0	2.62	1.39
GO:00330	tetrapyrrol	Biological	0.52	0.70	Solyc04g0	Solyc04g0	3.78	1.92
GO:00047	pyruvate k	Molecular	0.52	0.70	Solyc09g0	Solyc09g0	2.88	1.53
GO:00309	potassium	Molecular	0.52	0.70	Solyc09g0	Solyc09g0	2.88	1.53
GO:00099	histidine p	Molecular	0.52	0.70	Solyc01g0	Solyc01g0	2.26	1.17
GO:00060	cellular alc	Biological	0.52	0.70	Solyc01g0	Solyc01g0	0.22	-2.18
GO:00480	UDP-gluc	Molecular	0.52	0.70	Solyc09g0	Solyc09g0	4.17	2.06
GO:00071	G protein-	Biological	0.52	0.70	Solyc01g1	Solyc01g1	0.44	-1.20
GO:00198	protein kin	Molecular	0.52	0.70	Solyc06g0	Solyc06g0	4.81	2.27
GO:00517	misfolded	Molecular	0.52	0.70	Solyc06g0	Solyc06g0	2.09	1.07
GO:00800	fatty-acyl-	Molecular	0.52	0.70	Solyc09g0	Solyc09g0	0.14	-2.85
GO:00101	regulation	Biological	0.52	0.70	Solyc10g0	Solyc10g0	2.14	1.10
GO:00000	acyl carrie	Molecular	0.52	0.70	Solyc03g1	Solyc03g1	4.55	2.18
GO:00319	vesicle	Cellular C	0.52	0.70	Solyc04g0	Solyc04g0	2.07	1.05
GO:00161	sucrose sy	Molecular	0.52	0.70	Solyc09g0	Solyc09g0	2.71	1.44
GO:00704	rRNA base	Biological	0.52	0.70	Solyc07g0	Solyc07g0	0.40	-1.31
GO:00096	cold acclin	Biological	0.52	0.70	Solyc01g1	Solyc01g1	14.86	3.89
GO:00171	Golgi trans	Cellular C	0.52	0.70	Solyc04g0	Solyc04g0	0.26	-1.94
GO:00068	sodium ior	Biological	0.52	0.70	Solyc10g0	Solyc10g0	17.76	4.15

GO:00323	mismatch r	Cellular Co	0.52	0.70	Solyc01g0	Solyc01g0	0.49	-1.04
GO:00152	ligand-gate	Molecular	0.52	0.70	Solyc08g0	Solyc08g0	0.42	-1.27
GO:00083	asymmetric	Biological	0.52	0.70	Solyc05g0	Solyc05g0	5.37	2.42
GO:00041	aminopept	Molecular	0.52	0.70	Solyc12g0	Solyc12g0	1818.26	10.83
GO:00009	retrotransp	Cellular Co	0.52	0.70	Solyc11g0	Solyc11g0	0.13	-2.96
GO:00052	voltage-ga	Molecular	0.52	0.70	Solyc02g0	Solyc02g0	19.78	4.31
GO:00068	chloride tr	Biological	0.52	0.70	Solyc02g0	Solyc02g0	19.78	4.31
GO:00452	proton-tran	Cellular Co	0.52	0.70	Solyc10g0	Solyc10g0	0.03	-5.05
GO:00001	1,3-beta-D	Cellular Co	0.52	0.70	Solyc11g0	Solyc11g0	0.23	-2.12
GO:00038	1,3-beta-D	Molecular	0.52	0.70	Solyc11g0	Solyc11g0	0.23	-2.12
GO:00060	(1->3)-beta	Biological	0.52	0.70	Solyc11g0	Solyc11g0	0.23	-2.12
GO:00715	CAAX-bo	Biological	0.52	0.70	Solyc05g0	Solyc05g0	1370.75	10.42
GO:00085	proteasom	Cellular Co	0.52	0.70	Solyc01g1	Solyc01g1	0.00	-9.07
GO:00043	fructose-bi	Molecular	0.52	0.70	Solyc02g0	Solyc02g0	3.52	1.82
GO:00485	recognition	Biological	0.53	0.71	Solyc05g0	Solyc05g0	0.43	-1.22
GO:00485	recognition	Biological	0.53	0.71	Solyc10g0	Solyc10g0	0.08	-3.68
GO:00485	recognition	Biological	0.53	0.71	Solyc02g0	Solyc02g0	394.05	8.62
GO:00485	recognition	Biological	0.53	0.71	Solyc02g0	Solyc02g0	517.85	9.02
GO:00083	cation tran	Molecular	0.54	0.72	Solyc01g0	Solyc01g0	0.33	-1.61
GO:00083	cation tran	Molecular	0.54	0.72	Solyc06g0	Solyc06g0	2059.41	11.01
GO:00070	mitotic sist	Biological	0.54	0.72	Solyc01g0	Solyc01g0	2.56	1.36
GO:00070	mitotic sist	Biological	0.54	0.72	Solyc06g0	Solyc06g0	0.31	-1.70
GO:00065	GPI ancho	Biological	0.54	0.72	Solyc01g0	Solyc01g0	0.35	-1.51
GO:00065	GPI ancho	Biological	0.54	0.72	Solyc03g0	Solyc03g0	6.17	2.63
GO:00080	tRNA proc	Biological	0.54	0.72	Solyc09g0	Solyc09g0	0.05	-4.37
GO:00080	tRNA proc	Biological	0.54	0.72	Solyc05g0	Solyc05g0	0.03	-5.29
GO:00301	pyridoxal p	Molecular	0.55	0.73	Solyc01g0	Solyc01g0	2.46	1.30
GO:00301	pyridoxal p	Molecular	0.55	0.73	Solyc04g0	Solyc04g0	3.78	1.92
GO:00301	pyridoxal p	Molecular	0.55	0.73	Solyc11g0	Solyc11g0	0.50	-1.01
GO:00301	pyridoxal p	Molecular	0.55	0.73	Solyc07g0	Solyc07g0	6.21	2.63
GO:00301	pyridoxal p	Molecular	0.55	0.73	Solyc05g0	Solyc05g0	2.50	1.32
GO:00301	pyridoxal p	Molecular	0.55	0.73	Solyc08g0	Solyc08g0	3785.25	11.89
GO:00301	pyridoxal p	Molecular	0.55	0.73	Solyc04g0	Solyc04g0	1711.14	10.74
GO:00301	pyridoxal p	Molecular	0.55	0.73	Solyc08g0	Solyc08g0	0.13	-2.97
GO:00301	pyridoxal p	Molecular	0.55	0.73	Solyc06g0	Solyc06g0	0.00	-9.45
GO:00481	Golgi vesic	Biological	0.56	0.73	Solyc03g1	Solyc03g1	0.33	-1.60
GO:00063	transcriptio	Biological	0.56	0.73	Solyc09g0	Solyc09g0	0.18	-2.45
GO:00086	phospholip	Biological	0.56	0.73	Solyc06g0	Solyc06g0	0.41	-1.30
GO:00346	cellular res	Biological	0.56	0.73	Solyc06g0	Solyc06g0	2.09	1.07
GO:00071	reciprocal	Biological	0.56	0.73	Solyc09g0	Solyc09g0	0.35	-1.51
GO:00436	cortical mi	Biological	0.56	0.73	Solyc02g0	Solyc02g0	2.29	1.19
GO:00194	aromatic co	Biological	0.56	0.73	Solyc03g0	Solyc03g0	0.39	-1.35
GO:00003	plant-type	Cellular Co	0.56	0.73	Solyc06g0	Solyc06g0	15.51	3.95
GO:00457	negative re	Biological	0.56	0.73	Solyc06g0	Solyc06g0	2.87	1.52
GO:00057	mitochond	Cellular Co	0.56	0.73	Solyc03g0	Solyc03g0	2.19	1.13
GO:00102	response to	Biological	0.56	0.73	Solyc02g0	Solyc02g0	1506.28	10.56
GO:00167	hydrolase a	Molecular	0.56	0.73	Solyc11g0	Solyc11g0	0.46	-1.12
GO:00514	regulation	Biological	0.56	0.73	Solyc12g0	Solyc12g0	4.99	2.32
GO:00057	mitochond	Cellular Co	0.56	0.73	Solyc01g0	Solyc01g0	0.08	-3.59
GO:00070	cytoskeleto	Biological	0.56	0.73	Solyc04g0	Solyc04g0	553.61	9.11
GO:00068	phosphate	Biological	0.56	0.73	Solyc12g0	Solyc12g0	4.94	2.30

GO:00068	phosphate	Biological	0.56	0.73	Solyc11g0	Solyc11g0	0.49	-1.04
GO:00068	phosphate	Biological	0.56	0.73	Solyc06g0	Solyc06g0	0.32	-1.62
GO:00068	phosphate	Biological	0.56	0.73	Solyc09g0	Solyc09g0	0.38	-1.39
GO:00510	actin filam	Molecular	0.56	0.73	Solyc03g1	Solyc03g1	0.46	-1.13
GO:00510	actin filam	Molecular	0.56	0.73	Solyc08g0	Solyc08g0	21.56	4.43
GO:00510	actin filam	Molecular	0.56	0.73	Solyc04g0	Solyc04g0	2.37	1.25
GO:00510	actin filam	Molecular	0.56	0.73	Solyc07g0	Solyc07g0	0.45	-1.16
GO:00466	anchored c	Cellular C	0.56	0.73	Solyc01g1	Solyc01g1	14.06	3.81
GO:00466	anchored c	Cellular C	0.56	0.73	Solyc10g0	Solyc10g0	3.83	1.94
GO:00466	anchored c	Cellular C	0.56	0.73	Solyc07g0	Solyc07g0	0.42	-1.26
GO:00466	anchored c	Cellular C	0.56	0.73	Solyc09g0	Solyc09g0	0.49	-1.04
GO:00466	anchored c	Cellular C	0.56	0.73	Solyc03g0	Solyc03g0	0.34	-1.57
GO:00466	anchored c	Cellular C	0.56	0.73	Solyc11g0	Solyc11g0	0.28	-1.82
GO:00152	purine nuc	Molecular	0.56	0.74	Solyc02g0	Solyc02g0	2.41	1.27
GO:00152	purine nuc	Molecular	0.56	0.74	Solyc02g0	Solyc02g0	2.29	1.20
GO:00167	phosphotra	Molecular	0.56	0.74	Solyc12g0	Solyc12g0	3.17	1.66
GO:00167	phosphotra	Molecular	0.56	0.74	Solyc11g0	Solyc11g0	2.98	1.58
GO:00000	ribosomal	Biological	0.57	0.74	Solyc09g0	Solyc09g0	4.17	2.06
GO:00000	ribosomal	Biological	0.57	0.74	Solyc11g0	Solyc11g0	0.00	-13.41
GO:00000	ribosomal	Biological	0.57	0.74	Solyc12g0	Solyc12g0	0.04	-4.72
GO:00012	DNA-bind	Molecular	0.57	0.75	Solyc11g0	Solyc11g0	0.50	-1.01
GO:00012	DNA-bind	Molecular	0.57	0.75	Solyc06g0	Solyc06g0	0.00	-12.03
GO:00012	DNA-bind	Molecular	0.57	0.75	Solyc12g0	Solyc12g0	5.33	2.42
GO:00012	DNA-bind	Molecular	0.57	0.75	Solyc04g0	Solyc04g0	1227.78	10.26
GO:00063	mRNA prc	Biological	0.57	0.75	Solyc09g0	Solyc09g0	0.05	-4.37
GO:00063	mRNA prc	Biological	0.57	0.75	Solyc12g0	Solyc12g0	0.00	-13.90
GO:00063	mRNA prc	Biological	0.57	0.75	Solyc05g0	Solyc05g0	0.03	-5.29
GO:00063	mRNA prc	Biological	0.57	0.75	Solyc04g0	Solyc04g0	2.47	1.30
GO:00037	translation	Molecular	0.58	0.75	Solyc11g0	Solyc11g0	0.10	-3.26
GO:00037	translation	Molecular	0.58	0.75	Solyc10g0	Solyc10g0	2.10	1.07
GO:19015	organonitr	Biological	0.58	0.75	Solyc04g0	Solyc04g0	3.78	1.92
GO:19015	organonitr	Biological	0.58	0.75	Solyc08g0	Solyc08g0	0.13	-2.97
GO:00380	signaling r	Molecular	0.58	0.75	Solyc09g0	Solyc09g0	0.27	-1.90
GO:00380	signaling r	Molecular	0.58	0.75	Solyc09g0	Solyc09g0	8.11	3.02
GO:00041	serine-type	Molecular	0.59	0.75	Solyc01g0	Solyc01g0	2.08	1.05
GO:00041	serine-type	Molecular	0.59	0.75	Solyc12g0	Solyc12g0	4.50	2.17
GO:00041	serine-type	Molecular	0.59	0.75	Solyc05g0	Solyc05g0	1063.88	10.06
GO:00046	polygalact	Molecular	0.59	0.75	Solyc10g0	PG2	9.48	3.25
GO:00046	polygalact	Molecular	0.59	0.75	Solyc04g0	Solyc04g0	8.84	3.14
GO:00046	polygalact	Molecular	0.59	0.75	Solyc02g0	Solyc02g0	7.09	2.83
GO:00046	polygalact	Molecular	0.59	0.75	Solyc03g0	Solyc03g0	11.56	3.53
GO:00329	protein-co	Cellular C	0.59	0.75	Solyc09g0	Solyc09g0	0.27	-1.91
GO:00442	cellular lip	Biological	0.59	0.75	Solyc08g0	Solyc08g0	4.51	2.17
GO:00040	carbonate	Molecular	0.59	0.75	Solyc02g0	Solyc02g0	11.09	3.47
GO:00045	beta-galact	Molecular	0.59	0.75	Solyc03g1	Solyc03g1	2.20	1.14
GO:00434	protein his	Molecular	0.59	0.75	Solyc01g0	Solyc01g0	2.26	1.17
GO:00096	cytokinin r	Biological	0.59	0.75	Solyc10g0	Solyc10g0	3.13	1.64
GO:00427	D-xylose n	Biological	0.59	0.75	Solyc09g0	Solyc09g0	4.17	2.06
GO:00464	phosphatid	Biological	0.59	0.75	Solyc01g1	Solyc01g1	0.24	-2.06
GO:00484	plant ovule	Biological	0.59	0.75	Solyc11g0	Solyc11g0	2.29	1.19
GO:00062	DNA topo	Biological	0.59	0.75	Solyc01g0	Solyc01g0	0.39	-1.35

GO:00001	MAPK cas	Biological	0.59	0.75	Solyc08g0	Solyc08g0	0.41	-1.30
GO:00009	cytokinesis	Biological	0.59	0.75	Solyc01g0	Solyc01g0	0.00	-10.07
GO:00005	proteasom	Cellular C	0.59	0.75	Solyc01g1	Solyc01g1	0.00	-9.07
GO:00458	negative re	Biological	0.59	0.75	Solyc06g0	Solyc06g0	2.87	1.52
GO:00168	strictosidir	Molecular	0.59	0.75	Solyc05g0	Solyc05g0	2.44	1.28
GO:00046	prenyltrans	Molecular	0.59	0.75	Solyc06g0	Solyc06g0	12.77	3.67
GO:00162	lipase activ	Molecular	0.59	0.75	Solyc04g0	Solyc04g0	9.52	3.25
GO:00300	metal ion t	Biological	0.59	0.75	Solyc03g0	Solyc03g0	0.42	-1.27
GO:00300	metal ion t	Biological	0.59	0.75	Solyc10g0	Solyc10g0	2.91	1.54
GO:00300	metal ion t	Biological	0.59	0.75	Solyc06g0	Solyc06g0	4.45	2.15
GO:00300	metal ion t	Biological	0.59	0.75	Solyc04g0	Solyc04g0	0.39	-1.37
GO:00300	metal ion t	Biological	0.59	0.75	Solyc02g0	Solyc02g0	27.11	4.76
GO:00516	proteolysis	Biological	0.60	0.77	Solyc03g1	Solyc03g1	2.16	1.11
GO:00516	proteolysis	Biological	0.60	0.77	Solyc04g0	Solyc04g0	2.20	1.14
GO:00516	proteolysis	Biological	0.60	0.77	Solyc12g0	Solyc12g0	4.50	2.17
GO:00516	proteolysis	Biological	0.60	0.77	Solyc03g0	Solyc03g0	30231.28	14.88
GO:00098	ethylene-a	Biological	0.60	0.77	Solyc09g0	Solyc09g0	0.25	-1.98
GO:00098	ethylene-a	Biological	0.60	0.77	Solyc01g0	Solyc01g0	0.42	-1.24
GO:00098	ethylene-a	Biological	0.60	0.77	Solyc03g0	Solyc03g0	0.46	-1.13
GO:00098	ethylene-a	Biological	0.60	0.77	Solyc04g0	Solyc04g0	732.58	9.52
GO:00466	response to	Biological	0.60	0.77	Solyc04g0	Solyc04g0	2.70	1.43
GO:00466	response to	Biological	0.60	0.77	Solyc01g0	Solyc01g0	550.66	9.11
GO:00062	base-excisi	Biological	0.60	0.77	Solyc09g0	Solyc09g0	2.14	1.10
GO:00062	base-excisi	Biological	0.60	0.77	Solyc09g0	Solyc09g0	2.29	1.20
GO:00162	iron-sulfur	Biological	0.60	0.77	Solyc05g0	Solyc05g0	2.96	1.57
GO:00162	iron-sulfur	Biological	0.60	0.77	Solyc12g0	Solyc12g0	2.58	1.37
GO:00069	cellular res	Biological	0.60	0.77	Solyc04g0	Solyc04g0	2.35	1.23
GO:00069	cellular res	Biological	0.60	0.77	Solyc03g0	Solyc03g0	0.02	-5.67
GO:00056	nucleus	Cellular C	0.62	0.77	Solyc09g0	Solyc09g0	6.00	2.58
GO:00056	nucleus	Cellular C	0.62	0.77	Solyc09g0	Solyc09g0	0.05	-4.31
GO:00056	nucleus	Cellular C	0.62	0.77	Solyc06g0	Solyc06g0	7.71	2.95
GO:00056	nucleus	Cellular C	0.62	0.77	Solyc01g1	Solyc01g1	5.46	2.45
GO:00056	nucleus	Cellular C	0.62	0.77	Solyc03g0	Solyc03g0	0.24	-2.06
GO:00056	nucleus	Cellular C	0.62	0.77	Solyc11g0	Solyc11g0	0.36	-1.47
GO:00056	nucleus	Cellular C	0.62	0.77	Solyc01g0	Solyc01g0	2.68	1.42
GO:00056	nucleus	Cellular C	0.62	0.77	Solyc04g0	Solyc04g0	2.73	1.45
GO:00056	nucleus	Cellular C	0.62	0.77	Solyc12g0	Solyc12g0	9.07	3.18
GO:00056	nucleus	Cellular C	0.62	0.77	Solyc01g0	Solyc01g0	3.72	1.90
GO:00056	nucleus	Cellular C	0.62	0.77	Solyc01g0	Solyc01g0	2.26	1.17
GO:00056	nucleus	Cellular C	0.62	0.77	Solyc02g0	Solyc02g0	2.17	1.12
GO:00056	nucleus	Cellular C	0.62	0.77	Solyc05g0	Solyc05g0	0.46	-1.12
GO:00056	nucleus	Cellular C	0.62	0.77	Solyc07g0	Solyc07g0	2.17	1.12
GO:00056	nucleus	Cellular C	0.62	0.77	Solyc11g0	Solyc11g0	0.22	-2.21
GO:00056	nucleus	Cellular C	0.62	0.77	Solyc09g0	Solyc09g0	0.40	-1.34
GO:00056	nucleus	Cellular C	0.62	0.77	Solyc09g0	Solyc09g0	0.32	-1.63
GO:00056	nucleus	Cellular C	0.62	0.77	Solyc06g0	Solyc06g0	0.48	-1.05
GO:00056	nucleus	Cellular C	0.62	0.77	Solyc01g0	Solyc01g0	0.34	-1.54
GO:00056	nucleus	Cellular C	0.62	0.77	Solyc08g0	Solyc08g0	0.47	-1.08
GO:00056	nucleus	Cellular C	0.62	0.77	Solyc05g0	Solyc05g0	2.40	1.27
GO:00056	nucleus	Cellular C	0.62	0.77	Solyc09g0	Solyc09g0	0.44	-1.18
GO:00056	nucleus	Cellular C	0.62	0.77	Solyc08g0	Solyc08g0	2.38	1.25

GO:00056	nucleus	Cellular Co	0.62	0.77	Solyc09g0	Solyc09g0	0.34	-1.58
GO:00056	nucleus	Cellular Co	0.62	0.77	Solyc11g0	Solyc11g0	2.29	1.19
GO:00056	nucleus	Cellular Co	0.62	0.77	Solyc06g0	Solyc06g0	0.11	-3.19
GO:00056	nucleus	Cellular Co	0.62	0.77	Solyc09g0	Solyc09g0	0.27	-1.90
GO:00056	nucleus	Cellular Co	0.62	0.77	Solyc06g0	Solyc06g0	0.46	-1.11
GO:00056	nucleus	Cellular Co	0.62	0.77	Solyc01g1	Solyc01g1	2.32	1.22
GO:00056	nucleus	Cellular Co	0.62	0.77	Solyc02g0	Solyc02g0	2.01	1.01
GO:00056	nucleus	Cellular Co	0.62	0.77	Solyc01g0	Solyc01g0	0.10	-3.35
GO:00056	nucleus	Cellular Co	0.62	0.77	Solyc01g0	Solyc01g0	0.44	-1.18
GO:00056	nucleus	Cellular Co	0.62	0.77	Solyc01g1	Solyc01g1	0.44	-1.20
GO:00056	nucleus	Cellular Co	0.62	0.77	Solyc06g0	Solyc06g0	4.81	2.27
GO:00056	nucleus	Cellular Co	0.62	0.77	Solyc02g0	Solyc02g0	6.74	2.75
GO:00056	nucleus	Cellular Co	0.62	0.77	Solyc07g0	Solyc07g0	0.46	-1.11
GO:00056	nucleus	Cellular Co	0.62	0.77	Solyc02g0	Solyc02g0	2.62	1.39
GO:00056	nucleus	Cellular Co	0.62	0.77	Solyc04g0	Solyc04g0	2.47	1.30
GO:00056	nucleus	Cellular Co	0.62	0.77	Solyc09g0	Solyc09g0	0.35	-1.51
GO:00056	nucleus	Cellular Co	0.62	0.77	Solyc08g0	Solyc08g0	0.20	-2.35
GO:00056	nucleus	Cellular Co	0.62	0.77	Solyc02g0	Solyc02g0	0.49	-1.02
GO:00056	nucleus	Cellular Co	0.62	0.77	Solyc06g0	Solyc06g0	0.46	-1.11
GO:00056	nucleus	Cellular Co	0.62	0.77	Solyc02g0	Solyc02g0	0.42	-1.24
GO:00056	nucleus	Cellular Co	0.62	0.77	Solyc11g0	Solyc11g0	0.50	-1.01
GO:00056	nucleus	Cellular Co	0.62	0.77	Solyc01g1	Solyc01g1	2.18	1.13
GO:00056	nucleus	Cellular Co	0.62	0.77	Solyc11g0	Solyc11g0	2.31	1.21
GO:00056	nucleus	Cellular Co	0.62	0.77	Solyc05g0	Solyc05g0	0.22	-2.15
GO:00056	nucleus	Cellular Co	0.62	0.77	Solyc08g0	Solyc08g0	3.77	1.91
GO:00056	nucleus	Cellular Co	0.62	0.77	Solyc09g0	Solyc09g0	0.44	-1.19
GO:00056	nucleus	Cellular Co	0.62	0.77	Solyc06g0	Solyc06g0	2.48	1.31
GO:00056	nucleus	Cellular Co	0.62	0.77	Solyc05g0	Solyc05g0	4.20	2.07
GO:00056	nucleus	Cellular Co	0.62	0.77	Solyc11g0	Solyc11g0	18.46	4.21
GO:00056	nucleus	Cellular Co	0.62	0.77	Solyc04g0	Solyc04g0	0.21	-2.27
GO:00056	nucleus	Cellular Co	0.62	0.77	Solyc02g0	Solyc02g0	0.32	-1.64
GO:00056	nucleus	Cellular Co	0.62	0.77	Solyc01g0	Solyc01g0	3.17	1.66
GO:00056	nucleus	Cellular Co	0.62	0.77	Solyc01g0	Solyc01g0	2.30	1.20
GO:00056	nucleus	Cellular Co	0.62	0.77	Solyc06g0	Solyc06g0	3.13	1.64
GO:00056	nucleus	Cellular Co	0.62	0.77	Solyc01g0	Solyc01g0	0.39	-1.35
GO:00056	nucleus	Cellular Co	0.62	0.77	Solyc04g0	Solyc04g0	6800.94	12.73
GO:00056	nucleus	Cellular Co	0.62	0.77	Solyc06g0	Solyc06g0	2.54	1.35
GO:00056	nucleus	Cellular Co	0.62	0.77	Solyc06g0	Solyc06g0	0.35	-1.53
GO:00056	nucleus	Cellular Co	0.62	0.77	Solyc10g0	Solyc10g0	5392.75	12.40
GO:00056	nucleus	Cellular Co	0.62	0.77	Solyc11g0	Solyc11g0	0.50	-1.00
GO:00056	nucleus	Cellular Co	0.62	0.77	Solyc09g0	Solyc09g0	2.14	1.10
GO:00056	nucleus	Cellular Co	0.62	0.77	Solyc04g0	Solyc04g0	0.41	-1.28
GO:00056	nucleus	Cellular Co	0.62	0.77	Solyc01g0	Solyc01g0	34.25	5.10
GO:00056	nucleus	Cellular Co	0.62	0.77	Solyc04g0	Solyc04g0	2.35	1.23
GO:00056	nucleus	Cellular Co	0.62	0.77	Solyc02g0	Solyc02g0	0.42	-1.26
GO:00056	nucleus	Cellular Co	0.62	0.77	Solyc12g0	Solyc12g0	2.41	1.27
GO:00056	nucleus	Cellular Co	0.62	0.77	Solyc09g0	Solyc09g0	0.25	-1.98
GO:00056	nucleus	Cellular Co	0.62	0.77	Solyc12g1	Solyc12g1	2.30	1.20
GO:00056	nucleus	Cellular Co	0.62	0.77	Solyc06g0	Solyc06g0	0.46	-1.11
GO:00056	nucleus	Cellular Co	0.62	0.77	Solyc10g0	Solyc10g0	2.26	1.18
GO:00056	nucleus	Cellular Co	0.62	0.77	Solyc06g0	Solyc06g0	2.01	1.01

GO:00056	nucleus	Cellular C	0.62	0.77	Solyc04g0	Solyc04g0	0.07	-3.79
GO:00056	nucleus	Cellular C	0.62	0.77	Solyc06g0	Solyc06g0	0.39	-1.37
GO:00056	nucleus	Cellular C	0.62	0.77	Solyc03g0	Solyc03g0	4.18	2.06
GO:00056	nucleus	Cellular C	0.62	0.77	Solyc02g0	Solyc02g0	0.00	-11.29
GO:00056	nucleus	Cellular C	0.62	0.77	Solyc12g0	Solyc12g0	2.29	1.20
GO:00056	nucleus	Cellular C	0.62	0.77	Solyc03g0	Solyc03g0	2300.48	11.17
GO:00056	nucleus	Cellular C	0.62	0.77	Solyc12g0	Solyc12g0	0.33	-1.61
GO:00056	nucleus	Cellular C	0.62	0.77	Solyc11g0	Solyc11g0	2.24	1.16
GO:00056	nucleus	Cellular C	0.62	0.77	Solyc08g0	Solyc08g0	2486.58	11.28
GO:00056	nucleus	Cellular C	0.62	0.77	Solyc11g0	Solyc11g0	0.00	-10.83
GO:00056	nucleus	Cellular C	0.62	0.77	Solyc02g0	Solyc02g0	25.47	4.67
GO:00056	nucleus	Cellular C	0.62	0.77	Solyc05g0	Solyc05g0	2.76	1.47
GO:00056	nucleus	Cellular C	0.62	0.77	Solyc10g0	Solyc10g0	0.40	-1.31
GO:00056	nucleus	Cellular C	0.62	0.77	Solyc02g0	Solyc02g0	2.66	1.41
GO:00056	nucleus	Cellular C	0.62	0.77	Solyc03g1	Solyc03g1	0.06	-4.16
GO:00056	nucleus	Cellular C	0.62	0.77	Solyc06g0	Solyc06g0	0.00	-10.94
GO:00056	nucleus	Cellular C	0.62	0.77	Solyc01g0	Solyc01g0	1296.75	10.34
GO:00056	nucleus	Cellular C	0.62	0.77	Solyc07g0	Solyc07g0	2.02	1.02
GO:00056	nucleus	Cellular C	0.62	0.77	Solyc01g0	Solyc01g0	2.57	1.36
GO:00056	nucleus	Cellular C	0.62	0.77	Solyc03g0	Solyc03g0	20.56	4.36
GO:00056	nucleus	Cellular C	0.62	0.77	Solyc06g0	Solyc06g0	0.00	-12.03
GO:00056	nucleus	Cellular C	0.62	0.77	Solyc01g0	Solyc01g0	4.12	2.04
GO:00056	nucleus	Cellular C	0.62	0.77	Solyc02g0	Solyc02g0	0.39	-1.37
GO:00056	nucleus	Cellular C	0.62	0.77	Solyc04g0	Solyc04g0	2071.20	11.02
GO:00056	nucleus	Cellular C	0.62	0.77	Solyc03g0	Solyc03g0	0.00	-11.07
GO:00056	nucleus	Cellular C	0.62	0.77	Solyc02g0	Solyc02g0	8.19	3.03
GO:00056	nucleus	Cellular C	0.62	0.77	Solyc09g0	Solyc09g0	2.32	1.21
GO:00056	nucleus	Cellular C	0.62	0.77	Solyc04g0	Solyc04g0	0.48	-1.06
GO:00056	nucleus	Cellular C	0.62	0.77	Solyc12g0	Solyc12g0	5.33	2.42
GO:00056	nucleus	Cellular C	0.62	0.77	Solyc03g1	Solyc03g1	2361.02	11.21
GO:00056	nucleus	Cellular C	0.62	0.77	Solyc01g0	Solyc01g0	5.28	2.40
GO:00056	nucleus	Cellular C	0.62	0.77	Solyc12g0	Solyc12g0	3.71	1.89
GO:00056	nucleus	Cellular C	0.62	0.77	Solyc10g0	Solyc10g0	0.29	-1.77
GO:00056	nucleus	Cellular C	0.62	0.77	Solyc03g1	Solyc03g1	734.02	9.52
GO:00056	nucleus	Cellular C	0.62	0.77	Solyc12g0	Solyc12g0	0.39	-1.35
GO:00056	nucleus	Cellular C	0.62	0.77	Solyc07g0	Solyc07g0	42.77	5.42
GO:00056	nucleus	Cellular C	0.62	0.77	Solyc03g0	Solyc03g0	2638.78	11.37
GO:00056	nucleus	Cellular C	0.62	0.77	Solyc02g0	Solyc02g0	1446.12	10.50
GO:00056	nucleus	Cellular C	0.62	0.77	Solyc09g0	Solyc09g0	2.29	1.20
GO:00056	nucleus	Cellular C	0.62	0.77	Solyc01g0	Solyc01g0	6.82	2.77
GO:00056	nucleus	Cellular C	0.62	0.77	Solyc01g1	Solyc01g1	3.24	1.69
GO:00056	nucleus	Cellular C	0.62	0.77	Solyc10g0	Solyc10g0	2.60	1.38
GO:00056	nucleus	Cellular C	0.62	0.77	Solyc04g0	Solyc04g0	1227.78	10.26
GO:00056	nucleus	Cellular C	0.62	0.77	Solyc08g0	Solyc08g0	3.40	1.77
GO:00056	nucleus	Cellular C	0.62	0.77	Solyc02g0	Solyc02g0	0.06	-4.15
GO:00056	nucleus	Cellular C	0.62	0.77	Solyc06g0	Solyc06g0	15.65	3.97
GO:00056	nucleus	Cellular C	0.62	0.77	Solyc06g0	Solyc06g0	2.87	1.52
GO:00056	nucleus	Cellular C	0.62	0.77	Solyc01g0	Solyc01g0	626.38	9.29
GO:00056	nucleus	Cellular C	0.62	0.77	Solyc01g0	Solyc01g0	0.48	-1.06
GO:00056	nucleus	Cellular C	0.62	0.77	Solyc06g0	Solyc06g0	8.85	3.15
GO:00056	nucleus	Cellular C	0.62	0.77	Solyc06g0	Solyc06g0	0.45	-1.16

GO:00056	nucleus	Cellular Co	0.62	0.77	Solyc10g0	Solyc10g0	0.26	-1.94
GO:00056	nucleus	Cellular Co	0.62	0.77	Solyc01g0	Solyc01g0	0.42	-1.24
GO:00056	nucleus	Cellular Co	0.62	0.77	Solyc02g0	Solyc02g0	1506.28	10.56
GO:00056	nucleus	Cellular Co	0.62	0.77	Solyc08g0	Solyc08g0	2.07	1.05
GO:00056	nucleus	Cellular Co	0.62	0.77	Solyc02g0	Solyc02g0	4.91	2.30
GO:00056	nucleus	Cellular Co	0.62	0.77	Solyc03g1	Solyc03g1	356.83	8.48
GO:00056	nucleus	Cellular Co	0.62	0.77	Solyc11g0	Solyc11g0	0.47	-1.09
GO:00056	nucleus	Cellular Co	0.62	0.77	Solyc08g0	Solyc08g0	3.29	1.72
GO:00056	nucleus	Cellular Co	0.62	0.77	Solyc11g0	Solyc11g0	0.46	-1.12
GO:00056	nucleus	Cellular Co	0.62	0.77	Solyc01g0	Solyc01g0	0.24	-2.09
GO:00056	nucleus	Cellular Co	0.62	0.77	Solyc01g0	Solyc01g0	0.00	-9.77
GO:00056	nucleus	Cellular Co	0.62	0.77	Solyc06g0	Solyc06g0	0.45	-1.16
GO:00056	nucleus	Cellular Co	0.62	0.77	Solyc02g0	Solyc02g0	0.06	-4.03
GO:00056	nucleus	Cellular Co	0.62	0.77	Solyc06g0	PTI6	0.43	-1.22
GO:00056	nucleus	Cellular Co	0.62	0.77	Solyc08g0	Solyc08g0	0.41	-1.27
GO:00056	nucleus	Cellular Co	0.62	0.77	Solyc08g0	Solyc08g0	2.98	1.58
GO:00056	nucleus	Cellular Co	0.62	0.77	Solyc01g0	Solyc01g0	0.45	-1.14
GO:00056	nucleus	Cellular Co	0.62	0.77	Solyc01g0	Solyc01g0	312.22	8.29
GO:00056	nucleus	Cellular Co	0.62	0.77	Solyc03g0	Solyc03g0	0.00	-9.94
GO:00056	nucleus	Cellular Co	0.62	0.77	Solyc10g0	Solyc10g0	2.42	1.27
GO:00056	nucleus	Cellular Co	0.62	0.77	Solyc04g0	Solyc04g0	732.58	9.52
GO:00056	nucleus	Cellular Co	0.62	0.77	Solyc05g0	Solyc05g0	0.00	-10.39
GO:00056	nucleus	Cellular Co	0.62	0.77	Solyc06g0	Solyc06g0	0.08	-3.60
GO:00056	nucleus	Cellular Co	0.62	0.77	Solyc09g0	Solyc09g0	0.22	-2.22
GO:00056	nucleus	Cellular Co	0.62	0.77	Solyc02g0	Solyc02g0	0.00	-10.40
GO:00056	nucleus	Cellular Co	0.62	0.77	Solyc02g0	Solyc02g0	7.52	2.91
GO:00342	carbohydra	Biological	0.62	0.77	Solyc12g0	Solyc12g0	4.94	2.30
GO:00057	peroxisom	Cellular Co	0.62	0.77	Solyc02g0	Solyc02g0	0.50	-1.01
GO:00012	DNA-bind	Molecular	0.62	0.77	Solyc08g0	Solyc08g0	2.38	1.25
GO:00450	innate imm	Biological	0.62	0.77	Solyc02g0	Solyc02g0	0.38	-1.41
GO:00068	water trans	Biological	0.62	0.77	Solyc06g0	Solyc06g0	2.02	1.02
GO:00009	cell morph	Biological	0.62	0.77	Solyc02g0	Solyc02g0	6.74	2.75
GO:00098	pollen tube	Biological	0.62	0.77	Solyc01g0	Solyc01g0	0.49	-1.04
GO:00181	peptidyl-th	Biological	0.62	0.77	Solyc11g0	Solyc11g0	2.57	1.36
GO:00068	calcium io	Biological	0.62	0.77	Solyc06g0	Solyc06g0	2059.41	11.01
GO:00084	beta-gluco	Molecular	0.62	0.77	Solyc06g0	Solyc06g0	2.25	1.17
GO:00071	transmemb	Biological	0.62	0.77	Solyc10g0	Solyc10g0	4.22	2.08
GO:00195	protein me	Biological	0.62	0.77	Solyc12g0	Solyc12g0	1818.26	10.83
GO:00100	thylakoid r	Biological	0.62	0.77	Solyc06g0	PSBS	2.99	1.58
GO:00199	protein do	Molecular	0.62	0.77	Solyc06g0	PSBS	2.99	1.58
GO:00088	fructokina	Molecular	0.62	0.77	Solyc12g0	Solyc12g0	3.17	1.66
GO:00160	cellular res	Biological	0.62	0.77	Solyc01g0	Solyc01g0	0.21	-2.28
GO:00052	calcium ac	Molecular	0.62	0.77	Solyc06g0	Solyc06g0	13.67	3.77
GO:00053	inorganic p	Molecular	0.62	0.77	Solyc09g0	Solyc09g0	0.38	-1.39
GO:00096	salicylic ac	Biological	0.62	0.77	Solyc01g1	Solyc01g1	0.00	-10.11
GO:00800	methyl ind	Molecular	0.62	0.77	Solyc01g1	Solyc01g1	0.00	-10.11
GO:00800	methyl sali	Molecular	0.62	0.77	Solyc01g1	Solyc01g1	0.00	-10.11
GO:00800	methyl jas	Molecular	0.62	0.77	Solyc01g1	Solyc01g1	0.00	-10.11
GO:00064	translation	Biological	0.62	0.77	Solyc11g0	Solyc11g0	0.10	-3.26
GO:00064	translation	Biological	0.62	0.77	Solyc10g0	Solyc10g0	2.10	1.07
GO:00084	3'-5' exonu	Molecular	0.62	0.77	Solyc09g0	Solyc09g0	6.17	2.63

GO:00084	3'-5' exonu	Molecular	0.62	0.77	Solyc01g1	Solyc01g1	2.98	1.58
GO:00086	lipid biosy	Biological	0.62	0.77	Solyc12g1	Solyc12g1	0.00	-10.11
GO:00086	lipid biosy	Biological	0.62	0.77	Solyc02g0	Solyc02g0	8.45	3.08
GO:00312	anchored c	Cellular C	0.62	0.77	Solyc03g0	Solyc03g0	0.00	-9.17
GO:00312	anchored c	Cellular C	0.62	0.77	Solyc08g0	Solyc08g0	13.23	3.73
GO:00718	potassium	Biological	0.63	0.78	Solyc01g1	Solyc01g1	0.29	-1.80
GO:00718	potassium	Biological	0.63	0.78	Solyc05g0	Solyc05g0	0.49	-1.03
GO:00718	potassium	Biological	0.63	0.78	Solyc12g0	Solyc12g0	369.30	8.53
GO:00324	Rab protei	Biological	0.63	0.78	Solyc10g0	Solyc10g0	2.15	1.10
GO:00324	Rab protei	Biological	0.63	0.78	Solyc06g0	Solyc06g0	2.20	1.14
GO:00324	Rab protei	Biological	0.63	0.78	Solyc09g0	Solyc09g0	0.44	-1.20
GO:00435	ADP bindi	Molecular	0.63	0.78	Solyc02g0	Solyc02g0	0.47	-1.10
GO:00435	ADP bindi	Molecular	0.63	0.78	Solyc10g0	Solyc10g0	0.39	-1.34
GO:00435	ADP bindi	Molecular	0.63	0.78	Solyc10g0	Solyc10g0	0.39	-1.36
GO:00435	ADP bindi	Molecular	0.63	0.78	Solyc08g0	Solyc08g0	0.34	-1.57
GO:00435	ADP bindi	Molecular	0.63	0.78	Solyc01g0	Solyc01g0	0.49	-1.03
GO:00435	ADP bindi	Molecular	0.63	0.78	Solyc02g0	Solyc02g0	0.43	-1.23
GO:00435	ADP bindi	Molecular	0.63	0.78	Solyc08g0	Solyc08g0	0.35	-1.51
GO:00435	ADP bindi	Molecular	0.63	0.78	Solyc08g0	Solyc08g0	0.42	-1.25
GO:00435	ADP bindi	Molecular	0.63	0.78	Solyc07g0	Solyc07g0	0.38	-1.40
GO:00435	ADP bindi	Molecular	0.63	0.78	Solyc08g0	Solyc08g0	9.19	3.20
GO:00435	ADP bindi	Molecular	0.63	0.78	Solyc11g0	Solyc11g0	0.22	-2.19
GO:00435	ADP bindi	Molecular	0.63	0.78	Solyc07g0	Solyc07g0	0.19	-2.41
GO:00435	ADP bindi	Molecular	0.63	0.78	Solyc05g0	Solyc05g0	0.41	-1.28
GO:00435	ADP bindi	Molecular	0.63	0.78	Solyc12g0	Solyc12g0	0.00	-8.12
GO:00435	ADP bindi	Molecular	0.63	0.78	Solyc05g0	Solyc05g0	0.36	-1.48
GO:00435	ADP bindi	Molecular	0.63	0.78	Solyc09g0	Solyc09g0	362.34	8.50
GO:00435	ADP bindi	Molecular	0.63	0.78	Solyc12g0	Solyc12g0	0.00	-10.19
GO:00422	ribosome b	Biological	0.64	0.78	Solyc09g0	Solyc09g0	4.17	2.06
GO:00422	ribosome b	Biological	0.64	0.78	Solyc04g0	Solyc04g0	2.32	1.22
GO:00069	response to	Biological	0.65	0.78	Solyc04g0	Solyc04g0	2.70	1.43
GO:00152	symporter	Molecular	0.65	0.78	Solyc12g0	Solyc12g0	4.94	2.30
GO:00056	transcriptio	Cellular C	0.65	0.78	Solyc11g0	Solyc11g0	0.22	-2.21
GO:00064	translation	Biological	0.65	0.78	Solyc12g0	Solyc12g0	2.35	1.23
GO:00046	phospholip	Molecular	0.65	0.78	Solyc01g1	Solyc01g1	0.24	-2.06
GO:00096	systemic a	Biological	0.65	0.78	Solyc03g0	Solyc03g0	0.24	-2.06
GO:00089	phospholip	Molecular	0.65	0.78	Solyc01g0	Solyc01g0	2.38	1.25
GO:00068	regulation	Biological	0.65	0.78	Solyc10g0	Solyc10g0	17.76	4.15
GO:00313	regulation	Biological	0.65	0.78	Solyc12g0	Solyc12g0	3.08	1.62
GO:00994	plant orgar	Biological	0.65	0.78	Solyc03g0	Solyc03g0	2300.48	11.17
GO:00349	response to	Biological	0.65	0.78	Solyc06g0	Solyc06g0	0.23	-2.13
GO:00057	mitochond	Cellular C	0.65	0.78	Solyc03g1	Solyc03g1	2773.71	11.44
GO:00058	lipid dropl	Cellular C	0.65	0.78	Solyc12g0	Solyc12g0	2.17	1.12
GO:00083	galactosylt	Molecular	0.65	0.78	Solyc02g0	Solyc02g0	0.49	-1.03
GO:00903	nucleic aci	Biological	0.65	0.78	Solyc05g0	Solyc05g0	20.96	4.39
GO:00102	hydrotropi	Biological	0.65	0.78	Solyc03g1	Solyc03g1	0.42	-1.24
GO:00065	polyamine	Biological	0.65	0.78	Solyc08g0	Solyc08g0	0.00	-9.72
GO:00428	protein hor	Molecular	0.65	0.79	Solyc04g0	Solyc04g0	2.70	1.43
GO:00428	protein hor	Molecular	0.65	0.79	Solyc04g0	Solyc04g0	3.53	1.82
GO:00428	protein hor	Molecular	0.65	0.79	Solyc01g1	Solyc01g1	4.98	2.32
GO:00036	chromatin	Molecular	0.65	0.79	Solyc01g0	Solyc01g0	3.72	1.90

GO:00036	chromatin	Molecular	0.65	0.79	Solyc08g0	Solyc08g0	2.38	1.25
GO:00036	chromatin	Molecular	0.65	0.79	Solyc01g0	Solyc01g0	2.17	1.11
GO:00097	response to	Biological	0.65	0.79	Solyc09g0	Solyc09g0	0.05	-4.31
GO:00097	response to	Biological	0.65	0.79	Solyc04g0	Solyc04g0	2.70	1.43
GO:00097	response to	Biological	0.65	0.79	Solyc09g0	Solyc09g0	3.41	1.77
GO:00097	response to	Biological	0.65	0.79	Solyc01g1	Solyc01g1	14.86	3.89
GO:00097	response to	Biological	0.65	0.79	Solyc03g0	Solyc03g0	17.00	4.09
GO:00002	magnesium	Molecular	0.65	0.79	Solyc03g0	Solyc03g0	4.05	2.02
GO:00002	magnesium	Molecular	0.65	0.79	Solyc09g0	Solyc09g0	2.88	1.53
GO:00002	magnesium	Molecular	0.65	0.79	Solyc01g0	Solyc01g0	0.03	-5.12
GO:00002	magnesium	Molecular	0.65	0.79	Solyc08g0	Solyc08g0	0.00	-12.86
GO:00002	magnesium	Molecular	0.65	0.79	Solyc12g0	Solyc12g0	0.00	-11.26
GO:00002	magnesium	Molecular	0.65	0.79	Solyc04g0	Solyc04g0	0.41	-1.30
GO:00002	magnesium	Molecular	0.65	0.79	Solyc01g0	Solyc01g0	2.02	1.02
GO:00002	magnesium	Molecular	0.65	0.79	Solyc12g0	Solyc12g0	1818.26	10.83
GO:00002	magnesium	Molecular	0.65	0.79	Solyc08g0	Solyc08g0	8.90	3.15
GO:00002	magnesium	Molecular	0.65	0.79	Solyc06g0	Solyc06g0	12.77	3.67
GO:00081	methyltran	Molecular	0.66	0.80	Solyc03g0	Solyc03g0	2.53	1.34
GO:00081	methyltran	Molecular	0.66	0.80	Solyc06g0	Solyc06g0	2.05	1.03
GO:00081	methyltran	Molecular	0.66	0.80	Solyc08g0	Solyc08g0	2.64	1.40
GO:00081	methyltran	Molecular	0.66	0.80	Solyc03g0	Solyc03g0	0.28	-1.86
GO:00081	methyltran	Molecular	0.66	0.80	Solyc12g0	Solyc12g0	2.20	1.14
GO:00081	methyltran	Molecular	0.66	0.80	Solyc01g0	Solyc01g0	2.17	1.11
GO:00081	methyltran	Molecular	0.66	0.80	Solyc05g0	Solyc05g0	0.44	-1.19
GO:00081	methyltran	Molecular	0.66	0.80	Solyc01g0	Solyc01g0	0.32	-1.65
GO:00081	methyltran	Molecular	0.66	0.80	Solyc10g0	Solyc10g0	2.14	1.10
GO:00081	methyltran	Molecular	0.66	0.80	Solyc03g0	Solyc03g0	0.39	-1.35
GO:00081	methyltran	Molecular	0.66	0.80	Solyc02g0	Solyc02g0	34.22	5.10
GO:00081	methyltran	Molecular	0.66	0.80	Solyc09g0	Solyc09g0	529.32	9.05
GO:00081	methyltran	Molecular	0.66	0.80	Solyc01g0	Solyc01g0	23.29	4.54
GO:00160	lipid catab	Biological	0.66	0.80	Solyc06g0	Solyc06g0	2.40	1.26
GO:00160	lipid catab	Biological	0.66	0.80	Solyc10g0	Solyc10g0	11.34	3.50
GO:00310	stress-activ	Biological	0.66	0.80	Solyc07g0	Solyc07g0	2.15	1.11
GO:00310	stress-activ	Biological	0.66	0.80	Solyc02g0	Solyc02g0	2.27	1.18
GO:00310	stress-activ	Biological	0.66	0.80	Solyc07g0	Solyc07g0	2.88	1.52
GO:00483	root develo	Biological	0.66	0.80	Solyc11g0	Solyc11g0	0.50	-1.00
GO:00483	root develo	Biological	0.66	0.80	Solyc03g0	Solyc03g0	0.00	-12.14
GO:00483	root develo	Biological	0.66	0.80	Solyc11g0	Solyc11g0	0.47	-1.09
GO:00508	response to	Biological	0.67	0.80	Solyc12g0	Solyc12g0	4.94	2.30
GO:00508	response to	Biological	0.67	0.80	Solyc11g0	Solyc11g0	0.49	-1.04
GO:00508	response to	Biological	0.67	0.80	Solyc06g0	Solyc06g0	0.32	-1.62
GO:00508	response to	Biological	0.67	0.80	Solyc09g0	Solyc09g0	0.38	-1.39
GO:00166	oxidoreduc	Molecular	0.67	0.80	Solyc04g0	Solyc04g0	6.11	2.61
GO:00181	peptidyl-ty	Biological	0.67	0.80	Solyc02g0	Solyc02g0	0.44	-1.19
GO:00060	pentose-ph	Biological	0.67	0.80	Solyc01g0	Solyc01g0	3.54	1.82
GO:00072	small GTP	Biological	0.67	0.80	Solyc02g0	Solyc02g0	6.74	2.75
GO:00068	anion trans	Biological	0.67	0.80	Solyc03g1	Solyc03g1	0.39	-1.35
GO:00062	DNA repli	Biological	0.67	0.80	Solyc05g0	Solyc05g0	2.54	1.35
GO:00422	response to	Biological	0.67	0.80	Solyc06g0	Solyc06g0	0.15	-2.74
GO:00700	metalloam	Molecular	0.67	0.80	Solyc12g0	Solyc12g0	1818.26	10.83
GO:00083	voltage-ga	Molecular	0.67	0.80	Solyc03g1	Solyc03g1	2773.71	11.44

GO:00301	sphingolip	Biological	0.67	0.80	Solyc12g1	Solyc12g1	0.00	-10.11
GO:00328	regulation	Biological	0.67	0.80	Solyc06g0	Solyc06g0	2.87	1.52
GO:00096	jasmonic a	Biological	0.67	0.80	Solyc01g1	Solyc01g1	0.00	-10.11
GO:00045	ribonuclea	Molecular	0.69	0.82	Solyc11g0	Solyc11g0	0.41	-1.28
GO:00045	ribonuclea	Molecular	0.69	0.82	Solyc03g0	Solyc03g0	4.17	2.06
GO:00045	ribonuclea	Molecular	0.69	0.82	Solyc07g0	Solyc07g0	0.46	-1.11
GO:00067	glutathione	Biological	0.69	0.82	Solyc09g0	Solyc09g0	2.37	1.24
GO:00067	glutathione	Biological	0.69	0.82	Solyc09g0	Solyc09g0	2.07	1.05
GO:00067	glutathione	Biological	0.69	0.82	Solyc06g0	Solyc06g0	0.15	-2.74
GO:00067	glutathione	Biological	0.69	0.82	Solyc05g0	Solyc05g0	7.28	2.86
GO:00198	rRNA binc	Molecular	0.69	0.82	Solyc05g0	Solyc05g0	0.09	-3.44
GO:00198	rRNA binc	Molecular	0.69	0.82	Solyc11g0	Solyc11g0	0.00	-13.41
GO:00198	rRNA binc	Molecular	0.69	0.82	Solyc12g0	Solyc12g0	0.16	-2.65
GO:00198	rRNA binc	Molecular	0.69	0.82	Solyc09g0	Solyc09g0	0.25	-2.00
GO:00037	actin bindi	Molecular	0.69	0.82	Solyc10g0	Solyc10g0	2.14	1.10
GO:00037	actin bindi	Molecular	0.69	0.82	Solyc08g0	Solyc08g0	21.56	4.43
GO:00037	actin bindi	Molecular	0.69	0.82	Solyc11g0	Solyc11g0	3.66	1.87
GO:00037	actin bindi	Molecular	0.69	0.82	Solyc07g0	Solyc07g0	0.45	-1.16
GO:00190	SCF ubiqu	Cellular C	0.69	0.82	Solyc07g0	Solyc07g0	2.29	1.20
GO:00190	SCF ubiqu	Cellular C	0.69	0.82	Solyc07g0	Solyc07g0	5532.77	12.43
GO:00063	chromatin	Biological	0.69	0.82	Solyc06g0	Solyc06g0	3.14	1.65
GO:00063	chromatin	Biological	0.69	0.82	Solyc01g0	Solyc01g0	3.72	1.90
GO:00063	regulation	Biological	0.69	0.82	Solyc09g0	Solyc09g0	6.00	2.58
GO:00063	regulation	Biological	0.69	0.82	Solyc06g0	Solyc06g0	7.71	2.95
GO:00063	regulation	Biological	0.69	0.82	Solyc03g0	Solyc03g0	0.24	-2.06
GO:00063	regulation	Biological	0.69	0.82	Solyc09g0	Solyc09g0	0.25	-2.02
GO:00063	regulation	Biological	0.69	0.82	Solyc11g0	Solyc11g0	0.36	-1.47
GO:00063	regulation	Biological	0.69	0.82	Solyc04g0	Solyc04g0	2.73	1.45
GO:00063	regulation	Biological	0.69	0.82	Solyc02g0	Solyc02g0	2.17	1.12
GO:00063	regulation	Biological	0.69	0.82	Solyc01g0	Solyc01g0	3.08	1.62
GO:00063	regulation	Biological	0.69	0.82	Solyc12g0	Solyc12g0	2.65	1.40
GO:00063	regulation	Biological	0.69	0.82	Solyc09g0	Solyc09g0	0.32	-1.63
GO:00063	regulation	Biological	0.69	0.82	Solyc08g0	Solyc08g0	0.47	-1.08
GO:00063	regulation	Biological	0.69	0.82	Solyc05g0	Solyc05g0	2.40	1.27
GO:00063	regulation	Biological	0.69	0.82	Solyc04g0	Solyc04g0	0.38	-1.40
GO:00063	regulation	Biological	0.69	0.82	Solyc09g0	Solyc09g0	0.34	-1.58
GO:00063	regulation	Biological	0.69	0.82	Solyc11g0	Solyc11g0	2.29	1.19
GO:00063	regulation	Biological	0.69	0.82	Solyc06g0	Solyc06g0	0.46	-1.11
GO:00063	regulation	Biological	0.69	0.82	Solyc10g0	Solyc10g0	0.07	-3.89
GO:00063	regulation	Biological	0.69	0.82	Solyc01g0	Solyc01g0	0.44	-1.18
GO:00063	regulation	Biological	0.69	0.82	Solyc06g0	Solyc06g0	3.01	1.59
GO:00063	regulation	Biological	0.69	0.82	Solyc06g0	Solyc06g0	0.49	-1.03
GO:00063	regulation	Biological	0.69	0.82	Solyc08g0	Solyc08g0	0.49	-1.02
GO:00063	regulation	Biological	0.69	0.82	Solyc08g0	Solyc08g0	0.20	-2.35
GO:00063	regulation	Biological	0.69	0.82	Solyc06g0	Solyc06g0	0.46	-1.11
GO:00063	regulation	Biological	0.69	0.82	Solyc02g0	Solyc02g0	0.42	-1.24
GO:00063	regulation	Biological	0.69	0.82	Solyc08g0	Solyc08g0	3.77	1.91
GO:00063	regulation	Biological	0.69	0.82	Solyc09g0	Solyc09g0	0.44	-1.19
GO:00063	regulation	Biological	0.69	0.82	Solyc05g0	Solyc05g0	4.20	2.07
GO:00063	regulation	Biological	0.69	0.82	Solyc04g0	Solyc04g0	0.21	-2.27
GO:00063	regulation	Biological	0.69	0.82	Solyc02g0	Solyc02g0	0.32	-1.64

GO:00063	regulation	Biological	0.69	0.82	Solyc06g0	Solyc06g0	3.13	1.64
GO:00063	regulation	Biological	0.69	0.82	Solyc09g0	Solyc09g0	0.49	-1.02
GO:00063	regulation	Biological	0.69	0.82	Solyc11g0	Solyc11g0	2.73	1.45
GO:00063	regulation	Biological	0.69	0.82	Solyc06g0	Solyc06g0	0.35	-1.53
GO:00063	regulation	Biological	0.69	0.82	Solyc11g0	Solyc11g0	0.50	-1.00
GO:00063	regulation	Biological	0.69	0.82	Solyc08g0	Solyc08g0	4.49	2.17
GO:00063	regulation	Biological	0.69	0.82	Solyc02g0	Solyc02g0	0.42	-1.26
GO:00063	regulation	Biological	0.69	0.82	Solyc05g0	Solyc05g0	31.58	4.98
GO:00063	regulation	Biological	0.69	0.82	Solyc09g0	Solyc09g0	0.25	-1.98
GO:00063	regulation	Biological	0.69	0.82	Solyc08g0	Solyc08g0	0.37	-1.44
GO:00063	regulation	Biological	0.69	0.82	Solyc06g0	Solyc06g0	2.01	1.01
GO:00063	regulation	Biological	0.69	0.82	Solyc04g0	Solyc04g0	0.07	-3.79
GO:00063	regulation	Biological	0.69	0.82	Solyc06g0	Solyc06g0	0.39	-1.37
GO:00063	regulation	Biological	0.69	0.82	Solyc02g0	Solyc02g0	0.00	-11.29
GO:00063	regulation	Biological	0.69	0.82	Solyc03g0	Solyc03g0	2300.48	11.17
GO:00063	regulation	Biological	0.69	0.82	Solyc12g0	Solyc12g0	0.33	-1.61
GO:00063	regulation	Biological	0.69	0.82	Solyc07g0	Solyc07g0	2.33	1.22
GO:00063	regulation	Biological	0.69	0.82	Solyc11g0	Solyc11g0	0.00	-10.83
GO:00063	regulation	Biological	0.69	0.82	Solyc02g0	Solyc02g0	25.47	4.67
GO:00063	regulation	Biological	0.69	0.82	Solyc02g0	Solyc02g0	2.66	1.41
GO:00063	regulation	Biological	0.69	0.82	Solyc06g0	Solyc06g0	0.00	-10.94
GO:00063	regulation	Biological	0.69	0.82	Solyc03g0	Solyc03g0	20.56	4.36
GO:00063	regulation	Biological	0.69	0.82	Solyc06g0	Solyc06g0	0.00	-12.03
GO:00063	regulation	Biological	0.69	0.82	Solyc02g0	Solyc02g0	0.39	-1.37
GO:00063	regulation	Biological	0.69	0.82	Solyc02g0	Solyc02g0	8.19	3.03
GO:00063	regulation	Biological	0.69	0.82	Solyc02g0	Solyc02g0	0.38	-1.40
GO:00063	regulation	Biological	0.69	0.82	Solyc03g1	Solyc03g1	2361.02	11.21
GO:00063	regulation	Biological	0.69	0.82	Solyc04g0	Solyc04g0	0.00	-12.04
GO:00063	regulation	Biological	0.69	0.82	Solyc10g0	Solyc10g0	0.29	-1.77
GO:00063	regulation	Biological	0.69	0.82	Solyc03g1	Solyc03g1	734.02	9.52
GO:00063	regulation	Biological	0.69	0.82	Solyc07g0	Solyc07g0	42.77	5.42
GO:00063	regulation	Biological	0.69	0.82	Solyc10g0	Solyc10g0	683.10	9.42
GO:00063	regulation	Biological	0.69	0.82	Solyc02g0	Solyc02g0	1446.12	10.50
GO:00063	regulation	Biological	0.69	0.82	Solyc03g0	Solyc03g0	3218.95	11.65
GO:00063	regulation	Biological	0.69	0.82	Solyc01g0	Solyc01g0	6.82	2.77
GO:00063	regulation	Biological	0.69	0.82	Solyc01g1	Solyc01g1	3.24	1.69
GO:00063	regulation	Biological	0.69	0.82	Solyc10g0	Solyc10g0	2.60	1.38
GO:00063	regulation	Biological	0.69	0.82	Solyc04g0	Solyc04g0	1227.78	10.26
GO:00063	regulation	Biological	0.69	0.82	Solyc06g0	Solyc06g0	15.65	3.97
GO:00063	regulation	Biological	0.69	0.82	Solyc01g0	Solyc01g0	0.48	-1.06
GO:00063	regulation	Biological	0.69	0.82	Solyc06g0	Solyc06g0	8.85	3.15
GO:00063	regulation	Biological	0.69	0.82	Solyc05g0	Solyc05g0	3.77	1.92
GO:00063	regulation	Biological	0.69	0.82	Solyc06g0	Solyc06g0	0.45	-1.16
GO:00063	regulation	Biological	0.69	0.82	Solyc02g0	Solyc02g0	1506.28	10.56
GO:00063	regulation	Biological	0.69	0.82	Solyc08g0	Solyc08g0	2.07	1.05
GO:00063	regulation	Biological	0.69	0.82	Solyc03g0	Solyc03g0	0.46	-1.13
GO:00063	regulation	Biological	0.69	0.82	Solyc11g0	Solyc11g0	0.47	-1.09
GO:00063	regulation	Biological	0.69	0.82	Solyc08g0	Solyc08g0	3.29	1.72
GO:00063	regulation	Biological	0.69	0.82	Solyc01g0	Solyc01g0	0.24	-2.09
GO:00063	regulation	Biological	0.69	0.82	Solyc06g0	Solyc06g0	0.45	-1.16
GO:00063	regulation	Biological	0.69	0.82	Solyc02g0	Solyc02g0	0.06	-4.03

GO:00063	regulation	Biological	0.69	0.82	Solyc06g0	PTI6	0.43	-1.22
GO:00063	regulation	Biological	0.69	0.82	Solyc08g0	Solyc08g0	0.41	-1.27
GO:00063	regulation	Biological	0.69	0.82	Solyc01g0	Solyc01g0	0.45	-1.14
GO:00063	regulation	Biological	0.69	0.82	Solyc04g0	Solyc04g0	2.34	1.23
GO:00063	regulation	Biological	0.69	0.82	Solyc10g0	Solyc10g0	2.42	1.27
GO:00063	regulation	Biological	0.69	0.82	Solyc01g1	Solyc01g1	0.37	-1.43
GO:00063	regulation	Biological	0.69	0.82	Solyc05g0	Solyc05g0	6.61	2.73
GO:00063	regulation	Biological	0.69	0.82	Solyc05g0	Solyc05g0	0.00	-10.39
GO:00063	regulation	Biological	0.69	0.82	Solyc09g0	Solyc09g0	0.22	-2.22
GO:00063	regulation	Biological	0.69	0.82	Solyc11g0	Solyc11g0	0.00	-9.22
GO:00063	regulation	Biological	0.69	0.82	Solyc02g0	Solyc02g0	7.52	2.91
GO:00324	positive re	Biological	0.69	0.82	Solyc03g1	Solyc03g1	0.35	-1.54
GO:00166	oxidoreduc	Molecular	0.69	0.82	Solyc06g0	Solyc06g0	2.15	1.10
GO:00325	mitochond	Biological	0.69	0.82	Solyc05g0	Solyc05g0	0.09	-3.44
GO:00507	regulation	Biological	0.69	0.82	Solyc06g0	Solyc06g0	4.81	2.27
GO:00167	cellulose s	Molecular	0.69	0.82	Solyc12g0	Solyc12g0	3.03	1.60
GO:00066	protein tar	Biological	0.69	0.82	Solyc05g0	Solyc05g0	2.29	1.20
GO:00182	peptidyl-L	Biological	0.69	0.82	Solyc05g0	Solyc05g0	2.29	1.20
GO:00047	protein tyr	Molecular	0.69	0.82	Solyc04g0	Solyc04g0	0.18	-2.51
GO:00301	integral co	Cellular C	0.69	0.82	Solyc08g0	Solyc08g0	6.46	2.69
GO:00096	cytokinin b	Biological	0.69	0.82	Solyc11g0	Solyc11g0	0.46	-1.12
GO:00076	circadian r	Biological	0.69	0.82	Solyc08g0	Solyc08g0	0.36	-1.48
GO:00058	integral co	Cellular C	0.72	0.84	Solyc12g0	Solyc12g0	4.94	2.30
GO:00058	integral co	Cellular C	0.72	0.84	Solyc11g0	Solyc11g0	0.49	-1.04
GO:00058	integral co	Cellular C	0.72	0.84	Solyc04g0	Solyc04g0	0.41	-1.29
GO:00058	integral co	Cellular C	0.72	0.84	Solyc04g0	Solyc04g0	5759.74	12.49
GO:00058	integral co	Cellular C	0.72	0.84	Solyc09g0	Solyc09g0	0.00	-8.11
GO:20003	regulation	Biological	0.72	0.84	Solyc04g0	Solyc04g0	0.22	-2.15
GO:00801	regulation	Biological	0.72	0.84	Solyc09g0	Solyc09g0	0.27	-1.90
GO:00325	DNA dupl	Biological	0.72	0.84	Solyc01g1	Solyc01g1	2.32	1.22
GO:00197	protein-cy	Molecular	0.72	0.84	Solyc05g0	Solyc05g0	2.29	1.20
GO:00168	intramolec	Molecular	0.72	0.84	Solyc12g0	Solyc12g0	2.17	1.12
GO:00469	pectinester	Molecular	0.72	0.85	Solyc03g1	Solyc03g1	5.44	2.44
GO:00469	pectinester	Molecular	0.72	0.85	Solyc03g1	Solyc03g1	0.44	-1.20
GO:00168	ligase acti	Molecular	0.72	0.85	Solyc03g0	Solyc03g0	0.34	-1.54
GO:00168	ligase acti	Molecular	0.72	0.85	Solyc01g0	Solyc01g0	550.66	9.11
GO:00058	trans-Golg	Cellular C	0.73	0.85	Solyc02g0	Solyc02g0	3.63	1.86
GO:00058	trans-Golg	Cellular C	0.73	0.85	Solyc05g0	Solyc05g0	0.44	-1.19
GO:00058	trans-Golg	Cellular C	0.73	0.85	Solyc12g0	Solyc12g0	3.10	1.63
GO:00058	trans-Golg	Cellular C	0.73	0.85	Solyc12g0	Solyc12g0	3.03	1.60
GO:00058	trans-Golg	Cellular C	0.73	0.85	Solyc01g0	Solyc01g0	0.19	-2.40
GO:00058	trans-Golg	Cellular C	0.73	0.85	Solyc02g0	Solyc02g0	0.49	-1.03
GO:00062	DNA repli	Biological	0.73	0.85	Solyc01g1	Solyc01g1	2.32	1.22
GO:00062	DNA repli	Biological	0.73	0.85	Solyc05g0	Solyc05g0	2.54	1.35
GO:00062	DNA repli	Biological	0.73	0.85	Solyc03g0	Solyc03g0	0.00	-11.07
GO:00062	DNA repli	Biological	0.73	0.85	Solyc09g0	Solyc09g0	2.32	1.21
GO:00062	DNA repli	Biological	0.73	0.85	Solyc01g0	Solyc01g0	312.22	8.29
GO:00062	DNA repli	Biological	0.73	0.85	Solyc02g0	Solyc02g0	0.00	-10.40
GO:00015	RNA meth	Biological	0.74	0.85	Solyc03g0	Solyc03g0	0.28	-1.86
GO:00039	NAD+ kin	Molecular	0.74	0.85	Solyc03g0	Solyc03g0	2.33	1.22
GO:00057	mitochond	Cellular C	0.74	0.85	Solyc05g0	Solyc05g0	0.09	-3.44

GO:00441	protein fol	Molecular	0.74	0.85	Solyc06g0	Solyc06g0	2.09	1.07
GO:00070	cell cycle	Biological	0.74	0.85	Solyc10g0	Solyc10g0	2.14	1.10
GO:00080	ATP-deper	Molecular	0.74	0.85	Solyc09g0	Solyc09g0	0.35	-1.51
GO:00164	myosin coı	Cellular C	0.74	0.85	Solyc10g0	Solyc10g0	2.78	1.48
GO:00056	U5 snRNP	Cellular C	0.74	0.85	Solyc06g0	Solyc06g0	0.46	-1.11
GO:00038	3-hydroxyi	Molecular	0.74	0.85	Solyc01g1	Solyc01g1	4.73	2.24
GO:00047	MAP kina:	Molecular	0.74	0.85	Solyc08g0	Solyc08g0	0.41	-1.30
GO:00001	exocyst	Cellular C	0.74	0.85	Solyc06g0	Solyc06g0	0.16	-2.68
GO:00001	exocyst	Cellular C	0.74	0.85	Solyc10g0	Solyc10g0	0.13	-2.96
GO:00159	large ribos	Cellular C	0.74	0.85	Solyc05g0	Solyc05g0	0.09	-3.44
GO:00159	large ribos	Cellular C	0.74	0.85	Solyc04g0	Solyc04g0	2.32	1.22
GO:00302	carbohydra	Molecular	0.75	0.86	Solyc03g1	Solyc03g1	2.20	1.14
GO:00302	carbohydra	Molecular	0.75	0.86	Solyc08g0	Solyc08g0	2.18	1.12
GO:00302	carbohydra	Molecular	0.75	0.86	Solyc02g0	Solyc02g0	2.97	1.57
GO:00302	carbohydra	Molecular	0.75	0.86	Solyc04g0	Solyc04g0	0.37	-1.44
GO:00302	carbohydra	Molecular	0.75	0.86	Solyc10g0	Solyc10g0	4.22	2.08
GO:00302	carbohydra	Molecular	0.75	0.86	Solyc04g0	Solyc04g0	0.21	-2.27
GO:00163	kinase acti	Molecular	0.75	0.86	Solyc05g0	Solyc05g0	4.45	2.16
GO:00163	kinase acti	Molecular	0.75	0.86	Solyc09g0	Solyc09g0	2.88	1.53
GO:00163	kinase acti	Molecular	0.75	0.86	Solyc03g0	Solyc03g0	2.33	1.22
GO:00163	kinase acti	Molecular	0.75	0.86	Solyc02g0	Solyc02g0	0.44	-1.19
GO:00163	kinase acti	Molecular	0.75	0.86	Solyc01g0	Solyc01g0	0.42	-1.26
GO:00163	kinase acti	Molecular	0.75	0.86	Solyc03g0	Solyc03g0	2.06	1.04
GO:00163	kinase acti	Molecular	0.75	0.86	Solyc08g0	Solyc08g0	2.43	1.28
GO:00163	kinase acti	Molecular	0.75	0.86	Solyc10g0	Solyc10g0	4.22	2.08
GO:00163	kinase acti	Molecular	0.75	0.86	Solyc12g0	Solyc12g0	3.17	1.66
GO:00163	kinase acti	Molecular	0.75	0.86	Solyc06g0	Solyc06g0	912.98	9.83
GO:00163	kinase acti	Molecular	0.75	0.86	Solyc08g0	Solyc08g0	0.41	-1.30
GO:00163	kinase acti	Molecular	0.75	0.86	Solyc02g0	Solyc02g0	307.12	8.26
GO:00163	kinase acti	Molecular	0.75	0.86	Solyc07g0	Solyc07g0	2.39	1.26
GO:00163	kinase acti	Molecular	0.75	0.86	Solyc03g1	Solyc03g1	356.83	8.48
GO:00168	isomerase	Molecular	0.75	0.86	Solyc04g0	Solyc04g0	3.78	1.92
GO:00168	isomerase	Molecular	0.75	0.86	Solyc09g0	Solyc09g0	0.37	-1.45
GO:00168	isomerase	Molecular	0.75	0.86	Solyc12g0	Solyc12g0	2.17	1.12
GO:00469	proton-trar	Molecular	0.75	0.86	Solyc06g0	Solyc06g0	15.51	3.95
GO:00469	proton-trar	Molecular	0.75	0.86	Solyc11g0	Solyc11g0	0.22	-2.19
GO:00057	vacuole	Cellular C	0.75	0.86	Solyc03g1	Solyc03g1	2.20	1.14
GO:00057	vacuole	Cellular C	0.75	0.86	Solyc10g0	CHI9	0.28	-1.84
GO:00057	vacuole	Cellular C	0.75	0.86	Solyc06g0	Solyc06g0	0.40	-1.32
GO:00057	vacuole	Cellular C	0.75	0.86	Solyc06g0	Solyc06g0	2.02	1.02
GO:00057	vacuole	Cellular C	0.75	0.86	Solyc07g0	Solyc07g0	2.40	1.26
GO:00454	xylan bios	Biological	0.76	0.86	Solyc01g1	Solyc01g1	0.30	-1.73
GO:00095	chloroplas	Cellular C	0.76	0.86	Solyc01g1	Solyc01g1	6.83	2.77
GO:00704	NAD+ bin	Molecular	0.76	0.86	Solyc09g0	Solyc09g0	4.17	2.06
GO:00310	heat shock	Molecular	0.76	0.86	Solyc06g0	Solyc06g0	2.09	1.07
GO:00468	phosphatid	Biological	0.76	0.86	Solyc04g0	Solyc04g0	0.41	-1.30
GO:00464	phosphatid	Biological	0.76	0.86	Solyc10g0	Solyc10g0	2.19	1.13
GO:00472	polygalact	Molecular	0.76	0.86	Solyc02g0	Solyc02g0	0.46	-1.13
GO:00001	protein ph	Cellular C	0.76	0.86	Solyc06g0	Solyc06g0	10.22	3.35
GO:00150	glucuronos	Molecular	0.76	0.86	Solyc09g0	Solyc09g0	0.44	-1.19
GO:00087	S-adenosyl	Molecular	0.76	0.87	Solyc05g0	Solyc05g0	0.44	-1.19

GO:00087	S-adenosyl	Molecular	0.76	0.87	Solyc06g0	Solyc06g0	2.08	1.06
GO:00087	S-adenosyl	Molecular	0.76	0.87	Solyc03g0	Solyc03g0	0.39	-1.35
GO:00301	integral co	Cellular C	0.77	0.87	Solyc04g0	Solyc04g0	4.53	2.18
GO:00301	integral co	Cellular C	0.77	0.87	Solyc08g0	Solyc08g0	6.46	2.69
GO:00070	microtubul	Biological	0.77	0.87	Solyc07g0	Solyc07g0	3.21	1.68
GO:00070	microtubul	Biological	0.77	0.87	Solyc03g1	Solyc03g1	2.39	1.26
GO:00056	obsolete ce	Cellular C	0.77	0.87	Solyc07g0	Solyc07g0	9.18	3.20
GO:00056	obsolete ce	Cellular C	0.77	0.87	Solyc04g0	Solyc04g0	2.43	1.28
GO:00056	obsolete ce	Cellular C	0.77	0.87	Solyc05g0	Solyc05g0	3.22	1.69
GO:00056	obsolete ce	Cellular C	0.77	0.87	Solyc10g0	Solyc10g0	2.07	1.05
GO:00166	oxidoreduc	Molecular	0.77	0.87	Solyc04g0	Solyc04g0	2.43	1.28
GO:00001	negative re	Biological	0.77	0.87	Solyc11g0	Solyc11g0	0.22	-2.21
GO:00055	phospholip	Molecular	0.77	0.87	Solyc04g0	Solyc04g0	0.22	-2.15
GO:00482	clathrin co	Biological	0.77	0.87	Solyc04g0	Solyc04g0	0.22	-2.15
GO:00302	clathrin bi	Molecular	0.77	0.87	Solyc04g0	Solyc04g0	2.07	1.05
GO:00525	cell wall p	Biological	0.77	0.87	Solyc05g0	Solyc05g0	0.44	-1.19
GO:00085	obsolete p	Molecular	0.77	0.87	Solyc03g0	Solyc03g0	0.49	-1.02
GO:00068	endocytosi	Biological	0.77	0.87	Solyc11g0	Solyc11g0	2.57	1.36
GO:00167	transferase	Molecular	0.77	0.87	Solyc09g0	Solyc09g0	3.57	1.84
GO:00163	phosphatid	Molecular	0.77	0.87	Solyc10g0	Solyc10g0	2.19	1.13
GO:00300	CCR4-NO	Cellular C	0.77	0.87	Solyc07g0	Solyc07g0	0.40	-1.31
GO:00082	serine-type	Molecular	0.78	0.88	Solyc01g1	Solyc01g1	0.44	-1.18
GO:00082	serine-type	Molecular	0.78	0.88	Solyc07g0	Solyc07g0	2.17	1.12
GO:00082	serine-type	Molecular	0.78	0.88	Solyc09g0	Solyc09g0	0.12	-3.08
GO:00082	serine-type	Molecular	0.78	0.88	Solyc04g0	Solyc04g0	0.43	-1.23
GO:00082	serine-type	Molecular	0.78	0.88	Solyc09g0	Solyc09g0	3042	11.57
GO:00082	serine-type	Molecular	0.78	0.88	Solyc01g0	Solyc01g0	0.48	-1.06
GO:00167	glycosyltra	Molecular	0.78	0.88	Solyc09g0	Solyc09g0	0.05	-4.31
GO:00167	glycosyltra	Molecular	0.78	0.88	Solyc01g1	Solyc01g1	4.75	2.25
GO:00167	glycosyltra	Molecular	0.78	0.88	Solyc09g0	Solyc09g0	7.65	2.93
GO:00167	glycosyltra	Molecular	0.78	0.88	Solyc08g0	Solyc08g0	2.92	1.55
GO:00167	glycosyltra	Molecular	0.78	0.88	Solyc06g0	Solyc06g0	2.15	1.10
GO:00167	glycosyltra	Molecular	0.78	0.88	Solyc05g0	Solyc05g0	4.78	2.26
GO:00167	glycosyltra	Molecular	0.78	0.88	Solyc09g0	Solyc09g0	2.71	1.44
GO:00167	glycosyltra	Molecular	0.78	0.88	Solyc05g0	Solyc05g0	0.00	-11.21
GO:00167	glycosyltra	Molecular	0.78	0.88	Solyc09g0	Solyc09g0	0.47	-1.09
GO:00167	glycosyltra	Molecular	0.78	0.88	Solyc02g0	Solyc02g0	0.46	-1.13
GO:00167	glycosyltra	Molecular	0.78	0.88	Solyc11g0	Solyc11g0	0.25	-2.02
GO:00167	glycosyltra	Molecular	0.78	0.88	Solyc12g0	Solyc12g0	0.00	-9.95
GO:00167	glycosyltra	Molecular	0.78	0.88	Solyc02g0	Solyc02g0	0.49	-1.03
GO:00167	glycosyltra	Molecular	0.78	0.88	Solyc03g0	Solyc03g0	0.43	-1.23
GO:00167	glycosyltra	Molecular	0.78	0.88	Solyc09g0	Solyc09g0	0.44	-1.19
GO:01403	ABC-type	Molecular	0.79	0.88	Solyc09g0	Solyc09g0	4.52	2.18
GO:01403	ABC-type	Molecular	0.79	0.88	Solyc06g0	Solyc06g0	0.42	-1.25
GO:01403	ABC-type	Molecular	0.79	0.88	Solyc06g0	Solyc06g0	0.00	-9.49
GO:01403	ABC-type	Molecular	0.79	0.88	Solyc12g1	Solyc12g1	249.94	7.97
GO:01403	ABC-type	Molecular	0.79	0.88	Solyc03g1	Solyc03g1	0.00	-8.90
GO:00001	ubiquitin li	Cellular C	0.79	0.88	Solyc03g1	Solyc03g1	0.35	-1.54
GO:00041	endopeptic	Molecular	0.79	0.88	Solyc04g0	Solyc04g0	0.43	-1.23
GO:00066	fatty acid b	Biological	0.79	0.88	Solyc10g0	Solyc10g0	2.27	1.19
GO:00058	proteasom	Cellular C	0.79	0.88	Solyc03g0	Solyc03g0	30231.28	14.88

GO:00064	protein N-l	Biological	0.79	0.88	Solyc07g0	Solyc07g0	3.48	1.80
GO:00102	cellulose n	Biological	0.79	0.88	Solyc03g0	Solyc03g0	0.00	-9.17
GO:00167	phosphatas	Molecular	0.79	0.88	Solyc03g0	Solyc03g0	2.27	1.18
GO:00167	phosphatas	Molecular	0.79	0.88	Solyc06g0	Solyc06g0	0.18	-2.44
GO:00167	phosphatas	Molecular	0.79	0.88	Solyc05g0	Solyc05g0	3.20	1.68
GO:00167	phosphatas	Molecular	0.79	0.88	Solyc10g0	Solyc10g0	2.23	1.16
GO:00167	phosphatas	Molecular	0.79	0.88	Solyc12g0	Solyc12g0	2.88	1.52
GO:00167	phosphatas	Molecular	0.79	0.88	Solyc06g0	Solyc06g0	0.14	-2.82
GO:00167	phosphatas	Molecular	0.79	0.88	Solyc07g0	Solyc07g0	8.04	3.01
GO:00167	phosphatas	Molecular	0.79	0.88	Solyc09g0	Solyc09g0	25.41	4.67
GO:00167	phosphatas	Molecular	0.79	0.88	Solyc09g0	Solyc09g0	3.37	1.75
GO:00167	phosphatas	Molecular	0.79	0.88	Solyc01g0	Solyc01g0	25.34	4.66
GO:00167	phosphatas	Molecular	0.79	0.88	Solyc07g0	Solyc07g0	7.27	2.86
GO:19026	proton tran	Biological	0.79	0.89	Solyc10g0	Solyc10g0	0.03	-5.05
GO:19026	proton tran	Biological	0.79	0.89	Solyc06g0	Solyc06g0	15.51	3.95
GO:19026	proton tran	Biological	0.79	0.89	Solyc11g0	Solyc11g0	0.22	-2.19
GO:19026	proton tran	Biological	0.79	0.89	Solyc12g0	Solyc12g0	4.99	2.32
GO:00985	defense res	Biological	0.80	0.89	Solyc07g0	Solyc07g0	2.29	1.20
GO:00985	defense res	Biological	0.80	0.89	Solyc10g0	Solyc10g0	3.83	1.94
GO:00985	defense res	Biological	0.80	0.89	Solyc09g0	Solyc09g0	0.47	-1.07
GO:00985	defense res	Biological	0.80	0.89	Solyc03g0	Solyc03g0	0.34	-1.57
GO:00985	defense res	Biological	0.80	0.89	Solyc02g0	Solyc02g0	307.12	8.26
GO:00985	defense res	Biological	0.80	0.89	Solyc05g0	Solyc05g0	0.36	-1.48
GO:00985	defense res	Biological	0.80	0.89	Solyc05g0	Solyc05g0	0.31	-1.68
GO:00985	defense res	Biological	0.80	0.89	Solyc11g0	Solyc11g0	0.30	-1.71
GO:00043	helicase ac	Molecular	0.80	0.89	Solyc07g0	Solyc07g0	2.20	1.14
GO:00043	helicase ac	Molecular	0.80	0.89	Solyc01g1	Solyc01g1	2.32	1.22
GO:00043	helicase ac	Molecular	0.80	0.89	Solyc03g0	Solyc03g0	0.44	-1.19
GO:00043	helicase ac	Molecular	0.80	0.89	Solyc06g0	Solyc06g0	0.46	-1.11
GO:00321	activation	Biological	0.80	0.89	Solyc07g0	Solyc07g0	2.15	1.11
GO:00321	activation	Biological	0.80	0.89	Solyc02g0	Solyc02g0	2.27	1.18
GO:00321	activation	Biological	0.80	0.89	Solyc07g0	Solyc07g0	2.88	1.52
GO:00036	double-stra	Molecular	0.80	0.89	Solyc09g0	Solyc09g0	0.49	-1.02
GO:00036	double-stra	Molecular	0.80	0.89	Solyc10g0	Solyc10g0	5392.75	12.40
GO:00036	double-stra	Molecular	0.80	0.89	Solyc01g1	Solyc01g1	0.37	-1.43
GO:00007	chromatin	Cellular Co	0.80	0.89	Solyc01g0	Solyc01g0	2.56	1.36
GO:00420	protein ref	Biological	0.80	0.89	Solyc06g0	Solyc06g0	2.09	1.07
GO:00510	chaperone	Biological	0.80	0.89	Solyc06g0	Solyc06g0	2.09	1.07
GO:00170	cytochrom	Biological	0.80	0.89	Solyc11g0	Solyc11g0	0.00	-13.30
GO:00045	ribonuclea	Molecular	0.80	0.89	Solyc01g0	Solyc01g0	0.31	-1.69
GO:00198	protein pho	Molecular	0.80	0.89	Solyc06g0	Solyc06g0	10.22	3.35
GO:00102	vegetative	Biological	0.80	0.89	Solyc11g0	Solyc11g0	0.47	-1.09
GO:00171	negative re	Biological	0.80	0.89	Solyc07g0	Solyc07g0	0.40	-1.31
GO:00097	response to	Biological	0.81	0.90	Solyc11g0	Solyc11g0	0.28	-1.84
GO:00097	response to	Biological	0.81	0.90	Solyc01g1	Solyc01g1	0.37	-1.43
GO:00097	response to	Biological	0.81	0.90	Solyc01g1	Solyc01g1	4.02	2.01
GO:00097	response to	Biological	0.81	0.90	Solyc07g0	Solyc07g0	8.01	3.00
GO:00097	response to	Biological	0.81	0.90	Solyc06g0	Solyc06g0	4013.78	11.97
GO:00097	response to	Biological	0.81	0.90	Solyc03g1	Solyc03g1	0.32	-1.65
GO:00097	response to	Biological	0.81	0.90	Solyc09g0	Solyc09g0	13.30	3.73
GO:00097	cytokinin-a	Biological	0.81	0.90	Solyc01g0	Solyc01g0	2.26	1.17

GO:00097	cytokinin-a	Biological	0.81	0.90	Solyc10g0	Solyc10g0	0.50	-1.01
GO:00054	SNAP rece	Molecular	0.81	0.90	Solyc06g0	Solyc06g0	16.51	4.04
GO:00054	SNAP rece	Molecular	0.81	0.90	Solyc09g0	Solyc09g0	2.01	1.01
GO:00096	response tc	Biological	0.81	0.90	Solyc09g0	Solyc09g0	10879.94	13.41
GO:00096	response tc	Biological	0.81	0.90	Solyc12g0	Solyc12g0	3.08	1.62
GO:00007	double-stra	Biological	0.82	0.90	Solyc01g1	Solyc01g1	2.32	1.22
GO:00037	RNA helic	Molecular	0.82	0.90	Solyc06g0	Solyc06g0	9.47	3.24
GO:00037	RNA helic	Molecular	0.82	0.90	Solyc11g0	Solyc11g0	0.47	-1.10
GO:00037	RNA helic	Molecular	0.82	0.90	Solyc06g0	Solyc06g0	0.46	-1.11
GO:00302	polysaccha	Molecular	0.82	0.91	Solyc01g0	XTH1	5.24	2.39
GO:00302	polysaccha	Molecular	0.82	0.91	Solyc03g0	Solyc03g0	2.01	1.01
GO:00057	endoplasm	Cellular Co	0.82	0.91	Solyc02g0	Solyc02g0	0.50	-1.01
GO:00057	endoplasm	Cellular Co	0.82	0.91	Solyc01g0	Solyc01g0	0.35	-1.51
GO:00057	endoplasm	Cellular Co	0.82	0.91	Solyc09g0	Solyc09g0	3.57	1.84
GO:00057	endoplasm	Cellular Co	0.82	0.91	Solyc06g0	Solyc06g0	2.08	1.06
GO:00057	endoplasm	Cellular Co	0.82	0.91	Solyc04g0	Solyc04g0	5759.74	12.49
GO:00057	endoplasm	Cellular Co	0.82	0.91	Solyc12g1	Solyc12g1	0.00	-10.11
GO:00057	endoplasm	Cellular Co	0.82	0.91	Solyc02g0	Solyc02g0	8.45	3.08
GO:00465	U4/U6 x U	Cellular Co	0.83	0.91	Solyc06g0	Solyc06g0	0.46	-1.11
GO:00164	palmitoyltı	Molecular	0.83	0.91	Solyc05g0	Solyc05g0	2.29	1.20
GO:00104	regulation	Biological	0.83	0.91	Solyc11g0	Solyc11g0	10.55	3.40
GO:00104	regulation	Biological	0.83	0.91	Solyc07g0	Solyc07g0	0.39	-1.35
GO:00150	protein tra	Biological	0.84	0.92	Solyc06g0	Solyc06g0	0.16	-2.68
GO:00150	protein tra	Biological	0.84	0.92	Solyc10g0	Solyc10g0	0.13	-2.96
GO:00150	protein tra	Biological	0.84	0.92	Solyc11g0	Solyc11g0	0.02	-5.38
GO:00150	protein tra	Biological	0.84	0.92	Solyc03g1	Solyc03g1	0.49	-1.02
GO:00150	protein tra	Biological	0.84	0.92	Solyc11g0	Solyc11g0	0.09	-3.49
GO:00150	protein tra	Biological	0.84	0.92	Solyc11g0	Solyc11g0	0.03	-5.20
GO:00150	protein tra	Biological	0.84	0.92	Solyc10g0	Solyc10g0	0.06	-4.16
GO:00097	embryo de	Biological	0.84	0.92	Solyc10g0	LE25	6.62	2.73
GO:00097	embryo de	Biological	0.84	0.92	Solyc11g0	Solyc11g0	18.97	4.25
GO:00069	nucleocyto	Biological	0.84	0.92	Solyc11g0	Solyc11g0	0.05	-4.30
GO:00150	potassium	Molecular	0.84	0.92	Solyc05g0	Solyc05g0	0.49	-1.03
GO:00304	production	Biological	0.84	0.92	Solyc07g0	Solyc07g0	0.46	-1.11
GO:00009	RNA polyı	Molecular	0.85	0.92	Solyc06g0	Solyc06g0	7.71	2.95
GO:00009	RNA polyı	Molecular	0.85	0.92	Solyc11g0	Solyc11g0	2.29	1.19
GO:00009	RNA polyı	Molecular	0.85	0.92	Solyc11g0	Solyc11g0	0.47	-1.09
GO:01406	ATP-depeı	Molecular	0.85	0.92	Solyc07g0	Solyc07g0	2.20	1.14
GO:01406	ATP-depeı	Molecular	0.85	0.92	Solyc07g0	Solyc07g0	2.62	1.39
GO:01406	ATP-depeı	Molecular	0.85	0.92	Solyc01g0	Solyc01g0	2.62	1.39
GO:00057	endosome	Cellular Co	0.85	0.92	Solyc02g0	Solyc02g0	3.63	1.86
GO:00057	endosome	Cellular Co	0.85	0.92	Solyc05g0	Solyc05g0	0.44	-1.19
GO:00057	endosome	Cellular Co	0.85	0.92	Solyc12g0	Solyc12g0	3.10	1.63
GO:00057	endosome	Cellular Co	0.85	0.92	Solyc02g0	Solyc02g0	0.49	-1.03
GO:00163	phosphory	Biological	0.85	0.92	Solyc02g0	Solyc02g0	0.44	-1.19
GO:00163	phosphory	Biological	0.85	0.92	Solyc01g0	Solyc01g0	0.42	-1.26
GO:00163	phosphory	Biological	0.85	0.92	Solyc10g0	Solyc10g0	4.22	2.08
GO:00163	phosphory	Biological	0.85	0.92	Solyc12g0	Solyc12g0	3.17	1.66
GO:00163	phosphory	Biological	0.85	0.92	Solyc11g0	Solyc11g0	2.98	1.58
GO:00163	phosphory	Biological	0.85	0.92	Solyc06g0	Solyc06g0	912.98	9.83
GO:00163	phosphory	Biological	0.85	0.92	Solyc08g0	Solyc08g0	0.41	-1.30

GO:00163	phosphory	Biological	0.85	0.92	Solyc02g0	Solyc02g0	307.12	8.26
GO:00163	phosphory	Biological	0.85	0.92	Solyc07g0	Solyc07g0	2.39	1.26
GO:00163	phosphory	Biological	0.85	0.92	Solyc03g1	Solyc03g1	356.83	8.48
GO:00426	ATPase-cc	Molecular	0.85	0.92	Solyc04g0	Solyc04g0	7.72	2.95
GO:00426	ATPase-cc	Molecular	0.85	0.92	Solyc03g1	Solyc03g1	0.00	-8.90
GO:00454	cell redox	Biological	0.85	0.92	Solyc10g0	Solyc10g0	4.23	2.08
GO:00454	cell redox	Biological	0.85	0.92	Solyc07g0	Solyc07g0	9.18	3.20
GO:00454	cell redox	Biological	0.85	0.92	Solyc04g0	Solyc04g0	2.43	1.28
GO:00454	cell redox	Biological	0.85	0.92	Solyc05g0	Solyc05g0	3.22	1.69
GO:00454	cell redox	Biological	0.85	0.92	Solyc10g0	Solyc10g0	2.07	1.05
GO:00001	nucleotide	Molecular	0.85	0.92	Solyc08g0	Solyc08g0	3.99	2.00
GO:00001	nucleotide	Molecular	0.85	0.92	Solyc02g0	Solyc02g0	0.44	-1.19
GO:00001	nucleotide	Molecular	0.85	0.92	Solyc01g1	Solyc01g1	2.32	1.22
GO:00001	nucleotide	Molecular	0.85	0.92	Solyc06g0	Solyc06g0	2.09	1.07
GO:00001	nucleotide	Molecular	0.85	0.92	Solyc09g0	Solyc09g0	0.35	-1.51
GO:00001	nucleotide	Molecular	0.85	0.92	Solyc04g0	Solyc04g0	0.00	-11.98
GO:00001	nucleotide	Molecular	0.85	0.92	Solyc04g0	Solyc04g0	0.00	-13.91
GO:00001	nucleotide	Molecular	0.85	0.92	Solyc10g0	Solyc10g0	0.04	-4.70
GO:00001	nucleotide	Molecular	0.85	0.92	Solyc12g0	Solyc12g0	0.00	-13.90
GO:00001	nucleotide	Molecular	0.85	0.92	Solyc05g0	Solyc05g0	0.00	-14.18
GO:00001	nucleotide	Molecular	0.85	0.92	Solyc08g0	Solyc08g0	2.67	1.41
GO:00001	nucleotide	Molecular	0.85	0.92	Solyc10g0	Solyc10g0	0.00	-12.76
GO:00001	nucleotide	Molecular	0.85	0.92	Solyc10g0	Solyc10g0	4.22	2.08
GO:00001	nucleotide	Molecular	0.85	0.92	Solyc12g0	Solyc12g0	3.17	1.66
GO:00001	nucleotide	Molecular	0.85	0.92	Solyc03g0	Solyc03g0	2.40	1.26
GO:00001	nucleotide	Molecular	0.85	0.92	Solyc08g0	Solyc08g0	0.41	-1.30
GO:00001	nucleotide	Molecular	0.85	0.92	Solyc02g0	Solyc02g0	307.12	8.26
GO:00001	nucleotide	Molecular	0.85	0.92	Solyc12g0	Solyc12g0	467.31	8.87
GO:00001	nucleotide	Molecular	0.85	0.92	Solyc06g0	Solyc06g0	15.51	3.95
GO:00001	nucleotide	Molecular	0.85	0.92	Solyc04g0	Solyc04g0	0.06	-3.97
GO:00001	nucleotide	Molecular	0.85	0.92	Solyc07g0	Solyc07g0	2.39	1.26
GO:00001	nucleotide	Molecular	0.85	0.92	Solyc11g0	Solyc11g0	0.22	-2.19
GO:00001	nucleotide	Molecular	0.85	0.92	Solyc03g1	Solyc03g1	356.83	8.48
GO:00001	nucleotide	Molecular	0.85	0.92	Solyc12g0	Solyc12g0	4.99	2.32
GO:00001	phosphorel	Molecular	0.85	0.92	Solyc01g0	Solyc01g0	3.08	1.62
GO:00086	cellular an	Biological	0.85	0.92	Solyc01g0	Solyc01g0	2.09	1.06
GO:00051	structural r	Molecular	0.85	0.92	Solyc03g1	Solyc03g1	0.34	-1.56
GO:00161	diterpenoic	Biological	0.85	0.92	Solyc08g0	Solyc08g0	8.90	3.15
GO:00150	DNA integ	Biological	0.85	0.92	Solyc10g0	Solyc10g0	0.00	-10.24
GO:00905	RNA phos	Biological	0.85	0.92	Solyc01g0	Solyc01g0	0.31	-1.69
GO:00068	exocytosis	Biological	0.86	0.93	Solyc06g0	Solyc06g0	0.16	-2.68
GO:00068	exocytosis	Biological	0.86	0.93	Solyc10g0	Solyc10g0	0.13	-2.96
GO:00053	carbohydra	Molecular	0.87	0.93	Solyc12g0	Solyc12g0	4.94	2.30
GO:00053	glucose tra	Molecular	0.87	0.93	Solyc12g0	Solyc12g0	4.94	2.30
GO:00057	mitochond	Cellular Co	0.87	0.93	Solyc07g0	Solyc07g0	3.93	1.98
GO:00153	peptide:pro	Molecular	0.87	0.93	Solyc07g0	Solyc07g0	2.63	1.40
GO:19046	peptide tra	Molecular	0.87	0.93	Solyc07g0	Solyc07g0	2.63	1.40
GO:00468	inositol ph	Biological	0.87	0.93	Solyc04g0	Solyc04g0	0.41	-1.30
GO:00483	leaf develo	Biological	0.87	0.93	Solyc09g0	Solyc09g0	8.04	3.01
GO:00037	mRNA bin	Molecular	0.87	0.94	Solyc12g0	Solyc12g0	2.04	1.03
GO:00037	mRNA bin	Molecular	0.87	0.94	Solyc01g0	Solyc01g0	34.25	5.10

GO:00037	mRNA bin	Molecular	0.87	0.94	Solyc04g0	Solyc04g0	2.74	1.46
GO:00037	mRNA bin	Molecular	0.87	0.94	Solyc05g0	Solyc05g0	2143.40	11.07
GO:00037	mRNA bin	Molecular	0.87	0.94	Solyc06g0	PSBS	2.99	1.58
GO:00037	mRNA bin	Molecular	0.87	0.94	Solyc04g0	Solyc04g0	2.47	1.30
GO:00037	mRNA bin	Molecular	0.87	0.94	Solyc06g0	Solyc06g0	659.59	9.37
GO:00086	hexose tra	Biological	0.87	0.94	Solyc12g0	Solyc12g0	4.94	2.30
GO:00151	carbohydra	Molecular	0.87	0.94	Solyc12g0	Solyc12g0	4.94	2.30
GO:00431	cation binc	Molecular	0.88	0.94	Solyc03g0	Solyc03g0	2.27	1.18
GO:00431	cation binc	Molecular	0.88	0.94	Solyc04g0	Solyc04g0	1564.01	10.61
GO:00356	oligopeptic	Molecular	0.88	0.94	Solyc03g0	Solyc03g0	0.28	-1.86
GO:00356	oligopeptic	Molecular	0.88	0.94	Solyc07g0	Solyc07g0	2.63	1.40
GO:00057	Golgi appa	Cellular Co	0.88	0.94	Solyc02g0	Solyc02g0	0.21	-2.23
GO:00057	Golgi appa	Cellular Co	0.88	0.94	Solyc10g0	Solyc10g0	2.15	1.10
GO:00057	Golgi appa	Cellular Co	0.88	0.94	Solyc12g0	Solyc12g0	5.32	2.41
GO:00057	Golgi appa	Cellular Co	0.88	0.94	Solyc09g0	Solyc09g0	2.71	1.44
GO:00057	Golgi appa	Cellular Co	0.88	0.94	Solyc12g0	Solyc12g0	3.10	1.63
GO:00057	Golgi appa	Cellular Co	0.88	0.94	Solyc01g0	Solyc01g0	2.42	1.27
GO:00057	Golgi appa	Cellular Co	0.88	0.94	Solyc12g0	Solyc12g0	3.03	1.60
GO:00057	Golgi appa	Cellular Co	0.88	0.94	Solyc05g0	Solyc05g0	2.29	1.20
GO:00057	Golgi appa	Cellular Co	0.88	0.94	Solyc09g0	Solyc09g0	0.49	-1.04
GO:00057	Golgi appa	Cellular Co	0.88	0.94	Solyc02g0	Solyc02g0	3.38	1.76
GO:00057	Golgi appa	Cellular Co	0.88	0.94	Solyc01g0	Solyc01g0	0.19	-2.40
GO:00057	Golgi appa	Cellular Co	0.88	0.94	Solyc09g0	Solyc09g0	0.47	-1.09
GO:00057	Golgi appa	Cellular Co	0.88	0.94	Solyc07g0	Solyc07g0	0.01	-7.50
GO:00170	structural c	Molecular	0.88	0.94	Solyc11g0	Solyc11g0	0.05	-4.30
GO:00150	proton tran	Molecular	0.88	0.94	Solyc10g0	Solyc10g0	0.03	-5.05
GO:00050	guanyl-nuc	Molecular	0.88	0.94	Solyc02g0	Solyc02g0	2.47	1.31
GO:00168	ATP hydrc	Molecular	0.89	0.95	Solyc09g0	Solyc09g0	0.05	-4.40
GO:00168	ATP hydrc	Molecular	0.89	0.95	Solyc06g0	Solyc06g0	0.43	-1.22
GO:00168	ATP hydrc	Molecular	0.89	0.95	Solyc05g0	Solyc05g0	0.19	-2.39
GO:00168	ATP hydrc	Molecular	0.89	0.95	Solyc01g0	Solyc01g0	0.30	-1.75
GO:00168	ATP hydrc	Molecular	0.89	0.95	Solyc06g0	Solyc06g0	2.09	1.07
GO:00168	ATP hydrc	Molecular	0.89	0.95	Solyc06g0	Solyc06g0	2513.21	11.30
GO:00168	ATP hydrc	Molecular	0.89	0.95	Solyc04g0	Solyc04g0	0.00	-11.98
GO:00168	ATP hydrc	Molecular	0.89	0.95	Solyc02g0	Solyc02g0	0.49	-1.02
GO:00168	ATP hydrc	Molecular	0.89	0.95	Solyc01g0	Solyc01g0	0.49	-1.04
GO:00168	ATP hydrc	Molecular	0.89	0.95	Solyc10g0	Solyc10g0	0.04	-4.70
GO:00168	ATP hydrc	Molecular	0.89	0.95	Solyc08g0	Solyc08g0	2.67	1.41
GO:00168	ATP hydrc	Molecular	0.89	0.95	Solyc12g0	Solyc12g0	2.58	1.37
GO:00168	ATP hydrc	Molecular	0.89	0.95	Solyc08g0	Solyc08g0	2.38	1.25
GO:00168	ATP hydrc	Molecular	0.89	0.95	Solyc10g0	Solyc10g0	0.00	-12.76
GO:00168	ATP hydrc	Molecular	0.89	0.95	Solyc09g0	Solyc09g0	0.07	-3.89
GO:00168	ATP hydrc	Molecular	0.89	0.95	Solyc04g0	Solyc04g0	0.06	-3.97
GO:00168	ATP hydrc	Molecular	0.89	0.95	Solyc09g0	Solyc09g0	0.35	-1.53
GO:00168	ATP hydrc	Molecular	0.89	0.95	Solyc12g0	Solyc12g0	4.99	2.32
GO:00430	ribosome t	Molecular	0.89	0.95	Solyc12g0	Solyc12g0	0.36	-1.46
GO:00056	nuclear po	Cellular Co	0.89	0.95	Solyc02g0	Solyc02g0	0.25	-2.02
GO:00098	plant-type	Biological	0.89	0.95	Solyc03g0	Solyc03g0	0.00	-11.24
GO:00316	ubiquitin p	Molecular	0.89	0.95	Solyc01g0	Solyc01g0	0.42	-1.24
GO:00316	ubiquitin p	Molecular	0.89	0.95	Solyc02g0	Solyc02g0	2.53	1.34
GO:00086	carbohydra	Biological	0.90	0.96	Solyc12g0	Solyc12g0	4.94	2.30

GO:00457	nutrient re	Molecular	0.90	0.96	Solyc11g0	Solyc11g0	7.96	2.99
GO:00322	methylatio	Biological	0.90	0.96	Solyc08g0	Solyc08g0	2.64	1.40
GO:00322	methylatio	Biological	0.90	0.96	Solyc09g0	Solyc09g0	529.32	9.05
GO:00063	RNA proc	Biological	0.91	0.96	Solyc11g0	Solyc11g0	0.41	-1.28
GO:00063	RNA proc	Biological	0.91	0.96	Solyc03g0	Solyc03g0	4.17	2.06
GO:00063	RNA proc	Biological	0.91	0.96	Solyc11g0	Solyc11g0	0.46	-1.13
GO:00063	RNA proc	Biological	0.91	0.96	Solyc07g0	Solyc07g0	0.46	-1.11
GO:00063	RNA proc	Biological	0.91	0.96	Solyc04g0	Solyc04g0	2.47	1.30
GO:00057	peroxisom	Cellular C	0.91	0.96	Solyc06g0	Solyc06g0	2.33	1.22
GO:00057	peroxisom	Cellular C	0.91	0.96	Solyc03g0	Solyc03g0	0.00	-11.43
GO:00513	cell divisic	Biological	0.91	0.96	Solyc10g0	Solyc10g0	2.14	1.10
GO:00167	transferase	Molecular	0.91	0.96	Solyc06g0	Solyc06g0	12.77	3.67
GO:00062	DNA repai	Biological	0.91	0.96	Solyc01g1	Solyc01g1	2.32	1.22
GO:00062	DNA repai	Biological	0.91	0.96	Solyc09g0	Solyc09g0	0.35	-1.51
GO:00062	DNA repai	Biological	0.91	0.96	Solyc11g0	Solyc11g0	6976.37	12.77
GO:00062	DNA repai	Biological	0.91	0.96	Solyc09g0	Solyc09g0	2.14	1.10
GO:00062	DNA repai	Biological	0.91	0.96	Solyc03g0	Solyc03g0	0.00	-11.07
GO:00062	DNA repai	Biological	0.91	0.96	Solyc09g0	Solyc09g0	2.32	1.21
GO:00062	DNA repai	Biological	0.91	0.96	Solyc09g0	Solyc09g0	2.29	1.20
GO:00062	DNA repai	Biological	0.91	0.96	Solyc01g0	Solyc01g0	312.22	8.29
GO:00062	DNA repai	Biological	0.91	0.96	Solyc02g0	Solyc02g0	0.00	-10.40
GO:00063	chromatin	Biological	0.91	0.96	Solyc07g0	Solyc07g0	2.20	1.14
GO:00041	ATP-depe	Molecular	0.91	0.96	Solyc09g0	Solyc09g0	0.12	-3.08
GO:00081	sulfotransf	Molecular	0.91	0.96	Solyc05g0	Solyc05g0	0.42	-1.24
GO:00150	protein-dis	Molecular	0.92	0.97	Solyc04g0	Solyc04g0	2.43	1.28
GO:00150	protein-dis	Molecular	0.92	0.97	Solyc03g1	Solyc03g1	2.16	1.11
GO:00150	protein-dis	Molecular	0.92	0.97	Solyc10g0	Solyc10g0	2.07	1.05
GO:00083	O-acyltran	Molecular	0.92	0.97	Solyc05g0	Solyc05g0	5.36	2.42
GO:00036	single-stra	Molecular	0.92	0.97	Solyc01g0	Solyc01g0	0.49	-1.04
GO:00166	CCAAT-b	Cellular C	0.92	0.97	Solyc06g0	Solyc06g0	0.00	-12.03
GO:00090	biosyntheti	Biological	0.92	0.97	Solyc05g0	Solyc05g0	0.06	-4.07
GO:00090	biosyntheti	Biological	0.92	0.97	Solyc09g0	Solyc09g0	0.06	-4.05
GO:00090	biosyntheti	Biological	0.92	0.97	Solyc06g0	Solyc06g0	0.41	-1.30
GO:00090	biosyntheti	Biological	0.92	0.97	Solyc05g0	Solyc05g0	2.44	1.28
GO:00090	biosyntheti	Biological	0.92	0.97	Solyc06g0	Solyc06g0	0.00	-9.45
GO:00063	DNA-temp	Biological	0.93	0.97	Solyc06g0	Solyc06g0	3.01	1.59
GO:00425	cell wall m	Biological	0.93	0.97	Solyc08g0	Solyc08g0	2.92	1.55
GO:00425	cell wall m	Biological	0.93	0.97	Solyc03g1	Solyc03g1	0.44	-1.20
GO:00425	cell wall m	Biological	0.93	0.97	Solyc10g0	Solyc10g0	2.16	1.11
GO:00002	microtubul	Biological	0.93	0.97	Solyc12g0	Solyc12g0	0.48	-1.07
GO:00056	spliceosom	Cellular C	0.93	0.97	Solyc06g0	Solyc06g0	0.46	-1.11
GO:00197	calcium-m	Biological	0.93	0.97	Solyc03g0	Solyc03g0	2638.78	11.37
GO:00905	RNA phos	Biological	0.93	0.97	Solyc05g0	Solyc05g0	20.96	4.39
GO:00226	cytosolic l	Cellular C	0.93	0.98	Solyc09g0	Solyc09g0	4.17	2.06
GO:00226	cytosolic l	Cellular C	0.93	0.98	Solyc05g0	Solyc05g0	0.09	-3.44
GO:00226	cytosolic l	Cellular C	0.93	0.98	Solyc09g0	Solyc09g0	2.84	1.51
GO:00226	cytosolic l	Cellular C	0.93	0.98	Solyc09g0	Solyc09g0	0.47	-1.10
GO:00226	cytosolic l	Cellular C	0.93	0.98	Solyc12g0	Solyc12g0	0.04	-4.72
GO:00068	retrograde	Biological	0.94	0.98	Solyc09g0	Solyc09g0	2.01	1.01
GO:00453	aspartyl es	Molecular	0.94	0.98	Solyc03g1	Solyc03g1	0.44	-1.20
GO:00180	histone-lys	Molecular	0.94	0.98	Solyc12g1	Solyc12g1	2.30	1.20

GO:00710	catalytic st	Cellular Co	0.94	0.98	Solyc06g0	Solyc06g0	0.46	-1.11
GO:00058	cytosol	Cellular Co	0.94	0.98	Solyc04g0	Solyc04g0	0.06	-4.01
GO:00058	cytosol	Cellular Co	0.94	0.98	Solyc01g1	Solyc01g1	5.46	2.45
GO:00058	cytosol	Cellular Co	0.94	0.98	Solyc04g0	Solyc04g0	2.70	1.43
GO:00058	cytosol	Cellular Co	0.94	0.98	Solyc05g0	Solyc05g0	2.80	1.49
GO:00058	cytosol	Cellular Co	0.94	0.98	Solyc01g1	Solyc01g1	4.98	2.32
GO:00058	cytosol	Cellular Co	0.94	0.98	Solyc03g1	Solyc03g1	2.44	1.28
GO:00058	cytosol	Cellular Co	0.94	0.98	Solyc12g0	Solyc12g0	9.07	3.18
GO:00058	cytosol	Cellular Co	0.94	0.98	Solyc11g0	Solyc11g0	0.50	-1.01
GO:00058	cytosol	Cellular Co	0.94	0.98	Solyc01g0	Solyc01g0	3.54	1.82
GO:00058	cytosol	Cellular Co	0.94	0.98	Solyc10g0	Solyc10g0	2.23	1.16
GO:00058	cytosol	Cellular Co	0.94	0.98	Solyc12g0	Solyc12g0	0.45	-1.15
GO:00058	cytosol	Cellular Co	0.94	0.98	Solyc11g0	Solyc11g0	2.07	1.05
GO:00058	cytosol	Cellular Co	0.94	0.98	Solyc04g0	Solyc04g0	0.43	-1.23
GO:00058	cytosol	Cellular Co	0.94	0.98	Solyc01g1	Solyc01g1	14.86	3.89
GO:00058	cytosol	Cellular Co	0.94	0.98	Solyc03g0	Solyc03g0	6.17	2.63
GO:00058	cytosol	Cellular Co	0.94	0.98	Solyc06g0	Solyc06g0	3.04	1.60
GO:00058	cytosol	Cellular Co	0.94	0.98	Solyc03g0	Solyc03g0	0.02	-5.67
GO:00058	cytosol	Cellular Co	0.94	0.98	Solyc05g0	Solyc05g0	2143.40	11.07
GO:00058	cytosol	Cellular Co	0.94	0.98	Solyc04g0	Solyc04g0	1711.14	10.74
GO:00058	cytosol	Cellular Co	0.94	0.98	Solyc09g0	Solyc09g0	8.04	3.01
GO:00058	cytosol	Cellular Co	0.94	0.98	Solyc02g0	Solyc02g0	2.66	1.41
GO:00058	cytosol	Cellular Co	0.94	0.98	Solyc09g0	Solyc09g0	25.41	4.67
GO:00058	cytosol	Cellular Co	0.94	0.98	Solyc05g0	Solyc05g0	2.69	1.43
GO:00058	cytosol	Cellular Co	0.94	0.98	Solyc12g0	Solyc12g0	3.17	1.66
GO:00058	cytosol	Cellular Co	0.94	0.98	Solyc03g0	Solyc03g0	0.39	-1.35
GO:00058	cytosol	Cellular Co	0.94	0.98	Solyc12g0	Solyc12g0	0.39	-1.35
GO:00058	cytosol	Cellular Co	0.94	0.98	Solyc11g0	Solyc11g0	45.49	5.51
GO:00058	cytosol	Cellular Co	0.94	0.98	Solyc06g0	Solyc06g0	0.38	-1.41
GO:00058	cytosol	Cellular Co	0.94	0.98	Solyc06g0	Solyc06g0	10.22	3.35
GO:00058	cytosol	Cellular Co	0.94	0.98	Solyc11g0	Solyc11g0	0.46	-1.12
GO:00305	pectinester	Molecular	0.94	0.98	Solyc08g0	Solyc08g0	2.92	1.55
GO:00305	pectinester	Molecular	0.94	0.98	Solyc04g0	Solyc04g0	2.29	1.19
GO:00305	pectinester	Molecular	0.94	0.98	Solyc03g1	Solyc03g1	0.44	-1.20
GO:00311	SCF-deper	Biological	0.94	0.98	Solyc07g0	Solyc07g0	2.29	1.20
GO:00064	translation	Biological	0.94	0.98	Solyc12g0	Solyc12g0	2.35	1.23
GO:00042	serine-type	Molecular	0.94	0.98	Solyc01g1	Solyc01g1	0.44	-1.18
GO:00042	serine-type	Molecular	0.94	0.98	Solyc07g0	Solyc07g0	2.17	1.12
GO:00042	serine-type	Molecular	0.94	0.98	Solyc09g0	Solyc09g0	0.12	-3.08
GO:00042	serine-type	Molecular	0.94	0.98	Solyc04g0	Solyc04g0	0.43	-1.23
GO:00042	serine-type	Molecular	0.94	0.98	Solyc09g0	Solyc09g0	3042	11.57
GO:00042	serine-type	Molecular	0.94	0.98	Solyc01g0	Solyc01g0	0.48	-1.06
GO:00009	P-body	Cellular Co	0.94	0.98	Solyc07g0	Solyc07g0	0.40	-1.31
GO:00071	cell surface	Biological	0.95	0.98	Solyc06g0	Solyc06g0	7.83	2.97
GO:00004	protein pe	Biological	0.95	0.98	Solyc09g0	Solyc09g0	4.54	2.18
GO:00459	positive re	Biological	0.95	0.98	Solyc06g0	Solyc06g0	7.71	2.95
GO:00459	positive re	Biological	0.95	0.98	Solyc11g0	Solyc11g0	0.22	-2.21
GO:00459	positive re	Biological	0.95	0.98	Solyc11g0	Solyc11g0	2.29	1.19
GO:00459	positive re	Biological	0.95	0.98	Solyc11g0	Solyc11g0	0.47	-1.09
GO:00001	phosphorel	Biological	0.95	0.98	Solyc01g0	Solyc01g0	2.26	1.17
GO:00001	phosphorel	Biological	0.95	0.98	Solyc10g0	Solyc10g0	0.50	-1.01

GO:00084	transamina	Molecular	0.95	0.98	Solyc04g0	Solyc04g0	3.78	1.92
GO:00057	mitochond	Cellular C	0.95	0.99	Solyc05g0	Solyc05g0	19.89	4.31
GO:00057	mitochond	Cellular C	0.95	0.99	Solyc04g0	Solyc04g0	2.70	1.43
GO:00057	mitochond	Cellular C	0.95	0.99	Solyc01g0	Solyc01g0	2.46	1.30
GO:00057	mitochond	Cellular C	0.95	0.99	Solyc03g1	Solyc03g1	2.22	1.15
GO:00057	mitochond	Cellular C	0.95	0.99	Solyc01g0	Solyc01g0	0.32	-1.65
GO:00057	mitochond	Cellular C	0.95	0.99	Solyc06g0	Solyc06g0	0.41	-1.30
GO:00057	mitochond	Cellular C	0.95	0.99	Solyc06g0	Solyc06g0	2.01	1.01
GO:00057	mitochond	Cellular C	0.95	0.99	Solyc02g0	Solyc02g0	2.62	1.39
GO:00057	mitochond	Cellular C	0.95	0.99	Solyc07g0	PDF1A	4.20	2.07
GO:00057	mitochond	Cellular C	0.95	0.99	Solyc10g0	Solyc10g0	2.18	1.13
GO:00057	mitochond	Cellular C	0.95	0.99	Solyc02g0	Solyc02g0	3.06	1.62
GO:00057	mitochond	Cellular C	0.95	0.99	Solyc01g0	Solyc01g0	0.42	-1.26
GO:00057	mitochond	Cellular C	0.95	0.99	Solyc01g0	Solyc01g0	2.02	1.02
GO:00057	mitochond	Cellular C	0.95	0.99	Solyc04g0	Solyc04g0	39.10	5.29
GO:00057	mitochond	Cellular C	0.95	0.99	Solyc02g0	Solyc02g0	0.19	-2.37
GO:00057	mitochond	Cellular C	0.95	0.99	Solyc08g0	Solyc08g0	8.58	3.10
GO:00057	mitochond	Cellular C	0.95	0.99	Solyc08g0	Solyc08g0	7.54	2.91
GO:00057	mitochond	Cellular C	0.95	0.99	Solyc01g0	Solyc01g0	550.66	9.11
GO:00057	mitochond	Cellular C	0.95	0.99	Solyc11g0	Solyc11g0	0.22	-2.19
GO:00057	mitochond	Cellular C	0.95	0.99	Solyc04g0	Solyc04g0	32.87	5.04
GO:00057	mitochond	Cellular C	0.95	0.99	Solyc11g0	Solyc11g0	0.09	-3.52
GO:00057	mitochond	Cellular C	0.95	0.99	Solyc08g0	Solyc08g0	4.19	2.07
GO:00458	negative re	Biological	0.96	0.99	Solyc11g0	Solyc11g0	0.22	-2.21
GO:00458	negative re	Biological	0.96	0.99	Solyc10g0	Solyc10g0	0.10	-3.35
GO:00458	negative re	Biological	0.96	0.99	Solyc03g0	Solyc03g0	0.11	-3.20
GO:00469	protein din	Molecular	0.96	0.99	Solyc06g0	Solyc06g0	7.71	2.95
GO:00469	protein din	Molecular	0.96	0.99	Solyc03g0	Solyc03g0	2.53	1.34
GO:00469	protein din	Molecular	0.96	0.99	Solyc09g0	Solyc09g0	0.25	-2.02
GO:00469	protein din	Molecular	0.96	0.99	Solyc06g0	Solyc06g0	2.05	1.03
GO:00469	protein din	Molecular	0.96	0.99	Solyc08g0	Solyc08g0	0.47	-1.08
GO:00469	protein din	Molecular	0.96	0.99	Solyc10g0	Solyc10g0	0.40	-1.34
GO:00469	protein din	Molecular	0.96	0.99	Solyc11g0	Solyc11g0	2.29	1.19
GO:00469	protein din	Molecular	0.96	0.99	Solyc08g0	Solyc08g0	0.49	-1.02
GO:00469	protein din	Molecular	0.96	0.99	Solyc11g0	Solyc11g0	0.50	-1.01
GO:00469	protein din	Molecular	0.96	0.99	Solyc11g0	Solyc11g0	17863.20	14.12
GO:00469	protein din	Molecular	0.96	0.99	Solyc10g0	Solyc10g0	2.14	1.10
GO:00469	protein din	Molecular	0.96	0.99	Solyc11g0	Solyc11g0	0.50	-1.00
GO:00469	protein din	Molecular	0.96	0.99	Solyc08g0	Solyc08g0	0.18	-2.47
GO:00469	protein din	Molecular	0.96	0.99	Solyc10g0	Solyc10g0	0.33	-1.59
GO:00469	protein din	Molecular	0.96	0.99	Solyc03g1	Solyc03g1	0.44	-1.20
GO:00469	protein din	Molecular	0.96	0.99	Solyc12g0	Solyc12g0	5.33	2.42
GO:00469	protein din	Molecular	0.96	0.99	Solyc03g0	Solyc03g0	0.39	-1.35
GO:00469	protein din	Molecular	0.96	0.99	Solyc01g0	Solyc01g0	0.32	-1.65
GO:00469	protein din	Molecular	0.96	0.99	Solyc03g0	Solyc03g0	3218.95	11.65
GO:00469	protein din	Molecular	0.96	0.99	Solyc04g0	Solyc04g0	1227.78	10.26
GO:00469	protein din	Molecular	0.96	0.99	Solyc01g0	Solyc01g0	626.38	9.29
GO:00469	protein din	Molecular	0.96	0.99	Solyc02g0	Solyc02g0	34.22	5.10
GO:00469	protein din	Molecular	0.96	0.99	Solyc11g0	Solyc11g0	116.23	6.86
GO:00469	protein din	Molecular	0.96	0.99	Solyc11g0	Solyc11g0	0.47	-1.09
GO:00469	protein din	Molecular	0.96	0.99	Solyc01g0	Solyc01g0	0.10	-3.31

GO:00469	protein din	Molecular	0.96	0.99	Solyc05g0	Solyc05g0	6.61	2.73
GO:00469	protein din	Molecular	0.96	0.99	Solyc05g0	Solyc05g0	0.00	-10.39
GO:00485	meristem c	Biological	0.96	0.99	Solyc07g0	Solyc07g0	0.00	-11.47
GO:00485	meristem c	Biological	0.96	0.99	Solyc07g0	Solyc07g0	0.00	-11.40
GO:00485	meristem c	Biological	0.96	0.99	Solyc03g0	Solyc03g0	4.97	2.31
GO:00002	protein pol	Biological	0.96	0.99	Solyc03g1	Solyc03g1	0.35	-1.54
GO:00100	meristem r	Biological	0.97	0.99	Solyc07g0	Solyc07g0	0.00	-11.47
GO:00100	meristem r	Biological	0.97	0.99	Solyc07g0	Solyc07g0	0.00	-11.40
GO:00100	meristem r	Biological	0.97	0.99	Solyc03g0	Solyc03g0	4.97	2.31
GO:00103	terpene syı	Molecular	0.97	0.99	Solyc08g0	Solyc08g0	8.90	3.15
GO:00082	zinc ion bi	Molecular	0.97	0.99	Solyc04g0	Solyc04g0	0.09	-3.54
GO:00082	zinc ion bi	Molecular	0.97	0.99	Solyc09g0	Solyc09g0	6.99	2.80
GO:00082	zinc ion bi	Molecular	0.97	0.99	Solyc04g0	Solyc04g0	2.70	1.43
GO:00082	zinc ion bi	Molecular	0.97	0.99	Solyc04g0	Solyc04g0	3.53	1.82
GO:00082	zinc ion bi	Molecular	0.97	0.99	Solyc02g0	Solyc02g0	11.09	3.47
GO:00082	zinc ion bi	Molecular	0.97	0.99	Solyc09g0	Solyc09g0	0.18	-2.45
GO:00082	zinc ion bi	Molecular	0.97	0.99	Solyc05g0	Solyc05g0	0.48	-1.05
GO:00082	zinc ion bi	Molecular	0.97	0.99	Solyc09g0	Solyc09g0	0.44	-1.18
GO:00082	zinc ion bi	Molecular	0.97	0.99	Solyc05g0	Solyc05g0	2.11	1.08
GO:00082	zinc ion bi	Molecular	0.97	0.99	Solyc06g0	Solyc06g0	0.46	-1.11
GO:00082	zinc ion bi	Molecular	0.97	0.99	Solyc06g0	Solyc06g0	0.14	-2.80
GO:00082	zinc ion bi	Molecular	0.97	0.99	Solyc04g0	Solyc04g0	0.00	-13.91
GO:00082	zinc ion bi	Molecular	0.97	0.99	Solyc02g0	Solyc02g0	0.00	-11.89
GO:00082	zinc ion bi	Molecular	0.97	0.99	Solyc08g0	Solyc08g0	0.00	-12.86
GO:00082	zinc ion bi	Molecular	0.97	0.99	Solyc02g0	Solyc02g0	0.31	-1.68
GO:00082	zinc ion bi	Molecular	0.97	0.99	Solyc04g0	Solyc04g0	2.35	1.23
GO:00082	zinc ion bi	Molecular	0.97	0.99	Solyc03g1	Solyc03g1	0.41	-1.30
GO:00082	zinc ion bi	Molecular	0.97	0.99	Solyc01g0	Solyc01g0	2.35	1.24
GO:00082	zinc ion bi	Molecular	0.97	0.99	Solyc08g0	Solyc08g0	0.03	-5.23
GO:00082	zinc ion bi	Molecular	0.97	0.99	Solyc02g0	Solyc02g0	0.29	-1.80
GO:00082	zinc ion bi	Molecular	0.97	0.99	Solyc05g0	Solyc05g0	3.62	1.86
GO:00082	zinc ion bi	Molecular	0.97	0.99	Solyc07g0	Solyc07g0	2.02	1.02
GO:00082	zinc ion bi	Molecular	0.97	0.99	Solyc03g0	Solyc03g0	20.56	4.36
GO:00082	zinc ion bi	Molecular	0.97	0.99	Solyc02g0	Solyc02g0	0.07	-3.82
GO:00082	zinc ion bi	Molecular	0.97	0.99	Solyc03g0	Solyc03g0	794.30	9.63
GO:00082	zinc ion bi	Molecular	0.97	0.99	Solyc04g0	Solyc04g0	15.69	3.97
GO:00082	zinc ion bi	Molecular	0.97	0.99	Solyc01g0	Solyc01g0	0.12	-3.03
GO:00082	zinc ion bi	Molecular	0.97	0.99	Solyc03g0	Solyc03g0	0.44	-1.17
GO:00082	zinc ion bi	Molecular	0.97	0.99	Solyc02g0	Solyc02g0	1446.12	10.50
GO:00082	zinc ion bi	Molecular	0.97	0.99	Solyc02g0	Solyc02g0	0.50	-1.00
GO:00082	zinc ion bi	Molecular	0.97	0.99	Solyc11g0	Solyc11g0	5.06	2.34
GO:00082	zinc ion bi	Molecular	0.97	0.99	Solyc08g0	Solyc08g0	5.12	2.36
GO:00082	zinc ion bi	Molecular	0.97	0.99	Solyc06g0	Solyc06g0	0.35	-1.52
GO:00082	zinc ion bi	Molecular	0.97	0.99	Solyc02g0	Solyc02g0	394.05	8.62
GO:00082	zinc ion bi	Molecular	0.97	0.99	Solyc11g0	Solyc11g0	281.82	8.14
GO:00082	zinc ion bi	Molecular	0.97	0.99	Solyc12g0	Solyc12g0	2361.68	11.21
GO:00039	GTPase ac	Molecular	0.97	0.99	Solyc11g0	Solyc11g0	0.10	-3.26
GO:00039	GTPase ac	Molecular	0.97	0.99	Solyc10g0	Solyc10g0	2.15	1.10
GO:00039	GTPase ac	Molecular	0.97	0.99	Solyc01g1	Solyc01g1	0.44	-1.20
GO:00039	GTPase ac	Molecular	0.97	0.99	Solyc02g0	Solyc02g0	6.74	2.75
GO:00039	GTPase ac	Molecular	0.97	0.99	Solyc06g0	Solyc06g0	2.20	1.14

GO:00039	GTPase ac	Molecular	0.97	0.99	Solyc08g0	Solyc08g0	0.49	-1.03
GO:00039	GTPase ac	Molecular	0.97	0.99	Solyc03g1	Solyc03g1	0.00	-9.23
GO:00039	GTPase ac	Molecular	0.97	0.99	Solyc09g0	Solyc09g0	0.44	-1.20
GO:00039	GTPase ac	Molecular	0.97	0.99	Solyc01g0	Solyc01g0	312.22	8.29
GO:19909	ribonucleo	Cellular C	0.97	0.99	Solyc05g0	Solyc05g0	2143.40	11.07
GO:00801	gene silenc	Biological	0.98	1.00	Solyc01g0	Solyc01g0	2.62	1.39
GO:00065	cellular am	Biological	0.98	1.00	Solyc03g0	Solyc03g0	0.44	-1.19
GO:00165	protein deu	Biological	0.98	1.00	Solyc12g0	Solyc12g0	0.39	-1.35
GO:00165	protein ubi	Biological	0.98	1.00	Solyc07g0	Solyc07g0	2.29	1.20
GO:00165	protein ubi	Biological	0.98	1.00	Solyc03g1	Solyc03g1	0.35	-1.54
GO:00165	protein ubi	Biological	0.98	1.00	Solyc08g0	Solyc08g0	0.29	-1.78
GO:00165	protein ubi	Biological	0.98	1.00	Solyc03g0	Solyc03g0	2.92	1.54
GO:00165	protein ubi	Biological	0.98	1.00	Solyc05g0	Solyc05g0	2.24	1.16
GO:00165	protein ubi	Biological	0.98	1.00	Solyc03g0	Solyc03g0	0.34	-1.54
GO:00165	protein ubi	Biological	0.98	1.00	Solyc02g0	Solyc02g0	0.18	-2.48
GO:00165	protein ubi	Biological	0.98	1.00	Solyc11g0	Solyc11g0	0.24	-2.07
GO:00165	protein ubi	Biological	0.98	1.00	Solyc02g0	Solyc02g0	2.53	1.34
GO:00165	protein ubi	Biological	0.98	1.00	Solyc11g0	Solyc11g0	0.46	-1.12
GO:00048	thiol-deper	Molecular	0.98	1.00	Solyc12g0	Solyc12g0	0.39	-1.35
GO:00975	glutathione	Molecular	0.98	1.00	Solyc08g0	Solyc08g0	2.43	1.28
GO:00042	metalloenc	Molecular	0.98	1.00	Solyc05g0	Solyc05g0	1370.75	10.42
GO:00096	chloroplas	Biological	0.98	1.00	Solyc01g1	Solyc01g1	0.37	-1.43
GO:00058	ribosome	Cellular C	0.98	1.00	Solyc11g0	Solyc11g0	2.27	1.18
GO:00058	ribosome	Cellular C	0.98	1.00	Solyc03g0	Solyc03g0	2.43	1.28
GO:00058	ribosome	Cellular C	0.98	1.00	Solyc06g0	Solyc06g0	0.29	-1.81
GO:00058	ribosome	Cellular C	0.98	1.00	Solyc05g0	Solyc05g0	0.09	-3.44
GO:00058	ribosome	Cellular C	0.98	1.00	Solyc10g0	Solyc10g0	2.18	1.13
GO:00058	ribosome	Cellular C	0.98	1.00	Solyc10g0	Solyc10g0	0.16	-2.66
GO:00058	ribosome	Cellular C	0.98	1.00	Solyc07g0	Solyc07g0	2.00	1.00
GO:00058	ribosome	Cellular C	0.98	1.00	Solyc09g0	rpl33	0.05	-4.19
GO:00058	ribosome	Cellular C	0.98	1.00	Solyc09g0	Solyc09g0	2.84	1.51
GO:00058	ribosome	Cellular C	0.98	1.00	Solyc05g0	Solyc05g0	2.32	1.21
GO:00058	ribosome	Cellular C	0.98	1.00	Solyc09g0	Solyc09g0	0.47	-1.10
GO:00058	ribosome	Cellular C	0.98	1.00	Solyc11g0	Solyc11g0	0.00	-13.41
GO:00058	ribosome	Cellular C	0.98	1.00	Solyc12g0	Solyc12g0	0.08	-3.72
GO:00058	ribosome	Cellular C	0.98	1.00	Solyc02g0	Solyc02g0	7.75	2.95
GO:00058	ribosome	Cellular C	0.98	1.00	Solyc05g0	Solyc05g0	10.06	3.33
GO:00058	ribosome	Cellular C	0.98	1.00	Solyc12g0	Solyc12g0	0.04	-4.72
GO:00058	ribosome	Cellular C	0.98	1.00	Solyc12g0	Solyc12g0	0.16	-2.65
GO:00058	ribosome	Cellular C	0.98	1.00	Solyc01g0	Solyc01g0	0.08	-3.59
GO:00058	ribosome	Cellular C	0.98	1.00	Solyc09g0	Solyc09g0	0.25	-2.00
GO:00050	GTPase ac	Molecular	0.98	1.00	Solyc07g0	Solyc07g0	2.26	1.18
GO:00510	unfolded p	Molecular	0.98	1.00	Solyc10g0	Solyc10g0	0.09	-3.53
GO:00510	unfolded p	Molecular	0.98	1.00	Solyc06g0	Solyc06g0	2.09	1.07
GO:00510	unfolded p	Molecular	0.98	1.00	Solyc02g0	Solyc02g0	2.46	1.30
GO:00063	regulation	Biological	0.98	1.00	Solyc11g0	Solyc11g0	2.29	1.19
GO:00063	regulation	Biological	0.98	1.00	Solyc10g0	Solyc10g0	0.33	-1.59
GO:00063	regulation	Biological	0.98	1.00	Solyc04g0	Solyc04g0	0.07	-3.79
GO:00063	regulation	Biological	0.98	1.00	Solyc04g0	Solyc04g0	1227.78	10.26
GO:00063	regulation	Biological	0.98	1.00	Solyc01g0	Solyc01g0	0.48	-1.06
GO:00068	endoplasm	Biological	0.99	1.00	Solyc04g0	Solyc04g0	5759.74	12.49

GO:00068	endoplasm	Biological	0.99	1.00	Solyc01g0	Solyc01g0	2335.77	11.19
GO:00064	translation	Biological	0.99	1.00	Solyc11g0	Solyc11g0	0.10	-3.26
GO:00064	translation	Biological	0.99	1.00	Solyc11g0	Solyc11g0	2.27	1.18
GO:00064	translation	Biological	0.99	1.00	Solyc03g0	Solyc03g0	2.43	1.28
GO:00064	translation	Biological	0.99	1.00	Solyc06g0	Solyc06g0	0.29	-1.81
GO:00064	translation	Biological	0.99	1.00	Solyc05g0	Solyc05g0	0.09	-3.44
GO:00064	translation	Biological	0.99	1.00	Solyc04g0	Solyc04g0	2.32	1.22
GO:00064	translation	Biological	0.99	1.00	Solyc07g0	PDF1A	4.20	2.07
GO:00064	translation	Biological	0.99	1.00	Solyc10g0	Solyc10g0	2.18	1.13
GO:00064	translation	Biological	0.99	1.00	Solyc10g0	Solyc10g0	0.16	-2.66
GO:00064	translation	Biological	0.99	1.00	Solyc07g0	Solyc07g0	2.00	1.00
GO:00064	translation	Biological	0.99	1.00	Solyc09g0	rpl33	0.05	-4.19
GO:00064	translation	Biological	0.99	1.00	Solyc09g0	Solyc09g0	2.84	1.51
GO:00064	translation	Biological	0.99	1.00	Solyc09g0	Solyc09g0	0.47	-1.10
GO:00064	translation	Biological	0.99	1.00	Solyc11g0	Solyc11g0	0.00	-13.41
GO:00064	translation	Biological	0.99	1.00	Solyc12g0	Solyc12g0	0.08	-3.72
GO:00064	translation	Biological	0.99	1.00	Solyc02g0	Solyc02g0	7.75	2.95
GO:00064	translation	Biological	0.99	1.00	Solyc05g0	Solyc05g0	10.06	3.33
GO:00064	translation	Biological	0.99	1.00	Solyc12g0	Solyc12g0	0.04	-4.72
GO:00064	translation	Biological	0.99	1.00	Solyc12g0	Solyc12g0	0.16	-2.65
GO:00064	translation	Biological	0.99	1.00	Solyc01g0	Solyc01g0	0.08	-3.59
GO:00064	translation	Biological	0.99	1.00	Solyc09g0	Solyc09g0	0.25	-2.00
GO:00001	Golgi mem	Cellular C	0.99	1.00	Solyc03g1	Solyc03g1	0.33	-1.60
GO:00001	Golgi mem	Cellular C	0.99	1.00	Solyc01g0	Solyc01g0	2335.77	11.19
GO:00055	GTP bindi	Molecular	0.99	1.00	Solyc11g0	Solyc11g0	0.10	-3.26
GO:00055	GTP bindi	Molecular	0.99	1.00	Solyc10g0	Solyc10g0	2.15	1.10
GO:00055	GTP bindi	Molecular	0.99	1.00	Solyc01g1	Solyc01g1	0.44	-1.20
GO:00055	GTP bindi	Molecular	0.99	1.00	Solyc02g0	Solyc02g0	6.74	2.75
GO:00055	GTP bindi	Molecular	0.99	1.00	Solyc06g0	Solyc06g0	2.20	1.14
GO:00055	GTP bindi	Molecular	0.99	1.00	Solyc08g0	Solyc08g0	0.49	-1.03
GO:00055	GTP bindi	Molecular	0.99	1.00	Solyc03g1	Solyc03g1	0.00	-9.23
GO:00055	GTP bindi	Molecular	0.99	1.00	Solyc06g0	Solyc06g0	2266.68	11.15
GO:00055	GTP bindi	Molecular	0.99	1.00	Solyc09g0	Solyc09g0	0.44	-1.20
GO:00055	GTP bindi	Molecular	0.99	1.00	Solyc01g0	Solyc01g0	312.22	8.29
GO:00226	cytosolic s	Cellular C	0.99	1.00	Solyc01g0	Solyc01g0	0.08	-3.59
GO:00037	translation	Molecular	0.99	1.00	Solyc01g1	Solyc01g1	6.83	2.77
GO:00064	protein de	Biological	0.99	1.00	Solyc12g0	Solyc12g0	2.88	1.52
GO:00037	structural	Molecular	0.99	1.00	Solyc11g0	Solyc11g0	2.27	1.18
GO:00037	structural	Molecular	0.99	1.00	Solyc03g0	Solyc03g0	2.43	1.28
GO:00037	structural	Molecular	0.99	1.00	Solyc09g0	Solyc09g0	4.17	2.06
GO:00037	structural	Molecular	0.99	1.00	Solyc06g0	Solyc06g0	0.29	-1.81
GO:00037	structural	Molecular	0.99	1.00	Solyc05g0	Solyc05g0	0.09	-3.44
GO:00037	structural	Molecular	0.99	1.00	Solyc04g0	Solyc04g0	2.32	1.22
GO:00037	structural	Molecular	0.99	1.00	Solyc10g0	Solyc10g0	2.18	1.13
GO:00037	structural	Molecular	0.99	1.00	Solyc10g0	Solyc10g0	0.16	-2.66
GO:00037	structural	Molecular	0.99	1.00	Solyc07g0	Solyc07g0	2.00	1.00
GO:00037	structural	Molecular	0.99	1.00	Solyc09g0	rpl33	0.05	-4.19
GO:00037	structural	Molecular	0.99	1.00	Solyc09g0	Solyc09g0	2.84	1.51
GO:00037	structural	Molecular	0.99	1.00	Solyc09g0	Solyc09g0	0.47	-1.10
GO:00037	structural	Molecular	0.99	1.00	Solyc11g0	Solyc11g0	0.00	-13.41
GO:00037	structural	Molecular	0.99	1.00	Solyc12g0	Solyc12g0	0.08	-3.72

GO:00037	structural c	Molecular	0.99	1.00	Solyc02g0	Solyc02g0	7.75	2.95
GO:00037	structural c	Molecular	0.99	1.00	Solyc05g0	Solyc05g0	10.06	3.33
GO:00037	structural c	Molecular	0.99	1.00	Solyc12g0	Solyc12g0	0.04	-4.72
GO:00037	structural c	Molecular	0.99	1.00	Solyc12g0	Solyc12g0	0.16	-2.65
GO:00037	structural c	Molecular	0.99	1.00	Solyc01g0	Solyc01g0	0.08	-3.59
GO:00037	structural c	Molecular	0.99	1.00	Solyc09g0	Solyc09g0	0.25	-2.00
GO:00037	peptidyl-pı	Molecular	0.99	1.00	Solyc09g0	Solyc09g0	4.54	2.18
GO:00064	protein gly	Biological	0.99	1.00	Solyc09g0	Solyc09g0	7.65	2.93
GO:00064	protein gly	Biological	0.99	1.00	Solyc08g0	Solyc08g0	2.92	1.55
GO:00047	protein ser	Molecular	0.99	1.00	Solyc12g0	Solyc12g0	2.88	1.52
GO:00065	ubiquitin-c	Biological	1.00	1.00	Solyc07g0	Solyc07g0	2.29	1.20
GO:00065	ubiquitin-c	Biological	1.00	1.00	Solyc03g1	Solyc03g1	0.35	-1.54
GO:00065	ubiquitin-c	Biological	1.00	1.00	Solyc07g0	Solyc07g0	0.41	-1.27
GO:00065	ubiquitin-c	Biological	1.00	1.00	Solyc03g0	Solyc03g0	794.30	9.63
GO:00065	ubiquitin-c	Biological	1.00	1.00	Solyc03g0	Solyc03g0	0.44	-1.17
GO:00065	ubiquitin-c	Biological	1.00	1.00	Solyc01g0	Solyc01g0	0.42	-1.24
GO:00065	ubiquitin-c	Biological	1.00	1.00	Solyc01g1	Solyc01g1	0.00	-9.07
GO:00616	ubiquitin p	Molecular	1.00	1.00	Solyc03g1	Solyc03g1	2.10	1.07
GO:00616	ubiquitin p	Molecular	1.00	1.00	Solyc03g1	Solyc03g1	0.35	-1.54
GO:00616	ubiquitin p	Molecular	1.00	1.00	Solyc05g0	Solyc05g0	2.24	1.16
GO:00616	ubiquitin p	Molecular	1.00	1.00	Solyc07g0	Solyc07g0	0.41	-1.27
GO:00048	ubiquitin-p	Molecular	1.00	1.00	Solyc03g1	Solyc03g1	0.35	-1.54
GO:00048	ubiquitin-p	Molecular	1.00	1.00	Solyc05g0	Solyc05g0	0.48	-1.05
GO:00048	ubiquitin-p	Molecular	1.00	1.00	Solyc03g0	Solyc03g0	2.92	1.54
GO:00048	ubiquitin-p	Molecular	1.00	1.00	Solyc03g0	Solyc03g0	0.34	-1.54
GO:00057	nucleolus	Cellular Co	1.00	1.00	Solyc01g0	Solyc01g0	3.72	1.90
GO:00068	intracellula	Biological	1.00	1.00	Solyc10g0	Solyc10g0	2.15	1.10
GO:00068	intracellula	Biological	1.00	1.00	Solyc06g0	Solyc06g0	16.51	4.04
GO:00068	intracellula	Biological	1.00	1.00	Solyc06g0	Solyc06g0	2.20	1.14
GO:00068	intracellula	Biological	1.00	1.00	Solyc04g0	Solyc04g0	5759.74	12.49
GO:00068	intracellula	Biological	1.00	1.00	Solyc09g0	Solyc09g0	0.44	-1.20
GO:00068	intracellula	Biological	1.00	1.00	Solyc02g0	Solyc02g0	107563.96	16.71
GO:00064	protein fol	Biological	1.00	1.00	Solyc10g0	Solyc10g0	0.09	-3.53
GO:00064	protein fol	Biological	1.00	1.00	Solyc06g0	Solyc06g0	2.09	1.07
GO:00064	protein fol	Biological	1.00	1.00	Solyc02g0	Solyc02g0	2.46	1.30
GO:00057	endoplasm	Cellular Co	1.00	1.00	Solyc02g0	Solyc02g0	0.50	-1.01
GO:00057	endoplasm	Cellular Co	1.00	1.00	Solyc09g0	Solyc09g0	0.41	-1.27
GO:00057	endoplasm	Cellular Co	1.00	1.00	Solyc09g0	Solyc09g0	3.41	1.77
GO:00057	endoplasm	Cellular Co	1.00	1.00	Solyc01g0	Solyc01g0	0.49	-1.04
GO:00057	endoplasm	Cellular Co	1.00	1.00	Solyc03g1	Solyc03g1	0.33	-1.59
GO:00057	endoplasm	Cellular Co	1.00	1.00	Solyc05g0	Solyc05g0	2.29	1.20
GO:00057	endoplasm	Cellular Co	1.00	1.00	Solyc04g0	Solyc04g0	5759.74	12.49
GO:00057	endoplasm	Cellular Co	1.00	1.00	Solyc10g0	Solyc10g0	0.37	-1.43
GO:00003	mRNA spl	Biological	1.00	1.00	Solyc06g0	Solyc06g0	0.46	-1.11
GO:00161	vesicle-me	Biological	1.00	1.00	Solyc06g0	Solyc06g0	16.51	4.04
GO:00161	vesicle-me	Biological	1.00	1.00	Solyc02g0	Solyc02g0	107563.96	16.71
GO:00036	nucleic aci	Molecular	1.00	1.00	Solyc06g0	Solyc06g0	9.47	3.24
GO:00036	nucleic aci	Molecular	1.00	1.00	Solyc09g0	Solyc09g0	2.34	1.23
GO:00036	nucleic aci	Molecular	1.00	1.00	Solyc09g0	Solyc09g0	0.18	-2.45
GO:00036	nucleic aci	Molecular	1.00	1.00	Solyc11g0	Solyc11g0	0.41	-1.28
GO:00036	nucleic aci	Molecular	1.00	1.00	Solyc05g0	Solyc05g0	2.32	1.21

GO:00036	nucleic aci	Molecular	1.00	1.00	Solyc11g0	Solyc11g0	4.62	2.21
GO:00036	nucleic aci	Molecular	1.00	1.00	Solyc09g0	Solyc09g0	6.17	2.63
GO:00036	nucleic aci	Molecular	1.00	1.00	Solyc02g0	Solyc02g0	0.49	-1.04
GO:00036	nucleic aci	Molecular	1.00	1.00	Solyc11g0	Solyc11g0	0.47	-1.10
GO:00036	nucleic aci	Molecular	1.00	1.00	Solyc01g1	Solyc01g1	2.32	1.22
GO:00036	nucleic aci	Molecular	1.00	1.00	Solyc06g0	Solyc06g0	0.40	-1.33
GO:00036	nucleic aci	Molecular	1.00	1.00	Solyc10g0	Solyc10g0	10.66	3.41
GO:00036	nucleic aci	Molecular	1.00	1.00	Solyc09g0	Solyc09g0	0.45	-1.15
GO:00036	nucleic aci	Molecular	1.00	1.00	Solyc06g0	Solyc06g0	0.31	-1.69
GO:00036	nucleic aci	Molecular	1.00	1.00	Solyc06g0	Solyc06g0	0.14	-2.80
GO:00036	nucleic aci	Molecular	1.00	1.00	Solyc01g1	Solyc01g1	2.98	1.58
GO:00036	nucleic aci	Molecular	1.00	1.00	Solyc09g0	Solyc09g0	0.32	-1.62
GO:00036	nucleic aci	Molecular	1.00	1.00	Solyc06g0	Solyc06g0	2.54	1.35
GO:00036	nucleic aci	Molecular	1.00	1.00	Solyc06g0	Solyc06g0	2.07	1.05
GO:00036	nucleic aci	Molecular	1.00	1.00	Solyc10g0	Solyc10g0	2.20	1.14
GO:00036	nucleic aci	Molecular	1.00	1.00	Solyc02g0	Solyc02g0	0.31	-1.68
GO:00036	nucleic aci	Molecular	1.00	1.00	Solyc04g0	Solyc04g0	2.35	1.23
GO:00036	nucleic aci	Molecular	1.00	1.00	Solyc09g0	Solyc09g0	2.93	1.55
GO:00036	nucleic aci	Molecular	1.00	1.00	Solyc04g0	Solyc04g0	2.74	1.46
GO:00036	nucleic aci	Molecular	1.00	1.00	Solyc06g0	Solyc06g0	0.46	-1.11
GO:00036	nucleic aci	Molecular	1.00	1.00	Solyc05g0	Solyc05g0	2143.40	11.07
GO:00036	nucleic aci	Molecular	1.00	1.00	Solyc01g0	Solyc01g0	2.35	1.24
GO:00036	nucleic aci	Molecular	1.00	1.00	Solyc02g0	Solyc02g0	0.29	-1.80
GO:00036	nucleic aci	Molecular	1.00	1.00	Solyc05g0	Solyc05g0	3.62	1.86
GO:00036	nucleic aci	Molecular	1.00	1.00	Solyc11g0	Solyc11g0	1008.81	9.98
GO:00036	nucleic aci	Molecular	1.00	1.00	Solyc10g0	Solyc10g0	0.00	-10.24
GO:00036	nucleic aci	Molecular	1.00	1.00	Solyc08g0	Solyc08g0	3.18	1.67
GO:00036	nucleic aci	Molecular	1.00	1.00	Solyc05g0	Solyc05g0	3.77	1.92
GO:00036	nucleic aci	Molecular	1.00	1.00	Solyc06g0	Solyc06g0	0.35	-1.52
GO:00094	RNA modi	Biological	1.00	1.00	Solyc04g0	Solyc04g0	0.09	-3.54
GO:00055	protein bin	Molecular	1.00	1.00	Solyc04g0	Solyc04g0	0.09	-3.54
GO:00055	protein bin	Molecular	1.00	1.00	Solyc04g0	Solyc04g0	0.06	-4.01
GO:00055	protein bin	Molecular	1.00	1.00	Solyc09g0	Solyc09g0	0.05	-4.40
GO:00055	protein bin	Molecular	1.00	1.00	Solyc01g0	Solyc01g0	2.18	1.13
GO:00055	protein bin	Molecular	1.00	1.00	Solyc07g0	Solyc07g0	2.29	1.20
GO:00055	protein bin	Molecular	1.00	1.00	Solyc07g0	Solyc07g0	2.80	1.49
GO:00055	protein bin	Molecular	1.00	1.00	Solyc05g0	Solyc05g0	0.02	-5.33
GO:00055	protein bin	Molecular	1.00	1.00	Solyc03g0	Solyc03g0	2.38	1.25
GO:00055	protein bin	Molecular	1.00	1.00	Solyc01g0	Solyc01g0	0.31	-1.67
GO:00055	protein bin	Molecular	1.00	1.00	Solyc09g0	Solyc09g0	2.34	1.23
GO:00055	protein bin	Molecular	1.00	1.00	Solyc11g0	Solyc11g0	9.52	3.25
GO:00055	protein bin	Molecular	1.00	1.00	Solyc10g0	Solyc10g0	2.09	1.06
GO:00055	protein bin	Molecular	1.00	1.00	Solyc06g0	Solyc06g0	0.29	-1.78
GO:00055	protein bin	Molecular	1.00	1.00	Solyc09g0	Solyc09g0	4.03	2.01
GO:00055	protein bin	Molecular	1.00	1.00	Solyc01g1	Solyc01g1	0.29	-1.80
GO:00055	protein bin	Molecular	1.00	1.00	Solyc08g0	LOX1.1	2.60	1.38
GO:00055	protein bin	Molecular	1.00	1.00	Solyc02g0	Solyc02g0	3.63	1.86
GO:00055	protein bin	Molecular	1.00	1.00	Solyc09g0	Solyc09g0	2.37	1.24
GO:00055	protein bin	Molecular	1.00	1.00	Solyc11g0	Solyc11g0	0.41	-1.28
GO:00055	protein bin	Molecular	1.00	1.00	Solyc02g0	Solyc02g0	0.50	-1.01
GO:00055	protein bin	Molecular	1.00	1.00	Solyc11g0	Solyc11g0	20.23	4.34

GO:00055	protein bin	Molecular	1.00	1.00	Solyc07g0	Solyc07g0	2.04	1.03
GO:00055	protein bin	Molecular	1.00	1.00	Solyc02g0	Solyc02g0	0.47	-1.10
GO:00055	protein bin	Molecular	1.00	1.00	Solyc07g0	Solyc07g0	2.17	1.12
GO:00055	protein bin	Molecular	1.00	1.00	Solyc11g0	Solyc11g0	3.90	1.96
GO:00055	protein bin	Molecular	1.00	1.00	Solyc09g0	Solyc09g0	2.40	1.26
GO:00055	protein bin	Molecular	1.00	1.00	Solyc02g0	Solyc02g0	0.49	-1.04
GO:00055	protein bin	Molecular	1.00	1.00	Solyc10g0	Solyc10g0	0.40	-1.34
GO:00055	protein bin	Molecular	1.00	1.00	Solyc03g0	Solyc03g0	3.18	1.67
GO:00055	protein bin	Molecular	1.00	1.00	Solyc06g0	Solyc06g0	0.29	-1.81
GO:00055	protein bin	Molecular	1.00	1.00	Solyc09g0	Solyc09g0	0.09	-3.42
GO:00055	protein bin	Molecular	1.00	1.00	Solyc09g0	Solyc09g0	0.44	-1.18
GO:00055	protein bin	Molecular	1.00	1.00	Solyc02g0	Solyc02g0	2.12	1.09
GO:00055	protein bin	Molecular	1.00	1.00	Solyc09g0	Solyc09g0	0.34	-1.58
GO:00055	protein bin	Molecular	1.00	1.00	Solyc03g0	Solyc03g0	2.16	1.11
GO:00055	protein bin	Molecular	1.00	1.00	Solyc03g0	Solyc03g0	7.56	2.92
GO:00055	protein bin	Molecular	1.00	1.00	Solyc09g0	Solyc09g0	0.22	-2.17
GO:00055	protein bin	Molecular	1.00	1.00	Solyc03g0	Solyc03g0	0.31	-1.67
GO:00055	protein bin	Molecular	1.00	1.00	Solyc01g0	Solyc01g0	2.00	1.00
GO:00055	protein bin	Molecular	1.00	1.00	Solyc11g0	Solyc11g0	0.39	-1.35
GO:00055	protein bin	Molecular	1.00	1.00	Solyc09g0	Solyc09g0	3.53	1.82
GO:00055	protein bin	Molecular	1.00	1.00	Solyc09g0	Solyc09g0	20.47	4.36
GO:00055	protein bin	Molecular	1.00	1.00	Solyc03g0	Solyc03g0	2.06	1.05
GO:00055	protein bin	Molecular	1.00	1.00	Solyc04g0	Solyc04g0	0.43	-1.23
GO:00055	protein bin	Molecular	1.00	1.00	Solyc02g0	Solyc02g0	0.45	-1.14
GO:00055	protein bin	Molecular	1.00	1.00	Solyc04g0	Solyc04g0	0.41	-1.29
GO:00055	protein bin	Molecular	1.00	1.00	Solyc07g0	Solyc07g0	4.78	2.26
GO:00055	protein bin	Molecular	1.00	1.00	Solyc02g0	Solyc02g0	0.50	-1.00
GO:00055	protein bin	Molecular	1.00	1.00	Solyc02g0	Solyc02g0	0.45	-1.16
GO:00055	protein bin	Molecular	1.00	1.00	Solyc03g1	Solyc03g1	0.34	-1.56
GO:00055	protein bin	Molecular	1.00	1.00	Solyc08g0	Solyc08g0	0.20	-2.35
GO:00055	protein bin	Molecular	1.00	1.00	Solyc09g0	Solyc09g0	0.50	-1.00
GO:00055	protein bin	Molecular	1.00	1.00	Solyc09g0	Solyc09g0	0.49	-1.04
GO:00055	protein bin	Molecular	1.00	1.00	Solyc10g0	Solyc10g0	2.78	1.48
GO:00055	protein bin	Molecular	1.00	1.00	Solyc08g0	Solyc08g0	0.12	-3.05
GO:00055	protein bin	Molecular	1.00	1.00	Solyc05g0	Solyc05g0	0.50	-1.00
GO:00055	protein bin	Molecular	1.00	1.00	Solyc05g0	Solyc05g0	20549.37	14.33
GO:00055	protein bin	Molecular	1.00	1.00	Solyc03g1	Solyc03g1	0.43	-1.21
GO:00055	protein bin	Molecular	1.00	1.00	Solyc09g0	Solyc09g0	0.00	-16.52
GO:00055	protein bin	Molecular	1.00	1.00	Solyc02g0	Solyc02g0	2.08	1.06
GO:00055	protein bin	Molecular	1.00	1.00	Solyc01g0	Solyc01g0	3.70	1.89
GO:00055	protein bin	Molecular	1.00	1.00	Solyc09g0	Solyc09g0	2.07	1.05
GO:00055	protein bin	Molecular	1.00	1.00	Solyc02g0	Solyc02g0	0.47	-1.08
GO:00055	protein bin	Molecular	1.00	1.00	Solyc04g0	Solyc04g0	0.30	-1.76
GO:00055	protein bin	Molecular	1.00	1.00	Solyc01g0	Solyc01g0	0.49	-1.03
GO:00055	protein bin	Molecular	1.00	1.00	Solyc09g0	Solyc09g0	0.47	-1.07
GO:00055	protein bin	Molecular	1.00	1.00	Solyc09g0	Solyc09g0	0.48	-1.05
GO:00055	protein bin	Molecular	1.00	1.00	Solyc02g0	Solyc02g0	2.05	1.04
GO:00055	protein bin	Molecular	1.00	1.00	Solyc06g0	Solyc06g0	2.07	1.05
GO:00055	protein bin	Molecular	1.00	1.00	Solyc11g0	Solyc11g0	0.46	-1.12
GO:00055	protein bin	Molecular	1.00	1.00	Solyc01g1	Solyc01g1	0.00	-11.43
GO:00055	protein bin	Molecular	1.00	1.00	Solyc01g0	Solyc01g0	34.25	5.10

GO:00055	protein bin	Molecular	1.00	1.00	Solyc07g0	Solyc07g0	0.00	-12.30
GO:00055	protein bin	Molecular	1.00	1.00	Solyc03g1	Solyc03g1	2.61	1.39
GO:00055	protein bin	Molecular	1.00	1.00	Solyc06g0	Solyc06g0	0.15	-2.74
GO:00055	protein bin	Molecular	1.00	1.00	Solyc09g0	Solyc09g0	4.06	2.02
GO:00055	protein bin	Molecular	1.00	1.00	Solyc09g0	Solyc09g0	0.47	-1.10
GO:00055	protein bin	Molecular	1.00	1.00	Solyc06g0	Solyc06g0	0.15	-2.77
GO:00055	protein bin	Molecular	1.00	1.00	Solyc12g1	Solyc12g1	2.30	1.20
GO:00055	protein bin	Molecular	1.00	1.00	Solyc04g0	Solyc04g0	2.84	1.50
GO:00055	protein bin	Molecular	1.00	1.00	Solyc03g0	Solyc03g0	0.34	-1.54
GO:00055	protein bin	Molecular	1.00	1.00	Solyc02g0	Solyc02g0	0.27	-1.89
GO:00055	protein bin	Molecular	1.00	1.00	Solyc01g1	Solyc01g1	0.45	-1.15
GO:00055	protein bin	Molecular	1.00	1.00	Solyc09g0	Solyc09g0	4.54	2.18
GO:00055	protein bin	Molecular	1.00	1.00	Solyc12g0	Solyc12g0	0.27	-1.89
GO:00055	protein bin	Molecular	1.00	1.00	Solyc09g0	Solyc09g0	2.37	1.25
GO:00055	protein bin	Molecular	1.00	1.00	Solyc06g0	Solyc06g0	1942.11	10.92
GO:00055	protein bin	Molecular	1.00	1.00	Solyc04g0	Solyc04g0	0.46	-1.11
GO:00055	protein bin	Molecular	1.00	1.00	Solyc09g0	Solyc09g0	1363.92	10.41
GO:00055	protein bin	Molecular	1.00	1.00	Solyc08g0	Solyc08g0	0.50	-1.00
GO:00055	protein bin	Molecular	1.00	1.00	Solyc09g0	Solyc09g0	0.00	-11.38
GO:00055	protein bin	Molecular	1.00	1.00	Solyc04g0	Solyc04g0	0.39	-1.37
GO:00055	protein bin	Molecular	1.00	1.00	Solyc07g0	Solyc07g0	5532.77	12.43
GO:00055	protein bin	Molecular	1.00	1.00	Solyc03g1	Solyc03g1	57.07	5.83
GO:00055	protein bin	Molecular	1.00	1.00	Solyc05g0	Solyc05g0	21.99	4.46
GO:00055	protein bin	Molecular	1.00	1.00	Solyc07g0	Solyc07g0	2.02	1.02
GO:00055	protein bin	Molecular	1.00	1.00	Solyc05g0	Solyc05g0	4.22	2.08
GO:00055	protein bin	Molecular	1.00	1.00	Solyc02g0	Solyc02g0	2.60	1.38
GO:00055	protein bin	Molecular	1.00	1.00	Solyc08g0	Solyc08g0	0.27	-1.90
GO:00055	protein bin	Molecular	1.00	1.00	Solyc07g0	Solyc07g0	1880.17	10.88
GO:00055	protein bin	Molecular	1.00	1.00	Solyc04g0	Solyc04g0	0.15	-2.72
GO:00055	protein bin	Molecular	1.00	1.00	Solyc08g0	Solyc08g0	0.42	-1.25
GO:00055	protein bin	Molecular	1.00	1.00	Solyc05g0	Solyc05g0	7.28	2.86
GO:00055	protein bin	Molecular	1.00	1.00	Solyc08g0	Solyc08g0	4.09	2.03
GO:00055	protein bin	Molecular	1.00	1.00	Solyc03g0	Solyc03g0	2.40	1.26
GO:00055	protein bin	Molecular	1.00	1.00	Solyc02g0	Solyc02g0	0.18	-2.48
GO:00055	protein bin	Molecular	1.00	1.00	Solyc07g0	Solyc07g0	0.47	-1.10
GO:00055	protein bin	Molecular	1.00	1.00	Solyc04g0	Solyc04g0	0.18	-2.51
GO:00055	protein bin	Molecular	1.00	1.00	Solyc06g0	Solyc06g0	0.34	-1.57
GO:00055	protein bin	Molecular	1.00	1.00	Solyc12g0	Solyc12g0	467.31	8.87
GO:00055	protein bin	Molecular	1.00	1.00	Solyc04g0	Solyc04g0	2.47	1.30
GO:00055	protein bin	Molecular	1.00	1.00	Solyc07g0	Solyc07g0	2.39	1.26
GO:00055	protein bin	Molecular	1.00	1.00	Solyc02g0	Solyc02g0	0.50	-1.00
GO:00055	protein bin	Molecular	1.00	1.00	Solyc06g0	Solyc06g0	0.00	-9.64
GO:00055	protein bin	Molecular	1.00	1.00	Solyc09g0	Solyc09g0	73.03	6.19
GO:00055	protein bin	Molecular	1.00	1.00	Solyc06g0	Solyc06g0	0.45	-1.16
GO:00055	protein bin	Molecular	1.00	1.00	Solyc11g0	Solyc11g0	2.15	1.10
GO:00055	protein bin	Molecular	1.00	1.00	Solyc08g0	Solyc08g0	2.56	1.36
GO:00055	protein bin	Molecular	1.00	1.00	Solyc07g0	Solyc07g0	2.77	1.47
GO:00055	protein bin	Molecular	1.00	1.00	Solyc06g0	Solyc06g0	10.22	3.35
GO:00055	protein bin	Molecular	1.00	1.00	Solyc12g0	Solyc12g0	369.30	8.53
GO:00055	protein bin	Molecular	1.00	1.00	Solyc07g0	Solyc07g0	0.11	-3.12
GO:00055	protein bin	Molecular	1.00	1.00	Solyc04g0	Solyc04g0	0.34	-1.57

GO:00055	protein bin	Molecular	1.00	1.00	Solyc01g0	Solyc01g0	0.08	-3.62
GO:00055	protein bin	Molecular	1.00	1.00	Solyc08g0	Solyc08g0	4.19	2.07
GO:00055	protein bin	Molecular	1.00	1.00	Solyc01g1	Solyc01g1	2.45	1.29
GO:00055	protein bin	Molecular	1.00	1.00	Solyc03g0	Solyc03g0	0.36	-1.46
GO:00055	protein bin	Molecular	1.00	1.00	Solyc05g0	Solyc05g0	0.31	-1.68
GO:00055	protein bin	Molecular	1.00	1.00	Solyc08g0	Solyc08g0	0.11	-3.25
GO:00055	protein bin	Molecular	1.00	1.00	Solyc08g0	Solyc08g0	474.28	8.89
GO:00055	protein bin	Molecular	1.00	1.00	Solyc07g0	Solyc07g0	0.39	-1.35
GO:00055	protein bin	Molecular	1.00	1.00	Solyc03g0	Solyc03g0	0.39	-1.37
GO:00055	protein bin	Molecular	1.00	1.00	Solyc12g0	Solyc12g0	0.00	-10.33
GO:00055	protein bin	Molecular	1.00	1.00	Solyc09g0	Solyc09g0	0.00	-9.75
GO:00055	protein bin	Molecular	1.00	1.00	Solyc03g0	Solyc03g0	0.10	-3.29
GO:00055	protein bin	Molecular	1.00	1.00	Solyc09g0	Solyc09g0	0.22	-2.22
GO:00055	protein bin	Molecular	1.00	1.00	Solyc09g0	Solyc09g0	740.17	9.53
GO:00037	RNA bindi	Molecular	1.00	1.00	Solyc04g0	Solyc04g0	0.09	-3.54
GO:00037	RNA bindi	Molecular	1.00	1.00	Solyc11g0	Solyc11g0	2.27	1.18
GO:00037	RNA bindi	Molecular	1.00	1.00	Solyc07g0	Solyc07g0	6.72	2.75
GO:00037	RNA bindi	Molecular	1.00	1.00	Solyc11g0	Solyc11g0	4.62	2.21
GO:00037	RNA bindi	Molecular	1.00	1.00	Solyc02g0	Solyc02g0	0.49	-1.04
GO:00037	RNA bindi	Molecular	1.00	1.00	Solyc05g0	Solyc05g0	0.09	-3.44
GO:00037	RNA bindi	Molecular	1.00	1.00	Solyc04g0	Solyc04g0	0.13	-2.94
GO:00037	RNA bindi	Molecular	1.00	1.00	Solyc11g0	Solyc11g0	0.46	-1.13
GO:00037	RNA bindi	Molecular	1.00	1.00	Solyc10g0	Solyc10g0	10.66	3.41
GO:00037	RNA bindi	Molecular	1.00	1.00	Solyc10g0	Solyc10g0	2.18	1.13
GO:00037	RNA bindi	Molecular	1.00	1.00	Solyc09g0	Solyc09g0	0.05	-4.37
GO:00037	RNA bindi	Molecular	1.00	1.00	Solyc09g0	Solyc09g0	0.32	-1.62
GO:00037	RNA bindi	Molecular	1.00	1.00	Solyc06g0	Solyc06g0	2.07	1.05
GO:00037	RNA bindi	Molecular	1.00	1.00	Solyc10g0	Solyc10g0	2.20	1.14
GO:00037	RNA bindi	Molecular	1.00	1.00	Solyc09g0	Solyc09g0	0.47	-1.10
GO:00037	RNA bindi	Molecular	1.00	1.00	Solyc05g0	Solyc05g0	0.03	-5.29
GO:00037	RNA bindi	Molecular	1.00	1.00	Solyc04g0	Solyc04g0	2.74	1.46
GO:00037	RNA bindi	Molecular	1.00	1.00	Solyc05g0	Solyc05g0	2143.40	11.07
GO:00037	RNA bindi	Molecular	1.00	1.00	Solyc08g0	Solyc08g0	3.18	1.67
GO:00037	RNA bindi	Molecular	1.00	1.00	Solyc12g0	Solyc12g0	0.16	-2.65
GO:00037	RNA bindi	Molecular	1.00	1.00	Solyc05g0	Solyc05g0	20.96	4.39
GO:00037	RNA bindi	Molecular	1.00	1.00	Solyc09g0	Solyc09g0	0.25	-2.00

pval	qval	regulation
0.00	0.00	up
0.00	0.00	up
0.00	0.00	up
0.00	0.00	up
0.00	0.00	up
0.00	0.00	down
0.00	0.00	up
0.00	0.00	up
0.00	0.00	down
0.00	0.00	down
0.00	0.00	down
0.00	0.00	up
0.00	0.00	up
0.00	0.00	down
0.00	0.00	up
0.00	0.00	up
0.00	0.00	down
0.00	0.00	down
0.00	0.00	down
0.00	0.00	down
0.00	0.00	up
0.00	0.00	up
0.00	0.00	up
0.00	0.00	down
0.00	0.00	down
0.00	0.00	down
0.00	0.00	down
0.00	0.00	down
0.00	0.00	down
0.00	0.00	down
0.00	0.00	up
0.00	0.00	down
0.00	0.00	down
0.00	0.00	down
0.00	0.00	down
0.00	0.00	down
0.00	0.00	down
0.00	0.00	down
0.00	0.00	down
0.00	0.00	down
0.00	0.00	up
0.00	0.00	down
0.00	0.00	down
0.00	0.00	up
0.00	0.00	down
0.00	0.00	up
0.00	0.00	up
0.00	0.00	up
0.00	0.00	up

0.00	0.00	down
0.00	0.00	down
0.00	0.00	up
0.00	0.00	down
0.00	0.00	down
0.00	0.00	down
0.00	0.00	up
0.00	0.00	down
0.00	0.01	down
0.00	0.01	down
0.00	0.01	down
0.00	0.01	down
0.00	0.01	up
0.00	0.01	down
0.00	0.01	down
0.01	0.02	up
0.01	0.02	down
0.01	0.02	up
0.01	0.03	down
0.02	0.05	down
0.00	0.00	up
0.00	0.00	up
0.00	0.00	up
0.00	0.00	up
0.00	0.00	up
0.00	0.00	up
0.00	0.00	up
0.00	0.00	up
0.00	0.00	down
0.00	0.00	up
0.00	0.00	up
0.00	0.00	up
0.00	0.00	up
0.00	0.00	up
0.00	0.00	down
0.00	0.00	up
0.00	0.00	down
0.00	0.00	up
0.00	0.00	up
0.00	0.00	up
0.00	0.00	down
0.00	0.00	up
0.00	0.00	down
0.00	0.00	up
0.00	0.00	down
0.00	0.00	down
0.00	0.00	down
0.00	0.00	down
0.00	0.00	up
0.00	0.00	down
0.00	0.00	up

0.00	0.00	up
0.00	0.00	down
0.00	0.00	up
0.00	0.00	down
0.00	0.00	up
0.00	0.00	up
0.00	0.00	down
0.00	0.00	up
0.00	0.00	down
0.00	0.00	up
0.00	0.00	down
0.00	0.00	down
0.00	0.00	up
0.00	0.00	up
0.00	0.01	down
0.00	0.01	up
0.00	0.01	down
0.00	0.01	down
0.00	0.01	down
0.01	0.03	down
0.01	0.03	up
0.00	0.00	up
0.00	0.00	up
0.00	0.00	up
0.00	0.00	up
0.00	0.00	up
0.00	0.00	up
0.00	0.00	up
0.00	0.00	down
0.00	0.00	up
0.00	0.00	up
0.00	0.00	up
0.00	0.00	up
0.00	0.00	up
0.00	0.00	down
0.00	0.00	down
0.00	0.00	up
0.00	0.00	down
0.00	0.00	up
0.00	0.00	down
0.00	0.00	down
0.00	0.00	up
0.00	0.00	up
0.00	0.00	down
0.00	0.00	up
0.00	0.00	up
0.00	0.00	down
0.00	0.00	down
0.00	0.00	down
0.00	0.00	down
0.00	0.00	down

0.00	0.00	up
0.00	0.00	up
0.00	0.00	up
0.00	0.00	up
0.00	0.00	down
0.00	0.00	down
0.00	0.00	down
0.00	0.00	down
0.00	0.00	down
0.00	0.00	down
0.00	0.00	down
0.00	0.00	down
0.00	0.00	up
0.00	0.00	down
0.00	0.00	up
0.00	0.00	down
0.00	0.00	up
0.00	0.00	down
0.00	0.00	down
0.00	0.00	up
0.00	0.00	down
0.00	0.00	down
0.00	0.00	down
0.00	0.00	down
0.00	0.00	down
0.00	0.00	up
0.00	0.00	down
0.00	0.00	down
0.00	0.00	down
0.00	0.00	down
0.00	0.00	up
0.00	0.00	down
0.00	0.00	up
0.00	0.00	down
0.00	0.00	up
0.00	0.00	down
0.00	0.00	down
0.00	0.00	up
0.00	0.00	down
0.00	0.00	down
0.00	0.00	up
0.00	0.00	up
0.00	0.00	down
0.00	0.00	down
0.00	0.00	up
0.00	0.00	up
0.00	0.00	up
0.00	0.00	down
0.00	0.00	up
0.00	0.00	down
0.00	0.00	down

0.00	0.00	up
0.00	0.00	down
0.00	0.00	down
0.00	0.00	down
0.00	0.00	down
0.00	0.00	up
0.00	0.00	down
0.00	0.00	up
0.00	0.00	down
0.00	0.00	down
0.00	0.01	down
0.00	0.01	down
0.00	0.01	down
0.00	0.01	down
0.00	0.01	up
0.00	0.01	up
0.00	0.01	down
0.00	0.01	down
0.00	0.01	up
0.00	0.01	up
0.00	0.01	down
0.01	0.02	up
0.01	0.02	down
0.01	0.02	up
0.01	0.03	down
0.01	0.03	up
0.02	0.04	down
0.02	0.05	down
0	0	down
0.00	0.00	up
0.00	0.00	up
0.00	0.00	up
0.00	0.00	up
0.00	0.00	up
0.00	0.00	down
0.00	0.00	up
0.00	0.00	down
0.00	0.00	up
0.00	0.00	up
0.00	0.00	down
0.00	0.00	down
0.00	0.00	down
0.00	0.00	up
0.00	0.00	down
0.00	0.00	down
0.00	0.00	down
0.00	0.00	down
0.00	0.00	down
0.00	0.00	down
0.00	0.00	down
0.00	0.00	down

0.00	0.00	down
0.00	0.00	down
0.00	0.00	up
0.00	0.00	down
0.00	0.00	up
0.00	0.00	up
0.00	0.00	down
0.00	0.00	down
0.00	0.00	up
0.00	0.00	down
0.00	0.00	down
0.00	0.00	down
0.00	0.01	down
0.00	0.01	down
0.00	0.01	down
0.00	0.01	down
0.01	0.02	down
0.01	0.03	down
0.01	0.04	down
0	0	down
0.00	0.00	down
0.00	0.00	up
0.00	0.00	up
0.00	0.00	up
0.00	0.00	up
0.00	0.00	up
0.00	0.00	down
0.00	0.00	up
0.00	0.00	up
0.00	0.00	up
0.00	0.00	up
0.00	0.00	down
0.00	0.00	up
0.00	0.00	down
0.00	0.00	down
0.00	0.00	down
0.00	0.00	down
0.00	0.00	down
0.00	0.00	down
0.00	0.00	up
0.00	0.00	down
0.00	0.00	down
0.00	0.00	down
0.00	0.00	down
0.00	0.00	up
0.00	0.00	down
0.00	0.00	up
0.00	0.00	up
0.00	0.00	up
0.00	0.00	up
0.00	0.00	down

0.00	0.01	down
0.00	0.01	down
0.00	0.01	up
0.00	0.01	up
0.00	0.01	down
0.01	0.02	up
0.01	0.02	down
0.01	0.03	up
0.01	0.03	up
0.01	0.04	down
0	0	up
0	0	up
0	0	up
0.00	0.00	up
0.00	0.00	down
0.00	0.00	down
0.00	0.00	up
0.00	0.00	up
0.00	0.00	up
0.00	0.00	up
0.00	0.00	up
0.00	0.00	up
0.00	0.00	up
0.00	0.00	up
0.00	0.00	down
0.00	0.00	up
0.00	0.00	up
0.00	0.00	down
0.00	0.00	up
0.00	0.00	up
0.00	0.00	up
0.00	0.00	down
0.00	0.00	up
0.00	0.00	up
0.00	0.00	up
0.00	0.00	up
0.00	0.00	up
0.00	0.00	up
0.00	0.00	up
0.00	0.00	up
0.00	0.00	down
0.00	0.00	up
0.00	0.00	down
0.00	0.00	up
0.00	0.00	up
0.00	0.00	down
0.00	0.00	up
0.00	0.00	down
0.00	0.00	up
0.00	0.00	up
0.00	0.00	up

0.00	0.00	down
0.00	0.00	up
0.00	0.00	up
0.00	0.00	down
0.00	0.00	up
0.00	0.00	down
0.00	0.00	up
0.00	0.00	up
0.00	0.00	up
0.00	0.00	up
0.00	0.00	up
0.00	0.00	down
0.00	0.00	up
0.00	0.00	down
0.00	0.00	up
0.00	0.00	up
0.00	0.00	down
0.00	0.00	up
0.00	0.00	up
0.00	0.00	down
0.00	0.00	down
0.00	0.00	up
0.00	0.00	down
0.00	0.00	up
0.00	0.00	up
0.00	0.00	up
0.00	0.00	up
0.00	0.00	up
0.00	0.00	down
0.00	0.00	up
0.00	0.00	up
0.00	0.00	up
0.00	0.00	down
0.00	0.00	up
0.00	0.00	up
0.00	0.00	down
0.00	0.00	up
0.00	0.00	up
0.00	0.00	down
0.00	0.00	up
0.00	0.00	down
0.00	0.00	down
0.00	0.00	down
0.00	0.00	up
0.00	0.00	up
0.00	0.00	down
0.00	0.00	up
0.00	0.00	up
0.00	0.00	down
0.00	0.00	up
0.00	0.00	up

0.00	0.00	down
0.00	0.01	down
0.00	0.01	up
0.00	0.01	down
0.00	0.01	up
0.00	0.01	up
0.01	0.02	up
0.01	0.02	up
0.01	0.02	up
0.01	0.02	up
0.01	0.02	up
0.01	0.02	down
0.01	0.02	down
0.01	0.03	up
0.01	0.03	up
0.02	0.05	down
0.02	0.05	down
0.02	0.05	up
0.00	0.00	down
0.00	0.00	down
0.00	0.00	up
0.00	0.00	up
0.00	0.00	up
0.00	0.00	up
0.00	0.00	up
0.00	0.00	up
0.00	0.00	up
0.00	0.00	up
0.00	0.00	down
0.00	0.00	up
0.00	0.00	up
0.00	0.00	up
0.00	0.00	up
0.00	0.00	up
0.00	0.00	down
0.00	0.00	up
0.00	0.00	up
0.00	0.00	down
0.00	0.00	down
0.00	0.00	up
0.00	0.00	up
0.00	0.00	up
0.00	0.00	up
0.00	0.00	up
0.00	0.00	up
0.00	0.00	up
0.00	0.00	up
0.00	0.00	up
0.00	0.00	up
0.00	0.00	up
0.00	0.00	up

0.00	0.00	up
0.00	0.00	up
0.00	0.00	down
0.00	0.00	down
0.00	0.00	down
0.00	0.00	up
0.00	0.00	down
0.00	0.00	up
0.00	0.00	down
0.00	0.00	up
0.00	0.00	down
0.00	0.00	down
0.00	0.00	up
0.00	0.00	down
0.00	0.00	down
0.00	0.00	down
0.00	0.00	up
0.00	0.00	up
0.00	0.00	down
0.00	0.00	up
0.00	0.00	down
0.00	0.00	up
0.00	0.00	down
0.00	0.00	up
0.00	0.00	down
0.00	0.00	down
0.00	0.00	up
0.00	0.00	down
0.00	0.00	up
0.00	0.00	down
0.00	0.00	up
0.00	0.00	down
0.00	0.00	up
0.00	0.00	down
0.00	0.00	down
0.00	0.00	down
0.00	0.00	down
0.00	0.00	up
0.00	0.00	up
0.00	0.00	up
0.00	0.00	down
0.00	0.00	down
0.00	0.00	down
0.00	0.00	up
0.00	0.00	up
0.00	0.00	down
0.00	0.00	up
0.00	0.00	up
0.00	0.00	down
0.00	0.00	up
0.00	0.00	up

0.00	0.00	down
0.00	0.00	up
0.00	0.00	down
0.00	0.00	down
0.00	0.00	down
0.00	0.00	down
0.00	0.00	up
0.00	0.00	up
0.00	0.00	down
0.00	0.00	up
0.00	0.00	down
0.00	0.00	down
0.00	0.00	down
0.00	0.00	down
0.00	0.00	down
0.00	0.00	up
0.00	0.00	down
0.00	0.00	up
0.00	0.00	down
0.00	0.00	down
0.00	0.00	down
0.00	0.00	down
0.00	0.00	up
0.00	0.00	up
0.00	0.00	down
0.00	0.00	up
0.00	0.00	up
0.00	0.00	up
0.00	0.00	up
0.00	0.00	down
0.00	0.00	up
0.00	0.00	up
0.00	0.00	down
0.00	0.00	down
0.00	0.00	down
0.00	0.00	down
0.00	0.00	down
0.00	0.00	up
0.00	0.00	down
0.00	0.00	down
0.00	0.00	down
0.00	0.00	down
0.00	0.00	up
0.00	0.00	down
0.00	0.00	up
0.00	0.00	up
0.00	0.00	down
0.00	0.00	up
0.00	0.00	down
0.00	0.00	up
0.00	0.00	down

0.00	0.00	up
0.00	0.00	up
0.00	0.00	up
0.00	0.00	up
0.00	0.00	up
0.00	0.00	up
0.00	0.00	down
0.00	0.00	up
0.00	0.00	up
0.00	0.00	down
0.00	0.00	down
0.00	0.00	down
0.00	0.00	up
0.00	0.00	up
0.00	0.00	up
0.00	0.00	up
0.00	0.00	down
0.00	0.00	up
0.00	0.00	down
0.00	0.00	down
0.00	0.00	down
0.00	0.00	up
0.00	0.00	down
0.00	0.00	up
0.00	0.00	down
0.00	0.00	up
0.00	0.00	up
0.00	0.00	up
0.00	0.00	down
0.00	0.00	down
0.00	0.00	down
0.00	0.00	up
0.00	0.00	down
0.00	0.00	down
0.00	0.00	up
0.00	0.00	down
0.00	0.00	down
0.00	0.00	down
0.00	0.00	down
0.00	0.00	up
0.00	0.00	down
0.00	0.00	up
0.00	0.01	up
0.00	0.01	up
0.00	0.01	down
0.00	0.01	up
0.00	0.01	down
0.00	0.01	down
0.00	0.01	up
0.00	0.01	up
0.00	0.01	down

0.00	0.01	down
0.00	0.01	up
0.00	0.01	up
0.00	0.01	down
0.00	0.02	down
0.00	0.02	up
0.01	0.02	up
0.01	0.02	down
0.01	0.02	up
0.01	0.02	down
0.01	0.02	down
0.01	0.02	up
0.01	0.02	down
0.01	0.02	down
0.01	0.02	down
0.01	0.02	down
0.01	0.03	down
0.01	0.03	down
0.01	0.04	down
0.01	0.04	down
0.01	0.04	up
0.02	0.05	up
0.02	0.05	down
0.00	0.00	up
0.00	0.00	up
0.00	0.00	up
0.00	0.00	up
0.00	0.00	up
0.00	0.00	up
0.00	0.00	up
0.00	0.00	up
0.00	0.00	up
0.00	0.00	up
0.00	0.00	up
0.00	0.00	down
0.00	0.00	down
0.00	0.00	up
0.00	0.00	up
0.00	0.00	up
0.00	0.00	up
0.00	0.00	down
0.00	0.01	down
0.00	0.01	up
0.01	0.03	up
0	0	down
0.00	0.00	up
0.00	0.00	up
0.00	0.00	up
0.00	0.00	down
0.00	0.00	down
0.00	0.00	down

0.00	0.00	down
0.00	0.00	down
0.00	0.00	up
0.00	0.00	up
0.00	0.00	up
0.00	0.00	up
0.00	0.00	down
0.00	0.01	down
0.00	0.01	up
0.01	0.03	up
0.01	0.04	down
0	0	down
0.00	0.00	down
0.00	0.00	down
0.00	0.00	down
0.00	0.00	down
0.00	0.00	down
0.00	0.00	down
0.00	0.00	down
0.00	0.00	down
0.00	0.00	down
0.00	0.00	down
0.00	0.00	down
0.00	0.00	down
0.00	0.00	down
0.00	0.00	down
0.00	0.01	down
0.01	0.03	down
0	0	up
0.00	0.00	down
0.00	0.00	down
0.00	0.00	down
0.00	0.00	up
0.00	0.00	up
0.00	0.00	down
0.00	0.00	up
0.00	0.00	down
0.00	0.00	up
0.00	0.00	up
0.00	0.00	down
0.00	0.00	down
0.00	0.00	down
0.00	0.00	up
0.00	0.00	up
0.00	0.00	down
0.00	0.00	up
0.00	0.00	down
0.00	0.00	up
0.00	0.00	up
0.00	0.00	up
0.00	0.00	down

0.00	0.00	up
0.00	0.00	down
0.00	0.00	down
0.00	0.00	down
0.00	0.00	up
0.00	0.00	up
0.00	0.00	up
0.00	0.00	up
0.00	0.00	up
0.00	0.00	down
0.00	0.00	down
0.00	0.00	up
0.00	0.00	up
0.00	0.00	down
0.00	0.00	down
0.00	0.00	up
0.00	0.00	up
0.00	0.00	down
0.00	0.00	up
0.00	0.00	up
0.00	0.00	down
0.00	0.00	up
0.00	0.00	down
0.00	0.00	down
0.00	0.00	up
0.00	0.00	up
0.00	0.00	down
0.00	0.00	up
0.00	0.00	up
0.00	0.00	up
0.00	0.00	up
0.00	0.00	down
0.00	0.00	down
0.00	0.00	up
0.00	0.00	up
0.00	0.00	down
0.00	0.00	up
0.00	0.00	up
0.00	0.00	up
0.00	0.00	down
0.00	0.00	down
0.00	0.00	down
0.00	0.00	up
0.00	0.00	up
0.00	0.00	up
0.00	0.00	up
0.00	0.00	up
0.00	0.00	down
0.00	0.00	up
0.00	0.00	up
0.00	0.00	down

0.00	0.00	down
0.00	0.00	down
0.00	0.00	up
0.00	0.00	up
0.00	0.00	up
0.00	0.00	down
0.00	0.00	up
0.00	0.00	up
0.00	0.00	up
0.00	0.00	up
0.00	0.00	up
0.00	0.00	up
0.00	0.00	up
0.00	0.00	up
0.00	0.00	down
0.00	0.00	up
0.00	0.00	down
0.00	0.00	up
0.00	0.00	down
0.00	0.00	up
0.00	0.01	down
0.00	0.01	down
0.00	0.01	up
0.00	0.01	down
0.00	0.01	up
0.00	0.01	up
0.00	0.01	up
0.00	0.02	down
0.01	0.02	up
0.01	0.02	up
0.01	0.02	down
0.01	0.02	up
0.01	0.02	up
0.01	0.04	down
0.02	0.05	up
0.02	0.05	up
0.02	0.05	up
0	0	up
0.00	0.00	up
0.00	0.00	up
0.00	0.00	down
0.00	0.00	up
0.00	0.00	down
0.00	0.00	down
0.00	0.00	up
0.00	0.00	up
0.00	0.00	up
0.00	0.00	up
0.00	0.00	down
0.00	0.00	up
0.00	0.00	up

0.00	0.00	down
0.00	0.00	up
0.00	0.00	down
0.00	0.00	up
0.00	0.00	up
0.00	0.00	up
0.00	0.00	up
0.00	0.00	up
0.00	0.00	up
0.00	0.00	down
0.00	0.00	up
0.00	0.00	down
0.00	0.00	down
0.00	0.00	down
0.00	0.00	down
0.00	0.00	down
0.00	0.00	down
0.00	0.00	up
0.00	0.00	up
0.00	0.00	down
0.00	0.00	up
0.00	0.00	up
0.00	0.00	up
0.00	0.00	down
0.00	0.00	up
0.00	0.00	up
0.00	0.00	up
0.00	0.00	down
0.00	0.00	up
0.00	0.00	down
0.00	0.00	down
0.00	0.00	down
0.00	0.01	up
0.00	0.01	down
0.00	0.01	up
0.00	0.01	up
0.00	0.02	up
0.01	0.02	up
0.01	0.02	down
0.02	0.05	up
0	0	down
0	0	down
0.00	0.00	down
0.00	0.00	down
0.00	0.00	up
0.00	0.00	down
0.00	0.00	up
0.00	0.00	up
0.00	0.00	up
0.00	0.00	up
0.00	0.00	up

0.00	0.00	down
0.00	0.00	down
0.00	0.00	down
0.00	0.00	up
0.00	0.00	down
0.00	0.00	down
0.00	0.00	down
0.00	0.00	down
0.00	0.00	down
0.00	0.00	up
0.00	0.00	down
0.00	0.00	up
0.00	0.00	up
0.00	0.00	up
0.00	0.00	up
0.00	0.00	down
0.00	0.00	up
0.00	0.00	down
0.00	0.00	down
0.00	0.00	up
0.00	0.00	up
0.00	0.00	down
0.00	0.00	up
0.00	0.00	up
0.00	0.00	up
0.00	0.00	up
0.00	0.00	down
0.00	0.00	down
0.00	0.00	up
0.00	0.00	down
0.00	0.00	up
0.00	0.00	up
0.00	0.00	up
0.00	0.00	up
0.00	0.00	up
0.00	0.00	down
0.00	0.00	down
0.00	0.00	down
0.00	0.00	up
0.00	0.00	down
0.00	0.00	down
0.00	0.00	up
0.00	0.00	up
0.00	0.00	up
0.00	0.00	down
0.00	0.00	up
0.00	0.00	up
0.00	0.00	down
0.00	0.00	up
0.00	0.00	up
0.00	0.00	down

0.00	0.00	down
0.00	0.00	up
0.00	0.00	down
0.00	0.00	down
0.00	0.00	down
0.00	0.00	up
0.00	0.00	down
0.00	0.00	up
0.00	0.00	up
0.00	0.00	up
0.00	0.00	down
0.00	0.00	down
0.00	0.00	up
0.00	0.00	up
0.00	0.00	up
0.00	0.00	down
0.00	0.00	down
0.00	0.00	up
0.00	0.00	down
0.00	0.00	up
0.00	0.00	down
0.00	0.00	down
0.00	0.00	down
0.00	0.00	down
0.00	0.00	down
0.00	0.00	down
0.00	0.00	up
0.00	0.00	down
0.00	0.00	up
0.00	0.00	down
0.00	0.00	up
0.00	0.00	down
0.00	0.00	down
0.00	0.00	up
0.00	0.00	down
0.00	0.00	up
0.00	0.00	down
0.00	0.00	up
0.00	0.00	down
0.00	0.00	up
0.00	0.00	down
0.00	0.00	up
0.00	0.00	up
0.00	0.00	down
0.00	0.00	down
0.00	0.00	down
0.00	0.00	up
0.00	0.00	down
0.00	0.00	up
0.00	0.00	up
0.00	0.00	down

0.00	0.00	up
0.00	0.00	up
0.00	0.00	up
0.00	0.00	up
0.00	0.00	down
0.00	0.00	down
0.00	0.00	down
0.00	0.00	up
0.00	0.00	down
0.00	0.00	down
0.00	0.00	down
0.00	0.00	up
0.00	0.00	up
0.00	0.00	up
0.00	0.00	down
0.00	0.00	up
0.00	0.00	up
0.00	0.00	down
0.00	0.00	up
0.00	0.00	down
0.00	0.00	up
0.00	0.00	down
0.00	0.00	up
0.00	0.00	up
0.00	0.00	down
0.00	0.00	up
0.00	0.00	up
0.00	0.00	up
0.00	0.00	up
0.00	0.00	up
0.00	0.00	down
0.00	0.00	up
0.00	0.00	up
0.00	0.00	up
0.00	0.00	down
0.00	0.00	up
0.00	0.00	up
0.00	0.00	down
0.00	0.00	down
0.00	0.00	down
0.00	0.00	down
0.00	0.00	down
0.00	0.00	up
0.00	0.00	up
0.00	0.00	down
0.00	0.00	down
0.00	0.00	down
0.00	0.00	down
0.00	0.00	down
0.00	0.00	down
0.00	0.00	up

0.00	0.00	up
0.00	0.00	up
0.00	0.00	down
0.00	0.00	down
0.00	0.00	up
0.00	0.00	up
0.00	0.00	down
0.00	0.00	down
0.00	0.00	up
0.00	0.00	up
0.00	0.00	up
0.00	0.00	down
0.00	0.00	up
0.00	0.00	down
0.00	0.00	down
0.00	0.00	up
0.00	0.00	down
0.00	0.00	down
0.00	0.00	up
0.00	0.00	up
0.00	0.00	down
0.00	0.00	up
0.00	0.00	up
0.00	0.00	down
0.00	0.00	down
0.00	0.00	down
0.00	0.00	down
0.00	0.00	down
0.00	0.00	down
0.00	0.00	down
0.00	0.00	up
0.00	0.00	down
0.00	0.00	down
0.00	0.00	down
0.00	0.00	down
0.00	0.00	down
0.00	0.00	up
0.00	0.00	up
0.00	0.00	down
0.00	0.00	up
0.00	0.00	down
0.00	0.00	up
0.00	0.00	down
0.00	0.00	down
0.00	0.00	down
0.00	0.00	up
0.00	0.00	up
0.00	0.00	down
0.00	0.00	up
0.00	0.00	up
0.00	0.00	up

0.00	0.00	down
0.00	0.00	up
0.00	0.00	down
0.00	0.00	up
0.00	0.00	up
0.00	0.00	down
0.00	0.00	down
0.00	0.00	up
0.00	0.00	up
0.00	0.00	down
0.00	0.00	up
0.00	0.00	up
0.00	0.00	up
0.00	0.00	up
0.00	0.00	up
0.00	0.00	down
0.00	0.00	up
0.00	0.00	down
0.00	0.00	down
0.00	0.00	up
0.00	0.00	up
0.00	0.00	up
0.00	0.00	up
0.00	0.00	down
0.00	0.00	up
0.00	0.00	up
0.00	0.00	down
0.00	0.00	down
0.00	0.00	down
0.00	0.00	up
0.00	0.00	up
0.00	0.00	down
0.00	0.00	down
0.00	0.00	down
0.00	0.00	up
0.00	0.00	up
0.00	0.00	up
0.00	0.00	up
0.00	0.00	down
0.00	0.00	up
0.00	0.00	up
0.00	0.00	down
0.00	0.00	up
0.00	0.00	down
0.00	0.00	up
0.00	0.00	up
0.00	0.00	down
0.00	0.00	up
0.00	0.00	down
0.00	0.00	down
0.00	0.00	down

0.00	0.00	up
0.00	0.01	up
0.00	0.01	up
0.00	0.01	down
0.00	0.01	up
0.00	0.01	down
0.00	0.01	up
0.00	0.01	up
0.00	0.01	up
0.00	0.01	down
0.00	0.01	down
0.00	0.01	down
0.00	0.01	down
0.00	0.01	up
0.00	0.01	up
0.00	0.01	up
0.00	0.01	up
0.00	0.01	down
0.00	0.01	down
0.00	0.01	down
0.00	0.01	up
0.00	0.01	up
0.00	0.01	down
0.00	0.01	up
0.00	0.01	up
0.00	0.01	down
0.00	0.01	down
0.00	0.01	down
0.00	0.01	down
0.00	0.01	down
0.00	0.01	up
0.00	0.01	down
0.00	0.01	up
0.00	0.02	down
0.01	0.02	up
0.01	0.02	down
0.01	0.02	up
0.01	0.02	up
0.01	0.02	up
0.01	0.02	up
0.01	0.02	up
0.01	0.02	up
0.01	0.02	down
0.01	0.02	up
0.01	0.02	down
0.01	0.02	down
0.01	0.02	up
0.01	0.02	down
0.01	0.02	up
0.01	0.02	down
0.01	0.02	up

0.01	0.02	down
0.01	0.02	down
0.01	0.02	down
0.01	0.02	up
0.01	0.02	down
0.01	0.02	down
0.01	0.02	up
0.01	0.03	up
0.01	0.03	down
0.01	0.03	down
0.01	0.03	down
0.01	0.03	down
0.01	0.03	up
0.01	0.03	up
0.01	0.03	up
0.01	0.03	down
0.01	0.04	down
0.01	0.04	down
0.01	0.04	down
0.01	0.04	up
0.01	0.04	up
0.02	0.04	up
0.02	0.05	up
0.02	0.05	down
0.02	0.05	up
0.02	0.05	down
0.02	0.05	down
0.00	0.00	up
0.00	0.00	up
0.00	0.00	up
0.00	0.00	up
0.00	0.00	up
0.00	0.00	down
0.00	0.00	up
0.00	0.00	down
0.00	0.00	down
0.00	0.00	up
0.00	0.00	up
0.00	0.00	up
0.00	0.00	down
0.00	0.00	up
0.00	0.00	up
0.00	0.00	down
0.00	0.00	up
0.00	0.00	up
0.00	0.00	up
0.00	0.00	up
0.00	0.00	up
0.00	0.00	down
0.00	0.00	up
0.00	0.00	up

0.00	0.00	up
0.00	0.00	up
0.00	0.00	up
0.00	0.00	up
0.00	0.00	up
0.00	0.00	up
0.00	0.00	down
0.00	0.00	down
0.00	0.00	down
0.00	0.00	down
0.00	0.00	down
0.00	0.00	down
0.00	0.00	up
0.00	0.00	up
0.00	0.00	up
0.00	0.00	up
0.00	0.00	up
0.00	0.00	up
0.00	0.00	down
0.00	0.00	down
0.00	0.00	down
0.00	0.00	up
0.00	0.00	up
0.00	0.00	up
0.00	0.00	down
0.00	0.00	up
0.00	0.00	down
0.00	0.00	down
0.00	0.00	down
0.00	0.01	up
0.00	0.01	down
0.00	0.01	up
0.00	0.01	down
0.00	0.01	up
0.00	0.02	down
0.00	0.02	up
0.01	0.02	down
0.01	0.03	up
0.02	0.05	up
0.02	0.05	up
0	0	up
0	0	up
0.00	0.00	up
0.00	0.00	down
0.00	0.00	up
0.00	0.00	up
0.00	0.00	up
0.00	0.00	up
0.00	0.00	up
0.00	0.00	up
0.00	0.00	up

0.00	0.00	up
0.00	0.00	up
0.00	0.00	up
0.00	0.00	down
0.00	0.00	down
0.00	0.00	down
0.00	0.00	up
0.00	0.00	down
0.00	0.00	up
0.00	0.00	up
0.00	0.00	down
0.00	0.00	up
0.00	0.00	down
0.00	0.00	down
0.00	0.00	down
0.00	0.00	down
0.00	0.00	up
0.00	0.01	up
0.00	0.01	up
0.00	0.01	up
0.01	0.02	up
0.01	0.03	up
0.01	0.03	up
0.02	0.05	down
0.02	0.05	up
0	0	up
0	0	up
0.00	0.00	up
0.00	0.00	down
0.00	0.00	up
0.00	0.00	up
0.00	0.00	up
0.00	0.00	down
0.00	0.00	up
0.00	0.00	down
0.00	0.00	down
0.00	0.00	up
0.00	0.00	up
0.00	0.00	up
0.00	0.00	up
0.00	0.00	up
0.00	0.00	up
0.00	0.00	down
0.00	0.00	up
0.00	0.00	down
0.00	0.00	up
0.00	0.00	up
0.00	0.00	up
0.00	0.00	up
0.00	0.00	up
0.00	0.00	up

0.00	0.00	down
0.00	0.00	up
0.00	0.00	down
0.00	0.00	down
0.00	0.00	down
0.00	0.00	down
0.00	0.00	up
0.00	0.00	up
0.00	0.00	up
0.00	0.00	up
0.00	0.00	up
0.00	0.00	down
0.00	0.00	down
0.00	0.00	up
0.00	0.00	down
0.00	0.00	down
0.00	0.00	down
0.00	0.00	up
0.00	0.00	up
0.00	0.00	up
0.00	0.00	up
0.00	0.00	up
0.00	0.00	up
0.00	0.00	down
0.00	0.00	up
0.00	0.01	down
0.00	0.01	up
0.00	0.01	up
0.00	0.01	up
0.01	0.02	up
0.01	0.03	down
0.02	0.05	up
0.02	0.05	up
0.00	0.00	up
0.00	0.00	up
0.00	0.00	up
0.00	0.00	up
0.00	0.00	up
0.01	0.03	up
0.00	0.00	down
0.00	0.00	up
0.00	0.00	up
0.00	0.00	down
0.00	0.00	down
0.00	0.00	down
0.00	0.00	down
0.00	0.00	up
0.00	0.00	down
0.00	0.00	up
0.00	0.00	up
0.00	0.00	up

0.00	0.00	down
0	0	up
0.00	0.00	down
0.00	0.00	up
0.00	0.00	down
0.00	0.00	up
0.00	0.00	up
0.00	0.00	up
0.00	0.00	up
0.00	0.00	up
0.00	0.00	up
0.00	0.00	down
0.00	0.00	up
0.00	0.00	up
0.00	0.00	up
0.00	0.00	up
0.00	0.00	up
0.00	0.00	up
0.00	0.00	up
0.00	0.01	up
0	0	down
0.00	0.00	down
0.00	0.00	down
0.00	0.00	down
0.00	0.00	up
0.00	0.00	down
0.00	0.00	down
0.00	0.00	down
0.00	0.01	down
0.00	0.00	up
0.00	0.00	up
0.00	0.00	down
0.00	0.00	down
0.00	0.00	down
0.00	0.00	down
0.00	0.00	down
0.00	0.00	up
0.00	0.00	up
0.00	0.00	up
0.00	0.00	down
0.00	0.00	down
0	0	up
0	0	up
0.00	0.00	down
0.00	0.00	up
0.00	0.00	up
0.00	0.00	down
0.00	0.00	up
0.00	0.00	up
0.00	0.00	up
0.00	0.00	up

0.00	0.00	up
0.00	0.00	up
0.00	0.00	down
0.00	0.00	down
0.00	0.00	up
0.00	0.00	up
0.00	0.00	up
0.00	0.01	up
0.00	0.00	up
0.00	0.00	up
0.00	0.00	down
0.00	0.00	up
0.00	0.00	down
0.00	0.00	down
0.00	0.00	down
0.00	0.00	up
0.00	0.00	up
0.00	0.00	up
0.00	0.00	down
0.00	0.00	up
0.00	0.00	up
0.00	0.00	up
0.00	0.00	up
0.00	0.00	up
0.00	0.00	up
0.00	0.00	up
0.00	0.00	up
0.00	0.00	up
0.00	0.00	up
0.00	0.00	down
0.00	0.00	down
0.00	0.00	up
0.00	0.00	down
0.00	0.00	up
0.00	0.00	up
0.00	0.00	up
0.00	0.00	up
0.00	0.00	down
0.00	0.00	up
0.00	0.00	up
0.00	0.00	up
0.00	0.00	down
0.00	0.00	up
0.00	0.00	down
0.00	0.00	down
0.00	0.00	down
0.00	0.01	up
0.00	0.01	down
0.00	0.01	up
0.00	0.01	up
0.00	0.01	up

0.00	0.02	up
0.01	0.02	down
0.02	0.05	up
0	0	down
0.00	0.00	down
0.00	0.00	up
0.00	0.00	up
0.00	0.00	up
0.00	0.00	up
0.00	0.00	down
0.00	0.00	up
0.00	0.00	up
0.00	0.00	up
0.00	0.00	up
0.00	0.00	down
0.00	0.00	up
0.00	0.00	down
0.00	0.00	up
0.00	0.00	up
0.00	0.00	down
0.00	0.00	up
0.00	0.00	up
0.00	0.00	up
0.00	0.00	up
0.00	0.00	up
0.00	0.00	up
0.00	0.00	down
0.00	0.01	up
0.00	0.01	down
0.01	0.02	down
0.01	0.03	up
0.02	0.04	up
0	0	down
0.00	0.00	down
0.00	0.00	up
0.00	0.00	down
0.00	0.00	down
0.00	0.00	down
0.00	0.00	down
0.00	0.01	down
0.00	0.00	up
0.00	0.00	down
0.00	0.00	down
0.00	0.00	up
0.00	0.00	up
0.00	0.00	down
0.00	0.00	down
0.00	0.00	down
0.00	0.00	down
0.00	0.00	up
0.00	0.00	up

0.00	0.00	up
0.00	0.00	up
0.00	0.00	up
0.00	0.00	up
0.00	0.00	up
0.00	0.00	up
0.00	0.00	up
0.00	0.00	up
0.00	0.00	up
0.00	0.00	up
0.00	0.00	up
0.00	0.00	up
0.00	0.00	up
0.00	0.00	up
0.00	0.00	up
0.00	0.00	up
0.00	0.00	up
0.00	0.00	down
0.00	0.00	down
0.00	0.00	down
0.00	0.00	down
0.00	0.00	up
0.00	0.00	up
0.00	0.00	up
0.00	0.00	up
0.00	0.00	up
0.00	0.00	down
0.00	0.00	up
0.00	0.00	up
0.00	0.00	up
0.00	0.00	down
0.00	0.01	up
0.00	0.01	up
0.00	0.00	down
0.00	0.00	up
0.00	0.00	down
0.00	0.00	down
0.00	0.00	down
0.00	0.00	down
0.01	0.03	down
0	0	up
0.00	0.00	down
0.00	0.00	up
0.00	0.00	down
0.00	0.00	up
0.00	0.00	up
0.00	0.00	up
0.02	0.05	up
0.00	0.00	up
0.00	0.00	up
0.00	0.00	up

0.00	0.00	up
0.00	0.00	up
0.00	0.00	up
0.00	0.01	up
0.01	0.03	up
0.00	0.00	up
0.00	0.00	up
0.00	0.00	down
0.00	0.00	down
0.00	0.00	up
0.00	0.00	down
0.00	0.00	down
0.00	0.00	down
0.00	0.00	down
0.00	0.00	down
0.00	0.02	down
0.01	0.03	up
0.01	0.03	down
0	0	up
0	0	up
0.00	0.00	down
0.00	0.00	up
0.00	0.00	up
0.00	0.00	down
0.00	0.00	up
0.00	0.00	up
0.00	0.00	up
0.00	0.00	up
0.00	0.00	up
0.00	0.00	down
0.00	0.00	down
0.00	0.00	up
0.00	0.00	up
0.00	0.01	up
0.00	0.00	down
0.00	0.00	up
0.00	0.00	up
0.00	0.00	up
0.00	0.00	up
0.00	0.00	up
0.00	0.00	down
0.00	0.00	down
0.00	0.00	down
0.00	0.00	up
0.00	0.00	up
0.00	0.00	down
0.00	0.00	down
0.00	0.00	down
0.00	0.00	up
0.00	0.00	up
0.00	0.00	up

0.00	0.00	down
0.00	0.00	up
0.00	0.00	down
0.00	0.00	up
0.00	0.00	up
0.00	0.00	up
0.00	0.00	up
0.00	0.00	down
0.00	0.00	down
0.00	0.00	down
0.00	0.00	down
0.00	0.00	up
0.00	0.00	down
0.00	0.00	up
0.00	0.00	up
0.00	0.00	down
0.00	0.00	up
0.00	0.00	up
0.00	0.00	down
0.00	0.00	up
0.00	0.00	up
0.00	0.00	down
0.00	0.00	up
0.00	0.00	up
0.00	0.00	up
0.00	0.00	down
0.00	0.00	down
0.00	0.00	up
0.00	0.00	up
0.00	0.00	up
0.00	0.00	up
0.00	0.00	down
0.00	0.00	down
0.00	0.00	down
0.00	0.00	down
0.00	0.00	up
0.00	0.00	up
0.00	0.00	down
0.00	0.00	up
0.00	0.00	up
0.00	0.00	down
0.00	0.00	up
0.00	0.00	up
0.00	0.00	up
0.00	0.00	down
0.00	0.00	down
0.00	0.00	up
0.00	0.01	up
0.00	0.01	up
0.00	0.01	up
0.00	0.01	up

0.00	0.01	up
0.00	0.01	up
0.00	0.01	down
0.01	0.02	up
0.01	0.02	down
0.01	0.02	up
0.01	0.02	down
0.01	0.03	down
0.01	0.04	down
0	0	up
0.00	0.00	up
0.00	0.00	up
0.00	0.00	up
0.00	0.00	up
0.00	0.00	up
0.00	0.00	up
0.00	0.00	down
0.00	0.00	down
0.00	0.00	up
0.00	0.00	up
0.00	0.00	down
0.00	0.00	up
0.00	0.00	down
0.00	0.01	up
0.00	0.01	down
0.00	0.01	up
0.00	0.01	down
0.01	0.03	down
0.00	0.00	up
0.00	0.00	up
0.00	0.00	up
0.00	0.00	up
0.00	0.00	up
0.01	0.03	up
0	0	up
0.00	0.00	down
0.00	0.00	up
0.00	0.00	down
0.00	0.00	up
0.00	0.00	up
0.00	0.00	up
0.02	0.05	up
0.00	0.00	down
0.00	0.00	down
0.00	0.00	down
0.00	0.00	down
0.00	0.00	down
0.00	0.00	up
0.00	0.00	up
0.00	0.00	up
0.00	0.00	up

0.00	0.00	up
0.00	0.00	down
0	0	up
0.00	0.00	up
0.00	0.00	down
0.00	0.00	up
0.00	0.00	down
0.00	0.00	up
0.00	0.00	up
0.00	0.00	up
0.00	0.00	down
0.00	0.00	down
0.00	0.00	up
0.00	0.01	up
0.01	0.03	up
0.01	0.03	up
0	0	up
0.00	0.00	up
0.00	0.00	up
0.00	0.00	up
0.00	0.00	up
0.00	0.00	up
0.00	0.00	up
0.00	0.00	up
0.00	0.00	up
0.00	0.00	up
0.00	0.00	up
0.00	0.00	down
0.00	0.00	down
0.00	0.00	up
0.00	0.00	down
0.00	0.00	up
0.00	0.00	up
0.00	0.00	down
0.00	0.00	up
0.00	0.00	down
0.00	0.00	up
0.00	0.00	up
0.00	0.00	up
0.00	0.00	up
0.00	0.00	up
0.00	0.00	up
0.00	0.00	up
0.00	0.00	up
0.00	0.01	up
0.00	0.01	down
0.01	0.03	down
0.01	0.03	up
0	0	up
0.00	0.00	down
0.00	0.00	up

0.00	0.00	down
0.00	0.00	up
0.00	0.00	up
0.00	0.00	up
0.02	0.05	up
0.00	0.00	up
0.00	0.00	down
0.00	0.00	down
0.00	0.00	up
0.00	0.00	up
0	0	up
0	0	up
0.00	0.00	up
0.00	0.00	down
0.00	0.00	up
0.00	0.00	down
0.00	0.00	up
0.00	0.00	up
0.00	0.00	up
0.00	0.00	up
0.00	0.00	down
0.00	0.00	up
0.00	0.00	up
0.00	0.00	up
0.00	0.00	up
0.00	0.00	up
0.00	0.00	down
0.00	0.00	down
0.00	0.00	up
0.00	0.00	down
0.00	0.00	up
0.00	0.00	up
0.00	0.00	up
0.00	0.00	down
0.00	0.00	up
0.00	0.00	down
0.00	0.00	up
0.00	0.00	down
0.00	0.00	up
0.00	0.00	down
0.00	0.00	up
0.00	0.00	up
0.00	0.00	up
0.00	0.00	up
0.00	0.00	up
0.00	0.00	up
0.00	0.00	up
0.00	0.00	up
0.00	0.00	up
0.00	0.00	up
0.00	0.00	up

0.00	0.00	up
0.00	0.00	up
0.00	0.00	up
0.00	0.01	up
0.00	0.01	down
0.01	0.02	up
0.01	0.02	down
0.01	0.03	down
0.01	0.03	down
0.01	0.03	up
0.00	0.00	up
0.00	0.00	up
0.00	0.00	up
0.00	0.00	up
0.00	0.00	down
0.00	0.00	down
0.00	0.00	up
0.00	0.00	up
0.00	0.00	up
0.00	0.00	up
0.00	0.00	up
0.00	0.00	down
0.00	0.00	down
0.00	0.02	up
0.01	0.02	down
0	0	down
0.00	0.00	up
0.00	0.00	down
0.00	0.00	down
0.01	0.02	down
0.00	0.00	up
0.00	0.00	down
0.00	0.01	down
0.00	0.01	down
0.01	0.02	down
0.00	0.00	down
0.00	0.00	down
0.00	0.00	down
0.00	0.00	up
0.00	0.00	up
0.00	0.00	down
0.00	0.00	down
0.00	0.00	up
0.00	0.00	up
0.00	0.00	down
0.00	0.00	up
0.00	0.00	down
0.00	0.00	up
0.00	0.00	up
0.00	0.00	up
0.00	0.00	up

0.00	0.00	up
0.00	0.00	up
0.00	0.01	down
0.00	0.01	up
0.01	0.04	up
0.02	0.05	up
0.00	0.00	up
0.00	0.00	up
0.00	0.00	down
0.00	0.00	down
0.00	0.00	down
0.00	0.00	up
0.00	0.00	up
0.00	0.00	up
0.00	0.00	down
0.00	0.00	up
0.00	0.00	up
0.00	0.00	up
0.00	0.00	up
0.00	0.00	up
0.00	0.00	up
0.00	0.00	up
0.00	0.00	up
0.00	0.00	down
0.00	0.00	down
0.00	0.00	down
0.00	0.00	up
0.00	0.00	up
0.00	0.00	up
0.00	0.00	up
0.00	0.00	down
0.00	0.00	up
0.00	0.00	up
0.00	0.00	up
0.00	0.00	down
0.00	0.00	up
0.00	0.00	down
0.00	0.00	down
0.00	0.00	down
0.00	0.01	up
0.00	0.01	down
0.00	0.01	up
0.00	0.01	up
0.00	0.02	up
0.01	0.02	down
0.02	0.05	up
0.00	0.00	down
0.00	0.00	up
0.00	0.00	up
0.00	0.00	up
0.00	0.00	up

0.00	0.00	up
0.00	0.00	up
0.00	0.00	up
0.00	0.00	up
0.00	0.00	down
0	0	up
0.00	0.00	up
0.00	0.00	up
0.00	0.00	up
0.00	0.00	down
0.00	0.00	down
0.00	0.00	up
0.00	0.00	down
0.00	0.00	down
0.00	0.00	up
0.00	0.00	up
0.01	0.02	up
0.01	0.03	down
0.00	0.00	up
0.00	0.00	up
0.00	0.00	up
0.00	0.00	up
0.00	0.00	up
0.00	0.00	up
0.00	0.00	up
0.00	0.00	down
0.00	0.00	up
0.00	0.00	up
0.00	0.00	down
0.00	0.00	up
0.00	0.00	up
0.00	0.00	up
0.00	0.00	down
0.00	0.00	up
0.00	0.00	down
0.00	0.00	up
0.00	0.00	up
0.00	0.00	down
0.00	0.00	down
0.00	0.00	down
0.00	0.00	down
0.00	0.01	down
0.01	0.02	up
0.01	0.02	down
0.01	0.03	down
0.01	0.03	down
0.01	0.03	down
0.02	0.05	down
0.00	0.00	down
0.00	0.00	down
0.00	0.00	down

0.00	0.00	up
0.00	0.00	up
0.00	0.00	up
0.00	0.01	up
0.01	0.02	up
0.00	0.00	down
0.00	0.00	down
0.00	0.00	down
0.00	0.00	down
0.00	0.00	down
0.00	0.00	down
0.00	0.00	down
0.00	0.00	down
0.00	0.01	down
0.01	0.03	down
0.00	0.00	up
0.00	0.00	up
0.00	0.00	up
0.00	0.00	up
0.00	0.00	up
0.00	0.00	up
0.00	0.00	up
0.00	0.00	up
0.00	0.00	down
0.00	0.00	up
0.00	0.00	up
0.00	0.00	up
0.00	0.00	up
0.00	0.00	down
0.00	0.00	up
0.00	0.00	up
0.00	0.00	down
0.00	0.00	down
0.00	0.00	down
0.00	0.00	down
0.00	0.00	down
0	0	up
0.00	0.00	up
0.00	0.00	up
0.00	0.00	up
0.00	0.00	up
0.00	0.00	up
0.00	0.00	up
0.00	0.00	up
0.00	0.00	down
0.00	0.00	up
0.00	0.00	up
0.00	0.00	up
0.02	0.05	down
0.00	0.00	up
0.00	0.00	up

0.00	0.00	up
0.00	0.00	up
0.00	0.00	up
0.00	0.00	down
0.00	0.00	up
0.00	0.00	up
0.00	0.00	up
0.00	0.00	up
0.00	0.00	up
0.00	0.00	up
0.00	0.00	up
0.00	0.00	down
0.00	0.00	up
0.00	0.00	down
0.00	0.00	up
0.00	0.00	down
0.01	0.02	down
0.02	0.05	down
0.00	0.00	down
0.00	0.00	down
0.00	0.00	down
0.00	0.01	up
0.00	0.01	down
0.00	0.00	up
0.00	0.00	down
0.00	0.00	down
0.00	0.00	up
0.00	0.01	down
0.00	0.00	up
0.00	0.00	up
0.00	0.00	down
0.00	0.00	up
0.00	0.00	down
0.00	0.00	up
0.00	0.00	down
0.00	0.00	down
0.00	0.00	down
0.00	0.00	down
0.00	0.00	down
0.00	0.00	down
0.00	0.00	up
0.00	0.00	down
0.00	0.00	up
0.00	0.00	down
0.00	0.00	up
0.00	0.00	up
0.00	0.00	down
0.00	0.00	up
0.00	0.00	up
0.00	0.00	down
0.00	0.00	up

0.00	0.00	up
0.00	0.00	up
0.00	0.01	up
0.00	0.01	up
0.00	0.01	up
0.00	0.01	down
0.00	0.01	up
0.00	0.01	up
0.00	0.01	up
0.00	0.01	down
0.00	0.01	down
0.00	0.01	up
0.00	0.02	down
0.01	0.02	down
0.01	0.02	down
0.01	0.03	down
0.02	0.04	up
0.02	0.05	down
0.02	0.05	down
0.02	0.05	down
0.02	0.05	up
0	0	up
0.00	0.00	up
0.00	0.00	up
0.00	0.00	up
0.00	0.00	up
0.00	0.00	up
0.00	0.00	down
0.00	0.00	down
0.00	0.00	down
0.00	0.00	down
0.00	0.00	up
0.00	0.00	down
0.00	0.00	down
0.00	0.01	down
0.01	0.03	down
0.00	0.00	down
0.00	0.00	down
0.00	0.00	down
0.00	0.01	up
0.00	0.01	down
0.00	0.00	down
0.00	0.00	down
0.00	0.00	down
0.00	0.01	up
0.00	0.01	down
0	0	up
0.00	0.00	up
0.00	0.00	up
0.00	0.00	up
0.00	0.00	down

0.00	0.00	down
0.00	0.00	up
0.00	0.00	up
0.00	0.00	up
0.00	0.00	up
0.00	0.00	up
0.00	0.00	up
0.00	0.00	up
0.00	0.00	up
0.00	0.00	down
0.00	0.00	up
0.00	0.00	down
0.00	0.00	up
0.00	0.00	down
0.00	0.00	down
0.00	0.00	up
0.00	0.00	up
0.00	0.00	down
0.00	0.00	down
0.00	0.00	down
0.00	0.00	up
0.00	0.00	down
0.00	0.00	up
0.00	0.00	down
0.00	0.00	up
0.00	0.00	down
0.00	0.00	up
0.00	0.00	up
0.00	0.00	down
0.00	0.00	up
0.00	0.00	down
0.00	0.00	up
0.00	0.00	up
0.00	0.00	up
0.00	0.00	up
0.00	0.00	up
0.00	0.00	up
0.00	0.00	up
0.00	0.00	up
0.00	0.00	up
0.00	0.00	down
0.00	0.00	down
0.00	0.00	up
0.00	0.00	up
0.00	0.00	up
0.00	0.00	up
0.00	0.00	up
0.00	0.00	down
0.00	0.00	up
0.00	0.00	up
0.00	0.00	up

0.00	0.00	up
0.00	0.00	up
0.00	0.00	up
0.00	0.00	up
0.00	0.00	up
0.00	0.00	up
0.00	0.00	up
0.00	0.00	up
0.00	0.01	down
0.00	0.01	up
0.00	0.01	up
0.01	0.02	up
0.01	0.02	down
0.01	0.02	up
0.01	0.04	up
0.02	0.05	down
0.00	0.00	up
0.00	0.00	up
0.00	0.00	up
0.00	0.00	up
0.00	0.00	up
0.00	0.00	up
0.00	0.00	up
0.00	0.00	up
0.00	0.00	up
0.00	0.00	up
0.00	0.00	up
0.00	0.00	up
0.00	0.00	up
0.00	0.00	up
0.00	0.00	up
0.00	0.00	down
0.00	0.00	down
0.00	0.00	down
0.00	0.00	down
0.00	0.00	down
0.00	0.00	up
0.00	0.00	up
0.00	0.00	up
0.00	0.00	down
0.00	0.00	up
0.00	0.00	down
0.00	0.00	up
0.00	0.00	up
0.00	0.00	up
0.02	0.05	down
0.00	0.00	down
0.00	0.00	up
0.00	0.00	down
0.00	0.00	up
0.00	0.00	down

0.00	0.00	up
0.00	0.00	up
0.00	0.00	up
0.00	0.00	up
0.00	0.00	up
0.00	0.00	up
0.00	0.00	up
0.00	0.00	down
0.00	0.00	down
0.00	0.00	down
0.00	0.00	down
0.00	0.00	down
0.00	0.00	down
0.00	0.00	down
0.00	0.00	down
0.00	0.01	down
0.00	0.01	down
0.01	0.03	down
0.01	0.03	down
0	0	up
0.00	0.00	down
0.00	0.00	up
0.00	0.00	down
0.00	0.00	up
0.00	0.00	up
0.00	0.00	up
0.02	0.05	up
0.00	0.00	up
0.00	0.00	up
0.00	0.00	down
0.00	0.00	up
0.00	0.00	up
0.00	0.00	up
0.00	0.00	up
0.00	0.00	up
0.00	0.00	down
0.00	0.00	up
0.00	0.00	up
0.00	0.01	up
0.00	0.00	up
0.00	0.00	down
0.00	0.01	down
0.00	0.00	up
0.00	0.00	up
0.00	0.00	up
0.00	0.00	up
0.00	0.00	down
0.00	0.01	down
0.01	0.03	down
0.00	0.00	up
0.00	0.00	up

0.00	0.00	up
0.00	0.00	up
0.00	0.00	down
0.00	0.01	down
0.01	0.03	down
0.00	0.00	up
0.00	0.00	down
0.00	0.00	down
0.00	0.00	down
0.00	0.00	down
0.00	0.00	down
0.00	0.00	up
0.00	0.00	up
0.00	0.00	up
0.00	0.00	up
0.00	0.00	up
0.00	0.00	down
0.00	0.00	up
0.00	0.00	up
0.00	0.00	up
0.00	0.00	down
0.00	0.00	up
0.00	0.00	up
0.00	0.01	down
0.00	0.00	up
0.00	0.00	up
0.00	0.00	up
0.00	0.00	up
0.00	0.00	up
0.00	0.00	up
0.00	0.00	up
0.00	0.00	up
0.00	0.00	up
0.00	0.00	up
0.00	0.00	up
0.00	0.00	up
0.00	0.00	up
0.00	0.00	up
0.00	0.00	up
0.00	0.00	down
0.00	0.00	down
0.00	0.00	up
0.00	0.01	down
0.00	0.00	up
0.00	0.00	up
0.00	0.00	down
0.00	0.00	up
0.00	0.00	up
0.02	0.05	down
0.00	0.00	down
0.00	0.00	up

0.00	0.00	up
0.00	0.00	up
0.00	0.00	down
0.00	0.00	up
0.00	0.00	up
0.00	0.00	up
0.00	0.00	up
0.00	0.00	up
0.00	0.00	down
0.00	0.00	up
0.00	0.00	up
0	0	up
0.00	0.00	down
0.00	0.00	down
0.00	0.00	down
0.00	0.00	up
0.00	0.00	down
0.00	0.00	up
0.00	0.00	down
0.00	0.00	up
0.00	0.00	up
0.00	0.00	down
0.00	0.01	up
0.00	0.01	up
0.01	0.03	down
0.02	0.04	up
0.00	0.00	down
0.00	0.00	down
0.00	0.00	up
0.00	0.00	up
0.00	0.01	up
0	0	down
0.00	0.00	up
0.00	0.00	up
0.00	0.00	down
0.00	0.00	up
0.00	0.00	up
0.00	0.00	up
0.00	0.00	down
0.00	0.00	up
0.00	0.00	up
0.00	0.00	down
0.00	0.00	down
0.00	0.00	down
0.00	0.00	down
0.00	0.00	down
0.00	0.00	up
0.00	0.00	up
0.00	0.00	down
0.00	0.00	up
0.00	0.00	down

0.00	0.00	up
0.00	0.00	up
0.00	0.00	down
0.00	0.00	down
0.00	0.00	down
0.00	0.00	up
0.00	0.00	down
0.00	0.00	up
0.00	0.00	down
0.00	0.00	down
0.00	0.00	up
0.00	0.00	up
0.00	0.00	up
0.00	0.00	down
0.00	0.00	up
0.00	0.00	up
0.00	0.00	up
0.00	0.00	up
0.00	0.00	down
0.00	0.00	down
0.00	0.00	up
0.00	0.00	up
0.00	0.00	up
0.00	0.00	down
0.00	0.00	down
0.00	0.00	down
0.00	0.00	down
0.00	0.00	up
0.00	0.00	up
0.00	0.00	down
0.00	0.00	down
0.00	0.00	up
0.00	0.00	down
0.00	0.00	up
0.00	0.00	up
0.00	0.00	down
0.00	0.00	up
0.00	0.00	up
0.00	0.00	down
0.00	0.00	down
0.00	0.00	up
0.00	0.00	down
0.00	0.00	up
0.00	0.00	up
0.00	0.00	down
0.00	0.00	down
0.00	0.00	down
0.00	0.00	down
0.00	0.00	up
0.00	0.00	down
0.00	0.00	up

0.00	0.00	up
0.00	0.00	up
0.00	0.00	down
0.00	0.00	up
0.00	0.00	down
0.00	0.00	up
0.00	0.00	up
0.00	0.00	up
0.00	0.00	up
0.00	0.00	up
0.00	0.00	down
0.00	0.00	up
0.00	0.00	down
0.00	0.00	up
0.00	0.00	down
0.00	0.00	up
0.00	0.00	up
0.00	0.00	down
0.00	0.00	down
0.00	0.00	up
0.00	0.00	down
0.00	0.00	up
0.00	0.00	up
0.00	0.00	down
0.00	0.00	down
0.00	0.00	up
0.00	0.00	up
0.00	0.00	up
0.00	0.00	up
0.00	0.00	up
0.00	0.00	up
0.00	0.00	up
0.00	0.00	up
0.00	0.01	up
0.00	0.01	up
0.00	0.01	down
0.00	0.01	up
0.00	0.01	down
0.00	0.01	up
0.00	0.01	down
0.00	0.01	up
0.00	0.01	up
0.00	0.01	down
0.00	0.01	down
0.00	0.01	up
0.00	0.01	down
0.00	0.02	down
0.01	0.02	down
0.01	0.02	down
0.01	0.03	up
0.01	0.03	down

0.01	0.03	up
0.02	0.04	up
0.02	0.04	up
0.02	0.05	down
0.02	0.05	down
0.02	0.05	up
0.00	0.00	down
0.00	0.00	down
0.00	0.00	down
0.00	0.00	down
0.00	0.00	up
0.00	0.00	down
0.00	0.00	down
0.00	0.00	up
0.00	0.01	down
0.00	0.00	down
0.00	0.00	down
0.00	0.00	down
0.00	0.00	up
0.00	0.00	down
0.00	0.00	up
0.00	0.00	down
0.00	0.00	up
0.00	0.00	up
0.00	0.01	up
0.00	0.01	down
0.01	0.02	up
0.00	0.00	up
0.00	0.00	up
0.00	0.00	up
0.00	0.00	up
0.00	0.00	up
0.00	0.00	down
0.00	0.00	down
0.00	0.00	up
0.00	0.00	down
0.00	0.00	up
0.00	0.00	up
0.00	0.00	down
0.01	0.02	up
0.00	0.00	down
0.00	0.00	down
0.00	0.00	up
0.00	0.00	down
0.00	0.00	down
0.00	0.00	down
0	0	up
0.00	0.00	up
0.00	0.00	up
0.00	0.00	up
0.00	0.00	down

0.00	0.00	up
0.00	0.00	up
0.00	0.00	up
0.00	0.00	up
0.00	0.00	down
0.00	0.00	down
0.00	0.00	down
0.00	0.00	down
0.00	0.01	down
0.01	0.02	up
0.01	0.03	down
0	0	up
0.00	0.00	up
0.00	0.00	up
0.00	0.00	up
0.00	0.00	up
0.00	0.00	up
0.00	0.01	up
0	0	up
0.00	0.00	up
0.00	0.01	down
0.02	0.04	up
0.00	0.00	down
0.00	0.00	up
0.00	0.00	up
0.00	0.01	up
0	0	up
0.00	0.00	up
0.00	0.00	up
0.00	0.00	down
0.00	0.00	down
0.00	0.00	down
0.00	0.00	up
0.00	0.00	down
0.00	0.00	up
0.00	0.00	down
0.00	0.00	up
0.01	0.03	up
0.00	0.00	up
0.00	0.00	up
0.00	0.00	up
0.00	0.00	up
0.00	0.00	down
0.00	0.00	up
0.00	0.00	down
0.00	0.00	up
0.00	0.00	up
0.00	0.00	up
0.00	0.00	up
0.00	0.01	up
0.00	0.01	up

0.00	0.01	down
0	0	up
0.00	0.00	up
0.00	0.00	down
0.00	0.00	up
0.00	0.00	up
0.00	0.01	up
0.00	0.00	up
0.00	0.00	up
0.00	0.00	down
0.00	0.00	up
0.00	0.00	up
0.00	0.00	up
0.00	0.01	up
0.01	0.03	up
0.00	0.00	down
0.00	0.00	up
0.00	0.01	up
0.00	0.00	down
0.00	0.00	down
0.00	0.01	down
0	0	down
0.00	0.00	up
0.00	0.00	up
0.00	0.00	down
0.00	0.00	down
0.00	0.00	up
0.00	0.00	up
0.00	0.00	up
0.00	0.00	up
0.00	0.00	up
0.00	0.00	down
0.00	0.00	up
0.00	0.00	down
0.00	0.01	down
0.00	0.01	down
0.01	0.02	down
0.01	0.02	down
0.01	0.03	up
0.01	0.03	up
0.00	0.00	up
0.00	0.00	up
0.00	0.00	down
0.00	0.00	up
0.00	0.00	down
0.00	0.00	up
0.00	0.00	up
0.00	0.00	down
0.00	0.00	up
0.00	0.00	down
0.00	0.00	down

0.00	0.00	down
0.00	0.00	down
0.00	0.00	up
0.00	0.00	up
0.00	0.00	down
0	0	up
0	0	up
0	0	down
0.00	0.00	down
0.00	0.00	up
0.00	0.00	up
0.00	0.00	up
0.00	0.00	up
0.00	0.00	up
0.00	0.00	up
0.00	0.00	up
0.00	0.00	up
0.00	0.00	down
0.00	0.00	up
0.00	0.00	up
0.00	0.00	up
0.00	0.00	down
0.00	0.00	down
0.00	0.00	down
0.00	0.00	down
0.02	0.05	up
0.00	0.00	up
0.00	0.00	down
0.00	0.00	down
0.00	0.00	up
0.00	0.00	up
0.00	0.00	down
0.00	0.00	down
0.00	0.00	down
0	0	up
0.00	0.00	up
0.00	0.00	up
0.00	0.00	up
0.00	0.00	up
0.00	0.00	up
0.00	0.00	up
0.00	0.00	up
0.00	0.00	down
0.00	0.00	up
0.00	0.00	down
0.01	0.02	up
0.00	0.00	up
0.00	0.00	up
0.00	0.00	up
0.00	0.00	down
0.00	0.00	up

0.00	0.00	up
0.00	0.00	up
0.00	0.00	up
0.00	0.00	up
0.00	0.00	up
0.00	0.00	up
0.00	0.00	up
0.00	0.00	up
0.00	0.00	up
0.00	0.00	down
0.00	0.00	down
0.00	0.00	down
0.00	0.00	up
0.00	0.00	up
0.00	0.00	up
0.00	0.00	down
0.00	0.00	down
0.00	0.00	up
0.00	0.00	up
0.00	0.00	up
0.00	0.00	up
0.00	0.00	up
0.00	0.00	down
0.00	0.00	down
0.00	0.00	up
0.00	0.00	down
0.00	0.00	up
0.00	0.00	down
0.00	0.00	up
0.00	0.00	down
0.00	0.00	down
0.00	0.00	up
0.00	0.00	up
0.00	0.00	up
0.00	0.00	up
0.00	0.00	down
0.00	0.00	up
0.00	0.00	down
0.00	0.00	up
0.00	0.00	down
0.00	0.00	down
0.00	0.00	up
0.00	0.00	down
0.00	0.00	down
0.00	0.00	up
0.00	0.00	down
0.00	0.00	up
0.00	0.00	down
0.00	0.00	up
0.00	0.00	up
0.00	0.00	down

0.00	0.01	up
0.00	0.01	up
0.00	0.01	up
0.00	0.01	up
0.00	0.01	up
0.01	0.02	up
0.01	0.02	up
0.01	0.02	up
0.01	0.02	down
0.01	0.02	up
0.01	0.02	down
0.01	0.02	down
0.01	0.03	up
0.01	0.03	down
0.01	0.04	down
0.02	0.04	up
0.00	0.00	up
0.00	0.00	up
0.00	0.00	up
0.00	0.00	up
0.00	0.00	up
0.00	0.00	down
0.00	0.00	up
0.00	0.00	down
0.00	0.00	up
0.00	0.00	down
0.00	0.00	up
0.00	0.00	up
0.00	0.00	down
0.00	0.00	up
0.00	0.00	up
0.00	0.00	up
0.00	0.00	down
0.00	0.00	up
0.00	0.00	up
0.00	0.00	down
0.00	0.00	up
0.00	0.00	up
0.00	0.00	up
0.00	0.00	down
0.00	0.00	up
0.00	0.00	up
0.00	0.00	up
0.00	0.00	down
0.00	0.00	up
0.00	0.01	up
0.01	0.02	down
0.01	0.02	down
0.01	0.03	down
0.01	0.04	up
0.00	0.00	up

0.00	0.00	up
0.00	0.00	up
0.00	0.00	down
0.00	0.01	down
0.01	0.03	down
0.00	0.00	up
0.00	0.00	up
0.00	0.00	down
0.00	0.00	down
0.00	0.00	down
0.00	0.00	down
0.00	0.00	down
0.00	0.00	up
0.01	0.02	up
0.01	0.02	down
0.01	0.03	down
0.01	0.03	down
0.01	0.03	down
0.00	0.00	up
0.00	0.00	up
0.00	0.00	up
0.00	0.00	up
0.00	0.00	up
0.00	0.00	up
0.00	0.00	down
0.00	0.00	up
0	0	up
0.00	0.00	up
0.00	0.00	up
0.00	0.00	up
0.00	0.00	down
0.00	0.00	up
0.00	0.00	up
0.00	0.00	down
0.00	0.00	up
0.00	0.00	down
0.00	0.00	up
0.00	0.00	up
0.00	0.00	down
0.01	0.02	up
0.01	0.02	down
0.00	0.00	down
0.00	0.00	down
0.00	0.00	up
0.00	0.00	up
0.00	0.01	up
0.02	0.05	up
0	0	up
0.00	0.00	up
0.00	0.00	up
0.00	0.00	down

0.00	0.00	up
0.00	0.00	up
0.00	0.00	up
0.00	0.00	up
0.00	0.00	up
0.00	0.00	up
0.00	0.00	up
0.00	0.00	up
0.00	0.00	up
0.00	0.00	up
0.00	0.00	up
0.00	0.00	up
0.00	0.00	down
0.00	0.00	down
0.00	0.00	up
0.00	0.00	up
0.00	0.00	down
0.00	0.00	up
0.00	0.00	down
0.00	0.00	down
0.00	0.00	down
0.00	0.00	up
0.00	0.00	up
0.00	0.00	up
0.00	0.00	down
0.00	0.00	down
0.00	0.00	down
0.00	0.00	down
0.00	0.00	up
0.00	0.00	down
0.00	0.00	up
0.00	0.00	down
0.00	0.00	up
0.00	0.00	up
0.00	0.00	up
0.00	0.00	up
0.00	0.00	up
0.00	0.00	down
0.00	0.00	up
0.00	0.00	up
0.00	0.00	up
0.00	0.01	down
0.01	0.03	up
0.00	0.00	up
0.00	0.00	up
0.00	0.00	up
0.00	0.00	up
0.00	0.01	up
0.00	0.00	down
0.00	0.00	down
0.00	0.00	down

0.00	0.01	down
0.00	0.02	down
0	0	down
0.00	0.00	down
0.00	0.00	down
0.00	0.00	up
0.00	0.00	up
0.01	0.02	up
0.00	0.00	up
0.00	0.00	up
0.00	0.01	up
0.00	0.00	up
0.00	0.00	up
0.00	0.00	up
0.00	0.00	down
0.00	0.00	up
0.00	0.01	up
0.00	0.00	down
0.00	0.00	up
0.00	0.01	up
0.00	0.00	up
0.00	0.00	down
0.00	0.00	down
0.00	0.00	down
0.00	0.00	down
0.00	0.00	down
0.00	0.00	down
0.00	0.00	down
0.00	0.00	up
0.00	0.01	down
0.01	0.03	down
0.00	0.00	up
0.00	0.00	up
0.00	0.00	up
0.00	0.00	up
0.00	0.00	up
0.00	0.00	up
0.00	0.01	up
0	0	down
0.00	0.00	up
0.00	0.00	up
0.00	0.00	down
0.00	0.00	down
0.00	0.00	up
0.00	0.00	down
0.00	0.00	up
0.00	0.00	up
0.00	0.00	up
0.00	0.00	down
0.00	0.00	down
0.00	0.00	down

0.00	0.00	up
0.00	0.00	up
0.00	0.00	down
0.00	0.00	up
0.00	0.00	down
0.00	0.00	up
0.00	0.00	down
0.00	0.00	up
0.00	0.00	down
0.00	0.00	down
0.00	0.00	up
0.00	0.00	down
0.00	0.00	down
0.00	0.00	up
0.00	0.00	up
0.00	0.00	up
0.00	0.00	down
0.00	0.00	down
0.00	0.00	up
0.00	0.00	up
0.00	0.00	up
0.00	0.01	up
0.00	0.01	down
0.00	0.01	down
0.00	0.01	up
0.00	0.01	up
0.00	0.01	up
0.01	0.02	up
0.01	0.02	down
0.01	0.02	down
0.01	0.02	up
0.01	0.04	down
0.02	0.05	down
0.00	0.00	down
0.00	0.00	down
0.00	0.00	up
0.00	0.00	down
0.00	0.01	up
0.00	0.02	down
0.00	0.00	up
0.00	0.00	down
0.00	0.00	down
0.00	0.00	down
0.00	0.00	up
0.00	0.00	down
0.00	0.00	up
0.01	0.02	up
0.01	0.02	down
0.00	0.00	up
0.00	0.00	up
0.00	0.00	down

0.00	0.00	up
0.00	0.00	up
0.00	0.00	up
0.00	0.00	up
0.00	0.00	up
0.00	0.00	up
0.00	0.00	up
0.00	0.00	up
0.00	0.00	up
0.00	0.00	down
0.00	0.01	down
0.01	0.03	down
0.00	0.00	up
0.00	0.00	up
0.00	0.00	up
0.00	0.00	up
0.00	0.00	down
0.00	0.00	down
0.00	0.00	down
0.00	0.00	up
0.00	0.00	down
0.00	0.00	up
0.00	0.00	down
0.00	0.00	up
0.00	0.00	up
0.00	0.00	up
0.00	0.00	up
0.00	0.00	down
0.00	0.00	down
0.00	0.00	up
0.00	0.00	up
0.00	0.00	up
0.00	0.00	down
0.00	0.01	up
0.00	0.01	up
0.00	0.02	down
0.01	0.03	down
0.01	0.03	up
0.00	0.00	down
0.00	0.00	up
0.00	0.00	up
0.00	0.00	up
0.00	0.00	up
0.01	0.03	up
0.00	0.00	down
0.00	0.00	down
0.00	0.00	down
0.00	0.00	up
0.00	0.00	up
0.00	0.01	down
0.00	0.00	down

0.00	0.00	up
0.00	0.00	down
0.00	0.00	up
0.00	0.00	up
0.00	0.00	up
0.00	0.00	up
0.00	0.00	up
0.00	0.00	up
0.00	0.00	up
0.00	0.00	up
0.00	0.01	up
0.02	0.04	down
0.00	0.00	up
0.00	0.00	down
0.00	0.01	down
0.00	0.01	down
0.01	0.02	down
0.00	0.00	down
0.00	0.00	down
0.00	0.00	down
0.00	0.00	up
0.00	0.00	up
0.00	0.01	up
0.00	0.00	up
0.00	0.00	up
0.00	0.00	up
0.00	0.00	down
0.00	0.00	up
0.00	0.00	up
0.00	0.00	up
0.00	0.00	down
0.00	0.00	up
0.00	0.00	up
0.02	0.05	up
0	0	up
0.00	0.00	up
0.00	0.00	up
0.01	0.04	down
0.00	0.00	down
0.00	0.00	up
0.00	0.00	down
0.00	0.00	up
0.00	0.00	down
0.00	0.00	up
0.00	0.00	down
0.00	0.00	up
0.00	0.00	up
0.00	0.00	up
0.00	0.00	down
0.00	0.00	down
0.00	0.00	down

0.00	0.00	up
0.00	0.00	down
0.00	0.00	down
0.00	0.00	down
0.00	0.00	up
0.00	0.01	down
0.00	0.01	down
0.00	0.01	down
0.00	0.00	up
0.00	0.00	up
0.00	0.00	down
0.00	0.00	down
0.00	0.00	up
0.00	0.01	up
0.01	0.02	up
0.00	0.00	down
0.00	0.00	up
0.00	0.00	down
0.00	0.00	down
0.00	0.00	down
0.00	0.00	up
0.00	0.01	down
0.00	0.01	down
0.00	0.01	down
0.00	0.00	down
0.00	0.00	down
0.00	0.00	down
0.00	0.00	down
0.00	0.00	down
0.01	0.02	up
0.01	0.03	down
0.00	0.00	down
0.00	0.00	up
0.00	0.00	up
0.00	0.00	down
0.00	0.00	down
0.00	0.00	down
0.00	0.00	down
0.00	0.00	down
0.00	0.00	down
0.00	0.01	down
0.00	0.01	down
0.01	0.03	down
0.00	0.00	up
0.00	0.00	up
0.00	0.00	up
0.00	0.00	up
0.00	0.00	down
0.00	0.00	down
0.00	0.00	up
0.00	0.00	down

0.00	0.00	down
0.00	0.00	down
0.00	0.00	down
0.00	0.00	up
0.00	0.01	down
0.00	0.01	up
0.01	0.02	down
0.01	0.02	down
0.01	0.03	up
0	0	down
0.00	0.00	up
0.00	0.00	down
0.00	0.00	down
0.00	0.00	down
0.01	0.02	up
0.00	0.00	up
0.00	0.00	up
0.00	0.00	up
0.01	0.02	up
0.00	0.00	up
0.00	0.00	up
0.00	0.01	up
0.01	0.03	up
0.00	0.00	up
0.00	0.00	down
0.00	0.01	down
0.00	0.00	up
0.00	0.00	up
0.00	0.00	up
0.00	0.00	up
0.00	0.00	up
0.01	0.02	down
0.00	0.00	up
0.00	0.00	down
0.00	0.00	down
0.00	0.00	up
0.00	0.00	up
0.00	0.00	down
0.00	0.00	down
0.00	0.00	down
0.00	0.00	up
0.00	0.00	up
0.00	0.00	up
0.00	0.00	up
0.00	0.00	up
0.00	0.02	down
0	0	up
0.00	0.00	up
0	0	up
0.00	0.00	up
0	0	up

0.01	0.02	down
0.00	0.00	up
0.00	0.00	up
0.00	0.00	up
0.00	0.00	down
0.00	0.00	up
0.00	0.00	down
0.00	0.00	up
0.00	0.01	down
0.00	0.00	up
0.00	0.00	up
0.00	0.00	up
0.00	0.00	up
0.00	0.00	down
0.00	0.00	down
0.00	0.00	up
0.00	0.00	up
0.00	0.00	up
0.00	0.00	up
0.00	0.00	up
0.00	0.00	up
0.00	0.00	up
0.00	0.00	down
0.00	0.00	down
0.00	0.00	down
0.01	0.02	up
0.01	0.03	down
0.00	0.00	down
0.00	0.00	down
0.00	0.00	down
0.00	0.00	up
0	0	down
0	0	down
0	0	up
0.00	0.00	down
0.00	0.00	up
0.00	0.00	up
0.00	0.00	down
0.00	0.00	up
0.00	0.00	up
0.00	0.00	up
0.00	0.00	up
0.00	0.00	up
0.00	0.00	up
0.00	0.00	down
0.00	0.00	up
0.00	0.00	up
0.00	0.00	up
0.00	0.00	up
0.00	0.00	up
0.00	0.00	up

0.00	0.00	up
0.00	0.00	down
0.00	0.00	down
0.00	0.00	up
0.00	0.00	down
0.00	0.00	down
0.00	0.00	down
0.00	0.00	up
0.00	0.00	down
0.00	0.00	up
0.00	0.00	up
0.00	0.00	up
0.00	0.00	up
0.00	0.00	up
0.00	0.00	up
0.00	0.00	down
0.00	0.00	up
0.00	0.00	down
0.00	0.00	down
0.00	0.00	down
0.00	0.00	down
0.00	0.00	up
0.00	0.00	up
0.00	0.00	up
0.00	0.00	up
0.00	0.00	up
0.00	0.00	up
0.00	0.00	up
0.00	0.00	up
0.00	0.00	down
0.00	0.00	up
0.00	0.00	up
0.00	0.00	up
0.00	0.00	up
0.00	0.00	up
0.00	0.00	up
0.00	0.00	up
0.00	0.00	down
0.00	0.00	down
0.00	0.00	up
0.00	0.00	down
0.00	0.00	up
0.00	0.00	up
0.00	0.00	up
0.00	0.00	up
0.00	0.00	up
0.00	0.00	up
0.00	0.00	up
0.00	0.00	up
0.00	0.00	up
0.00	0.00	down

0.00	0.00	down
0.00	0.00	down
0.00	0.00	down
0.00	0.00	down
0.00	0.00	up
0.00	0.00	down
0.00	0.00	up
0.00	0.00	up
0.00	0.00	up
0.00	0.00	up
0.00	0.00	up
0.00	0.00	up
0.00	0.00	up
0.00	0.00	up
0.00	0.00	down
0.00	0.01	up
0.00	0.01	up
0.01	0.02	up
0.01	0.02	up
0.01	0.02	up
0.01	0.02	up
0.01	0.02	up
0.01	0.02	up
0.01	0.02	up
0.02	0.04	up
0	0	up
0.00	0.00	up
0.00	0.00	up
0.00	0.00	up
0.00	0.00	up
0.00	0.00	up
0.00	0.00	up
0.00	0.00	down
0.00	0.00	down
0.00	0.00	down
0.00	0.00	down
0.02	0.05	up
0.00	0.00	up
0.00	0.00	up
0.00	0.00	down
0.00	0.00	down
0.00	0.00	down
0.00	0.00	up
0.00	0.00	down
0.00	0.00	up
0.00	0.00	up
0.00	0.00	up
0.00	0.00	down
0.00	0.00	down
0.00	0.00	down
0.00	0.00	up

0.00	0.00	up
0.00	0.00	up
0.00	0.00	up
0.00	0.00	down
0.00	0.00	down
0.00	0.00	down
0.00	0.00	down
0.00	0.00	up
0.00	0.00	down
0.00	0.00	down
0.00	0.00	down
0.00	0.00	down
0.00	0.00	down
0.00	0.00	up
0.00	0.00	up
0.00	0.00	up
0.00	0.00	down
0.00	0.00	up
0.00	0.00	down
0.00	0.00	down
0.00	0.00	up
0.00	0.00	down
0.00	0.00	up
0.00	0.00	up
0.00	0.00	down
0.00	0.00	down
0.00	0.00	up
0.00	0.00	down
0.00	0.00	up
0.00	0.00	up
0.00	0.00	down
0.00	0.00	down
0.00	0.00	down
0.00	0.00	down
0.00	0.00	up
0.00	0.00	down
0.00	0.00	down
0.00	0.00	up
0.00	0.00	down
0.00	0.00	down
0.00	0.00	down
0.00	0.00	up
0.00	0.00	up
0.00	0.00	down
0.00	0.00	up
0.00	0.00	up
0.00	0.00	down
0.00	0.00	down
0.00	0.00	up
0.00	0.00	up
0.00	0.00	down

0.00	0.00	down
0.00	0.00	up
0.00	0.00	down
0.00	0.01	up
0.00	0.01	up
0.00	0.01	down
0.00	0.01	up
0.00	0.01	down
0.00	0.01	up
0.00	0.01	up
0.00	0.01	down
0.00	0.02	down
0.01	0.02	up
0.01	0.02	up
0.01	0.02	up
0.01	0.02	down
0.01	0.02	down
0.01	0.02	up
0.01	0.03	up
0.01	0.03	down
0.01	0.03	up
0.01	0.03	up
0.01	0.03	down
0.01	0.03	down
0.01	0.04	up
0.01	0.04	up
0.02	0.04	up
0.02	0.04	down
0.02	0.05	down
0	0	up
0.00	0.00	up
0.00	0.00	down
0.00	0.00	down
0.00	0.00	up
0.00	0.00	up
0.00	0.00	up
0.00	0.00	up
0.00	0.00	down
0.00	0.00	up
0.00	0.00	down
0.00	0.00	up
0.00	0.00	down
0.00	0.00	down
0.00	0.00	down
0.00	0.00	up
0.00	0.00	down
0.00	0.00	down
0.00	0.00	down
0.00	0.00	down
0.00	0.00	down
0.00	0.00	down

0.00	0.00	down
0.00	0.00	up
0.00	0.00	down
0.00	0.00	down
0.00	0.00	up
0.00	0.00	up
0.00	0.00	down
0.00	0.00	up
0.00	0.00	down
0.00	0.00	up
0.00	0.00	down
0.00	0.00	up
0.00	0.00	down
0.00	0.00	down
0.00	0.00	down
0.00	0.00	down
0.00	0.00	up
0.00	0.00	down
0.00	0.00	down
0.00	0.00	up
0.00	0.00	down
0.00	0.00	up
0.00	0.00	up
0.00	0.00	down
0.00	0.00	up
0.00	0.00	down
0.00	0.00	down
0.00	0.00	up
0.00	0.00	down
0.00	0.00	up
0.00	0.00	down
0.00	0.00	down
0.00	0.00	up
0.00	0.00	up
0.00	0.00	up
0.00	0.00	up
0.00	0.00	up
0.00	0.00	up
0.00	0.01	up
0.00	0.01	down
0.00	0.01	up
0.00	0.01	up
0.00	0.01	down
0.00	0.01	up
0.00	0.01	down
0.00	0.01	down
0.00	0.02	down
0.01	0.02	down
0.01	0.02	down
0.01	0.02	down
0.02	0.04	up

0.02	0.04	up
0.02	0.04	up
0.02	0.04	up
0.02	0.05	down
0.02	0.05	up
0.00	0.00	down
0.00	0.00	up
0.00	0.00	down
0.00	0.00	up
0.00	0.00	up
0.00	0.00	down
0.00	0.00	down
0.00	0.00	down
0.00	0.00	up
0.00	0.00	down
0.00	0.01	down
0.00	0.01	down
0.00	0.01	down
0.01	0.04	up
0.00	0.00	down
0.00	0.00	up
0.00	0.01	up
0.01	0.03	down
0.02	0.04	up
0.00	0.00	down
0.00	0.01	up
0.00	0.00	up
0.00	0.00	up
0.00	0.00	down
0.00	0.00	up
0.00	0.00	up
0.00	0.00	up
0.00	0.00	up
0.00	0.00	up
0.00	0.00	up
0.00	0.00	up
0.00	0.00	up
0.00	0.00	up
0.00	0.00	up
0.01	0.02	up
0.00	0.00	up
0.00	0.00	down
0.00	0.00	up
0.00	0.00	up
0.00	0.00	up
0.00	0.00	down
0.00	0.00	up
0.00	0.01	up
0.00	0.00	up
0.00	0.00	up
0.00	0.00	down

0.00	0.00	up
0.00	0.00	down
0.01	0.02	down
0.00	0.00	down
0.00	0.00	up
0.00	0.00	down
0.00	0.00	up
0.01	0.02	down
0.00	0.00	down
0.00	0.00	up
0.00	0.00	up
0.00	0.00	up
0.00	0.02	down
0.00	0.00	down
0.00	0.00	up
0.00	0.00	up
0.00	0.00	up
0.00	0.02	down
0.00	0.00	down
0.00	0.00	down
0.01	0.02	up
0.01	0.03	down
0.00	0.00	up
0.00	0.00	up
0.00	0.00	down
0.00	0.00	up
0.00	0.00	up
0.00	0.00	up
0.00	0.00	up
0.00	0.00	up
0.00	0.00	down
0.00	0.01	down
0.00	0.00	up
0.00	0.00	up
0.00	0.00	up
0.00	0.00	up
0.00	0.00	up
0.00	0.00	up
0.00	0.00	up
0.00	0.00	up
0.00	0.00	up
0.00	0.00	up
0.00	0.00	up
0.00	0.01	up
0.00	0.00	down
0.00	0.00	down
0.00	0.00	up
0.00	0.00	down
0.00	0.00	up
0.00	0.00	down
0.00	0.01	down

0.01	0.03	down
0.02	0.05	down
0	0	down
0.00	0.00	down
0.00	0.00	down
0.00	0.00	up
0.00	0.00	up
0.00	0.00	up
0.00	0.00	up
0.00	0.02	down
0	0	up
0.00	0.00	up
0	0	up
0.00	0.00	up
0.00	0.00	up
0.00	0.00	down
0.00	0.00	up
0.00	0.00	up
0.00	0.00	down
0.00	0.00	up
0.00	0.00	up
0.00	0.01	up
0.00	0.00	up
0.00	0.00	up
0.00	0.00	down
0.00	0.00	up
0.00	0.00	down
0.00	0.00	down
0.00	0.00	down
0.00	0.00	up
0.00	0.00	up
0.00	0.00	up
0.00	0.00	up
0.00	0.00	up
0.00	0.00	up
0.00	0.01	up
0.00	0.00	up
0.01	0.03	up
0.00	0.00	down
0.00	0.00	down
0.00	0.00	down
0.00	0.00	down
0.00	0.00	down
0.00	0.01	down
0.00	0.00	up
0.00	0.02	down
0	0	up
0.00	0.00	up
0.00	0.00	up
0.00	0.00	up
0.00	0.00	down

0.00	0.00	down
0.00	0.00	down
0.00	0.01	down
0.00	0.00	down
0.00	0.00	up
0.00	0.00	down
0.00	0.00	up
0.00	0.01	up
0.00	0.00	up
0.00	0.00	down
0.00	0.00	down
0.00	0.00	up
0.00	0.00	up
0.00	0.00	up
0.00	0.00	up
0.00	0.00	up
0.00	0.00	up
0.00	0.00	up
0.00	0.00	up
0.00	0.00	up
0.00	0.00	up
0.00	0.00	up
0.00	0.00	down
0.00	0.00	down
0.00	0.00	up
0.00	0.01	up
0.00	0.00	up
0.00	0.00	up
0.00	0.00	up
0.00	0.00	up
0.00	0.00	down
0.00	0.00	down
0.00	0.00	up
0.00	0.00	up
0.00	0.00	up
0.00	0.00	down
0.00	0.00	down
0.00	0.00	up
0.00	0.00	down
0.00	0.00	up
0.00	0.00	up
0.00	0.00	down
0.00	0.00	up
0.00	0.00	up
0.00	0.00	up
0.00	0.00	down
0.00	0.00	up
0.00	0.00	up
0.00	0.00	down
0.00	0.00	down
0.00	0.00	up

0.00	0.01	up
0.00	0.00	up
0.00	0.00	up
0.00	0.00	down
0.00	0.00	up
0.00	0.00	up
0.00	0.00	down
0.00	0.00	down
0.00	0.00	down
0.00	0.00	up
0.00	0.01	down
0.00	0.01	down
0.01	0.02	down
0.00	0.00	down
0.00	0.00	up
0.00	0.00	up
0.00	0.00	down
0.00	0.00	up
0.00	0.00	up
0.00	0.00	down
0.00	0.00	down
0.00	0.00	up
0.00	0.00	up
0.00	0.00	up
0.00	0.00	up
0.00	0.00	down
0.00	0.00	down
0.00	0.00	down
0.00	0.00	up
0.00	0.01	up
0.00	0.00	up
0.00	0.00	down
0.00	0.00	down
0.00	0.01	up
0	0	up
0	0	down
0.00	0.00	up
0.00	0.00	up
0.00	0.00	up
0.00	0.00	up
0.00	0.00	down
0.00	0.00	down
0.00	0.00	up
0.00	0.00	up
0.00	0.00	up
0.00	0.00	up
0.00	0.00	up
0.00	0.00	up
0.00	0.00	up
0.00	0.00	up
0.00	0.00	up

0.00	0.00	up
0.00	0.00	down
0.00	0.00	down
0.00	0.00	up
0.00	0.00	down
0.00	0.00	down
0.00	0.00	up
0.00	0.00	up
0.00	0.00	up
0.00	0.00	up
0.00	0.00	down
0.00	0.00	up
0.00	0.00	up
0.00	0.00	up
0.00	0.00	down
0.00	0.00	up
0.00	0.00	up
0.00	0.00	down
0.00	0.00	down
0.00	0.00	down
0.00	0.00	up
0.00	0.00	up
0.00	0.00	up
0.00	0.00	down
0.00	0.00	down
0.00	0.00	down
0.00	0.00	up
0.00	0.00	up
0.00	0.00	up
0.00	0.00	up
0.00	0.00	up
0.00	0.00	up
0.00	0.00	up
0.00	0.00	up
0.00	0.00	up
0.00	0.00	up
0.00	0.00	up
0.00	0.00	up
0.00	0.00	down
0.00	0.00	up
0.00	0.00	up
0.00	0.00	down
0.00	0.00	down
0.00	0.00	down
0.00	0.00	down
0.00	0.00	up
0.00	0.00	down
0.00	0.00	up
0.00	0.00	up
0.00	0.00	down
0.00	0.00	up

0.00	0.00	down
0.00	0.00	down
0.00	0.00	up
0.00	0.00	down
0.00	0.00	up
0.00	0.00	down
0.00	0.00	up
0.00	0.00	up
0.00	0.00	up
0.00	0.00	up
0.00	0.00	up
0.00	0.00	up
0.00	0.00	up
0.00	0.00	down
0.00	0.00	down
0.00	0.00	down
0.00	0.01	up
0.00	0.01	up
0.00	0.01	down
0.00	0.01	down
0.00	0.01	up
0.00	0.02	down
0.01	0.02	up
0.01	0.02	down
0.01	0.02	down
0.01	0.02	down
0.01	0.02	up
0.01	0.03	up
0.01	0.03	down
0.01	0.03	down
0.01	0.03	down
0.01	0.03	down
0.02	0.04	up
0.02	0.04	down
0.00	0.00	down
0.00	0.00	down
0.00	0.00	down
0.00	0.00	down
0.00	0.00	up
0.00	0.00	up
0.00	0.00	down
0.00	0.00	up
0.00	0.01	down
0.02	0.05	up
0.00	0.00	up
0.00	0.00	up
0.00	0.00	down
0.00	0.00	down
0.00	0.00	down
0.00	0.00	up
0.00	0.00	down

0.00	0.00	up
0.00	0.00	up
0.00	0.00	up
0.00	0.00	down
0.00	0.00	down
0.00	0.00	down
0.00	0.00	up
0.00	0.00	up
0.00	0.00	up
0.00	0.00	up
0.00	0.00	down
0.00	0.00	down
0.00	0.00	down
0.00	0.00	down
0.00	0.00	down
0.00	0.00	down
0.00	0.00	down
0.00	0.00	down
0.00	0.00	up
0.00	0.00	up
0.00	0.00	up
0.00	0.00	down
0.00	0.00	up
0.00	0.00	down
0.00	0.00	down
0.00	0.00	up
0.00	0.00	up
0.00	0.00	up
0.00	0.00	down
0.00	0.00	down
0.00	0.00	down
0.00	0.00	up
0.00	0.00	up
0.00	0.00	down
0.00	0.00	down
0.00	0.00	down
0.00	0.00	down
0.00	0.00	up
0.00	0.00	down
0.00	0.00	down
0.00	0.00	up
0.00	0.00	down
0.00	0.00	down
0.00	0.00	down
0.00	0.00	up
0.00	0.00	up
0.00	0.00	down
0.00	0.00	up
0.00	0.00	up
0.00	0.00	down
0.00	0.00	down

0.00	0.00 up
0.00	0.00 up
0.00	0.00 down
0.00	0.00 down
0.00	0.00 up
0.00	0.00 down
0.00	0.01 up
0.00	0.01 up
0.00	0.01 down
0.00	0.01 up
0.00	0.01 down
0.00	0.01 up
0.00	0.01 down
0.00	0.02 down
0.01	0.02 up
0.01	0.02 up
0.01	0.02 up
0.01	0.02 down
0.01	0.02 down
0.01	0.02 up
0.01	0.03 up
0.01	0.03 down
0.01	0.03 down
0.01	0.03 up
0.01	0.03 up
0.01	0.03 down
0.01	0.03 down
0.01	0.04 up
0.01	0.04 up
0.02	0.04 up
0.02	0.04 down
0.02	0.05 down
0.00	0.00 up
0.00	0.00 up
0.00	0.00 down
0.00	0.00 up
0.00	0.01 down
0.00	0.00 up
0.00	0.00 up
0.00	0.00 down
0.00	0.00 up
0.00	0.00 up
0.00	0.00 down
0.00	0.00 up
0.00	0.00 up
0.00	0.00 down
0.00	0.00 up
0.00	0.00 up
0.00	0.00 down
0.00	0.00 up
0.00	0.00 up

0.00	0.00	down
0.00	0.00	up
0.00	0.00	up
0.00	0.00	down
0.00	0.00	up
0.00	0.00	up
0.00	0.01	down
0.00	0.00	up
0.00	0.00	down
0.00	0.00	down
0.00	0.01	down
0.00	0.01	down
0	0	up
0.00	0.00	up
0.00	0.00	up
0.00	0.00	up
0.00	0.00	up
0.00	0.00	up
0.00	0.00	down
0.01	0.02	up
0.00	0.00	up
0.01	0.02	down
0.00	0.00	down
0.00	0.00	up
0.00	0.00	down
0.01	0.02	up
0.00	0.00	up
0.00	0.00	down
0.00	0.00	up
0.00	0.00	up
0.00	0.00	down
0.00	0.00	up
0.00	0.00	down
0.00	0.00	down
0.00	0.00	up
0.00	0.00	down
0.00	0.00	up
0.00	0.00	down
0.00	0.01	up
0.02	0.04	up
0.00	0.00	up
0.00	0.00	down
0.00	0.00	up
0.00	0.00	up
0.00	0.00	down
0.01	0.02	up
0.00	0.00	down
0.00	0.00	up
0.00	0.00	up
0.00	0.01	down
0.00	0.00	down

0.00	0.00	up
0.00	0.00	down
0.00	0.00	down
0.00	0.00	down
0.00	0.00	down
0.00	0.00	down
0.00	0.00	up
0.00	0.00	down
0.00	0.01	down
0.00	0.00	down
0.00	0.00	down
0.00	0.00	up
0.00	0.00	up
0.00	0.00	up
0.00	0.00	up
0.01	0.02	up
0.00	0.00	up
0.00	0.00	down
0.00	0.00	up
0.00	0.00	up
0.00	0.00	down
0.00	0.00	down
0.00	0.00	down
0.00	0.00	up
0.00	0.00	down
0.00	0.00	up
0.00	0.01	down
0.01	0.02	up
0.00	0.00	down
0.00	0.00	up
0.00	0.00	up
0.01	0.02	up
0.00	0.00	down
0.00	0.00	up
0.00	0.00	up
0.01	0.03	up
0.00	0.00	up
0.00	0.00	up
0.00	0.00	up
0.00	0.00	up
0.00	0.00	up
0.00	0.00	down
0.00	0.00	down
0.00	0.00	down
0.00	0.00	down
0.00	0.00	down
0.00	0.00	up
0.01	0.02	up
0.00	0.00	up
0.00	0.00	up
0.02	0.04	up

0.00	0.00	down
0.00	0.00	down
0.00	0.00	down
0.00	0.00	up
0.00	0.02	down
0.01	0.03	down
0.00	0.00	up
0.00	0.00	down
0.00	0.00	up
0.00	0.00	down
0.01	0.02	down
0.00	0.00	up
0.00	0.00	up
0.00	0.00	down
0.00	0.00	up
0.00	0.00	up
0.00	0.00	down
0.00	0.00	up
0.00	0.00	up
0.00	0.00	down
0.00	0.00	up
0.00	0.00	down
0.00	0.00	down
0.00	0.01	up
0.00	0.00	up
0.00	0.00	down
0.00	0.00	down
0.00	0.00	down
0.00	0.00	down
0.00	0.00	down
0.00	0.00	up
0.00	0.00	down
0.00	0.01	up
0	0	up
0.00	0.00	up
0.00	0.00	up
0.00	0.00	up
0.00	0.00	down
0.00	0.00	up
0.00	0.00	down
0.00	0.00	up
0.00	0.00	up
0.00	0.00	up
0.00	0.00	up
0.00	0.00	up
0.00	0.00	down
0.00	0.00	up
0.00	0.00	up
0.00	0.00	up
0.00	0.00	up
0.00	0.00	up

0.00	0.00	up
0.00	0.00	up
0.00	0.00	down
0.00	0.00	up
0.00	0.00	down
0.00	0.00	up
0.00	0.00	up
0.00	0.00	up
0.00	0.00	down
0.00	0.00	up
0.00	0.00	up
0.00	0.00	down
0.00	0.00	down
0.00	0.00	up
0.00	0.00	up
0.00	0.00	down
0.00	0.00	up
0.00	0.00	up
0.00	0.00	up
0.00	0.00	up
0.00	0.00	up
0.00	0.00	up
0.00	0.00	up
0.00	0.00	up
0.00	0.00	up
0.00	0.00	up
0.00	0.00	up
0.00	0.00	up
0.00	0.00	down
0.00	0.00	up
0.00	0.00	down
0.00	0.00	down
0.00	0.00	down
0.00	0.00	down
0.00	0.00	up
0.00	0.00	up
0.00	0.00	up
0.00	0.00	down
0.00	0.00	down
0.00	0.00	up
0.00	0.00	up
0.00	0.00	up
0.00	0.00	down
0.00	0.00	up
0.00	0.00	up
0.00	0.00	up
0.00	0.00	up
0.00	0.00	up
0.00	0.00	up
0.00	0.00	up
0.00	0.00	down

0.00	0.00	down
0.00	0.00	up
0.00	0.00	up
0.00	0.00	up
0.00	0.00	down
0.00	0.00	down
0.00	0.01	up
0.00	0.01	up
0.00	0.01	up
0.00	0.01	up
0.00	0.01	up
0.00	0.01	up
0.00	0.01	down
0.01	0.02	up
0.01	0.02	up
0.01	0.02	up
0.01	0.02	up
0.01	0.02	up
0.02	0.05	down
0.00	0.00	up
0.00	0.00	up
0.00	0.00	down
0.00	0.00	up
0.00	0.00	up
0.00	0.00	down
0.00	0.00	up
0.00	0.00	up
0.00	0.00	up
0.00	0.00	up
0.00	0.00	up
0.00	0.01	up
0.01	0.02	up
0.00	0.00	up
0.00	0.00	up
0.00	0.00	up
0.00	0.00	up
0.00	0.00	down
0.00	0.01	down
0.00	0.00	down
0.00	0.00	up
0.00	0.00	up
0.01	0.02	up
0.00	0.00	down
0.00	0.00	down
0.00	0.00	up
0.00	0.01	up
0.00	0.00	down
0.00	0.00	up
0.00	0.00	down
0.00	0.00	down
0.00	0.00	down

0.00	0.00	up
0.00	0.00	down
0.00	0.00	down
0.00	0.00	up
0.00	0.00	up
0.00	0.00	up
0.00	0.00	up
0.00	0.00	up
0.01	0.03	up
0.00	0.00	down
0.00	0.00	up
0.00	0.00	up
0.00	0.00	up
0.00	0.00	down
0.00	0.00	up
0.00	0.00	up
0.00	0.00	down
0.00	0.01	up
0.00	0.00	down
0.00	0.00	up
0.00	0.00	down
0.00	0.00	down
0.00	0.00	up
0.00	0.00	up
0.01	0.02	up
0	0	up
0.00	0.00	up
0.00	0.00	down
0.00	0.00	up
0.00	0.00	up
0.00	0.00	up
0.00	0.01	up
0.00	0.01	up
0.00	0.00	up
0.00	0.00	up
0.00	0.00	up
0.00	0.00	up
0.00	0.00	up
0.00	0.00	up
0.00	0.00	up
0.00	0.00	up
0.00	0.00	up
0.00	0.00	up
0.00	0.00	up
0.00	0.00	up
0.00	0.00	up
0.00	0.00	down
0.00	0.00	up
0.00	0.00	up
0.00	0.00	down
0.00	0.00	up

0.00	0.00	up
0.00	0.00	down
0.00	0.01	down
0.00	0.02	down
0.00	0.00	down
0.00	0.00	down
0.00	0.00	down
0.00	0.00	down
0.00	0.00	down
0.00	0.01	up
0.01	0.02	up
0.00	0.00	down
0.00	0.00	down
0.00	0.00	down
0.01	0.03	down
0	0	down
0.00	0.00	up
0.00	0.00	up
0.00	0.00	down
0.00	0.00	down
0.01	0.02	up
0.00	0.00	up
0.00	0.00	down
0.00	0.00	up
0.00	0.00	up
0.00	0.00	up
0.00	0.01	up
0	0	down
0.00	0.00	up
0.00	0.00	down
0.00	0.00	up
0.00	0.00	down
0.00	0.00	up
0.00	0.00	down
0.00	0.00	up
0.00	0.00	up
0.00	0.00	up
0.00	0.00	up
0.00	0.00	up
0.00	0.00	down
0.00	0.01	down
0.00	0.00	down
0.00	0.00	up
0.00	0.00	down
0.00	0.00	down
0.00	0.00	up
0.00	0.00	up
0.00	0.00	up
0.00	0.00	down
0.00	0.00	down
0.00	0.00	down

0.00	0.00	up
0.00	0.01	up
0.00	0.00	down
0.00	0.00	down
0.00	0.00	up
0.00	0.01	up
0.00	0.00	down
0.00	0.00	down
0.00	0.00	up
0.00	0.01	up
0.00	0.00	up
0.00	0.00	up
0.00	0.00	down
0.00	0.00	up
0.02	0.05	up
0.00	0.00	down
0.00	0.00	up
0.00	0.00	up
0.00	0.00	up
0.00	0.00	down
0.00	0.00	up
0.00	0.00	up
0.00	0.00	up
0.00	0.00	down
0	0	up
0	0	down
0.00	0.00	up
0.00	0.00	down
0.00	0.00	down
0.00	0.00	up
0.00	0.00	up
0.00	0.00	up
0.00	0.00	up
0.00	0.00	up
0.00	0.00	down
0.00	0.00	up
0.00	0.00	down
0.00	0.00	up
0.00	0.00	up
0.00	0.00	up
0.00	0.00	up
0.00	0.00	down
0.00	0.00	up
0.00	0.00	up
0.00	0.00	up
0.00	0.00	up
0.00	0.00	up
0.00	0.00	down
0.00	0.00	down
0.00	0.00	down
0.00	0.00	down

0.00	0.00	up
0.00	0.00	down
0.00	0.00	up
0.00	0.00	up
0.00	0.00	up
0.00	0.00	down
0.00	0.00	up
0.00	0.00	up
0.00	0.00	up
0.00	0.00	up
0.00	0.00	up
0.00	0.00	down
0.00	0.00	up
0.00	0.00	down
0.00	0.00	down
0.00	0.00	up
0.00	0.00	up
0.00	0.00	up
0.00	0.00	down
0.00	0.00	down
0.00	0.00	up
0.00	0.00	up
0.00	0.00	up
0.00	0.00	down
0.00	0.00	up
0.00	0.00	up
0.00	0.00	down
0.00	0.00	down
0.00	0.00	up
0.00	0.00	down
0.00	0.00	up
0.00	0.00	up
0.00	0.00	up
0.00	0.00	down
0.00	0.00	up
0.00	0.00	down
0.00	0.00	up
0.00	0.00	down
0.00	0.01	up
0.00	0.01	up
0.01	0.02	up
0.01	0.04	up
0.02	0.04	up
0.02	0.05	down
0.02	0.05	down
0.00	0.00	up
0.00	0.00	down
0.00	0.00	down
0.00	0.00	up
0.00	0.00	up
0.00	0.01	up

0.00	0.00	up
0.00	0.00	up
0.00	0.00	up
0.00	0.00	up
0.00	0.00	up
0.00	0.00	up
0.00	0.00	down
0.00	0.00	up
0.00	0.00	up
0.00	0.00	down
0.00	0.00	down
0.00	0.00	down
0.00	0.00	down
0.00	0.00	down
0.00	0.00	up
0.00	0.00	down
0.00	0.00	down
0.00	0.00	up
0.00	0.00	down
0.00	0.00	up
0.00	0.00	up
0.00	0.00	down
0.01	0.02	down
0.00	0.00	down
0.00	0.00	down
0.00	0.01	down
0	0	up
0.00	0.00	up
0.00	0.00	down
0.00	0.00	up
0.01	0.03	down
0.00	0.00	up
0.01	0.02	up
0.00	0.00	down
0.00	0.00	up
0.00	0.00	down
0.00	0.00	up
0.00	0.00	up
0.00	0.00	up
0.00	0.00	up
0.00	0.00	up
0.00	0.00	up
0.00	0.00	up
0.00	0.00	up
0.00	0.00	down
0.00	0.00	up
0.00	0.00	down
0.00	0.00	up
0.00	0.00	down
0.00	0.00	up
0.00	0.00	up

0.00	0.00	down
0.00	0.00	down
0.00	0.00	up
0.00	0.00	down
0.00	0.00	up
0.00	0.00	up
0.00	0.00	up
0.00	0.00	down
0.00	0.00	down
0.00	0.00	down
0.00	0.00	up
0.00	0.00	up
0.00	0.00	up
0.00	0.00	down
0.00	0.00	up
0.00	0.00	up
0.00	0.00	up
0.00	0.00	down
0.00	0.00	down
0.00	0.00	up
0.00	0.00	up
0.00	0.00	down
0.00	0.00	down
0.00	0.00	down
0.00	0.00	down
0.00	0.00	down
0.00	0.00	up
0.00	0.00	down
0.00	0.00	down
0.00	0.00	down
0.00	0.00	up
0.00	0.00	down
0.00	0.00	down
0.00	0.00	up
0.00	0.00	up
0.00	0.00	up
0.00	0.00	up
0.00	0.00	up
0.00	0.00	down
0.00	0.00	up
0.00	0.00	up
0.00	0.00	up
0.00	0.00	down
0.00	0.00	down
0.00	0.00	down
0.00	0.00	down
0.00	0.00	down
0.00	0.00	down
0.00	0.00	down
0.00	0.00	down
0.00	0.00	up

0.00	0.00	down
0.00	0.00	down
0.00	0.00	up
0.00	0.00	up
0.00	0.00	down
0.00	0.00	down
0.00	0.00	up
0.00	0.00	up
0.00	0.00	down
0.00	0.00	down
0.00	0.00	down
0.00	0.00	down
0.00	0.00	up
0.00	0.00	up
0.00	0.00	down
0.00	0.00	down
0.00	0.00	up
0.00	0.00	up
0.00	0.00	up
0.00	0.00	down
0.00	0.00	down
0.00	0.00	up
0.00	0.00	down
0.00	0.00	down
0.00	0.00	down
0.00	0.00	down
0.00	0.00	up
0.00	0.00	down
0.00	0.00	up
0.00	0.00	down
0.00	0.00	up
0.00	0.00	down
0.00	0.00	down
0.00	0.00	down
0.00	0.00	up
0.00	0.00	down
0.00	0.00	up
0.00	0.00	down
0.00	0.00	down
0.00	0.00	up
0.00	0.00	down
0.00	0.00	down
0.00	0.00	up
0.00	0.00	up
0.00	0.00	down
0.00	0.00	down
0.00	0.00	up
0.00	0.00	up
0.00	0.00	down
0.00	0.00	down
0.00	0.00	down

0.00	0.00	down
0.00	0.00	up
0.00	0.00	up
0.00	0.00	up
0.00	0.00	down
0.00	0.00	down
0.00	0.00	down
0.00	0.00	up
0.00	0.00	up
0.00	0.00	down
0.00	0.00	down
0.00	0.00	down
0.00	0.01	up
0.00	0.01	down
0.00	0.01	up
0.00	0.01	down
0.00	0.01	up
0.00	0.01	up
0.00	0.01	up
0.00	0.01	down
0.00	0.01	up
0.00	0.01	down
0.00	0.01	up
0.00	0.01	down
0.00	0.01	down
0.00	0.01	down
0.00	0.02	down
0.01	0.02	up
0.01	0.02	up
0.01	0.02	up
0.01	0.02	up
0.01	0.02	down
0.01	0.02	down
0.01	0.02	up
0.01	0.03	up
0.01	0.03	down
0.01	0.03	up
0.01	0.03	up
0.01	0.03	down
0.01	0.03	down
0.01	0.04	up
0.01	0.04	down
0.01	0.04	up
0.02	0.04	up
0.02	0.04	down
0.02	0.05	down
0.00	0.00	down
0.00	0.00	down
0.00	0.00	down
0.00	0.00	down
0.00	0.01	down

0.00	0.01	down
0.01	0.03	down
0	0	down
0.00	0.00	up
0.00	0.00	up
0.00	0.01	down
0	0	down
0.00	0.00	up
0.00	0.00	up
0.00	0.01	down
0.00	0.00	up
0.00	0.00	up
0.00	0.00	up
0.00	0.00	down
0.00	0.00	down
0.00	0.00	up
0.00	0.00	down
0.00	0.01	up
0	0	up
0	0	down
0.00	0.00	up
0.00	0.00	up
0.00	0.00	up
0.00	0.00	down
0.00	0.00	up
0.00	0.00	down
0.00	0.00	up
0.00	0.00	down
0.00	0.00	up
0.00	0.00	up
0.00	0.00	up
0.00	0.00	down
0.00	0.00	up
0.00	0.00	up
0.00	0.00	up
0.00	0.00	up
0.00	0.00	up
0.00	0.00	down
0.00	0.00	up
0.00	0.00	down
0.00	0.00	down
0.00	0.00	down
0.00	0.00	up
0.00	0.00	up
0.00	0.00	up
0.00	0.00	down
0.00	0.00	up
0.00	0.00	up
0.00	0.00	down
0.00	0.00	down
0.00	0.00	up

0.00	0.00	up
0.00	0.00	up
0.00	0.00	up
0.00	0.00	down
0.00	0.00	up
0.00	0.00	up
0.00	0.00	down
0.00	0.00	down
0.00	0.00	up
0.00	0.00	up
0.00	0.00	up
0.00	0.00	up
0.00	0.00	down
0.00	0.00	up
0.00	0.00	up
0.00	0.00	up
0.00	0.00	up
0.00	0.00	down
0.00	0.00	down
0.00	0.00	up
0.00	0.00	up
0.00	0.00	down
0.00	0.00	down
0.00	0.00	down
0.00	0.00	down
0.00	0.00	up
0.00	0.00	up
0.00	0.00	up
0.00	0.00	up
0.00	0.00	down
0.00	0.00	up
0.00	0.00	up
0.00	0.00	up
0.00	0.00	up
0.00	0.00	up
0.00	0.00	up
0.00	0.00	up
0.00	0.00	up
0.00	0.00	up
0.00	0.00	up
0.00	0.00	up
0.00	0.00	down
0.00	0.01	down
0.00	0.01	up
0.00	0.02	down
0.01	0.02	up
0.01	0.04	up
0.00	0.00	up
0.00	0.00	up
0.00	0.00	up
0.00	0.00	up

0.00	0.00	up
0.00	0.00	up
0.00	0.00	up
0.00	0.00	up
0.00	0.00	up
0.00	0.00	up
0.00	0.00	up
0.00	0.00	down
0.00	0.00	up
0.00	0.00	down
0.00	0.00	up
0.00	0.00	down
0.00	0.00	up
0.00	0.00	up
0.00	0.00	down
0.00	0.00	up
0.00	0.00	up
0.00	0.00	down
0.00	0.00	up
0.00	0.00	down
0.00	0.00	up
0.00	0.00	down
0.00	0.00	up
0.00	0.00	up
0.00	0.00	up
0.00	0.00	down
0.00	0.00	up
0.00	0.00	down
0.00	0.00	down
0.00	0.00	up
0.00	0.00	up
0.00	0.00	down
0.00	0.01	down
0.00	0.01	down
0.00	0.01	up
0.00	0.01	down
0.00	0.01	down
0.00	0.01	up
0.00	0.01	down
0.01	0.02	up
0.01	0.02	up
0.01	0.02	down
0.01	0.04	down
0.00	0.00	up
0.00	0.00	up
0.00	0.00	up
0.00	0.00	up
0.00	0.00	down
0	0	up
0.00	0.00	down
0.00	0.00	up

0.00	0.00	up
0.00	0.00	down
0.00	0.00	down
0.00	0.00	down
0.00	0.00	up
0.00	0.00	up
0.00	0.00	down
0.00	0.00	up
0.00	0.00	down
0.00	0.00	up
0.00	0.00	up
0.00	0.00	down
0.00	0.00	up
0.00	0.00	down
0.00	0.00	down
0.00	0.00	up
0.00	0.00	up
0.00	0.00	up
0.00	0.00	down
0.00	0.00	up
0.00	0.00	up
0.00	0.00	up
0.00	0.00	up
0.00	0.00	down
0.00	0.00	down
0.00	0.01	down
0.00	0.01	up
0.00	0.01	up
0.00	0.01	up
0.00	0.02	down
0.01	0.03	down
0.02	0.04	up
0.00	0.00	up
0.01	0.02	up
0.00	0.00	up
0.01	0.02	up
0.00	0.00	up
0.00	0.00	down
0.00	0.00	up
0.00	0.00	up
0.00	0.00	down
0.00	0.00	up
0.00	0.00	down
0.00	0.00	down
0.00	0.00	up
0.01	0.02	down
0.00	0.00	up
0.01	0.03	up
0.00	0.00	down
0.00	0.00	down
0.00	0.01	up

0.01	0.02	down
0.00	0.00	down
0.00	0.00	up
0.00	0.01	down
0.00	0.01	down
0.01	0.03	up
0.01	0.04	down
0.02	0.05	down
0.00	0.00	up
0.00	0.00	up
0.00	0.00	down
0.00	0.00	down
0.00	0.00	up
0.00	0.00	down
0.00	0.00	down
0.00	0.00	down
0.00	0.00	up
0.00	0.00	down
0.00	0.00	up
0.00	0.00	down
0.00	0.00	up
0.00	0.00	up
0.00	0.00	up
0.00	0.00	up
0.00	0.02	down
0	0	up
0.00	0.00	up
0.00	0.00	up
0.00	0.00	up
0.00	0.00	up
0.00	0.00	up
0.00	0.00	down
0.00	0.00	up
0.00	0.00	up
0.00	0.00	down
0.00	0.00	up
0.00	0.00	up
0	0	down
0.00	0.00	up
0.00	0.00	up
0.00	0.00	down
0.00	0.00	up
0.00	0.01	down
0.01	0.02	down
0.00	0.00	down
0.00	0.00	down
0.00	0.00	down
0.00	0.00	down
0.00	0.00	up
0.00	0.00	down
0.00	0.00	down

0.00	0.00	up
0.00	0.01	up
0.00	0.01	up
0	0	up
0	0	up
0.00	0.00	up
0.00	0.00	down
0.00	0.00	down
0.00	0.00	up
0.00	0.00	up
0.00	0.00	down
0.00	0.00	up
0.00	0.00	up
0.00	0.00	up
0.00	0.00	up
0.00	0.00	up
0.00	0.00	up
0.00	0.00	down
0.00	0.00	up
0.00	0.00	up
0.00	0.00	up
0.00	0.00	up
0.00	0.00	up
0.00	0.00	up
0.00	0.00	down
0.00	0.00	down
0.00	0.00	up
0.00	0.00	up
0.00	0.00	up
0.00	0.00	up
0.00	0.00	up
0.00	0.00	up
0.00	0.00	up
0.00	0.00	up
0.00	0.00	up
0.00	0.00	up
0.00	0.01	down
0.00	0.01	up
0.00	0.01	up
0.02	0.04	down
0.00	0.00	up
0.00	0.00	up
0.00	0.00	up
0.00	0.00	up
0.00	0.00	up
0.01	0.02	down
0.00	0.00	up
0.00	0.00	up
0.00	0.00	up
0.00	0.00	down
0.00	0.01	up

0.00	0.00 up
0.00	0.00 up
0.00	0.00 up
0.00	0.00 up
0.00	0.00 up
0.00	0.00 up
0.00	0.00 down
0.00	0.00 down
0.00	0.00 down
0.00	0.00 down
0.00	0.00 up
0.00	0.01 down
0.00	0.00 down
0.00	0.00 up
0.00	0.00 down
0.00	0.00 up
0.00	0.00 down
0.00	0.00 down
0.00	0.00 up
0.00	0.00 down
0.00	0.00 down
0.00	0.00 down
0.00	0.00 up
0.00	0.00 up
0.00	0.01 up
0.00	0.01 down
0.01	0.02 down
0.01	0.02 up
0.01	0.03 up
0.01	0.03 up
0.01	0.04 up
0.00	0.00 up
0.00	0.00 up
0.00	0.00 up
0.00	0.00 up
0.00	0.00 up
0.00	0.00 up
0.00	0.00 up
0.00	0.00 down
0.00	0.00 down
0.00	0.00 down
0.00	0.00 down
0.00	0.01 down
0.02	0.05 up
0.00	0.00 up
0.00	0.00 down
0.00	0.00 down
0.00	0.00 down
0.00	0.00 up
0.00	0.00 up
0.00	0.00 up

0.00	0.00	down
0.00	0.00	up
0.00	0.00	up
0.00	0.00	up
0.00	0.00	down
0.00	0.00	up
0.00	0.01	down
0.00	0.00	down
0.00	0.02	down
0.00	0.00	down
0.00	0.00	down
0.00	0.00	up
0.00	0.00	up
0.00	0.00	up
0.00	0.01	down
0	0	up
0.00	0.00	up
0.00	0.00	down
0.00	0.00	up
0.00	0.00	up
0.00	0.00	up
0.00	0.00	up
0.00	0.00	down
0.00	0.00	down
0.00	0.00	down
0.00	0.00	up
0.00	0.00	up
0.00	0.00	up
0.00	0.00	up
0.00	0.00	down
0.00	0.00	up
0.00	0.00	up
0.00	0.00	down
0.00	0.00	down
0.00	0.00	up
0.00	0.00	up
0.00	0.00	up
0.00	0.00	up
0.00	0.00	down
0.00	0.00	down
0.00	0.00	down
0.00	0.00	up
0.00	0.00	up
0.00	0.00	up
0.00	0.00	up
0.00	0.00	down
0.00	0.00	up
0.00	0.00	down
0.00	0.00	up
0.00	0.00	up
0.00	0.00	up

0.00	0.00	down
0.00	0.00	down
0.00	0.00	up
0.00	0.00	up
0.00	0.01	up
0.00	0.01	down
0.01	0.02	down
0.01	0.02	down
0.00	0.00	up
0.00	0.00	up
0.00	0.00	up
0.00	0.01	up
0.00	0.00	up
0.00	0.00	up
0.00	0.00	up
0.00	0.00	up
0.00	0.00	down
0.00	0.00	up
0.00	0.00	up
0.00	0.00	down
0.00	0.00	up
0.00	0.00	down
0.00	0.00	up
0.00	0.00	down
0.00	0.00	up
0.00	0.00	down
0.00	0.00	up
0.00	0.00	down
0.00	0.00	down
0.00	0.00	up
0.00	0.00	down
0.00	0.00	up
0.00	0.00	up
0.00	0.00	down
0.00	0.01	up
0.00	0.01	down
0.01	0.02	up
0.01	0.02	up
0.01	0.02	down
0.01	0.02	up
0.01	0.03	up
0.01	0.03	down
0.01	0.03	down
0.02	0.04	up
0.00	0.00	down
0.00	0.00	up
0.00	0.00	up
0.00	0.00	up
0.00	0.00	down
0.00	0.00	up
0	0	up

0	0	up
0.00	0.00	up
0.00	0.00	up
0.00	0.00	down
0.00	0.00	up
0.00	0.00	up
0.00	0.00	up
0.00	0.00	up
0.00	0.00	up
0.00	0.00	up
0.00	0.00	up
0.00	0.00	down
0.00	0.00	up
0.00	0.00	down
0.00	0.00	up
0.00	0.00	down
0.00	0.00	down
0.00	0.00	up
0.00	0.00	down
0.00	0.00	down
0.00	0.00	up
0.00	0.00	up
0.00	0.00	up
0.00	0.00	down
0.00	0.00	up
0.00	0.01	up
0.01	0.02	up
0.01	0.03	up
0.02	0.05	down
0.00	0.00	down
0.01	0.02	up
0.00	0.00	up
0.00	0.01	up
0.00	0.00	up
0.01	0.02	up
0.00	0.00	up
0.00	0.00	down
0.00	0.00	up
0.00	0.00	down
0.00	0.00	up
0.00	0.00	up
0.00	0.00	up
0.00	0.00	up
0.00	0.00	up
0.00	0.00	down
0.00	0.00	up
0	0	up
0.00	0.00	down
0.00	0.00	down
0.00	0.00	up
0.00	0.00	up

0.00	0.00	down
0.00	0.00	down
0.00	0.00	down
0.00	0.00	down
0.00	0.00	down
0.00	0.00	down
0.00	0.00	up
0.00	0.00	down
0.00	0.00	down
0.00	0.00	down
0.00	0.00	up
0.00	0.00	up
0.00	0.00	down
0.00	0.00	down
0.00	0.00	up
0.00	0.00	up
0.00	0.00	down
0.00	0.00	up
0.00	0.00	up
0.00	0.00	down
0.00	0.00	up
0.00	0.00	up
0.00	0.00	down
0.00	0.00	up
0.00	0.01	down
0.00	0.01	down
0.00	0.01	up
0.00	0.01	down
0.00	0.01	up
0.01	0.02	down
0.01	0.02	down
0.01	0.04	down
0.02	0.05	down
0.02	0.05	down
0.00	0.00	down
0.00	0.00	up
0.00	0.00	up
0.00	0.00	up
0.00	0.00	up
0.00	0.00	up
0.00	0.00	up
0.00	0.00	up
0.00	0.00	down
0.00	0.00	up
0.00	0.00	down
0.00	0.00	down
0.00	0.00	down
0.01	0.03	up
0.00	0.00	up
0.00	0.00	up
0.00	0.00	up

0.00	0.00	up
0.00	0.00	up
0.00	0.00	up
0.00	0.00	down
0.00	0.00	up
0.00	0.00	down
0.00	0.00	up
0.00	0.01	down
0.01	0.02	down
0.02	0.05	down
0.00	0.00	up
0.00	0.00	up
0.00	0.00	down
0.00	0.00	down
0.00	0.00	up
0.00	0.00	down
0.00	0.00	up
0.00	0.00	down
0.00	0.00	up
0.00	0.00	down
0.00	0.00	down
0.00	0.00	down
0.00	0.00	up
0.00	0.00	down
0.00	0.00	down
0.00	0.00	up
0.00	0.00	up
0.00	0.00	down
0.00	0.00	down
0.00	0.00	up
0.00	0.00	down
0.00	0.00	up
0.00	0.00	down
0.00	0.00	down
0.00	0.00	up
0.00	0.00	up
0.00	0.01	down
0.00	0.01	down
0.01	0.02	down
0.01	0.02	down
0.01	0.02	down
0.01	0.02	down
0.01	0.03	up
0.02	0.04	down
0.00	0.00	up
0.00	0.00	up
0.00	0.00	up
0.00	0.00	down
0.01	0.04	up
0.00	0.00	up
0.00	0.00	up

0.00	0.00 down
0.00	0.00 down
0.00	0.00 down
0.00	0.00 up
0.00	0.00 down
0.00	0.00 down
0.00	0.00 up
0.00	0.00 down
0.00	0.00 up
0.00	0.00 up
0.00	0.00 down
0.00	0.00 down
0.00	0.00 up
0.00	0.00 down
0.00	0.00 down
0.00	0.00 down
0.00	0.00 up
0.00	0.00 down
0.00	0.00 down
0.00	0.00 down
0.00	0.00 up
0.00	0.00 up
0.00	0.00 up
0.00	0.00 up
0.00	0.00 up
0.00	0.00 up
0.00	0.01 down
0.01	0.02 up
0.01	0.02 up
0.01	0.02 up
0.01	0.02 down
0.01	0.02 down
0.01	0.02 up
0.01	0.02 up
0.00	0.00 up
0.00	0.00 down
0.00	0.01 up
0.02	0.05 down
0.00	0.00 up
0.00	0.00 down
0.00	0.00 up
0.01	0.03 up
0.02	0.05 down
0.00	0.00 up
0.00	0.00 up
0.00	0.00 down
0.00	0.00 up
0.00	0.00 down
0.00	0.00 down
0.00	0.00 up
0.00	0.00 up

0.00	0.00	down
0.00	0.00	up
0.00	0.00	down
0.00	0.00	down
0.00	0.00	up
0.00	0.00	down
0.00	0.00	up
0.00	0.00	up
0.00	0.00	up
0.00	0.01	down
0.00	0.00	up
0.00	0.00	up
0.00	0.00	up
0.00	0.00	up
0.01	0.02	up
0.00	0.00	up
0.00	0.00	up
0.00	0.00	up
0.00	0.00	down
0.00	0.00	up
0.00	0.00	up
0.00	0.00	down
0.00	0.00	up
0.00	0.00	down
0.00	0.00	up
0.00	0.00	down
0.00	0.00	down
0.00	0.02	down
0.01	0.02	down
0	0	up
0.00	0.00	up
0.00	0.00	up
0.00	0.00	up
0.00	0.00	up
0.00	0.00	up
0.00	0.00	up
0.00	0.00	up
0.00	0.00	down
0.00	0.00	up
0.00	0.00	down
0.00	0.00	up
0.00	0.00	up
0.00	0.00	down
0.00	0.00	up
0.00	0.00	up
0.00	0.00	up
0.00	0.00	up
0.00	0.00	down
0.00	0.00	up
0.00	0.00	down
0.00	0.00	up

0.00	0.00	down
0.00	0.00	down
0.00	0.00	up
0.00	0.00	up
0.00	0.00	down
0.00	0.00	up
0.00	0.00	up
0.00	0.00	down
0.00	0.00	down
0.00	0.00	down
0.00	0.00	down
0.00	0.00	up
0.00	0.01	down
0.01	0.04	up
0.00	0.00	down
0.00	0.00	down
0.01	0.02	up
0.01	0.03	up
0.00	0.00	down
0.00	0.00	up
0.00	0.00	up
0.02	0.05	down
0.00	0.00	down
0.00	0.00	up
0.00	0.00	down
0.00	0.00	down
0.00	0.00	up
0.00	0.00	up
0.00	0.00	down
0.00	0.00	up
0.00	0.00	up
0.00	0.00	up
0.00	0.00	up
0.00	0.01	down
0.01	0.02	down
0.00	0.00	down
0.00	0.00	down
0.00	0.00	down
0.00	0.00	up
0.00	0.00	down
0.00	0.00	up
0.00	0.00	down
0.00	0.00	up
0.00	0.01	up
0.00	0.01	up
0.00	0.01	up
0.00	0.02	down
0.01	0.02	up
0.01	0.02	down
0.01	0.04	up
0.00	0.00	up

0.00	0.00	down
0.00	0.00	down
0.01	0.03	down
0.00	0.00	down
0.00	0.00	up
0.00	0.00	up
0.01	0.02	down
0.00	0.00	up
0.00	0.00	up
0.00	0.00	down
0.00	0.00	down
0.00	0.00	down
0.01	0.02	down
0.00	0.00	up
0.00	0.00	up
0.00	0.00	up
0.00	0.00	up
0.00	0.00	up
0.00	0.00	down
0.00	0.00	down
0.00	0.00	down
0.00	0.00	down
0.00	0.00	up
0.00	0.00	up
0.00	0.00	down
0.00	0.00	down
0.00	0.00	down
0.00	0.00	up
0.00	0.00	down
0.00	0.00	up
0.00	0.00	up
0.00	0.01	down
0.00	0.00	down
0.00	0.00	up
0.00	0.00	up
0.00	0.00	up
0.00	0.00	up
0	0	up
0.00	0.00	up
0.00	0.01	up
0.00	0.01	up
0.00	0.00	down
0.00	0.00	up
0.00	0.00	up
0.00	0.00	up
0.00	0.00	up
0.00	0.00	up
0.00	0.00	up
0.00	0.00	down
0.00	0.00	up
0.00	0.00	down

0.00	0.00	down
0.00	0.00	down
0.00	0.01	down
0.00	0.01	up
0.01	0.02	up
0.01	0.02	up
0.01	0.03	up
0.00	0.00	down
0.00	0.00	up
0.00	0.00	up
0.00	0.01	down
0.01	0.02	up
0.00	0.00	up
0.00	0.00	up
0.00	0.00	up
0.00	0.00	up
0.00	0.00	down
0.00	0.01	down
0.00	0.01	down
0.02	0.04	up
0.00	0.00	up
0.00	0.01	up
0.00	0.00	up
0.00	0.00	up
0.00	0.00	up
0.00	0.00	up
0.00	0.00	up
0.00	0.00	down
0	0	up
0	0	down
0.00	0.00	up
0.00	0.00	up
0.00	0.00	down
0.00	0.00	down
0.00	0.00	up
0.00	0.00	up
0.00	0.00	up
0.00	0.00	up
0.00	0.00	up
0.00	0.00	up
0.00	0.00	down
0.00	0.00	up
0.00	0.00	down
0.00	0.00	down
0.00	0.00	down
0.00	0.00	down
0.00	0.00	down
0.00	0.00	down
0.00	0.00	up
0.00	0.00	down
0.00	0.00	up

0.00	0.00	down
0.00	0.00	up
0.00	0.00	down
0.00	0.00	down
0.00	0.00	down
0.00	0.00	up
0.00	0.00	up
0.00	0.00	down
0.00	0.00	down
0.00	0.00	down
0.00	0.00	up
0.00	0.00	up
0.00	0.00	down
0.00	0.00	up
0.00	0.00	up
0.00	0.00	down
0.00	0.00	down
0.00	0.00	down
0.00	0.00	down
0.00	0.00	down
0.00	0.00	down
0.00	0.00	up
0.00	0.00	up
0.00	0.00	down
0.00	0.00	up
0.00	0.00	down
0.00	0.00	up
0.00	0.00	up
0.00	0.00	up
0.00	0.00	down
0.00	0.00	down
0.00	0.00	up
0.00	0.00	up
0.00	0.00	up
0.00	0.00	down
0.00	0.00	up
0.00	0.00	up
0.00	0.00	down
0.00	0.00	up
0.00	0.00	down
0.00	0.00	up
0.00	0.00	down
0.00	0.00	up
0.00	0.00	up
0.00	0.00	down
0.00	0.00	up
0.00	0.00	down
0.00	0.00	up
0.00	0.00	down
0.00	0.00	up
0.00	0.00	up

0.00	0.00	down
0.00	0.00	down
0.00	0.00	up
0.00	0.00	down
0.00	0.00	up
0.00	0.00	up
0.00	0.00	down
0.00	0.00	up
0.00	0.00	up
0.00	0.00	down
0.00	0.00	up
0.00	0.00	up
0.00	0.00	down
0.00	0.00	up
0.00	0.00	down
0.00	0.00	down
0.00	0.00	up
0.00	0.00	up
0.00	0.00	up
0.00	0.00	up
0.00	0.00	down
0.00	0.00	up
0.00	0.00	down
0.00	0.00	up
0.00	0.00	down
0.00	0.00	up
0.00	0.00	up
0.00	0.00	down
0.00	0.00	up
0.00	0.00	up
0.00	0.00	up
0.00	0.00	up
0.00	0.00	down
0.00	0.00	up
0.00	0.00	down
0.00	0.00	up
0.00	0.00	up
0.00	0.00	up
0.00	0.00	up
0.00	0.00	up
0.00	0.00	up
0.00	0.00	up
0.00	0.00	up
0.00	0.01	up
0.00	0.01	down
0.00	0.01	up
0.00	0.01	up
0.00	0.01	up
0.00	0.01	down
0.00	0.01	up
0.00	0.01	down

0.00	0.01	down
0.00	0.01	down
0.00	0.01	up
0.00	0.01	up
0.00	0.01	up
0.00	0.01	up
0.00	0.01	down
0.00	0.01	up
0.00	0.02	down
0.00	0.02	down
0.00	0.02	down
0.01	0.02	down
0.01	0.02	down
0.01	0.02	down
0.01	0.02	down
0.01	0.03	up
0.01	0.03	down
0.01	0.03	up
0.01	0.04	down
0.02	0.04	up
0.02	0.04	up
0.02	0.05	down
0.02	0.05	down
0.02	0.05	down
0.02	0.05	down
0.02	0.05	up
0.00	0.00	up
0.00	0.00	down
0.00	0.00	up
0.00	0.00	down
0.00	0.00	up
0.00	0.00	up
0.00	0.00	down
0.00	0.00	up
0.00	0.00	up
0.00	0.00	up
0.00	0.00	up
0.00	0.00	up
0.00	0.00	up
0.00	0.00	up
0.00	0.00	up
0.00	0.01	down
0.01	0.02	up
0.01	0.03	down
0.01	0.03	down
0.01	0.03	down
0.01	0.03	down
0.01	0.03	down
0.00	0.00	down
0.00	0.00	up
0.00	0.00	up

0.00	0.00	up
0.00	0.00	down
0.01	0.02	up
0.00	0.01	down
0.00	0.01	up
0.00	0.00	down
0.00	0.00	down
0.00	0.01	up
0.00	0.00	up
0.00	0.00	up
0.00	0.00	down
0.00	0.00	down
0.00	0.00	down
0.00	0.00	down
0.00	0.00	down
0.00	0.00	down
0.00	0.00	down
0.00	0.00	down
0.00	0.00	down
0.00	0.01	down
0.00	0.01	up
0.00	0.01	down
0.00	0.01	down
0.01	0.02	down
0.01	0.02	down
0.01	0.02	down
0.01	0.03	up
0.02	0.04	down
0.00	0.00	up
0.00	0.00	up
0.00	0.00	up
0.00	0.00	up
0.00	0.00	down
0.00	0.00	up
0.00	0.00	down
0.00	0.00	down
0.00	0.00	up
0.00	0.00	up
0.00	0.00	up
0.00	0.00	up
0.00	0.00	down
0.00	0.00	up
0.00	0.00	up
0.00	0.01	down
0.01	0.02	up
0.02	0.05	down
0.02	0.05	down
0.00	0.00	up
0.00	0.00	up
0.00	0.00	up
0.00	0.00	up

0.00	0.00	up
0.00	0.00	up
0	0	down
0.00	0.00	up
0.00	0.00	up
0.00	0.00	up
0.01	0.03	up
0.00	0.00	up
0.00	0.00	up
0.00	0.00	down
0.00	0.00	down
0.00	0.00	down
0.00	0.00	down
0.00	0.00	up
0.00	0.00	up
0.00	0.00	up
0.01	0.02	up
0.00	0.00	up
0.00	0.00	up
0.00	0.00	up
0.00	0.00	down
0.00	0.00	up
0.00	0.00	up
0.00	0.00	down
0.00	0.00	down
0.00	0.00	up
0.00	0.00	down
0.00	0.01	up
0.00	0.01	up
0.02	0.05	up
0.00	0.00	up
0.00	0.00	up
0.00	0.00	up
0.00	0.00	up
0.01	0.02	up
0.00	0.00	down
0.00	0.01	down
0.00	0.01	down
0.00	0.00	up
0.00	0.00	down
0.00	0.00	down
0.01	0.03	down
0.00	0.00	up
0.00	0.00	down
0.00	0.00	up
0.00	0.00	up
0.00	0.00	down
0.00	0.00	up
0.00	0.00	down
0.00	0.00	up
0.00	0.00	up

0.00	0.00	down
0.00	0.01	up
0.01	0.03	down
0.00	0.00	down
0.00	0.00	up
0.00	0.00	down
0.00	0.00	up
0.00	0.00	up
0.00	0.00	down
0.00	0.00	up
0.00	0.00	down
0.00	0.00	down
0.00	0.01	down
0.02	0.05	down
0.00	0.00	up
0.00	0.00	up
0.00	0.00	up
0.01	0.02	down
0.00	0.00	up
0.00	0.00	up
0.00	0.00	up
0.00	0.00	up
0	0	up
0.00	0.00	up
0.00	0.00	down
0.00	0.00	down
0.00	0.00	down
0.00	0.00	up
0.00	0.00	up
0.00	0.00	up
0.00	0.00	up
0.00	0.00	down
0.00	0.00	down
0.00	0.00	up
0.00	0.00	down
0.00	0.00	down
0.00	0.00	up
0.00	0.00	down
0.00	0.00	down
0.00	0.00	down
0.00	0.00	up
0.00	0.00	down
0.00	0.00	down
0.00	0.00	down
0.00	0.00	down
0.00	0.00	down
0.00	0.00	up
0.00	0.00	down
0.00	0.00	up
0.00	0.00	down
0.00	0.00	down

0.00	0.00	up
0.00	0.00	down
0.00	0.00	up
0.00	0.00	down
0.00	0.00	down
0.00	0.00	up
0.00	0.00	down
0.00	0.00	up
0.00	0.00	down
0.00	0.00	down
0.00	0.00	up
0.00	0.00	down
0.00	0.00	down
0.00	0.00	down
0.00	0.00	up
0.00	0.00	down
0.00	0.00	up
0.00	0.00	down
0.00	0.00	up
0.00	0.00	up
0.00	0.00	down
0.00	0.00	up
0.00	0.00	down
0.00	0.00	down
0.00	0.00	up
0.00	0.00	down
0.00	0.00	up
0.00	0.00	down
0.00	0.00	down
0.00	0.00	up
0.00	0.00	up
0.00	0.00	up
0.00	0.00	up
0.00	0.00	up
0.00	0.00	up
0.00	0.00	up
0.00	0.00	up
0.00	0.00	up
0.00	0.01	up
0.00	0.01	down
0.00	0.01	up
0.00	0.01	up
0.00	0.01	down
0.00	0.01	up
0.00	0.01	up
0.00	0.01	down
0.00	0.01	down
0.00	0.01	up
0.00	0.02	down
0.01	0.02	down
0.01	0.02	down

0.01	0.02	down
0.01	0.02	down
0.01	0.03	down
0.02	0.04	up
0.02	0.04	up
0.02	0.04	down
0.02	0.04	up
0.02	0.05	down
0.02	0.05	down
0.02	0.05	down
0.02	0.05	up
0.00	0.00	down
0.00	0.00	up
0.00	0.00	down
0.00	0.00	up
0.00	0.00	up
0.00	0.00	up
0.00	0.00	up
0.00	0.00	down
0.00	0.01	up
0.00	0.02	down
0.01	0.04	down
0.00	0.00	up
0.00	0.00	down
0.00	0.00	down
0.00	0.00	up
0.01	0.02	down
0.00	0.00	down
0.00	0.00	down
0.00	0.00	up
0.00	0.00	up
0.00	0.00	up
0.00	0.00	up
0.00	0.00	down
0.00	0.00	down
0.00	0.01	up
0.00	0.00	up
0.00	0.00	down
0.00	0.00	up
0.00	0.00	up
0.00	0.00	down
0.00	0.01	down
0.00	0.00	up
0.00	0.00	up
0.00	0.00	down
0.00	0.00	up
0.01	0.03	up
0.02	0.05	down
0.00	0.00	down
0.00	0.00	up
0.00	0.00	down

0.00	0.00	up
0.00	0.00	up
0.00	0.00	down
0.00	0.00	up
0.00	0.00	down
0.00	0.00	up
0.00	0.00	down
0.00	0.00	down
0.00	0.00	down
0.00	0.00	down
0.00	0.00	up
0.00	0.00	up
0.00	0.00	up
0.00	0.00	up
0.00	0.00	down
0.00	0.00	up
0.00	0.00	down
0.00	0.00	up
0.00	0.00	up
0.00	0.00	up
0.00	0.00	down
0.00	0.00	down
0.00	0.00	up
0.00	0.00	up
0.00	0.00	up
0.00	0.00	up
0.00	0.00	up
0.00	0.00	down
0.00	0.00	up
0.00	0.01	up
0.00	0.01	up
0.00	0.00	up
0.00	0.00	down
0.00	0.00	up
0.00	0.00	up
0.00	0.01	down
0.00	0.00	up
0.00	0.00	down
0.00	0.00	down
0.00	0.00	up
0.00	0.01	up
0.00	0.00	down
0.00	0.00	up
0.00	0.00	up
0.00	0.00	up
0.00	0.00	down
0.00	0.00	up
0.00	0.00	down
0.00	0.01	up
0.02	0.05	down
0.00	0.00	down

0.00	0.00	up
0.00	0.00	down
0.00	0.00	up
0.00	0.01	up
0.00	0.00	up
0.00	0.00	up
0.00	0.00	up
0.00	0.00	up
0.00	0.00	up
0.00	0.00	up
0.00	0.00	up
0.00	0.00	down
0.00	0.00	down
0.00	0.00	down
0.00	0.00	up
0.00	0.00	down
0.00	0.00	down
0.00	0.00	up
0.00	0.00	up
0.00	0.00	up
0.02	0.05	down
0.00	0.00	down
0.00	0.00	up
0.00	0.00	down
0.00	0.00	down
0.00	0.00	up
0.00	0.00	down
0	0	down
0.00	0.00	up
0.00	0.00	up
0.00	0.00	up
0.00	0.00	up
0.00	0.00	up
0.00	0.00	up
0.00	0.00	down
0.00	0.00	down
0.00	0.00	down
0.00	0.00	down
0.00	0.01	down
0.00	0.01	down
0.01	0.02	down
0.02	0.05	down
0.00	0.00	up
0.00	0.00	down
0.00	0.00	down
0.00	0.00	up
0.01	0.04	down
0.00	0.00	down
0.00	0.00	down
0.00	0.00	up
0.00	0.00	up

0.00	0.00	up
0.00	0.01	down
0.00	0.00	up
0.00	0.00	down
0.00	0.00	up
0.00	0.00	up
0.00	0.00	up
0.00	0.00	down
0.00	0.00	up
0.00	0.00	up
0.00	0.00	up
0.01	0.03	up
0.02	0.04	up
0.00	0.00	down
0.00	0.00	up
0.00	0.01	down
0.01	0.02	up
0.00	0.00	up
0.00	0.00	up
0.00	0.00	down
0.00	0.00	down
0.00	0.00	up
0.01	0.02	down
0.01	0.03	down
0.01	0.04	down
0.00	0.00	up
0.00	0.00	up
0.00	0.00	down
0.00	0.00	down
0.00	0.00	up
0.00	0.00	up
0.01	0.02	up
0.00	0.00	down
0.00	0.00	up
0.02	0.04	down
0.00	0.00	up
0.00	0.00	up
0.00	0.00	up
0.00	0.00	down
0.00	0.00	down
0.00	0.01	up
0.00	0.01	down
0.02	0.05	down
0.00	0.00	down
0.00	0.00	down
0.00	0.00	up
0.00	0.00	up
0.00	0.00	up
0.00	0.01	down
0.01	0.02	up
0.00	0.00	up

0.00	0.00	down
0.00	0.00	up
0.00	0.00	up
0.00	0.00	up
0.00	0.00	up
0.00	0.00	up
0.00	0.00	up
0.00	0.00	down
0.00	0.00	down
0.00	0.00	up
0.00	0.01	up
0.00	0.00	down
0.00	0.00	down
0.00	0.00	up
0.00	0.00	up
0.00	0.00	up
0.00	0.00	down
0.01	0.02	up
0.00	0.00	down
0.00	0.00	up
0.00	0.00	up
0.01	0.03	down
0.00	0.00	down
0.00	0.00	down
0.00	0.00	down
0.00	0.00	down
0.00	0.01	down
0.00	0.01	down
0.01	0.02	down
0.00	0.00	up
0.02	0.04	up
0.00	0.00	down
0.00	0.00	down
0.00	0.00	down
0.00	0.00	up
0.00	0.00	up
0.00	0.01	down
0.00	0.00	up
0.00	0.00	up
0.00	0.00	up
0.00	0.00	up
0.00	0.00	down
0.00	0.00	up
0.00	0.01	down
0.00	0.00	down
0.00	0.00	down
0.00	0.00	up
0.00	0.00	up
0.00	0.00	up
0.00	0.00	up
0.00	0.00	down

0.00	0.00	up
0.00	0.01	up
0.00	0.01	up
0.00	0.01	up
0.01	0.04	down
0	0	up
0.00	0.00	up
0.00	0.00	up
0.00	0.00	up
0.00	0.00	up
0.00	0.00	up
0.00	0.00	down
0.00	0.00	up
0.00	0.00	up
0.00	0.00	down
0.00	0.00	down
0.00	0.00	down
0.00	0.00	down
0.00	0.00	down
0.00	0.00	down
0.00	0.00	up
0.00	0.00	down
0.00	0.00	up
0.00	0.00	up
0.00	0.00	up
0.00	0.00	down
0.00	0.00	up
0.00	0.00	up
0.00	0.00	up
0.00	0.00	down
0.00	0.01	up
0.00	0.01	down
0.00	0.01	up
0.01	0.02	up
0.00	0.00	up
0.00	0.00	up
0.00	0.00	down
0.00	0.00	up
0.00	0.00	down
0.00	0.00	down
0.00	0.00	down
0.00	0.00	down
0.00	0.00	up
0.00	0.00	up
0.00	0.00	up
0.00	0.00	up
0.00	0.00	up
0.00	0.00	down
0.00	0.00	up
0.00	0.00	up
0.00	0.00	up

0.00	0.00	up
0.00	0.00	up
0.00	0.00	up
0.00	0.00	up
0.00	0.01	up
0.00	0.00	up
0.00	0.00	up
0.00	0.00	up
0.00	0.00	up
0.00	0.00	down
0.00	0.00	up
0.00	0.00	down
0.00	0.00	up
0.00	0.00	up
0.00	0.00	up
0.00	0.00	up
0.00	0.00	up
0.00	0.00	up
0.00	0.00	up
0.00	0.00	down
0.00	0.00	up
0.00	0.00	down
0.00	0.00	down
0.01	0.02	down
0.00	0.00	down
0.00	0.00	down
0.01	0.03	up
0.00	0.00	down
0.00	0.00	down
0.00	0.00	down
0.00	0.00	down
0.00	0.00	up
0.00	0.00	up
0.00	0.00	down
0.00	0.00	down
0.00	0.00	down
0.00	0.00	down
0.00	0.00	up
0.00	0.00	up
0.00	0.00	up
0.00	0.00	down
0.00	0.00	down
0.00	0.00	down
0.00	0.01	down
0.01	0.02	up
0.00	0.00	down
0.00	0.00	down
0.01	0.03	down
0.00	0.00	down
0.01	0.02	up
0.00	0.00	up

0.00	0.00	up
0.00	0.00	up
0.00	0.01	up
0.00	0.00	down
0.00	0.00	up
0.00	0.00	down
0.00	0.00	down
0.00	0.00	up
0.00	0.00	up
0.00	0.01	down
0.00	0.00	up
0.01	0.02	up
0.00	0.00	up
0.00	0.00	down
0.00	0.00	up
0.00	0.00	up
0.00	0.00	down
0.00	0.00	up
0.00	0.00	up
0.01	0.03	up
0.02	0.05	down
0.00	0.00	up
0.00	0.00	down
0.00	0.00	down
0.00	0.00	up
0.00	0.00	up
0.00	0.00	up
0.00	0.00	up
0.00	0.00	down
0.00	0.00	down
0.00	0.00	down
0.00	0.00	down
0.00	0.00	down
0.01	0.02	up
0.01	0.02	down
0.00	0.00	up
0.00	0.00	up
0.00	0.00	down
0.01	0.02	up
0.00	0.00	down
0.00	0.00	down
0.00	0.00	up
0.01	0.02	up
0.00	0.00	up
0.00	0.00	down
0.00	0.00	up
0.00	0.00	down
0.00	0.00	down
0.00	0.00	up
0.00	0.00	down
0.00	0.00	up

0.00	0.00	down
0	0	down
0.00	0.00	up
0.00	0.00	up
0.00	0.00	up
0.00	0.00	up
0.00	0.00	up
0.00	0.00	up
0.00	0.00	down
0.00	0.00	up
0.00	0.00	up
0.00	0.00	down
0.00	0.00	up
0.00	0.00	down
0.00	0.00	up
0.00	0.00	up
0.00	0.00	up
0.00	0.00	down
0.00	0.00	up
0.00	0.00	up
0.00	0.00	up
0.00	0.00	up
0.00	0.00	up
0.00	0.00	up
0.00	0.00	up
0.00	0.00	down
0.00	0.00	down
0.00	0.01	up
0.00	0.01	down
0.00	0.01	up
0.00	0.02	down
0.00	0.00	up
0.00	0.00	up
0.00	0.00	down
0.00	0.00	up
0.00	0.00	up
0.00	0.00	down
0.00	0.00	up
0.00	0.00	down
0.00	0.00	down
0.00	0.00	up
0.00	0.00	down
0.02	0.05	down
0.00	0.00	up
0.00	0.00	up
0.00	0.00	up
0.00	0.00	down
0.00	0.00	up
0.00	0.01	down
0.00	0.00	up
0.00	0.00	down

0.00	0.00	up
0	0	up
0.00	0.00	up
0.00	0.00	up
0.00	0.00	up
0.00	0.00	down
0.00	0.00	down
0.00	0.00	up
0.00	0.00	up
0.00	0.00	up
0.00	0.00	up
0.00	0.00	up
0.00	0.00	down
0.00	0.00	up
0.00	0.00	up
0.00	0.00	down
0.00	0.00	up
0.00	0.00	up
0.00	0.01	up
0.00	0.01	down
0.00	0.01	up
0.00	0.02	down
0.01	0.02	up
0.00	0.00	down
0.00	0.01	down
0.01	0.04	down
0.00	0.00	up
0.00	0.00	up
0.00	0.00	down
0.00	0.00	up
0.00	0.00	down
0.00	0.00	down
0.00	0.00	up
0.00	0.00	down
0.00	0.00	down
0.00	0.00	up
0.00	0.00	up
0.00	0.00	down
0.00	0.00	down
0.00	0.00	down
0.00	0.00	down
0.00	0.00	up
0.00	0.00	down
0.00	0.00	down
0.00	0.00	up
0.00	0.00	up
0.00	0.01	up
0.00	0.01	up
0.00	0.01	up
0.00	0.01	down
0.01	0.03	down

0.02	0.04	up
0.02	0.05	down
0.00	0.00	down
0.00	0.00	down
0.00	0.00	up
0.00	0.00	down
0.00	0.00	down
0.00	0.00	down
0.00	0.00	up
0.00	0.00	up
0	0	down
0.00	0.00	up
0.00	0.00	up
0.00	0.00	up
0.00	0.00	up
0.00	0.00	down
0.00	0.00	down
0.00	0.00	down
0.00	0.00	up
0.00	0.00	down
0.00	0.00	down
0.00	0.00	down
0.00	0.00	down
0.00	0.00	down
0.00	0.00	down
0.00	0.00	up
0.00	0.00	down
0.00	0.00	up
0.00	0.00	down
0.00	0.00	down
0.00	0.00	up
0.00	0.00	up
0.00	0.00	up
0.00	0.00	down
0.00	0.00	up
0.00	0.00	up
0.00	0.00	down
0.00	0.00	down
0.00	0.00	up
0.00	0.01	down
0.00	0.01	up
0.00	0.01	up
0.01	0.02	down
0.01	0.02	up
0.01	0.03	up
0.01	0.03	up
0.00	0.00	down
0.00	0.00	up
0.00	0.00	down
0.00	0.00	up
0.00	0.00	up

0.00	0.00	down
0.00	0.00	down
0.00	0.00	down
0.01	0.03	up
0.00	0.00	up
0.00	0.00	up
0.01	0.02	down
0.00	0.00	down
0.00	0.00	up
0.00	0.00	down
0.00	0.00	down
0.00	0.00	up
0.00	0.00	up
0.00	0.00	down
0.00	0.00	down
0.00	0.01	down
0.01	0.02	up
0.01	0.04	down
0.00	0.00	down
0.00	0.00	up
0.00	0.00	up
0.02	0.04	down
0.00	0.00	up
0.00	0.00	up
0.00	0.00	down
0.00	0.00	down
0.00	0.00	up
0.00	0.00	down
0.00	0.00	up
0.00	0.00	down
0.00	0.00	up
0.00	0.00	up
0.00	0.00	down
0.00	0.00	down
0.00	0.00	down
0.00	0.00	up
0.00	0.00	up
0.00	0.00	down
0.00	0.01	down
0.01	0.02	down
0.02	0.05	down
0.00	0.00	up
0.00	0.00	down
0.00	0.00	up
0.00	0.00	up
0.00	0.00	up
0.00	0.00	down
0.00	0.00	down
0.00	0.00	up
0.00	0.01	down
0.00	0.00	up

0.01	0.02	up
0.00	0.00	down
0.00	0.00	up
0.00	0.00	up
0.00	0.00	down
0.00	0.00	down
0.00	0.00	up
0.00	0.00	up
0.00	0.00	up
0.00	0.00	down
0.00	0.00	up
0.00	0.00	down
0.00	0.00	up
0.00	0.00	down
0.00	0.00	down
0.00	0.00	down
0.00	0.00	up
0.00	0.00	up
0.00	0.00	down
0.00	0.01	down
0.01	0.02	down
0.02	0.05	down
0.00	0.00	down
0.01	0.02	up
0.00	0.00	down
0.00	0.00	up
0.00	0.00	down
0.00	0.00	up
0.00	0.00	up
0.00	0.00	down
0.00	0.00	down
0.00	0.00	up
0.00	0.00	down
0.01	0.03	up
0.01	0.02	down
0.00	0.00	up
0.00	0.00	up
0.00	0.00	up
0.00	0.00	up
0.00	0.00	up
0.00	0.00	down
0.00	0.00	down
0.00	0.00	up
0.00	0.00	up
0.00	0.00	down
0.00	0.00	up
0.00	0.00	down
0.00	0.00	up
0.00	0.00	down
0.00	0.00	down
0.00	0.00	down

0.00	0.00	up
0.00	0.00	up
0.00	0.00	down
0.00	0.01	down
0.01	0.02	down
0.02	0.05	down
0.00	0.00	up
0.00	0.00	up
0.00	0.00	up
0.00	0.00	up
0.00	0.00	up
0.00	0.00	down
0.00	0.00	down
0.00	0.00	up
0.00	0.00	down
0.00	0.00	down
0.00	0.01	down
0.00	0.00	up
0.00	0.00	down
0.00	0.00	up
0.00	0.00	down
0.00	0.00	down
0.00	0.00	down
0.00	0.00	up
0.00	0.00	down
0.00	0.00	up
0.00	0.00	up
0.00	0.00	up
0.00	0.00	up
0.00	0.00	up
0.00	0.00	down
0.01	0.02	up
0.00	0.00	down
0.00	0.00	up
0.00	0.00	up
0.00	0.00	down
0.00	0.00	down
0.00	0.00	up
0.00	0.00	down
0.00	0.00	down
0.00	0.00	up
0.00	0.00	up
0.00	0.00	down
0.00	0.00	down
0.00	0.00	up
0.01	0.02	up
0.00	0.00	up
0.00	0.00	up
0.00	0.00	down
0.00	0.00	down
0.00	0.00	up

0.00	0.00	up
0.00	0.00	up
0.00	0.00	down
0.00	0.00	down
0.00	0.00	up
0.00	0.00	down
0.00	0.00	up
0.00	0.00	down
0.00	0.00	down
0.00	0.00	down
0.00	0.00	up
0.00	0.00	down
0.00	0.00	up
0.00	0.00	up
0.00	0.00	up
0.00	0.00	down
0.00	0.00	up
0.00	0.00	up
0.00	0.00	up
0.00	0.00	down
0.00	0.00	up
0.00	0.00	up
0.00	0.00	down
0.00	0.00	up
0.00	0.00	up
0.00	0.00	down
0.00	0.00	up
0.00	0.01	up
0.01	0.02	down
0	0	down
0	0	down
0	0	down
0.00	0.00	down
0.00	0.00	up
0.00	0.00	up
0.00	0.00	up
0.00	0.00	down
0.00	0.00	up
0.00	0.00	down
0.00	0.00	up
0.00	0.00	up
0.00	0.00	up
0.00	0.00	down
0.00	0.00	up
0.00	0.00	down
0.00	0.00	up
0.00	0.00	up
0.00	0.00	up
0.00	0.00	down
0.00	0.00	down
0.00	0.00	up

0.00	0.00	up
0.00	0.00	down
0.00	0.00	up
0.00	0.00	up
0.00	0.00	up
0.00	0.00	down
0.00	0.00	down
0.00	0.00	up
0.00	0.00	down
0.00	0.00	down
0.00	0.00	down
0.00	0.00	up
0.00	0.00	down
0.00	0.00	up
0.00	0.00	up
0.00	0.00	down
0.00	0.00	down
0.00	0.00	up
0.00	0.00	down
0.00	0.00	up
0.00	0.00	up
0.00	0.00	up
0.00	0.00	down
0.00	0.00	down
0.00	0.00	down
0.00	0.00	up
0.00	0.00	down
0.00	0.00	down
0.00	0.00	down
0.00	0.00	down
0.00	0.00	down
0.00	0.00	down
0.00	0.00	up
0.00	0.00	down
0.00	0.00	down
0.00	0.00	up
0.00	0.00	down
0.00	0.00	down
0.00	0.00	up
0.00	0.00	up
0.00	0.00	up
0.00	0.00	down
0.00	0.00	down
0.00	0.00	down
0.00	0.00	down
0.00	0.00	down
0.00	0.00	up
0.00	0.00	up
0.00	0.00	down
0.00	0.00	down
0.00	0.00	up

0.00	0.00	down
0.00	0.00	up
0.00	0.00	down
0.00	0.00	up
0.00	0.00	down
0.00	0.00	down
0.00	0.00	up
0.00	0.00	up
0.00	0.00	down
0.00	0.00	down
0.00	0.00	down
0.00	0.00	up
0.00	0.00	down
0.00	0.00	up
0.00	0.00	up
0.00	0.00	down
0.00	0.00	up
0.00	0.00	down
0.00	0.00	down
0.00	0.00	down
0.00	0.00	up
0.00	0.00	up
0.00	0.00	up
0.00	0.00	up
0.00	0.00	up
0.00	0.00	up
0.00	0.00	down
0.00	0.00	up
0.00	0.00	down
0.00	0.00	down
0.00	0.00	up
0.00	0.00	up
0.00	0.00	up
0.00	0.00	down
0.00	0.00	down
0.00	0.00	down
0.00	0.00	down
0.00	0.00	up
0.00	0.00	up
0.00	0.01	up
0.00	0.01	down
0.00	0.01	down
0.00	0.01	up
0.00	0.01	down
0.00	0.01	up
0.00	0.01	up
0.00	0.01	up
0.00	0.01	up
0.00	0.01	up
0.00	0.01	down
0.01	0.02	down

0.01	0.02	down
0.01	0.02	up
0.01	0.02	up
0.01	0.02	down
0.01	0.03	down
0.01	0.03	down
0.01	0.03	up
0.01	0.03	down
0.01	0.03	down
0.01	0.04	down
0.02	0.04	down
0.02	0.04	down
0.02	0.05	down
0.02	0.05	up
0	0	down
0.00	0.00	up
0.00	0.00	up
0.00	0.00	up
0.00	0.00	down
0.00	0.00	down
0.00	0.00	down
0.00	0.00	down
0.00	0.00	up
0.00	0.00	up
0.00	0.00	down
0.00	0.00	down
0.00	0.00	up
0.00	0.00	up
0.00	0.00	down
0.00	0.00	down
0.00	0.00	up
0.00	0.00	up
0.00	0.00	up
0.00	0.01	down
0.01	0.02	up
0.02	0.05	down
