# Supplementary material for: Transcriptome reveals differential expression of flavor and color in closely related strains of tomato (Solanum lycopersicum)
Source: PeerJ. 2025 Oct 7;13:e20113. doi: 10.7717/peerj.20113 (PMC12513376; doi:10.7717/peerj.20113)
Supplement: Supplemental Information 9 [file peerj-13-20113-s009.pdf]

Table S7: KEGG pathways of differentially expressed genes in Br19-vs-Br20.

Pathway_	KEGG_L	KEGG_L	Pathway_	P.value	Q.value	gene_id	gene_nam	fc
sly01100	Metabolisr	Global and	Metabolic	0.00	0.00	Solyc10g0	Solyc10g0	0.03
sly01100	Metabolisr	Global and	Metabolic	0.00	0.00	Solyc09g0	Solyc09g0	0.01
sly01100	Metabolisr	Global and	Metabolic	0.00	0.00	Solyc02g0	Solyc02g0	0.23
sly01100	Metabolisr	Global and	Metabolic	0.00	0.00	Solyc02g0	Solyc02g0	0.02
sly01100	Metabolisr	Global and	Metabolic	0.00	0.00	Solyc05g0	Solyc05g0	0.01
sly01100	Metabolisr	Global and	Metabolic	0.00	0.00	Solyc11g0	AOS2	0.12
sly01100	Metabolisr	Global and	Metabolic	0.00	0.00	Solyc01g0	Solyc01g0	0.27
sly01100	Metabolisr	Global and	Metabolic	0.00	0.00	Solyc11g0	Solyc11g0	0.11
sly01100	Metabolisr	Global and	Metabolic	0.00	0.00	Solyc09g0	Solyc09g0	0.27
sly01100	Metabolisr	Global and	Metabolic	0.00	0.00	Solyc06g0	Solyc06g0	2.65
sly01100	Metabolisr	Global and	Metabolic	0.00	0.00	Solyc01g1	Solyc01g1	2.32
sly01100	Metabolisr	Global and	Metabolic	0.00	0.00	Solyc07g0	Solyc07g0	0.47
sly01100	Metabolisr	Global and	Metabolic	0.00	0.00	Solyc07g0	NCED1	0.30
sly01100	Metabolisr	Global and	Metabolic	0.00	0.00	Solyc10g0	Solyc10g0	0.06
sly01100	Metabolisr	Global and	Metabolic	0.00	0.00	Solyc07g0	Solyc07g0	0.16
sly01100	Metabolisr	Global and	Metabolic	0.00	0.00	Solyc01g0	Solyc01g0	0.31
sly01100	Metabolisr	Global and	Metabolic	0.00	0.00	Solyc12g0	Solyc12g0	0.34
sly01100	Metabolisr	Global and	Metabolic	0.00	0.00	Solyc12g0	Solyc12g0	0.40
sly01100	Metabolisr	Global and	Metabolic	0.00	0.00	Solyc05g0	Solyc05g0	0.22
sly01100	Metabolisr	Global and	Metabolic	0.00	0.00	Solyc02g0	CHI3	0.24
sly01100	Metabolisr	Global and	Metabolic	0.00	0.00	Solyc05g0	Solyc05g0	2.01
sly01100	Metabolisr	Global and	Metabolic	0.00	0.00	Solyc03g0	Solyc03g0	0.11
sly01100	Metabolisr	Global and	Metabolic	0.00	0.00	Solyc05g0	Solyc05g0	2.02
sly01100	Metabolisr	Global and	Metabolic	0.00	0.00	Solyc08g0	Solyc08g0	0.21
sly01100	Metabolisr	Global and	Metabolic	0.00	0.00	Solyc03g0	Solyc03g0	0.18
sly01100	Metabolisr	Global and	Metabolic	0.00	0.00	Solyc04g0	Solyc04g0	0.48
sly01100	Metabolisr	Global and	Metabolic	0.00	0.00	Solyc03g0	RBCS-2A	0.34
sly01100	Metabolisr	Global and	Metabolic	0.00	0.00	Solyc07g0	Solyc07g0	0.49
sly01100	Metabolisr	Global and	Metabolic	0.00	0.00	Solyc03g0	Solyc03g0	0.15
sly01100	Metabolisr	Global and	Metabolic	0.00	0.00	Solyc05g0	Solyc05g0	0.37
sly01100	Metabolisr	Global and	Metabolic	0.00	0.00	Solyc05g0	Solyc05g0	0.45
sly01100	Metabolisr	Global and	Metabolic	0.00	0.00	Solyc04g0	Solyc04g0	2.15
sly01100	Metabolisr	Global and	Metabolic	0.00	0.00	Solyc01g0	Solyc01g0	0.29
sly01100	Metabolisr	Global and	Metabolic	0.00	0.00	Solyc01g1	Solyc01g1	0.28
sly01100	Metabolisr	Global and	Metabolic	0.00	0.00	Solyc08g0	Solyc08g0	0.34
sly01100	Metabolisr	Global and	Metabolic	0.00	0.00	Solyc02g0	Solyc02g0	0.36
sly01100	Metabolisr	Global and	Metabolic	0.00	0.00	Solyc03g1	Solyc03g1	0.41
sly01100	Metabolisr	Global and	Metabolic	0.00	0.00	Solyc05g0	Solyc05g0	0.37
sly01100	Metabolisr	Global and	Metabolic	0.00	0.00	Solyc07g0	Solyc07g0	2.29
sly01100	Metabolisr	Global and	Metabolic	0.00	0.00	Solyc09g0	Solyc09g0	0.40
sly01100	Metabolisr	Global and	Metabolic	0.00	0.00	Solyc04g0	Solyc04g0	0.41
sly01100	Metabolisr	Global and	Metabolic	0.00	0.00	Solyc11g0	Solyc11g0	0.49
sly01100	Metabolisr	Global and	Metabolic	0.00	0.00	Solyc06g0	psaD	0.43
sly01100	Metabolisr	Global and	Metabolic	0.00	0.00	Solyc01g0	Solyc01g0	0.41
sly01100	Metabolisr	Global and	Metabolic	0.00	0.00	Solyc06g0	Solyc06g0	0.22
sly01100	Metabolisr	Global and	Metabolic	0.00	0.00	Solyc02g0	Solyc02g0	0.36
sly01100	Metabolisr	Global and	Metabolic	0.00	0.00	Solyc01g1	Solyc01g1	0.31
sly01100	Metabolisr	Global and	Metabolic	0.00	0.00	Solyc05g0	Solyc05g0	0.15
sly01100	Metabolisr	Global and	Metabolic	0.00	0.00	Solyc05g0	Solyc05g0	0.44

sly01100	Metabolisr	Global and	Metabolic	0.00	0.00	Solyc01g0	Solyc01g0	0.29
sly01100	Metabolisr	Global and	Metabolic	0.00	0.00	Solyc11g0	Solyc11g0	6.34
sly01100	Metabolisr	Global and	Metabolic	0.00	0.00	Solyc09g0	Solyc09g0	0.44
sly01100	Metabolisr	Global and	Metabolic	0.00	0.00	Solyc11g0	Solyc11g0	0.33
sly01100	Metabolisr	Global and	Metabolic	0.00	0.00	Solyc10g0	Solyc10g0	0.49
sly01100	Metabolisr	Global and	Metabolic	0.00	0.00	Solyc05g0	Solyc05g0	0.26
sly01100	Metabolisr	Global and	Metabolic	0.00	0.00	Solyc03g1	Solyc03g1	0.46
sly01100	Metabolisr	Global and	Metabolic	0.00	0.00	Solyc10g0	CHI9	0.27
sly01100	Metabolisr	Global and	Metabolic	0.00	0.00	Solyc05g0	Solyc05g0	0.26
sly01100	Metabolisr	Global and	Metabolic	0.00	0.00	Solyc07g0	OPR3	0.47
sly01100	Metabolisr	Global and	Metabolic	0.00	0.00	Solyc02g0	Solyc02g0	0.49
sly01100	Metabolisr	Global and	Metabolic	0.00	0.00	Solyc12g0	Solyc12g0	0.43
sly01100	Metabolisr	Global and	Metabolic	0.00	0.00	Solyc01g0	Solyc01g0	0.45
sly01100	Metabolisr	Global and	Metabolic	0.00	0.00	Solyc02g0	Solyc02g0	5.84
sly01100	Metabolisr	Global and	Metabolic	0.00	0.00	Solyc08g0	Solyc08g0	0.11
sly01100	Metabolisr	Global and	Metabolic	0.00	0.00	Solyc01g0	Solyc01g0	0.18
sly01100	Metabolisr	Global and	Metabolic	0.00	0.00	Solyc06g0	Solyc06g0	2.01
sly01100	Metabolisr	Global and	Metabolic	0.00	0.00	Solyc02g0	Solyc02g0	2.89
sly01100	Metabolisr	Global and	Metabolic	0.00	0.00	Solyc08g0	Solyc08g0	0.47
sly01100	Metabolisr	Global and	Metabolic	0.00	0.00	Solyc11g0	Solyc11g0	2.55
sly01100	Metabolisr	Global and	Metabolic	0.00	0.00	Solyc02g0	Solyc02g0	0.36
sly01100	Metabolisr	Global and	Metabolic	0.00	0.00	Solyc12g0	Solyc12g0	2112.44
sly01100	Metabolisr	Global and	Metabolic	0.00	0.00	Solyc01g0	Solyc01g0	0.42
sly01100	Metabolisr	Global and	Metabolic	0.00	0.00	Solyc06g0	Solyc06g0	0.24
sly01100	Metabolisr	Global and	Metabolic	0.00	0.00	Solyc02g0	Solyc02g0	0.46
sly01100	Metabolisr	Global and	Metabolic	0.00	0.00	Solyc08g0	Solyc08g0	0.44
sly01100	Metabolisr	Global and	Metabolic	0.00	0.00	Solyc03g0	Solyc03g0	0.00
sly01100	Metabolisr	Global and	Metabolic	0.00	0.00	Solyc07g0	Solyc07g0	0.45
sly01100	Metabolisr	Global and	Metabolic	0.00	0.00	Solyc01g0	Solyc01g0	2.13
sly01100	Metabolisr	Global and	Metabolic	0.00	0.00	Solyc09g0	Solyc09g0	0.34
sly01100	Metabolisr	Global and	Metabolic	0.00	0.00	Solyc01g1	Solyc01g1	2.42
sly01100	Metabolisr	Global and	Metabolic	0.00	0.00	Solyc01g0	Solyc01g0	4.89
sly01100	Metabolisr	Global and	Metabolic	0.00	0.00	Solyc12g0	Solyc12g0	0.30
sly01100	Metabolisr	Global and	Metabolic	0.00	0.00	Solyc08g0	Solyc08g0	0.00
sly01100	Metabolisr	Global and	Metabolic	0.00	0.00	Solyc06g0	Solyc06g0	0.39
sly01100	Metabolisr	Global and	Metabolic	0.00	0.00	Solyc09g0	Solyc09g0	8.52
sly01100	Metabolisr	Global and	Metabolic	0.00	0.00	Solyc06g0	Solyc06g0	0.30
sly01100	Metabolisr	Global and	Metabolic	0.00	0.00	Solyc09g0	Solyc09g0	2.43
sly01100	Metabolisr	Global and	Metabolic	0.00	0.00	Solyc07g0	Solyc07g0	0.34
sly01100	Metabolisr	Global and	Metabolic	0.00	0.00	Solyc05g0	Solyc05g0	0.00
sly01100	Metabolisr	Global and	Metabolic	0.00	0.00	Solyc12g0	Solyc12g0	0.13
sly01100	Metabolisr	Global and	Metabolic	0.00	0.00	Solyc01g1	Solyc01g1	2.95
sly01100	Metabolisr	Global and	Metabolic	0.00	0.00	Solyc11g0	Solyc11g0	0.38
sly01100	Metabolisr	Global and	Metabolic	0.00	0.00	Solyc03g1	Solyc03g1	0.34
sly01100	Metabolisr	Global and	Metabolic	0.00	0.00	Solyc09g0	TD2	4.53
sly01100	Metabolisr	Global and	Metabolic	0.00	0.00	Solyc08g0	Solyc08g0	799.01
sly01100	Metabolisr	Global and	Metabolic	0.00	0.00	Solyc03g0	Solyc03g0	2.14
sly01100	Metabolisr	Global and	Metabolic	0.00	0.00	Solyc06g0	Solyc06g0	9.98
sly01100	Metabolisr	Global and	Metabolic	0.00	0.00	Solyc01g1	CAP10A	0.35
sly01100	Metabolisr	Global and	Metabolic	0.00	0.00	Solyc02g0	Solyc02g0	2.29
sly01100	Metabolisr	Global and	Metabolic	0.00	0.00	Solyc01g0	Solyc01g0	2.28

sly01100	Metabolisr	Global and	Metabolic	0.00	0.00	Solyc09g0	Solyc09g0	3.02
sly01100	Metabolisr	Global and	Metabolic	0.00	0.00	Solyc01g1	Solyc01g1	0.00
sly01100	Metabolisr	Global and	Metabolic	0.00	0.00	Solyc04g0	Solyc04g0	0.27
sly01100	Metabolisr	Global and	Metabolic	0.00	0.00	Solyc07g0	Solyc07g0	0.37
sly01100	Metabolisr	Global and	Metabolic	0.00	0.00	Solyc03g0	Solyc03g0	0.04
sly01100	Metabolisr	Global and	Metabolic	0.00	0.00	Solyc02g0	Solyc02g0	0.10
sly01100	Metabolisr	Global and	Metabolic	0.00	0.00	Solyc11g0	Solyc11g0	0.47
sly01100	Metabolisr	Global and	Metabolic	0.00	0.00	Solyc10g0	Solyc10g0	0.28
sly01100	Metabolisr	Global and	Metabolic	0.00	0.00	Solyc06g0	Solyc06g0	0.00
sly00941	Metabolisr	Biosynthes	Flavonoid	0.00	0.00	Solyc09g0	Solyc09g0	0.01
sly00941	Metabolisr	Biosynthes	Flavonoid	0.00	0.00	Solyc02g0	Solyc02g0	0.02
sly00941	Metabolisr	Biosynthes	Flavonoid	0.00	0.00	Solyc05g0	Solyc05g0	0.01
sly00941	Metabolisr	Biosynthes	Flavonoid	0.00	0.00	Solyc11g0	Solyc11g0	0.11
sly00941	Metabolisr	Biosynthes	Flavonoid	0.00	0.00	Solyc06g0	Solyc06g0	2.65
sly00941	Metabolisr	Biosynthes	Flavonoid	0.00	0.00	Solyc05g0	Solyc05g0	0.22
sly00941	Metabolisr	Biosynthes	Flavonoid	0.00	0.00	Solyc01g0	Solyc01g0	2.13
sly00941	Metabolisr	Biosynthes	Flavonoid	0.00	0.00	Solyc01g0	Solyc01g0	2.28
sly00941	Metabolisr	Biosynthes	Flavonoid	0.00	0.00	Solyc01g1	Solyc01g1	0.00
sly00941	Metabolisr	Biosynthes	Flavonoid	0.00	0.00	Solyc04g0	Solyc04g0	0.27
sly00941	Metabolisr	Biosynthes	Flavonoid	0.00	0.00	Solyc03g0	Solyc03g0	0.04
sly00195	Metabolisr	Energy me	Photosyntl	0.00	0.00	Solyc01g1	Solyc01g1	0.46
sly00195	Metabolisr	Energy me	Photosyntl	0.00	0.00	Solyc02g0	PSBO	0.48
sly00195	Metabolisr	Energy me	Photosyntl	0.00	0.00	Solyc02g0	Solyc02g0	0.35
sly00195	Metabolisr	Energy me	Photosyntl	0.00	0.00	Solyc06g0	psaD	0.43
sly00195	Metabolisr	Energy me	Photosyntl	0.00	0.00	Solyc06g0	Solyc06g0	0.20
sly00195	Metabolisr	Energy me	Photosyntl	0.00	0.00	Solyc06g0	Solyc06g0	0.24
sly00195	Metabolisr	Energy me	Photosyntl	0.00	0.00	Solyc08g0	Solyc08g0	0.21
sly00195	Metabolisr	Energy me	Photosyntl	0.00	0.00	Solyc06g0	Solyc06g0	0.31
sly00195	Metabolisr	Energy me	Photosyntl	0.00	0.00	Solyc06g0	Solyc06g0	0.06
sly01110	Metabolisr	Global and	Biosynthes	0.00	0.01	Solyc10g0	Solyc10g0	0.03
sly01110	Metabolisr	Global and	Biosynthes	0.00	0.01	Solyc09g0	Solyc09g0	0.01
sly01110	Metabolisr	Global and	Biosynthes	0.00	0.01	Solyc07g0	Solyc07g0	0.17
sly01110	Metabolisr	Global and	Biosynthes	0.00	0.01	Solyc02g0	Solyc02g0	0.23
sly01110	Metabolisr	Global and	Biosynthes	0.00	0.01	Solyc02g0	Solyc02g0	0.02
sly01110	Metabolisr	Global and	Biosynthes	0.00	0.01	Solyc05g0	Solyc05g0	0.01
sly01110	Metabolisr	Global and	Biosynthes	0.00	0.01	Solyc11g0	AOS2	0.12
sly01110	Metabolisr	Global and	Biosynthes	0.00	0.01	Solyc11g0	Solyc11g0	0.11
sly01110	Metabolisr	Global and	Biosynthes	0.00	0.01	Solyc06g0	Solyc06g0	2.65
sly01110	Metabolisr	Global and	Biosynthes	0.00	0.01	Solyc07g0	NCED1	0.30
sly01110	Metabolisr	Global and	Biosynthes	0.00	0.01	Solyc07g0	Solyc07g0	0.16
sly01110	Metabolisr	Global and	Biosynthes	0.00	0.01	Solyc12g0	Solyc12g0	0.40
sly01110	Metabolisr	Global and	Biosynthes	0.00	0.01	Solyc05g0	Solyc05g0	0.22
sly01110	Metabolisr	Global and	Biosynthes	0.00	0.01	Solyc05g0	Solyc05g0	2.01
sly01110	Metabolisr	Global and	Biosynthes	0.00	0.01	Solyc03g0	Solyc03g0	0.11
sly01110	Metabolisr	Global and	Biosynthes	0.00	0.01	Solyc05g0	Solyc05g0	2.02
sly01110	Metabolisr	Global and	Biosynthes	0.00	0.01	Solyc08g0	Solyc08g0	0.21
sly01110	Metabolisr	Global and	Biosynthes	0.00	0.01	Solyc04g0	Solyc04g0	0.48
sly01110	Metabolisr	Global and	Biosynthes	0.00	0.01	Solyc03g0	RBCS-2A	0.34
sly01110	Metabolisr	Global and	Biosynthes	0.00	0.01	Solyc07g0	Solyc07g0	0.49
sly01110	Metabolisr	Global and	Biosynthes	0.00	0.01	Solyc05g0	Solyc05g0	0.37
sly01110	Metabolisr	Global and	Biosynthes	0.00	0.01	Solyc05g0	Solyc05g0	0.45

sly01110	Metabolisr	Global and	Biosynthes	0.00	0.01	Solyc04g0	Solyc04g0	2.15
sly01110	Metabolisr	Global and	Biosynthes	0.00	0.01	Solyc11g0	Solyc11g0	0.16
sly01110	Metabolisr	Global and	Biosynthes	0.00	0.01	Solyc01g1	Solyc01g1	0.28
sly01110	Metabolisr	Global and	Biosynthes	0.00	0.01	Solyc08g0	Solyc08g0	0.34
sly01110	Metabolisr	Global and	Biosynthes	0.00	0.01	Solyc02g0	Solyc02g0	0.36
sly01110	Metabolisr	Global and	Biosynthes	0.00	0.01	Solyc09g0	Solyc09g0	0.40
sly01110	Metabolisr	Global and	Biosynthes	0.00	0.01	Solyc04g0	Solyc04g0	0.41
sly01110	Metabolisr	Global and	Biosynthes	0.00	0.01	Solyc01g0	Solyc01g0	0.41
sly01110	Metabolisr	Global and	Biosynthes	0.00	0.01	Solyc02g0	Solyc02g0	0.36
sly01110	Metabolisr	Global and	Biosynthes	0.00	0.01	Solyc01g1	Solyc01g1	0.31
sly01110	Metabolisr	Global and	Biosynthes	0.00	0.01	Solyc05g0	Solyc05g0	0.44
sly01110	Metabolisr	Global and	Biosynthes	0.00	0.01	Solyc01g0	Solyc01g0	0.29
sly01110	Metabolisr	Global and	Biosynthes	0.00	0.01	Solyc11g0	Solyc11g0	6.34
sly01110	Metabolisr	Global and	Biosynthes	0.00	0.01	Solyc11g0	Solyc11g0	0.33
sly01110	Metabolisr	Global and	Biosynthes	0.00	0.01	Solyc03g1	Solyc03g1	0.46
sly01110	Metabolisr	Global and	Biosynthes	0.00	0.01	Solyc07g0	OPR3	0.47
sly01110	Metabolisr	Global and	Biosynthes	0.00	0.01	Solyc12g0	Solyc12g0	0.43
sly01110	Metabolisr	Global and	Biosynthes	0.00	0.01	Solyc02g0	Solyc02g0	5.84
sly01110	Metabolisr	Global and	Biosynthes	0.00	0.01	Solyc08g0	Solyc08g0	0.47
sly01110	Metabolisr	Global and	Biosynthes	0.00	0.01	Solyc02g0	Solyc02g0	0.36
sly01110	Metabolisr	Global and	Biosynthes	0.00	0.01	Solyc01g0	Solyc01g0	0.42
sly01110	Metabolisr	Global and	Biosynthes	0.00	0.01	Solyc08g0	Solyc08g0	0.44
sly01110	Metabolisr	Global and	Biosynthes	0.00	0.01	Solyc03g0	Solyc03g0	0.00
sly01110	Metabolisr	Global and	Biosynthes	0.00	0.01	Solyc01g0	Solyc01g0	2.13
sly01110	Metabolisr	Global and	Biosynthes	0.00	0.01	Solyc12g0	Solyc12g0	0.30
sly01110	Metabolisr	Global and	Biosynthes	0.00	0.01	Solyc06g0	Solyc06g0	0.39
sly01110	Metabolisr	Global and	Biosynthes	0.00	0.01	Solyc09g0	Solyc09g0	8.52
sly01110	Metabolisr	Global and	Biosynthes	0.00	0.01	Solyc06g0	Solyc06g0	0.30
sly01110	Metabolisr	Global and	Biosynthes	0.00	0.01	Solyc09g0	Solyc09g0	2.43
sly01110	Metabolisr	Global and	Biosynthes	0.00	0.01	Solyc01g1	Solyc01g1	2.95
sly01110	Metabolisr	Global and	Biosynthes	0.00	0.01	Solyc11g0	Solyc11g0	0.38
sly01110	Metabolisr	Global and	Biosynthes	0.00	0.01	Solyc09g0	TD2	4.53
sly01110	Metabolisr	Global and	Biosynthes	0.00	0.01	Solyc03g0	Solyc03g0	2.14
sly01110	Metabolisr	Global and	Biosynthes	0.00	0.01	Solyc06g0	Solyc06g0	9.98
sly01110	Metabolisr	Global and	Biosynthes	0.00	0.01	Solyc02g0	Solyc02g0	2.29
sly01110	Metabolisr	Global and	Biosynthes	0.00	0.01	Solyc01g0	Solyc01g0	2.28
sly01110	Metabolisr	Global and	Biosynthes	0.00	0.01	Solyc09g0	Solyc09g0	3.02
sly01110	Metabolisr	Global and	Biosynthes	0.00	0.01	Solyc01g1	Solyc01g1	0.00
sly01110	Metabolisr	Global and	Biosynthes	0.00	0.01	Solyc04g0	Solyc04g0	0.27
sly01110	Metabolisr	Global and	Biosynthes	0.00	0.01	Solyc03g0	Solyc03g0	0.04
sly01110	Metabolisr	Global and	Biosynthes	0.00	0.01	Solyc02g0	Solyc02g0	0.10
sly01110	Metabolisr	Global and	Biosynthes	0.00	0.01	Solyc10g0	Solyc10g0	0.28
sly01110	Metabolisr	Global and	Biosynthes	0.00	0.01	Solyc06g0	Solyc06g0	0.00
sly00940	Metabolisr	Biosynthes	Phenylpro|	0.00	0.01	Solyc06g0	Solyc06g0	2.65
sly00940	Metabolisr	Biosynthes	Phenylpro|	0.00	0.01	Solyc05g0	Solyc05g0	0.22
sly00940	Metabolisr	Biosynthes	Phenylpro|	0.00	0.01	Solyc03g0	Solyc03g0	0.11
sly00940	Metabolisr	Biosynthes	Phenylpro|	0.00	0.01	Solyc02g0	Solyc02g0	5.84
sly00940	Metabolisr	Biosynthes	Phenylpro|	0.00	0.01	Solyc01g0	Solyc01g0	2.13
sly00940	Metabolisr	Biosynthes	Phenylpro|	0.00	0.01	Solyc09g0	Solyc09g0	2.43
sly00940	Metabolisr	Biosynthes	Phenylpro|	0.00	0.01	Solyc01g1	Solyc01g1	2.95
sly00940	Metabolisr	Biosynthes	Phenylpro|	0.00	0.01	Solyc03g0	Solyc03g0	2.14

sly00940	Metabolisr	Biosynthes	Phenylpro[	0.00	0.01	Solyc06g0	Solyc06g0	9.98
sly00940	Metabolisr	Biosynthes	Phenylpro[	0.00	0.01	Solyc02g0	Solyc02g0	2.29
sly00940	Metabolisr	Biosynthes	Phenylpro[	0.00	0.01	Solyc01g0	Solyc01g0	2.28
sly00940	Metabolisr	Biosynthes	Phenylpro[	0.00	0.01	Solyc09g0	Solyc09g0	3.02
sly00940	Metabolisr	Biosynthes	Phenylpro[	0.00	0.01	Solyc01g1	Solyc01g1	0.00
sly00940	Metabolisr	Biosynthes	Phenylpro[	0.00	0.01	Solyc04g0	Solyc04g0	0.27
sly00940	Metabolisr	Biosynthes	Phenylpro[	0.00	0.01	Solyc03g0	Solyc03g0	0.04
sly00940	Metabolisr	Biosynthes	Phenylpro[	0.00	0.01	Solyc02g0	Solyc02g0	0.10
sly00196	Metabolisr	Energy me	Photosyntl	0.00	0.01	Solyc03g0	Solyc03g0	0.18
sly00196	Metabolisr	Energy me	Photosyntl	0.00	0.01	Solyc05g0	Solyc05g0	0.15
sly00196	Metabolisr	Energy me	Photosyntl	0.00	0.01	Solyc10g0	Solyc10g0	0.49
sly00196	Metabolisr	Energy me	Photosyntl	0.00	0.01	Solyc05g0	Solyc05g0	0.00
sly00196	Metabolisr	Energy me	Photosyntl	0.00	0.01	Solyc01g1	CAP10A	0.35
sly00071	Metabolisr	Lipid meta	Fatty acid	0.00	0.01	Solyc08g0	Solyc08g0	0.21
sly00071	Metabolisr	Lipid meta	Fatty acid	0.00	0.01	Solyc04g0	Solyc04g0	0.48
sly00071	Metabolisr	Lipid meta	Fatty acid	0.00	0.01	Solyc01g0	Solyc01g0	0.41
sly00071	Metabolisr	Lipid meta	Fatty acid	0.00	0.01	Solyc03g1	Solyc03g1	0.46
sly00071	Metabolisr	Lipid meta	Fatty acid	0.00	0.01	Solyc08g0	Solyc08g0	0.44
sly00071	Metabolisr	Lipid meta	Fatty acid	0.00	0.01	Solyc06g0	Solyc06g0	0.39
sly00945	Metabolisr	Biosynthes	Stilbenoid,	0.00	0.01	Solyc06g0	Solyc06g0	2.65
sly00945	Metabolisr	Biosynthes	Stilbenoid,	0.00	0.01	Solyc05g0	Solyc05g0	0.22
sly00945	Metabolisr	Biosynthes	Stilbenoid,	0.00	0.01	Solyc01g0	Solyc01g0	2.13
sly00945	Metabolisr	Biosynthes	Stilbenoid,	0.00	0.01	Solyc01g0	Solyc01g0	2.28
sly00945	Metabolisr	Biosynthes	Stilbenoid,	0.00	0.01	Solyc01g1	Solyc01g1	0.00
sly00945	Metabolisr	Biosynthes	Stilbenoid,	0.00	0.01	Solyc04g0	Solyc04g0	0.27
sly00945	Metabolisr	Biosynthes	Stilbenoid,	0.00	0.01	Solyc03g0	Solyc03g0	0.04
sly00730	Metabolisr	Metabolisr	Thiamine r	0.01	0.10	Solyc07g0	Solyc07g0	0.47
sly00730	Metabolisr	Metabolisr	Thiamine r	0.01	0.10	Solyc11g0	Solyc11g0	0.49
sly00730	Metabolisr	Metabolisr	Thiamine r	0.01	0.10	Solyc11g0	Solyc11g0	0.38
sly00730	Metabolisr	Metabolisr	Thiamine r	0.01	0.10	Solyc11g0	Solyc11g0	0.47
sly00592	Metabolisr	Lipid meta	alpha-Lino	0.01	0.10	Solyc11g0	AOS2	0.12
sly00592	Metabolisr	Lipid meta	alpha-Lino	0.01	0.10	Solyc08g0	Solyc08g0	0.21
sly00592	Metabolisr	Lipid meta	alpha-Lino	0.01	0.10	Solyc04g0	Solyc04g0	0.48
sly00592	Metabolisr	Lipid meta	alpha-Lino	0.01	0.10	Solyc07g0	OPR3	0.47
sly00592	Metabolisr	Lipid meta	alpha-Lino	0.01	0.10	Solyc02g0	Solyc02g0	0.00
sly00520	Metabolisr	Carbohydr	Amino sug	0.04	0.31	Solyc01g1	Solyc01g1	2.32
sly00520	Metabolisr	Carbohydr	Amino sug	0.04	0.31	Solyc10g0	Solyc10g0	0.06
sly00520	Metabolisr	Carbohydr	Amino sug	0.04	0.31	Solyc02g0	CHI3	0.24
sly00520	Metabolisr	Carbohydr	Amino sug	0.04	0.31	Solyc08g0	Solyc08g0	0.34
sly00520	Metabolisr	Carbohydr	Amino sug	0.04	0.31	Solyc07g0	Solyc07g0	2.29
sly00520	Metabolisr	Carbohydr	Amino sug	0.04	0.31	Solyc11g0	Solyc11g0	0.33
sly00520	Metabolisr	Carbohydr	Amino sug	0.04	0.31	Solyc05g0	Solyc05g0	0.26
sly00520	Metabolisr	Carbohydr	Amino sug	0.04	0.31	Solyc10g0	CHI9	0.27
sly00520	Metabolisr	Carbohydr	Amino sug	0.04	0.31	Solyc07g0	Solyc07g0	0.45
sly00520	Metabolisr	Carbohydr	Amino sug	0.04	0.31	Solyc07g0	Solyc07g0	0.37
sly00561	Metabolisr	Lipid meta	Glycerolip	0.07	0.45	Solyc12g0	Solyc12g0	0.34
sly00561	Metabolisr	Lipid meta	Glycerolip	0.07	0.45	Solyc01g0	Solyc01g0	0.41
sly00561	Metabolisr	Lipid meta	Glycerolip	0.07	0.45	Solyc11g0	Solyc11g0	6.34
sly00561	Metabolisr	Lipid meta	Glycerolip	0.07	0.45	Solyc03g1	Solyc03g1	0.46
sly00561	Metabolisr	Lipid meta	Glycerolip	0.07	0.45	Solyc07g0	Solyc07g0	0.34
sly00561	Metabolisr	Lipid meta	Glycerolip	0.07	0.45	Solyc03g1	Solyc03g1	0.34

sly00903	Metabolisr	Metabolisr	Limonene	0.07	0.45	Solyc01g0	Solyc01g0	0.41
sly00903	Metabolisr	Metabolisr	Limonene	0.07	0.45	Solyc03g1	Solyc03g1	0.46
sly04626	Organisma	Environme	Plant-pathc	0.08	0.45	Solyc11g0	Solyc11g0	0.46
sly04626	Organisma	Environme	Plant-pathc	0.08	0.45	Solyc07g0	EIX1	0.33
sly04626	Organisma	Environme	Plant-pathc	0.08	0.45	Solyc01g0	Solyc01g0	0.00
sly04626	Organisma	Environme	Plant-pathc	0.08	0.45	Solyc09g0	Solyc09g0	8.52
sly04626	Organisma	Environme	Plant-pathc	0.08	0.45	Solyc11g0	Solyc11g0	2.48
sly04626	Organisma	Environme	Plant-pathc	0.08	0.45	Solyc06g0	Solyc06g0	0.48
sly04626	Organisma	Environme	Plant-pathc	0.08	0.45	Solyc09g0	Solyc09g0	0.43
sly04626	Organisma	Environme	Plant-pathc	0.08	0.45	Solyc03g1	Solyc03g1	0.34
sly04626	Organisma	Environme	Plant-pathc	0.08	0.45	Solyc01g0	Solyc01g0	2.66
sly04626	Organisma	Environme	Plant-pathc	0.08	0.45	Solyc02g0	Solyc02g0	0.49
sly04626	Organisma	Environme	Plant-pathc	0.08	0.45	Solyc02g0	PTI5	0.07
sly04626	Organisma	Environme	Plant-pathc	0.08	0.45	Solyc06g0	Solyc06g0	3.20
sly00380	Metabolisr	Amino acid	Tryptophar	0.08	0.45	Solyc01g0	Solyc01g0	0.41
sly00380	Metabolisr	Amino acid	Tryptophar	0.08	0.45	Solyc03g1	Solyc03g1	0.46
sly00380	Metabolisr	Amino acid	Tryptophar	0.08	0.45	Solyc06g0	Solyc06g0	2.01
sly00380	Metabolisr	Amino acid	Tryptophar	0.08	0.45	Solyc02g0	Solyc02g0	5.03
sly04016	Environme	Signal tran	MAPK sig	0.09	0.45	Solyc10g0	Solyc10g0	0.06
sly04016	Environme	Signal tran	MAPK sig	0.09	0.45	Solyc02g0	CHI3	0.24
sly04016	Environme	Signal tran	MAPK sig	0.09	0.45	Solyc08g0	Solyc08g0	0.39
sly04016	Environme	Signal tran	MAPK sig	0.09	0.45	Solyc10g0	CHI9	0.27
sly04016	Environme	Signal tran	MAPK sig	0.09	0.45	Solyc06g0	Solyc06g0	0.41
sly04016	Environme	Signal tran	MAPK sig	0.09	0.45	Solyc01g0	Solyc01g0	0.35
sly04016	Environme	Signal tran	MAPK sig	0.09	0.45	Solyc06g0	Solyc06g0	0.48
sly04016	Environme	Signal tran	MAPK sig	0.09	0.45	Solyc06g0	Solyc06g0	0.39
sly04016	Environme	Signal tran	MAPK sig	0.09	0.45	Solyc04g0	Solyc04g0	0.34
sly04016	Environme	Signal tran	MAPK sig	0.09	0.45	Solyc09g0	Solyc09g0	0.49
sly00906	Metabolisr	Metabolisr	Carotenoic	0.09	0.45	Solyc07g0	NCED1	0.30
sly00906	Metabolisr	Metabolisr	Carotenoic	0.09	0.45	Solyc08g0	CYP707A1	0.26
sly00906	Metabolisr	Metabolisr	Carotenoic	0.09	0.45	Solyc10g0	Solyc10g0	0.28
sly00710	Metabolisr	Energy me	Carbon fix	0.10	0.45	Solyc03g0	RBCS-2A	0.34
sly00710	Metabolisr	Energy me	Carbon fix	0.10	0.45	Solyc01g1	Solyc01g1	0.31
sly00710	Metabolisr	Energy me	Carbon fix	0.10	0.45	Solyc05g0	Solyc05g0	0.44
sly00710	Metabolisr	Energy me	Carbon fix	0.10	0.45	Solyc02g0	Solyc02g0	0.46
sly00710	Metabolisr	Energy me	Carbon fix	0.10	0.45	Solyc12g0	Solyc12g0	0.13
sly00053	Metabolisr	Carbohydr	Ascorbate	0.10	0.47	Solyc07g0	Solyc07g0	2.29
sly00053	Metabolisr	Carbohydr	Ascorbate	0.10	0.47	Solyc01g0	Solyc01g0	0.41
sly00053	Metabolisr	Carbohydr	Ascorbate	0.10	0.47	Solyc03g1	Solyc03g1	0.46
sly00053	Metabolisr	Carbohydr	Ascorbate	0.10	0.47	Solyc01g0	Solyc01g0	0.18
sly00053	Metabolisr	Carbohydr	Ascorbate	0.10	0.47	Solyc02g0	Solyc02g0	2.89
sly00340	Metabolisr	Amino acid	Histidine r	0.13	0.57	Solyc01g0	Solyc01g0	0.41
sly00340	Metabolisr	Amino acid	Histidine r	0.13	0.57	Solyc03g1	Solyc03g1	0.46
sly00010	Metabolisr	Carbohydr	Glycolysis	0.15	0.59	Solyc05g0	Solyc05g0	2.02
sly00010	Metabolisr	Carbohydr	Glycolysis	0.15	0.59	Solyc04g0	Solyc04g0	0.48
sly00010	Metabolisr	Carbohydr	Glycolysis	0.15	0.59	Solyc01g0	Solyc01g0	0.41
sly00010	Metabolisr	Carbohydr	Glycolysis	0.15	0.59	Solyc01g1	Solyc01g1	0.31
sly00010	Metabolisr	Carbohydr	Glycolysis	0.15	0.59	Solyc11g0	Solyc11g0	0.33
sly00010	Metabolisr	Carbohydr	Glycolysis	0.15	0.59	Solyc03g1	Solyc03g1	0.46
sly00010	Metabolisr	Carbohydr	Glycolysis	0.15	0.59	Solyc08g0	Solyc08g0	0.44
sly00010	Metabolisr	Carbohydr	Glycolysis	0.15	0.59	Solyc06g0	Solyc06g0	0.39

sly04712	Organisma	Environme	Circadian r	0.15	0.59	Solyc09g0	Solyc09g0	0.01
sly04712	Organisma	Environme	Circadian r	0.15	0.59	Solyc05g0	Solyc05g0	0.01
sly04712	Organisma	Environme	Circadian r	0.15	0.59	Solyc12g0	Solyc12g0	0.32
sly00100	Metabolisr	Lipid meta	Steroid bic	0.18	0.68	Solyc03g0	Solyc03g0	2.46
sly00100	Metabolisr	Lipid meta	Steroid bic	0.18	0.68	Solyc01g1	Solyc01g1	0.28
sly00100	Metabolisr	Lipid meta	Steroid bic	0.18	0.68	Solyc02g0	Solyc02g0	0.36
sly00020	Metabolisr	Carbohydr	Citrate cyc	0.23	0.74	Solyc05g0	Solyc05g0	2.01
sly00020	Metabolisr	Carbohydr	Citrate cyc	0.23	0.74	Solyc05g0	Solyc05g0	2.02
sly00020	Metabolisr	Carbohydr	Citrate cyc	0.23	0.74	Solyc07g0	Solyc07g0	0.49
sly00020	Metabolisr	Carbohydr	Citrate cyc	0.23	0.74	Solyc04g0	Solyc04g0	0.41
sly00902	Metabolisr	Metabolisr	Monoterpe	0.24	0.74	Solyc11g0	Solyc11g0	0.16
sly00261	Metabolisr	Biosynthes	Monobacta	0.24	0.74	Solyc06g0	Solyc06g0	0.30
sly00350	Metabolisr	Amino aci	Tyrosine n	0.24	0.74	Solyc04g0	Solyc04g0	0.48
sly00350	Metabolisr	Amino aci	Tyrosine n	0.24	0.74	Solyc08g0	Solyc08g0	0.44
sly00350	Metabolisr	Amino aci	Tyrosine n	0.24	0.74	Solyc06g0	Solyc06g0	0.39
sly01200	Metabolisr	Global and	Carbon me	0.25	0.74	Solyc10g0	Solyc10g0	0.03
sly01200	Metabolisr	Global and	Carbon me	0.25	0.74	Solyc05g0	Solyc05g0	2.02
sly01200	Metabolisr	Global and	Carbon me	0.25	0.74	Solyc03g0	RBCS-2A	0.34
sly01200	Metabolisr	Global and	Carbon me	0.25	0.74	Solyc07g0	Solyc07g0	0.49
sly01200	Metabolisr	Global and	Carbon me	0.25	0.74	Solyc04g0	Solyc04g0	0.41
sly01200	Metabolisr	Global and	Carbon me	0.25	0.74	Solyc01g1	Solyc01g1	0.31
sly01200	Metabolisr	Global and	Carbon me	0.25	0.74	Solyc05g0	Solyc05g0	0.44
sly01200	Metabolisr	Global and	Carbon me	0.25	0.74	Solyc11g0	Solyc11g0	0.33
sly01200	Metabolisr	Global and	Carbon me	0.25	0.74	Solyc02g0	Solyc02g0	0.46
sly01200	Metabolisr	Global and	Carbon me	0.25	0.74	Solyc12g0	Solyc12g0	0.30
sly01200	Metabolisr	Global and	Carbon me	0.25	0.74	Solyc12g0	Solyc12g0	0.13
sly01200	Metabolisr	Global and	Carbon me	0.25	0.74	Solyc09g0	TD2	4.53
sly00360	Metabolisr	Amino aci	Phenylalar	0.27	0.74	Solyc08g0	Solyc08g0	0.11
sly00360	Metabolisr	Amino aci	Phenylalar	0.27	0.74	Solyc08g0	Solyc08g0	0.00
sly00360	Metabolisr	Amino aci	Phenylalar	0.27	0.74	Solyc08g0	Solyc08g0	799.01
sly00904	Metabolisr	Metabolisr	Diterpenoi	0.28	0.74	Solyc07g0	Solyc07g0	0.17
sly00904	Metabolisr	Metabolisr	Diterpenoi	0.28	0.74	Solyc01g0	Solyc01g0	0.29
sly00310	Metabolisr	Amino aci	Lysine deg	0.28	0.74	Solyc01g0	Solyc01g0	0.41
sly00310	Metabolisr	Amino aci	Lysine deg	0.28	0.74	Solyc03g1	Solyc03g1	0.46
sly04075	Environme	Signal tran	Plant horm	0.29	0.74	Solyc03g0	Solyc03g0	4.24
sly04075	Environme	Signal tran	Plant horm	0.29	0.74	Solyc11g0	Solyc11g0	0.47
sly04075	Environme	Signal tran	Plant horm	0.29	0.74	Solyc06g0	Solyc06g0	0.41
sly04075	Environme	Signal tran	Plant horm	0.29	0.74	Solyc06g0	Solyc06g0	2.00
sly04075	Environme	Signal tran	Plant horm	0.29	0.74	Solyc01g0	Solyc01g0	0.35
sly04075	Environme	Signal tran	Plant horm	0.29	0.74	Solyc04g0	Solyc04g0	0.18
sly04075	Environme	Signal tran	Plant horm	0.29	0.74	Solyc01g0	Solyc01g0	0.50
sly04075	Environme	Signal tran	Plant horm	0.29	0.74	Solyc08g0	Solyc08g0	0.38
sly04075	Environme	Signal tran	Plant horm	0.29	0.74	Solyc06g0	Solyc06g0	0.39
sly04075	Environme	Signal tran	Plant horm	0.29	0.74	Solyc04g0	Solyc04g0	0.34
sly04075	Environme	Signal tran	Plant horm	0.29	0.74	Solyc09g0	Solyc09g0	0.49
sly00630	Metabolisr	Carbohydr	Glyoxylate	0.32	0.74	Solyc10g0	Solyc10g0	0.03
sly00630	Metabolisr	Carbohydr	Glyoxylate	0.32	0.74	Solyc03g0	RBCS-2A	0.34
sly00630	Metabolisr	Carbohydr	Glyoxylate	0.32	0.74	Solyc07g0	Solyc07g0	0.49
sly00630	Metabolisr	Carbohydr	Glyoxylate	0.32	0.74	Solyc12g0	Solyc12g0	0.30
sly00900	Metabolisr	Metabolisr	Terpenoid	0.33	0.74	Solyc04g0	Solyc04g0	2.15
sly00900	Metabolisr	Metabolisr	Terpenoid	0.33	0.74	Solyc12g0	Solyc12g0	0.43

sly00900	Metabolisr	Metabolisr	Terpenoid	0.33	0.74	Solyc11g0	Solyc11g0	0.38
sly00920	Metabolisr	Energy me	Sulfur met	0.33	0.74	Solyc09g0	Solyc09g0	0.44
sly00920	Metabolisr	Energy me	Sulfur met	0.33	0.74	Solyc02g0	Solyc02g0	0.49
sly00908	Metabolisr	Metabolisr	Zeatin bios	0.33	0.74	Solyc01g0	Solyc01g0	0.42
sly00908	Metabolisr	Metabolisr	Zeatin bios	0.33	0.74	Solyc10g0	Solyc10g0	3.52
sly02010	Environme	Membrane	ABC trans	0.33	0.74	Solyc02g0	Solyc02g0	0.34
sly00300	Metabolisr	Amino aci	Lysine bio	0.33	0.74	Solyc06g0	Solyc06g0	0.30
sly00531	Metabolisr	Glycan bio	Glycosami	0.38	0.75	Solyc03g1	Solyc03g1	0.41
sly00905	Metabolisr	Metabolisr	Brassinost	0.38	0.75	Solyc02g0	Solyc02g0	0.36
sly00450	Metabolisr	Metabolisr	Selenocom	0.38	0.75	Solyc01g1	Solyc01g1	2.42
sly00591	Metabolisr	Lipid meta	Linoleic ac	0.38	0.75	Solyc01g0	Solyc01g0	3.58
sly00280	Metabolisr	Amino aci	Valine, leu	0.38	0.75	Solyc01g0	Solyc01g0	0.41
sly00280	Metabolisr	Amino aci	Valine, leu	0.38	0.75	Solyc03g1	Solyc03g1	0.46
sly00280	Metabolisr	Amino aci	Valine, leu	0.38	0.75	Solyc12g0	Solyc12g0	0.43
sly00750	Metabolisr	Metabolisr	Vitamin B	0.42	0.80	Solyc06g0	Solyc06g0	0.22
sly00330	Metabolisr	Amino aci	Arginine a	0.43	0.81	Solyc02g0	Solyc02g0	0.36
sly00330	Metabolisr	Amino aci	Arginine a	0.43	0.81	Solyc01g0	Solyc01g0	0.41
sly00330	Metabolisr	Amino aci	Arginine a	0.43	0.81	Solyc03g1	Solyc03g1	0.46
sly04130	Genetic In	Folding, sc	SNARE in	0.45	0.81	Solyc09g0	Solyc09g0	2.28
sly04130	Genetic In	Folding, sc	SNARE in	0.45	0.81	Solyc01g0	Solyc01g0	0.19
sly00770	Metabolisr	Metabolisr	Pantothena	0.45	0.81	Solyc01g0	Solyc01g0	0.41
sly00770	Metabolisr	Metabolisr	Pantothena	0.45	0.81	Solyc03g1	Solyc03g1	0.46
sly00565	Metabolisr	Lipid meta	Ether lipid	0.49	0.86	Solyc08g0	Solyc08g0	0.47
sly00260	Metabolisr	Amino aci	Glycine, se	0.52	0.86	Solyc12g0	Solyc12g0	0.30
sly00260	Metabolisr	Amino aci	Glycine, se	0.52	0.86	Solyc06g0	Solyc06g0	0.30
sly00260	Metabolisr	Amino aci	Glycine, se	0.52	0.86	Solyc09g0	TD2	4.53
sly01040	Metabolisr	Lipid meta	Biosynthes	0.52	0.86	Solyc01g0	Solyc01g0	0.27
sly00130	Metabolisr	Metabolisr	Ubiquinon	0.53	0.86	Solyc05g0	Solyc05g0	0.22
sly00130	Metabolisr	Metabolisr	Ubiquinon	0.53	0.86	Solyc03g0	Solyc03g0	0.11
sly00290	Metabolisr	Amino aci	Valine, leu	0.55	0.86	Solyc09g0	TD2	4.53
sly00030	Metabolisr	Carbohydr	Pentose ph	0.57	0.86	Solyc01g1	Solyc01g1	0.31
sly00030	Metabolisr	Carbohydr	Pentose ph	0.57	0.86	Solyc05g0	Solyc05g0	0.44
sly00052	Metabolisr	Carbohydr	Galactose	0.57	0.86	Solyc11g0	Solyc11g0	0.33
sly00052	Metabolisr	Carbohydr	Galactose	0.57	0.86	Solyc01g0	Solyc01g0	0.45
sly00480	Metabolisr	Metabolisr	Glutathion	0.58	0.86	Solyc01g0	Solyc01g0	0.31
sly00480	Metabolisr	Metabolisr	Glutathion	0.58	0.86	Solyc02g0	Solyc02g0	2.89
sly00480	Metabolisr	Metabolisr	Glutathion	0.58	0.86	Solyc09g0	Solyc09g0	0.34
sly00740	Metabolisr	Metabolisr	Riboflavin	0.58	0.86	Solyc03g0	Solyc03g0	0.15
sly00500	Metabolisr	Carbohydr	Starch and	0.59	0.86	Solyc12g0	Solyc12g0	0.40
sly00500	Metabolisr	Carbohydr	Starch and	0.59	0.86	Solyc05g0	Solyc05g0	0.37
sly00500	Metabolisr	Carbohydr	Starch and	0.59	0.86	Solyc05g0	Solyc05g0	0.37
sly00500	Metabolisr	Carbohydr	Starch and	0.59	0.86	Solyc11g0	Solyc11g0	0.33
sly00410	Metabolisr	Metabolisr	beta-Alani	0.61	0.86	Solyc01g0	Solyc01g0	0.41
sly00410	Metabolisr	Metabolisr	beta-Alani	0.61	0.86	Solyc03g1	Solyc03g1	0.46
sly01212	Metabolisr	Global and	Fatty acid	0.63	0.86	Solyc01g0	Solyc01g0	0.27
sly01212	Metabolisr	Global and	Fatty acid	0.63	0.86	Solyc08g0	Solyc08g0	0.21
sly00240	Metabolisr	Nucleotide	Pyrimidine	0.63	0.86	Solyc01g0	Solyc01g0	0.29
sly00240	Metabolisr	Nucleotide	Pyrimidine	0.63	0.86	Solyc11g0	Solyc11g0	2.55
sly00040	Metabolisr	Carbohydr	Pentose an	0.65	0.86	Solyc09g0	Solyc09g0	0.27
sly00040	Metabolisr	Carbohydr	Pentose an	0.65	0.86	Solyc05g0	Solyc05g0	0.26
sly00040	Metabolisr	Carbohydr	Pentose an	0.65	0.86	Solyc01g0	Solyc01g0	4.89

sly00620	Metabolisr	Carbohydr	Pyruvate n	0.65	0.86	Solyc05g0	Solyc05g0	2.02
sly00620	Metabolisr	Carbohydr	Pyruvate n	0.65	0.86	Solyc01g0	Solyc01g0	0.41
sly00620	Metabolisr	Carbohydr	Pyruvate n	0.65	0.86	Solyc03g1	Solyc03g1	0.46
sly00270	Metabolisr	Amino aci	Cysteine a	0.65	0.86	Solyc02g0	Solyc02g0	0.23
sly00270	Metabolisr	Amino aci	Cysteine a	0.65	0.86	Solyc07g0	Solyc07g0	0.16
sly00270	Metabolisr	Amino aci	Cysteine a	0.65	0.86	Solyc03g0	Solyc03g0	0.00
sly00270	Metabolisr	Amino aci	Cysteine a	0.65	0.86	Solyc06g0	Solyc06g0	0.30
sly00073	Metabolisr	Lipid meta	Cutin, sub	0.66	0.86	Solyc12g0	Solyc12g0	0.47
sly03440	Genetic In	Replicatio	Homologo	0.66	0.86	Solyc06g0	Solyc06g0	2.41
sly00564	Metabolisr	Lipid meta	Glyceroph	0.67	0.86	Solyc12g0	Solyc12g0	0.48
sly00564	Metabolisr	Lipid meta	Glyceroph	0.67	0.86	Solyc11g0	Solyc11g0	6.34
sly00564	Metabolisr	Lipid meta	Glyceroph	0.67	0.86	Solyc08g0	Solyc08g0	0.47
sly03410	Genetic In	Replicatio	Base excis	0.70	0.89	Solyc11g0	Solyc11g0	0.43
sly00650	Metabolisr	Carbohydr	Butanoate	0.72	0.89	Solyc12g0	Solyc12g0	0.43
sly00062	Metabolisr	Lipid meta	Fatty acid	0.72	0.89	Solyc09g0	Solyc09g0	8.52
sly04146	Cellular Pr	Transport	Peroxisom	0.73	0.89	Solyc10g0	Solyc10g0	0.03
sly04146	Cellular Pr	Transport	Peroxisom	0.73	0.89	Solyc12g0	Solyc12g0	0.30
sly04144	Cellular Pr	Transport	Endocytos	0.80	0.95	Solyc10g0	Solyc10g0	0.47
sly04144	Cellular Pr	Transport	Endocytos	0.80	0.95	Solyc08g0	Solyc08g0	0.47
sly04144	Cellular Pr	Transport	Endocytos	0.80	0.95	Solyc03g1	Solyc03g1	0.00
sly04144	Cellular Pr	Transport	Endocytos	0.80	0.95	Solyc05g0	Solyc05g0	0.43
sly04144	Cellular Pr	Transport	Endocytos	0.80	0.95	Solyc02g0	Solyc02g0	2.27
sly00051	Metabolisr	Carbohydr	Fructose a	0.80	0.95	Solyc01g1	Solyc01g1	0.31
sly00051	Metabolisr	Carbohydr	Fructose a	0.80	0.95	Solyc11g0	Solyc11g0	0.33
sly01230	Metabolisr	Global and	Biosynthes	0.83	0.96	Solyc07g0	Solyc07g0	0.49
sly01230	Metabolisr	Global and	Biosynthes	0.83	0.96	Solyc01g1	Solyc01g1	0.31
sly01230	Metabolisr	Global and	Biosynthes	0.83	0.96	Solyc05g0	Solyc05g0	0.44
sly01230	Metabolisr	Global and	Biosynthes	0.83	0.96	Solyc06g0	Solyc06g0	0.30
sly01230	Metabolisr	Global and	Biosynthes	0.83	0.96	Solyc09g0	TD2	4.53
sly01230	Metabolisr	Global and	Biosynthes	0.83	0.96	Solyc06g0	Solyc06g0	0.00
sly01210	Metabolisr	Global and	2-Oxocarb	0.84	0.96	Solyc07g0	Solyc07g0	0.49
sly00250	Metabolisr	Amino aci	Alanine, as	0.87	0.97	Solyc12g0	Solyc12g0	0.30
sly00400	Metabolisr	Amino aci	Phenylalar	0.87	0.97	Solyc06g0	Solyc06g0	0.00
sly00562	Metabolisr	Carbohydr	Inositol ph	0.90	0.99	Solyc05g0	Solyc05g0	0.45
sly00860	Metabolisr	Metabolisr	Porphyrin	0.91	0.99	Solyc09g0	Solyc09g0	0.40
sly00190	Metabolisr	Energy me	Oxidative	0.94	0.99	Solyc04g0	Solyc04g0	0.41
sly00190	Metabolisr	Energy me	Oxidative	0.94	0.99	Solyc12g0	Solyc12g0	2112.44
sly01240	Metabolisr	Global and	Biosynthes	0.94	0.99	Solyc07g0	Solyc07g0	0.47
sly01240	Metabolisr	Global and	Biosynthes	0.94	0.99	Solyc01g0	Solyc01g0	0.29
sly01240	Metabolisr	Global and	Biosynthes	0.94	0.99	Solyc07g0	Solyc07g0	2.29
sly01240	Metabolisr	Global and	Biosynthes	0.94	0.99	Solyc01g0	Solyc01g0	0.41
sly01240	Metabolisr	Global and	Biosynthes	0.94	0.99	Solyc06g0	Solyc06g0	0.22
sly01240	Metabolisr	Global and	Biosynthes	0.94	0.99	Solyc03g1	Solyc03g1	0.46
sly03013	Genetic In	Translatio	Nucleocyt	0.95	0.99	Solyc11g0	Solyc11g0	0.13
sly03013	Genetic In	Translatio	Nucleocyt	0.95	0.99	Solyc06g0	Solyc06g0	2.11
sly03040	Genetic In	Transcripti	Spliceoson	0.97	0.99	Solyc10g0	Solyc10g0	0.47
sly03040	Genetic In	Transcripti	Spliceoson	0.97	0.99	Solyc03g1	Solyc03g1	0.00
sly04141	Genetic In	Folding, sc	Protein pro	0.97	0.99	Solyc04g0	Solyc04g0	0.49
sly04141	Genetic In	Folding, sc	Protein pro	0.97	0.99	Solyc10g0	Solyc10g0	0.47
sly04141	Genetic In	Folding, sc	Protein pro	0.97	0.99	Solyc03g1	Solyc03g1	0.00
sly03015	Genetic In	Translatio	mRNA sur	0.98	0.99	Solyc06g0	Solyc06g0	0.33

sly03010	Genetic In	Translatio	Ribosome	1.00	1.00	Solyc09g0	Solyc09g0	3.66
sly03010	Genetic In	Translatio	Ribosome	1.00	1.00	Solyc09g0	Solyc09g0	6.55
sly03010	Genetic In	Translatio	Ribosome	1.00	1.00	Solyc11g0	Solyc11g0	5.73

log2(fc)	pval	qval	regulation
-5.15	0	0	down
-7.13	0	0	down
-2.13	0	0	down
-5.62	0	0	down
-6.95	0	0	down
-3.01	0	0	down
-1.87	0	0	down
-3.13	0	0	down
-1.88	0.00	0.00	down
1.41	0.00	0.00	up
1.21	0.00	0.00	up
-1.09	0.00	0.00	down
-1.72	0.00	0.00	down
-4.12	0.00	0.00	down
-2.62	0.00	0.00	down
-1.69	0.00	0.00	down
-1.57	0.00	0.00	down
-1.33	0.00	0.00	down
-2.15	0.00	0.00	down
-2.08	0.00	0.00	down
1.01	0.00	0.00	up
-3.22	0.00	0.00	down
1.01	0.00	0.00	up
-2.27	0.00	0.00	down
-2.46	0.00	0.00	down
-1.06	0.00	0.00	down
-1.58	0.00	0.00	down
-1.04	0.00	0.00	down
-2.71	0.00	0.00	down
-1.44	0.00	0.00	down
-1.15	0.00	0.00	down
1.11	0.00	0.00	up
-1.78	0.00	0.00	down
-1.84	0.00	0.00	down
-1.55	0.00	0.00	down
-1.46	0.00	0.00	down
-1.27	0.00	0.00	down
-1.42	0.00	0.00	down
1.19	0.00	0.00	up
-1.33	0.00	0.00	down
-1.27	0.00	0.00	down
-1.03	0.00	0.00	down
-1.22	0.00	0.00	down
-1.30	0.00	0.00	down
-2.19	0.00	0.00	down
-1.49	0.00	0.00	down
-1.69	0.00	0.00	down
-2.75	0.00	0.00	down
-1.18	0.00	0.00	down

-1.77	0.00	0.00	down
2.66	0.00	0.00	up
-1.20	0.00	0.00	down
-1.59	0.00	0.00	down
-1.02	0.00	0.00	down
-1.92	0.00	0.00	down
-1.12	0.00	0.00	down
-1.88	0.00	0.00	down
-1.96	0.00	0.00	down
-1.10	0.00	0.00	down
-1.03	0.00	0.00	down
-1.20	0.00	0.00	down
-1.15	0.00	0.00	down
2.55	0.00	0.00	up
-3.24	0.00	0.00	down
-2.51	0.00	0.00	down
1.01	0.00	0.00	up
1.53	0.00	0.00	up
-1.07	0.00	0.00	down
1.35	0.00	0.00	up
-1.49	0.00	0.00	down
11.04	0.00	0.00	up
-1.26	0.00	0.00	down
-2.07	0.00	0.00	down
-1.12	0.00	0.00	down
-1.20	0.00	0.00	down
-11.21	0.00	0.00	down
-1.16	0.00	0.00	down
1.09	0.00	0.00	up
-1.56	0.00	0.00	down
1.28	0.00	0.00	up
2.29	0.00	0.00	up
-1.74	0.00	0.00	down
-10.99	0.00	0.00	down
-1.35	0.00	0.00	down
3.09	0.00	0.00	up
-1.75	0.00	0.00	down
1.28	0.00	0.00	up
-1.55	0.00	0.00	down
-11.15	0.00	0.00	down
-2.93	0.00	0.00	down
1.56	0.00	0.00	up
-1.39	0.00	0.00	down
-1.57	0.00	0.00	down
2.18	0.00	0.00	up
9.64	0.00	0.00	up
1.10	0.00	0.00	up
3.32	0.00	0.00	up
-1.51	0.00	0.01	down
1.19	0.00	0.01	up
1.19	0.00	0.01	up

1.59	0.00	0.01	up
-9.69	0.00	0.01	down
-1.87	0.00	0.01	down
-1.43	0.00	0.01	down
-4.71	0.00	0.01	down
-3.34	0.00	0.02	down
-1.08	0.00	0.02	down
-1.85	0.01	0.02	down
-9.26	0.01	0.02	down
-7.13	0	0	down
-5.62	0	0	down
-6.95	0	0	down
-3.13	0	0	down
1.41	0.00	0.00	up
-2.15	0.00	0.00	down
1.09	0.00	0.00	up
1.19	0.00	0.01	up
-9.69	0.00	0.01	down
-1.87	0.00	0.01	down
-4.71	0.00	0.01	down
-1.13	0.00	0.00	down
-1.07	0.00	0.00	down
-1.53	0.00	0.00	down
-1.22	0.00	0.00	down
-2.31	0.00	0.00	down
-2.07	0.00	0.00	down
-2.23	0.00	0.00	down
-1.71	0.00	0.00	down
-4.04	0.00	0.01	down
-5.15	0	0	down
-7.13	0	0	down
-2.54	0	0	down
-2.13	0	0	down
-5.62	0	0	down
-6.95	0	0	down
-3.01	0	0	down
-3.13	0	0	down
1.41	0.00	0.00	up
-1.72	0.00	0.00	down
-2.62	0.00	0.00	down
-1.33	0.00	0.00	down
-2.15	0.00	0.00	down
1.01	0.00	0.00	up
-3.22	0.00	0.00	down
1.01	0.00	0.00	up
-2.27	0.00	0.00	down
-1.06	0.00	0.00	down
-1.58	0.00	0.00	down
-1.04	0.00	0.00	down
-1.44	0.00	0.00	down
-1.15	0.00	0.00	down

1.11	0.00	0.00 up
-2.69	0.00	0.00 down
-1.84	0.00	0.00 down
-1.55	0.00	0.00 down
-1.46	0.00	0.00 down
-1.33	0.00	0.00 down
-1.27	0.00	0.00 down
-1.30	0.00	0.00 down
-1.49	0.00	0.00 down
-1.69	0.00	0.00 down
-1.18	0.00	0.00 down
-1.77	0.00	0.00 down
2.66	0.00	0.00 up
-1.59	0.00	0.00 down
-1.12	0.00	0.00 down
-1.10	0.00	0.00 down
-1.20	0.00	0.00 down
2.55	0.00	0.00 up
-1.07	0.00	0.00 down
-1.49	0.00	0.00 down
-1.26	0.00	0.00 down
-1.20	0.00	0.00 down
-11.21	0.00	0.00 down
1.09	0.00	0.00 up
-1.74	0.00	0.00 down
-1.35	0.00	0.00 down
3.09	0.00	0.00 up
-1.75	0.00	0.00 down
1.28	0.00	0.00 up
1.56	0.00	0.00 up
-1.39	0.00	0.00 down
2.18	0.00	0.00 up
1.10	0.00	0.00 up
3.32	0.00	0.00 up
1.19	0.00	0.01 up
1.19	0.00	0.01 up
1.59	0.00	0.01 up
-9.69	0.00	0.01 down
-1.87	0.00	0.01 down
-4.71	0.00	0.01 down
-3.34	0.00	0.02 down
-1.85	0.01	0.02 down
-9.26	0.01	0.02 down
1.41	0.00	0.00 up
-2.15	0.00	0.00 down
-3.22	0.00	0.00 down
2.55	0.00	0.00 up
1.09	0.00	0.00 up
1.28	0.00	0.00 up
1.56	0.00	0.00 up
1.10	0.00	0.00 up

3.32	0.00	0.00	up
1.19	0.00	0.01	up
1.19	0.00	0.01	up
1.59	0.00	0.01	up
-9.69	0.00	0.01	down
-1.87	0.00	0.01	down
-4.71	0.00	0.01	down
-3.34	0.00	0.02	down
-2.46	0.00	0.00	down
-2.75	0.00	0.00	down
-1.02	0.00	0.00	down
-11.15	0.00	0.00	down
-1.51	0.00	0.01	down
-2.27	0.00	0.00	down
-1.06	0.00	0.00	down
-1.30	0.00	0.00	down
-1.12	0.00	0.00	down
-1.20	0.00	0.00	down
-1.35	0.00	0.00	down
1.41	0.00	0.00	up
-2.15	0.00	0.00	down
1.09	0.00	0.00	up
1.19	0.00	0.01	up
-9.69	0.00	0.01	down
-1.87	0.00	0.01	down
-4.71	0.00	0.01	down
-1.09	0.00	0.00	down
-1.03	0.00	0.00	down
-1.39	0.00	0.00	down
-1.08	0.00	0.02	down
-3.01	0	0	down
-2.27	0.00	0.00	down
-1.06	0.00	0.00	down
-1.10	0.00	0.00	down
-11.78	0.00	0.00	down
1.21	0.00	0.00	up
-4.12	0.00	0.00	down
-2.08	0.00	0.00	down
-1.55	0.00	0.00	down
1.19	0.00	0.00	up
-1.59	0.00	0.00	down
-1.92	0.00	0.00	down
-1.88	0.00	0.00	down
-1.16	0.00	0.00	down
-1.43	0.00	0.01	down
-1.57	0.00	0.00	down
-1.30	0.00	0.00	down
2.66	0.00	0.00	up
-1.12	0.00	0.00	down
-1.55	0.00	0.00	down
-1.57	0.00	0.00	down

-1.30	0.00	0.00	down
-1.12	0.00	0.00	down
-1.12	0.00	0.00	down
-1.59	0.00	0.00	down
-12.11	0.00	0.00	down
3.09	0.00	0.00	up
1.31	0.00	0.00	up
-1.07	0.00	0.00	down
-1.22	0.00	0.00	down
-1.57	0.00	0.00	down
1.41	0.00	0.00	up
-1.03	0.00	0.01	down
-3.77	0.00	0.01	down
1.68	0.01	0.03	up
-1.30	0.00	0.00	down
-1.12	0.00	0.00	down
1.01	0.00	0.00	up
2.33	0.01	0.04	up
-4.12	0.00	0.00	down
-2.08	0.00	0.00	down
-1.34	0.00	0.00	down
-1.88	0.00	0.00	down
-1.28	0.00	0.00	down
-1.51	0.00	0.00	down
-1.07	0.00	0.00	down
-1.36	0.00	0.01	down
-1.54	0.01	0.02	down
-1.04	0.01	0.03	down
-1.72	0.00	0.00	down
-1.93	0.00	0.00	down
-1.85	0.01	0.02	down
-1.58	0.00	0.00	down
-1.69	0.00	0.00	down
-1.18	0.00	0.00	down
-1.12	0.00	0.00	down
-2.93	0.00	0.00	down
1.19	0.00	0.00	up
-1.30	0.00	0.00	down
-1.12	0.00	0.00	down
-2.51	0.00	0.00	down
1.53	0.00	0.00	up
-1.30	0.00	0.00	down
-1.12	0.00	0.00	down
1.01	0.00	0.00	up
-1.06	0.00	0.00	down
-1.30	0.00	0.00	down
-1.69	0.00	0.00	down
-1.59	0.00	0.00	down
-1.12	0.00	0.00	down
-1.20	0.00	0.00	down
-1.35	0.00	0.00	down

-7.13	0	0	down
-6.95	0	0	down
-1.65	0.00	0.00	down
1.30	0	0	up
-1.84	0.00	0.00	down
-1.49	0.00	0.00	down
1.01	0.00	0.00	up
1.01	0.00	0.00	up
-1.04	0.00	0.00	down
-1.27	0.00	0.00	down
-2.69	0.00	0.00	down
-1.75	0.00	0.00	down
-1.06	0.00	0.00	down
-1.20	0.00	0.00	down
-1.35	0.00	0.00	down
-5.15	0	0	down
1.01	0.00	0.00	up
-1.58	0.00	0.00	down
-1.04	0.00	0.00	down
-1.27	0.00	0.00	down
-1.69	0.00	0.00	down
-1.18	0.00	0.00	down
-1.59	0.00	0.00	down
-1.12	0.00	0.00	down
-1.74	0.00	0.00	down
-2.93	0.00	0.00	down
2.18	0.00	0.00	up
-3.24	0.00	0.00	down
-10.99	0.00	0.00	down
9.64	0.00	0.00	up
-2.54	0	0	down
-1.77	0.00	0.00	down
-1.30	0.00	0.00	down
-1.12	0.00	0.00	down
2.08	0.00	0.00	up
-1.08	0.00	0.00	down
-1.28	0.00	0.00	down
1.00	0.00	0.00	up
-1.51	0.00	0.00	down
-2.51	0.00	0.00	down
-1.01	0.00	0.00	down
-1.40	0.00	0.00	down
-1.36	0.00	0.01	down
-1.54	0.01	0.02	down
-1.04	0.01	0.03	down
-5.15	0	0	down
-1.58	0.00	0.00	down
-1.04	0.00	0.00	down
-1.74	0.00	0.00	down
1.11	0.00	0.00	up
-1.20	0.00	0.00	down

-1.39	0.00	0.00	down
-1.20	0.00	0.00	down
-1.03	0.00	0.00	down
-1.26	0.00	0.00	down
1.82	0.02	0.05	up
-1.57	0.00	0.00	down
-1.75	0.00	0.00	down
-1.27	0.00	0.00	down
-1.49	0.00	0.00	down
1.28	0.00	0.00	up
1.84	0.01	0.04	up
-1.30	0.00	0.00	down
-1.12	0.00	0.00	down
-1.20	0.00	0.00	down
-2.19	0.00	0.00	down
-1.46	0.00	0.00	down
-1.30	0.00	0.00	down
-1.12	0.00	0.00	down
1.19	0.00	0.00	up
-2.40	0.01	0.04	down
-1.30	0.00	0.00	down
-1.12	0.00	0.00	down
-1.07	0.00	0.00	down
-1.74	0.00	0.00	down
-1.75	0.00	0.00	down
2.18	0.00	0.00	up
-1.87	0	0	down
-2.15	0.00	0.00	down
-3.22	0.00	0.00	down
2.18	0.00	0.00	up
-1.69	0.00	0.00	down
-1.18	0.00	0.00	down
-1.59	0.00	0.00	down
-1.15	0.00	0.00	down
-1.69	0.00	0.00	down
1.53	0.00	0.00	up
-1.56	0.00	0.00	down
-2.71	0.00	0.00	down
-1.33	0.00	0.00	down
-1.44	0.00	0.00	down
-1.42	0.00	0.00	down
-1.59	0.00	0.00	down
-1.30	0.00	0.00	down
-1.12	0.00	0.00	down
-1.87	0	0	down
-2.27	0.00	0.00	down
-1.78	0.00	0.00	down
1.35	0.00	0.00	up
-1.88	0.00	0.00	down
-1.96	0.00	0.00	down
2.29	0.00	0.00	up

1.01	0.00	0.00	up
-1.30	0.00	0.00	down
-1.12	0.00	0.00	down
-2.13	0	0	down
-2.62	0.00	0.00	down
-11.21	0.00	0.00	down
-1.75	0.00	0.00	down
-1.08	0.00	0.00	down
1.27	0.00	0.00	up
-1.06	0.00	0.00	down
2.66	0.00	0.00	up
-1.07	0.00	0.00	down
-1.23	0.00	0.00	down
-1.20	0.00	0.00	down
3.09	0.00	0.00	up
-5.15	0	0	down
-1.74	0.00	0.00	down
-1.09	0.00	0.00	down
-1.07	0.00	0.00	down
-11.76	0.00	0.00	down
-1.23	0.00	0.00	down
1.18	0.00	0.00	up
-1.69	0.00	0.00	down
-1.59	0.00	0.00	down
-1.04	0.00	0.00	down
-1.69	0.00	0.00	down
-1.18	0.00	0.00	down
-1.75	0.00	0.00	down
2.18	0.00	0.00	up
-9.26	0.01	0.02	down
-1.04	0.00	0.00	down
-1.74	0.00	0.00	down
-9.26	0.01	0.02	down
-1.15	0.00	0.00	down
-1.33	0.00	0.00	down
-1.27	0.00	0.00	down
11.04	0.00	0.00	up
-1.09	0.00	0.00	down
-1.78	0.00	0.00	down
1.19	0.00	0.00	up
-1.30	0.00	0.00	down
-2.19	0.00	0.00	down
-1.12	0.00	0.00	down
-2.91	0.00	0.00	down
1.07	0.00	0.00	up
-1.09	0.00	0.00	down
-11.76	0.00	0.00	down
-1.03	0.00	0.00	down
-1.09	0.00	0.00	down
-11.76	0.00	0.00	down
-1.61	0.00	0.00	down

1.87	0.00	0.00 up
2.71	0.00	0.00 up
2.52	0.00	0.00 up
