# Supplementary material for: Transcriptome reveals differential expression of flavor and color in closely related strains of tomato (Solanum lycopersicum)
Source: PeerJ. 2025 Oct 7;13:e20113. doi: 10.7717/peerj.20113 (PMC12513376; doi:10.7717/peerj.20113)
Supplement: Supplemental Information 10 [file peerj-13-20113-s010.pdf]

Table S8: KEGG pathways of differentially expressed genes in MF19-vs-MF20.

Pathway_	KEGG_L	KEGG_L	Pathway_	P.value	Q.value	gene_id	gene_nam	fc
sly00195	Metabolisr	Energy me	Photosyntl	0.00	0.00	Solyc02g0	Solyc02g0	3.57
sly00195	Metabolisr	Energy me	Photosyntl	0.00	0.00	Solyc07g0	Solyc07g0	4.02
sly00195	Metabolisr	Energy me	Photosyntl	0.00	0.00	Solyc12g0	Solyc12g0	2.97
sly00195	Metabolisr	Energy me	Photosyntl	0.00	0.00	Solyc04g0	PETE	2.69
sly00195	Metabolisr	Energy me	Photosyntl	0.00	0.00	Solyc09g0	Solyc09g0	7.18
sly00195	Metabolisr	Energy me	Photosyntl	0.00	0.00	Solyc02g0	Solyc02g0	2.31
sly00195	Metabolisr	Energy me	Photosyntl	0.00	0.00	Solyc12g0	Solyc12g0	2.10
sly00195	Metabolisr	Energy me	Photosyntl	0.00	0.00	Solyc02g0	PSBO	5.02
sly00195	Metabolisr	Energy me	Photosyntl	0.00	0.00	Solyc07g0	PSBR	3.05
sly00195	Metabolisr	Energy me	Photosyntl	0.00	0.00	Solyc02g0	Solyc02g0	3.41
sly00195	Metabolisr	Energy me	Photosyntl	0.00	0.00	Solyc06g0	PSBS	2.99
sly00195	Metabolisr	Energy me	Photosyntl	0.00	0.00	Solyc07g0	Solyc07g0	2.47
sly00195	Metabolisr	Energy me	Photosyntl	0.00	0.00	Solyc01g1	psbZ	0.17
sly00195	Metabolisr	Energy me	Photosyntl	0.00	0.00	Solyc08g0	Solyc08g0	1537.41
sly00195	Metabolisr	Energy me	Photosyntl	0.00	0.00	Solyc06g0	Solyc06g0	4.49
sly00195	Metabolisr	Energy me	Photosyntl	0.00	0.00	Solyc03g1	Solyc03g1	2.21
sly01100	Metabolisr	Global and	Metabolic	0.00	0.00	Solyc07g0	ACO1	2.82
sly01100	Metabolisr	Global and	Metabolic	0.00	0.00	Solyc10g0	PG2	9.48
sly01100	Metabolisr	Global and	Metabolic	0.00	0.00	Solyc01g1	Solyc01g1	4.83
sly01100	Metabolisr	Global and	Metabolic	0.00	0.00	Solyc01g0	Solyc01g0	0.09
sly01100	Metabolisr	Global and	Metabolic	0.00	0.00	Solyc04g0	Solyc04g0	6.11
sly01100	Metabolisr	Global and	Metabolic	0.00	0.00	Solyc03g0	Solyc03g0	4.05
sly01100	Metabolisr	Global and	Metabolic	0.00	0.00	Solyc10g0	Solyc10g0	4.26
sly01100	Metabolisr	Global and	Metabolic	0.00	0.00	Solyc04g0	Solyc04g0	6.77
sly01100	Metabolisr	Global and	Metabolic	0.00	0.00	Solyc09g0	Solyc09g0	6.99
sly01100	Metabolisr	Global and	Metabolic	0.00	0.00	Solyc07g0	Solyc07g0	0.35
sly01100	Metabolisr	Global and	Metabolic	0.00	0.00	Solyc02g0	Solyc02g0	0.08
sly01100	Metabolisr	Global and	Metabolic	0.00	0.00	Solyc01g0	Solyc01g0	2.62
sly01100	Metabolisr	Global and	Metabolic	0.00	0.00	Solyc05g0	Solyc05g0	0.06
sly01100	Metabolisr	Global and	Metabolic	0.00	0.00	Solyc02g0	Solyc02g0	3.57
sly01100	Metabolisr	Global and	Metabolic	0.00	0.00	Solyc01g0	Solyc01g0	2.46
sly01100	Metabolisr	Global and	Metabolic	0.00	0.00	Solyc11g0	Solyc11g0	3.25
sly01100	Metabolisr	Global and	Metabolic	0.00	0.00	Solyc02g0	Solyc02g0	3.08
sly01100	Metabolisr	Global and	Metabolic	0.00	0.00	Solyc05g0	Solyc05g0	4.45
sly01100	Metabolisr	Global and	Metabolic	0.00	0.00	Solyc11g0	Solyc11g0	0.28
sly01100	Metabolisr	Global and	Metabolic	0.00	0.00	Solyc09g0	Solyc09g0	0.06
sly01100	Metabolisr	Global and	Metabolic	0.00	0.00	Solyc05g0	Solyc05g0	2.80
sly01100	Metabolisr	Global and	Metabolic	0.00	0.00	Solyc06g0	Solyc06g0	2.22
sly01100	Metabolisr	Global and	Metabolic	0.00	0.00	Solyc12g0	Solyc12g0	0.25
sly01100	Metabolisr	Global and	Metabolic	0.00	0.00	Solyc04g0	Solyc04g0	2.62
sly01100	Metabolisr	Global and	Metabolic	0.00	0.00	Solyc04g0	Solyc04g0	0.35
sly01100	Metabolisr	Global and	Metabolic	0.00	0.00	Solyc01g0	Solyc01g0	2.01
sly01100	Metabolisr	Global and	Metabolic	0.00	0.00	Solyc11g0	AOS2	3.20
sly01100	Metabolisr	Global and	Metabolic	0.00	0.00	Solyc01g1	Solyc01g1	4.98
sly01100	Metabolisr	Global and	Metabolic	0.00	0.00	Solyc03g1	Solyc03g1	2.44
sly01100	Metabolisr	Global and	Metabolic	0.00	0.00	Solyc04g0	Solyc04g0	3.78
sly01100	Metabolisr	Global and	Metabolic	0.00	0.00	Solyc09g0	Solyc09g0	2.88
sly01100	Metabolisr	Global and	Metabolic	0.00	0.00	Solyc04g0	Solyc04g0	3.50
sly01100	Metabolisr	Global and	Metabolic	0.00	0.00	Solyc03g0	Solyc03g0	5.61

sly01100	Metabolisr	Global and	Metabolic	0.00	0.00	Solyc09g0	Solyc09g0	0.37
sly01100	Metabolisr	Global and	Metabolic	0.00	0.00	Solyc04g0	Solyc04g0	8.55
sly01100	Metabolisr	Global and	Metabolic	0.00	0.00	Solyc10g0	CHI9	0.28
sly01100	Metabolisr	Global and	Metabolic	0.00	0.00	Solyc04g0	Solyc04g0	0.40
sly01100	Metabolisr	Global and	Metabolic	0.00	0.00	Solyc01g0	Solyc01g0	0.22
sly01100	Metabolisr	Global and	Metabolic	0.00	0.00	Solyc08g0	Solyc08g0	3.48
sly01100	Metabolisr	Global and	Metabolic	0.00	0.00	Solyc03g0	Solyc03g0	2.33
sly01100	Metabolisr	Global and	Metabolic	0.00	0.00	Solyc07g0	Solyc07g0	3.93
sly01100	Metabolisr	Global and	Metabolic	0.00	0.00	Solyc12g0	Solyc12g0	2.97
sly01100	Metabolisr	Global and	Metabolic	0.00	0.00	Solyc12g0	Solyc12g0	2.20
sly01100	Metabolisr	Global and	Metabolic	0.00	0.00	Solyc06g0	Solyc06g0	2.40
sly01100	Metabolisr	Global and	Metabolic	0.00	0.00	Solyc01g0	Solyc01g0	0.35
sly01100	Metabolisr	Global and	Metabolic	0.00	0.00	Solyc06g0	Solyc06g0	2.47
sly01100	Metabolisr	Global and	Metabolic	0.00	0.00	Solyc06g0	Solyc06g0	3.62
sly01100	Metabolisr	Global and	Metabolic	0.00	0.00	Solyc07g0	Solyc07g0	0.49
sly01100	Metabolisr	Global and	Metabolic	0.00	0.00	Solyc09g0	Solyc09g0	3.61
sly01100	Metabolisr	Global and	Metabolic	0.00	0.00	Solyc10g0	Solyc10g0	2.91
sly01100	Metabolisr	Global and	Metabolic	0.00	0.00	Solyc09g0	Solyc09g0	5.85
sly01100	Metabolisr	Global and	Metabolic	0.00	0.00	Solyc04g0	Solyc04g0	2.89
sly01100	Metabolisr	Global and	Metabolic	0.00	0.00	Solyc11g0	Solyc11g0	0.50
sly01100	Metabolisr	Global and	Metabolic	0.00	0.00	Solyc01g0	Solyc01g0	0.32
sly01100	Metabolisr	Global and	Metabolic	0.00	0.00	Solyc12g0	Solyc12g0	7.81
sly01100	Metabolisr	Global and	Metabolic	0.00	0.00	Solyc07g0	Solyc07g0	0.48
sly01100	Metabolisr	Global and	Metabolic	0.00	0.00	Solyc09g0	Solyc09g0	0.47
sly01100	Metabolisr	Global and	Metabolic	0.00	0.00	Solyc02g0	Solyc02g0	2.31
sly01100	Metabolisr	Global and	Metabolic	0.00	0.00	Solyc08g0	Solyc08g0	2.18
sly01100	Metabolisr	Global and	Metabolic	0.00	0.00	Solyc09g0	Solyc09g0	0.36
sly01100	Metabolisr	Global and	Metabolic	0.00	0.00	Solyc01g0	Solyc01g0	3.54
sly01100	Metabolisr	Global and	Metabolic	0.00	0.00	Solyc12g0	Solyc12g0	2.10
sly01100	Metabolisr	Global and	Metabolic	0.00	0.00	Solyc06g0	Solyc06g0	2.01
sly01100	Metabolisr	Global and	Metabolic	0.00	0.00	Solyc05g0	Solyc05g0	0.27
sly01100	Metabolisr	Global and	Metabolic	0.00	0.00	Solyc05g0	Solyc05g0	3.22
sly01100	Metabolisr	Global and	Metabolic	0.00	0.00	Solyc03g0	Solyc03g0	0.18
sly01100	Metabolisr	Global and	Metabolic	0.00	0.00	Solyc02g0	Solyc02g0	3.06
sly01100	Metabolisr	Global and	Metabolic	0.00	0.00	Solyc02g0	Solyc02g0	2.54
sly01100	Metabolisr	Global and	Metabolic	0.00	0.00	Solyc10g0	Solyc10g0	2.23
sly01100	Metabolisr	Global and	Metabolic	0.00	0.00	Solyc09g0	Solyc09g0	2.71
sly01100	Metabolisr	Global and	Metabolic	0.00	0.00	Solyc03g0	Solyc03g0	0.22
sly01100	Metabolisr	Global and	Metabolic	0.00	0.00	Solyc11g0	Solyc11g0	2.07
sly01100	Metabolisr	Global and	Metabolic	0.00	0.00	Solyc02g0	Solyc02g0	2.56
sly01100	Metabolisr	Global and	Metabolic	0.00	0.00	Solyc05g0	Solyc05g0	2.50
sly01100	Metabolisr	Global and	Metabolic	0.00	0.00	Solyc06g0	Solyc06g0	3.53
sly01100	Metabolisr	Global and	Metabolic	0.00	0.00	Solyc06g0	Solyc06g0	2.33
sly01100	Metabolisr	Global and	Metabolic	0.00	0.00	Solyc01g0	Solyc01g0	0.06
sly01100	Metabolisr	Global and	Metabolic	0.00	0.00	Solyc03g0	Solyc03g0	3.84
sly01100	Metabolisr	Global and	Metabolic	0.00	0.00	Solyc01g0	Solyc01g0	3528.16
sly01100	Metabolisr	Global and	Metabolic	0.00	0.00	Solyc06g0	Solyc06g0	0.14
sly01100	Metabolisr	Global and	Metabolic	0.00	0.00	Solyc01g0	Solyc01g0	2.52
sly01100	Metabolisr	Global and	Metabolic	0.00	0.00	Solyc02g0	Solyc02g0	4235.90
sly01100	Metabolisr	Global and	Metabolic	0.00	0.00	Solyc03g0	Solyc03g0	5511.05
sly01100	Metabolisr	Global and	Metabolic	0.00	0.00	Solyc06g0	Solyc06g0	0.15

sly01100	Metabolisr	Global and	Metabolic	0.00	0.00	Solyc01g1	Solyc01g1	2993.63
sly01100	Metabolisr	Global and	Metabolic	0.00	0.00	Solyc11g0	Solyc11g0	0.38
sly01100	Metabolisr	Global and	Metabolic	0.00	0.00	Solyc02g0	Solyc02g0	2.01
sly01100	Metabolisr	Global and	Metabolic	0.00	0.00	Solyc09g0	Solyc09g0	3.57
sly01100	Metabolisr	Global and	Metabolic	0.00	0.00	Solyc06g0	Solyc06g0	2.25
sly01100	Metabolisr	Global and	Metabolic	0.00	0.00	Solyc01g1	Solyc01g1	4.37
sly01100	Metabolisr	Global and	Metabolic	0.00	0.00	Solyc02g0	Solyc02g0	0.48
sly01100	Metabolisr	Global and	Metabolic	0.00	0.00	Solyc01g0	Solyc01g0	2.02
sly01100	Metabolisr	Global and	Metabolic	0.00	0.00	Solyc04g0	Solyc04g0	1711.14
sly01100	Metabolisr	Global and	Metabolic	0.00	0.00	Solyc03g0	RBCS-2A	2.71
sly01100	Metabolisr	Global and	Metabolic	0.00	0.00	Solyc10g0	Solyc10g0	2.07
sly01100	Metabolisr	Global and	Metabolic	0.00	0.00	Solyc08g0	Solyc08g0	2.11
sly01100	Metabolisr	Global and	Metabolic	0.00	0.00	Solyc08g0	Solyc08g0	2.43
sly01100	Metabolisr	Global and	Metabolic	0.00	0.00	Solyc06g0	Solyc06g0	2.08
sly01100	Metabolisr	Global and	Metabolic	0.00	0.00	Solyc02g0	Solyc02g0	0.07
sly01100	Metabolisr	Global and	Metabolic	0.00	0.00	Solyc09g0	Solyc09g0	25.41
sly01100	Metabolisr	Global and	Metabolic	0.00	0.00	Solyc05g0	Solyc05g0	2.69
sly01100	Metabolisr	Global and	Metabolic	0.00	0.00	Solyc07g0	Solyc07g0	2.71
sly01100	Metabolisr	Global and	Metabolic	0.00	0.00	Solyc03g0	Solyc03g0	2.60
sly01100	Metabolisr	Global and	Metabolic	0.00	0.00	Solyc03g1	Solyc03g1	6.29
sly01100	Metabolisr	Global and	Metabolic	0.00	0.00	Solyc09g0	Solyc09g0	2.82
sly01100	Metabolisr	Global and	Metabolic	0.00	0.00	Solyc07g0	Solyc07g0	0.14
sly01100	Metabolisr	Global and	Metabolic	0.00	0.00	Solyc04g0	Solyc04g0	15.69
sly01100	Metabolisr	Global and	Metabolic	0.00	0.00	Solyc04g0	Solyc04g0	39.10
sly01100	Metabolisr	Global and	Metabolic	0.00	0.00	Solyc06g0	Solyc06g0	0.25
sly01100	Metabolisr	Global and	Metabolic	0.00	0.00	Solyc03g0	Solyc03g0	0.39
sly01100	Metabolisr	Global and	Metabolic	0.00	0.00	Solyc12g0	Solyc12g0	0.25
sly01100	Metabolisr	Global and	Metabolic	0.00	0.00	Solyc06g0	Solyc06g0	15.51
sly01100	Metabolisr	Global and	Metabolic	0.00	0.00	Solyc09g0	Solyc09g0	3.37
sly01100	Metabolisr	Global and	Metabolic	0.00	0.00	Solyc11g0	Solyc11g0	5.06
sly01100	Metabolisr	Global and	Metabolic	0.00	0.00	Solyc01g0	Solyc01g0	13.05
sly01100	Metabolisr	Global and	Metabolic	0.00	0.00	Solyc11g0	Solyc11g0	45.49
sly01100	Metabolisr	Global and	Metabolic	0.00	0.00	Solyc06g0	Solyc06g0	0.38
sly01100	Metabolisr	Global and	Metabolic	0.00	0.00	Solyc03g0	Solyc03g0	0.00
sly01100	Metabolisr	Global and	Metabolic	0.00	0.00	Solyc08g0	Solyc08g0	0.13
sly01100	Metabolisr	Global and	Metabolic	0.00	0.00	Solyc02g0	Solyc02g0	574.97
sly01100	Metabolisr	Global and	Metabolic	0.00	0.00	Solyc08g0	Solyc08g0	5.12
sly01100	Metabolisr	Global and	Metabolic	0.00	0.00	Solyc01g0	Solyc01g0	550.66
sly01100	Metabolisr	Global and	Metabolic	0.00	0.00	Solyc02g0	Solyc02g0	2.27
sly01100	Metabolisr	Global and	Metabolic	0.00	0.00	Solyc04g0	Solyc04g0	32.87
sly01100	Metabolisr	Global and	Metabolic	0.00	0.00	Solyc12g0	Solyc12g0	4.99
sly01100	Metabolisr	Global and	Metabolic	0.00	0.00	Solyc12g0	Solyc12g0	6.07
sly01100	Metabolisr	Global and	Metabolic	0.00	0.00	Solyc02g0	Solyc02g0	8.45
sly01100	Metabolisr	Global and	Metabolic	0.00	0.00	Solyc06g0	Solyc06g0	13.74
sly01100	Metabolisr	Global and	Metabolic	0.00	0.00	Solyc10g0	Solyc10g0	0.00
sly01100	Metabolisr	Global and	Metabolic	0.00	0.00	Solyc03g0	Solyc03g0	0.44
sly01100	Metabolisr	Global and	Metabolic	0.00	0.00	Solyc01g1	Solyc01g1	4.58
sly01100	Metabolisr	Global and	Metabolic	0.00	0.00	Solyc06g0	Solyc06g0	4.49
sly01100	Metabolisr	Global and	Metabolic	0.00	0.00	Solyc02g0	Solyc02g0	3.52
sly01100	Metabolisr	Global and	Metabolic	0.00	0.00	Solyc11g0	Solyc11g0	0.43
sly01100	Metabolisr	Global and	Metabolic	0.00	0.00	Solyc01g0	Solyc01g0	0.03

sly01100	Metabolisr	Global and	Metabolic	0.00	0.00	Solyc07g0	Solyc07g0	2.87
sly01110	Metabolisr	Global and	Biosynthes	0.00	0.00	Solyc07g0	ACO1	2.82
sly01110	Metabolisr	Global and	Biosynthes	0.00	0.00	Solyc04g0	Solyc04g0	6.11
sly01110	Metabolisr	Global and	Biosynthes	0.00	0.00	Solyc03g0	Solyc03g0	4.05
sly01110	Metabolisr	Global and	Biosynthes	0.00	0.00	Solyc10g0	Solyc10g0	4.26
sly01110	Metabolisr	Global and	Biosynthes	0.00	0.00	Solyc04g0	Solyc04g0	6.77
sly01110	Metabolisr	Global and	Biosynthes	0.00	0.00	Solyc09g0	Solyc09g0	6.99
sly01110	Metabolisr	Global and	Biosynthes	0.00	0.00	Solyc07g0	Solyc07g0	0.35
sly01110	Metabolisr	Global and	Biosynthes	0.00	0.00	Solyc02g0	Solyc02g0	0.08
sly01110	Metabolisr	Global and	Biosynthes	0.00	0.00	Solyc05g0	Solyc05g0	0.06
sly01110	Metabolisr	Global and	Biosynthes	0.00	0.00	Solyc01g0	Solyc01g0	2.46
sly01110	Metabolisr	Global and	Biosynthes	0.00	0.00	Solyc11g0	Solyc11g0	0.28
sly01110	Metabolisr	Global and	Biosynthes	0.00	0.00	Solyc09g0	Solyc09g0	0.06
sly01110	Metabolisr	Global and	Biosynthes	0.00	0.00	Solyc05g0	Solyc05g0	2.80
sly01110	Metabolisr	Global and	Biosynthes	0.00	0.00	Solyc06g0	Solyc06g0	2.22
sly01110	Metabolisr	Global and	Biosynthes	0.00	0.00	Solyc04g0	Solyc04g0	2.62
sly01110	Metabolisr	Global and	Biosynthes	0.00	0.00	Solyc04g0	Solyc04g0	0.35
sly01110	Metabolisr	Global and	Biosynthes	0.00	0.00	Solyc11g0	AOS2	3.20
sly01110	Metabolisr	Global and	Biosynthes	0.00	0.00	Solyc01g1	Solyc01g1	4.98
sly01110	Metabolisr	Global and	Biosynthes	0.00	0.00	Solyc03g1	Solyc03g1	2.44
sly01110	Metabolisr	Global and	Biosynthes	0.00	0.00	Solyc04g0	Solyc04g0	3.78
sly01110	Metabolisr	Global and	Biosynthes	0.00	0.00	Solyc09g0	Solyc09g0	2.88
sly01110	Metabolisr	Global and	Biosynthes	0.00	0.00	Solyc04g0	Solyc04g0	3.50
sly01110	Metabolisr	Global and	Biosynthes	0.00	0.00	Solyc03g0	Solyc03g0	5.61
sly01110	Metabolisr	Global and	Biosynthes	0.00	0.00	Solyc09g0	Solyc09g0	0.37
sly01110	Metabolisr	Global and	Biosynthes	0.00	0.00	Solyc01g0	Solyc01g0	0.22
sly01110	Metabolisr	Global and	Biosynthes	0.00	0.00	Solyc08g0	Solyc08g0	3.48
sly01110	Metabolisr	Global and	Biosynthes	0.00	0.00	Solyc07g0	Solyc07g0	3.93
sly01110	Metabolisr	Global and	Biosynthes	0.00	0.00	Solyc12g0	Solyc12g0	2.20
sly01110	Metabolisr	Global and	Biosynthes	0.00	0.00	Solyc07g0	Solyc07g0	0.49
sly01110	Metabolisr	Global and	Biosynthes	0.00	0.00	Solyc04g0	Solyc04g0	2.89
sly01110	Metabolisr	Global and	Biosynthes	0.00	0.00	Solyc11g0	Solyc11g0	0.50
sly01110	Metabolisr	Global and	Biosynthes	0.00	0.00	Solyc12g0	Solyc12g0	7.81
sly01110	Metabolisr	Global and	Biosynthes	0.00	0.00	Solyc07g0	Solyc07g0	0.48
sly01110	Metabolisr	Global and	Biosynthes	0.00	0.00	Solyc09g0	Solyc09g0	0.47
sly01110	Metabolisr	Global and	Biosynthes	0.00	0.00	Solyc07g0	Solyc07g0	2.20
sly01110	Metabolisr	Global and	Biosynthes	0.00	0.00	Solyc09g0	Solyc09g0	0.36
sly01110	Metabolisr	Global and	Biosynthes	0.00	0.00	Solyc01g0	Solyc01g0	3.54
sly01110	Metabolisr	Global and	Biosynthes	0.00	0.00	Solyc06g0	Solyc06g0	2.01
sly01110	Metabolisr	Global and	Biosynthes	0.00	0.00	Solyc05g0	Solyc05g0	3.22
sly01110	Metabolisr	Global and	Biosynthes	0.00	0.00	Solyc03g0	Solyc03g0	0.18
sly01110	Metabolisr	Global and	Biosynthes	0.00	0.00	Solyc02g0	Solyc02g0	3.06
sly01110	Metabolisr	Global and	Biosynthes	0.00	0.00	Solyc10g0	Solyc10g0	2.23
sly01110	Metabolisr	Global and	Biosynthes	0.00	0.00	Solyc09g0	Solyc09g0	2.71
sly01110	Metabolisr	Global and	Biosynthes	0.00	0.00	Solyc02g0	Solyc02g0	2.56
sly01110	Metabolisr	Global and	Biosynthes	0.00	0.00	Solyc05g0	Solyc05g0	2.50
sly01110	Metabolisr	Global and	Biosynthes	0.00	0.00	Solyc06g0	Solyc06g0	2.33
sly01110	Metabolisr	Global and	Biosynthes	0.00	0.00	Solyc01g0	Solyc01g0	3528.16
sly01110	Metabolisr	Global and	Biosynthes	0.00	0.00	Solyc01g0	Solyc01g0	2.52
sly01110	Metabolisr	Global and	Biosynthes	0.00	0.00	Solyc03g0	Solyc03g0	5511.05
sly01110	Metabolisr	Global and	Biosynthes	0.00	0.00	Solyc11g0	Solyc11g0	3.46

sly01110	Metabolisr	Global and	Biosynthes	0.00	0.00	Solyc01g1	Solyc01g1	2993.63
sly01110	Metabolisr	Global and	Biosynthes	0.00	0.00	Solyc11g0	Solyc11g0	0.38
sly01110	Metabolisr	Global and	Biosynthes	0.00	0.00	Solyc09g0	Solyc09g0	3.57
sly01110	Metabolisr	Global and	Biosynthes	0.00	0.00	Solyc06g0	Solyc06g0	2.25
sly01110	Metabolisr	Global and	Biosynthes	0.00	0.00	Solyc01g1	Solyc01g1	4.37
sly01110	Metabolisr	Global and	Biosynthes	0.00	0.00	Solyc02g0	Solyc02g0	0.48
sly01110	Metabolisr	Global and	Biosynthes	0.00	0.00	Solyc04g0	Solyc04g0	1711.14
sly01110	Metabolisr	Global and	Biosynthes	0.00	0.00	Solyc03g0	RBCS-2A	2.71
sly01110	Metabolisr	Global and	Biosynthes	0.00	0.00	Solyc08g0	Solyc08g0	2.11
sly01110	Metabolisr	Global and	Biosynthes	0.00	0.00	Solyc07g0	Solyc07g0	29.42
sly01110	Metabolisr	Global and	Biosynthes	0.00	0.00	Solyc06g0	Solyc06g0	2.08
sly01110	Metabolisr	Global and	Biosynthes	0.00	0.00	Solyc02g0	Solyc02g0	0.07
sly01110	Metabolisr	Global and	Biosynthes	0.00	0.00	Solyc09g0	Solyc09g0	25.41
sly01110	Metabolisr	Global and	Biosynthes	0.00	0.00	Solyc12g1	Solyc12g1	0.00
sly01110	Metabolisr	Global and	Biosynthes	0.00	0.00	Solyc05g0	Solyc05g0	2.69
sly01110	Metabolisr	Global and	Biosynthes	0.00	0.00	Solyc07g0	Solyc07g0	2.71
sly01110	Metabolisr	Global and	Biosynthes	0.00	0.00	Solyc04g0	Solyc04g0	1523.79
sly01110	Metabolisr	Global and	Biosynthes	0.00	0.00	Solyc03g1	Solyc03g1	6.29
sly01110	Metabolisr	Global and	Biosynthes	0.00	0.00	Solyc09g0	Solyc09g0	2.82
sly01110	Metabolisr	Global and	Biosynthes	0.00	0.00	Solyc04g0	Solyc04g0	15.69
sly01110	Metabolisr	Global and	Biosynthes	0.00	0.00	Solyc04g0	Solyc04g0	39.10
sly01110	Metabolisr	Global and	Biosynthes	0.00	0.00	Solyc06g0	Solyc06g0	0.25
sly01110	Metabolisr	Global and	Biosynthes	0.00	0.00	Solyc03g0	Solyc03g0	0.39
sly01110	Metabolisr	Global and	Biosynthes	0.00	0.00	Solyc09g0	Solyc09g0	3.37
sly01110	Metabolisr	Global and	Biosynthes	0.00	0.00	Solyc11g0	Solyc11g0	5.06
sly01110	Metabolisr	Global and	Biosynthes	0.00	0.00	Solyc06g0	Solyc06g0	0.38
sly01110	Metabolisr	Global and	Biosynthes	0.00	0.00	Solyc03g0	Solyc03g0	0.00
sly01110	Metabolisr	Global and	Biosynthes	0.00	0.00	Solyc02g0	Solyc02g0	574.97
sly01110	Metabolisr	Global and	Biosynthes	0.00	0.00	Solyc08g0	Solyc08g0	5.12
sly01110	Metabolisr	Global and	Biosynthes	0.00	0.00	Solyc04g0	Solyc04g0	32.87
sly01110	Metabolisr	Global and	Biosynthes	0.00	0.00	Solyc12g0	Solyc12g0	6.07
sly01110	Metabolisr	Global and	Biosynthes	0.00	0.00	Solyc02g0	Solyc02g0	8.45
sly01110	Metabolisr	Global and	Biosynthes	0.00	0.00	Solyc10g0	Solyc10g0	0.00
sly01110	Metabolisr	Global and	Biosynthes	0.00	0.00	Solyc02g0	Solyc02g0	3.52
sly01110	Metabolisr	Global and	Biosynthes	0.00	0.00	Solyc07g0	Solyc07g0	2.87
sly00073	Metabolisr	Lipid meta	Cutin, sube	0.00	0.00	Solyc07g0	Solyc07g0	0.32
sly00073	Metabolisr	Lipid meta	Cutin, sube	0.00	0.00	Solyc06g0	Solyc06g0	2.15
sly00073	Metabolisr	Lipid meta	Cutin, sube	0.00	0.00	Solyc01g0	Solyc01g0	6.67
sly00073	Metabolisr	Lipid meta	Cutin, sube	0.00	0.00	Solyc12g1	Solyc12g1	0.00
sly00073	Metabolisr	Lipid meta	Cutin, sube	0.00	0.00	Solyc01g0	Solyc01g0	13.05
sly00073	Metabolisr	Lipid meta	Cutin, sube	0.00	0.00	Solyc03g0	Solyc03g0	2.51
sly00073	Metabolisr	Lipid meta	Cutin, sube	0.00	0.00	Solyc01g0	Solyc01g0	1566.92
sly00941	Metabolisr	Biosynthes	Flavonoid	0.00	0.01	Solyc02g0	Solyc02g0	0.08
sly00941	Metabolisr	Biosynthes	Flavonoid	0.00	0.01	Solyc05g0	Solyc05g0	0.06
sly00941	Metabolisr	Biosynthes	Flavonoid	0.00	0.01	Solyc11g0	Solyc11g0	0.28
sly00941	Metabolisr	Biosynthes	Flavonoid	0.00	0.01	Solyc09g0	Solyc09g0	0.06
sly00941	Metabolisr	Biosynthes	Flavonoid	0.00	0.01	Solyc03g1	Solyc03g1	2.44
sly00941	Metabolisr	Biosynthes	Flavonoid	0.00	0.01	Solyc07g0	Solyc07g0	0.49
sly00941	Metabolisr	Biosynthes	Flavonoid	0.00	0.01	Solyc01g0	Solyc01g0	3528.16
sly00941	Metabolisr	Biosynthes	Flavonoid	0.00	0.01	Solyc01g0	Solyc01g0	2.52
sly00941	Metabolisr	Biosynthes	Flavonoid	0.00	0.01	Solyc07g0	Solyc07g0	2.71

sly00941	Metabolisr	Biosynthes	Flavonoid	0.00	0.01	Solyc10g0	Solyc10g0	0.00
sly00940	Metabolisr	Biosynthes	Phenylpro]	0.00	0.01	Solyc10g0	Solyc10g0	4.26
sly00940	Metabolisr	Biosynthes	Phenylpro]	0.00	0.01	Solyc02g0	Solyc02g0	11.82
sly00940	Metabolisr	Biosynthes	Phenylpro]	0.00	0.01	Solyc06g0	Solyc06g0	2.22
sly00940	Metabolisr	Biosynthes	Phenylpro]	0.00	0.01	Solyc03g1	Solyc03g1	2.44
sly00940	Metabolisr	Biosynthes	Phenylpro]	0.00	0.01	Solyc07g0	Solyc07g0	0.49
sly00940	Metabolisr	Biosynthes	Phenylpro]	0.00	0.01	Solyc03g0	Solyc03g0	0.18
sly00940	Metabolisr	Biosynthes	Phenylpro]	0.00	0.01	Solyc12g0	Solyc12g0	4.01
sly00940	Metabolisr	Biosynthes	Phenylpro]	0.00	0.01	Solyc01g0	Solyc01g0	3528.16
sly00940	Metabolisr	Biosynthes	Phenylpro]	0.00	0.01	Solyc01g0	Solyc01g0	2.52
sly00940	Metabolisr	Biosynthes	Phenylpro]	0.00	0.01	Solyc03g0	Solyc03g0	5511.05
sly00940	Metabolisr	Biosynthes	Phenylpro]	0.00	0.01	Solyc06g0	Solyc06g0	2.25
sly00940	Metabolisr	Biosynthes	Phenylpro]	0.00	0.01	Solyc01g1	Solyc01g1	4.37
sly00940	Metabolisr	Biosynthes	Phenylpro]	0.00	0.01	Solyc02g0	Solyc02g0	0.07
sly00940	Metabolisr	Biosynthes	Phenylpro]	0.00	0.01	Solyc07g0	Solyc07g0	2.71
sly00940	Metabolisr	Biosynthes	Phenylpro]	0.00	0.01	Solyc09g0	Solyc09g0	2.82
sly00940	Metabolisr	Biosynthes	Phenylpro]	0.00	0.01	Solyc06g0	Solyc06g0	0.25
sly00940	Metabolisr	Biosynthes	Phenylpro]	0.00	0.01	Solyc03g0	Solyc03g0	0.39
sly00940	Metabolisr	Biosynthes	Phenylpro]	0.00	0.01	Solyc10g0	Solyc10g0	0.00
sly00940	Metabolisr	Biosynthes	Phenylpro]	0.00	0.01	Solyc07g0	Solyc07g0	2.87
sly01200	Metabolisr	Global and	Carbon me	0.01	0.09	Solyc07g0	Solyc07g0	0.35
sly01200	Metabolisr	Global and	Carbon me	0.01	0.09	Solyc01g0	Solyc01g0	2.62
sly01200	Metabolisr	Global and	Carbon me	0.01	0.09	Solyc01g0	Solyc01g0	2.46
sly01200	Metabolisr	Global and	Carbon me	0.01	0.09	Solyc01g1	Solyc01g1	4.98
sly01200	Metabolisr	Global and	Carbon me	0.01	0.09	Solyc09g0	Solyc09g0	2.88
sly01200	Metabolisr	Global and	Carbon me	0.01	0.09	Solyc04g0	Solyc04g0	8.55
sly01200	Metabolisr	Global and	Carbon me	0.01	0.09	Solyc08g0	Solyc08g0	3.48
sly01200	Metabolisr	Global and	Carbon me	0.01	0.09	Solyc07g0	Solyc07g0	3.93
sly01200	Metabolisr	Global and	Carbon me	0.01	0.09	Solyc01g0	Solyc01g0	3.54
sly01200	Metabolisr	Global and	Carbon me	0.01	0.09	Solyc06g0	Solyc06g0	2.01
sly01200	Metabolisr	Global and	Carbon me	0.01	0.09	Solyc05g0	Solyc05g0	3.22
sly01200	Metabolisr	Global and	Carbon me	0.01	0.09	Solyc10g0	Solyc10g0	2.23
sly01200	Metabolisr	Global and	Carbon me	0.01	0.09	Solyc02g0	Solyc02g0	2.01
sly01200	Metabolisr	Global and	Carbon me	0.01	0.09	Solyc03g0	RBCS-2A	2.71
sly01200	Metabolisr	Global and	Carbon me	0.01	0.09	Solyc08g0	Solyc08g0	2.43
sly01200	Metabolisr	Global and	Carbon me	0.01	0.09	Solyc09g0	Solyc09g0	25.41
sly01200	Metabolisr	Global and	Carbon me	0.01	0.09	Solyc03g1	Solyc03g1	6.29
sly01200	Metabolisr	Global and	Carbon me	0.01	0.09	Solyc04g0	Solyc04g0	39.10
sly01200	Metabolisr	Global and	Carbon me	0.01	0.09	Solyc02g0	Solyc02g0	2.27
sly01200	Metabolisr	Global and	Carbon me	0.01	0.09	Solyc04g0	Solyc04g0	32.87
sly01200	Metabolisr	Global and	Carbon me	0.01	0.09	Solyc03g0	Solyc03g0	0.44
sly01200	Metabolisr	Global and	Carbon me	0.01	0.09	Solyc02g0	Solyc02g0	3.52
sly00603	Metabolisr	Glycan bic	Glycosphi	0.01	0.13	Solyc01g0	Solyc01g0	2.01
sly00603	Metabolisr	Glycan bic	Glycosphi	0.01	0.13	Solyc06g0	Solyc06g0	3.62
sly00603	Metabolisr	Glycan bic	Glycosphi	0.01	0.13	Solyc03g0	Solyc03g0	3.84
sly00250	Metabolisr	Amino aci	Alanine, a:	0.01	0.13	Solyc04g0	Solyc04g0	6.77
sly00250	Metabolisr	Amino aci	Alanine, a:	0.01	0.13	Solyc04g0	Solyc04g0	0.40
sly00250	Metabolisr	Amino aci	Alanine, a:	0.01	0.13	Solyc11g0	Solyc11g0	0.50
sly00250	Metabolisr	Amino aci	Alanine, a:	0.01	0.13	Solyc01g1	Solyc01g1	2993.63
sly00250	Metabolisr	Amino aci	Alanine, a:	0.01	0.13	Solyc04g0	Solyc04g0	1711.14
sly00250	Metabolisr	Amino aci	Alanine, a:	0.01	0.13	Solyc01g0	Solyc01g0	550.66

sly00250	Metabolisr	Amino aci	Alanine, as	0.01	0.13	Solyc03g0	Solyc03g0	0.44
sly00710	Metabolisr	Energy me	Carbon fix	0.01	0.13	Solyc04g0	Solyc04g0	8.55
sly00710	Metabolisr	Energy me	Carbon fix	0.01	0.13	Solyc01g0	Solyc01g0	3.54
sly00710	Metabolisr	Energy me	Carbon fix	0.01	0.13	Solyc10g0	Solyc10g0	2.23
sly00710	Metabolisr	Energy me	Carbon fix	0.01	0.13	Solyc03g0	RBCS-2A	2.71
sly00710	Metabolisr	Energy me	Carbon fix	0.01	0.13	Solyc08g0	Solyc08g0	2.43
sly00710	Metabolisr	Energy me	Carbon fix	0.01	0.13	Solyc09g0	Solyc09g0	25.41
sly00710	Metabolisr	Energy me	Carbon fix	0.01	0.13	Solyc02g0	Solyc02g0	2.27
sly00710	Metabolisr	Energy me	Carbon fix	0.01	0.13	Solyc02g0	Solyc02g0	3.52
sly00196	Metabolisr	Energy me	Photosynth	0.02	0.19	Solyc09g0	Solyc09g0	5.85
sly00196	Metabolisr	Energy me	Photosynth	0.02	0.19	Solyc10g0	Solyc10g0	2.07
sly00196	Metabolisr	Energy me	Photosynth	0.02	0.19	Solyc03g0	Solyc03g0	2.60
sly00196	Metabolisr	Energy me	Photosynth	0.02	0.19	Solyc12g0	Solyc12g0	0.25
sly00945	Metabolisr	Biosynthes	Stilbenoid,	0.02	0.20	Solyc03g1	Solyc03g1	2.44
sly00945	Metabolisr	Biosynthes	Stilbenoid,	0.02	0.20	Solyc07g0	Solyc07g0	0.49
sly00945	Metabolisr	Biosynthes	Stilbenoid,	0.02	0.20	Solyc01g0	Solyc01g0	3528.16
sly00945	Metabolisr	Biosynthes	Stilbenoid,	0.02	0.20	Solyc01g0	Solyc01g0	2.52
sly00945	Metabolisr	Biosynthes	Stilbenoid,	0.02	0.20	Solyc07g0	Solyc07g0	2.71
sly00945	Metabolisr	Biosynthes	Stilbenoid,	0.02	0.20	Solyc10g0	Solyc10g0	0.00
sly00910	Metabolisr	Energy me	Nitrogen n	0.03	0.21	Solyc04g0	Solyc04g0	0.40
sly00910	Metabolisr	Energy me	Nitrogen n	0.03	0.21	Solyc01g0	Solyc01g0	550.66
sly00910	Metabolisr	Energy me	Nitrogen n	0.03	0.21	Solyc03g0	Solyc03g0	0.44
sly00910	Metabolisr	Energy me	Nitrogen n	0.03	0.21	Solyc01g1	Solyc01g1	4.58
sly00630	Metabolisr	Carbohydr	Glyoxylate	0.03	0.22	Solyc01g1	Solyc01g1	4.98
sly00630	Metabolisr	Carbohydr	Glyoxylate	0.03	0.22	Solyc04g0	Solyc04g0	0.40
sly00630	Metabolisr	Carbohydr	Glyoxylate	0.03	0.22	Solyc07g0	Solyc07g0	3.93
sly00630	Metabolisr	Carbohydr	Glyoxylate	0.03	0.22	Solyc06g0	Solyc06g0	2.01
sly00630	Metabolisr	Carbohydr	Glyoxylate	0.03	0.22	Solyc05g0	Solyc05g0	3.22
sly00630	Metabolisr	Carbohydr	Glyoxylate	0.03	0.22	Solyc03g0	RBCS-2A	2.71
sly00630	Metabolisr	Carbohydr	Glyoxylate	0.03	0.22	Solyc03g1	Solyc03g1	6.29
sly00630	Metabolisr	Carbohydr	Glyoxylate	0.03	0.22	Solyc01g0	Solyc01g0	550.66
sly00430	Metabolisr	Metabolisr	Taurine an	0.04	0.25	Solyc11g0	Solyc11g0	0.50
sly00430	Metabolisr	Metabolisr	Taurine an	0.04	0.25	Solyc04g0	Solyc04g0	1711.14
sly00902	Metabolisr	Metabolisr	Monoterpe	0.04	0.25	Solyc11g0	Solyc11g0	3.46
sly00902	Metabolisr	Metabolisr	Monoterpe	0.04	0.25	Solyc11g0	Solyc11g0	5.06
sly00600	Metabolisr	Lipid meta	Sphingolip	0.06	0.35	Solyc03g0	Solyc03g0	2.33
sly00600	Metabolisr	Lipid meta	Sphingolip	0.06	0.35	Solyc06g0	Solyc06g0	3.62
sly00600	Metabolisr	Lipid meta	Sphingolip	0.06	0.35	Solyc03g0	Solyc03g0	3.84
sly04016	Environme	Signal tran	MAPK sig	0.06	0.35	Solyc07g0	Solyc07g0	2.29
sly04016	Environme	Signal tran	MAPK sig	0.06	0.35	Solyc10g0	CHI9	0.28
sly04016	Environme	Signal tran	MAPK sig	0.06	0.35	Solyc09g0	Solyc09g0	0.27
sly04016	Environme	Signal tran	MAPK sig	0.06	0.35	Solyc07g0	Solyc07g0	2.15
sly04016	Environme	Signal tran	MAPK sig	0.06	0.35	Solyc03g1	Solyc03g1	0.48
sly04016	Environme	Signal tran	MAPK sig	0.06	0.35	Solyc08g0	Solyc08g0	2.67
sly04016	Environme	Signal tran	MAPK sig	0.06	0.35	Solyc09g0	Solyc09g0	0.25
sly04016	Environme	Signal tran	MAPK sig	0.06	0.35	Solyc01g0	Solyc01g0	3.53
sly04016	Environme	Signal tran	MAPK sig	0.06	0.35	Solyc04g0	Solyc04g0	0.46
sly04016	Environme	Signal tran	MAPK sig	0.06	0.35	Solyc01g0	Solyc01g0	0.42
sly04016	Environme	Signal tran	MAPK sig	0.06	0.35	Solyc08g0	Solyc08g0	4.19
sly04016	Environme	Signal tran	MAPK sig	0.06	0.35	Solyc07g0	Solyc07g0	2.88
sly04016	Environme	Signal tran	MAPK sig	0.06	0.35	Solyc04g0	Solyc04g0	732.58

sly00130	Metabolisr	Metabolisr	Ubiquinon	0.07	0.40	Solyc03g0	Solyc03g0	5.61
sly00130	Metabolisr	Metabolisr	Ubiquinon	0.07	0.40	Solyc12g0	Solyc12g0	2.20
sly00130	Metabolisr	Metabolisr	Ubiquinon	0.07	0.40	Solyc03g0	Solyc03g0	0.18
sly00130	Metabolisr	Metabolisr	Ubiquinon	0.07	0.40	Solyc05g0	Solyc05g0	2.69
sly00130	Metabolisr	Metabolisr	Ubiquinon	0.07	0.40	Solyc03g0	Solyc03g0	0.00
sly00030	Metabolisr	Carbohydr	Pentose ph	0.10	0.47	Solyc08g0	Solyc08g0	3.48
sly00030	Metabolisr	Carbohydr	Pentose ph	0.10	0.47	Solyc01g0	Solyc01g0	3.54
sly00030	Metabolisr	Carbohydr	Pentose ph	0.10	0.47	Solyc10g0	Solyc10g0	2.23
sly00030	Metabolisr	Carbohydr	Pentose ph	0.10	0.47	Solyc09g0	Solyc09g0	25.41
sly00030	Metabolisr	Carbohydr	Pentose ph	0.10	0.47	Solyc02g0	Solyc02g0	3.52
sly04712	Organisma	Environme	Circadian r	0.10	0.47	Solyc05g0	Solyc05g0	0.06
sly04712	Organisma	Environme	Circadian r	0.10	0.47	Solyc09g0	Solyc09g0	0.06
sly04712	Organisma	Environme	Circadian r	0.10	0.47	Solyc09g0	Solyc09g0	0.40
sly04712	Organisma	Environme	Circadian r	0.10	0.47	Solyc12g0	Solyc12g0	2.29
sly00020	Metabolisr	Carbohydr	Citrate cyc	0.10	0.47	Solyc05g0	Solyc05g0	2.80
sly00020	Metabolisr	Carbohydr	Citrate cyc	0.10	0.47	Solyc07g0	Solyc07g0	3.93
sly00020	Metabolisr	Carbohydr	Citrate cyc	0.10	0.47	Solyc12g0	Solyc12g0	7.81
sly00020	Metabolisr	Carbohydr	Citrate cyc	0.10	0.47	Solyc05g0	Solyc05g0	3.22
sly00020	Metabolisr	Carbohydr	Citrate cyc	0.10	0.47	Solyc04g0	Solyc04g0	39.10
sly00020	Metabolisr	Carbohydr	Citrate cyc	0.10	0.47	Solyc04g0	Solyc04g0	32.87
sly00053	Metabolisr	Carbohydr	Ascorbate	0.11	0.49	Solyc12g0	Solyc12g0	0.25
sly00053	Metabolisr	Carbohydr	Ascorbate	0.11	0.49	Solyc09g0	Solyc09g0	0.37
sly00053	Metabolisr	Carbohydr	Ascorbate	0.11	0.49	Solyc01g0	Solyc01g0	0.22
sly00053	Metabolisr	Carbohydr	Ascorbate	0.11	0.49	Solyc02g0	Solyc02g0	2.54
sly00053	Metabolisr	Carbohydr	Ascorbate	0.11	0.49	Solyc01g0	Solyc01g0	0.06
sly00053	Metabolisr	Carbohydr	Ascorbate	0.11	0.49	Solyc11g0	Solyc11g0	0.43
sly00531	Metabolisr	Glycan bio	Glycosami	0.11	0.49	Solyc01g0	Solyc01g0	2.01
sly00531	Metabolisr	Glycan bio	Glycosami	0.11	0.49	Solyc06g0	Solyc06g0	2.47
sly00410	Metabolisr	Metabolisr	beta-Alani	0.12	0.49	Solyc01g0	Solyc01g0	0.22
sly00410	Metabolisr	Metabolisr	beta-Alani	0.12	0.49	Solyc11g0	Solyc11g0	0.50
sly00410	Metabolisr	Metabolisr	beta-Alani	0.12	0.49	Solyc02g0	Solyc02g0	2.01
sly00410	Metabolisr	Metabolisr	beta-Alani	0.12	0.49	Solyc04g0	Solyc04g0	1711.14
sly00410	Metabolisr	Metabolisr	beta-Alani	0.12	0.49	Solyc01g0	Solyc01g0	0.03
sly00240	Metabolisr	Nucleotide	Pyrimidine	0.13	0.52	Solyc02g0	Solyc02g0	3.08
sly00240	Metabolisr	Nucleotide	Pyrimidine	0.13	0.52	Solyc05g0	Solyc05g0	4.45
sly00240	Metabolisr	Nucleotide	Pyrimidine	0.13	0.52	Solyc01g0	Solyc01g0	0.32
sly00240	Metabolisr	Nucleotide	Pyrimidine	0.13	0.52	Solyc01g0	Solyc01g0	2.02
sly00240	Metabolisr	Nucleotide	Pyrimidine	0.13	0.52	Solyc06g0	Solyc06g0	0.38
sly00750	Metabolisr	Metabolisr	Vitamin B	0.14	0.53	Solyc03g1	Solyc03g1	2.22
sly00750	Metabolisr	Metabolisr	Vitamin B	0.14	0.53	Solyc06g0	Solyc06g0	0.14
sly00010	Metabolisr	Carbohydr	Glycolysis	0.15	0.53	Solyc07g0	Solyc07g0	0.35
sly00010	Metabolisr	Carbohydr	Glycolysis	0.15	0.53	Solyc09g0	Solyc09g0	2.88
sly00010	Metabolisr	Carbohydr	Glycolysis	0.15	0.53	Solyc01g0	Solyc01g0	0.22
sly00010	Metabolisr	Carbohydr	Glycolysis	0.15	0.53	Solyc08g0	Solyc08g0	3.48
sly00010	Metabolisr	Carbohydr	Glycolysis	0.15	0.53	Solyc05g0	Solyc05g0	3.22
sly00010	Metabolisr	Carbohydr	Glycolysis	0.15	0.53	Solyc10g0	Solyc10g0	2.23
sly00010	Metabolisr	Carbohydr	Glycolysis	0.15	0.53	Solyc09g0	Solyc09g0	25.41
sly00010	Metabolisr	Carbohydr	Glycolysis	0.15	0.53	Solyc04g0	Solyc04g0	15.69
sly00010	Metabolisr	Carbohydr	Glycolysis	0.15	0.53	Solyc08g0	Solyc08g0	5.12
sly00010	Metabolisr	Carbohydr	Glycolysis	0.15	0.53	Solyc02g0	Solyc02g0	3.52
sly00564	Metabolisr	Lipid meta	Glyceroph	0.15	0.53	Solyc06g0	Solyc06g0	0.26

sly00564	Metabolisr	Lipid meta	Glyceroph	0.15	0.53	Solyc09g0	Solyc09g0	0.47
sly00564	Metabolisr	Lipid meta	Glyceroph	0.15	0.53	Solyc11g0	Solyc11g0	0.38
sly00564	Metabolisr	Lipid meta	Glyceroph	0.15	0.53	Solyc09g0	Solyc09g0	3.57
sly00564	Metabolisr	Lipid meta	Glyceroph	0.15	0.53	Solyc06g0	Solyc06g0	2.08
sly00564	Metabolisr	Lipid meta	Glyceroph	0.15	0.53	Solyc04g0	Solyc04g0	1523.79
sly00564	Metabolisr	Lipid meta	Glyceroph	0.15	0.53	Solyc09g0	Solyc09g0	3.37
sly00604	Metabolisr	Glycan bic	Glycosphi	0.16	0.56	Solyc01g0	Solyc01g0	2.01
sly00460	Metabolisr	Metabolisr	Cyanoami	0.17	0.56	Solyc04g0	Solyc04g0	6.77
sly00460	Metabolisr	Metabolisr	Cyanoami	0.17	0.56	Solyc01g0	Solyc01g0	2.46
sly00460	Metabolisr	Metabolisr	Cyanoami	0.17	0.56	Solyc06g0	Solyc06g0	2.25
sly00380	Metabolisr	Amino aci	Tryptophai	0.18	0.56	Solyc01g0	Solyc01g0	0.22
sly00380	Metabolisr	Amino aci	Tryptophai	0.18	0.56	Solyc05g0	Solyc05g0	3.22
sly00380	Metabolisr	Amino aci	Tryptophai	0.18	0.56	Solyc06g0	Solyc06g0	3.53
sly00380	Metabolisr	Amino aci	Tryptophai	0.18	0.56	Solyc03g0	Solyc03g0	0.39
sly00561	Metabolisr	Lipid meta	Glycerolip	0.18	0.56	Solyc01g0	Solyc01g0	0.22
sly00561	Metabolisr	Lipid meta	Glycerolip	0.18	0.56	Solyc06g0	Solyc06g0	3.62
sly00561	Metabolisr	Lipid meta	Glycerolip	0.18	0.56	Solyc03g0	Solyc03g0	3.84
sly00561	Metabolisr	Lipid meta	Glycerolip	0.18	0.56	Solyc11g0	Solyc11g0	0.38
sly00561	Metabolisr	Lipid meta	Glycerolip	0.18	0.56	Solyc04g0	Solyc04g0	1523.79
sly00561	Metabolisr	Lipid meta	Glycerolip	0.18	0.56	Solyc09g0	Solyc09g0	3.37
sly04075	Environme	Signal tran	Plant horm	0.20	0.60	Solyc07g0	Solyc07g0	2.29
sly04075	Environme	Signal tran	Plant horm	0.20	0.60	Solyc01g0	Solyc01g0	2.26
sly04075	Environme	Signal tran	Plant horm	0.20	0.60	Solyc07g0	Solyc07g0	6.62
sly04075	Environme	Signal tran	Plant horm	0.20	0.60	Solyc09g0	Solyc09g0	0.27
sly04075	Environme	Signal tran	Plant horm	0.20	0.60	Solyc08g0	Solyc08g0	0.20
sly04075	Environme	Signal tran	Plant horm	0.20	0.60	Solyc07g0	Solyc07g0	8.01
sly04075	Environme	Signal tran	Plant horm	0.20	0.60	Solyc09g0	Solyc09g0	0.25
sly04075	Environme	Signal tran	Plant horm	0.20	0.60	Solyc12g0	Solyc12g0	3.08
sly04075	Environme	Signal tran	Plant horm	0.20	0.60	Solyc01g1	Solyc01g1	0.20
sly04075	Environme	Signal tran	Plant horm	0.20	0.60	Solyc06g0	Solyc06g0	4013.78
sly04075	Environme	Signal tran	Plant horm	0.20	0.60	Solyc06g0	Solyc06g0	0.45
sly04075	Environme	Signal tran	Plant horm	0.20	0.60	Solyc01g0	Solyc01g0	0.42
sly04075	Environme	Signal tran	Plant horm	0.20	0.60	Solyc03g1	Solyc03g1	0.32
sly04075	Environme	Signal tran	Plant horm	0.20	0.60	Solyc04g0	Solyc04g0	732.58
sly04075	Environme	Signal tran	Plant horm	0.20	0.60	Solyc09g0	Solyc09g0	0.22
sly04626	Organisma	Environme	Plant-path	0.21	0.60	Solyc04g0	Solyc04g0	0.35
sly04626	Organisma	Environme	Plant-path	0.21	0.60	Solyc03g1	Solyc03g1	8.55
sly04626	Organisma	Environme	Plant-path	0.21	0.60	Solyc01g0	Solyc01g0	0.34
sly04626	Organisma	Environme	Plant-path	0.21	0.60	Solyc11g0	Solyc11g0	0.24
sly04626	Organisma	Environme	Plant-path	0.21	0.60	Solyc03g1	Solyc03g1	0.48
sly04626	Organisma	Environme	Plant-path	0.21	0.60	Solyc01g0	Solyc01g0	3.53
sly04626	Organisma	Environme	Plant-path	0.21	0.60	Solyc11g0	Solyc11g0	2.24
sly04626	Organisma	Environme	Plant-path	0.21	0.60	Solyc04g0	Solyc04g0	0.46
sly04626	Organisma	Environme	Plant-path	0.21	0.60	Solyc08g0	Solyc08g0	0.42
sly04626	Organisma	Environme	Plant-path	0.21	0.60	Solyc01g0	Solyc01g0	5.28
sly04626	Organisma	Environme	Plant-path	0.21	0.60	Solyc03g0	Solyc03g0	2638.78
sly04626	Organisma	Environme	Plant-path	0.21	0.60	Solyc01g0	Solyc01g0	0.00
sly04626	Organisma	Environme	Plant-path	0.21	0.60	Solyc06g0	PTI6	0.43
sly00650	Metabolisr	Carbohydr	Butanoate	0.22	0.60	Solyc03g0	Solyc03g0	4.05
sly00650	Metabolisr	Carbohydr	Butanoate	0.22	0.60	Solyc11g0	Solyc11g0	0.50
sly00650	Metabolisr	Carbohydr	Butanoate	0.22	0.60	Solyc04g0	Solyc04g0	1711.14

sly00220	Metabolisr	Amino aci	Arginine b	0.22	0.60	Solyc04g0	Solyc04g0	0.40
sly00220	Metabolisr	Amino aci	Arginine b	0.22	0.60	Solyc01g0	Solyc01g0	550.66
sly00220	Metabolisr	Amino aci	Arginine b	0.22	0.60	Solyc03g0	Solyc03g0	0.44
sly01240	Metabolisr	Global and	Biosynthes	0.22	0.60	Solyc01g0	Solyc01g0	2.62
sly01240	Metabolisr	Global and	Biosynthes	0.22	0.60	Solyc02g0	Solyc02g0	3.08
sly01240	Metabolisr	Global and	Biosynthes	0.22	0.60	Solyc12g0	Solyc12g0	0.25
sly01240	Metabolisr	Global and	Biosynthes	0.22	0.60	Solyc04g0	Solyc04g0	3.78
sly01240	Metabolisr	Global and	Biosynthes	0.22	0.60	Solyc09g0	Solyc09g0	2.88
sly01240	Metabolisr	Global and	Biosynthes	0.22	0.60	Solyc03g0	Solyc03g0	5.61
sly01240	Metabolisr	Global and	Biosynthes	0.22	0.60	Solyc09g0	Solyc09g0	0.37
sly01240	Metabolisr	Global and	Biosynthes	0.22	0.60	Solyc01g0	Solyc01g0	0.22
sly01240	Metabolisr	Global and	Biosynthes	0.22	0.60	Solyc12g0	Solyc12g0	2.20
sly01240	Metabolisr	Global and	Biosynthes	0.22	0.60	Solyc01g0	Solyc01g0	0.32
sly01240	Metabolisr	Global and	Biosynthes	0.22	0.60	Solyc05g0	Solyc05g0	3.22
sly01240	Metabolisr	Global and	Biosynthes	0.22	0.60	Solyc06g0	Solyc06g0	0.14
sly01240	Metabolisr	Global and	Biosynthes	0.22	0.60	Solyc08g0	Solyc08g0	2.11
sly01240	Metabolisr	Global and	Biosynthes	0.22	0.60	Solyc05g0	Solyc05g0	2.69
sly01240	Metabolisr	Global and	Biosynthes	0.22	0.60	Solyc03g0	Solyc03g0	0.00
sly01240	Metabolisr	Global and	Biosynthes	0.22	0.60	Solyc07g0	Solyc07g0	2.40
sly00965	Metabolisr	Biosynthes	Betalain bi	0.23	0.60	Solyc01g0	Solyc01g0	0.12
sly01040	Metabolisr	Lipid meta	Biosynthes	0.24	0.60	Solyc01g0	Solyc01g0	0.09
sly01040	Metabolisr	Lipid meta	Biosynthes	0.24	0.60	Solyc11g0	Solyc11g0	3.25
sly00071	Metabolisr	Lipid meta	Fatty acid	0.24	0.60	Solyc01g0	Solyc01g0	0.22
sly00071	Metabolisr	Lipid meta	Fatty acid	0.24	0.60	Solyc04g0	Solyc04g0	15.69
sly00071	Metabolisr	Lipid meta	Fatty acid	0.24	0.60	Solyc08g0	Solyc08g0	5.12
sly00500	Metabolisr	Carbohydr	Starch and	0.29	0.71	Solyc04g0	Solyc04g0	2.89
sly00500	Metabolisr	Carbohydr	Starch and	0.29	0.71	Solyc07g0	Solyc07g0	0.48
sly00500	Metabolisr	Carbohydr	Starch and	0.29	0.71	Solyc08g0	Solyc08g0	2.18
sly00500	Metabolisr	Carbohydr	Starch and	0.29	0.71	Solyc05g0	Solyc05g0	0.27
sly00500	Metabolisr	Carbohydr	Starch and	0.29	0.71	Solyc09g0	Solyc09g0	2.71
sly00500	Metabolisr	Carbohydr	Starch and	0.29	0.71	Solyc05g0	Solyc05g0	2.50
sly00500	Metabolisr	Carbohydr	Starch and	0.29	0.71	Solyc06g0	Solyc06g0	2.25
sly00660	Metabolisr	Carbohydr	C5-Branch	0.30	0.72	Solyc03g0	Solyc03g0	4.05
sly00740	Metabolisr	Metabolisr	Riboflavin	0.31	0.72	Solyc03g0	Solyc03g0	0.22
sly00740	Metabolisr	Metabolisr	Riboflavin	0.31	0.72	Solyc08g0	Solyc08g0	2.11
sly00640	Metabolisr	Carbohydr	Propanoate	0.31	0.72	Solyc04g0	Solyc04g0	6.11
sly00640	Metabolisr	Carbohydr	Propanoate	0.31	0.72	Solyc05g0	Solyc05g0	3.22
sly00640	Metabolisr	Carbohydr	Propanoate	0.31	0.72	Solyc02g0	Solyc02g0	2.01
sly00280	Metabolisr	Amino aci	Valine, leu	0.33	0.74	Solyc04g0	Solyc04g0	6.11
sly00280	Metabolisr	Amino aci	Valine, leu	0.33	0.74	Solyc01g0	Solyc01g0	0.22
sly00280	Metabolisr	Amino aci	Valine, leu	0.33	0.74	Solyc05g0	Solyc05g0	3.22
sly00280	Metabolisr	Amino aci	Valine, leu	0.33	0.74	Solyc02g0	Solyc02g0	2.01
sly00051	Metabolisr	Carbohydr	Fructose ai	0.34	0.74	Solyc08g0	Solyc08g0	3.48
sly00051	Metabolisr	Carbohydr	Fructose ai	0.34	0.74	Solyc10g0	Solyc10g0	2.91
sly00051	Metabolisr	Carbohydr	Fructose ai	0.34	0.74	Solyc10g0	Solyc10g0	2.23
sly00051	Metabolisr	Carbohydr	Fructose ai	0.34	0.74	Solyc09g0	Solyc09g0	25.41
sly00051	Metabolisr	Carbohydr	Fructose ai	0.34	0.74	Solyc02g0	Solyc02g0	3.52
sly00310	Metabolisr	Amino aci	Lysine deg	0.40	0.85	Solyc01g0	Solyc01g0	0.22
sly00310	Metabolisr	Amino aci	Lysine deg	0.40	0.85	Solyc05g0	Solyc05g0	3.22
sly00904	Metabolisr	Metabolisr	Diterpenoi	0.40	0.85	Solyc07g0	Solyc07g0	2.20
sly00904	Metabolisr	Metabolisr	Diterpenoi	0.40	0.85	Solyc07g0	Solyc07g0	29.42

sly00511	Metabolisr	Glycan bio	Other glyc	0.41	0.85	Solyc01g0	Solyc01g0	2.01
sly00052	Metabolisr	Carbohydr	Galactose	0.46	0.89	Solyc08g0	Solyc08g0	3.48
sly00052	Metabolisr	Carbohydr	Galactose	0.46	0.89	Solyc06g0	Solyc06g0	3.62
sly00052	Metabolisr	Carbohydr	Galactose	0.46	0.89	Solyc03g0	Solyc03g0	3.84
sly00591	Metabolisr	Lipid meta	Linoleic ac	0.46	0.89	Solyc08g0	LOX1.1	2.60
sly00903	Metabolisr	Metabolisr	Limonene	0.46	0.89	Solyc01g0	Solyc01g0	0.22
sly00905	Metabolisr	Metabolisr	Brassinost	0.46	0.89	Solyc02g0	Solyc02g0	0.48
sly00260	Metabolisr	Amino aci	Glycine, se	0.49	0.91	Solyc07g0	Solyc07g0	0.35
sly00260	Metabolisr	Amino aci	Glycine, se	0.49	0.91	Solyc01g1	Solyc01g1	4.98
sly00260	Metabolisr	Amino aci	Glycine, se	0.49	0.91	Solyc06g0	Solyc06g0	2.01
sly00260	Metabolisr	Amino aci	Glycine, se	0.49	0.91	Solyc05g0	Solyc05g0	3.22
sly01230	Metabolisr	Global and	Biosynthes	0.49	0.91	Solyc03g0	Solyc03g0	4.05
sly01230	Metabolisr	Global and	Biosynthes	0.49	0.91	Solyc07g0	Solyc07g0	0.35
sly01230	Metabolisr	Global and	Biosynthes	0.49	0.91	Solyc01g0	Solyc01g0	2.46
sly01230	Metabolisr	Global and	Biosynthes	0.49	0.91	Solyc09g0	Solyc09g0	2.88
sly01230	Metabolisr	Global and	Biosynthes	0.49	0.91	Solyc04g0	Solyc04g0	0.40
sly01230	Metabolisr	Global and	Biosynthes	0.49	0.91	Solyc08g0	Solyc08g0	3.48
sly01230	Metabolisr	Global and	Biosynthes	0.49	0.91	Solyc07g0	Solyc07g0	3.93
sly01230	Metabolisr	Global and	Biosynthes	0.49	0.91	Solyc01g0	Solyc01g0	3.54
sly01230	Metabolisr	Global and	Biosynthes	0.49	0.91	Solyc02g0	Solyc02g0	574.97
sly01230	Metabolisr	Global and	Biosynthes	0.49	0.91	Solyc01g0	Solyc01g0	550.66
sly01230	Metabolisr	Global and	Biosynthes	0.49	0.91	Solyc02g0	Solyc02g0	3.52
sly00563	Metabolisr	Glycan bio	Glycosylpl	0.51	0.92	Solyc01g0	Solyc01g0	0.35
sly00592	Metabolisr	Lipid meta	alpha-Lino	0.57	0.97	Solyc11g0	AOS2	3.20
sly00592	Metabolisr	Lipid meta	alpha-Lino	0.57	0.97	Solyc04g0	Solyc04g0	15.69
sly00520	Metabolisr	Carbohydr	Amino sug	0.57	0.97	Solyc01g1	Solyc01g1	4.83
sly00520	Metabolisr	Carbohydr	Amino sug	0.57	0.97	Solyc12g0	Solyc12g0	0.25
sly00520	Metabolisr	Carbohydr	Amino sug	0.57	0.97	Solyc01g0	Solyc01g0	2.01
sly00520	Metabolisr	Carbohydr	Amino sug	0.57	0.97	Solyc09g0	Solyc09g0	0.37
sly00520	Metabolisr	Carbohydr	Amino sug	0.57	0.97	Solyc10g0	CHI9	0.28
sly00520	Metabolisr	Carbohydr	Amino sug	0.57	0.97	Solyc07g0	Solyc07g0	0.14
sly00520	Metabolisr	Carbohydr	Amino sug	0.57	0.97	Solyc06g0	Solyc06g0	13.74
sly00340	Metabolisr	Amino aci	Histidine n	0.59	0.97	Solyc01g0	Solyc01g0	0.22
sly00565	Metabolisr	Lipid meta	Ether lipid	0.59	0.97	Solyc09g0	Solyc09g0	0.47
sly00770	Metabolisr	Metabolisr	Pantothena	0.60	0.97	Solyc03g0	Solyc03g0	4.05
sly00770	Metabolisr	Metabolisr	Pantothena	0.60	0.97	Solyc01g0	Solyc01g0	0.22
sly00860	Metabolisr	Metabolisr	Porphyrin	0.60	0.97	Solyc04g0	Solyc04g0	3.78
sly00860	Metabolisr	Metabolisr	Porphyrin	0.60	0.97	Solyc09g0	Solyc09g0	0.36
sly00860	Metabolisr	Metabolisr	Porphyrin	0.60	0.97	Solyc12g0	Solyc12g0	6.07
sly00330	Metabolisr	Amino aci	Arginine a	0.62	0.97	Solyc01g0	Solyc01g0	0.22
sly00330	Metabolisr	Amino aci	Arginine a	0.62	0.97	Solyc02g0	Solyc02g0	3.06
sly00330	Metabolisr	Amino aci	Arginine a	0.62	0.97	Solyc01g0	Solyc01g0	0.03
sly00760	Metabolisr	Metabolisr	Nicotinate	0.63	0.97	Solyc06g0	Solyc06g0	0.38
sly00790	Metabolisr	Metabolisr	Folate bios	0.63	0.97	Solyc07g0	Solyc07g0	2.40
sly00620	Metabolisr	Carbohydr	Pyruvate n	0.65	0.97	Solyc09g0	Solyc09g0	2.88
sly00620	Metabolisr	Carbohydr	Pyruvate n	0.65	0.97	Solyc01g0	Solyc01g0	0.22
sly00620	Metabolisr	Carbohydr	Pyruvate n	0.65	0.97	Solyc05g0	Solyc05g0	3.22
sly00620	Metabolisr	Carbohydr	Pyruvate n	0.65	0.97	Solyc03g1	Solyc03g1	6.29
sly00290	Metabolisr	Amino aci	Valine, leu	0.66	0.97	Solyc03g0	Solyc03g0	4.05
sly00350	Metabolisr	Amino aci	Tyrosine n	0.66	0.97	Solyc04g0	Solyc04g0	15.69
sly00350	Metabolisr	Amino aci	Tyrosine n	0.66	0.97	Solyc08g0	Solyc08g0	5.12

sly00190	Metabolisr	Energy me	Oxidative	0.68	0.97	Solyc12g0	Solyc12g0	2.97
sly00190	Metabolisr	Energy me	Oxidative	0.68	0.97	Solyc04g0	Solyc04g0	39.10
sly00190	Metabolisr	Energy me	Oxidative	0.68	0.97	Solyc06g0	Solyc06g0	15.51
sly00190	Metabolisr	Energy me	Oxidative	0.68	0.97	Solyc04g0	Solyc04g0	32.87
sly00190	Metabolisr	Energy me	Oxidative	0.68	0.97	Solyc12g0	Solyc12g0	4.99
sly01210	Metabolisr	Global and	2-Oxocarb	0.68	0.97	Solyc03g0	Solyc03g0	4.05
sly01210	Metabolisr	Global and	2-Oxocarb	0.68	0.97	Solyc07g0	Solyc07g0	3.93
sly00360	Metabolisr	Amino aci	Phenylalar	0.70	0.97	Solyc10g0	Solyc10g0	4.26
sly00360	Metabolisr	Amino aci	Phenylalar	0.70	0.97	Solyc08g0	Solyc08g0	0.13
sly00670	Metabolisr	Metabolisr	One carbo	0.71	0.97	Solyc01g0	Solyc01g0	2.62
sly00730	Metabolisr	Metabolisr	Thiamine	0.71	0.97	Solyc11g0	Solyc11g0	45.49
sly00900	Metabolisr	Metabolisr	Terpenoid	0.76	0.97	Solyc04g0	Solyc04g0	2.62
sly00900	Metabolisr	Metabolisr	Terpenoid	0.76	0.97	Solyc06g0	Solyc06g0	2.33
sly00513	Metabolisr	Glycan bio	Various ty	0.76	0.97	Solyc01g0	Solyc01g0	2.01
sly03440	Genetic In	Replicatio	Homologo	0.76	0.97	Solyc01g1	Solyc01g1	2.32
sly00480	Metabolisr	Metabolisr	Glutathion	0.77	0.97	Solyc02g0	Solyc02g0	2.54
sly00480	Metabolisr	Metabolisr	Glutathion	0.77	0.97	Solyc11g0	Solyc11g0	2.07
sly00480	Metabolisr	Metabolisr	Glutathion	0.77	0.97	Solyc06g0	Solyc06g0	0.15
sly01212	Metabolisr	Global and	Fatty acid	0.77	0.97	Solyc01g0	Solyc01g0	0.09
sly01212	Metabolisr	Global and	Fatty acid	0.77	0.97	Solyc11g0	Solyc11g0	3.25
sly00906	Metabolisr	Metabolisr	Carotenoic	0.78	0.97	Solyc04g0	Solyc04g0	3.50
sly00920	Metabolisr	Energy me	Sulfur met	0.80	0.97	Solyc01g0	Solyc01g0	2.46
sly00908	Metabolisr	Metabolisr	Zeatin bios	0.80	0.97	Solyc10g0	Solyc10g0	3.13
sly03410	Genetic In	Replicatio	Base excis	0.80	0.97	Solyc09g0	Solyc09g0	2.14
sly00562	Metabolisr	Carbohydr	Inositol ph	0.80	0.97	Solyc06g0	Solyc06g0	2.40
sly00562	Metabolisr	Carbohydr	Inositol ph	0.80	0.97	Solyc09g0	Solyc09g0	0.47
sly00230	Metabolisr	Nucleotide	Purine met	0.80	0.97	Solyc09g0	Solyc09g0	2.88
sly00230	Metabolisr	Nucleotide	Purine met	0.80	0.97	Solyc01g1	Solyc01g1	2993.63
sly00230	Metabolisr	Nucleotide	Purine met	0.80	0.97	Solyc06g0	Solyc06g0	0.38
sly00062	Metabolisr	Lipid meta	Fatty acid	0.82	0.98	Solyc04g0	Solyc04g0	0.35
sly00040	Metabolisr	Carbohydr	Pentose an	0.82	0.98	Solyc10g0	PG2	9.48
sly00040	Metabolisr	Carbohydr	Pentose an	0.82	0.98	Solyc09g0	Solyc09g0	3.61
sly00040	Metabolisr	Carbohydr	Pentose an	0.82	0.98	Solyc02g0	Solyc02g0	4235.90
sly00270	Metabolisr	Amino aci	Cysteine a	0.85	0.99	Solyc07g0	ACO1	2.82
sly00270	Metabolisr	Amino aci	Cysteine a	0.85	0.99	Solyc09g0	Solyc09g0	6.99
sly00270	Metabolisr	Amino aci	Cysteine a	0.85	0.99	Solyc01g0	Solyc01g0	2.46
sly00270	Metabolisr	Amino aci	Cysteine a	0.85	0.99	Solyc02g0	Solyc02g0	2.56
sly04070	Environme	Signal tran	Phosphatic	0.86	0.99	Solyc06g0	Solyc06g0	2.40
sly04070	Environme	Signal tran	Phosphatic	0.86	0.99	Solyc09g0	Solyc09g0	3.57
sly04130	Genetic In	Folding, sc	SNARE in	0.87	0.99	Solyc09g0	Solyc09g0	2.01
sly00100	Metabolisr	Lipid meta	Steroid bic	0.87	0.99	Solyc02g0	Solyc02g0	8.45
sly00061	Metabolisr	Lipid meta	Fatty acid	0.88	0.99	Solyc11g0	Solyc11g0	3.25
sly00400	Metabolisr	Amino aci	Phenylalar	0.93	1.00	Solyc02g0	Solyc02g0	574.97
sly03050	Genetic In	Folding, sc	Proteasom	0.94	1.00	Solyc01g1	Solyc01g1	0.00
sly04146	Cellular Pr	Transport	Peroxisom	0.97	1.00	Solyc05g0	Solyc05g0	0.48
sly04145	Cellular Pr	Transport	Phagosom	0.97	1.00	Solyc02g0	Solyc02g0	6.74
sly04145	Cellular Pr	Transport	Phagosom	0.97	1.00	Solyc06g0	Solyc06g0	15.51
sly04144	Cellular Pr	Transport	Endocytos	0.98	1.00	Solyc11g0	Solyc11g0	0.30
sly04144	Cellular Pr	Transport	Endocytos	0.98	1.00	Solyc06g0	Solyc06g0	2.09
sly04144	Cellular Pr	Transport	Endocytos	0.98	1.00	Solyc06g0	Solyc06g0	2.20
sly04144	Cellular Pr	Transport	Endocytos	0.98	1.00	Solyc02g0	Solyc02g0	2.47

sly03018	Genetic In	Folding, sc	RNA degra	0.99	1.00	Solyc08g0	Solyc08g0	3.48
sly03015	Genetic In	Translation	mRNA sur	0.99	1.00	Solyc06g0	Solyc06g0	10.22
sly03013	Genetic In	Translation	Nucleocyto	1.00	1.00	Solyc11g0	Solyc11g0	0.10
sly03040	Genetic In	Transcripti	Spliceoson	1.00	1.00	Solyc06g0	Solyc06g0	2.09
sly04141	Genetic In	Folding, sc	Protein pro	1.00	1.00	Solyc06g0	Solyc06g0	2.09
sly04141	Genetic In	Folding, sc	Protein pro	1.00	1.00	Solyc02g0	Solyc02g0	2.46
sly03010	Genetic In	Translation	Ribosome	1.00	1.00	Solyc09g0	Solyc09g0	4.17
sly03010	Genetic In	Translation	Ribosome	1.00	1.00	Solyc04g0	Solyc04g0	2.32
sly03010	Genetic In	Translation	Ribosome	1.00	1.00	Solyc10g0	Solyc10g0	2.18
sly03010	Genetic In	Translation	Ribosome	1.00	1.00	Solyc07g0	Solyc07g0	2.00
sly03010	Genetic In	Translation	Ribosome	1.00	1.00	Solyc09g0	rpl33	0.05
sly03010	Genetic In	Translation	Ribosome	1.00	1.00	Solyc09g0	Solyc09g0	2.84

log2(fc)	pval	qval	regulation
1.83	0.00	0.00	up
2.01	0.00	0.00	up
1.57	0.00	0.00	up
1.43	0.00	0.00	up
2.84	0.00	0.00	up
1.21	0.00	0.00	up
1.07	0.00	0.00	up
2.33	0.00	0.00	up
1.61	0.00	0.00	up
1.77	0.00	0.00	up
1.58	0.00	0.00	up
1.30	0.00	0.00	up
-2.52	0.00	0.01	down
10.59	0.00	0.01	up
2.17	0.01	0.03	up
1.14	0.01	0.03	up
1.49	0	0	up
3.25	0	0	up
2.27	0	0	up
-3.43	0	0	down
2.61	0.00	0.00	up
2.02	0.00	0.00	up
2.09	0.00	0.00	up
2.76	0.00	0.00	up
2.80	0.00	0.00	up
-1.51	0.00	0.00	down
-3.73	0.00	0.00	down
1.39	0.00	0.00	up
-4.07	0.00	0.00	down
1.83	0.00	0.00	up
1.30	0.00	0.00	up
1.70	0.00	0.00	up
1.62	0.00	0.00	up
2.16	0.00	0.00	up
-1.84	0.00	0.00	down
-4.05	0.00	0.00	down
1.49	0.00	0.00	up
1.15	0.00	0.00	up
-1.99	0.00	0.00	down
1.39	0.00	0.00	up
-1.50	0.00	0.00	down
1.01	0.00	0.00	up
1.68	0.00	0.00	up
2.32	0.00	0.00	up
1.28	0.00	0.00	up
1.92	0.00	0.00	up
1.53	0.00	0.00	up
1.81	0.00	0.00	up
2.49	0.00	0.00	up

-1.45	0.00	0.00	down
3.10	0.00	0.00	up
-1.84	0.00	0.00	down
-1.31	0.00	0.00	down
-2.18	0.00	0.00	down
1.80	0.00	0.00	up
1.22	0.00	0.00	up
1.98	0.00	0.00	up
1.57	0.00	0.00	up
1.14	0.00	0.00	up
1.26	0.00	0.00	up
-1.51	0.00	0.00	down
1.31	0.00	0.00	up
1.85	0.00	0.00	up
-1.02	0.00	0.00	down
1.85	0.00	0.00	up
1.54	0.00	0.00	up
2.55	0.00	0.00	up
1.53	0.00	0.00	up
-1.01	0.00	0.00	down
-1.65	0.00	0.00	down
2.96	0.00	0.00	up
-1.05	0.00	0.00	down
-1.07	0.00	0.00	down
1.21	0.00	0.00	up
1.12	0.00	0.00	up
-1.46	0.00	0.00	down
1.82	0.00	0.00	up
1.07	0.00	0.00	up
1.01	0.00	0.00	up
-1.87	0.00	0.00	down
1.69	0.00	0.00	up
-2.45	0.00	0.00	down
1.62	0.00	0.00	up
1.34	0.00	0.00	up
1.16	0.00	0.00	up
1.44	0.00	0.00	up
-2.16	0.00	0.00	down
1.05	0.00	0.00	up
1.36	0.00	0.00	up
1.32	0.00	0.00	up
1.82	0.00	0.00	up
1.22	0.00	0.00	up
-3.97	0.00	0.00	down
1.94	0.00	0.00	up
11.78	0.00	0.00	up
-2.82	0.00	0.00	down
1.33	0.00	0.00	up
12.05	0.00	0.00	up
12.43	0.00	0.00	up
-2.74	0.00	0.00	down

11.55	0.00	0.00	up
-1.41	0.00	0.00	down
1.01	0.00	0.00	up
1.84	0.00	0.00	up
1.17	0.00	0.00	up
2.13	0.00	0.00	up
-1.05	0.00	0.00	down
1.02	0.00	0.00	up
10.74	0.00	0.00	up
1.44	0.00	0.00	up
1.05	0.00	0.00	up
1.08	0.00	0.00	up
1.28	0.00	0.00	up
1.06	0.00	0.00	up
-3.82	0.00	0.00	down
4.67	0.00	0.00	up
1.43	0.00	0.00	up
1.44	0.00	0.00	up
1.38	0.00	0.00	up
2.65	0.00	0.00	up
1.50	0.00	0.00	up
-2.88	0.00	0.00	down
3.97	0.00	0.00	up
5.29	0.00	0.00	up
-2.01	0.00	0.00	down
-1.35	0.00	0.00	down
-2.01	0.00	0.00	down
3.95	0.00	0.00	up
1.75	0.00	0.00	up
2.34	0.00	0.01	up
3.71	0.00	0.01	up
5.51	0.00	0.01	up
-1.41	0.00	0.01	down
-11.43	0.00	0.01	down
-2.97	0.00	0.01	down
9.17	0.00	0.01	up
2.36	0.00	0.01	up
9.11	0.00	0.01	up
1.18	0.00	0.01	up
5.04	0.00	0.01	up
2.32	0.01	0.02	up
2.60	0.01	0.02	up
3.08	0.01	0.02	up
3.78	0.01	0.02	up
-9.79	0.01	0.02	down
-1.19	0.01	0.02	down
2.20	0.01	0.03	up
2.17	0.01	0.03	up
1.82	0.01	0.04	up
-1.23	0.02	0.05	down
-4.98	0.02	0.05	down

1.52	0.02	0.05	up
1.49	0	0	up
2.61	0.00	0.00	up
2.02	0.00	0.00	up
2.09	0.00	0.00	up
2.76	0.00	0.00	up
2.80	0.00	0.00	up
-1.51	0.00	0.00	down
-3.73	0.00	0.00	down
-4.07	0.00	0.00	down
1.30	0.00	0.00	up
-1.84	0.00	0.00	down
-4.05	0.00	0.00	down
1.49	0.00	0.00	up
1.15	0.00	0.00	up
1.39	0.00	0.00	up
-1.50	0.00	0.00	down
1.68	0.00	0.00	up
2.32	0.00	0.00	up
1.28	0.00	0.00	up
1.92	0.00	0.00	up
1.53	0.00	0.00	up
1.81	0.00	0.00	up
2.49	0.00	0.00	up
-1.45	0.00	0.00	down
-2.18	0.00	0.00	down
1.80	0.00	0.00	up
1.98	0.00	0.00	up
1.14	0.00	0.00	up
-1.02	0.00	0.00	down
1.53	0.00	0.00	up
-1.01	0.00	0.00	down
2.96	0.00	0.00	up
-1.05	0.00	0.00	down
-1.07	0.00	0.00	down
1.14	0.00	0.00	up
-1.46	0.00	0.00	down
1.82	0.00	0.00	up
1.01	0.00	0.00	up
1.69	0.00	0.00	up
-2.45	0.00	0.00	down
1.62	0.00	0.00	up
1.16	0.00	0.00	up
1.44	0.00	0.00	up
1.36	0.00	0.00	up
1.32	0.00	0.00	up
1.22	0.00	0.00	up
11.78	0.00	0.00	up
1.33	0.00	0.00	up
12.43	0.00	0.00	up
1.79	0.00	0.00	up

11.55	0.00	0.00 up
-1.41	0.00	0.00 down
1.84	0.00	0.00 up
1.17	0.00	0.00 up
2.13	0.00	0.00 up
-1.05	0.00	0.00 down
10.74	0.00	0.00 up
1.44	0.00	0.00 up
1.08	0.00	0.00 up
4.88	0.00	0.00 up
1.06	0.00	0.00 up
-3.82	0.00	0.00 down
4.67	0.00	0.00 up
-10.11	0.00	0.00 down
1.43	0.00	0.00 up
1.44	0.00	0.00 up
10.57	0.00	0.00 up
2.65	0.00	0.00 up
1.50	0.00	0.00 up
3.97	0.00	0.00 up
5.29	0.00	0.00 up
-2.01	0.00	0.00 down
-1.35	0.00	0.00 down
1.75	0.00	0.00 up
2.34	0.00	0.01 up
-1.41	0.00	0.01 down
-11.43	0.00	0.01 down
9.17	0.00	0.01 up
2.36	0.00	0.01 up
5.04	0.00	0.01 up
2.60	0.01	0.02 up
3.08	0.01	0.02 up
-9.79	0.01	0.02 down
1.82	0.01	0.04 up
1.52	0.02	0.05 up
-1.63	0.00	0.00 down
1.10	0.00	0.00 up
2.74	0.00	0.00 up
-10.11	0.00	0.00 down
3.71	0.00	0.01 up
1.33	0.00	0.01 up
10.61	0.00	0.01 up
-3.73	0.00	0.00 down
-4.07	0.00	0.00 down
-1.84	0.00	0.00 down
-4.05	0.00	0.00 down
1.28	0.00	0.00 up
-1.02	0.00	0.00 down
11.78	0.00	0.00 up
1.33	0.00	0.00 up
1.44	0.00	0.00 up

-9.79	0.01	0.02 down
2.09	0.00	0.00 up
3.56	0.00	0.00 up
1.15	0.00	0.00 up
1.28	0.00	0.00 up
-1.02	0.00	0.00 down
-2.45	0.00	0.00 down
2.00	0.00	0.00 up
11.78	0.00	0.00 up
1.33	0.00	0.00 up
12.43	0.00	0.00 up
1.17	0.00	0.00 up
2.13	0.00	0.00 up
-3.82	0.00	0.00 down
1.44	0.00	0.00 up
1.50	0.00	0.00 up
-2.01	0.00	0.00 down
-1.35	0.00	0.00 down
-9.79	0.01	0.02 down
1.52	0.02	0.05 up
-1.51	0.00	0.00 down
1.39	0.00	0.00 up
1.30	0.00	0.00 up
2.32	0.00	0.00 up
1.53	0.00	0.00 up
3.10	0.00	0.00 up
1.80	0.00	0.00 up
1.98	0.00	0.00 up
1.82	0.00	0.00 up
1.01	0.00	0.00 up
1.69	0.00	0.00 up
1.16	0.00	0.00 up
1.01	0.00	0.00 up
1.44	0.00	0.00 up
1.28	0.00	0.00 up
4.67	0.00	0.00 up
2.65	0.00	0.00 up
5.29	0.00	0.00 up
1.18	0.00	0.01 up
5.04	0.00	0.01 up
-1.19	0.01	0.02 down
1.82	0.01	0.04 up
1.01	0.00	0.00 up
1.85	0.00	0.00 up
1.94	0.00	0.00 up
2.76	0.00	0.00 up
-1.31	0.00	0.00 down
-1.01	0.00	0.00 down
11.55	0.00	0.00 up
10.74	0.00	0.00 up
9.11	0.00	0.01 up

-1.19	0.01	0.02 down
3.10	0.00	0.00 up
1.82	0.00	0.00 up
1.16	0.00	0.00 up
1.44	0.00	0.00 up
1.28	0.00	0.00 up
4.67	0.00	0.00 up
1.18	0.00	0.01 up
1.82	0.01	0.04 up
2.55	0.00	0.00 up
1.05	0.00	0.00 up
1.38	0.00	0.00 up
-2.01	0.00	0.00 down
1.28	0.00	0.00 up
-1.02	0.00	0.00 down
11.78	0.00	0.00 up
1.33	0.00	0.00 up
1.44	0.00	0.00 up
-9.79	0.01	0.02 down
-1.31	0.00	0.00 down
9.11	0.00	0.01 up
-1.19	0.01	0.02 down
2.20	0.01	0.03 up
2.32	0.00	0.00 up
-1.31	0.00	0.00 down
1.98	0.00	0.00 up
1.01	0.00	0.00 up
1.69	0.00	0.00 up
1.44	0.00	0.00 up
2.65	0.00	0.00 up
9.11	0.00	0.01 up
-1.01	0.00	0.00 down
10.74	0.00	0.00 up
1.79	0.00	0.00 up
2.34	0.00	0.01 up
1.22	0.00	0.00 up
1.85	0.00	0.00 up
1.94	0.00	0.00 up
1.20	0.00	0.00 up
-1.84	0.00	0.00 down
-1.90	0.00	0.00 down
1.11	0.00	0.00 up
-1.07	0.00	0.00 down
1.41	0.00	0.00 up
-1.98	0.00	0.00 down
1.82	0.00	0.00 up
-1.11	0.00	0.00 down
-1.24	0.00	0.01 down
2.07	0.01	0.02 up
1.52	0.01	0.02 up
9.52	0.02	0.04 up

2.49	0.00	0.00	up
1.14	0.00	0.00	up
-2.45	0.00	0.00	down
1.43	0.00	0.00	up
-11.43	0.00	0.01	down
1.80	0.00	0.00	up
1.82	0.00	0.00	up
1.16	0.00	0.00	up
4.67	0.00	0.00	up
1.82	0.01	0.04	up
-4.07	0.00	0.00	down
-4.05	0.00	0.00	down
-1.34	0.00	0.00	down
1.20	0.00	0.00	up
1.49	0.00	0.00	up
1.98	0.00	0.00	up
2.96	0.00	0.00	up
1.69	0.00	0.00	up
5.29	0.00	0.00	up
5.04	0.00	0.01	up
-1.99	0.00	0.00	down
-1.45	0.00	0.00	down
-2.18	0.00	0.00	down
1.34	0.00	0.00	up
-3.97	0.00	0.00	down
-1.23	0.02	0.05	down
1.01	0.00	0.00	up
1.31	0.00	0.00	up
-2.18	0.00	0.00	down
-1.01	0.00	0.00	down
1.01	0.00	0.00	up
10.74	0.00	0.00	up
-4.98	0.02	0.05	down
1.62	0.00	0.00	up
2.16	0.00	0.00	up
-1.65	0.00	0.00	down
1.02	0.00	0.00	up
-1.41	0.00	0.01	down
1.15	0.00	0.00	up
-2.82	0.00	0.00	down
-1.51	0.00	0.00	down
1.53	0.00	0.00	up
-2.18	0.00	0.00	down
1.80	0.00	0.00	up
1.69	0.00	0.00	up
1.16	0.00	0.00	up
4.67	0.00	0.00	up
3.97	0.00	0.00	up
2.36	0.00	0.01	up
1.82	0.01	0.04	up
-1.97	0.00	0.00	down

-1.07	0.00	0.00	down
-1.41	0.00	0.00	down
1.84	0.00	0.00	up
1.06	0.00	0.00	up
10.57	0.00	0.00	up
1.75	0.00	0.00	up
1.01	0.00	0.00	up
2.76	0.00	0.00	up
1.30	0.00	0.00	up
1.17	0.00	0.00	up
-2.18	0.00	0.00	down
1.69	0.00	0.00	up
1.82	0.00	0.00	up
-1.35	0.00	0.00	down
-2.18	0.00	0.00	down
1.85	0.00	0.00	up
1.94	0.00	0.00	up
-1.41	0.00	0.00	down
10.57	0.00	0.00	up
1.75	0.00	0.00	up
1.20	0.00	0.00	up
1.17	0.00	0.00	up
2.73	0.00	0.00	up
-1.90	0.00	0.00	down
-2.35	0.00	0.00	down
3.00	0.00	0.00	up
-1.98	0.00	0.00	down
1.62	0.00	0.00	up
-2.35	0.00	0.00	down
11.97	0.00	0.00	up
-1.16	0.00	0.01	down
-1.24	0.00	0.01	down
-1.65	0.00	0.01	down
9.52	0.02	0.04	up
-2.22	0.02	0.05	down
-1.50	0.00	0.00	down
3.10	0.00	0.00	up
-1.54	0.00	0.00	down
-2.04	0.00	0.00	down
-1.07	0.00	0.00	down
1.82	0.00	0.00	up
1.16	0.00	0.00	up
-1.11	0.00	0.00	down
-1.25	0.00	0.00	down
2.40	0.00	0.00	up
11.37	0.00	0.00	up
-9.77	0.00	0.02	down
-1.22	0.01	0.02	down
2.02	0.00	0.00	up
-1.01	0.00	0.00	down
10.74	0.00	0.00	up

-1.31	0.00	0.00	down
9.11	0.00	0.01	up
-1.19	0.01	0.02	down
1.39	0.00	0.00	up
1.62	0.00	0.00	up
-1.99	0.00	0.00	down
1.92	0.00	0.00	up
1.53	0.00	0.00	up
2.49	0.00	0.00	up
-1.45	0.00	0.00	down
-2.18	0.00	0.00	down
1.14	0.00	0.00	up
-1.65	0.00	0.00	down
1.69	0.00	0.00	up
-2.82	0.00	0.00	down
1.08	0.00	0.00	up
1.43	0.00	0.00	up
-11.43	0.00	0.01	down
1.26	0.00	0.01	up
-3.03	0.00	0.00	down
-3.43	0	0	down
1.70	0.00	0.00	up
-2.18	0.00	0.00	down
3.97	0.00	0.00	up
2.36	0.00	0.01	up
1.53	0.00	0.00	up
-1.05	0.00	0.00	down
1.12	0.00	0.00	up
-1.87	0.00	0.00	down
1.44	0.00	0.00	up
1.32	0.00	0.00	up
1.17	0.00	0.00	up
2.02	0.00	0.00	up
-2.16	0.00	0.00	down
1.08	0.00	0.00	up
2.61	0.00	0.00	up
1.69	0.00	0.00	up
1.01	0.00	0.00	up
2.61	0.00	0.00	up
-2.18	0.00	0.00	down
1.69	0.00	0.00	up
1.01	0.00	0.00	up
1.80	0.00	0.00	up
1.54	0.00	0.00	up
1.16	0.00	0.00	up
4.67	0.00	0.00	up
1.82	0.01	0.04	up
-2.18	0.00	0.00	down
1.69	0.00	0.00	up
1.14	0.00	0.00	up
4.88	0.00	0.00	up

1.01	0.00	0.00	up
1.80	0.00	0.00	up
1.85	0.00	0.00	up
1.94	0.00	0.00	up
1.38	0.00	0.00	up
-2.18	0.00	0.00	down
-1.05	0.00	0.00	down
-1.51	0.00	0.00	down
2.32	0.00	0.00	up
1.01	0.00	0.00	up
1.69	0.00	0.00	up
2.02	0.00	0.00	up
-1.51	0.00	0.00	down
1.30	0.00	0.00	up
1.53	0.00	0.00	up
-1.31	0.00	0.00	down
1.80	0.00	0.00	up
1.98	0.00	0.00	up
1.82	0.00	0.00	up
9.17	0.00	0.01	up
9.11	0.00	0.01	up
1.82	0.01	0.04	up
-1.51	0.00	0.00	down
1.68	0.00	0.00	up
3.97	0.00	0.00	up
2.27	0	0	up
-1.99	0.00	0.00	down
1.01	0.00	0.00	up
-1.45	0.00	0.00	down
-1.84	0.00	0.00	down
-2.88	0.00	0.00	down
3.78	0.01	0.02	up
-2.18	0.00	0.00	down
-1.07	0.00	0.00	down
2.02	0.00	0.00	up
-2.18	0.00	0.00	down
1.92	0.00	0.00	up
-1.46	0.00	0.00	down
2.60	0.01	0.02	up
-2.18	0.00	0.00	down
1.62	0.00	0.00	up
-4.98	0.02	0.05	down
-1.41	0.00	0.01	down
1.26	0.00	0.01	up
1.53	0.00	0.00	up
-2.18	0.00	0.00	down
1.69	0.00	0.00	up
2.65	0.00	0.00	up
2.02	0.00	0.00	up
3.97	0.00	0.00	up
2.36	0.00	0.01	up

1.57	0.00	0.00	up
5.29	0.00	0.00	up
3.95	0.00	0.00	up
5.04	0.00	0.01	up
2.32	0.01	0.02	up
2.02	0.00	0.00	up
1.98	0.00	0.00	up
2.09	0.00	0.00	up
-2.97	0.00	0.01	down
1.39	0.00	0.00	up
5.51	0.00	0.01	up
1.39	0.00	0.00	up
1.22	0.00	0.00	up
1.01	0.00	0.00	up
1.22	0.00	0.00	up
1.34	0.00	0.00	up
1.05	0.00	0.00	up
-2.74	0.00	0.00	down
-3.43	0	0	down
1.70	0.00	0.00	up
1.81	0.00	0.00	up
1.30	0.00	0.00	up
1.64	0.00	0.00	up
1.10	0.00	0.00	up
1.26	0.00	0.00	up
-1.07	0.00	0.00	down
1.53	0.00	0.00	up
11.55	0.00	0.00	up
-1.41	0.00	0.01	down
-1.50	0.00	0.00	down
3.25	0	0	up
1.85	0.00	0.00	up
12.05	0.00	0.00	up
1.49	0	0	up
2.80	0.00	0.00	up
1.30	0.00	0.00	up
1.36	0.00	0.00	up
1.26	0.00	0.00	up
1.84	0.00	0.00	up
1.01	0.00	0.00	up
3.08	0.01	0.02	up
1.70	0.00	0.00	up
9.17	0.00	0.01	up
-9.07	0.00	0.01	down
-1.05	0.00	0.00	down
2.75	0.00	0.00	up
3.95	0.00	0.00	up
-1.72	0.00	0.00	down
1.07	0.00	0.00	up
1.14	0.00	0.00	up
1.31	0.01	0.03	up

1.80	0.00	0.00	up
3.35	0.00	0.01	up
-3.26	0.00	0.00	down
1.07	0.00	0.00	up
1.07	0.00	0.00	up
1.30	0.00	0.00	up
2.06	0.00	0.00	up
1.22	0.00	0.00	up
1.13	0.00	0.00	up
1.00	0.00	0.00	up
-4.19	0.00	0.00	down
1.51	0.00	0.00	up
