# Supplementary material for: Transcriptome reveals differential expression of flavor and color in closely related strains of tomato (Solanum lycopersicum)
Source: PeerJ. 2025 Oct 7;13:e20113. doi: 10.7717/peerj.20113 (PMC12513376; doi:10.7717/peerj.20113)
Supplement: Supplemental Information 11 [file peerj-13-20113-s011.pdf]

Table S9: The results of TF prediction in two de

Br		MF	
gene_id	TF_Family	gene_id	TF_Family
Solyc09g0	bZIP	Solyc09g0	bZIP
Solyc11g0	TCP	Solyc04g0	ERF
Solyc04g0	ERF	Solyc12g0	C3H
Solyc07g0	C3H	Solyc11g0	GRAS
Solyc06g0	GRAS	Solyc01g1	C2H2
Solyc07g0	ERF	Solyc02g0	bHLH
Solyc04g0	RAV	Solyc04g0	C2H2
Solyc04g0	C2H2	Solyc11g0	TCP
Solyc01g1	C2H2	Solyc04g0	GRAS
Solyc04g0	ERF	Solyc07g0	ERF
Solyc04g0	bZIP	Solyc06g0	GRAS
Solyc07g0	ERF	Solyc03g0	GRAS
Solyc05g0	ERF	Solyc09g0	ERF
Solyc03g0	ERF	Solyc05g0	ERF
Solyc10g0	MYB	Solyc05g0	ERF
Solyc12g0	B3	Solyc01g0	Trihelix
Solyc02g0	HD-ZIP	Solyc03g0	GRF
Solyc06g0	NF-YC	Solyc04g0	MYB
Solyc05g0	ERF	Solyc07g0	GRAS
Solyc03g0	ERF	Solyc03g1	GRAS
Solyc10g0	ERF	Solyc06g0	NF-YB
Solyc02g0	GRAS	Solyc02g0	SRS
Solyc05g0	bHLH	Solyc06g0	NF-YC
Solyc12g0	ERF	Solyc02g0	ZF-HD
Solyc06g0	C2H2	Solyc08g0	bHLH
Solyc02g0	ERF	Solyc03g1	Dof
Solyc03g0	GRAS	Solyc02g0	GRAS
Solyc03g0	ERF	Solyc10g0	G2-like
Solyc04g0	MYB	Solyc01g0	C2H2
Solyc12g0	C3H	Solyc07g0	ERF
Solyc02g0	ERF	Solyc03g0	bHLH
Solyc07g0	ERF	Solyc02g0	Dof
Solyc09g0	GRAS	Solyc01g0	GeBP
Solyc07g0	GRAS	Solyc06g0	C2H2
Solyc11g0	ERF	Solyc04g0	bHLH
Solyc10g0	G2-like	Solyc06g0	ERF
Solyc02g0	GRAS	Solyc02g0	ERF
Solyc02g0	ERF	Solyc07g0	ERF
Solyc03g0	bHLH	Solyc06g0	TCP
Solyc06g0	Dof	Solyc11g0	GRAS
Solyc06g0	Trihelix	Solyc06g0	ERF
Solyc01g0	Trihelix	Solyc08g0	FAR1
Solyc04g0	EIL	Solyc12g0	ERF
Solyc09g0	GRAS	Solyc05g0	bHLH
Solyc03g1	ERF	Solyc04g0	EIL
Solyc09g0	ERF	Solyc05g0	M-type_MADS
Solyc08g0	ERF	Solyc02g0	ERF
Solyc05g0	ERF	Solyc02g0	bZIP
Solyc05g0	C2H2	Solyc07g0	C3H
Solyc06g0	C2H2	Solyc05g0	GeBP

Solyc11g0	GRAS	Solyc09g0	GRAS
Solyc11g0	GRAS	Solyc02g0	bHLH
Solyc08g0	bHLH	Solyc04g0	M-type_MADS
Solyc10g0	WRKY	Solyc07g0	GRAS
Solyc05g0	ERF	Solyc04g0	bZIP
Solyc01g0	NAC	Solyc03g0	ERF
Solyc10g0	C2H2	Solyc03g0	ERF
Solyc08g0	FAR1	Solyc04g0	RAV
Solyc09g0	TCP	Solyc02g0	GRAS
Solyc04g0	ERF	Solyc06g0	Dof
Solyc02g0	ZF-HD	Solyc10g0	ERF
Solyc06g0	B3	Solyc01g0	MYB_related
Solyc03g0	bZIP	Solyc11g0	GRAS
Solyc04g0	bHLH	Solyc08g0	TCP
Solyc10g0	bZIP	Solyc04g0	ERF
Solyc03g0	bZIP	Solyc02g0	TCP
Solyc09g0	C2H2	Solyc12g1	Trihelix
Solyc02g0	bHLH	Solyc11g0	ERF
Solyc08g0	TCP	Solyc09g0	GRAS
Solyc02g0	Dof	Solyc01g0	GRAS
Solyc04g0	C2H2	Solyc03g0	ERF
Solyc03g1	NF-YC	Solyc10g0	ERF
Solyc04g0	GRAS	Solyc10g0	C2H2
Solyc03g0	GRF	Solyc02g0	TCP
Solyc04g0	MYB	Solyc03g0	ERF
Solyc07g0	ERF	Solyc02g0	GRAS
Solyc02g0	HSF	Solyc07g0	C2H2
Solyc03g0	EIL	Solyc03g0	bZIP
Solyc02g0	GeBP	Solyc10g0	MYB
Solyc10g0	ERF	Solyc07g0	ERF
Solyc02g0	ERF	Solyc06g0	C2H2
Solyc12g0	ERF	Solyc09g0	C2H2
Solyc07g0	C2H2	Solyc05g0	C2H2
Solyc01g1	MYB_rela	Solyc09g0	ERF
Solyc04g0	NF-YB	Solyc02g0	ERF
Solyc06g0	ERF	Solyc10g0	WRKY
Solyc08g0	C2H2	Solyc03g1	Trihelix
Solyc12g0	GRAS	Solyc02g0	GRAS
Solyc09g0	ERF	Solyc04g0	MYB
Solyc04g0	M-type_M	Solyc07g0	C2H2
Solyc02g0	bHLH	Solyc01g1	M-type_MADS
Solyc03g1	GRAS	Solyc11g0	C2H2
Solyc11g0	GRAS	Solyc02g0	ERF
Solyc11g0	Dof	Solyc02g0	B3
Solyc01g0	G2-like	Solyc04g0	AP2
Solyc08g0	BBR-BPC	Solyc08g0	BBR-BPC
Solyc02g0	Trihelix	Solyc03g1	LBD
Solyc03g1	ERF	Solyc01g0	GRAS
Solyc01g0	GRAS	Solyc05g0	ZF-HD
Solyc03g1	NAC	Solyc05g0	ERF
Solyc10g0	TCP	Solyc04g0	Dof
Solyc10g0	ERF	Solyc03g1	C2H2
Solyc01g0	GRAS	Solyc03g0	ERF

Solyc01g0	GRAS	Solyc04g0	NF-YB
Solyc05g0	C2H2	Solyc02g0	ERF
Solyc12g0	bHLH	Solyc08g0	ERF
Solyc05g0	GeBP	Solyc11g0	Dof
Solyc03g1	Dof	Solyc06g0	C2H2
Solyc08g0	ERF	Solyc05g0	ERF
Solyc03g0	ERF	Solyc08g0	ERF
Solyc10g0	C2H2	Solyc04g0	TCP
Solyc02g0	Dof	Solyc08g0	C2H2
Solyc11g0	GRAS	Solyc03g0	NAC
Solyc02g0	ZF-HD	Solyc03g0	G2-like
Solyc04g0	ERF	Solyc10g0	bZIP
Solyc02g0	bZIP	Solyc10g0	GRAS
Solyc09g0	ERF	Solyc02g0	LBD
Solyc10g0	M-type_M	Solyc11g0	C2H2
Solyc05g0	TCP	Solyc03g1	NF-YC
Solyc11g0	C2H2	Solyc06g0	M-type_MADS
Solyc02g0	C2H2	Solyc12g0	GRAS
Solyc11g0	ERF	Solyc02g0	TCP
Solyc08g0	ERF	Solyc03g1	C2H2
Solyc02g0	GRAS	Solyc02g0	Trihelix
Solyc02g0	GeBP	Solyc03g0	TCP
Solyc08g0	GRAS	Solyc06g0	ERF
Solyc08g0	C2H2	Solyc10g0	ERF
Solyc09g0	C2H2	Solyc08g0	ERF
Solyc02g0	GRAS	Solyc08g0	GRAS
Solyc02g0	NAC	Solyc09g0	MYB
Solyc11g0	C2H2	Solyc02g0	ZF-HD
Solyc03g0	TCP	Solyc06g0	GRAS
Solyc02g0	B3	Solyc05g0	bHLH
Solyc01g0	M-type_M	Solyc02g0	bZIP
Solyc06g0	M-type_M	Solyc03g0	bZIP
Solyc11g0	M-type_M	Solyc06g0	B3
Solyc03g1	LBD	Solyc05g0	GRAS
Solyc09g0	NF-YB	Solyc06g0	C2H2
Solyc02g0	ERF	Solyc10g0	bHLH
Solyc08g0	GRAS	Solyc10g0	ERF
Solyc09g0	MYB	Solyc08g0	ERF
Solyc04g0	AP2	Solyc04g0	TALE
Solyc01g0	MYB_rela	Solyc03g1	ERF
Solyc02g0	TCP	Solyc05g0	C2H2
Solyc01g0	C2H2	Solyc02g0	NAC
Solyc04g0	TALE	Solyc03g0	TCP
Solyc12g0	ERF	Solyc09g0	TCP
Solyc02g0	MYB_rela	Solyc03g1	ZF-HD
Solyc09g0	HB-other	Solyc04g0	ERF
Solyc02g0	NAC	Solyc08g0	Trihelix
Solyc11g0	C2H2	Solyc04g0	C2H2
Solyc02g0	GRAS	Solyc02g0	Dof
Solyc06g0	NF-YB	Solyc12g0	ERF
Solyc03g1	C2H2	Solyc03g1	C2H2
Solyc03g1	Trihelix	Solyc10g0	C2H2
Solyc04g0	Dof	Solyc01g0	NAC

Solyc08g0	ERF	Solyc12g0	bHLH
Solyc02g0	bZIP	Solyc01g1	MYB_related
Solyc08g0	Trihelix	Solyc02g0	HD-ZIP
Solyc07g0	C2H2	Solyc05g0	C2H2
Solyc02g0	SRS	Solyc01g0	B3
Solyc03g1	C2H2	Solyc07g0	NZZ/SPL
Solyc06g0	ERF	Solyc05g0	TCP
Solyc05g0	GRAS	Solyc03g0	EIL
Solyc03g1	C2H2	Solyc08g0	ERF
Solyc06g0	C2H2	Solyc12g0	B3
Solyc08g0	ERF	Solyc06g0	Trihelix
Solyc05g0	bHLH	Solyc11g0	ERF
Solyc01g0	GeBP	Solyc11g0	GRAS
Solyc03g0	TCP	Solyc09g0	ERF
Solyc02g0	TCP	Solyc01g0	G2-like
Solyc10g0	bHLH	Solyc08g0	ERF
Solyc08g0	ERF	Solyc03g1	ERF
Solyc06g0	GRAS	Solyc01g0	GRAS
Solyc08g0	ERF	Solyc02g0	GeBP
Solyc06g0	TCP	Solyc08g0	C2H2
Solyc05g0	ZF-HD	Solyc11g0	M-type_MADS
Solyc10g0	GRAS	Solyc10g0	M-type_MADS
Solyc11g0	NAC	Solyc02g0	GeBP
Solyc05g0	TCP	Solyc09g0	NF-YB
Solyc02g0	TCP	Solyc10g0	TCP
Solyc06g0	C2H2	Solyc11g0	C2H2
Solyc02g0	ERF	Solyc02g0	GRAS
Solyc03g0	ERF	Solyc02g0	NAC
Solyc07g0	GRAS	Solyc09g0	C2H2
Solyc06g0	ERF	Solyc03g1	NAC
Solyc12g1	Trihelix	Solyc09g0	HB-other
Solyc02g0	bZIP	Solyc08g0	GRAS
Solyc03g1	ZF-HD	Solyc02g0	C2H2
Solyc05g0	C2H2	Solyc12g0	ERF
Solyc05g0	M-type_M	Solyc01g0	M-type_MADS
Solyc02g0	NAC	Solyc02g0	ERF
Solyc01g1	M-type_M	Solyc02g0	NAC
Solyc01g0	B3	Solyc02g0	HSF
Solyc10g0	ERF	Solyc05g0	TCP
Solyc04g0	TCP	Solyc02g0	MYB_related
Solyc02g0	LBD	Solyc02g0	bZIP
Solyc07g0	NZZ/SPL	Solyc11g0	NAC
Solyc03g0	NAC	Solyc08g0	ERF
Solyc03g0	G2-like	Solyc04g0	ERF
